# Enormous expansion of the chemosensory gene repertoire in the omnivorous German cockroach *Blattella germanica*

**DOI:** 10.1002/jez.b.22797

**Published:** 2018-03-22

**Authors:** Hugh M. Robertson, Rachel L. Baits, Kimberly K.O. Walden, Ayako Wada‐Katsumata, Coby Schal

**Affiliations:** ^1^ Department of Entomology University of Illinois at Urbana‐Champaign Urbana Illinois USA; ^2^ Department of Entomology and Plant Pathology North Carolina State University Raleigh North Carolina USA

## Abstract

The acquisition of genome sequences from a wide range of insects and other arthropods has revealed a broad positive correlation between the complexity of their chemical ecology and the size of their chemosensory gene repertoire. The German cockroach *Blattella germanica* is an extreme omnivore and has the largest chemosensory gene repertoire known for an arthropod, exceeding even the highly polyphagous spider mite *Tetranychus urticae*. While the Odorant Receptor family is not particularly large, with 123 genes potentially encoding 134 receptors (105 intact), the Gustatory Receptor family is greatly expanded to 431 genes potentially encoding 545 receptors (483 intact), the largest known for insects and second only to the spider mite. The Ionotropic Receptor family of olfactory and gustatory receptors is vastly expanded to at least 897 genes (604 intact), the largest size known in arthropods, far surpassing the 150 known from the dampwood termite *Zootermopsis nevadensis*. Commensurately, the Odorant Binding Protein family is expanded to the largest known for insects at 109 genes (all intact). Comparison with the far more specialized, but phylogenetically related termite, within the Dictyoptera, reveals considerable gene losses from the termite, and massive species‐specific gene expansions in the cockroach. The cockroach has lost function of 11%–41% of these three chemoreceptor gene families to pseudogenization, and most of these are young events, implying rapid turnover of genes along with these major expansions, presumably in response to changes in its chemical ecology.

## 1 INTRODUCTION

The past two decades have seen the availability of genome sequences for numerous insects and other arthropods. Among the many insights these have provided is recognition of the extremely variable sizes of the various gene families involved in allowing arthropods to sense external chemicals. These gene families include the Odorant Binding Protein (OBP), Odorant Receptor (OR), Gustatory Receptor (GR), and Ionotropic Receptor (IR) families, as well as some smaller families (Leal, 2013; Benton 2015; Joseph & Carlson, 2015; Rimal & Lee, 2018). Arthropods vary enormously in the complexity of their chemical ecology, and their chemosensory gene repertoires covary with it. Extreme examples of this relationship are the most prominent. For example, the smallest chemosensory gene families are found in obligate parasites or mutualists with limited need for diverse chemical sensing ability, for example, the human body louse *Pediculus humanus* (Kirkness et al., 2010) and the fig wasp *Ceratosolen solmsi* (Xiao et al., 2013). In contrast, there are insects with complex chemically mediated social lives, like ants, with massive expansions of the OR family (Smith et al., 2011a, b; Zhou et al., 2012), many of which are now known to mediate perception of their highly diverse cuticular hydrocarbons (Pask et al., 2017), or highly polyphagous species like the moth *Spodoptera frugiperda* (Gouin et al., 2017) and the spider mite *Tetranychus urticae* (Ngoc et al., 2016), with massive expansions of the GR family. More subtle examples are also available, for example, the depauperate GR family in the honey bee *Apis mellifera* (Robertson & Wanner, 2006), thought to be commensurate with its mutualistic relationship with plants, and the considerable contraction of the OR and GR families of the tsetse fly *Glossina palpalis* (Obiero et al., 2014), commensurate with the reduced complexity of its chemical ecology compared to most flies. Finer‐grained examination of close relatives has revealed the on‐going processes of gene gain and loss that mediate these grander patterns, for example, in the monophagous *Drosophila sechellia* on the Seychelles islands (McBride, 2007; McBride & Arguello, 2007) or the unusual pestiferous *Drosophila suzukii* (Hickner et al., 2016). Genome sequences are nearly essential for discovering the size and complexity of these processes as these gene families commonly encode highly divergent proteins making them difficult to identify by screening methods, and they are commonly expressed at such low levels that transcriptome studies, even of chemosensory tissues such as antennae, palps and legs, will only detect some of them unless carried out at extreme sequencing depths, for example, the neurotranscriptome of the yellow fever mosquito *Aedes aegypti* (Matthews, McBride, DeGennaro, Despo, & Vosshall, 2016). Even then, there are gene family members not expressed in obvious chemosensory tissues, whereas pseudogenes are rarely transcribed and provide useful insights into gene family evolution (e.g., Smith et al., 2011a). The ongoing deluge of arthropod genome sequences promises to provide many more examples of the connection of chemosensory repertoire to chemical ecology, and one more extreme example is described here.

The German cockroach, *Blattella germanica* Linnaeus, is a widespread human commensal species (Schal, Gautier, & Bell, 1984; Schal, 2011), where it causes considerable problems beyond annoyance, including allergic responses leading to asthma (Gore & Schal, 2007; Rabito, Carlson, He, Werthmann, & Schal, 2017) and being a passive vector for potential pathogens. It is an extreme omnivore, feeding on almost any available foods, with a preference for “sweet” tastes (Schal et al., 1984). This biological preference was exploited in the development of insecticide baits that use sugars such as glucose (Schal & Hamilton, 1990). Resistance to these baits has evolved via the expected pathways of insecticide resistance (e.g., Gondhalekar & Scharf, 2012), but also remarkably by evolution of aversion to the sugar bait (Silverman & Bieman, 1993; Silverman & Ross, 1994). This aversiveness to glucose has been demonstrated to involve perception of glucose by the “bitter” neuron in each gustatory sensillum (Wada‐Katsumata, Silverman, & Schal, 2011, 2013). This switch might involve misexpression of a glucose receptor in these “bitter‐sensing” neurons or modified recognition of glucose by a receptor that normally senses a “bitter” compound. Thus, in addition to illuminating the chemosensory biology of this cockroach, documenting the major gene families encoding chemosensory proteins is a prerequisite to attempts to understand the molecular basis of this sugar aversiveness. Food preferences are also used in mate‐recognition and acceptance, as the courting male offers the female a nuptial gift in his tergal gland rich in sugars (maltose and other oligosaccharides) and phospholipids (Wada‐Katsumata, Ozaki, Yokohari, Nishikawa, & Nishida, 2009). This cockroach also uses chemoperception in other contexts, including long‐range mate‐finding with volatile sex pheromones (Nojima, Schal, Webster, Santangelo, & Roelofs, 2005), contact‐based sexual recognition with derivatives of cuticular hydrocarbons (Eliyahu, Nojima, Mori, & Schal, 2008), and aggregation (Wada‐Katsumata et al., 2015), so a complete documentation of its chemosensory genes and encoded proteins lays the ground for improved understanding of many aspects of the chemical ecology of *B. germanica*.

Three previous publications describe aspects of the chemosensory repertoire of this cockroach. Zhou et al. (2014) described partial sequences for 14 OBPs, two ORs, and four GRs from a whole body transcriptome conducted using pyrosequencing, whereas Niu, Liu, Dong, and Dong (2016) expanded the OBP total to 48 mostly complete sequences by performing an antennal transcriptome using ILLUMINA sequencing and also found five ORs and 5 IRs, albeit mostly partial sequences. The sequencing of the *B. germanica* genome as part of an i5k pilot project (Robinson et al., 2011; i5k Consortium 2013) allowed Harrison et al. (2018) to compare the repertoires of intact OR and IR genes of this cockroach with those of the dampwood termite *Zootermopsis nevadensis* (Terrapon et al., 2014) and two other termites they sequenced, revealing massive expansion of the IR family in this cockroach relative to the termites. Here we describe all four gene families in complete detail, including their many pseudogenes, and reveal that not only is the IR family massively expanded in this cockroach far beyond that known for any other arthropod, but the GR and OBP families are the largest known for insects, commensurate with the broad requirements of this cockroach to sense diverse chemicals in its environment.

## 2 MATERIALS AND METHODS

Searches for *B. germanica* chemosensory genes were conducted on the genome assembly of Harrison et al. (2018) using TBLASTN at the i5k Workspace (Poelchau et al., 2014) with proteins from the *Z. nevadensis* families and other insects (Terrapon et al., 2014) and E values up to 1000. Iterative searches with newly discovered genes and their encoded proteins were undertaken in an effort to exhaustively discover all gene family members. Gene models were built in the Apollo browser at the i5k Workspace, with supporting evidence from RNAseq data from three sources, all generated with ILLUMINA sequencing: the antennal set from Niu et al. (2016), a head set from Drinnenberg, Henikoff, and Malik (2014) available from the Short Read Archive (SRA) at NCBI as SRX682022, and a head set generated by A. W‐K. and C. S. (available in the SRA as SRX3189901/2). Partial gene models resulting from difficulties with the genome assembly were repaired when possible using a combination of the above RNAseq reads and raw genome reads from the SRA. Pseudogenes were translated as best possible, using Z for stop codons and X for frameshifts and other obvious pseudogenizing mutations like large insertions or deletions and splice junction mutants, but only named and included if they encoded at least half the length of a typical gene family member. Some pseudogenes were so badly degraded that despite being nearly full‐length they were not reconstructed and are not included in the protein sets, but were included in the pseudogene statistics. The same length criterion was employed for gene fragments that could not be repaired. Proteins from *B. germanica* and *Z. nevadensis* were aligned within each gene family, along with representatives from other insects, using ClustalX v2.1 (Larkin et al., 2007), and gene models were refined in light of these alignments. All protein sequences are available as Supporting Information, and the transcripts and protein sequences for all intact and contiguous models are available from the i5k Workspace.

For phylogenetic analyses, aligned protein datasets were trimmed using TrimAl v1.4 (Capella‐Gutierrez et al. 2009), using the “gappyout” option for the OR, GR, and OBP families, which are of reasonably uniform length, and the “strict” option for the IRs, which vary considerably in the length and sequence of their N‐termini, most of which was effectively removed from the alignment. Maximum likelihood phylogenetic analysis was performed with PhyML v3.0 (Guindon et al., 2010) using default settings with support for nodes evaluated using their approximate Likelihood Ratio Tests (aLRT). Trees were arranged and colored with FigTree v1.4.2 (https://tree.bio.ed.ac.uk/software/figtree/), and figures prepared in Adobe Illustrator.

Expression levels of the OBPs were compared between the antennal RNAseq of Niu et al. (2016) and our RNAseq from heads with antennae (SRX3189901/2). Trimmed reads were aligned to transcripts from our complete gene models, with 5′ and 3′ untranslated regions included, using the Burrows‐Wheeler Aligner (BWA) (Li & Durbin, 2009). Samtools (Li et al., 2009) was used to sort, index, and summarize the BWA. Read counts were standardized as counts per kb. These two libraries are of comparable size with 67,706,096 reads from Niu et al. (2016) and 77,726,077 reads from our heads‐with‐antennae RNAseq, so counts were not standardized by library depth as our comparisons are primarily within each dataset.

## 3 RESULTS AND DISCUSSION

### 3.1 The OR family

The OR family evolved from a lineage of the diverse GR family within insects (Robertson, Warr, & Carlson, 2003; Missbach et al., 2014; Ioannidis et al., 2017), with the wingless firebrat *Thermatobia domestica* expressing at least three members of the family in their antennae, but the slightly older lineage of the bristletail *Lepismachilis y‐signata* appearing not to have any ORs (Missbach et al., 2014). In all insects with a genome sequence examined to date the family consists of a single highly conserved gene encoding a coreceptor, known as Orco, as well as 4–400 “specific” ORs that mediate the specificity and sensitivity of insect olfaction (Leal, 2013; Benton 2015; Joseph & Carlson, 2015). The family ranges enormously in size in insects, from a low of five genes in the damselfly *Calopteryx splendens* (Ioannidis et al., 2017), which was previously thought to be anosmic, and 13 in the obligate parasitic human body louse *P. humanus* (Kirkness et al., 2010), through 60 genes encoding 62 receptors in *D. melanogaster* (Robertson et al., 2003), up to ∼400 in some ants (Smith et al., 2011a,b; Zhou et al., 2012), where up to half of them likely mediate perception of the enormously diverse cuticular hydrocarbons involved in nestmate recognition and other social cues (Smith et al., 2011a,b; Pask et al., 2017). The ligands of most of the *D. melanogaster* ORs are known (Hallem, Ho, & Carlson, 2004), as are those from *Anopheles gambiae* (Carey, Wang, Su, Zwiebel, & Carlson, 2010; Wang et al. 2010), and some others in other insects, for example, pheromone receptors in diverse species (Wanner et al., 2007; Leary et al., 2012; Andersson et al., 2016).

The OR family in *B. germanica* is of an intermediate size, with 134 potential transcripts from 123 genes in addition to the Orco gene. Five genes exhibit an unusual form of alternative splicing previously described in this and the GR family in many insects including *D. melanogaster* (Clyne, Warr, & Carlson, 2000; Robertson et al., 2003), in which tandemly arrayed long first exons are alternatively spliced into a shared set of exons encoding the C‐terminus of the protein, the most conserved region of the protein. Twenty‐nine of these genes or transcripts are pseudogenic (21.6%), leaving 105 apparently intact specific ORs. Niu et al. (2016) described only five ORs from their antennal transcriptome, and all are partial sequences, so the family here was named afresh, attempting to give related receptors, especially in arrays within scaffolds, consecutive names (their OR1 is Orco, Or2 is Or49, Or3 is Or33, Or4 is Or100, and Or5 is Or48).

**Figure 1:**
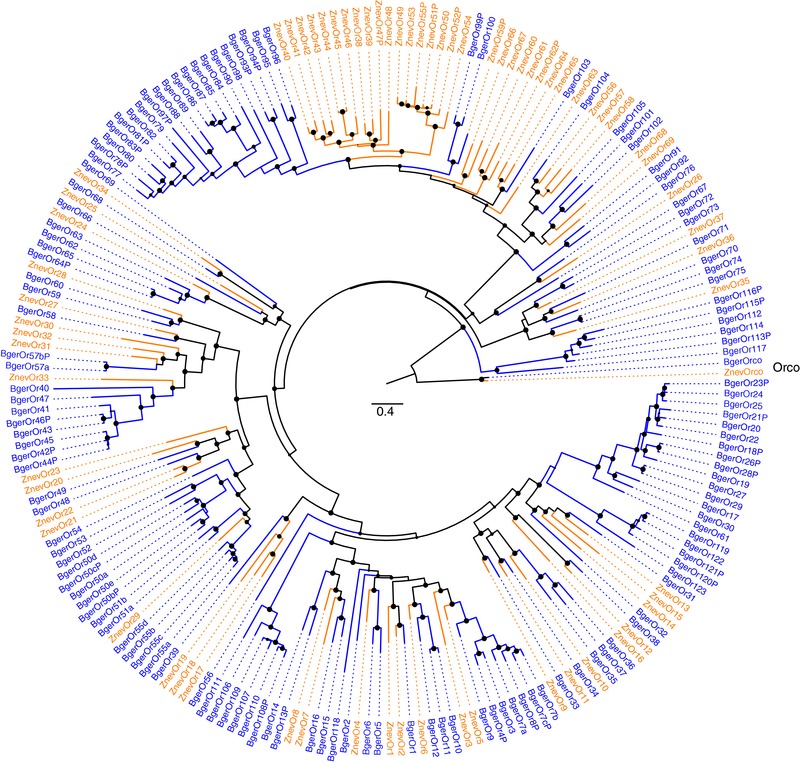
Phylogenetic relationships of the OR family. The tree was rooted with the Orco proteins, based on the conserved sequence and basal position of this protein within the OR family in analyses of the entire insect chemoreceptor superfamily (Robertson et al., 2003). *B. germanica* proteins are in blue and *Z. nevadensis* in orange, as are the branches leading to them. The suffix P after a name indicates that it is pseudogenic. The scale bar is substitutions per site, and filled circles on nodes indicate support levels as aLRT (approximate Likelihood Ratio Test) values from PHYML v3.0 ranging from 0 to 1 [Color figure can be viewed at http://wileyonlinelibrary.com]

Phylogenetic analysis of these OR proteins with their 70 relatives from *Z. nevadensis* (Terrapon et al., 2014), reveals the kinds of gene family evolution well known for this family (e.g., Benton, 2015) (Figure 1). Beyond the highly conserved Orco lineage, there are many instances of apparent 1:1 orthologs between the cockroach and termite, as expected from their close phylogenetic relationship, as well as many instances of gene losses and duplications in one or both lineages. The most extreme examples of the latter are expansions of 18 proteins in the termite and 15 and 20 proteins in the cockroach. Most pseudogenes are found in these and other smaller expansions, suggesting loss of gene function as these receptors sometimes became irrelevant to the species chemical ecology. The overall larger size of the cockroach OR repertoire is a combination of fewer gene losses and more duplications, many of which have short branches in the tree indicating that they are the result of recent duplications in the cockroach.

### 3.2 The GR family

The GR family consists of multiple divergent subfamilies (Clyne et al., 2000; Robertson et al., 2003) and dates back at least to the earliest animals where they are called GR‐Like genes (Benton 2015; Robertson, 2015; Saina et al., 2015; Eyun et al., 2017). It varies in size from a low of five genes in the obligate mutualist fig wasp *C. solmsi* (Xiao et al., 2013) and 12 in the honey bee *A. mellifera* (Robertson & Wanner, 2006), through 60 genes encoding 68 receptors in *D. melanogaster* (Clyne et al., 2000; Scott et al., 2001; Robertson et al., 2003), to 222 and 286 in the red flour beetle *Tribolium castaneum* (Richards et al., 2008) and the Asian longhorned beetle *Anoplophora glabripennis* (McKenna et al., 2016), respectively. The sugar receptors are the oldest distinctive recognizable subfamily of known function, dating back to crustaceans (Peñalva‐Arana, Lynch, & Robertson, 2009), whereas the distinctive subfamily that contains the carbon dioxide receptors of endopterygotes dates at least to the odonates (Iaonnidis et al. 2017). In *D. melanogaster* most of the remaining GRs are involved in perception of bitter tastants (Weiss, Dahanukar, Kwon, Banerjee, & Carlson, 2011), however some are implicated in perception of pheromones (Bray & Amrein, 2003; Watanabe, Toba, Koganezawa, & Yamamoto, 2011), Gr43a (a fructose receptor) and other GRs are involved in both peripheral detection and central monitoring of tastants (Miyamoto, Slone, Song, & Amrein, 2012; Chen & Dahanukar, 2017), and others are expressed in non‐chemosensory tissues (Thorne & Amrein, 2008) and Gr28b senses light and heat (Ni et al., 2013).

**Figure 2:**
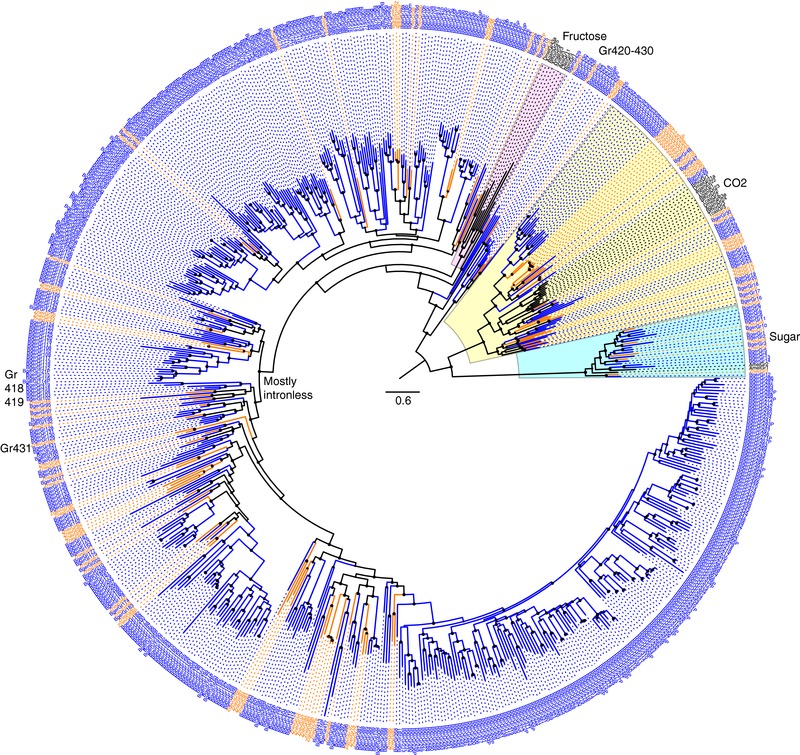
Phylogenetic relationships of the GR family. The sugar and carbon dioxide receptor subfamilies together rooted the tree, based on their basal location together in analyses with GRLs of other animals (Robertson, 2015). Major subfamilies are highlighted in colors, and the branch leading to the mostly intronless clade is indicated inside the circle. Non‐blattodean insect GRs are in black. Other details are as in the Figure 1 legend [Color figure can be viewed at http://wileyonlinelibrary.com]

*B. germanica* has the largest known GR family in insects, with 431 genes potentially encoding 545 proteins through alternative splicing of 26 genes, although 62 (11.4%) of these genes or transcripts are pseudogenic, leaving a potentially intact set of 483 GRs. Only the extremely polyphagous spider mite *T. urticae* has more GRs with 689 (Ngoc et al., 2016). The sugar receptor subfamily consists of 14 genes (BgerGr1–14), compared with six in the termite, through both loss of genes from the termite and duplications in the cockroach (Figure 2). The subfamily that contains the carbon dioxide receptors of endoterygotes is also larger in the cockroach, with 36 genes (Bger15–50) including a cockroach‐specific expansion of 16 genes, compared with 25 in the termite. A complex mixture of orthologs, gene losses, and species‐specific duplications has been involved in this subfamily. Although these proteins cluster confidently with the carbon dioxide receptors of endopterygote insects, their ligands remain to be determined and are unlikely all to be related to sensing of carbon dioxide.

**Figure 3:**
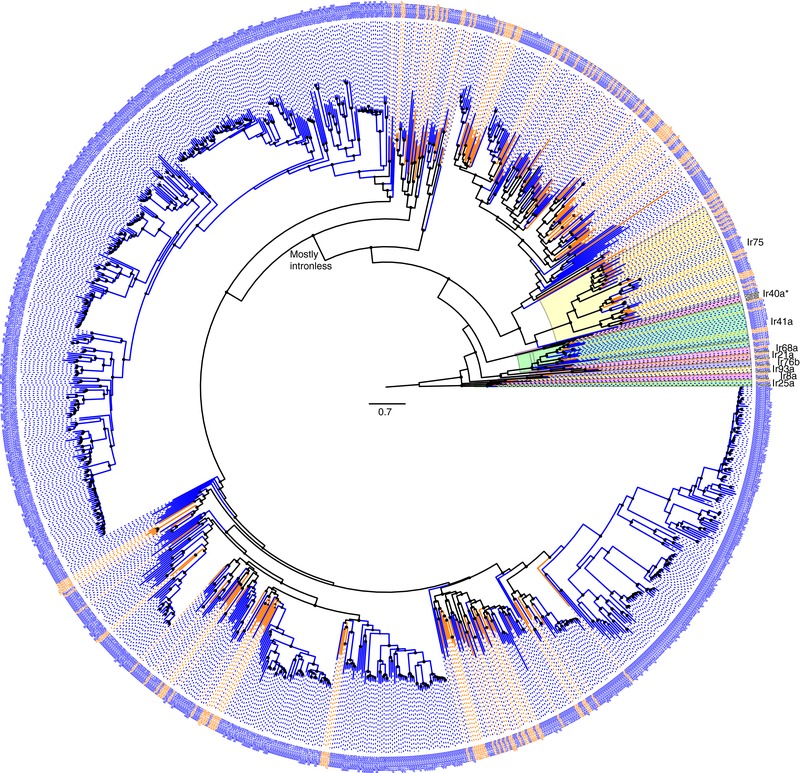
Phylogenetic relationships of the IR family. The Ir8a and 25a lineages were declared the outgroup as these two proteins are most similar to the ionotropic glutamate receptors (Croset et al., 2010; Terrapon et al., 2014). Major conserved lineages are highlighted in colors, and their names indicated on the outside of the circle (the asterisk on Ir40a indicates it is missing from both species). The branch leading to the mostly intronless clade is indicated inside the circle. Other details are as in Figures 1 and 2 legends [Color figure can be viewed at http://wileyonlinelibrary.com]

The remaining GRs fall broadly into two large groupings, as do the termite ones. The first is a set of genes that contain three phase‐0 introns near the 3′ end, in positions compatible with being homologous with the three introns inferred to be ancestral to the insect chemoreceptor superfamily (Robertson et al., 2003) and present throughout the GRLs in other animals (Robertson 2015; Saina et al., 2015). These genes are Gr51–94 and 418–430. Gr420–430 form basal clusters nearer the root of the tree (indicated in Figure 3), and Gr429 and 430 have acquired three additional introns interrupting the usually long first exon, so they have introns in phases 1‐0‐1‐0‐0‐0. Four relatives of the latter set were not previously recognized in the termite, so are newly described here, specifically ZnevGr88/89 are related to BgerGr424/425, whereas ZnevGr90 and 91 are related to BgerGr426 and 429, respectively. Enigmatically, Gr418/419 cluster in the tree with the intronless genes below. These intron‐containing genes are commonly alternatively spliced in a fashion similar to some of the OR genes, with multiple long first exons alternatively spliced into the three shared short final exons, commonly with RNAseq support for at least some of these alternative splices. The largest of these is Gr60a‐m encoding 13 distinct receptors, all apparently intact, whereas some isoforms encoded by other genes in this set are pseudogenic. Comparison of the Gr51–94 and 418–430 set with their termite relatives reveals major expansions of several gene lineages, for a total of 171 potential proteins versus just 18 in the termite. Again, although there are a few apparent orthologous relationships, the remaining relationships involve minor expansions in each species, apparent loss from the termite, and large expansions through alternative splicing in the cockroach.

The second and larger GR set are intronless genes, at least as far as the coding sequences are concerned (for some genes there is RNAseq evidence of an intron in the 5′ untranslated region), comprising a single lineage in the tree (indicated in Figure 2). This intronless clade consists of 44 termite and 322 cockroach genes (Gr95–417). Again, although there are nine apparent orthologous relationships, many other relationships involve duplications in one or both species, there are many apparent gene losses from the termite and very few from the cockroach, and most impressively, there is a massive cockroach‐specific expansion of 166 genes (Gr213–379). By comparison with other insects such as *D. melanogaster*, these two large clades of GRs are likely to be involved in perception of “bitter” compounds.

Finally, a single divergent gene named Gr431 was discovered while searching for relatives of the DmelGr43a fructose receptor lineage. This receptor lineage evolved from within the “bitter” clade of GRs (as opposed to being a divergent member of the sugar receptor subfamily), and is present in all neopteran insects examined to date as at least one gene and sometimes an expanded lineage, except for the termite *Z. nevadensis* (Terrapon et al., 2014). The damselfly *C. splendens* has a set of five proteins that might be related to this lineage, however the association is not robust (Ioannidis et al., 2017). Gr431 has a completely different gene structure than all the others, with introns in phases 0‐2‐0‐1, none of which correspond to any other GR introns in this species or the fructose receptor homologs in other insects, splitting the CDS into five roughly equal‐size exons. The termite has a previously unrecognized ortholog of this gene (ZnevGr92P) with the same gene structure, but it is a pseudogene with a stop codon in the middle of the fourth exon, whose existence is supported by both raw genome reads and some expressed sequence tags, although it is always possible this is a pseudogenic allele specific to the sequenced strain. These two proteins cluster phylogenetically well within the intronless clade (indicated in Figure 2), suggesting that all four of these introns are novel gains in this gene before the cockroach/termite split. This GR is nevertheless unlikely to be a fructose receptor as that clade is quite distinct in the phylogenetic tree.

### 3.3 The IR family

The IR family is a variant lineage of the ionotropic glutamate receptor superfamily of ligand‐gated ion channels (Benton et al. 2009; Rytz, Croset, & Benton, 2013; Rimal & Lee, 2018), present throughout the protostomes (Croset et al., 2010; Eyun et al., 2017). Like the other families, IRs are best understood in *D. melanogaster* where they have been shown to mediate a distinct set of olfactory capabilities, especially involving acids and amines (Ai et al., 2010; Silbering et al., 2011; Rytz et al., 2013; Ahn, Chen, & Amrein, 2017; Chen & Amrein, 2017), as well as perception of temperature and humidity (Knecht et al., 2016, 2017; Ni et al., 2016), whereas the large Ir20a clade are involved in gustation (Koh et al., 2014; Stewart, Koh, Ghosh, & Carlson, 2015). The family has three coreceptors, Ir8a, 25a, and 76b, involved in different sensory aspects, as well as various conserved lineages dating back to early insects (Ioannidis et al., 2017). The family ranges from 14 and 19 genes in the pea aphid *Acythosiphon pisum* and *P. humanus*, respectively (Croset et al., 2010; Terrapon et al., 2014) and similarly tens of genes in most Hymenoptera (Croset et al., 2010), through 62 genes in *D. melanogaster* (Benton et al. 2009), to 150 genes in the termite *Z. nevadensis* (Terrapon et al., 2014).

*B. germanica* has far and away the largest IR family known in any arthropod, with 897 genes, 393 (43.8%) of which are pseudogenes, leaving 604 intact genes. In addition there are many gene fragments encoding less than 50% of a related protein, as well as pseudogenes so badly damaged they could not easily be reconstructed and hence were not included in the naming or analysis. The naming of IRs is complicated. In their survey of IRs across various insects, Croset et al. (2010) gave clear relatives of the *D. melanogaster* proteins the same names and for the rest assigned sequential numbers to each IR independent of species, but this approach is not tenable in the long run. Terrapon et al. (2014) started an alternative approach of naming the remaining IRs in a series beginning with Ir101, which avoids confusion with the *D. melanogaster* names as they were named for their cytological locations and hence only go up to 100a. This approach has now been employed for several species, including the predatory mite *Metaseiulus occidentalis* (Hoy et al., 2016), the damselfly *C. splendens* (Ioannidis et al., 2017), the milkweed bug *Oncopeltus fasciatus* (Panfilio et al., 2017), the Asian longhorned beetle *A. glabripennis* (McKenna et al., 2016), the Colorado potato beetle *Leptinotarsa decemlineata* (Schoville et al., 2017), the tick *Ixodes scapularis* (Josek et al. 2018), and updated *Ae. aegypti* and *An. gambiae* genomes (Matthews et al. 2018). Thus the single orthologs of Ir8a, 21a, 25a, 68a, 76b, and 93a are given those names, and as in the termite, the expanded set of genes related to DmelIr41a are named Ir41a1–16, whereas the expanded set related to DmelIr75a–d are named Ir75a–z. Both the termite and cockroach do not have an obvious ortholog for the Ir40a gene, which is present in the odonate (Iaonnidis et al. 2017). Niu et al. (2016) identified five IRs in their antennal transcriptome, and like the ORs most are partial sequences, however their Ir4 is full length. Their IRs correspond with those described here as follows: their Ir1 is Ir41a11, Ir2 is Ir41a15, Ir3 is Ir41a12, Ir4 is Ir25a, and Ir5 is Ir76b.

Phylogenetic analysis of the IR family reveals the simple orthologous relationships of the conserved IRs, the absence of an Ir40a ortholog, and the expansions of the Ir41a and Ir75 lineages in the cockroach, once again involving some orthologs, gene losses primarily from the termite lineage, and duplications in the cockroach (Figure 3). The remaining IRs, much like the GRs, form two large groupings. The first set of genes generally contains eight introns comparable to those seen in these IRs in many other insects. In the cockroach, these are BgerIr101–196 and in the termite ZnevIr101–155. This set of genes again contains some potentially orthologous relationships, as well as the usual losses primarily from the termite, and duplications in one or both species, the largest of which are eight genes, BgerIr152–159, related to ZnevIr132 and 14 genes, BgerIr163–176, related to ZnevIr137/138.

The truly massive IR expansions in the cockroach, however, have occurred within a clade of largely intronless genes. Some of these genes have acquired idiosyncratic single, and rarely two, introns, commonly near their 5′ end and barely interrupting the N‐terminal coding region. It is possible that some of these were introns in 5′ untranslated regions that have now become coding. This massively expanded clade is BgerIr196–950 (755 genes) and ZnevIr156–222 (66 genes). As usual it contains a few apparent orthologous relationships, many gene losses from the termite and a few from the cockroach, and most impressively massive cockroach‐specific expansions. In stark contrast to the termite, where the largest expansion is just five genes, in the cockroach expansions include 17 genes (Ir226–239) related to ZnevIr163, 46 genes (Ir702–747) with no clear termite relative, 61 genes (Ir592–653) related to ZnevIr190–193, 147 genes (Ir751–870 and 924–950) related to ZnevIr213, and 347 genes (Ir262–567, 748–750, and 884–923) without a clear termite relative.

### 3.4 The OBP family

OBPs are small globular secreted proteins, which in the context of insect chemoreception are secreted into the lumen of sensilla from support cells at their base (Leal, 2013; Pelosi, Iovinella, Felicioli, & Dani, 2014). Not all OBPs are involved in chemoperception, however, with some expressed in other cells and tissues (e.g. Foret and Maleszka 2006; Pelosi et al., 2014). The Classic structure for an OBP is to have six highly‐conserved cysteines that maintain their tertiary shape via three disulfide bonds, however there are variants with four cysteines called Minus‐C, and ones with two additional cysteines, called Plus‐C, as well as apparent OBP dimers (Pelosi et al., 2014). The family ranges considerably in size up to 90 genes in the parasitoid wasp *Nasonia vitripennis* (Vieira et al., 2012), and appears to be largely an insect‐specific invention (Missbach, Vogel, Hansson, & Groβe‐Wilde, 2015), extending back to basal hexapods like Collembola (Pelosi et al., 2014), although similar if not homologous proteins are known from other arthropods (e.g., Renthal et al., 2017; Vizueta et al. 2017; Josek et al. 2018).

Niu et al. (2016) described 48 mostly full‐length OBPs from their antennal transcriptome of *B. germanica*, along with extensive data on their expression patterns. Those 48 names were therefore retained, whereas gene models were completed for their partial ones, and 61 models were added for a total of 109 genes, all of which are intact (OBP103 is a pseudogene in the genome assembly with a single‐base‐insertion frameshift in a homopolymer region of exon7, but examination of raw genome and RNAseq reads indicates it is polymorphic with an intact version, which was employed instead). Eleven additional models required repair of the genome assembly and all models are now full length. This is the largest known OBP family. Because Niu et al. (2016) described and named their proteins from a transcriptome, the genes encoding them have no logical order in the genome. The new ones were also added in no particular order, so unlike the chemoreceptor families, consecutive gene names have no meaning for this OBP family, including in a large array of 61 genes spanning approximately 1.6 Mb across four scaffolds (scaffolds1250, 997, 2545, and 116). The newly added OBPs mostly have RNAseq support from head RNAseq and less from the antennal RNAseq of Niu et al. (2016) (Supporting Information Figure 1), largely explaining why they were not included in their compilation. These OBPs might be expressed in gustatory rather than olfactory sensilla, whereas others might not be involved in chemoperception at all. Like OBP genes in other insects, most of these have a phase‐0 intron separating the first exon encoding the signal sequence from the rest of the gene, which typically consists of 7–9 short coding exons commonly spanning several kilobase pairs.

**Figure 4:**
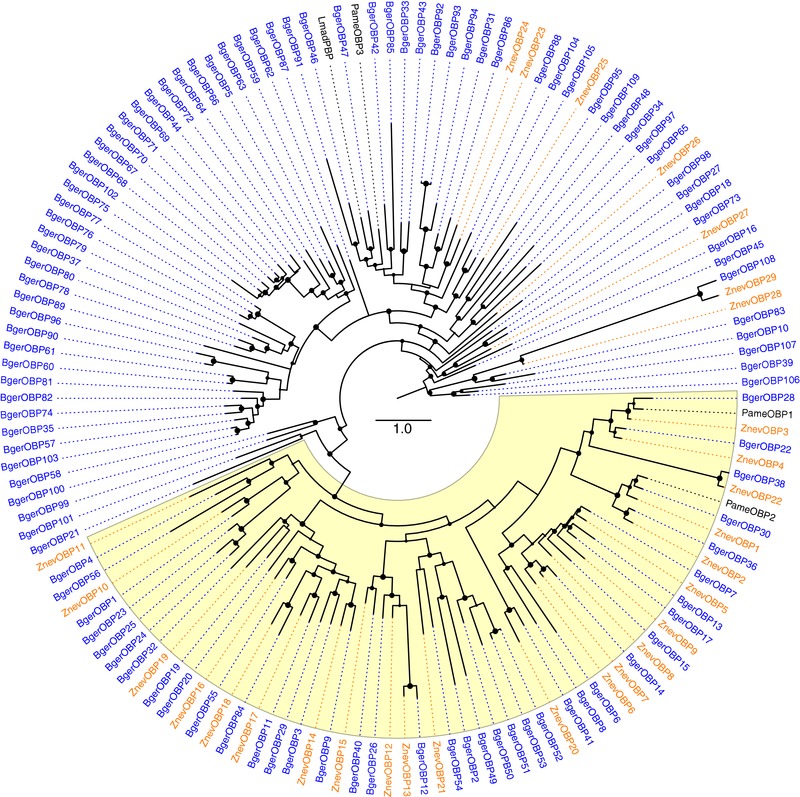
Phylogenetic relationships of the OBP family. In the absence of an established outgroup, the tree was rooted at the midpoint. The Classic and Minus‐C OBPs are highlighted in yellow, the remaining OBPs being Plus‐C and a distinctive clade. Other details are as in Figures 1 and 2 legends, except that here four additional cockroach OBPs from other species are in black [Color figure can be viewed at http://wileyonlinelibrary.com]

Phylogenetic analysis, along with the 29 OBPs from *Z. nevadensis* (Terrapon et al., 2014), three from *P. americana* (Li, He, Zhang, & Dong, 2017), and a pheromone‐binding OBP from *Leucophaea* ( = *Rhyparobia*) *maderae* (Riviere et al. 2003) reveals that there is a major clade of 71 genes, most of which are in the large 61‐gene array in the cockroach, including a 33‐gene cockroach‐specific expansion. These are all Plus‐C OBPs, while the Classic and Minus‐C OBPs cluster together (highlighted in yellow in Figure 4). Once again, several gene losses from the termite and almost no termite‐specific expansions are evident from the tree. The cockroach genes that cluster with this 71‐gene clade, but are not in the 61‐gene array, are OBP27 which is a singleton in another large scaffold, OBP85 in another large scaffold with other OBPs, OBP33 and 43 in yet another large separate scaffold along with other OBPs, and OBP39 and 104–107 in yet another large scaffold (OBP108 is discussed below). These genes have presumably relocated from the large array, as they clearly belong evolutionarily with it. Five of the six ZnevOBPs that cluster with this cockroach OBP expansion in the tree are also in an array spanning 100 kb across two scaffolds (ZnevOBP23–28 in scaffolds1046 and 631), so this expansion is an old one, and there are relatives in other insects not shown in Figure 4. ZnevOBP29, and its cockroach ortholog BgerOBP108, are not only elsewhere in their respective genomes but are far longer than the average OBP at 331 and 324 amino acids, respectively, with a long section of simple sequence between the N‐terminal secretion signal sequence and the OBP‐homologous C‐terminus. Finally, OBP109 is an even more unusual gene encoding a 596 amino acid protein, the C‐terminus of which has homology to OBPs, but the N‐terminus has no similarity to other proteins in the nonredundant protein database at NCBI, and the termite ortholog appears to have been lost. Like ZnevOBP29 and BgerOBP108, this gene model is deeply supported by RNAseq from antennae and heads, so its unusual length is real. OBP109 is at one end of the 61‐gene array and appropriately clusters phylogenetically with the others from the array.

### 3.5 Chemoreceptor pseudogenes

**Figure 5:**
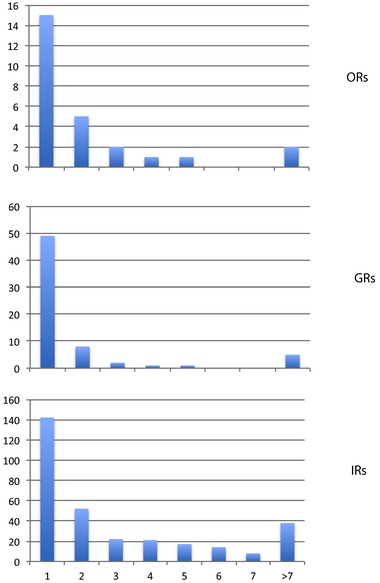
Histograms of numbers of pseudogenizing mutations in the three chemoreceptor families. The *x*‐axis is the number of pseudogenizing mutations and *y*‐axes are the number of pseudogenes in each category [Color figure can be viewed at http://wileyonlinelibrary.com]

The proportion of pseudogenes in the three chemoreceptor families is fairly high (21.6%, 11.4%, and 43.8% for the OR, GR, and IR families). These large numbers present an opportunity to examine them in more detail, specifically by counting the numbers of obvious pseudogenizing mutations in each gene. This approach previously revealed an excess of “middle‐aged” pseudogenes in the OR and GR families of the red harvester ant *Pogonomyrmex barbatus*, something not seen in the honey bee *A. mellifera*, suggesting that it had undergone a major shift in its chemical ecology in the distant past (Smith et al., 2011a). As noted in the methods, in addition to the many pseudogenes named and translated for analysis herein, there were some, especially in the IR family, that despite being near full length were not formally included in the families as they were too damaged to be easily reconstructed. For the purpose of examining the pseudogenes, these were counted as having more than seven pseudogenizing mutations, thus the numbers in the histograms are somewhat higher than the numbers given in the family descriptions above. The distribution of pseudogenes is clearly dominated by those with single mutations, with a clear reduction in numbers of pseudogenes with more mutations (Figure 5). This is the pattern to be expected if older pseudogenes are removed from the genome by deletions, although at 2.2 Gbp, this genome is not that small and hence pseudogenes are probably not removed as quickly as in smaller genomes like that of *D. melanogaster*, but would be removed more quickly than from a large orthopteran genome (Petrov, Sangster, Johnston, Hartl, & Shaw, 2000).

## 4 GENERAL DISCUSSION

The connection between complexity of chemosensory repertoire and chemical ecology of an arthropod species is now clear, as it is for vertebrates (Niimura, 2012), and this example of the German cockroach, and its comparison with the dampwood termite, demonstrates it abundantly. Termites evolved from cockroaches, and comparison of their genomes has revealed many genomic processes that parallel those of the independent evolution of sociality in Hymenoptera (Harrison et al., 2018). Termites have lost many chemosensory genes that their cockroach ancestors had, presumably because they were no longer necessary for their more specialized ecological niche. In addition, they have only expanded a few chemoreceptor lineages, in particular one of 18 genes in the OR family, which might be involved in their social behavior. In radical contrast, *B. germanica* has lost only a few gene lineages that once were present in the common ancestor with the termite, and has expanded many lineages in dramatic fashion, most spectacularly in the GR and IR families. These major expansions, many of them fairly young as indicated by the short terminal branches to many proteins in the phylogenetic analyses, along with reasonably high percentages of pseudogenes, especially in the IR family, suggests that these chemoreceptor gene families continue to undergo rampant evolution in this cockroach lineage.

Blattodeans are evolutionarily distant from the dipteran and other endopterygotan species for which evidence of ligand specificity is available for at least some chemoreceptors. Hence, it is only possible to make general inferences about the roles of most of these proteins. In the OR family, the well‐conserved Orco protein is present as a single ortholog, as is the case in almost all insects examined to date (the three named Orco proteins in the basal zygentoman *T. domestica* described by Missbach et al. (2014) are likely actually a single Orco and two “specific” ORs (Iaonnidis et al. 2017)). The remaining 123 cockroach ORs are therefore likely “specific” receptors that function as dimers with Orco, but their divergence from all endopterygotan ORs of known ligand specificity precludes any speculation about their functions.

The GR family consists of several subfamilies, the most distinctive and ancient of which is the sugar receptors, here with 14 genes. Although the ligands of these proteins are almost certain to be sugars, the precise ligand specificities of sugar receptors remain unclear even in *D. melanogaster* (e.g., Fujii et al., 2015). In any case, the sugar GRs in this cockroach do not have simple orthologous relationships with those of the flies, having mostly expanded within this lineage in two gene clusters of five and six genes in different large scaffolds, as well as a singleton and pair of genes in other scaffolds. Basal insects have an expanded GR subfamily to which the carbon dioxide receptors of endopterygotes belong, here consisting of 36 genes, however it seems unlikely that all of these are involved in detecting this gas, but rather represent the subfamily from which the carbon dioxide receptors evolved. The remaining GRs in most insects appear to function in perception of “bitter” chemicals, although some in *D. melanogaster* are pheromone and light sensors. These comprise the vast majority of the expanded GR family in this cockroach, including an intronless clade of 322 genes, and are inferred to primarily sense a wide diversity of chemicals in their diverse foods. Somewhat surprisingly, the otherwise well‐conserved fructose receptor lineage related to Gr43a in *D. melanogaster* is absent from both these blattodean genomes. This lineage was also not confidently identified in the odonate *C. splendens* (Iaonnidis et al. 2017) so it is possible that it originated from a “bitter” receptor at some point later in insect evolution, although its role in brain nutrient sensing would seem to be an ancient and essential role (Miyamoto et al., 2012).

The IR family has several conserved members whose functions can be assigned with some confidence by comparison with *D. melanogaster*, and are named for their fly orthologs. The Ir8a, 25a, and 76b proteins are coreceptors with other IRs, and involved in diverse aspects of IR function. The Ir21a, 68a, and 93a proteins have recently been demonstrated to mediate perception of temperature and humidity in *D. melanogaster* (Knecht et al., 2016, 2017; Ni et al., 2016), although these blattodeans have lost the fourth gene involved, Ir40a, as it is present in *C. splendens* (Iaonnidis et al. 2017). The cockroach has considerably expanded the Ir41a lineage to 16 intact genes. DmIr41a and 76a are close relatives and cluster with Ir92a, and Ir92a and Ir41a are involved in olfactory perception of amines, in cooperation with Ir76b, at least in the case of Ir41a (Min, Ai, Shin, & Suh, 2013; Hussain et al., 2016). Interestingly Niu et al. (2016), in addition to detecting the coreceptors Ir25a and 76b in their antennal transcriptome, identified three of these Ir41a lineage genes, suggesting that like in *Drosophila* they are olfactory receptors that partner with Ir76b to detect amines. The Ir75 subfamily in *Drosophila* consists of the Ir75a–d, 31a, 64a, and 84a proteins, which along with Ir8a are involved in perception of various acids (Ai et al., 2010, 2013; Grosjean et al., 2011; Gorter et al., 2016; Prieto‐Godino et al., 2016; 2017). This subfamily is considerably expanded in this cockroach to 26 genes, all but two intact, which can reasonably be inferred to also partner with Ir8a to sense a diversity of acids. The remaining 850 BgIrs, especially the 755 intronless Ir genes, are likely to encode gustatory receptors, by analogy with the large Ir20a clade of *D. melanogaster*, which are expressed in gustatory tissues (Koh et al., 2014; Stewart et al., 2015), and like the GR expansions presumably sense various chemicals in foods.

Finally, the OBP family is the largest known at 109 genes. This large number, many of which are expressed at low levels in antennae, is appropriate given the huge expansion of GRs and IRs, and because many of these nonantennal OBPs are expressed in head RNAseq where they might serve in gustatory sensilla in support of the numerous gustatory receptors. Li et al. (2017) described three OBPs from *P. americana*, and the first two are close relatives of BgerOBP28 and 30, respectively, and are also expressed in antennae and bind a variety of relevant odorants, so similar roles can be ascribed to these and probably many other of the 48 OBPs Niu et al. (2016) described. Their third OBP is a Plus‐C subfamily member, and Li et al. (2017) did not describe a close *B. germanica* relative, but it is related to the 71‐gene expansion in *Blattella*, and it is widely expressed in *P. americana* and did not bind any of 87 chemicals tested. Thus, it and some of the *B. germanica* relatives might not be involved in chemosensation. It is, however, also related to the Pheromone Binding Protein of another cockroach, *Leucophaea maderae* (Riviere et al. 2003). The biological roles of OBPs remain unclear as their assumed role of transporting commonly hydrophobic odorants and tastants through the sensillar lymph to the membrane‐bound receptors (Leal, 2013; Pelosi et al., 2014) has been challenged by the first direct mutational evidence for the physiological role of an OBP in *D. melanogaster*, where DmOBP28a is not involved in odorant transport, but rather in buffering the responses of the relevant olfactory sensory neurons (Larter, Sun, & Carlson, 2016).

In summary, *B. germanica* has the largest chemosensory repertoire known for any arthropod, including the second‐largest GR family, the largest OBP family, and by far the largest IR family. It therefore supports the contention that chemosensory repertoires evolve in concert with the complexity, as well as presumably the details, of the chemical ecology of the species. Complete documentation of these four large gene families will facilitate future work towards identifying the roles of some of these proteins in various aspects of cockroach biology, from their sex and aggregation pheromones to the remarkable evolution of sugar‐aversive strains.

## CONFLICT OF INTEREST

None.

## Supporting information

Supporting Information

Phylogenetic relationships of the GR family. The sugar and carbon dioxide receptor subfamilies together rooted the tree, based on their basal location together in analyses with GRLs of other animals (Robertson, 2015). Major subfamilies are highlighted in colors, and the branch leading to the mostly intronless clade is indicated inside the circle. Non‐blattodean insect GRs are in black. Other details are as in the Figure 1 legend.

Phylogenetic relationships of the IR family. The Ir8a and 25a lineages were declared the outgroup as these two proteins are most similar to the ionotropic glutamate receptors (Croset et al., 2010; Terrapon et al., 2014). Major conserved lineages are highlighted in colors, and their names indicated on the outside of the circle (the asterisk on Ir40a indicates it is missing from both species). The branch leading to the mostly intronless clade is indicated inside the circle. Other details are as in Figures 1 and 2 legends.
