# Supplementary material for: Enormous expansion of the chemosensory gene repertoire in the omnivorous German cockroach Blattella germanica
Source: J Exp Zool B Mol Dev Evol. 2018 Mar 22;330(5):265–78. doi: 10.1002/jez.b.22797 (PMC6175461; doi:10.1002/jez.b.22797)
Supplement: Supplementary file 1 — Supporting Information [file JEZ-330-265-s001.pdf]

Expression levels of the *B. germanica* OBPs.

Figure S1 shows RNAseq read counts from the antennal library of Niu et al. (2016) (blue) compared with those from our RNAseq from a heads-with-antennae library (orange) of comparable size. Figure S1A is OBP1-48, that is, the OBPs described by Niu et al. (2016) from their antennal transcriptome, while Figure S1B is OBP49-109, that is, the new OBPs we describe. The y-axis is logarithmic to accommodate the extreme variation in expression values, which range from 0 for OBP96 to 356624 for OBP33, both from the antennal library. Note that the y-axis for Figure S1A is to 1 million, while that for Figure S1B is only to 100,000. Amongst the Niu et al. (2016) set of OBP1-48, almost all are reasonably well expressed in antennae, with more than 100 reads for all but OBP43, which makes sense as they were able to identify mostly full-length transcripts for these OBPs from their antennal transcriptome. Comparison with our heads-with-antennae RNAseq library, which has slightly more reads, reveals that 31 of these 48 have higher expression in the antennal RNAseq (in part presumably because antennal OBPs would be diluted in the heads-with-antennae library). Of the remainder, only a few are dramatically more highly expressed in our heads-with-antennae RNAseq, most obviously OBP3, indicating that they are expressed in additional sensilla on the head. The expression levels for OBP49-109, those newly identified herein, reveal that while a few are highly expressed in antennae, none have more than 10,000 reads (19 of the OBP1-48 set do), and only about half (31) have more than 100 reads. It is nevertheless somewhat surprising that these OBPs that are reasonably well expressed in antennae were not recognized in the transcriptome of Niu et al. (2016). Of these 61 genes, 38 are more highly expressed in our heads-with-antennae RNAseq, indicating that some of them might primarily be expressed in gustatory rather than olfactory sensilla.

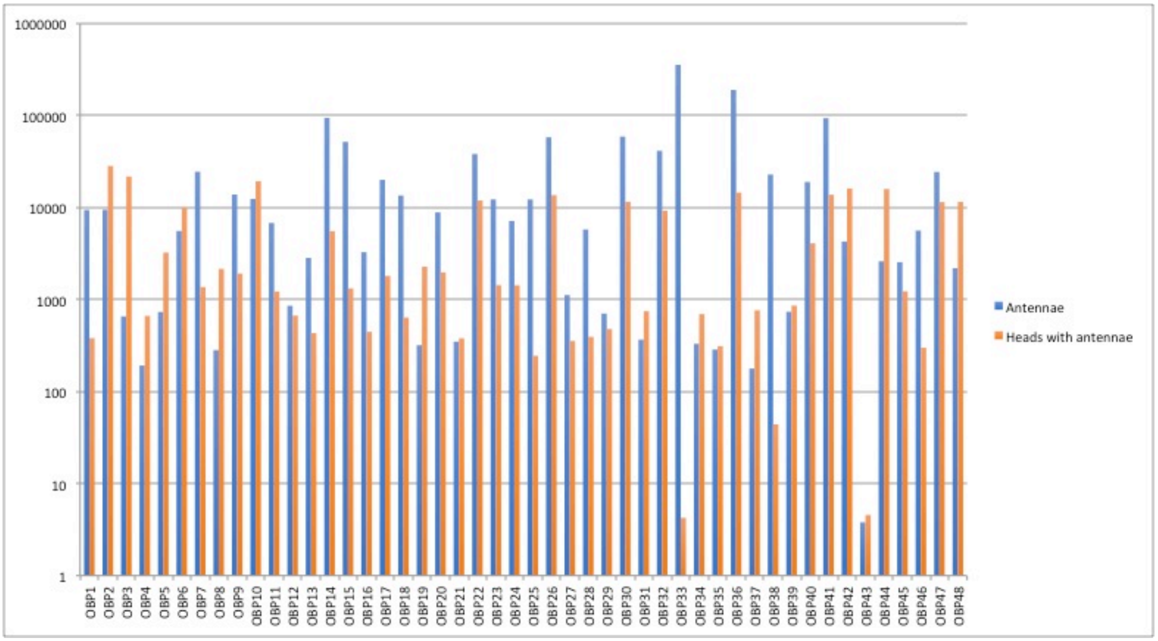

Figure S1A. RNAseq read counts per kb for OBP1-48 from Niu et al. (2016) from their antennal library and our heads-with-antennae library.

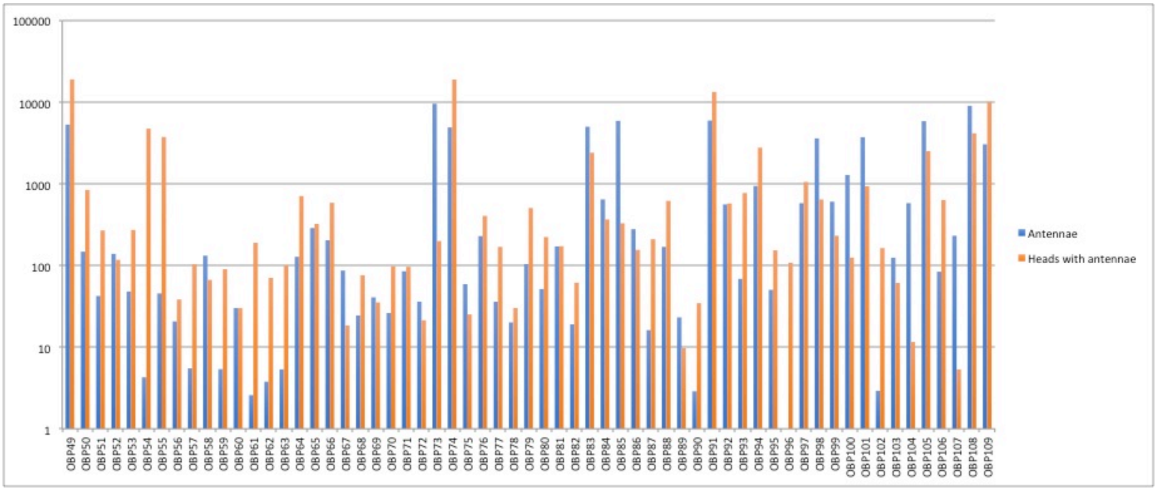

Figure S1B. RNAseq read counts per kb for OBP49-109 newly-described herein from their antennal library and our heads-with-antennae library.

All *B. germanica* protein sequences employed herein, and five newly recognized ZnevGrs. Lower case letters identify alternatively-spliced isoforms from the same gene for the ORs and GRs. Suffixes after names are: C – C-terminal region missing; F – assembly repaired; I – internal region missing; J – model spans two scaffolds; N – N-terminal region missing; P – pseudogene.

134 BgerOR proteins

>BgerOrCo
MYKFRLHGLVADMWPLIRMMQMTGFFLLDYYEDMSFGFTSMRAGFSGTVGVLMVAQFG
CLFLNLMHQADEVNDLTANTITVLFFVHSLTKFFYFAIRRAKFYRTLATWNNANSHPLFS
ENNSRHHQASVGSIRKLVLYVSIGVILSVVSWTSITFVGESVREITDPDNTNETMKVEIPRL
MVRAWYPWDAMGGAGYFISFTYQFIWLILALSHSMLTDVMFCCWLIYTCEQLLHLKEIM
KPLMELSASLDTVVPYSAELFRAVSATGNQPLPGTDGDGIRSIYSNQHDFSQFRLNTGTIA
NVKQGGVGPNGLTKKQELLVRSAIKYWVERHKHVVRFVSNIGDTYGAALLLHMLTSTVTL
TLLAYQATKIDQVNVYAASVIGYLFFTLAQVFLFCFFGNRLIEESSSVMEAAYSCQWYDGSE
EAKTFIQIVCQQCQKAMSISGAKFFTVSLDLFASVLGAVVTYFMVLVQLN
>BgerOr1
MTTQTGEVLDLLSKLHSFAPLLRLLNLSGMLPPLSIYKVPWKNFLYNIFSLLTFLSFIPLIIM
QLTGFYVYGNKLNLILVIAFQLAAYFDGMVTMAYFVWNRKRLLTMFLLMETKFIPLMSKV
GRPEKQKTILAKNWRFCKVITTVAIGAFLADMATWCVLPSVVRYMAYLHEEEEVEGEPE
HKMEYFVLVMWLPQNATAFPRYEMLHVFQFFSVWGVVANYAAGKLIIVTLFYHLATHFN
ILSSAIQDIDDICVNELSANEDMRLVVKQLAISHETNDSSVIEGALNLNLRSKIVQEKRLCPV
DLDPDNINVKESDIFGRLTYPDEVTYLTDCVIYHQELLKFCREINSFLTNILLFDLVIFQILM
CLPPLQLVLGQDASTFRFVSSIVDTGIWPLLICFWGEAVSQESLGVRKAAYDCQWYNRSQA
VKRLLQMVLMRTREPVRMTAGQFYNLSLETFADIENKVYAYFTLLKNMQG
>BgerOr2
MENRGESTLEKLNLLKKQYQFLHVSGILPYSSVCKSFWKTSLYNSFTILCMFYLPFTIVTFL
LAIEQFGDDLDIFVSILYEVFLFMYIVFELYYWILNRKYLLKLYNDLESKFIPHITKLNLSPD
CIQDSINFYNKLMIIIVIICVADIFAWAGVPFLLWCFETSEDYKNMEGNEFWKYFSNICWLP
ANATQTPMYQVVYSHQAFSSYLCGIHLTSCNVVMFSVIYHVATHFKLIIAALEKVDSRFLV
KESKQEERYLSKKDIETNEDQSKNDKRANEDLSVTPENFKNQRSSVIDEEKIYQYLIDCVK
YHQDLLEFCKEANTLMSPMLLATLVYIQISVCVPLLLVALGRYNGFAKYIVAVVEQFVMPC
MICYYGDYVIEQSLNVQKAAYGCIWYHRSPEVKKLLQIIIMRAQKPVQFTANSFYVVSLETL
GDIFNKVYAFFTVLKGMFE
>BgerOr3
MIGHKKQNLLNRNLIALYISGFLCPPNWQKNLCKKILYQIFTLETIAVFPAIITVQTIELFHR
FDDLFATTAILFQLACFISVYINFLYFLYYKPELIKLMDRIEIDFVPLMERVGSSKRRETILAE
RHEKSSSITILMISMHIFVTTAWGILPWVLSYIDYFIKTEEELAEVEIRNYFGCLMWLPENV
MKSPKFEVMQLFHFWGIYGIVSNITSCYMIMFMLTFHTATIFRLICAAFEDIDEFERSLRNE
ENFKKNSCVSDGKCVFVEIPSDEGFNENSNEEHDNISRNRKQENAQPFINDANGSNVSNP
QVNEEIRRRMNEYLINCIEFHQAAITYIEDLNDLVSPMVFIFFIFTEVMLCLSSFQLALAKW
DEKKIKFLSSVSCVFTWPLLLCIYGDDLKSSAVKESAFSIRWYCQSKTFNKLLQMVMIRAQ
QPVCIRAGKFYVATLETFSDLCHKVYAYLTLLRQMYDNS
>BgerOr4P
MTGKKRQHFLNRNLVALYISGLLCPPNWKNNWYKKILYKIFTLGTVVVIAAIIVVQAVELF
HRFDDLFATTAVLFQXTCSTSVYINFLYFLYHKSELMKLMDNIEIEFVHHMERVGSPGRIK

TILSERYEKSSSISILMISMHAFVTTAWAVLPWILSYIDYFIKTEKELRELEIERYFGCIMWF
PENVMKSPKFEFVQLFHFCGIYGIVSNITSCYMIMFMLTYHAATIFRLICASFQDIDEFERSL
RSEEHSKLNSFISDSVKMPLNKLRELESLDENSNDHDYVSRNRKQENVQPSISDVYVSTVS
NPHTNEEIRRRMNEYLINCVKFHQAAIKYIEDLNDLVSPMVFIFFIATEVMLCLSAFQFALA
KWDEKKIKFLSSVACVFTWPFLLCIYGDDLRSSAVRQSAFGIRWYCQSKTFKKLLQMVLIR
AQQPVCIRAGKFYVATLETFADLCHKVYAYFTLLRQMYDDK
>BgerOr5
MEPKKSTEEKWKGAKGVTANKLELMNFQLKLLSWFALLPPMNAKRDSWTYRFHLLFCC
TLLFWYIPMLIADTMAIPQNWGNLPLVIEVIFQISASISAMIGGYYITYHKYRVVEHFKMLE
TRFMGFINNSTVSEEHLSNTLAKVTKQAKLSSYLLILNVGTILLSWTGLPYIRMSKARASEN
ILDQSSPDFWGYFCFVMWLPENPIESPKYEFLYLFQLPCVAVVIFHITGLNMIFYFTILYISF
YFELLTKSLQDIDKRFPLESEHGPQIIENKLILNEEEDASNQQYIQTHDLSVVKNEDSYSRK
NLETSYLFNNVNQNETPEEYVANFGDKSPEFLSLEENAIEHLKQCIKYHQSLLEYHKLAND
LLSPMFLAFFMSNEISMCLSVFQILVNENSGILKTLSSGLTVCTWPFIISYSGEYLTNKGKEF
ESVIYDMQWYKRSEKFKKLLLMNLAGAQKPVRLSAGNFFDVSLESFAEIVNKVYAYFTIVK
KMYDG
>BgerOr6
MLSKEPNNGKCNDNKENNRKKLDLVSLQLKILTYFAILPPANAKRDSWISRFYLAFCYLLL
FWYIPMFAFDMMAIPQNPGNLPLITEVIFEMSASTTAGIASSYFIFNRHRVTEIFEMLETRF
EKFINNKTVSETNFKCIFMKVSKIAKIVTYTIAINVCLLVAAWICLPYTRRIMDETSGNVHD
ESKPEFWGYFCYIMWIPEKPLESPTYECLYLFQISCVLMVICHYTAMNTIFFFIIIYTLAYFQ
LLTTCITDIDKRFPIENGSKCDSEINRRLFRNLHGHVPKESHYKTKSDSCRDDALVLTKDT
NDGYLYDLHNDKLTAVNEELANGLEDDAVEHLKQCIEFHQSLLEYYKSVNSFLSPIYLAFF
LSNEISMCVSVFQLLVNENNGIMKIIIGAINACSWPLIISWCGDYLTEKSKELESAIYEMQW
YKRSERFKKLLFINLLGAQKPVRMTAGKFFDVSLQSFAEIVNKVYAYFTILKKMYDT
>BgerOr7a
MDFLKHQLFILYLSGLICPKLWVQNWCKKILYNMFTLAIVIVFPLILSVLMVTLMQQLDDL
STVIAIIYNMLCFISVYINFIYVLRNKSNLLNLIERIETEFIFYMERVGSPQRRETIILKRSKIA
KNITYLMLSMYLFVMFFWTIFPWFLGYKDYFMNTDEQINTDMQNYFGMPILLPENINKF
PIYEIVQIFHFVSAYSIVTNITACYMVIFILTYHAATLFKLIYAAFEYFDHFERNLRREYELED
DVISDCDSLKSDGSKTYDFHQSKTEERSANYGGNTNQDTANKSISINENELEFSINNSNTST
IQDPKNNEEYQRRLYKYLVDCVEFHQAAIRYTDDLNDLVSPMIFIFFIFTELMLCLSAFQLV
LAKMDERKLKFLTSSSTLFAWPLILCHYGDDLKSSAVKESVFSIRWYTQSKSINKLLQMVM
IRAQQPVTIRPGKFYVATLETFSDICHKVYAYLTLLRQMYDN
>BgerOr7b
MAEIKKNNFLEAPVFFLHLSGLLYPPNWKQNWYKKIFYDVFTLAIAVFIPLMEIVLLMHFI
ANLDDLFRATAVLFQILCLMSVHIIFFYFLRHKPKLLKLIDRIETEFDFYMERVGSPERREAI
LSERYENLKKIVNGMWLVYLFSMLFWTIFPWLLCYIHYFIKTEELSKEDTEKYFGLEMWL
PENINKFPIYEMFQMFNLMTVYCNVTNMSACYLIMFVLTYHTATLFRLICSAFEDFDYFES
TLRSEYEIYDTKNQSNSFKSDETERRSPARERSGHHDGYVDSFNQDSVGTNKSIDRNKFKV
IDTVTDVSNISNLEDNEEIKTRLNNYLIHCVKFHQAAIRYTDDLNDLVSPMIFIFFIFTELML
CLSAFQLVLAKMDERKLKFLTSSSTLFAWPLILCHYGDDLKSSAVKESVFSIRWYTQSKSIN
KLLQMVMIRAQQPVTIRPGKFYVATLETFSDICHKVYAYLTLLRQMYDN
>BgerOr7cP
MAELKRNNFLKVPLFFLHLSGHLYPPNWEQSWYXKDIVRYIYTLAMAIWSPSISVVLMVY
LIVNMDDLFRATAVLFQILIIISVHINFLIFLRHKSKLLKLIDRIETELFFYMERVGSRERIETI

LSERYEKLDKIVYCMWLVYLFAMLFWTIFPWFLRYIQXFIVTEELSKDTTQKYFGMLMWL
PENINTSPTYELFQIFNFMTLYGSVSNMSASYMIMFVLIYHTATLFRLICSAFEDFDYFESTL
RSEYELEDNTKSQFHSIESNQLKIYNILPLSAKKQSKHCDGLSGNTNQDHTVAFKTRHENK
FKLFINDTNVSINLNFENNEEFKERLNKYLVHCVEFHQATIRYTDDLNDLVSPMIFIFFIFT
ELMLCLSAFQLVLAKMDERKLKFLTSSSTLFAWPLILCHYGDDLKSSAVKESVFSIRWYTQ
SKSINKLLQMVMIRAQQPVTIRPGKFYVATLETFSDICHKVYAYLTLLRQMYDN
>BgerOr8P
MKRKNLFKRQLFVLHLSGILCPPNWEQNWFKKTLYYTYTLGVAICVPSMFTVLMMYLIN
HIDDLFKVTAVLFPNLCFITVYINFIYFMRNKSKLLNLIDRMETEFLVHMERVGSPERREVI
LSRYQNSKKITYLMLSIYVFAMLFWTIFPWSLGYLDYFIATEEISKADAEKYFGLPILLPENI
NKFPIFEIVEVCNLMSVSCNVTLMTACYMIMFILTYHAATLFRLIRAAFEDFDYFENKLRN
EYELGDDTKSQCHSIRSNHSKMHNFFGSPGKERSEHCDDGNTNQEHTDAFRSTKENKFK
SSINNTNVSIEFNPKNNEEFQNRLNKYLVHCVEFHQAVIRYTDDLNDLLSPMIFIFFIFTEL
MLCLSAFQLVLAKMDEKKLKFLTSSSTLFTWPLILCNYGDDLKSSAVKESVFSIRWYAQSK
SINKLLQMVMIRAQQPVTIRAGKFYVATLETFSDICHKVYAYLTLLRQMYD
>BgerOr9
MEGTKRLRLLHLQLIALYLSGILYPPNWEQNRCKKFIYDIFTFITIIWFPPTIFLQMIFLLDC
LGDLRTATGILFQVSCYISTYIIFLYFVWHRKALVKLIDRIETEFVFHLERVGTPSRRDAILA
EDYRKQKIITCLMLGLCFLVEAAWGVIPGMIGYIEYFIKTEEELANNEKGKYFGLAIWLPD
NVNKSPTYEMVHCFHFIAVYTVVSNITACYMTMFMFAYHTTTIFKLTCAAFEDADDFENT
LLVENESNHDIRLMVGSSSSKETMRSYLNSMAANRWNEHSDRMLDKKSKEDNNHGFTS
NELQTSNFNSTVSTGDDVDVSHQKMFNEDLKRSMQKYLENCISFHQAIIQYTSDLNELLSP
VMLVFFVFSEAMMCLSTFQLALGKMDEKKFKFLWSVLLASVWPLLVCLYGDDLMSIAVK
QSVFNTRWFCQTTAYNKLVLMVMMRAELPICLRAGKFYIATLQTFADIGHKVYAYFTLLR
QMYDD
>BgerOr10
MEPQTNKREVFGLNDTLIEYDEETQMKRLKLLGIQRRLLYTCTGLPTFSTLQSKFKMILFH
LVSNVLVFWFIPHTITQFMALYQHMNDIELLADLVFQIALYIQTGTMAFFFRFTRKELSNY
FELLETQFVPNLKELGLSIEHKQMVKNHIRYGNFMIRILFGHWCWILFSWAILPAFTGYYD
MYTQFDQFRNESLTLTNASITITDEYRKYFGILIWLPNNIDQFPVFELFYLFDFVGTYGVSS
WCASQVSVSLVLMHNLSLHFKLLIASIDNLDIVILKTLDMQKVSHTTISESHDKSAVDSQFQ
PDLAANQERSNRIQNLTKATSKVSTAKNNLIISSSRDQQLHLSREHEEIVFNYLVQCIKYHQ
ALLGFCEDINKYLGPVLLIFFLGYEAMVIFSAVRLAMGLSEGNFKFFSSVVYVIFWPLVICSV
GQDLTNQSENVQFAAYNSRWYNHSTRIKKQFQMMIMRAQKPVKISVGKFYSVSLETYSD
MGNKVYAIFTLLKRMMAE
>BgerOr11
MELQTTEREIFRSNDALLEYDEETQKTRLKLLGNQRRLLYACTGLPTISTLNSKFKSNLYH
FVSNVLVFWFIPHTITQFMALYQHMNDIELLADLVFQLALYIQTGTMAFFFRFTRKELSNY
FELLETQFVPNLKEIGLSIEHKQMVKNHIRQGNFIIRILFGHWCWILFSWAILPAFTRYYEL
YTQFDQFRNESLTMTNASTAITDEYRKYFGILIWLPNNIDQFPVFELFYLFDFVGTYGVSS
WCSGQVSIYLVLMHNISMHFKLLIASIDNLDIVILKTLDIHYDPHTTISEFKGESVEDSKFRS
DHSASQAGSNRIQNVTKDTTEVPTPKENLTISSSSKQQLDISREHEEMAFNYLVQCIKYHQ
ALLEFCEDVNQYLGPILLLFLLGFEGMVIFSAVRLAMGLSEGNFKFFASVIYVVFWPLVICIV
GQDLTNQSENVQFAAYNSRWYNHSTRIKKQFQMMIMRAQKPVKIPVGKFYSVSLETYSD
MGNKVYAIFTLLKQLMAE
>BgerOr12

MEDQPSSAILTISENLIRFDPKTHIKRMNLLGIQPQLLYVCGILTPNWFSKSTFRSVIFKFYS
NLLLLWFLPHITSQIMALYQHMNRFATLTDLIFQLALFIHTVIITFYFVTYKSQLAKILDLLE
SQFTPYFERICILNIHKPLIEDVTKQGTIVTKALLGVLSSCLLSWGVFPAMVRYYDLYFTTD
QEKDESLNITEEYIKYFGLVVWLPPNVNEFPVYELVYIFDFIGTVGVACNCSGVQSIFLVIMF
TISTHFKIIIASIQNIPIKFPEILKQKKYVFENNGPNTNLNILSMNVIALHPNRPDTMIDSDTE
YNNMKSNSDIIHSEDLLNPELEERVYKYLVNLTKYHQILLEFCKDVNDFLSPVLLVFVLGCE
AMVCFAAFRLALELNEGNFKFFTSAVSMVIWLLIICWYGEHLIEQSIKVQVNAYDYNWIN
QSTRIKKLLQMIIMRAQNPVKLTAGKFYPVSLQTFAEIGNKVYAFYTLLKQMLADSEITG
>BgerOr13P
MQLSSKKKNQEYKIVDFSTYERLKTFYKIQLIILHYTGIFPRPEINRNEMIRKFYNTCTALVI
ICSLTSPVAMCTFLYQNWGLITIATGVIFQTTFYTVNASVYAYMALNRQRLWKVISSSEVD
SGMWNSFPKGTSIILQSRTNRWKFXEAFNLNKVLTWSIVIANSAGFFSWIILPITLWFVQG
EETDNPEETGYNTGQWKYFIYMMWLPENAVEYPTYYYIYLYQTYICVMLITVYTAYNIMF
SSLVIDIISKFDSLMMLLKNIDNMNLLEEDNIDSKICKNTDMPNRIANDILNHKEEFKVTFR
RENVETQREISIKDIDSDTSINITEEEKKYYYLVNCIKYHQHILRQAELMNKMFSPVLFIVFT
LGLIMMCTTVFQITLGSGQGTSLKIASAMFATWFPISLICYFGDKLTQKSLKVQEIALSTRW
YDRSPRYKKLFHVLIMRTQKPIQLTGGKFFIASLETFSD
>BgerOr14
MKTFYDIQLKMLHFTGILPLTDIYDITWKRVCYNIYTIIVIMLEIPAPFAQFVTIYQNWGVL
EITTGVIFQVCFYFNNASIYMYLLFNRNRLRHATCSVARKFVEQFGLKELDIYDTIRKKAFA
FNKVLLWVILLINSVTYFFWLIMPFTLWCTEYQNLNDVEEMNGYNTGQWRYFIYMTWV
PQNALEYPIYNFIYLYQWFALSVILVLYYGYNLMLFSQIIDISSQFNALLVFLKEIDKMTLCM
DNVLLQDDESNSKSTIFNKVSNMATTTHLAAANMNLKNIEEFTNSHIKERFDIMDDLYM
DNIFTEPIIDNLNKKEKELYSYLINCIKFHQYILKRVEEINKVYSPILFVVFTIGMVMMCTTV
FQVTVGSGQGTNMKLLTATITVWFPISLICYFGDKITQKSLEVQKVILSGQWYEKSIRFQKL
MLILIMRAQKPVQLTGGKFFTASLETFSQVANKVYAYYTVLKQMKDSE
>BgerOr15
MFCWKRKSENNNKQDDDETSAFLTVQLTFLKFGGILPSKSIFNSPLKIKIYNFFATISMLW
YIPIVVAQGVAAYQHWGNVSFVTGLLFESAAVLNNLMISSYLMMNRHKISSIICKVNSAFK
NQTEQLPFEEKHQQSLSDASNKNLKFAWILIITNILSSTLWVLVPFLLWYTYDGDDIYEHD
ESQEKEIHWEFFSQKSWLPSNVYETPYYQIIWFYQTVPVYSILINFTGYNMFFYSVTTFAST
HFQILADLLRNANKYIESTTLIQSSLEHQALTRGNLNTENEHIQVSDVHELNISDENMHGK
MEIKSEFNYSINKNERKETYQDVAYLVKCLKYHQALLEFCADVQNLFGPILFIFFCMNGIM
MCITVFQATLPSEEEGFIKFSMASLSCWFPIFLLCWYGDHLTIQSLEIEERAYGCLWYNSST
TFKKILQFLIKRSQEPVQLNGGGVFPISLKGFADIANNVYAYYTILKKMQQA
>BgerOr16I
MLSRKVVSEDNQKHEKRNKSNFLTMQLTFLKIGGILPPETIFNSPWKLKLYNTYALTIML
WYLPIMSSQFFAVYQNWGNLNFVTALVLEIAASLNNVAISSYLMSKRKSIADLISKVNTSF
KNQTKLLPFGDKHKKILDHASSRLSKFTWVIIVVNVSTGIAWVVGPFISWYTYDGDDIYKP
NRNDEIDIHWEFFSQRSWLPPNVPVYETPYYQIFCLYQAMPVFSILVNYTGYFTLACSLVT
FTTAHFQVLADLLQNVYKHIETTKLNQEYPEQQSITDEALVMDEENNFQQLSSDIKENKDI
HNEEEIKIQYDYSIKGNESHEDVHYLVNCLKYHQIILEPDQDDGFMKFLTACLSCWLPPFV
LCWYGEQLTTQSLEVQQRAYDCPWYKSSTEFKKNLQMLIMRAQKPAQMNGRWKFSISL
KGFADIANNVYVYFTILKQIQ
>BgerOr17

MATKIVTTNKDPEFRSEMDLLDFMYKFFHLTFIAPMLKTTDNSWKKRLYNLVRQILLITY
FPSFLGIVLGLYKFWGDMKTVTNMFVTGVPLIMGFCAGLYFYWYWKEIRDFMESIERESN
FINPFVHSKTKLLQIVEDTKRKCKFITQFIAIFQFFAFVAFFIKPFILDFSEESELKIAEEEYR
NEQWKRLIYIMWLPIDITSPEMYYYTYAYQLITSLALYCHTATTITFIFVSSRYAAAQFTIVC
EALNDVSILNIFSVCRYEMNTGGRDLLNSQSMPEELHPNLRKSMDSCVNGLADKEIKSNE
VHIYIRECIQIHQSAIRYAMKLNNLLSPVFVMYLSLLTIGLIMGTIQLAVVDELDQKFPYFGS
VSVTLTDLFAFCWHGQILINESLSVEQAAYNMLWYNHCSSVKQLVRFIIFRAQKMTELKRS
GFLNLSIRTFSAVLNFSYKWFTILLEMHDD
>BgerOr18P
MDRPKQAGTTSHIPRECGRRMDLIGFMFRFLHFTFLGPSQEIKNNTWKXYCIIQFVMVFS
QILSILGIALALNKFQANVGDIIDILVTGSALSTSFLCALYFIKNWDSIZNFMNSMETESCFT
NPFVRSNGDLLQIIEETKRKCTLLTKVLVMTELIGICTFFFKPFILEYLEDPNKNLTEEENIM
NHWKKMIFVIWFPLDPTNPITFYCIYICQLFAALAFXCHTASIINFNFVLIRYSALQFTLSSK
ALSQTDIITSSSNLKSXENELEVMKNENISSEMAVESQSFLRDRSDTYIDEETRGQKEAESN
EVFYYVRGCIXFPKQMNKLLSPLFVLYLLLPSIILVVSTFQLTMGGGIEKNMHNLSVSTIVLS
DTFALCWFGQTLMDESLAVKQAVYNTNWYDQPSSVKKLLSLIMVRSQNETGFPNISIATF
SMILNASYQWFTVLLKMLPE
>BgerOr19
MDIQTRLSAIHLSTECDRRMDLLGFMFKYLHFTFLGPSQETMNNTWKRRLYSLVQVIILFS
YIPAIIGQAFGLCKYREDVEDISDILVSIVPLITFFPCSLYFITNWDNIKRFMLSMETESCFAS
PFVQSKNNLLQIIEGTKKKCIILTKVIVITEICGTCCFLFKPFVLDYLENQNRNLSEEEHVKN
QWKKLMFLMWLPVDPRDPLIFYSIYTYQLFVCFVLFCHSTSIINFNFVLIRYSFIQFTLTCR
AISETDVLTSCSKLESKNDSEVVKNENISSEIVAKSQSLSRDSNDIYINEETTEQADTESSEV
YNYVRECTILHQSAISFAKEMNKLLSPLFVMNLLILSTVVVVSTFQLAMGGGVEKSLPYLSA
SMVVLMDTFALCWFGQQLMDESLAVEQAVYNTNWYDQPNSVKKLIYFIMMRSQNVVEI
KETGFPNLSIETFSVILNASYQWFTILMNMVHE
>BgerOr20
MESQASRSSIHLSGESDRRMDLLGFMFSYLHFTFLGPSQETRDTKWKKVVYNLVQFVMLF
LYIPTFLGIALGFYKFRENVTDMTDILVTGLSYVIAFPCAVYFIKNWDEIKNFMVLMETRSC
FTNPFVHSNSNLLSIIEETKRKCTFIAKVIVIIEFIGVFTFYLKPFILNYFEDPNIKLTDEEQIM
KQWKKMIFVAWFPVDPRKPVIFYSIYAYQVLTAFTFFCHTSAVMNINLVLIRYSVVQFTLT
SKALSEIHIQTSSSTLESKTHSENEKDSSSETTIEPQSTLKDSTLLYTTEETMEQADIESNEV
FNNIRECIILHQSAISFAEQMNKLLSPLILISLLISSINLVVSTFQLAMGGGLQENMPYLSVSSI
VLLYAYAFCSFGQTLIDESLAVNDAVYNIHWYNRPSSVGDMISFMIMRSQNAVEIKETGFP
NLSIATFSMIVNSSYQWFTVLLNMIHD
>BgerOr21P
MESQASRSSIHLSKESERRMELLGFMFKYLHFTFIGPSQETRDTKWKKVLYNLIQLVMLFF
YIPNFLGIALGLYKFRENVADMTDIFVIGVSYVTSFPCALYLIKNWDEIKNLMVSMERRSCF
TNPFVHSNCNLLSIIEERKRKCKFLTKAFVMTVFIAYFTFVLKAFILDYLEDPNINSTDEEHI
MKQWKKMILVVWFPLDPRNPVIFYSIYAYQVLTVFTFFCHSSAVMNFNVVLIQYSSLQFTL
TSKAISEIHNQTICSTLESETYSETMEQADTKSNKVFNNVRECILLHQSAISFANQMNSLLS
PLFVMYLLTPSITLVVSSFLLAMGGGLEENLPYLSVSSIVLMYAFVVCSFGQTLIDE
>BgerOr22
MESQASRSSIHMSRESERMDLLGFMFKYLHFTFLGPSQETRDTTWKKLLYKLVQFVMLFL
YIPTFFGIALGLYKFRENVADMTDVLVTGVAYVTSFPCALYFIKYWDEIKNFMVSMETRSC
FTNPFVYSNSNLLSIIEETKRKSAFFTKVLVIIEFIGLWTFYFKPFILDYLEDPNINLTDEERI

MKQWKKMIFVAWFPVDPRKPVIFYSIYAYQVLTAFTFFCHTSAVMNFNLVMIRYSAVQFT
LTCKTLSEIHIQTNSSTLESKTFSENGNFSSSETAVEPQSILRDLTHLNITEETTEQADTESN
EVFNNVRECIILHQSAISFAKQMNSLLSPLFTIYLLMPSITLVVSTFQLAMGRALEENLPYLS
VSSIVFLYCFELCSYGQTLIDESLAVNEAVYNIQWYNDPSSVKDLISFIIMRSQNAVEIKETG
LPNLSIATFSMIVNSSYQWFTVLLNMIH
>BgerOr23P
LENQTGRTAINFPRESERRMDLLGFMFKYLHFTFLGPSQETKDTTWKKVLYNLIQFVMLF
LYIPTFLGIALGLYKFRENIADMTDILVTGVSFMTAFPSALYFVKNWDEIRKFMVLMETKS
CFTNPFVYSNSNLLSIIEETKRKCTLLTKAFVITEFIGICTFLCKPFILNYLEDPNINLTDEEYI
MKQWKXMIYVIWFPVDLRESVIFYSIYTFQVLATMTFFCHTSSIMSFNLVMIRYSAMQFTL
TIEALSEIDNVTNSPTLESKTYSENINISSSDMTVESFLRDLSPSHIPEHTLEQADSNEVFNY
FRECILLHQIAISFAKRMNKLISPLITIWLLMPSIPLVVATFQIAMGGGLQKNAPYLNVSLVA
LSYLFALCSFGQTLIDESLAVDHAVYNIQWYTHPRCVTDLISFMIMRSQNAVEIKETGFPNI
VNASYQWFTLLLNMLHE
>BgerOr24
MENQTGRTAINFPRESERRIDLLGFMVRYLHFTFLGPSQETKDTTWKKVLYYLVQFVMLF
LYIPIFLGIALGLYKFRENIADMTDILVTGVSFMTAFPSALYFVKNWDEIRKFMVLMETKSC
FTNPFVYSNSNLLSIIEETKRKCTLLTKAFVITEFIGICTFLCKPFILNYLEDPNINLTDEEYI
MKQWKKMIYVIWFPVDLRESVIFYSIYTFQALAAMTFFCHTSSVMSFNLVMIRYSAMQFT
LTIEALSEIDNVTNSPTLESKTYSENRNISSSDMTVESFLRDLSPSHIPEHTLEQADSNEVFN
YFRECILLHQIAISFAKQMNKLLSPLFVIYLLLPSISLVVSTFQLAMGGGLEKNAPYLSVSSIV
LLYTFALCSLGQTLIDESLAVDEAVYNVNWYTHPRCVKDLINFIIMRSQNAVEIKESGFPNV
SIATFSMIVNASYQWFTVLLNMLHE
>BgerOr25I
MENQTGRTAINFPRESERRIDLLGFMFRYLHFTFLGPSQETEDTTWIKVLYNLIQFVMLFL
YIPTFLGIPLGLYKFRENIADMTDVLVTGVSFMTAFPCALYFVKNWDEIRKFMVLMETRSC
FTNPFVNSKSNLLSIIEETKRKCTLFTKVFVITELIGVCTFLCKPFILNYLEDPNINLTDVEYI
MKQWKKMIFVIWFPVDLRESVIFYSIYTFQILAAITVFCHTSSVMSFNLVMIRYSAMQFTL
TIEALCEIDNLNNSPTLESKTYSENSNISLSDMTIESQSFLRELSPLHIPEDLTEQADTESNE
VFNYVRECILLHQSAISFANQMNKLLSPLFLIYLLVPSISLVVSTFQLAMIVNASYQWFTLLL
NMLRE
>BgerOr26P
MDVQMKRTVVRQSKKCERRMDLLGFMFKVLHFSFLGPSQEIQDNTWKKTLYRLVQFIMI
FTYIPAFLGIALGLYKYQEDFGDITNILVPTLPLITYFHCALYFVKNWEAIKHLMQSMETQS
CFTSPLVHSNGNLLQIIEETKSKCKFLTKIIVIMEIFGIRXPFIIDYLEDQNISLTEEEGMKNQ
WKKLMFLIWLPVDPRNPIIFYSIYTYQSYVAIVFFCHTISIININFVLIRYAALQFTLTTKAIS
ETDILASSFILESKSDSENSTESEIDTESQSFVNDLTDIYIDEEAESNEVFNYLRECIILHQTAI
SFAKQLNTLLSPLFLMNLLIISTTAVVSTFQIAMGGGVENNIHYVLVTLIVLMDTFVLCWFG
QKLIDESIAVEEAVYSIHWYNYPSGAKKLINFVIMRSQYAVEIKEPGFPNLSIATFSMIVNAL
YQCFTVLLNMFHE
>BgerOr27
MDVKTGSTVHQSTEYSRRIDLLGFMFKYLHYTFLGPSQETMDSTWKRRLYSLVQFIMLFS
YIPALLCTALGLYEFRHDIRDITDMLVTIVPFLAAFPCGFYFIRNWDQIKQFMVSMETDSYF
TSPFVHSKDNLLKIIEDTKKKCVFLTKFLVIAEFIGLTSFFFKPFILSYLEDSQINLTEEESIRK
QWKNMIYVIWLPVDPRKPLVFYSVYIYQLFTALVFFCHSSSVININFVLIRYSAVQFTLTCK
ALSQTDILTSSSTLKSKNEMLINEYHAQSEIDSESQSFSGDLTDSSFSQDTSAQKEKESDESF

NYVRQCIILHQSAISFATEMNALLSPLIVMYLLNPSFALVVSSFQFAMGGGIEKNMPYFWV
SVVSLAETFALCWFGQKLIDESLAVEQAVYNMQWYYQSSSVKEIVSFIIMRAQNAVELKET
GFPNVSIATFSMIINASYQWFTVLLNMLNE
>BgerOr28P
MDVQMRTTLVRQSKKCERRMDLLGFMFKVLHFSFLGPSQEIHDNTWKKTLYRLVQFIMI
FTYIPALLGIALGLYKYQEDFGDITNILVPTLPLITYFHCALYFVKNWEAIKHLMQSMETQS
CFTSPLVHSNGNLLQIIEETKSKCKFLTKIIVIMEIFGIRXPFIIDYLEDQNISLTEEEGMKNQ
WKKLMFLIWLPVDPRNPIIFYSIYTYQSYVAIVFFCHTISIININFVLIRYAALQFTLTTKAIS
ETDILASSSILESKSESENSTESEIDTESQSFFNDLTDIYIDEEAESNEVFNYLRECIILHQTAI
SFAKQLNKLLSPLFLMNLLIISTTAVVSTFQLAMGGGVEQNIHYVLVTLIVLVDTFVLCLFG
QKLIDESLAVNEAVYNIQWYNHPSSVKMLINFIIMRSQNAVEIKEPGFPNLSIDTFSMIVNA
LYQCFTVLLNMLHE
>BgerOr29
MDVKTRSTVHQSTEYSRRIDLLGFMFKYLHYTFLGPSQETMDSTWKRRLYSLVQFITLFSY
IPALLCTALGLYEFRHDIRDITDILVTTVPFLTSFPTGFYFIRNWNQIKKFMVSMETDSYFT
SPFVHSKGNLLRIIEDTKKKCVFLTKFLVITEFIGLTSFFFKPFILSYLEESQINLTEEESIRKQ
WKNMIYVIWLPVDPRKPLVFYSVYIYQLFTALVFFCHSSSVININFVLIRYSAVQFTLTCKA
LSQTDILTSSFTLKSKNDSEMLINEYPTQSEIDSESQSFLGDLTDSSFHQDTSAQQGRETEE
SFNYVRQCIILHQSAISFATEMNALLSPLIVMYLLNPSFALVVSSFQFAMGGGIEKNMPYFC
VSVVALAETFALCWFGQKLIDESLAVEQTVYNMQWYYQSSSVKEIVSFIIMRAQNAVELKE
TGFPNVSIATFSMIINASYQWFTVLLNMLNE
>BgerOr30
MEESTTQLSPRFEKKMDLLTFIVKFCHFAFVGPADYIAETPWKRRCYRLVQLTLIGSYIPAF
IGIVLGLYTFWGDIEVITNILVTGCPLFTGAFIGVYCFIYWDEFKLFIDSIESKSCFSHYFVHS
KKNLLEIIEDTKLKGKLLTKFVATVEFIGLASFFFKPFLLEYLEGSNINLTEEEQIMEQWKK
MIFIIWLPTDPRQPQIFYSVYVCQFISCLAFYSHTATIITFIFVSTRYVASQFTIVTEALKEVD
VLTSIPNYRTETDTKLLGNKFETEQKNQMESRTYLEDFTESYVNENILEDEENNSKEVHD
YIRECIKIHQSSIRFAMQLNQLLSPIISFLLVDLTIVIVTAMFQLAVGDGIQNDFPYLGVAIVA
LTDLFGLCWHGQELINESQAVEFTAYNIHWYNHSSNVKYLVRFIILRAQKAVEIKGSGFINL
SINTYSAVLNFCYQWFTLLLKLHDD
>BgerOr31
MENQHNEDLEENYVVNQRLGLFEMNLKLLSVTGIVPNRNITCSKWKLKTYRLLQFLSLLI
YIPVLILQVLGLCFYWGNITLTTDNICITCSLLIGYIPALYQAVHAENLHRVIDMIEQQSLFS
MKAVKENSAYTTIVNDAKKTASYLTWLTSISLSVTGILWTSYPLVMHYFQNNSSDDNSLD
HQFKYLVFVMWLPFEISQPDCYRLTYLLQVIVFLTAMAYIIGILTLYLNIMVYLKAQLKIVT
AAIRELDKAYFFEKEEDMNQGHTFEYDKQFRQEIVQEVINETYKNKENRNTLQAVEESDI
VRKSEDIQLYSIASLELLTTKIESNNVLNGEENSDKYIVQCIKFHQSAISFSKEANNILGFGLI
VAIVCNVVLIITETFQLSLVVTTKYCSGFFTSLTQQFIFSYFGQGIINQSLAVGAAVYKVKWY
CLPVRFQRLLLLILARAQNPVKITFGKIFPLSLILFTQVLNVSYKFYTVLLQVNDP
>BgerOr32
MVNLLLCNFQSHLQTMKEDDGLKFKIVELNLKLLNASGMIPSGGIKSTVWKSRLYSIYQIF
QYLLYFPILLSQLLALYYNKNDIIVLIDNICALTIPSSSYIAAILLKFKGNAVFKLIQNVENSYI
YDLPEVKNNEKCIEALKSSKKMCRIMFWFTIGSGLSGMFFWVLSPILLDFAEQVIYDESNS
NDRNTTENKFVFAMWLPFELESSSSYVTVYIVQTVIFFVSGNALFGFFAFSLTLLIHAANQF
EIVTLMVKDIDDELSKYATDEFHSNTLPLKIRFKNQNEITKLRLDVYKTSPYYINTKLNSRG
REYEKEHAMELYHRNTSNVYYFKTDLKIEMDAEEHHATNYLRQVAKAHQIAIQNMTNTE

EIIGLPLFCITFLALTCIVGSTFILSMNPGFQQMMKSFATLTFALAQFFFFCIFGEAVITQSLA
VGEAAYSCQWFERGPAFKRLLLIVLTKAHKPVYLTLGKLLIQSMEMFAWVVQSSYKFFNL
LLQMNEQI
>BgerOr33
MTAERVDLIPLNLKLLFSAGVVPTSDIRSSIWKLRVYRIYPMFMSFLYSMVLTAQCLAIYKY
WGHLDVITDIGFTMVGIFMCYVMAGYAIKNTERILQLIKMLETELPTIEEPVKEAVRKSRIF
TYIMFFLVHGMLSTWIAAPILLRYAQDEKEEPETDEPYPYFCFVIWLPFDATQSPGYELVY
TVQTLCFLMASLYYTSINTLFITFIIHVAAQFQILVQSLKCLDDHPHDEYFINCIKHHQTIIKF
SKELDLVLSPLLFFFFCCSQMIMCVVTFQVVLTWADGTIQVKLILGLMAALCGPLMFCWF
GTVMIQEGLAVEQAVYDCKWYERPTNFRRLLGMVLMRSQKPVRLTAGQFYDVSLTSFTQ
MLNAVYTYFAVLKQLYDE
>BgerOr34
MEALSLNLRLLQISGIVEPPSVSKSGWKHVIFSMYMTTAVIIFIPILVGELLAFYHFWGDLV
VITNNMFTIVGNITFFWEALYIIVRRGAFNRLVATLQRMLKDMPRWNLKQQTIAKNSIKR
GRRLTWFMVIIVYMVPFSWSIAPLIGMLLPENEEEIQSVPEDEVEDFWKSLISIMWLPLDA
TKSPVKEIIYTCQFMIFILTASYYSSVNTVFVTFIVNLTGQLETLTATVEDMDQIIDQYDDKE
LHNVFVDIIRHHQSIIDFSQELNAVMSPLLFFYFFSTQVMMCVMAFQMVLTWGEQSNFVK
FFFGLLCVLAGPFFFCWQGNILTEQSVQAEKAVYNCQWYERSQRFKKMVLTVIMRGQKPI
ALTAGSLYVLSLDTFAKMLNSVYYYFAVLKQLHEE
>BgerOr35
MTENAIPEQFHEYEQNIFDLNKKWLYRAGIVPLYSIKRNPFKMLLYKLFILMSYILYFPTLI
GQLLALYHFWGQLNILINGLYNMVACLMCYIIGTYGLFKKNEITQLFVAFEHEILPKMANV
ILNERKIEIFKTASKRARKITWIVIIVLDIFAILWIPVPLTNHYLKERNGTASELDDGKTWIH
FCFLIWFPYDIRVTPYYEIMYLSQVILFFTACSYLKAVSMSIASLMVHIAGQFEILSETLADID
ELLTNTAIQKAAQIQNNQFLHNNIQEMADELPTSTPKTSNANLSTVVKPLSQFESKEENLE
LIADSLEQKKCFINIVKYHQSILWFLEDLNRVSGPVILASLFSCQFLGCLMIFLMTLEWAQE
KNSSNFARYIFAFICAMSFPISFCWYGNTVTESANNVRDAIYKIQWNRHTKAFNKDLLLLG
EGARKQVYISGMQFYKLSLETLREMMSTMYSLYTLLHKIYHT
>BgerOr36F
MPQSKTTETFEDMKEKALLLNLISLQKLGIALPSNVKSNPLQKMKYYLMIFFSCLVYIPTFI
GQMYALYHFWGNLDILVHGLYNMIAIFMCHLLGMYGLIMKDEIANLFVTYKDEVLTKVEK
VGFKNSTNEIFSKTSKKAHQISSSAMISLQIFFILWAPVPLATHYLENNDETMSAEDDEKR
WLHFCFLIWLPYDIRVAPYYEIMYLTQIIMFYTASCYLCYVSMTMASFIIHVAGQFEILYKAI
EDMNLFLKTAEGQKETQMQNKVTMNENIKHFPSASDTMMEYFENIDSSVHYLSTKGSAD
HLEIILDSQEHREYFLNIIKLHQAILQFADEVKKIAGPVIFLILFTCQFLGCLMIFLMTLEWK
LGSRKGENFARFGIAGICALFYSYIFCSNGDIATQNCRKVASALYENRWYHYTTKFNRDLL
MALRCAQKDIYFSGANIYKLSLETYGQMLNTMYTLYTLLQKAYEG
>BgerOr37
MPKSNTIETFEDIKKKSLHSNMESLQRIGIIQSPSTKKNPLKKMICNLLTAFTFLIGIPTLIGQ
ILALFHFWDNLFILFSGLFNFVALVLCYILGVHGVIMKDDIMQLFVTFKDELLIKTENVGIK
EKTREIFAETSKKAHHMTMYILTILVTLMVLWAPVPFYTHYTEDRNATRSAEEDEARWL
HFCFLIWFPVDIRVTPFYEIMYLSQCLIFYYVNLSHIHSVGITMASFIVHIGGQFEILSKAIEDM
DTFLETTDIQDKMQRNQTMMRKTMKEHAKHSPPHFHIKTGVPKDLNENIHALSIYGSDG
DKLEFILDYPEQREYILNIVNLHQAVIEFSREVNRVAGPVILALLFICQFLTCLMIFLLTLEW
KQGTRKAENFARYAIAGLCAISFSFELCWHGENAKQSSRRLADAIYESQWYRHTVTFNRD
LFMIKELAQKEVTFSGGNFYVLSLETFGEMMNTMYTLYTLLQNAYEG

>BgerOr38
MLEALFDNSPPQVADIDEMESHELGLMNVVRLELCIVGLIPTNGIVNDRWKLKLFRYYQN
VMICVYIPVMLGQLMAIQHFWGDLDTVTDCAGMFSVFVACFFDYLYLIEHEKTILHVCEVL
EETPIPKVKNPRLIEKYLAIVEICRTEIRIVMEIFWGLAAIGAIKWLLYNPIENLIIDRHFLNH
TLKEDKPNADFVFIIWFPFDATWSPLFEIIYMFQSILLVMATCHNICANSTFLTFMVHAWG
KLEFVECTVSSIDDELNPKRVGDEAVEDKISEIFGEFDDEDTIPAADDTNDDDTTVTLEEE
AEQETGLNGDLQVFSPEGANTNSDVVPVENGVEVAMDAHVEYLKRCVKQHQDAIDFVHE
LDDLVSTWLAVRMFAFQMVAAGEVFQLIVNMDDWEKLVGHGTILFFIFAQILIYCWFGEQ
ILQGGWDVDRAVYETPWFTYSQTYRKNLILIIMRAQRPVEVTVGHYYSLSLQSCELILQNIY
FFSMFLNQINNKASKQAALAELEA
>BgerOr39
MFKQNQNLPDHKGNSDEPETGREVMSFCINLLRIAGLTPIYERNPIIAHAHNFFIFALLMT
STILQIVGLIECWGDFKPMCFISGTLSGCSAITVNYILLIKNRDAIFKVIHVLKIEYISRVNPKY
MGYVRNSEKEVVKSAILKILFGTALTGLGTIIPLINKSIDDGGSVTGNLTKYQYMTKYQIFVT
YTPLDIQQSPQFELNYVVLVILTAFVVGTDAVIDSLYYALLSHLTAQFKILVAVLDDMDENL
NFIDNEESINYSKTTSKKCEVKQRYSTDISKEEMDSIQLNEVSNVYPDGKDIHKAYLKDCIR
RHQALLSFSDDLNSMMSFAAFLQVISTPVMICMGGFLMTTSLTVFTDVIKYSSLVALAFYK
LLIYCMYAEDLTQASLKVREALYNLNWYGLSMQCQRMLPIMIIRSADAVVPKASVFYELS
MDSFGTVLNTAYTYFTLLLQMSDS
>BgerOr40
MPDINDNDIKKLNLIRPNIRLLAIFGLLIRDLTPWKQTLRRCGVYLFIVAAFFNIVSQTIEAC
RVTDARLMSEGGLWVIFGVKGVTKVITIFYYEDEIWKLINIFEENIFKNAHLLTSTEKKSAK
ESLNFSKRMLSYTLAASLLSNGTLALDILPPSEEGLQALEAASAPDWNSPTKYAAPFKGMR
NRYWYNRVGYIFSMSYLTYYPMMFIYSFNCQLIMYLATHFAILSDSLENAARNVQEMMKR
STSTSSLQVFSTNENMKEEIMTSLDREMSSKDNLEMTEDCAVRDQRFDEEMYKYLLERT
KQHQEYLLLFHRLNKITRVVVFTDILGAAVYIAACLMGMAEIHETYEVLEFGFLLMFAVVE
LGVFCWVGNYLTTQTERVGAAAYASLWYVQSTKYQRHLQLVIMRSQRPLKLSVGPFGVVS
LELFAKIMNTAYTYYTLMREFVSPKKEEAGERHHERSE
>BgerOr41
MNRNELPDDIQKLNALRANIKFLKFLGLLRTTDAMSPHWKQIAHRCLKYSIGIPISIFALGA
IVEAYYFRDNSREIIQAITAFLSVLKSVIKYLSFILHEEEFKEISEHCDGNFIIEGNDLTQRER
KMIENDMAITKKLTYSTWFMCLLLLSGLTFNVFPPSEEEIENSDRDYVPAWRSLNRYAIPF
QSARSPYFFFRFLYSVFVEICAMVPFILINTMNALVITYLTTQFSVLSDALEHIEENVQAVLE
NEGSQIVTQENGRFREESHENRRVITQDALFHWEMEMYLRRCIIHHQKLLQFFDILNNAM
RTTLFADTLVASILISMMSFSLLIATDMGDVIQNLGILTLTTTELWFFCWLATRLSVQSERI
GEAVWSSLWYKQRIKFQQHSKFILTRAQRPAQFSIYLFGTVSLELFSKIMNTAYSYFTIIKE
M
>BgerOr42P
MPGIELPDDIQKLNVFRVNVKVLQFLGFFRTSYVNTPHWKKIVHRCLKYSIGITAFIFCFGA
LVEGXRCRDYPLEVIEAITAFLTGLKALIKYLSFIVHEKEFKEIAEHCDDNFIIEGNDLSERQ
RNIISKDMATTZKATYLFWFLAVITLVTATFNVFPLSEQELENYDRVFLPAWGSLNRYAIP
LQNARSPYFLIRVIYCTFVIILACVSFIIINTMSVLVITYLTIQFAVLSDSLKNIEENVQAVLER
GGSQFVTQGQCRLEDDWHGNREVVTQNEAFHLEIERYLRCCIKHHQKLLQFFEVLNNVL
RTTLFVDILVASVLISMLSFSFLISSDFSNVIQNIGILYFITIELWFFCWIGTRLSTQSEMIGGA
VWSSAWYKQRVKFQQRSAFILMRAQRPVGFSIDIFGTVSLELFSKIMNAAYSYFTIIKQMI
>BgerOr43

MEMHNIELPADIQKLNVLRVNIKLMKLLGLLKTYDEKTSPWKKLAHRCLKYSIGLPVFIFS
LGAIVEAYRFRNYPLEIVQAITGFLSALKALIKYLVFIIHEEKFKEITERCDGNFIIEGNDLTE
RERKIIEKDMAISRKLTHFVWLMSFLMLSGMAFNVFPPSEEEIQNSERDYVPAWNSLIRFA
IPFQSAKSPYFFPRVIYSVFVEMCACVPFILLNTMNVLVITYLTTQFAVLSDSLKNIEENVLV
ILEREGSQIVPQGKARLEEGWQENRELLTRDDLYHKKMEMYLKRCIKHHQKLLQFFEILK
NAMRTTLFVDTLVASGLIAMMSSSFLIATDFVAVMENLGALTFITMELWFFCWIATRLST
QSEKIGEAIWSSPWYKQRVKFQQHSKFILLRAQKPVGFSIDIFGTVSLELFSKIMNTAYSYF
TIIKEMI
>BgerOr44P
MSGIELPDDIQKLNVLRVNIKVLQFLGFFRTSYIDIPHWKQIVHRCLKYSIGITAFIFCFGAL
VEGYRCRDYPLELIXGLKALIKYLSFTVHEKEFKEIVEHCDDLTGRQRNIISKDMATTKKAT
YLFWFLAVITLVTATFNVFPLSEQELENYDRVFLPAWGSLNRYAIPLQSARSPYFFIRVLYC
TFFTILACVPFSIINTMNVLVITYLTIQFAVLSDSLKNIEENVQAVLERGGSQFVTQGQCRLE
DDWHGNREVVIQNEAFHLEIERYLRCCIKHHQKLLQFFEVLNNVLRTTLFVDILVASVLIS
MLSFSFLISSDFSNVIQNIGILYFITIELWFFCWIGTRLSTQSEMIGEAVWSCAWYKQIIKFQ
QRSTFILMRAQRPVGFSIDM
>BgerOr45
MPRIELPDDIQKLNVLRVNIKVLQFLGFFRTSCIDIPHWKQIVQRCLKYSIGITAFIFCFGAL
VEGYRCRYYPLEVIEAITAFLTCLKALIKYLSFTVHEKEFKEIVEHCDDNFIIEGNDLSERQK
KIISKDMATTKKATYLFWFMAIVTLVTATFNIFPLSEQEIENSDRDFLPAWGSLNRYAIPL
QSARSPYFFIRVLYCTFVDILACVPFIILNTMSALVITYLTIQFAVLSDSLKNIEENVEAVLER
GGSQFVTQGQCRLEDDWHENRVVVPQNEAFHLEIERYLRCCIKHHQKLLQFFEVLNNVM
RTTLFVDILVASVLISMLSFSFLISSDFSNVIQNIGILYFITIELWFFCWLGTRLSTQSEMIGEA
IWSCAWYKQRIKFQQRSTFILMRAQRPVGIYIHIFGTVSLELFSKIMNAAYSYFTIIKQMI
>BgerOr46P
MEMHNIALRADIQKLNVLRLNIKLXLGLLKTYDERSSPWKQIAHRCLKYSIAIPVFIFSLGAI
VEAYRYRNYPLEIVQAITGFFSGMKALVKYLVFIIHEEDFKELTERCDGNFIIEGNDLTDRE
RKIIEKDMDISRKQTHFVWFMSFLMLSGMAFNVFPPSEEEIQNSDRDYVPAWSSLIRFAIP
FQSAKSPYFFPRVIYSVFVEMCACVPFILLNTMNVLVITYLTTQFAVLSDSLKNIEENVLVIL
EREGSHIVPQGKGRVEEGWQENREILTRDDLYHKEMEMYLKRCIKHHQKLLQFFEILKNA
MRTTLFVDTLVASGLIAMMSSSFLXWFFCWIATRLSTQIMNTAYSYFTIIKEMI
>BgerOr47I
MEEYNLPEDIEQFNIMRFMIRFAGFIGLNFSSRKHSERWEIVYNVMSKTMKCFIYFCMLFS
TISSLINCFYIIKRDMKEFIEAIISFVGCLKTLMKFSIIIFHEDKLKELLNIYIQNFFIHGKYLTE
SEKKIIKDSLRTAKKVTKFIWVASIVGLAGILTNVSPPTPEELEENEYLPAWKSQSRFVIPF
NEGRSPFYLLRVICTELIAVGVMSAMIWHIPFIFLLITYLTTQFSTLTDSISNIADNVREQLF
YSKLDYVLNYNFFTDAMSTSAMISILGFAVLMSSNTAQVLQYLGMLVHFSMEFWCYCFIA
NRLSTQSSMIGETAYAISWYQQTTVFQRQIMMIIMRAQRPVEMSAGLFGKMSLELFSKIM
NTAYSYFTLMKETIS
>BgerOr48
MEPVNVKRCLDLNLKLLTFSSVRPTDVGSSPLANKLYSAFTVFTLLVFSLISSGILPWLIYT
DYTLEDLIEVISILITQIRCLTRFLTFIVFRKGLRNLISTLYDNFNIHGRDFDSEEKLIINNTM
ENCRKITKYYVGLFCCTGISMVIQPLTAPEPDELEDHSNSSLPHKPLPFKAYYPINTMKSPQ
YEIVYIAQSYLALIESWGIGSLDSFCVAILMYVTCQYELLCGSLMNMKRNVAIRMQMMAHS
KMVAFQERVRTEPISDEEIQVHEDRNLPREETPVLYRNVVCNTNLCLIEEEFNTYISKCIIH
HQAMLKYVDDFNACFRPMFFSILLTASILMCMMGFQVIVIPPKGIMFVRVVLHLLCTIFEL

GFFCWFGSDVMRKSASVCDAAYFSEWEDLSSDAKKNIGMIIMRSQQPVAIQAGLFGALCL
PTFGTMMKNAYSYLALLKQLHEGDMEE
>BgerOr49
MQSHIVINFQVRKVLTHEMDSESECLKLNVIFLKIGAAWPGDVSPVTWGNTLYSAYKILYR
FLFMLCTLGIVLYCFFAYETLEDLTENISVFMTQTSLCGRFFVLAMYRKELLVLIRTVANNF
YAPGPEEKAMVQRTIAICQKLGKSIFILYVFTTLAMISHPLTADERVLPFKSWYPMMNSSV
SPFYELQYMAQASLTIMEGWFAGAMDSFVISMLLYGALQFELVAHGIRKSKSVEELRTCIQ
YHQAALQYGEDMCTALNPVFTVQVFSDNLLICVLGFQVMVMETDGIKIIRVLLHLVCAIFQ
IWFLCAFCSHFTTQSSSVYEAAYESTWYDQKPAFKQMTWMVVERARKPVALSAGLFGEM
SLPLFSAIMRSSYSYMALLSQMNEENFN
>BgerOr50aJ
MDFQTCDFEFKDCTKFVVKLLQFSGLWCTSDENVLIYRVYKVYSITVAITMFYLVFSEVLV
FFSQNEAHDIIESIIIFIANLTTLSKMLIYFLKEKEVKHILDSVNRNFSINGGTLSIENRSIIKST
LELSKRLCIAYAIMTYSGVTFYVDVVPLTTIQFSEGKNFTHQTVRKLQYSIWSPIDIQSSFNN
YIFMYIVITILSHIMANILVSTQAAFLTLIICLTGQFKLLCESLRNMSKNVRCRLQRSSGSTK
WKSKELEEDASYSDEPTDLFLAEAEIYLKECIKHHQSLIEFAEKFDNVWKTTFFVQFLTAS
FLVCFLGFQAMLMPFGLNLIKMLIFIFVELFELGVACKFGSDLMTQSEDVYRAVYDSDWY
NQSTRFKQSARMLIMRAQKPVRLTAGRLGILSQPLFAAILRSSYSYLALLRQMHDD
>BgerOr50bP
MTFDKNYCEHNNCANLAVKFZKIAGLWSESDNSSNGPMYYVYLFYSKTVLLVQLYFWFA
FIAQLFIGKTELEETIELIILIITQCHIYFKLRIFYFKKEEVVPLVGALKSNFLIHKENISTENRD
TIKSTLILTRKMCILYAIMINGGITFYTDFVPIASSSFESQGSNETNSTQRVMPFSIWLPIDM
NTSQYYLEVYLLLFAGTHCIALVLMATQAFFLTLIICLTGQFQLLCDALRNLSMNVQIRLQS
TEGSTKWKSKELEEDASYSDEPTDLFLAEAEIYLKECIKHHQSLIEFAEKFDNVWKTTFFV
QFLTASFLVCFLGFQAMLMPFGLNLIKMLIFIFVELFELGVACKFGSDLMTQSEDVYRAVY
DSDWYNQSTRFKQSARMLIMRAQKPVRLTAGRLGILSQPLFAAILRSSYSYLALLRQMHD
D
>BgerOr50cP
LVDSDQAQTVWLHRLHQHRCLYRMFSIIVLLYQLYFFFGFVVELFISNTLEDTIEVLILSISC
FHILFKIHICHFKKEQLNILIERVKNNFFIHKEYLTTENREIIHSTIHTSKTICIIYAFLVNISTV
FYIDFVPIANSIFYSQSKNSMNATHRELPFKIWVPIDLPTTPYXYIHVYAFLAIGSHAMTNV
VVATETMFCSLIICLTGQFKLLSDSLRNVFKRVEMRLHPRTGSTKWKSKELEEDASYSDEP
TDLFLAEAEIYLKECIKHHQSLIEFAEKFDNVWKTTFFVQFLTASFLVCFLGFQAMLMPFG
LNLIKMLIFIFVELFELGVACKFGSDLMTQSEDVYRAVYDSDWYNQSTRFKQSARMLIMR
AQKPVRLTAGRLGILSQPLFAAILRSSYSYLALLRQMHDD
>BgerOr50d
MNTCEKHSRYYSEHEECTKIAVMFLRIGGLWSLNSGFIFVNRLYYIYSSFTKFMTFYFLIAFI
LELFANNTLEETMEILILWITSIHLLAKLLIFHFNKQQIEILFETVKNNFSVHKHYLTTENR
KIILSTMQMSRKINIFYAVLLNLGMTFYVDFVPLVTSVTYTNGTTIMERKLPFKVWVPLDI
TTSAYYVHAYVFLTVASHILATLLVATQSVFLTLIICLTGQFELLCDALRNMLTNVNYRLQH
NSDKGSTKWKSKELEEDASYSDEPTDLFLAEAEIYLKECIKHHQSLIEFAEKFDNVWKTTF
FVQFLTASFLVCFLGFQAMLMPFGLNLIKMLIFIFVELFELGVACKFGSDLMTQSEDVYRA
VYDSDWYNQSTRFKQSARMLIMRAQKPVRLTAGRLGILSQPLFAAILRSSYSYLALLRQM
HDD
>BgerOr50e

MMSFDCNDESDHYSCANLAVKFIKIAGLWPTSEHFSVRNCVYKLYYVMVFLIQLYYFFGLT
IQLFFVDTVLEETFEIIIFLITEVHILYKTRIFYFMKKEILSLLGSLRINFVIHKEFLTTENKQII
HSTAVLTRNMCLLYAIMINGGITFYCEFVPIATSVYDYCVHNESNILPRKPPYSIWLPIDMA
NSDYYIEIFLMLTLASHCIALVLMSTQAIFLTLIICLAGQFELLSDALRNLSKNVQCRLQSTM
GSTKWKSKELEEDASYSDEPTDLFLAEAEIYLKECIKHHQSLIEFAEKFDNVWKTTFFVQF
LTASFLVCFLGFQAMLMPFGLNLIKMLIFIFVELFELGVACKFGSDLMTQSEDVYRAVYDS
DWYNQSTRFKQSARMLIMRAQKPVRLTAGRLGILSQPLFAAILRSSYSYLALLRQMHDD
>BgerOr51a
MKTGFDDELNKRVDLCMEFPILMLKNSGVWTSVFTTRPQLHKIHQRVLLVLITVMTITGV
TGIYVYRKRIEVVFELLGLLISHSVLICKLYIFVYCKEDVEDILNRVRTNFTIHEQRLTVENK
DIIKEVINKTRMIVLVFVSLSGFTTIFYFIVTPLVNIYMQNKLIFLQNDTEIYEPPLKILPVQL
YIPFEIDKSPVYELVYLLVSILAVNECFTFTAIETIIMSLLIYIPSQYSLLCDSLRNATGNVKM
RLQQNTIISDVCEFDSSPKHSEEKKEDNIDCDKDINEVSYNNINDNDGKNITEQIFQQEME
KYLRECVAHHQKLLEFTEKLDQLWEFAFFFQFMTVSLLICFIGFQAMSGPLDANLFKMLG
YLVSVLFQIFIYCAFGSNLTAKSAEVFNAVYDTDWYNQNNSYKLITKMMIMRSQKPVFLT
AGRFGALCLPLFTSMIRSSYSYLALLRQMQDS
>BgerOr51b
MYGILHTSLKISGVWCGAKNTMTLSNRLHQRFLLVVMAYAAIGIVMGIYTHSKRFQVVIEL
VCLSISNLVINLKLYVFIFRSEDFHYVLQGVRTHFFVHDRQLTLENKTIITDILNKVRNLLM
VYVSIMTYSSGTFIFITPFVNIYLEKNKSFMNNTETDETPLRILPIQLWLPFDINESPAYELG
FLIILITAVVLCIAFTAAEATIISLMVYIIGHYDVFCDSLRNATRNVKTRIQQKNIMADVCEF
DSSPKHSEEKKEDNIDCDKDINEVSYNNINDNDGKNITEQIFQQEMEKYLRECVAHHQKL
LEFTEKLDQLWEFAFFFQFMTVSLLICFIGFQAMSGPLDANLFKMLGYLVSVLFQIFIYCAF
GSNLTAKSAEVFNAVYDTDWYNQNNSYKLITKMMIMRSQKPVFLTAGRFGALCLPLFTS
MIRSSYSYLALLRQMQDS
>BgerOr52
MTSMKVHNSFNLVEKMMHISGLWEKQYDCILSARLYRCYTYIVKFFMIYFAIGIPMKIIYPE
STEKAMEMVIMGIAHVILLFKLYLFYFRKKELEQVMSDVKKNFHIHGNRLTTENQKIIYET
MLKARYICVTFAIMLYFSFFVYCDILPLVTIKNHEYTFNFESTGCLIHNITITYSEMRLPIDV
WLPYSLKKTFVFQLTYTLLLIGCQVEAFNYISTDALFITLILYISGQFELLCDSLRSMPKNLEI
RLAKIYTKVPECNDLRFDVLQREAEMYIRECAVHHQHLIRESRRVESLWRRVFFAQFLIES
LWICLMRFRAMTMENRTDILMMILMLVCILMQMSLYCSFGSQLLTQSENVSNAVYSTDW
YNQSENFKLTARMIIMRAQKPVRISAGLFGTISMPLLTKILRSSYSYLTLLLNLTEA
>BgerOr53
MKRAMSRFSTKNNFYSKPRIKTQDVENCFRLIHKLLVLSGTWELKNTSTFWIITYQCYSLF
TKFSFVFLAFALPLKIINSEQPTDEVESFITSIAHSILLFKVSIIYFHKEDLKCIMTLVKRNFYI
HDKKLTTENEDIIVVTLQKAKFVTIVFVTMITSTFFIYAGILPFLTATENEYGIMSEFDDPLT
HNTTFRELHMRLPVNVWIPLDVNKSLNYELVFVPLLVGCVVEGLNLTVIDSLYFTLMIYM
TGQFELLCDSIHKISQDTTQRFLETKENTLDNQGCEPEWKSLQKEAEIYIREYAVYHQSLIL
ASEKIDKLWGRMFFVQFLVESIWLCLMGFEVMRMEMDSNRMIMIMLLICCLVQMGLYCI
FGSNLMQQSENVYKAAYGSDWYNQSKYYKQTTRMMIMRAQKPVKITAGLFGPVSMPLFA
YILQSSYSYLALLRQLNEQ
>BgerOr54
MTSKGEEGNYDDCLKVALFLMNLVGLGYNTNFSKVTGKLYRIYQVFTQMGVIIFTVGVTV
EMYVLRDDTENALEMIGWIITHIILCYKLYVFVIRKPEINYLISTLPKNFVIDGKNRRIDNKD
LADIVMKGARSVVMTYAIVMGSSIVLYVDISPLINYMTSAAEVQNITLFNQTEGAQRELPV

RLWLPFDTTETPMFEITYVYLAICAHTEGMLSCSIDVFCMSVIIILTGQFELLCDALKNSTN
YSLARSESTTQKGVNYDNQQKIMDNDIPNISNYDLEEAEDHLEECIKHHQKLIDFSERLNS
LVSSIFFLQFLTASIMICMLGFRLTTMDFDINLMKLLSYLLTCICQLCLYSIFGSNLMTQSEA
VHNAVYDCEWYDQSNHFKKSITMIIMRAQKPVTIMAGQFGSLCLPLFASMMQSSYSYLAL
LMQLNEEVEDE
>BgerOr55a
MDKSCEWSDIETEKQARNRINFYVKLLKIGGLWHMGKSELSEFLSSFYKKVLIIWFFIHGL
SIYISLCESRNNFDDLVEVLPICISTTIFYIKIYAFFFRRIEMEHIVQNVRDNFFIHRNGLTLE
NKTIIMSTLNQGKKFTIIYLSWNCFVNCLFSVVTPLLATAPETANGTDAVSPLIIKIWTPVD
HTVSPNYEIISLYISITCIMLGFDIFVTDLFIMILIIYCTGQFELLSDSLSKASVNVKKKLLQEE
PIKLLKIEGEQEVKMEENMSSTLSTKLMHSETEKYLMDCIRHHQSLIEFAARVEDLWKTY
FFAQFLTASFLICFLGYKSMTMDMDVNLLKNMGYLGSVIFQLALQCLFGSNLMTESSAVY
DAVYSSDWYNQSNKYKFCSRMMIMRAQKPVQIRAGRFGIMSLPLFASMMRSSYSYLALLK
QMQEE
>BgerOr55b
MSKRSEWSDAEIEKNARNRMNTFVKLLKFGGIWDMRKSRLFGVFSSLYNKILIFWCGLHG
FTVYASLCESRNNFDDFVEVMPLCISMTIFYLKLYAFIFRRNEIEDIIQKVRENFFIHGNGLT
SENKTIIMSTIKQGKKFTIIYLSWNGFVNVFYGVVSPLLAPVPEMANGTDAIYELPMKIWT
PLDLSVSPNYEIVSVYVSLTCIVIGFNFFLTELFIMVLIIYCTGQFELLCDSLSKASTNVKRKL
LQEESKSDTLLKIGEIEGEQEVKMEENMSSTLSTKLMHSETEKYLMDCIRHHQSLIEFAAR
VEDLWKTYFFAQFLTASFLICFLGYKSMTMDMDVNLLKNMGYLGSVIFQLALQCLFGSNL
MTESSAVYDAVYSSDWYNQSNKYKFCSRMMIMRAQKPVQIRAGRFGIMSLPLFASMMRS
SYSYLALLKQMQEE
>BgerOr55c
MDKTSELSDAQIEISVKNRMNLYVKLLKFGGLWQLKKSKLTGFLIFFYNKILLMWFIYHGF
SIYVSLYESRNNFDDLVEVLPLCISMTLFYLKIYAFIFRRKEIENIVQKVRENFFIHRNRLTLE
NKTIIMSTIKQGKKFTIIYLSWNGFVNFIFSVVTPLLATVPETANGTETVSPLLVKIWTPVD
PSVSPNYEIISIYISVTCIVIGFDFFVTDLFIMVLIIYCTGQFKLLCDSLSKAQANVNRKLLKEE
LKTHTLLKCAEVGGEQEVKMEENMSSTLSTKLMHSETEKYLMDCIRHHQSLIEFAARVED
LWKTYFFAQFLTASFLICFLGYKSMTMDMDVNLLKNMGYLGSVIFQLALQCLFGSNLMTE
SSAVYDAVYSSDWYNQSNKYKFCSRMMIMRAQKPVQIRAGRFGIMSLPLFASMMRSSYSY
LALLKQMQEE
>BgerOr55d
MNSDSELSDFEIENNVRNCMNLLVKLLKIGGLWCTEKSGYTLNFFYNKIVIILIYIHGVAVF
MKLYESRNNFDELLEVMSVSISVSMYFLKVNAFIFKKTEIEHILQNVKENLFIHKNKLMIE
NKKIIMSIIKHGRKITVIYMSWNLTINILYTLVSPLLAPIPETVNATDFVRPLAMKLWTPFD
QTVSPNHEIMTAYVSIAGILHGFNFFVTDLFIMIMMVYCSGQFVLLCDSLFETTANVKKMI
LDDQNKSFNQPTLNVSLKKQKRKGEQEVKMEENMSSTLSTKLMHSETEKYLMDCIRHHQ
SLIEFAARVEDLWKTYFFAQFLTASFLICFLGYKSMTMDMDVNLLKNMGYLGSVIFQLAL
QCLFGSNLMTESSAVYDAVYSSDWYNQSNKYKFCSRMMIMRAQKPVQIRAGRFGIMSLPL
FASMMRSSYSYLALLKQMQEE
>BgerOr56
MACGNQKRPSSYKEELIINTFKLNRTFLYLSGLVPSDAIVAVPWKLGLYRVYTFFTLFIISTG
IIATFVSIFEHWQDVERAGESAFVCISFVLVFLGSIYILLYWDELQKLMRTAEHEILSNIQKT
YVKHLHEAERFSRFLTRFLFISCLSVAVTRSYVPLIIHYVRKYVYGAGGTNRHTLTLLFDM
WFPFHIDNTPLFLLAYIIQMLIQCISVAHNVSVITYFFTLFIYSCTRFKILQAALKSIDKFIPEY

EEYDHKLSSELIQTDNCEFSDSSKTVNASSISEYNLIEEKTLSDIRFANVENEELNDSPGYDV
TFPGYYYERDFTHGRSSKMSEEIRNTNSKSSCSKSSEVPRPNLKDAGYNIPGMSAQYPNQV
IQSENSIYTVSIDVYCKNKNAENITEHIELEVVETKRTSVTVIDKRIVHEEDMDSSTFVEEE
MSVPNDKEPQNRKAERYFIECIREHQDVISFVDNLNNIISTWTFLYSWLEHCTVYYSILYLV
ESMENMKFFLRALFGLIGELIRVYILCFFGENIIHKSLEIHEALYECRWYNCCPKLKRLFHIS
MMRAQKPSVLKTGKWGFLSNETFSELITNAYICFILILELRISNHHIS
>BgerOr57a
MYFIKLFRSPEKVNVLQMNLGFLRIFGVLAQQSVNTSRLRVLIDYLLKWAMAVLILFFLFG
TTVRLSFVLDDFSKTADTLLFVITHVKSTVKISSLIAFREKFLNLITSIEENTYIKGINPLKREI
SCVDGYKKLARKICQFTWVTFFLSVSTWFTKLPQKPDLDLLANSTLGEYRRESFFNLWLP
VKGAESPYFELFNIFEYLAIGAVYLFVTLMNSSIIVLIIHKTAQFALLAETIENTNIHADEQN
QMKKATSNNEITKKESTKASEEMSTPARKVKFSSLEVEEVLLFDSCNPPPEVDELYINQVD
THMRLCVQYHQNLLKQAKILDKALALIMFTQILSSSFLLAIIGVLMATAKDSSTMTMGATY
LSYVLMETGLLCWIATQLRIQSEMVGQAAYNCMWYGYPKRIKLSLRFIIKRSQKPVILSLGP
FGSLSMELFGKILNSAYSYFTLMKDVSRN
>BgerOr57bP
MYFIKLFRSPEKVNVLQMNLAFLQFFGVLAQQSVNTSRLRVLIDYLLKWTMALLILFFLFG
TTAQLSFVLDDFSKTADNLVYVITYVKTTIKISSLIAFRDKFLXNLITSIEENTYIKGINPLKR
EISCVDGYKNLARKICQFTWVTFSLSISTWFTKLPQKPDLDLLANSTIKYRRESFFNLWLP
VKGAESPYFELVNIFEYLSTSVVYLFVTLMNASITVLIIHKTAQFALLAETIENTNIHADEQT
QMKATSNNEIAKKESTKASEEMSTPARKVKFSSLEVEEVLLFDSCNPPPEVDELYINQVDT
HMRLCVQYHQNLLKQAKILDKALALIMFTQILSSSFLLAIIGVLMATAKDSSTMTMGATYL
SYVLMETGLLCWIATQLRIQSEMVGQAAYNCMWYGYPKRIKLSLRFIIKRSQKPVILSLGPF
GSLSMELFGKILNSAYSYFTLMKDVSRN
>BgerOr58
MEVVIKPLPNDLRKLSPFGMILSILRCGGLLVDTSSIKNWSSAIGYSLRKTLTITCLFIVGIGF
LVETYLSRHNVEEFSECLTLVLKQTRNTTRLVSLFFHRKKILDLIKSIENGFFIHDRELNKE
EYSMIRHYLRNSRRFSYLYWLQWFLVLLFEVSAQRPPEESLETQNISMPISNQMPIKLWVP
FDTAESPFYEIGYFYNTFFCVIGSLLAAVTDTLIFGFLFSLTSQFNFLGLSLRNMSRDVAMT
MHPETLETINALVIQGQLNDDGTNNDDLTSDGTNFNEEIIKHMARCIRYHQQLLDNVEIF
NSFLSPIEGIEVLSASVLIALSGFQIIAGQFNAVHLPRQVSFLSFIVLALGLHCWFANRLTNQ
SEEVYQDMYASEWFLLPVNVQHSVPFIIMRAQRPVLLSAGPFFKLSLETFGKIMQTSYSYL
TLLTQFYAED
>BgerOr59
MEEDTAPKWLLEEDALIVIKNLLRFCALLPGKTKREILQRKITVNVILILMIFTVIGAFIEAY
NTRSNFMGFIQCGTVCITLVKCLFKIWYTRFKEKDLRYLLDTLPRNFYVKDNVHQEKILST
IRAKKKTAWILSVPYTSIFIFTIFLIALDKMSSLTYRPPEGTIIVNGTSNASLFIRTLPLRIWLP
LDEQKSPYYEIGYLYQLTIFTYQIYSTCVIDACIAVLVMYASVQFELLASTIEHAKQNVKELL
EENAADGRQVLSGEVTDDEEPNIKDSKWRSEMDNYLKLCVKRHQALLEYIRRLNIISSPIE
LLQALTSSILICTLGFTAITSGNTAILPKVILYTACAFLQIGLPCYCATQLQTQSMAVAEAAY
NCAWYEEPVSFQKSISLILMRAQKPELIFVGPFGTMSLELFAGVAQSAYSYLTLLRQVYE
>BgerOr60F
MGENTVPTWLLEEDALSLHKQIFPYFGLLPGNTTSEKRRTRIIGIISCTLMTGMVIGSLIEA
YQNLSSFKGRIASLSASITQIKCLLKTLVLIYYEDDVRYLLDKIMENFQVCSDIEKEKIISTIRA
KKRTAWWITVLYIGCFLGSILLVPIESIPDLLYRNPLATNGTNNTLEIKEFKRRLPLRVWLP
IDEQKTPYYEIGFFYQMIFFTYEILTACSIDTFIAILIMYASVQFELLGSAIEDKEEKVKTLLE

RKLSNEMIGSQNGPRMRSSEAETTKHIGKINLEKYISQSLNDDIDNIEEHASDEWNKEMF
NYLGLCVKRHQSLLEYVERLNIISSPIEFCQAFSSSLLICTTVFVVITLTKVSALPQYIMYASL
ALLQIALQCIYATELQIQSEKVADAAYNCAWYSDTLSFQKSLIFIIVRAQKQVVLEVGPFGTL
SLEFLGWVVQTAYSYLTILLQAYD
>BgerOr61
MKMEHTLHSDKQESGTMDLLGFMIKLSHYCFIVPKKTMVDSSWKYKLFRLTQMILVLLF
PPTLFTKMIAMNHFWGQMEVITYILVTGLPMYIGFHGGAYFFKNCKEIRNIVDQMETNSV
FTHPLVHSKDHLKQILIDTKRKCINITLLVTVSECITLFGFIMKPFIVSHLLDKEGKTDEEIV
DEIWKNIIFTMWLPIDPRDPLTYYILYTFQVVIALAYFCFTGPIVALIFVLARYASAQFAVVS
AALKDVDVLFNFPKPHLTIDTKLKNQFEMKQELQRASLIMEVSESILVNEDVNTNEMYSY
VRECVRLHQSAIRFAKEVNDVFSPIFFLYLSVHTAILVVVVFQIAVDSSTGSRVLFGCLASVI
LMHCFGFCWHGQELIDQSLAVERAAYNILWYNHPRSVKELFLLIILRSQKEVELKGLGYIN
LSINTFSLILNSVYQWLTVLLNMHDE
>BgerOr62
MDQLPSHVYMSTQLKVSRIFGIWPYPEGTPLWKKVASKFVFYVLLGLKVASSYLVLYHIYY
EWGHLPDPIDTVINITAHFNTTFGMMYIPCKIKKFQALLKLMDRTFMVSEDKSEDDFEKD
RQRLFERAMKSAAFVTKLFISVGLMTGVFYSIQPLGQQDGERKLPYRVTFPFGIDVSSGTN
YWLAYSLLMANFFGMCINGTINCDMFVSLIIKTTCQFSFLKHMLLNIRESVRRQKNTFNIR
LESADSKVEEKSLKVSTHKEEEDKTSKNEIEFLDDQENELIVKRLNECLKFHQDMLLFVKE
MDEHVSLFMLAQIGVYVGFLCMNIFALSIVPMLSPLFLQVAMFITSISCLLLVFSWYGNELQ
LKSLEVADAAYNCDWVNGPEPFKKSLIMLIWRAQKPVKLTAWKFFTVDLNLFITVMKTS
YSYYQVMHTMYSEE
>BgerOr63
MEQLPSEFYLKTQLKFSRNWGVWPWTEETPLWKKIFCKIIFYITLFLKLAAFACLLYHVYV
EWGHLPNPIDNIILASALLNCSFGMIYIPYKIEGFLDLLKSMDRIFVIPKDESDDELERFKKQ
TFERYMKSAALVTRLFLGSSLATGIMSGIEPLAYEEGKRPFPLLVSFPFDCSSGIHYWYAYF
FLMASWMSCCINGTINCDLFVSLIIKTTCQFHFLELGLLKIRDTAIKQKRTRTESVKQLKIK
FLDPSEEELDAEEDDLMVQNLKVCIKYHQDLLGFAAALEAHVSPFVFMQMFVYFTFLCM
NIFAISLVPVASMEFMEQLLFIICITCLLLILSWYGNELKLKSMDVAKAAYDCDWLNGPLEF
KKSLVTLILRAQKPVILTALKFFTVDLNLFIVVMKTSYSYYQVLHTMYSKES
>BgerOr64P
MGEQPTPASTNETFEMALPDMAVRSSEVYLEKQLKVQRFFGIWPHSEDAAPWKKVSSKV
LFYVMLVMKLVSLPLILYHIYDEWGALPNPIDTMVVIASHFNTIFGMIYIPYNYGDFQSLVG
LIDKTFVIPRSGSGDESQHRVFKKTMRTASFLTNLYVGLAVTLSVLYGIQPXAFPPEERPLP
YRLKVPFGIDVSSGRNYWIAYCVTLANWMGITINAAIVCDFLISMTIKTTCQFKVLELRLLQ
IRDFVRKQKQEKEQPAATVEENQLLLRRLRECLKYHQDILTFVQELDYHVSPFILGQISVYL
VFICMNIFALSFLPVLSVMFMQCAMFIVCILFLLLMFSWFGNELRLQSETVAEAAYNCDW
VDAPQDFRRALVLLIWRAQKPVKLTGWKFFTVDLNLFISVLQTSYSYFQVMRTVYSEEQE
>BgerOr65
MAVRSSEVYLEKQLKVLRFFGIWPHSEDAATWKKVSSKVLFYVMLVMKLVSLPLILYHIY
DEWGALPNPIDTMVVIASHFNTIFGMIYIPYNYGDFQSLVGLIDKTFVIPRSGSGDESQHRV
FKKTMRTTSLLTNLYIGMAISLSVLYGIQPFAFPPEERPLPYRLKVPFGIDVSSGRNYWIAY
CVTMVNWMGMTINGGIVCDFLISMTIKTTCQFKVLELMLLQIRDFVRKQKQEKEQPAATV
EELEELESELLLQRLRECLKYHQDILTFVQELDYHVSPYILGQISVYLVFICMNIFAMSFLPV
LSVMFMQCAMFVVSTLFLMLMFCWFDNELRLQSETVAEAAYNCDWVDAPQDFRRALVL
LIWRAQKPVKLTGWKFFTVDLNLFISVLQTSYSYFQVMRTVYIEEQE

>BgerOr66
MESFLLIMKNKLQAIDLNQSKNDLSINVNTNIQYLKMLAIWPLKEDIQIWKRAIMYVVFVI
GVFVQFIIICSQILDIVVGVDEHGELTDNIFMTGIAFNGLFKQVYVAYRKKSFQSLVKSIDKV
FYKAQEPFKNEKKIILENSLFYGKLVTWSIICVCIFSGFCYPLIPLSAGFHSLVDSNSTAPRPL
PHSGWFPFDKNESPYYEIVYAIMSFNAFYIALYASSTDTLIISLMIHTFKQFEILQFSIRNVK
QYAINRINAPNNQFGQNERHLSITSDTTVYSYNSNNISLIKKTNKNGFQKLNNENYMTDK
ESREKFNEELNICLGLCIKKHQELLSFSEHLNDLASPFLLVQLVVDVSFLCILTLHMTVVPV
MSFKFISAFGIVMAVLNIVGLISWCSGELSVKSEGIRDAAFECEWETTSSHFKNSLKFLMLR
AMKPCTIKAGKFMDFNLANYAMVLKASYSYFTVLQRVYHDK
>BgerOr67
MEDSSKKEEMSAHKYLGAHLALLEAGAMWPLRKLKTPTHNQIGMVGLIISVICQILLMIM
EQGYLYSSTSDPLEIIAYIGTACLRIEALGKLLYMLFIRKKVKNLLQSLDTCFKLSIHGENEG
EELDRKEEVFSIMKSWSYGAKIMAVSWTGLCTFGGTQWALVPFGINGYVTVTLDGDEHL
HQGNLTNFTEIGPRTETFYLRVLPLRGWYPFNETLTPAYETIFLIQGIGNICTAFAVGIFDQ
FYCAVALLLCGQFECLKNSLRNINIKDKSAILYDISANLTQLKFERNEKDLIANKNDEFSIQY
SLHDGNIENIKELIFRLTHKFDDDLENDYEETTREKTLNSEQMAMEDELKACIKHHQKIVS
CASELNMVYSPMMFMQCQKSLLALCLVAFQSTMVNGDLLQLFSLVGYVMLLVIQLFLFC
WCASELTERGKSVQDAAYDSGWPDANFGYKSSLIIMIHNCQVPILLTGGAFYILTMELFIEL
LRLSFSTYTVLKEMHEGQQD
>BgerOr68
MDLVSPKMIKFLGIEPERGAKEFLNFNLQRLRYLGIWRWDVPHFKLNQAFACVQIIAIFLF
SVTEILSAFDNLNDLDHLTKLCITTVFVYLLIFKNIYLAMRMDYACELIDMLENKFFTSSRP
PTQEQLMIVNRYGARAKFFTILRSNMALSIITFWLCAPLKDMLTEFTSDISFNEDYHSEEV
ESVVNKTSERQLPFVATFPFDIQNSLFNYIIGYVFQVFCGFTVFIGMPAWDMMFVSIFIHTS
GHFKALQHVLFNLQRNLSQTKMRQDYYSQAVQSSLLAEEQDQCNTNTEALDTSSMQVIIA
NQDLDDNMLETLNNCIQHHQLILNFVEKLEGLLGPVMLMQMMASVIAFCVIGFQMSVIPI
STESVKFVKLSVSLVSALVEQGMFYWFGGELLEESAQTLNAAYHCEWYSTDMKFRQNLR
LMMERAKRPVKLTAGGFSLLTLESFASVVQSSYSYFTMLKAVHDEDQE
>BgerOr69
MEYCLVFNINLLHYFGIWPSEDTRKPWKKAAYRMVSIFLLLQLILFYITELAGFYMNWGDI
RKMTEAMCQITANTHLICKISYLLFNHKRFHKLLQSLNNISSHYPGIKNNNNLVFVKNCES
VIKGFTFAYIGAGWLVGIFWVISPLIDNESGHSELPFHSWTPFNVSDPVLYSVMYTLHVMH
ASLFSTYIPSCSMFIFGLICHASARFKILHSLLLQSEISMESDKRMDKKEHEHKGLELYNYAF
PEVEVKGNRKDTNNYKNLLNEELTTLQECIIYHQSILRFCKELEEILSPIMFMEIITTGIALC
VVAFQVTEVPVYSLRFFTMATFLISVLFECGIFFWAGEQLITESLKTADIIYGCMQWYHNCS
LFSRGIPIIIACSQSEVKLTCGKFYILSLSNYANVIKTSYSFYAVLKSLHDS
>BgerOr70F
MAKVLDEEFNRKQLMNLNIKTLQLVGLWRGSMFAMKYKWRKMAYNVYGWLLWLVLF
MNLISQSMDVFLTRRNLEEMANNGCTTLTYAAAVAKQFMFLLNHKKILNLVEALHSGTL
SSSLKWSADQDNVLRIAHFQCRFVSWCYYCFGIVTLLLIDLTAITKSFPEAFPFLEVTPSNQ
TVKFLPFNAWYPFDVQAPLNYEMVCTFQIVMGMFGPTVNIGIDTFIVSLIICCSGHFRVLKY
SLRAIDSVSLSHLEIEDKDYICLIECIHHHQPLLKLVGDIEEVLSSCLFVQFLSSSCTICLLLFII
AVRIDSDGIVAQLSSIQFLSISFLQLLLFCWYGQQLIYQSDSLTTAMYESPWYDSSKGYRRSL
CIMMSQTMKLCSLTGGKLYILSLETFRAILTASFSYYTVLRNLNFGQ
>BgerOr71

MTLFNKNEKIYRNNNKTFMNINVTALQIVGLWPNINIPPKYEWIKMIYVYYGWTFFVVN
VLNVATQIADIAYTWGDLENLAANGGVTLMYIACILKQLNFFIYRRKIAEMVENIQNGFFS
DSLTWDDERNRIANSSNKFALTVSWTYFGVTACTVGTFILGGFLSSYPEIFGVEPLMVGNQ
TIKVLPLKEVFPFDIQKRGLFEIAFIFQFILLTLGPLLNAGMDNCMTSLIVHCCAQFKILKYA
LRNIDKRAYVLLGYEKTDETEDSNFTAKANDGEISNLTTNKLEYGERFPERVNEKAYKCL
RECIQHHQNVLEFFDDLRNVLSMYLFGQFLCSTSVLCLILFYIGMGVESENGFAEKVGFYQL
FVTATMQILMYCWFGNDLTYESDSLTKAMYDSPWYEASVEYRKNLCIMMARTVKLCYLT
GGQLYIASLETFRSIMGASFSYYTVLSSMKVEEN
>BgerOr72
MFSNKNEDESRFPDDFLRVNIKTLWMCGLWNCFGIQRKENFTYTLFSIMASFVLLHQSVT
QTAYLMFNMKYFAILISSAGLILTYLTLLFKRFVYLTVEQRIYRLVNGLRDGDLSSSSNWTE
EQVEMAKDYDRRARNMSWSYYWLGVVCLFFLSMSGIVNGPLKEDKSVNSTSWRNLPYN
EAIFEFDIQNVVYYGIALVLQYLAVFFGPTTNIGIDTMFVALVIHASGQIQILKLSLTKMKER
AIRMEPDDLNIRNTDRPIWDKIDPDSITEELKLIESFNDQFDEDNLESTQYENDEGRNYLS
NLLQVKLSMCLNDCIRHHQEIAKFTTELEYIFSPMSLIQFLSSSVTLCLIIFSIAVSDVSRLPT
NIVFLSISITQLLLYCWFGSELTYQFESLVAAIYDAPWYGSSLNFQKNILTMLIRATKSFILT
GGTIYLMNLNTFLTMMKASFSYYTMLKEMDIS
>BgerOr73
MSGKNEDKRKFPDDFLRVNIKTLKLCGLWNCFGIERRGKHLYTVFSGIGSFIILHHNLTQIA
YAIYNRHDFVKVISVAGLIVTYGTLFLKRILFLLLNKRIYRLVYKLRDGELSSSDNWTEEEA
DMAKNYDQRARMMSWSYYWLGIVCLFFFCLTGMLTGVIEEEEFMNKTTTSIRNLPYNEA
VFEFDIQKGQYYAIALLLQGLVVFFGPTSNIGQDTMFVALVIHASGQIKILKLALRKMKERA
IRMKDTFKSEKSSQFNIHNTDIFNKDETDSINEDLNLIEAFKNEFNEDIHLKIYEDQSYLTR
LLQVKLAMCLNDCIRHHQEIAKFTKELQDIFSPISLIQFLSSSVALCVITFTIAVSDASQIVTYI
VFLAISIMQLLFFCWFGSELTYQFETLGEAIYDSPWYESSLDFQKNIHTMLIRSTKSFILTGG
NIYLMNLNTFLTMMKASFSYYTMLKEMDVS
>BgerOr74
MDLHSYLKEKALTPYEPQEYDFMAINIKAIHVVGLWNYHWKKKNQWRYYAYCLFSSIQIS
ILAVHTVTQFLDLCINSHDMATYATTAWLTINYFAATTKQVFFVYHRDELQDTTLKLKGG
ILSKGLRWSKEQDDIALKTHKQVRTLSLIYDWMGVICVIGIVSIAIQSSYYKLYAEFTGLQNY
TGQETEFQLPLQAWLPFDIQKPGNYLIAFTFQVTTLLIGPIVNIGTDGFMAGLMIHACGEF
RILKHSLKMLKRRARQLQTEETEIRKLSSECPSELQPGMDIQEIDESTAKNDDSMLNRTSG
DLRPYLYKALVECIQHHQEIHKFISELEEIFCSLMFIQFLSISVRLCLNVISMTLSGSKLLVQM
TNVPLISVTFMQGLLYCWFGSELTYQSESVARVIYETPFLEASYRFKRNIIIMMMRAQKRT
QMTGGKIYVLSLDTFQALVYASFSFFRLLQEFA
>BgerOr75
MAINIKAIHVVGLWNYHWKNKNQWRYYAYCLFSSIQISILAVHTFTQFLDLCINRHDMAT
LAFTAWSTINYFAATAKQVFFVYHRDELQNVTMKLKGGILSKGLRWSKEQDDIALKTHK
QVRTLSLIYDWLGVFCVIGTVSIAIQSSYYKLHAEFAGLQNYTGQDTAFELPLQAWLPFDIQ
KPGNYLIAFTFQVTTLLIGPIVNIGTDGFMAGLMIHACGEFRILKHSLKMLKRRARQLQTE
ETENRKVPLDAENPSELQSRMDIQEIDESTRKNVDSIVHRTPQSEDLRPYLYKALVECIQH
HQEIHKFISELEEIFCSLMFIQFLSISVRLCLNVISLTLSGTKLLVQMTNVPLISVTFMQGLLY
CWFGSELTYQSESVARVIYETPFLEASYRFKRNIIIMMMRAQKRTRMTGGKIYVLSLDTFQ
ALVYASFSFFRLLQEFS
>BgerOr76

MEELNSASHLRFNFWVLSVVGMWPASNYIILGLSYILTVIIFAFWLSFLALGISVRNDMNR
LPVCVGFVCGYFVSLYKWSRIVVYRKDIQDLIRSFTRCFKLGFVAAKDDSDSWRQILEATR
KKVRNLSVGWVTYLMYISHHGVLFALSLNGMKVPIPKTETEKPLFNDNVNITELHTDESA
NQTKSEEMFMTLKFLPFGDWPFVDTRDSPAYELVYFLQATGAIVHAWAHAATDVLFLAI
STLVCGQLEILILTLSKVGKSNNYKEFSSCIRHHQQILSIAEVINDIYSPIMLVQFFNTMMAL
CTFAFEASKMEGGGNQIWVQADFFIATALQVYLFCSVGSRLTTLSLEVADSCYESDWVNK
PKFWKKSVEIVIMRAQKPLKLVGGPFYVISKETFLALIQLSFSYYTVLRNVQEEKE
>BgerOr77F
MTCSRQIHDRFGLYLVFLRMVGIPVFMKKSKLYFAYEIVANICIYTTSLACWLQILEEKHDL
KEMMATLRIAMSMVLVNILHFYMRFYLKAFEELLQITDTFTWEELPARSPTTGKLTAVG
WIDALKKFMKYTLIYVLIFHCIQTAYRMWYVENSMVFRAWYPYNYTVSPAYELTNFSQAI
SSVCGATTVLAFPGLYSTMVAIGRCQFDKIRIMLQAIYSQESSASDAQNQLKECIVLHQQVL
EYLRKIEDVLSNCLGAVLFLQMTDLCVLAFSLIISWGDYADMSQSFFIYLVWMTNTFIICW
SANELSDAADSVKGSAYEVDWIGAPIPFQRSILLMITRTNQSFVLTAGKFVPVNNETMMNI
FKESLSLFMFLLEMKDKRGE
>BgerOr78P
MQTDNLLIQKRFGLYLLYFQVSGIRICMKEKSKLYFAYEIIAIICFYGTFLACCIHILDAMDDL
KKMMASLRIAMVMGLIIVQHLYIMIHVYTLYIDWMGNIFIICWCANELSDAAERVKVSAYE
VDWIGALIPFQRSILLMITRTNQSFVLTAGKFVPVNNETMMNIFKESLSLFMFLLZMRNKR
HEQIVILCYGMF
>BgerOr79I
MTTGSPLLESRFGLYFVYLRLAGIPIYMKEKSRYYLIYQSFVSIVFYGTCLGFCIQILEDKHDL
KKMIASVQVSMASVFVAVLSFQIRIDLKSLEELLQLTDNFSWEEMPLRNPVSGDFTAKGWI
CVIRMVIKKTFVGADIIYITLSLLRMLYIQDSMVLPVWYPYNHTLSPIYELTNLSQNWGDY
ADMAQSAVMYVLITATSFLVCWPGTELTDKSESVKDAAYSSEWVGAPITFQKSILLIMTRA
NSEFTFTAGKFVPVNNSTLMNVLNESMSLFMFLMNVQEKQIAV
>BgerOr80
MKFNIGSPLVHNRFGLYLLCLRIAGIPVFMKEKSKLYILYEIFVNVCYHGMALACCIHVIEVK
DDLKEMLATLRIAMSVAMVSVLHIFIRIRVKEFEELLQLTDMFTWEEVPARNPETGKLTS
VGWMDMIRKIVKHALISTLIFHSTQSLYRLLSAQDSLVFHAWYPYNYTTSPGKELTNISQA
LASVFGMATIFAFPGLYATVVAIGCCQFHKLKMMMQVIFQKENSVDTENQLKKCIVLHQN
ILEYMSKMEEVLNNILGALLFTLMTNICVVAFSVIISWGDYVDMAQSIFIYVNWMTQVFIIC
WSANELSGAAEDAKRTACETDWIGAPVYIQRSILLMMTRANQPFVLTAGKFVPVNNETM
MNIFNESLSLFMFLLEMKDKHDVEINA
>BgerOr81P
MGNESQNGNPLIKNRFGLYLIYLQIAGIPIFMKEKSKIYFAYEIFVNICIYGTLLACWIQLLEV
KHDLKAMMATLRIAMAMGLAVLIHFYIRIHRLKFEELIQLTNDFTWEELPTRSPTTGNLT
AAGWMDIIRKVIKFSVICVLIFHAIQSMYRICTQGSLVFYAWYPFNYTVSSSYELTNLSQVL
VSVVLAPTFFAFPGIYLTTVAIGCSQFDKLKKMLKSINHKQSSLTDSKDELKKCVVLHQQIL
HPKZLLGCCSLLIMIIICLNWGDYEDMAQSFVIYINMMTTISTVCWPANELSAENVKLAAY
EVCWIGAPISFQKSILLMITRTNQPFFLTAGIFVPVNNVTMMNILNESLSLFMFLLQMKVT
HDEQD
>BgerOr82F
MEKELHNGSPLIKNRFSLYLVYLRIAGIPIFMKEKSKLYFAYELIVNVCVYGTLMACCIQLLE
FKHDLKVMMTTLRVAMSIVLGTLVHFYIRIHTLKFEELIQLTNDFTWEELPTRSPTTGNLT
AAGWIDIIRKVIKFTVNCILIFHTIQSLYRICTQGSLVFYAWYPFDYTVSPAYELTNLSQALV

SVVVTYGFCAFQGIYLTIVAIGCSQFDKLKMKLKSINLKQNSVTDSKEELKKCVVLHQKILR
YLIKMEEVLSNCLGAVLFLMMINICLDSFSVIIWGDYADMAQSFVIYTVLMATIFTICWPAN
ELSVAAENVKLAAYEGCWIGAPISFQKSILLMITRTNQPFFLTAGKFVPVNNATMMNILNE
SLSLFMFLLQMKVKHDEQN
>BgerOr83P
TGNNANVDSTLTQNRFGLYLVCLRIAAIPVFMKEKSKLYFVYEIFVTICFYGTLLSCCIHILE
TIDNIKEMMATLRVAMALGIVAVMRILIRXHTQGFEELLQLTDSFMWEELPHRNPKTGNL
SAVGWIATIKKISKYGIIFSCFFHFTQSFYRILXQDLVMFPAWYPYNYTVSPAQELTNFSQS
VASVLAIATIFGFFSLYSTIVAMGCSQFDKFKIMMQAICQKENSINTQNQLRTCIVLHQHVL
RYLSKMEEVLNISLGCILFLEMAILCIVAFAVIINWGDYADMAQSLFIYINSMIHIFIICWSAN
ELSDAAESAKLAAYEVDWIGTPISFQRSILLIISRTSKPFILTAGKFLPVNNATMMNIFNESL
SLFMFLLLHMKDKHDEGIKT
>BgerOr84
MERDLTRTRFNIIIFCMRISGIPIFIERKTKMYLAYEILVYVCGCSSFIACWLDVLLNQENLK
EFLASVRVSIQILISAWILFYMKFHVKSVENLLRFTEQFTWEELPQKDPENGKITSAGVALII
QKMVKFSLIAILTFHFIQSTYRMLTKHEMIYLSWYPYDFKASPAYELTNLSQVLASIVAATS
LHFYFGFYGVTVSIACSQFYKLKLALLNIRDKQEPSTEELTNCIRHHQIILMFLDEIELAFNR
SIFGILFVEMTVACLCAFSVTTNWDVYADLLQALLILAISMGGISMLCWGGNILTESAESVK
EAAYSVDWVEAPETFKRSLLLMITRTNKEFTLTAGKFIPVNNKTMMNIFNEICSLILFLLE
MKNRYIQVD
>BgerOr85
MEGDLTHSRFKIIIFLMRLSGLPVFMERKTKIFLAYEILIELCGYSTFIGSWLNLFLNTENFK
ERLVSVHLPLQLLIAAWIHLDMRLHVKTIESLLRFTEQFTWEELPQRDPENGDITSSGVAL
ILPKMVKYSSLGLLSFHLLQSTYRMLTKHEMIYIAWYPYDFKASPGYELTNLSQVFASIQSA
AIFCSYLGYYSLSVSVACSQFYKLNLDLQKMNAKQDFSTEELNNCIKHHQTILVFLDEIELA
FNKSICGVLLVEMTVSCLCAFSITSGWGEYADLVQALAVFVFNMAGISMLCWAGNKLTES
AENVKEAVYSIDWVGTTETFKRSVLLMITRTNKQFTLTAGKFIPVNNETMLSIYKETWTLI
LFLLEMKNKYVQVD
>BgerOr86
MEGDLMHSRFNIIIFCMRVSGLPVFMERKAKIYLAYEILIELCGFSTFIAFWLDVFLNTENL
KEFLASVHLPIQSLIVAWIHLDMKLNVKTIESLLRFTEQFTWEELPQKDPENGDITPTGVT
LKLPKILKHSLIGVLSFHFIQSIYRMLTKHEMSFVSWYPYDFMASPAYELTNLSQVVSSIQY
AAIFWSYLGLYSLSVSVSCSQFYKLNLGLRKMNAKQGFSTEELNKCIKHHQTILAFLDEIEL
AFNKSICGVLLVEMTVACVCAFSITSNVEYADLLQAIVLFSLNMATISILCWAGNKLTESAE
SVKEAVYSTDWVEATESFKRSVLLMITRTNKQFTLTAGKSIPVNNETMMNIYKQTWTLIL
FLLEMKNKYVEAD
>BgerOr87
MEGDLTYSRFKIIIFYMRLSGLPVFMERKTKIYLAYEILIELCGFSTFIASWLEVLLNTEDFN
KFLTSVHQPIQFLIIAWIHLDMKFHVKTIESLLRFTEQFTWEQLPQRDPESGDITPTGVTLK
LPKILNHSLIGALSFHFIQSIYRMLTKHEMSFVSWYPYDFMASPAYELTNLFQVFASIQYVA
IFWLYVGLYSLSVSISCSQFYKVNLDLLKINAKQDFSTEELNNCIKHHQTILVYLDKIELAFN
TSICGILLFQMTISCLCAFSITSGWGEYADLLQAIVLLAINMTGISILCWAGNKLTESAESVK
EAVYSTDWVEATGSFKRSVLLMITRTNKQFTLTAAKFIPVNNETMMNIFKEIWTLILFLLE
MKNKYVEAD
>BgerOr88

MEVSSRTKSRFDLYIFLLRAAGIPFFMKQKSFLFLAYELFMYASTIITLVSTWICVLEGKQDI
KVNMATLRVSMGALVIIPFNFCFRFQMKDFEQLTKMTELFTWEELPTKDPDSGEITSAGL
VAPLRKLVKYSFIGGITGHTFQCLYRIIWNHELIFVIWSPYDWQVSPAYEITNVMQMAGSL
MVFCSLCGYLGLYCTLVAVACSQFDKLKMNMLQVYHGEEETRDKRLNACIKHHQLVLQF
LDDMENALTAGMCIALLVLMTALCVIAFSAVTSVGDPIDMIQIIMLYIIWMSAICIICWFGN
ELTDKADSVREAAYDGEWIGKPISFQRSLLIIMTRCNKSFNLTAGKFVLLNNETMMNILKE
TLSLFMFLLEMKDKSIEEGM
>BgerOr89
MEENFPRTKKRFSLYVFFLRAAGVPIFMKRKWKAYIAYEIILYSCSVALLITSWLRVVNER
DNIREMMATMRIAAGMLVIVPFNFCVRFQLKKFENVIEMTEGFTWEEMPFKDPIMGTLT
SAGMLPLIWKIIKYGFMTMGYGHLIQSFYRIIVNHELMFRTWCPIDCHASIVFEVTNISHIM
TSILAACSFFGYMGIYCSLVAIGCSQFDKLKMNMLSIYHTEQYNSNMDYHLQACVRHHQQI
FQFIREMENVFTIGMCLTLLVLMTSTCIAAFSAVISRGEVEDVVQIIMLYVAWMTSICVICW
TANELSDKAESVKNAAYEGEWIGTSISFQRSVLLIITRSNKIFNLTAGKFVLVNNETMMNIL
NETLSLFMFLLNMKDKTVE
>BgerOr90
MGTKAQSESLLTITRFRFNILFMRVGGIPIFMQMSIGYFMFVMVWYFCTFVTLAGVWLDA
FLGHHDLKDILASLRMAIAFTVTVWVHFCLRLNVKSIEQLFRCTEVFSWEEMPSKDPSTG
QLTSLGYWQWFQKGLKYMVIVNWFMHLVPCCHRIFIEHRMMLPVWLPIDLKASPAFELT
NLCQFLGTVQAGASFYSLLGLYSTTMFIVYCQVDKLKMSFKNVKSEPSELNQNEIELNKCII
HHQQILEYLPKMESTLNICIGGVLFLEMACCCTCAFSAVISWGDISDMAQAMLIYFIYMTSI
CCVCWAGEQLSNRVESVRDVVYEGHWIEAPISFQKSIMLIITRTNARFNLTAGKFVPVNNK
TMMNILQQTLSLFMFLLEIKDKNADEKNTINRRG
>BgerOr91
MKDDIDDNEDFDIYMKICKVLGYAPSNSFLYRIYSFCVMFLGNACTLSILIDMWLYLGDLE
HVMMSARIGLPLSISTCIDIFIKLRNYDMREVIKHSTNFTTIDSGTGALIPKFRYLVTRGFPL
AYSIHCLFTIITIITKDGRPLAMNSWFPYDTSYTPVYQFTVIAQIVATGIHTYRFVVFLGIYFT
LVMIACSQLEQLRALILGNMKNSQIELNQCIRYHQRILEFINHMEDVFNITLLSQLLFIMGS
VCFSAFSIVNCNGNTELLGEAIVTIVVMLEILFIYCWSGEQLMQKSKELEEAIWESNWVGA
PVVFQQCVLFVGAASSKELKFTAGKFVPMSNNTMLNILQSCYSYLMCLMSMSSE
>BgerOr92F
MADDVATSALELYLKILNYIGYSSSNSLVWSVYNFGVIFLAESSVIGVLVDMWLYLGDMEH
LMVTARIAFPLSISTCINVFLMFRGSALKEVINHSSLFITSDYGLGALIPKFRNMATKGMTII
FLLHSVFTIITITTREGRPLALNSWFPFNATRRPIYELVNIVQIISTGIHTARIVAYLGIYFTLV
ITACSQLEQLCELLMKMENTQSELNYCVRRHQMILKLVRHIEDVFNLHLLSHFLLLMGSM
CFSSIAIVMGKDNTELLGEAVLTQGVMLSILFCYCWSGEQLRQKADAVALAAWETEWVGA
PIAFQRSMLFVISAASKEIIFTAGKFVSVSNVTLVKILQQTLSLITFLLSLSE
>BgerOr93P
MTLRRGLLKSKFLKRDRFAMATEKFRSRFKFLILLIQIGAVPLFMEKKSLYYKVYEVIALFM
GYSVLLVTLLDATFNNEDIQRTMGSLRIFFPCLMIIWLQTILRVRMHAVEDLFRCTDYFTW
EDLPTKTSDGRLTNAGLVPVTQKLVKGFIGFIWGIHSLQSLARIIFAHDMVFDSWYPFDVS
ETPIYEIANLIQLLGSVILQSIFAAFTGLFATLIQVACSQIEKLKIFLNNIDKEDMYRELLQCIR
HHQQVLKYMRNMEEAFSWCVAGIFLINIMSLCAIAFSAVQSWHDAVALIQIVAIYFSMISE
NWVMCIVGEELTQQASSVGEAVYAVDWIGQPVHIQRLIYFMIANANSGLKQLSAARFVPV
SKGTFVKFMNETMSLFMFLLQVQERGDG
>BgerOr94P

MEAEDINENKFHKRFKFLIFITRLGAVPLFMEKKPLYYKVYEVIALLMGYSCMVVTFLDAA
CNNEDIQRTMGSLRICIPSCMIIWLQTILVQKHAVEDLLHATDSFTWEDLPTQTLDGHLT
NAGLIPVTQKLLKCFIPFIWGMHTLQSLVRVIFAHDMVFDSWYPFDVTESPIYEIVNLIQLL
GSMVLQSIFTAFLGLFATVIEISCSQIEKLKISLKNIGEQDIHKELIQCIRHHQQVLKYVREIE
EAFSWCVAGIFLINIISLCVIAFSAVQSWHDAVALVQIVVIYITMLLQNWVMCSLGEELMQQ
MCIRDSHISFMIANTNSGLKQLSAARFVPVSKGTFMKFMNETMSLFMFLLQVQERDDGKL
IEKY
>BgerOr95
MKGRELSEKRFRKLFLMMRGAGIPLYMKNTSIFFKVYSYLVTVCMLCTIITIFIGTVTNSDC
LKRIMQNVRVSFAMLDMSVAYIFIRFFHDRIEYLISLTEEFTWDELPKSNEFRMTSWIERV
QTLGKYICIIVLSYHYIQSTVRIITLQEIVFDAWFPFDTSVSPTFEIIIFFQLLASVAIICIFAGYL
PLNCIFIAVACSQLENLAFELKSLKQNCEIEEQLRNWILYHQKTLRFVKAIEDMLSTYIGVQ
MIPLYLGPCLSAFSAITSWNDFTDQTQAFVAVSVFFCQASVLCWFGTQLTESAENVKRAA
WECDWIGAPIPMQKTILFIISRGNKDTEITAGKFVPLSNVTLLHMMNDCVSIFMFLLNVKE
RKERQHD
>BgerOr96
MTGSALSEKRFGVQFRLLRACGIPLFMKNPSIFFKLYSYLVVVCMTSLSITVLIGTVTNSDTL
KRFMQNARIFFGIFGVTVAFMFNRFFLDKVEYLINLTEQFTWEDLPKTESFRMTSWIPRV
QTAGKYTFIVIFIFHYVQSAVRVLTLEELVFDIWFPFDTSKSPTFEIIVFFQFIASLTVVAIFA
GYLPLNCALMAVACSQLEKLAVDLIELKQDHDFMLQLQNWIRCHQQALGYVKAMEDSLS
MYMGVQFIPLFLGPCIFAFSAITSWGDFTDQTQAFVAFTAFIGQACVLCWFGAQLTEAAEN
VKRAAWGCDWIGTPIPVQKTILFIIARGNTDVTITAGKFVPLSNVTLMHMINNCLSMFMF
LLNVKDRKEIA
>BgerOr97P
MKLIYTLVLFYFKLRMVRGEEGKSVTETRFKFLIQFMRVGGIPIFMETQTKLYKAYQLVLF
TCTITTIISSWLHLIYENTMNDALKIILLPIAFVFVAAYSIFIRFDIKSIEKLLKSTENFTWEE
MPSRDSKTGNLTAAGALGIGTIYFKRVILGLAIYQSLICVIQFFMRVMFFPSWYPYDACASL
AFELTFLSQLLGFVAQFVSYLSFFASYCLFVAIGCSQCDKINTSLLSLNHNHNSTSENHQEL
CKCIILHQQVIEYINKMEXHLYLCGALLFQVTTLGVSALAILINLDDRFAAVQALATYVTMSI
SAFLVCWPGTKLSGKVESVKTAVYDVDWIGTPISFQKSLLFITKMADKPFKLTGGKIKPIN
NETLMDILNNTYVMFTFLLNMQNKQSEKEV
>BgerOr98
MESQSTDLIMKRFKFIIYLWRFVGVPMFLRKPSMVYKIYQAVTWLLMVSFPIFNCLDAYE
NRDNVARAAISLRTAFPMCIMLMPHTAVSLNLELFETLFHKTDTLLWEDMPEKDPVTGQ
LTIAGWIPRTMTLVKCGIIGSIFAHAVQVTDRIRSHELTTYSWYPFDCYSSPAYEITNFVQVI
QALMAICSMYAFIGVYALHLEIACTQLDKLTSSLLAIRQTSDSQEDFKAIEQELNRSIVHHQ
QILEYMQALENAMNFSICTIFLFLMATLCIISFSAVKVLNDPVELEQVIHLNIVFTIYLFILCG
FGTLLTNKAQYIAEAAYGVDWVGTPLSIQSSILIIITQSNKEFTLTAGKFVPVSNKTMLSILS
EAWSLFMFLLQVQDPEANN
>BgerOr99NP
FYRTDVVNVLILTNSFTWDEKEAKDPDTGGLSIATWIPRIKKITRNLFMTMWALHASQTI
LRIIFSDKPPLTLRAWYPFDTTSNPGYGVAMFLQLYSSLVLSAYMYGFPPFYATLVCIACSQ
LEKLRINLSRVNQDQELLNTSIRHHZKIIEYMKAMEVAFAPCLLGWFLSITAGLCISAFTAV
VSLENFSLLMQSVLIYTVLIANVFAFCWFGSELTDQAQKVSDAAFGMDWLGSQIPFQRCIV
FMISQANKEFKLTAGKLMPVNKITMKNMIEQSVSFFMFLVQVKDPKD
>BgerOr100N

FYRTDVVNVLKLTNSFTWDDKEAKDPDTGGSSIAAWIPRIKKFAFNLFIIIWIVHVSQTAL
RIIFSDERHLTVRAWYPFDTTSNPGYSVAFCHQLYASLVLSIYMHCFPPFYATLVCIACSQL
EKLRVNLSRVNQDQELLNACIRHHQKIIEYMNAIEVVFAPCLLGWFLLITTGLCISAFTAVV
SLGNFTWFLQALMIYTLLLANVLEFCWFGSELTDQAQKVSDAAFGMDWLGSSIPFQRCIV
FMIAQANKEFKLTAGKLMPVNKITMKNMIEQSVSFFMFLVQVKDPKD
>BgerOr101N
MMVDACMHLGDLGHIIENVATLLPHLCGIWIDVFLRFRRPAIERLIYHTDEFVWEDWPAR
DEITGSLTMAGLFPRIQKVIRVLVLVIWGPHGVYMVYRGIMDRELLGFNAWFPFDTFSSPT
HEIVILIQVYCSFALTTNFLANLGLYASLLTVACTQLTKVQNGLIQLGSCTFMDLIKVQFFCR
SENILCLGKQYFRDCSLGNVGALYEIIVVFNGQMMLLAAYCLFGTELSTQAEEVKFAAYSC
DWTGCSLSEQKSLMFIMAVASKEFIITAGGVIPVNRETMLAVMNQAFSYTMFLLNFKDT
>BgerOr102
MSTKDVMGDARKRFPIFRRLYKLSGNAIFGDGKTILYKTYSYISRVVVYLNWLAIVVDTIFN
LNNLERLIENVSVILPHLCSVWLDIFLQFRKSSMEKILMETNMFAWEDYPLRDEVTGWPT
LRFVIVNVPKICFCNVWFTVISHSSYMVYRGVTSRDILGFNAWFPFDSTLSPMHELVIAMQ
IYYSFMMTAHFYNNVAVYSTIVSIGCIQLNNLLKDIHSLQLDDLDDRKKKTKFKECVRYHQ
KILNYLGIMQDSLNVLMFGHFLIIIIGMAFAAFSAVMSWQNLGAMFQLIVVFFGQMILLAV
YCILGTHISTLTEDVRFAIYESDWIGSPVPTQKSMVFMMAAANNEFKLSAGGFIPVTRETM
LAMLNQMFSYTMFLLNFKDEEE
>BgerOr103
MALYRTRHVSKYVEDYYGFFISWFWLSATPIIKYNRLSLYGIASILLMINAYVCVATIIIDIFA
VPHDLQYMVENTRVAVPASNALWFHFIISFHKSEITELMAFQWEGFETTVLGPMTLLLPR
IQPMTVKTNIVFFGIHSIYSFLRVILTKTRQLAVNSYWGFDVTPSPIYQLIFVAQYCMEWICF
MLFFGFTGFYAYTVATACSHLETLREDLIRTKESSDIKKHLQKCIRHHQKILAYVGLVEKVS
SPVLLGQFLLILGGMCLSAFSAAMSWRSPLHIAQAFLVYSSFVAQLFVYCWFGSELSDELQL
VSEAAYNSDWVGIPLSEQRSIQFIIVAANKEVLLTAGKFVPATRKTMQNVINQTISFLMFLI
NVSNDDVQV
>BgerOr104NF
KEIFQFLCFSFFYVIISTTSERNNYGGKFGTDPRFQILKRLFKIAGTPLFPEKESNLYILYRNV
VVVSGYLTLLTTFIGIVQNITDLEYVLGAARVSFVMINLIWMHFFISFNIQKVRNLLNMIGH
FTWSDLPLHDHEGSISMAGWIPKIQNLLWKFNFCDWGFHCLYLVLRGVSSGSYHPLFFDA
WSPFNTENIMGYAVVLLIQGFGSVMIGTSLFAVMGLYISSVAVACTQLQKIQAALVNIKQK
DETLLNDELILCIRHHQQVLRYIKLLEETFNPVLLGPFMSVVAALCFTAYAAITIAGRFVEII
QIFLISCAMVFQIQAFCWFSTELTIETVKIRDAAWHTDWVGAPSSFQRSVLFMIAVSKEFT
LTAGKILPVSRRTFMMVVNETYTYLMLLLHFVDTSTQNITYKEL
>BgerOr105I
MDLSETDKRFKTLQNQFVRVGLPLWHIEKRFYNFYKICIIFCGNTTFIGAVINLIINIDDKSY
ILENIQMNFSMFNCIWIHLILSYKINELRNILKIVEEFTWSEESSDFMSRWIARAQNFSDKG
SVLAIGCFNLYFVLRNVLSEGRKVALETWAPVDLRENLPAYIITVIMQGIACIMYGNIVFAG
VILYTVLVAVACTQFHKLHSALLNIRTHEISESEMSHQLAKCVKHHQRIYEFVKGLEDTFNI
AILGPVFIVAAILCFSAFTAMKTGRVGDVAWESDWIGAPIPYQKSISIIIAASKEFVLTAGKII
PITRHTFMTIMNQTYSLLMFLLKF
>BgerOr106
MVELLKLQRSILGGVCFLGSKGKWYIYLGVSCWLLVLMAQLYGVHKFWGNIDAMAEGIGV
ILMLGLLIVQAAHCLKHSDQLQHIIDFLENLFNQFENEECREIVLRTQALTNKVIKSLVPIFV
TDVSFWIFPPFIRYYFDEDKHGRPRADNEPFPYFCFIMDFPFDATHSPIFEVVFLVQLTCTT

VLALFDIALLTIVFSTILFTAAYLKALATMLRKLVDFEPGDKEIEDPEAYLNNCIRLHQQLLS
FSVDLQQYLSPILIFFLKPFELFLCIAVYEAVQMTDKVSTFVVSAIALLSGISVLCAFGEHLKE
QSELLEKEVTACRWYLHSTRFKRHHLLLLAQCQRTIRLKVGFFYPLTLETFIKILNTTYAYF
NLLQQLR
>BgerOr107
MAELLRIQKTILTYSSFLGSRSKWHVYLVVCCWLFLTTGQVYGIRSFWGNIDAIAEIVGIGV
LCIFLIIQAFHCLRNKDQLQHIIDSLENLFKQFENDECRQIVEKTDKLNNKITKMLVPIYVT
DTVLWFIIPFIRYYFDVDKHERPRADNEPFPYFCFIVDFPFDATHSPIYEFVILVQSTITAILT
LYNTAFLTIVFSTILFTASYFKALAAMVRKLVDFEPGDKEIEDPEAYLNNCIRLHQQVLSLV
SDLQEYISPILVFFIKPYEVFLCIAVYETVLMTDKASAFIEGTFGLLFCIAVFCSFGEHLKKQS
ELLEAEVTACRWYVQSTRFKRSHMILLAQCQRTVCLQVGLFYPLTLETFIKILNSTYAYFN
LLQQLR
>BgerOr108P
MAELLRIQKIIFTYTCCIGSIGKWHFYLAVCCWLFVFMGQIYGIHSFWGNIDVVAEIIGITVL
YIFLIIQAFYCLMHKDQFQHIINTLENLYIQFENEECREIVMKTNQFNNKMTKILVPIFVTD
VTLWIITPFIRYYFDENKHERERADNEPFPZFSYIVDFPFDATHSPIYEFVFLVQFTMTSIM
SLYNIAFLTIVISTILFTASYFKALAAMVRKLVDFEPEDKEIEDPEAYLNNCIRLHQQLLSLL
RDLQEYISPILVFFIKPYELFLCISVYEAALMTDKVTTYIEGAVGMLFCICIFCSFGEHLKKQA
EVAACRWYVQSSRFKRSHMILLAQCQRTVCLQVGLFYPLTLETFIKILNSTYAYFNLLQQIK
>BgerOr109
MAEILRLQKTILTYTSFLGSRSKWHIYLAVGCWLFVIMGQVYGIRSFWGNIDAIAEIIGVAV
LCIFLIMQTFHCLRHKDQLQHIIDSLENLFKQFENEECHQIVMKTTQFNNKMTKMLVPIFA
TDMTLWIIIPFIRYYFDEDKHERERADNEPFPYFCFIMDFPFDATHSPIFEFVFLVQFTITA
VLTLYNIAFLTIVFSTILFTASYFKALAAMLRKLVDFEPGDKEIEDPEAYLNNCIRLHQQLLS
LVSDLQEYISPILVFFIKPYEVFLCISVYEAALMTEKVSAFIEGAVGALFCIAIFCNIGEHLKKQ
SELLEAEVTACRWYVQSTRFKRSHMILLAQCQRTVCLQVGLFYPLTLETFIKILNSTYAYFN
LLQQLQ
>BgerOr110
MAELLRLQKTIFTYTCCIGSNSKWHFYLAIGCWLILFMGQVYGIRSFWGNVDAIAEIVGVA
VLCTFLFIQAFHCLRHKDQLQHIIDSLENLFKQFENEECRQIVMNTNQFNNKMIKILVPIFV
TDVTLWVIIPFIRYYFDEDKHERKRDDNEPFPYFSYIVDFPFDATHSPIYEFVFLVQFTMTS
IMSLYNIAFLTIVISTILFTASYFKALAAMLRKLSDFEPGDKEIEDPEAYLNNCIRFHQQLLS
LMSDLQDYIGPILVFFVKPYEVFLCISVYEAALMTEKVSAFIEGALGMLFCIAIFCSFGEHLK
KQSELLEAEVTACRWYVQSTRFKRSHMILLAQCQRTVCLQVGLFYPLTLETFIKILNSTYA
YFNLLQQLQ
>BgerOr111C
MAQLLRVQRKILSYLCFFDFKYVPYFLLGFCSWIFVFSGQLYGLYQFYGNMDVIVEGLGVTL
YFIMLIMQALYCMTHQSQLARIVNFFEYKYDELENEEARAIMMKTSNLCNKLCLALIPTFI
VDIIVWSAIPLAQYYDKSSKCHQERADDEPYTCFIYVLVFPFDFTYSPAYECAFLIQMICTFF
VMFINLGVMVLVFTTMLYCAAYFKVLATMVRELGDSDQFDDPNEEVVKTCIDNPEAYLD
KCIHLHQQLLHFLKELQYFIGPVLAMFLKPYELLLCIAVYEAALMNDKSTIFILTATVMLIF
VYIVCAFGEHLQQQGRLVEKEVLACAWYDQSRRFKRHRLIFMAQAQRDVLVHVGVFYPL
TLESFIM
>BgerOr112
MKRSLGVDIAIHLKIMFWVCLWVPSNTYIAKLLYGSFVLFTVVFVMSLLLAQLLYLVIEHF
DLILFVNSLTTLTSNIKILLIMTRFRFMKEELQDLIKNFNALRSNRKSVSESENKILETAEKE

SRVLTNGLLSGFITLFILMVAKPLFVQYTSTHHSNSTDLCPERSFIMLSWYPWGTCSTTIYA
AIYVSQIYALFMIIMQIAVYQTLNTSILIHIAAEVDIIYDKFISVLNQNNRKISEERNNKFPNL
DIDTNRSKNNEKLKQCYALNKDIVCAETNSTALKNNHIEGIVGILDNVSVAYECVKDPISN
MFSNDYDKSEEEGEQEEEEEVYNRLIEIVKHHQSVFRFSEEVNNMFSPFYLFQFSTVIIGLC
FASFLASMPSLDSETRMQFIIFTFLLMAQLLLPCWYGQRVTDQSERIRDAVYGCRWYDKS
PRFKKAVQIIIMRCQRPICFSVGGFAVISRETWLSLINFTYSLLAVLRQMDEDTLQEN
>BgerOr113P
MKRSLGVDLAIHLKVMFWLALWVPSESNIAKFLYGIFTLFTGVMACVFLLFQLLNVAYGN
LNLILLVHSLAIIAPSIRVVLIFIKLTFTRKDLKNLVQNFNTLRSNWKSSSETENKILESAEKE
SRIITNGLLSVFIILMILMSVKPFVFQYTSMNNTNLTDVHPERSFAVPSWYPWDFSSTTSM
FAVTYISHIYGIYLLTXAIFQAFTSAILIHIAAEFKIIYEKFISVLNQNKHAEESKIDFSRASNST
FDIKRNQNKNTVRKRRNEIREDISSEINSRNSEYANASVSNISSINYEKPENEDENEAYERL
IEVVKHHQNVLNFCEEFDSIFSSLYLLQFSSIIVCLCISCFIASLSYVDSGTRMQFINITGTAM
AQLLLTCWYGQRVTDQSELIADAVYGCGWYNASPRFKKAVHIIIIRCQRPVCFSVGGFAVIS
RETWLSILNFTYSLLAVLRQMDEDKLPEN
>BgerOr114
MKRSLGVDIAFHLKVMFWVALWVPSDSNIAKFLYGIFTLFTGAMVCGFLLSQLLNIALGHF
NLILFVHSLATIAPCVKVLLILIKFTFTKEDVENLIKNFNTLRSNWRSSSETENKILDSAEKE
SRLMTNGLLSGYVILMIFMSVKPLVFQDTYMNNTNLTDIHPERSFVVLSWYPWDTRSTT
MYAVTYISQIYALYVVIIQVAVFQTFNTAILIHIAAEFKLIYQKFTSILNQNTYNHAEESKNIF
SHASNSTCDVKRSQNKNTIRKRRCEIRKGIRSEIKSKNHVKEGGEKPYSEYANDSGSNIFSI
NYDKPENEAYERLIEIVKHHQNVLRFCEEFGTIFRSLYLFQFSTVIFGLCFSCFLASLPSVDS
ETRMQFIIFTGLLMAQLLLPCWYGQRVTDQAELIRDAVYSCRWYDESARFKKAVHIIIMVC
QRPISFSIGGFAAISRETWLSLINFTYSILAVLRHMDVDKFAEN
>BgerOr115P
MKRSLGVDIAIHLKIMFWAGLWVPSNSSIAKILYGFFILVTGVMAAALLLSQLLNLGLGDF
NLILFANSLATITSNIKILIILIRFTYMKKELQNLINNFITLRTNWKSYSETENKILENAGKES
QKLTKVILSGYAVLMTIMSVQPLVSQDNYINQTNSTDIFPERLFVVPSWYPWDTTSTTMY
AVTYISQIYAFFMVCMQIILYLTLTSSIFIQIAAEVDIIYEKFIAVLNPNIYKNSEEZKYKFSQA
SNSTVQNKRCHNNNEKLKRRYALNEGDVSAETNSSVLNENHLEETHEKLDFTSVSFEYA
NDPISNISLNSYDRSEEEEEEEEEEEEEEEEEEEEEEEEEEEEDETYDDYDDEAYRRLIEIVK
HHQYIFSFSKEVDTMFRPIYLFQFASITFGLCFSCFLAASPSSDSETKMQFIVFTGLLMAQLL
LPCWYGQRVTDQSERIQDAVYGCRWYEESTKFKKAVQIIIMRCQRPICFSVGGFAYISRET
WLSIIHFTYSILLVLRNMDEEKLSAN
>BgerOr116P
MNTFLAVESVSTFNFNMNTSLGVDVGIHLKIMYWGALWVPSKNTSXKFIYGLFILITGVTV
TFLLFCQISYLIFKHFDLILFARSLTTLTSFIKILLIMTRFTFMKQELQNLIDNFVTLRSSWKT
YSESEIKILEIAGKEXTKLTIVILSGYLILMTIMVTKPIIFQDISTNLANTTFMHIERSYXLSWY
PWDTTTMMYALTYISQVYGYFMLTLQIVVYLTLITSILIQITAEVDIVHNTFTSVLNPDNCK
NLEESENNFTEVTRFTVDIRGDQRKKRNSNKNIVKIKILMLQSQIRQLLKGILGXEYGNGS
MSHSSSMSYNKSEVEKKEEIEDETYKRLIEIIKHHQDVFSFSEEVDTMFNPLYLFQFSSVIF
GLCFSCFLASSPSSDSETKTQFIVIAVLMVVLLLLPCWCGQRSERIGDAVYGSGWYDESPRF
KKAVRIIITRCQRPISFSIGGFSDISRETWLSITNFTYSLLLVLQNFDKEKLASN
>BgerOr117F
MLISGNISMTSEELPADSKCNKFRTSGIQLKINRKILWAMGLWYPRKARSSQYRDYWLFS
WFMTFGLFAHISTEIVAMYVYSNDIRIMLNIFCTMISGLGAFYKALYTVYRKYQMQDLVNK

LEGSTFAFDHQIGEEQQRIIRSACRLSNFITALFEILCVFIVTLWMIAPMVFEDPTSPLEQPL
PLPASFPWDLRNTMGYSAAYIFQLESLAVTLEKLVESLNSEHKRISIRDISTMNRSSERKIQ
EKAERRETSQERNRHSGELKSHQDTYAISENLTNEDLIQEFTERELESYQKLKECIKHHNE
ILSFAEEVQTHFGPVMLFQCGSSTLILCFVAFLLTESSGDIGTLIKCIMYLVVGTFELWLYCV
FGDRVIELSGNISNAAYGSEWIVMSQRFKKALTMMIMRAQKPIIFKGGFLYTVSLETFMSLI
NASYTYFTVLQQAKE
>BgerOr118
MVTGSPTASSLEVNQESNKKWLRAKQFHLSFLNIIGILPSQSISSNKYKLFIYNIFCVINMSI
KFPIAILVFCNISHAWGDFSDVSDTVHITNAVSLDGVMFTYLFFKRKELENIFNQMQSKFM
KITEQIVSPQIYQTVVDRTTRRCWILSTIFITWLSFMIAVWTVLPFSLLFVPHLMEVGSTLK
NETSYVFMLPMWWPEELKMTPIYEIVFVIQLFETFYCLTIIAAFGTVYLYIVNGIVMRFEIL
GYCIEHTEANIRYWLENASEQSGNIENKLTNSDNQIKFEERERLQQLEKGIHEVDNNLQQ
DFEIEVSLINQLMDTNKTREEEEEEEEEMFRIYLLQWIKIHQDLLDYCEEIKIFFNVLLMAF
FTTISLNMIFLAFGLAQDSRNSIVAVTLSAIYAFGCPYLLCLYGDKLTEASLNIKDAAYSCEW
YTKSPVIKRMLQMIIMRAQSPVVLKAGWYSISLEQFSEIINTVYAYFNIMRENANKN
>BgerOr119
MKKGSDSLNTSIREGSCELDLMNLQQKCLILAAIEPSNEIKINFWKSILFRIYQIIIVSTYIPLL
VIQIFGCYHYRYDFLTLLDGIVPIGLASIGFFVPLSANWKIATELVRKFERDSVFIKAINQNN
TKNINLLNEAQRFTKFLTCTLLVCITLSAGIWFTQPHIFAVLESFTNKKENSNSTIIMDPLK
MYPLVIWIPYVDITATIPYMFISTLIGTCIFIVSTRGATFISYSVSIMIYTTTQFKMVATCMNE
IDNVDDNELEFTDNNIISNNNGLSKQVLNCPNNKSPDELKRTHEEHLDDVKKQIKRKKFN
VYKNIKLQDFEYEKCLLCPEEDEVKAITQLINSVKDHQQILKIINEVNDAFSSMFFMTIMLG
ALSLSLALFVVAVNPDFSNKLKNASAVMVLLIYGWIIFSNGEDVKEEGIKIHHTAYNLQWF
KHSTKFKKILQIIIMRSQKPCGIMLGPLMDMTIENYSNILNTAYSYFTLLVQFESKDSEK
>BgerOr120P
MKKENDSLNTIVREGACELDLMNLQQKCLILAAIEPSNEIKINFWKNILFRIYQIIIVSTYIPL
LFLNICGSYHYRNDLLTLLDGIVPIGLASIGYFVPLSAKWKIATELVRKFZRDSVFVKAINQN
NTKNINLLNEAQRFTTFLTRTLLVCTYISAVMWSIQPHILAVLESFTNSKKNLNSTIVDPLK
IYSHVIWIPHVDITANKPYMFITALIVTCAFVISTRAAAFISYSVSIMIYTSTQFKIVSTCLKEI
DNMDDNELEFMENNIISNNNDLSKQVLNWPNDEPPDEFKHFHEEHHDGVKKRIKRKKS
NVYKNVKLKDSEYEKCVLCPEEGEVKAITQVINCVKDHQQILKTIKEINDAFSSMFFVTIMI
GALSLSLALFQIAANPDFSNKLKNASSGMAILIAGWIIFSNGEDVKEEGINIHHTAYNLHWF
KHSTTFKKILQIIIMRSQKPCGIMLGPLMDMTIENFSHILNTAYTYFTLMVQFESKASEKW
DHTST
>BgerOr121P
MKKGSDSLNTNIREGSCELDLMNLQQKCLIFAAIEPSNEIKINFWKKILFRFYQIIIVATYIP
VFFFNICGCYHYRHDLLTLLDGIVPIGIASIGYFVPLSAKWKIATELVRKFERDSVFVKAINQ
NNTKNINLLNEAQRLTTFITRTLLFCTLFSSGIWCIQPQIFAVLESFTNSKENSNSTIVDPLK
MYPLVIWIPYVDITAIKPYMFITTLIVSXCIFIVSARAAAFISYSVSIMIYTNTQFRIVATCMN
EIDNVDDNEPEFTENNIISNNNDLSNQVLNWRNNKPPDEFIHSDEEHLDGGKKRIKRKKS
NAYKNVKLKDSEYEECVLCPEEYEVKAIIQLINSVKDHQQILISIKEMNDAFSSMFFITILFA
VLSLSLALFVVAVNPDFSNKLKNASSVMAILIAGWIIFSNGEDVKEEGIKIHHTAYNLHWF
KHSTKFKKILQIIIMRSQKPCGIMLGPLMDMTIENYSNILNTAYSYFTLLLQFDS
>BgerOr122F
MKKGSDSLNTSIREGSCELDLMNLQQKCLILAAIEPSNEIKINFWKSILFRIYQIIIVSTYIPLL
VIQIFGCFHYRYDFLTLLDGIVPIGLASIGFFVPLTAKWKIATELVRKFERDSVFVKAINQNN

TKNINLLNKAQRFTKFLTRTLLVCTTFSAGIWFTQPHIFAVLESFTNSKENLNSTIVDPLKI
YPLVIWVPYVDITATIPYMFITTLIGTCIFIVSTRIAAFISYSVSILIYTSTQFKIVATCMNEIDN
VDDNEPEFMENNIISNNNDLSKQVLNSLNDKPPDEFKHSDEEHLDGGKKRIKRKKSNAYK
NVKLKDSEYEECVLCPEEDEVKAITQLINSVKDHQQILTIINEVNDAFSSMFFTTIMIGALSL
SLALFVVAVNPDFSGKLKNASAVMVTLIAGWIIFSNGEDVKEEGIKIHHTAYNLHWFKHST
KFKKILQIIIMRSQKPCGIMLGPLMDMTIENYSNILNTAYSYFTLLVQFESKDSEKWDHGST
>BgerOr123
MNTIKDSKIMREDNIELPFCKRLETTYEIELMGFQNRCLLYLGILPLKDTNEIWKIKLYRFY
QVLGYIMYIPVILMEIYGCFHYRNDLNVVIDAIVPIGLGCIGFFVPLTVNWDNVLNHFEKME
TKSAFIRIINDNNRKKIAILEEHYHWSRFFTKTFVLIANITGITWGLQVYLLEFLETLTGEN
HSNSTINEEFPALKLYPIVVHLPYVDMTDTVICIILNVIIMILIIMTCNRAGMTIVYFSTLLLY
HSTLFTLVAVSLEEVDTEDSINIESNFNTSHDIGQPVIRKENNRFQDSFENYVLQKTQCNN
LDIVNKRPRPIKSSMFNYSDNENQTEIANEEQEIRAIDLLKDAIKDHQEALQYVDRVNDLIS
SYLFSEISVGAVVLALALFESVVNPNFAAKAKHVASALAVVIGGWIVFRSGATVTDQGQKV
CEAACNTLWYKHSMKFRKLLYILIWKSQKPVIVVAGPLIEMTEKNFADVVNGSYSYFTLLL
QFESTKS

545 BgerGr proteins

>BgerGr1
MDLTKIHNPAYIQNGDKTNSFQNAMSSILITAQCFGVMPVTGITGCSAVSLRFRWCSFRTA
FCIITTLGAFLNMVFSFALMKSGLNFAQSDNVVFFGGVSIMYLLFLQLARHWSTLAIEWEN
LELSQKHYGYPKSLKLKIKIIATIILVGALVEHLLSKASRIAAAAKNCNGVSEVLHNYFTNTA
TYNQIFVVMEYNAVTAILTILSNFISTFVWNFTDLFIILVSVSLAARFRLYNRQLQSLRGKM
FPESFWIKIREEYNSLSYLTRKLDSCISKIIFVSFGNNLYFICRQLLFSLSPMEGLIDMIYFCCS
FGFLLFRMILLSLYASSIYEESKKPRHILYSVPSESYQTEVSRLLIQVVTDNVALTGLGFFYVT
RTLLLTLAGTIVTYEIVLVQFNNLNSVGNSSDPKINATEVCHCQQ
>BgerGr2
MDSSEELIDKNTSTSASNSAVEAKGRLQKLVDSPVVNPLSSNMLNDNMESLKAKDEYND
RGNSFQNAMCSILVTAQCFGLLPVCGITSQSANSMRFTWRSFRFFYSIIGIFGTCFNIIISILLI
QAEGLMYERVDKIVFYGTSALMYFQFLVLGKNWPNIASEWEIVEESQKHYGYPKKLRLKT
KILTAVVLLGALVEHALGHVNILRWAGCCQGDISGMLKRYFVTVHSQLFKFIPYSVPLAVT
NALLNIITTFTWNFTDLFIMLISLCLAERFSIFNVNLEKVKGKVLEEKVWKRLREEYNSLAI
LTKLLNSHVTVIVLISYASNLYFICRQLLSSLNPLGNSTEVAYFYWSFGFVLFRTITVSLSAA
RVNEESQIPKSVIYSVPSESYGTEVSRLIQQITTDNIVLTGMNFFSVTRTLLLTLAGTIVTYEI
VLVQFNNINGAGSSTEGNSTNTGTANGTNS
>BgerGr3
MMMRDLPNGGKSGNNVKNSNEDANGFHRVMKPILITAQCFGLMPVSGIASKQASDLRFT
CCSIRFVYCIFSLAGLMTMTVLNALEVWNNKVSFATARGTIFFTSTTLNSLLFLQLASNWR
ALVLEWEFVEQKHIHYGYPRNLRCKINSTTILILLMAAGETILANINAMMWATTCERKGE
GNLIYCYAMSSYKYIFKYMNYSTVVAILAIFFNTIATFTWSFIDLFIMLVSISLTERFHLLND
HLRYIQGKIIKEASWNRLREEYNNLSSLTKILNSFVSKIILLSYGTNLYFICTQLLNSLTPIDS
TLEKCYFYLSFGYLLLRAGISCLCAAEIYEESKVPRDILYCIPSGSYCAEVLRFERQVTTDEVA
LSALNFFSVTRSMLLTLAGTIVTYEIVLVQFNNINTGGSCSITSTAAPPVTTVPNTNAV
>BgerGr4
MKELILFDSEFLLSRRKSKTDGDFTVCRGLPDYMGTNVSSSIVRYDKSVTELSPHNQIKINR
GSVSESDSFQHTIRPVIRIAQIFSLMPVQGFTGPNASSIRYRWFTFRVLYSLLVIFGLLTMVV
FCGIRFVKLGADFQNTPCVVFFGCATAVAILFHQLAKTWPLIATKWEETESALAHYGYPK

HQHLRFSIITSGVLFMALVEHCLSYSRILRLSIRCGKKEEFDGFKTFSVNSYPHIFDFISYKL
WNAVFALVLNVIATFSWNFMDLFIILISIALANRFRQINSLLRSIKGKPLPHTFWCQIREDY
NLISSLTKIVDNAVSRIVLVSFGNNLYFICLQLLHSMHTPPQNTMQTIYFYLSLYFLLFRAVA
VSLCAASINNESREPRSILYSVPSATYSEEVHRFLHQVTTDEVALTGYKFFSVTRTLLLTVA
GTIVTYEIVLMQFNKMTDDERDSVETNVTNKFC
>BgerGr5
MMKFKKESVSELDSFHRATNKIIIIAQCFGLMPVIGIKGQNASHLRFKWLSLRVLYTILTLM
GVSFMTAMCIKRFYKYDRSFKETRECSVFYGCSFLRVLLFLILAKHWPILSSIWEQVEIDLA
HYGYPRHQHRKFGLITALILFVAFLEHVLSHVRVLRLAVLCAVEDKMDGICTFFFNSFPHV
FDYVPCSLWSGIIIFIINMECAFGWTYIDLFIILISTALADRFKQLNRCLKSVEGQTLSPEFW
HQMRMDYNTLATLAKTMDSYISKIIFLSYANNLYSICMQLLNSLHPPENAAQMFYFCLSFG
FILVRIAAVSLCAANVNEQSNHPKAFLYSVPSSGFSIEVDRFLQQVTTDDVVLTGMSFFSVT
RTLLLTVIGTMVTYELILVQFSGIHADERDLAAHSVAKRYCF
>BgerGr6
MIYGSGGILRRRPTESRMTPKFIKDIPCGNSMNLNSMHTSMGTIIVIAQCFGLLPVQGVTGK
NVENIRFSWKSFRVLYTLLIMVMMVVNMVFTIARISSIDLNFQTAGAFIYYLIAWFCCFLFL
NLAKKWPDLSLLWYKTERSQERYGYPKNLNKKLRIMTSVIFFLAIVEFLLAQLASGLIASY
CSTGGMDLLRRYFIVYNTHIFLVTKYNSALAIFAFASNLLAAFYWSFSNLFVMLICVGLTSR
FHLLNQHLNEVRGKVMAESFWRGIREYYNGVSYLTYIVDTHVSSIIFLCFATNLFYICQQLL
YSIVPSKHMLRSFYVYMSFGYLILRTIAMALYASRVFEESKKAKVVLYAVPAQNYQVEVHR
LLVQVATDNVALTGLNFFPITRTVLLTLAGTIITYEVVLVQFGNASSEPTNSTELCLNLTEF
AITM
>BgerGr7
MQMSMSFVIVIAQCFALFPVQRITSSQADDIRFTWRDFRIVYAVFSTSAMTYNLFLVIYWLI
KVGFNFKTAASLVFYLNGLLCTLLFFKIAMKWPEVARLWQELELRQLHYGYSRNLRFRIKI
FTIFLLMVALVEHLLSHLYQLNIASRCSNGFWDYIRRYLILYHNQIFEVIRFSPYLGALTSISA
FGAAFYWSFPDIFVIMISIALTSRFRQLNKQLILVRGQVKKEEFWKEVREDYGALAFLTKT
LDSCISGVVLLSFATDLFYLCQQLLYSMNSTYTTIQEVYVYTSFIYLAARTTVKSLCAARVYE
ESKIPKYILYSVPSESYNAEVYRFLDQVTTDDVSLTGLNFFPVTRMALLTLAGTIVTYEIVL
MQFGTASGASPSANLTQLCLNNSRDHRF
>BgerGr8F
MSLVLVIAQCFSLCPVKGITSHQAEDLRFTWRTYRMYYSIFVTIIMFINIIFVILWFVKVGLN
FRKAGFLIFYLNGFLCCIIFIKIAMKWPVIVQLWQTIERSQLQYGYPKNLRFKIWTLTVVILI
VAVVETSLSHLSNLHQASQCSSGVTDLIRRYFIIKDRNVFQIVKFSPVLPVFTLFSNLMAVFY
WSFTDIFIIVISIALSDRFKLINKQLMHIRGQAMTQTYWKGIREDYEILAYLTKVLDSGISSLI
LLSFATDLFYICQQLLYSMNHIMSSVHRAYIFVSFSYLTVRTCAKLLSAAKVYEESQVPKEV
LYCVPSEGYNIETHRFLCQVTTDSVALTGQNFFTVTRTVLLTLAGTIVTYEIVLVQFGTTSE
GVDVNLTELCFNLTRSLDR
>BgerGr9
MITAVFERPSSCPRIFHFLPFCINILKHYLFISGPYRNSMIERNNDFIVTRRRVTTGYNKIPF
KELNNAQTRDLKRERGTSRFQRSMFLALFVAQCFGVFPVIGITSKKAQDLRFSWWSFRSIY
SMAVIILMLISIGFIVAWLNMFGFSIITAGNLIFHLCALVHCMLFIRVAAKWPDFVILWEKT
EIRMKKYGYSKYLKLKMNTISIIILGMALIENVLFSSKTAQKASSCSSDTAAIIRRYLVLREP
HVSQVVGYKPVLLVLATINKVIAYFCRSFVDLFVLITSAAVSNKFELFNNHLKHFSGKVMPI
EFWKSSREDFNALSTLTRQLENLVSPIILIALFSNLFYICKRLLEEPKSSATVADIVYYWVSF

AFLIIRIIATNLYAAGIYEEGIATKDAIFSVPRNHYNLEVHRFLLQIKTDDVRLTGMDLFPIT
RTLLMTLAGTIITYEVVMISVVGNKEESNLNATSIIDESVNITSLCHNLTLIIKD
>BgerGr10
MLIMYAGHMRSREANRLSFIKLYNSNTDVNPSITVLQKNDFQGATRLLMFSAQCFGLFPIQ
GIRNNSRESIRFVYLSVQVLYSTLIMLGLLSINGFIIIYYIKGFLNFNNTVLLSFYGSGLLCCML
YMRLAANWRDILEAWHEVEIIQKGIGYPENIHQKIKKYIVIIISFSLVEHSLSWANQILLEIN
CSRSISEAITRALIHAHRHIFEFVEFSTVIGIVIEVLSFFATFYWNFNDLFIIAISIALSSRFKCL
NEYILEEIGKAKSQEFWRKIRIQYNALSNLTKYMDSCVSGIVLLSFGTNLYFITQQLYNSMV
QTRGITETVYLVFSLGYLIYRTVVVSLFTASIYEESQATRFPLYSIPPEYYRIEVHRLLFQITT
DKVALTGFNFFTITRTLLLTLSGTMVTYVVIMVQFSGTEDKSSNHTDNACISFSG
>BgerGr11
MKGLKVSPEGIISTKTIHDVMDVPRRRITLGVIEVKPEVLLKETTIENNLNSMHRAMMVVL
VVAQCFGICPVKGITGEKAEGLRFTWASFKIVYQCVALLGILVSLGFCVIWFGKIGFNFQSSS
DCVFHLVSVMCCILFLKIARRWPQFALMWQNIERRQIIYGYPKNLRKKIRRVSSVILTLALV
EHLLAQNSSWGTAGCCTSGTMERIRMFIILSRVQVFQVFGYNFLTAIFTVFINTATAFYWS
FIDLFIIAISMCLAARFQVLNQHLKTAKGKDMSEEFWKKIREDYNDLSLLTKTLDNHISGV
VLLSFSSNLFYICLQLYNSMTNTVTLVETVYFYVSFGFLLGRAIAVNLYASSIYDESISAKNV
LYAVPPNNYQVEVHRFLVQISTDSIALTGLNFFPVTRTLLLTLAGTIVTYEVVLVQFHTPTG
NTFKNISEDCIQLHYLL
>BgerGr12
MLGHPSLLRSTEGIEMRALSRTRQNVWERTDDRNSFQRAVCPMLVVGQCFAIMPVDGITS
RKGSDLRFSWLSIRVLYTFFSLCGTFALFYLQTWDLMSLPSITYSACAGCIFFSITFVSVTMF
LRLATKWPDLVSKWEELEKDMLPSCGYPKNLAFKCRMTSAVLLTLSTAEHLGAIISEIIRA
LPCAKGGILDIFRTFYMVWFGTVFRHTKYALWKALILQFSNFLNTFTWSYMDVFVSVISIA
LVAKFNQINERLERMRGKEAPWWQWREIREHYDKLCSLTQTVDDHISHLILLCIFSDLYF
VCLQLFNSLQNVKSLYGYIFFSYSFGYLLVRAITMCLSAAAVNEASKIPKHALFSVPSKSYNE
EVDRFIDQVTTGEVALTGFRFIQINRRFLLTVIGSIATYELLLVQQNIGGNPHPELSVNATN
TSFGGCPVFD
>BgerGr13
MLRQSNVKMNANIPTPLTTKTDQKKGEEFELQNGNKMWNFIKSQKWTIVGKLNLKETS
TKIKIENEKEMQSSFHSAMGPILVMAQCFGILPVRGVLNCRPHQLRFRYFSARVAYTFLFL
LILSLTACFSVGYFFVYVDRDGTRVLRNLRTAVWGGIFYGNAFLSITLFLKLASKWPNLMI
KWYDMEERLQNWAFPSQRKQFYTITIVILITAFCDQMLHTFLSVPEYFSSNEGNLNNPCP
YDKNFKVNDSSPTIKEIFQVHMCRTHWFIFESIPYDEYIGAVLFVSSKLALFIWNFTDIFIILI
SSGLTERFSQFNERIRTTVNEKTDKNEWKAIREHYNILSRLVKNVDEEISGIVLLSFANNLC
CLGLQLMKTLSDDSIALERIYFSLAFAILTARTVFVTLSAARVNEESKDSLPYLYDCPSDAYC
KEVKRLQTQMTTDEVALTGFRIFSITRSLILVVAGCITTYVVVLLQFYVAIQDMKTMMEEK
MNEANITNITSI
>BgerGr14N
NKEHFHISIRWILKTAQVFGLLPVRGILEQDPNKLEFNLISFRVVYTTLFLICLLAIETLAMV
ELCDSVQKHGSKVLKNLKDVAGATLFYGNAFIATFLFLKLASRWKTMILNYWYEVENNV
KDFKFCNLTRRFSLMAAFIMVFALVDHILSLRVNYEKAIGNMRDGSDLFEEYMKESHPFIF
KNSAFIKYNPYLGGIITGCCTIATFIWNFTDLFVILISMGLSQHFVHFNDCVYKARQNASER
EWKNLRLHYHSLSTLVKNTDKEISGIVLLSFMNDTCWIGLQLLNVLIRCNNMERVYFSVSF
LFLLVRTIILTRYAARIHEESRACLLDIYSCPSENYCIELQRLETFVVTDEIAITGLRIFPVTRN
FIFVVCGCIFTFTVVLMQFYIALNGENTSHSNKI

>BgerGr15
MPPPQRKVASELPQDCGAEAKKWSQFGEDFFFKDFLGGQKELKTSSDDSEPTTEFFDELK
PLLLSMKAFGLFPLQRRNSDESNFRRCKLSLLYSILFYVGMSLYAFYVSRVRIEYLRSSETQF
DELLFSVVHFLYVLPHFYLIPCHWIEVTKVGRYFTHWYEFQNQFQHVTGKPLYLGQRKNA
LLSVVLIPIVSVIYIVTELLLVNPKHMIWEIGFYFYITVIISLHIGWWWVICSALQGAMDAFN
RNLFKDCSIFKHETSAVMVAEYRSLWITICKLSRETGYFMCYTYGHLTIFAFAIMTLQLYGA
LSNLNDGMYPRHAGLAITVLLTAVMIYTIANSAHHASDQISDVFSERLSRVCTTDKQVRRE
LKIFSQVISVNSPAINLGGYARMDRGFLLNFASQMVTYLIVLLQFRLGILSLNPSSNSTEQTT
HSSVSQLLK
>BgerGr16
MFTLSDEMDRLFYRREKGKQTPPQAEDYLEAVRLKNPEDYNPIRKEIRFPILMLHLLGAL
PFQLNHKAEVRIVSLMSLYSLAVVSVMTTATVVVMSERYRIMTSTASSFGNKVLEAASIIFF
IPAVAVPVTHWYEAHKKAAYINSWKHIQVEFHKVTGKPLLVNLKATTRKVKASVAIIVAV
LTTLLFVAERGFVWWQGLAFAYACCLCAVCTGAWCIMCRSLMEVADAIKAELNQFLQRK
KGCNYPEKLRQYKTLWLSLSDLTQEFGNALGYSYGFQMLIYFVQQVLAVYGLLAELQRGG
GFDAVKATFVASAIMFTYAIFMICDCSQRATEQVGNVFQRNLQRLGNFYSFSFDEARYEIS
LFINAIVANPPVIKFAGFVEVNRGLIGSLMAAMVTYLVVLIQFQMTSISTCTANMTDFSQ
>BgerGr17
MFQETSSYARTSLELTSDEVRPVLNYNFKGEPWAVPSRKRNSHVYLENLKPLLFVAKALG
VLPINPDQNGEMQFRLFSLGTLYAGAVQVLMLSLTVFTIFERVHRIRSEVLDFGDLVLEVG
CIICCSFTFLAPMNHLPEAKEKAQFLNDWKTLQDNFHRVNGRPLDFNLRRRIVVNLGVCT
AVAAGFTSILFGLNIGYKWWQGIAFFYSSFLSFFMCVYWVFTMDAFVIVANSMAENLKDK
MKTDSSVQGKTVEAYAQLWLELSKLTQDMGGGMGYSYGGLITLYFSLQLIGTFGFISQVNQ
DRVTITLGFLSTSIIYAVLVFLLCSAAEKATNQVGISFQEKIRSIIFTSPMNDSTKTELYSFMR
AISAYPPVINLRGFVQVNRSLQTSLMSATVTYLVVLTQFQQSS
>BgerGr18
MHPQQEVPPSFPTHMRERKWKGSQICPGDSPIQQEIRHFFHEIKPLLVMYRLMGLLPFHI
NEAGDVVIKVSSPLMLYTVVLQAGMTACAFIVCKHRIDFIQSEKPDFSRLLIETTFIGAFVP
MILSPFSQLPEVCKKACFLNDWIKFQKCYLSSGGKPLNTSLGACVKVMLVVSVLIAITITVF
PFVTLDNMELWHSVPFFYSIYICILATVMWYINSWALVKAARILTADLEEIVVTKDYNCRE
MIAEYKVLWLQLSKVAQGQGTSSGFTYAAQLVAYFFEQVLAVYGILTNIQEGLVVTNLVCG
ASTVIFSTLIFVTCNSAQMISDEVGTNVQEKIQSLCHRIPLHCAEAKQEARLFVETIIENPPTI
NLAGFVAVNRGLQTSLISTMVTYLVVLIQFQMSTEAPGNSTFTEPPTTETTRPS
>BgerGr19
MTYFQEGRNFHTFQEEFHNAGISNRSVKRTTTHLKTVSPENTATSQEYSTFYEEFKILFW
MARALGVFPYHVDESGELSIKLCSIELLYSVAVVTCVAVTSAYVTIDWKSHLKSGINFRESV
NIYTLWLFIDSYVLIPLNHWPEMWKKVRFINNWKKFQHNFAETTGNPPRLEMRRKLAT
ALVCYAAIEVFVVAQICLGNLIHQWWHVFIYLHVFALLFLMPGIWVFTCVSFSKMADLLSA
EIKKDARSPASATAHRLKQYRKLWSRLSELTEELGTSSGITFGGITLLLFTASLLFAYSFLVT
IESHFNYALFCVIVWINVVIFAQCDAARAAMDTVGSSFSKSIEALTENLPANDVANIQELRI
LLSTIARNRPVISFANFVTVNRGLFVSLISTTMTDLIVLVQFQISDDQDTKKQNNTS
>BgerGr20
MYSGGSESFRNADEKKITVPFGNVQRSVFYRDMRPVILLMRVLGVFPYSVDDAGEMQFRV
LSVVVAYSLLLQASMMAMAGCVLAERVGSILEQKAAFIGHLLDGGFIAVISTIFFIPLTYWL
EVTKMLRFIGNHAVFEEEFRGLMRRDVQSFLKRRVCVFMCIAAVVVVLLEINGFVIERGLS
RWWHGIPFGFSITLMVLMPFVWTSTCRELIVIARDMAQHIETHVNEFAWSVRAAGYRKL

WLKLSYLTQELGQSVGFTYGTLNLIFFTIELIATYGVLSEVSKGFTPTVMVLLSASILFPALIF
IQCNMAQHATDEVGSKFQLKLENITSQVVNTNRTVTCNVRSFLKTIALNPPIITSSGFVDV
NRTLLVSFLSAKVTYLIVLIQFQMSSPNSLSQNKTNEARQ
>BgerGr21
MSTKGLGISTIHCQRQKRPFRNNFVEEETENVLFREMRPILFTLRLIGTLPFGVSPSDGELT
FNCNSLTTIASVILQVLVSIGAILRSKHLLQNIQQYAFDTRVWMYLGAFTYLSRVIMVPLVQ
WPEVHKTVRFFNEWKSFQLRYSQVTGRSMKLRLYKKAWIYLLGVATYVALQGCPLVASG
NGLDGFWVTPLLLFCMFSVHVMNIPWMLLCSALQSGAKCAAHEMEKHISACSFGRGEKIS
KYRELWLQIYRLTRNFGNAMGLTFGGHILLLFTAQVLFIYYTLSKFSSDPTAGDLAVKCFF
PFTFTVYLLGLCNTAHGAVREIGTKFQDRLQMLEIKYATTNNKALTEIRRFYRAMARHQP
TICLCGFASVDRGLQAALMSALVTYMIVLVQFRMEATAISQPGCNNMSSSTD
>BgerGr22
MRRPDEEEGKVDSRRSRRRRRKRRQLWRHEATGNAFYEELKPIFLTLRFMGTFPYSVSSS
GALVFEPLSPSTVYSVVLQLTLTVGATVRERSKVESIGHQTGLEGRIWVYLALILYVATTVC
VPLLHWPETCKKVRYVNSWASFQEEFARLTGKDLVLGLRRTSMACLAAVSVYSVVQYSPL
IVGEKPSALLWVGPLILVSTLFINGHTVPWVMMCSALGTAARLLAQEMELQVSVRGFGNIS
RFRQLWLQLHRLTRDFGSSMGLSFGGHFLVYFSGQVLFTYYTLSKLATGPADLVFRWALP
LMYSLFIFAFCNAAHKACNEVGHNFHGRLQALEVIYPSMNKEAECQVQRFFEAMARHPA
KITFSGFASVNRGLQTAMMSALVMYMVVLVQFDIESSTEEEPLNCTTTT
>BgerGr23
MPYHPRWQQRKTSPRDLFMMKVNAAYHANGGNALLKEMRPILWILRGLGLFPFTVSST
DEMAFRALSPVTLFSVVMVVGIIGWAVYVHYNYLLFHSKGSNFNNVLIASGTVLFLMPTYL
SPLIHWPEVNKKVKFFNSWMEFQLAFLITTGKPLKVGIRYAVRFTLIVSIVVSVSCYAFVAL
TTPEVTAWMLWPLVPSTFLFSIMPGIWIILCSLLTKTSKQMIQLLEQNGRRCCAPAVLQDY
RQLWLKLCALAQDLGNSMGLTFGSLTLIYFILEVLNMFIVMFNVRQGYASEFAFSLFLTVFI
ECLIFLQCNVAHNVTQEVGSRFMKRVQSLEETYPPLTKGSQEELQNFLETMKRTEPVISYS
GFVAVDRSLCTSLASAMVTYLIVLLQFQLSL
>BgerGr24F
MSPAVNEYIRTDESTFYNNMKSVLFTLRLMGLLPYHITQQGRMEFSYFSPAAIYSLLVMVT
VTVGVIYRGMLAMEDIYSVSFVSRMFAYISLVVYCSPVILIPLVHWTETRKKIEYINNWSCF
QYEFRRVTGKLYHLRLHYKVLLGLAAVMAYVGLQCIPFILNIATAHLWWALPTMLFCTFF
HMLIAIPWVSFCSSLRIAGKSMRMEMEAHVGTCGFGNGSKISEYRVLWLQLSRLTQDLGN
SMGMTFGAHIVVYFTGQVLFMYYILSMLSLRLDEFIVKFLIPVAISIYIYIVCNAAQSVTDEIG
ESFLECLETLEAKHPPKNAESDNEIQKFLNTMERNPPVISFCGFANVNRSLQTSLMSALVT
YMIVLLQFNMGSPNPLCASNVS
>BgerGr25
MPIFSTSNSHLQARNDLFIREVAPVSIFSTDKFHKPPAKNEETTQNKFNASKDMLPETLNS
YNRDLSAIIKVLQASGIFPFKKTLRGTYVYSPFSPLMIYAVCLYVSMLIILWFTTGVKLQDL
MKIHHHFNVMFFVFMAVAYFLIILAIPFINWPEANNIARYLSNWTKFQEKYCKLTGKPLN
LGMRKKVFILMCILPVLIFSFEVIYQLTNGDDVWVDWWVAVKEWYLDVVRFYIITYWHLT
CGALSSAARNLWRNFIKEYEESTKYIPDIINLHRCLWVDLSRLVRETGTSMTYTYGFYILY
MFLSLTFFVYHTMSNLARQLRADQVLMAIECCVMEYTLFLLCDSASKATREVGDEIRDGIL
KVRWATKDPVIQKELDKFLKSLDTLPPIVSLGEFTIINRGLFISLGTMMTTYLIVLLQFNMS
SSDSHGYNITLQI
>BgerGr26

MIDKNLQSKIIKVKPIQTAMKPDNLGSINCISNSETRDIYYIDLKAIFIHLTLIGIFPISIDPNG
NAIFKVLSITSIYCILLYTGNVFLLLMSYNNLATSRKFDDSLFSVAYYYYFSILVLLPLIAWRN
TSNLTSYMTRWKHFQELVYKRTGKELPSALRFRSIVLVLTISVALAMIEIARQLIQSKSLKW
NLFFVNYFINLLRYSLTSFWWLQCNFIRRTSVDLEGRFKKKIKENLKVEEVVVFRNLWWE
LNQLLHQKGKILYFTYGFLILYLFGKIIFNMYFVLINVMFEESDKFTVCYTFLMTILDYLML
YTVCDQAHRTSVQVKDSWREEILALSMNLSTSETLNEVNGFLHIIQRYGADIDLNGYVIVN
RKLFVSLTTMMVTYMVVLMQFKLTSFSNEQNAATNCTT
>BgerGr27
MASKICVDIRKIYSSIKSPNIQISSEHIKATDVTSTIYELEHSMKPILLVLRTIGIFPIARGSSGE
WQLRLMSLTSLYSLCVYVLSLVLLWFTTGVRLREFTSKYSQFDVMFFVLTCISYFIIVIILPLI
VWTESHGIASYLNQWDRYQEYYFELTGQRVGLNFKRRAMFLCCSEFVLIISFELVYQVTNE
DGILVDWLVALNEWYLDVVRFFVLTAWYLVCVALSTAADNLWKNFNKELSFRASCQVVS
AYRNLWLELSRLVQKTGRTLCYSFSFYILYMFVTLTLFVYHTLSNLTHQLRGDLVLMAIEC
LVMLLALYVICDSANRTTQQVGDLFRDGLLTERSYITSTSVKEELETFLRIIDTSPPRISLGD
FVIVNRALFVSLGSMMTTYLVVLLQFNLSDDGNHLYNISLQI
>BgerGr28
MYPNYFHEEISSARLSSRPFCILESRKSYSTNFGVLYLNMKPVIFALRVLGVLPISTTNSKME
FKLRSKSMAYSVLVYMCMMALWISQAMKIFAQEKRFDHVVNKLSTMIYFTILIGVPVIMW
TGVHTLIVYFNSWKQFEERFSQLLQQHLELHLQRRSLVSALVAMVTAFALETTYMGMSRC
FVWSRWITNMYQTILRYLLGIFFYLLCCSIVRTADSILNLFRLDVKHGSSKTGKLVAKYKIV
WLELSRLVNLTGTACNYTFGFYILYLFLNLILCLYLTATYTGSKKKIVVNVETTISTSLLLYF
MCNAGQSVKRQVVEVFQDELLKMRSGTCHVMSRREVDSFLNMIKIMKVSVNVGGFIIIRR
SLLVSLGSSLVTYLIVLVQFQFQVAGNV
>BgerGr29
MKMSYPDTLERTVASLFKEDKLFTKTANIYLKEDLFYNDVRPVANLLKLMGVLPLQRIGT
ENRFRILSLQMLYSMGVYVAMFVLLWIVHGHELQSQHYDDSILALYHMVLMLLLVAIPFT
NWKDSPSVAKLLSSWLDFQVDFCNVTGQVLMFNLKKRSLILSVTMPCGSVLLLELCYQLLD
VSINAVNLITDVYLLTLRLLLAVMWHLYCLALAKAGDEVLDKMKQKLKTVVVPTNEIIKY
RTLWFNLGKLTKQTGTSLCYTYGFFILYMFMKMTLNIYWTLNSLLFVPNKLFIVVYILISIA
IDITIMFLFCNDAQLVERKVYDAFVNTLMDYKLSTSNSILNDEIDVFLYTINTTQPVLNLGD
YVTVNRSLVISLGSIMVTYLVVLLQFKLSNMSPEGHNSTTVSQNLTPSEALLTEAN
>BgerGr30
MDKHEHCFQKANEIIFPQCKMTRLENAVLFTHNPLLIHKRTTPSAQLQKDHIYEELKPLW
LELRVLGVLPVEKTTNEYGLPTFRCSSFALLYSITFFVVIGIFISYAVYMKILELNVIGSEHFD
QAVHIYMHIIYLIPHYTIPLGHWKHGKEIARLLNNWTTFQVQFNQTKFDGKCRKSRFNSV
FITSVAVPLLESSLMIIYWIVLPEFTFQDIFYYTYTNALVHLHIVLWFLMCCKVAKFANHLA
SILNKDILSRKIIEDCHSLWQELSCICRQIGDSFCYVYGMIIVLWFIVATLSLYGSLTSIIDKGG
SWREIGLLINGAGCMVLLFIICDAGYRLSNEMSSTFYSNLQALHLKGNKKIEQEVNCFKYD
VVLKSPIVSLGGYVTVNRSLLISLITTMVSQMVILLQFRIGIEVPNNSTFTCNETCLCSHVT
>BgerGr31F
MAAMRYPSFYREVQMTNADMGKMRKIPREYQRVSPMTDNFFKFGLTGSTRYDDEEFFH
WHLKWILWLLRVMGAFPVKFDSSGFPEFKLLSLATAYSMCFFLLINSLTWILITDFCSDIQ
KLGADLSDSIYSVLFIASFTFCIWIPLLTWLDARSTCRYFKAWAEFQLKFRAVTKDRVQLNI
RKYSIVAFLPVVGSGLFVVLLSRMTPHSEPWKMPVPITGCVHFSTLVILKIFMFIALHKAGT
ALDSAFKRDLALKPTSPNLVEEYRLLWLRWSRLVSQEEDMFGTVYAFTLVKMLFIVVITL
YCSLMDLKTGQVNYERQLNTCSPCAFGMATLYFICRNAHRLSDLGKEFQKNLEIISPSTVSS

YVWLEVKFFLDTIRMNPPVVTAIGFTNVNKPLFVALMSSMVTYMIVLLQFDRGDSSSLRLS
SNSTNESPTSTTSFS
>BgerGr32
MKPINMTGIRFPKDKNDPSLDIGKARESSPVFTMTDNFFSGGLSSYNTKESLFYHLKPSLC
LLRVLGAYPAQFEASGLLPRFKMLSAITAYTVSFYVLLKLPTLAALKDYYFELVTSGADLSE
SIFLVILMSMFTYNIWLPLLTWSDVKRTCNYFKDWNEFQMAFISVTRSHLQLNIRKYLLPL
VLAICTMSFLPIFVMGLDLWRSAVLMVSSLYFADLLLLKIFLFKALHKAGTTLINSFKGDLQ
RKSFAANMLEGYRLLWFRWSRLVSEEDCMFGTLYAFFMIAMLFITVITSYCSLMDLKTGR
ADLFRLGNSAPTAVGGVLILYFMCRNGHSLSDLGKEVQKDLDRIGFSSLPKQDRRVVKAFL
ATIKMNPPVVSAVGFTNVDKTLFIALMSAVVTYLIVLLQFGGG
>BgerGr33F
MYNSIRYPHEIKSGHELDKTTDLSHDFNHAHPMRGDFNFGNVRTSTFNFKDSFIYQMKPS
LYVLRIIGAFPVTFGSSGLPKFKFLSAIAVYSVCFYVLLNIPTWALLRDYFLAIKSSGGSLSDSI
YVVMFMTIFMFNVWVPSLAWVDVKRACQYLADWTKFQMEFVAVTKEHTGLTLRKHSIL
WVANSFLGSLIPVVLFNQIPVMTLWRLSTPMVATIYSTITMSLKAFISLALYKAGVALRNQ
LKRSLQKKPISPSVVEDYRLLWLRWSKLVSQEDHMFGTVYAFTFIKMLFIVVITLYCSLMD
VKNGSVDLFRQTNTLGACCVGALILFFFCRNAHVLTDLGNKFQRDLQFISPSTVSSQAWSE
VKAFLNVIETNPPVVTAIGFTKVDKTLFVALMSTMVTYMIVLLQFDKGELNFIRSDANTT
MGTNT
>BgerGr34C
MFQKFDSLMKMRKSLHQLQTVCPKANNYFFKFGRRETYDVKESFHYHVKPTLCLLRIMG
AFPVAFKPSVAGLPEFKLLSPITVYSACFYIVLNTLTWSLLSDFYVEMKKFGGDLSDSMYII
MFMTFLVSNLWYPLLCWLDVTNTCQYFVNWRQFQIEFDAVTKDRIQLNIRKYSFWIFVA
TPISGGLVLLFLRILTSAKIWKAPALMVAGIYFSNAVFLKMFMLAALHSAGTALFNAFKRA
LEKKPLSANLVEDYRLLWLRWSRLVSEEEFMFGSVYAFSLVSISCVVIIHLYCFLVDMRSRK
LDAKRLFNTVAPWLNGTAILFLVCRNAHALSNL
>BgerGr35
MPPGRQAHVYRGAHIIKPKESFMSSMNFRNPEFSDSSAMYKEEDSFYFNIRAYFFVLKILG
AFPANHDTSGLPRFRLASVVMAYSACMYLAVAANGCLTMNEYYLDIARSSQPWSHSIFSV
MTITLLTTVVYVQIFAWNDTNKICHYVQQWREFHKLFVAVTRKRMLLDLKTYAVVMTVG
TATFSVASGLWFMIEPSVKLWTLPAFELSFLFLGIFMSVKKSTFDALQRSGACLRTSFQCE
LQKKPLSAEMVEGYRVLWIRWSRLVQHEDHMFGSMFAFAMIVMIFTVVITVYSLLGMMR
EDSLDVYRLLNTFIPCVVSTSNLYRMCNASQRLTDQGMEFLNDLQMIAPKEISRKAVDEV
TYFLDTITLTPPVISANGFARVDRALFRAFTSTMVTYMIVLLQFDKAGTNLNS
>BgerGr36
MILNEDEIFSTTSQSKYGGKPRAELSALSNSDEFNEYESFYYNVKPNLFMLKILGAFPVIDD
GSGIPRCQLFSAVTIYSVCLHLVMSVITCLNIKDYYLDMLKSNGVLYDSIYPLVMITIALIGLY
VPTFSWFDIRASCHYIKIWREYQTMAETITKEKMNLNLRRTSIVMVSICLLLSMFGAFMH
VLDPSLTAWKVPAFILSSLFASILTTLNNLVFQALHKSGKQLRKSFQRELQNKPLTLEILEG
YRVLWLRWSQLVQQVDHLLGAVYSFAIVEMLFTVVITLYYLLTGLRSNSPELHRLINAFTP
FITCTANLFLVCRDAHNILQLGKSFRDDLHRIAPSEVSRNVMEEVLYFLDTIRMNPPIISSL
GFAIVDKSLFRACMSVMVTYMIVLLQFDRGSLKTSASTTCQCSN
>BgerGr37I
MRPTSKIVFVKRSDWFRDSVPNYPLTPKISYTATKQVQVRQEDYFHEQISVLLLLLRLLGA
FPVRMSPTDVPVFQLFSPVMAYSILVVLFIGGPTFVAARDYLLQMEGENFSDNVFTILFVAI
LVTIVYMPVLSWFDVKTTINYLRQWTRFQRMFLGVTNKRINLNLRRKSQVFTAICSIMNC

LSILLHFYYPYIELWKVPLNIISGVAVCAAIILKWANFRALINAATNFNSCFKRDLEDKGPTS
EMIAKYRNLWLQWSQLVTEEGGVLGTMYVAFLLTMLFILILSLYSTLDKLISGNMGIFFM
MNAMVPCAMCAFTLFALCNFGYQLHVLLLSATVTYMIVLLQFENGEKNSKETTSTETSSP
VR
>BgerGr38
MDSKNLKMRYKSRKLFVKRSAWLQDSVPSISIVANCLTETLELRQEDYFHEQIKILLCTLR
ALAAFPVRMSSTDVPVFRLFSPVTVYSLLVYLTMCSKLICLLRERFQEMGNVDFSENVFGIF
FMAVLMTLIYMPLICWLDIKNSVNYLKDWVIFQKNFLDVTRKCLVLNLRGICRIQIAVSIVI
NSSFVWMHFYYPYLELVEVFLNILSGVVVSCTILLKRTIFQALKISSANINSCFKEDLHARGP
SPELIAKYRQLWLQWSHLVSQEGNFLGTLYAVFFVAMLFILTLGLYSALVGVRIGYNSISW
TMSTVMPSLICCGTLYIFCDDGYQVSMSGMEIKNDLQLIAPIHTTHQVMAEVEAFLNSLVM
NPPVLSIMGFSNVNKGLFNSFMSATVTYLIVLLQFKNDQPSTSTASSHHQ
>BgerGr39
MDLRKVKMRPKSNSLFLRKRHWIHGTAASRLSSMPKISSVGRYYGEISTESVEVHREDYF
HEENKSLLLTLRVFGAFPVRMTSTGVPTFRLCSPVTAYSIVVYLFFGALTCVALREDYLQM
AKEDFGHRINKIMLMTLLFTTLYLPLLSWFDINNTLLHLQQWAQFQRMFLKVTNERANL
NLRRKNNIVLAVSAIMSVSMVTMHYFYPYSDLWRIPIYTFSTMLSSVAIFFKSAVLRALSIA
GTKFNSCFKKDLQERGPSPDMVAKYRLLWLQWSKLVSEQGRAMGKMYLVNLVIALFVFII
SSYSCITSLLADKIELVLLLSTFFPAFGTSSMLYGFCNAGYQLSLLGEEVKHDLQLIIPAHTTY
QVMMEVKAFLSAIAMNPPKASINGFSNLDRGLFNSFMSTTVTYMIVLVQFGTSQTKENCT
NASFTSSPEE
>BgerGr40I
MEPRQLKVRPECEEALFVRRAGYIDDHFSGTKSALYGVKRREPLQMLQGSYFHEEVKFPL
QVLRILGVFPATMNYPDVPTFRLFSATMAYAFSFYLCVSVPTAIAMRDHYLKMAEGNFGE
RVYNTLLLALLMTMVYMPPLSWLDMKNTISYLHEWTRFQRDLQERDLTPEMIANYRLL
WLQWSQLVSLEGRRMMGIMNGLGLLVMFFVVILSLYSTLVGLMTKDLAVSWVMNTIVPC
LSTAAILYTICNDGYRLSALGSDVKNDLQLIIPANTTPQVMTEVKLFLNVILMNPPVPSIMG
FSNLDRALFSSFASATITYMIVLLQFDKEETEWKTSTESSL
>BgerGr41C
MTLYSEHRTQRYEMGLRQVKMRPKSKFLFVKKRVPSISLIRGKRMKALEMRPKEYFHEQ
FKFLLLALRLIGAFPAKMSDSDVPTFRLFSPVTAISVSTHLFLAVLSFSAIRDHHLRMANED
FLQKIHSLLLVTLLVVMLYLPLLCSLSVKNAIHYLNEWAQFQRMFLEVTNKHINYKFRRIYI
RNFIVMLIGTNLMLFLHFYYPFLELWKVPLCMLSCIVFSAAMLFKSAILAALFQTGANINSY
FKMDLQEREPTPEMVAKYRRLWLKWSKLVRLEGRVMGAVQGLNFLLMLILSLYSFLTAV
IVEHNDPVLVSRTITPGLGSALFLYGFCNQGYQLSLV
>BgerGr42NI
MFLNVTRRRLNLKLRRKFKIFFILFAIGNSSSVLLHYHYPFIEVWKIPLYTLSGLAFCFSVFL
KRALFRGLIAYGTNLNRSFKVLSLYSTLSVLLTEEINWIWLVRTFVPGLANTVILYCYCNDG
YRVAAMAVEIKNDLLLIAPVNTSPDVMIEVRAFLNVITTNPPKASLMGFSNLFMSATVTYII
VLLQFDKGEKGRNETSTALALLQG
>BgerGr43C
MDPRESNMRPRRTVAWIRNNDFGVSNKLSISRFEEIPVESYEMHPKNYFHQQVKVFLVLF
RLLGAFPARFSPTEVSLFGLFSPVSVYSILIYLFIGAPTFLLICEHCTELRRTNNFTDDINIVIT
IALLTTMLYFPLLSWFDLRSSVNHLNEWAQFQMLFLKVTNEDLDLKLRRNSITSLTLSFM
LSLTCVFLHYYPPYLEWWKIAVYMFSGVAFGTVLIYKRFMIYSLQSTGIKLNKCFKKDLKE

RGPTPELIAKYRCLWLQWSQLVSQEGHVMGTLYGFILLLLLFLHVSSLYTFLVGFRSDEISI
LEQINRFAPSVGSICTLYVVCNGGHRISVM
>BgerGr44I
MDPMKLKMGTNSNTLFVRRSAYTRRSMTTKMLFMSRYNQANEPGLQREGYFHQEIKPL
LICFQVLGAFPVTIGTSDVPTFKLFSPVTAYSMVVYMLTSLLILLAVRDHYYDLLKLSNLND
KIYLVTIIIFIIVSLFLPPLSWFDLKHTIKYLHEWVKFQRLFSKVTDGGTGFKLRRCPLIALVV
VPFAAFQMTLLHVYYPYLELWKIPLYSISSFGYCILIFFKMSLFDALWTTGKKVTAYFKQDL
KKRSLTPEMIAQYRCLWLHWSRLVSQEGTVMGRLYGCFILTLLFNHILALYSCLMGFRSEE
IDIFWQVNTLTPCLGCAFTLYLICNEGYQLSVMFMSTMVTYMIVLLQFDNTGAKLKH
>BgerGr45F
MKSNSKALFVRRSAWTVDSGLPPFSFTQSASLEMRQKDYFHEELGLVLLTFRVLGAFPVR
MKSPDVPSFRLISLITAYSISIYLIGAVLISIAMRDQYLLMSTENFTDKIYSTLYMVLLSTMLY
IPLLSWFDLKNTINYFRGWSEFQRMFLNVTRRRLNLNFRRKFKIYFILFAIGNSSSVLLHYH
YPFIEVWKIPLYSLSGLAFCFSVFLKRALFRGLIAYGTNLNRSFKRELQERGPTPELIEKYRF
VWLQWSQLVSQEGRVLGTMYGFGILSMLFVLVLSLYSSLSVLLTEEINWIWLVRTFVPGLA
NTVILYCYCNDGYRVAALSVEIKNDLLLIAPVNTSPDVMIEVRAFLNVITTNPPKASLMGFS
NMDRTLFTSFMSATVTYIIVLLQFDKGEKGRNETSTALALLQG
>BgerGr46
MDPRKSKIRPRSKSYRRSSAWIRSRDFGIPNKLSISGLEEIPVRSIEMRPENYFHQQVKLFL
VVFRLLGAFPARLRSSDVSLFQLVSPVSAYSILVYLLVGAPTFRIILEHCTELRQTDNFSDDI
YIIIRISLLMTMLYFPLFSWFDLRSSVNYLNQWAQFQRLFLKVTNEDTDLKLKRRCLAALS
LTLALSVTSVLMHFYPPYLETWKIPFYFFSCVAFSIVLFYKRFMIHSLQLTGIKLNNCFKKD
LKERGPTPELIATYRSLWLHWSQLVSQEGRVMGTLYGLLYLLLLFLHVSSLYTFLVGFRAD
EISIWVQFNRLAPSASSIGTLYIVCDGGHRISVKGVEIKMELLLIAPMYSTHEVMALVKAFL
NTIAMSPPSASVMGFATMDRTLFTSFMSATVTYMIVLLQFDKGNTPDPTASPVH
>BgerGr47C
MNSKQNFAVFNMKQHMYPEPPSRGCFLKERIDVTASTILGVDSKTEDIMKAYFGHIYTKQ
SIHNQFKPLLNLFRILGLCPVAVSQYGTLKFSFCSVIMLYTIAMQCIDVVLVYLNLPDRTSQL
MARNVSFDETVFNLLLLLGLIGAASSPVLMWMDLPDIVKSADRWMAFQFQFKSVTKRCL
RIQIRTRCLLSTVYCVTVGLVAFGLTLVPPYIPIWRIPPYYCSVTMLNCEAINVLLMITTMTQ
AGTCLTKQFRKDMQDNKDTLTVISDYRILWLHLSKLIQQVANSMGRSFICYLVMLISCLTI
TLYGSLGGITREVESRLRVFGMYVISIGVIVPFCLVTNNGHKLTENVTSDMEEILYNIPTKGG
NKAYLEEVNFFIQTIKNNPPVLSMLGYLTINRKLFKG
>BgerGr48
MYRGENYFPVVTKKSRSPINPKNPGFILNSVKFVESRPESEITEYGKRNSGPKYQLFLNHM
RYVFLIMQTLGWLPIEYRPTKTGDLRFRLCSPIMTYSVCLYVTITVTCLKCFSDVVPLLLTD
KYFVLSVTHMTMIVCGVTCVIVPVMCWASANKFIDYINQWNRFQEQYFRVTRQPLNFYL
KLYENPQCSIAVATMTGLPLLFFFFYLPNATLWALPSYYIFITTVTTTGAMWMSTFRVLIL
SGKAREAQENLSPPLIANYRILWLHLSYLVSQTGLLLGKTMLFLIVVFLYLTTTMIYGSLVTI
TMDMHIESIGMGVFGTLMALSTYRFCDLASAVTSLVKNRMQQELHMIRHHTVNEAVATE
VNAFLQTIYMNDPVISISGFTKINNGLFKTYVSSAVTYMIVLLQFDGNGGFKSKKHPNCSST
D
>BgerGr49NI
EPSLSKILLLYSVCIHGPLTVVGCFIIQDCINGVILLNMDYITTLLYVLIVTATAASMTTFLLV
WLELRKFIKYLNSWNHFQLEFQRVTGSRFQMALRVPSTANLVRCIVAASASIIFHFLSPFIS
LGRVPCYFILLGQTNAHIAMWIMMCKSLTKAGKLLSREFKNSLTKEERSAKRISEYRLLWL

QLSRLVSDT
VKAFIHTIETNPPVVTVGDVIRVERGQFTSFISRIVTYLIVLVQFNTEQPSRTSTGDCSSNET
QPLNSNSDIG
>BgerGr50
MLTMQKGLFYKRHKIPVSTKGIAWPQITISAAEAETNAKLQKIQEDITNESYHNQMKPLM
WLLRLFSCLPLDMKGTGIPTFRLFSMVTAYSLSFWITLVLSCYLVLLEHWDNYINDMGQL
RPSIFDSIQMIGMSSIPLVSTTSAIDIKPFITHMEKWLEFQMLFTEVTGNVLRLSIKRGCFLS
LAFCGLAAIGASWFHFYPPYIEVRHMFPYICLVVMTCAVKFFWYFTYRCLYVSNEQLISCF
KHDLRTKENTYNVLYKYRLLWWKLSQLVTDIGYARGTLLVMYHVFVLISLALALYGCMT
GITMKEDDRCFRIIFMTVSSFIIGVPFYFLCNYADKVSNKVKNEVMEILQQDKMMRRNIQV
IQEVKLFLLTILTNPPVITVHGFTPLNKSLFKAYGSALITYLIVLLQFDIGQDEDKQ
>BgerGr51a
MNGQKSNLITILKPVWILSRILGLNPFSFIQRQNCIKYSTQKSLLLYCFIILVVTNFGMIYSLL
EWFLNVPDNLSEIMSILHCIYLVLGSFLSFATCVFHNRNFPELIKQLNSIKLLNFPCVYSKAL
HISSTKYLALLILLIFCNATIYVGVRIVMIQRALIWMLSQDMIEFQMLFTELHFYITLKVLKD
YFLQVNYELSLLKIKWKYNAASHNQSFPKSVANLVKDMSVVHEKLYNISVKISEIYSVFMIP
MLMTDISFTTYSTHVILSNFMNPSGVKDNLYNGSHLRICLYTLKLVTLLAGSNFTKIEADR
TSKLVHKILVRTEDPRMREELHRFSLQLFHNKFRLTAWNFFALDYTLLYMIVGAVATYLII
LIQFHLMYKTEIQENN
>BgerGr51b
MRNNFRETGVKELMPLTYLLPRAIGLMPFSVTSENKSLKFSSKWTVFCLFLSLVPLLVFICS
LNEWLYESPREFSMYLTLMHPTVALITIFVGYVTSFTQCRKLHRLLSQFNFLEVTNCYSMY
SSLRRRYFTRITLGITGIGSSITVTYSFIKMIMKYSAILWLISACLVEFQLLVIEMQFTLVLDL
VFRHMNILNSELINYLNQDASSGSTKIKIVKINWSQFNSAPVSKYDWGSIHEHLCYIAEEVN
KIYSPFMIFIITTDFVMLTFAIQVIIADHVIVQSTVVARLLYFAGIPWTMFYIIKLVGCLHCCT
TARDEADRTSKLVHKILVRTEDPRMREELHRFSLQLFHNKFRLTAWNFFALDYTLLYMIV
GAVATYLIILIQFHLMYKTEIQENN
>BgerGr51c
MDPILLRLCVFQKVVGLLPYSLTYSNGMLYCKRSLKSMVFSLTLTSVSTISFCYSKWEWYQ
NTAFSIPKVASTFRPVFILMSIFVGYITLSIHYQKYLDLIEKFQCVVLHDCSPYYFSTKFRCLS
LFILDLVILISGNFIMYLCIVDQTLTASVLWLISSTLIGMQELMFDIAFITIVNILYEHFSVLNA
WLNCSENISLRKEETVVGKKNVVRYSFVPQVLTSNAYLIHSNFQSNLCDIAEEINKMYSSYL
ITRVASDVLLMIYSFYMVLGNFIAQNTILNRQYVSAFPWMIFSTLNVMSLLHVCNLVKSEA
DRTSKLVHKILVRTEDPRMREELHRFSLQLFHNKFRLTAWNFFALDYTLLYMIVGAVATY
LIILIQFHLMYKTEIQENN
>BgerGr51d
MEQGSHSSTNLIYYISRFLCLPLFLRKTTSTIYTFCSICIFIVTNTIIVSLPYNNVTHNGYAVIA
SFSIFCTTSVVTNIVGLTFDIIFRKRLSITFSNIYELETLMHAEKYFDAVVCCKSVVLIMLIIES
LTLIGSVVISWLSVGAKVQFIHSYICIFLTEEYSFLLELQFISSVIRISELFKTVNEMLIQNKEA
RFIFIGPHQRKIFHNEINKSPTFISITTLARCHEYLCLVIKTINAIYSPVILLTIGKLLFGISTSLF
VIVIQIIDIFLVGKSNVNNQLYLWLVRDSINLTFLIWCSSSCVFQADRTSKLVHKILVRTEDP
RMREELHRFSLQLFHNKFRLTAWNFFALDYTLLYMIVGAVATYLIILIQFHLMYKTEIQEN
N
>BgerGr51e
MAEGIHPSLNIIYYTSRIFCTPFFLEKGTNLFYTLCSICLFIATNSMYIMLPFASRGKENYVVI
VNMSIFCFISVLTNITGFICDVILHKRMSTAFRIVSEIYAEMLLHAKQDFHGAVHWKIVVVV

VVIGTLSHLASLILSWVTLGAHIQYALMFLCMFLIEQYAWILEIKFLTFVDLIRELFKIVNNS
LLLIKESKFKIRESYQFKIYHNKNIEINKLSSITSLGNIHENICLIAKQFNTMYSPFMLITIIKM
FCGISSSLFYTLIKIIQIFANEKNHANNELFLWLIRDSLNLFYVIWCCSSCVFEADRTSKLVH
KILVRTEDPRMREELHRFSLQLFHNKFRLTAWNFFALDYTLLYMIVGAVATYLIILIQFHL
MYKTEIQENN
>BgerGr52aP
MKFFETVDIYTAFSPIFILSKFLGLWPLKLVSHNGIKEYHVTFSALMYSFLVMFLVLIKIALF
VGHLFINENIITLFQTTSTFETVMCNFTSSLFFFLCVLNRHKMVKIFRKLAEFDQKLVSPKS
CKNTLIFIMVQIFFQIVYLCSAGIATLVSGEFHLDCELIAYVCISYNMSVNTSVDLAITNLMF
ALKQRFHTINNKIAELLQSDDLEFTISKQNFKKVTFPVTTVSHILRAGGXVNNFSKLHDSLS
DIYEFVNSTFSIHVLLAIALKFVGIIFNVYFRLLRMLNYNTGRFDTGSYEGIMVAMLFWNVF
QLIVLVWSCDSATEEANRTAVLVHKLLSKTRDPDIKEELQLFSLQLLHRKVQFTACGFFPL
DFTLLYSIVGAVTTYLVILIQFQLSFTNQEHKNETTTIAPNAGNYSFSITTLNP
>BgerGr52b
MKWFQTVDIFSSFKPIHVLSKFLGVWPLRVVKQNGIREYEASSAALSYSSLVMFFVFIKIAL
FLVNLFRNEHLITLLQTASTFEAVTCNLTATSLFFLCLLNRHRNVKIFRMLAEFDRKLISIH
SYKNLLIFISAQIFFQIAYLGSVGITVVLDGEIEAWDIVIYICIVFNVSIIASVDVIITSLMFALK
QRFQAINNNMEYLLRSKDFEFTTTKQRSKKMFFPVSTVSQVLIDGAFWEFPFQKSKNFSE
LYDYLCDIYESINSTYSVHALFAVALKFIGIIFNVYFRLLRVLQYNTGTFEGKSYEGVMVAM
LCWNVFQLTLLVWSCDSATEEANRTAVLVHKLLSKTRDPDIKEELQLFSLQLLHRKVQFT
ACGFFPLDFTLLYSIVGAVTTYLVILIQFQLSFTNQEHKNETTTIAPNAGNYSFSITTLNP
>BgerGr52c
MRSEASDLHSVIKPLYCVSKLVGLLPLTVITRYGTLVYESSTTDIMYSISVLIVVAGCQIFTL
WKNACVENLEAVFGTANIIESVSYNITTFLLHVLCILARSNVSKIIQSFDTFDQLSQDISSWD
YNKIRCFLIKEITICLLVFVVLAFNVFLSIEMPSVLHTISHVMFYTCIISVFIVDLQHINLILLLR
QRFQAINAQMVSLIDEACNSIFLMKLEKYPNINIDPIALISSSLYPIQARIDLSPTSLQYLKNL
HDFLCDLSEKINSTYSVRVLLTIALKFVAIVFYIYYCMLRILKHDVGEYGHAAWVGFTFSW
TCWYIVIITATSWACSSTASTANRTAVLVHKLLSKTRDPDIKEELQLFSLQLLHRKVQFTA
CGFFPLDFTLLYSIVGAVTTYLVILIQFQLSFTNQEHKNETTTIAPNAGNYSFSITTLNP
>BgerGr52d
MESFKPLKTLFYFSRFLGLTPFNVNHKNQIINFYLSKVWLTYSILLIISVSLVLVYHISWVTE
THSTEFPITKLVFIIRSSSNLSMYSMSFILSYLYLNRFGHVVRKLLSLEELHFYSYKKLRFVSR
AQLCIFMSFIVIIICIEIHKLELEFGKCLFLMANVITDFTCIVRELIFVSCVLYIQQQFSCINEQL
KECKHILLKRSMSKSLSNRSLDRLRKSRSAFTRFKPQSVWCTRNSKMVSIQNYFRNISDNI
VINTIQRVRNLHMSFCGLVNKANSIYHFQILLNITNAFVNIVYVMHISLNFLMGSYHDIDDL
KFTSLIIWGMFYALKIIWLVISCCIATQQVSHTANRTAVLVHKLLSKTRDPDIKEELQLFSL
QLLHRKVQFTACGFFPLDFTLLYSIVGAVTTYLVILIQFQLSFTNQEHKNETTTIAPNAGNY
SFSITTLNP
>BgerGr52e
MNRIPMCLKPVFYISKFIGIAPCTIQENGILVVSKVELCYSIVFLLLSALSMLRYVLNPVFHLF
SSYNLIITYLCHLGMTVIFSIAVIVSCIMMFAKFKLMRNVVFRIVFLETNLQIKFSYKTNISIII
TMLFALLPDISDLMEDVKAFNNANDILDLYLHNAIFIMQMIYFQFITLVYIMKQYFLYLNE
LITATGFVDKHVKNHFKNKHSQMKQIKKARFLFNKKFVMIFRRIHLFLSETVDEINRAYSV
QIFFNLTTVFFGWLYRLYYFAVLLLSLQILVKYHFLKRLKFVMQIIYNLMILLYVCADTTKE
ANRTAVLVHKLLSKTRDPDIKEELQLFSLQLLHRKVQFTACGFFPLDFTLLYSIVGAVTTYL
VILIQFQLSFTNQEHKNETTTIAPNAGNYSFSITTLNP

>BgerGr53aF
MYLTGEFDIYSEISPLIWVMKVLGVCPVRLVGNMNSREIICSKMTLAYSFLVFILMSVLKPL
SVLKLVDKFELKIDELNLTFIYSIILDILGVITVISSNFENLKIAGIYKYLSIFDCDFLFIPSNYK
KSFYSVLTRVLIAIGILNLAMFSTFLVPDVSLLEIVLSGYSFAVGLLQDLLFCNIVLSINNRFT
MINLQLTSYIKTPDNAFLYLQFQDESRLRPVKQLSHLYEILKEASEFVNSVFAFRNLTFNAS
KLMYLTYSLYIELSRSLRPKSSVDHLEFSLAIYVLLVYVVASLIYVVWCCDSTSSQANRTAVL
VHKLLNKTKDPTTREELELFSLQLLHRKVQFTACGFFPLDFTLLYSIAGAVTTYLVILIQFQ
LTFTEKENNATISSFPTTTTTTTFNYI
>BgerGr53bFP
MAFIKFVDTLSEMIPLFWTLRIHGLFPYIVFGDSFRRLKLSKFWLTYSVIIFILKLLDVYKRQ
LMWYSMMNLQLEQVNXIKVICAVTSGLASCCMLLDVIPSERNWSTLFSKLTEFDRMSHYR
IEIWRRVIKSVGLIGRKFFVIFCCIYLLYVHSHFSEFLFVLSCFQDYMNKISFLSYDSFVLTIAS
MIKERFQHLNAKLVKIRQCQETNEKSYNGSRDNNFLPESIVYRVKNLTRSHDLLCDVCDM
LNTWYSLRIVCSVAAKLLTSLIFSFRIVTIFCEGLDIEATPSLIFVSSMFVVGPLVLLFAILQAC
YLAGYEANRTAVLVHKLLNKTKDPTTREELELFSLQLLHRKVQFTACGFFPLDFTLLYSIA
GAVTTYLVILIQFQLTFTEKENNATISSFPTTTTTTTFNYI
>BgerGr53cF
MYLTGEFDIYSEISPLIWVMKVLGVCPVRLVGNMNSREIICSKMTLAYSFLVFILMSVLKPL
SVLKLVDKFELKIDELNLTFIYSIILDILGVITVISSNFENLKIAGIYKYLSIFDCDFLFIPSNYK
KSFYSVLTRVLIAIGILNLAMFSTFLVPDVSLLEIVLSGYSFAVGLLQDLLFCNIVLSINNRFT
MINLQLTSYIKTPDNAFLYLQFQDESRLRPVKQLSHLYEILKEASEFVNSVFAFRNLTFNAS
KLMYLTYSLYIELSRSLRPKSSVDHLEFSLAIYVLLVYVVASLIYVVWCCDSTSSQANRTAVL
VHKLLNKTKDPTTREELELFSLQLLHRKVQFTACGFFPLDFTLLYSIAGAVTTYLVILIQFQ
LTFTEKENNATISSFPTTTTTTTFNYI
>BgerGr53dF
MYFFGEYDNFSEIIPWIWTMKIFGLCPFHIEGNIHHRKIKLSKMSLLYSVIVSILIAVVNLISI
LDFYNNFRVKLFNIKVVSTVGRSLSVFCLVIVLFNLIEANKLPIIYKKLSIFDRKISINHTYYR
KSFKNSVILLFIALIVIIITFVVTAFRILHPTVIIIYYKLFVLSSNFVFQSYIILELKRRFETVNLQ
LINLKFNKKYGNLAKSLLYKDKKQELNLIKELSYMHTYFADICDQINSIFCARNIISVYCIFI
MTTSLTFNAALPNLISMVVLRRALFIFALYAQLGLVLFAFFFNIWCSDTACSQANRTAVLV
HKLLNKTKDPTTREELELFSLQLLHRKVQFTACGFFPLDFTLLYSIAGAVTTYLVILIQFQL
TFTEKENNATISSFPTTTTTTTFNYI
>BgerGr53eFP
MNSSGDFDVYSEILPLVWALKIFGLFKFSLKGNVGSREFKISKISLFYSSVIAFSLLILQYFED
NVFIGAFNYTIHDSKVVFVSRLIKNAVSYVMIILTLFQNSQIPLIYXYKRLSIFDKKKVIAHTE
LRKSFFIGVIGCISILSTILIALFVILSIITKKYAIVIIVTYYNLIIVLAQVIFFSKVLLTIKSRFLVI
NSLIIDYNNKKVDENCSSKFNDKYRFQLKSLSELKHMHQFLSDICEMMNYGFSLKNILFVV
YSFIIFTYYMYNTVFLRIISITSFHNKVLYTTFCARFCTLLITFVYVNWCZKTTSFEANRTAV
LVHKLLNKTKDPTTREELELFSLQLLHRKVQFTACGFFPLDFTLLYSIAGAVTTYLVILIQF
QLTFTEKENNATISSFPTTTTTTTFNYI
>BgerGr53fF
MKIFGLCPFHIEGKIHFRKIKLSKIYFLYSVIISTLAAGVNLISILDFFDDFRVKFFNINVVSTV
NRSLNVLCSLIVLFNLIDSHKIPDIYRKLSIYDRVIFVSNTDYNRSFKNSAIFLFITVCVIVMSF
FVTLLNVMHPIVVVIYYKLFVVLSHFIFQYFFIKAIKKRFEAINRELTHFRFRKNTKIFRVNV
LSKRNTLTRIKEWSCMHRYFTEICDQINSLFSIRNVFSISYIFIITTSMTFNVAFPSFNSISVF
NRSPFIFALFAQVGLVVVAFLFNVWCTDTTCSQANRTAVLVHKLLNKTKDPTTREELELF

SLQLLHRKVQFTACGFFPLDFTLLYSIAGAVTTYLVILIQFQLTFTEKENNATISSFPTTTTT
TTFNYI
>BgerGr53gF
MCFFGECDNFSEIIPWIWTMKIFGLCPFHIEGNIHHRKIKLSKISILYSVIVSILAFMMNLFV
VFNSYNEFDLKIFKFRAISAIGKCLNVFCSVIVLLNIFEVNKIPIIYRKLSIFDREVAVKHTDC
NRSLKNSLILLFMAFSIIISTFVSTIHVSKVTGFVAIVHYYNTIVLLSHFKFQSCLILEMKNRF
RAINNLLVHNEIDRNYENLPKTSFDNEKKYKLKILSYIHAYYVEICDQINSIISIRNLFSVSFIL
IVLILLTFDFAIPSLSSTGMLNRAPVTFVLLALLGVILAAFFFNIWCNDTACSQANRTAVLV
HKLLNKTKDPTTREELELFSLQLLHRKVQFTACGFFPLDFTLLYSIAGAVTTYLVILIQFQL
TFTEKENNATISSFPTTTTTTTFNYI
>BgerGr53hF
MNRLGEFDVFSEIIPLIWTMRFFGVFQYSLQGKVHARQLKFSKLSLLHTLAVVFLLLVLEFF
QNQFFLAIYLLKAHESKVIFCLGLFRNILCYIIIILTMFQNSQIPLIYKLLLTFDKRMFNCHYM
QRKSFLVCLIQCITIFGTVLVAFFVSLCIVINKPVFVIILTYYNLIIVISQVIFFSKILLTIKTRFL
VINSLIVDYKNISYENSTLEFSHKCRFKLKSISELSYMHQLLSDICEVLNSSFSIRNMLFVVYS
FIIFTYYIYNVVSLSSISVTPFYNSELYLTFSARFCAALVAFVYVSWCCKTTSYQANRTAVLV
HKLLNKTKDPTTREELELFSLQLLHRKVQFTACGFFPLDFTLLYSIAGAVTTYLVILIQFQL
TFTEKENNATISSFPTTTTTTTFNYI
>BgerGr53iF
MAFRTLSDIFDEIHLFLWFLRIHGMFPYVVIRQKGVHILKLSKFWIFYGIILLSARIIIEILWL
QETLHTNSLWSNTRKITMFGYIIHCSLCVCNMFLNVIGVYKIRDFFSKLVQFSNLVGFDNK
SSDMFKKSFVSILYIVIVAAISQFALYSQNYDGVSNIMIVSFEDYFDVVPTLSSDAYLVIIVIFI
KVRFVHINSILDKLTENGDIDGTSNKNQRFRKITQINNSSLLSSRLKNLISSQNLLCNLCELT
NSLFSLRILISFAIKFVASTIFWYTCITNKDIPYGHDFTLITAAIFLSVFVLVPSCVILFSCNSTS
SEANRTAVLVHKLLNKTKDPTTREELELFSLQLLHRKVQFTACGFFPLDFTLLYSIAGAVT
TYLVILIQFQLTFTEKENNATISSFPTTTTTTTFNYI
>BgerGr53jF
MARSFQKDYYTFLLLFHLSRIMGFIPFFNETKSTSRTNATMYFTMLRSSCVTFAVVLNMCL
LIWFILSRTDLDVVVTVILIIQNIMYIIIFIGTPFYFLFKMDSLRELLSKMSSFEKSFGNTYAN
TRLFSAQLRHQNLINMFLIFVFITAHILFTVGNPGLSFLSVLFIIFSSLEHFFIDLLLLNLMLLI
KNRFYVINVLINDLKIIENSKHVEHDNDLHLEGSVCNLKRNHSFACDIFELSNSTFDFPLLL
SMLSKCVTLILYIFFQMLTVFNKTKTDIFQNYYFIVASLLSLMCSASNLVCMLWSGDYATR
EANRTAVLVHKLLNKTKDPTTREELELFSLQLLHRKVQFTACGFFPLDFTLLYSIAGAVTT
YLVILIQFQLTFTEKENNATISSFPTTTTTTTFNYI
>BgerGr54aJ
MAFLDRKDLYCLITPFFYVSKCLGLAPFYVKISFSARKFKISDIAMAYSIFVAIVPFTIHVISH
WYFLYDENMEAVFLSSKIIQSFAFLLIIFLIPITFLLESLSLCKLVNNFLMLDLLFQNFEDKYS
RYYAKARAITISEIIFLCIIVLQHVGMILVNDELEFWYFIISIFFVISDLIIYLNDMHTMDGTM
LLKYYFSIFNLILDNELENKNYSKSRNHARSINKVQRIGYKPLITVHKHLLDTNDIADTIFG
KLVLYSMLMKFLIVVLYMYFYIISFLEFDTAGYNGLVWNLNFSLPMIWSVLFLIIISWSSNG
VSFEANRTGVLVHKLLAKTKDPEMREELQLFSLQLLHRKVQFTACGFFPLDFTLLYSIVGA
VTTYLVILIQFQLSYNDDGIQNTTNSPFLATTMPTLSYVNDSLR
>BgerGr54bJ
MATKKQDVYYAFYPLFCLSKAFGITPFNYNSQVSDEKLKFSLPATVYGAILSFLFVSVQFIA
NWNMVQRMGYQNFIIKTYSIKVCTDVLSSTVVYTSFLINTKAFPKLIHSLSHFDRLLSTVED
LTADFYRISRIVALSFLFGCLCLYFSVGFVICYIITSLTLRMPVTSIFVFFSNFGSFIIDLHVVNL

NNLLKQRFNKINNIVFDLHNTTANRRSLNSRQIDPRNVGKLYSQLCDICERSNSAYGLQLL
VGILRKCAVVLTDTYFLTLIILKQDRGPLANKMWYIPNNSLLCFNVLALLFLLWSCHGMTQ
EANRTGVLVHKLLAKTKDPEMREELQLFSLQLLHRKVQFTACGFFPLDFTLLYSIVGAVTT
YLVILIQFQLSYNDDGIQNTTNSPFLATTMPTLSYVNDSLR
>BgerGr54cJ
MSSVAKDVYYVIYPLFCVSKVFGFTPFSYNLKSYNEKLKYSKPAAVYVILLLLINVTNETISIS
SNVQIMYDNKILLTTFLIQTIVDTFTIVLNQSIFLLKSSLFINLYRSLAEFDCSLSRVKILNEN
FYKIPRLVILSHLIIYVLLQIFLPVLFTYLMLIFNIPHFVTYFCIYINNLGTFIIDLHVIIIINLLK
QRFYKINEVLYDITFIDSSKIGKPFSRLKSLSQLGNLHSQLCDTCKIANDAYNIQLLIGILRKC
TVILIKFNLLAIIIVNGCGPFTSYMWYIPSNSLLCWYIVTLILLAMSSQKLSHEANRTGVLVH
KLLAKTKDPEMREELQLFSLQLLHRKVQFTACGFFPLDFTLLYSIVGAVTTYLVILIQFQLS
YNDDGIQNTTNSPFLATTMPTLSYVNDSLR
>BgerGr54dJ
MSSAAKDVYYVIYPLFCVSKVFGFTPFSYNLKSYNEKLKYSKPAAVYVILLLLINVTNETISIS
SNVQIMYDNKILLTTFLIQTIVDTFTIVLNQSIFLLKSSLFINLYRSLAEFDCSLSRVKILNEN
FYKIPRLVILSHLIIYVLLQIFLPVLFTYLMLIFNIPHFVTYFCIYINNLGTFIIDLHVIIIINLLK
QRFYKINEVLYDITFIDSSKIGKPFSRLKSLSQLGNLHSQLCDTCKIANDAYNIQLLIGILRKC
TVILIKFNLLAIIIVNGCGPFTSYMWYIPSNSLLCWYIVTLILLAMSSQKLSHEANRTGVLVH
KLLAKTKDPEMREELQLFSLQLLHRKVQFTACGFFPLDFTLLYSIVGAVTTYLVILIQFQLS
YNDDGIQNTTNSPFLATTMPTLSYVNDSLR
>BgerGr54eJ
MSEKPNVFYVVYPLFVVSKFLGFSPFEYKIQGTRKTLQLSLPAVAFTVILSFFIILNQVFLLY
KSFKYLKSQTLLLTESCILGSVNILTTVTSLSMFLIQNSSHRNLLNRLSNFDELISDIKVYVK
NDYNIPRLIILCHLYTSFIVSMVASVITYYLFKSIEINLFLSGFLFSIYKLRSFVIDIHVISFNLLI
LQRFRKINEIIKCLIKLSTSRKSVAQFKFSRNQIVLPRKLGNLHSNLLDISVLANSIYGLHVLI
GILRKCVIILYAVYFLAVTILKEDVGPYKDIMWFIPPNLAMFWSILLLINLLLSSQYTSEEAN
RTGVLVHKLLAKTKDPEMREELQLFSLQLLHRKVQFTACGFFPLDFTLLYSIVGAVTTYLV
ILIQFQLSYNDDGIQNTTNSPFLATTMPTLSYVNDSLR
>BgerGr54fJ
MSRSQQDLYYVLYPLLYVSKLIGLTPLCLNRQFSNNSFKVSPPALTYAILLTFLIVTKQAHR
FWKQLNYPDVHLNVVWIVIVSILISVNIVTTLITHSVFIVKSSSFRKLFQRLSEFDELVSTMN
DIKELDHDVSRFRIMCHVCMALFFCVFYFVFSTFIESTSVVEVNYFTDQFVLFIINFTSSFTD
IHVFIISQLIKCRFLKINNIIETRYFVIKMKKYTLTNSKLALEFKNNKINLKTLENLHSHMCN
VCQLANDTYDVQMLVCVFRKCSLLLLLAYYFASTVHKIANGAYAVHMWSSSKCFGLCFCA
VHLTYLVWSGHTMAKEANRTGVLVHKLLAKTKDPEMREELQLFSLQLLHRKVQFTACGF
FPLDFTLLYSIVGAVTTYLVILIQFQLSYNDDGIQNTTNSPFLATTMPTLSYVNDSLR
>BgerGr54g
MSRSQQDLYYVLYPLLYVSKLIGLTPLCLNRQFSNNSFKVSPPALTYAILLTFLIVTKQAHR
FWKQLNYLDVHLNVVWIVIVSILISVNIVTTLITHSVFIVKSSSFRNLFQRLSEFDELVSTMN
DIKELDHDVSRLRIMCHVCMALFFCVFYFVFSTFIESTSVVEVNYFTDQFVLFIINFTSSFTD
IHVFIISQLIKCRFLKINNIIETRYFVIKIKKNTLINSKHALEFKNNKINLKTLENLHSHMCNV
CQLANDTYDVQMLVCVFRKCTLVLLFAYYFASTVHMIANGAYAVRMWSSSKCFGLCFCAV
HLTYLIWSGHTMSKEANRTGVLVHKLLAKTKDPEMREELQLFSLQLLHRKVQFTACGFFP
LDFTLLYSIVGAVTTYLVILIQFQLSYNDDGIQNTTNSPFLATTMPTLSYVNDSLR
>BgerGr54h

MWRSQQVLYRLLYPLLCISKLIGFTPLTYTKQFSESSLKFSPQAVAYAIILLLLIVTKQAYHL
WRNLNYPDTHLNVVWIVIVCIQISVSIFTTLVTHTGFIIKFSSFRKLFLSLAEFDKLVTAMN
DFNELDNDVSRLRVMWHIYMALFFCAFYFILATFIESTHLFSIEYFADQFIFFVANLSSSIIDI
HLLIMSQLIKCRFLKINCIIINFTDIVSEVEMNAVINNEHFKCKNNKINPKTLGNLHSLLCNT
CEIANDTYGAQLLVGIFKKWTLILLFVYYYALTVHNINNGAYANQRWSISNYFALCFCVKF
VFYLARVENLLRILQRANRTGVLVHKLLAKTKDPEMREELQLFSLQLLHRKVQFTACGFF
PLDFTLLYSIVGAVTTYLVILIQFQLSYNDDGIQNTTNSPFLATTMPTLSYVNDSLR
>BgerGr54i
MSGSQQELYRLLFPLLFVSKLLGFTASVCSRKFSENSLKLSPLAVFYAIILTFFIIAEQLHAL
WYQLCHPDWHLNVVWRVIVSIQISVSIFTTLVTHAVFIIKTSSFRKLFLSLAEFDELVSAVN
DGKELDHDVSRLRIMFPIFIGLSMCALYFVFCISVVPPNMLDTDFFVDQLLLFVTNLSLTVI
DLHVVNMSQLIKRRFIKINKIVVKLASELKINGIKYLSFRKTIIKARSLCHLHSFLCNVCELV
DSIYSLQIFISIIKKCTLILLFVYYFALTIGHLYRGPYNDNLWCIPIYFALCCCVVNLASLIWSS
HAMSQEANRTGVLVHKLLAKTKDPEMREELQLFSLQLLHRKVQFTACGFFPLDFTLLYSI
VGAVTTYLVILIQFQLSYNDDGIQNTTNSPFLATTMPTLSYVNDSLR
>BgerGr55F
MQKYLGIMKLFHNISTILGLSPYRLSFGRALKQSRVAFLYAVSCLVLTFISNATGLYFMLLQ
DIEYHALVKGTIRLICFTISIVTYSICSIERIVKRKKISQFSNKLSYLCSRCTGMSEVGSDQWYI
LVYSTAGLVLIVFFHFTHWYDLCLSFKSMYMPFFFIRLLCTFGSHVTFCQLLYYYKIINTAIL
HINRKMYLNRKVLSCNKTSPASLLTNLNGQDDTKTELLNRISDVVQLHNILCEVVKSMNY
CFALQILFTIAGICVTFTYNVYEVVLGILNFQIDESVHTILYNSSWAMWKFFKVILLVKFCD
DCTEEANRTPDLVHKLMEKNKETEIRQELQLFSLLLIHKKIHFTACGFFPLDYTLLYSIIGA
VTTYLVILIQFQITFSEHKTTFESMFNNITVNTTE
>BgerGr56a
MATQSIYYVIYPIFIASKILGYTPFSYDGQIRHRALRFSLPATAYSAILITIIAMALCYALWLA
VYINSNGVFIVASFIQIFVSTVTSVVIQLAFIFKQNSVLKLYESISEFDETLQQIEENHTVIRLA
SMLHLLTSLFFFVIFSAMDLSLFGLLTIETIATQLALCISSFNVFVIFILVISTSHFIRKRFSKIN
SKIVDMHLKSTTYHVNINNNNRNFDMKLQSNEKTKQLQVLYSNLCNVCDVANSAFGVQL
LMVILKTWLVIVDEIYLIIMPVIDEAKVVLSSDKWYIPSNIYLIWSIVNLIIFLWSCNGLSQEA
KRTGILVHKLLIKTKDPEIREELQLFSLQLLHRKVQFTACGFSPLDFTLLYSIVGAVTTYLVI
LIQFQLSFNGDVKNTTNSTTYFYSSTAPSFFNITSG
>BgerGr56b
MNTKRDIFSTTYPLFYVSRLFGCFPFSYNGQSSLNSLKFSAIATSYSATITTVIISGYTLQLW
NILNNAIFPTNTAFSTSASILAFINMMIIIVIQSTFYGGNELFRKLFIGFSEYDKIFVQSRLNEI
NDRSRLLMLCHLFIPIFVCAIVIVYDFVFSNIFNIGRLAQELLLFTTEIRVFLIDIHIVNVTQLI
KQRLENINTTVIKLSDLINCKFTNNSQNIRALGNLHSRVCDISELANRVYSLQSLTVLCRKCI
IILSDAYFVTLYVLEEERGSNSAGWYVIPTTLSAIWCATNLFIFIWSSYAMLQEAKRTGILV
HKLLIKTKDPEIREELQLFSLQLLHRKVQFTACGFSPLDFTLLYSIVGAVTTYLVILIQFQLSF
NGDVKNTTNSTTYFYSSTAPSFFNITSG
>BgerGr57
MLDVFSEINPIFWMLRINGMFCFNLRGKIGFRELKFSKILGIFNVIMLLTRLIVQLKWIQDL
LVQQDFSLGGSIVVFLSVTIGSLSSTFCMGLNLIQNHKLLLFFNKLSEFDRSVTQIEDRHFKT
LTKTLIMVFVTLLTTGLSLLEKLIRKYSDPIDEIIIVYVDYLSMMVFLSQDAFFLICSQMIKER
FEVLNLEINSLMECKQGTNKLRDNCRKRKIFVSATNKKLISLPNRLEKLIYLHDLLVDVSEL
LNSIFSLRNLISVAIKFIVLTSYFYVMVTWAVYYLNFSFIFLISNILAGLCLAPLIAVLFICSSAC

QEAKYTAVLVHKFLQKSEDLEVRRKLQIFSLQLMHRKVVFTACGFFPLDFTLLHSIAGAVC
TYLVILIQFQPTFSLKLLNPHSQRVNNTYTDNLLGPGVETVYRTNVLS
>BgerGr58aF
MHFLGDFDIYSSISPLIWIMKIHGICQLSIEGKIHCREIKLSKISLIYSFIIFLLMFIVQLIIFQEF
VFSFQFKIHELKVSYISMSIGNILFLIGMNLAMFKNYKIPDVYKLLSLFDDLKVINRDIYKKS
FVNIVKQLILGFSIILHPFIIHIYRREYILHSLDNYIFLLILLTQNLLFTNLVLAVKNRFILLNTE
IENYTVDKRQESDLLDKNTHFLKEFKRLVHSHELLQKVCDLINCTFSIRNLLSISYTHLDVY
KRSHELLQKVCDLINCTFSIRNLLSISFTFMLITYCIYIVMSQCLTSGTYLNDSINSLSLFINFL
TSLGTAISIIWSCDSTTTQANRTGILVHKLLKKTDDPELREELQLFSLQLLHRKVQFTACGF
FPLDFTLLYSIVGAVTTYLVILIQFQLSYNDDEKQNSATSTMLISTTQHYDNML
>BgerGr58bF
MNFMEEFDIYSEISPLIWIMKLHGFCSIRFEGKVHYREIKFSKVALIYSFLVGIFMLFLGLFII
KMFVRYDLEALEMKLSLVLLLGTRILSLAIMFSSIFQKSKLPQLYKFLSTFDEKMSVNPFQY
KKAFIQISTKLILSFVAISGQFYFNHQMHYGLADYFDLGILLTNGLLFSSLVLALKKRFQLLN
LKLLKWSRLNNHENIFFGENEFELGAFQQLISFDKSLRDCCDLINSSFSIRNLFLFIHMLIFV
IYAIYVLLISRGFRQDTPLNHPNVAFSIFANFAICLFTLVDVVWSCRSTSSEANRTGILVHKL
LKKTDDPELREELQLFSLQLLHRKVQFTACGFFPLDFTLLYSIVGAVTTYLVILIQFQLSYN
DDEKQNSATSTMLISTTQHYDNML
>BgerGr58cF
MKDLHCILSSLFNISRVLGYFPLFREANNKISKCLNVMYSVFITLIISLNLSFITWIVIFQNDL
DIVVSITTVINNFIHVTVLLIIQFSMLFKFNSLQQLLRKLFLFDKLFWNTYTTAKLSSFKYQI
FINVMLIIIFTTAHYFYIICNMGIPLVCIFLLVVLCMKGFIVDLQLINLMLLLANRFSAINSIID
NEYDMKQNEKCVESSEKMKKFKSSLASRSNILFNCNLMRFHSFACDNSEIANSTFDVSLLL
AVLPKCVVIILYVFFQSLLVFKTDGGPLAGNKCKETSVSVVVLNVCGLVAILWSCTSVSRQA
NRTGILVHKLLKKTDDPELREELQLFSLQLLHRKVQFTACGFFPLDFTLLYSIVGAVTTYLV
ILIQFQLSYNDDEKQNSATSTMLISTTQHYDNML
>BgerGr58dF
MDKRKDVYYILYPLLCVSRIFGFTPVSYNGVLSHKTLKCSIPTAVYSAILMIIVVTNELFVVY
NYLYNSKSNLVFVTTTTMQNCVNIFSWIINQSVFLIKMNSLLRLLRYLSEFDELLVRLKKLS
NRDYRIPRLFILCHVVVTVCLCIVLCILIFYVLNFVFIQMIVSELLLLLNLGSFVVIDVHVITIT
SVLKQRFKKMNSIVVDVSHFVNGHTKISMLRSLHSRLCEICELANSIYNIPLVVGIIKKFTIIL
VAMYFISLAILQQNVGPYTDLLWSFITSSFMFWCLLNLVIVLWSSHAMSQEANRTGILVHK
LLKKTDDPELREELQLFSLQLLHRKVQFTACGFFPLDFTLLYSIVGAVTTYLVILIQFQLSYN
DDEKQNSATSTMLISTTQHYDNML
>BgerGr58eF
MAPTKRDIYYAIYPLIVVTKAIGLTPFSYNGQISHKSLKFSFSAATYAIILTFWLVINEAIVLF
TTLNYSMNIILTAASIIQNSLDILTLIVIQSTFLAKATSILKLFRCFKKFDESLASVYEMKEED
YKIIRLFIVFQSFLNVFTHVVLIILAVSSMHSFHVCTFLSEFGLGINRFSIFCIDLHVVNIVHLL
RQRFRIINENIVILSNVSGMVEYARMKNDNVARKLENLHSHLCDMCEFANSTYSAQLLMA
LFRKWLIIMGCMYFSVLIFVKVNKGLFSGTLWYLPINLMLCYSILSFGLLLWSSRALSKEAN
RTGILVHKLLKKTDDPELREELQLFSLQLLHRKVQFTACGFFPLDFTLLYSIVGAVTTYLVI
LIQFQLSYNDDEKQNSATSTMLISTTQHYDNML
>BgerGr59a
MAPREGTVFYYTIAPLFYVSKFLALAPFSVEVSAGSTKFKFSRLALMFSVALAVILTFNQLIP
IWYNFNDLQYEPVFLVFTVIEGVLYFTATIFMRFGYMLKIGVLHELLRRFASVFEVLCLIRK
EIFKIYRCFRAIVIFEIFVVFCFTCPPIFWVLAFANQSVLIYVSNFFSVFNFLVICITDLQAIHF

AMLLKHSFFTINEVLNDFNQVLNSASRERLQTQKKASKVLQSQPKTNIYHPNSCSILRRVH
SDLFDISEFMNEIYSLQALPSILMKFVMTVINMYYWSITVLDNDRGGYAESDWNLQYAGL
QIWGVIFLVLLSWSNTAAFDEANRTAVLVHKLLNKTKDPIISEELQLFSLQLMHRKVQFTA
CGFFPLDFTLLYSIVGAVTTYLVILIQFQLTFEDRGNATSSTTQVNFTSESLMTQKI
>BgerGr59bP
MTLKCTSDVFDEINPLFLLMRVHGLFPFVLAREKNCRKLKSSKFWLIFSIVLFIVKIVIANFA
FKDLVHENEFSSYSRKVHVFGFVCASVLCSCIVFINILPSPRLLELFRRMVDYDNEVSEVFKI
KKHVFSILYIITISNISYLILYFANNDGLITAIFISFEDHLDVVGILCNDVFYVVIVSILKRRFQH
LNLKLEKLLQFHKSESKFGKICKRNRIAILSARRQTSYIVLKVKNLTYLHHLLCEKCQLLNZ
LFSIRMLFSVANKFIISTVEYDYIMTSLLFRPKATIILVFTAIMQVSSSMGPLAAIIISSTSAAR
VANRTAVLVHKLLNKTKDPIISEELQLFSLQLMHRKVQFTACGFFPLDFTLLYSIVGAVTTY
LVILIQFQLTFEDRGNATSSTTQVNFTSESLMTQKI
>BgerGr59c
MSMDFLGEFDIYSEISPIIWTMKIFGLCQFQYKGKIHSRKMEFSKFSLVYTLIIFSCMLLIQPL
VAQMLVLNYPLKVHNFKILLIFECFFNFLSHVLVILSIFQNSRIPDFYKLISKFDAILSQNNSQ
CRKLFRNNTILVILTVIIMLFTFVVTSEFLFGLIGSYDFIIMLTQDMLFSNLILTLKRRFNSIN
LNIQGHIKFKNEKHSNYLAFHDENNHKLKQIEELTQLHELIRENSDLVCSIFSFRNLLSVIF
KFIFITHAIYIEISEITCPQMKVDVEVFSVLFLTQLFYILGSLVFVIWCSSSASKEANRTAVLV
HKLLNKTKDPIISEELQLFSLQLMHRKVQFTACGFFPLDFTLLYSIVGAVTTYLVILIQFQLT
FEDRGNATSSTTQVNFTSESLMTQKI
>BgerGr59dP
FYYNANSRYCMLFSNLILALKRRFNSINLNIQGHIKSKNTKQSNLLLFHDENNHKLKRIEEL
TRLHELLRKNCDLVCSIFSIRNLLSVIFKFIFITYAIYIEISEITCRQMKVNLEVFSVLFLTQLF
YILGSLVFVIWCSSSASKEANRTAVLVHKLLNKTKDPIISEELQLFSLQLMHRKVQFTACGF
FPLDFTLLYSIVGAVTTYLVILIQFQLTFEDRGNATSSTTQVNFTSESLMTQKI
>BgerGr59e
MDFLGDFDIYSEISPLIYTMRFFGLFQYGLRGKINYREMRLSKFSLVHSLIVFTFLLFLHGM
ALPILFKIPLTKHEFRMNFSIQISMNSICFLLVLLPMFQNYIIPSVFKLLLKFDGVIRPNLNYY
RKSFVNNIVQLILVSSSIFITLASTKYGLFSVIAVYNITIMLSQDLLFCNLTLALKKRFALLNS
HIERYISFKKDNARVYDLKKLKYLYELLTYTHDLVHSAFSVRNLLSTALKLIFLTYVYFIIFC
QSFQICTMTDPNVLSVLSIVVVQFLCLFGVLVFTVWSCRSTSVQANRTAVLVHKLLNKTKD
PIISEELQLFSLQLMHRKVQFTACGFFPLDFTLLYSIVGAVTTYLVILIQFQLTFEDRGNATS
STTQVNFTSESLMTQKI
>BgerGr59f
MAAEMTSDVLDKFNPLLWLMRVHGSFPYTIFKDKHYRYLKYSKFWLFYSMIIIAFNTISQ
TAILPTLYFSDPQSSYIEKLALLILILPYFLSGFAMVSNVIPSALLQKLLENTMDFDRKCLHF
KNSCYKTWAFVLYITVIVAYGHIHLYSYDYYTFNWILITFENHVYIILMLSQDAYIILIFSIVK
ERFQYLNLEICKLLNYEVNIGAENEVVLRKNISNPKYLMRLQDSLYTIFELSNSFTSARSLFS
ISQKYFLTTTYLYSISIWYVHGRQFIIAELLPYAVLVQLLFVIPLIATIYASTSTYYAANRTAV
LVHKLLNKTKDPIISEELQLFSLQLMHRKVQFTACGFFPLDFTLLYSIVGAVTTYLVILIQFQ
LTFEDRGNATSSTTQVNFTSESLMTQKI
>BgerGr59gP
MTSGVLEEFNPLLWLMRVHGSFPYTVIRDKHYPYYKYSKFWMLYSMIIIIAKIITQTTIIPIL
YYSETRPPSIEKLALLVLILPCFLSAFWMLTNVFQSTNLQNMTKKIMEFDRKTHFQISFNK
AWVLVSYFIIVLAYGHFHLYICGYFTGTFEGYLIVFDSHZNLVLHLTQDSFIVMVFLMIKER
FQHLNLEIGKLFKSEFRISLENDKVRASNTSDLCIGKNIPNPKHLMRLQDSLYTIFELSKSLI

SIRSLFSISLKYFLIVTYLYNLIIWYVRGREFVVIEFLPYAIIVQVLFMAPLVVEIYAASSTSYV
ANRTAVLVHKLLNKTKDPIISEELQLFSLQLMHRKVQFTACGFFPLDFTLLYSIVGAVTTYL
VILIQFQLTFEDRGNATSSTTQVNFTSESLMTQKI
>BgerGr60a
MGAPQRKDYYHTIAPFFYVSKFLGMTPFSVTTVNHSKIFKISRTAMAYSILMAAATFIRHV
LYLWFTLDKYKFENVAFITGTIFSLTLLVIVISIQVFYLLKSDQICEIWNSFSKLDFIFWTFSD
KRFSTRRVDRAVVLFEMFILVIIFSALVLKYLSFNNRGILFITSYFLFSFTNFIQYVNDLHFIN
STLLLKRYFNLFNHKLLSNHAFGIDYKCLRMIHIHLYRVFELVNTIYGVLVLLGMPVKFVTV
ITHLFYWTFVLLEYQLFGFGVDHWILTFAVHLIWSVLMIIIVPWSSNEFVCEANKTGVLVH
KLLTKTKDPEIREELQLFSLQLLHRKVQFTACGFFPLDFTLLYSIVGAVTTYLVILIQFQLSY
NDDGKRNETSSSMAPFTTSSYGDVSLA
>BgerGr60b
MTYAYNKDVLYVFLPIFHFSRILGFVPFFHKVNSAGKLLTASFTVLYTMSVTVILLCNEFSA
IWGFFYEIDAEITFVVITIINSLFSMVHTSLIPTAIFLKRHRLRLYLERLLAFNFSFCNFSFNA
KELCSFNLKRQLFLIGTIFLVFVIFFPLFVMCNGSLPIWWRIVFCIDTLKAFLIDLVVCNAML
SLYNRFSLINVVIDNLAEYESEQNRYKPLLNYVKILRESHIYVCEIGELSNSTFSLITLLSLAS
KCFFVTTYFFFKILTFLQYDRGLFSGEICNVGSVVALVFGLLNMVFILWSSMMAALEANKT
GVLVHKLLTKTKDPEIREELQLFSLQLLHRKVQFTACGFFPLDFTLLYSIVGAVTTYLVILIQ
FQLSYNDDGKRNETSSSMAPFTTSSYGDVSLA
>BgerGr60c
MTFTYHKDVLYAFQPIFYFCRILGIVPFFQEVNNTRQLLIASFSVLYTVCVTLIFLSNEFLLIR
SFLYDIDAEITFTVIIVMNSLFSMVNTLSIPSIIFFKRRSVHLFLKRLYDFNFSFCNVSFKSKE
VFTINFKRQLYLNVGIVFVYIGFFPIFVMCNDSFSTGWRIIFTMDILKVFLFDQIVTNAMILL
YNRFSLINLVIMNLVQDQNKIPRERYKHSLNYYKSLRESHFYACDISEILKTAFSMIILLSITA
KCSFLTTFTFFKILILLQYDRGLFGGGICQTGSIIVLVLTFLNLVSILRSSSMTSREANKTGVL
VHKLLTKTKDPEIREELQLFSLQLLHRKVQFTACGFFPLDFTLLYSIVGAVTTYLVILIQFQL
SYNDDGKRNETSSSMAPFTTSSYGDVSLA
>BgerGr60d
MSSGHQRLYHAVSPLLCVSKIFGFVPLSYNGETSKRRLGLSTAYVAILSSVIVLVQVFKLWD
TFSYINEHTVFNSAAGVQNCVYVVTSVVIQATFLIKHNSLPKLLQFLSEIDESVSILKNFSDR
DYATPRLVILSHLALSSVLGIFFSVMVVYFFEFTSIAIFICYIVITLNTLGTFIIDLHVITVGIFL
KQRLTKINSIVLESVSLIPNTRCPRKVNLQLKVRSTILGNLHSRVCDVCEFVNESYGIQVLAC
VLKKCTIMLIEIYFAAVYILKEDVDTFTNILWQIPSNVLLSWCVFNLILLLWTSQSMTEEAN
KTGVLVHKLLTKTKDPEIREELQLFSLQLLHRKVQFTACGFFPLDFTLLYSIVGAVTTYLVI
LIQFQLSYNDDGKRNETSSSMAPFTTSSYGDVSLA
>BgerGr60e
MSTKPNVYYVLHPLFCVSRALGLTPFSFNKQISNKPLKFSLPATVYAISLALIMIGVEIFQF
WYFLVSLKSVFLTATLIEVSLNTFISIVTTGSFLTSNYLFQKLFQAFYEFDESLWSIIKQKNE
YKNIRFLNICHLLLFSLPCLILSTLNMYLLHILSFKTSVANLFIFLSIFGMFVVDCHVINVTQL
LRQRFKTINEIVLDLSFNIQSEIRPSVMNPEVEDISEKTRKIASLYTRLSDICDLANSTYNIQI
LMSLLRKLTLIILRVYFLTLVILKQDLGPFANRQWNFSASLNLFLSAVDLIFLAWSSHSMSQ
EANKTGVLVHKLLTKTKDPEIREELQLFSLQLLHRKVQFTACGFFPLDFTLLYSIVGAVTTY
LVILIQFQLSYNDDGKRNETSSSMAPFTTSSYGDVSLA
>BgerGr60f
MAFSDEKDYRYIFLPFFNICRALGFIIHFQEVKYDPKQCLIALFTIVYTSIVTVVLLCNGFLS
MWFLMYRTKVEITLSVIIIAKLLIYFANILFVPVNFFLKRDSLRIILRSLRTVDLSCFKRCYGT

TWLSSLNLQRQILVNLIFICVFTLFCSIYMRCNGGYPIWSRIIIFGHNLKIALIDLNIINLMML
LYNRFSLYNFIMNKIVEDENSNINIRKTYCGKQENWRKWIITKLRKTHSSLCEVSELTNST
LSLVILLSVLVKFIIIICFSYFMVLTALEYQRGLFAGKDCFVGSLLSLTFSIFDLVFVLWSSSRA
SYEANKTGVLVHKLLTKTKDPEIREELQLFSLQLLHRKVQFTACGFFPLDFTLLYSIVGAVT
TYLVILIQFQLSYNDDGKRNETSSSMAPFTTSSYGDVSLA
>BgerGr60g
MHFYCALSPVFYLSRVLGLFPFRVVEENGTKEMKLAHFHLYYSLIFQINYICLQCYSVWGNI
MNENVPIIMKITIFIQDFTSFVSYLFLLGFLVFKHHDVPNLLNKIYNVGEVLDVNENSSKVF
LLQIFFIILNMPAVYIIYASANAVQYVPDRMVFLPYFVSCLNTVTGMVLDLQMLIVISIFEQR
FSRINSLLDITSLSRKRSSLTNESVTSRLKLINRVNVLVSDVFDVVNSVYGLHMLVATINIFL
TIIFNAFFVCTSFLKTDRGLFNGEVWTMSACATTVMCSVDFTWILWTSCLASKQANKTGV
LVHKLLTKTKDPEIREELQLFSLQLLHRKVQFTACGFFPLDFTLLYSIVGAVTTYLVILIQFQ
LSYNDDGKRNETSSSMAPFTTSSYGDVSLA
>BgerGr60h
MPYSYQKDFYYIFKPLFYVCRILGFMPFFDEVQGGVCEVLKACLIVGYTMAVSITLVVNALS
SEWFLISKSNIQMSLMIITSAKALGNIVNYLSIPHFILTKLQLFRILLKKLPAFNILFSKLVLKS
SVDYVPAFKRQIFINVSFILVFSIYCSVLQMCDGGFPIWTSILVGVGDLQTCLVDLNIINVLIM
LRNRFSWINSLISNLFHYNYINQPSQKVNFKTVILKLQENHSYLCDISDIAISTFNLKILLMV
TANLIIVTSFSWFLVLTFLGYDRGTLAGDMCVLGSVLAVIFHAVDLVFIVWCFSSVSYEANK
TGVLVHKLLTKTKDPEIREELQLFSLQLLHRKVQFTACGFFPLDFTLLYSIVGAVTTYLVILI
QFQLSYNDDGKRNETSSSMAPFTTSSYGDVSLA
>BgerGr60i
MTYSYQRDFHYIFLPLFHISRVFGCMPFFHEVRGNVKQTATAVLIVVYSILVTIIFSSNALYV
VWNLVFIANIDTPFLVAAVIHKLGSVVDILLMPSIVFFKLNRLRILLKQLSVTNFFNSSFGNI
DRVCSGNSRRLTCLNILSLLAISTCCVLFLSCRPNVNYPIWSPVITFLSIVEESLMDLNIINLM
LLLYNRFFIINVIAENIIENNSNCAPIPKYWIVFNLRKWHTNVCEMAELTNDTFNSLILISVL
KKCIVITTFSYFKVLIGLKFDRGPYTGKTCLLGSISTLAFSASSLVLILWSFNVTSSEANKTGV
LVHKLLTKTKDPEIREELQLFSLQLLHRKVQFTACGFFPLDFTLLYSIVGAVTTYLVILIQFQ
LSYNDDGKRNETSSSMAPFTTSSYGDVSLA
>BgerGr60j
MSSKRQDVYYVLRPLLGISKVFGLSAFSYNEDVANTFKFSPLAAAYAAIFTLLSLWNQVHY
SLVTSRRLTSSADTSVFLKTSTVSNVVMFLTCVICHTVCLVKHDSMSKILQCISEIDQSLQQF
HFSSYRINRLIILSHLALSSALVIFFTSINCFLMQRSNAGVLVSCFFIIIETQSLFLMDLHIIAIT
GLLKQLFNKINTIISIVICSNSKNNNNESDIKKISNLLHFETSGVNPPLTLTLIGHLQSRLCDI
CELVNSSYSAQALIGVLRKCAIILVQSYYLLLCLLDEDRGEYTDNIWHIPSNVLLSWSVLNL
VLMLWSCQRTSQEANKTGVLVHKLLTKTKDPEIREELQLFSLQLLHRKVQFTACGFFPLD
FTLLYSIVGAVTTYLVILIQFQLSYNDDGKRNETSSSMAPFTTSSYGDVSLA
>BgerGr60k
MPRQRDIYYVLEPLLFISKILGFSPFSYNDKFTTRPLQFSLPAAIYSAILISSMTVCLIFVFLIS
SKSHPNLVYIIASSIQIGVNVTSSLAIVASFLIKSESFLDLLDRLHEFEKCLSRIKKFNEDAYK
AARLAIQYHLLSTSLYCAMYSVICYLLLNFVLSEQFLSCLVLFIGNFSLVIVEMHLITFSKLLK
QHFKTINENIISNISSTTSKFKSHFIGLKNTQVKPIKLAHLYSSLCDICELTNSTYNVQLLMSL
IRKWCIIMDCMFYLALVILRVDRGQYSNSFWSIIPNMLLFWCSLYLILSLWTSRDLTQEAN
KTGVLVHKLLTKTKDPEIREELQLFSLQLLHRKVQFTACGFFPLDFTLLYSIVGAVTTYLVI
LIQFQLSYNDDGKRNETSSSMAPFTTSSYGDVSLA
>BgerGr60l

MPRQRDIYYVLEPLLFVSKILGFTPFRYNDKFTTRPLQFSLPAAIYAVIFKLIIMTYQCVVFC
ISAMFHPNYIYIITAAIQLTITVFSSVSILLSFLIRSELFLKFLEYLNEFEKYLSQIKKFNEQDY
KSERLVIKYHLLFVSVYIGLFSTIIASLITNLSLFETFLTSVLLFLVTFNTSIVEIHVINISWLLK
HRFKTINDSMSIASKIKFTENIELIKNIQTNPRKLADLYSRLCDICEHVNSTYNVQFFMYVF
QCWFVLIDNMYFLALGLLKEDRGDYSDTIWNIIPTMSLFLCILNLTLSLWASNSMSQEAN
KTGVLVHKLLTKTKDPEIREELQLFSLQLLHRKVQFTACGFFPLDFTLLYSIVGAVTTYLVI
LIQFQLSYNDDGKRNETSSSMAPFTTSSYGDVSLA
>BgerGr60m
MEVSRTLKMLFKISQIVGIAPFCVEDTFKVRPSKRALAFSVILCLAVVVLEGSGFYEYIWDN
STVSFIGVILVTFSSIVTHFTCCMLALNNSTELILILDELTAMNHLLATNGKSFKIFRSTIIHII
LGFLSILVHKISFILSHDRKLYLSLLSQLVSTFGDFIIIMQFKTLVQLCKYHLIALNSRLSDLE
NIFSSIDNTNIITVLQSKPHLDAKSKINVQDIVYFPKSEFKFNPGLRSQALSLTVDHNKICDV
VFRVNKAFRLQILFLTMKIFIFITFSLYFIANNVIAGRYYFQHGSDAMHLFVIAVYKLLLIAE
PCFSAVEEANKTGVLVHKLLTKTKDPEIREELQLFSLQLLHRKVQFTACGFFPLDFTLLYSI
VGAVTTYLVILIQFQLSYNDDGKRNETSSSMAPFTTSSYGDVSLA
>BgerGr61
MSDALDVFSPLLWVMRVHGSFPYTIIGDKRCRNLKYSKFWLIYSIIILISNIVSQSAMMFILH
YSDSIFSDRERLSVLVILFPYALSVFSMISNAIPSTKLQKLFQKVMKFDRESIFQNSYSKAWV
MLLYIIIVVGYGHFHMYISDFYEFNAILITFENHFYLISLLTQDAYIILIFLMIKERFEHLNFQL
AKMLQYEETKSKIVTKNVNGRITNALEVVNRKTISKPKHIMRLQDLIYSIFELSNSLNSLKT
LYSISLKYFLIMIYLYSVINWHLEGHEFVVIHFLPYAILIQVLFVVPLVATIFSSSSTSYEANRT
AIFVHKLLNKTKAPNIREELQLFSLQLMHRKVQFTACGLFPLDFTLLYSVVGAVTTYLVIII
QFQLTLQERGNITATATEVNFTSE
>BgerGr62aP
MTTIYSMLDSTIILLKVVFSPLAFTFERKFTEIAAVIWSIGICIIALLFMLYALDEVSQITVNIC
NFSNNILNCLVTLEIIVSQITSHFNREKRAQAILQLNSIINKIKFFTWKRTKCFISILIFTKISIL
LATMLVQYFTDIDQFPLYTVFLFFYFELLVNILVIQFEVFLVIVRHLFEILNIKLEEVTEXYN
RTETRLKQICENYETLCEISESVNYSYSFVGFLHFAYYMCTMLIFNDVLCNRASTHGYSYSV
WLLYGMTYFLAIVYLAQSAACEGNRMTILVNKAMERTNVPALKEQLQNFSQLLLHRKVK
FTACGFFPLDYTLIYGLVGGVTTYLVFLIQFQQALKHPTDNNTEATAVID
>BgerGr62b
MKMSSIYSMLDSTIILSKLVFSPFAFTYERKFTKILAVIWSIGICIFASLLMLYALDEVSQITV
NICNFSKNILNCLVTLELIISQVTSHFYHVRRTQEILQLNSIIKKIQFFTWKRTKCFISSTIFIK
MFILFVTTIVQYFSDVDQFPLYTVLIFFYFELVVNISVIQFEVLLVIVRHLFESLNIKLEEVTE
KYNGAVTRLKQICENYETLCEISESVNYSYSAAIFILVSRAFVGFLHFAYYMCTMLIFNDVLC
NRASSLGWSYCLWQLYAMTYFLPIVYLAQSTACEGNRMTILVNKAMERTNVPALKEQLQ
NFSQLLLHRKVKFTACGFFPLDYTLIYGLVGGVTTYLVFLIQFQQALKHPTDNNTEATAVI
D
>BgerGr62c
MYSSEDHPSVYSLFGPIILLSKCFFTAHFIFDDLKPITKTSVAFSIGMCMVLYFMILFSYDNV
EISTDANMLCQIVQYALCCLIPLAVIVNNINSFLNSKKTAEQFSKLNTIFKNVPLSIWIKSKQ
CRSVILHFNVVGVLIIYLIDYLTFSKKYPIYTVATFIYLELCCNIPIIQFKGILIVLKYSFRTLNM
KLEESFYKSNLNYRIKLCEASDTKRNFNKEMDLSTSNCILKNIYENYVALCDISESLNRFYS
VVILVWVGRSWTTLIYTLYFVCTAVIFNDVCYSSPAPTYLMWFSFYFIYIFAFVYLAESAVK
EGNRMTILVNKAMERTNVPALKEQLQNFSQLLLHRKVKFTACGFFPLDYTLIYGLVGGVT
TYLVFLIQFQQALKHPTDNNTEATAVID

>BgerGr63a
MTIMHRPTTKKIKSVYSLLCPTIILSKCLLSAPFITMYDRNHVKIANIICCFGVCIVSAKIVMG
TLDYYIFRYTLNVCSYGDALLCASMVIGALVSYANSYVFYENTAIQFARLNEVLNRIKFSM
WSKSKNCNRMYLFVNCIPILILHLYEYSATMIDYPFYKAMTYVYIDFCSYISAIQFSGILIILK
YSFKTLNMQLKNTNKNHERFNRFLKNSGHLRVHEINRNANIYRCDMQMKPPEIDLKNIF
DNYETLCDIAERFNRSYSLILLFWTGKNFSSSVQAAYYLYTVVLIPHSLCDTGTTGVYASTM
IYTLLNLLAIVYFTQSTANEANRITILVNKAMVRTNIPTLKEELQHFSQRLLHRKLRFTACG
FFPLDYTLIYGFVGGITTYLVILIQFQEALYPAKNSTIPVPGSQ
>BgerGr63b
MHSTKQNNTVYSVLGPTILLSKCLWSAPFITMYQRKFIAIGNIICSIGVCIYSIKMVIDAMKY
HVFRQTLNVCSYDEAVLSYMFLLIILVSYGTSYVFYKNTVLQFSRLNELLNRMRFSMWCES
KNCNTIFFWVNLIPVFLLYLLEYYTSNVQYALTIAIIFAYLEFCTYISVIQFDGIMIILKYSFKY
LNKELKNMIKNHMSVKRLSRKYEVIINREVKCNAHFHGDGVAVRPPETYLKTIFANYETL
CDIAERINLSYSLVLLLFVGKLFGCLVHNVYYLYTVVIFPHSLCNNGTTPVYASWTLYFFMN
LFGMVYLTQLTVNEANRITILVNKAMVRTNIPTLKEELQHFSQRLLHRKLRFTACGFFPLD
YTLIYGFVGGITTYLVILIQFQEALYPAKNSTIPVPGSQ
>BgerGr63c
MPKVYSVLGLTILLSKLFWAAPFITMYERKLTAWAYMICAFGVCIGSFLLNLASLKYFITEF
AQQVCNFSETVLCSLLVLQLIVTYVNSYLFYKNTALQFARLNALLKRINFSMWSQTKNYN
ALYISANFITVILISIFQYFTTITEYPLYIPVIFNYIEFCWKIYGIQFHGILIVLKYAFKTLNIKLN
YINVKYKNANRHSRNSQVISVHDMKDCDNVRGDGVLSETDLKPILENYENLCDIAEYFNLS
YSCVLLLWVARTFISLVHIVYYLYSVILLPDYLCDVGTTPAYVSWMIYYSFNLLGLVYFTKM
TTDEANRITILVNKAMVRTNIPTLKEELQHFSQRLLHRKLRFTACGFFPLDYTLIYGFVGGI
TTYLVILIQFQEALYPAKNSTIPVPGSQ
>BgerGr63d
MYTNKERPNVYSVLGPIILLSKCLFSAPFITMYKRKLVALTNIICTFGMCIGTLIMTISAVEY
HIDLLALNVCNFSEAFLCCLLLLMVMVSYATSYLYYKNVALQFSRLNALLIRVRFSMWSQS
RKCNTVYFSINFIPVILITTLEYYSSTTGYPFYILVMFIYTEFCSNILLIQFNGILIILKFSFKALF
MQLNGFNENQRNFIRHEQIQEVIKNGVNIHSAVRLNNTETGLTPIAENYETLCNIAESFNR
SYSFVLLVWIGRLFICLVHDVYYMYTAVLFPDVLCEIGSASAYLSWLVYYLFGLLSTVYFTQ
TTTKEANRITILVNKAMVRTNIPTLKEELQHFSQRLLHRKLRFTACGFFPLDYTLIYGFVG
GITTYLVILIQFQEALYPAKNSTIPVPGSQ
>BgerGr63e
MNSPRKMPSVYSILGPMIILSKCLWSAPFIIMYKRKVFALVNVICSTGVCVGCIKLIIDAFGF
YNQRLKYNVCNFSELFLFALLGLVITVSYITSYMFYKNTAVQFSRLNTALNRVQFTMCSNS
KNIILIFVVFEIIAIVITYFLEYCFLSVKYPIYMLCAFIYLELCTNISIIQFNGIMIILKYSFRSLNV
KLKYKIENNTKTSKFSKNYLQEIDLRHVFKTYEELYDIAESFNTSYSFVNLIWVGKLFICMV
HTVYYLFTAILLPYALCDEGTSPAYFSWMTYYCFSLFTTVYFTQSTSIEANRITILVNKAMV
RTNIPTLKEELQHFSQRLLHRKLRFTACGFFPLDYTLIYGFVGGITTYLVILIQFQEALYPAK
NSTIPVPGSQ
>BgerGr63f
MESSNKKTGAYSILSLTVVLSKCLCSAPFIIPYNKKITVIASAIVSIGICTCSIYLVLQSFFYYVL
RLNINDINVCNFSQTVLSSFLSMGIIVSYITSYLFYKTIAWKFSQLNKIFERDPICFQAKMYN
FIYIIVNAVTVTFTNLFEQYLVKGRVGHAILFSYLELCSIVPAIQFTGILIVLKYSFLILWTQLN
ELVKNYARSCISIKKLNESNLHEIYSLHQEFKQVIDYYEELCDTAESINHSYSCSILVLIVRIFI
FLIHMVYYLFAVGLLPKMCEEGSTLAYFIWFSYYIFIFYGLVYATQSVTYEANRITILVNKA

MVRTNIPTLKEELQHFSQRLLHRKLRFTACGFFPLDYTLIYGFVGGITTYLVILIQFQEALYP
AKNSTIPVPGSQ
>BgerGr64aP
MAVQMSFPILADIFSEINPVLWIMKIHGLFPYTLIGDPGRRVLNFSKYWLIYSILIFVLKIVT
QLLVIIDKFNSQQQFIPNIVLHVMFASSVFSTICIVFNFIPNDKLAIVFKXISIFDNVVEYPKSR
FLKTLLINFSLMCLMFMRLSYSLKLIQNLLEEKYLAILVSFQYYYDALSLFCQDSQLAISALM
IKERFQYINEFLVEIKEREAVNQNTTRKFFRKRQNNVATLVNVAEMRLLMSSQNLLCEVC
DLINSLFSFRVLISVLIKFLISLKYSYHTVDIVMKSSSFGVLLTGGILFGTPILTLFGLLYCCSR
TCFEANRTSVLVHKLLNKTRDPKVREELQIFSLQLMHRKVQFTACGFFPLDFTLLYSMVG
AITTYLVILIQFQLTFNNDSSPMFMNSTNSSVI
>BgerGr64b
MVRKLIKMVVSASHDVYTQINPLFWVIRIHGFFPYALVGGIGNRCLKISKTWVMYSIPMLM
FNITCSLISCKNMFNTNGITSGKDKIAVIFAIVVRITYSCGMILNTVPQKVLIIFFKKLSEFDS
AANYSVIIFWKTIKRYCVVFLYFIFTIFLSARWLILFVCVKTNMKIIPFVQFYFQLISILSQDIF
IAVSVSMVKDRFEYINLLINRSIKTQLLNYNDVGGTFNIRSKSCVTQKCSHHRSLMELHNFL
CDVTEIINSQFSLIAIGSILFKSFDCIAYFYVTISYYDKFSVEFVLLWGVSTLCLSVISFVSTLCC
YRSANYEANRTSVLVHKLLNKTRDPKVREELQIFSLQLMHRKVQFTACGFFPLDFTLLYS
MVGAITTYLVILIQFQLTFNNDSSPMFMNSTNSSVI
>BgerGr64c
MLSKMGITTSHDVYSEIKPLFWVMRIHGLFPYTLVGDLRSRCLKISKFWLLYSIPIFVLNILG
RILCCEELLYNAESKFSTKEKITVIVLFVTSALSSCGIFLNIVPLKQVINLFSKLSEFDNTAHY
AQKLYWKILERYFVFILYFNMTLYLMLYPVIQFEFDGTSRMFLRCVQYYSEVILILSQDIFIV
LTLSMIKERFKYVNFPIDQTGEGRFSNFTYMRGTHNFRWRSTIAQSAFFLVDLMKLHNCL
CEVAELINSLFSFRIIVLVLYKLFHSVVYLYILISTVEDLNVEATLIWGFGILGLSVISFICTLFG
YTSALYEANRTSVLVHKLLNKTRDPKVREELQIFSLQLMHRKVQFTACGFFPLDFTLLYS
MVGAITTYLVILIQFQLTFNNDSSPMFMNSTNSSVI
>BgerGr64dP
MKLYDSRDYXLFWVMRIHGLFPYVLIENRGVRCLQLSKIWLFYSSMIFLIKIVTAIIWILMV
YHAVPCVNKVHTTNTLIFLIAPCLSTFGMFLNIIPNDKFSILFIKLHAFDITIRNYQVPFCKKI
ITKIVIGISVLFIIMYCGFRTLSTLLNLFLYFILTIFQYYMDEINILSQGMFLTFTCLIIKDRFTY
INTVLTKMERAHENTTMQWGNKQXANRTSVLVHKLLNKTRDPKVREELQIFSLQLMHR
KVQFTACGFFPLDFTLLYSMVGAITTYLVILIQFQLTFNNDSSPMFMNSTNSSVI
>BgerGr64e
MSNILFNSYGEIIPLFWIMRLHGLFPFNLSEITNDRGLILSKSWFIYILLILTSKIISQIVWTLH
ILCDIVFGYRKSSINRLVWIGVNWMVSFCTILNMIPNEKLLHIFRILSKFDSIANETMQFKK
RRMKIVLFIIFITFFTYLGLYDRYTTLYHTFQIWALECFEFYFGMFTVIPQFTLQIIICLLIKER
FRYINKLILKIRQKVDHNIGLIQSHRRQEHKNFYLNSAVTVSEINHLMWLRYLINEVCDLTS
SLLQLRILLSFFSGFYTFASFVFWVISCLTQGGIHMMLSESMIGNIVIGITPYSSFIIMIHICRS
TCIEANRTSVLVHKLLNKTRDPKVREELQIFSLQLMHRKVQFTACGFFPLDFTLLYSMVGA
ITTYLVILIQFQLTFNNDSSPMFMNSTNSSVI
>BgerGr64f
MFSNIRGSRRDAYSVVNPLVWAMRIHGLFPYTLVDDVRNRGFKISKFWLFYSIPIFLWNII
TRLLYFEDLYNIVFSSKDKIMVIFIIVTSTLFCCGIFLNTFPQKEPINLFRKLSEFDSVAHYAP
VVFWKTLKTFSMFIFYLILTLYLSLYTVINVNIDVLRLHNIYLVLIYYEVISILSQNTFICLTLS
MVKERFQYVNLLLSQTDEIQFINNVNGTHNIQTTTQSCFHLWYLMKLHEHLCRLTDSVNS
LFSVRIICFASYTLFITVNYLYFLIVGLENLTVSTGIIWGFSTLNLSVISFVCTISCYSSAHHEA

NRTSVLVHKLLNKTRDPKVREELQIFSLQLMHRKVQFTACGFFPLDFTLLYSMVGAITTYL
VILIQFQLTFNNDSSPMFMNSTNSSVI
>BgerGr64gP
RYTFLTSTINNYFVTVSKFYFITQILYTQLPRSLPHKLIMNKVVLSVNNISIIVSSCLCSLCMF
FSIIPNKKLALLLRKLZIIDSTLPSDDAKFRKGTGKLVIIIFLFSLICLCLNCTFLSRINNNSGVI
LSCYQFHFDMITTISQYLFIITITSMIKERLLHINRQFDTINQNTKNNTNVSLRSSKENRKLL
NCVANRTSVLVHKLLNKTRDPKVREELQIFSLQLMHRKVQFTACGFFPLDFTLLYSMVGA
ITTYLVILIQFQLTFNNDSSPMFMNSTNSSVI
>BgerGr64hP
MVLKKLSDAYEDITSFFWIMRIHGFFPYVLSRPTTDLGQVLNPSKIWFLYSVIVFLSKIITQI
LWIPQMIEVNFSSSQTSNINLSIYVVFSSFSTFCMLLSIIPDKNLTLLSRKLSVFDSALEGVEI
CDKTTKILIRVYSIFFLISMGLIFKNGSEYLDNYQLVVKSFQAYSDMVIILSQHLFLIITVLMV
NVRAVIFNRLFDGMNVKENANLNINSKVWNRENMHNISKGNASTVXCMDIQKLTTLRE
MLDEMCDLVNSVFSFRELASIGFVFPVSSSFLYWTISYQTSEEFNIVFTSIFGISFLGMVPLA
SLMAMLYSCTSTCFEANRTSVLVHKLLNKTRDPKVREELQIFSLQLMHRKVQFTACGFFP
LDFTLLYSMVGAITTYLVILIQFQLTFNNDSSPMFMNSTNSSVI
>BgerGr64iP
MAIHVYSETVIPFWVLRLHGLFPYVIITKTGDCCLKZSKIWFLYSILILFSKIISEIIWICYIVN
KTSYKDSIERINGLLFIVSHCLSSLCLALNIIPDDKLSLVFKKLCAMEFKILNVSYRSRKIRVXI
FILIYLICIEIFSCTSIIHVLWFNTSLHITVSSGIQYYFDAITVQLHDMFLIILFSIIKERFLYINA
VFDRLNLRESSTQNKFQITKITETYEYNRSLRNIKSQIAFRGSDVTHLMSLHDLVKDAFELF
NSLFSFRVVVSIIFEFLTSSTHLYWEISVAYLAMENFSLXPIAVIGMLIYALLPLASLTVLLYF
CNSTCLEANRTSVLVHKLLNKTRDPKVREELQIFSLQLMHRKVQFTACGFFPLDFTLLYS
MVGAITTYLVILIQFQLTFNNDSSPMFMNSTNSSVI
>BgerGr65aF
MNLLGECDIYSEISPIIWVMRLLGLCQLRLEGKIHSREIKFSVISMIHSFLIALMLLIFECLIIK
HFIENFQLDIPELKVTLLTSTILHYVSLIIILLTVFQNSKFILVYKLLRTIDDKSFTNPCQYRQ
LFLNRIILLFFIFGAVVSPLFVDVFMGNNVPKVFVLSIFVNWYFLISVLTQVIFFSNLILAIKV
RFTLLNLRLEESIKRSSEGNLSFEENKLQLGQLNILHNLLSDVCDLISSIFSFRNLLCTAYAF
TVITFFVYIMVTQSLRSRTEIDETRVSCLIVIHTAIVMDPFIFGVANRTAVLVHKLLNKTKD
PHIREELELFSLQLLHRKVQFTACGFFPLDFTLLYSIVGAVTTYLVILIQFQLTFTANDNNN
ATYLIPPSNYTS
>BgerGr65b
MRMTMKFLEDFDIFSEISPFIFMMRILGSCQFHLDGQIHSRQIKFSNFSFVYDILVVIISLIPF
LATINIGIYDYSFDIHGFKITFITHIALWIISFVHVIFMMLQNTKISHLYELLSAFDKTFPNDI
QYKASFMNASIQLLLVASACLLAEEYILFLPNSTFYALVDFRCIINTLTHDLFFSNVIMGINK
RLSTINLATIRCFKLKNKENSNNLLSLHDKIDRDNITFQQLSYFHESLREVCVQTCSIFSAG
NLLSIAHKFVALTYCMYIGVSRSIRPHTSLDEGNISFTLIIHSSCLLATLIFVAWNCNNISQA
NRTAVLVHKLLNKTKDPHIREELELFSLQLLHRKVQFTACGFFPLDFTLLYSIVGAVTTYL
VILIQFQLTFTANDNNNATYLIPPSNYTS
>BgerGr65cF
MNFLGEFDIYSEISPLIWTMRILGLCQFRLEGQIHSREMKFSKYSMIYTLIMAALQFVVLSIT
TITFINLVMLHFIEVRVAFVFSLIMSALTPTILLSTISQNFNISIVYNMLSIFDKRFLTISSQYR
TSFIRVTIQLIFQISFIFIAFVPSHFIQNNVIYGILAFYQLTSVIMQDMFFFNLILIVKRRFVVFG
EAMVDYVSKKNQDHLLSPTIMTFINEGKSDLKLSDQLTYSFELLTQVCDLLSCTFSIRNILS
VSFKTVQAIFCVYIAVSGSIRSSEILDDQSLYLALSIQFSCCLVALFLVVWCCSSTSMQANRT

AVLVHKLLNKTKDPHIREELELFSLQLLHRKVQFTACGFFPLDFTLLYSIVGAVTTYLVILIQ
FQLTFTANDNNNATYLIPPSNYTS
>BgerGr65dF
MNFFRDFHIYSEISPLIWTMLIVGFCPFHLEGQIHSREIKLWKFPLVYNIILLVIVIILRPFAK
QTFILDRPLTIHEYKLSFISSIILDSISLILLVSMIIQNSKITYIYKLLSKFDQNTVSNYYQYRISF
ITVTVEVVLAVTATVLAFVTTHAPKDVIFEIIGIHDVTTVLIHDIFVSNLMLSAKRRFEMVN
LTLVHCIRHKRRGNLTALPSYHNENGYNPKMFELLSQNYALLREASDQVSSAYSTTNLLSV
AYKFIYFTFCIFMTCTRTIRSHTSLDEEPIASSIIMQISYKLLALIFVVWSSCTTSTEANRTAV
LVHKLLNKTKDPHIREELELFSLQLLHRKVQFTACGFFPLDFTLLYSIVGAVTTYLVILIQFQ
LTFTANDNNNATYLIPPSNYTS
>BgerGr65e
MADTDIYSAFIPLLCFLKQIGMTPMTVKGPIGSRKFTFSKTSIAYSVAAVSLVIGVRYTILKL
LVNYNESSSLLVTVLNLGDICHFIVTSTLVFLGLLNSFNSAQIFESLSKFDKILGGLNYLYLRA
TRFTRIQMYLGISSCLIFIVVLSSNDLLHLDRFTLLISILIDLFVIFIVTMTLIIKILLLRQRFHY
LNKVLLTSLEYNQPSPSQFQRIHLLPGSVPATHAPNNLAIELQFLRKMHSFLCTICEMFNSF
YSVQAVFVIAVSLMVATLNLYISFITCTFWNVSLLSVYETSWYIGNCGVLILSAVFTAQEAN
RTAVLVHKLLNKTKDPHIREELELFSLQLLHRKVQFTACGFFPLDFTLLYSIVGAVTTYLVI
LIQFQLTFTANDNNNATYLIPPSNYTS
>BgerGr65fP
MLFQLFFCIIMWNSSTILPLLSSRRDFLYLGIFIQDIMKLSVLIMINVINNFLYNIFEEISRRES
SLKEIFRDQFRNELTLVLIYSAAVIVTLSFCSFQCCYSTDILFRVLLNTARFSADIYIFFALQV
FILHTRLLQHSFSYINRKMSSEIRKEMSKSLESLDEHQRNTIAQLASFHNMLHDFVHLVNY
AFGPQILLNIAELIVTLTCDMYYIFLFSWENYMKHVLVMVYAGFYSTKLLQLIRSCSYTTKQ
ANRTAVLVHKLLNKTKDPHIREELELFSLQLLHRKVQFTACGFFPLDFTLLYSIVGAVTTY
LVILIQFQLTFTANDNNNATYLIPPSNYTS
>BgerGr66a
MHLKKALHRAIYCSDDSFLCFSRLFGVIPFRILTNTESEKYEISNIWFAYSVFLLIVHFAMHI
NFFLESSVENQIPFVLSKSTIYISTFNFICMVVIQIFSLKYSRALILIANKLSFINIDSNFINLKN
RKLIFSSINNTIIIINIIMLILVIYTSPSYVFRIDFIFVWLLAIGLILVAELQFIHFIIALKEKFNLI
NMNIKCMLNFSQNNKKSNLRYFSSCRVSQSKHESSITNKLIKKRIYTLANSHNLLCQIAAN
LNQAYSVQITVISTVNFLQIIISIYYIVSLMILCNETFHPTVMQTSMIVTFLCFFSLKITFVVCA
CSFTSTEANRTGILVHKALGKAKDEYIREELELFSMQLLHRKVQFTACGLFPIDCTLLYSIA
GAVTTYLVILIQFQQTYDKLDHSSNVTTPSP
>BgerGr66b
MDVNADMKFMLTVSRAFGFAPFSFNSTRYHYKLSKFWSLYSVFVIIVLVFINFALDLVYKF
YDKKLLIMRLSSTFISYFVFLSAIGSPVICLVNAKKNLAVIHKLSSYATILKISQISSRKLSKKY
LFCLFATFFPKMSTIIWQIVQTRKYIHYKEVRSSVKFIILLSIDDIFIWLVNLQLIFFLLLLKEY
FYVFNMNLMVSLMNNANKNIRNSLIAAGTGNDIFCYNFDFVSKISLRKVFMLHNSLCDTV
EIVNKLFSIQILPYLTFIFSEIVISMYTSCNLILYPYKENTISAKVTSPIHPFIWFLIFLLQLFILS
SVCNSTSYEANRTGILVHKALGKAKDEYIREELELFSMQLLHRKVQFTACGLFPIDCTLLYS
IAGAVTTYLVILIQFQQTYDKLDHSSNVTTPSP
>BgerGr67aI
MKNISEMKIFRDRDTDFAFKFILYLSKIIGLIPFSWNYNGVCFELRSSHLSFIWSLFIFLTFSY
FMFFTLVNNILNNLESVPTMIYAFTQVPIAISYPVSILSIWCQREKINSLLRIILECDCTLHRK
STRNLKSVIIQLLSSCTLTSVSLWLHVQNETKEDSLSLIFYVPPVISITVIQTHFVNCVYLIKQ
YFGDLKISLQGLICSGRQKDSKMVNSAMAFHNSLCEIIDLNSSIYSYPLLFNSLLFFSVAIFNV

YYLASNLLNAMRPTNNIAMVISLCYSVMNFLLLTQSCNSCCQELQLFSLQLFHRKVNFTAC
GFFRLDYTLLYSISGALTTYLVILIQFHQSFEVRDPCGNVTCVCPND
>BgerGr67bI
MKLFEAKETDLAFKIILYLSKMFGLIPLSWNYNKYSFELNISLMSFIWSSLMFLILLLVLIIN
TIQIFSNSFNSFLSLIAVFVHFPIVVCYFVSIFSIWFQKKKINNLLRMLLICDCTLHIKNTRNLI
YVILQLLISFTLVTVSSLLHTLYDVVYETLVLIPIMFSILIVQSQFINCVCLLRQYFQSIKMSLE
HLIDSNREGYSREIDFCMTFHNILCDIVLDSGTIYSYPLLLNTLLLFIFGIFNVYYLTTNFMD
NNATINNVAMTISFIFVAFNLLLLTQACNNCCVELQLFSLQLFHRKVNFTACGFFRLDYTL
LYSISGALTTYLVILIQFHQSFEVRDPCGNVTCVCPND
>BgerGr67cI
MKFFEDNETDLAFKIILYFSKIFGLIPFSCDNDKHILKLKLSLVSFMWSSLVFFITIYFLFKST
ISLNDTDNRNPFSNSIGVFVHLPMVFCYPVSVLSVWFQRKKINNLLRMILICDITLHLKNTK
NLMYVILQLFILMMSVLISSILHIPYNTKCEMLVVIPVMFSIVIVQSYFVNCLCLVRQYFQFM
KTSIENLIDNDRKIYSRVIDIYMYFHNILCDILEESNIIYSYPLLLNISLCFIFGIYNIYYLTTNL
LDVISPINNVAMIVSLLFSMFSFLLLTQSCNNCREELQLFSLQLFHRKVNFTACGFFRLDYT
LLYSISGALTTYLVILIQFHQSFEVRDPCGNVTCVCPND
>BgerGr67dI
MKVFHDKETDCAFKIILYISKIVGLIPFTWESSDTNFEFRTSFIGLIWASFLFATFLYNLISIM
VHDFAAYNDTTLQVVGVLVKLPFFICYPVSTLSIWLHRDKINAFLKNLLICDQTLNVKTPH
ILIYIIIQIISSLILLILSTVLFVYNDGNVRIIIYIPIMISISIVQLHFVNCVHLIRQYFQFLNLSLEN
VSCFRGKIYSQVIEFAMSVHNTLCEIIELGNAIYSYALLQNTLLLFFNATFNLYFMVGYYLQL
MPRNYNTAMICSLCFSMLNFLLLTQSCNKCTYELQLFSLQLFHRKVNFTACGFFRLDYTLL
YSISGALTTYLVILIQFHQSFEVRDPCGNVTCVCPND
>BgerGr67eI
MKIFQAKQTDSAFKFLLYISKICGVIPFTWKYNEVNFEFKSSLISLIWPLLLFLVLSYFALILL
VKNPLGYQIPISVLIGKIVQLPILIAYPVSVLSMWLQRKKFNELLTNILSCDRILNIKKTNNL
MSVIIQLLISFILITLSSILFIRFKSGDQMYIYIPILICITIIQIHFVNCIYLVRQYFQCLNVSLQKL
TINDHKTDILLLKHAMFFQNSLCNIIELSSAIYSYSLVSTVLFLFLLAMFNVYYLTANFLDV
MPQSNNISMIFSLCVSVYNLLLLTHSCNTCTRELQLFSLQLFHRKVNFTACGFFRLDYTLLY
SISGALTTYLVILIQFHQSFEVRDPCGNVTCVCPND
>BgerGr68a
MYNCEIVYRFDWIKALVFKSKLLQLFPLSIKRNSNGLMYSETSYSKLLICFSFHSLVCIVYYM
STLHWISNTQGMTKISGIYVGTDIIVSYALTIYMCIRYSNKMYNFILKACVSETSISKLSIEGS
SRQQSLLMVIESILVAFCSITSIIQSKYNFTISVNQCTWYVVFMSGFVINCQFLTFIDIQKKRF
EKINSDISNVMETSLLYKNIPSKIIINSSKTKAILSKMNAIMTCHGAFCNDIEEVNGLYSFFLL
LEVIKLFEDITFVGFLIIQLIFFDMSSTSIEGILNRRIQSLHLLLWGVAEAIKFNTILNKCRSSA
KEANRTAQVVHQLLNHTIDPKLKEELQLFSMQLLHRKVKFTACGFFPLDYSLLCSIVGAVT
TYMIILMQLEFPN
>BgerGr68b
MNKYFEKVYSFDWIKILMFKSKLLHLFPLSIKMNPYGVIQCETSYSKLILSMAFHLTVLIVY
YGTAMTYISGADGVMKTSSIFVGTNIVLYHALTIYMCLRYSNKMYNFIIRNCGSKPNSCNLS
YKQNGHKLSFLIFIESMLLASSTICASVHSKGSLVTMVNQCCWYVIFMSDFIITCHLLTFNDI
QKNRFEKINIQISKMVETNRLHKHVQCGTSYNLKCTNYKKSLAEVMSNTDVIVKIHSLFCK
EIEEINRDYSIFLLLQVKNIFETITFFGFLIIRLLFLSKDSTNILHAESVHLLFSTAAEIIKFNAI
LNSCRSTTNEANRTAQVVHQLLNHTIDPKLKEELQLFSMQLLHRKVKFTACGFFPLDYSL
LCSIVGAVTTYMIILMQLEFPN

>BgerGr69
MYQEFEKVYSFKSIKRLFFVAKLFQLIPVSIHKDSQGIKYSITSIPKVILSLLFHLPLCLVCITT
TTVWLSDLPDMVTKISGCFLTFQVGIIHVLTIYVTIRYSNKICNFILSACIYERNVEVNSTYM
ENRWKQLILLIMVSLPRIIPMISGIFYLTDGDLMAFSEGCWYLVNMSDMITIYLFLTFVDIQ
KRRFQKLNSAISKMVEIKRKIFCKKNAISQNYKTNSITTNNVDTFPSNRMLMACHCHFCD
VTEEISSLYSTFLLIKITKIFIVNTYCAFILIQLLFNSVNESFEGMARSIQIIQFTFWSISEMVKL
FDILYTCGSSAKEANKTPQIVHQLLTYTVNPKQREELEVFSMQLLQRKVQFTVGGFFPLDF
TLLYSIVGAISTYMVILTQMQSQTEV
>BgerGr70a
MFIAVRMTILISQIIGLIPLKIYVKKNGSINYENSFILLSYSVILFVCLSIFCLQCNPYSWSKTSD
PVDNLSNYFIYLNYMMCYIIGYISCILKYYKILELFAELQPFEKVCNISNIKFNILQISSSVIILL
HSVVCLSRCFIKRNSEIFFSTTLIALYSFMIENQFISLCFILFIKFQIVNENMIETAVLCSNYKI
PLKKLISTASRVHSNLCNAMKKVNDIYSIFILLVLARIFYFLSICFYAYAYFIIYDIRIIDLEAGT
RLVLWTSLNIVRLYQTVEGCQSATTEANRTAVLVHKVMMKTKDPILKDELNLFSMQLLH
QKVQFTACGLFPLDFTFLYSIAGAVTTYLVILVQFQETYKMHNVNNSTGITNSSIIETAQ
>BgerGr70b
MYNFLRFTLFLSQVIGLLPYRQYIKQNGTLFYKRSSLLLLYSILLFLTINCTLFIRFNIWFCLI
GQNPVKNTSNSIVFVGYIVSYAIGYVACVLNSSLIFPLFKEFERFTKSCNKYTGYIRIFSTLQL
IMGLIFLMDTFCLVSLPLGKVQKEIFISSAFISLCNYTVENQFYSLCLILFIYFKVLNDTIVKE
NNLNLSREINVCKVVRGEKSFSAVARFHSSLCDILTKVNGIYSIYILVALARILYGITLCFFVY
ANCLIYQLKIGTFAYVQATFWAVYNTLNMYQLFYCSTLAANEANRTAVLVHKVMMKTKD
PILKDELNLFSMQLLHQKVQFTACGLFPLDFTFLYSIAGAVTTYLVILVQFQETYKMHNVN
NSTGITNSSIIETAQ
>BgerGr70c
MYNFLRFTLFLSQVIGLLPNRQYMKQNGTLFYRRSSLLLLYSILLFLTINYILFIRCNLWYW
LIGQNPVKNTSNSIVFIGYIVSYTVGYVACVLNSSLILPLFKEFERFTKSCNKYSGYVQNFSAF
QLIMGLIFLMDTYFLLSQPLDNTQIEIFISSAFIFVCNYTVENQFYSLCLILFIYFKVLNDAILK
ANSLNLSREIYVCKVVCGENSISAIARFHSSLCDILKKVNGIYSIYILVALARILYGIIFCFFVYA
CCLIYQLKMETFAYVQTTFFINYFIANRTAVLVHKVMMKTKDPILKDELNLFSMQLLHQK
VQFTACGLFPLDFTFLYSIAGAVTTYLVILVQFQETYKMHNVNNSTGITNSSIIETAQ
>BgerGr70d
MCIYVRSNLLISQLIGLIPSKVHEKKTGLVTYKRSQFSLAFSITVFVTLNYLYISCNPYGLGTT
DEAVDNTSNLIEYLAYLFSFIIGYLTCVLNSSKIISLFQELRLCEKSRSASNVITRFCVLKFLVL
MFFSVLNLLAIVRLFTNRRYIDVYIYTILISVYNYTVENQFISLCFILFIKFRNLNDCILNLNQ
EICIGSVRIKSVFVAAKVHSKLCCIMKSVNDVYSLYIVLALARTFYGLTFCLFGFLYCIIYFITF
EIWTNVELFFWIGYHTLQLYQIIHSCTLAATEANRTAVLVHKVMMKTKDPILKDELNLFS
MQLLHQKVQFTACGLFPLDFTFLYSIAGAVTTYLVILVQFQETYKMHNVNNSTGITNSSIIE
TAQ
>BgerGr71a
MTNSATKALYFIYSLSKLFALYPCIYSTKDGSFAFKYSRRSFIFSLIFHLSILVFPCIRVINSWK
LALSIKEVTVIRLAGALIETFGILQYLLSALTILYNNKNLHSLLSAMVFWNKQYRTNKYFHI
QIVSLFLLGVLLICMFALHAWYVSSKKLLYMVFFLYHSYILISILVTQISFVTFVKHFKFLIEN
INLHMSDIYSAKTNHKFTNVNSISVRKIQKLAKSYNLLYENVELTNYIFCIPILIFMASCFEFI
TFSIYYLFWGFLLFDRFLYNFPFFVIFMITYYTLKLYLVTSSAHECKFEANRTGKLVHKILN
KTSDPRLEEELQLISLQLLHQKIQFTACGFFPLDFTLIYSIIGAVTTYLVILIQFQQTIVLQNR
NTTGLKFQ

>BgerGr71b
MGPSVDSALCAIYYIAKIFGYIPNIYLEKNSKFIFKFSKGWIIFCIILNSFVMLYPTCFSIVMFI
RSWPFHTMERLSGHISQITVLLENLAALICLIFNNKEIQKFLNLLNIIINSQRIKRQEFEGKR
YIFIQICSVTIVALNALYFSLDNSQNFWSEEAFYFVISTLRVYSSYVSQLIFVTLITMLKNSFS
QLNLEFQRIIRESVSSTMISAETMKLAAMFHMNLCYLSQMVNSIFSVPILFTCSRMLFFMT
YGIYILISHLLNVDLTSKVTTVVISVILYCAFNIMLLTHACEICCFEANRTGKLVHKILNKTSD
PRLEEELQLISLQLLHQKIQFTACGFFPLDFTLIYSIIGAVTTYLVILIQFQQTIVLQNRNTTG
LKFQ
>BgerGr71c
MTVYMESCIYIIYYISKLFGCFPNIHVKKGFCFVYRFSKSWMTYSVVLQLGLTLLPTVGAILG
LITSLSEHSVSRLTGFCSQLIIYLEGMVYFVCVSSNNKEIEKILNVLNTTIYNLRFRKQRFKG
KYLILIQLCLLTILVLFSLYLFLGIRKRYWSGFSQPIYFTLSCIRVYFVYISQLQFVCLIIMLKYS
FARVNSEFQEISLKTIKNTKLTTQQVKLAGIFHKYLCVMSQKVNYIYSMPILFSTIHVFIQAT
SALYLIISQLYSIEAIKDMQMLSFIILSYNIYNLSSLSYACDSCCIEANRTGKLVHKILNKTSDP
RLEEELQLISLQLLHQKIQFTACGFFPLDFTLIYSIIGAVTTYLVILIQFQQTIVLQNRNTTGL
KFQ
>BgerGr71d
MAASIDLSISIMYYISKCFGFFPNIQMEKGVCFVYRFSKFWTIFSVFFQLLQTLYPIFNIILELI
SLWTNHSMSQLSGYSSQFIIYLEGLVYFLCVVNNNKEIEKILNSLSTIIYTLRFRKLKFKGKYI
ICFQLCLLTIISFLLLYFFLNIRKNMVSKFTLVHVTLSALRLYSVHVAQFQFVSLIIMLKYSFL
QTNSELQELVIKPTTSTNVSVRKVKLAGILHNHLCILSQKVNYIYSLPILFSTIHKFIEATSAL
YLIISRFYNIEIVKDIQTIAFLVFSYNIFNLSSFSYACDSCSIEANRTGKLVHKILNKTSDPRLE
EELQLISLQLLHQKIQFTACGFFPLDFTLIYSIIGAVTTYLVILIQFQQTIVLQNRNTTGLKFQ
>BgerGr71e
MTAYIDSSMYIIYYISKVLGYFPNINIEKGVFVYKFSKPWVIYSILFQTILILYPAVFLVFSFIIH
WSYPSMALISSLSAQCIATCESMICFLCVVINNRDIEKVFNLLNTRTKIYRLRIEKQIFKATN
TIALQVCLLTVASFFILHYLLTHKKTSEYIYLTLSSLRLYSVYINQFQYICFIILLKYSFAQVNL
ELKESFMKQDTNIKVDFSIDQVMSSGVFHEHLCILQQKINGIYSLPTLLAIVHMLSQATSAL
YLFISKLYNIETVFGMHVGFFIFSYSMFNLISFSHACKSCCSEANRTGKLVHKILNKTSDPRL
EEELQLISLQLLHQKIQFTACGFFPLDFTLIYSIIGAVTTYLVILIQFQQTIVLQNRNTTGLKF
Q
>BgerGr71f
MEDYIDSSILIVYYISKLFGYFPNINIEKRNCFVYSISKPWIAFPVVFHATVSVYPLICMILYFS
DIESLSMLTITNLVSECAVFVGHLVSSVCLAFKNKKIHKLLNLINTIICKLNICGRNFSGRYTI
ILHLCFQSITVISASLLCLIDIKYTWESITIFAILFNIKVYSVYVIQFQFLSFIIILNYFYSQVNVA
FQEIVHKTVRNIENITKKVKLADALQRHLHVLTETVNDVYSVPMLCSVIHMCIQATGCLYL
ILLKLHNFEAEPPLYQQNTGYIVCSYSIFYLTTFAYACNSCCLEANRTGKLVHKILNKTSDP
RLEEELQLISLQLLHQKIQFTACGFFPLDFTLIYSIIGAVTTYLVILIQFQQTIVLQNRNTTGL
KFQ
>BgerGr72P
MSVSVDSMLYIIYYNSKLFGYFPNIHFERGMCFIYRMSIPSIAYSVVFQLIFSLYPTAFLLSEM
NIISSFSSMINYIGFISEFTIFFEHFVCLLCVIFYNKDIEKLLNFINFVINRLKIRETKFNEZYIII
FQLCLLCIALLSTYFISLGYKKGVGFQISLCYIILNIRLYSVYITQFQFLHLVITFKYFFAQINAE
FQEIFHKIFSNMKVSSQEIQIATTFHSQLYILSRVINCIYSLPILFSMVHLFIQATYCLFLFISK
LCNLSLAEGVHNVGLIGFSYTILNLIIFTHSCRSCCFEANRTGKLVHKILNKTKDSRLEEELQ
LTSLQLLHQKIQFTACGFFPLDFTLIYLIIGAVTTYLVILIQFQETIVFRSRMNTTNNDI

>BgerGr73a
MNRNVNIYVFIKPFYYVSKIVGLAPFYFNRRENSVTVLNTSKFGLIYSFLLLSIFGAPFTVAM
LQGNQAYRVTSIQDFSGILEFITAFLAYIIVNILNLYNFRRNKEIINKLCLMERLLQPRHKSY
KMESIFSSLELGLGLLPIALRQIIAGSSVPYLTIAIYYTIATMLFYYSIFILLFQFINLLTVLRMH
FRDINLQLDSQDESTNKLMRGFIKRVSPINRIRYGGLSALVTRYDEICGVAEILNKTFALELL
CIVARTFVCLVCELYGESSYLFTDKSHYGVVDTLLSELWITFNAVLLVSLSYACSSVMAEAN
RTAVLVHKLLNKTRDPAIREELQLFSLQLLHRKVQFTACGFFPLDFTLLYSIVGAVTTYLIIL
IQFQLSYENKDSNLGNGTTALPSPVNNASS
>BgerGr73b
MYIFVFIKPLYYVSKALGLAPFNTRKDNGIKKARCSKFAIWYSFFLWSIFLGPILISFLIKEDG
LFTSTSVRAFSENLENSFNVGVYLVVSIMALIKRKNIVELVNKLRLIEQFTDQRTQSLKLLFI
SSFLQLVLGLFPGALHKLVSYSSYFSSINITIYIIITTILYDYCMFILLSQFTNLLSVLHRHFRD
VNKKLKFVIESSEKRNENFIQSVRSITNNKNLGLLPKLFTKYDFICDLTQITNKTFDVQLLSI
VARIFISLVCTLFMESSFYLTSKSHPDFLHTLASKIWILFNAVLLVWVSYFCSSLLTEANRTA
VLVHKLLNKTRDPAIREELQLFSLQLLHRKVQFTACGFFPLDFTLLYSIVGAVTTYLIILIQF
QLSYENKDSNLGNGTTALPSPVNNASS
>BgerGr73c
MNRGVDIFVFIKPIYYVSKILGLAPFCTGKERYVPKSTGKERDKNLSITAFSNRKEIYIRNLR
TKFALAYSLFLWIIFVMPVLVIIMYAIIDGEATQMTSIRDFNESVEVFFAFGTYAIVSIMSLC
RRSNIIEIVNKLCLMEHSRSESPKLKSLLSFMQVVLGITPIAFRYFVEWWISLRHIFLIFTILV
SMLYDYSSFVLVSQFTNLLSVLRMHFRNINSKLSSRDESCTGPGKGVVQRVVDTRSREHDG
LSPFVISYDEMCDVAEMVNNAFSFQLLCIVAKTFVCLVCGLYLESSYHFTKKSHPDFTKTL
ASVSWIIYNAVILVWLSYSCRALRAEANRTAVLVHKLLNKTRDPAIREELQLFSLQLLHRK
VQFTACGFFPLDFTLLYSIVGAVTTYLIILIQFQLSYENKDSNLGNGTTALPSPVNNASS
>BgerGr73d
MEVYNSAKTIIYISRFFGVAPFSYINELQGNLKYSISWTIYSILWGCLIIISGFSSLFYLEIQSAI
NRICYTSGIMLRVIFFIVVAIVQIWSLACGNKVIAIFKKISEFDKLVHLNIFYKCYILKYLLIQL
LFVGIWSILLIIYGILYIADLSLEAILAVLYSLAVSLSARAIMWVVDCQFNYFTSLLYERFINL
NQEIGKYVSLPSSCTPHEHDTLLLNTNIEKLDISYLRGVHSRLCDISKYINEAYSRQTLLILA
EYFFEITWTSYAFIIMVFNPDSFFKKIVFNDIVYFMLMWILLLVTKICTTLLICTSTAKEANR
TAVLVHKLLNKTRDPAIREELQLFSLQLLHRKVQFTACGFFPLDFTLLYSIVGAVTTYLIILI
QFQLSYENKDSNLGNGTTALPSPVNNASS
>BgerGr73e
MDLYDVMNPIFIISKVIGLAPFILIKNSTNRIAQYSRIWTCYSISFSIFMNSALPIIYYMQTDVI
STEFYFSYSMIVTIAFFVVTDCKQILCLLNTNRVLEIFNELYDFDILLGVKQPHCHFIKLVKY
QLILVVSLFLFHGYFYVISFMVSSVELSCVIYSFFACLCCICMNWTIDSQFSYFIITIFQRFSHL
NIIIQENLEIDDMLISDIQHEQLQLLNSNLDEMSNIIHFRKLYDSLCEISNSLNNVYSGQNLL
SVALTFFQLTFLLYGYITCLLIPNIERFPNNEISHLHLLCWKIMSLTKMVTTLAVCEVTIREA
NRTAVLVHKLLNKTRDPAIREELQLFSLQLLHRKVQFTACGFFPLDFTLLYSIVGAVTTYLI
ILIQFQLSYENKDSNLGNGTTALPSPVNNASS
>BgerGr73f
MDIFDIVNPIFNISKVVGLAPYILIENSSIKGFKYSIIWTFYSILFSVFVNCALPINYYMQSDAT
SNDFYFSFSMLSTVMFYVVTDGMQILCLLNVNRIITIFKELHEFDCIFKARQPHCYLFKLVK
YQLFAIVGSFVLYGYYYIIFFKEYSAVGISSVIFAWVVGICCICMNWTAESQFSYFIIAIYQRFR
YLNNIINDNTENESTLNSHSQHKPNINLDDVSSIVQLRKFHETLCQISTSLNTSYSGQTLLS
VTQTFFQLTLLIYGSLTLIFIPNKYTEDFIDSFARCLHLTIWLVLSIMKTITTLTACEFTKRA

ANRTAVLVHKLLNKTRDPAIREELQLFSLQLLHRKVQFTACGFFPLDFTLLYSIVGAVTTY
LIILIQFQLSYENKDSNLGNGTTALPSPVNNASS
>BgerGr73g
MDIFDIMRPILNISKIFGLTPLIVVENALSKGFEYSRIWACYCILWIFIINASMPSVYYMNAGI
LSNDFYFSYTSISSMYFMIATDAIQIISLVKTKKLIGIFNEFHDFDILIEFRQPDSQLLFKIVKC
QIFYILCLQIVNIFFLYVRTYNIKYSYEELYYFCCWIVILCCICMNWTADSQFSCFLITLYQRF
LYLNKILQRNLEYECKFKCLLRRESVQIFNQSLMDSSSILQIAKLHETLCGISKHLNNMYSS
QSLLLSLSHTLFQLTFSLYGAITSSFVPNGRFPFNAITYCGMSLWILMHVMKMVTMLGMCTI
TKLEANRTAVLVHKLLNKTRDPAIREELQLFSLQLLHRKVQFTACGFFPLDFTLLYSIVGA
VTTYLIILIQFQLSYENKDSNLGNGTTALPSPVNNASS
>BgerGr74a
MEHTMDVYDVARPIIILSRISGLNPITVTAEGKFQYSIWWLIYSVVLTFVIASALPTTVINYC
KLFLNNVYFITSIAMTLVSYGSTIVIQMSCIATCKRTFIFLNNISGFDKSLGQKVTYSNLFTIV
IVETVLVMFSALFTSICSSELIDGLLNISIVSAINCWVMLLISFAIVTVVDSQFIFCLIILYNRFF
ELNKILEQGLELGEDTVRARIDSSCSGVPVHNIPLLKLANKNKASIVWYVRNLHGKLCDIS
NFTNTTFSFQNLMGLIIPLLNCIWCFYSFILFLYLPNLSKNVVKYKSSAYTMPFWIFLAIEKI
LKILVACEFTGNEANRTAVLVHKLMNKTRDREVKEELQIFSLQLLHRKVQFTACGFFPLD
FTLLYSIVGAVTTYLIILIQFPSRCV
>BgerGr74b
MDVYEVARPIIILSRISGISPLTICEGKFRFSISWLIYSIILSLVIITSVPFTLCNFYGLVLSDVYF
MSSMGIALTSYCVIIVTHILCIVTATRTIKFLNRVGEFDRCLGQKQPNSKIFKIVILEPILVEIS
VMLLSIVTSKFLIPQLQYGIFTAIHSWIVMLISLSITTVFDSQFLFFLIIIYQRFLHVNSMLGCN
TGLTENIIESTVDSSCTETRNPQTSLFKYDNEVNTKHILNVRALHDRLCDISNFTNRTYSFQ
ILIGMIVSLLQITWCLYSFILFLFLPNLSGNMVRYKPTAYSMPFIILVYSVKIMKILIGCRFTES
EANRTAVLVHKLMNKTRDREVKEELQIFSLQLLHRKVQFTACGFFPLDFTLLYSIVGAVTT
YLIILIQFPSRCV
>BgerGr74c
MEVYEVARPIIILSRISGMSPLTICEGKFRFSIFWLIYSIILSLVIITSLPSTLYNIYVLLFSDVYF
MSSIGIKFTTYSVIIVTQILCLVTSERTIKFLNRVSEFDRCLGQKQPNSKIFKIVILESILVLISA
MLLAIITYQFLAPRVKLGIFTAIHSWIVMLIILSITTVFDSQFLYFLIIIYQRFLQINKMLGCNK
MLTENLKDSTVDSSRTETHNSTTSLIKCDGEINTMQVLNVRALHDKLCGISSFTNRTYSFQ
ILSSMTVSFLQITWCLYSFILFLFLPNLTGNMVRYKQTAYSMPFFILVSSVKIVKILTGCRFT
ESEANRTAVLVHKLMNKTRDREVKEELQIFSLQLLHRKVQFTACGFFPLDFTLLYSIVGAV
TTYLIILIQFPSRCV
>BgerGr74d
MDVYEVARPIIILSRISGMSPLTICEGKFRFSIFWLIYSIILSIVIISSLPSTLYNYYALLLSDVYF
ISSIGITFTTYFVIIVTQILCLVTTKRTVKFLNRVSEFDRCLGKKQPNSKIFKIVILESILFEISA
MILTIITYQFLTPHLKIGIFTAIHSRIVMLISLSIATVLDSQFLFFLIIIYQRFLHVNNMLGSNT
RFTEDNIGSTVDSSRTEAHNSTTSLIKCDSEVNTTHVLNVRTLHDKLCGISSFTNRTYSFQI
LISMMVSFLQITWGLYGFILFLFLPNLTGNMVRYKQTAYSMPFFILVSSVKIVKILIGCRFTE
SEANRTAVLVHKLMNKTRDREVKEELQIFSLQLLHRKVQFTACGFFPLDFTLLYSIVGAVT
TYLIILIQFPSRCV
>BgerGr75
MAYFVNKFGRATDLFSAVFPLFVVSKLFGTVPFVKDNRFVKFSRGASIYVVFFIVIIIALWF
HFNFPTLLGPINMMTAVGTFETSTITFINITHRLICFINYNKILKLLSRVLEIDEFMIESKQSY
KSIFRISLVVVLVSLFLILVFVIGVRIKAVHSISELILRVIKSIIIRYSFLLIDIQYVSLVFVLYQKF

KYLNKRIEDNILNCIKNSLKSCRLNVILSETRNKNVHDWSVIHSEMCNICEMINSDYWIQN
LVNTTTAVVSVTFSLYFCLCRLIQPEGQIIESTGRSLLTDILTFYWAMLPSITVFLKVLAAQT
TTKEANQTAILVHKLINRTSDPELNEQLNLFSLQLLHRKIEFTAGNIFPINFSLLYSIVGTVT
TYLVILMQFQLSIKDEETKPEVPCLNNSSYARNSV
>BgerGr76aJ
MNVLQIYDIYSVIKPMLYISKLLGMAPFSLKDEIGSRNLEVSNPATVYSILILLILFIRQYWIF
NNLYFEVVDSVFKTVAKIETLTISLTTLGSIIILLYKRHYICIIFQHISQFDSRLCTLTKFNESA
LLLLIGQICFHSVISTALCLSVTIHIGFLNFIKFINSSPLSNMLIILVIDIQVTNLLTLIWQRFKA
INQQFENEINNGIFFINQESSNKINTSFIISNVENKWFKNSFIKFKELKDHHYNLCSICGTVN
NTYGILMLMNVLFKFVDIVFNAYFRILRVLKYSKGQYDHDIWEWTMTALMCWYIVKLAIT
LWSYSSVTYQANRTAILVHKLLNKTKDPAVREELQLFSLQLLHRKVKFTACGFFPLDFTLL
YSIIGAVTTYLVILIQFQLSFANAPAGRNDTTSPTTTYDPGYFNLTSPNTSS
>BgerGr76bJP
MCWVHGGKMAKLVTNLFVYNKIFGNTKFISNIGFFAYMFIILLGFIYVSSIIAIDLLSISSVTP
IILKIEVIIVKIQYHFLAVSPFLVLIQFSSTVILYGQFFTFLNEALTELFSDGDYSVHLSKRESQ
NSFPEYCSLCKKLGKKEFSLETTDIVMPNIGKQLKPVERNCAVQSSSNVDFLFMNRRRFFN
QISKVRPFNSHTLDKLPWILLDQHFSSQLSRITDLHGRLCDEIYKVNIVYSVQLLVGTADSF
VTFTVGLFCILLPFFPNTIHIPPVTLHLFSLSCCLFVNYCLLLHIMLFKGKWSIYEANRTAIL
VHKLLNKTKDPAVREELQLFSLQLLHRKVKFTACGFFPLDFTLLYSIIGAVTTYLVILIQFQL
SFANAPAGRNDTTSPTTTYDPGYFNLTSPNTSS
>BgerGr76cJ
MHTFLVIFKPLLFLSQILGTTPILNSGGDNYTWSLILTVVYSFILTGVIVCITSVMFFFLVKTFF
FGASVVSEIGILFMYVSVTIHYIASLFMSWLHSREIAKIVMNLFVINNFFSNPKFVSNNFFAT
YMISILLAFIYALVLVIIYSYVDVGVTPVTMGAEVVFVKIQFFLLTASPFLIQIQFVSMVIFYD
YFFSLLNEELIELFTGRDSILHLPTKHHHFIKMLPKYAYIAGNVGQLAFSKEVSLEINKNKSL
THRTKQNKHVDNWVAQSSSNSKFNYNREIYSKQISRVQALNSTSSARMAWFRTTSDKNL
IFQLLRISELHSQLCDGITEVNAVYSVQLLVGTASRFLTSTVCTFCILLLFFPNTLSTPLSNIV
LHLFSFVWNIIGLIITVLPCTSCITKANRTAILVHKLLNKTKDPAVREELQLFSLQLLHRKVK
FTACGFFPLDFTLLYSIIGAVTTYLVILIQFQLSFANAPAGRNDTTSPTTTYDPGYFNLTSPN
TSS
>BgerGr76dJ
MSLLIKPLFISKFLGFAPFICSRNSGSFRLSKIYLCSSFIITTFVSVYLLSYIVQVNDFLMDSWA
VTVENWFFLLFSSIFTYWASCFSSFTHLSEMITILNNLNISTKNSSLKRLEVIFLLEIYGGMFI
LSALYYCEWIHGLYEDIFEVIPYFVIELSNYLYEMQFIDIVLLLKYLMSMLEIRIIELANNEPK
INTFLPRYKYAQSEINSKLTYPSEVIKKFSCIYEKLCEAVDQLNRIFSFMILICTAAALIGITNG
LYIASITLIEVIHEDDKVVNPIIPTLAWSFYYFIIFGSIVCTSSSASDKANRTAILVHKLLNKTK
DPAVREELQLFSLQLLHRKVKFTACGFFPLDFTLLYSIIGAVTTYLVILIQFQLSFANAPAGR
NDTTSPTTTYDPGYFNLTSPNTSS
>BgerGr76e
MYTFLVIYKPLYYFSKFFGTLPISNLGDGNVTWSILVSIYSVILTGVCAYFSVGIFNVLIHYLL
FTTKSDIMSELAVFIIFGMAEINYFIAVVMSWINCKNVVKVFASLFVYNKIFGNLHLMPKKY
YCAYMFLILLGFIYLISINVIETNINDAEYNVFLSVHLIFQIIHYWLVVLCPYSILIQFTSIVILY
GCFFSRLNEELVELLNDWDMRVHSSETKQQNLDIVSPHYGSLLNPHTSQELHLETEEMPV
NLLFYSHRHIHSQIENDQTQIRSNFIFNSSKIYLNSPSRVKAFKYTPLNRLPPILLNRTLRFG
LYRVIELHSCLCDEINFVNATYSVQLLTGTSEKFVSFTISIFSVFLTFSPNYHIPPDAMPVFIV

TIIWNIVSLLITVFSCSTCSLKANRTAILVHKLLNKTKDPAVREELQLFSLQLLHRKVKFTA
CGFFPLDFTLLYSIIGAVTTYLVILIQFQLSFANAPAGRNDTTSPTTTYDPGYFNLTSPNTSS
>BgerGr76f
MYTFLVIFKPIYYISIVLGATSVSNSRGEPFTWSFLLIIYKVILCSTVTYTFTEALILFVQNFYV
ANSVIVSETGLLLVYTVLGIHYFITVVMSLLGCKKLAKVITSLFVCNNIFGNPKFISKIFTCTY
MIIILLGFIYLFLISCVSMCNFGVHGTIQTINFIGLFKLLKLSIFILCPYTILIQFISNVMLYCHLF
GCINEELVKLLNSWNSVIYSTEKELQNSIPIHPQCSTLLDCPRASSQFYLDTEKDLSANQYIC
TQIGTDLVAQSSSNSDFIYENRKNCINQSSKVKPFQPPSNATCRFGLSRLMDLHGLLCDEV
NELNAAYSIQLLVGTVERFINFTVSIFFVFIMVYPNIANVYAILLFMIFMIWNMFGLMITVSP
CVSCSEQANRTAILVHKLLNKTKDPAVREELQLFSLQLLHRKVKFTACGFFPLDFTLLYSII
GAVTTYLVILIQFQLSFANAPAGRNDTTSPTTTYDPGYFNLTSPNTSS
>BgerGr76g
MYTILVIFKPVYYLSIILGISPISNSGSEKFRRSFLLTIYSVVLAGGSAYFSIELYLIVAPYFFKN
KASLVSELGLILTYAVSEIHYFMATVMSWVFCRKLMRVVSNLLTYNRIFCNPKFISKKYYCL
YMIIISFGFIYLLLLHVTDIYTHRKKPFNLTLKNIIRTILYWVWLLFPYLILIQFASIVILYGCF
FTYINEELLELIKCWDSTVHPSLVSSQYSSFLKNLFPYLFPPEYSIDTGGMSVSLHSKHYIHG
HVKKHCTAQLSYHSNFFNSNRKKYINLSSGVASVDSVPLDKFSSVLSNRNHRLVLSVIMKL
HGSLCDELNEVNATFSIQLLIGTAERFVTLTLSIFFIFLPFYNKVNVPLHTGTIPIFLLTFAWS
LVNLMITVYSCSSCSSKANRTAILVHKLLNKTKDPAVREELQLFSLQLLHRKVKFTACGFF
PLDFTLLYSIIGAVTTYLVILIQFQLSFANAPAGRNDTTSPTTTYDPGYFNLTSPNTSS
>BgerGr77
MSEQRTFFKPEKLFLAQSLDSTMNPAFCMAKCFGLWHFTYQTHHGILKSKLSIPLTIFHVV
LLLIANSAYAIHFVLWVNDMPKYPIVSFSLVLQSVVSMVCNVIAFYTCCVHNQKIMSIFHLI
NDLEQYLKNSKAVYSGKRRMLIFQIMFVMIGTFISIVVSWQSVGANTEYIFMYMCFFVND
MCNSSLELQYLTFVSLIREHFCAVNIKLDEGRELTNVLSPNKCSKVYNMQRITLRRTFQKK
CTKMNVSKSTVKKAKMSLKNVIMYHDSLCDIVQLVNNVYSIFVLLSITKMFLSVSYTLFITL
SKILKPSYTRSDNVLDIVTFRNEVTPWCTIYTIKFFYLIYMCSITSDEANKAAVFVHKLITIT
NVRTIKEQLQHFSLQLIHRKVQLTASGFFPVDFTLLYSIITAVATYLVILIQFQLSVE
>BgerGr78a
MKVLKLLFLVSKIFGFAPFVIKENAQLKPSRKALICSIILYLAALFTEGNVVVGTLWNFDFLS
IIGLILLDLCGILTYVSSVFLVLKKYKKVILIVDELIMFNDYSHNPCYTVKLLRQIILQICLGFV
FVSILVVSFHFTDLKMYCDNIMTFIRMFGDYIIILQFANLISICRYNLMELNSRFEYLNNLCL
RDKTLIGKCFMRNSISCFPQSMRHLKNKCSSDLTSLIINSVTYHHKICNILQSINEIYSVQILL
STMKIFICVTYNLHFMERIIVYQLKEHIDFFLAALFSCIWCIYQLLLIILHCHSAIEEGRKTGV
LVHKLLSHTKDPIVREELHLFSLQLLHRKVRLTACGFFSLDFTLLYSIVGAVTTYFVILMQF
QLSIYNERKGNETISSMSSKL
>BgerGr78b
MEEKNGILKILFSVSKLFGFAPFLVKKNGQLKPSKRALIYTRILCLTVLLAEGNVFVEKLWN
SDPLSITGFMLLSSSSILTHFSSIFLVFKQNKNLILIVNQLITLYDHSNKSNCFCKLQRNIILQF
LLGVITVALIIVCMNLINLKTYENIVTFMRMFGDYIIILQFGSLISICRCQLKKINSRFEHLVEL
YSSDVSYKRKWYMANSIGYFPQSKTRSSDRFNSYLKNLVLENVIDHNKISDVLQSINEVYS
VQILLNTMKSFIFITFTLYFIETLILLYDKLKEGLHLLFIGILFCFWSVCQLLLITFPCQFIVEE
GRKTGVLVHKLLSHTKDPIVREELHLFSLQLLHRKVRLTACGFFSLDFTLLYSIVGAVTTYF
VILMQFQLSIYNERKGNETISSMSSKL
>BgerGr79

MQRYKEATRIVLQLSKFVGYFPFTYSKNNTFEIARQVWILALCMNCVLLVVYFIGLIITLKH
FHNMEERGVFQIVMEIHRAVSILAYIVSANSIFFISPKLIKTFNNLECLDLEFKYINVSTWKD
FHMYSRSKIILLIFIIINLMFFHPNGMRSTIQYIVVLPLFILKNLSLILILLLFADLIILLNYYFD
VINGILIDIRQKPFSLKVFNSCNMMYLMDSQNPTTTYNIHVKNYFAINPIANLHCRLCDVF
EDINSIFSLAILSNLVDILSLISSSVFSGIRILVNNQEYTIIDVYMLIIYLFWGIIKLIILLYPCKLC
VGKVKYSSRLITKLAYKIRNPVVEEELQSFSLQLLNRKIEFTICGMFPLDFTLLYSMAGAVTI
YIVIFIQFELFWEQSLKREIFTMFPPTLTTHNSTDLQLSY
>BgerGr80
MDEYFKEENVPILIKLVHYSSKYLIILPLRIVRSKEGYRRIYCEISILKFIISIIFHISLNVYKFKA
VHDTVMHLTFGNILHFIGHISISVNVLCSVISIFIFTINSKDIYNLLFNVYTRKIKEDISTMNR
VLLRRHMMIVLLTYLLIIACLINALMHFQVKISIFVGYFIWCIVYTSTFTVDLVFIILVDYFRI
HIEHLNSLICGMKQSTVLRHNYSHRKKQKNNKRKNKLKIKLNFDQYNLRNIMTSYNTLCN
ITDEVNRIFSPLLLTESARIFVIITFCAFAVLTSVQRSGLNLNEIFNFVTWAVVETCQLSSVI
WACTSAMKQANRTPIFVHKLINSTGDPTVREQLEQFSLQLLHRKVQFTACGFFPMDFTLL
YSIIGAVSTYFIILIQFHESIADKE
>BgerGr81a
MDFRSGSVFKHAVFMSQILGIFPFKINIDKVKGLHHVDFSSNLIIYSVVIISYVFILKVYSIICW
IASDQETVFKISGSIEAGCIIYSYLTGLFTSIRNFGNMQTTISELSFWKEFNNEYETTFKRNF
VIRVSLGTFAILIYTLAHWKGAPFIFEDLNTSISFFLIYITVYAIEVQFITLVSILYEFFYLINHE
LVYITNRKREQQFKDLEFLEESVSMCPNFKVNLNAKISLRALADLHRHLSDLADKLNSYYS
FHNLVDIAVLYLGLASALYNIFWCTLIFRTLYAQLCGSVLYNFVDICLTVDSLVPGNTIANR
TAVLIHKLLKRTKDDLDREELQLFSLQLLHRKVEFTACGFFPLDFTLLYAIVGAVTTYLIILV
QFELSHYQGNNYNSTDQPL
>BgerGr81b
MNIGPQSTLGLTVFMSQVLGIFPFKIIINRKSGLHHVHCSSNLIIYSVLIVFYVCIMKLYCIIC
WIVSDQETVFKISGSIEAGCIICSHLTGLYTSIRNFRKMHTAISQLPFWKEFNIQYEEIFKKN
TIIRMSFGVLAFSAHTLALWRGSPFSFKGSNILISFFLIYITLYAFELQFITLVSILKQYFYLIN
QELMCFTNRKREQQFRETVIFGEYVFMNSRLRVNANIRLTLTGLADLHSRLCDLTNKLNN
NYSFQNLVDIALLYFGLASALYNIFWWTKIIKLLTVRNMFPIVGFFCSTMKLVQILSNTSNI
RNEANRTAVLIHKLLKRTKDDLDREELQLFSLQLLHRKVEFTACGFFPLDFTLLYAIVGAV
TTYLIILVQFELSHYQGNNYNSTDQPL
>BgerGr82aFP
MDAFSNARIIIYFCRVFSVAPLKIKNESANSKFVHCKYWTCYSIFTILFVLSSFPIILNLQIRN
KQDGSSVYQSAFTLMTCTFFITVLTLQMFGFLIRNRLIKTLNELNELDIFLPSPTKKTFYYK
VIYYQIYFLLLLNTVFAYVTLQYVSKHFNGGFIIAIHFSFIFLFGAGVLWTADSLFECFVFMV
FQRFQKLNEILELAKQIAINSSNEETKPGDNQREHILRITPNTIASKNSVISIIRALNCLHVRL
CEIAEFINRTHSFQNLLSTTVSFVLIVWCCYVFAMQTLIPHSELSMPQMKRSASYSLPLWIL
YLANRTADLVHKLINDTKDPEIREELQLFSLQLLHRKINFTACGFFPLDFTLLYSIVGAVTT
YLIILIQFQLSYDSKGYPLPSAMMAAVHNISSLTTINDTVT
>BgerGr82bF
MDVFNNARTMIHFGRIFGVGPYNVITEFPNIKFERSKFWTCYNYLTLFFLLSSFLIILTTQV
RIKEGTVYHLTFTLGLCTLFIAVLFLQVCGFCIGNRLSKILNNLRDFDLLFSTPPRRTFYYAL
IFFQLYFILICNVVFCYISVIYISKHFYVSLVTAVHFWIVMFFSTGVIWTADSIFECFIVMVYQ
RFKKLNEIFVLVVQIQENLTDNENKHGNCESENNFRLLMNEVIKDTSTFSIIRILSCLQDRV
CDIAEFINKTYTFQNLLSTIISFVHIIWSSYIFAMQTMIPHSEPTMPQLQKFAFTLPIWAFSF

VAKLLTMLQICNTVRTQANRTADLVHKLINDTKDPEIREELQLFSLQLLHRKINFTACGFF
PLDFTLLYSIVGAVTTYLIILIQFQLSYDSKGYPLPSAMMAAVHNISSLTTINDTVT
>BgerGr82cF
MDDYNSVKPIIYLTKIFCLAPYKLVNGPVCEELRHSKLGVCCVVFLDCFMLSCIPHIWKVQD
HIKLFAVYYSSYSILTCGIIIIIVSLQTFYLTIGSKISNSIQSFVEINKLFSRPPKCTHYYKLLYSQ
IVLILIINSALIYFMVQYLTNYFSGGILVGLLSWLSFLYSCLFIWSCNAVFNCFVIMHYQRFQ
QINEMFIPKSRNEIKLVYTKTDNVQYFPMTILFKVGNKSLIKRGIYLHDQLCDIAEYVNSTH
SFQILLTIGLSVIQVTWLSYSFAMHTMLIPDSVFNTEYMKRISFCLPLWTLIAVYRIWTTLF
VCVSTSNDANRTADLVHKLINDTKDPEIREELQLFSLQLLHRKINFTACGFFPLDFTLLYSI
VGAVTTYLIILIQFQLSYDSKGYPLPSAMMAAVHNISSLTTINDTVT
>BgerGr82dF
MDVYEVVKPMVYVCRIFCSAPCKMVENSKRMQYSKFWIGYTLLVTLVTICCFPNIRQAQE
YFKANELFYSSCTVILYVFFSTVTVLQVVCFFKWNRVIIVLERLNEYDKASLIPLWNTPYFK
LVFSQIFCVVFFNLVFSFPSMSFWTNYFSGGLITAVQFWFVLVFSCCSLWAMDSLFDYFLI
MLYQRFQQINKIFVVTFGNKSSILINIKPVTFIKNRDNLQNSSLGLQHASVVTFLRNLQDQL
CDIANFVNGTHCLQMLLSISLSFFQVTWFSYLFFISILNSHLMETSMKIHVASLLLWILLFIV
KTSTTLLLCVTTTNEANRTADLVHKLINDTKDPEIREELQLFSLQLLHRKINFTACGFFPL
DFTLLYSIVGAVTTYLIILIQFQLSYDSKGYPLPSAMMAAVHNISSLTTINDTVT
>BgerGr82eFP
MNVLNIAKPYIYVSRILGTAPFKIVEGSKIERINLSKYWICYSVFIILSTTFSFPNIWDVQEIY
KMELFYTSSTIMLYVFFTTATILQVICFSRGRRVIIALERLTQYDSVLVTPPKETVYFKIVNF
ZCVFIIVSNTLLSFNAINFYAHYFSGGFVTGIQFWFIVVTRGFSLWTMDSIFDIFVIIIYQRFQ
QINEIFVVPTENNNSHQVGIKQLHSTHEQGENIEKYSLNLLQGKTKVISSIRILRKLQEQLCD
IAIFVNETHCLQILFSISLTFFQITWFTFAFITFFLNPYSHLVDQSAKIHIYSAAFWISLCILKI
LTTLRVCAITKAEANRTADLVHKLINDTKDPEIREELQLFSLQLLHRKINFTACGFFPLDFT
LLYSIVGAVTTYLIILIQFQLSYDSKGYPLPSAMMAAVHNISSLTTINDTVT
>BgerGr83
MSMDIYKVVRPVFILSRVSGINPFTFTEEGTFQFSKCWFIYSILVSSIIFSSVLFSLFCSHETFV
NTFFFISGKAITYTTSCIDISTQILCFFCCKRILIFINQISKFDSLLGLKQPNSNLFTIVILEESFF
IISSVLSSIVTQYNTPVLKLGVSSAMHFYAETLLSMSTAAAADSQFLYSLIILYQRFLCLNKM
LEEYDCNVKMYYSKPNSQKHYIPLLKVANKNDANVISYITKLHNNLCDISNFTNKTYSFQI
LIGMILSFLHITWGLYSFILFIFIPDVEGGTILYKPTGYLMPFWILKGSLKTMKILFACRFTEK
EANRTVMLVHKILNKTRDKKVKEELELFSHQLLHRKVQFTACGLFPLDFRLLYSIVGAVTT
YLIILIQFQLS
>BgerGr84aJ
MNCEIILKPINNFLEVLGVSPYLYSNIDGYKFKLKLIYNVLILNTILFFYIKDITNDYSVYTSD
TKLTTTAICICFEKLITIAQYLITIILDSIFLTRKHSFIMKSLTKNNLLNEIQILKSKKLIIIQLW
VYTLSCLSLYSIDVNMYSSEIISFCIIASFGILDLISSLREFLFITLVKILEQNFYIFNGNLPNFII
FRTAIYLNSDVNTKIISKYYHILNQKTSMSGDQNQRIEIVYNKLYFIAQTVNAAFSTELFIHV
IRYIIFTHSISYIIITHVFFAEYFGTFKEIYWKIVVTMWMIYYQFRFLWLTYSCWSSSREANK
TSAVVHKILNKTRNPEVREELELFSLELLHRKVQYTAGGFFPLDFTLFYMIVGAVTVYLVIII
QFQQIFMNQTINGDNSTTSSSQVLG
>BgerGr84bJ
MNCHMVLKPIDDFLTVLGLSPYLFFNSNYKINLKVIYNLSILFVTLFIQIKDMTNEHLVYAV
GFKLRTAAICVYIENFINFSQYIIILVITNFVHNSRVGLHSFIVRSLSKNSLLNNFQLIESRKLV
KIHLWIYTVSLVLMYVSDLIMFSSEFFSFCFIISYGFLDLVSSVRECLFITLIKMLKQNYYNFN

TNLPNIVLSRTGNHHTGYCEFFSSYINVLNQNKLVFGDKNLGIGMIYNQLCLIAESVNSAFF
IELFISVIRYFIITISHFYILFTHFFFYDYFTVTNNANWTKILVLWMIYYQSRYLWLIYSCWSS
SKEANKTSAVVHKILNKTRNPEVREELELFSLELLHRKVQYTAGGFFPLDFTLFYMIVGAV
TVYLVIIIQFQQIFMNQTINGDNSTTSSSQVLG
>BgerGr84c
MNCHMVLKPIDDFLTVLGLSPYLFFNSNYKINLKVIYNLSILFVTLFIQIKDMTNEHLVYAV
GFKLRTAAICVYIENFINFSQYIIILVITNFVHNSRVGLHSFIVRSLSKNSLLNNFQLIESRKLV
KIHLWIYTVSLVLMYVSDLIMFSSEFFSFCFIISYGFLDLVSSVRECLFITLIKMLKQNYYNFN
TNLPNIVLSRTGNHRTGYCEFFSSYINVLNQNKLVFGDKNLGIGMIYNQLCLIAESVNSAFF
MELFISVIRYFIITISHFYILFTHFFFYDYFTVTNNANWTKILVLWMIYYQSRYLWLIYSCWS
SSKEANKTSAVVHKILNKTRNPEVREELELFSLELLHRKVQYTAGGFFPLDFTLFYMIVGA
VTVYLVIIIQFQQIFMNQTINGDNSTTSSSQVLG
>BgerGr84dP
MNCLIVLKAINDFLTVLGMSICXNYKIKLQSVYNIVIFVVILSIQLKNITKIFLCICQKLYXYW
YLISVTSNVMFRHKVYTFILLRLLKNHILNSZQLLKSRKLLIIQLWTHTLSISFFYTTEVISYH
SELFSLCFILSSATLDLISLLRECLFITLIKILEENYYSLNTKIGLFINGIVSATVHSEVRFIKKY
CDNFNQQQKLRIKKLGKIHSELYFIAENVNSIFSAELLMYMIRFFIYILSNVYFVMTHIFFDE
HYSTHKGAIWTTIMTLWTMYYQLRLLWLIYSCWSSNKEANKTSAVVHKILNKTRNPEVR
EELELFSLELLHRKVQYTAGGFFPLDFTLFYMIVGAVTVYLVIIIQFQQIFMNQTINGDNST
TSSSQVLG
>BgerGr84e
MKNYHEILNPINNFLAVFGMSPYQFPNCNTNIRKVKLRSIYNILIFAILLFIQIKTITDNYYGS
YIIDNQLSTVIISCYLQSISNICAYLISEILNIIFHQKVHSTILEQLSKNDLLNSEQLSKSRKLVI
TQLWAYNLFFIFLYTSEAIWNHTGLFSVFYVISSAILDLVSSLKECLFINLVKILQGNFYSLN
TNIFLVQYKESNGTGHSFNKVRFFPIHYQELKYNQIKTKDHTVRIKKLGKIYNELCIMAETI
NSVFAVELLMYTTRFFVYILSNVYFVITHIFFDDLYSTHKDMTWGVVMALWAVYYQCRCL
WLIHSCWSSSKEANKTSAVVHKILNKTRNPEVREELELFSLELLHRKVQYTAGGFFPLDFT
LFYMIVGAVTVYLVIIIQFQQIFMNQTINGDNSTTSSSQVLG
>BgerGr84f
MKNYLLILSPINNFLAVLGMSPYKFHNCNTNIYKVKLQTIYNILIFAILLFIQIKTLTENYYGT
YIINNHLNTVVISCYLQSISNICAYLISEILNIIFHHKVHFTVLERLSKYDLLNSEQLSKSRKLV
IIQLWAYTLSFILLYTSEAISNHRGFFSLCFIMSSAILDLVSSLKECLFINLVKILQGNYYSLNT
NILLVQYTESTATGHNFNEMRFFPIHYLKWKYNQLKTKDQRMRIKKLGKIYNELCIIAETI
NSVFAVELLMYTTRFFIYILSNIYFVMTHIFFDSQYSTRKNVLWGGVMALWAIYYQCRCL
WLIHSCWSSSKEANKTSAVVHKILNKTRNPEVREELELFSLELLHRKVQYTAGGFFPLDFT
LFYMIVGAVTVYLVIIIQFQQIFMNQTINGDNSTTSSSQVLG
>BgerGr84g
MNCHMILKPIEDFLTILGISSYLLLHDNPVGYKMKLHVIYNIGIFIVILFIHIKDITSDYPLYAS
GFELGTVIICVYIQNIIHFGQYLIIAISNFVWQSKVHSFIVKSLSKNSILTTFQLVESRKLVIIHL
CIYIISVLLLYMIDLVVFSSELFSFCFVVSYGILDLIISLRECLFITLVKILSQNYYSFNANLPNIII
SRTTNHVNDYCDTRHFSSYIHILKLNKPTPENKTFRAGTIYSQLCFIAELVNSAFSIELFISV
TRYFIFSISHFYFLFTHIFFNDYFTTMDDVKWTIISTFWMIYYQFRFLWMIYSCWSSSMEA
NKTSAVVHKILNKTRNPEVREELELFSLELLHRKVQYTAGGFFPLDFTLFYMIVGAVTVYL
VIIIQFQQIFMNQTINGDNSTTSSSQVLG
>BgerGr84hP

MKNYHMILSSINNFLAVFGMSPYQFPNCNTNIRKVKLWSIYNIIIFVILLFIQIKTXNRQLL
WLIYYNHLSTVIISCYMQSISNICAYLISEILNIIFHHKVHFTILGRLSKNDFLNSEQLSKSRKL
VKIQLWAZTLSFILLYTSEAIZNHRGFFSICCIMSSAILDLVSSLKECLFINLVKILXMNYYSL
NANIFFVQYTDSTATGHNLNEVSFYAIDYQKLKDSLLKTKDHTVRIKKLGKIYNELCINAKS
INSIFAVELLMYTARFFIYIFQTFTLXFDGQYSTCKNVLWVCDMALWAIYYQRKFLWLIHS
CWSSSKEANKTSAVVHKILNKTRNPEVREELELFSLELLHRKVQYTAGGFFPLDFTLFYMI
VGAVTVYLVIIIQFQQIFMNQTINGDNSTTSSSQVLG
>BgerGr84iP
MNCHMILKPIEDFLTILGISSYRLLHDNPVGYKMKLHVIYNIGIFIVILYIHIKDTTNDYPVYA
SGFELGTAIILVYIQNIIHFGQYLIIAISNFVWQSKVHSFIVKNLSKNSILTMFQLVESRKLVII
HLCIYIISVLLMYMIDLVVFSSELFSFCFVVSYGILDLISSLRECLFITLVKILSZNYYSFNANLP
NIIISRTTNHVNDYCDTRHFSSYIHILKLNKPTPENKTFRAGTIYSQLCFIAESVNSAFSIELF
ISVMRYFIFSILHFYFLFTHIFFNDYFTTMNDVKWTIISTFWMIYYQFRFLZMIYSCWLSSL
EANKTSAVVHKILNKTRNPEVREELELFSLELLHRKVQYTAGGFFPLDFTLFYMIVGAVTV
YLVIIIQFQQIFMNQTINGDNSTTSSSQVLG
>BgerGr84j
MVKCHLILLVKFINNFLIVLGMSPCIFTNKNSGHKIKLYSIYSISILALILYFQIKLIIENYTTYT
SDTQLNTVVITFYVQTLANIVPHIISVILNIRLIYHQMVTSILERLSKNDFLSSEQFYKSRKL
MLIQLWTYILTLLLLYVSDFVQYGSDFLSFCFIMTTGIYDLISSLRESLIITLVKILEEHYYSFN
LKICNFLNSTSNLNNYCEMRFIKKYYHSLKEEQLMNENQTFRIKQMSMIYNELCLISETVN
SVFSAELLMYVTRFFIFILSNILFVFTRILFAEYYSASNGAFWVFIMALWTIHYQIRLLWLVY
SCWSLRKEANKTSAVVHKILNKTRNPEVREELELFSLELLHRKVQYTAGGFFPLDFTLFY
MIVGAVTVYLVIIIQFQQIFMNQTINGDNSTTSSSQVLG
>BgerGr85
MKTSQIFNFSKSLEPIFISSNIFGVSPIIINEKSRYNCSQIQLWNVISLCAIFYLMITEDFWWP
FAISATICGTSTNLNTFVQFSAYFLIVFSGFYNQKKIIGIRIELLEVQSELNCDSSKKLYFNFTL
QIISFAILHFISATIWYFKSQGSFGLVTLTCWSFISFGFCLSSLQFTCFVAILKEFFRSVNLQCC
NLKDNLPGRYFKLSSNTSMKREVISRFHKLENIHNFLINVAENINSLYNLIIISSMAKSFMTI
VTVSYAVIGEALFTEYSFICADGALISLCIDISNEALLVLWIIINSASTEKEATRTANIMQKMC
KRVQDPLIREEFQLFILKSQHQNFKFTAFGLYPLDFTLLYSFMGSTTTYIIFLIQLQIGSGEN
DSQNTTCTTELLNYEYSVQVN
>BgerGr86
MTIFRLVFFFQKPLKEKMEDSTKKEYLYMPNVMRFLQCMSEILILTPLKITRNTFGCMICEI
SKTKLITSLLLHSTINVLNLITLSTWLVNMRNTNVLRVSGAMSVFSDFLCCTVATLTRIKYS
KKVYHLIFNVLFLEINLNILPMFKVVNRKYAVILLSSFLLLFSCCTCVLAMSRPNSVTLISFS
TWSFIYISTLFVDMMFIILVDYFKIHMKYLNYTIKVLYESIRSVRMKNIKKVNYVKQNLNM
HLNVSKVKILVSRHTFLCNIAEEVNDMFSPFLLTKALKMLVIVTHCSFTLSTIRKGTRSIKD
KINVISFAGLEAIALSSLLWICHSSVKEVNETPKLIHKLLNISEDPAVQEELQQFSLQLLHRK
IRFTACGFFPMDFTLLYSIFGAIATFFVILMQFEGNFHY
>BgerGr87
MEELLAAIKFFYYFSKTLGLISFIYKHENNVLNLRLSKPSIVYCLTLPIIIIFANIYVIKGQYVQI
MELKKMNPVTFVVLIVVYTFNFTTHILSALILHKHRGSLNFILRTLLLLDKSLKQGKIFLWI
SLQLSLEFFHVFLTCLMYIIISNDFAGLLVYTVSFLSIYIIQLQFTNLVILLKKHFSSINEKLKT
LSESDLSGIPLKLSLNSLETNYEILCKLADEINSVYSFPILLGMTYFIFMDMHSLYFCALKIIQ
LYDGPSYFHLSVLLNSTYMLCFLAWACNSTKNANKTGEYVQKLMMKTTEPAATEELQLF

SLHLGHRHVQFTACGFFPMDYTLLYSIAGAMTSYLVILIQFHQTFIEKLGNGSSTLLTRLNC
TCMQ
>BgerGr88I
MIPKIIIVLHYILNILILSPLKMYMNRNGRWECEKSSKKFIICLIFHTTMNAINTSSSYIWMT
RVENSGMVHVTATINLFSNFICNIITTCTCLIHSKTIYNLIFDVFLPNICLKMLSESRMIDYK
YISILLTSFLLLITCSINALYMFKRTIFLIIGFVTWVIDYMSCLVVYLIFIVLIDYCATYIKILNS
DLLNMRNSIKCAIFEKNVMIKEEIVTPQKIYKSNTEIIIKIEDVIKSYNSIFDTTEKLNKIFSPL
LLAKSVELFVVITYCAFFALLFGHGTEMNINEIYNFVSWAILEILSLIIILYVCTTTTREANHT
PKLVHMLLKVIVDPIAEQKITGAVTTYLIILVQLHKTYEITL
>BgerGr89
MDAFNALKPLYIISKFLGLIPGHFIRYKGEIISFKLSTPFFIYSLFVQIFLRSYQIFTYVNWYLR
NYTVGSEFVELTSMIIKSTCNLCIGISSIFITILNSHKIFKLISALNYCCESLQFRHSTYNNIRRF
VLIHIFAWISTVITLCILTASFLPSKPMDIVTFINAYHCLLCTTLVEVNLCMILYIINNCFEAIN
SILREGTFSSKKTSIRTQGLKKQLQISTENFTRKFKERIKPSNKLNSVENSLNLSGDTRFISIY
RKMMDCHYLLLKIIENLTEIFSPYLLSFALLSLFSETYYLHNLMTQLIFMNPNNFRATINSCI
WSMLYATKMVYVIHHCSSTVKQANKTRRVVNEILNKTTKSILKSELQLFSLQLLHQHTEF
TAYRFFSIDFKLLYSIVGAVTTYLVILLQFQMPS
>BgerGr90
MQLFKSFDIFSELIPLLSIMRWHGLVAISFEGEVGFRKLVTSKFLYVYCILFFLLKILIQVLWF
QSRTSIYITPHLCNFSSGVFIVRGILSSCIMVLTIIFSKTISESFKALLEFDSAKSQIRSSYKKRF
TNTFKFICIYSIFLILTVSLHIFAPIGNSFEKFITFINMYFDSIILVSADLFFMQSVTIAKSRFQV
INSELEKIDWLRVSNKVWLHKNNSSISVLSYKNSNFIEILQIKNLIYFHSLICDVCDRINYFSL
RILITVTQKFISITMFVYFIGIELSYDINNYLVTIYPLCTLLCIDIFPLLHLVKCSCNTCHEGNQ
AAVLVHKLINRMNDPEIRHELHLFSMQLFHRKVQFTACGFFALDYTLLYSIIGAVTTYLVIL
MQFQINIK
>BgerGr91
MSAALKMDFYYVMEPLFNFSKFLGLTPFVFKVSLGSRKFIISKIAMTYSIMMLIIPFLSQVID
LCFTLYNDNMEGVFLASKIIESFTYLFVTFTIPLMYLCKNQSLCKLLNSLLEIGNILCRFRAK
NLKMFKKYKIFALCELFTIFMVLFPNIMFYFLFEKNGIVYFIKRFLLFFNELVNFINDIHSIN
VVILLKSAFYLLNDMLNNFIDKFGCTQFVKRNDREGSILKTVSHQHLQIQNYKHLRIIHSHL
HDICDLANSTFGIQILASILFKFIFIILNLYFDALFVLAQDRAGYSMISWMLDFGPAMLRSVV
YIILFPWAVREISTEAARTSILVHKLLGKTRHAGVKEEFQLFSFQLLHRKVEFTAFGFFPLD
FTILYLIVGAVTTYLVILVQFQLSSTTHVFNITSPDSANVSLTM
>BgerGr92C
MSPKEPMNLLSALNPYYYASKVFGLFPALGSLKDNTKKSNFGYIYTFTLYTALLFLFTYMIF
QMKYTAAPGLSAVDVVTFAFRTWSTTIIVVTFICKSFWREGLIMSLLKNFQKIEMQFSVSLI
KIPHRMVYISSIFYIFVGVVLIFVANAMFTFLIFSWKRMILWNLALSLTYVTSKLILYFCLSE
FTLCAFILRNYFKALNEVVNSFFPKEMERKSIDIQHIDFNNMWVEVPLIFVKSHSIHVSIKLI
EVIRHLHDSLSDIAFQLNATFSLSNFICLAAVFCDILISLYTVLIIYIKQGSLTNSWFMIAFLY
WTILEFIQLIITAGTCTSAIEQASYMGTLIHRLLRNTEDRNIRSQLRTFSLQVLQRKIHFSICG
LFDMDLTLITT
>BgerGr93
MKRAKVQSMNMSYPAAIKYSLLLSKVLGLAPTITITRTNINLSLFGYIFIALLYGAFIYIYIDT
SKKPPANKYFKSPTNRMSLTVRVYMDLIVFTVIIVSHSFNQKRLYEFIKDVICIDTVLKKIGY
SVSYSRIRCLSVTQVWVSLALSWGVDFIVFSILFSDDSLKDFLDLRRVVRYIIPRHIIIQSMSE
FLLFLFTIYKRFEIVNHNISKLSEKETTNDIIQYTLMQKSVKKLRRTDGTKKFKLEILQSLHD

ELSDLSAILNSLYSLPLLVTMITVFVDCTVNLYITIIVFMHGIDNLFVTMYVIRNMYRCFLAV
FIAWIVAAGCDATSNAANDTETIIHKVISEAKTVSQHVYEELELFSMQLLHRKLEFTVCGFF
RLKFSVLSTMAGSIATYLIILIQFYTSGRTNCSNKIT
>BgerGr94
MGTMEEAKDIYSSVYPLFWFGRLFGLVPISLNTTSIFGTSIKSILYSVLFLSANVASFVVYNIT
TGESRFSLGSRLTKIVFEIREYVDTTITVIFILSSCLNHRKILSFLSSVDYVDRALVNIGVSVPF
NLPIRLFKIQIILTVLVMVAPCVSLCCYLAFANGNINMVYGILIYAFPKVISCWMQSIFFDCM
LAHRQRFQIINTQISDLKTDNPSEDLMKDCSIFGPLIRETLTPTVQDPPEKSTLRSRTTTSAI
APVTPMIESQKNEDLPFSLSTYRNIPLNMLEVDMSRTVPQLNSRNKIIQVGEADEFAGMSR
VRILGHLHDTLCDSALVLNSSFSFQNLLCSAVCLIDMTVTFYCCFVVLTTIQERDALHVQVV
FYALYWTFLSMLQTIAVVASCAFTAQEANRAGVYVHNILRETSEPDTKKELKEFSLQLLHR
KLQFTACDFFPLDFTLLHSMVSAVTTYLVIVIQFHLASKEEKK
>BgerGr95
MASRNVFFVDIAMKPIVIISKILGIVPLKCIPKYVSSNGCHNSNRLKSYKSRRLSIINLCYSLLI
FSAVSYLYYISIHWKLNNIYKKIKFMVLLIDIVSSTLTFISISVIMIQHFFPHRRNWEDILQIL
HNIDVSINNKFNRRSYRRSLICVTLQLFVTIVVYGSLFAFDYSMLVEITAPILLYAKYFEHTL
RIVQNILFVNLNIIIESKFLILNTQLITIFNFNSEIDLNEFIHEHSEARLLNKNIRGQIKVHRGE
LHKNNKDVQTKAREVWKVEKPVSEINVKKLRIDYIQINSIARIVIEYYELYLLFEFLSIGSDT
VMHMFIAIVFPDQLPRCVFWIIVNYLALFIITSSYRSIRISNNCTTNLVNKLKLVSGLKSNIL
RQLERFSRQLFNTKLRFTACDFFLLDKEILGEIVKLTILYMVILLQNM
>BgerGr96
MSNFLLAIRPLFYISRFLGLLPIYYKKESDIHKSSYISAFGMAHSLTIMTILLCCFIPFVSDRIS
YVYESSTFTYIIFDFLSWVLSFTGSTVAFFQASIYYRHELKEILNNILLIDSVLLQSESLTFYK
RTFYIVLAEILMSFSYLSILIGFDSITIQRHSEWYYCLIKYYVIIVGNVIFLQYVDICMVIGHRF
HVLNKELNRIYNVLSNHNLPSNININTQSGIYGDSLFLEFLRDLPNFSLSSYHKGSRSVRYF
GNIWLDHKNENLLSIRALRKIHLQLQDLVTNFNNCFGLQIMLILLSSSAKITVNIHIAIILLLE
DLASSLDLDAKAYIFVNLAWSFPGLIKLFGITSCCEISKNVALRTSQIIHKILLLPNLDPVVFD
EMKLFSDQLLHCDIQFSASGFFNVDFSFLCSTFGSIATFLVVLTQFWETYQHEHRSLNGLV
SHMSPED
>BgerGr97
MAIRHTVDLFSAITPLHYLSKAVGLSPFIIGRKILDDTVITEFENSAVGAIYSLGVLVISIIIFPF
IIQWKRQVLYTRVIDDGLTFIAVDFIGTTLQFLTAVICIVEVSFRKSTKIKRIISNMKQIDNM
LKVNLLQRHYDRIKCFTAVQVSTCLAFLFILYYYDSVLWNENISFVTSISRYQTYIIYLVMV
MQFVNFTYYLKRRFQMLNIDIINIFGFRDEIELDEYLQHLSRNLLLDAPYVGFKKELLLNNL
KNHNIINEGSVKDSTSCNIRLLREVNFKLHHMATSLNSIYQIPLLFLIMTIVLKVAMKIHLEI
VTIFFENVTELPLALFLTIQFSWTTLYFLKLFLITTVCQRLQRQAFHTIILLHKLATLTPLHK
DIVLEVDMFIQQVMHNKLQFSPCGFFQLDHSFLSKCVEVLVAYVVILLQFTQSDESTN
>BgerGr98
MNSGYQEQLKDILFAISPIYYFSKVFGLAPYCWPPATGFKKMFDIAYTLFIFSGVLAWFIYS
MIWNIRNVYAGSSYTIIVPHVFKTILSFTSVLQALLLCTTLHRRKYEFILKKVCSVDDVILKY
PMERTSVYKKMKIFLIVEIFIVMLIYMVLFIYDRAIWTGSIGEPHYVLGYCVHVIGTTMNLQ
FIDFVLLVFHRYKTANRQLLNLFDIPDNEMEWSVLPERHSKSRHNITLTKTGLYTGEFTR
NTRSPMNDAVKTKSRPEEIFQNVKIENHIRTLSKVHSTLYDIADLVNSCYGLPMLVELTFIF
GALIHSLYNSMIETFNLEDLHTFGGPTESTILFILWAVVRFSKVLAVTTACQKASNEANRT
PILVQKLLLDRESHGLRLFSQQLLHYRLQFTACGFFNLDSTLIHSLVGSLTVYLVILLQFQFV
AS

>BgerGr99
MANWLNANNNILMAASPLYYISKIIGIAPISWPYKSKRKPYATIAYSAIVFIAVLAWFIYAIV
WNVNYEYKKITYTIIIPHIFKMVSSFTTVLAILLLCATVNRRKYEAILVKISAVDRILLNNTTI
MNLVYKRIKISLVIQLVVVIFVYFVLFYCDRAVWTGDIGDPHHLVEYCVHLIGTVMDMQFV
NLVLLIKQRYVMLNKQLLSLYRVPKDELEWSILPLPEFLNVRKTKKILADIEKGITENKCQF
RNVKDQKCVKIFAEFESEEVKILKLQKIHCSLFDIASLVNSCYGIPILFELTFIFGALINSLYSA
LLTALQLDNLQTYSGPGLAVTLFLSWATVRLLKMICITTVCQLASGEANRTGILVNKLLLG
GDCNCLRQFSHQLLHYKLRFTACGFFTLDSTLLYSLAGAVTTYLVILVQFQFIASAASSQIG
NSC
>BgerGr100
MSKTLLFTFESDDIYSSISPISYLSKIIGMAPINCSNGQPTRAATAYSLTVFITIFVWFMYCLL
FNIWNVYPTSTYTVIVPHVFKLMLSFTTVLTSILLLSTVHRCNFNSILKRITSIDEILLKPRSL
SRDVYKKTKIFLTVQLLTVIPIYLTLSCFDRFIWTGGVGDLHQIFEYIVHIIGTILEMQFMDF
ALLLKHRYACLNRELLSLHGISEDETDWAILPVPASLTLDRLMKPSKEYQIKTVHSITREGF
AEKQFLKGVHSEYKNETDVTLKKVSTEDSNQNEIILKLRKVHCTLYDAAALINCSYGIPILL
ELTFIFGALIQNLYNALLMSFKLDNLHHTGSVGEAVAVFLTWACVRVMKMICMTTVCQIA
SNEANHTGVLVQKLLLTRESESLRRFSEQLLNYKLQFNAGGFCTLDSTLLHSLAAAVTTYL
VILLQFQFVASPSSC
>BgerGr101
MNYIGYKIFSRFKNFNMGNNYIAQTDIFSAITPLLYVSKILGLAPFNSSTGAPTLKGVIYSCIF
FVFILASFVYSLFWNVWYYYHQTSYTVVIPQIFKLCCSFTRVLVTLILNVTANKNKFRAILS
RISEVDQVLMKSKQVKSSVYNRARNFLTVQLLVMLPLFIALFYFDRMVWTGVIGEPHHLV
EYLLDVIGTVMDLQFLNFVLVLKQRYCLLNKELLLLHSVMDETEWTIISKTDVRPKQNIRK
VYPIPNNFWLDSKQTSEKSFSSEKHFISTTKTKHTYIHTLLKVHISLYDTAKLVNYCYSLPL
LLEVTFIFGSVIQDIYNILIVSLDFEDLQTHGTAIEAAVLFIIWTVFRFLKIVCITTACQLASDE
ANRTGILVQKLMLREDSKSLRLFSEQLLHYKLSFTACGFFALDLTMLHSFLGAITTYLVILL
QFQFVAKSRPK
>BgerGr102
MANFWQRRYTIFENDIFNPKDIFSAIKPLMYTSKLFGLAPFVYADNPNCEKGLKISVRWTV
HCYVMCLSLAILVLATFENSVDFAFSNFNLTIGIVHMIDLCLSSFTLIVAYLLCATVNRQRVI
KFFSLVSKVDKHLFKSTNVYKRVHLSLLLCITLYILANVMDFVLCTINPIMKESYLQIQIMLF
LIHFLDSVSMFHFVFTVVTLRQRFSELNQQFLNQFSIRDETLLDENQFVSFMNGNVTSEES
TPIQHKLINNSLTDDTRNNIENFKNVKNTNSNISAQEGLSIKKISVLHNTLCDASELTNCTY
QIQVFMDLISIFVEITSCLYATLIYITKLLTCKLDNPSRWNLLTLFILWTVINLTKLLAVTASC
RNTSKVANHTAILVHKIMVARPLDTETKAELQDFSQQLLHRKLQFSACGFFEIDFTLIYSM
VGAVTTYIIILLQYSGRDIDDIKELCNKTSHNAI
>BgerGr103
MLDTKDIYCATKPLYYLSKVLGLAPFSYNKDRYQKASDKKLKISTLGTLHSWGMCVLISNF
LIFLFISNMKKEFPTLVIDIVNISDSYFSSLSVIISYALCATVNRNKVRTLFALLSRVDKCLVK
SDCSYRITGATLLMGVSIFLFAHGIGYYVVCIFIPTEAKDYHIQFVNFSIYVVGAVTIMQFVYS
VLILKDRFEMLNKELLNLFEIEDEDQLEGKLLNICHFTTSRKLDDDSGDHETCKMHVNKS
TIEMKLLESCDREAECSEENFNLSYAKRKERNLRLRCLRGVHNVLCDAAELTNSTYQVQV
LMDLIGILIEITTCLYLALTYTAQILTCRPTDAPRWNLLGLYVVWVALNLSKLLAIMISCRT
ACQKANHTAVLVHKLLVVQPLHSEITTELQMFSQQLLHRKLQFSACGFFNIDFTLLYSMAS
SMTTYLIILVQYTGQNIGEARIVELCNTTSQTTV
>BgerGr104

MDKIRSSILFNFVSDILQTRDFYSALKPLYYTYKFLGLAPVSLKTQNNSKSKNNFQMSTPSA
IHSAFMCIFLLILFLKIFAWHAGFIFTKHKLVISIVIICEKCLFSLAVIISYACCATVNGKKIVRF
LNLMSQVDSCIITSKNSHTRMFVFILLSVFSLVLFHFINFFLRLYILYMSHKVLYTSKHLVHF
MFTVLGEFVVLQFVFTTLLLKNGFEKLNKYLTSIFEFDEDIALNEWQKENFKSLTKNELKT
ISQNMSNVQARHISSTVTAVNLNEVQLLHSLRGVHNILHETAELVSSMYQFPILIILLEIFVK
ITLSLCVSLDCISKALTCKPLETLNVGLLQWMLLWTSLKCIRLLVITWSCQCVVQKAQSTAI
LVHKLLAFKALKPSTAEELRLFSRQLLHRKLEFTVCGLFQLDFTLVTTMASAVTTYLVILL
QHRGTTIEEIVDICNSAFLQFFGEGNS
>BgerGr105
MDTKDLIRSILPLHYFSRIFGLPIISDKTKGNRKRKIISEILDITLIFLNIIVMVTTVLIPLSSKL
LHVYSKWIMTDVLTDIILWVFSALSAVICLFSHVKGTRRKISKAIEKMYQVDQSLIPEPTKV
YGKTKVILLTEIICVSLLFGFLFGCDSFVWYEELDNNVYFVGLYLSHTIMCISNLQFGNIVIIL
WYRLKVLNEKINALGKYTSTPNNGQSNYRRYGPAPKITRHRRIGVLPNDYIIKKSSRMKED
ELNNLNIFYDDLKDISLLINSAFGLQILFNISTSLLNITVYLYEGLAVHLNLKKYLSENPKVD
LVMFINFLWAIFLAVQLIFIAAACSIASKESNETAFILQSILLNNDMTLKETEELQLFCDHVL
NRKLQFSACGIFTIDLSLLCSIAGTVTTYLVILIQTAK
>BgerGr106
MDEIDIFVLARPLYLISKCLGLAPFTCVSRENTKRFNVSYIGSLYTGTMIILIGVCFITSISAR
MTWVYPTVKTTTTVADVLILCSLVVTAIVNLIFSAITNRRGIDTILKKIADIDIQLFSNPKIM
YLKTKIFLYVQIGCIFSVLGILYCYDCWVWVETFGYRNMYYAVSYLVFTVNYVMNMQFVN
FVLLLRHRFNVVNSKLIFPDGTIFPNISCKENQTNPSYTATDIEDIPTSQDQLHYNASAFNN
HVVILVSEESAPSPAVFSRMRQVFFNLRILHDMLCDVAALVNDTYGFQILLDIATTLINITT
YLYFCLLYTLHLHSYDSEIIHFGHMLSLHLFWLAMHLFKIVCIVVSCHAASVASSRTGVLIQ
KLLLLHELDAESVYELEAFCRQLQPRQLCFTACGFFSLNLSVLCSIAGAVTTYLVILLQFQIS
Y
>BgerGr107P
MIMVICLIYFHTIKRLRNMLNKISKCDNIYTALSPLLYVSKIFGLAPFTIVRRSSSNEEVPIQK
QNKNWKNVYAXLVITVVLCCSILTVIGRVLFVYTSTNSTIKIVDSFILFSVLLTSVSSILSGLIV
TRGNFESIIFKLAKVDETLLNSPEEVYTKTFILLFLQQVVLIFWLSICFGYDSWVWVYVQRS
TRNVYYLIIYLLYTINIIMDIQLIDLVLLLKQRFAILNKTFPFMTVKEENAVESWSKFSETYG
RNYDVESVELENSYIFKNSSDTCHFITKSKHLLTKKQKILTIWKLHDELCDIGALINSTYGF
VMLLDLTSSFINIITYLQGLASVFNMETYDSKTREENCMLSLNIIWAILYSLKIVGVTSACSM
AREEGMKTMVLIHKYILRNDHELEVINLLEKFSRHVLYRNVRFSACGFFNLDLPLLCSVAG
GVSTYLVILLQFQKSF
>BgerGr108
MDIYKVFKHIQRLALTIGLDLFSQTTHINSFRRIIFSICFMFYRITLLLVTVLTASYVVFVSVN
QKDIEQIHLFQMPITVITSFLSLFASFFKLGQDLHTTVDRISLLDNLMKINYKMNNTSIYRR
HPNVHWIFIIFAFTLLTFIDLTIFVRSWFALAYVVSIDLGTYIHMVIAVQYLHFILLITERYRI
LNNYIILPKIINTSSRIALYNYLGILESNTEISNIIISEDFLHLDMKLQNRSLFEKRGEENKSHK
VKLQNETSHFQMLRVVHEVMYGISSSVNKMYGIQILFFFILLSTELTSNLYYAILFVVPTDN
LDRTFLSMMVPISWAVFYFILIFVFTGACKLAVDEANKTVILLQKMLLVPELDPGTMKEIK
MFLLQSFNRNIKFTACDIFNIDFHLLGSIVGAVTTFLVVLIQLRKIV
>BgerGr109
MVGPMGIYKDFKYIQRLAQTIGLSLVSYQTNEKTFSRIVSKTSFAFYSIILQSFTILSCSYVLL
TGFGQNDVMTEDLEQIHLSQMPITVVTGIFNVIVSCFNLPKEIHTIVDRISFLDNLMKINYT
KSNKSSPAMRLLTFIIVTFIGLSFYDFIFFVTNTLSLAYSISINIGIYMHLVLAVQYLYLISIIKE

RYKLLNNCIMLPKIINASSRIPLYNFLGIIQLNTPRINTVMSDEFLNLERKLKSHSFFRNTTQ
EIFTQKSHFQQEMSHFQMLRVMHEVVSEISSSLNKVYGIQILFFFIMISTELTSNLYYAILFI
ASADHKEIALLSMMLPFLWATFYFLLIFIFTRACKLAVDEANRTPVLLQKLLLIPELHTGTV
EEIKLFLSQTVHRKIRFTAYDIFTIDFQLLGSIVGAVTTFLVVLVQFRKIV
>BgerGr110
MDINSVFKHIRRLGITIGLDLVAPTTSKTFFSRTMISIFFTFYRITLLAVIISSIGYVEYTGIKQ
KGEMRDDVEHIHLFQIPVTTITAVFNLLISSFKLAQDVHTMKDKISLLDNLMKIKYNKFPS
LSLLLGFFVISAFTILTIYDLNLFSWNKLSLCYTVGINLGIYMHLVLAVHYLYLISLISERYKIL
NNYIMLPKMINLNTRKTIYNDLGIIQLYSEKKNTLLSEEFLSLDKKLIKHTLFENTTQENFT
NKTNFQREISHFQMLRVIHEVISEISSSLNKMYGIQILFFFIMISTELTSNLYYAILSIASTDH
REITLLTIALPFLWAMFYFMLIFIFTRACKLAVDEANRTSVLLQKMLLIPELHPGKVDEIKV
FLFQAVHRKIRFTAYDIFTIDFQLLGSIVGAVITFLVVLVQFRKIV
>BgerGr111
MNVHSVFRPIQLITKIVGIDLLSKTSNKKSLRGRIVSILFRFYRLALIAFTILNTVYVVINNTK
EEWINNGDIEQIHVFQMVITVTTNFLIFCASIFKQGPELYLTLNRIFIFDTLINVDRKVFKKF
TTQLMKVVIFILTTAFLLISMDILTYVNNLISLSYTVGVDFTVYMHLITELQYLYFILLILDRH
KILNDFIMLPSIINTCPKKMLYDYLGVIHLEKMKYVNFLSTECVYNVSLKNHPHMGNESQA
VILKHMDCFKKQQLHFQMLRVFHEVLCEISSSINKMYGLQILFFFITLSTELTSNLYYGILNV
VSPEMRRPPVLNVMVPCFWAVFYFLLIFIFTGVCKLTNDESKKSAILLQKLLLVPELHPAIK
EELKLFLQQVLHRRVKFTACDIFTIDFKLLGSIVGAVTTFLIVIVQFQKIV
>BgerGr112
MKEKERIQTIFALLKPLHLVSKIMGLILLPNVHNESSGLVKYSLLNKIYIFTLMGASTCLTLC
IVGSEIYNRDKRWFFEDICLSQISFTVSTAMVNTIASVIQLRQCRHILYSLSMLDHLLKVPKK
VYEENRRTVVLCVGTVCVTNIVLFIADVYPFVYSDINIFCFICVIDFTVFVHFLIATQYLIFTSI
MKTRFVLLNDYMLLPELLKNNSKRKLFDILGFIEPSKNSFTKTGLSEDDRKASVCQEIFKV
SDSPSAIIVLNEGQSYEEHFEVSRIAHELLSNITSSLSNMFGFQILLFILMTFTEATSNTYYVL
RALSINPHPDLWTLSRFLLSTTWVLIYLISLFLVVRSCSLVTSENTKTSILLQRLLLIPELQPE
TVLNIELYLEQVLNIKIKFTAWGVFNVDFKLLGSVMGAIVTLLVLLIQFHKIA
>BgerGr113
MEKRIKDLYSAFELLNSLFKVLGFVLFEITSDEKGHVVVVRTNYTICRKIHVLILIFFNVFVL
LSIIMYRLYFSNSKFQVEDVHLGQLSITMFTALIYLVVFVLKVRNTLPKIVYKIAVIDQILKV
QNDAYTNSKTSIRKITLGFLLILFTLTLSDFSSCVYLFELFYILFSIMIDCCIYIHFLVAVQYVIL
ISILRDRFKIVNNYIMSSKIVYENSRKLLYERLDIMNSSIGKEIELLSDEFLEDYTISERRLSVF
PFTEQFQYPLETYRNFRIHFQILRIAHEALCTICSSVNEIYGLQILLLMITVFTEITYNLYQSIR
IITSYTDMEVFVICSSLLTIFWAIWHLSIAFITTGVCESLNDEANRTVILLQRLLLIPELHDDA
VIEIRRFLRQAINRRIKFTAWDFFSMDFKILASLVGAVTTFLVLLVQFHKIV
>BgerGr114P
MLNMIENDNAKYLLVVFKPLHLVSKILGLVLLPESSKDLHGIVKYSILNKIYIFALTSTICFST
VFIILSGVSTMEKLIYIEDIHFFQFSFTVLAGVINIVVSAVKLRRCKNLLYTLSILDGLLKVNE
TMYKQNQRAITGYIKXTLTKIFFVLVIDFTLFVHTLVAAQYLAFISVIKTRFCLLNNYIILREL
SKRHPTRKLFDILEITETLNDYIFNGKITHYLRLQATNHQEFSGANIDFFCLLGRSSMDGEY
FQVLRIAHETLCNIISSINVLFGFQILLFTLTMFTESTSNIYYVIHLVSITKLKPWMFVKLVFN
VLWVLIYLSSLYLVTRACNLVTSENSRTSIILQRILLMSEVKNYKVIPIELFLKQVLNRKVKF
EAWRIFTIDFQLMGSILGAIITLLVILVQFHKIA
>BgerGr115

MIENFNAKYLFVVFKPLHLVSKILGLVLLPESPKNLQGIVKYSIFNKIYIFTLASMISFSTVFI
TLSGISTINNISIEDIHFFQFFFTAWAGVINIVVSAVKLRRCKHLLYTLSILDDLLKVNETMY
KQNQCSITRYIKVLFLINILIFSVDVSLTLFTSISIFFLLIIDFTVFIHTLVAAQYLAFISIIKTRL
CLLNNYIILRELSKRHPKRKLFDILEITETLNDYIFKGKITDYLRRQITNNQEFWGANIDSFC
LLGQHNMDGEYFQVLRIAHESLCNIISAINVMFGFQILLFTLTMFTESTSNIYYVIRLVSITK
LKPWMYVKLVFNVLWVLIYLSSLYLVIRACNLVTIENARTSIILQRILLMSEVENGKIIQME
LFLEQVLNRKVKFEAWRVFTIDFQLMGSLLGAIVTLLVMLVQFHKIA
>BgerGr116
MTENVNAKCLFVVFKPLHLASKILGQTLLPESPTELQGNVKYSILNKIYIVALASTIGFSTVF
ITLSGVSTLDKNISFEDIHFFQFSFTVLTGVINIVVSAVKLQKCKHFLYTLSILDDLLKVNETI
YKQNQLSVTGYVKNLLLINLLLFSADLSICPFKSISFLFSLCFLLVIDFTVFVHTLIAAQYLAFI
SVIKTRFCLLNDYIILWELSKKNPRRKLFEIFDISQSSNDFIFKRPTTDDLSHQVTNNQEIW
RENFSAFCRYGQHNTDDHCFQVLKIVHETLCNIISYINVMFGFQILLFALTMFTESTSNIYY
VIHLVAITKLKTWMVMKLVFNTLWSLIYLSSLYLVIRACNLVISENTRTNIILQRILLISELR
NDKVIPIELFLEQVLNRKVKLEAWRVFTIDFQLMGSLLGAIVTLLVMLVQFHKIA
>BgerGr117
MEKRIQQLCMPFKPINFACKVLGIVLFELFQNEKGNAVVKGVKHTIIRIIYIFTLLALNIITVL
MILILRFSSSSKKFNTQDVHLFQLPITVLTAMCYLFVFIIKLRNTLPNVLYRIAVIDELLKVK
PGLYRSNDAQIRKNIGFHMLVFSILVFGDFTIFDSGFETFFVLYIVVIDFGVYIHLLTAIQYVI
LIFLLRERFKTLNCYIISPKLMNNSRKRLFERLDIMYANSGLERDLLTEEFHEVNHIFERRF
PAIPFSEQDVYLQEYFQNMKAHFQILRIAHEGLCTVCSSINDGYGFQILLLMIVVFTEITSNL
YFSIHLVTFSNNFDPVDIITCISFFFWGIWYLIIAFITTGICKSVNDEANRSVVLLQRLLLIKE
LHQDTVLEIRRFIRQAINRKVKFTACDFFSMDFKVLASLVGAVTTFLVMLVQFHKIV
>BgerGr118
MEKNTVEYDLYSVTRLLHILSKCVGLSPISLLHLSDFKSCKSFKLRLCDVSYVIAISLMLLTS
VCVIVVLNLIRYNENDILAQVNLCQATVSIISSIVCLILAGVKLPNEIIMILNKLSIIDSIFGVQS
WKYERNVKTIRKYITIFISIYFTICSMDVLEVVKGPISLFYIAVVNYSIFVLFVTILEFTGLVIL
VKQRFKLLNCCIASFSESFENARPNMNLCQKLGLVNFNRKKTNISFRVNLRPENDNSVFP
GTHVQRINIRVLRIIQESLCDISCLINSTFGVQMLFTVVSIFLEITMNCSMSIILVESQSSFVQK
ALSALLTVAWVLLYFFMLFLMAGMCNSTCIESNNSKIILQRLLLTAELHPDIVGEITQFLQQ
VANRKVRFTACDFFTIDYRIMGSLVGAVTTLLVIFIQFQLTS
>BgerGr119
MSNMREKDRFKNMITLLRPLHWVSKIVGLTLLPGEQTDVLRTGTTKYSVLNKVYMVSLIC
ITICCTILIMINKASTMETDGVIEDMHLFQIAFTVSTAMINIIATALKIEKCKRLLSTLPILDQ
LMKVPNSLYTENSRIVRINVKTLCFVSISMFVIDIHTFLLDPFNFFFLSVIDFAIFVHFLIATQ
YVTFISIIKTRYILLNNYICLPELLNKNPRKKLFEILEIMDSSNNRILQIDQPKDQTTAYQHI
WNVNLYIKTVNTFNKSVNYGQHFEILRIVHETLSNVASSMNDIFGFQILLYTLMVFTEVTS
NIYYVIRLVVLQSSVDLQIISRSFFCLIMPVVYVITMFLLVRVCNLVTSENTRTSILLQRLLLIP
ELKQSTVLDIELFLKQVLNRKVKFEAWGVFTMDFGLLGSFLGAIVTFLVILIQCHKIV
>BgerGr120
MKKDTVEYNLQSVTRLFSILSKCVGLSPISLFSVNDFKSYEKFKPKLYDFIYVFMVSLALLT
ALCLVSIVALIRFDEKDDLAEVNIFQALVSASSGLMCLILGVIKLPNTIAEILCKISLIDEEFKV
ENWSYKRYTKLVRRRLLIFTTVYIILFCADIFIVVEGQLTLCYILIVNYSISVLFVTVLQFVGF
VHLLKQRFKLVNYHIVSLGESMMHTCHSIDLCQKLGLVTTAKIFKLSNQISSRVLFTKEKIN
NQCNISFSWTQRQKFDIGILKIIHESLCDITSSLNSAYGVQMLLAIVAIFIEITMNCNSSVIAL

HRNVNTEKIARSLLLTVLWVCMYFYVLFLMAGACRAACKEANRSGILLQRVLLIPELHPVI
SGEINQFLQQVTNRRVKFTSCDFFTIDYRIMGSIVGAITTLLVIFIQFQMATW
>BgerGr121P
MREAERITSIYMVFKPLHLVSKILGLSLSPNFNTTSPFAVKYSVLNQVYFFFLIFIICCSSVFV
VINEFSQEDNLLEEIHLIQFSFTVLAAAVNTIICAIKLRRCRHMLYILSNLDHLLKVTQRLYK
QNEKFVKQQVIVICIVRFVMFLADFIQEYISFSMFIYICAINFTLFIHFLIATQFLIFTSIIKTRF
SLLNDYILLPNLLGKNSRRKLLQNLGICESVKSQVFKEKSLNLMPTSLHXNGIWKTNINES
AICQYNSSKMYEEHFQLLRVAHECLCNIVSSMNDMFGFQILLFTLTVFTETTSNLYYVIRLL
VLSGDFTIWTMLKVPIDMFSVFVYLIFLYLVVRTCNMVTSENRRTSVILQRLLLISELDETI
VRVIEQFLEQVLNRKVKFTAWNVFLMNYELLGSVFGGIITLLAILLQFHKIA
>BgerGr122
MAANDVYSSAKILYYLSKTAGVAPVTLTTETGNNRMKFLNLSKKSLYEVLPGIFLTIIIIVAL
ILTFLDNKKLPPSAKNFLQIEVSLTTITTVACLILSITKVRKEIGPILSKVKILDEILMTNETIH
KKNMRCVFLQIIVLFPLLVLRDAYDLWAWTKVWNIRGHILHIIYVCAYVDIVVICQFVNLV
LLMKQKIYLINCCLTSSVGEEEKFIGYMPNVWEELLRICNFPNAGILNGNVIRSHEFYRILD
KRYINYSKKSSWNYIKITFLQRGRSHVRAMRIIYDVLCDLCTSVNSIYGVQLLMFMVTKFLE
ITTNLNYSIIVIYKGVEFNFISIIYAIETIIWALLDFLTLVIITGTCETICQEVNNLGNMLQKLL
LIPELHSDTADELRLFWQQVNGRKIKFTACGFFTINKAFLGSIIGAVTTLLVIIVQFEPSVNF
>BgerGr123
MGEYPMNIYSSITPLYRLSEMIGLASLALVKKGSVYVGFCKSRKRVILNIFITTFLIINSLFLM
AHIYLYDALHKMLKMFNIEQLLSVPMSLIMLFSSIFKIQKGIAKIFKKFSQVDRFLLPQPDKI
YRLSAKLLKIQVCCLLTEVIFMTFYDFWAWHDDIWWYSLYISSEANSLIHQVLVAQYVNIV
LLLYHRFRALNNFLIKSFSESSSGKNPTDTLLNTAEIGQPIIFSPHTLIIDALNEEEITNVKLP
NMSNNIRNQSLLPAATMHNDQNVVNSRLFEISYRNRSKHIPNTALRKHVGHRIYILCILRD
ILCDIVSSVNEMYGFQMLIIIVMILVKLTINLNYAVVSYNHMGLTHKTVATCLWASLVFFIL
FAVTGSCNLASHEASRTAVHLQKLLLLPEAYQSANQEIQLFLQQTVTRQVNFTACNFFRID
FGIFGSIIGATASFLIILLQFQKLA
>BgerGr124
MGEYPMNIYSSITPLYRLSEMIGLAPLALVKKGSVYVGFCKSRKRVILNIFITTFLIINSLFLL
AHIFLYDGEYKMQGMFNIEQLLSLPTSLIVLFSSIFKIQKDIAKIFKKFSQVDRFLLLKSNKT
YLLSSKLLKIQVFCLLIEVIFMAFYDFWTWHDDIWWYSLYILAEANSLIQQVLVAQYVNIVL
LLYHRFQTLNNLLIKSSPEWSSGNNSTDTLLNTAGIGQPIIFSPHTLLIDALNEEEITNFKLP
NMTNNIRNQSLLPAATMQNDQNVPNSKLFEISYCNRSKHIPNTVLRKHKAHRIYILCILRD
ILCDIVSSINEIYGFQMLLVIVTISVKLTANLNYAVVSYKHMGLTHKTVATCLWAGLVFFILF
AVTGSCNLASLEASRTAVHLQKLLLLPEANQSVNQVMQQFLQQTVTRQVNFTAWNFFRI
DFSIFASIIGATASFLIILLQFQKLA
>BgerGr125
MEEVYDIVKPSLYLSKIIGLFPLSIKRLIGENAEKRDKSYVRRWCKLYSLAILVFLTSSNIYVL
FSLAIFHNNYSEYGGIYFLDILLSGIANVVCLIKATTDFPKRMLKVLHNLSNISNFFKTRERP
LKTNKVIYHCQIVILITTNCISVVLDAIFWYRSEFIWNLYVAHAEISMWVQNILILQFISFVIV
VRNFIRGMNKLLILSDDNLHTSHKHNNINSILLVKPRKLQVFTLNKTAKYSSKVVFNSLNE
ANKEVYSKEQKNIIGVCIHDIRLIQDILCDTSTLLNSIYGIQILVVVLTFIVCMISNLNFTVNQI
FPISSLTTKKFASGSISLLWVLVMFLMLLYLTLTCNSTVKESQRTGELIQKLLLNSEMECSS
KSELRNFAEQVKVRKIKFTACDFFDLNHHILGSVVGGITTALVILIQIQSP
>BgerGr126

MMTKYSLFSAVRPLYYLSKTLGLAAFSYDLNLDKKLHAVEYFQRKDIIWPSFMILIILSCFV
FRMLWNFVLNPSEYSSNVIVTTTLSRILLYGACLTSLVLGATVNRRIVVQILNKVANVDSTL
LKDTDPDKIYRKTFLFLMTELVILISLLTAIGYYDIYVWGSGYNFIFEIMDDYAHLLNVTVAI
QLINFVQLLKQRFTILNAQLSNVTSSENSSAAINVVEKTMDSISKVIRRSAVPRIEETVEMN
SVDSQIQQMSFRVQTISPSPVTPVNKVHSLRVIHSDLYDVSEMVNENFGFSLLMELTYNFI
DLVSSLYFTMDVIINNLKEVNQNPDSIHHMLSSTVWAVVCVVKIVSITSACHTTSVEAQKT
SVLVHKLLLEQDLGLDTKAELQLFAAQLSNTKVVFTAHGFFSVNFALLYGIIEAAATYIIILL
QFRS
>BgerGr127
MNFKTSTKPLYYLSLAVGLASFSYHRVCDENLFNEEKRLLWALCSPYIWSIFLLLLHLVGY
ASLMLWNITYDYTHYSRNVIIPDVMIILLIYSTCVLSLVMITRIHKKKIETLLKKIVSLDQILL
HETRAGIYRQTFIFLTIEVTAVVIISLIFYCYHVYTWTSGISCIYFISKDFAHFCNTVMVLQYV
NMAQLVRHRFRTLNQQLESVAADHEIRISSPGSTKNRLCDDHVKQNGHRMASPMEQRHF
LSELSMPINSQRSYESARIHTLRRIYSDLFDISELINAIYGLQMLLEIAYHFLSLVSHLYYALE
KLYGSEKLGENRESGVSGIFEVVSSLCWVANSSVKVLCITGFCHAASRESQRTGNVVHRLLL
LQSLKGDASEELQLFSMQLTTNSVHFTAGGFFPVNLSLVYSIVGAATTYIIILLQLK
>BgerGr128
MSLLRPNDVHSAIVPLYLVSRMLGLASYKYLKQSSKHPSASSSNTNDAYESERQFRTSKCG
VLYTAILLLSLLAWLTYSLVCKILYDSSDLKLTYVVTQVMTLCLSAATTVVSLGLELTLNRK
RLEKIMLKIAQVDRILMSEANSGHRRMSCFVIFELVLFLLLLGARHGYELWSRGGNHYLNV
VVRFIVHFMSTVMIVQFVSFIQVLKQRYDCMNKLLGLIGGIDDRGLSVEQGRVSFTQHRLG
MASFNSTVIVPHVDDTPSGPVSGTNSIFISDSEVNQSNPSPSKFPSKILPHDVPNIHTLRYV
HTILYDIAGLINSVYGLQILLAMAYIFMSIVKYFHVVMVTNINSNHEQINSFKVNGIFPLVCV
VSIHVANLLWVTIACNSACCEAEQTTGLVNKLLLIQPLAADTSAELQLFSQQLLHSKLQFT
AFGFFHLDFTFLYGFVGGATTYIVILLQFQ
>BgerGr129
MFSAGISRPFYTTKKETYFNPRYFHSSIKPLYQLSCLIGLAPYRFVQDSMSFDASKLQRTIST
SIPAIISIIVTLTWLVYTFYNIITKKAFKTHVILAASQITTVLLIGTCCLVSMILNLTVNRRKCE
VILQKVVYIDKILLRSVKIYRKTVFILLAQIIICSAVLFIFHLYEFSKKKNPNLVIQIARVLVHF
NNVYMIIQFVNFVLLLTQRLSLVNQELSLLSDESKEIAKISEMSKYIRTTVHTSEFLPTSRSE
NLTFRITSSPTEAERLGTRLQKRNLLNRCQDLRNVHHIICDILAKEVNSIFGISLLIVVTYMF
VSFVKYIHVIFIMVKDPASETLEALSVNGASVMVCFILMHSAKVLYISYACQTAYQESKKTS
SVINELLLAHIPGSELSMELQLFSLQTFQSKLEFTAFDVFPLNYTFLHMYFAGLATYTVILL
QFAQ
>BgerGr130
MCSECVSCLPFKNIKDTYWNPCWFYTSTKPLYHWSRFVGLAPFKFVVDFATTDSSQIRRT
LLTAAPSVVCTIVVFLRLLHVLISFGLNNKFDSHTTMVVSQVITLCLSALTALVSLITSLTLNI
RKWEIILSKTVYIDKILIRNPLSVYRRTIAILIAQLSFSMAIIGIFHTYELLIKKKKNINHAVRIL
VHMNSVTMILQFANFALLLTHRLKLMNDKLKELNSSSKQTPHKSSHINYNRTHTIRNINN
YSTNKPKARTIESFHHEELYPPLHIDSILVSNKSDLLKELKSLREIHHIICDVMAVAVGSIYG
LPLLINILYMFVSCVKYLYVILVMVRDPTSEFLDALDTNGAAAMVYLVLIHFMKVFYISFVT
NLAEQESKKTKAIINDLLLVYDSGNDVCCELQLFSLQALHSKLHFTAFNFFPLDFTFLHMF
FAGLTTYTVILIQLT
>BgerGr131
MHPEYQQRLGATNYKESFFAPQSFKSSLRPIFYWSKIMGIAPYKYVSDPVTGEAMALKSSF
YSKIPTFVLFILIFSWVLYKLIFTIVEKAYVGHETIVISQTITMCLSSAACLSSLLRSATVNIKK

WQRIVLKVIYVDGIILRNRAIVYKKTTVVLLLEVLLSVIILAAFHTYEIMTKEKKQLSHALRV
FVHTNNVTMIMQFLNLVVLLTHRLKLVNERLSELADTYTNHSKPKRPGYRLKTRGHMDY
NISRILRSLRSDRQNFEQDLANTEFEPHLGGSKLSSLDLIKELEQLREVHHIICDILADLIDSL
YGVPLLLALTYMFMSMVKYVLVIFVMMKDPESERLSALNVHGALPMVILIIIHLVKVVLISA
LCNSASAASKRTTDALNELQIAHSSSASVSSELQKFSLQAMSTKLRFTAFDFFVLDYTFLHT
FVAGLTTYTVILLQFS
>BgerGr132
MGDDLLFFSSFKPVYYISKILGLSPWSVCSENGRTKFKSTTPGIVYTFLIFVVFLVWQIYIIN
WRVNNDYEGASLSYIVPQLLSICSSFITAVSSLLMTITVGRKSVEKIVQSILVVDEALLLNNA
ETSRKYKSIAWLEVVLFVAVTGLLYGYDLWVWFGKKGDNYVVNRGLVYAVNALTIIQYVD
FIYLLKHRFQVLIRQLHTTIAVEMDVSFTEIIRKTDKIPSETKSLETAGVFKIRDEIFPGSTPG
LQPTSHLFTRRSKLDKKVANIHKYREIYSILYDISHLVNSAFGIQNLLQFITIFIVIVKGLHFL
LTTLDDPTVVKVSHEVQFHAAMLVLLWAGFHAIQLLWISIVCHQSCAIGNSTGYVVQKLLL
KELTEESRTELMNFSNQLVHSKLTFTAIGFFTVDLNVLCAISGAIMTYTIILNQAQ
>BgerGr133
METNIFSASRGLYLLSTAVGLAPRRNFAGIIWSIILLFLVSTGFLFSTIYYVLHLYPDRAPNLI
SADILSRVLLYGTSAICLLGSLVLQKGALSKLLNLIGDIDHDLYEVPVYIYTQTRKWLTAAII
TLIFLITPFFCFHAHVYGKGTNLYNILFDELAHIIILVTDTQFIIIVLLLWSRCRHLNFNLRNI
PNEGINSVYLQKRFSNATCRIHSLRKSYSDLHDICLLTNKTFGFALLLELTCIFISVVSTFYN
LMFYFAQTLKLGEAVGAGTTLCHIMWVLLYLAKVVGITASCHFAASENNVSGSIVQKLLLQ
KYIALDVVDELQSFFVQISAHRLEFTACGIFPINLSLLHSILGAAATYIIILLQFG
>BgerGr134
MCGKRSLYAAITPLYYISKLSGLAPFSYSKNPNTDTNGIDPNKSFGIFSVLWSLLLTAIIICGF
IISMNDKMSHAFDKMASKVIVADVCTSVLLFGTTISSLILGASLNRRKLWKILSKLSTLDER
LLYNKMANVYRKTFLFLIAEISAVFLFILLLYTVHISAYESNAITSVPEIMAHTVNLVNVLQF
VDVELLLRFRYLTINQLIPLALNQMKIASSTCQEASLNIPNQQELPKITSPNRSTWQQSYSA
SQKPLENEEMNVTYYNNFWNMSNQKQYAQDEQLNNRTTKQWEISSQKQTKSSAHVKN
EPPKNQLWNISNRVDGQISKCHRSNIKQILQLRTLREMHSQLYDVVELVDFKFGFPILMEL
GCNFVSLVSTLYTAMTFMKSVQQIGDTHYMQKIMAFVFWTVLYLAKAGAVTISCHAASEE
AAKSVAVVQKMLLDQNLAADLSTELHLYLTQLCNNRVDFTACGFFSVNLSLLHGIVGATA
TYIIILIQLN
>BgerGr135
MTDIYSIVRPLYHVSKVFGLAPFTLPHKSKHGWEELNYRFVDVIWTIFWLLWFLAGLPLH
MLHDILYKQDELPTKICISLDIYLISLYVTSIASLSLAGTLNRKKVPQMLEKVSEVDTYLHQH
CDCSSMLKSAKLYLVLELIVLSIPLGFLHCYNVYSFFDGTIWGCFVMISENFSYLLNTLVVIQ
FVNSVIMVRQRYKCLNSFLTISTVSLDDNTLKDIKMLLSVETIQISDPEVNNESKLCKNIKT
SELVRGLRAAHFALHDTVVLIISNYGFLVFMTTFWIFMTIVFVLYYGLFSLQEVITSSTTDD
HHEVILSLCWCIFCIFLLVAMTLSCHLTTQDANFTIVLVEKLLLCRDLDEDTVDEFKNFSTQ
LSNTKIEFSAFGFFTLNLSFLYAITGLICTHIIILAQFN
>BgerGr136
MRNMDFYDTIKPIYYISKIFGLAQYSFDTGIASESRLQTETPNIHIRSSGTLWTFSILCVSVC
GLFSVIAWNLKIDYTDYSLNVIISDTFSVLLLFGSSITSLLGGLLQRRNTISMLRKFSQLNKLL
LLQEERTSFYKKIHTFLLIQMSVCISISFIAYAFHIYVWGSGLTHFLNAAKDLEHFVGVITLL
QFINIVLFLGQLFRILNNKLSLQFNNLRLIPINLDNILLHKIVSFEDLGIEDVNLEGGGELLTK
LELPWGRRGTIAGNCRSDIRMCRILHGELVDSAELVNRIYGVQLFALLIYYSACLISSVYYLV

EKFTSMDRSDEISWTTIEGPISTSFWVVVVYFIKIVVVTKVCAGTCENARRTTREVQRLLLCR
NVPISELKLFYLQLLGNKVDFFTACGLFSINLTFLCTVVGAIITYVIILFQMND
>BgerGr137
MDLNRAQDIYAAVKPFYIVSKIFGLAPYPLKGKAKTIRQINGFMFLNLLWTSLWVIFFIFG
MIMHVTNLRVFQNEEVSMKRLVTFSVFIFCAYCSNIVSLILEATVCRRYIPKILRKISAVDSI
LMEQSKEVIVYSKTRFILVTEVLILSVMLAIFSYCYIWVFCDNTISCICAVSQTLSMVSNVLM
IFQFINFTILLKQRYKILNSWITPIYKSNNQRTHDVSNPKPPENKHATKLFLLIQSYRRFKEI
IRNHQEGTSDGCDEVIVENKTTKLANILQNSHVFKDGSRVYVLRIIFSELYEIVLLVNKNFG
WSLLVGILWMFVSIVCNVYFSVDKINAMSNSKDQISQCKEISLYIVTLCCSLALMAIVTACS
HLAFVETRKSEALVHKLLLLPEIGPDLTEELNLFASQASVMKAKFTACGLFVVNLRVLFAII
SLTATYMVFMGLID
>BgerGr138
METPNVLQAIRSLSVVSQALGVAPVSSRFFKMYNVCLTAFLVISMIFQIISIDQNSPATVLFT
TSIVKIVSSAATVSALTLSWRRKDFSQLIRKINLCDKILLTEPSVDYSIMRKFLFLQIIVTFSIL
IVHFSYDCWVWKELDKDPWTFLTYSTEIIRFVVILQFVDAVILLRSRFSILNKLLLQNFEKS
VLPIKPNSAVWYNDTNIGNKIDENLSLDINSRLFNTNSVITNRYIFVNEASYLPTIPIPNKKK
TAVQQIRSVYNYLCDIVNLMNRIYGIPMLLQVVLLLCGLVRTLYYIIVTIFRPNIANFSVRTM
AYSVSVITCWFLFRLLTLTAVAISSERMRNEALRTAEVVDKIIIFEKDLISELNYFSLQLLLA
RRKLELTAFGMFSLDCKLLHSVAGAVTTYIIILIQMHEPDVIPEETSTSSIQNV
>BgerGr139
MKKKKMTKNSLRLFKKAILPFQLWYQVLGLAPKSKLYSTMVIICVIAFFIYELYSKIKSKFV
RLRMTLLIPQIFVALCAFLATVASVTLGFRRVAFNRIIEKLSFCDHVLLSEPSAVYRKMRRF
LTFEILITLLVIVAMCCYHYSVIKGPPTNFILSYVMELIRNCMVIQFISMVVIVKNRFEILNEM
ILEFGTGENDKNYKGFLKNNYPEEREKVYFSRNSSFSQFKRFRLRLIRNTELLIPLTIIEARK
AHLKKLQTVYNSLRDIVETMNSLYGLPVLLQLTLVLTGNIRCLYETITYVFQSSKRCPAKSS
THCIVLLILFFLLRSLILVFVSVVCDSLHSEAQRTSVVVEKVSLTETALLRELKNVVQQLLLA
RPRVNLTALGMFSLDRRLLHSVIATMTTYILILIQMQQMSQNEDQVVSTTNVTKQY
>BgerGr140
MLHSKMKQLIKLKNINQCTNIYTGIKPMYYFSKMIGLAPYSFKIKSDKIIYAYINSFPVYELII
VLIIVLCTAAIFFGRISYLYVDGREKGSVLDATVWCCLSSLALSSVIFGLIFRRIMVISLNRIAK
LDQHLLKDCKVYKKATFIEYIIIIFLILLISIVIIDFLWMSQEFNKNFMFFALPTYIIYLEYYTM
EAQFLLLVLNIWYHYKVLNSKLINKIQPNATEIPCSFQNSVNIWQFSNKNVTNDNKIKWN
SNEFWKLQGMHSTLYDIASSVNLIYGFQILLDIAFTLFDIIVSLYFLVTFKLGTIPNNRFISNL
LWSLISIVRITAVMTICHLTSNQANGTGEILQKIIYMKTHRCEEIKEFLQELSNRKLKFTACG
FFTLNLSTLCTMIGSVITYLVILLQYN
>BgerGr141
MAKSKNTENGVNKFEVTEGFFSAIQVLYYFSKIIGMAPYSISNIITPEGKIKTILYKAQLISIY
TFIFLLLVFISIIISITMRVLYAYPDAMKIVLVPDALLWGSLSLTTVISLVMSLIHKQKMVYIL
HKISKLDIIILTKHKEIYRNSRIICIFSLLFLITTIFTLLIAHCSSLMERSQSNFRILIIPGYIVYIIN
NTMILQYILILVCIYHRYKVINSYLVNETIKLWNNGIRYNSSNESRLDKMTSVFTIGNSENA
RGKHDREKEAISNTTYFSLNCRTDEMTSHEIEHNKKDLSYNLIPANIFLSYSGREEFGMSQ
FRCKQNIKNLRNFHDILHDIVTVINTIYGVQIMLDMTNTFVDIVKELYCVTLHAMQMEGT
NHITNHGMVLSVCWVLLFILKFTSITTICHVASNEANGTTVVLQKMLLIDDLNSDMEKEV
QGFLQQVLNRKMVFTACGLFTINLSTLCAVVGTVTTYIVILLQSSPHI
>BgerGr142

MHMSTGSRKSESGSNSLKIPKTPFDLYTAITPLYYVSKAMGLAPFSYKRQKTYNGQKTLIQ
RSKAGRLYTVFLMLLICVCQGVNLHGRLTFMYPIQATTIAVTDFMASLSSVICTAASFIAGA
TFGYWKLVRILKKMSYIDEILLVNPDSIYRKMTIFLNIQLPICYTVIIVLCIYDYYVISVVLGN
ELLYYVPPVYLIFLVMYTMDIQFVNLILLIWHRYKILNLQLETKYNFDKRLKIWKTDYLSV
HKENKNLTYDKDKNTAFQTVKPQETIDLQHDIFSMANHFHQFSFEKIRTLRVLHNLLTDC
AFLTSSMYGFQLLLDMGNTFIRTTTCLYKSVTFAVDMQSNDGQEDIVMKTHVFILYTSWI
PIFVLKLMCIALSCQLASNEANKTVTLLQRMLLVENIEQNVRIEINEFLHQVVNRKLEFKAC
SFFKIDMSMLRGLFVAVFTYLLILMQFYIPK
>BgerGr143
MGLAPFSIVTTAQNGERMQEIKISRNALIYSQIILILILFSIGISLALKIQHEFSHQFITSVIPEI
MLFISHNVTVIVALVLSITYKEKIMKIMSLILKINRIVLVDSNNYYGSLQRKLFWKLILIIILVI
TVILYDCIVWSLAAGIENLSYWQMYIDVAVDWAVIMQFMVFTNILQDMLTILNSCLLEFSK
HALCPTTQTIDDVINISPIKFSVVLNNENAQLTHKNMFTYNLAHDLLCDIASLIKSIYEIQIF
LSLINAFVSMTIWSYFSFCILYGYIEAPNVQVDIIYLVTSDLLWVFLNLIKIICITMPCHSVNI
QMANTRVVLRKLLLIRSIDSAIMKELEIFSQHLILRRFKFTVLDIFNLDFSLLCSMIGAVVTY
LVVLMQFKISENIPCEHLFNFTQ
>BgerGr144
MSRPLSFCSFRRMNMKYKEDIYKAMKPLLYVSRCLGLMPYPLLFSKQEIKSWPIVGAIYQL
FIVILTITAYIAAIAFKADGYYQTKSHARILSSFFYWSSHSITAVICILLSRIYHEKIENILKKLT
MTDPYSLSNPTTFYRRINHFVTAEVSIIYPLIMIISFYHSWTWVCMINTNFHFLGNFLFISIN
VTVTIQYINLVFFLYYKLKALNKCLKPVYSYDHKNKEIQKDKIILDVYKRQCLKPVYSYDHK
NKEIQKDKIILADTSFNNETDEQFKYDSHSTFNPIQKNRINIFFSLSSIRKLRQQQHFLTNIA
DNINSVYGFQMLWIILSYFIRLTANLYTPLYYAKNEANNVDNDPQAEMHILCVNLLWAAL
TSIELTCVSAICHVTKEEAKHTGILLQRLLLTETVGSPLSLEIRSFLEQVVHRPLQFTANGFF
NIDLSFLCAFLSALVTYIIVYLQFN
>BgerGr145P
MEDICLFIKPIYYISKFLGLAPFEYFEHQQNGRSLRRLRISRFGVYHSFLMCVIVLSVLSIAMV
SKFNYFYPTKTQQDTITSVMYWISYTVTALSSLLGTVIHRRKLLNLYSHFQNEREIFVDMN
KIYNQRYHFIKLGMFVIYAVVIVLSCYDTWVWITILGSKWDFIGFYLVYLIISTMEMQFLNI
VYVLYNRFKILNTCIEHNVIEVQDMTNTKENGTFYTTYKNIKTTSKDIQESGRNFQAISKA
QTDLRSVVPEDNEAQTTRTKHFEFDTSNKGIKVQYELNCTLRTLRRSHYQLSNLSDEINCI
YGFQILFDIITSFISITRNLYFGLYFILKKSRILDYPFAEKYVLLAELSWSLLSAMVLISLTTIC
QLTSSEAXVTAVILRQFLLTETVNSPLSEEIKGFLEQINNKPLRFSALRLFNIDLPFLCSFTG
AVTTYLIVLLQLH
>BgerGr146
MKVSFVIESLYCSLRPLFYVSRFLGMIPFSLRIVTVGTRTGYLKKSAFLRTWEFFQTLLVLS
MLVSCAVISTYKRLNHKNIVFMVIASDVVKLSVFSATSIASLILCGMLNGRKMKLILSKIIQV
DKVLLINPTWDYRKLYVLLCIEMVLIYFSLCVLFVFDGWVWTHGIRNHSFQYYVSTYPFHV
VNMTMAMQFINFVLILRSRLHYLNDQLYSLLRTLKIKSLPSIRGHFSEQEPWFFNIHYTPTI
PYQHMSSRICELRSLYDKLCDVASLIESCYGLPLLFDIAGSFIDVTSIFYLVLIVALGYQEVTA
NAKGLVVSLLLSWATLYLVKLFGITGSCRHTSVEATHTAVLVNKLLLLRPLRPDLEAELRL
FSLQLLHRSVRFTAFGFFTLDFTLLFTMAGAVTTYLVIYLQFKT
>BgerGr147
MKHVSTLYTSLLPIHLSSLVLGMSPISLRHSTEGTIRKYDRNFGICRRFYTLTIIMIVLTCSSL
SIYNRIEDSDIIVPVIAGDIVKIVLSTLTSIMTLVICGILNYRRVILILKTLSKVDNLLFVDLTN
MYRKIHRISLSVVIIIYSLLLLLFFFDGYVWKTGIQDISILYFITTYPFHIINVTLALQFVNIVLL

LGNKVFHLNKKLFFLYNKYEQQTGSTFSEQVRVYDFHLISSAFTKENMSLSQITFRIKELM
LIQDALVDVSSLINTCYGLPLLFDFTSTFIDLTSILYMTSAILLGYQKMTGNSAMLMVMLKF
FWITLFVLKLLLVTGSCRFTCTQFSRTATMVHKLLLQCHQRTVLSAQLELFSQQLLHRQVR
FTAFGFFDVDLRLLIAIAGSATTYLVIYMQLNE
>BgerGr148
MKTILNPKNFACSIRPLYYISRLVGLSPIAMDGSNNLKEVSIKWKIYSAFVLLILVISSTFSIV
QRVKQSELVATVIVNEFLMMSVGAIKAITSVLLCLTVNRSQVRRAICKIVKIDSSLLNNPEE
TYRKMYLFTLIQTSVVSIYTTFLFVYDTIVWTHAVEQMDSWYLITGYPHRIVNITSVVQFC
DFVLLLRNRIKSLNFKLIAILKATDLSKKTMALSSSNRFSSTSSYNILNHKKSSKEQTPKFISI
ADSNKVFTHVEPTPIKTSEVDTLHNLRELYDELCDVTSLINSIYGLLLLLELGVTTIELTSSL
YLLMATLLKIQTVEIDIMSQFTSLMIAWLIQYSFKLICITAPSQAATNEMENTIVLVHKLLL
AKRFDHGTIMELKLFSQQLLQRKVKFTAFGFLNLDYSLLFTIIGGVTTYLVIAMQYKK
>BgerGr149
MTDATMKFKNSVLFLDRTFISFNKRTFVASIKPLYLISLIFGVSPFSISSSNDTTELSTSKKV
YSIVVLIIVTVFGIFSFIERVGNTQLLPTIIVTESLLMCMGTLNASLSIIMSLTVNQKTARKLIT
KLIDLDKYILPDPNREYQKTFISIVVHFSIVCICTIFLFAYDSWVWTHELGIWYLISGYPHRIV
NLILVSQYCQFVLIIRRRLKSLNKKLKYFLKGVDGCHKRVLFITDFSDVYVTRITSLSGSQLN
TIPVQAPKIINYDLVRQMRKQYDALCDSVLYVNSMYGFLVLLELGVASIELTLGSYLILATIF
RIHIVDVTLFTTNQTIWLTQYFFKIISLTAPCQAVNTETEHTVILIQKLLLLQNLDNDTKLE
LKLFSEQLLHRKTKFTALGFLNLDYSLLFTIIGGVTTYLVIAMQYRS
>BgerGr150
MVQHIYTFSSSLKPLLYFCQIFGLAPVQVYNGNARDQGYLWNVYNVVILLTVMVCGICSLIE
RFNLSELLKTALVGEVFVMLIGAMKAITAIFLCTTLNRTKFKKLTSILIKLDSVLTADQEVY
KKHFNFTIVQIILVYVYLSVLFIFDLFVWHIYMNQMSIWCLVAAYPHRIVNLACVLQYSDLV
LFLKGKLHALNLKLSSAVGNGGNKYSINSKAPLQSSKLFLYVNSSQVKELSNKDEKKHVIHI
IRNLYDELCDANTLINSIHGPQLLIELGLSGVELTLALYLVLSAIIGIQEVNMGMFLILQVLW
LTIHIFKLISVTTSCQLVNNEYNETVVLVQKLLLQKFDEDVLKQLHFFSFQLFHRKIRFTVL
DFLSVDYSVLFTIFGAVTTYLIFALQYK
>BgerGr151
MAYNYVFSSSIKPLYYFCRILGLAPVSIDKSNVVKGQSLTWKVYCIMVLISVISFGTIAIIERF
NEAKLLTTALVGEVIVILIGAIKALTAILLCNTVNSVKFKKLTSIVIKLDSLVLKNDQEVYKK
SFTFTLMQTVILCAYLVTLFSYDIFVWNISMNKMSLWCLATGYPHRIVNLACVLQYCDLVL
LLKSRLHALNMILKSTLGYNNTQVSTLSQFQVPKIFLPIQSSQMKITSRKLNIMKDIHTIRN
TYDELCDVNALINSIHGPQLLIELGLSGIELTLALYFLLSTILGIQQVEVSIFVVLQVLWIVHH
AFKLISVTAPCQSVNNEYDETVTLVQKLLLKRLDDESIMQLHTFSFQLFQRKIKFTVLDFLS
IDYSVLFTILGAVTTYLVFALQYK
>BgerGr152
MEDINTVIKDIEYSLRPLYVISRILAICPYTQLSEMGKRCNTFKKLCMAYPIAVLWMILMFI
MGTMAFIFRIIITFVKPPSDPSIVISTVFAMPLSHISGLISIIMSSIHNRYKMAELVYKLAIIDK
CLCINTNKSTYKRHNFIVLCSVGMTVLLLAAFYCFNIWVWSDGFNYTIVYEIVIHVCYLVNG
VVFLQYLNWIEILHYRLWRFTQQIPKKDWVVNQSLINEISRISVPNIIKTHNRSRNIIKVKP
MIDNNLFFVPMKKYNYKTSRSESNYENEFTKYILNLRTLYNHIFEASILIESMYGFIMLLEV
AANVLYFISNMCSTITVFINFTKNSPVIFIASYCGWSLISLTRIISIVVVCHKIDKEHDRCVDR
VQKLLLRRDLSESAFTQLDLFSDQLEYNKIRFSAFGFFQFDVSLLSDIMTAATTYIIVLIQYR
NNMDTDSEKY
>BgerGr153

MNKMSIYTVIKPFYYISRLMGLVTFRIRKANDNSEYVDDAISRNIAGMIWSAVLTTTISCAF
LYSLSRFTDSSHRSANDFVEAALSLQFSFLPSTVTMIMGLSVNRRKAAQLFKKMTMVDNA
LLTDKIPKTYKKMKCFLCFQSVFLMLVCFPLFCFDEYLWNENGEKSVVYEGILRLSHFITLV
IDIQFTNLVLYVIYRLRNINENVLVFPNINASPKNNIKSKITISAENIQIVDCDNSVAPLEND
NTNTFANTLTFKTPQNHLLIHFIRCSYNNIYDVVNIINSMYGLHILIETFSNFITSIEVYYITY
KYFLFPDSDNLHLVSILGWAGFFLGKQLAITFPCHLVFQETKKLMDQTQNLLLIYPIKDEFL
QQARLFKSQLTKNKIEFSAFGVFNLSMSVLLTFIASAFAQLILIIQLKVV
>BgerGr154
MDSCQIKPCNILYWLKPLYLMSKCVGLAPFRLTVDLLICEDVKEDDLKCNLRYFIWPAIVF
AVILMGFIDCVSHLHLHSSNPGCIAHMLTLPLTYITALVTMISTITVNRHNVIKLTRMLCKI
DSVLLFGKCKGIYKKARFYLILEISVLFLVVVPFMAYDSYLWDTWTNTSYVFEIITRVSTLIN
LVIIVQFVNVVHFIKHRLKMLNKLFRDNQIMNTLENNLQPCDITDSMKEFMTLGNFTRFR
SKLKESIIQRKNRIGSENLKKIKKNIFKSSRRIKVARVLSACKDDRNNLPSLSIIFANDKHLN
VAQILKLRIVYKFLYETLKLVNSIYGLPILLLVTFCFGGTVSNIYAILDSLLGSGTKAMAPSYII
WMVVLSGSATAICASSHFAQIEAKKTVDEIQTDLLQNPLKKSVCKQLKLFSSQISKNPIEIT
GYGFFSVNLSLLFTFIGSATTYTIILVQFKLNK
>BgerGr155
MIRTSFIPTFEMSKINKSSAVGYDCKIIYYLFKVTGTAPFTLKVATRGQLRETVSTDLRANIF
SIIWTFCLFSSSFIGLCLTMKRDIPFNEPGFLFGYIITLPSCYLIMLFIIIHHNVILKKEKVKLM
DMFVKLENDLLQEREPGNLAKKILMSIFVFLNAVYLVFDAWYWKESSSFKCEFMMRFFK
FIHLLFILQYSYIAKFLHKILEQITHSLRQTKSIPFGGINIIISELIPVRSQVALFNTRALMKLS
NFDKASEIRKLMKYYFKIYETVKLVNSVYGFIMLILVTWQMCEDIIFTYGILSYLLGNSMHH
DMFFRYDFVLQSGTWILFTTIHLIIVTNNTKSVVQETKDLADEVMKELLEHSVESNVRKQL
KLFSYELASNKLDFNAIGFSFSPKFVCTLLASVLTCNCPCSI
>BgerGr156
MSKTSTMGYDYKILYYLFKITGTAPFTMKVKHRGQRVETVVTNLREDIFGIVWVLFLVSGS
FMGLYWSFLRTIPSNDPGFLFGYIIALPSCFFIMLFIIIYHNIMLKKEKVKLMECFVKLENDL
MQERKAENVTKKIMMSIFVILNAVYLILHSWYWKEMSSFKYEILMRFIKFIHFLFILQYRYI
AGFLHKVLAQITFSLRQTKNISFGGNSTTVSEIIPVRSQLEFLNTRSFIKFSSFDKASEIQKLM
KYYFKIYEAVKLVNSVYGLVMLILVIWKMCEDIIYTYGIFGFLIGNPTYHDRFFQYDYVLQS
GIWILFTTVHLIIVTHNTNSVTEEAKDLSDVLMKELLEHTVESDVKEQLKLFSYELACKKL
DFNAIVFSFSPRFICTLLASVVTYSVVLIQFKQ
>BgerGr157
MEKHNTKLLLSDCRKLYFMFKITGTAPFSLKRTGFHNEETLSTSPRTNVFGIVWTLCLVT
AALFGLVWTYEREIQNSADPGFMFGYRITMPATFCTLIVMLLNHNIIARRNNVKLMEALV
KLELELVGTRITESKIRKLAILSFGVFVGALAIFESWYYKDMSNLFYESTLKLIKFIEILVVLQ
YSCIAIFLQKPLARITYFLKMETRISKRNKNVSQISPAANEPTIFEAPPSPWHSNETARIQKL
MKDYFKIYETVKLVNSVYGFTMLVMVIRTSIEGTNSLYAGYATATGILGYNDRFFRGDFMF
LAVTWIFNTTVVLFISTYCTNLVVVEANELGDEVMRHMLEYPAFDVGLEQLKIFSNELALK
KIEFTAVGFTFSPSFLCTLLASICTYAIVLVQFRK
>BgerGr158
MSNMCEYRKLYILFKITGSAPFSLTYSAGKAEETVVTHLRATIFGIVWTLCFFSAALFGVIW
SYEREIQNSTDPGYIFSYRIFLPGTFCIMIVLLLNHNVISREKNIKLMEKLIGLEKEFKVARSP
EINTKNVVLLIFVFSNAGLFLYETWYYKSMSNIYYEFIIKLIKFIETLVILQYSSIALVLYKFLA
HVTSSLKMHKIVVPHRITGIQDFDRKTTVKLTNSGKKSKIQKLMEDYFKIFETVKLVNSVY
GFTMLVLVARQSIEEIDTVYAWYADAAGVSGYYDRFFQGDFMILALMKIVGTALTLIITTY

CTNLAVSEANELGDEVVRQIVQHPASNSDVEQLKTFSVELLLKKIEFSAVGFSFSPSFLCTL
LVSICTYAIVLVQFRK
>BgerGr159
MGKVCECRKLYLLFKITGTAPFSLTTVAGQDEPSMSVHPRASISSILWTLCLFIAAILGLVW
TYQREVQNSADPGFIFGYRVSLPTSFLIVIILIVNQNIICRKNNIKLMDALIRLEHEIMDERM
FESKLRNVTILTFVFVNITLFIFEVWYFNDMSSFIFESTFEFIEFIQILVMVQYSFIAVFLQKA
LATIRRCLEMKNRIHVRNIHISDLSVVNIRAQTFEKQTFPNLWITSKSSKIQKLMKDYLTIY
ESVKLVNSIYGFPMLLLVARQAIEGINNLYAWYAMTSGIFGFYDRLFHGDFMILEGIKIICV
ALSLVMITYCTNLAVSEANEVGDEVMKQLVENSATGRDLKQMKIFSNELMFKKIEFTAVG
FTFNPSFLCTLLVSVCTYATVLVQFKK
>BgerGr160
MNKCDDNEFESKRLYYLLKTTCTAPYSLCTTRNKDKFTSTKENVSGIIWTLSLFCIAFLSIV
WSYVGRPGNRSIDPGIIFGEKLVLPANFLVTIILILTHNIFLRKKNILLMELLIQIEHELPQKL
NSGSKAVRILNYCFLVLAGIMFLLESWYWESASSFCYELDLRFNRFVHLLVIYQYCTISHFI
KRVLYQLTNTITLKQENFNIKVQVTPKIVPATQNVIFGNFINSQDLEYEGKIKIESFTEFYFK
IYKTTKLVNSVYGITILILVLRYSIEQTCSLYTVFALVYKTSGYDDMYFPGDFMLMTVIAIIRS
IVVLCIVTFCTSSAVEQYKTLSDEVLNIVFNHRSSAQEVEGWSLFANHLSCMKIEFSVVAFS
FSPSFLFTLLASMFTYALVLVQFRQ
>BgerGr161
MQSSDGNSSVPERRVFYYMSRLVGPVPFCLKQSIEKTNIRLIIPEIVNISAHLWILFLTSTASI
GLFWSSRREVVGKVNDPGVIFLYRFNQPITFLVFLPILFVHNILKRRQSEELMDVFTKIHV
MPRRGHEALKKARHILIAVFLVLHAVFFVFESWLWRWVTSFYYELILRFFKFIGMVIVLFY
CEMVIYLRSKLCELRLALKTINANESHTISKTFIANSSSNTSKILAIPYNFKRRNIEGLLQMR
KDYLKVYKVCQLINSVYGFTVLLLVASNCTDLIMLYYELVFTCYSKVCRLQVFTPRHLLFVA
YSSFHHVVILLALMYSAQSTVEQAKKLFHNLQKKVLDDSLDYEQLEQLELFSNQLLHNKM
EFKTVAFSLNLSFLFHLVTTICSYGLVLYQFKH
>BgerGr162
MPLRKEYKKQSFLYKITDTASFSIVPINIGNSIILRTSPIPNVSDVLWIVILFLSALLGLVWTY
SRSRVGKGQDPGFIVTYNVCLPTNFVVTLLILINHNVVKRKQSRKIMDMFLKFERGPSQGV
RQKGIHQVLKLIGLALHILCLVIEAWYWRDVASIYYELLLRFFKFLHTLIMVQHSEMALFIK
RRLSEIRTSLSPVKSKFAQNLSMTVCEVKPIVKEKVNLGDKSTEAVNAGEILKLRHEYCRIY
TSVQVINSVYGFTMLLLILRNCVDLIDNLYTGFAIASGSKLHGDRYFIGDRILLILSWFTNVV
VLLYGVTYSSHLAAEEGKLLDDQVQERLLQYPIASEVKEQLKFFSNQLLHNKVEFFAVGFC
LNPSMFCTLLTSICTYVIVLVQFSKS
>BgerGr163
MFGISKPQFTAVSTYTLNYYLFKITGTQPFSITPMFIKDNEIIKDHLLSKIVTLIWSCTLMCL
AIVGIVWTIFRKSVRDDDPGYVVIYRICLPINFISSIIIFFIHNLFKRKDTTKIMTLFLKYELET
PNYNHNTCGRTQIYKCIAICIHLLLLIQESWYWRVMESLYYELLLRFFKFIQSLIMVQYIEIIL
FLKYRLRRLTELLSSNNYIVKNEPKLTLFRVEQGETSEALKIFNLRQEYSEIFAAVQLINSLY
GFTIWLTIIRNSIDVISSIHSWGIDVISRHMSYFKESFVGEELLVLTVWLFNDVAILFSLLWIC
DSVTAEANKLLHVIQQKLLDYTIAFDKEKQLKMFMLQVSQNKTQFSAMGFTLNLSFLCTI
LASIFTYVIVIFQSN
>BgerGr164P
MMGLIKLNFPAISPYTLHYYLFKLTGMESFSIRSTCSNNKDLVYTSQDVISFIWKLFLICLAL
FGLLWTYYIDYKGMGDNYAAFVVSFRITFPLYFSISLIIMVVFGLLKRKEITKLLDLFLKYEV
ERGFKYPSPSGKTQILKLVAIGVHLLLLLQECWYWRHTZCILYELLFRMFKFIQSLNMIQYT

EMVFFFRNILRHVEKSILISPNNLYNTSDCKVSFLVTELTVEKKHTCKSTEILKLRIKYSEIF
YAVQLINSLYGFSIWLLIIRCSISCINALQSFGVVILRREVYFDESFAGEAAILFSIWIVNMMVI
LFVMLFSCDSIAQEGKKLLSKINYRLLEQNMSLEEERQMKLFSLQVSQNQVQFTAMGFTL
NLSFLCTLLASMVTYVTVIIQLYH
>BgerGr165
MEYLVENKMFSISKQYIKNWSNWKNHFYLFKITGRAPFSICTTSEKNTEVLKVSPVPSFST
IMWTCLLICAATIGQGWTYIRKTTSGDDPAFYFTYKFRLPANFFVSLCVIFIHNLAKRKMLI
KLMKLLIDGLMGPDKGKLAKIELKQMVNIVTIVLYLLILIFESWYWRVVESVYYEALLRFF
NFIQTLIMFHYSEVVLLFRRRLCEIRKSLTRETIEETGKLEIKFLHVKSAEFLNISAKQTYTR
SEANEILKCRKLYSNIYEAVQLTNSLYGFTMLLLILRMSVDLISYIHNLFGVSKGTRTYYDLV
FKGDGIIMVVITICIILTILFSTIYASHTAMEEGTRLIAEVQKRLLEKSTTIEEENQLKLFALQ
MSQNKIKLSVMGFTLNLSLLCTGLTSIFTYVILLVQLDIR
>BgerGr166P
MFKFPKHNFLKWPQWKLHFYLFQLTGSAPFSVVKISEKNKEILKVSPLPNIISVIWTCSLIF
ATGLGLVWTWLRKTHDESNNPAHFLSYKLRLPANFVISLCIIFNHNIIKRKLSMKLMNRFL
KCYMTCPEENMNHIELKQIFNCIGIFLYILILIFESWYWRKLESLYYEMLLRFFSLIHTFIIIQ
YNEIVLLFRRKLQNLRRLASSKTMNAKQYLNITFLKVKSASIVNSVSPKVTTTNIHAEEILK
HRRTYSDIYEAVNLINSAYGFTIMSLIIKLCVDLICYVHHFFNISQGKNLYYDVFIVGDKIMLI
VIPISSMLIILFSAVYASSSTMEEYKILVDEIQKRLLEQDLTTEEKNQLKLFASQMSRNEIKF
FALGIFXFLCTWITSIFTYVVVLIQFDKQ
>BgerGr167
MLRQKNSSPFSRHAYKTLIYLSKLTGTVPCSILSSNSKRKSNFTTSRITKIAEIIWISFLFFSSL
VGTVCTYMGNGEFANDPGYIFTYNLFLSSNFIISTVIIIIHNIVFRKESTRILEIFLKYEQRNFE
RNIPKSTIKNILILVGVIIQPLQLLLECLYWRTIFNMPYKILVRLFKFQHTVILIQHSLITCFFK
RRLAKARASLQPSKLKTSEVCLLQLKSSDNDKLWHLNREITDVLNTRLKYSKIYSSVQMC
NSAYGFTMLLLILGHSIELIDILYKCFAIFAGSEFYTDRYFKGDRSLILTTRGLNVIAVLFTVI
YCSHLASEEGIHLDDDVQKMLLQYPMQLEKRKQLELFSVQLSHNTIRFSAMGVSLSFNFLC
TLLTSIFSYVIVMVQFRH
>BgerGr168
MSSIDEYKFILFLFKLSGTSPSSITITTNTKQRLIIKRLLEIADIAWVCFLVCFAILGIVWTCLR
NRMVVADPGYIFTYNLCLPANFIISLTLIILHYVINRNQSIRMMEVLLRYLAVRGDGSAQKS
YMKTFCTIIGITLHLIYLLLESWYWRNVFTIYYEVLLRLFKFLHTIIIVQHSVITYFFKYRLSK
MRHSLLSNNNIPTGNLKHILFEVKPARHDAKTPDESSSNEEIAVLKTRLEYSDTYNYVQLF
NSVYGFTIFMLIAGLCIELIDNMYKLITVFSGSAFYVDKYFKGDRILVLTAWVINILTILFVVI
YSSHSATSEGKKLNDDVQIMLLQHPIDVGKRKQLELFTTQLSNNKIEFSAVGFSLNFPLLCT
LLTSIFTYVIVMVQLRK
>BgerGr169
MSSIDEYKFILFLFKLSGTSPSSITITTNTKQRLIIKRLLEIADIAWVCFLVCFAILGIVWTCLR
NRMVVADPGYIFTYNLCLPANFIISLTLIILHYVINRNQSIRMMEVLLRYLAVRSDVSAQKS
YVKTFCTIIGITLHLIYLLLESWYWRNVFTIYYEVLLRLFKFLHTIIIVQHSVITYFFKYRLSK
MRHSLLSNNNIPTGNLKHILFEVKPARHDAKTPDESSSNEEIAVLKTRLEYSDTYNYVQLF
NSVYGFTIFMLIAGLCIELIDNMYKLITVFSGSAFYVDKYFKGDRILVLTAWVINILTILFVVI
YSSHSATSEGKKLNDDVQIMLLQHPIDVGKRKQLELFTTQLSNNKIEFSAVGFSLNFPLLCT
LLTSIFTYVIVMVQLRK
>BgerGr170

MKKSINSVQNKDGWRNIFYLSRIIGMSTFTLQSNTNQIILNTKQKFNILGYAWAVFLFFSFL
VGLLWTVQKRAPFVLDRGVFVNQFVVIPGSLLAAVVILPLHNVFLKKQYLQLMETLMRLG
FQYPNGRKLKNKIKEYLAVLFLLLCILYLIWESYYWWSYTGSLCYELLFRFCKFIHLLTVCH
YIEITFFIRQILSEIIQSLPAGDGTLKNKQKPEIKKMTKKDGPKIFTIYPVLPTCDSGTVKTSE
PVIPKYRKERKVEIIYELRQDYFNLYNIVELLNSVYGCTMIILISLFLANMVIAVYTSFGIFCNI
FEFEQYANGVVGLQTVVWITIFVSSLSVVAYATNSVIQQKLLGVEVQERMFHQTLSVEEMR
QLELFSDQLYQNEVEFTAVGFPLNHCFLCTLLASSFSYGIILIQMKQ
>BgerGr171
MQGNFHRSVFNQFKPLFYLSKILGLAPFILQGKTVNTEVKKLWVYLVWTILFMTYTSGAII
YIISTGIFVHQISLSAILTQVFNWPLNFVACSIAVIQLFAKRKTLAQALELLENSNNILENVTS
AEKFATMELMTFSVFGLSVCFDAYLKYSNNFAFQLTYRMPYLLNFLVILQFSFFAKITSQQ
LSAISKDLILNLKHKEEEELSSFRSYLKFSSKYYKVLTNDAEDILNKRKAYNCIYKSSKLLNS
VYGLPLTLIILRHASGLVTMICSLINIMKGNFHGRRYPTLSPPEYCILLLYHSTFSIYTLFHVS
YTSQIVKSKSIEIQDVIQEHMLMPSSRSPNVQDQLQLFSSQVSSSKIEFICLWVLKVDMSLLF
TVLASVMTYIIVLFQMQPN
>BgerGr172
MNHSSRQTISESRHRTLFYLFKLTCTGPSSLNSLNGNNKQSSRYIFIAELIWVCALSCIAVFG
FVYTYTRDGAQLESDPSHIFTYKIILPGVFILPFLVFLNHNLIKRKGSQKIIEILKSFDLEDCG
TRTKQDEAFDILTFVGIICHFLFLVFESWYWKNASSLCYESLLRMYKFITLLIMIQYSEMTK
IIKMKLKILRNSVKVLYCAPSYGNNSVKEITGNSAAVYMKISEIQRLRKKFFQIVSVVHRINS
VYGFTVFIMVIEISLELIENLYNGFRILNRSSTYDKYNLILSRIPLIMYSAVYFTTVLFTVNFSI
QSVQDERRKLGNKIQKVILNPYLSVKEEKQLKKFAILLLHNKIEFTAIGFSLNLSFLCKLLTS
IFTYVVVLVQFKV
>BgerGr173
MTKIEDKRVTNNIIVVSSKENNIPAREYNILKRKYKVIYYMFLLAGIGSFSLQNNSSMKSEEI
ISTDLVSNGLSIIWMLFSTISAVISFVWTCLRMEIDSTDPGIIVTFYLLLPSTVLVTLFIILTHN
FIKRQKSVKLMQMIMKLDLQSEETFVARDNVRFILKLLLVILHTIVLLMESWYWRKDVSL
VYELLWRFCKFNQMIILILYDDIAVFIKHKLSLIYKALADDVQSDNTVNVTHTSCAKTCLPK
PNTNTSTLNISNAKIIDIIVVSREKYFEVYNTGQVINSVFGLSMFLLIMNMSVDLISNVFTIFS
TAGQKKGYSIEQYGLIILCIPIYWSSHFFLLLFILTHSSQSVCDEVINIGDVIQKRLLDRSSGM
NVKQELNLFASQLNYNKVELHAFGFTLNSSFLCTFLTSICTYVLVLVQFR
>BgerGr174
MWKHRKCTTAILKDLLPILFMSRIMGLFPVSFSINKQTQQSYVDASLKGNISGILCSVLMCF
LMISGITTTVFRFYVSDLEDPGEFVNNAFCQPLNFVVGLVSIILSLTINRKKNADIISRLCVID
QRIMQHLKPGDIKVSKCNFNRSSLMTLNLIVVPFLWCDIIFWGSRYSYIVSFLLRTSHFLDM
VMIQQFCKLVQTIKVSLGKIVYLMEEDESFNKKENVLERIHVTKFEGIANVLPTEVQKYTES
KSIINIRVSSPSSEAIKSINILQYRQIYKEIYDTVLLINSAYGVQVLLEFIRQTIGFTTNFYQLLK
MLLDEEEHPDRPFYCILSKSLWCGMFLATVLCMTATCHLAMKETRKIMDQIQKHLLRHPI
EKGVFQQLQLFSGQIAQNQINFTAFWFFVVDMSLLCTFLASAITYVVILIQFKE
>BgerGr175
MVVVKLVPKIFDTPSPKSNPENSKMHTSYTALKPLYYLSKLVGLAPYRYTQGKLNSDFSSN
KFNTGWALLIINFIMGSHLYWILRFIQRPHDTIGDVIHSILLVPSVQLTALVSLILSITVNKSL
ILQFFRIIEKVDTDLRQNVTYSKLNLCCLVHCGFIILAVVPFFVYDVYLWGDTLGHVYVGLE
GVSFIIKQLVIFQFVHLMWLVKMQMSKLNEEFRIICSSMKFEQNFEDSMKIDRSSTSLGRLF
TRRQFPNRKVIQINLPISVCEYHPTLGNILKLRRLYFNIYNATKLLNLMFGLPLLLELVSSVL

YAIYCSYTAIETIMGSLQTDLRSMPPKQLIFCLCVFVIINVFHMAAIVFSCQATVQEEKLTSD
LVQEALLHIGTASDLLTQLQLFSQQLSINRLYVTALEMFKIDSKFFSMYIGCIVSYSN
>BgerGr176
MYTIPRKYRRQEVKPMLKPRSNYAISINTDLGMTVLNIFAKCVGLQSFSSRTHYKNPKPNA
NRFLRITEVIWCILMTLYMLIGFVINATSILYSSPSSNFEVVAFLISMPLGYTSCLIGLLAGQF
FHQDNIHKFVKKISDIDYYLLKSKKMLVYKQNSRSILFQLVGLALIAIPFYCLDYYFFSSEVL
WFYWHIQTIRFIREIINLQFVNTLSIVTRRFKFLNREISIRNSEFFQRDIVPKPEFGLTAPHV
FIVRQVFSYLVEVMYLINQIFGFHILLEVTYNFTNAVVSTYALVTVNFSSLETKDSKTEKSYS
WVYMCWILVSLGKIILISYRGHLASQEVNKFVHNVQKVLLRESSNKDTARQLKLLERQTA
NNSVTFSACGLFPVDFTLFYKFVASAATYFIVLVQLK
>BgerGr177
MNLLDVFRPMYLLSRFFALSPYIPVLNKISSEKKCCRFFHNLWTLIVLSIVTTGLIMCINNIH
MTLSNNPGKIVSHMFSTPINFISSIIGITIKSTVKRKSFFILVDKLQYIDEVLSVSNEEKMYEN
AKRKMVAELTIILVILVPFLCNDSYFFGRSSSFIYETFSRISVLINIIVVLQYIYIMRYLAHRLQ
LLNKQLSYEFASIYKNEHIHLQVNGLVNAWQPDKGPQQKPLQPYVIYPYTKLSGIHKLRVY
YNEIYEACQMINSVYGVSILLAFIYCFVSMVANTYFLFMDTVRNYETLQSKISSADYYSITHL
CWIGISLGKCIAICTSSQMVLEKASGLKDNLQKLQLFQPVKKEVLEDLQRFSYQLSENKIRF
TAFGFFSINLSLLYAFTASSITYVIILIQFK
>BgerGr178F
MWAQVSSKIYSDMKPLLIISKYLGLFPYTVVINNLTKEGGLDTRAKKNVLILIWTFMIFLL
MGFGLCDTFSQFKFIRWSTVSDTLNRKFCLPMFFTVTMISIFINSTFNKNKFPEILKMISNI
EKEIFRLRKDILFEARLKIFKDNLDIFVTVFIMIPYVIYETIIFGHGWMIIHSSFLKLSLLINIFS
ILQYCKFVYFIRRSLAAMNDIIIEVHELDSSKLSNQNEQYLHKTKLQRWISPAEDVILAVRR
LYLQICDVAHLLNKMYGLIVLLELALSFIVIITNVNTLTGIVKEKNIPHDGNRLAPIISNVGW
LVISTAITTVIIVNCQLTTWKSKELSSVICKLSLRMAPRSDSQRQLKMFGNQICYNAFEFSAF
WLFRLDSSLLCTIFASAVTYTAVLAQFN
>BgerGr179
MRLDILSEFKPVIKLCTYLGMFPYEIHKHFSSGDIIFDYSIKTNYCTVLRGTFIVLSALVMSII
GLVDRLQGIQNSISIILQNVCSLPLYFIECYVVIIMNATVNRKKMALFFTKLAEIDDVLNQFD
ISAVVKVKRLLSHIDIFALVCLVVPFVILDTFLWSTNSNVVYEIAFRYCFIINVSIVILYSNCAS
AIRKRLIFLSRIVKEHVKTSYLVTCMKVFNDLCGLVDTMNSIFGLQILLEILRNVTNIVSSIT
KILKDDIPEKNHYLYVVSLVSWNLIGIPVLAYLVIESHLTKQQVKILNRRVHEVALKYPLTIS
SSQHLILFSQQIHTCGLEFTALGLFKIDCSLLCKVIASVVTYVVVLSQFN
>BgerGr180F
MWAQVSSKIYSDMKPLLLISKYLGLFPYTVVINNLTKEGGLDTRAKKNVLILIWTFMIFLL
MGFGLCDTFSQFKFIRWSTVSDTLNRKFCLPMFFTVTMISIFINSTFNKKKFPEILKMISNI
EKEIFRLRKDVFFETTLKIFKDNLDIFVTVFIMIPYVIYETIIFGHGWMIIHSSFLKLSLLINIF
SILQYCKFVYFIRRSLAAMNDIIIEVHELDSSKLLNQNEQYLHKTKLQRWISPAEDVILAVR
RLYLQICDVAHLLNKMYGLIVLLELALSFIVIITNVNTLTGIVKEKNIQHDGNRLAPIISNVG
WLVISTGITTVIIVNCQLTTWKSKELSSVICKLSLRMAPRSDSQRQLKMFGNQICYNAFEFS
AFWLFRLDSSLLCTIFASAVTYTAVLAQFN
>BgerGr181
MRLDILSEFKPVIKLCTYLGMFPYEIHKHFSSGDIIFDYSIKTNYCTVLRGTFIVLSALVMSII
GLVDRLQGIQNSISIILQNVCSLPLYFIECYVVIIMNASVNRKKMALFFTKLAAIDDVLNAFD
ISAVVKVKRLLSHIDICALVCLVVPFVILDTFLWSTNSNVVYEIAFRYCFIINVSIVILYSNCAS
AIRKRLIFLSRIVEEHVKTSYLVTCMKVFNDLCGLVDIMNSIFGLQILLEILRNVTNIVSSITK

ILKDDIPEKNHYLYVVSLVSWNLIGIPVLAYLVIESHLTKQQVKILNRRVHEVALKYPLTISS
SQHLILFSQQIHTCGLEFTALGLFKIDCSLLCKVIASVVTYVVVLSQFN
>BgerGr182
MSIPAEDCLPDLKTNASPNNKNSPIYWDMKNLHNLFKLFGIVPCKLDKECFDSPENNTRY
FFWDRSVLWTFLIICMILTSFTSISIVNEVNVWHYVIFGSETLLATFQLVYVSLKRKGIWQY
CHWLKCADSILMEHAQSVPVSSLKWQYGLVKMCIRDSSILMEHAQSVPVSSLKWQYGLVI
LHLLILLLDSYSNNLNIFCSFILTIPYFLNMLFVLRYCNMVYFLQKRLTALKKVLALSNMLFS
ISTDSSRPTYLSDWKTVYDMRRVYSNIFKGVQHINCVYGVPIFLIIMRMSIHLSTSVILVASF
VAGKPNVENFTSRMSYKQYIGLELVWSVVSFTHLTAILCLNHITFSVSQVLIEIIQDRLLSQK
NKNQSVRQLKDFRYQINDNRIKFTTLWIVPLDLQLWFTVITSIVTSTIFSVQPKV
>BgerGr183
MSKFSNNLHKELRPLYYLSKVIGLAPFSFTRNQETGEYFIDTNLSTNVVGVIWTGMVFCV
MVGGFVYTKVRYQLYSIIHPGEAVNNAFSFPMNFIIAMVALINILLKGVKIPKLVKKLAAFD
EKLSKYRNDNCFMYADVIVVLVVFVLVSYDTLISEKGLAVLHDVIYRLAYLIGLVTVMQFC
KLVQLIQSRLTAIKKALSYILKLASLQNVKELGTGKDVESCNLGKTTDDTTIIQILHLRQAY
NSIYESTKLLTSIYGFPILLLLIRTSIALVTNVHGLIWSPVKTPANKEYFASLPVWIATFLGMI
VVVTVSCQMATLECKEVADRVQKLMLQESVTDKSLKQLKFFSMQLACNKIEFSAFWFFTI
DLSLLCTILASATTYIVILVQFKI
>BgerGr184
MSKTHPIMNFSKYSEMKNLRILYLLFGLAPYSFTEENTRKRLDIEILRVIWTFLVLVIMSIG
TIYVIIVPSPFTVPYAKILTIWWITYSAAVVHLFQIVIKRNEITVLYEHLQLAESILVQHLQVF
KRNSLHWQKVLIVVFILLLCVDSYVPERNFFFSLTFQLPDFVNMLLLLKYCNIVYCLRRRLE
ALEAVLSHKNLQISNLDDGLFISSDWRTVYDMRRVYSHLYKATQQINSVYGFPILVLIFVM
TSISAINMIFVLATLKFMVVFKIDLFSDFNAVKLTILLVVRAVEYFIKTSVLLFFNQLTKSRT
FKILDVIQDRLLLQAKSNESANQLTLFSRQIQDNKIIFLAFWVVALDLNLFSMMVVNIPYLII
VLYPDLRLSIGPTASNSNQIPDLPSNFSAFFSKPNE
>BgerGr185P
THKSVTIEDGLVDIRTEKQYCSIVRIVLTVILMLSILLYEITIKIHTILGAPSIVHLLITVPIIFVS
ASVAIIMNATINRTKSAKFFSKIFMIDNNLNRLCISGVREKEFHFDVIWLIFIIPFLVYDCSL
WSWNTNIPSILFEIVFRYCYAIDLAMMIIYCKFISIIRKRLIHLRRIIDKGVELRNVLSCMGTF
SKICELSGDINNMYGVQILLHTIRNFSLTITSISLLMSPAVEFNLQIKFDFVVSVLISFLTLTR
TVMQCHLTVQEMKILNNSVQNYLLKYPAKISAWQILPTFSQQISTSSLEFTAVGLFRVDNS
LLCTFLASVVTYVVVLSQLK
>BgerGr186
MPQKRISIYSDMKPLLTVSKCLGLFPYSIITNVISEERNINNSYRRNIIKVLWTLVVFGTMTF
GLCDTFSQFKFMRHVTVLDTLNKKLYLPMLFTVTLTAILMNFSFNKRKFPEIMKMISNID
KELDRLRKEYSFDEGNFYRCLVFIPIAAFFLILMPFYILESYIWGIDFLLVHSTLVRLCEFVNI
AVVVQYCAFVGFLGRRVNSLNKILSVLEKIDTSTIGRKYEGIQQNLTPPLSSSRSAATVLSLR
RLYLQICKVVQLLNQMYGLVILLELASRFIVIITNVTTLTSVLKSSRRKKMFPAFSLPFMSTT
AGLLISSGLIINIIAHCQLTTWKSKELSNKILRLSLKLPTKNSTQRQLKLFSCQIIHNSFEFSA
FGLFRLDASLFCRIFASAMTYTAVLAQLN
>BgerGr187
MEVKWLLLLCFIMKLFGVAPFSLRENFEVDCSFKSNYISITWAVLIAVTIILVQLIQIFNYRIL
SPFEELDVVTKITVPLLTLALVTALLMINCFRRNKFADIFSDLRYIDKVLFGYSNEHHYKIR
NIIICEILIYFFLSALVGCDSYAWVQWNKNVYLFELGYRVNSILIVPPFLFYFNAVCYIVHILY
VINNKINSLLSIGLINITEDIAFSRNSIRNFGRHSKLFLNTRYRGQTLLLDENIDKSFEITMLR

KIYSRLLSFSDGIHSVLGVYIMLETTKNFLSIVAHIYMLIIGMRIEYKPFLSSIGWILWYLGKQ
LALCFVCSMASSLIQNIQKKLQDILLLDEKEHLHYQIKHFSTQISRNKLVFSACGFYNINLQL
FHGYILSAASYLVILLQFREC
>BgerGr188
MSQDDRIRLNFRSLYYFSKLLGLSPYSFKSKTISSQLLVTTNVTANNIALFWSLCLFSVPIIT
LVQSVNELRFSDISRTNDLVVLAILGPITIITAAIIKGISIMNRKKVVNLFYYLSKLYPRILIFK
ESVWKIIFYTLRQPFYAIVTLYYSLFLGIGMGYWSSLYHNIAIQILQRLPKLEQFAIVLQFCEL
VLFIKRSLERMNEELFSFTTEQPLNEYPKYINNSAVNFKTVSIVCQPRKDKQNKYDSLMAT
VRLMRKQYRDINEAVNEIKSIFCTSIVIIVIKFAIDIVIFAYLVFVRDPYYADDHDLFIGMALH
ALGLVTAFLHVVYICLSCHMVNTEAERQHNAVHKLLLDVCIREDVYQELKMYSHQLVYSR
LEFSALGFTLEMGLLYTILASVFSYVIFLIQVKG
>BgerGr189
MSKRFYLHFRAIHYFSKLLGISVYSFRFNPVTHKQEIVTDISSNKCCFLWNLIIFCAVLGGFV
FTILRPQFSQSTPPDVFVTFNIELPACFITTLLLMIFSITTIRKKIWPMLMSLSKLSEGVITFQ
ESKLEILYFAIRHPLYIFYIFIYIPFLVYESYLWSGDSCIFYEMTLRTCEIVKLVTVFKFCSLVL
FIKGCLAEMNDDLNCSLTSKIVPHSGQKNDHQISHLEAAAWYTSDQNGIQLMKTILHHRK
RYKEIFEAVKQLNSVYGLSIIIIILQSTVDMVTSSYLVFVTNHPYAITDNETLRMAVLVIWM
VSSLICPIATTTCCHLAVNETKELHNGVQTMLLCYPVKEDALMQLFLLSNQIVNCKIQFGA
LGLMLDMRMLYTLLASVITYCIVMYQSIK
>BgerGr190
MAPKISDEKHFSLSFRTLYFISKLFGLSVYSFKRNPVTGKHDITIGVYSNIFGFVLWLAVFTS
GLAGFVQTILRPEFMNYSRPDIVSVYTISLPFNFLTCLVILLIAVTVGRIKMAALFKSLSKLA
DGFMTFEDTKREMLYFAITQPWCVIYVFIYIPYLIIESYLWSESTSMCYELTLRICRSVCILT
VIQFCLLVLFIKHSLVQLSLDLSSDLKEYTYNYIAIHVNTFPPFQAVAYNCPEEKNKGVTKIV
LKYRKRYNDICEAVEQISSVYGVPIITVILQSSVDLVTNCYLIFVQHNPVAMVENQQLRITLF
AIWMAMSLTCLVAITGCCQLAMGEVKKMCSTVQKLLLRYSMKEEVLSQLFLFWNELTHN
KTQFTALGLVLDIRMFYTVLASIVTYLIFMYHSR
>BgerGr191P
MARNTSKIKYFRLYFRSLYYISKFFGLAVYSFQRNSTTGKHYIIVGVSSNITGFIWWLVVFTS
VLSGFVLTMLRPEFLTNSEPDIVVMYSICLPSHFITCLXANGATVGRGKMVALFDSLSKLSE
GLTEFREPMWEVLYFSIRQPLFVIYIFIFIPFQIYASYFWSQSSSMFYEVILRSCRAISTLTVIQ
FWSVVLFIKDLLAQLNLDLNSSLKLATCNVPLVHVKTFSPLGVTYNFSEENCKGISTLTLKY
RKRYSDICEAVEQINSVFGIPTIILVLLYTVDVIVNCYLLFVHHDPVAMLENQHLRMILYSIR
QMLSLTCLVAITVCCQSAMDEVKKISSNVQKKLLLYPIKENVLSQLFLFSNQIMHNKIEFSA
IGLVFDIRMLYAVLASVVTYFIVMYQSRT
>BgerGr192
MAEKISKGKYFSLCFRTLYYISKFFGLAVYSFQRNPATGKYYIIVGVSSNISGFIWWLTVFTL
VLSGLVLTMLRPQFMTNSEPNTVAVYSISLPMYFITYLYLLVIGVTIGRGKMAALFNSLSKL
EKELMYFGNRKLEVLIFLIRQPLYGAYIFIYIPFLIYASYLWSESSSMFHELTLRTCRAISILT
VIQFCSVVLFIKNFLAKLNLDLDYNLQLKTCKVSLVRVNKFSSPEGVTYSYSEENGKGISTL
TLNYRKRYNDICEAVEHVNSVYGIPIIILVLLYTLDVIVNSYLIFVHHIAAAMFENHRLRLTM
FSISMMVSLTCLVAITVCCQLAMVEMKKISSNVQKKLLLYPIKENILTQLFLFSNQIVQRKIE
FSAVGIIFDIHMLYTVLASIVTYFIVMYQFRT
>BgerGr193
MTMKISKGKYFSLCFRTLYYISKFFGLAVYSFQRNPATGKYYIIVGVSSNISDFIWWLTVFT
LVLSGLVLTMLRPEFMTNSEPDIIAMYSICLPCHFITCLSLLIISATIGRGKMVALFNSLSKLE

KELIDFGNTKWEVLHFLIRQPFYGTYIFIYIPFLVYASYLWSQSSSMFYELTLRTCRAICALT
DIQFCSVVLFIKNFLAQLNLDLDSSLKLETCKVPLVHVNKFSPPEGVTYSYSEENGIGISTLT
LNYRKRYNDICEAVEQVNSVYGIPIIILVLLYTVDVIVNCYLIFVHHDPVAMLENHRLRSTM
YSIWMMVSLTCLVATTVCCQSAMVEVKKISSNVQKKLLLYPIKENILTQLFLFSNQIVRSKL
EFSAIGLVFDIRMLYTVLASVVTYFIVMYQSRS
>BgerGr194
MTMKISKGKYFSLCFRTLYYISKFFGLAVYSFQRNPATGKYYIIVGVSSNISDFIWWLTVFT
LVLSGLVLTMLRPEFMTNSEPDIIAMYSICLPCHFITCLSLLIISATIGRGKMVALFNSLSKLE
KELIDFGNTKWEVLHFLIRQPFYGTYIFIYIPFLVYASYLWSQSSSMFYELTLRTCRAICALT
DIQFCSVVLFIKNFLAQLNLDLDSSLKLETCKVPLVHVNKFSPPEGVTYSYSEENGIGISTLT
LNYRKRYNDICEAVEQVNSVYGIPIIILVLLYTVDVIVNCYLIFVHHDPVAMLENHRLRSTM
YSIWMMVSLTCLVATTVCCQSAMVEVKKISSNVQKKLLLYPIKENILTQLFLFSNQIVRSKL
EFSAIGLVFDIRMLYTVLASVVTYFIVMYQSRS
>BgerGr195P
MTEKISKWKHFSLCFRSLYYISKCFGLSVYSFQRNAVTGKHDLIVGIYSNIYSFVWWFVVFT
TISAGFVLTFLRPEFMDNYEPHMVAVYVISLPCDFLTCLLIMIISITSQRRKMGTLLKSLSKL
SAGFMNFDETKLEIICFCIRQPLYAGYIFIFIPLLIYLSYIWSSSSSFFYELMMRTCRAMSTLV
VLHFCLLVLFIRNSLVQLNLDLKSSLTVRNCSALSRHLNSSLLRSLPTSNTEYNSRIITRITIN
YRRRYNNISEAVEQVNSVFGVPIMILVLQXTSLMCLVAITVSCQLAMSEVKKMCNIVQKML
LRYSMKEDIVSHLFLFWNQLTHNKTQFGAIGLIFDVRMLYTLLASVATYFFVMYQSR
>BgerGr196
MSQETFFRLNYRTLFYFSKFLGLSPYSLRTNRTKAQYDIATDISSNIYGFIWSLIVFSFIATG
MILTFLRPEFMDFSSPDVFAVYTISLPTDFLVCLLIILTSITINRKKMKALLISLSKLDEGFFS
FEVSTWGILSFITKQPLYVLYLVVYTPFTIFECYLWSAGSGLFYELSLRVCRTISMVTLLNFC
SFVLFIKSCLAQMNFDMDTTLAIKTQNSHQTQTSSSPHITFLAKSVHNTISFETGKDTNKIF
TYRKRYSDICDAVGELNSVYGVSIIILIFQYTIDIVTSCYLIFVVDHSVAVINSQFLRRGIFLVW
LVCFLVCLFAVPICCHLTMLEVKKMSGKVQKMMILCPIQEDYLTQVYLLLKQLVHNRIEFS
AMGLTLELRMLYTLLASIFTYFIIMDQLRN
>BgerGr197
MSQETFFRLNYRTLFYFSKFLGLSPYSLRTNRTKAQYDIATDISSNIYGFIWSLIVFSFIATG
MILTFLRPEFMDFSSPDVFAVYTISLPTDFLVCLLIILTSITINRKKMKALLISLSKLDEGFFS
FEVSTWGILSFITKQPLYVLYLVVYTPFTIFECYLWSAGSGLFYELSLRVCRTISMVTLLNFC
SFVLFIKSCLAQMNFDMDTTLAIKTQNSHQTQTSSSPHITFLAKSVHNTISFETGKDTNKIF
TYRKRYSDICDAVGELNSVYGVSIIILIFQYTIDIVTSCYLIFVVDHSVAVINSQFLRRGIFLVW
LVCFLVCLFAVPICCHLTMLEVKKMSGKVQKMMILCPIQEDYLTQVYLLLKQLVHNRIEFS
AMGLTLELRMLYTLLASIFTYFIIMDQLRN
>BgerGr198
MSKPTLRRRHSDFYKLYHLAKIAGIASFSINPDYPSSSKTFMIWPLILFFTILVGCACTFFRE
EFSDNSDPGVIMNYKVTLPSNFIVALIIMIVHNTINRKKLLHLLLSIIKMDKVIPSKQPLSNE
TPKSIKHTLKHCFKLITVFIFICFNIIMLMFESWYWRLESNLVYESLVRMCKLLLMMIIVLH
CRFANFIKIKVYKINQSLGWNQDPNFGKKEYTSSASVSLVGSFESMQVKNIRRIRQKFYLIY
SLTQRINSVFAFSMLLLVVKYSIELIDTLYTVFMFQSYRVDLYDRYIDTGRIVLVGFWFFNIL
LSLFAVTYCSQSVVDEAQKLSDQVQERLLRNGLTSEEVKQLKLFANQLFHSNMELNAVGF
SLNLSFLCTLLTSICTYLIVLIQFKR
>BgerGr199

MFTMKIRYKIIYYMMKSAGLASFSIKNNKITNKETVSGEPFSNIGGIIWILISTILTLIGYIWT
CIRSEYNTTDAGYVVTFKVYIPSIFIVMLLIIAMHNLIKRGKSIKLMKMIIKLGLESNEIMDSK
EVLMCVFKLLMVIAHILFLSYEAWYWREDVALLYEMILRFIKFIEFLILVLHGEISVFIRHKL
SLINQSLKDQEINEGLDITNIYVKPKTVFGEKNCVFHSNLTDKILKLRQKYYTVYNIGQDVN
SVFGLSIMLLIMVDSIDLVGNLYAIVSISEGKKEYDLEKYGAVRLFIPVYYSIHILLILILVTYSS
ESVASEVKNMVDQIQKIMIQPSTNVVVKRELKSFTSQLFFNRLELNAFAFAINSSFLCTLLT
SICTYVIVLAQFR
>BgerGr200
MSNSFHIMQGNYHRSVFHQFKPLFYLSNFFGLAPFTLQKETVSAELKQIWLFVLWTFLTIT
FSSGFILYIMIKHNNLNKIPPSEILSQIFTWPLNFFSAIASVVQLFAKKKTLARVLQLLKISND
NLGNVATAQHFVQMELIYFTFYCFSLLFHAYISNKNNIVTEVVYRLPYLLNVLFVLQFCFFA
KITTLQLTTISSDLVLKLKNIEENTIKSYNNYFKFSSKYCTVLTNEAVDILNKRKVYNCIYKS
SKLLSSIYGLPLMLIIIRHGSTLIIMASAIIQMSDGHLKLRRCQNFSYAECSAILAFFILLSICIL
FHLSFTSQMAKNKANELQDVIQELILTSSRNPRVQDQLIQFSNQVSNCKIEFTCLWVLKVD
MQMLFTVLASVFTYIIVFIQMGS
>BgerGr201
MYNKETTMPNKCTMQRRYKTIYYLIKSAGLATFSLKNNKATNKETIYAEPISNKGAIAWIV
IITILSLVVFVWSCVRNEYNTNDAGYVVNFRLSIPSFSMVLLFIIPIHNLIKRKKSINLMKMI
MKLDLESNEVINCKRNVLIFAFKLVMGITHLLFLSVEAWYWRKDVALLYEIILRFCKFIELL
ILILHGEIAVFIRHKLSLIYESLKEEDINASLNIRNICVKPNILFEQQSCVFHTKLVDKILELRQ
KYFTVYNTGQAVNSVYGLSVLLLVIVESIDLVGNLYALVCISEGKKEYDLETFGVMRLFFPA
YYSIHIPMILIFVTYCSESVSNEVKNIVDQIQKTMLHRSTNVVVKRELKLFASQLFYNKLELS
ASALTLNYSFLCTLLTSICTYVIVLVQFK
>BgerGr202
MAIARPIINFGIYSNNKVFFCILKFVGLFPFTFSTDPITGIQKIDRPIKVYPNIGNLLWSAAVF
SFILGGFVSYSIALSSILNSSPSYVTTFLISLPLTNIMALLFILLNMTVNRARISKLVNKLEAIK
TELHKHTPQSHTAHTISSIECETIIMCGIVIFLGFDLWFCMNRMGFFAELGLRLSHFIQIVN
VVQYCKLTVFIRNSLRELNDTMQSYFIGEYNKQIQHKSRLPIKSVEKVVSELSRLTDFNDCG
TNNSNIQIDHELPQSTFLRKCRQLYYNIYNCVNCINSIYGLPILLELMRNTLATIANMFPVIG
ILTGTTEYAYRYGKDTPGYLILFTGWVLIFVLKQMTISAFCSKALVEAARTLDTIQRLLLSSS
LSENVIRQLERFSNQVILNKIRFSAFGFFNISLSTFSTFIVSSITYIVVLIQNK
>BgerGr203
MVITINYFEIELKVMDILWKVVGIIPFSVSNRSEYFIVKSIGRYLWIFVLVTAGVIGFVWCWL
RGINYNENAGSVYMYGLSLPAHFFVGLLIIFVHNVIKRNKSMQLMHLIQNTDLISTTEQDN
GCKIRHIFLVSWSLLNLSFLVYESWYWSWVTSIYYEMLMRMFIFLQFFIMIIYAETVFLVR
NKLFQINLNLSVSNVESHTHRSSSKKKIPVYQFKNDCGLFSVSDSYKNGEELEINYSKSVIL
HENTNNVYKMSNHFKNKICDNILSARKQYYGIYISVRLINSAFSLTMLFIILNQCLTHIHNM
YKVLQNLLLRDNFYHTGSRDEDLYILSIWSLNVLINLFIVTYSTNSTVGEAKKLSSIVQKRLL
CKSLHPMEVEQLQLFTAQLHHNKLEFNAVAFTLNLSFLCKVVASIFSYGLVLVEFRE
>BgerGr204
MRDIDRQIQMLQFVAKFVGIFPCSVKKNPKYILLSDIGRFLWVLLLVSAAIFGFVWSWFRE
VKFDEDVGSVFMYGLSLPANFLVCLFIIFVHNVVKRKESLKFMDVIKNTELILTKDQDMKS
KIRQILVLLWSFINLAFLFFESWYWGWVTSMYYEFLMRFFKFVQFIIMMFYGEIILIYRNKL
LNIHSFLSNIQSHKQQDSSGNFRNNSLKKTGDTFSISDHYYEEGFKLSENRISDSHNGILPVS
NDFKESLVTKILLARKGYNEIYTSVKLINSAYGLSMLFLILNHCFSLIYNTYSILAHLTLPINI

RPRFDVGFTSLLSVWSFSLLLSLFIVTHSTQSTINEAQKLNDTVQKLLLSHTSEDLEHLELF
AIQLFHNKLEFKAGAFILNLSFLCKVVASIFTYALVLVQFGTQ
>BgerGr205
MPGQVCSSVYVDFKPLAIISKWLGIFPYTIRKNKYNGERHIDNSARNNIFALLWTTVLSLIM
FFGLFDTISHFNFKGWGKVTDSVRKKFFHPMLLTITIVVIVTNSTFNKYKIPKVLKIISVIDK
QIHNLRKNNSSERKMNKCLRNIDTIILVFVIIPYLIYDGCFRNHEMMIITQILTKTAILINTGI
MLQYSKFVSFVRQRVAAICGIISDIQINGNDDKLIFDKSTAYISSVGRMIFILRRLYIQISDVV
HMLNVMYGFIVLLVISVLFGIFITNICLAIDMFKQKSAAELPVTIIISRTVSPIVSASLLTVTIM
QCQLTAWKTKELVNAILRLSLKLPSKNEAQTQLKGFYQQINFNSMEFTAFWLFRLDSSLL
CKIFASAVTYTIVLLQFN
>BgerGr206
MQVRVRSPVYSDIKPLAIICTVLGLFPYSINTGNSNEEGNIKRDTRALLWTGVLSLIMVSGF
FDLVSQQQSVKIKNISDTLNRRFSRPFFYIITVVSIAITSSFNKDKMPKLIKMIHDIDKGIYRL
KNDAKLHDKKFKNYIENVIVLFFVCTLTSILLCSANNLSDPLVILNYSALLLSSFVNTIFILLY
CKLVCFIRKRISLICHIVSNIQNDHLTTTRVDSHVFQISKTITSPPVIVTLEVRRLYVQICEAV
NMLNVMFGFIILLVIAIFLIFIIINFSLIIQISYQNISSDPSNYSSSKLITTLMWSALIINTILQCQ
LTSRKSKELTDAVCRVSLKLSAKNAAQKQLMQFTKQIYHHSIEFTAFWLFRLDSSLLCTIF
ASSVAYTVILVQLK
>BgerGr207
MQLHSSSPVYSEMKPFAIVCKLLGLFPYKIRINNYSMEGSIDIRARKNIVALAWTAVLSSLT
MFGLWESLSQFKSMKWENITDSLDKLCIFPLFLIITVLALLLNSMFNRHKIVEVMNIISTID
KEIRLLSNNMDLTDGGITKYVDIMFMLLLVIIFLPMFFLNVETWKNPFISTIPFIVSFFVNFA
IIMLYCKFVHFVRRRMIMICDIIYDIEEINSGRCDKKDSAIFNFPRMYRNSYTIVIITLRQVYA
QICDIVHMLNLMYGVIMLLVIAIFLVFFVTNVSLLIEILLEKSPSELTTSNFAKLITIIIWFIMI
SNVIVQCQLTTCKSKELIYALYRLSLNISPNNEAQKELLRFSQHIFHNAFEFTAFWLFRLDS
SLFCTIIASIVTYTAVLVQFN
>BgerGr208
MQTHISIEVYTDIKLLLGFCKFVGLFAYKICVNHFTGEGHIDTRIRRNFVSLLWTTVVFCGM
GFGLVCTFVNLELKNCTTLADTLNSTVCLPMTFAVTIVAIIVNVTCNKQKFSEILKMISNID
KEIHRLTRVMIMTEGKIKKCLFNFDVIIIAFVLTPFLVYDVMVWEQGWLIIHSSLLRISNFIN
YLAIFLYCRFVSFIQRRMSSLCKILSDIELHSSERGRTENLIYHCTTVDSCFSATKVIIALRRL
YTQIYDVVHLLNKMFGLIVLLTISLYYTLIITNVTTIMRFIQLFPIVLMPFISLVTFLVASMGL
ITYCIINCQLTVWKSLELDNIICELSLNVPAGSDMERQLRLFSNNIHKSSYSFTAFWLFRLD
SSLLCTIFASAVTYITVLVQFSSSSSA
>BgerGr209
MALDIVSDMKFVNILCRILGLAPYRMYKNYISGLVVVDFKFKNNYLVLVQSAVSVVIVLSILI
YSMIIRVPDFLSKLSEMVYIFLGWPILYLNVCLAFVMSAIFNKQKMILFFTKISEVDDKLNR
LSYDVRMEKNFHFDIVFLLFFVIPFCIFDSWIWSTDYNVSYEICFEYCFIIEFAMVIHYSKCV
SIIRKRLMCLSKTLDEKIISFQLTKCMDIFSGICEAAKVLDALYGFQIFSEIMANILYIISSIALL
LEQLKVDSKDGLHFFSIITWDVMNLGTLAYLVMHCHLTVEQVKNLNHAVHKIALKIPKPV
NTSQELALFSLQILTSNLQFTAFGLFRIDCAFLCNVITSLITYVVILSQFS
>BgerGr210
MSFDVISDLKWANRLCTVFGIHPNRIHKVYSSKNSAADFKIKTNYFAYMYSGIIFIVMLGFL
ISTVYYDMPKLITNFSIVTFGRMLHFIVPLAGIVLNATGNKYKFGEFLTKLSRIDQTLNKFY
QDGDSKKVRGQHFGTFVFIFFIVPFFVFDLCTSLMRHVEFYSVGVRFCYVLEAGIVLPYCSC
VAIIGKRLKYLSSALNGHIQVYQLTSLRNSFVEICEATDLVNSMYSFTILMEIMNLIIWLING

TSILLKFTSYINNIPMLSILITWYGIVLVTMVYIVVHCHLTVQELKSVKHAVHKLGLNQQLM
HASSQQLTFFVQQISTCDIGFTAFGLFNIDCSLLCIVLTAVFTYAIALSQFN
>BgerGr211
MQQDLKIPTPLSTSRQKWKNPSPKPTDIYDVIKPLYYVSNIVGLAPFSFAEHPDRILGTPGK
KLWSAVVAIAQLVSFVVVAMWSFMEDYPQYSMTVVVPDALAVFLQYLSCLSALCHGVLA
NGGNICRLLQKMALIDSLQPCVQTGYVTTRRWLWLELVALIGVLVAFYTYNVNVWGTGIT
RIYYALQNIANFSTIVFILQFIHVMIFLMHRASILNDALEKQQAHNTSPEIMRKLRSIHIELC
ELATMTNAVYGFTLLVAITHQFVFLVSSLHYSLSGMADIDKIQSIYEILSDTVWSVIFIFTML
ALTGSCQWAGDETDRAADYLQKMLVNNSRDGDMALELELFSMHLIGNKVEFTACGFFAV
DFSLLCSITGAITTYLVILFQFK
>BgerGr212
MLEPIWKPLAVMNRGPSTSPESYVPFAELLAALWPLYCVSKILGLAPFSVEHNSTFLKVHF
GGTLDSLWSLLMCLLVSLGGVFSLVWSALHDYPKLKPSVVITDALSVFLQFSSSLVALVYAL
ANRTHLETLLSKLREVSLSNVVDHDEIYRRSGKALKMHLTAKMVSLSLLFSMAYYLLGAH
DGWIMLVPKHVATYINATLVFQFVTMTLLLKRTFKGLNELLEEAMHHARGRKESVAESR
QWLTPILVATPHKALAFVDDSHVLFRDVRGLHSQLHEVSEMVNAMFGLPLCVCLAADFM
SLISSLHIPIGLLVAGVEAQGKKRSFSILLAGVILSWALLFLAKMVLVSSCGQRTSDEANRTV
VVVHRLLLNQNVSSSSDELRDFALQLANNRVEFSACRFFSVNHSFLCGILGATTTYLVILLQ
FS
>BgerGr213
MAVSNHIYSSELKILYYLTGTFGLTNFSFSKTEVYFPVFKLIWTLFCLSVNLLGVVMYSLFIL
TEEFFICPLKIFIPYRLNYLFMHFTVVSSYIICGIMKRRQLLKIMEDIQEIDENFSQREISQMR
NTTRKFVLSLGTFVIIMLVTTEPVYLIPECPNFTCLCFSLILDSFTRMINSLSLVNVIAIVVAI
RERFETINKRLSDHCCQIENCSNCIARINRQGTVDCCSSLSGREVRSFKLLYNRLFTICQSLM
QCYGVFMFCAVMWIFLCSVSVSYLAIFLSQRNIISHFNVIQIFLWSLYLMLLMLVLNISCHK
TVEVSRATIFHIQNLLLHRILPNETEDELTHMTSLIFGMRLDFSVCGLFSLNLPFFTSLVGVI
ISHFVLLCQIQ
>BgerGr214
MTDFNNIMLTLGVKLIFYMSQAFGITNFMLDQTKISLSYLRLCSTIVYVFIAISVPAICFIFTV
VDHCGTISSKFCIADLIYVLARYLSYIAYLICCGICKRQQMARIVEEIREIDGTFLSEKERFLV
QNKLNKCSLLFVIYIILANVSDDMTYVISNCKISSLDCINAIQECISGFINSLIISNIVAMVIALR
ERLKIVNKRLIGHLQINEGYSKATMDLMTVRCLFKENRQISVTHSYCSGREVRSLRFIYGKL
NAISQEVSSLYGIPLLFSIFWILMSAVDTFYSGTYYFGTEEGYMYVVMYIAWSFYCISLLVIL
TASCHSTVEASRDLAFNAQLLLLQSNLRIEAKDELDAMILQMSTLKIEFTVCGMFSLNLPF
LSRMLGVIVSYIIVMSQLQ
>BgerGr215
MTESSANFISAMKPVFFISQVFGVINISLDKNTQNFSRFKLYLTGCFALLMIFGTIIYYSTIIS
NNIQRYPLKLIVAGFLFFSSSYFSSFISLVYTGMYKRRKLAKIIDELREINNSLLQKTQAPDV
YSHLQKLCIFLPIYVVVEIFILAALYVTDGCKTFTFTCVHFLILDISGMVILFTIINFCSFVLVIK
ANLKIMNKILRFHGNTYRINKGKSMEFNQRISIFVPAGCLTVQDLRTLRTLYARLYVVSQE
LTCCYGLPLLGAILWIFLDSIVCFYFTSLYSELQFGVNTALVRNGVCCASLLSFMVILTCSCH
VTVDEFCHTVFQVENLLLRTEFGNEFREELKEMASCLGRLKIEFSVCGFFTLNNSFLCAFIG
VICSYVVIMLQID
>BgerGr216
MADGHNIIVTVGIKLIFYLSQIFGIINFTLDKNKVSFPFLRLVITIVYTCVVVFWSLSSLIYSIP
FYCVGYQLKLCIADSFYLISCYLAYIVYLISCNIYKRQQMARIIEEIRKIDEILFLSERERFLLQS

KLNKFSFIFATYIIFANLLDNVIYIISNCKISSLDCTIVIQETICALMNSLIIANIVAMVIALRQR
LKIVNKRLIGHLQINEGHSKATMDLMIVRCFFKGNRQIRVTRSLWFGREVRCLRFVYEKLC
TISHEFSSLYGISLLFSIFWILMSAVDTFYSGVYYFGSEEGYVYVVVIYIAWSIYCISLLVILTAN
CHSTVEATRDIAFSAQLLLLQSNLRIEAKEELDALILQLSTVKMEFTVCGMFSLNLPFLFRM
LGVIITYVIVMSQLH
>BgerGr217
MSNTNTNFITAMKPVFYLSQVFGFTIQSSESNILKLRLKQIWTLLCILLICLGVVIYSSMLYE
DFFKMPLKVFITYCLSFYSVYLTSIISLIYSGYFRRKQIAKIVKELSVVCELLSCQKHSSKQLR
NLKLLCWFLEFYLMVQTLGVAMWYMLETCKSYTKGCFKMFMYGFSVDVTALMMINFST
FVLVIRANLKLINQILFDLDHQCEKFVDSATKWPVIFVNAKFSVEELNNLKNSYARLCAVS
GSLSSCYGVPLLGAIIWIVLNSLVSFYTATTFDLLEFGAFTMFIRTLCWYITGSSFVIILSTCC
QTTANERNKAILQVHTLLLRPNLNDEFSNELKDFGYYLQNLKVEFPVCGFFKLNHSFLSAI
LGFISSYIIIMLQIK
>BgerGr218
MKPVFHLSQVLGQSNETNAFRIRLKQMWAVLRIVFVCLGLVIYSTKVYEDFSNSIFKLFITR
CLSFYSVYLTAMLSLIYSEYFRRRQIAKIVKELSEIWDMLICQTNASKLLRNFRKLRYVLQM
CLLLVTTGLCIWFMTVQFKSYNYFIIVSIYAFSSYIIAEMIINFSTFVLAIWVNLKVINQILRD
LDHQCDNFDKLRSMPFQNWPKIKQRTTKYSVDELKILKMSYTKLYAVSETLTSTYGVPLL
GATIWIVMNSLVTFYAITTIHLVETRIYTPLIRMVCWYLNVWCFVIILCTCCQVTANERNK
MIVRLHSLILKTKFCDEFTHELKDIVSYLKNLKLEFSICGFFRLNHSFLSAFLGFICSYIIIMLQ
IN
>BgerGr219
MSKTNTNFITALNPHFYLSQFFGYTRLEIETNASKNRIKQTWTVLIMLLITISPLKYTTTIYE
DFCTMHLKVFIARTLCYYTLYTTAMLSLIYSEYYKRGQIAKIIDEVSDICDLLISQTDASKLL
RNLKSLCWFMQIYITLITMGIMIWYVLEQCKSCMDLLMLVSYGFSSHAIVQMIINFSSFVLV
IRTNLKVMNQILCDLEHQCGAFDKLGSITFQHWPMIKKKTNISVDELHNLKISYTRLYAAT
ESLTSSYGAPLLAAATWIVLNSLVNFYAAITFPLSDARTYTQVIRITFWYINVWGFVAVLCT
SCQVTVNERNKIFLQVHNLLLKSKFSDEVTHELKNIIYYLKNLKLEFSVCGFFKLNHSFLSA
FLGFICSYIIIMLQIN
>BgerGr220
MTMKSSKFSSAMKPVFNVSQIVGFTCTSKEKTVKRKKLYWTLFCCLLITLGPVLYSTIIKQ
NYESLPFKLFVTYLSYLLTGYLTSFTSLMYTSVYQRRNMSKIIQELTEITDSLLSRQETSKIQ
TNLQRFCKFFTIYVLLIACLISGWSVCHSCTVPGRECLHYVIHSFVINVISLTILKFTAFVMVI
YANLKVINGTLSLYSPKYNNLEVIITRYHHTGIKNVTPVFTLSADDLQYVTKMYIRLFIATS
KLSKCYGIPLLLTTLWVFLNSIVSFYFGTTYRDLELNVYEALTANVIWCVCVLSFMFIMNSC
CHLTEHERSVAIVKLHNLLLQSNTADQLRVELKETIYYLRNLKLEFSICGFFTLNHKFLCTF
MGVIFSYIIIMFQLN
>BgerGr221
MPPQSGNIFFDTMKPVFYISQIFGLVNFSLDKRIRVHWSKIKVYWTILWILVLVFGLGLYTT
ITPLDYSRYPLKLFIAYILHVQTKYYTAFASLIYTTIFKRKTFAQINRDLAEISDILLSKVEAK
TLQRNLRILCIFLPMSAGLFITVVVLWYLIQSSEPFLMANLHFVIQAIAGLIDCLMIFSFSTYA
LFIRANLKVINKILCSYKGLNLTKRKLSIHAQLNILPVVNLSVAELRNINMMYARLHAVSKS
LASCYGLSLFLATIWIFLNSIVSFYFVTYYYELSMKNYLTLTRHILWCVSILSVIIIMSIICHQT
VEESSTTVSHVQNLILESANSYEFKKVLKEMARYLRSSKIEFSVCGFFNVNHQFLSAFFGVI
CSYIALILERS
>BgerGr222

MIKSTNIVSSIEPVFKISQFLGLTNIHQQRTNTVFYNVKLLQTVFLFSMCVISPILHFQFIPTK
KMDTNTLKIPITYYLCHVSGYFSSAVSIIFLGILKKQQLVEIMEDLNEMDTFLISENTLSKM
DIQLRKVLILLLAYVTFMNIISDLLYFSENCHCFSWTCSLSIIDAIATFYSMLMIASYISLVLAI
KQKLQIIAQSISNLGHQFQIPQKDASNTKLFTVGLTTRNYNPSREVRLLRIMYTDLYKVSQT
LASSFGFPMLVTTSWLIITIIQAFYYSAYGFGNLQTDYKSLLESTIWSGFCATLIYIINLSCHM
TVEERKEIFYQIQTLLLNSNIESKTEIELKDMMIQVKNLNIEFTICGLFKLDLPFLGTVLGLV
VSYIIIMYQFK
>BgerGr223
MARNLISSMKPVFVVTQMFGLSNFSFKKTKLHFPRVLLIWTLLCSVCPVVNLSLYFFRFVS
VYYDYYPFKLATAYTIHNMFNALTCFTAYIYPGIIKRRQLAEVFAEIAEIDALLLSHRRGQE
MHRKQKKYCLFLLAYNFLSIGGSHIWHACLICQPFSFMCVFVLFNSESSIANSFLIIYFVTIV
AAIKRRLQFINALFRFYGDELKDPTNNIASAMKQNTRCRGIQVSCLTDQEVVKLRAACGRL
YGVTQSLTSIFGMPVLLATIWVTLNSVLAFYFAIFFTQAGSSNYSRLMLILMWCFGTLSLMI
VLAATCHNTVHEYYEILYNVEYLIVQPNIPFAVKRKLKDMCIVFSDLKMEFSACGFFVYNL
PFLTTFLGVIVSYIVVMVQMK
>BgerGr224
MTCSEMTENSNTFLTSMKPVFYISQILALTNFSIDRKHVTFPKYKTLWTILFILVGVIIPILS
CVYETSIILNTSTTLKLKISHYFLLFFKYFAYLVSLIFSGMYKRRELATILKEFHLVDTALMS
KLGYAPESKVKLLSVLLLVYLFAIQASNSVIYLVIACKSLSLICIHKIIFNYNSFVMMIMIVIYV
AIVLAMRERYKTINTTFVDFLTKSDHYCYSTNSGTRKLSVPTPKFITKQDLKQLRVVHRKL
FVISRSLTSGYGLPIFASTFWIFQSCVQCFYASIYSYQKGEGFILVVDLALWVLFCVSLIFALT
LSCQMTVDECGMTRFHIQTLLLEESLSSELKEELKDIRSQLVDMKLEFTVCRMFRLDLSFL
SSFLGVICSYIIIMCQFE
>BgerGr225

MFRTRTHPSSHKKSSELQAYFISTTKPVFVASQVFGLTNFSFTRNVISFPTVRLLWTLICLA
ILVIGVAFATLNSLDYEVSADYPAEVFITEVIYIYSSLFTAFLGLLFCGICKRQQLRFIIEDFVQ
IDALLSNFERFQDKSVFNKLYCFALIYSFAGNIIDAVLYIPDTCDSWDVNCIKTILEVFSGFIN
SKIIIVYISLVLAVQERLQIINKRLFYYRGICEMCTQDFFIVTDIIPKVRQRIFQHNPFPAHEV
RIMKLVYAKLHNITLFLSSCFGFPIVSVTFWILVSIIDTFYYSLSAIQGMDTTVIAILYNVIWS
LCCIGFLFIYTVICHVTVSESHKVLFNIQALLLNTSLSKETLEELKEMSSEVNNLKLEFSASG
MFNVDLPFLCTVLGAVSTYVLLMYQMK
>BgerGr226
MPLQGSDLISALKPHLYVSQIFGLTNFSLVKNTLKPSTFRLCWSVIITFVISICLVRRSMSILD
IDTSSKPLKEFICYYMYSLCGFLTAVTSLLYLGISKRQKLASIFDGFFETYVSPTNENESSEQF
GKLKKLCLLLLFYIIAVNAITDGIYFAAKCSSSACLCLDVFTSTLPPFLNSLMVVNYVFAILT
MRVKLKTINNSLLAYITKLQDKKLPILESTILFFEQKMITTTYMPKQEIHSLMLAYERLYEV
SQTITTCYGLPLLGTVSWIVIGVVETFHRTAHRLQNETYDLAMILETICWTLYCVSLIFILAI
SCHVTMDESCTTMFRVQTLLWQSNLLSGARKVLKDMRYQLHYLKIEFPVCGLFNMDLPF
VCTILGLVASYIIIMSQIK
>BgerGr227
MMLNFSSKFYSSTKVLHFVSRVFGFVTDSSYMNRKLFGILRLFWAVVALLNLILYAISIVSK
EQTGLSLKTLIAYVSFIVCNSLTTISSVICYSKIQRIAKLYNCIQVIDALLIQVGVPRTHINGIE
HGFILVVTVFMANVVSVILFVRQGCVVQCGIWGCLHVVMDFLCTFVNSTMIVNYIFLVFDI
QGRLKIVNNLLIGDGRRCQTENPVPVGIPVVNFRSICNVYFLRKVRLLRLVYFRLYAFSQDL
MSLFGFPMLNATLWIILSCIDAIYYIVIYPGVSLEISVDLVFLTFWILFYGSLLYILTFACDRT

VAESEATASNVQMLLLGNNLDSSVEKELKKMCHLQDCLKMTFSACGVFCFNLPFFCQVLG
TLISYIVVICQIK
>BgerGr228
MFGFTRSCVRFMVFHPFMILLHAALYPFGFINYFLLLVNKYNGNFSLKMFVSDILYSACSF
MTSFMYFVFTVIHKRKLRSILIADLQEIDDLLMVAPGSFDVSCTKNKVCLTMFIYFVVANIG
IGVKPIVQECYYLRLWCVYTIQDIVCGCTISLMVFNFITVVLGLRERLKFIKELLKDHDEKEK
LHDRNKLISSLLSDDSTVGCAYLSESEVRKFRLVYGKLFAVSRTLTSCYSVPLFFATSTTFFS
SVHTFYFSTYYLLEENAAYIELLPDVLWSLYCCTLMILLTFSCHLTVNESLELSIHVESLLLR
ANLSKSIKKELAGFGSQLTKLKMSFQVCGVFNLNLSFLSSLLGIIISYILVMCQIV
>BgerGr229
MPHIRHGMHLKFAMKPLLCVSKVFGVANFSGNKNVACTKIFELVWTFLLMFINASGPIMY
FIFIAPQGCNIYPLKLCISHTGYTIASYSMSFLYLVLCRVKKRYEMGRIFEAIQQVDIMFLSAE
ESVVIQTKTKFYCLAVLIFISLTSSLNGVFFIVSIHKSLTWCCVNTIQEIISAFMNSLIIVNFAT
VVFGIGQRFKVINNRLYSQYQNTECYLKGTLYVKEFVPVVKMISSSNFDSAIRPLRLVYVKL
YEISRNLRSFYEIPILFAIFWIFLSTVDGFYCAMYSMGKDIVSNSINVFALNYSWSVYCISLLL
ILTVTCHVTLEESFSILFNIEKLLLQSDLPVEIKEELNALGFQLSRLKMEFAVCGLFKLNLPF
LSSAVGVIASYVVLMSQLG
>BgerGr230
MVYPRRNVFLSSKVLRDVSNIFGFRIMNEKYSVVDLLCTVVHVSIYLLALFLYFTRIAPDYY
KLLSLKVCVSDVLYTSTCAITSVLYLLSAFYKRRQVSKIILDFEMVDNSLWLIPSKSKVDAK
KGCFLILVFVVLANYGTCLGVLCEICPRFSPSCVFTILISMSGCVNTLLIVNFVAMVLAIRERL
TAVKEVLSGHVEEQPNLRVVRKIFSLETNIISMSECSDKYNQLSDIQVRGIRLVHKKLLSVS
KILMSCYGIPLLFYVTSTLFCAVQVFYSFGFHLATNSLKFNLIVEEVIWSIYCFCFLVILTISCQ
ITVDESFEILSTVEDLLLGSNIPFKTKEELKALSCQLSKFKIRFVVCGVFNLNISFLFSFIGMIA
TYIIVMWQTN
>BgerGr231P
MIYPRRSVFFSSRVLLVTSMIFGLISNSKKCSIVYLLCTAVHISIXPLAFLFYFILIAPDYYQQIP
LKVCXCFMTSAAYLISAFYKRRHLSKIVVDFEVIDNSLLLVPGKSKVFALRLCAVIIGCISLAN
FGFYIGLLSETCPSLSYVCPYTILICMSGCVNTLLIVNFVVLVLAIRERLKTVNELLSSHVGKQ
YNAHKKLFVLEKTPNSKICEKSNQLSEIDVREIRLVHKKLLCVSKTLNSCYGIPLFFCISSILS
GTVEVFYFSGSYMLMHSFRFFVVTEEVYFTIYYFSLLGILTVSCQITGDESFEILSTVEDLLLG
PNVPIETKKELKAFSSQLNKFKIKFSICGLFNLNMSFLGTVIGMIATYLIVMYQTK
>BgerGr232
MIHPRRSVFFSSKVLLVTSLIFGLRSNSKKCSIVHLLCTAVHISIYPLAFLLYFILIAPDYYEEI
SLKVCVSDTLFTSACFMTSVTYLIPSFYKRRQLSKILEDFEIIDNSLLLVPAKTKGYAKRFCT
VSIICTSLANFGFYIGLLSETCPYGSFVCPFTILICMSGCVNTLLIVNFVVLVLAIGERLKAVNE
LLSGHVEKQFNAHKNIFFLVKTPTSMICEKCNHISNVDVREIRLVHKKLLCVSKTLNSCYG
VPLFFCIASILSGTVEICYFSGYYMLMHSFRYSVVTEEILVTIYYFCLLGILTVSCQITGDECFE
ILSSVEDLLLGPNIPIETKKELKAFSCQLTKFKIQFSISGLFNLNLAFLGSVIGMVATYLIVMY
QAN
>BgerGr233P
MVFAANFISPMRSVLYISQIFGIANFTAKENKISYTSFQILWTIFWICVMVSGLVVYFAFLVS
DYCSMDSLKICVAQTLYLFESYFTGAWYLIVCGIXYNRHELARIIQDYMQIDEMYFSKVENY
EVLRKMKRYCLGLVVYVVLVNLVVWVLFFVYSCDTLSWYCLSAIQETISGCVNSLIVVNFV
CMVLAVRGRLKVLNNRLEGHLQNVEGYSREIVFVEFVQMPSLKIVSPWFCSGNDIHCLRLI
YGRLLVLSRKITSFYGQPLLLAIFWIFFSTLLAFYSATEYMADVDVDLFSVSGVLWAVFCVS

LTLILTTSCHLNKDERFDILLKVETLLLETNLSVEAKEELCAMSGQLSKLKIDFTLCGLFNL
NLSFLIGFMGVIXSYIVLMSQMN
>BgerGr234P
MNLIEVKTASYLDTHDSKVSFQMYGSTSKLMSSPKILFYVSLIFGVTTDGKFSAFRLLWSLL
HISVYAIQPILYFVFLAPERYETLPLKVFISSTLYTVSSFLSPLIIFILXSIYKKHKLSQLIDDFQI
IGRLLLITQNDELFAKRNKISSLLLGYITLANFSLDVASFERRCSSLSLLCVYSIVDAMSGFVI
SLVIVNIVSIQFLIWERLKTINKLLCGYVEKYDGYSNREKLPSKKIYTGITVVPTSVASSYISG
HQIRDLRLAYWKLFEISRSLASCYGVPLLITTLYVLLSSIHAFYLGVSVTKKGESGVGGIYWF
FYGLSLMTILSVSCQRTTDESLEILVHVERILLQSGISVSTKKELKALREETRSLKIEFAICGM
FTLNLGLLCNIFGLTLSYIIIMWQIT
>BgerGr235P
MKTHKRKSVEYSVTMSRNSHTTTNFILSALKPVLYFSQIFGVVNFSRNKNKIYFNYFNLFW
TILYICIVITGLVMYCIFTLSGYCVDFPLKLCISHTAYVFAHYSTSILYLTICGLYKRHGYARIIE
ELIHIYIFISEVEANKVQSRLKKVYLLNIFYITIVSFCVSVFYIARSCEYWTGSCVQTLLECLSG
FVNTAIIVSFVAIVIAVREKFNVINKRLHGQLQNSEGYPKEIIMVSVASENVRRTSSYCTGHE
VRCFRLVYGRIYELTKTLLSFYGFPLLSAILWISXFYNAVHSYSEEKYFSSIVDNFLWSLYCV
SLLTIITVTCQMVVDESLATVFNIEQLRLQPGVPTEAKEELKEMGCQL
>BgerGr236P
MLKLSSDSFSATNLLLFVCQLFGLSAAPNNNNSKLLYILTLFWFILPLLNIISYVTPILFGEK
YNSLLVSLISYSLYVISSSITSIASLISFSKNQQXELLRVIQEIDITLLQIKNAGIVVGRIKYGFFV
ICYTLLANILMFVLYIYYGCVCNNKHLICLHLILDAICTFISSTMVISFVLMVLEIRERFKVIN
KLLHDHGNNSGSKNTVSLLIPRAHMKLGTMFVHKMRYLRIAYVGLYAVSRELMSIFGFSL
LSATCWFVFSCLSSFYFVITSSFMDLMFYTRLVFLYVWGFYSWSLLFVLCTSCGLAAKECQ
TSIFYIQMVLLGINLDSAVEEELKRMCSLLNCLKINFCVCGIFNFNLSFFSQLLGLLVSYIVVI
CQIV
>BgerGr237
MAEYFSRSLSAMMRLLMFSEIFGLSTSSYTRQLSVQIIKYFWMFVAFFNFFPYVVSLVFNE
KNTPSLHWMIVYFSYVFFNSITAITSIFCFGKKTQMQKIFEGIKQCDAILIKFGIPEVNMGGI
KYGFFLLIYVVVLNIITGFLYVGTAQFTWIRFHYVLDCICCFINSILVVNIFGILLAIRERFNVI
NIFLHNQTFCSEHKSNVSGIFHTKHFGVPLCNFDCIHNLRFLRIVYFQLFVVSKDLMSMFG
VPLLSATLWIILTCLTSVYHIVFVLRLESSMSLNLALYAMWTIFTGSLLCALTFACSLILDDR
RKIIFYVHMLLLDRVVGSSLEKELKKMCTQLHCLRMNFSVCGMFQLNLQLFCNVLGLIISYI
ILLSQFK
>BgerGr238
MTVFGTSFISAMSPLFYVSQASGLVSFSLKRNAMEVSVFRLIWTYFLLFILILDSVFYIVFRIS
DENTYPVKMYVVVYHLYFISCHFTCITSLIICSIFKKKQLMICISGLNLLSTFKFAARECHAVH
SKLRKIYILLVAYVCFFNISCDADYLIVVCNSILTCYVGITNSFSAFAASVVIINFTFLVLFFHA
KLKLINKQISTYRCKLHQEMTATSSANVSSSANVIHVFTRNIYSCLVPRGELRCMRLAYGLL
QENWQKLSSIFGFPLLCAFLWIVVGAVLTFYEALYAFQGRFESVLWFIFSVSLMFVLTVSC
HMVVGESRLILFNIESLMCETNLVRSMKEEFDDLRCQLSIQKFEFSVCGLFTMDLKFLCTR
FGILFSYILLISQIK
>BgerGr239
MSDTNFLVSSNFLLYFSQMFGFTGNKTTLSKLHLFWVLLHVSFYGLAPIMYFTIIAPAKYK
TIPLKMCIVEALYTSSSSLTSLVCLLYVGIFKRHNLTNLIEEFEDIQEIMLSDTEISGISDKRN
LFLVLLGYILLVNFVLDVHAFTRECTVLGWRCLYCVEDSMSAFVNSLLVLNFVSLVLTICGR
FKIVNMLLSDHIIREKVYSKQIRVETNQNSLPRECNIHFTSLSGLQVRNYRLVCRKLFEISRT

VTSCYGVPLFITTLYIFLSSVHTFYLGIIFTSNTDNYGFTNGLGGITWSIYCISLMAILTVSCQ
MTLDQSSEILVNIESLLFQSNISMKTKEELKALRSQFRSLKVDFSICGMFSLNLGSLCSILGII
LSYIIIMCQMSL
>BgerGr240
MFKSYCVRMSGRFYSNMSRTSSTNRFISSMKTVFCLSQFIGIINFSIDRKNNNALKFPVIRT
LWTVVMVLIGIGSVMMYFMSNVSNFVHPSSLKIKISYIFMYTFKYCTYFSSLIYSGMYKRKQ
TSIIVGEFQFIDNLLSSTTGSVPESGVRKMSYLLLTFLASIKFIDDLLYITSKCDYFVIVCIHCV
IYNYNTYVIMFMILMFVCTVLAMKERYKTVNRILSSFSNLETKHSKGSLIFIHSLSSVVPLTP
ASVHKVRCLRLVCKSLYTISLALLSCYELSLLCATCWIFGDCVHGFYSSAYSLQEGNEFYLIV
IDSVIWALFCMSLMFILTSSCQGAADESRLISINIQNLLLEANLSKDVRDELKDFRCQLNDM
KLEFKICNMFKLNSSFFCHLVGIISSYIIIMCQVE
>BgerGr241
MAHTQTSFISLMKPIFCISHITGLTNFAIDKRNGNKITFPFIRLLWTILFVIVGAWSLPLCFV
HNTTYFDYSVTLKFKITYNIFLLSKYCAYMLALIYTGIYKRRKIVFIVKQFQLIDKLLSSQFSC
NMDDRARKSYLFLLIYICCIKCMGDITLTSSKCKHFTLICIHSLVFNYNSYVIFFLVVTFIAIV
LILRARYQSINKIILGFCNNLEVCHEQDSIASVDCPSLLVGLPSVNAVRSLRIVYKRLYEISRA
VIAYYEISLFASICWIIIGCVICSYVGAFSLQDKGNSDYIMQMDMALWSLLCISVIFSLSLSSQL
AVNESRVARVHIQSLLLVENLSSHVKDELNAMRSQLIDLDLDFTVCGLFRLNLSFFCNFVGI
ICSYLIIMCQL
>BgerGr242
MSKTSSIYISSMKPLFILSQFVGVSNFVIDKRSNTLKFPISRTLWTILFIAVGAGSFMLSPFDN
LANFNYSKTLKIKIPYLLMLFFKYFAYFSSLMFPGIFKRKQFARMLQEFQFIDKMVMSEIGF
IVESKVKKWSVLLLTYLFFIKFIDDLLYVISHCTSLSTLCVHSFIYDYNSYAILTLILIYICTVLA
LKERIRIINKIFSGHCSNFEMDNNNCLIVFQRSSFSSVPWGTLSVRQLHYMRIIYSKLFQTSR
DLISCYEIPLFASTGWTFLGCVHGFYSSAYSLQNGNGYYIKMVDSTLWSLFCVSLMFVLTFA
CQMAFDEYQVTKVHVQNLFLESRLSKEVKNELNTMRSQLKDMKFEFNICRMFNLDLSFL
WSFIGIICSYIIIMCQIE
>BgerGr243
MTSTSNTFISSLKPTFYVAQFFGVINFSIDTTKNIVKFPILNICWTIVLISLGFVTLALNFFYS
TSNFDHSSSFKLILSYSFLTFLKYLAYMSSLLVSGILRRKEQAEIVTRFEYIDKFLFSRNYSLK
NRRVKHLSCLLIVYLIFGKTLDDLAHIVGKCNSLSLICFHAVIYNSHTYVILILISMYISIVLAL
VERFKTMNRILSSFCSTSELHHVGASVISKPCPVLSLSKVKSLRQVYTSLFLISCNLISCNQFS
LLFGICWIFSSCLYCTYVSAYAIQKSYGDFVFMLELMLWVFFVASFIFFLTATCHRAVEESRI
MRNQIQILLLGSNFSREVKDELNAFNEQMTVMKFKFEICGMFELDLFFFRSFVGILCSYIIIL
CQFD
>BgerGr244F
MKTSNTFISSMTPIFCVLQVFGMINFSRERFCNAAFPVFRTIWTIFSLVVGTSTMILSIIYPN
ADIDYSISLKLSISYNILSFFKIIGYLSPLLMLGIYKRQDIATIVTEFEFVDQLLSSHINSAAQIK
LKRQCLVLLVYFVSIKFIHDLGDISYLCKSFSLKCLHSVVYDYISYVILITIVGYVSIIFSMRERF
KVVNSILFGYCSNFDLRLCKGTVTSKISRTSWCFPSADELLCLRLVHRKLFTIAHALESSFGS
SLLAGIGWFFSSCVHFCYLAAYLMQSGSKNYISMLDMIIWAPFCVSMIYILTSSCQKTVDEY
RTCTVHVQTLLLQPNLPKDVEKQLNALNSQLITMNLEFSLCGMFRLNLSFFCNFIGIICSYII
ILSQFE
>BgerGr245F
MIHPRRSVFFSSKVLLVTSLIFGLRSNSKKCSIVHLLCTAAHISIYPLAFLLYFILIAPDYYEEI
SLKVCVSDTLFTSACFMTSVTYLISSFYKRRKISKIIVDFEVIDNSLLLVPAKTRVYAKRFCA

VIIGCISLANFGFYIGLLSETCPYGSFVCPYTILICMSGCVNTLLIVNFVVLVLAIGERLKAINEI
LSGHVQKQYNIHKKLFVLVKTPTSMICEKCNHLSDVDVREIRLVHKKLLCVSKTLNSCYGI
PLFFCIASILSGTVEICYFSGYYMLMHSFRYSVVTEEILVTIYYFCLLGILTVSCQITGDECFEI
LSTVEDVLLGPNIPIETKKELKAFSCQLSKFKIQFSICGLFNLNLAFLGSVIGMVATYLIVMY
QSN
>BgerGr246P
MKTHKRKSVEYSVTMSRNSHTTTNFILSALKPVLYFSQIFGVVNFSRNKNKIYFNYFNLFW
TILYICIVITGLVMYCIFTLSGYCVDFPLKLCISHTAYVFAHYSTSILYLTICGLYKRHGYARIIE
ELIHIYIFISEVEANKVQSRLKKVYLLNIFYITIVSFCVSVFYIARSCEYWTGSCVQTLLECLSG
FVNTAIIVSFVAIVIAVREKFNVINKRLHGQLQNSEGYPKEIIIVSVASENERRTSSYCTGHEV
RCFRLVYGRIYELTKTLLSFYGFPLLSAIXVDFDAFYNAVHSYSEEKYFSSIVDNFLWSLYCV
SLLTIITVTCQMVVDESLATVFNIEQLRLQPGVPTEAKEELKEMGCQLSRLRIEFTVCGLFN
LNLPFLCSSMGVIASYLIIISQMS
>BgerGr247
MNQSGKDFILSSKLLLYVSRMFGINGNKSTLSNIRLLWTLVHVTIYTLCPILYFTFLAPKKY
EESPVKISISDALYVSSSCLTAVLYVALLGIFKRRKMVEILQTFQTMDVFLQSENVKVKSRD
NLSLVLFVYVTLVNICLDVTTFGRDCTVLSSKCLFDIADAMAAFINSLMVLNFVAVVFAIGE
RFKVINTLLYSHILKDKYYWIKMGETTDLISAISEYKLNPSPLSDHQVRNYRLVCRKLFDSS
RSSTSCYGFPLLFATLYILFAFVHTFYLGIMLSSGNLLFVSGIMGVISSIYDLSLMVILTVSCQL
TLDASFEIIVSIERLLQQSNISVKTKKELKAMRYQLRSLKVQFSVCGMFSLDTKLLCSIFGIIL
SYIIIMWQTS
>BgerGr248
MLNPSSKSYSSTKIILFVSKIFGFTSYSRQTNSLSFIILRCFWVVVSLLNFVSFVLSLYEHPEFS
TQSLISYTFYVATNSITSISSLICYSKKRRIRKILDCIEDVDGILLQIGIPSTNISYNKHGAFLSV
YIILANVVSILLYISEGCLCQNCTWSCVHVVLDLACVFINTAMIVNHVFVVFSMWHRFKVL
TKLLRAEDHRAKTKQVRSNKITEAFIKVSLNICFLRKLRFLKLVYLRLYVLSRELMSLFGVP
LLSATFWCVVACLAAMYDATSEVVDWEMYFLMTCLSLWVLYCCTLLFVTSLASDLIVKES
HAISSCIQMLLLDNYLDSTVEKELKKMCYLVDCLKMNFSVCGVFSLNLSFFCQVLGTMISYI
VVICQTK
>BgerGr249
MMLNFSSKFYSSTKVLHFVSRVFGFVTDDSSYMNSKLFGILRLFWAVVALLNLILYAISIVS
KEQTGLSLKTLIAYVSFIVCNSLTTISSVICYSKIQRIAKLYNCIQVIDALLIQVGVPRTHISGIE
HGFILVVTVFIANVVSVILFVRQGCVVQCGIWGCLHVVMDFLCTFVNSTMIVNYIFLVFKIQ
GRLKIVNTLLIGIERRCQTENPVPDHIPVVNFRSNSNAYFLRKLRLLRLVYFRLYAFSQDLM
SLFGFPMLNATLWIILSCIDAIYYIVIYPGVSLEISVDLVFLTFWILFYGSLLYILTFACDRTVT
ESEATASNVQMLLLGNNLGNVIEIELRKMCHLLDCLKMNFSACGVFSFNLPFFCQVLGTLI
SYIVVICQIK
>BgerGr250
MFGFTGSCARFMVFHPCMILFHAALYPFGFVNYFLLLVNKYDTFSLKMFVSDVLYSACSF
MTSFMYLVFTVNHKRKLRCILIADLQEIDDLLMVAPGSFDVSCTKNKVCLTMFIYFVVASF
GIGVMPMVQECYSLRLWCVYTIQDIICGCTISLMVFNFITVVLGLRERLKFIKELLKDHDEK
EKLHDKKKLISSLLSDDSTVGCAYLSESEVRKFRLVYGKIFAVSRTLTSCYSVTLFFATSTTF
FSSVHTFYFSTYYLLEENAAYIELLPDVLWSVYCCTLMILLTFTCHLTVNESLELSIHVESLL
LRENLTKNIKKELTGFGSQLTKLKMSFQVCGVFNLNLPFLSSLLGIIISYILVMCQIV
>BgerGr251

MLHIRPGMNLMLAMKPLLCVSKVFGVANFNDIKNVACTSMFEFVWTFLLMFINASGPIM
YFIFIAPQGCNIYPLKLCISHTGYTIASYSMSFLYLVLCRIKKRYETGRIFEAIQQTDIMFPSAE
ESVVIQTKTKFYCLAVLIFISLTSSLNGVFFIVSNHKSLTWCCVNTIQEIISAFMNSLIIVNFA
TVVFSIGQRFKVINKRLYSQCQNTECYLKGTLYVKEFVPTVKKISSSNFDSAIRPLRLVYVKL
YEISRKLRSFYEIPLLFAIFWIFLSTVDGFYCAMYSMGKDIVSNSINVFALNYSWSVYCISLLL
ILTVTSHVTLEESFSILFNIEKLLLQSNLPVEIKEELNTLGSQLSRLKMEFPVCGLFKLNLPFL
SSAVGVIASYVVLMSQLG
>BgerGr252P
KKRISFLKVLRDVSNIFGFRIMNEKYSVVDLLCTVVHVSIYLLALFLYFTRIAPDYYKLLSLK
VRVSDVLYTSTCAITSVLYLLSAFYKRRQVSKIILDFEMVDNSLWLIPSKSKVDAKKGCSLIL
VFVVLANYGTCLGLLCEICPRFSPSCVFTILISMSGCVNTLLIGNFVAMVLAIRERLTAVKEV
LSGHVEEQRNLRVVRKIFSLETTIISMNECSDKYNQLSDIQVREIRLVHKKLLSISNILMSCY
GIPLLCYVACTLLCAVQVFYSFGFHLATNSLEFNQIVEEVIWSIYCFCFLVILTISCQITIDESF
EILSTVEDLLLGSNIHFKTKEELKAFSSQLSKFKIRFVICGVFNLNVSFLFSFIGMIATYIIVM
WQTN
>BgerGr253
MIYPRRSVFFSSRALLVTSMIFRLTPNTKKCSIVHLLCTVVHVSIYPVAFLFYFILIAPDYYQ
QIPLKVCVADTLFTSTCFMTSAAYLISVFYKRRQLSKIIVDFEIIDNSLLLVPAKSKVYAKRF
CAVIVGCIVLANFGFYIGLLSETCPYGSLTCPYTILICMSGCVNTLLVVNFVVLVYAIGERLKA
VNEILSGHVEKQYNVHKKLFVLKKAPISKIYEKSNQLSEIDAREIRLVLKKLLSVSKTLNSCY
GIPLLFCISSILSGTVEVFYFSGSYMVIHSFRYIVVTEEVFFTIYYFWLLGILTVSCQITGDESF
EILSTVEDLLLDPNVPIETKKELKAFSSQLTKFKIQFSICGLFNLNMSFMGTVIGMVATYLIV
MYQTN
>BgerGr254
MIYPRRNVFFSSKVLLVTSLIFGLRSNSKKCSIVHLLCTAAHISIYPLAFLLYFMLIAPDYYEE
ISLKVCVADTLFTSACFMTSVTYLISSFYKRRKISKIIVDFEIIDNSLLLVPAKSKVYAKRFCA
VIIGCISFANFGFYIGLLSETCPYGSFVCPFTILICMSGCVNTLLIVNFVVLVLAIGERLKAVNE
LLSGHVEKQYNSHKNIFFLVKTPTSMICEKCNHLSDVDVREIRLVHKKLLCVSKTLNSCYGI
PLFFCIASILSGTVEICYFSGYYMLMHSFRYSVVTEEILVTIYYFCLLGILTVSCQITGDECFEI
LSTVEDLLLGPNIPIKTKKELKAFSCQLSKFKIQFSISGLFNLNLAFLGSVIGMVATYLIVMY
QAN
>BgerGr255P
MVSTANFISPMRPVLYISQIFGIANFTAKENKISYTCFHILWTIFWICVMVSGLVVYFAFLVS
DYCSKDSLKICVAQTLYLFESYFTGAWYLIVCGXNYNRHELARIIQDYMQIDEMYFSKVEN
YEVLRKMKRYCLGLVVYVVLVNLVVWVLFFVYSCDTLSWYCLSAIQETISGCVNSLIVVNF
VCMVLAVRGRLKVLNNRLEGHLQNMEGHSREIVFVEFVQMPSLKIVAPWFCSGNDIHCLR
LIYGRLLVLSRKITSFYGQPLLLAIFWIFFSTLLAFYSATEYMADVDVDVFSVSGVLWAVFC
VSLTLILTTSCHLNKDERFDILLKVETLLLETNLSVEAKEELCAMSGQLRKLKIEFTLCGLF
NLNLSFLISFVGVIGSYIVLMSQMN
>BgerGr256F
MYGSTSKVMSSPKILFYVSLIFGVTTDGKFSAFRLLWSILHISVYAIQPILYFVFLAPERYETL
PLKVFISSTLYTVSSVLSPLIYLFFVSIYKKHKLAQLIDDFQIIGRLLLITQNDELFAKRNKIST
LLLGYITLANFSLDVASFKRRCSSLSLLCVYSIVDAMSGFVISLVIVNFVSVQFLIWERLKTIN
KVLSGYIEKYDGYSNREKLPSKKIYSGITVVPTSVASGYISVHQIRDMRLSYWKLFEISRSLA
SCYGVPLLITTLYVLLSSIHAFYLGVSVTKKGESGVGGIYWFFYGLSLMTILSVSCQRTTDES

LEILVHVERILLQSGISVSTKKELKALREETRSLKIEFAICGMFTLNLGLLCNIFGLTLSYIIIM
WQITELG
>BgerGr257
MKTHKRKSVEYSVTMSSNSHTTTNFILSALKPVLYFSQIFGVVNFSRNKNKIYFNYFNLFW
TILYICIVITGLFMYCIFTLSGYCVDFPLKLCISHTAYVFAHYSTSILYLTICGLYKRHEHARIIE
ELIHIYIFISEVEANEVQSRLKKVNLLNIFYITIVSFCVSVFYIARSCEYWTGSCVQTLLECLSG
FVNTAIIVSFVAIVIAVREKFNVINKRLHGQLQNSEGYPKEIIMVSVASENVRRTSSYCTGHE
VRCFRLVYGRIYELTKTLLSFYGFPLLSAILWISLSTVDAFYNAVQSYTEEKYFSSIVDNVLW
SLYCVSLLTIITVTCQMVVDESLATVFNIEQLRLQPGVPTEAKEELKEMGCQLSRLRIEFTV
CGLFNLNLPFLCSSVGVIASYLIIISQMS
>BgerGr258P
KYEESPVKISISDALYVSSSCLTAVLYVALLGIFKRRKMVEILQTFQTMDVFLQSENVKVKS
RDNLSLVLFVYVTLVNICLDVTTFGRDCTLLSSKCLFDIADAMTAFINSLMIINFVAVVFAIG
ERFKVINALLYSHILKDKYYWIKMGETTDLIPVISEYKLNPSPLSDHQVRNYRLVCKKLFDS
SRSLTSCYGFPLLFATLYILFAFVHTFYLGIMLSSGNLLFVSGIMGIISSIYDLSLMVILTVSCQ
LTLDVSFEINVSIERLLQQSNISVKTKKELKAMRYQLRSLKVQFSVCGMFSLDTKLLCSIFGI
ILSYIIIMWQTS
>BgerGr259
MEHYKPSVILISSLKPVYYVSQVFGIASFTLGSDQIHFSVLKLIWTAVYTCMVAASPVIYFVF
LNPEYCSEIPANLCLANALYTVSTYTVSILYIFICGIYKRRELGRIFAEFMHIDDVRLFEDEK
HALKSNTRTFSVMFLVYIMVVNITDTSLRIFYTRESSFWIKVSVIQEGLSAIINTLAIVNFVT
LVIAMRERFKLMNKRLLGGNSANTEDRASKAIIVGDFFKRIRENDVPISSCTYREVRCLRLV
YRRLYDVSQRLTSFYGIPITAAVIWIFLSTIDAFYAATFALSRGVGEVEVNILWSCYCVSLLVI
LTTLCHWCVQESLTVVFNVETLLLQSNLDLRTKEELNSLGFQLSRLKINFILCGVYNLNLPF
LCSVMGVIASYVIVISQMN
>BgerGr260
MSKCSSNSYSASKLLFFVCQIFGFSVSLHHRGKVMHIITLFWLLLAIVNITSYVIHLVKYENY
STSLQPVIAYMFYVISSLVTTIASILSFSKNIQFGRILQVMREIDAILLEIGNPGIMVVGRIKLG
LFLIIYAVVTNLALLVLFIRDGCRFKFWVCLHLSVDNACSFINSMMVISFVLFVLDIRERFKL
LNKLLNDQGNSFGSKNTASLLIPKRHMMLGHSSYFLHKLLSLRIAYVSLYSVCKELVSIFGF
SLLSGICWIVLSTLSSFYFAVVSANMGVELSMKMFFLYVWSFFSMSLLYLICNSCHITVRDS
QKSIFYIQMLLLGINMDSCIEEELKRMCSLINYLKLNFSVYGVFSLNLAFFSQVLGLMISYMV
VICQVK
>BgerGr261
METAGSDLISALNPIFYASKCTGLMNWSFGRKKNSVWRYGWTIFVTSALVGNCFLWLWT
KISEGFPVHYYATKVILSYELEFLLNHLSSITCLVYSGFFKRCQLKKLILDLSCVDGSQKGTSP
KYANVKASSVLLLMFFIGINIVCDAFLATEECQSTTLKCTVAIGNYLSTAVICVMVLNFVSFV
LVIREKLKYINHQLTNYKRKLEMTSSEKTIHLLVQSNIFRHEIRCLRMIYGRIFENAQVLSSC
YRFPLLCTWGWMIIKFVDSQYFSLYSLQEDYDNVLLMVAGVLCSFSSLTIIYIMCLACQKTT
DETHKIVFGTHELLCLPNLSIGIREELTEMSSQLTILKLEFSVFGLFSLDLKYMSKTFGLLLS
YLILMHQMK
>BgerGr262
MSDTNFLVSSNFLLYFSQMFGFTGNKTTLSKLHLFWVLLHVSFYGLAPIMYFTIIAPAKYK
TIPLKMCIVEALYTSSSSLTSLVCLLYVGIFKRHNLTNLIEEFEDIQEIMLSDTEISGISDKRN
LFLVLLGYILLVNFVLDVHAFTRECTVLGWRCLYCVEDSMSAFVNSLLVLNFVSLVLTICGR
FKIVNMLLSDHIIREEVYSKQIRVETNQNSLPRECNIHFTSLSGLQVRNYRLVCRKLFEISRT

VTSCYGVPLFITTLYIFLSSVHTFYLGIIFTSNTDNYGFTNGLGGITWSIYCISLMAILTVSCQ
MTLDQSSEILVNIESLLFQSNISMKTKEELKALRSQFRSLKVDFSICGMFSLNLGSLCSILGII
LSYIIIMCQMSL
>BgerGr263
MPRASTSFAKTMGPAFSLSQMFGLSVFSKDKQKRTFTFPKYRILWTVILVVFGATFVVLSF
IGHVSHFDDRLPLKLFIVHKILLFFKYLAYMSCLLFCGIYKKREVVSIMDEFQFIEQIFSSELG
CKVYFRSKRYLLCLLLLKMTSDLVHFGSNCIPFNIQCLHFMMFSSNSYVLFVLVIVYTSFVL
ALKEGFNAVNNRLSSLWSSREPYKKKVIVYNSIFSPFAHWCCPLVHEVRTLRLVCRRLFVI
SRLLVSCYGFPLFSTICWIFLGCVHYFYIAAYALGEGGNDSAVVTHGLWALLNIAMLFVLTF
SCHVSLEEYSKTTIHIQTVLLEANLSNDVKDELNAMNSQLNTMKLEFKLCGIFRLNLSFLS
NFVGFICSYVIIMCQFE
>BgerGr264
MSKTSSIYISSMQPLFFLSQFVGVSNFVIDRRSNTLKFPFSRTLWTILFVAVGAGSFMRCPF
DNLTNFNYSKALKIKIPYYLMLFFKYFAYFSSLMFPGIFKRKQFARMLEEFQFIDNIFMSEI
GFMVESKVKKWSVLLSTYLFFVKFVDDLLYIISHCRSVSTHCVHAFVYNYNSYVILILILIYIF
TVLALKERIRIINKILSSHCSNFEMENNSCLVTIFQHNSFPSEPWGALCDRQLHCIRIIYNKL
FQTSRDLIFCYEIPLFAATGWAFMSCVHCFYSSAYSLQNGNEYYINMVDSTLWSLFCLSLM
FVLTFSCQMALDEYQVTIVHVQNLFLESRLSKEVKNELSALRSQLKDTKFEFKICRMFKLD
LSFLWSFVGIICSYIVIMCQIE
>BgerGr265
MYSTSNILLPSFDSLIFTSQIFGLSNFAIEEKNKNKMKFPKLRLLWTISILIVGIVTMVLCFGP
YAAGINYSISLKLNVTYNLVISLKYSSFIASLMFLGIYRRRELATMVREYEFIDRILATKFRYA
KYTWKKNSFLCIYFIVIIKFSCDVINISAMCQMYSVTCVTCVLNDSCSYVVLVMVVSYIYLVL
SLREKYKFINKILSNFCNDFDDCHKIGNITFVHDLSANVVHSLRLVSGKLFGISRTLVTCFGC
PILLTICWAFISCVHFFYVCAFNFGGSTIIYADMGLWVIFCLSLLCTLTTACHVTVEEFRTAT
VHIQTILLDLNLSKEVVEELHGLNSQLCTMKLEFILCGMFRLDLKFLCNFIGIICSYFIIMCQI
E
>BgerGr266
MTCISSTFLSSMEPLFYLSKIFGVINFTIDKHNNAIKFPFIRLLWTISLVIVGGIAMMVLFLYH
VFHLDYSIPIKLNISYSGLVFFDCFTYLSAMAFSGIYKRKELAIIMKELVAVDRILIADTGLEI
GKDFKNSILYLLIFSFVTKVTDDVLYAIPYCGTFCIKCIHCIVFSFNSNMTLLIIIMYVSIVLAL
KDRYKATNKILSEFCSNFNTHNDKCTTFPKHSLKFVNHPNFPNIFVLISLRQVYKKLFGVS
LALTSCYQLPLLAVTCWISVSCVHCAYIAMNELQSGGQGLLVVLHMSLWTFLCMSLMIVLT
CACQMALNEYRMTSVHIQTLLLESSLPNDIKEELKNMSFQLKTMYLEFTVFGMFRFNSTF
FCNFVGIIFSYLIIMCQLQ
>BgerGr267
MSVTSNTFIATMAPIFYASQIFGLINFSKNRFKNASFPVFRTVWTVFSIVVGTSSMVLSLIYP
DSDIDYSTSLKLSISYNVLSFFKCLGYLSSLVILGIYKRQEIATLVREFEFIDQLLSSNNNSAA
QINLKRWLLLILSYFISIKVLDDLLNISFMCKSCSLKCLHSVVYEYISYVLLITIISYVSIVLSMR
ERFKAANKILSDYCSNFELRHYKGTSISTFSLSSWRLPSADELLCLRRVHVKLFTIASALESS
FGFSLLSGIGWIFSSCVYYCYLAVHLMQSGSENYISMLDMTLWTLFCVSLIYVLTSSCQRTV
DEYRTCTVHIQALLLQPNLPRYVEKQLNAMNSLLITLNLDFSLCGMFKLNLSFLCNFIGIIC
SYIIILSQFE
>BgerGr268
MVPKKTSFITSMEPIFCVSQIFGLTNFSVDKRNNIIVITFPFLRIIYTFLITILGLAILLLGFVFG
TSNIDNSTSLKLCISYHSRLFFKSLAFLTSLMFSGIYKRHEIAAMLKELHFVDQVCLELGFVT

YRVKKFSLFVLSYFILTKSIDDVIHLTTRCDSLSSLCIYTLMYNGISYVILLMLIIFVSVVFALT
ERYRSANKILSGYCTEFEVQHNKDNVIWDCESHVSLVLPQCPISVHELRCLKLVYRRLSKIS
RLLLSSFGVPILAATLWIFVSCVYDAYVSAYAMKIGRESYVIITDMLLFALFGVSVMFVLTFL
CQMALDEYRMTKINIQTLLWEPNQLSAVTIELNAINSQLNCMKLEFTICGMFSFNLSFFCK
FIGIMCSYIIIMCQIE
>BgerGr269
MDSTQFTFILSMKPIFYVSQIIGLTNFYIERNSKFVLKYPLFRLLWTIFFVIIGGASLILCFVYT
TSQFDYSISLKLLVSYNLRLCFKFFAYCSCLVYSGIYKRRKIATIVKEFQFVDEILRSNLGYIV
QSRVKVLSLFLISYVIVTKGVDDVLHNTAKCKPLSLMCLHSIIFNCNSYLILIMIVTYICIVLA
LRERYMAISNILCSALTNLDIVPFKDTMTSSPSQPLLAPCVCPTVNEIRVLRLVYRKLHAISR
NLVSCYGLSLLFAICWIFLSCVYGFYVSAYSLHAGGDVYLVVIETALWAFFCMSLMFVLALS
CHLTVDESRDLSVHVQTLLLAANLSNDVKEEVNALTYQMGIMELEFTICGMFKMNLPFFC
NFVGIFCSYVIIMFQFE
>BgerGr270
MGSTPSFVSSLGPLFYVSQIIGLINFSIDKKNSTTISFPITRSLWTIFFLIVGGGLLLLCFTYTT
SHFNYSVSLKLYVSYKLKLFFKCVAYFSCLIFSGIYKRQEIAKMVKEFHFVDELLSSQIGFAA
NSRVKLLSLVLLAYTIITKSIDDMFHYSDVCRQLSLRCVISIIYNYNSYLILIMFITFICIVLALR
ERYSTTNKILSDFCTNFETNIDKGTITSVHHHTCFVPWSCPAPEDIRIVRLVYRKLFDISRR
LTACYGLPLLSSMSWMFVGCVYGFYVAGYTSMVDDLFLVVTDMIVWSFFCMSLVFVLALS
CHMTVDESRMTTVIVQTLLLESNFSKEAKEELNALSYQLGSMPLEFSIFGMLKLNLSFFCN
FFGIFVSYVLIMCQFQ
>BgerGr271P
MDKTSSTFLSAMAPAFCMSQCFGLINFSVVTKKRTMIKFPIYRIVWTIFLAIIGSISLVFCVID
LTSHFDYSISLKLNISNKILHFXKYISYFSTLLYSGIYKRKSILKIIEEFHIIDKGLSSLIGYTVES
RIKKLSLLLILFLVFVKCLDDIMYTSIMCKQNYSMCIHYINYNYNSYVVLTLIILYVSFVLSLR
ERYQTANTVLLGLRTNFLSFPNTSAIFSDSTFLLIIPCRSQSVNELYSLRLIYRRLFTISNTLI
NCYGLPILAALGWIFLGCVLYFYIVAYLLQQGNYTFVMVVDMLLWIIFCMSLLLVLTLSCHR
TVDEFNASIVHIQSLLLEKSLSTDVKDELNAIRSQLIDLKLEFTVCGMFKLNLSLLSKFIGIIC
SYVIIMCQMN
>BgerGr272P
MKLKMSVASSSFVSSMAPIFYISQIMGLISFSIEKRNNDSIRVKFPTWRVLWTILQAVVGGG
ILILSIVYSPTHYDYSZSLKLTISLNVKLFFKYFAYFSSIIYSGIYKRYEMANVLKQFHSMDKL
LFKLTNHAIDSNIKKHYFFLLAYLVSIKCVDDLIDVTTKCTSSYVICMHAVIFNLNSHAILIM
VVMFVSTVLAIGERIKTTNRILSDFGTNHNKQNISGSSLVHSCTSSVNKIHCIRIVYKRLFNA
EQSLMSCYGLSLLATICSIFIGCVRGSYVSALSLHKDNYGFLVMVDMFVWSFLCISLMFIMT
SSCHKTMDKHRKTKVHIQAMLLESNLCSKLKEELNAMISQLGTMKLGFSVCGMFRLNLSL
FCNFVGIICSYVIIMCQIE
>BgerGr273
MSVTTASFVSSMNPIFCISQIMGLINFSIEKRNNEEIRLKFPIWRILWSILLAVVGVVTLILCF
VYNSAFFDYSKSFKLVLSFNIKIFFKYFAYFSSMIYSGIYKRHKMATIVKQFQCIDTLLFRHT
GHEMSTKLRKHSLLLLAYFVCVKCIDDSIDVALKCAYSYAVCIHTGVYNLHSYGIMLMIVSY
VSTVLAIRERIKYTNGILSDLGSKFLTNHNKGNIISGASVENSCSAFVSKVRCIRIVYRRLFSV
EQYLTSCYGISILGTICLIFLGCVHTSYVSVYSLHEGNFGFLIVVDLFAWNFFCFSLMFVLTT
ACQKTLDEQRMTVVHIQTLLLEFDLCSKLKEELNAMSAQLSSMKLEFSVCGMFKLNSSLF
CNFVGIICSYVIIMCQVD
>BgerGr274

MATNVPKRNFISSVKTLFFISQFVGTSNFRLEKNSIEFTRFRLVRTIIFLIITILGPVFYFVFSI
SSYCSGMPVKVCAANLPYTLSTFLTSVIFLFHCAIRKRQKLVQIIQDFIHIDEMFLSEAENLS
VQDNLKENCLVLLIYILLSNVVATAFFISSNCQPFSWMCVTAVQKCISGVINTFIIVNFVAM
VMAIRERMKIINKRLLDRIENQEGHATKTVIVGEFLQTVRHKMVVSSSCCSREVRSFRLVY
GRLFTLSRELTSFYGIPLILVITWIFLNAVNSFYFVAYSLGKEGMINYNSITGNIGWSIFCFSL
LVILTVSCHLAVEECNVTVFQLEVLLLQSDLALQAEDELNSFSFQLNRLKLCFSVLGLINLN
LSFSFYVLGILIYYIILIIQIT
>BgerGr275F
MTVNIRPINFISAIDTVSCLSQIFGVTKFTVGKNKNKLPWLSIFWTSLYICIIGLGPFVVYFAFT
FSEYCDGIPVKLCTSNSMYIVCSFVTSFFYLLLCGVCKRRLFARIIEEFLLMDDLLLTGPENL
DLQKNLKKFCFYFLIYVFLLFFGVGGFFIIVSCETFSWTCVNGVQEWISGFMNSLIIVNFVS
MVIAMRQRLKIVNKRLLGYLQKVEGCSKASSIVEGSVLATKRKIVKFSSFSAHKVLCLRLIYS
KMFTVCRRLVSFYGVPLLLSILWIFLSTVNAFYTAAYCLAINTNYNSIIINILWSSFCLSLMII
LTVSCHLCVDESLATVYNVEVLLLGFNLPVETKKELSYLRLQLSSLKIDFTVCGLFKLSLPFL
CSELGVIASYIIVISQIEIGRASCRE
>BgerGr276P
YDRFFFLRLLWTILLGVVYALTPFIYFTVGTPLHLQNMPLKMCIADVLYTSSISVTSVLYLV
HTGIYKRGLLAKAADQFHEIDDMLFTRCENSSRNTSKRNSLILFAYMLLVNLVEDIVCLQA
SCISPNWLCVYTAQDGLCNFLNSMMVVNFVSMVLVIRERFNTINIILSGHTDKKMVAMTP
FLTEDIDRILTCRLVFTLRVAYRKLFEIQRVLMSCYGIPMLFLISSIFSGSVQTFYFAAYNYG
NLDQSLEIANVIWPIYCVSLMVILTITCQKTMDERFEMLTHVEGLLLDPKICIKTKKELKGF
SSLLSKLKVDFSVCGVFSLNLLFLFSILGIILSYIILMCQIS
>BgerGr277P
MTHPEMNLLSALKPVYSVSLIFGLVNFTVDNDKIKFPIFKLFLTGFYITIVTLGPALFFLLSA
EDYCDGNPLKLCLADTAYILTNYSTSILYYLSCGIYKRRELGRIINELIHIDEMFLNEQEKFT
VQSSAKRYSZIILIYVITANSVNNICYISSKCESFTWNCVNDIQECLSGFINSLLIVNFVTMVIA
IRERFKVINKRLLGHYEHLEGYGTGNIIVGYFLRSLREKSVTYSSCTGLEVRCYRLVYARLYA
VSKSLTSFYGLALVSSIFWIFLSTVDAFYTAAYLLGKSDTDYTIFSLNIAWSIYCVSLMVILTV
SCYKSVEESLKTVFNVEILLLQSGLPIEAKEELNAMSWQLNRLKISFIICGLFSLNLSFLCST
MGVIASYIIVLSQIG
>BgerGr278
MATNSGQDVNQALQPLIYFMQTFGIFRFTVGRKKTFLDCLKMMWTILVMSMVGIGPVLY
FVLKLHVYCNGVLLKFCLWDTLYIISTYASPILFLFIYGLCKKRKMYGLIQELTEIDQTFFSK
TDNNDLQVKFNKFNKLNIAYVIISNVLSAIVYIGTSCPTFSSKCMLNLQDVFNGFVNSLIIVH
FVAIVTALRYRIEIINKRLIGYYRHIEGRYIETVTMGDFIKTSRLRNARSSSCTGHQVHSLRL
LYSKLYGISKNITSLYGLNLFLVIIWIFLSSIDAFYLGATNKDNYGAVIVSISWTSISISLLVML
TASCDWCVNESLATLYHAEELHLQKTLSVEAKEELNAFSFQVSRLKMDFTVCGFFSLNLK
FFWGTIGTLASYIVIISQFS
>BgerGr279
MAYKTEVEDFISALKPLFYLMQIFGISVLKAKWPLNFFQLIWSIFMMVIVGSGPLVYFTLSP
LDYCKQNPLIACIAKALYMLSSYTSSTYIVIFYGLYKRQEVKSIIKEFIEIDKMFLSKVENCEL
QTKFKRFCVVITTCIVMTNITGDIIYFQSECRSSSHTLVNCAQETISTFINSLMIVSFVTMVT
ALRYRFEIINKRLIGYYRNIQGHSVTTVTMENFVKSCIHNNTRSSSCTGHEVRCLRMFYGKL
YSISRICNSLYGFSLFLVFIWIFLGSIEAFYFGVFSFHQSDVPYVSFAVSLTWSFYSVILLLILSS
ASHLCLVESLATLVHVELILLGSNLSLETKEQLNAFSFQLSRLKIEFSVCGLFSMNMKLLFS
ALGAIISYIIVISQLS

>BgerGr280
MRMYFNPRRKIFVQSAEFDNNLDKKCSSTNFLSSLRLLFNISKGFALVNFSLDQKNINFSSL
RVFWTLLYLFILVIGLIIYFTSTVPEYCTTVPLKLCVSNAAYMVASYSTCILYFILCAIYKRRE
YAKIISEFIQIDDLILSEAENTSVQTDIKKFNIFILVYIIFLNVSDGVFYTASNCETFSLTCINSI
QENLSGFINSLIIVNFVAMVLALRQRFKVINSRLLAHAEDIDGHAKKTTILGDLIQPRQNSF
SSCSAREVRCMRIVYIRLYANSRVIMSFFGFPVLAAIFWIFLSVVDAFYSAAYFLEQGDDDY
VAIGANVLWSFYCLSLMVILTVLCQLSVDESLGTAFHVETLLLDSGLPFDAKVELNAFSFEL
SRLKMDLTVCGLFNLNLPFFCSVMGVIASYIIIISQIN
>BgerGr281
MVPNNIYAKELKFMYYVLQVFGLTNMSFHRNTISFPAIKLFWTLICLFVSLIPPVLYIFFIIL
QEIRYFPVKWLLPHTSNLLIRYITVMLVYLRLGFVKKHQLAKIIKEFQEIDLEFSQVEIQGLR
NRIKKRVSTLLIFVISIPSVINILITNSCSKFMMTCISSAVNFFPIIVNVITVINYSSIVFSIGERF
KIINHRLAHKGSLTGKYNSNNYFQSFLCSPLSASEIRSIKTLYMRLIDISRSLSNCYGVILLGI
NSWIFLCTFSVCYAAMIHFEMHRITFWAASVIVTWAFYALFLVFILCFSCHSTVEANRSTM
FHIQKLLLQRNLTKETMEELRQMALHVTTLKVEFPVCGMFSMNRPFFCRMVGFIISYFIIL
Y
>BgerGr282P
MASPSNGFITSMEPLFKMSQIIGIVTFSIDKKSKDISFPVLRIFWTILVVIFGAVGLVLSFLDY
TLSFQNSNSTKFRISYGIKIYFMYCAFWSSLIYTGLYKRKEILVIIKEFNFVDKELSCITRHPV
KSTIKILSFILVMYFATVKGIDDLVNTASSCKHLSIKCIHSIVZHQNNYVIIFSLILFVSMVTA
MSERHKTANKILSNFNTKFPTNQNSSTNFSEPNLPFKILSISDIQSLRLVHKRLFIISRQLICC
YEPTLLATICWILGGCVHCSYASAYSMQERHGDYSVLVDFVFWVLFCVSMMFILAYICHKA
VEECLMTRYHTQALLLEPGLGYEVKEELKGLHKQLNTMKVEFKICGMLRLNLSFFCNLVG
LICSYIVIMCQFE
>BgerGr283P
MAEISFISSMRPIFLLSQIVGITNFAIDRKNNTITFPFLKTLWTFLFATIGVGSVLLCFAFSPS
FFDYQVSVKLYLSHNIKQFFKFFTYISSLIFSGLYRRRQNLIILKEFQSADMLLYSLVGYKAPS
RVKKLSTYLAAFLVSIKFIDDFLYISMKCKDFSIRCISSVVYQINIYTALVVILVYISIVLSLTER
YKTTNKILSNFNTKLKPHPNITFVTADRTTTSPSVGDIRNLRQIHKCLIVISRALLTCYGLPI
FAVTGWIFFSCVHCFYVSAHYLQKGYQDYVVVXSDTLVNFCVLLIYFLALTSQLAVDEFRK
TGVHIQILLLDSNLANGVRDELNIMDLQLKSVKLEFAICEMFRLNLSFLCNFVGIICSYIVIM
CQLQ
>BgerGr284
MVRPSTVFTSSLKPIFLISKVFGLTPSSIRRKTNNVTIPFSGICWTTTLVILGAIGLLLCFFQN
TSEFNYSKSLKLSISYCIKLVLKYISYFSTLLYSGIYKRQEIVTITKEFQYIDHELVRNGYTIET
KVKKLSIMLIAYLTLIKFTDDVMDIISMCQLSSLKCFHMVIYDFNSYEIFVMAVIYVTIVLAV
RERYRAANKILSNFSTNFKTAPNVGAIISDPTLTIIKPWAIISKPDIQILRQVYRSLFTISNSL
VSCYGVSLLAAISWIFIGGVYCSYTTAYSLQVESKTIVVIVDMGLWAIFCVSLMFVLTISCQL
TMDEHRATVVHVQTLLLEPSLGNELKNELNAMNTQLTSMNLEFRICNMFRLDVTFLCSF
LGIMFSYIIIMCQFE
>BgerGr285
MAQTSTTFVSSMEPIFRVSQIVGLVNFSIDRKTKQIKFSFSRTLWTVFCVMLAVLGFVLYFF
VKSFKFDYSITFKIGISYNIKLFFRYFAFFSSLIISGVYKREQMLVIIKEFQYVDSIFSKLHYKV
DTKVKTVSLILLAYFSFSKLVDDIIDIVTGCKISTIKCIHTTIHDSHSYALFLIVITYVCIVVALK
ERYKTINKILTDLCSNFENVQTCNVISERHRKVVLQWNDSSNYEIQILRQVNKRLFVISRAL
SSCYGVTLFAIVGWAFLGSVHYSYITANSMQGDRENLLMKFDLALGAMFCLNLMFVLSYS

CQLTLDEFYETPVHIQTLLLTPSLRSGMKEELNAMNSQMNSLKLEFRVCSIFRLDLTFFCN
FLGIMCSYIIIMYQFE
>BgerGr286
MARISNTFISTMGPIFFISKTIGLINFEIDKNRKIIKFPFFRLLWTILFLIYGMIGFVLCVTYKT
FYFNYTPTLKVGISYGIKLLIRYFSYYSSFFYTGILKRQEFVTIFHEFQAIDQELSKIGYSVESN
VKNLSLLLLSYLVLIKFIDDMIDIVSICHYSATKCIHTIIYNSNSYAILIMVNTYVCIVLEIKERY
KIINRILSDFCASFKTVHQKTFVISERNIKCVSPWNLPTVSDIHVLRQVYRKLFIVSRALLSC
YGLSLISAIGWLFVATVHYSYITAYFLQGGSERYIIILDTGVWSLFCVSLMFVLTLSCHLAMD
ECRTSGVHIQTLLLEPNLRSDVKEELNAMNVQLATLKLELKVCGMFRVDLNFFCNFVGIIC
SYLIIMYQFE
>BgerGr287
MARMSNTFISTMQPIFFISKIIGLMNFSIDKNRKKIKFPFFRLLWTILFLIYGTIGFVLCVTYR
TFYVNYTPPLKVGISYGITLLLRYFSYYSSLFYTGIIKRHEFVTIFHEFQAVDQELSKIGYSVE
SNVKNLSLLLLSYLVLIKFIDDVIDMVSTCQYSTMKCIRTIIYDSNSYAILIMVVTYVCIVLET
KERYKTINRILSDFSASFQAVHQKTFAISERNITYVSPWNLPTVSDIRGLRQVYRRLFTVSR
ALLSCYGLSLISAIGWIFVGCVHYSYITAYFLQGESEPYIIILDTGVWSVFCVSLMFVLTLSCH
LAMDECRTSGVHIHTLLLEPNLGTDVKEELIAMNSQLASLKLELKICGVFRVDLNFFCNFV
GIMCSYIIIMYQFE
>BgerGr288
MIRPSSKIITELKTVFFMSKVFGLINFSVQGCKVSFPVPNLLWSLICFCALISSFVVIALFVFA
VEKARLSLIVFIPRLLYVISMYLVSTSTIMLSGVYKRRQVMNILEEFFKIDDLLYNQIGCSHH
KSNLNTINLILVLYMFIAGGALELQFILKSCGSISWKCFNAFSQTYMAFVCSLMIFNFVAFAL
SIHDRYKTINKRLIYYCEKFDEQPNKAFTVSIFNPKVVKLDEVFSSPSLHEIRCLRLIYRKLNI
ISSTLTSCYSFPLLFFICCVFINSVGLLYMVFFKFNFLSVGYGVIMWCLYLFSLVFILLLVCQL
VLDESNSTMTLVQTLVSKPNLNSRAADELKDMNLHSSGMKITFSIFGLFSLDMSFLSAFLG
MVASYLVIMCQIN
>BgerGr289
MALHNSITMIHPTNNMISTIKPLLVVAQIFGLINLSFNKNKIYFPKFRLILTILFSLFCVISFV
GYFIFLITYINLPTKIIVSRTLFLFFNDFASFASFVFTGVYKRKHFALLIEDFLMIDELLVLRW
GSVEYRKVQRNLKHIVLVLLAYPLLIGSSLKLVVFGNSSLEAFWMYLYFVLDSTFEIINALTI
VNFVSFVVFIRGRYQLINKYLFDQYITPERYTLNDFLANDGPKIVKCIKLSADDMRFIRFVY
KKLYTTCGLLSSIYGFPLLFGIFWILSCSLTIFYFSVYFMKVNSWLDYFIFAWCAYCIALLVIL
TISCNLTVEEGAKCMVHVQALLVHSGVSNEATDELKDMSFHSSRFNVEFSVCGLFSINSSL
LCTFVGALVSYYVVLCQFD
>BgerGr290
MNVPKTKLISTIKPLLLISQLFGLINFSLNKNSTSLVKIKIFWTLLYSAVSLSGIVLYYITVLPD
NHFTLKIGIAQSLFISFNFISSLTSLIYTGIYQRQQLKKLIKEFLEIDDVLFSQWGGFVYSKEQ
KKLRNIFVCLLFYVIVFGIIVKGLIMNAFRGLNTLQWVHAGLECISGIINSIVILNYVTLVLAL
RVNYRVINAYLFEQFLNSETRHMGLSSKFPPKSMMVSSRSMSVGEIRLLRSIYRNLYTYSGS
LSNCYGLTLLFITLWIVLNALSVFYFAVSYIDLIKHSYYVVFAWCLYCILLLVILTASCHVTVE
ESLKCIVNIQTLLMCSSLSSEVLEELKAMSIHSSSFNVEFSVCGMFNLNLPFLCTVVGSLVSY
FVIMCQFN
>BgerGr291
MNMPTSNFLSTMKPVFFVSQVFGLINFSYSRNYLSFSVLRLSWTLLLSFFSSLGLLVYITFIL
PTETIPLKLIVVRTLFYISQVFTAVASLIFSGIYKRNEFAKIVEEFKKIDDILWLFTVKHKRIY
VDSNLKIYYFALLAYIFCVGFVVDVVYICDDGFFWGTLQKTMDTFSSIVNSIVIVNFVIMVL

AVRERLRMINELLCKELQIQEKSTIDSTIRTNAIPKCTIKVIKSLSSHEVRVIRLIYRNMYTVS
RMLMTYFGFPLFGVVLWVFLSCLCVFYFGIYSFNSKITGISSLVWSLYCFVLFFILSLSCHLT
EDENYDVMVHVESLLVYSNLSSDVLEELKVFNIHSGSLKIRFSVCGLFNLGLPCLCTLAGVI
TSYLLVMYQIN
>BgerGr292
MSLPESKLVSSMKPVLLVSQFFGLVNISSNEHNIYLPIFKLACTLVLASFIVLDALVYIIVFLP
NEAIPLKLIVVNVLFNSLQNFTAVTSLIFTAIYRANLFVKIIEALVKIDGILFSFIDKFESYKSH
RTIRRYCFSLLSFTTLVGCVLNLVFICSDEISLYTLNKLLSSLSSIINFLMIINYVIIVLVIHERC
RIINKLLSKQLANIQVIQIDSTIRTNFLQNYTSQVKTQIISYRDVQYIRFVYQSLYDVSRLLST
CYGFSIFCATLWIFLSCLCAFYSGIYSSDAKVFTLAVFWCFYCIVIFFILTLSCHVTEGENYDV
MVHVQRLILHSSLSSDVLDELRDFNMHSGNLKMKFTVCGMFNLGLPCLCTLAGVLVSYLL
VMYQLN
>BgerGr293
MCTSTRNFIATIKPVFFASQVFGLTNFSLDEKLSFPVPRLLWTLLLGLLNIFGSLVYFVLHL
PYDEKPLKLVLTHSLFLAAKDITTILSFIFVGVYKRRHLSTIIKDFLKVDEILSSEAKFLHRSN
AHCKLLKYSFLLLTYTFLLGGTLEVSFIVIRCCGFVMPSGCAHTTFDALSSFINSVMFINFLV
MVLAMLEKYRVINNILQDQLNSLSIYPRQTSSTTDSNPMCSYPCEYLTVQEIHSIRLVSRKL
YSVSQLLMACYGLPLLFGILWILMESFTVLYFTIYFSGIDQLSILYVFGSLYHLLFLFLLTSVC
HATVDASKDCMVHVQTLLLYSELPNEVLEQLREMSLHSGWFRIEFSVCGWFSINLPFFCS
VVGVIVTYFLIMYQIN
>BgerGr294
MTSKNAFISTLRPMFLISQAFGLTNFSSNSPSTLQQFWTYILASLSASGSIVYVVLVFPYEEL
PLKLIVARSLFFFTQGYTSVICLTYSGLFRRIQLNKVVEELVKIDEILSPFVINHRSRLKKWY
FGLLVYVVCLGFVIDVVYIFSKPFSWGHIHQVLDTMSSNINSVMIVNFIIMALAIKERLVIIN
KFLSQQLHYLDDLPEMATSVFITKSKPCTFIKFHQLHTLRLVYKKLFFVSRLLTTCYGLPLF
CTVAWVLLGTLTCFCMGIYTLDAKSFSISSLVWSLYCLVLLFFVALTCNVTEEEANEFLVH
VQSLIVNTNLPNDVLDELNDISVHSHSLKIHFSVCGLFNMNLPFLCTVIGVLSSYLLIMYQL
N
>BgerGr295
MTEKTSNLISEMKPVLFVSQVFGLINFTLNKIFSFSLLRTTWTIILALLSIFGLFICYTIIVPYQ
DMSFKMKVFRFLFHTSQFCTTVACLIYSGIYKRKQLVKALEEIIKIDEILWSEFESQNSNLK
KHSLILLVAIFFVGVFLDVTYYYIPAVTRDIIFGVFYTVSSIIDSLTVAIFILMVEAVRERLEIV
NKQLSDQLKALEVLQATKMREFMPKYRPRFINSLPFLSFHKLQTVRFIYRKLYIVCQLLSSC
FGFALLCTTLWVLCAILTVSYYGIYSLQNKILGTSSLIWSLYCAFLFFALCLSCNATQDQASK
FLEHVQSLIMYSTLPSDVLDELKDISVHSSSLKIRFSVCGMFNLNLKLLCTFASIISSYWVIM
YQIN
>BgerGr296
MFPSRKKTLSVQGYQLAEEYRRFNTSKFVIMNTNSYVKTMKPVFFVSRLFGLINFRIGKQI
SFPNLLLSWTILLAIVSLSSILVYLVLVIPNEAFPVKILTTRTLFFCIQNFTAISCLIFSGIYKRR
EIVKTVEEFHKIDETLSMYFGSPQTNLRKYYYILLAYVILVGVNSDVIFIYSMPYSWDRFHQI
CDTISSTINSLLVVNVSIMALAIRDRLKFLNKILSDQLLVHEIHPEKTSMTSGSNRKYIPKMI
KSCNTLTCHELCAVGFIYRKLYTVCQLLSTCYGFALFCSILWTFLGSLTVFYYGMYFIGSKTI
GISSVIWSLYCVGLFFVLTLSCHLAEHEGCQFLVQVQTLILHSSLPSDVVEKLKDMSMHSTN
LRIQFSICGLFNLNLPFSCTIISVVISYLLILYQLN
>BgerGr297

MNTPRSNFLATMKPMIFVSQFFGFINFSITENNTRFPKLRLCWTLVLALLSILMGSHLVLV
HPYDIPFKLHVLQDFFYSTLCFTVVSSYIFTGVYKRKELVKTLEEFMKVDEDLWYYGGNTR
TNLRKYYYILLAYIFFVRVLVDFVLTSLIPFSDTYVHQLFGTVILALNCVLIANFVLLVLALR
ERLITLNTFLSDQLRFLDSVSAHTTMACVPKNTTRINPSRAITYHQAQYMRIIYRKLYSICQ
LLTSTYSIPLLSSILWILFGSLSVLYFGIVTSEPVSLMWSLYCLFLFFILTLVCNVTEDEASGFL
GHVQTLLLDTSLPNDVLQELKDISVHTSNFKIRFQICGLFNLNLPFVCTIVGVITSYMIIIHQF
N
>BgerGr298
MSTSASYFNSVMRPLIMVSQAFGLIHFNNNKNNNTCFQIAEMFWTLLLALLCSSGTVVYIV
FVMPNEDFPLKLIVLRTLFFLSVGSTAVTSLIYSAIFKRRQLEKTLDEFVKIDNALWPYTAN
NKILKKYYFALLAYIVFIGFSLYIVFIYDLPFCYEYIHQVSDMVSSFITTLMIINFVTLVLAIRE
RFKIINTLLLDQLEELEEHPMKISMTSQSNPKQVPSFSLLSRHELRCIRHVHRKLYSVCNLL
MKCYSFPLFCAILWVLLGSLSVFHNAIIYSLWRSTFGISSLGWDIYCIVLILILTLSCHVTEDQ
SREFILNVQTLILHSSLSSDVLEELRDISVHSGSMKIQFSICGLFNLNLPFFCTLVGVIMSYLL
VMFQLQ
>BgerGr299
MKSSTNNFIVTMKPLFVISQVFGLINFSLEKGISFPVVRLLWTVLLGSVNFVGSLIYFVFFLP
YDPHPLKLVVTHTLFLACNDLTSFLTFIFTGIHKRKEVRAILKEFLRIDEILCYEDFNTTKV
HLSLRKYIYILLSYAVFAGGTLNCLYIVSRCKDLLRPAGCVHTIFDAFSSFINYLMFVNIIILVL
ALRERYRVITLLLCEQIHIFETFPRKVSKYRPSLFPYKYITTNQIISIRLAYSKLYKISRLLMNC
YGAPLLFGILWILMKSFTLLYFTVYTSGRNSIGIGSVLGSVCDLIFLLVLTISCQLTIDASQEC
MVNVQTLLLHSVRFSDTSEQLQEMSLHSRGFRVQFSVCGLFCIDLSFLCSVIGVIVSYFFLLC
QLN
>BgerGr300
MNSPDKFITSVKPLFYVANILGLANFTFDKNQIVFSTVLYIWSIFCLFVPIIGFSLFFYFVIYE
NPDSLSFKIRLSTALYGVASYITSIFNYIFLSFYKRKKLATIIKSFKEVDALLTSTIGITDFKITL
KNTSVRLIAYFFITNALESILYFLVSSETSKWQSLSSVFQSYIAFANSITVAYFVTMVMAVRE
RLKIVNKCLSSTNKNTAFKKNCIQPVLILTNKHRSLIPFSKVRIIRRVYEKLFEISLMLSSCFG
VPLLIANLWIFLDSVDTFYTTAAIFQKEEIDFVEIAQLCIWTVFLLSLMLVICASCQITMNEV
YEANFHIEYQLLHSNFYSATFDELEELMCQINTLKIQFSAAGFFTFNLSLVCAMLGTIITYIII
MCQM
>BgerGr301
MPSMDFVSKMKPVFCITKLFGVTNFYVEKNRIYFNVFNLYWTILCFILFCIGSAFFMAFSIA
DTTYTLKLKVIDVLQDISSVSTGILYFVFPGICMRRRYAKIIQEFISIDTVQLTKTQNKNILKQ
YPFLLILYAVFGNLSCDVVFMITLYKSPPTDVLSGLLDCFIAFINSLLIVHYVAIVMAVRERL
KSINKCLSEQYGKFEGHEKKMYMKPVFSRSAKLYPCVFSQKLHQLRCVYERIYLVSLDLSSC
FGIPLLSAIFWTFLNTIQAFYLSAFSFGKDLGYREILQECLWCFMAISLMIMLTTCCQVALN
ENYKTVHLVETMLMRSDIDPDTEAELKGFRSQLNTLPMEFSTCGFFSLDLSFLYNIFGTLC
TYIIIMCQFNN
>BgerGr302P
MSSIDFITSLKPIFYFTNIFGLTNFSINVTGIHFPIVRQFWTIIWLLLAISGTISEYFNNSISNSL
PLKLRIITILFAMSSFLTVLLYYIFVGIFKRHYFLKIVKNFKEIDMFFKTETKKPKKHAKAVA
ISTNFVVYXVLNSCMFIIYMTTFHGLSSVKTIQGSFRCLVCFLNSLLIVHYVAMVIAVRQRFKI
FNKCFSGHIKNQEGREMKLYMKFQTDKQSTSISYNEIKNFRRVHEKLYEISQHLSSFFGFPI
LMAMLWIFLCIIQAFYKTIYFVNEGHFPFGVVLHKGFLTAICTSLLIILTFSCHVTVDESYKT

MYILMNLILNSDLNEQMKYELKELRSQLNCLRIEFSVCGFFNINFPFLCSICGAVCTYLIIMC
QFNN
>BgerGr303
MSSMDFITSLKPVFCVTNIFGLSNFSINENGIYFPVFRIFLWIFWLSLMFLTACWGYYDNM
LDNSTPLKFKICLNLYGTAHFLTSILYFIFPGFVTRHCFVDLVKELKNIDIYFQNGTTARKN
VFKQCPFILIMYITFVNVSDDVMYIKKAYDASIYNLFQAILQCFVTFINCLLIVHFVSIVTAVR
ERFKMMNKCLSGHCKNQEGREKKSSVQPTFIRREIQLSSISHHDVRHLRCVHEKLYRVSQI
LATYFGIPLLCAKLWIFLGCVEAFYVSAYSFGSENIDYSTLVEECVWTFMSASLMVILTGAC
HVTVDEVYKTVSHVEYILLNSVLDSATEGELKEFRSQLNSLRIEFSACGFFTFNLPFLCGIFG
AVCSYIIILCQFNN
>BgerGr304P
KCRLWIFTSLKPIFYFTNIFGLTNFSINVTGIHFPIVRLFWTIIWLLLAIZGTISEYFNNSISNS
LPLKLKIITILFAMSSFLTVLLYCIFVGIFKRHNFLKIVKNFKEIDMFFKTEPKKPKNMLKQS
PFLLILFIIVINSCMFIICMTTFHGSSAVKAIQGSFRCLVCFLNSLLIVHYVAMVIAVRERFKIF
NKCLSGHSKNQEGRDMKLYMKIQTEKQSTSISYHEINKFRRVHEKLYEISQHLSSFFGFPIL
MAMLWIFLCIVQAFYKTIYFINEGHFHFGVVLHKGFLTAICTSLLIILTFSCHVTVDKSYKT
MHILMILILNSDLNEEMKYELKELRSQLNCLRIEFSVCGFFNINFPFLCSICGAVCTYIIIMCQ
FNN
>BgerGr305
MSSVDFITSLNPVFCVTNIFGLSNFSINENGIYFPVFRIFLWIFWLSLMFLTACWGYYDNML
DNSTPLKFKICLNLYGTAHFLTSILYFIFPGFVTRRCFVDLVKELKNIDIYFQNGTTVRKNIF
KQCPFILIVYITFVNISDDVIYIKKAYEASMYNFLQAILQCFVSFINCLLIVHFVAIVTAVRERF
KMMNKCLTGHCKNQEGREKKFSVQPAFIRREIQLSSISYHDVRHLRCVHEKLYRVSQILAT
YFGIPLLCATLWIFLGCVEAFYVSAYSFDSENIDYGTMLEECVWTLMCASLMVILTSACHV
TVDEVYKTVSHVEYILLNSVLDKATEGELKEFRSQLNSLRIEFSACGFFTFNLPFLCGIFGAV
CSYIIILCQFNN
>BgerGr306
MSSMDFITSLKPIFYVTNIFGLTNFSINEKGMYFPIGRFMWTIFCLLVNFLPPVFYYMYNVF
ENSYPLKLKIIISLYETFHFLTLILYYFFVAVFMRKEFVKIVKEFNKVDALFGCLCVNVEEKL
LIRQYPFMLVVYIIVGNGLDDILFIIQNYGSPISKFMQSLCDCYISFLISLLIVHYVAMVMAVR
ERFKMINKCLSGHCKNLGGHGKKFSVQPAFIRSQRHFSSFSCHDVRQLRRVHERLYSVCQS
VGTCFGIPLLSVTLWIFLSTVQTFYVSAYSVQYENTEHSKIHVIQESIWCFFSTSLVVILTASC
HATVDEVSETILQVECVRLHRELDRDTEEELKEFKTQLNRLRIEFSTCGFFSLNLPFLCSIL
GAVCTYIIIMCQFNNA
>BgerGr307
MIQTETNVISALKLLFCVSLVLGLNNLSFYRNTVSFRVRKLLWTYFCVFLTALGPVIYCTFI
MSSHYVLYPLKISIARSMYLTACYTTSLISYLFLSVYKRREMASIIDTLLKIDNLLLVQREET
TVHLKVFSILLTSFVTLVGTILYFVRFLAQCNGFSWGCLHWLINYLSCLTNFMMIANYITIL
FAIRQRFNVINKRLYIYHCELDDDLKSKNTVGFDLSNRLLRSSFGSLSRRQVRCFRIVFSDL
HAVSETLTACYGLPILSAIVWIFLCCVAVFYLASFDYWMHPYIAYNNIAWTVFSLILIAVLS
GSCHWTIEASRATVAHVQSLLLSSKLSNETKEELRDMSCHLNSIKVKFPVCGFFNLNFSFF
TTFVGVIFSYIVIMLQLT
>BgerGr308P
MVGNIQSNLLSSLKIVLYFLQGFGMVSLRTGENKMHFSRMRIIWTAVHLCIIVFAFVTLFLY
RIPRDCHVMIPKVCITNIVYLVSSFTPAILYFIFCAIRKRRELVAIIDRFNEIDDIXISDWNEN
GGFQINLKKRSLLLLFYAAIANLVIVGIHSANNQCLTFSWSCVNNLLEIYSGIINSLVIISFVA

MVTAIRQRLQIITKILTGLVQNIDGYEKTIVNDFVHTIRRKRVVFSPNFDRKVHFLRIIYGKL
YRLSQILTTYYGIPVFSATFWILLSAVFAIYAATYFLAEEHVKTAIGICISWTSFCLSLMLILC
VTCDFCAYESDTALLQVDEILLNSELSVLTREELKGLAIQFVRFRMDFNICGMFSLNLPFM
CSIMGVIASYVIIMSQFN
>BgerGr309
MAVKTKNRLFSDLKIPFYILRGFGMLNLTTDENRVSASVLSFMWTVVHICVVFIMMFVFRI
PHDCEASSRKVCIANAVYLVSSYTTTILYFLVCTVWRRKDLIAIFDRLNQIDIISVSLNITSVF
EINLKKRSIMLILYGVIGLLVLGGIHAATGCSDPISWKCVTFILEIYSGIINSLVIVSFVTMVIAI
RQRLQIINKILSSFDIQGSGEKTFMGDFVHTIRYKRFSSNNGREVHCLRLIYGKLYTVSQML
TNYFGIPILSATAWIFLSAVYAFYIAMYLSGLEHLSYYDIADNVSWTGFCLSLLMILCIACDF
CARESGTTVHLVEELLLNSDLCLETREELNAMGEQLARLKMEFNICGMFSLNLPFMCKYR
WSHSILYHHYQ
>BgerGr310
MVGNIQSNLLSSLKIVFYFLQGFGMVSLTTGENKMQFSRMRIFWTAVHLCMIVSAFVTLFI
FRISRDCHVMTRKLCITNTVYLVSSFTAAILYFIFCAIRKRRDLVAIIDRFNEIDDIISQTWNE
NGGFQINLKKRSLLLLFYVAIANLIIGGLHTATDYCLTFSWSCVNNLLEIYSGVINSLVIISFV
AMVTAVRQRLQTITKILTGLVQNIDGYEKTIVSDFVHTIRHKRVVLSSNYDPEVHCLRIIYG
KLYRLSQMLTTYYGIPVLSASSWILLSAVYAFYAATYLLGQQPVKYTVIGVCISWTSFCLSL
MLILCVTCDFCGYESDTALLQVDEILLNSELSVLTREELKGLAIQFVRFRMDFNICGMFSLN
LPFMCSIMGVIASYVIIMSQLN
>BgerGr311
MVGNTQNPFLSATKIVFNFLQGIGMVNFTIDKNKIYFTRLRILWTSVHVCLIVFSFITLGLRI
IEDCQVTSRKLCIVSTAYLISTYTTAILYFIICAIRKRKQLVAIIDRFKQIDDVIAGIQSNSGDID
NYKKYNILFVLYGISGNVIVGGLHATTDFCKPVSWSCVNVILETYAGTVNSLAIVSFVVMVI
SVRQRLQVITKTLAGFGQNIEGLHKKTMVGDFVQTIRHKRTAFSSNYIREVHCLRLIYSGLF
AVSQMLSSYAGVPLLTGFVWIFLSAVYAFYSAAFLFGEENVEYTYIAECIIWTVFCFSLMVIL
CMTCGFCAQEGDTPVHQIEELLLNSELSVQTKEELNSMGVQLARLKMEFNICGMFSLNLP
FMCSIIGVIASYIIIMTQIN
>BgerGr312
MIQPKTKLIFCSKWLFYASQVFGIINFNVETFFFIRLLWTISLAVFCAVTPFLYYTMLALPN
LETTPLQNLIADILYTSSIPITSVLYLVHTGIFKRIQLAKVTDELGQIDDVLFSRRESASRNTS
KTMSLVLFAYVALTNLVLGTLFIPKHCRSPTWSCLYNVQECLCNILNSLMVVNFVSVVLVI
LQRFKAIRKVLSGQIDETIVTCRQVLKLRVACKQLFEVQRTLMSCYEVPLIVQIFCIFSVSVE
TFYYDAYNFGKGELEQQIANVIWPIYCVSLIVIPTISCQKTVDETFEVLTRVESLLLDPRLCI
KTKAELKNFSSQLNKLKLEFSVCGVFNLNVAFLFSILGIIVSYVILMCQLG
>BgerGr313
MVTNPQSYFLSASKIVFNILQGFGMVNITIDKDRIHLSNPRLLWTMFHLCVILFGFVAMLH
FRISVDCPELSLKICVAGTAYLVSSYTTSILYFITCAIRKRKELASIINRFSQIDIFISLTLNQNG
LFQINANKHSLQFLIYAASANLIIGAIHSSSGYCDVFSWSCINSLLENYVGTINSLVVVSFVT
MVISIRQRLQVISKILTGLAQNTEGYHMKPFMSDFVRTIRLKRVIFSSNYDREVHCLRIVYG
KLYRLSQMFTSHYEVPVFSAIAWIFLSAVYAAFFVGQENVDYLIIADCVLWTVFCLSLVMIL
CAACSLCAREGDSLVMQVEELLLNSDLGLETRDELNAMGMQLTRLKMEFNIFGMFSLNLP
FMCSIMGVVASYVVIMSQMN
>BgerGr314
MVGNHQNRLISALKIVFRFLQGFGMVSFTIDKSKINFSQRRVFWTISHIYIILFAFVTMFFFR
VPNDCEEKSLKLCIASTAYLVSTYSTAILYFIFCAIRKRRQLVALIDLFNEIDDIMSLWLNEN

GDFLINLKTRSLLFLFYVVIANLIIGGLHNITDFCRPISWSCVNCILESYSGTINSLVIVSFVTM
VIAIRQRLQVINKNLTGFGNNTEGYQKKTSDKRVAFSSNYDHEVRCLRLLYGRIYTLSQKL
TSYYGIPVCSATVWIFLSAVYAFYAAAYLLGQENIDYVYIADNLAWTVFCLSLMVILCVTCD
FCAREGGIAVQHVEELLLTSELSVQTKEELNAMGVQLTRLKLEFSICGMFSLNLPFMCSIVG
VIASYVIIMSQFN
>BgerGr315
MVSFTTDKIKINFSNLRIFWTIVHICVTLFGFITMLVFRVPNDCQQTPFKLCIANTVYLVST
YTASILYFIICAIHKRRDLVAIIDRFKQVDDLIFLTLNQNCDYQINLKKRSLLFLLYAVILNLII
GAIHSTTGYCEPISWSCVNSALEVYTGTINSLVIVTFVTMVIALWQRLQMINKILNSLGRDI
EGSGKKTIVGDFVQTIRHKRVVFSSNYDREVHCLRLIYGRMYTVSQMLTNYYGIPVLSAIV
WIFLSAVYAFYAAMYLFERESVEYEFIADCIAWTVFCLTLLVILCVTCDFCAREGGTTVHLV
EELLLNSDLGLETREELNAMGVQLTRLKMEFNICGMFSLNMPFMSSIMGVIASYVIIMSQM
ND
>BgerGr316
MGKSTVLSRQSSDLMFCSKWLFYTSQIFGLINFGRGDTLFFLRLFWAISLLVFYALTPVLYF
TVVAFEVLKTSPLKIRIVDVLHTSSISLTSVLYLIHTGVFKRGQLSNIADEFDQIDDILCSRRE
SFSRNTSKRTSLMLFAYVSLTVLVVETVCISIKCQSPNWWCLYIVQDCFSSFLNSLMVVNFV
SVVFAIRERFKTINKILSGHVFTKGHYQIVTCREVFNLRVVFGKLFEVHRSVMTCFGVPMM
FQILSIFSGTVQTFYVAAFNLGKKQGALDFVNIIWPIYCVSLMVILTISCQKTVDVWYDVLA
QVEGLLLEPRLCDRTKEELEDFSSQLSKLKVEFSVCGVFSLNLPFLFSSLGIILSYIILLSQIS
>BgerGr317
MSTSDFMARLKPLLFVTNFFGVTNFSIKEDKYNFPTARLIWTFLCMAVIIIGAGLYYAFNIF
DHSYPLKLTIIKILYDSSHFLTAILYYIFIGMCKRRQCARIIQELLKTDNYLVFQPKNTKVERI
LSQYPLLLLLYIVIGNVCDDLIFIILSFESRPWKFVQSLIDCLTAFINSLLIAHYVSMVMAVRE
KLKAINKCLSGHRREIKDNKQMQPLFIRKQKKMNSLTCEEVFYLRCVHKTLYEVGKTLSST
FGIPLLCAILYIFLSSVEAFYLAAYIFQEGIHDYVKIIQESLWSLYCLSLILVLTMFCNLASNEN
DKTAYEIECVLLYSNLEPETEKELKEMRSQVNSLTIEFSVYGFFSLNFSFLNSTLAAMCTYII
IMCQFNN
>BgerGr318
MTTPGIDFILSMKPVFVISQLFGFSNVSSERDVHISFIRVFWTLLWISAIPVIFFEYVIYASVE
DYISIPTKLFIAYSFFLVSYNCTAVVSILYSGILKRRELEEIFDDLLRTNELLATKFYICDSRSK
LRKHLVILLIYAFLQNCCYDIAYLRLACGAVSYTCVHCVMHCCLNVVVLLMMAQFISLVLCI
LETLKLINNLLTVGCHKTDPHCSVSVSKVRCLRSVYGALYASSQSLSNCYGAPLLCATASIFF
LSMMTFYYALYSLQMEPVQYDYVTYSVLWGFYQASCVVILVVPCQMILDQQRSMVYRIET
LLLHSNLPKETEEELTKFSSQITKLKIEFCVCGFFSLDRPFLCGLFGVIASYIVIMCQFPT
>BgerGr319
MASSSNAFLSSIKPIFRISQVFGLSDITSNKKLYFPYLRAFGALLCICVILGDFIFYFKFVSLEE
VQSFPVEILWAYCFYLTVYNLTVVTSIVYSNSFKNQQLKCIFDELLYCNELLTSKFKVGELG
NKVRNVALGFLVYVFLQRLSNDVYAIKGACGLYPKNCIHSVMQTFVGLAYSLTMIRFVSLV
LFMLQTLKLVNKILSDIDKEDTFRCFVPDSSIRCLRHVYGKLHGISQSLSSCFDLPLLLTCTSI
SFMVIDTFYYSLYGFRGKLFSYVDLLPHVFWGLYHASLIFPLAVPCQMTIDESFTIMCRLES
LILHTNLSSETENELSKMSSQIGKLKIEFSVCGLFTLDLPFVCGFFGLLLSYIVVVSQYSS
>BgerGr320
MTSEHAFILSLKPLFYFSQFFGLSDFSLNKKTQFPYLYILWPVLCLSLLLAMSVLYFTFIVVE
DYIAIPTRLFYAHCMFLSTYVTTVAVSILYSSMFKNNELKHIFQELVYYNKLLVSTFKVKKM
GNGVRYLNIGMLGYLFLQKFANDIFNIIFTCRKLSLRCVCGVLQSFSGFVYLLTIVHFTSLVF

FILETLKLVNKVISDMFQEDNFHAYVPVAKIRCLRLVDGKLRIMSQALSSCFGVPLLLITVC
TFFISIVTFYYALFSLHNYSFTLVDILPAALWGFYHASFIFIVTLACQMTMNESSAIMYRIES
LLLESKLPTEVEDELTKMSSQIRKLKIQFSVCGFFTLDLPFVCGFFGVLLSYIVLVSQQVA
>BgerGr321
MSSTSKVIISAMQPVFYVLQIFGLVNFTVHEHGISFPARKLLWSILWTLFFGLGPVLYTALR
FQNRFEGSVKLYVATVLYSASNYFTALTSFLYPAIFRKRRLGKVLLEFIKCDSILSSLSKDST
KKNRLNKVLFLLLSYVVAVNACDSGLYAFLECRKYVTHRCVHSTIEGLALVINSLLIVNYVS
YVLAMKEMYALINKRLCQYCHKLSFFSSPSIREVRSLRLVHGRLFAVSQTLSHCYGVPLLGV
FSWMLLSCVTTFYMSTNQLENQSNDFREVIINVLWSIYCGSLMVILTSSCHSTVEESKMTV
FRVQSLLLCPNLSFDAEYELKEMRSQLRALKIDFSVCGLFSINLPFLSTIVGVTASYIIIMCQV
>BgerGr322
MVLQESNAYSTMKPVFLVSQVFGLINFSLNTKQTSCIRLVYTIATLAVVTSGLILYFAVLIFE
DYNDLLLKQLLAYICYMSTAHISAILSYVFISIYKRQKLANILKEFDQMVDLLNSQNFKSKSS
LKVFLTTFLVYIITGNIADDAFYVDSSCKMSASHCVHSLIDCLSALVSSLMILTYVSIVLSMR
EILKTINERISGSVVCSREVRCMRSLYQKLLVISREINTIFGVPLIVAISWVFFCSVLAFYFSIY
NLQLEKVPYRMIVVSIVWACICSSLIVLLTVSCHLALDESCSTKIRIQGLLLQPNLSSDVEEE
LKAMNCQLASAKMEFSTSGLFNLNLPFLYAFFGVIASYIIIICQMN
>BgerGr323
MTLSNTKLTSKLKPIFYVSQAIGLITFSLGTKNRYFRCVGALWRIVCSSFITVFFCVFLRSAN
REILDKFTLKLHIADVLYVSANYCSALASLFYLGIYKRSQLQRIIKNFEEIDASITRGSEMSAL
YCKSKMRYLLLLAYVFLIYTFFNAFYMCNSRGFLTNKCADLLAARICSFVASQMIFSYIAFV
LGIQERLHAINKRLSSFPETFQNSKLYLGSSFVAVDMFLARSSASVHEVRFFRLIYVKLQSLS
RDISSCFGVPLLASIVWIFFACVYVLYFLCYFQHYIRSYQDFLSSCMWPFYCTCLIFALTKCC
HLTVDESYTTMFHVQNLLCQPNVPTETISELNAMSGHLNNLKIEFSICGLFSLNLPFLCTIF
GVIVSYIIVISQFS
>BgerGr324
MIGRVNFISAISLIIFASQCVGLFSFSFLRGVFNFPILRLLYTFVFIALNAFGATVYSVFLLYDD
FCFLPIKVFIAHSIYYFFTYFTSVISLVFSGIYRRREVTNVMQDLVDIDCLLNPLVDMGIVHN
RLKHISKLLLAYAALMLAVDCTLSGILVCVPFSPFSWSCPHSFLDSLSGMINSVMLLDFIAV
TLAIRDRFKIINGYLTGASLTSRCTVKLQSLRCVYSRLCALSRALTSCYGVPLFGATAWIFVG
SLETFYHVAHSVQTENDDYEDIIETVLWFVYCISLMIVLTTCCDLTVEESRLTLFNVQKLLV
QSDLSIETQEELKQMSLHSSSLTVEFSACGLFTLNLNFLFAFLGFITSYFIIMYQLN
>BgerGr325
MAPVEPSIITTLKPLLFVLQFLGLTSFSLDKNKFTFSTIRYFYTMLVALLDASGFLTYLLIGIA
SSYDKSPINRFVADILFVSSNYCTSLMYYLITGIWKRRKLCTIMKKFEHIEQDFTQIEFSARY
LKMRKWCLLLLSYVFVVNTSAMLVYTYYVCESFSPYCVTGVIMELSAFLNSLMIVFYVIIVL
VIREWLKVINDIMSKCCQKFEECSTKILFAPGFISSDISSKEILYLRLLYGKLVSISRDLNCYC
GIPLSSAVSWIFFSCVYVFYCLWHDLKTATLVHKMLLVIWPIYCPSLVVILSFCCHITVEESG
STVFYVQTLLLQPNLLPGTREQLNAIGSQLGSLKLEFSTKGIFTLNLKFLFAFAGFILSYIIVM
SQFK
>BgerGr326
MPKSEANFQSTLRPVLLVSQALGITSFKHSPSSRFLQASSTILCLSVLIFGLCVYVFVTVTDS
YKEMTLKFFLAEMVYLIFNYLTAFLCLLTPYIYKSDQIKIMKKFHQIDACSRSVSFEGHRKL
RKRYYLLIGYIIFMTVIVGALYAFQDCKRSPRHCAQAVAGCFSSLINSQMTGHYMALVLAVE
EILKSVNKSLLHYSQMFERYPKKSLALPYKDFVHYSNFVSAHELRTLRLVYAKLQSISRDLS
TYYGMQIAGVICWTFFACVYIFYYFAANIKVEQSTLVMASLIWPLYCLSMIIMLTASAHNTV

AESNTTMFHVQTLLLHSSLSTEAVIELKLLSSQLTCTKLEISAIGLFNLNLSFLATMFGFIFS
YIIIMCQFE
>BgerGr327
MSEDRMSFLSTLKPLVYVSEAVGITNFSLDSHQRLRLTWTIFCASLIGFVSIVFISVDVRNM
YSLLTLKFFVAEVLYSSVSYFTALLYLLTCCIFRRSQMSIIIKKFDQIEAFHRSSFNEASSEAN
RKLQKSYRILLMYVILFLTPDTVLFLYHNCHCMSRFCVQSIIGSYASILNSQITINFIALVLAL
RQRFKYINRRMDNYFQLMEDSLSRDTFGVPSQDIRKLRVLYAKVQSLSVDLMHFYGVPLV
AVIIWLFLSCVYVFYYVAYMIASQSVLRIANSVIWTVYSVTLIIVLTVSCHVTAAETKATMSY
IQNLLLHPNIPTETINELKSMSSQLRCLKLKFSACGVFTLDLTFSRTMFGVIISYIIIMCQFQ
>BgerGr328
MSGGRVSFLSTMKPLVYVSEAVGITNFTLDSHQKLRLIWTCICTLLIASGSLMYISVIVEQIY
GEMSPKLFIADVLYIMANYSTSLLYLSICCIYNRKQLSKMIKKFDQIGPFLRTSDLSVSKEISS
GTHRQLQIYYRILLTYIILFSTPDTVLFLRKQCECLSWICFHATIGNYSSFLNSLITINFIALVL
ALRQRLKYINSRMANYCQIIEDSEQVFPHSRTTDCISSEDIHKLRVLYAKVHSLSEAIVHFY
GVPLAGVIIWLFLACVYVFYYVAYMIASQSVYRIANSVIWTTYIVTLIIVLTVSCHVTAAETK
ATMSYVQNLLLHPNLPTDTRYELKSMSSQLLCLKLQFTTCGLFTLDLFFLRTMFGVIVSYII
IMCQFK
>BgerGr329
MDCANSPTNLISALKPLLYVSQAFGTVSFSLGKNVMNFSCFRLIWTFVFIFVIGTGPILYFTF
TVPEHCESSPLKLCIANIVDTVASYSASFLYLLICGVYKRRELGRIIIEFAQIDETLLSQTEKYE
VRNILRKSSTLVLGYIILVNGPDFIFFISSSCESFSWGCVNTIQESLSAFINSLIIVNFVAMVIA
MRERFKIMNKRLLNHYENSEDSPKKAVIVGDFVKRIRPKNVPIGSHSGREVHCLRLVYGRL
FAVSRTLTSFFGIPVASAILWIFLSAVEAFYSAAFSLGQGNSDYSSVAINVIWSSYCVSLMVIL
TVFCHLNVDETSAMIFNVETLLLHSTFSDESKEELNSMSFQLNRLKLDVTVCGVFNLNLPF
LCSVIGVIASYIVLISQMN
>BgerGr330
MKPVNYISQIFGLNIFLFNTSNLYLFILRLLWSSLNSLLVIVGSVLYFTQSVSGPSAPLTLTIV
RSIYMFLNYCTCILYFFNDIYKRHEFRRLYKAFIEIDCSFLSNSENHKVQINLKKFSSRVLAYI
ILINVLNNAFYINSECAINSWKCVSAVLEIISGFINSLLIVNFIIVVYSTLQRLRVINELLSRHC
QKLEEKQDAIDMVTNFCPKLGNRNIETEYISSSEVHILRLIYLRLHELSQELTTCYGPPMLM
LISWTFICIIEAFYSAIYSMQSDSNNYNFIFINCFWSVYCVSLLIILIVTSQMCVDERLETIFIL
ETLFLQPKLSAEVLEELNAMRCQCSALKVEFEICGLFPLKLSFLCSVLGVIVSYIIVMCQLG
>BgerGr331
MKPVIFISKAFGLLDLSFGTNQAFFHFLGLIWRIVCLLLVIIGGVCNVIDYISIVSIYKGGVQVL
IASIANVLSNFLSSIISLIVLGFHKRQIYEKLIVGLEGSLVREKDFNFKSTCSKFTFLILSYFVGT
NICMYILTAVLRNCSWCNIVCYLFQGMSSIINSLLIINYLDIVIAIRDRLRICNKCLSDYCSLRE
QENRSNMLGFIVRVGQNVISLKTLSPNTVHLLRLEYGCLYAISETLKSCFGVPVLGIVCWIFI
NTVHAFYFVTRYLETGVMEQWRIAVGIVWSISFLLLLFILNLLCHLAGNESKMIICHIQTLV
LDTKLPIETKNELKDMNDQISYLKLKFSICGMFDLNLSFFFSQLCIIASYVILMCQHK
>BgerGr332
MNNSAMNFISGIKPVLNTSRFCGICNTSVDRNVKFPLHQPFWTSLQISLMLLGFILYSVFTL
PVLYDSLPLKILISHAAYSVLGYLVAITSLLFCGIFKRRQLRLTIEEMFEIDCLLFSQEEKFEI
MGKLKKLSAILLTFVILENIADDALYLHEVCHSLTWSCLQGILECISVFMNIVLILLYICIVSA
MREILKIINKRLLQHCNNIEGNMKQFAFVSEFPLTISAVRSSLSGSEVRTFRHVYARLCKISR
MLESCYGFPLLVVILWIFLSSIDAHYIALDIWQKEPINYGSLIMNGLWGVNLIIIFVVLFIPCH

LTSDESFLTSIHVDTILMNSNSVEVKEELIAMRSQLRGLRMNFSVCGFFNLNLQFMCSTLG
AIVSYILLVSQLK
>BgerGr333
MIVSATNFISAIKQVVYISQIFGLSNFHADKNIAFAMIRIFWTLLYLSLMSTGLVLYFMFTFS
ENYNSLILTLFITDAAFTISNYVASVLWVLSCSIFKKRQLRTIIEQFILIDNIVLSRKENIELKK
SLRKFSVLSIYLVVENVIGSADYLRKNCDCFSRKCINSVLGCISVSINFIMVVQFTCIVSVVREI
LKIINKRLLDHCKNIEDYHQTRMFDSHVSQLNSIIIRSSLSGREVRCLRLVYAEQYRISKILES
CYGFPLLNVVFCLFVDSIGAYYDVVDYFQMTSINYMSFVMFILRCVRLLTDITVLVVPCHM
VTDESYETLVQVETLLLSSNLSVEAKEELKAMRSQLRGMRVHLSLCGIFKLNLQFLSSFLGT
IISYCIVVSQLK
>BgerGr334
MYYTKTNHTVSKTRRVTVKKSARIFTESHKSESPFVSSLKPVIIVSQIFGLINFTINKNTIFFP
VLRLVWTVFCTSLIAGGPILYSVFVVPYYYHLFPMKLFVTYYFYLNSGYMTSIISLLYSGIYK
RRHLAQVIQKFMEIDNLLFYQTRDFKGQRKLKNIFLIAIMLIFLTTALYVIYIIARCKYFSWV
CIHALIRSLSGLENSMMTVGFFSIVLSMRERYKVINKLIQQYCLVYQNSEKNVFAVELYPRP
KNWFSQQSSLTVQQVRNLRIVCAKLQEISRGLSTYYGFPLLGIISWSFISFLSLFYLIVFCIET
QAIGYRLMYISIVSCLLLITINFIFVLTCHITVNENFETIFHVQTLLLQPNLPIDVREELNAMC
TQLGIMKIAFSICGLFNLDLAFYFSMLGVLSSYVILMCQLE
>BgerGr335
MYSNSEERKQSVNLDAMAQQSSKFISTMRPVILASQIFGISNFSIDGKTVSFPKLKVIWTLV
MLSICAYFPISFLSIDIEYFHRSYPIKLFVTYRINMVTNNFTSIATLLFTGLYKRREIARIIQEFI
EIDRLFSETKFLQFSNKLKNLFLFFTLYVLLMNFCSSMGFIVLRCTSISLACLHNIIIAICNAI
NMTLLINYIVMVLYIRDKLKHINERLRQYYLQKEAYPDELFILDESPGKTHRITAYVQELHK
FRQVLGKLYELSRKISSCYGLSVLGITLWGSSCIVSAFHFTILFLETQITGYFTIFLVICSCFHC
ELITVALFVSCHLTVDERRETMICVDKLLIQPEFSTEVREELKEMLFHLGTFKIEFSAFGFF
TLNLQFLSTYIGLTCTYIIIIMQLV
>BgerGr336P
MKMTLLPSDFISTIKPVIMFSKVFGLLNFTVNRNKIYFPILGPIWTLLCIGINIFGFTYYYLVT
YLQYYSLLSLKLLLTYCFNISSSYVTSITSLLYCGVFKRQKIPKIIEQFSQIDEMINQSKSFEIN
RKLHKVCIILLTYVFLATGITNTIYLIYQCETSTFKCLHSIIECFSGYINSQIVVTYVVLVFAIRE
RFQSINKWLIDCNEKCNSCAISLHDLHCLKWIYLRFYAVSSEIMSYFGFPLIGIILWTFLSFF
DSFYLSGDTLGKGNFDYMRLISNISWSFYCFSLTAILTVSSHLSLNENHKMMFNIQVLLLQS
DFSIGVKQELKDISIQFTGLNIQYSVCGFFNVDLPFLZTFVGVIVSYVIIMLQFG
>BgerGr337
MEYTDANFISAVKPLIYILQMFGLVNFSLNGGNAIYPLPRLFWTLFCTSLVSVDMAVFFVFI
FPEYYYSFSTKAFLAFSLYMTFTYFTYITCFLFLGVLKRRKVVKIIKEFSQIDSLLLSETAVIC
MHRHMKNFYLAFLTILVCCEIGLYAWHYTCHCTYFDWQCVRYTILIFYGFLTSLVIIGFLTL
VIALKQRLQAINRILFSQYSQDLKSTEKLISVNTRSIVSGELASFSVRDIRKLRKVHDKLHSIS
LDLTSSYGLVFLAVFCWNFFSCIIVFYMLIFHSRTQNAGKSMLNALFECVFSSSFTAMLAIF
CHLTLDEEKTTSIHVQKLLLYSNLSTDTQEELRKLKSQLKCLKMNFSACGFFSVNLPFLAT
LLGLICTYVVILLQFK
>BgerGr338
MVPLEESLISAMKPLIHLLQIFGIVSLSFEGNKIIFPTRRLLWTISLCSLVIIDPLLILTPFFLSE
SYIPIIASLKVLIAFYLHVGLTYLCYITIFLSLGIFKRQRFANIFQELSEIDTLLSSQTSTTQLHR
QLKRSNLALLLFLVGIQVVLFLWHMLSNCLPFESYCFQFLIQLFYGFICSLMVISFLSLVIAL
RQRLKVLNRILSTQYCRSDITSNEEAVSFGPRKARAYSLSVMDVHNLRKVHEKIYTVSLHIS

SCYGLIFLITMLWSVINCIIICYSAITLSQENKPSHAIFAFCWSIFALSSILNTTLLCHVTVNEE
RRITFNVQRLFLYSNLGSKVKEELKNLSLQLLCLKMEFSACGIFCINLPLLATVLGLICSYIIII
MFQFT
>BgerGr339
MAPLDENFISATKPLICLLQIAGLVNFSIDGNNITFPLWRLLWTLFWTSLVAVDPIVYLTFV
ISEFINSYSLKILISIHLYLIFMYFAYINSFIYFGILKRRLLGNIFQEFLTIDSLLPKSVVLSSLHR
QLKKSYFSLVVYIVGFHIVLFVWHSISNCSLLNWRCVHFSLQSFYGLSCTFVIINFISLVLALR
QRFKAINRILSDYYHQNSNKKNISITVNLMQNITCTTRKYSLSVQDVRNLRKIHEKLHLISR
EITSCHELTLFTVLCWSFFYVIIVSYMVSFYIKDEIIAFRAVYPLFWSIYVLSVTVIITICCHFT
VDERKMTMFHVQRVLLYCDIYGELEEEFEKLRAQLKYLKMEFSICGFFSLNLPFLGTMLGL
ICSYFVVMLQLT
>BgerGr340
MTEENFISAIEPLINILQIFGIFNFSFERNVIIFPFRRLIWTFLCASLVSVDLFMNFTLSISDYY
YYVSNLKVFLAINCYLVFTYLSYTTSFIFVSILKRKLFTNIIQEFLQVDDLLLPRSAATHLHR
QLKNAYVLLFLYIVGVHAVLFTWRLASICTPFQWKCLHFTLQSFYGLVCSLMIISFLVFVLA
LLQRLKATNQILSSQYCRQDLSSCQKLVFIHRIVTNRNYLSVRDVNNLRKVHEKLHTITYD
LISCYGVTLLVIFCWIFFYCILAFYMTVVYTQAHYSGHRSLQPLCWCIFALSFIAVMTTCCHL
TVDESKKTKFHVQRLLLYSKLCVETEKELQNLTSQLKTLKLEFSICGLFTLNLPILGTMLGL
LCSYIVVILQFS
>BgerGr341
MEYTDANFISAVKPLIYILQMFGLVNFSLNGGNAIYPLPRLFWTLFCTSLVSVDMAVFFVFI
FPEYYYSFSTKAFLAFSLYMTFTYFTYITCFLFLGVLKRRKVVKIIKEFSQIDSLLLSETAVIC
MHRHMKNFYLAFLTILVCCEIGLYAWHYTCHCTYFDWQCVRYTILIFYGFLTSLVIIGFLTL
VIALKQRLQAINRILFSQYSQDLKSTEKLISVNTRSIVSGELASFSVRDIRKLRKVHDKLHSIS
LDLTSSYGLVFLAVFCWNFFSCIIVFYMLIFHSRTQNAGKSMLNALFECVFSSSFTAMLAIF
CHLTLDEEKTTSIHVQKLLLYSNLSTDTQEELRKLKSQLKCLKMNFSACGFFSVNLPFLAT
LLGLICTYVVILLQFK
>BgerGr342
MVPLEESLISAMKPLIHLLQIFGIVSLSFEGNKIIFPTRRLLWTISLCSLVIIDPLLILTPFFLSE
SYIPIIASLKVLIAFYLHVGLTYLCYITIFLSLGIFKRQRFANIFQELSEIDTLLSSQTSTTQLHR
QLKRSNLALLLFLVGIQVVLFLWHMLSNCLPFESYCFQFLIQLFYGFICSLMVISFLSLVIAL
RQRLKVLNRILSTQYCRSDITSNEEAVSFGPRKARAYSLSVMDVHNLRKVHEKIYTVSLHIS
SCYGLIFLITMLWSVINCIIICYSAITLSQENKPSHAIFAFCWSIFALSSILNTTLLCHVTVNEE
RRITFNVQRLFLYSNLGSKVKEELKNLSLQLLCLKMEFSACGIFCINLPLLATVLGLICSYIIII
MFQFT
>BgerGr343
MAPLDENFISATKPLICLLQIAGLVNFSIDGNNITFPLWRLLWTLFWTSLVAVDPIVYLTFV
ISEFINSYSLKILISIHLYLIFMYFAYINSFIYFGILKRRLLGNIFQEFLTIDSLLPKSVVLSSLHR
QLKKSYFSLVVYIVGFHIVLFVWHSISNCSLLNWRCVHFSLQSFYGLSCTFVIINFISLVLALR
QRFKAINRILSDYYHQNSNKKNISITVNLMQNITCTTRKYSLSVQDVRNLRKIHEKLHLISR
EITSCHELTLFTVLCWSFFYVIIVSYMVSFYIKDEIIAFRAVYPLFWSIYVLSVTVIITICCHFT
VDERKMTMFHVQRVLLYCDIYGELEEEFEKLRAQLKYLKMEFSICGFFSLNLPFLGTMLGL
ICSYFVVMLQLT
>BgerGr344
MTEENFISAIEPLINILQIFGIFNFSFERNVIIFPFRRLIWTFLCASLVSVDLFMNFTLSISDYY
YYVSNLKVFLAINCYLVFTYLSYTTSFIFVSILKRKLFTNIIQEFLQVDDLLLPRSAATHLHR

QLKNAYVLLFLYIVGVHAVLFTWRLASICTPFQWKCLHFTLQSFYGLVCSLMIISFLVFVLA
LLQRLKATNQILSSQYCRQDLSSCQKLVFIHRIVTNRNYLSVRDVNNLRKVHEKLHTITYD
LISCYGVTLLVIFCWIFFYCILAFYMTVVYTQAHYSGHRSLQPLCWCIFALSFIAVMTTCCHL
TVDESKKTKFHVQRLLLYSKLCVETEKELQNLTSQLKTLKLEFSICGLFTLNLPILGTMLGL
LCSYIVVILQFS
>BgerGr345
MAYLDNSFVSTLKPIIRLSQIFGLINFSFETNYIVFPLPRLLWTVFCACLVSVDPFVFFTFVY
SDYYKLYNIKIFIIINLYLISTYFTYLTNLFFVGILKRRQVVKIIQEFSKIDNLLLTRKATSLHR
QLKNYYFLLLTFSAGFQLIHFILFIKINCPVFNWNCLHQTIQTFYGLVGTLVAINFLTLTLAL
RQRLKALNQILSLQHSEDIISPNEKSDFIISSTSHKISNVTKYTLSIQNIRKLRKMYEKSQTIS
CDLTACFGPSLLTVICWSFFFCITVFYMIISNVHIGTNPLGYTGFYVITFSWCVYALCFIAVL
TVCCHLTEDEGKNTMSHLQRILLYCNVYGEMERELERFISQVKCFKIEFSICGFFLVNFPFL
GAMLGLMCSYVIVILQFN
>BgerGr346
MIHCEESFISSLKPLISILQIFGFINFSFEGENIIFPVRRFLWTLFWLSLIFLDPILFFNFVIYDY
YQMHILNAFLVANLYIICTYLTYTSNLIFVGIFKRRRLVKIIQNFIEIDSMILSPETASDLYRQ
FKSLSLLFIICVLGFQTFLSVWHTLLYCDSLYWRCLHCIILSVYGLSCSLLIVNFISLVFALRQ
RLKILNQILFKQYGHQYPNTDEKLFSFNTTVTQRSSLSVRDVRNLRKAHEKLHAISHDLSS
CYGLSLLISFLWSCFYCVLAAYIAIFSTNPNAHDRNGYRALQPIFGCFFALSFIAFLTINCHFT
VDEGKRTIFHTQRLLLYSNYCYKVEEELKNLSFQVMFLKMEFSVCGFFSLNLHFLGTMLGL
ISSYIVLMIQFK
>BgerGr347
MTHPEENFVSALKPLINVLQIFGLVNFSFEGNIVSFPHHRLYWTLFCTSLVSVDPVIFFTLLI
SDYYHLYSLNGFLTVNLFISFTYFTYTTGFLYVGIFKRRQLAKIIQDFSKIDNLLLSRTEVAV
LNRKLKYSYFLISIYIVGFLACLSIWQTILVCIEFSFCKCIHYAIHFLYSLVCSLMIIYFMSLVFG
LRQRIKALNLMLQTQYRHQEFCPSEKIIPVNSKAKVNFLLSIRDVRNLRKVHEKLHAVSRD
LTSSYGLAVLTALCWSFFYCILVFYMAIFYSNTQNFGCSALNHLFWCIFCLSFIVILIVCCHL
TADEGKMTIVHVQRLSLYSDLGTELEEELMKLTSQLKCLKIEFSACGFFTLDLPLLATMVG
LICSYIVVMIQFS
>BgerGr348
MDYQMPHCKENFISAMKPLISVLQIFGLINFLFEGNNIIFPFHLLLRTIFYASLVFIDPVVFY
TLTSANSDRYNAAHTTKVLIAVNSHIMFTWFTYIINLIYVGIFKRRKLAKIFQEFSQIDSFLF
QVTTSSTHKQLKNSYLLLLIYIIGCQIVLFIWYIFSDCIPFGWECFHYSCQSFYGIISSLMIISFI
ALVLALRQRLKTLNEILSLQCCHQDLSFSGKIVSIKHRVTKPNFISVQNVHNLRKVYEKLHA
ISYDLTSCYGLALLIAMCWSFFYGILVFYLAIYYIQTENAANPICWSIYSMGFTAILTVSCHV
TVDEGKITMFHVQRLILYSNLCSEAKRELKELKSQLKCLKIEFSACGFFSLNLPFLASMLGL
LSSYIVIMLQFS
>BgerGr349
MTWPTGSFISSMTPLFYVSQFFGLTNFSFDTNNHVISFPVYKRIWTCIFIIVTTVSMGMYIM
SLILDFEYSLPLKLHIAGNIFILCKYFAIFSSLIIPGICRKQEVVKIIMDFLEIDDLLASHRVLVD
SKTKLFFLLLFYIFFSKSIEDALFIFRRCPSPTFSCIHFILYNFTSYIIILIVITFVSIALATRQRL
VSINKVLTDICSNLEGHRKRSPQFLEFAPLHNQISFSIDHEVRSLRQVYGILFATSRALSSIYG
IPILGAISWIFSCCVYSFYVIAYILERGDEHYVLIIDLALWAGFCISLIVLLTTVCHITVDENST
TMFHVRSLLLQSSLTIEEKEELNAMSFELSYLKVEFPICGMFNVNLTFLCNFIGIIFSYIIIMC
QFQ
>BgerGr350

MIYNSALNRLPYSDFLNFTISSNMNHARSNFISSLKPLIYASQFFGITNFTFYRGDIVFHVSK
LFWTIFLLIIVAVFLGFYLVFGITFNYSKPRPLKLYISWYSTLFSKYFAVFSSLIYLGICRRRLL
AATFKEMLVIGDLLVFPTGYVADSKTPKKYALLLTYFFIFKLFDDVLDIMVNCKASSLECIY
CIFFTFSSYIAFLMTINYICIVLVMRDRFIIINQFLAEFNFQLEERSTKPIFISELIPTSKRKSGH
FSAVHRLRLAFDKMHTVSRYLSSIFGIPLLGATMWIFCTCVHAFYTLIFTNYEQLISFTKTL
DFVIWIPFCASIIFLITLSCHMTMDERNKIVFHIHKLLLQSNLSIQEQKELNSMLLQVTFLK
MEFSSCGMFTFNLSFFTNFIGIICSYIIIMTQIQ
>BgerGr351
MEETSLRMNRSTSSFISSLKPLFYISQIFGVYNFSIDKNNLYFPVYKLLWTVFFTLLSAIHMV
LKAVRTNLFFDHSLPLKLYIAYNILIACKSTAILTSLIFIGIYEKQKLATMVKDLIIIDDILSTY
VRYTLVSKMKRLCWGVLLYVVIFKLFTDSFYVLLESESLTLSTLPPLLYSCNSFVVFLMICN
YVYIVLYIREVFVVINDILAKRFLSVEECDMNLTTRNGCKNARYPSEFPRQLRSLRFVYGKL
FAVSRTHSQIYGVPLLFTAFWFFSSCVQTFHVEAHLFQNKNTNVISLLSTISWIVFIISLMFL
ISLSCYKTEYECYMTKFHVHSLLLTNLTNEEKKELLLFRSHLKSLKIKFSICGMFTMNITFFS
NFIGIIFSYVIIMCQIH
>BgerGr352P
MNRLTSNFISSLKPLLYVSQSLGVTNFSIDKNNVYFPVYKLIWTVIXIILTVINQGSRAYYYFK
SNFGQSVPFKLHVVFYILMLAKFTAIFSSLLFLGIYKRKQVAAIIKDLLVIDHTLASNKGYHG
DTKTKKLSWVLILYVLLYKIISDVFFIVHHCNLDTCAWHCIYTALYSSNSDVVFLMIINYLF
TVLAMRDRLIIINEILAAHCQNLDEYHKENFGLIKNHPATRQRNESCINFTCQLHSFRFVYG
KLFAISRELSHIYGIPVLATALWYFATSVESFHYEAYFLQIKDANFSYLSMRLLYTISWIICCI
TVMLLMTVSCHMTEDECCKTMVLVQNLLMEVNLFNEEKEQLMLLSSHMKSLQIRFSLCG
MFSMNLSFFSNFIGILFSYIIIMSQIH
>BgerGr353
MDPAKSYLVSSYKTLFHISQICGVINFSFGRNDINFPFKKLIWTVFVLVFSSGNVVLYFCTFF
TEPDFFLRPVKLTIASTILVISKCVAIFATLIFLGIYKRQVLASIFKNLLKVDDILALCSSCAFV
GKMKKTYVILLYYVLLFKIVGDVYYLRSHCTSFSWACVRNVTYIANSDVIIVLILNYVYIVLA
LRERFIVMNDVLLEQRSKGLHKDISELLPVSIRRRTGKWTTTFVNQLHSFRLVYGKLFAIC
KQLTSIFGVPLLFTTLWVFSTSVEAFHYEAYAFQMGNIEFIPISNTIVWMLYCISLILLITSSC
HHTMSECETTMFHIQTLLMDSGLSNDEMHELHLMSSHLNSLKLNFSLCGMFSLNLSFFCD
FIGLLCSYIIIMSQIQ
>BgerGr354P
MYQPSSNFISSFKPLYRISQFFGIINFSFYGNSVYFPAYKLFWSVLFVVFSLGVQGLHTVYLI
KHFDYTRPLKFLVIFNILVVSKTFAIITSLVLLGIFKRQQIVTIIXNLLVIDDILAPYVTNVIGD
KMKYVCRALLLHVFLFKAFDDVLYILMECRNPARSCLRPIVYTCNSYVVFLMIINYVHIVLV
IRERLIVINKILSGYCQNMEDSQSKNALRSSKFFQSVHRNTIRMTPFIRQLHSLRFIYIKIFLI
SRKLSNICGVPLLATTLWFFSTCVKTFHYEAYSLQSGKIDYISMSNTILWMSYCVSMMLIPA
VACHITTSECYTTMFHVQTLLMQTNVTKEETDELIMMCSCLNSLKLEFSVCGMFNLNVSF
FANFIGIVCTYVILMSQIQ
>BgerGr355P
MPNLEANFLLSMTPMLTVSQFFGLINFSFDGEKINFPLHRYFCTLVSTLIITLDPILYGATGF
FYFYNSIPFKLFIMQNFDLIARLLTALAGLIISGIYHRRKLVTIIQHFLEIDILLIHKLDRLKAH
SNQKTFCILLLVYSLVIXFGWFLSTNVCSTFNWGCFRTFSITLTELVNALSTVIFVSVIIALQI
RLKVINLTLLQYYRQYESCSSDTIVIIDFNSKVSQRIEETISISCEAVRNLRKVHGRLYEISEN
LTSCYGLPILGVTLRIFTYSIVAMYFSLCISQTDLIGVNCTSKIQLTAKIIFTVSLMIIMTLTCQ

LALNENKAIIFNIQRLLLETRCPRETEEELKRMCRQMTTLKLEFSICGFFTLNMQFLGAFV
GLITSYILIMVQFSA
>BgerGr356
MGTTSTFISSTKPIFCLSQIVGLTSFSIDKNSNTITFPKFRLLWTIFSITIGWGSLLFSFIYSPT
DFDYSVSLKLNISYCVLSLFKWIAFLASLIFLGIYKRKDVVIIIKEFELVDKLLYSQRDNTAES
KLKKLCLFLLAYLVSIELFDNLVDVTSLCKTFSLKCAYSITNEYNSYVILVVIISYVSIVLSLRE
RCKATNKILFDFCSSFETHLYNDVIVPKPCTSLLITWCFPSVRELCYLRLVYRKLFVISRLLE
SCYGFSMFTVICWVFLSCVHYCYLAAYSLQSGFKDYVMMIDMALWAPFCVSLIFILTASCQ
MTMGEYRLTTGHIQTLLLHSSLPKDVKKELKAMNSQLIAMNLEFTLCGTFRLNLSFLCNFI
GIICSHIIILSQFQ
>BgerGr357P
MSRISTNILSSMAPIIYISQIFGLINFSVAIRKRSVIKYPIITIFWAILLAIIGCGSIVSSLFYSTSH
LDYSISLKLNISYKMLNVFXYLTYLSSLIYSSVYKRKNILNMIKEFQVIDKSLFSEIGCTVRKT
EXQLSLFLIIYLVFIKCVDDLLFLTSMCKSVSIMCVHYISYSYSSYIILIMIVTYVTFVLSLRERY
KTTNRILEDLYSNFRTRHNKNVIISEACFLLVLPWNPPSVNELRSLRVVYKRLFTLRERYK
TTNRILEDLYSNFRTRHNKNVIISEACFLLVLPWNPPSVNELRSLRVVYKRLFTFLTLSCHA
TVEEYRMSIVXLLLEPSLSTDMKEELNAMRSQLTDMKIEFTVCGMFRLNLTLLCNLIGITC
SYIIIIMCQVN
>BgerGr358
MKGFMETNDFVNAIQPLFLICKVFGLVSFSVTRNKSKFDFFWAVLWIICFVIALIGHIWNK
LTDCVTIIPMKIKAVDIFYEISVYGTSIVSLFFCNILKTKQTSQIIRKIQFIDEMFIGKSEASKL
NKRLKLFSVAQLSITLLANIILSSIYMYLNCQSTKVQCLVAATESVSSVTNSLVLVIFITFVAT
LRERYLYINRYLYNFCCKLQSDDKYRHNSSRTTPEVRKVISKISEAVFKNLDTYALSPSEIRY
FRLLYYKLHTLSRSINTCYGFPILCATFWTFISIIDIFYNGVFSLNASYSKSSFTHYGNLVVCF
GWSIFSAVLIFVTTTVCHMTVEETNTTMVHVESLLLCSNLTVNAMDELKNFSFQLSRLKH
EFTACGMFTLNLPFLYALIGVTCTYFVILCQLN
>BgerGr359
MATSSDIISAMKPVTGISQVFGLTYMFPQKKSHFSTFRIFWTLCFVAMFLATLIVIGIFELPE
IYRVFTLKFFISGSLYIVSTYLSCLVSLVYPGLYRRKKFAELRQKWLDMDELLSSHIENSRTY
KRLKTLCVLLVLYFLVANIILSTMHIVSEFRLSTFSGMVGILECLACLVNCLLVANFVSVVLA
IWQTLTIINKHLSHYFLKLGFQDETYIFIPSLKTTGCPDISLRERASQSNFLSGFEIRLLRLMY
CNLYNISRVLLFCYGFPILLVITWIFMSTVSAFHYAMYFLQVGVPSGHIKTSITWSVFCLSLL
FILTLSCHLAVSETNLIVINIQSLVLVSNLSTEAKEELYAMNSLLCCLKMKFIVCGAFCLDTP
FFCSLLATIVSYVILMCQLR
>BgerGr360
MVESSADITSAMKPIIFVSKVFGFNNIYLQRKTDSISIFGTIWTVFVALLFKVGFILWITLTLP
VFYRYVQLQFFISRVLYIGCGYISCMLGLIYPGLYRRKYFVKLRQTWFSNEQLLLPRMENRI
HLKTLKKLYLLLLLYILIANLTLGAVHVFIEFQNTIYKGMRCILECLVSLVNSLMIVNFISIVL
AIRQRLKSINKCLSVYSLRTEEKQETFYFISLNLGGNDLFLWQQEKDNMINNSNIIGGHGRF
LRQRIQPFKVLSGFEVRLLRMLYNNIYKITRILVFCYGLPLLLAMFWTFLTTVSTFHFIVFVI
KQNINQTNIIININISWCFFFLSLAFILTLACHLTEEESNLIITHIQTILLDSNLSIETKEQLNAM
NSQVSSLKLNFPLCGTLFLNIPFFCSLLTAVLTYVVLMSQIQ
>BgerGr361
MARTNMNIILAMKPVIFVSQVFGLTNFSFDGKDFSFPFFKLAWSVVCTSLIIFGVILFYTHRI
FNYCYTIHVKIYISSILFVSSNYLTALLSLMFLGFIKRRQLAIAIEKYLRIESEMWSKVSDNNR
LHIILFLTFIILANFFEDVLHIISEWGCSPWNSVHVIIETISAFINALCFVNFVVLVLAVRDRL

KMINNRLSASRRELEDSLRTNNITFVLKTTTGNIIPRSYISDTEVRSLRLLYGRLYEVSRMIT
SCFGIPLLAAISWVFLSSVYAFYFAVYSIQTERIEVIQMINFLTWPLLCLISLLIFTVSCQMTT
DEGKLIIFNVQSLLLDSSLSSAVLDELKEMNSQVCSLKLEFAVCGVFTLDLQFVWAFLGVIT
SYIVIICQFS
>BgerGr362
MTRPSVIFISSMTPLIHFLQICGLIHFSFQKDFVSYSSLRLLRTIICITVIVSDLVLYHIFVNEQ
HYQDYLPFKLYVTYNLYLLIIHVTSIACFVCPCIVNRRRLTKIFKEFNKIVNILIFQTGNIDLN
KKLKKLCTVLLLSFLSMTFVTWTWYLSTKCVTLSWLCFHATVVTFSGFINGVIIVVFVTMV
LTIRVMSNEINTILSNYSNFSNNNISLWTSDHSARRWVEESNRVTKEKVLCLRVVYVKLYA
ISRELNSCYGFPLLGVTAWIFLSAILLFYFVIYFTETQVMNGITLLRITVWGFYTVSLMVIVA
ICCHMTVKENDKTMFHVQKLLLQTDVSSEVKKELKTLSTHLTNLKIEFTSYGFFTFNLQF
MCAFFGVISSYIMLMCQLN
>BgerGr363
MTWTYNSFISAMKPIFMVSKAFGLMNISLTRENFSFNGPTLFWPILWIFIVALNPVIFCVFI
VTNYYDSIPLNLFIAYNLYLVSLYFTSFLSLICSILRRRQLANIIEEFTQICNILVCRSEGAEIFR
KMRHVSLILLLYILVIGSGLIFYIVPNCRSISWKCLYRIINSCSGFINSMNIINFTSIVLAIRECL
NIINKILCNFGQQLQIYNVSMRPIVRPSIIIFGGKITGYEYLFEQEMRCMRRMQGTLFALSQV
VMSVYGLSVLGSISWIFLCCIVTFYKIIFLSQAKVIEHVALGGSVIWCLMTVSLIVFATICCHA
AVDESDAMLIHVHYLLLETNLSKAAKDELKGIYLHLNSMKIEFSAYGCFVLNRQFLVSFFG
LICSYILIMLQLN
>BgerGr364
MTVSKNEIISALKPMFFISEIFGLVHHSSFFEDKTILSFIQLSRTIIYTTIVAIYCVLSFILVLPN
YTNNVPPKLFIAHSFYTIITAAAMIISIIFSILKRRVLFQIFQDLRDIHRVLFFKRAMHQDYS
MFKNFNLVLLLYLIVFFVIDDVWFITMQYQSFSFATATFAFGTVCEMTGNLVVLNFINVVF
IIQQGFRSLNQILHEYWKHDQDYNKNVIFALEFKSVSKSGMKHTTSVTARDVRQLRIIQRK
LFEVSKMLLSCYEVPLVATILRIFLASVDGIHYCIYYYQTENNFMEPLIWWIAILSLIFVLTFC
CHKAQNESCATMLYAQNLLLQPNLSSDVQEELLGMTFFMNNLKLRFSTCGLFTLNLPFLG
TFIGLISSYILVMLQFN
>BgerGr365
MSDVTDHKHASLSPKWPSFTRSTNMNSALKPIFFISQLFGIMNHTSLGESNVCVSFFRLC
WTLMYITLTSLYPVIYFLTFSREYCYKVPIKLFIIHSVYWTALSITATACLVSSVYKRKLIVKI
FKELRDIHDSLALRTGTYLDYTKIKNFCLILVFYLLVVFGTSDTWFIEMYCRTFSLETLHRT
FEMTSELIVALTVVNFIIIVFIIEVCYKCMNNFLSELNVQLDKNNKDPIMLDYKKERLNTPN
IKQYGKLTAKDIRQMRNIQRRLFSLSRTLLSCYGPLILGRTSLNFLSSIDGVHYYIYYFESGG
KFLETTLWWGSNFSVIVVLTICCHLAQNESSATMFYIQNILLHRNLSREIKDELKEMSLFM
NNLKGQFSICGCFTPNLSFLGRYIGLISSYIVILVQLN
>BgerGr366
MIQHTTDIVAALKPLLYISEFFGLWSHASFIKGITIHPFLRVFWTIVLMTITGIYLTVYCSLII
PDYYHKLPLNLFIVHSCYWTILSVSALASLIFNIIKRQELSAMYQELRDIYKVLVFDRGLFGS
YSKVKKICLLIVAYLIVVYGMFGVLFIVARCKFNSIRSLTCIFGAISDIIANLVVLNFSVVVLIIR
EAIKSLDNILSQVCQNGREFKTIFASDFEALYITRITKHGSSAGKELRLLRVVHGRIFSVSRV
LLSCYGILLVLAIIRIFLSSVDGIHFCIYFSHSAFDLLESVAYWFGNFSLVVILTFCCHLAENES
YETMFNIQNLLLQTNLSVEIREELKEMSSFISSVKFQFSTCGFFTLNLSFLSTFIGLICSYILIM
LQFN
>BgerGr367

MQKQSNEIVTALKPMFYISELFGLLTHVSFGQSAIASSLRLSWTFLNVLLIAMHPVIYFAFL
ATDYNNRNPFKLFVVHSMYWAVLTLTAATSLIFSILKKQILAKIFHELRNIYKTLSLLEGAC
EDFGKIKNFCLIVVVYFVFVFGINNTWFLVASPKISFTKSYIRILGITSEFIVILVILNFILVVFT
IREAFKLINKVLFEACAEITLVLDFKTTHKPKTKHVGILKAKYIRELRIVQSKLFGISKALLSC
YAFPLLGATLHIFIASISGIHYSIYYLNFKYLLVLQSVLWWLGSFSLMVLLCIYCHLTENECN
ETIFYVQNLLLQPNLASDVEEELKKMSSFMNSVKLKFSSCGFFTFNLSFLGRYLGLISSYILV
ITQFN
>BgerGr368
MALFRNEIVTALKPIFCISEIFGHANHCSFFDGRNTRITTFLQLSKTIIYITLVAIHSVLFYVFF
LPDYYHKVPLKLFISHSIYTALTAVVMIVTLIISVLKRQVLFFILQNLREIHRVLFFRRGMHE
DYSKFRTFTLVLLLYFIVFFGIDDTWFLVMEFTTFSPSTMTFAIGTICEITVTLAVLNFINILF
VIQEGFRSLNQILHESLEHGQDYNKNVMFSIDFKSVSKSDKKRTNSLTARDVRQLRIIQRRL
FEASRTLLSCYGVPLVGAILRTFLASVYGIHFCIYYYQAQGNFMEPLLWWLGNLSLIVVLTL
CCHMAQNESSATMFYVQNVLLQPNLSGDVQEELLGIFSFMTNLKLRFSACGIFRLDLPFLG
TFIGLISSYILVMLQFN
>BgerGr369
MSSSTAFIFSLKPLFYISQFFGLSDFSSNKKMLFPHLKLFCAVLCISMVLISYVLYFTFIAMKD
YNTIPARLFYAYCIFLTAYTSTAVVSILYSNIFKTNQLKSIFEDLVNYNELLVSTFKVRKIGSD
VRNLNIVLLVYVFLMKLCNDVFNGGFSCRKLSFRCMFGVMQGFIGFVYLLTIIHFISLVLFV
LHILKLINKVIEDIFQDSSFNFFIPVSKIRCLRLVDGKLHLISQRISSCYGLPLFLITVCIFFITIV
TFYFALFSMRKFSFDYVDVLPTALWGIYHASLIFVLTLPCQMTINESLAIMYRIESLLLQTK
LPNEIEDELRKMSSQIRKLKIKFSVCGFFTLDLPFMCGFFGVILSYIVVVSQELS
>BgerGr370
MNRGRMVVTNLNSWVKHLFRISQFFGLISFSFENDKPYPKLGIRGTIINIVISLLEMILYTLH
NILYFEISKPIILYISFNLFYICQQLAIITSLVFLGIYKREQLSNAIKEILIINRNSLSEIKQLKSSD
KTYKIILFITTYVIFAKVFSHILYIVRKCDSFSLRCSQTIIASYIQSTIILMVFIYIAIVLSMRERFI
VINSRLFKYCSNSENQRKKISVLILTERDLKQNSPTVFEVRFMRKVYVRLYIVLRALNSCFG
VPLLCTTSYICLACIDVFYKTTHTFRIPTDDYILVLNFVLWISLFVSLMYALTLSCHLTMDE
VQGTLVNIQNLLLKSNLSRDAKEELRAMNSQLTNLKMHFTVCDIYMLNLPFLCSFLGIISSY
VIVMAQFQQKS
>BgerGr371
MTETNFITSIELMSKVSQVFGIFSLTFANENTSVHILKLLWTLLNALIITLYPVIYFTFVLPH
LYTTYHLKVIVVFTIYILSSYFTSMVNLFYFEIEKRQQLKIFLKNIQQIDDLLLSKTHNNVHS
NLRKRYLYILQYISIMSGSGQVLYVFTRCTNSPSCFCILTFCDCYILFINCVMILNLAILIYAIS
ERLKYINACLSNYCSEKENLPEKSFFAPNCAPKFRELVIRTAITERDVRKLRLVYGKLYSVS
QNLQTCCGFPALCAIAWIFISLVEAFYFVERTLENHDDFIVIVTAGLWLSVTTSLIVILTSDC
QKTIDERSTMMFHVQNLLLQPHLARETRDELKEFCNQLKCLRMDFKACGLFSLNLPLLCT
VFGVVVSYIILMCQIK
>BgerGr372P
MTETNFITSIMLMSKISQVFGIFSLTYVDENIPVHILKRLWTRLNALIFTLYPSLFFTFVFPL
LYPSFHLKDTVVFTLYVLSSYLTCMVYLFYFEVKKRHQLKIILKQMQQVDNLLLPKTHNSV
HSNLQNRYLYLLQYISILSGSGQVFYVFKRCSSSPFCFCILSIFDCYINFINSVMIINLXTSFYVI
SERLKLITYSLSNYCFEQETLPEKSFFAPNCAPNFRDMITRTAITERDVRKLRLVYGKLYSV
SQNLQECCGFPALCAISWIFVSLVEAFYFVERTLENHDDFIIIVTAGLWLSVVISLIVILTSYC
QKTIDESSTAMFHVQNLLLQPYLPRETRDELKEFCNQLKCLRMDFRTCGLFSLNLPLLCTV
FGVMLSYIILMCQIK

>BgerGr373
MVNNFVSTVKLISTFSQYFGLINFAWNDEKNTLQILRVLWTFLLILFSAIHPIVYFTCILPD
MYRTSPLKLTLAFTLYSSVDYFYCMVNLIFFGFTKCRKLKNIVKQIDQFDDLFLSKENSVV
HTNLQKICLYLLGYILILLFSGLAMYIYTSCDRFSWFCIFTILDSYITIVNTLMIVNFVVMVFA
IRERLKCINRSLFVFSHERQICPSKAFFVSNVLNSRELSSLTSISEKDVRNLRLMYSRLYSISQ
KLEDCYGFPTLCSIAWIFISIVESFYYIDGIIQEHTTGFLSIIVCCFWTTMDVSLIILMALSGHG
TIQECCRAMFYTQNSLLQPYLRSDTREELKEFGHQLSCLRIEFTTSGLFTLNLQLLCTVFGV
VISYIILICQIQ
>BgerGr374
MSYPENIVSILKPVLYISQVFGLTNFSIHGNSIYFPFYKVFWTVVCSLSALTSFILYLIFFDMY
PDSTPLSLVISANSFICCGQIGSLTSLIISGIYKRQKIALFIKELQLTDIYLIKFPSFTETKSIKKY
CVLFSLYVIVMQAINDINYLIGICGDISFDCVRAILLCSVECVIPFMIVIYVCMVLAIRARFKII
NTWLTVFSFKDNSHRTNSVDSKFPTLMTRPRITPLIDTSKANATDLKQLYVRLYGASQAL
TSCFGVPLLVSIAWIFLSCLSTFYYVAYIIQESEGYIPIFVNVFWSFYTTSLMVVLTTACHLTV
DEAQTIKLNVQILIHQLDFPRSLENEMNTLCSYVNNLKIEFSTSGFFTLDLQFMFSFIGTIIT
YIIVMCQFD
>BgerGr375
MASAPTNIISLLKPLIYIYQAFGLITLSFNNNTVFYSKIRILWSILWLLLIIPGIVVTYILDISDN
YIMFNQQCVKILSQVIHYITSILALLFIGIYKRQQLSNILQEFVKIDESIFKLTGNTEKYKQFK
TSLFLTVGIISIYSFLIVSFIYTNCKEVTWKCSYVFVYVSILINSLMVSIFVTMVIFIQRRYKIIN
SFLVERRHGNQGLFEKIDIDLDNKMISPMLVQNRSISVQDVRILRALHVRLYILSLDIVSLFG
FALSTYIVGEFFLCLRYLHLMLYSTKEFNAFMLRFTTTFVRILQIAVLNICCYLVVEERNISS
TYIQSILLDRNLSGHVIEELNGLAFCLKSLRLEFKIGGFFKLDMPFLSAFLGLICSYTAIMFQV
G
>BgerGr376P
MASQEINVISSLKSVFYIYQVFGLTTLSFSKSGLNFSYIGLLWSLLWFFFNFVGTIEFCLTIDD
YISVYPLNLKILTILYDFSCYLSYIVTLFFIVVYKGHEIRKVIQQFIQIDHILQKQNAHLYKVSK
LSLFLTIGIILAGICMLYTLLVINQYNCLAWICVHVVTSFCLNFTLNSLMVSQFVAIVMIVRE
RFRIININIILWNCYKQYQPYFEMSVPYSHERVVPSNEQCRHHTLTVQDICILRYLHMKLLINS
KKLTSIFGPSFFLSMTATFLCCVRYIQIIIFYSQKSEVSIPFLPLLHITVMLVTNTFLVIALNVC
CHMTLNERSEAMIHVQNLILHPHVSKYVXELNSFAFCLTNLKMEFNVCGFFKLGLPSLGSF
FTFICSYIVIISQLS
>BgerGr377P
MASQETNVISSLKSLFYIYQVFGLTIFSFSTSGLNFSYIGLLWSLLWFFLNFVGMIAFCLIIDD
YISVYPLSLKILTILFNCSCYLSYIVSLFFIVVYKGREIRKVIQQFIQIDHILQKQNAHLYKVSK
LSLFLTIGIILAGICMLCISLINQYNCVSWICVHIITSYCFNFTLNSLMVSQFVAIVMIVRERFK
IINIILWNCYRQYQPYFEMSVPYSHERVLPSNEQCRHHTLTVQDICILRYLHMKLSXINSQK
LTSIFGPSLFLSMTATFLYCVRYIQIIIFYSQKNEVSISFLPLLHITVALVTNIFLVIALNVCCN
MTLNERSEAMIHVQNLILHPHVSKYVKEELNSFAFCLTSLKMEFNVCGFFKLGLPLLGSFF
TFICSYIVVISQLN
>BgerGr378
MSMLAGKLVSALKPIIYASQILGLTNFTIDRKIYFPIRRLLWTILWLVITLLGFVLCLIHLQSY
DVHSFKRNASFNIHFFSSYLTTIMSLVFLGIYKRQKIVICLEELSKIDDLLILRKNCPSLDKNT
KTVTLGFLIYIITIPAFSNTMQLFKMCSPWTLFCVQGILYFYHTYITCLIIVMYTILVMTIGSR
LKIMNKLLSIFCDKLEKELNKSTFKTEFRQWITPKENQVKRTSYISELVPATLQRLILKIND
HEFRDFRKMYSKLFAISQALISCYGFPLLAVTLSLFFSCIDGFYYAAYYFKYGSEGYIVKTSY

VLWTVYSSSFMLIITVASHDTVNESKETLYLVQKILMHSNLTHTVREELNELLSQSKSLKIR
FSIYGIFSMDLPFICNFFGVIASYIILMCQLI
>BgerGr379
MITPQESFIPAIRPVLFLSQFCGIMNLKLERGRINFTVPRKIWTCLCTLYLIIGVIAYLAYYM
DTYYDLLPLKLYIVVNIYTATTYFGALSSLLFLSMYKIPTLKNILQEFINLDNITISQTGFVEK
ARMLKTWNLLLFGYIVVPKLCLTVWYVLALDDTMGLVHLFPEVVDDVMIVNFIVLVLEIR
KRLEIIDKSILQYRLQIEVLSEKALFTLRRSPRIVHGPILWIPLTFKEVYNWRKIYSRLYVISG
ELSSSFGVPLLGASYRSFVFPIMHLYLGLLVVAESIPFAVVVSVHYLSLIAIMTIICHITAQKR
SDILFNTLTLVSDSNLSIETEEELRKLFVSLSKLKIQFTVCGFFELNVPALFAFLGTILSFILVI
SQLNV
>BgerGr380P
MMAMNQNKISDPHFESGKRHYFFSFKLIHSVFIKIREKVFHKHIISDIFVIIEILIMILVFLYL
LIMDDTEYFMKWYRWIKSIAASVYLTQSFVTRKKIPLIKDNLSTVELILSQHAQVVVVSTLF
WQKLFITTYLISLTVDIYITETNVFFGILLRFADLIVMIAIFSYCTLVYFLKGKLISLEYVLAQK
NLKFYNLEDGVFLRSDWRKVYDMRRVYSHLYKASQLMNSVYGFSMLIIFILITVSEVLNY
WHLLYITRYIVFSPKXSSNVGLLKEELVIYLRITIYIVMKTTILLVNQIAVSQSHKLFEVIQER
LLLEQVENEATKQLDKFSRQLQDNKMVFIAL
>BgerGr381
MISYTDIHSSVKPWLYLLKFFGLGMYVHGYKTESACEVFVSCFDILWPTILISFILGGSIINLY
SSLTDDNSNIPSKIRFVFAFNEICLYLTCILSIITASTVYRHNLLRILQKLSEIDRTLIPAGERIR
TNKNVKTVLVVESVVFVVVHVTYISSLVYSFTRNGREKYMKIIMDAICHVIMMLYITAFMN
IVWMLKRRYKDLNILLSYNLTDLEYKIGYVGQEYNINLAYRKRQILDGRLMFSKMHEVVV
LTNSTFSVLNLMSMVWILASCVSSTYILFEFSIPHLIFELVISVGLLVCITIICHLACNEVETSK
VVIEKMLIQLGTGSNSLHDLRALLSQLKNMKVEFTACGLFQLNLPFLYSYINIICTYIVILAQ
FK
>BgerGr382
MKSVMSPYTDIRLAVKPWLYLFKISGLSIFISNKSEDKKEENLRCYDILWPIIVTFIHLAGLV
LDIYAATNTVTWRFTETKMRVMMLLYTSTLHLTGSLSIFSITILNRNNVSTIFQDIAQIDRT
LYTQSESIRKNKKARSVLVLQTIILLVICCVFFSTRKLHFYNLSFLQTFLHEIGYIIMIVQLLLF
INIVWLLKQRYKDLNKSVSSNVKGYGHGIQIHETYTKNKIHLERKVFSKLHDISRLTNSTFG
VLILTSMFWILVTSVLTIYLMTKGIVVLELILECAANAVLLISTTGVCHAVGTEVDNSKVIIE
KILLQFGDRSPSLNELRLLSMQLNNMKVEFTACGLFEIHLPFLYSYINMICTYIVVLAQF
>BgerGr383P
MIPHTDIRLAVRPWLYFLRFCGLPVFSYEEGKIKNEVIVKFSDILWPVILILCHVGGLVWDI
HSTTMSIFWMRIESKLKFMMILYTATLHLAGALSIFGLAIMNRYNIGKILHMITEVDKVLY
TQRESIRKNNNARLTLVVETILFMIITCIFIGTKSYTYGTMNLIVKTLLQGLCHVILTTNILVF
INVVWLLKQRYSDLNKSLSRNIQQHGQDIASIGHEIQVYEIYRKRKIHTERELFSKLHDVAR
LTNCTFGVLNLTSMFWVLVTSVLTIYIVTIRLIIASLIIECIICAILLLCMTVVCHAACNEVENS
KVIIEKVLIQSGDINQALKELRLLSTQLXNMKVEFTGCGLFVIHLPFLYSYINMICTYLIVLVQ
F
>BgerGr384
MKVFGTADQIRHVLKPLYIVCKVFGLNSYFDNSKRGKELKLICISTNIFYSIMWIVLYATVF
PLHVKFSIESFELDKKIVAFSIYLSSTYVTSIVSICYLSIVKRKLFDTIINKIEEIDAEIYTSEERI
KENSIMRYYVVLDLLLSTISVSVLSIFNNICHPKSGFLEALIQNTENLSSICNSVLMCQICDTA
LILKARYKHLNNKLIKYLKTENIDLFKNSREKFSDIQPNEVGSLHISTPSNDILIRRKFHEIR
LIYSELYKTFLLVNEYYGLSMLAEIYWILASVVFFTYITMQGLNKIDYNDLCFCIGWNLLLF

RLVFTCHITTNEWKTSKFLVQETLLNINLGVATTKELRLFGDQLNVMSIEFSACGFFSVHM
PLLCTMVSVIFTYIIIMVQL
>BgerGr385C
MKVFGTADQIRHVLKPLYIVCKVFGLNSYFDNSKRGKELKLICICTNIFYSIMWIVLYATVF
PLHVKFSIESFELDKKIVAFSIYLSSTYVTSIVSICYLSIVKRKLFDTIINKIEEIDAEIYTSEERI
KENSTMRYYVVLDLLLSTISVSVLSMFNNICHPKSGFLEALIQNTENLSFICNSVLMCQICD
TALILKARFKHLNNKLTNYLKTENIDLFKNSREKFSDIQPNEVGSLHISTPSNDILIRRKFH
EIR
>BgerGr386N
LSTISVSVLSIFNNICHPKSGFLEALIQNTKNLSSICNSVLMCQICDTALILKARYKHLNNQLI
NYLKTENIDLFKNSREKFSYIQPNEVGSLHISTPSNDILIRRKFHEIRLIYNKLYKTFLLVNE
YYGLPMLAEIYWILASVVFFTYITMQGLNKIDYNDLCFCIGWNLLLFRLVFTCHITTNEWK
TSKFLVQETLLNINLGVATTKELRLFGDQLNVMSIEFSACGFFSIHMSLLCSMVSVIFTYIII
MVQL
>BgerGr387
MKMFKTVDQARQAFSPLYYVGKLLGLNSYFCIFHGGKRLAFIYSTINILYPILWIFLYATVF
PLHFKTAVESRNLDKRIIAFSIYLSSTYITSIVSISFLSVVKRKLFDKITIKIEIIDAAIYTSEERI
KANTKMKCAIIIDVLISATVAVFLTIFNTVNHPRTGFKNMLIQHTENLSFICNSILISQICITIL
ILKTRYERVNKSLFRNFKTQDLKTFEDSIGKFNDHQSIEISQASSSNDAIIINRKINKLRVIYS
ELHKTVLVFNELYGLSVLAEIYWILSSIVYFTYSTMQGLNNIDYVDVCFCVSWVVLLFRLIL
TCHVTINEWKTSKLLVQEALLSSRLGEETTKQLQLLADQLNAMVVEFSACGLFSLDMPFL
CTVISAIFTYIIIMIQL
>BgerGr388
MNLFSSADQVRHVFKPLYYFGKVFGLNSYFDHSHGGKRQKHVYYALNVVYCMVWITLYV
TVYPLHVKTTVESGEQEKTIVAYSIYLSSTYITSIASICYLSIIKRKLFEQIITKIEEIDAVIYTSE
ERIKTNSKMKNYIILDVVFSTIVVSVISVFNILYNTESGFLNNLIQTTENFSFVCNVILICQICN
TALILKARYKHLNKGLIRNLKIVNLNTYKDFYGNLEVDKINEVPNSPIPTKVTELNLKINK
MRVIYSELYKTVSLVNDYYGLSIFAEMHWILASIVFFTYNYIKQGQNHTDYIEKWVCLSWI
VLLFRLVFTCHNSANEWRKSKLLVQEALLDFHLEQTTRNELQLLGDQLKVMVVEFSACGL
YSLQMPLLCSVFSVIFTYIIIMVQL
>BgerGr389
MKIIDYTVVGCDKFKMLNLLRSSDIYTTILPLAYLSEIFAINPFPWLPACLKRFNKVVIMLD
HIWTVVVWASYLIACIYMYNGHDYVDYTNYIISSLNVSDIDSNVKLVSSIKRMSIINFIYNNSL
FVSTIVVVVFSSKYSRRKLTEAFIKFSDVDYLLFTEDEKIRMYIRGKVIIIVKLAILIILSVLYL
NYSLYWCGKYHMSCILIAMEFVTMFMNSLVLFQFTSVVILLRNKYKALNNLLDSSVEHEK
RSCEFRSNGSNSHSSSTTIRRPSHSNMVQQYRKLRIAFGHLYDSAQLIDSSYSLPILLVIFCLF
MHIISVVNLGIEYGIESEGLSESLIYCAFTITLLGGVAAVCSLVSKESKNSSIITQKIMLSFSES
HILRNELKEMYEQFNSMKIEFDVLGLFSMNLSFFSSFIAITCSYIVVLIQLA
>BgerGr390
MGNLASSSDVCSTILPLRYASAFFAVNPYQTVFDCSGKIWKMLTILDHFWSLIVVSFLSFST
YMHTTYLFSNYATFIKSDQNGTSVSGKIYESNVMKFLFVKCLYYGCLYLTSMVFVINASSVR
RKMSQIIDKFNVVDNILLTEEHHIKVYTAMKKYIVMQLGIIIFLWFSFMEYTCSGKQRKEC
RLWILESIAIFINSLVVLQVANILMFLRKEYHSLNSSLVTSCKFQFRYIKTNVFTYDSNETQ
MNASGNSNSSSQRHYRKMRLVFNHLYDIAQLINSCYRVPILAITFWVFMQVITIVNICLED
GIDVSSLTETILFAGYSVVMYAAIATACTLTVNESKNSSIIIQKFLLDSSENFMALKELRAMH
VQCATMKTKFDVLGLFTMDLSFFSSFVAVTCSYIVVLIQFS

>BgerGr391
MTNLLRSSNVLSAVLPFGCLCAVFSVNPYPIRSIIGNKYKNIARFIERIWTVTILIALIIVLYLQ
NYMAYSYFSLLTKHSENKSVVPDEYLKRRGRKNGFKILIINFIYFNALILTTITSIVTNVMIIR
PKMKEIIRKFNIVDEILFTEEERTIICTARKKMIYIKMFIIMILWTVCSIYAAYDHAIVVVFSE
LLHFFSVLIIVFFVEQFTCIIMILTTRYKFFNRLLLPATNFEFQPFEEGFLSEEMKINSTGIPQ
FATLRQYRKMRLAFSHLHDIGQLITSSYSFPVLAISFLLFMHVISAVNFYIEDANKPTENLFL
VGVSVTLFGTMTRACSLASNESKKSSVITQKIMLNHRGNFMFRYELREMYEQFLNMKVEF
EILGLFKMNLSLFSSFIAIACSYIVILVQVS
>BgerGr392
MADHLSVMFPLSCISTIFSVNPFPIYIVNNKKYKKLLLFLDRVWTVVILLSVIFFMYIRNDEA
YSVYLRLTENSHIHLNATEEIEDHSMKNFILNLMHDNWLIFARLICIIVTSYVARPKMSSIL
TKFTIVDNILFSQEKNNISYSRNRKHILFKTMILTILWVGYLHFIVYSCMYIGICYILISLWIT
VFTNSLIIIQFTGILMVLRNQYKSLNSLILSTTSNSTFEEFEHSLQELEVPKRIGLDSSTIRLC
RKQELEVHKRIGLDSSTIRLCRKIRIAHIHLYEASQLISSSYSFPILTITFSIFMQVINVVNYVF
AYKVTVIDVSETLTFCGFSVALFGAMAISCSLTTNEWKNSLISIQKVMLNHTASVRNEMRE
MYEQYSSMKVEFEIAGLFTMNLSMFSSFIAVTCSYIVIIMQFS
>BgerGr393
MLNVWHSSDIISAILPLRCVSALLAVNPYPVLSLRTGKCKNILCFVDRLWTVAAVSLLAFFI
FSKNYSTYLNYEMLTHISYNSTSNSSEQIDARREIKMRILNLLYDNSLYLTDIVSVIVTPTIA
RPKMSEILRKVNILDNIFFTKEERTKIYTEKRIYIIIQLLTPIILWGWYSYYINEWCSIFETNC
YYVIIVWISVFTSSVVILQFNSILMILKSHYKNINTILLSSATNLEFLNIEQSWNKSENINFRK
GHDEDINTIRLCRKIRSAHIHLHDICQLINSSYGFQILTIIFYIFTHILVAVNFSFEYELNLMNI
YVSIVFCGYSVVLFVTMAASCTVSTNEWKNSLTIIRKIMLNHGGSVRNELREMYEQCSTMK
VEFDILGLFTMNLSLFSSFVAVTSSYIVITMQLA
>BgerGr394P
MLNIFRSSDIISTVLPLKCVSAIFSVNPYPQHYQCTNKYKXILFFLDRLWTCIMLLYLSISICI
KNYSAYSIFKNLSNGSFNITSNVPGGDGIQVKLKTFFLKLLFDNVLNICSLFNVIITSTIVRPK
MSEVFRKFNIVDKILITQEERIKMYTKNKIFILITLSIPTIMWESYIYYIFSFCTNFVMCCIVV
MEWISVFTNAIVVLQFTSILRILRSKYQRFNTLILSSASNYEFKNIEQCWNKSDQIKLNSNH
FIGINTVMFFRKIRSTYIHLYDISQLINSIYNLQILAITCFIFLHIMSSIDYSIEYYKTDLAYMYV
TLMFCGHSVALFVAMATSCTLTTKEWKNALISIQKIMPMHTVSVRNELRAMYEQCLSMK
VEFDILGLFTMDLPLFSSFVAVTCSYIVIMVQIS
>BgerGr395P
MGNILKCSDIYSTVKTLGYVSILFAVNPYPGVFVCLENNKPMLILYKMYKVFLILSMTVCIC
LDNVYTISGSFEHADSFHASTLNESDLFKGRKVMKVEVLICIYHNLLYATNIVCITVNSYIIR
RRMPDILMKLDVVDKTFSRSTLEEITLKYSRNKLLITSKLLFVYAVLLAYIAYIFFWSTNSR
LHYYSLIVKSVSVLIIAVIIVRITSILMLMRSQYLSLNSALLSTSTAIRNPDLSDSDVGNRSISC
SSAIERYRRIRSSFNMLYDVAKLVNSSYCILILFVAFCVFIHVITLVILTIEYGIIGTDMAENLX
YCVFAIVLFGGIATACSVVSNESENSCVIIQKVILDSNESLLIQNELRQMFEQCKSMKVGFDI
LGLFKMNLSFFSSFLTVVCSYIVVLMQIV
>BgerGr396
MKIKRAIHKNIIICNSLKIFYIVCALFGLAPYYSYLSNVEKKRIKTFSNLKMKLLALAWVCMY
VVGLYLNISYNHCENNLIQRPQKLIIASDIVYIIIFGSSIACVVVADIKKFPLIHRILSEVNDNF
YTSADFSLQPKLWNIGTVADTVIMLILPILIFLSSLLFVATLDIWRFVGVCLLLSSAMANILV
ISQFSIAVVLVQRKHAVLNIYLEKQLSYQKVLNKNINNSHIAIANESFVDIALLQISTNKFSR
NLSYLRMLHCELYDVVSKINSQFGLPVLLVLCWLNMQLIFTIYYAVFFFAIANMAILFYCLE

IFMILVRLPMLCHSAVCTFQNANVLVQKLMLYNGNCSKTSRELKQLSIQLNSMKVRYSAY
GIFTIDLPFLGSVVSLVCTYFLLLVQFK
>BgerGr397
MKPHFSLLLPKMKPKKHNDLSFGFKIFYNICGIMGMAPNYFSSFDTEFQRTNKRCNFRSTI
VSLFWICVQMFGFYQSILDVNTINLKNQDTEKLLVTCNIVYILIFTSSITATIVGIFKKFPSVH
QRISEIDVSPFTSLEDSVQFKISKPILFADILIFLFLPIFVFGSHIYFIRYRELKKLCIVILMFCSA
LNTLLMICQFVIAVMLVQQKHSVLNSYLEKQLNHQKLLLSKNVNSTFKNHKIFSEVSLSLL
TTSSFSRNLHNLRSLYSDLYDVVSIINSHYGLPILVALSWLIVQLVYIVYCAIYYFQDPHLMV
MFYCFIEFMILLRLPWVCHYAVSTFIDAKILVQKLTMYYEISTTVNSELNQLSIQLNTMKVE
YTACGIFTIDLPFLGSVLSVVCSYVFLLVQFK
>BgerGr398
MGGLPVISNIYNEIKPSYLLCKITGLAPYSFVNNQLINRPANYYAFWMVCCCATILFPLFSRL
SNLRFTQFTTSLSVDAVLTLHLIINTIGGLSSIINVAVRRGHLVKAFNLIIEADRIFERCNAKF
KVNSWKNLYVVIHALITILSLTTMFSFLTTITISIEYSYTDIAYLLARFFLDLTMTSMVLFLV
NMVWFLKTRFHHLNNLLIYLKNLDFLSSARIDVNKLNDLIFIQSVAISEMRPIPKQLLITDN
LRRLRLLHSILHEAVQHINTTFGLPVLFITFWVFIRIISTLYYIIDKVRPSEDHSNYISFTISAC
VYTLYLLIQITYSCHVTAEEDTSTILNIQRVLFRQDLARNVVKELKEFRLQVITEAVKFSACG
MFNLDLTFLYQSLGVFATYFLVLAQLIY
>BgerGr399
MKGNTLSEIICRVFKPVYYSCKITALAPYTFTKSEIHDHIGLFYMLWVSLCLIILFASTVNDV
LFNNQKLQVKLVRIIKLKTILICITAASSIFNLSIIKRKNVTEILKVLTQVDLLQEKFSMTRKM
TRHKAYTIIHLLVTVLSFTVLRSIILYNSWTDPIEIQVIDITRTATMMPINMMIVLYVDLVW
FLKFRFQRINEFLRNLYNNKQTLHDPRQLSETKRCNIVSDLVSALENSSNTNSLTVSDILEI
RKMYNQLQNCVQLINSTFGLPMLLSMFLAFVKIIVSLYVFLGKDKSATESELFAILSSSSCVF
SFITLIQVTASCHFTKEESECTARHIQHIVLSNNFPYETVQEFEKFYAQVTTSGIKFSACGLF
DVDFPYLYSFIGVICSYFVVLVQLSDFN
>BgerGr400P
MSLKQFSGVFFTLLWFVLFLGALFLSIWLMPVSELPSARTMYIYSILNFVILTINLSTFVGFS
TINRKIIPRILADFYHVDTCCHYETQITRIRHRHMLYFMLHLILVVIFLEIPYGVFFHMRFVE
NPWFISMKILDKLNGISNILLLVTFNVFVTLSKEKCKNLNRFISHIAGYIKKGKIRTRSGHLT
NSVKLNRRYKLETFTCKAHIHSSSVIKIIRIIKSTYAHLQDAVHLINSAFGFSILVSSFWIMLT
NLFALYLTFYSVEVRAEDLAATAAWCIYILLLLVRIILSCHFTTEQAGNMMQLVRKILLCPN
IEQDTFTAMEKLLFQIRTSKIEFTTYGFFRLDFHFLYLTLGIIS
>BgerGr401
MKSGIYFRLRPLFFVGTIFGLSLRQHGYKSTNYTLSAIWIIFLIVNECIELYFDVQQQESLKE
MGLMLLFTLSLHSSGISSNIALTVLGRTVLPSIVEIVKNVDKLIALINEKFLPSENKVAVKIQ
VILLCVYTFIQMAFDIFNSIDPWSHYLMVADNIASFISSLTVLYFINLVLILRNRFKYVNSFLE
KLSSGSRHFPIYETVKPENNGLTFSIINHSEIQAIREIWRSLYDSACLINYLFGFPLMITTCFA
LLNILSVISHMLYENDLLFSLEYYTWFFLPVLLQLTVSVVCELTVRESHKAPITVQRILLRQ
DLKWDMEKDLQKLYIQQNCMKLGFTACGMFEYKAGFISSLVGVIVTYTIIVVQIPF
>BgerGr402
MASEMNCTGELASGIYSRLQPLLCVERIFGLSIHSRRKKTENFFIDHILSILWIIFLVVNLCIE
CYWNVLGDHTLKDKALRTLFTLSLYLSGISSLFAISLKGKSALSSIMQIMTNVDNNIASIDK
RYVPPGSKLPIIVEIVILFAYNVLILIFDSIAEVEILGIRSYKLIPDSIALFISCLTILYFVNLVLVL
RDRFMYLNSVLEEFSTESNFSTNFVIMKTTNIDYVNELINISKIRTIRNILRDLYQVSTLINA
YFGFPLLTITLFAFLNIVSLIAHAFSIIDVLIAFQTYLWSFLSVVLQMTVSVTCEMTVRQSLR

APVIVQIILLRKDLNWVIERNLQKLYTQLNCMKCEFSACGMFVYNAAFISSIVGVTVTYMVI
VSQMK
>BgerGr403
MKPNIRESQLYLYLKPIFIVQKMYGIIITDQRKEHFSFLTHALLPLSWLVGFFICILLFIANLL
TSKENLKGKVLDSMFYLTLYLSSIISIIMGRTVNRNKLTKIFEMFASIDNTIMTLDSNAKLM
RENKLSLKMQLLLLFLFVAILIISALLFDCQFSHVFCCFKNAMDILSSILNFSIVLLFSNIVFQL
DKRYRYLNSILECTADESNICCYNTANGPLFEKDRLKNVTEKMCFCRNRCLIPLWRLLFSK
LFDIAHVTNSCFGFLLLNITLWAFINILSAISFLVHVAQEYESVSIFIEKGWIVFLWFTASIGL
MLLVTITCTRAVNECKRSPKIIQKILLRRGLSEKDVKDFERFYAELTCFEIEFSANGLFAYDL
KFFHGMLGVSLSYIIIIVQLK
>BgerGr404
MADECRMSSSDVYSALAPLCFVSQIFGLAPLRWTPEKGKFLRQAWSVVAFIILFSSSCAEFL
FLPTITREGSMRQVLGTVLLMFSLHFCALSVFVSCAFINRHKMTEIVKRMESIDRNLERKL
GTLDLYRHAHCLTLMEILVVIFMEFVMGCLVVYTVSKRDTTIIYLVSTETLPAIEINIVIVQFI
NIVIILRKKYKILNMYLSKVNATNEDICFTNVKQILRVSTNKNFIVHIPELREIYIQLYEAHQ
LINEKFGVGTLAALLWPSARFISEVAFSVRGISVMSVVKCLVVIIQLAIVTVSGHNASEEANK
ALILVQVLHLSKGYGPDFRKDLDDLACQLERMPVRFTAAGFFTLDKPCFCTIMGIICTYVA
VMGQLR
>BgerGr405
MDTNNSSADNFEISIKPLILISKVFCMLPISLTIKSYERGRKEKFVDTVFVCVFLIFYGMNFC
YASYVYLFLHRSKEEKIIITIFIYFIALYITNISTLICLNFKGVNVLTILSKLYSVDNIFENNRVF
FIYRYCRSRTFIYYIIVFIVLFYVSCGMYFSYYANSKYILPYVGEYASTVMNSVLGVQCISIVT
MLKTRFVELNRKIISNLLKKQVVIPKTRLACFYHVSFGNMENIRPEKSTLLYLEEIKEIRLKY
FELYDCIQIFNSIFGTPLLMLSLTIFATVVNATRFATIAFKFFDLEELQSYILVIYLLVIIALGL
FLIMYLALLCHRTCDEVNKTVQNIHRVLTVCIISYDLQRELHKLQNQLKDMKVEFTVCGFF
TLNLPYVCTFVTGIVMYVLIVIQL
>BgerGr406
MSPSRMSMADLYNSIKPLYFMSKLLGLASFIFVTDKDTSEVTIKNTSALSSLWCLLVSVIIF
GSCVHHVYFFATYIYINSNMNYTAAEFLSTVILYVTPTVSVITSYLKRNTMVDIIRKLASIDE
KLLESNKAVVYKRTYWIMGIELIVATISNVVIFFFNNYAYNTRFVDVKFYFQSVAHSMIVYS
VIQMVNIVWCVKNRFSVLNTKLEEFFICEKGLVKNNPVKIQTIDDVNTLRTIHLVRIVHSEL
YEVVISLNSYYGLFVVLVSLNILINFSSHVFYIIMNFNSALSIPTSSGQFYIFASSYIPWITLYLC
ELLSITVFCHLTKREAFNLVTLVQKIILYPILKEEVSRELQELCFQVVNQKVAFNACGVFPID
MTLFHSVFSSAITYIFILVQT
>BgerGr407
MLISKASEFYSAIKPLCFMTRLLGLLVLPIDTVCFKICNIIWCLIVAVCIVGVCCHHLYVFWYI
IYFKGAIISTAEDIFAKILLYGSPVMAIILGPVLNNRKMYIILEFISYIDRKLIKNSSVYSSVLKI
LIVEILFGVFLITSLFTLNIYAWKGFAYLIFYECCIHLITLFNILIYVNIMLYVSYLFNKINYELY
NFGKNKVQVFGSLSLSEEFACDYIYRLKILHCACFDVIKDTISYFGWQLLFERANIFVQFTA
SLYNTIRVSKHCLQPDHKNNFCTSQSTSLILWMSFYVLKIFLISFSSYLAKKESYKTLIIIQKL
LLKNLGITQVSIELEKFSLQVSGTHSNISTSKKFPVDMKLFLRMIAASVTYVVMLLSFE
>BgerGr408
MATENYYGIDMTTVKTNKYLHRESLHAANMPFYFVTKLMGLTSYSYGANNSDTFAYISVF
WSSLVFCSVVGACSHHVYHFLKTVYVRETTNFMISDLLSKILWYFSPASAMLVGLLIRRKR
SDIERSISLIDTKLLGKECSKIYVSTFFVQILAISFCILCIICMTLYSTWAWSSKFESVFEYYET
SSMFLSLLMDVHYICLIYCIKHRYGVLNRELEISMGMRRKPRLLQNIVKLRHAHSELYDILQ

LVNSCFGFQILIEIASMFIQVVSRSYLDINMAKHLLTLKGDIFYDYLPKVIAVLNVVIFNVLK
MLGVTIACHGATRTANYTVTVIHRLLIRRGLSGYICSELLEFAKQVVNNRATITAFGVFPLD
MSAFLGFSGASITYIIVLVQT
>BgerGr409
MRNPKAMVYMSVKSIYFVSILWDLAPYTFKVNKQNRKEEFSYSNRNLMLPLLMIFILLIGC
ITELIYVFKTDVLTSSPTRWIPEILYLTFIHSASLLSLVFGITFNRRLPQKIIEKIDVIDFLLLEE
NYRIKLYQNQHVLSVKCLFFLIMFLFIIHGINILNSDDYYEMLFKIIETWTCLIVTVLIIQFVN
VVIMILQRLKIINCQIVLLINSGKDPLNTVDEHGNYFIQASNILFRKRNFLNNQKYLEHVDQ
DCTYSLERIYTALYDISVYLNSGYGFQLLFLLTGIFINIVTCLYDGLDFLLNQISPSRHHYTVT
VQEMTNGFLQTFASVTYTIVIVICCHMTSQESIKLTVLARRLLLCHHLKSYDFEKLSTLTSQ
MNIEFTACGLFKLNLALLTNVMGVIMTYIIILFQNH
>BgerGr410
MIIVTPEAPSLQQAYQTAGLEPLSMYGIVWTGILLLYELFGLCYMICVVLLKPEASAFSIIRGI
ITIPLSHISSMISLACALSLIHHNTRVLIHNQISKIDKYLSYKKTKDHTLIIVTSLVIFLIPFHAI
LIYIWSYHTGMSLSTVHQIILHFSNFMSTILTLKYIAYVQMLGLRMQILNKKLDCKDVNCFQ
ELYNILYDISNLINSAYGRQILFAMLSNCSTFISNMYGAIRFVEAVLNNYADEREFGHDMAY
IFWTFIGLFKMAAITLSCENTQRECKVLLNHIQKLLLTEDLHINSMYQLKLFCKQVTRNKIE
LTLHGICVLNLSLMYKIIGISTAYIIVFMQVH
>BgerGr411
MVPPPSLYDAVSPLNYLSLIFGMPAFKRSPSAWSLRFQLFWTLILIAESVSGCIWTSSKRIN
GNDTGGRPIVQIIMMELYMSGIYVVSTSALLLSIVRLRSQIPFILEQLTTIDNFLLDNRCKKS
VYRKTKVYLYVVIVTMIAIFISLRFHAATIWVKDFSIVNFLPVIPVLVGQITNIQIVTLLLLIK
QRFRFLNERLKEYEAKSNTEIFHSEILFHEVNQEVTIRNIRKWYSDLHNISNIATQCYGPYL
VLQLLHATVCFIISLFYMIILSYKIVQTSLTFRFFMHMINLISLFLLRLARVYVTLNTCQTLSS
EASGILDTLDALLFKKVSNVTDNREIRLFRNQLSVQTVKVVPCGLFTLDFSVLHSIIGITVTY
LVIILQY
>BgerGr412
MVPPLSLYDAVSPLNYLSIVFGMPAFKGSPSAWSLRLQKFWTLILIVENITGCIWISLRRIT
DNHADGIVHILMNEFYMSGMYIVSTSALLLSIGRLKSHISFILEQLTTIDNFLLDNRCRKSIY
RKTKVYLYVVIVTMIAIFICLRFHVGTLWVKTFSIDNILPVIPVLVGQTVNIQIVTLLLLIKQR
FRFLNERLKEYEAKSNNEISFHEVNQEVNIRNIRKWYSDLHNISNIATQCYGPYLVLELLH
TTVCFVAVLFYMILLSYKIVQSFIDLRLFMHLLNLMSLFFIRLSRVYITLNTCQTLSSEASGIL
DTVDALLLKKFCSKTDNREIRMFRNQLSVQIVKIVPCDLLTLNLPLLQSIIGITVTYLVILLQ
Y
>BgerGr413
MAQHYGLNLLYLVSVLFGQMPIRAENDKLLYKHIIYVIMWFICLIFMLCFSIYAYLKIVPESR
KMFSIFLAWTAYVILKFSVCLSNSFLNLTIYREHLSIIYCHFNEVDSRLYEAKYLNTLYTRKA
KSVRFQIIIVFLIPIIINICDCFSQEGAPVSSIATVMLGTLPLNLNSVMFIHYLNYVLLLKEKIM
KINSSLSNHLASQDMSDIKFHYTHGAGLQLRHYSSEKWKIRDLRIAYSELYSASQVINKHFG
IPIFLGVLTIVLRVISAAFEGFYIFRIASRKVADYLNATQQLCLCAYYLMFALALALCCGATE
VEALRVTTSIQKRLLYSKMNYNEFEELGMFLSQVKDMTVQFDVCGLFTINGRLLGALTGIC
VTYFVVLYQVNHVG
>BgerGr414
MAGYVISNCLYYSLQPILILAKFFGIFPFSFTRKGRYEKHVLYNLKKHLFPSFLWLIILAITGT
FSLYYSLYLEDHSLNLTTGYCIYMILLHITTFVSICKICKSRKTIPLIFEKFYLIDQLFHEENTT
IIYHNTRFWIIKAILCLFLYLTMIFGCLLCSYYDGTVRSVFIAVCEHLSATINIVVMFLFIVIVK

MLNQRYEQLECRINNLDNNTYKRLPVLIHEYILNFNNSLHHQRMNLKRSLEYHKITTVHQ
QHFHLYETAFFINKVFGIPLLLEILSAQTNCVTSLYDGIDILKSGNIRFEGYLESVSVIILGSW
FIVSMIWMILLCHTTPSHIEKQSLAIRRLLVNRAFCQETELELKTLLAEIKAMTPHFTAGGF
LTLDLPFLGTSFSCIFTYFVIMSQF
>BgerGr415
MKSGARQHDLYSSMKILLQISKAFGVFPPLVFKKINSEENIIVRNSFIVFGTLWTTFHLGGIS
LAVYNIAVLKSYIEPKLQVMNVLLIVILYSTSLVSIMSLCCIKRFKIIKILKLITLADQYFPHQE
VFILHKNTRTYINKKIILVFIFLVPEFISTGVYYYDGTILAFTQTIWDFSSVICNIIVMFQYAIL
VRSVGLRYNLLNKFLINFHRITNNKKLDCRSRNLFVFTAWKEFSQNKLTVARNRLQSISDE
RIFIIGQQYFHLFELVTLINDVYGFPILMNIVLALFNSIVSLYEGTVRIEQVETQNFTFFNLET
VAISVLGILFFVFLLWFLKECHKTVHEANIGIIYIQKVITTCDLHFSVAIELKEVLYQLNNTK
VSFTAAGLFEIGLPFLNTTFSCILTYIIIMAQFR
>BgerGr416P
MNSDLEELRYLGLSPFITILKCFGVVPIVGLKSHYVKRHHFQKLLTFSWIAMCVFAFSWHL
NILRIAIFNGLVKKTNFLVISYRIAMYMKPVILLTKIXIIDDLEKIYKIDCQLFDLRKLVRIYKS
KRIYVILKVIVLIIFNLLITGEYMILTWSFWMLLLDTFIVFFNCMTGLLHIELIKLIMERFKQ
MNWTFLCLKKNSKYTEGAWNFNIVYVTNIVTVLNESLALKIWEFIDIYCVLWSVLYKFNN
LFEIFNALNGCIFLCDAITMFYHFFIQSIYYESGYTNILHTVAQVCVLFLKAIFIISISYTCYGT
EKESGSLLVNAQQLVLLPKLQQSTKIVLNKFICQLKIMTVRFTVYGIFVINLKYLCTLCGILV
TYSQILHDL
>BgerGr417
MEIKLKKEDNSIGLSPLLLITKAFGLTPIEKSVIFGKKKSITSFFAKLPTLWVIIPCIGFLHSTC
HDLFVAIATGSLDRFTVLGITFLITRYSKPIVLIYQNVRNSHSVSTVVMTINSIDLQLFSYKR
RSFMYKRKKLFVVLHILITFLPTCYAFYACLIYYRNIPFSTMIFVSDLMNFLSKLLYNQLVIA
FKDRFKSLNKILQTKKLITNKPSISTDKIRTLCHFYTDLWNSLNQLNKHYELFHALDILSYL
ADIISVSYLLHVEGKHSFFFHTHKNVFREIEGILWWGLWYALCTFFVLHTAYSCYKTQDEV
DCLKITVLKLSMKSGIGANMNSEIENFLSILKVLKVKFSIYGIFSLNLQLMCTLCGILVTYIVV
LQQM
>BgerGr418
MVKENFEIYSFFQPAMYIYKIFGLAPFSIHTGKNGSKYISTTKIDLIYNVSLALLCLLPLVALN
EYFQSTAYNEESHITRFADYFSLYSLQFSSFSSVIYSLINIRKFINFFTYINEINSKTSRISLIVS
YKYGRIFIFYEVILKILIDIAFTTQSYFKEIKVYSYPFLKYCFVITVPMLIGCVMESQFIALLYV
LKLNLESLNKYTETFPCSDMFGGPRSLYNNQQTLQIAFPVADLSAVHHSLVVCAQLLNDM
FSFQILLRFTFIFFLITCSIYYTIYIIIFTESKELYFCTLWAIWTFARLLIITFFSKKVCHEVKRT
SFIIHRLINRRICSSSENNKLMELSQQLLHCNFNFSPCRLFQLDFTLIYYLVGFVTTNVIILTQ
FEATK
>BgerGr419
MAKIQESTKLYPFPSSGFYLYKSFGLAPFTISTKESGEIYISTTKTDIFYNICLALLSSVAFFSL
DEYFLSEKYRAQSYITLLVDNFTLYCLNSCFLVSVVLALIKQKRFIELLRHLYEFSSRTSQLNI
TVSYMDEKIPLVLKILLPIMMEAAISVFCYFCVNTNYNYPFLKHVLVNTFPSMVTSVMETE
FHELATALKSNFVLLNAFISEFISYPQANETKFRSVCSEALKQRTFPVKHFTIIYRSVVYDAQ
LLNGIFSYQLLLRVIHSLMIVINMLFYIIFMNIFSEFKGINPELCLCIFWAIWNSGRLLFIISVC
SNIYHEVKQTPIIIHRVINKGVLNNTENSQLMEFSQELLHYNLNFSICGFFNLNFNLVYIMIG
FLTTNIVILIQFEEYNQALRSPMKQ
>BgerGr420aP

MKTWERNNVLENLLRRFRIGTQNEEKIYRSFYSNMKPLAILLKIFGLFPFRNVFEDDGRLL
QYNFFSLHTFWGLFLMCICLVSNFGSFFWWGIIILIDTVLLIFTSICFDKFFPKIIQQIENFDL
AIILTRRPYNPRTILTSGIFWMTLSIIGIVAQFACIVWLWMYEGNFSMVTHILGITAAVLNG
VFKVLSIELFLFZWLNISARFRCIRQNFCVLLKARNDYQELDKMLEEVRLLYSQLSKIVDEI
NHYNGPRLASFLSIIFFQIVIDLFVSYTEGGQASPIILLAILMQLGSLEILAFAGDDVTRAAQE
MVDELKGISLLNLSEKSQYQIQMFAWQLNASPIEISAYGFCAINRKLSVPFTMALATYFIAV
IQLQTQFKNLSQYFWSNYSNYHLID
>BgerGr420b
MKPWSSWEKNLCTRFRIGSEHKHNTVATFYSNMRPLALFCKAFGAFPLTNAFQTDGRKL
HYKILSLHALWGPFLMVICLFFGTQVVQHKWVKIIVSLDISFIFITSLYFDKFLPEAIHEIENF
DASSKILGKCFSPQTFMTNGRFWAVLSAISIVTVILGCARLWVDVKDDPLSSQIFKVIVTIP
DVIYRVLYIELCLFLCCNTSSRFRYVKQNLQNITTGTRHEFYTRLIQIRLLFNKLTKCVDTFN
RCFGPRLAWLLFIMFVEIQADLYAFYLGWQDSSSLVFILLNLFNLQLLTFAGEDLKNSAQE
MVDELKGISLLNLSEKSQYQIQMFAWQLNASPIEISAYGFCAINRKLSVPFTMALATYFIAV
IQLQTQFKNLSQYFWSNYSNYHLID
>BgerGr421a
MKKDGWVKPNNISKNLSNSRIFPTNEHHDTFYVNIRPTAIICKLMGGFPLKNALKKDGKSI
KYQLLSFDVFWTLILPLICSIGYVQYITVPWNILIIPIALSRAVASSLLCIYHDRILPEIIQRLEY
FDKKYKILTGSAPSKNSFGNTLWGMLTLLSFIAAIVGIMSMLAKDPRRTMLRKSLETAFIL
LPWFQYQAHCIFYLLLCFVIGSRFRDLKSHLRYIVKSSTDDQLASISTIDSKFQQNVDIRIEN
TRILHAHLALTVDRVNKCYGARLTSYVILCFLQITSDFYQLIFGQEMDITTAMIFFITHAGCV
FILGCFTDSLCKSAHSVIQELRLIPVTKLNNSALNQVEMFIYQLQTCPIEITTCGFSVISRKFSI
SFLLALTTYLIAIVQLAPQINDTLQETFNACVLLSNKRLSNEN
>BgerGr421b
MKTNGWMNCNDNEKETKQQLSELSIFARYVKKGSWNNSIHDIQTKKLSRSTGPSKKKHN
ETFYNNIRSTAIVCKIIGSFPLKNAFKNDGNLLKYQLLSLETFWRVFLPLVCSIGYALYVTKP
WIMLIFPIYLFRAAAFSILCVYHDRILPEVIQRLEYFDRSYKTLTRVAPSRHFFGNGFWCTS
TLLSFMAAITGIMWMLAGDPRRTFFRNSLETAIILIPWFQCQAHYVLYILFCFTVAIRFRDL
RSQVRYIVQCHYQQPWMFITDSRAQQDFDIRIENIRILHSHLALTVDRLGKCYGMRLTCYV
IICFLQTASDFYQCTLRSDMDFKTATVFIVTYIGCIYIMGTFTDCLKNSAHSVIQELRLIPVT
KLNNSALNQVEMFIYQLQTCPIEITTCGFSVISRKFSISFLLALTTYLIAIVQLAPQINDTLQE
TFNACVLLSNKRLSNEN
>BgerGr422I
MAKMFNTKPKSFYSNLKPIAIFCQYLGEFPLKNVYQSEVQHLQYKYISLQTLWNIVLTIFFV
FCFSKLKKIHWWNFSMPFNSTKVFMSCCANIYYSQHLPDLIKEVDNFDKHYNKVHSLILV
EKKTNGYSWLILSITILVALPPCVLHMLLEVECIAKAVPDTALAILPWYQFQASVILFLFFCI
NISSRFTFLKELWQCTLATIHQRKDFKEPELNASRKSEAISSSERLEEIRILHAQLCSAVDKL
NTCYGIRIAIYLAFNFGDMLYDFYEFIQGGITSNGPIGFFVYHIAIVFFIGAITQRLSDSVKLFI
KQVTASAVYASAGGFCALSMGFITSYLLALTTGYIAVVQLDTQLDQQSFNTTT
>BgerGr423
MAKIFNHKPVSFYSNLKPIAIFCHYLGEFPLKNVYQREVQILQYKFISLATLWNITLTLMFG
FFYWRLIIIRWWPLSIPFNIARSSIDVCASIYYNQHLPDLIKEVDSFDKYYNKINSPIMVEKK
TNGYTWLLISIIALISIPPCVLHMLLEAECSASALLYTLYATLPWIKFQASTILFLFFCVNISS
RFTFVKDLWRTTLEQGRDKESELNVSRKSKIFRSAERLEEIRILHAQLCSAVDKLNNCYGT
RMAIYLAFHFQELLCDFYQCIEGGLSSKGPIGFFIYHIASVFFIGVITQGLFDSAKEVLKEVG

MLKISKLDDQSISQVKLFIKQVTASGVKASAGGFCTLSMGFTTSYLLALTTGYIAVVQLDAQ
LDQENSNITT
>BgerGr424
MKVVPNGKYNSPETKTFYKELRVIMLICKFFGVFTLQNVLEESGYHLRHKMLSWSSAWG
PFVIGIVPIICMDFDKGEFHIYFKAMHCTRGIICCILSSYYDQYLPDIIHKIQYIQLKTDYYCAT
NSRHIKCPGTSKILINLMFYGSIAFIVLNAAIEQLVNGKSILIAIREVFGVLNLLPRQAYVMLY
IFICQRITRLYCEIKLCWKIYIQKVLESYAVGERSEEQLEKLRLMHAELAKIVRVLNNAYGT
RMAFYIAGIFVEVLLDLYIFFFRNKYSFVQALYYIFNGVTLYTLTSAADSLSDASRSIVFDLA
KTPITKFRIESQNQLKIFLRQIVVQRVQMSAAGFCTINRRIIISILTAVTTYFIVIYQQFQPTN
>BgerGr425
MESSLKVNLVTKYGIVESMNSVEHLKKTDTMIFVNRGAHSNINRTNEIFKGLKASKLQVG
RKYSPMDVSFYRDIRPIAIFCKFLGLFPIGNVSKNSGQLQFQVLTPDTLWGVLFIGNITGLCL
YHAILLNVCLPVDFILQTEGFLMVLLSCYYDKYQIDLIKQIEEFEERIKFLDNITYRKFIIKPN
AKYILYGVCSSYVILSIIDIFTICYTAPFTALDVITEICSFFIFLPRHIALMIYVYYCYNIKLILRA
LTNLWTVNTRSIVSYKSSNIPIAWEKLLENLRILHAEVVDVVLLLNSAYGVRLLYFVFLSCM
EVIFTLYELSFGDIRLKYECSVINIITTYIVISYTEDISEENEILAESIIDVPISSLDEACQQQVEI
FLNQIMMKKLEISAAGFFSINKKLIISISMGVTTYFIAIYQLAQET
>BgerGr426
MVSCMHLGVHLWAYCNFSIPFNCTEITLLDLIVASILVTRAVYKSPNTAQCLNLLSNVAEQ
NQCTLKTASIAFLPFSLLQFSISVFMTTTSFHSILLNYFFPLVAIAVENIVGIMEFVLEVRFTS
INKELESLLLQKAEGCGFRKSNVCTMPFRVITNQKQALARSKRARMGFKTCIKWIRRLQNI
FQRGEIEPSKRAVCPVTFCESIRRFEQSGSAIRPFPGDAQAANDPLQQLFKNHGRNFNINT
WDYLQDLRVLHCRLCSVFTCVNSRYGLEMLTVTTVNFCQLILIMFLGIRELRKQETIPFVSF
TIMLVHFIFHMARLFYVCYRCGKVLSEAEKTTELLLKLSDRNTEPMLKQEISVFLLQLHSM
KITFSACGFFNLKLSLIRPIIGAVTTYLVMLLQFQSNGTTGNSTSIHQNVSSLENEDSY
>BgerGr427
MKPLLAIIFFVSKVLGVLPLSCTKSLFFKRSITGIALAIINSLNIFIYIYICTRSFDVGFIVCCFAS
MVCIINSLWFQTKLANYLSEIAEITRKLNVYFNTILMFLILSFEIFQIVSPIYLVSGQATPTIPI
AQAIWIFAIPIAMECQYTVFCLILQYCFKSINFQLKSFLNRSSCKGEETISNNKWNLRLHQY
SNQNIRQNIFKIPWHNQIFPFDKSKTFRAHYRHFSASDITKLKDFYEQLYVLCTHLNDIYGF
QMLVTTTMSVSVVIVSAYNFVESISVKNAIWSLLILIHRLLKLSYICYRCDKTRDEASKTLSV
LHELSYADCYPSEVQQFERQVKASQVTFSAGGFYTFEIPLIHSILATTITYLVIMIQFNSK
>BgerGr428F
MALKFTKLPLIFLPIYYTSKIFGVFLARYTAFPRQNEGNFKFYSLNLIPTLISTLLWIYSLVFF
FTSEPIYLLSENKRYPKILSGYAIATMKGISLMSSFTGLSVAIFRAKFIIDAFNKCLNIHARSQ
MLLKSCKFSVLLVSFSVLLFISVYVYHICFVVIVSSNSASGVYELGLSLYFWCNFIPIVVEHQF
VTGCFILGETFRGVNEVLTSFKKKNQCAEKLFEDFSISQIRSCKTCKSLEFVRMLHKDSCEM
SLDLNLAYGPVILINFLACKVALILELYIWVTTFITGKLYLGKSSNVALYYTAEGFHMCYVF
ARMFSVCYWCNATAKEASRTKHQLAIIEGKNFHPKFKFELEKFEKQLKLFTVKFSACRVF
PLNLPHICTIVATTSTYLVILLQFQSAGD
>BgerGr429
MYKMEYNNIIYQAFRPVFYFSKVVGFMPASYKRKNFKKLKTIHVHPTSLSLEWSWLGVVY
SCVWIAFLVLFRFVFVPAWGRPPPPPPPPPLDMFNSSEFHLNGSEGNDTFRGPAFPRMNV
IASLNQILDAICSVLIVLTGVFGVRKLPSVFRNIHLLDDYVDEDGFYLLGLVERKTLLRRGIIS
SIIFVVSCFIYTFSGTFCASYVAVVHGGGHLKDITPFMLQDIIKLGNAAGEAQFLTLSVFLRH
RFKILNNGLKFLHTNINKVINNSETKPTLQIWSLRPVSSDWYSRTATGRIMPINPLKIPLPH

NRQRDGPRSTTLFNQDPSMDTRDSIADVLSRLCRWHRRLCDIIDMVVSCYQLTLIVSIAYC
FGSSVFRIYTFLTNIDTLHIVSTTPEIVWCLSFNCRMVLLSVIPALAVKEASKSRTLVERLNN
RSLDKNSKEEIAIFISHLSSRDISFSICGFFTLDLQFLRSIAVAIVTYVILLIQFKIK
>BgerGr430
MASISINSRSTNEISANCADNSFLPIYYLCRLLGTTALFRRSRHSLKYKVSVFASLYMILCICL
AILSCHMVINFFNTVEVYVATPKSKGSIYFPIYMEPVATCIVNAVITMNLLLKSAQIVTLLQT
IHNFDNANVGICMSLHNESFNFCLALSIAYPLMYLLVKIIRHIHDILNINVLITFFLVDLSHM
STMSAEAQFLALCYLVKQRYKMLNSKLEYICSSGYHILWKQESKWSHNEVQDFNGKLPHT
LELIRKSHIQLYDMIEQLNCIYGVQFFFLIVINFIQIPTNMYNVAKLKNGDMTLTITMSIWCL
SFIIRFFMALKMCSLTTYEASRSSIIVENMKRRLLQPDFKEELLLFSRHLANHQVSFTACGF
FKMDMPLLITVIAAGCTYLVIMIQM
>BgerGr431
MSVYTSVQPLLRVSQILGLAPVTWPRLKPSGLGSAYTCAVFLGFLAWFVYTTCLAVTRDYP
GRIATYVVPDLLNSGALYLTSLTSLALGATLMRSKPEAIMRLLMQVDRVVCPSAAHKRAH
YVVSLQIGVLVVSLSVLAVFDNWVWTGRLWNLHDYTGRFLAHLVNAVVVLQFSNLALLL
RHRFGKLNHRLLLQQDEDMFDDFVPEKHHRATNNVVFEKSPTPVAARPNAPVTVRPIRT
SETESDDNIFALRRAHALLCEVACMANAMFGAQILLALTSEFIGIIWLLYLTLVSGGSQKTL
CLSAFWILFRCAKVLFVVCACHVAQAEALLTGTLVHRVLLHKEALKQQGGRLVQLQRFSQ
QLMHSRHQVHFSACGLFSLDMTLLHSIASAVTTYIIILMQFHAK

897 BgerIR proteins
>BgerIr8a
MILRTALTVFVIFPPLLAQTSVKFLMLVEEQQKKVPNLYADGLSEAQKGNNDLALNEQTIY
INREKEEETFKQVCNEIQKAVTVIVDLTWTGWSRIRVLSESTGIPYIRADATIGGFVQVIDA
YLTERQSTDAALIFQNEEELDQALYYLIGNSIIRVIVLDQLPGNSTEKLLAMRPVPSYFVIFA
NTENVIKLFKKAQDGKLVTRSERWNLVFTDFKYAEFQESSLDILTSLLTLQKDVCCRLSGL
EKGCNCPDDFEILPAYLKEVMLFLSDLLMEMKRSGSDLSIPVSDLGECQNLDAKQADNST
RDKFYETMQQVLNQHTALGFNQSRWLLYYRNDMDIVVANSSQRDKVATWTMEDGLMV
KPNLTLAPTRRFFRVGTAEAVPWSYRKLNPTTKEVMKDDKGNEMWEGYCIDLLQRLSVL
MEFDYEIVPPANGGFGTRSPDGTWDGMVGDLAMGETDMVIAPLTMTSEREEVIDFVAPY
FDQSGISIVIRKPVRKTSLFKFMTVLRLEVWLSIVGALTVTGIMIWILDKYSPYSAQNNKGL
YPYPCREFTLKESFWFALTSFTPQGGGEAPKALSGRTLVAAYWLFVVLMLATFTANLAAF
LTVERMKSPVASLEQLARQSRINYTVVNNSDTHEYFKNMKNAEDVLYNVWKDITLNSSS
DQSKYRVWDYPIKEQYGHILQAITQTGPVENATIGFQKVIDQEEGKFAFIHDAAQIRYEVS
RNCNLTEVGEMFAEQPYAIAVQQGSHLQEEISRKILDLQKDRYFETLSGKYWNSSAKGDC
PNNDDSEGITLESLGGVFIATLFGLALAMITLAGEIFYYKRKKMTMVSAPKPVNEPPKKQI
TIGKEFRPVMEKTMPRVSYISVFPRNQLY
>BgerIr21a
MGVVFKIIVTIIIICITSEQMPCVLIVNGSEVNVNGVKSNSYDRRQHNKCFVKMQNSEEPLE
HLKGRFERSQNLRQTGSTNNKINTNRNKNQTHNSADQKSGFVNIMLSRVDLITGSSSYNF
GKIEKDNSGNQSGFTRYRKLQNCSHNNLPAVVNLIKFISKEYYKNCATVMLYDEYYAKFH
RLMTPLIKEHPHSFLHGATQKHNFSKIHDKTCRHFILFLRNLHETTTVVGDLVFSYVAVVT
DVSKWKVKEFLQSASSRKVINLIVIRTDDILQQEANNQEILMYTHELFVDGLGASRLQVLT
AWRCERLTRPQVNLFPEKLKSGFKGHQFVISTGYQPPFVVQRGFYIDGPKRIPIWDGLEIR
LLYLLSEVLDFTMTFIQNDDARYIARASNAVVKDLKEGRADIGVAGIYVTPERNKVITLSSA
HSQDYATFLTITSTALPRYQAIMGPFQWTVWLALTLTYLLAIFPISFSDRHSLMHLLKDP

WQVENMFWYVFGTFTNCFTFKGKDSWTNSKKAATRMLIGWYWAFSIIISACYTGCIIAFV
TLPVYPARIDNLKQVIGHRMHVGTLSEGGWRYWFNDSADPLTTELFEKMEYLPDLETAM
FNVTQAYYRDYAFFGSFAQLEYIVQINYTQRSQRKTYLYLANEELVPFSVSLAFPYQAPHS
NIMNQKILRAVQSGLITKLKRDLEWDLQRSSSGKFLAAVSSAFKPTKMRERKLTLEDVTG
MFLLMGAGYGLGAAILTIETISWLIKLVNRKCLNGKNPEDARQTSERYNDQQAGPAFMQI
ESTNTRYAQRSFTEHSPYNLEYLQNELVPRRRYRDSISLNRSVRDNSIPNRAHVEDAECSV
AVDYFGGMVQPCVSRQHGYHDSISLQLVPLKENTDENRAFTF
>BgerIr25a
MALENRLLFLLLAMFFDFSASQKINIMILNEERNWEASDSFRAAQEYFKQTPTLGVEIGNL
MNVTGNTTDAKTFLEMMCTEYNNSIAEKNPPHIVLDFTMSSVPSETAKSFTAALGLPTIS
TSFGQEGDLRQWRTLDEREKKYLIQIMPPADIIPEVIRSIVVSQNITNAGILFDDSIVMDHK
YKSLLQNIPTRHMIVKVDQQGPRAQLERMRSRDIFNYFIVGRMTTIKKVFDVAYKKKYFM
RQFAWHGITSDSGNVYCDCKNATVFYVKPRPNQEYTDRYDLLKTKFGLSGTPEITVSFYF
DIAVRTLMAAKTIMKGKPYWENYVTCNDYDEKSRPVPDVNLMKAFTEVTEKPSYGPFVL
KSNGQSMMKFNMDMTLVTINNKIPVKSVEVATWEADLVKGLDIKNGDKMVENSAVTVY
RIVTVVQKPFVMYDGVDDKNRTKFKGYCIDLIDEIRNITKFDYDIFEAPDGKFGNMDENGE
WNGMIKELILKNADIALGSLSVMAERENVVDFTVPYYDLVGITILMKKPKAATSLFKFLTV
LENDVWLCILAAYFFTSFLMWVFDRWSPYSYQNNREKYKDDEEKREFNLKECLWFCMT
SLTPQGGGEAPKNLSGRLVAATWWLFGFIIIASYTANLAAFLTVSRLDTPVESLDDLAKQY
KIQYAPLANSSAQTYFQRMADIESRFYEIWKDMSLNDSLTDVERAKLAVWDYPVSDKYTK
IWQAMKEAKFPDSLEAAVARVMESKSSSEGFAFIGDATDIRYLVLTSCDLQMVGEEFSRKP
YAIATQQGSPLKDQFNNAILQLLNKRKLEKLKEQWWNQNPEKKRNCEKQDDQTDGISIQ
NIGGVFIVIFVGIGLACITLAFEYWWYRFKRNPQVVDTGNVVVQPRTIPTAGGGKMDPLT
MPGFRPRNTGFSERPFTQRNAALAAVNNP
>BgerIr68a
MPILHDINCLAIINDDIHQDVFGGYFFKQLHLPYYKVLIQEKEDLQSPNFKTLSVIRQVKRS
GCKVYILLISNGSKVARFLKFGDRYRVFDTRARFILLHDQQLFQTNLHYLWKKIVNVIFLR
QHRSSYQPGVFSDKLVFPWYDISTVPFPMPIRKLLIPVRLDTWHEDSLFRKNAELFPDKTS
NLKGENLRVVTFQHLPGSVKLAINNVRLDVKILNTLGRVMNFKPNVYQAEHADLEKWGR
KQLNGSYTGLIGEVTSGKADVALGNLQYIPYYLELMDLTVPYTAECFTFLTPEALTDNSW
MTLILPFKPVMWAAVIVAMALIGFVFYALANYEQYVIQFKPPSSRVILVKERQREDKTEPT
EEKQTKTIQTAEKRVTGEGLYLFSELANGILYTYGMLLMVSLPKFPTGWSLRVMTGWWW
LYCILLVVSYRASMTAILANPAPRITIDTLEQLVEADITCGGWGEDQKQFFVTSLDPAGQRI
GFKYEVTQDVNDAVERIARGEFAFYDNIYFLQFLRIKRQVEQRKNQTSANATEKAEMKKL
PQERNLHIMHDCIIHMPVSLGLLKNSPLKPHMDKYIRRIIEAGFIKKWLNDAILRVMSEEIK
EDKDDVKALMKLEKLYGAIVALGIGYFISICCLIGELIYWESVVKKDPFYDKYDMRMYYKL
KKTTKKQIRFK
>BgerIr76b
MGIGAKLVGALLAHVCVNHDVIIKNGTNLQGEHIPCDLWTPKALHGQHFLIAALDQPPLS
KRVGDKNYTGIVFDFIDILKEKYGFTYDVTFPPSAEENIMGDADSGIIGRVYKHEVDMAAA
FLPVFHDLDKLVNFSTTLDESNWVVLMKRPSESATGSGLLAPFDETVWLLILISLIAIGPTI
YAIVFIRAIVCKNDEVLATVVPLDNCIWFVYGALMKQGSTLMPVADSTRILFATWWIFITIL
TSFYTANLTAFLTLSKFTLPIEGPKDLAKNRAGWISHKGTSLEYQVENNKDYEYLNKTVR
DGRGRFLIEKDEDMLLMVKNKKFSLLRERRAVEYFMFRDYLTKAENNVPENERCTYVVT
PKSFMAHGIAFAYPKNSTLAKLFDPIFQALVETGIVKHLLKKDLPPTEICPLNLKSTERQLR

NGDLFTTYMVVVIGFIAGIAAFIGEVMYTTVKRCAAGTKIEVPDTDWNGNVGYKKSQLFP
PPYSVFMQQQPPPFGKHQNINGRDYLVINNKTGGPQLIPVRSPSAFLFQYSA
>BgerIr93a
MILRKWIVSFFLCFNLSSGADSELSGFTATLALVVDKNFKGVNYQNFSMKMRHYLRETIA
QHLKHGKLYVKIFTPSDINMPKDTLAVLTIMPCENTWDLYYLFQKEEFLHIAITEGDCPRL
PRNEGITVPLVTIHHSSSQVILDIKTTQLSSWTTCTVLYDDSTDIQMVERIIKSLSVPSLGRE
DNVATVSVFKIGGSHAEWERRKSVTDLLQDFPTRILGSNFVVSVSNDIVGILMKASKSVGL
SNPENQWLYLISDLSALSHNMSGIISLLTEGENIAFVYNTTSTDKNCKGGVYCHAEELLKSF
IIALDRTLVDEIEMMEQVSVEEWEYIRPSKSKRRSDILHLMQVDMNDGGHCDDCSSWTLE
AGDTWGLDYLPTEQSRQLIPVGKWTPQKGIEVVGHLFPHILHGFGGRILPLVTLHDPPWQ
IIGYNETGQYANYKGFMFKIINEMSKHLNFSYTVMFPTDSKEGWTNDTGVIKERDANSTS
TFIETEKIIDLIRKKKVFLAVGAFTVTERRKTLVNFTIPISIEAATLLTSRPGEISRALIFIAPF
TYDTWICIIIMIAIMTPIINYFHRHSPYYEYFYGTSIKGGLNSIFNVLWYVYGALMQQGGMH
LPEADSGRILVGAWWLVVLVIVTSYGGNLVAFLTFPRYKDSITNLEDLMANKGKVSWGIL
KDSSIEDHLKDSDLPRFKNFLQGCRAHEMEDPEVMAQVRSGSHVYVDWKINLLFLMKKE
FLSTKNCDFTLGEEDFLEDQVAMMMKLKSPYLGMVNEQLRRLHQSGLIYKWYVDHHPR
KDRCWGTSRIMEATTHTVNLDDMQGSFIVLGLGCVSAMIIIMLEFCYHFYKLSKEKRVVKP
YT
>BgerIr41a1
MIRFFLLVLAVLSRIFFVISRGKQNTIIHHQEELSELKPMILNIIERYFKNTRCICSITAGSHSV
LEDFPMWFPTFSINIRNISYLYFNIDSEGSETINKFESIIMKAISEECTPFVVQVKNINGVIRA
IDRASRRSITRYRKQYIYLPVNFEEPIEVRSIFSLTEMNNMPDTLLARFVNEPSSYIALSKPR
TDEQINDAKCFMTFPCLEENIANTNDTMNAVQHGHAIELLTHKFVGRNPFSEIVLDVWIN
DSFLNYNDLFPDKISNLKGKHLKCSSMPYIPYTVFTENEQIYSGSELQIILELSKKINFTWEI
VTDDHLWGTIWPNGTGNGVVGKVAGKQADFGIAGLYPWFSTYQWADFSHVHTTSSIVC
MAPKPHMRPRWISLYLPLSQNLWSAVITALALISFTNYYLHKASAMYLVLIKCCKSVTDTS
AFESFNTIINSFLTTLGMFFQQPPPNKVTVIGPLRLFILCVLIFCLFISSVYSGGLASILTIPRF
ESPIDTREELAESGLPWVNVHEAWVWSLLDSEDDVSKTLVRNFQIHSEEEMTKEATQGK
TVFSLEKFNSGLYSIPEHINEETIQTLRVMKEELYGGHVILIAQKSSPYIKHLNELVDHLYES
GILRYWESKVLREFLSQRLQIAVAQSVILNTQNGPTKLLTDHLQGAFIMLLLGISLASLSFLV
EMVTAKCAS
>BgerIr41a2
MVYNARYLFHCFLINLSLGNILIGELVNEIILKYFINYRCIAFITETDQDILYYVPSGIEIFHIRI
EDELGVIENLEENDTLDSAKVSEGTKIFERLLIQTLDAGCQGFIVQVRNFRSIVYSLSRATRR
CVTRYDRRYVFLPLNSEYDVNGIFSMKEMLSMPNLIIAKFSNNKDCLFTNRKQFESSIGIHL
KTQKEIHFKISNQNYTGQSETLNYNFYVSNNKFNSSNKFNIIDNRGSFRSSNEVCINIITHKF
AGNSNSNEEILLDVWGQFGFKNQFLYNRYIFPDKIKNLEGKQLRLIAFPWAQFVVSMQQE
DPPIEDGMEVLIFKEFSRCFNSTWKLTLDTKYLWGHISDNGSGTGLLGNVVTDQADFAFA
ALYVWHSTFQWLDFTRIYSKGAVTCLAPKPKMLPKWMTPIRPFTFSVWMTVFGAMLIVA
ITFYFFSRLSHFLIGAEGPQRFNNIVESTIYTFGLFLEQTPSGDSVTGTPFHNLFRRFIASVM
VFALLLNTAYDSGLSSVLTVPKFEDPIETPEQFAAKNIKWAANHESWTWSIKESDDPVIKA
IIKNFKVMDSEKITSDASKEKISFPIEMTSGGYYAVGLNLTTEILRNLRIMKEPLYWSPIVAL
SRKNSAYLEHLNTLISRLLSGGIIFYWEGETIRKYYSLTLQTAIARSADLVKDDGPIKLTVGH
IEGTFFILFIGLFISFVAFVFELILNLFKKPVSSTVTYYIKGPIR
>BgerIr41a3

MKLLWLCFVAAVWGNPPLPEDEESVVGDAVQHVVGKYFSQSFCVGVVYERRSTFAHLVR
GVPLVLHEASDDSAATLEEFLDQKCLDIMVQVADVRAMVQRLCRASRASKVRSTRRFLFL
PNAAAVQEANANGVFAMEEMAVLPDLVVAKLAEGNASLQFVTHRYAGDHPEEEVALGE
GELFPDKLRDLEGKELALAAVPDYPPYTILDLGASPPLYEGVELRVAKDFAKTLNFSFHVL
VDADAWWGEVWPNGTGTGMWGMVATDVAALGFGATYAWLENYPYLDYSLPYFRSSVR
CITPRPRQLPGWMTPLLPFDALMWTAVGASLGVTIAALYLATNAIMRLLDSDTKERLYST
LTSSVMWSWGLLLLQSPSVETQPLYVPLRHLVTWLMVYYLLVNSIYSGGLAAVLTVPRFE
HPINSVADLAGSNMIWAATLDAWVYSIEEATEPDLKHIVRNYRTLGEEELLRRAFKGDTA
FAVERLLGGNYALPSYINNETVPLLRAMQDDIYSGSPVFNVRKASPFLKSLNMLILRLNAA
GLLLNYEVQVVREFVGQRIQLALAQNDQVAKDSGPVALRVDHIQGPLFFLGFGLTAAFLVF
VSEVLKMVVIKKKALNAK
>BgerIr41a4
MANKKLITLEIKILCIFQALNNCLLLPSTGDNLIEESIGQMIREIVRKYYNASYCVGIVSENG
HPVAYILGEIPTVLIVNSKIPEMDYSEEYGNLRDETAIFDKIMVQMLDLSCSDFIIQVSDAQN
VVQYFSRSSRRSQNRFHRRYLYLPLIQENRFQNPDASIIFNMKEMIYMPDLITAHVLEKDN
VLKKCNRDNKRSLYQNNESYTEIQHLCLETIEIELKTHKYIGHDNAAEIKLDKWSPYIGFQS
GVSLYPNKIKNLQGKQLNITTIVEYPPYVVTDLTASPSLYEGIEFRIAKDYETFINCTFEVVIE
IEGEWGELWPNGTGTGMWGKLTMDFAVLGFGALYAWLEPYPYVDYTLPYTGSYVLCMT
PHPKLLPGWMTPFLPFTIEAWVAVAASQFITGISLYLTSKAQFLIHDSRNKIVKTFLKTTDC
IFWSIGLLLLQAPICFKNPPLRQLLTWLMLFYILVSTIYISGLASLLTTPRFENPINTVADLA
KSGLKWVANADAWVYTILEATEPDMVTIVNNFRVLKENELLQHAYDGDRAFALERLMD
GTYALPSYLNNETLPHLRVMREDIYSGTPLFNVRKGSPFLESFNTLLLRLHASGITHYYELK
VVQDFGEPKLQLMVDKDEDVSTPVKLQISHIEGPIFFLLFGLLGAFLIFIGEILKPPKKILKR
REEKQM
>BgerIr41a5
MLKAPVLFLFLILILKVAKSKLNLDEDIVTRSIGEFVHKIVSRDFNDSYCIGIITEDNPSLINYI
PPHIYKYHFHLGKNSPMPELEDSPTYDYRILSNATIMLETLVLKSWNYGCQLYIMQVSQPR
AVIRCMIRLSRRAMFRANRHHLYLPVIKNGEIFPVDESIFSLKEMDCLSDLTVARIVQNKTV
IYRNVTNYEGSQYFKTKAENVTNEENVLNRKECNVTLLEDKNYSYGDFVLEIRTHKYVGT
NRSENILLDVWVPGNGTCSGGFLYSGIVFPDKTRNFEGKTLIVTTWIYPPFVIMNFNRKPP
LLDGIEFRILYEYLKFVNATYRVTTDPLHFWGENYPNGTGNGVTGVVGMDKADIGFSAYY
SWPKGFQAVDFADTHMRSSVVLVTARPKLKPGYLVPLMPFDGVMWGAVAGSLVGCIYAL
YGLQKFCDIVLGRPEDRLSPYSNLDYSALRSIAMLLNQGPVFAWDSSIPRHVPARHFVGWF
QFMGEVISDTYNAGLASVLSSPRYEKPIETIHDLATKNVIWAANHISWIWSIEEDESPELQ
KITRNFRLLSEEEMIAAGKKNGEMGFVLEKMQGGHFTTESHINEDTVLQRRVMKGTLYW
SHLYFLLRKGSPLMRHFNILVGRVRESGLAYYWEADVIRNHLSNRLQLQIAKSRILYDDTG
PRRLNLSQLQGAFFLLALGEIVGLVAFLLEHSWSRAKKPPIKVVSRKK
>BgerIr41a6
MLKNLSLWLFLTFLGMSISQINVEKEVLTKSLGEFVFKFINRDFSGAYCIGVITEEDPAIVDY
IPKYLYRYHFQIGGQNSPIQEVQDSSTYDYRTLNNETILFETFLVQSLNSGCPLYVMQVSNP
RAVIRCFIRSSRRAMFRLSRFYIFLPVIKNGEIISVNEDIFFMKEMNCLPDLVVCRIVQNTTS
TTKNLLNPKGLEYFDTKSENITNNYDYLNRRECSAENLNPTESTNTSFGKFVIEFRTHKFV
GSNRSESVLLDVWIRGTDTCYGYFQQSGIIFPDKIRNVEGRQVILITWYYPPFIIMDFNAKP
NLFDGIEFRIIQEFMKFINATFRVTTDPGNYWGEGYENGTGDGVLGFVAMDKADIGFSAY
YSWPKGYRVLDFATPHMRSSVTMITPRPSLKPGWLVPLMPFDGVMWGAVAFSLLGCSYT
LYGLQKISDALLGRPEDRLSPYSTLDYSALRSYAMLLNQGPVFEWDSSIPRHVPARHFVG

WFQFMGEVISDTYNAGLASVLSAPRYEPPIETIHDLAVRDVKWGANHISWIWSIQEDEHP
DLQKVTSNFRLHTEDQMIRIGSRSGDMAFGLERLQGGHFTTEPHINEATVSHRRIMKGTL
YWSHLYFLLRKGSPYMRHFNTLVRRVREAGLPFYWEGDVIRHYMSERLQVQIVSSKVLFD
DTGPIRLNITQLQGPFIILGFGLMAAFLSFLAEHFFALSKKNAVKFSKKTVTKKNMSRHVT
KQTHRLSNNIHTRK
>BgerIr41a7
MNDTESKWNLYQYTPVDALGELTNLITKTHFPQYTCVGVITEGSTGIMDYVPHSNFRFHI
HIGDDSPISKLEETAELDYNKLDGNTLRFEELLIVSLDAGCPFYVVQVSNPKAAVHCFARAS
RRAQFRSSRKYLFLPMFKNGEIVDLKEDIFLMKEMDYFPDLVIAKLLSQEQDSQDSYDNLE
INSEINSMKFHYKIELYTHQFKGREKSKILLVDTWRNNENSLRSMNIFPDKVRNVEGREVI
IVTWYYAPFIVLNFNKRPPLYDGIEFRVAREFLIFVNASVRITMDPNNYWGDAFDNGTGN
GVLGLMAMDKGDVGFSAFYAWPSSFKVVDFSNTHMRSSVTMMAPRPKLKPGWLMPIMP
FSGAMWMAVGACYLGATFTLYKFQNFADKLLGRPGDAEVPFSKLDYTALWLFAMLMSQ
GPDYKLDSSIPRQVPTRHFIAWFLVIGEVICDTYSAGLAYVLSTPRFEPPIEWVEDLWTRH
VIWAGNHVAWIWSIEKDESPMLKDITANFRVMTEESMNTTGRKSGDIAFGIERLQGGHF
TTESHINEGTVAHRRIMKDTLYWSHLYFLLRKGSPYKQHFNTLVNRLREAGLLLYWEGD
VIRHYMSERLQVAISTSRILHDEDGANKLTLNQLQGAFFLLILGGILGTIAFAAELIVRPEKK
QQMKLNSNDEKSVNENKLESTRLN
>BgerIr41a8
MFEIIISNKMDFVLGALFVLLFPVYSTSTNEEVIGNVIRNISEIHFSHYQCLIIITEEFSLFPLF
LPAEIPRVGIQIHNTNFDNTLVKAMDLGCMGYIIHCCDCKSVFVTLAKSTRIALLRGGKKII
VYPECTEVQDIAQIMHMEELELFPDTVFLQVTKTVLGSSISTQKSILLVETSSTVKDSKGLG
TTKPENISDSCSLDSPNVEEESSNIESTINIGNNTLECLSEGVIIRGSSENTSVDYKYTIHNFE
LEYSDLPVESTFSAYSNDTVPVDSTSTYTEKIGRIISNKIQTNKNVNRIIQTFTDVSFGVLKFI
THKFVGENPAEELLLESWSSDQDEFPSVSQLFPDKTQNLLGKSLVVAAVDYTPYSVLGKDI
NSSDGVEVRLLAEFSKQINFTWEFLAENVNLWGTVHPNGSGHGVIGDVAEGKADIGISAII
STIYLIWNWTDFGTPHMSSATTGVVPAPTILPRWHTLILPFSTEIWVAMGVTFLVSAFGT
YCVSRLAHKFLGTMGPYTTLFNTALLVFGMLVMQVYEGAHEENRYVVLRALTALLDMMI
FTLSSIYTGGLASVLTIPRYGRPIDTLREMADSNILWAAQDEAFVFSIRGTGDPILDHLNEN
FRVLDEEELAVRALRGDMGFVMEVLSGGSCCFSSHLTPRAMAKLRIMREPLYWSHIIFIFR
KSSPYIEKFNNLVYKMRESGIMYYWEGEVARKFNSGCGPGPQYDAGPTKLQLGHVQGAFF
ALGFGLMASLLVFCWELLKAGGGKRIKNRRHKGPC
>BgerIr41a9
MENLGLAAMIVLGTTAVTAREEKLMGTMTREVILKFFTNIKINQCICIVTEESNDFLDFIFP
IDYPTFHVNISLNLMKNSNPLDEPFEGYDWDTLFLVPINSGCLAFIVQTSDIKFMVKTFAR
LFHTPKSIYRSNRKYLYLPAKNVDFENVIKDLFQMREIDFMPDLVLAKLKIKEEERKIKNNI
LKFIKDDSHFADIELITHRFVGPRPGSSETVLLDTWTSSEGFTKKADLYPDKMSNLMGKEL
ILCSLHYPPAAVINDIVDPPIYDGTEFRFMNEFSRTVNFSWKVLHKQDEWWGDVWDNGT
GTGPVGYVSMDQADFGFSLLYLFDHEHHFMDFSTYYYHSSLTTALPKPKLLPEWQVIICPF
NFDMWLAILISILISTAALLYTSKLSMRFLVIGKGDIIPSIYSTWTECGFRTMGLVVLQVPPD
ERDWSTPRYVPMRHLVTWLILFYFVVTTGYGGGLASVLTLPRFEPPIDTPAEMADRGSIW
AGTSDNYIAFLKESTNPKLKIMGKHFYVLKEEALADIAETGKIGIALERMLGGNFVLPYYIN
ERTMKNFRVMRDNYFSGHCAFYMRKGSPYMKHINIVVRNAREAGLFFYWEYLTVRLYM
NARDQLAVSSSRQIEDPGRTPLRVSHGSLYLLLYGLVLAGITFVLEIAKQRYLKKKEKKLFR
DIPVH
>BgerIr41a10

MIFKVIILINFVFGRGIDIKNNFNSILGVMSRDLVIKYFMESRCLGQVTEDSGKIMDFIFPLN
LPIYHVHISLDMLKNSERLGTEFEEYTWEEPLLQTINEGCLAFIIQTSELRSMIRAFPRLFHS
PKSVYRANRKYLFLPIGNELMNNKTFEETILDVFSMREMDFMPDVVIAKLLIENNKPNFT
PKTMDSADISPAEEEVYVELITQRFVGKTTKSDRVSLDIWDPKNGFRYGTNLYPDKISNLM
GKKLRVAGIAYEPLTVIDWEAEPSTYDGSDLQFMFQFAKKLNFTFEYVHDEYYYGEIWSN
GSGNGALGLTAMDIADYVFCATFIWEYEHRYLDFSTSYYETTIILVTPKPKLLPGWMVPVL
PFNYNMWMAVAVSVLFCTSLLYITSSASVRCLANESGKAVVNMYSSWIECAFRTTGLLVL
QVPPDERDWSTPRFVPMRHLVTWLILFYFVVTTAYCGGLATVLTVPRYEKPIETVSDLAK
HNVKWTGIYDAYLVSIKDSVNPDIQKVVKNWRFGDYDWLEAKTKTGDMSFVLEKMLGG
HFFIPPFIQEETMDYLRIMKEDLYSCACVYVMRKGSPFMKPLNTLLMRARDAGLYYYWES
FVVRTRLNSRRQLSVINSRVQYDVGPTKLQLHHIVGSFYMLAYGILLGSTVFILELIHFKFM
KCTIE
>BgerIr41a11
MRWMRFLKKQEMIINISILMIFVSVKGFDITDKINRILGVMSRDVVMKYFTESRCLGLVTE
DSDKIVEFILPLNLPIHHVHISLEMVKSSEQLDTEFEGYVWEERLLQPINDGCLAFIVQTSEL
RSMMRAFPRLFHSPKSVYRANRKYLFLPIGNELNTSDSFEETIVDVFSMREMDFMPDVVI
AKLLVDYNTSDFTARKLKSFGGFSPLLFFTDISPDDEEDVCVELITQRFVGETTKSDRVSLD
IWDPKIGFRYGTNLYPDKISNLMGKKLSVTGIAYEPLTVIDWEAEPSTYDGLDLQFMFQFA
KKLNFTFEYVHDEYYYGEIWPNGSGNGALGLTAMDIADYVFCGTCIWEYEHRFVDFSTSY
FQTTVVLVTPKPKLLPGWMVPVLPFNYNMWTAVAVSVLICTSLLYFTSSASVRFLARGTG
ANMVNMYSSWIECAFRTTGLLVLQVPPDERDWSTPRFVPMRHLVTWLILFYFVVTTAYS
GGLATVLTVPRYEKPIETVSDLARHNVKWTGIYDAYLVSIKDSVNPDIQKVVKNWRFGDY
DWLEAKTKTGDMSFALEKMLGGHFFIPPFIQEETMDFLRIMKEDLYSCTCVYVMRKGSPF
MKSLNTLLMRARDAGLYYYWESYVVRTHLNSRRQLSVINSRIQNDAGPTKLQLHHIVGAF
YLLAYGLMLGSTVFILENLYLKFITVIDTSKLFVPKSNAKNLIKNISSYNKQNIYNK
>BgerIr41a12
MLLYALAVMTIVIGKNGAQAMDLTEVLGTLAREIVLKYFMELPTENRCLCIITEDNDDLLS
YLLPLNITTYNVHISHDEYKFSKPLDTLYEEYIWEPPIMETVNAPCMGYIIQTSDIWWMSK
TMGRLWRSETAIQRYNRTFLYLPVVSRLMSDDEFEDHIRTVFEMKEMDFMPDLNVVKVT
KEMYVEPPILNTVKPNTVGSPLDNLTIEIVTHKFVSVYSSERISLDWWNRKKGFLMGTNL
YPYKMDNLMGKNITLVTINYPPLTVIDWDKDPRTYDGLEPQFIFEWARRLNFTYGWAHD
NEFWGHIYKNGSGVGIFGILSMDKADLAFNAFYLWEPEHHFLDYSTSHFKTSIILICPKPKL
LPGWMVPILPFSYTMWVAVIISVFGCTTALYIFSVASVRFLARGTGATMANMYSSWIECAF
RTTGLLVLQVPPDERDWSTPRFVPMRHLVTWLILFYFVVTTAYCGGLATVLTVPRYEQPI
KTPKDLADHNIKWTGLYDAYLISIRDTTNPVMARVVKNFYLEGEGEVVIPWLAELCKLQK
YCFVLEKMVGGTFFMPPFITEDTMNYYRIMDEELYSSLAIFGTRKGSPLMHHVNKILGKAR
DAGLFKYWEDLTVRNYLSTRRQLSVIQSRIQFDEGPTKLQVHHIHGAFYLLAFGLSSGTVIF
IFEPLVYKMSAIKQNILLKKKKNASEEILDNYNQRKTK
>BgerIr41a13
MYKFIQTISLVTMAVYCSPLAIQITEELARMSLQIVTNHFNNSKCVALITEDNADIVDFLGP
LHLPIVHSQIPSKILKDSKPLQTGFEQYEWDIFLVNLMDASCLAFIIQTSDISFMVKTFARLA
HSPLSLQRAVRKFLYLPTQEVPDDIFESNLRELYTRREMDFMPDVVVAKLITEFDVISEND
TSLYADDTDTELLKIELITHRFVGPKSSDRISLDIWSPDEGFLQHADLYPDKITNLMGKELS
FTTILYPPLSAVYVDVNPPIYDGLEFQIMHAMSRRNNFTFRMAYRPEEWWGAIWENGSG
TGMSGLVSMDMADIGFGAIYLWENEYRFTDYSTVYFRTSLTAVLPRPKLLPGWMVPIHPF
SKSMWCAVAVSVIVCTTILYLISQGSVRLLGGTGQTVVNMYSTWLECAFRTMGLLVLQVP

PDERDYSTTRYVPLRHLVTWLILLYFLVTTGYSAGLASVLTLPKYEPPIETPVEMVDRGVV
WGGMDIAYVFFLQKSLDPKMRRLAASFVEATEEYLTKKAATGEMGFVIERMLNGHFFLP
PYVTEKTMKKLRVMKEDYVYGHCVYMVRKGSPYKGIINQIVHKVRDTGLVLYWEDRTVR
RYMSTRRQLSVINSRKDIDSGPTQLLLRHVTGSFYLLCYGLTMSLVVFILELLRYYFFPQNK
NTFDVKKK
>BgerIr41a14
MLVLLIIAINGLLASENVPEETVEILGYMTRDISVKYFNTSKCLAFLTEDDNRFLDFVLPLDI
PTYMVQIPLSVLQNSQRITPISDFEEYAWESFLLPPLDAGCLAFVIQTSNITFMVKSFARLA
HSPKSTYRANRRFLYLPASETPGSLFESHIKDLYVKREMDFMPDIVVAKLIITDKEWEMD
QDNSSETPGLPGERTYETNITIELITHQYVGPISSQRLSLDTWTLHKGFNYNNDIYPDKLSN
LMGKRLVMTSFSYPPVSVVIPDEKSPIYDGLEFRIINEWSIYNNFTWSVHFRPEEGWSIIW
DNGSGLGLTGNVASDKVDVGFGAIYLWERDHLYVDYSSIYFTTFLTTVVPRPKLLPGWTVI
VSPFALDMWLAIGIAFILITFIVYWTAYSSMKLLGRGKIFTSMYSTLQECAFRNLGLLVLQV
PPDERDWSTPRFVPMRHLITWLILFYFVVTTAYSGGLATVLTLPRYEKPIETPHDMADHK
TVWGALDDVFVAFLKDSYDPKMREVAANNIVESEEYFEKLIPTGEIGFVIEMMQNGHFAM
APFINEQTMEKLRLMREMYVAGHCAYALRKGSPYMSIMNPILRKLRDAGIVLYWEDSTVR
KFMSTRDQLAVINSRRDPENSPTQMQLRHLTGSFLLLMTGEILSFFVFSFELSQQFWERLN
MRNDVQNSSLSALKQTSKKDTKEEKEGKVFPLLKWFNWNK
>BgerIr41a15N
VTEINNSNSDYIYDYFPNGIVPTIHISFSRKTENSKGYLEIFYESAEDEYIFEKLILASLNERCL
GYIVQVPDLRFMVDVFTEITRWASQRVNRYFLYLPTVSGASIPTSEEWNTDINDIFSLRAM
DFMPDLVVAKFLERNSEYVNEERNNASPGENDFAQYSYMSEDADCLQDAECSIEGDSVER
RRMKKFKSQHDNLDDVDIFEEISKNSQLIIQESSAESLSSEYHFELVTHKFIWPQPEEQIFL
DIWEANIGFVQGTNLYPDKITNLMGKPFIITTVPYPPFAVLDIEIEPPLNDGFEFRVVYEYV
KKHNMSYKGVYDLDNWWGKIWPNGSGMGLTGMVAIDEADIGFAAVYLWNDEYRFTDY
SHAYGWSGITVVAPKPVMLPGLMVPVLPFAPEMWMAVGVYLVVITFVLYGMSNASQKFL
GGNIANRYSTLVECIFRTLGLLVLQVPDDERDQSVARHVPMRHLVNWLILQFLVITTAYAG
GLAMVLTLPRFTPPIETKEDLASSGLHWSALDGAFVFGIKESPDPVLRTLTRNFYVGSEEE
LNRRTRTGDMAFVVERLQGGRFALPPHITEEAMAFLRLMKEDIFWGHCVFMVRKGTPH
MEKFNQVVNHLKEAGITLHWEVQVVREFLSERQQLSVILSRAPLDVGPTKLLLDHVQGAY
ILLFLGLGLGFITFFVERIYSRLSERAKKKTSFWKNIFSKETEKL
>BgerIr41a16
MRLKFVHVIFYAFHLCHVTKEDLVSWHIKNKVDLTAEEALGQMSGKIAAEYFTDFYCTAV
VTEINNSNSDYIYDYFPNGIVPTIHISFSRKTENSKGYLEIFYESAEDEYIFEKLILASLNERCL
GYIVQVPDLRFMVDVFTEITRWASQRVNRYFLYLPTVSGASIPTSEEWNTDINDIFSLRAM
DFMPDLVVAKFLERNSEYVNEERNNASPGENDFAQYSYMSEDADCLQDAECSIEGDSVER
RRMKKFKSQHDNLDDVDIFEEISKNSQLIIQESSAESLSSEYHFELVTHKFIWPQPEEQIFL
DIWEANIGFVQGTNLYPDKITNLMGKPFIITTVPYPPFVVLDIEIEPPLNDGFEFRVVYEYV
KKHNMSYKGVYDLDNWWGKIWPNGSGMGLTGLVAIDKADIGFAAVYLWDDEYCFTDYS
HAYGWSGITVVAPKPVLLPGLMVPVLPFAPEMWMAVGVYLMVTTFVLYGMSNASQKFL
GGNIANRYSTLVECTFRTLGLLVLQVPDDERDQSIARHVPMRHLVNWLILQFLVITTAYAG
GLAMVLTLPRFTPPIETKEDLASSGLHWSALYDAFVFGIKESPDPVLRILTRNFYVGSEEEL
NRRTRTGDMAFVVERLQGGRFALPSHITEEAMAFLRLMKEDIFWGHCVFMVRKGTPHM
EKFNQVVNHLKEAGITLHWEVQVVREFLSERRQLSVILSRAPLDVGPTKLLLDHVQGAYIL
LFLGLGLGFITFFVERIYFRLSERAKKKTSFWKNIFSKETEKL
>BgerIr75a

MNSKQYFQMFCSFIVFYISKFRDMNQVVFFGTILFIVGRLRATSAKMNSAESRMVMSYLNE
RRWSAAVLFICEDTAENLKWMKQLSSFDIQATIYSPMADNIVPTSLTTRYHLVAAVVDLS
CDGVVALLTKASEWKMFSSLHYWLLVSENLNSEDVSTLLNPLNIALNSHVTLAQLGKEPF
SLQDMYRIRTTEDLTFTSSLKWNPGQVLRSRPPRDDYRGVSLTATVLIYNDTWENFLDIA
QRDQNTEAKFHYVLMAHVAEMLNFRMNRSITDIWGYPLNGSKCFNGIIGTIQEDCGESDI
SATGLMWKTERLEVVDYFTDTFKYKGAFIFLKPSLSEVSIIYELPFSISVWVTFSVTMAALT
IFLVYVQRTENHIHSKRKNEVDESLNWSDAVLDTIGIVCQQGTGNTPKYLAARMIFIFLLLL
SLFLVTSYSAIIVSLLQTTSSAINTLSDLMNSPFKLSLCEMLINEVTDPTVEKVFKEKLYTQP
YHEAFMSEEEGMEKIRKGLYAFHALFAAHKIISDTFEEHEKCRVKQIPMFLANHIGFPIRK
GSPYAEHMRRSIQWMRETGILGREINRWYYQEPKCISGGQNFVSVGIQEFYPTLVILSYGIL
LSIGVFVLEAVHHRYRHAL
>BgerIr75b
MRRLLQYCFIVQIFAYASIFDEEAITQSIISLLNDNFIKFATFITCASDAADVAKSFAEKGMR
LKVQGPDKASDVESLLDPKDHLLAVIVDYRCGRHSTQFLAQVSEHKLFTSLHRFLLLLPDQ
TKKNQLYEELSKLDLPIYSQVTVASYKQGDVVVELQDVYRVRKEVPLTITPVQKWAPGER
FPRGPKRDNFGGIVFNSSTVLLEGEAWETFLAESNKHINTLTKFHFVLVQEIARMLNFRLY
TWPETSWGYPNGTNYDGVIGTLQRGEAEIASVALILKKVRMDYLDYVGETVRYEGAFYFL
KPSLSDVSNIYLLPFSRSVWITYTLAGFIFAVVFYWTQRIEDAMHQHELHKTITFTDSALIA
ISVVCQEGSSRDPRNLSARILFLSLLLLSLFLTVSYSAIIVSLLQTTSDSIKSIRDLMDSPFKLG
MKDVVSNHRYVNETKDQTIKELYFKHIFTQPWHEAFTSTKVGVDRMRSQLYAFHGDTDA
LREIGRTFEEHEKCRLNRVLMFTSTRLSFCARKGSPYKELLRRKAQWLKEAGIIDREYKM
WLTQKPACQGGVEGFASVRIKDFYPALLVWIYGLIFSTALLVLEYGRNKLTHSRENVPHGF
SIEK
>BgerIr75c
MVLLVLLALLCSNTFAMDLERTTLLVQDFMRCNELSRASALVCWNKRDEVNMMKRFSE
KELPLHIFNDVHDENLLHLLNVKYTHLAVLLDMKCEGAIVLLEKAFEHKMLKRLHFWLL
LNSNGSDILLRLPLDSQVTTAEFGHEVVLGDVYSFADRPNIVTEPRIWSPGKKFPKTPGRD
DLGDIVLKAGVTVIQDPWEHFMDLRYRHLNTMSKLSFVLTGYIGQMLNFRMEPVLTDAW
GYPINNTEMYAGIAGQLQRGEVEIGATTLLIKNARLNIIDYAAEAFPFVGNFMFLRPSLSEV
SNIYTLPFTRAVWITILLLVTVLTVMLVLSLKAHIRFSPHGAEDSIWNSWSESVITTFAIVC
QQGTQEAPNSASSRLIFLFLLLLSLFLVASYSAIIVSLLQTSSTAIKTVEDLMNSNFKLSMRNI
TYSITYVNDTTDPTVRRLYFEILYAQPFSKSFTSNEIGIENVRKGLHAFQSDADAYKEMSDT
MEEHEKCRYEEIVLFTANVLAYPVRKGSQYKELIARKARWLRETGFVDREYTRWYHPKP
KCQDNSQGFVSVRLQDFYPALLVFLWGALLAVLVFFLEIGYNIGRERCCCNKNEMWVD
>BgerIr75d
MRRLATAASLLLLLLLPLWCHCTAASSMRYFPAIRAFLTHNRVRCAVLYTCPQDDSAALS
KKLSDDGFAATSAHKNINDYNVSKLLNVEWHHLSIIVDSGCDGSTTFLTEASRGNKFGIQH
HWLILPRDEGPVLSALTALNVPVDGHVTVAVPGSLPGRVILKDVYHLGEQPFLTEKPSGS
WAPGDRWPSEPPRTNYGAMHIKTAVIVVQDPWEHFFDMRYKHLNTLSKNNYALMAYV
AEMLNFRMNLTMTDSWGYPVNGSKDYAGIVGLMQRGEVEIGAAGLLVKETRMDYVDYA
GEIVTFRGAFMFLKPSLSEVSIIYTLPFSNSVWATFVATVIILTVGLEFSQRLFKKVNPSESN
HRSEWSEAVVNSIGIICEQGAETAPEDVSSRIIFLALLLMSVFVLTSYSASIVSLLQTSSDAIN
TLSELMDSGLHLSMRDIGFNTNYANDTTDPVIRRLYRDYLFAQPYHKAFTTQEEGVEKIR
TGLNAFHGDAGAYKVMSDTFEEYDKCRLKEIKMYSTDKLAFPVKKGSQFREHITQKARW
LREVGLVGREYKRWFSQRPKCDSNSQGFVSVKLLDFYPVLMVLVFGMAAAVAIFFGEVLK
SALKQGACRLERNPLQEITKNRR

>BgerIr75eNF
MMKFAAGQNVMVAIEDPIQAVADTWRLLQSPYSHIAVIVDLECEASIRFIADTSNESAALA
NLELLVDSQVSILHYKPGERFAMLQDVYRLRKDRPLKFTELQEWNPGQKHIPKQRRDNF
DGLALKAGIVMRNKSEDFLDKRYRHKDSVGKSNYVIMTLVAEMLNFTMNLTFMTTWGF
PKKGTDCYDGVVGGLQCGDLEIGALGILYKIPRLERVDYAGNTFSFEMSFKFLKPSLSEVSV
IYALPFSLSVWLTYLLFVALMGLALFVTETVESRMNPDADDAPMIHDATLNAFAIMCGEG
TPRAPRNVSSRIVFFFFLLLALFLETSYSAIIVSLLQTPSEAINSLTELIDGPFKLAMKAISYNI
DFVNDTTDPEIRRLYFKKLYARPFHEAFVSEEKGVESIRRGQNAFYTDSTGYKVMSDTFEE
HEKCRLKEIVINPSNVLAFPIKKKSPYRELIIRKVRVLKEFGMIDREYRVWFEKRPKCGGNS
QGFVSVRIKDFYPTLVVLSIGILCSIAVFFVELFYHKIKEGVYWPAVEEIGLA
>BgerIr75fN
AFEKKYLKALQHWLVLAANEGVFDEFAALKLLLDSHVTVAQSDTDQKTIILREVYRIGQQ
PLRKTPPQSWKPGLKWPPRRDRRNYGGIPVNTAVVVGVIGDTWENFLDMRYKHLNTQS
KCSFTLMTDISIILNFTLNLTQTDTWGYPKKNSPCYDGIVGLLECGDIELSCTGLLQKDARL
NRIDYAGETFDFGGKFMFLKPSLSEISIIYTLPFKKTVWIAYISAVSILTVVFYASQRFEWRV
NPSHEPVLFGDVALNSLALICQEGTPRAPDCYSSRICFLNLLMLSLFLTASYSAIIVSLLQTSS
NAINSLKELIDSSFTLSMRDIAWNTKFVNDSRDPLVKKLFYEKLYKMQYRDAFVSQETGV
EKMRSGLFAFYGDAEAYKIMSDTYEEDEKCQLKEITINPSNSLALPVRKASQFKEHVTQKI
LWLREAGMIKREYKRWFIQRPKCDSDSRGFVSVRIKDFYPVLVILSFGIAGSLMFFALELLH
HKTVHGTFYPKAIIL
>BgerIr75gN
RLKTITDQSHYGLQVNEQSELQTRLSTILRTKYYPLVVVADMEDENINHILEQMQVKLYSL
CFIFQASNDKLFSPLHHWLMFSNNIQESITILEESYLPINSHVTLAFENSPTKNIILQDVYHV
GRNQSLIISQPLNWTIGQRIPQENRRSDYRGISIPTVIVLAQGTWKDFSDERYRHLNTESKV
QYTLMTSIAEMLNFRITPCFTNTWGYPINGSHCFNGAVGLLQCGNCEIGATGLLFKAERM
PVIDYAGETLNFRGAFMFLRPSLSEVSVIYELPFSTSVWVTYIGTVTVLTIVLQITRHIQQHI
SSSKEEPISWSDTIMDSMAIVCQQDALHSPKNASSRIVFIFMLILTVFVLTSYSAIIVSLLQTS
SDAINTLTDLMNSPLKLSMIDIFVNETSDSEVTRVFKEKLYTQPFKEAFTTEDVGIEKIRKG
LYAYHGLSGSYKIISDTYEDHEKCRFKEITMFTSLHISFTARKHSPYIQHIKERTCWLREVG
LVDREDKRWFHQKPKCESAGQNFVSVGIQDFYPALVVLTYGILGSIGLLLVELLYFKKWKI
NCYSPIDNKSD
>BgerIr75h
MKMTWIKLLLSIILILFSKGNARDMIKDSISIIKQFYEHKKVSLVTAVVCWGKYELSLMKAL
ALSGIRVNFAKEPDTLAKVLDHDTHYRSGAVVDMACKHSHAALKHASFKKQFDIHKFWL
LLSPPEENAIPYGNTSYDVVYSVDRNMFPDGVLKDEEIFNVSISLTEFEIGALSGLRILENSE
VILMQNPQIINESDQIKILYPHQFTYWEDMTLRHVDKWTKIHWPVFGYLSEQMNFTFDV
NYQEDNYGWLINGSFNGMMGYMQREEVEFPSTGIFIRKDRFAVTDYCADTFKLRATVMF
RQPSLSTVSNIFMLPFDTAVWMCCLAYCLIIMIILGLQITFTIRQKIEQDMINSGWSDILTFV
LGAICQQGFHITPSTLAGRTTVFILTLSSLFLFTSYSANIVALLQTPSTSFKTVKDLTESSMV
LGVENQTYNRVYMQETTDAELKEFYFKKIAPMGEKVYCDPVIGMANIKKRLHAFQVDTT
AAYKIMSETYEEYEKCGLQEIDLFPSPVFTIAVTRGSPLRDYFSVRVRWYREIGLLGRLFKI
WMPQKAKCESSAGGFVSVGMTEFYPALMVLQYGTLVAVIIFVAEKLYFYRTHMASKVLQI
CPRQKIKTDRKPPKADNGVKNTKQLLNKQNNWLT
>BgerIr75iP
MKMTWIKLLLSIILILFSKGNARDMIQDSISIIKQFYELKKVSLVTAVVCWGKYDLSLMKAL
ALSGIRVNSAKEPDTLAKVLDHDTHYRSGAVVDMACKHSHAALKHASFKKQFDIHKFWL

LLSPPEENAIPYGNTSYDVVYSVDRNTFPDGVLKDEEIFNVSISLTEFEIGALSGLRILENSE
VILMQNPQIINESDQIKVTYVDKWTKMHWPVFEYLSEQMNFTFDITYQEDSYGYLNNGSF
NGMMGYVQREEVEFPSTGIFIRKDRFAITDFCADTFPLRATVMFRQPSLSTVSNIFTLPFD
TAVWMCCLAYCLIIMIILGLQITFTNRQKIEQDMINSGWSDILTFVLGAICQQGFHITPSTLA
GRTTVFILTLSSIFLFTSYSANIVALMQTTSTSFKTVKDLTESSMVLGIENQTYNRVYLQETT
DAELKEFYLKKIAPMGEKAYCDPVKGMANIKKRLHAFQVDTTAAYQIMSETYGKYEKCGL
HEIDLFPAPVFTIAVTRGSPLRDYFSLRVRWYREIGLLGRLFKVWMPQKAKCESSAGEFVS
VGMTEFYPALMVLQYGTLVALIIFVAEKLYFYRTHMASKVLQISPRQKIKTVSKHPKADKG
VKNTKQLQIKQNNWLT
>BgerIr75j
MLKTRFILFSMLISWLVPNNESIELNTEDKFIISLLTFHSKHSLPAQNSMFLCHNTDSTYQL
FRELSKNYIELSILTKTQSILNQLRETKIESRCIFILDMSCPNSSAILQQAQVLALFNKKNKW
ILLNNYYPNVYDSENITDTSMTSVFKNSNSKSAKMRVTANQQLINELTRINLLIDSDVSVA
TRISRNRFILEEVYKRREESNLVTNEIGYWVEEFRLVIYNAKSLSSRRMDFQKSLIKVVMVV
INKDTMKHLTDAVDRHIDSLSKANYALFHHIKEIMNASIEILTVDSWGYEINGTWTGECG
YLQRRETDIAITPMFITKQRLTIVEFMSAPTPTRVEFIFRQPPLSFVQNIYTLPFSGLVWLCS
FLLFSLMGILFYCALKWEFCTKENESSADNMPITWSDIVMSTVGAICEQGTLMEGQRIPSR
IILTFLFVTVIFLNTSYSAYIVALMQSTTNSIRTLNDLLNSGLTLAAEDIVYNRYFFSVAKDP
VRRAIYTKKINPPGGPQRFYPIAEGIERVRKGFFAFNLELGIAYKVISDTFNEEEKCNLQTM
NFLIEVRDPYLAISKNSPYREIFLIAYRMVHERGLQQRQNLRFYNKKPKCNSPGTAFMSTGI
VDFYPVLVTLGIGLVSSVCIFILEIILHRKLCTRKHL
>BgerIr75k
MLKSNVLLFSIILSWLVVSNESTELNTVDKFIVSVLRFHSKNYLPAQNSAFLCRDAYSTHQL
FRELSKHFIKLSILTQAQSIVDQLRATKTESRCTFILDMSCPNSNAILQQAQFFTLFNKRNK
WILLNNYHSDVHDSNNISDTSMASVIENSNSNSSKMGVTANQQLINGLTRINLLIDSDVSV
ATRISRSRFILEEVYKRREESNLVTNEIGFWVEDFRLVIYNAKSLSSRRMDFQKSLIKVVMV
ITHKETMKHLTDAVDRHIDSLTKANYALFHHVKDIMNASIEILPIDSWGYEINGTWTGEC
GYLQRRETDIAISPMFITKPRLGIVDFIAAPTPTRVEFIFRQPPLSFVQNIYTLPFSGLVWLC
SFLLFTLMGILFYCALKWEFCTKENESSSDKMQITWSDIVMSTVGAVCEQGTLMEGQRIPS
RIILTFLFMTVIFLNTSYSAYIVALMQSTTKSIRTLNDLLNSGLSLAAEDIVYNRYFFSVAKD
PVRRAIYTKKINPPGGPPQFYPIAEGIERVRKGFFAFNLELGTAYKVISDTFNEEEKCNLQT
MKFLVDVSVPYLAVSKNSPYKEMFLIAYRMVHERGQQQGQILRFYNKKPKCNSQGTTFMS
TGIVDFYPVLVTLGIGLVSSLCIFILEIIVHRKPCTRKSL
>BgerIr75lP
MSFLTIKSSCELTNMLKSNVMLFYIILSWLVVYNESTELNTVDKFIVSVLRFHSKKYLPAQN
SAFLCRDADSTHRLFRELSKHFIKLSILTQTQSIMDQLRATKTESRCTFILDMSCPNTITILQ
QAQXFTLFNKRNKWILLNNYHSDVHDSNNISDTSMALVIENSNSNSAKMGVTANQQLIN
ELTRINLLIDSDVSVATRISRNRFILEEVYKRREESNLVTNEIGFWVEDFRLVIYNAKPLSSR
RMDFQKSLIKVVMVITHKETMKHLTDAVDRHIDSITKANYALFHHVKDIMNASIEILPIDS
WGYEINGTWTGECGYLQRRETDIAISPMFITKPRLGIVDFIAAPTPTRVEFIFRQPPLSFVR
NIYTLPFSGLVWLCSFLLFALMGILFYCALKWEFCTKENESSVDKMQITWSDIVMSTVGAV
CEQGTLMEGQGIPSRIILTFLFVTVIFLNTSYSAYIVALMQSTTKSIRTLNDLLNSGLSLAAE
DILYNRYFFSVAKDPVRRAIYTKKINPPGGPPQFYPIAEGIERVRKGFFAFNLELGTAYKVIS
DTFNEEEKCNLQTMKFLVDVSVPYLAVSKNSPYREMFLIAYRMVHERGQQQGQILRFYNK
KPKCNSQGTTFMSTGIVDFYPVLVTLGIGLLLSVCIFILEIIVHRKPCTRKSL
>BgerIr75m

MSTVLNFRVLKMSQLLHFVHFLVYLVVSTHIVKTNKIITTEFIVDVLKFQAKVSPPARVNT
FLCWDFGDLRYLMNYMSQRQVQLAVHTSLDGFLSQLHRADESKHEIFILDIDCEDVELILS
EAHNSFKFRPWLRWLLLDTSTYLQNNKSLELIDTKAMEEPDAVDYLLMDTDVLLNSEVT
VGKQISENMFTCLEIYKRGKMEPLIKNILGEWNVNEGMKTMAPLVTSRRRTNIHKSLLKA
VMVVTVNDTLNHLDNTINKHIDTITKVNDALFHHIVDILNASISMVIVDTWGYPDENNN
WSGLAGYLQRSDADIGTTGMFVTKQRLPFVRYIASTSETKNSFIFRQPPLSFVENIFTLPFN
RSTWWASAGLITLTGFILYIAFKWEFKKKKITDGASNERERVQGSDVLLMSLGAICQQGTS
QEAESIPGRILMFFLFTSVIFLYTAYSACIVALLQSSTNRIRTLKDLLNSGLTLGVEDVVYNH
HYFPAATDPVRHAIYTEKVNPPGGPNRFMSLEEGLKRVRKGLFAFHVELGPGYEVISDTFF
EEEKCGLETINFLIEIVEPWVGVSKTTPFTEILSVAYRMLKERGLQHRENVRFYQKKPECVS
QGSLFVSVGLRDCYSAMVVLLYGILFAISTLILEILYFKWNHHKNKSNENYLSP
>BgerIr75n
MLRKSIFSTIILYWLMINGESVAMKTTQKFIVDMLTDLKSNYLPVHISIFLCWEVGSLHELV
HELSMNYVTHSVHVSNWDIMEQLHASNNKTRSTFVLDLACPNWIEFLYEAQTLNLFNRQ
KKWILLNSCNTKFNYSDKSTNNNGIPPIKIDSTASAITIDACQKQTNELIHLNIFIDSDVSVA
KRISENKFVLVEVYNLKNQKGFVQKEIGYWREDLGAVIKKSWSQSISRMNLQRSVIKAVIV
VTNNDTKEHLTHTTNRHIDTISKVNYALFQHIGDILNAXVEIIVVKSWGYPENGSWSGMS
GYLYRGEADIGISSMFITKEHMEFVEFITSTTPTSAQFIFRQPQLSFMENVFTLPFSRTVWL
FSTLLIAINGVLFYFALKWEIHEDSGSNDKPIPRIITWSDMILSAVGAVSQQGTEVEGKKISG
RIILLMLFVVVIFLYTSYSASIVALLQSTTNSINTLSDLLHSGLTLGVHDTDFNRHFFSMVKD
PVRRAIYTRKVKSQGQSEHFYPMDYGLERMRTDFFAFHVELGPAYKVISETFQESEKCNL
QTINYLIEVKDPWLAVSKKSPYKEALTRAYRMVHERGLQSRENLRFYHRKPECSSQGSNF
VSVGIKDCYVVLLVLLYGILFAVGIMLLEIIAIKKPCTRRIN
>BgerIr75o
MMICVKSETVPKDNIMDTISFIGDFLKVQRRAAPPAIVTAFLCWESGNIHKLTRFLSQDFV
FISVHSTTEKDVLAQYQRLQRDVYKTFILDLECQDATDILAEANKRDMLRHQHRWLLINR
SFNNNKYESVPSDNHEIDLISANMEFEIENDVLYSIEFVTFFQTLNILLDSDITIAIKMTDQL
FHLYEIYRRGPSEKVIINKIGHQEQTLEILMIASSLPAKRRLNLHQTTLRVGTVVTDKDTLE
HLDDGVNKHIDSMSKSNYLKYKHAAMVINVNTQNVFTNTWENFVNGSWTGLSVYLERN
EIDLGTSPIFLLEGRLKTLNYIHAPHGGSSFFLFRKPQLSLMRNIFILPFSSSVWLASGAILILI
GVLLYGIFKWEAQNNDTTRNNKEGLDIMITGSEEQKARTASWSDIILISFGQICQQGSEME
ARQPTGRIISLLLYIAVMFLYTAYSACIIMLFQSTSDSIQTLKDLYESGMELAVCDNEYNRHF
FPMVNDPIRKAIYNDRIKSSENFMPLEDGIDKVKNAFFAFHASEIAFKTVSETYREEERCGL
KSIPFFDFFPAYVGASKESPFKEVLSFAYRRMQETGFQIRIYTQFYTKKPKCDGSSSIYVSVG
ITDCLAVILVLVYGIPCAIVVLLLEIIVHKILKKRGMPQMDMDLNNHKSTAIRKF
>BgerIr75pI
MVCVKSESVSKDRMDTITFIGNFLKLHRRVAPPSIVTAFLCWETGDVQKLTRFLSQDFVFI
SVHSATEKDVLAQYQRLQRDVYKTFILDLQCQDSTDILAEANERNMLRQQYRWLLISQSF
YNNYRSLPSDTSNYENDVASSNTKFEIENDKLYATDYITFFEEMNVLLDSDITLAIKLADKL
FHLYEVYRRGASEKVIINKVGHQEETSEILMTDSLLPAKRRLNLNQTTLRVGTVFTSKETIQ
HLEDGVDKSIDSLSKSSYLRFKHAAIVINVKPQNVFVDSWGSIKNGTWTGLTGCLQRNEID
LGTTPIFLLKERMKILNYIHAPHAGSEMEARQPTGRIISYLLYITVMFLYTAYSACIIALLQST
SDSIQTLKDLYDSGMELGVWDNQYNRHYFPMVNDPIRKAIYNYRIKPSQRFLPLEDGIAR
VKNGFFAFHASETGYKIISETYLEEEKCGLEILPFFDFFYPHVGVSKQTSYKELLSIAYRRMQ
ETGFQIRLHTQYYSKRPKCDGSGSFYVSIRITDCLGVIQVLAYGTTCAVGLLILEIIVHKMLK
QRGMLETTDFNTHKSCFSNS

>BgerIr75qN
LFILGTRCESNHIDNTNSFIRDFLKSQTKAAPPTPVSAFLCWKLGDILKIIKFLSQNQILLSV
HSTTENVLSQYEPKNQLADAYNTFVLDLDCEAAHQILMQGNITKMLRRQNKWLLLRSPF
YNITQELSSRFGKLVVLLDSDVTVAFNISKRQYDMYEVYWRGHSEIMVVNNIGHWEESSGI
CMNTSRVVAMRRINFHQATVKAVTVVTVNDSINHLEDGVNKKVDTVSKCCYMMFKIVLD
MLNARADMVFTNTWGINKNGSWTDLSGYLQRNETDIATTGMFVLKERMKVVDHIAPCL
GGPFFIFRKPPLSLVENIFTLPFSRMVWISCAVLMSILATLLYGVYKWEVFKSNQRTFNKD
NAFLRSDQPSVSDVVLLTLGTICQQGSALEAQQPISRMVTLWLYITVIFLYTAYTAFIITLLQ
SSTNSIQTLKDLYESGMELAVWDTPYNRHFFTTADEPLKKAIYENRIKGRGRPDKFLTIEE
GTEKVRKGLFAFHSSFLSFHQISETYQEEEKCDLELVKYFENFCLIHVATSKETPFKEIFKIG
YRRLAETGLQLRTQRSFYLKKPQCEGRGSFYMSVGITDCYPILLAFVYGLVCSILALITEIVV
NNRLRRREIERLLKQLYTE
>BgerIr75rC
MFVQYFHFNLIFSMLMIWVTAGHISENDIVISFIGDILNFQKKVAPPAIVTAFLCWKSEQLR
AMDRFLSTNYVFLSIHSMAEKHILHKYNFHKRDGYKTFILDMGCKDAKKIVFQANEMNM
FRGQHRWLLLNKYFDYYNVTLTNVFSNNSENVHSTSSTKKDYEISNISYLSKMFNSTNILI
DSDVTIASRESDTKICLYEIYRRGPLEKTVINKIGIWENQIGIQLTVPTAVATRRFNLQQTTL
NAITVITDDSTIDHLEDGVDRDTDAFSKTTYVQTKHVTDMINATSWGAFRNGSWDGLTG
YLERQEADIGTTPMFVMKERLAVAYYISSPVPGLPFFLFRKPPLSVVKNIFTLPFSQSIWIAS
GVLLFGICILLYGIFKWEAWSTETSENIENGNNLNYNAETLLKMATLSDVILISVATICEQG
TEIEAEKPTGRIVTLLHYISIMFLYTAYTACIVALLQSTTDSIRTLKDLYDSGMEIGVWDTPY
>BgerIr75s
MLLINVKAQQVSEEYDNIITFIGDFLKLQRKVAPPATVTAFLCWKPEQRQILNRYLSENYV
LLSMHSIKENYILPQYHFQKRDGFETFILDLDCENANNIIHQANATNMFRGQYRWLFLKK
SIDYNPTLSNSHLDNSKDVPTTSLKTDCEINNIINNLSRRFNNTSILVDSDVTIALRVTNKIC
LYELYYLGPKMVINKIGFWEDNSGIQLSVPIVVASRRFNLQQTTLNGVTVVTYEDTINHLD
DGVDKHIDTFTKSYYLQCKHIFDLINASMNYTVERSWGILKNGTWDGLTGVLARHEADIG
LTPLFILKERTHIVTFIASSKLQLPFFVFRKAPLSVVQNIFILPFSRSVWMASGLLVFVIMTIL
YGIYKWEAWTTIKSEKVSQNVNHNGNNLIVRTATLSDIILSSIATICEQGTETEPEKSTGRI
VILLQYISIMFLYTAYTASIVALLQATTDSIQTLKDLYDCGMEIGAWDTPYNRHYFPNAIDP
IKKAIYQDRMNSKDGRKNFLSLEEGMDKVRKGLFAFHTTTHSYNIMAATFEEEEKCGVQE
IVYFDDQFMTHTAISKQTPYKEILSLAFRRMDESGIQTKLINDYYRKKPKCDGSGAYYLSVG
LLDCYPVMIIFVIGTSCSILVFVLEFFVHRV
>BgerIr75tP
MLLINVKAQQVSEEYDNIIKFIGDFLKLQRKVAPPATATAFLCWKPEQRHVLNRYLSENYV
LFSMHFIKENYILPQYHFRKRDGFETFILNLDCENANNIIHQANTTNMFRGQYRWLFLKQ
SIDYNPTLSNSQSDNSNDVPATSIQTDCKNTTIINNISRRFNNTSILVDSDVTIALRATNKIC
LYELYYLGPKMVINKIGFWEDNSGIQLSVPIVVASRRFNLQQTTLNGVVVVTYEDTINHLD
DGVDKHIDTFTKSCYLQCKHIFNLINASMNYTVERSWGIFKNGTWDGLTGVLARHEADIG
LTPLFILKERTHIVTYIASSKSKLPFFVFRKAPLSVVQNIFILPFSRSVWMASGLLVFVITTIL
YGIYKWEAWTTIKSEKVSKNVNDNGNNLIVRTATLSDIILSSIATICEQGTETEPEKSTGRIV
ILLQYISIMFLYTXYTASIVALLQSTTDSIQTLKDLYDCGMEIGAWNTPYNLHYFPNAIDPIK
KAIYQDRMNSKDGRKNFLSLEEGMDKVRKGLFAFHTTIHSYNIMAATFEEEEKCGVQEIV
YFDDQFMAHTAISKQTPYKEILSLAFRRMDESGIQTKLMYDYYRKKPKCDGSGAYYLSVGL
LDCYPVMIIFVIGISCSILVFVLEFFVHKV
>BgerIr75ZF

MVLLLMILLWLIEARFNEYTTSLVVDYFSYKQTRIVTIFACSIQDSLALSKALLSAGKFWIT
TIAPEEGGSLTHRNKMESDTLAVYYTAHGVFIDYDCVENKDTFRKMSDRFLLNASFNWLI
WSKDPAPSDLETCNLNIDSEVTWATPEMSTIKLFDLYKVNYTRPLISTPSGIWEKTNGLD
YHLNEFKYLRRSDLQGLKFNAATAVTSIPEGSIEAYLTSTTHRYTDSIAKYNYALIRQLATT
FNFTLQLYVTNSDGYPDTNGQFDGMMGMLQRGEAHLGINSLIMGPHRLGAVDFTGPTW
KFRVAAIFRHPLTVGKSDSLVRPFSEKAWAAIILSWVIMAIALQFVKWLEAKRQHLSKQN
MDYAWSASLLAVSGAATEQGTELESKMVSWRIAYLATLVQTTLVNAYYGACLVSDLLQPP
PKNILTMRDLIKSKLQVAYVDNRFNRELFQEISDPIVLELYQKKMKSDSSAALPFDIAIDKL
HKQEYAIHDEAIALYPIIEDTFNNDDKCAIAEIAFFQPMMTYNAIQKGSPYRKLLTYGFRK
MMEDGIMARELSTWCSDPPKCQPLDKFYPVKTETVTAALALFAGGVFAAFVFICLERFQN
FRYGSNILSLNEI
>BgerIr75v
MSDNRSRRLDFSRRTEALMGQHKMWLILIFLCLFSQEYVFGSLNETTIQFIEDYFKYKDVR
FVATFTCSKYETYQLTKALASKNFRVVSPDPEKPASAILKSVMKRSYHKYGIFLDYDCECSA
GLFLNGSFNWLVWSEQSFPSSIETLNLNIDSEFMWANPEESGKVILYDLYKITYTNPVNAT
FAGDWDPEGGLNYILTQYIIRRRQNMQKVIFKVGLVVNNIPLENIEEELVKPENRPLDSMA
TYNWGFFLFLKQMYNFTPEIHITNSFGLVVEEEGNVDGLALMLKNKEIEFGINSYLMNRP
RTLLTDYSSTATWDFRICAIFRHPTVTEKLGLLLKPFEPSLWLGCGLLWLLMMAALRFISF
FESQYVDVLESGANTGSEQQGAWTWSDTFVIIMGALSQQGSTMDSEWLTGRIVFLNTHIL
ALMMNTFYSAFIVSSLLSAPPKTIKTVRNLIDSPLQFGAEDINYERGYFELSNDPLVQELFQ
KKMAPPKKGYFSRELGLHKVLTGLFAFHTEAINVYPTIEATFTDQAKCDLTEILIFPIERGY
MPVPWGSPHKERITYSFRKFIEGGLINYQNLQWRAAKPPCLIADEIPAVAVNTVSFALWL
QLFGVIVAIVIFIFEAILGSR
>BgerIr75wF
MNCLKISVIILTILSQTSGQITEEIANFVHDYFTYKYARTVSCITCSKMDSLYLTKYLMSVGS
YWMVSPDPDESPTELVPQIMKRDYRRFGVFVDYDCAVAKKYLAENSDRKLLNATFYWLI
WSKNKFTSEIEYLNLHMDTEMTWAYPDETLNQTLLYDLYKITYKWPINATLAGHWTSEE
GVVWNLTEYKYSRRRDMRGVTLRTGIVVNNIPIENLEDELVKPENRILDSMATYNNKIFL
MMMDFYNFTREMIITHQFGYVVEDGNYEGMVKLVHEKEVDVSISAMLLNKARMENVDY
CPVETWKLRLVLPICALFRHPPITGAYSVLLKPFEPSLWLGCGLMWFLMMVALRFISFFEN
QYHVEDNEDAPEEEAWTWSDTLVIIMGAVSQQGSTMDSKWVTGRIVFLNTHILALMMN
VYYSAFVVSSLLSAPTQSIHNTRDLIDSSLDFGAEDINYNRPYFETNTDPLVHELYTKKMAP
PHKGYFSRELGLHKVATERFAFHTEVINIYHIIEETFPEEAKCELTEILIFPIEPCYPFIPKSSP
LKELFTYSYRRAVESGLVDHMNQHWRPVKPPCSSKTEYRRVGIDTILFGIWLLGAGLALA
NIFFTMELIVKRRFCLKKKKQISTLDFPPPQQKKVSALPWIEDVKPRVVTSSLPLDGALLYR
KNKVK
>BgerIr75x
MKLVTSVIVLSTLQYFSSALLDSTTLRFIEEYFIFKTAKIITIFTCSQHDAFRLQKALTTSAGK
FWVVTPDPSKPIDKAIDHIIMRTYNKFGIFVDYDCDAGKDYLFENSERRSLNDSYNWLVW
SEKDSPKVAEDLNLHFDTDMTWAKPDDDLIILHDLYKINYSWPLNSTFAGFWEPGKGITY
TLKQYKYARRQDMRGVILTAGLAVNNIPLDDLKGELEKPKNRKMDAMATYNYRIFTMLA
QFYNFSSFIKNSHLYGYVVENGREEGLILMLKNHDVEYSISAIQTNRARYRCTDFISVPTWK
FRIVALFRHPPVTGYYGQVLLQPFETNVWTTSAGMWAIVILTIRFVSWVEIKTFDFVPTSN
EDETVRSWSDTLMIMIGAISEQGTTMDSKWTSWRIGFLAAFILSMMVNINYGASLVSSLLS
TPPKSIKTTRDLIDSQLTFGAEDISYNVPFFALNSDPLIHELYKKKMAPPHQAYWARDVAL
RKMRKEYFAFHTEAIKVYPEIEDNFTPKEKCELSEVLVFVPEKCYNPIPWGSPHKEAFTFA

MRKISENGLVNYQDRIWFIPKPSCTIKEEFVSVDMDKISPAFLIVLAGVFVSSLLMMFEIFS
HRKKNAQNMLQAPVPVQVKVEKSAKPRETDVFRQNYRYFSQRGNYFD
>BgerIr75y
MKVVLFLLLSNLRFFVVARLDASTIQLVKDYFSYKGVRIVRSFTCSVEDKISITKALQSTGRI
WTILSDPEDSSFPTEDLLNWERSPFIADYYKFGVFLDYDCPSSKRSFNESSEKNFLNLRHQ
WLIRSVKGYLEIEHGRLNIDSEVTWATLSDPTSGQINLFDLYKINYTWPLIATPVGHWNAS
GLHYNITKYKYIRRENLQHLMFDMAIATFNPKNGVEDVHAHLIMENEKNFTTIMSKCSYA
QTLMLQKKFNFTTQLHMTDSSNFAPFDDGFFNGHIGMLQKNQATFSPCPLLVFVAREKVI
DFVAPTWYFDVAAMFRHPPVRGMGNAFLEPFSFAVWMCTLTSWIMAFITLKFAAWAES
SLQEESEIDSSWSGTVLATVGAISEQGSEMESQSVSWRIMFLFILLQVVILNNYYGGGIISSL
LAEPEKTIKTLQDLINSPLAMGYEDVNYNRGYFKESNDPLIHILYTKKIFPQKQQAPNEYRI
DIGVKKMKDEPFIFHGEQIGAYPFIDATFTDVEKCALTEIRIFPSMVGPVYTMVQKGSPYKE
IFVYGYRQIWERGLMRYQLLKWQPAKPRCLVVTEVLSVELGTILFAYHILFGGLLIATTLM
LIEMMYFKRCCLKLSIKKIKKKPRNNPDLPENGSSVKTPRQFLI
>BgerIr75zI
MGISNIILAAFFPIVISGALNTETIQFIKDYYTHKNIKAIATFTCSKHDAYLLAKTLTNKFWII
SADYEKPITKFIDPVVKRSYSRFGLFMDYDCPEGKQYLFQVNNIPTENLKEELVKPENRKM
DAMATYNYRIFDMLQQTYNFTVDIVPSKYFGYVVENGKLEGLTLMMKNHEVDFSVSAILI
NRARYRCMDFVPVPTWEFRIEALFRHPPVTGYYGQVLLQPFETNVWWTSGVMWIIVIVII
RFVSWVEIKTYDFVATSNEEETVRSWSDTLMIMIGAISEQGTTMDSKWIAWRISFLAAFIL
SMMVNINYGASLVSSLLSTPPKSIKTTRNLIDSQLAFGAEDISYNVAYFALNSDPLVHELYQ
KKMAPPNNRPYYSRDEALKKMRKERFAFHCESLKVFPEIEDTFSDKQKCELTEILIFPPEK
CYLAIPWGSPYKEAFSIAMQKAHERGLISYADRTWKFPKPACTIKEEFVSVEMDKIFPAFLI
VAMGVFLSFIMLVFEKLIKKRSVALEELNEKIHSLQENAMHLAKRHDSNKLHAPKEPHGS
VVRSWVP
>BgerIr101
MTAETLIIKAMNYTLYCAFIAEDGTDFALDFLRNVIPMITMPVTSGHCPGTSVGIIVAENDI
NVVHFTSKSTDHFKSFIILVSGTLHSVLEHPSVFTYISAYVVAANFSSVFYLSSPQNQEFLPL
KISDYIISKKLPNLQGRIVHAATFHCPPFSYFQDWAEEVHYGSFPLGIPDGVEMAVFRALA
WRLNFTWKLKEIKGADKWGHRDSNGSWSGGILGTLSRKRADIGFCGIFVDETSVQVLDLT
IPWTHYCLTFLVPLHASSFRFAILRTFHPLLWFAILTTTLGTTVIYCLLTHAKKEPQDLPKV
LITAVGILSLSFIPEHDFRILRPLMISWSIYSLLMATALSSCLVSHLTRPPPAKQLNSVQDLV
RAGITWGQDFKQEQTRLFNLKDPWHVQLAERFQLESSQCDRVARVRNGGYAILGGRLDG
IAPYFMEGDTLKDTGLISQLHVMRECLIRHYSAIGLTKRSSLTEPVSRVLRWLLETGLVEH
WQADLVRNYGNPEIKNLFHDFGYKGPQKLTWTHLEDIFLLLSIGLGISTVIFCLEYCWFTV
KSMEILKVSK
>BgerIr102I
MLQVENPLTDYGTIAYSIFDATFQRYCICVQVTLTGNIAESLVIAAAAYFCPILSLTFDRNIT
LCKDVSLLVTERIKDVEKAATQYTPLIVFNTGKTKLLFNDTTLAILVIIQEQDDFLVQDAID
DQILGQWNPTEGLVVMMETPWIQFIQKRRLSRMNTPRISMFECPPICSLWLTTDAPCGM
QLSTPWARQKGTFLAPKPQPLSRAILIYLPLECWTWVALLSVCIIFSLVLHIVGRLLPTQNR
YRSIRLCFLNTFRTLMLNGVPGFPTQVSLRWLLVGWSFFSLLTTTVYSSGYTSLLTSPLYSP
PIDTLGDLLEQGIYWGEESENTFAYLIRESGNSKLIEFGNRFKRELTPRDRENRILKGNYAI
FSKVMASAFVTETERLSQRARRMLRVMEEPVFTFYVGIGLRTNSPYETVIDSTIVRLHGAG
LLEFWQRLIIQKLGYRYMNTFFTLQIDDDNIHALTLNSVLGAFSLLGVGLVIASVVYVVEIT
TRYIKGGNTIPDKKKYYRKNSLFYINYQWA

>BgerIr103
MQLRKCCCCFFVTWILQPAISVLEIEMSEDDLKRKAYILTQLYFDLLVTSNCISVSLYGTKT
NNIATNIYKALPAPAVSGPIYSLKCENYMVIMDKSQSIRELKFILRLNPNTGIIILILDECLNN
DVNIVFAGKNKVILICIVNDTRYMMDNNGKFVALMNSTIHFKEEFSSGSPNYLGKRLVVGT
FNCPPFVFEDVTNTSSSNIESAENLDGIELQVFLEVIRRLNFTWKLLEPTDDNKWGWKME
NGSWSGGVIGQLVNEETDVGFCFLWMVSPQAEDIDLTFPLSSTCNTFMVPRPRPLKQLLA
VFKPFRASLWASVFGALCFISIVTWKFRRFRQNIHSLDWHFLEMLGILTMENVSPPRKEL
REARHIITWWAMFTVLVSTAYSSGLASHLTVPLYEPLLNSIQDLVLAKYYWSQAFIPNMK
NLFDISNVWHRQFIDTFQLDTSMLKATRSRHASLGTILEGAEYVTEGVKILPLEYTKLRRIE
ECISRYYTSLGLRKDSPYTEVFNHILIRLFETGIIGQWTRTITLKHANPAMTNIFSPDREVGT
KPKVLTLKNIQGAFLLWCVGIVTSICSFIAEKQIAKLKIG
>BgerIr104
MLQVAVFAFLLFPKYLTSEPVVLEEEYLSSTELESLMKILFPTNNVCNCHYIKSHNKIDRVI
GYIHQLQCISLVIGDTVKNSLCKEHIFIIGKESDFDELNVIFNSDYIRIIVLIIIPTCPADVLRYI
DRHKFGVGQIIISCVLINSIYIYHNSTTLMKMDKLTTSSGTILKLPNYGGKFLRVSTFNCPVY
SYGTEDNIRSSSNDLEQLDGIEMKIFLEISKRLNFTWRLEEPQELNKWGQKFSNGTWSGGI
VGALVKKSVDIGFCCLWLASPQAEDVDLTMPWNILCSTLLVPRPRRLQKLGAVFYPFTTSL
WAVLVAATFSISMILWCIQRVRQQAVSSGKVFSLCRNFQHFIGIISMGSMPSAYVQINECRH
ISTWWAVFALLMTTAYSSSLVSHLTVPLFDTPLNSVRDLVMADVHWSQSYFPAVDVIFDL
ENSWHRRFIKYFELDPETAKVHHAFHITDHASLGFVLEGDRPYFLEPIPMNPSLLPSLRVM
KECVSRYYISIGLTKNSPYTAALNEVMSRLIESGVIKYWQKDVVLRHTTPQMTQVFEDSDG
MVSGPEVLKLKNLEGAFLLLFCGLCFACVTMFVERYILNKNAK
>BgerIr105
MFFIFNWFAFLLFMKYVISELSITIEEERPSIDTELFMETIIPNDNSFNCYYINIHNKCERVIS
SVYQVQSISLVIGDAIFSVMCKQYIFIVENDGDIEELKLILNSDYNRIIVLIITLKYPENKFWN
MNNFTTQIKILHVPMNSLETLMKVNYVNVYSKHALKLPDYMGRLLRIGTFNCPVYSYGIK
EGMRSSSNAIDELDGIEMKIFLEVSKRLNFTWKIEEPQELNKWGQKFKNGTWSGGIVGAL
VEKVVDIGFCCLWLTIPQSEDIDLTLPWGIHCNTLLVPRPRRLQKLGALFYPFTKSVWILFI
AAIFFVSTTLWCLEKTRHEIIPGMKVLCLDRIIQDLIGILTMGNTSSPYIQIHESRHVSTWW
AVFVLLMTTAYSSTLVSHLTVPLFDSPLNSVRDLVMADIHWSQSYFPAVDVMFNLENPW
HRKYIRKFQLDPGREKFHHPFQAKDHASLGSVLEGDRPYFIEPVPMNPKFLSFLRVMKEC
VSRYYISIGLTKNSPYTSGVNEVISRLIESGIIKNWQSDVIIRRSSSQMTHVFEETDRKLSGPE
VLKVQNLQGAFLLLLCGLCFGSLMFLFEKFIVKLGRIYSYLYCLKQN
>BgerIr106
MYSRWETVFWLLLPQFVSNRLIINDTQTQNREYLQIVIRVLELFQCINMRIEGQQQYEEILS
SMLYENLTASMFIGQMPGLSSCNQYFIVVDKSEDLLLKTVLADITLVLLIHNRSCARSDEIK
QIEVINGKLILVCAFENELYINEDLSGLKKLLNFSDIYEINFNGARKYDGKELLVTTFDCPLF
SYGTEGDMNSSSSNIEKLDGIEMKIFLEVSKHLNFTWKLKEPMEINKWGSKLENGTWTGG
IKGPLASATADVGFCCLWLTLPHFKDFDLTIPWDILCNTFLVPRPRRLSDVGAIFTPFQRA
LWCLLVAIIFATANMLIWLERISSLMGFRRTGQGLCKHIMDLLGILTLASFPPPSVVLQERR
HVFASWTVFSLLMVTAYSSSLVSHLTVPLFEPALNSIRDLVLANIKWSGFTNFTSAKFLNT
EDPWQSRFLQNFYIDPEAAKKHHHFQVKDHAAYGFILEGGIPFFLEGGVQMNQSAYPFLR
VMKQCASRYYISLGLKKNSPYTSIFNKVIMHLLESGIVKHWKENVAYRHADKEITKLFADN
LAMSENPGSLKLENLKGTFCVLLSGLILGTVVLIFENLAKKINTRNLNLTRE
>BgerIr107

MHFLIETFFLLVNLQYAVTHFNSKLSKPYNRGYLELILQITESFPCLNIQVYNEIHDELYVNI
LKHLSIPTFVGNFSKHSLCTQQFLIVENETLFIETVSFDILVILLVLDSNCFRMNEMKSSCHG
VGKILVVCAFKNTVHLVTNSKLTSNLSEFSELINESTKQLPNYFGKSLLVFTFNCPLFSYGT
AGDIDSSSNNIGKLDGIEMKIFLEVSKKLNFTWKLREPTELNKWGFRERNGTWSGGIKGP
LANGTADVGFCCLWLIVPQFQDFDLTFSWDILCNTFVVPRPRRLNNVGAIFRPFENPLWC
ALMLVTFLTAFIMQRLQGVSCRTHLIKNERDLVKDSLDLLGILMLASFPPLRSECQERRHIC
AWWTVFALLISTAYSSSLVSHLTKPQFEPLLDSVRDLVLANIKWSGSETFAESSILSPEDHA
AYGFTLDGGIPFFLEGGVPMNPSAYPFLRVMKRCASRYYISLGLAKNSPYTSAFNKVIFRLI
ESGVIKHWKETIANRHIDPQMSKLFMDTVAHSLEPQPLKLVNIFGVFILLSCGLFIGTFVFIL
EKCLMINWKILIYSTLVMLIKE
>BgerIr108
MSLGCILLFLLLILRDVANRLDIKDIANIIFQYSEEEMELLQHIIDNPICIIYTFNYEKVHSISK
QYSSNLTYQTGRVNNLPCIPTITVGETYIQQVLNCSDIFIFVIEPAGSNTQLPNKIRFDTRQFI
FIRAAKNVAYFINVSQISTRVENISEISVPKSRHLPNYGGKLLNAMTFNCPVFSYGAEGGISS
SSRDIDKLDGIEMKIFLEVAKRLNFSWKLDEPTDPVNKWGQKSSNGTWSGGYKAALASG
KADVGFCCLWLMAEQYEDFDMTIPWDIQCSSFLVPRPRRLSNFGAIFSPFTTTLWVVIIVT
AILTLLILHCLQRVRRCFITRERKDRSMTTNSMHILGILTLSNLVTSRPEFDEGRHIFAWW
TVFALLITTAYSSGLVSHLTVPVFDHRLNTIRDLVSANVEWSEPYEPAMASLFDLQNPWH
QRFIEKFHLNPERAKKNYRLEDMNNVVFGFILEGGTPYFLQGGEPMDPSGLISLRVMKEC
VSRYYLTFGLVKNSPYKDAFNDVLFRLIESGIVNYWKKSIAYRRGDAEMATVFIDEVKINN
EPHPFILKNVIGTFIILIFGLILAILIYVAEICLR
>BgerIr109
MRWIPIVMILRKCQSNILLKKDLPNKFEFLVKIAVQGSFDNFWCLQLTTDSNVTEKMLPLI
YEECNSPITFTDSTCKGQIIISNKEASIPHIVGRRSTSNEFRFLILLIGNLNMILQDVSMFGEA
EVLLVDTDNLYSLVTSRTARHFLRMTSSKSELFPAKVTTLPDLMGRSLKVGTFKCPPFSYF
EGRADSKDVENLHGVSDKFDGIEMLAFLELAKRLNFSWNMYEPSDTNKWGTLYKNKTW
SGGIFGRLANNSIDIAFCALWVVEEQREISDFTVPWSQLCNTFIVPRPHLKYYWYSIFLSLK
PTVWVTFGIFVAATALLLHLFVKLHLRIIYSPKSHKYEEFGTSLLEALGMLMSTYSPTNEQ
QLGPARYVICTWCFITLMFLTAYSCGLVSHLTVPTFSDNLETVRDLVEADFTWGHTYFPSV
YSIFDDRNTWHQKFINRFRLQSDPERNVLLETGKHAVFGYKVEGTVRYFLETERMEPEA
WRQMCMIKECMSRYHIALGLTKNSPYTAAFNQGLIRLQKAGIMEYWQRNVILRRGNSNV
STIFYENSNIASDRPEKLTLLHLEGAFFVLFAGLTVAGMSFIAEIIARGPKSLLIRM
>BgerIr110
MSPKCIQVAVITMNERVTCQLHPNTAGRNPHLIMFYQGSTTQSLFLKKCSEEQQFGDKNF
WLFITNQSLTTDLFFILKGLNIQFNSNVFVATQTNGTQNMFTEVYTDGHDLKIEEDATIW
ENTMAGQTYNYAKIKRFRNLHGHRLTAIAVGVNKFYTEVKKSGNQFQIVGGYFGEVFKTL
AHLLNFTYSVKYLTGYAYGNTPTDDNSSWTGIIGYIQRNEADLSACEVSIVGDRLLVMDFS
MPVHISRRRLYIHEGIKVLHMDWYFRPFKMSTWCALIICLFFFTLINSFLHHISYKMEAVQ
RRVGILSNFFTVVTIMLQQGTEYPANMSVRLVLLSCAFMFLVVHVAYSAKLMSLATMYISP
PPLQHLDDILKSTSWKFSLTNGSLEYNIFKTAMKDSVLGQIWQQKLEPFPEQLVNDIRSGL
LEVLSESPTIFMGHEDGCENTLQNSFRPTEACQVLALPQTYFKGGMGFGLQRKSPYKDIIN
YSLMKMKSSGILQRLKLEWIPQPKECSRSLQIVTEWLDIVPPLTAYVTMLVLSICILIGEML
LHVVLKGGRKGKLKKIKQTGKTYFK
>BgerIr111NIC
SYELVPFTKLGWRNEEKAWLFMLGAVTSGTVDMGLDNVVITNERSMDMFFTLPIVQSMT
VNDTALRRLYFRGLVRNHGVAEASAGLQKASTGGYAFFVSARLARKALNSFIAQDKRCDV

QELTVEATKSAIALPMGLSSPYRRLINLSLLRMREAGVLGPIQERMLPPMPPCKSYSAFNS
ASITDVYSAFLVFGGGLATAVFMGLSEKIWKRRASLAKWIVVKWANKDKVKILFNEPEP
>BgerIr112I
MQLCCASQSILIIMVASTLCATLFMTLPITLCLPPVEIIESVLSNYHRGCVSLLYPLTPRLSYR
KALWNSSWLLFMDTNIEMKNFFRDIYIPMNCKFLVVQFNNGHYIMTEVYQIAKGYPLETF
QYGLWTQETGIALSSLSLYERRNNLNGFVFNASGITEPPLTVVKAINGTLEVRGFFGEVWK
ILETHLHFKTHLEEAQDNAYGSLDNGTWSGVIDMLINKEVEMGLGEFRVTSERLEVIDYT
LPIILSRYQIYIREPTSQNMMWGNFLEPFSSELWLTVVASIVIFSIFCAIIFYANHQLFYRNES
DMEELTIWDAIHIVFGAFCQQGYVITPRSNSCRMVLLCVYIIGAVLLAAYSAALVSFLAKRK
PILPFNNLEELVTDGTYKFGLLQKSAEFNVFYGSKDKLLNETFNRFIKPEKENLPTSSQEGL
TRTCQSKFAFFVSQDIAQGLLHKIPCKIIELPGDSFKGSVSMALIKNSPYRELINHYLLVLRK
GGVLGRLRNNIWPKHFPDSTSTWITVDLHDVTPLLTVLAAGILVSFLLLLLEQIFYFAINDS
QSSPFPWTL
>BgerIr113
MKLEAAKCFAMSMLVLAQGNVLFHNVKDYFGVITLVSNNIHPRCIIILHPSSNSTEKLLGA
LGGVRVSVVRLETYRETQNILCQNREQPLIVILSSTAIVKKKLELLSLDDGLSEATWLLFLN
KDFTLNTFFFDINIPFDCEFLIARSTGEHTRNIELIEVHRVTAETALLTYPFATWSNKESKV
TNTTLYERRNSLHGIHLKGVSTDDPPFTILENSEEKGIAIGGFMGDVWNILQGTLEFSTTF
QVENTFGVVTNGSWNGMIEMLRTKQIDVAVSEFTLTDERMQVVDFAIPIIYTRYQVFIQKP
RHENLTWSDFLEPFSRMLWLTVCFSILVLSLFLSILHSFGRRFGNEESIGPSRYTFNDSLHY
MFGIFCQQGHEFTPRAMSCRMVYFTAYIIAVVVYAAYSAALISFLTEKTATLPFRDLRGLLK
DGTYDLGLLRDSAEFNLFSNSNDSVLKEVYEKFLVPESSLPETDEEGIYRLCSRKYAYVSAG
LAMSGHTFPCDVTKLPDVGFPVSLSLAFVKGSPYKGLINYNLLELRDKGTMKRLRGLWW
TSKSAADENVWSTVAFNSVVPVLVVLAAGMVTAAVVLVMEYHWNNVQRLRSQKQSSPA
PNDFHHIKPKIYYNNSFEGEFREC
>BgerIr114I
MNLQATLYFVMVLRLSSFVKGNVLMEYTLSEYSGVVTLIFRKYHPACIIILHSTPNLLSEIGL
YDKWKIIEFEKLLLQVNVRITSIRVETYNHTLCQDGAQPLTVISSSTPSVKKKLQAPSWIST
LSDAIWILFLKSNLTLHTFFSEINIPFDCKFIVAQSHEEVIELTEIYRVAAGMSLLDGHYGK
WSENGSYITSVTLYERRKSLHGFKMKGVSIQNNPVTTIRYTNKNEITVGGLFGAVWRALQ
DALNFTTTFQVETTFGALNNGSWNGMIEMLRARTVDVALSEFTITQERMKVVDYVIPIIN
THEFTPRATSCRLVYLTAYLTAVILLAAYSAALISSLTEKTATLPFRDLRGLLKDGTYHLGV
LKDSAEFNLFSQSNNTVMQEAFQELILRENDLPESYEEGSKRICNGKYAFVASALAVIGSSV
ACGVSALPDVAFPVSLSLALTKNNPYKDIFNYNLHILRSEGTLKRMRDKAWTPKLPKVKS
PWNTVHLNSIVPVLVVLIAGILLAGVILAMERHLHRVYRLKHHKKVYNCTSLRFSRTPIKY
TDSGTLQKLCTK
>BgerIr115
MYVKLKTWVILDIFTMLMFALECLGKVDGVIFLEYTMSDYVGVITLVCKKYKSRCVSFLYS
YELNIDINLEQTKTITTLEKSLSSSDKSIFSSSSLLDNNSIFLDYKCPNKHRPLVVILFSNIEIR
QQLQMLSHQQSMSASAWLVFLSDSSTEDYFSDIYIPFSCQFLVAERRNDFIELRELYRVAD
GKPLITFPFGSWNSYGHNCTGTFLYERRNNLHGLQIKATTMNSPPVVLLYENENGIQGIDG
YFGNIWTNLETNFNFTTSFKKGKAFGVKKNGRWNGMIAMLMSNEVEVGVCEFTITPARL
KVVEYTTPIIFSSYEFFIRKPRNEGVQWNGFLQPFSNSLWFSICGWVVLLSILLTTFHFLGR
RYGNSEVDDPPKYRIVDSFHYMLGIFCAQGQDKEPRSPSCRLVWFIAYITSVTLLAAYSAAL
VSFLTREHEVMPFNNFKGLLEDGSYKFGVVGDSAEYDIFDKSRNRLIRKLHEKYTQFEENV
FPNYEQGMQKVCSSRFVFMASYLLQYRDNVACEMRYLTQEAFPVTMTISVAKHSPYKGIL

SYGLQKLRDKGALKKLQMVWLKPMQKKEDPWDEVDLKNVVPIFVVLLAAIVTACLLLLIE
REWHEVQRRRRSLHKSTETNSFSDLNITNKVLHNKIQFSDP
>BgerIr116P
MRLHLWQFVAAVFFNYTTQTRSDFDLEIAARVTLAMHHYYKTGCIFLLHTPGFKDSILQK
EMRRRLSEDETGTSIITFQMLEFKPHVNHCRYVPPLNVIMFHNNATLRSLRELHTSLYTK
VKWLLFMDSNYIIKETFEGLELPFNSEFVVAQQYDNYVNLTELFHINPEYPIQENQLGHW
TSERGLDFTDIPLYERRYNLMGTTIKTGYFQDVAVKVITNNNGEPKATGYFGEVWQALQN
RLNFRTKYSRSSTKAYGSLQENGNWSGLIGMITRNEVHVAVQSLTVTEERSNVVDFTLPLL
RSTQCILIQRPGRNGLHWGNFIEPFSTKLWMAVICCMVGLTVFLWATYYTLRFFSEELEQ
ANFTLPVSFAYVYTSFCQQGCITNPTSASARLVCIVIKLTATVLLGGYSAYLIAHLAVDEPDL
PFSDFKSLLKDGTYKLGVLANSFMTMYFDSSASTLMPRIYEKLVKPYSLPNSWGEGIRNIC
KDKKYAFWALDDMVTSARKKATCRISVAWQSNRRNAIAIAIAKGSPYRDIIEHNLNKMRD
SGILHRLKESLWPNPPVPWKNTYNSVQIEAVAPILLLPAIGVITSIFILVSECFIQRYNWRFH
NNEMIIG
>BgerIr117
MKNCFIILLLQTFLICTVTTIQLHLPSLVDIIVSLKNILYPKGIYLLFASNGVDMDEYPQIILLE
KQLSSRGVRSSAFEVDSFNFTNEKLQNSTNALFVLLFNGEEFQMKFQKISRELKLSSATWL
LVPRTEIKVNSLFSNTYIPFDCELMVVQQSSNHVSLTEVYQINESSSLLLFAFGNWTATQGF
KWCNLLMYKRRGNLGGHTIKVGFKYGDYVNPEDFLYDVVDRTDGVKVYFSILWAILEKIL
NFKSIYLRPADNNFGALKNGTWNGLIGMIVRKEIEIAVDGFLITAGRMDVVQYMRPLFRA
RNRPFIRKPETFERHWGDFMTPFSTELWLAVLATLVLLASFLSALYNVGHNYCGEAVVGV
TEYGFLDSLFYVFSSFCQQGHSVTPLSNSCRTVYVASYLIGMVLVAAYSASLISHLTVQRIEL
PFTNFEGLLKDGTYKLGVTESSATFNLFDKSNSSLMQAVYTTLIVPYDLPKTSGEGFKRVC
SDHKYAYMSPFDTPLSRYNGLECDMVEIPYASFSGSLTMPIAKDHPLLGLFNHVLLNMRV
GGTLDRLYTFSWPRKPEKWTSPLTSVELEEVFSLFSLLIGGMGFAAVTFIFEKCLCYITNIS
EEENDVKYRLGY
>BgerIr118
MAKVLFILIPLFQTTCTYYTPLNNAVEVVDSVQRIFKSGCIFLLFSKEESESLDGEQYVFFLK
KILAKRNIRTAATNVVSLNMDSKDYQCTDNIPMYVLLSSSNSTKERLKQVSRSSQMNAAV
WLLLVEYHSDLENVFRDIYVPFNCEFLVAQNRFDEVILTEVFRIGSTLPLQTYTFGRWTRA
HGFSWSPLKFYERRNNLEGIVLRAGYKYGDGIHKEEIRKAIDRRNGVKFYFSEVWLLLEE
MLNFTTEYYRPADNAWGGLLKNGSWNGMMGMILRNEVDVAVDAYLITSARISIVDYMT
TLLPTTSRIFIRQPDTIMYHWTDFLKPFSIKLWMYLVLSIFVLAFLLSRLFNMGRFSNSMD
HRQTLSYGLLDCIYYILASFCQQGQSSVPASMSCRIIIITSYLMAVVILSAYSASLISHLTNQN
VVLPFNTFKELLKVGSYKLGVIDKSAQLNYFDNATDRILRKLYKSLIKPHLRDLPVTEANG
LRRICQEMNYAFMTPFDTPLSRYNNFKCKMVEITGASIPGSLTMPMSKYHPFLGLINYTLE
NMRRNGMLNRLHSMAWPRRLRDFSIPWKSVNKEEVTPIFALLSSGFVIAIIIFFLEVFVKY
LRICVQTRQIHRTGMKRKAHFVYV
>BgerIr119
MSNVYTWLIMLTMCVHGDCFLSMELQAELIYLVQRRIQLGCLTIIQSSGSGIRNTAVTVKM
QKILITFRNFIQLKIVLLETYNYTEMACYNGVPPMYIILHPDENVKLHLKKILNQQEMSRTS
WLMFFDSATPLVEFFSDIYVPFSCTFLVAEPKGENITLTEVYRVAPGKPLLTNVFGNGSSG
MGYILSNDSLYERRNTLHGLELKGVSIMDPPITELILQNGRIIADGYLGDIWVHLEKQINFT
THFLNATADGQTALRNTSWNGMINMLHSRQVDIALSEFTLTVKRLEKVDYTIPLSISTYQL
FIRDPKDNYLHFGRFLEPFSLGLWLSVLGSIMIFSVLLALFHTIGRNIRNAEHGGPPKYRFR
DTILYMFGTFCMQGHDFTPRATSCRIIYITAYITSFIVLAAYSSVVISFAATRNPDELPFTNL

EGLYADKSYKLIVTRKSAMFNLFDVRIINTTDQTMSKLFERDLANIDNLPLSDIQGIKRACS
GKYAFMSSKKKVMGMKNVCDVVVVPGVAFPATFGMGLAKHSPYKGIIDFNLLAMKTEGII
QRIRYQTLGKMTIEELVPSNIVDVYYILPIMIILITGALGSILLLGIEVGWHRYQNYHSQNKEI
FKCRENRKIDLSTSHLKTISIYIQTRQGKSFYH
>BgerIr120N
NPCRLMWLLFLSDDICLEEEFSSIYVPFNCKFMISQLRNNTFYITEIYRINLEMPVFTYPFGF
WRTDNTWRLANNALYGRRDSLEGLTMKGNPPFTKIKTTKEGQIYVSGYIGRVWALLEEM
LNFTTIFRVAPQHGSFTKGNKSWTGLMEMLRTNEVDVAVTDMSITTQRMDVIDYTIPML
SMRLRVFLRQTPLDDQFLWTNFLRPFSKSLWLMVTASIFVFSIVFHFINIADCHFGKKGSR
RHNFFSFLYHFVGVFCLQGQNMQLFSNSSRIVYITSYFTAMILLAAYSSSVISSLATKNEGPL
HYRSIHDLLVDGRFSLGMRKNTATQDYFEDSGDPKTKKIFISLVKPHLENLPTSEEEGFAR
ACSKRYAHVSVSATASAMSLRCKVTSLPDEGFPISKAIGLVKNSPYKDILNYKLQQLRSQGI
LQRITNSWNKKTVNSRSTFNVVDLHSTVPILAVLIAGALCGIFLLVLESAWHYYRRTQLFY
STV
>BgerIr121N
ISSIVPSSIWLMFIDKKSTIQDVLQEASIPFSSEFLIAQEIGVNRFTLKEVYHVNSCLPQNVQT
FAQWTPTYGLIWQNKGLYQRRSNLKNVVINVGINVDDFFILKNRNGKDGFQITGFAMDI
WKTLEMRLNFKTKTSIILNNSWGVLLDNGTWTGMIGMVHRNEVEVGIGAFSMTSSRTNA
VNFFVPLLNVKSSVYFRATDGEGILSDNVFIKPFRLQVWIAMLTVITLLATGLSTTYNIGRR
IGNEEKYVPPVYKLLDSLFYVFTSFCQQGNKVTPRSTPCRLVYFTCYATAVTVITFYSAALV
SHLTVKRSPIPVTSLQEMLQAGTYELQVGKHSFELDYFNRSENPVMAQVYRKYLEPNSQS
LPESTLEGLQKICEKHGYAYMDYATPYLKYINCSVTQMTKDGFPFSLSMIINKKSPFLGIIN
HHLQQMRRSGLLKKLYDHHFPRISDEEATWRATSFEGIAPIVTLLCSGICFSWLCLIMERK
LWTIFNTRHRNKSAIRRFPFIN
>BgerIr122N
LDEKSTLLREFSRRHMQIGILNFNTAEEIRPGKCLNNKVLQVLLTTDEITKRALSEVSQDR
GVMSEAFWLLFVDVELDLRIFLEKVLIPVNCVFVVARRGYSETIILKEVYRMDQGLPLQET
WFGTWNVRSGLKATPHSLMKRRSNFHGYLITATAMDFPPLVLMKEDENNLTGGLFGVV
WKILEKRLNFRTVFKIPDERTWGVYHSNNTTTGMVDMLHSGKVEVAVFNMLMTSRRSL
VMDFSIPLLNPAYHIFIRRPDGMHLEWDTYLAPFSRKLWFAVLLLILLISLFLPVLFQIGRR
HGLAETTAVVRFRIKDSLFVVFGAFCQQGMEPGCPQQSSCRVVYLTMYLTAVILLAAYSAT
LISFLTIRSISLPFRNLEEILSIRTHKLGALKDSTMLHHFKQNSSGLQLYAEVYDHLLHERYG
SLVRDTKTGLQRVCDEKYLLMAMYTDVVTHLPSLHCRVMTLPSNYFRSSLAFGFRLQSPY
KPFINYHLQAMKDDGVLHKLRSHLEAPFKVHQNDLGESVSGWVSVEMEHLLPILTTLAA
GIVLGASFLFLECAGRSVLFRHIWRVRFRRSPPFSPYRVLSHAKSQLLNA
>BgerIr123
MRRRIMYKYEVIIYCLCLCFGGVCSGLSDQADLIAALKLHLRFGCIFFLGADYLDLLQREKV
SREVKFLSMHQIATTLLQNTHMVKCLRNKNIFIMSATEDSVSTFLHKFSFTKNMSDSAWL
LFLNSDADLDIIFSKVDIPFNCEFLVAQTQTDYVRLTEVYRVNQSLPLQKQYFGSWSSRKG
LNVHAGGMYTRRNDLQGILLRAGVMDDDYTAVLEEKDGKPIKLRGFGYEAWKVLQNRL
NFTTEFIKPEDCQYGVMEDDGNWNGLIGQILRNETDVSITPLIWNKQRNEVVTFIASMFKI
NLVLAVKTHDSSDIIWDNFLLPFSSGLWTSIIITILVTGFLLLFIEKIWNHHIREPIWKGTVS
SLSEALLTTFGAFCFQGHCKTPTCLSSRIMFLFIHITSVVVLCSYSGSLISKIATQVFSLPFND
LEGFSKDGTYQLGVLRASGDLTYFKESNNSMIKNIYDKFIAPYKSSSLLSSYLGGLTRACSQS
KFAFVFGDDKFTSLNSLKCSLSYLPIMQYDMSFLMRKHSLYKDLLSHSVHQMYESGIIRILL

HLYTPERYIKTSNDFSPVSINQITPVFVILVSGIAFAMLLFLGERTLRLFMHYEYHKNSQEIL
QNFCLKNTMKRHSPWQNTNFTNNTVTIQSTYIEYY
>BgerIr124
MVASSRGIFTVVVLLVAGSEGDDSASFTAGLILAIQGKYRSGCVVLLTTNEKGDLTHQEGLV
QVQLRKHLSQERVSVTARRIQSLNITALRCRKNIPLVVILNSDSDMKNELQEYSEKQNMAK
AVWLMTLSVGSTEQYFTDINVPFNCEFFVARTGVSVTLSEVYRASKNKPVQTFYYGRWN
NVSGLISDKPSIYIRRTDMEGVVLNVITADDPPILTIKDGGRNVSGFFGRVWNTLEGKMNF
KTNYTVTEDMTKGQELKKDDCTGMIGRLQCGEVDVALGAFSTSSYAEIDIKFTMPLIYTAY
QVFIKKPKGKTTEWNAFLMPFQPRLWLVLVATMLFISLYLATFYNIGRRIGNEEAGGPELY
SFYDSLLYIFGAFCQQGHDITPRSTSCRLVYITAYLTALVLLAAYSAALISSLTVSYSNLPFED
LEGILKNKRYKMGMVDKSEMFYTFSDEKEDGIFGEIYKKLMVQDPSNFPATTLDGLRRVC
SMRYAFFATPESVMPLLGKLNCTIIPLPYKSYPISLAIAFRPDNPFREFISYRLRNLRDGGIL
HKLRIDNWSFLSESATKSSLVSVDLEAIAPLLILLTSSVVVSIAVMFLERGKSALSNRDSDSS
KKKSRWERNSLRLQSTSKSLPYKKSSPSLFFWKLVPEKIIPPFQGWHE
>BgerIr125N
AIWLIFLNSDTILDEFFNDVEIPYDCQFLVAQPRDNYIILTELYRVATNFSLQSNHYGYWTP
EEGLTWPELTYYQRRSNLFGMTMKIGVTQEFYSERMALFRRMPTEPDLYADIWSILEREL
NFTTEYFLPDGEDLSIGDTLPNGSWTGLMGMLQRKEAEMCNAPLSMTAKRMQAIDFTVP
LLDIKTYLVIKKPSTYELKWNIFLMPFTQKLWAVILLVMLCLAVSYKAIGFLEIYFGIYHGQ
QISILGAFLTMVSAFCQQGNIDVPKASSLRLVFATSYLTAYVILACYSAAFITHLTLRKPHLP
FQNFEEFLEDGTYRLGMLQKSAQMDYFRQANVKLLKEIYQKMIEPYESTLYTSDKKGMED
VCRIDKFAYMVSTYSMMKRGFLPSCSLAVIPQAFYPGNIAIGITKKSPYLGIFNFKLNSMKQ
DTRYASMYKQYNFKLPRSEEDGSMAVNLYDAAPIMITLATGSVLALIVLVVEKVFNYVKY
HS
>BgerIr126C
MLTILFLMLTMPIASIGTDIKNLVNLVLAVQKYFTSGCIYFLHDHVKYWNSTHLTVQISKLL
FQRHIQLSMASKDGPIKCDQNRPLFVIPDGDRELTRSLVRDSSQTAIWLKFLWSQTPSDFF
RNKDIPFDCEFLLAIPKDEERLKYTLKEVYRVGPALPIKTYHFGEWDPLRGLTRPSVGFYE
RRNTLHGAVLRTAVQPDSTLFLPLDSNEYQLRKKDLFDEIWLVLRSVMKFKPEYRASDLN
VNGVKLSNGSWNGIIGLLDRKEAEVSCEGLSMTPGRAEVLDFMVPIKNDRHYVVIKKSNSI
ELSWNSFLLPFSITLWFAVLFTILILTSMLWITYKLCCLYWKDLQDEFNGSFRLQDSFFYIF
GSFCQQGLAKVPAPMSARMVCLVSYLTAVILLAGYSASLISNLTLRKPDLPFSTFEGLLASS
YKFGITPFSSSFQFFQEGKDPLFHEIYERLLRPYRHSLPSTSLEGLRRVCEIPKYTFLSDVAV
EDRQNEFSCHLFPLPEAYLPGSRAMAVVRNSPYLGLFSH
>BgerIr127N
FLAKENSPGTIWLVFSNENALLEKFVKDINIPFDCEFLVAKIEAEHVILNEVYRVHPNLPLL
THEFGKWSDNKVSWRRPVELYSRRENLQGLVLKTAVKEGFFDGEFYSSGLGRNYFCSQI
WFTLMKYLNFTSLYSYSNEMGNGVNLQDGLWTGILGMLHSGEAHVTAEGISMRKERLDV
VDYIFPIWTERVYPFMKATKVFKISWKNFLLPFELDIWIALMFIIILISSGFFLIGKRWSYFQ
KVMQERTVPGFSDSIFHVTGSFLNQVQDFNLQSPSSSILRLVSHWTSTVLIASYSAFFISFLT
LRNAVLPFDSFEELLKDGTYRLGILPYSSLQQVFQNANNNILGDVYRQVIVPYNRSLPTSSL
EGLQRVCSDVKYAHMYPLEGISKYTDKLKCQIMLAPHSKIETLKSMAIVKGSPYIGIFRHIT
YKMQESGIIQKLLLKTVTELTDVEEPQNIVDIKTVTPVLCVFILGLILAIFLLALELGFNRMK
PKPIFKLC
>BgerIr128NI

ISENINISKFIWLLFLESRSTLNTIFAEVNIPFDCLFFVAQSDDNTVYLTEVYRITPSYPLQTY
PFGNWTTKEGLRYPDIELYSRRNNLQGIYLKTGARKWRTKRGEDVDPGYYIGSVWNLLQ
TTINFTANYCIPEDNSSGSKMNNGSWSGVIGMMQKGELAISNIPLVMTPSRATVVNFTFPL
LSIHTYPMKNAAIIILFATTYLSAVVLLATYNAAFTSQFTVPRPWMPFTTFWDFLQDKSYK
LGVMPNTAQASYFEHTNDPLLNRIYDKKVVPYRSSLPRDDIEGLERLCSWDEYALATNTY
NLISMDFSPNCTITPVPQAFIPASIAIATSKDSPYRGIFNYKLETLRARGILQRLYKKQWKN
VYEEKGTLDMAVTFQEIAPVLIVLMTGIIAGIIFLLIEIIVYHFQEKYTSYAIHVDRESIKQRH
QPLIHI
>BgerIr129
MHSHTGNQSLILEDIALLAMTIQQSLYTDCVFLLHSENSGWNLLNKLINLEKILIKNGRMT
NIYSMEQLNHVQNRKKLNYESHENSCLTKRPIYVILAEDKDLFQELEIWPGPLWLLFLNST
VVGNLRNVYIPFNCKLVAVRKCAGNLFVLTEIYQVQEKLPLRQNHLGSWSEGKEMKWIRG
GLYHRRNNLQGLKISTAVIDVPGRMTVQRNEKNITIGDLFGTLWNMLERRLNFKMQHST
PEINSWGWMENNGSWNGVIGMIQRGDVHMGACAIFVTSARIETIDFTMPLFDFGYSIFIK
QTNPYKMAWDSLISPFSPAFWGICALAMIVMAFNLWMLQTSLNYLNKKNKDLNSIQYTF
GDSLFFLAGIFFQQGAEVNLISISERVVLFTASLTAITLYAGYSATLLAYLATDADALSFNNL
FEFLEEGGFQLGCVRESAEYFFLRDSKDPILKKAFMERLSPNLPNSNEEAMKMICNSSFAF
LAPNENFRPLIKNASCTIIAEFQEIFKMAIAIPIAKNSPYRELFDHSFLVLRDSGVLERLRKE
NWPKVMSPLEDSWVSVSIDDIVLATTVLATGIVVSIMLLVFEGVITKCKKHQIYKQMRRRK
RRINMEKIPAPKKFIMYC
>BgerIr130N
MQNYMTLNQLQKTLVRHVFSSATVPNSLLQSYLEKTLICKRNHPLHIVLSSSPDMKKFLQ
QMSIEKFMSKAKWLMFTDNAISLDDFFSDIYIPIDCEFFIVQRHYSTKEIVFLEVYNVGPTK
SLKVNQVGIWNSRGTLTWDNQSIYRRRRNLQGVVIRGAMEPDNMSIIVKEFTSGNKPSKV
EGYFGNIWNILEKEMNFKTELVQPDDHSWGSRLPNDSWNGMVGMVVRGEAEVAITAFT
MSTPRLDGVDFMAPYWSDGLTVFIRKPGAFQRTWVQYTLPFSPNLWLVIVISFILLGVGLS
TTYYIGRQFAGAREDQRVNYSILQSLLIALAILCQQSQDVSPKSISCRIVYYVTAYMTALIILCS
YSATFISILTVRYPKLPFTTFKGLLEDGTYRLGVLQSSAELDFFEKTNDSLMHLLHVEMIMP
EEEDLPDSHLEGLQRLCTRKKYAFLGPNSILSSLLKNVSCNVIPVPHASIPATSAMVISKESA
YTQFFKYSIQTMRCAGILSKLLLSMSPFRATETENTAKFIVTFEMIFPILSVLGAGIISAFFVL
FAELLRRKIIVHVKSQLGSSKRRPSVIWYYKDRSFTRKRYSYLKYD
>BgerIr131N
MYIPFDCEFIIAHISGSEIANFTEFYHVSGNLELQINHVGKWSSKSGLTWTKLQLNQRRESL
HGLIMKAAVIDHATSYVVSRDSIGKPLDFGGYPAIMWKILEKNLDFRTDLYYPPDKSWGV
LSGNGTWNGMIKMVLDGEVGVAAAAFSMTATRLQVVDFLTPMTKEKLMVHIQQPKSWE
WSWRQYLHTFAPEVWKNIFVVGLFLTLGLSATYFIGKRFELRALWDPFKYNLGDFALCIY
GMICAQGVQMESRLPIWSNRLVLSVAHFTALAIFTSYSATLISHLTVTKPKLPFMDFKGLY
DDTRFHFGILWASAQFSLFNESSDPVLEKIYNKHIGPHLERMPLSEDAGLDWICADDRYA
FLTGVISIKLLDAKNSLKCKMVPIKKAYIPVTVGMVIGKKNPFKRILNHKISLMNKSGLMKR
IITSMFSIEKSIEDEETAHPILMKQVKHIVAFLGIGIAASVLILAIECIFHAKKQSNERKTITFQ
QKRPAEI
>BgerIr132
MRFPQLLCLLTLTCNVVAMFPMNVTTGLIVSLYTRWNSHCVYLIHDIGNAPLLKGIRKELS
ELSIRTASIPLELLESILTGMTCDKKLPFLVILESNKTIKHVLEAVSQRTTLSRARWLVFLLE
ETVSTFLAHINIPFDCNFVVGMLSEFEVVTVTEVYRVYPTSKLSFLHLGKWSSNSGPIWAS
EDYDLRRLNLEGITINATVISDGTTSIVTKMVKGKPVEIGGYVGSIWRLLEHSLNFKTNFV

MPGDGTFGSMMKNGSWTGMVGMVQNGFAEIGTAAFSMTTQRIQVVDYLSPLVKEKLTV
FIQQPDFSDSKRNFYMNSYNYKLWLVVLAAIILLTFCLWISHFVFLQLGILDASEEVNAIEL
CFSIYGCFCFQGAHLAIKKCSLRIIMFVTYVTALIILATYSATFTSYLAVRNVEYPFTSFQGIL
EDGTFQFSALKGSAQLNYFENSKDPVLRTIYTRLIAPYKETLPSTELSGLLRLCDDRRRYAY
MCGIITVNRAKNSLKCNVVPITGAFVPVTLAMIIRKNSPYRKAFNHILQKLRREGFLHRTL
RNVTSTKISTKEEETVTAITIHDIAPLLALLASAVLLSCFILILECLLYHKGHRCTNILQTEFS
>BgerIr133N
MSITELSNIKWLLFMDSQPLENYFEGIYIPLDSQFIVARHFERTITFTEVYRVGKMEPLRTN
NVGEWRSSDKGPRWTTLTFYSRRGDLQGVRVKAAVISDGAICNIMEFVNKKPIKIGGFFGF
LWMALESRLNFTSDYFTPEDGGYGSLHADTWSGMIGMLVRKEVDFAIGAYVMTEQLTRY
VNFLSPMGATKITVFVRPTSTAPGTSWTRYLAPFNADLWITILAALILLALCLSTVSRIGCN
TNKSDAENYTFYNSLLYIFGSFCQQGHEGTPQSWPCRFMYLSAYLTAVVVLAAYSAIFISFL
TVRHIYTPFTTFEDILREGTYKLGILQRSVELNFFDQTAKDSVLHDVYNQLILPTLDESISDT
LTGLQRVCIEPNFGFVCDSTRAASVMNKVTCQLVAVSQAFIPATGTFIIGKHSPYRRIINNN
LESMRRHGELGRLFVHAMPANIYKDSSDSSLIHVRIQQVIPVLTILFASIIVATIFLFAERYAF
HGFSPRRRKRARNKHC
>BgerIr134
MKSAHVPLLFLLAVSCYGDELQEVMLATSLRAHFHASGVFLVHSTDFSTHQRSVRFLKYLS
NAGVSASSPDISQIRKFKSETSKSLLVLNEDLKKVMNSVIGSQFVWLVLQRRNSMLVELLK
DISIPFDCEFLVASPITEGGFNLTEVYRVSDEFPLQIHNFGSWSSAKGLSVSVGSLYSRRNNL
QGLQLRTGDLKYMSIKKLLQVRNICFSLQKKMTPSSEFFNEIWKSLERSLNFTSLYVMAPD
NSFGSRSKNGSWSGVMGMLQRRDVVASNIALMASASRLLTVDFSVPVLDIKTFLLMRQPD
FSVPKWNAYFMSLSTHVWWTILATVFLGAFCAAITGHCNQPLLMVFGAFCQQGQDVLQK
PTSVRCILMTSHLTALVLMVCYSGSLVSHLAMQQPELPFSNFEEFLDDDVYELGMIPMSAK
LDYFKKSKQPVLRRIYKKKVSAKESRLPKSEAEGVKRICSRDYVHVIASHNLMDVGHLVHC
QVTPIPDAFFPGVIAFAITKGSPYLGIFNHKLRTMRASGVLNGLAKRYSFDFSKSRTSAKNP
LSFSEVAAVFCCLGLGMVISILIFVLEVLVKWWSVRQKNYG
>BgerIr135N
DMYRLSREQKLLSSQGLATASVRHMDRVECSRNKALIVAEAGPHLVQALNTVPNSQFTW
LVLQNDIQVAKEMLQDTSISFDCELLVATPANSKVDLTEVYRVSDNSPLQTDKFGVWNSA
EGLKAPQKSLQGRRTSLHGLALKTGLKIEVPVSGDKTDERESSTGFFLEYWENMEKWMN
FTTEYLYPSDGAYGGKLENGSWSGVIGMLMSKEVDASSVAFEMTADRAQVVDFTLPLYNI
RSVMIIRMPSTVALTWNAFLNPFSHKLWGIVIFAMILCILCIATTKEFGFSTSSFLVAGIFCL
QGHSLSQRTTSLRLLLLTAYVTAVVIMASYSASFISSLTLRKPKLPFKNFREFLQDGSYQLS
MLPSTSRLNFFKSSQAEIIKQVYRKTISPSEKSLPTEELEGMERLCTHDNLAYIVSDKHLAY
FGHMMSCGYKVVPGAFVPGSVAIALRKGSPYKGLFNYMIRKLRRGGHVRIIYERFASKRKK
SLKGYVHYDINLPDVAPILFVLLWGVLQASCFLLIERCVYWLACKRYLYTFNPSSVD
>BgerIr136N
VSNTVELLSLQKAIMERGIPVGTSEVNSIGNIKCKRNKPLNILFLDKEKSTGIKLELGDNASR
AKWLLFMQNTSLEGMLQDMYVPFNCEFIVAQWENGYGYSLYEIYRVSRESSLQIDNFGF
WTIDKGLTCSLKSIYSRRWNLQGIILKTAVLKSVAVTVTKNKDGNITKIGGYLGRIWNTLE
ERLKFISHYMESIDGAYGSPNKSGSWSGVIGMIQRKEVDAGPADLAISVSRMEVVYFTSPIL
DLKIYLFVKQENISRISWTKYFLPFKWQLWIAVVITAFILANGFSLIYYMAHKQGLETLECP
FLEHNYLDSVSLVLSSLFQQGHSTTPSSMSCRTLYLTSYVVAVIVLAGYSAALVSYLASREVS
IPFQNYEEFLEDGTYQLDVLLNSFIHAFFSDGSTLTIRRVYAEMKDRESNFPRSVEEGMNR
LCFGKNKAFILQTYMVYRWAKYLKCNVMLAPGTIIPASLALPLVKGSPYIELFIYMINKMR

YAGILQILKERTLATLPPQPPKDFYSVELIDMAPLLTALGFFMLLSILLLIAEYTLCYIKKTEL
KNRNLSTPKPEI
>BgerIr137N
ISDFMGPVWILFAANSARAEQQLKGIFVPINTEFLVAKNTENENILIEEVYNIEKDKPKVW
HYFGEWETGSGLRITSSPLFERRSNFHGMTFHVISTDNPPLTTLKSEKGKLLAKGFVGSIW
NDMQKGLNFTTVYKNEGGEEIDGKWAAMIDLVQSAKYHAGVDALTMTSDRAKVVDYTL
SMVNAKYNVFIRKPDQQEVEWRTFLTPFSSGIWYTTVVYLIVTAVGLDVTHHFSKIYSQTE
INQKDMSAFSFKDSLFYVYGLLCQQDMVPHSYSSRMAFSTALFVAVVLLANYSATLISFLT
VRIEKLPFEDLEGLYADGTYKYGTQGKSAQYFYYKDSNNTFIRQLYEKTMASDPGNFPELK
LDGLRRVCDMKYGFYTNYDTAMSFRKKLSCKIVILPHELTTVPLSIILYKNSPYRRFFNHKL
LRMREAGLLDKHYRDILDMTQDEEPDLTSVTIEDITPILVVLAAGVGISCSILLVERLHSKW
KANNIEDITSSVEETRKRVVSPFVKRRRRTFHHRTASSSKSLRYMYKKPVKFYPRVTARIF
H
>BgerIr138I
MYSQIPIYWFPLAVIFSVTSSYYFDFGGNVLKNSLDNTATVVTSIQSLYHSNCIFLITLSTLS
DPDFEELSMLTELGKLLSKKKASNVIVSAAIYNYSERAFGCPRNRPLNVILSTNRTNSTSVS
QIKEIKGPVWMMFTEYNVYFDKLLEQLYIPLSAEFLVALSLPGGNFLINEVYRIDVYSPLK
WNTFGKWTVDEDLIVTEQNYFKRRENFHKTTFQAITTDTRFFTIQNNDLVSHKISGEWS
AIVEAVANSRVQFGLDAFAMTSSRALIVDFTIPIHLTKYKVYIRKPNKQEVYWSTFFTPFSK
TIWCTTVIFLGIVALSLNITQSLRRIFINFNASQEDSEAFSFKNSLFYVYGLVCQQGTDITPK
TYPCRIAFTTACLIAVVLLANYSATLISFLTIRIEKLPFRDLKGLLRDGSYQYGTTNASAHYT
TYKTPTDRFTEELYRKTMATSINNFPKTNLEGLKRVCRKRYGYLTAVDVASTLNRNISCEI
VQLPDENFDVALSFILTRNSPYTRLIDHRLLIMKEVGLIKRMFLTVMDPTKDEETDITSVTI
EDVSPILALLAGGIALSIIIFILEIRFQEWSKAPHKQKFTTMKGRSLRTHSYTRRRHRPLFRR
RIPNSSKVLSYYYKNQPTLQTRKLSEIFQNNFDTVYGYC
>BgerIr139
MKYRIFIIVILIPVITTIYGYIDKDVTLTEGNFISALRDYFQSGCVNLVYNKAKEYKGSHKYIT
DLMKHLSVLKVPTAVFVSSQMNTTAWRRCKHNRPINVILGDTPNLQPVLPEEIWILYQKT
NGTSAKNLLENVQAPFDSEFLIARSTGNATDISEVYQLYNNQSSLEYHFGSWTDHLGLKVT
NLTFNKRRSDLKGVTLSATVLHNPPSVRLLESDDGTLHVNGIIGHAFGILKDLTNFNIPRE
AKWGVPENGTWDGMIGEIQRKEVDMAVNGFFMTSKRMDILDFTLPLLYTRYSIFIREPD
NKSTQWGNFLAPFSSKLWIAVLTAIVVLALFLALLYYCGRRYGNQEAEDSSRYDLYDYFLY
IFGVFCSQGHYLSPRSNSCRLVYLLANLTAVVLLAAYSGTLISFLTVHNYNFPFTDLDGLLA
ENSYDLGVYNIPDEWYKTNESFPKLNKAYMKTMANDPQGYPMTALEGLQKVCNNKYAF
LLPMDTALALSNDINCSLVALPHGSFQASLSIALPKRSPYRGLLSYKLHELRDTGIIHRLRQ
REWAPQYEKPQPWKRVGLEEAIPIFVILIFGVLASCLLLFLERRYTSINLKMEVDKASTVTP
KHEPCKCLYRYQHFKYPLNQDKSVREAYPFFQ
>BgerIr140
MLIVLLAMVALMSSCCCSCCGNETIFTSTSGIVASLKKYYQSSCVFLLHTKQQSTSRTIESKL
EKLGRQISENGLPTALMSLSKFNHTSEKFHCKSSQPLVLISSTDQDAKDTLSKMSHRTSLS
GPIWLLLLDESSSIEDFLEETYIPFDCKFLVSQSSDNGLIILHEVYRVAEGLPLQIENFGYWQ
SETEYNFTQMSFYYRRSNLQELVIPAATLENPPIIEIRKTNGDITVDGYFGKVWKILEKNM
NFKTNFTFPKDGARGSVLENGTANGMIQMIADEEVHMAVDAFGMSGIRAKVVDFSIPLLS
TRYCVLIKPPDTMILQWDNFLSPFSSMLWVAMMITVLFIAIHLTVLYYLGRQYGNPEAEET
KLYTFSDSLLLVLGIFCQQGHELTPKSYSCRFVCISTYIIAVMIFGTYSATFISFLTVHDNKLP
FTNLRSLLLEGGYTFGVSSEHAQNYISKNSRDFLDKTFKGRHLDIHPTVLDGMQHVCNSK

HSFLIPLHTALAFKNKVDCNVEPLPDETSLFIPQGIAIAKNSPYKGLLNYNLNKLRANGVLF
KHKVEDYVRMFPKEKKTWTRVGLEEIAPVIGFLMAGIVAAAVVLLLEKITPAHLLQLQQR
DGVFCCLADVKHIGFIHETFNNHQPHRNQPGYQEFSQIGVLQVEQNLDRDNIHQSPELMV
>BgerIr141
MGILLQMILKVLLITTVQHSDALLGFFSFSSAIDILIDVRNHLHTGCVYLLHHSSTQTELSDF
YQVTKIGINLSTKGIPVSILDIQNIKKKQKCLRNLPIMVILSSTGQMQIALQKTIFLYIFKHSS
QKILRSSTWLLYLSTWSIEDYFKDVDIPFDCLFLVAEEHGNNSVRITEVYRIYPSLPLSFNTF
CTWKNGEGLSCSKYSMMMRRNTLQGLDMKIIVPNTTAVQDEGSKHLKEFFYTIWHDIAG
NINLNSLSTKRNDQTQGQNINGTWTGLIGDVTNGLAHLGVDPVGMKGPRTEVVDFTVPL
FTFKISMFLKEPDSALSWGRFLAPFGADLWLAVIASMLVLTLCLSLFHLIGRYTGNEEANG
PNTYSLYNSSLYVFGIFCQQGHEISPRSWSCRIIYWMTYLTAIILLASYSGTLVSFLAVKHHP
LPFNDIEGFLRDETYKTGMIRSYFQLFTKTKDIPISLQTPSQKLIRKVEKEKDNSPLLALDG
LKRLCKDKYALLITASVVPPLMDDVPCGVVSLPRFSIEIGMSYIIAKGSPYRSIINWKINALR
AGGILKRGFTDVDTTKFSKSHDGFMKVGMGEVVPILIMLGLGACLGCLILLAELYLHRWM
RRRRIRYVEYDDSMC
>BgerIr142
MKHAPWKNISLLLIMTTACQCQTQKSLSLLEATAVILGLQQYFHSGCVFFIHSAFTIHYELQ
DELRLLQLRKMLSRNNVATLAVAVETFQIPLGKMRCQKNRPVIVITNSGDTTKNALQQVA
EKPGFSQPIWLLLLNTETSISTYFSKIYVPVDSEFLVAQRPNEAIQLTEVYQVNKDYPLQLN
QFGEWSPNRGLIATKEPFCERRADLQGVTFKAITVSQHPYVFVTRFINGTSAVSGGFFGLV
WFEFEQKLNFKTDYTALDTVSSGTSTNETIADAFSKVMVEIRNKNYDVALAALRFTSERF
AIVDYSHLIHRSKFIIAIKPKDNEDEAWKHFFGPFQSTLWFGVLGCIVIIACCLSTFHYLGR
MLAYPGYEDPKSYTLFLSVFYVFTMFCQQGHDTTPKSCACRLVYWLGYVTALVILAAYSA
TLISFLTIQSTEAPVRTIKALAQDKTFKLGMLLIHLKIVREDPAYGISDEIYNNMIAPDPANI
PTTYLEGLQRVCKMKYAFMTSMEFVKQAGDVIGCNVAFLPEYSHLFNYAYVIRENSPYKRI
INFLITKMQAAGVLQKLKFESWAANVPEAQNAWNSVSVVQVSPMLFILMGGMLASIAFLL
MEMGFRRWKGQNEKRKDEWQMKFQKQFQSNQLLFHV
>BgerIr143
MAPNIIKFLITSVSIALRYCIAEDSNDLQDQVAVMTSLKQHFHSGCLFLIQSETTNFSLMEG
VLQTRLAKVLSERNVRSATVSCETMMTSPIHSIQCHKNRPIYTFTSVDNSTRRFLMNFKRI
SMRSSIWLLFMRNELSIEDFFANIYVTFDSEFLIARRESEETLLTEVYKVDEEWPLQKRLFG
LWSPQKGLDTVDQSFYIRRNDLQGFVMRGVTHETNQYTLLNYEGNQTKLKDGYFGFLW
NEFQEGLNFRTHFIQHKSTTDVNKRFSEMIIMIKNENLDVAVDAFPVSSTEFDYIDYTTPA
HNERFRILIRPQRKSDMGISTYLGPFSHRLWYVVGIFIILLASSLALFHHIGRIFISSEDFDWP
WRYSFSTSLFYVFGIFCQQGHDITPRSSGCRVVYWSAYLTSLVILAAYSGTLISFLTIHRDNF
PFNDLPGLLKDPTYKIGTLQKYMHYFQGNESGIMFEAYKKKMISKPIGSYVEGLKDVCEKN
MVFWASIDTSGLYMDNVACTILPIPKYGFDFNVAMVLAKNSPYRIFLDHMAALLRDSGILR
RKHQLEWFVRSPLAHDAWSSVSLEQVQPLLIIVAFGIILSSCIMGLEILLSRISRTNLHPSTT
NTITKQRLKRLPKYNTMSSFPVTNQLYYSKETNNDLFTLSMFQNK
>BgerIr144N
NKHFLIQIQKYLIQQSASSVIVSLNDMANRGYIKCYNNRPVNVLLSTSNETRRFLLQISTKL
HLSDAIWLLFSTFKWDDTKSLNIFFNDIYIPIDTQFLITYPQEEGQTHILFSEVYRVSKTRSL
RSNYFGTWTPNERIVETGVQFYERRNNLQGIVIKAASIMEPPVTILKRKEDNTYVVEGFFG
KIWRSLEKTMNFTTEYSKPADDAWGSLTKNGTWNGMIGMILRNEVEIAVAEFTMTALR
AGVVDFTIPLIDTRNCVLIRNPNAEEDISWLGFLEPFTSNLWIFVLTTIIVISLLLKLLYNLKL
WYIGQVPPETIDTYSIIYIIGAFCSQGLNIDSGHSSLRILYFSAHLTALVLSAAYSAQLISFLSIQ

TFSLPFNSLRELIDIGTYKLGVLANSGQLNNFNTANDSLMKEVYTKLIEPDIENLPVSIEEG
MQYICKYNKYAFMTSLDVVLGLLDHISCELMSVLGASIPESLAMSIKKRSPYRGLINFNLQA
MRRNGVLKRLRQEEWPTKLPLATSEWSSIDIYKTIPILFIIITGVIVGMFLLTLEWCRHVNR
KKRSRGNNRDFWSSCSYAIKQKMCQPGSNKVSNIQRTGRYQYLYKNNFMQN
>BgerIr145
MIDWRRWFSIAAMILFTSINGHSKDFNAQILPVIVEIITTVWRNPPSNHVVLHNFDQDLQ
KIHQLRELIKIMGHKEILAVSLHIDKMNIHLVEGNEIFVLVSVRKEVQQLFKNLSETHYSKP
TKWLFFVSDEGILDDLSTPINSHCIVLRRKNNSIVLTEKNNIHPQLPLQQREIRLALLKNSF
LTSWLNVGFFKKRDCLQNSTIKIVTALKRNNVNGKLSGFYGEVWSVLQEKLKFKENFLEP
RDGGSYGSQSMNGTWSGMMGMLVRNEADIAIGGFALTPERLEAIDFLPPMVEGSVTVYIR
QPDLDTFSMAWLLAPFNTNLWLVLLIIILCLSLELQATWKYGQQEFKNWIHSSWLHIFGS
FCQQGQPKTSQPASSTVLCVTAYLTAMVILSVYSATFTSFLAVQHVQMPFSSLEGLLEDGT
YQLGMLTRSATLNYFDKSNNSLMNEIYNKLIAPEKYSLPVTNEQGLTLICKRKKYAYMSPS
ITAEMEISKLPCRIIEVPNTDIPVSSSMVVNKHNPYRKLMDSYVQHMRRVGILSRIHKNIW
PTQSTGILENPSVYMAFASMAPLLSFLTIGIIVSIIILFIEYLRYKHVA
>BgerIr146
MHTRELKLWVSQPINDHYSSSMGPVLLLILIASVNAAVDLDIPALLTALQEHFHTSSVVVY
YGNTTGLEDQRQLKNFAKISKVPVGVFRQQKSKNLSHKASNSLYVILSPLHFGINILSEMFQ
DCHVDITWLLYFSTESYQYLLADVYIPLDCEVIVFQPDYTLSEVYHLQNRLEILSVGTWHD
TKGLVWTAKDFFQRRSDLKGITIKASTLNNPPITIVQEQNGTVMDVSGYFGDVWKVLEKE
MNFTTEYYLPLDGSFGAKRENGTWSGMIAMILNGEVELAMSEFTITPIRKEVLDFTMPLIF
TRFCVLIQQPSGIRSSWTSFLAPFSTNLWLTLLLVILLMSLHFAVSHQDFAINPGQKVIDAL
FQIFGIFCQQGQEETPQTLSGHILCMTTYLTAVILPAAYSAALTSFLTVQHTEMPFTTLPGL
LEIHSYQLGVIANSGQLNYFDQAKEPLMKKLNDELIHPHRNELPASVSEGLLRLCKQPKYA
FMASLDIAMALVNDFPCNVIPVTGASIPDTLAIAISKNSPYTQLINYNLEKMQKSGILARLRI
NAWPARISKSKPLWSEVGLETMQPVLCIVIVGILAAFWCLSIEYLINMRHTSIRYCFIKR
>BgerIr147I
MNLNYMSKTISTASMILYFCTQSVGISAKEEGESWLNSTVSTVISLKKRLNLGCLFVVHCAT
SDSVYDTEKQFQLGKGLTLQYQPIGIPDLDRMRNKIKCLKNRPLYIMLLINNTARECVQKV
SKKPQFSDGLWLIFLSSPTQEVFQSLPVPFNSFVVVAHYQRDSVLLEEVYRIAPHLPLIIRRV
GTWSKTTGGLSWTQQNVHSRRNSLRGMTLKAISDHTDWVAPAATLDGTEKISSRSSGML
HMISEQQVDAIVSDITITTEWEEIIDFTMPILSYKFNVYFRSLGKGEHLEWDTYTKPFNLEL
WISVFGTVIVLTILLSLIHQFGRENGNAEKMFPRLHYSLYEAFFYVLGAFSMQGGDVTPKA
SSCRVVYFLSFIIAVVLLAAYGGILISFIAIKHEELPFTNLEGILKFKKFQFGVIQNSTEFSFFK
DADKNSVQGRIFSTLIMKDPKNCPETDQEALERICSTKYAYMAPTEYVHHLMGSAKCEIQ
HYQEKYMSASLGIALTNNCEYKKLFNRNLQAMIDGGVLQKMWMDTWAAKPSPVEANW
EGVVVNHVTPLLAFFGISILLSLLFLLCERKLLLHRNTLPFSSEQTKQPIKKEWKPTPWMA
>BgerIr148
MHVGPNSGLKHFRTISIVVFLVFILRPSGNNAYAFLEEMVASVHEYFHSGCILMLQGGQQQ
PSLSAMTSSVRLQTLLKNVPVFAALPSRIGRLAVNCLPRLLLIVALSSNAEMLAEVLEDQRS
TPTAWLVLLERGTTLENVFDKVNVPFNLELLVAQPQGSHLVALTEVYRVESGGPLQTIHF
GSWTRLEGVVGPKTSFYGRRSNFQGLTFRVVGIMLSIGENSAGDIVGVSGHFGTLWKILEK
ELNFTSEFFFPKDNNYGTMVGNGSWNGMIGEVQRGEAHFCCSSILLTTARNGAVDFLSPL
VEVRMTFAIREPSEFVVTWREFLDPFTWQLWSAQVACMLLIGACFLAVSSFYGPKEGDAS
RRVSDAVFHVFSIFTLKGQSDAPASLSARTILWFSHFAATILLAGYSACLISHLTTKKVKMP
FSSLREFLESNIELGAIPFSALNDFFKNSSSPDMREVDARFISGHEASAPRSDSEGLRRVCQ

RSSYAYLTYLEVVSHAARGCDVGVVPGVFFPASLGFIANKRSPYRKIFNMKIHDLRISGVLQ
RLHQVFFPLRASSTDLPLPVVDFSDVKSIFAVLFAAVLASNAILLCERLGRSRM
>BgerIr149NI
MNVSEEETGGRGLQGFVFKAGVVLKSMTRKEEGFPGYTSGFIEAVWRELQAIHRFSTEYF
RPEDGTWGVILPNGSWNGMIREILSGRINVGVSDFLITSERVRVVDFLPPLINTMCTIYVTA
YSIASVMFSAYSAALVSILAVQTDVLPFTTFQGLLDDGTYKLGVLEGSAQISYLDRTEDSTL
RKVYEKLIAPQLSNMPSNVSVGLQRVCHNNKKYAFMVELANVWGLVRNLPCHVVRVPGT
EIPSVISIVLPKNFTYQHLLRQTVLQMRSAGILQRLHRMNIPAEKEMNDVHEVHLSLNSFLI
LLTLLGAGIVVSCIFLLVEILSYKLATLCARRRTFKLERKSRRFDLTRKIVTSRRVCAGQSRG
LYSKL
>BgerIr150C
MHLSISSILIKGIMVYVLLLEKYSCLELKERCRIVKDVIQQYQGQCVNLLKPKYEHDLWMQL
LTLKYFSEFAKDFFITSVIQDEWKPTKSMEICQPFVYTYSNWSCTQDILKMFTGSSYRSNV
KLMLFEDSLTNTKEFFENAYIPFDSELLVAKENSESSELTIHEFYHILPNTSLQTQCVATW
NSKNGLTWTSKPYSERRDNFHGTKINVGTMSFGKIIFVMETADGIKPVKIGGFISQMWDA
LSHRLNFTSDFCGVPDRAYGSRDANGTWNGLIGMVARREVDIGLMSFMMNTARWTVVD
HFAPIIESKYSIFIKKAQDEGSSIDQLIKPLSANLWFSSIIVMFLFSIFLTMTWFYSERKEEHK
IEPKFSLGTSIFYMFGIFCQRGLDTSPNSTPSRIVYINANMVAFVIFGAYSAILYAFLAVSKYE
IPIKDIKDLIEDGRYKLGLLANSAHFILFG
>BgerIr151N
FTETTYRANTKWIVFEDAIRNENEFLKNSYIPLDTEFLLAKQPPDTKDVEISELFHVLPNT
PFNSQTVGQWNLDTGLNWTNKSFSERRVDFHGSVIKATYAPFGKVLYFVENDKKLEFGG
FAFEIWNTLENTLNFTTTFQKPTTGRFGAQSANGSWDGVTGMLQSKKVDMSLAPLILMN
SRLSVIDYFVPVIRTRYQIYLKRPHTEEQMFDQVLKPLSGHIWFCLGLIMILISVLLTIAWLT
SERFSFHNAKTKHSLIDSLFHVFGIFCQSEWSIRLVIINTSVVGFFVYLAYSAMLFSFLAVRK
YEMPFKNLKELINDGRYKLGLIDNSGDFIFFAESKDPVIKEAYKKLVMGVKNHPKDPREGI
RRVCEEKRFAHLSLVVNEKHLIADSRARVRCSYVALPGFSVPFSVGNPCVKNSSLKRIINYY
ILKMSEGGLLKVFDERLIPLKLRKKALEDVGTVATMEKVSTIFIILVAGIISSIVVLFLEMCYA
RYGLSSNFEK
>BgerIr152F
MKGPPKEVYQCMVLNVAIWISVNAVDISTVIAITKQVVLKYNIEALYMLRSTNHPGKEYLS
VMKNFAVGFGVYASVRPVEEMSVRHEWVQHKPLYLIFQTNNVVRSYISKISRSGRLARSI
WLMFLEDSKTALEEFFNETYLPLDCGFLVVQGERRHLALIELYRVHFTRPLQMRHVANW
SSGSNLDWGQHSTLMRRNDLRDIVIRGVYVPEPPYISKEKEGYSGFCFDVWKELQSRTNF
RIEFHTPPEEISYGNLENGTWTGMVGDIIRDNADVGLNVFIISNSRLKVVDYLPPIFDTRMI
VHIRRPGLGRGTFQHLVILFSPTLWNATIVTCVLFALVMSATRNVATKYRGCLPDDGFKFK
DSWLYVIGIVCRQGHYTMPEPWSLRVLFLTIHVMLLVIFVAYTAQFISFLANRTPVLPFSTF
EDLLTDRTYSLGVIKNSAREAFFKNSVDPVLRGIFKKLIAPVQRRPNNDEDGLRILCADPQ
HGYFCSQISVRSLALKLPCSVAEIPEAYYSVSLAMTISKNSPYRRLLSYNLREMKRTGILKRI
EMLHWPKFADKLDYGAPSVSFGTVIIFYYLLLGGMLAAVAILIIEHIVRHLRRRTRRDAPKS
ISSILASEKRRQLKAKAVFPYGLNMDKVN
>BgerIr153I
MSQSGAFDMALMVRAYVILMSLSKVNNFDLTCMSRVAAEIIQKYQIQSVQFMFTTKTVTK
LENIRMHTFLVKLIQREKYISVNSINVYHDSTIEKRGQKQLTIISSSEQLNRHTLEEVLEKIK
VSHSALLLFLDVKRTVEEIFSDVFVPMNCEVLVAQGHAEVTLTKVHHIHPSRRPLQKIPLG
RWNALDGIKWANQVGQRQNDLKGAVIRGAFILEVIVHIKEPEVDNSNHDPLSPFTLRMW

ATVLGAMFLLALALTTIFSIRRPTVASQHLQECFLHIFGVFCQQMVIVAAYSATFVSFLAVT
RSHLPYTDFTDLLQDGGFKLGVRAKSSHEDYFKITQDQVLRQIYRNKILPQARPPTFMEGL
NRVCTQSKYGFVLAEVTFWGLQRDVPCKIVTVPKAYYAATTSFVFSKGSPYAAVFGREIQK
MRRAGIIERINNNFWPQRYQEDVQESSSKSADLEMMHPFFLLLGTATLLSIGLLLVEITFH
RMRKNKRDPVIDLI
>BgerIr154
MLFTEELFSIKNIFVVLALVQPLQVSTLTIEDKCRIARIVAQRYQSRCVFLLKPNDGPDMET
ELLSFKYLNQLSRMFMISSMIQDKWKNYLSRETCQPFLFAVHNISNIDAILEPFTESTYRAN
TKWLLFEDSIKKEENAFFNNTYVPLDAEFLLAQQSPANKEITVISEVFHILPNTTLRKQAV
GLWHPQKGLTWTNKSCSERRNDFQGNELKAAFMPFGKIIFIEEKGGKAMKFGGFLYQIW
ETLQNGLNFTTKIQRIAEGRFGVQDANGSWSGMTGMLQRKQIDIGLMTLMLSTSRLSVLD
YFVPVIKSSLSIFIKRARSEDNSLDQLLKPLSDALWVAVGCIVLLASALLTVAWLYADRGIH
RRGTETEYNLTNSLFYVFGIFCQSAHGLATNSWSTRLVIINSNALGFVVYGAYSAMLFSFLA
IRKYQIPFNDIKDLMDDGRYKLGLLTNSAHFLVFTETKDPIIMEAYKKLVLAEKSHPTSIEE
GLRRVCERKHYAYMMLKFFEKELRPGLHLSCNYVALPKFSAPTTLGIGCNKNSPLKRVINY
HIIKLHEGGLVKLFDERLIPLRFRAPPEEDVTIAASLEKVTTIFIVFLAGIVASIVILLVEICVY
RFKNVSAKIKGN
>BgerIr155P
MYLTSGFTFTQNRSVLFKIVLMVHIISGNVSSNKIEETCDVVKSLLQQYQAQCIFLLKPKDD
PDMRTELISFKFVGEFSKKFVVSSMIRDTWKNSTSREMCQPFLFAFYNVSHADLVLKPFT
ETTFRANTKWILFKEVIGNEEQWFNNSFIPPDTEFLLAQPSTDKAAMIISXVPNTTLRKQT
VGQWHPQKGLTWTNKSCSERRNNLQGNELRAAFLPYGKIIYILQQENGKIIKLGGFLPKV
WETLENGLNFTTDLQRIAHGGYGVPDANGSWNGMTGMLGNKSVDIGLMTLMLSNGRLN
VLDYFVPLITSRFLIHLKSGETEVNSLDQLLKPLSDGLWIGVGSVIALASILLTVTWFSSDGR
IHHSGGSDKFNLSNAILHVFGVFCQTGPGLASKFWPTRLVIINTYAIGFIVYVAYSATLFSFL
AVRKNEMPFKGIKDLMDDGRYKLGLFANSAHFLLFRETKDPIMKEAYKKLVLAEKSHPTS
IQDGLRRICDRKRYSYMMLEMFEEILRQDLGMSCSYVPLPQFALPAAMAIGCVKNSPLKRI
INYHIIKMYEGGLLRLYRDRLVPLQFRTREENDVAIVASLEKVTTIFIVFLTGLVTSILMLLA
EITLYRLKIKPEKTTV
>BgerIr156
MNLIVTLFVISISPFIQVVGMKTEDKCRIVKSILRRYQTTCVFLLNQDQGNDLESESLTFKYL
SEFSRDFIFVADKWRWHSTSREMCQPFVYAFHGVSQVDAILKTLTETSHRTNAKLVVFED
TIGNETQFFRDVYIPLDTEFLLVKKPNNNEEVAIDELYHVIPNTTLRTNEIGQWRPDKDKG
ILDWTTKSYSTRRNNFYGIELKAAFTPFVRMVYEVKNNDDKTIKLGGFVYEIWDILQEAL
NFKTHFQRVPEGRFGIEDDNGTWNGMAGMLQRKQVDIGLMALMLSTSRLRVVDNFHPV
KRSRFVVIIRRGTKDSTFDQIFKPLSRSVWISVVFTIILVSIFLEIIWSNSVQERRNATKCTIS
NSVLSVVATFSLRGPDWMPNAWPTRIVIISSSVIGVVAYSAMLVSILAMSRYEIPIRNIKQLT
DHGRYKLGLVQNSTAVVYFEDSRDPAMVKAYEKLVLTEKDHPQTIEDGLRRVCERNKYA
FITISQYHEQILPDLDLPCTQVYLPEVSFTTSHGIPCAKHSPFKRIINTYIIKMHERGLLEVQY
SRLRKNKLEEEEEEVEERAALSLETLSTMFILLFAGMASSTLILFLEICHKRLKKTK
>BgerIr157
MDTRNTFVVFIIGIVVIPMTSLRRTSSMEIEDKCKVVKSVLQQYQTQCVFFFKPEELQDWN
METELLSFKYFTEFSRNFYLSSMIHDKWKSPISREVCQPFVFAFSNISRLDAILEIFANSTYR
ANAKFIVFEDSSPSQDSFNNTYIPLDTEFLLARQLDKKDMSIDEFYHILPNTSLLAQTVGQ
WNSENGLNWTNKSYSQRRNNFQGTELKAVFYPFGKQLYIIENKDSKTIKFGGYNYQIWKT
LQNELNFTSNLHRVTEGGFGVRNATGSWNGVTGLLQRKDVHVSFITLVMSKSRLATFDYF

IPVIRTRDVIYLKRPEAEEYMLEQVLKPLSGHLWIGVGLVMILISILLTITWLTSGRICYQNT
KAKYSLSDSIFCVFGIFCQAGAEDVPNVWSIRLVIISACVIGYIVYLAYSAMLFSFLAVRKYE
MPFNNLKELIDDGRYKLGLIENTADFLFFKETNDPLLKEAYRKLVLAEPNHPTSAQEGLR
RICDQKRFAHMVLKVNERHLSADLDLRCSYVTLPRFSFSISLGFGCTKNSPLKRVFNYYILK
MSENGLLKIYDDRLVPLKFRDKAQEDDAMTVATLEKVTTIFFVIFAGIILSNALLLVEISYH
RCKFSAH
>BgerIr158P
MDTSKRFALFVIGIVIIPMMSFRRASSMKIEDKCKVVKSVLQQYQSQCVFFFKPEELQDRN
METELLSLKYFTEFSRNFYLSSMIHGEWKSPISREVCQPFVFAYSNISRLDAILEIFANSTYR
ANAKFLVFEDSSPSQNSFNNIYIPLDTEFLLARQQFDKKDMSIDEFYHILPNTSLLAQTVGQ
WNSERGLNWTEKSYSQRRNNFHGTELKAAFFPFVKIIYVLEYKDDKPIKFGGYVYHIWEA
LQHALNFTTDFVRVTKGGFGIRDANGSWNGITGMMEGKFVDVGLLTLALSASRLSVMDY
FVPVKTSRLLIFLKRDQTDNRLNQLLKPLSGGLWTAVGSVIILVSILLTITWLSSNIRKQISGI
RVKLTASSSLFYVFGIFCQTADAWPTRLVIINASVIAFIVYVAYSAMLFSFLAVSKYEIPFKN
LKQLTDDGRYKLGLLQNSADFLYFRESKDPVMMEAYKKLVLAEKSHPTGIDDGLRRICER
ERYAYMVMNLYQKQIRPDLGLRCSYVALPHFSIPASFGIACTKNSPLKRIINYYIIKAYEGGL
LKILYDRLEPLRFRAEVEDVTTVATMEKVATIFIILFTGIISSTVIILIEVCYHRWKIPCRPIAL
E
>BgerIr159
MDAKTSFILIIIVATAQHLGNVSSIGSEEKIRVVKSLLHRYQTQCVFLLKPQKDVGLETDLLS
VKYFSEFSKNFFVTAMIQDKWKNYLSREICQPFVFVFHKISQADPMLKTLTETTYRANTK
WILFEDTIQDLNHFFDKTYIPLDTEFLLAQKTSDDKTLVINEMYHIMPNTSLWIQNVGHW
HPDTGLNWTSETFSQRRNNFHGVEIKAVYMPFGKQFYIIENQDGKTIKFAGYNYQIWKTL
QNELNFTSNLHRVTEGGFGVRNATGSWNGVTGLLQRKDVDVSFLTLVMSKSRLSTLDYF
TPVIRTRDVIYLKRPEGEGQMFEQVLKPLSGHLWIGVGLFMILISILLTITWLTSERICYQNT
KAKYSLIDSIFCVFGIFCQAGAEDVPNVWSIRLVIINASVIGYIVYLAYSAMLFSFLAVRKYE
MPFHNLKELINDGRYKLGLIANSADFLFFTETNDPLLEEAYRKLVLAEPNHPTSAQEGLRR
ICDQKRFAHMVLTVNERHLSADLGLRCSYVTLPHFSFSISLGFGCTKNSPLKRVLNYYILK
MSESGLLKIYDDRLVPLKFRDKAQEDDAMAVATLEKVTTIFFVIFAGIILSNVLVLVEISYQ
RLKFSAH
>BgerIr160
MILHVMMIVLIALMNGHVSEFVAEAMANVAIGVFHKYHSTCIYLLSVSNREELSHWQRAT
LVQKSLSRRKITNLVISVENLGQFVRGTFCRPLIILLQPRAISEDLSHEISDALHASNVTWLL
FADNNEPIEEFFTKIQIPFNCIFLVAKIRTISSVSEVYHLPSLKSLQKVEVATWNMAHGFKW
SPALPFYERRGNLQGTVVKGAVIPYKPYLLSNSENSKEPRFSGYLMQLWKELERRMNFKT
EFIVLKDGVFGFLLENGSWNGAVGMVQREEADVGISFFAYLKDRLSAVDFLPPIWNVKLM
VHIKEPELEVSKFSHIMSPFVPSLWFIVVFCMVVFTALLSFTWYLRPKDQDSFDSTLYNIW
ESSLYVFGAFCQQGHDSTPRSPSCRLIYLMTYLLAVVMFVMYSAIFISFLTIKRYNLPFDDF
DGLLNDGSYEFGVLSGSARINYFKKSPDENLRRLYWKLLDPELPKQPVSDVDGLRRICNEK
KYSYLISGTTLRGLARNIPCSIVGVPHAYYTITASMIISKSSPYQRLFHHHVQEIRRSGILDRI
ERSSWPPKYEEILLDPPSSVSLETVTIFLIVLAIGILSAVAVLFLEIAAYKFRIWRLLKSKTPY
>BgerIr161NI
EIELHDMSRAKWLMFLDTRTTLEEFFENMYVPLDCELLAVQRSTTEDGGGDAISVSEVYH
THQAHDWNLQKHFQKHLLANWNTNSTVTWNNSTFYERRSDLKGHYLTAALIKQTVFKE
PFDDGWYGARDGNGSWNGVIGMIVDGNAEVGLNVLNYDTIRLDAVDYFPPLWNLKKML
YIRQPGLDTFHGEQLLRPFSDMIWWTIVISILTITFLLWSISQVKSLMQNNRENEIYSLYNS

AFYVLGLFCRQSPAIFPKSKALRCVFFTAAISAMILIVAYSDTIITHLTVHKFDLPFTDFKELL
QDGTYRVAIRKGSADKSYFQKSKDPVFNEIYEKLITPEIDKSPNTARSGLRRICKYKKYGFM
TSNTIFQGLQRQVPCNVIGVPHAYYSNTVSMVMHKASPYRRIFNYHIQELRRNGMLKRLM
RQTWPPKLKEIYIESPPAVSLEIVNAIFMLLFLGGAISFVILILECALSRALSRKHSSKTKTKK
MAQTYFEFMN
>BgerIr162N
VSKNRSISIGKWLMFMESKTNLDNFFQNINVPFNCEFLVAQQANDTIITISEVYHLSESTPL
QQNIVATWNNNEGLIWNSKHLFEHRGDLMGIQLTAAVGREPIITKDNRRKPTVLAGYTG
NVWKVIEQAVNASTDIYYSNDGKVGQLYPNGSATGLFQMVIKKDVQIGLETNPINPDYLQ
YVKFLYPTWRLRLSAFIKKPSLYDSPTDAVLAPFSVSMWLAVVASMVTFIAFLTGTWHMG
RKFAGGSTPQQESLSLYETWLYVFGLFCQQGQEVTPRSLSGRVVYLMTLCTALILYESYSAI
IISFLTIQKQDLPFTDFRTLIDNGHYKLGIAEHNAVIFEAATNPLLKETYEKMIMNSRNDLP
ADEPQGFTYLCQKDNYAYITAQFFHRVFRKSITCDLVEIPAVSIATYGSIIVDKSLPYIGLLN
KRLHDMTSYGIMKRISEDDSPSIVKDEPETELRAVTLGSVAPILSILILGVIAAIVLFLFEHLF
HRHSNRWQSEKFSRKIKVKNSQSVSNILTPSVFHLQFVTHFPEISEGNISKKSHKTLE
>BgerIr163
MKLLLILCALQVNSIDFSPESLIKVTVDVLANYHSTCLYILHSAPKLDYSQKHTNVEFMKNL
IKIKHLQIAVQSMKTLLVKNENVNCNEKLPFYIILTSEESVQLYLNLFSRRSIFTSGRWLMF
LGKNVQLEYYFSGIDFPYDCEFIIVQNTSQNILTLTEVYRHEIEESLHKYVLAEWFNNNLK
WTNLSLLRRRENLHGHQIRVSIFPEQEKYVKSNCNGFEKEEFCKAQYKIWRMIEREANAIS
VYISDVEETVDVGNKTWSGGIAQLILNKSEVAISPFGMTTERISVVNYISNAFPAKLTTYIRK
ESEGFSKWDHVIEPFSPSLWCTIFFSLIVFTILWSSTWYATVRMNISPESEEYTFENTWILV
IGCFCQQGHETTPQAWSCRLIFITAYFTYLIIISAYSAIFISFLTVEHHEIPFTTFQELLETESY
EFGTYGHELYVGMFEKATNPTLREVYERFLLHQVNLLPKESEDALRQVCERNEYAFAFVE
DDVEGMEPMCEILALPRTTLAMYASFIIRKNSPYHRFFNHHMENIRRFGFLQKINTMKTQ
LEEVQDVEPQAGLEDVLVMFVILIFGLLISLLCLAVEKYNFKYENLP
>BgerIr164
MLFPMISLMKMTARVLEKYHTICLYLLVSSYNPDYLQMRSSMDYVKNLIRWNNLQVTVIS
MQELLDRYKDMKCQENIPFYLIMNSDKNTQGYLRKFSSGSIFPSARWLMFLSNDVQIDHY
FKGIHIPHDCEYIVVQKESKSTVTLTEIYNLHPEDPLQKQTIAEWHNDSFKWTQVSLLKRR
GNLRGHQIRASIFDDEKVFLKSKCSGFEKESFCRQHYEIWNMIQRQANITTRYLPYSRNYT
NFGNDSWHVPIVKLIENEADVAVSSYGMTPERMREVSYISNAFPEKVKTYIKETDKISSLD
SFLEPFTMSLWCTIFISLVTLAALLSSTCWLKVRLKISSSEEDYSFNEAWMFVFGSFCRQGH
ENASIRRAWSSRLIFLTTHLTYLIVVAYYSAIVVSFLTVQHHYIPFSGYKGLLDIGTYDFGTY
RNKLYPGLFQRATNPAMRSLYEKFLAPRQGTLPEKPAQGLRLICKKKRYAFALVEEELDL
NKPTCGIMEVPQTTFYLFASFLIQKKSPYYRIFTHYLENLRRFGFLDKVNSMQSPRVQVTE
EHRTQADIENVLASFLILFAGCLISLVLLAAERCLFKC
>BgerIr165IC
MKLLLILYVVQAKALQFPMTILMKVTVAVLEKYHTICLYLLVSSYNSDYFQIRSSLDYVKNL
TRWNNLQVTVICMQKLLDSDKHMECQENLPFYLIMNSDNNTQGYLKKDNDRLKSKCSGF
EKEPFCRQYYEIWNLMQRQANTTNYTNLGNESWHRTIVKLIKNEADVAINSYGMTPERM
RVVSYISNAFPEKVKTYIKETDKISSWDSFLEPSTMSLWCTIFISLVTLTVLLSSTCWLKVRL
KISPSEEDYSFNEAWVFVFGSFCGQGHENTRIRRAWSSRLIFLTTDLTYLIVVACYSAIVVSF
LTVKHHYIPFSGYKGLLDIGTYDFGTYNTPLYPGLFQRATNPAMRSLYEKFLAPRKGTLPQ
KPAEALRKICQKKRYAFAFVEEELDLNKPTCGIMEVPQTTFYLYSSFLIQKKSPYYRIF
>BgerIr166C

MKLLLILYAVQVKALEFSMLAMISVTVGVLEKYHSTCLYLLDSTSNPDYFLKRTNMEYMK
TLTNRKNIQYTIMPMKMLLNTNVHYRCNENLPLFLILGNDDSVKSYLNMFSKKAILSRGR
WLMFLLKYDQLDSYFIDTNIPYNCEFIIVQNTSQKDLSLTEVYRQQPHQPLQKNILATSIEN
KLKWTEISLIERRGNLQGAPIRQAESTKRNCKVKNGEDYCKIYYPIWKIIQTKINATSKYLS
PPKNEFKSGNKIWPGVIGQLILDEADVAVELYGMSAERMAVVSYISNAFPKKVTTVIKREK
AEFSKWDTFLEPFSTGLWWTVFVSFLFLSITLSTTWRVTVRLNISPSGENYSFYNTWILVV
GCLCQQDSTPRAWSCRLVFLSAYLTYLIVVAAYSAVFISFLAVHRYYVPFSDFQTLIDAGTY
RFGTYRSTLYVGMFEYATNPTMKDVYEKLLKPEWDNLPEDSKSGIRRVCEESHYAFLMN
ADLVQISKPKECHVMEIAGASLSMPASFLISKRCPYYRLLSH
>BgerIr167
MKLLLVFYAFQVKVIGLSMATMMSVTADVLEKYHTTCLYILQSTPYPDYYALQRDMEYM
KSLIKWKNLQIAIIFMKTLLDKHEQGKCNENLPFFLIMSSEESVRTYLSKFSERSIFPSGRW
LMFLPMDVQIDSYFLDIDIPYNCEFTVVQNTPEKSYSLTEIYRQQPHQSLEKHILGKWTDK
HLQWTNLSLLERRKNLQEEEEFVLSNCSGFQKEEFCNKYYEIWSIMQKQTNSTSEYVMYS
YDEEYEAGNVNWSTAIGTLVSNQADVGLYSYGMTAERIRVVNYISNAFPTKVTTFIKRQR
AGFSKWDHILEPFSTSLWWTICFSLVTLPIILTNTWYVTVRYGISPRDEDYSFSNTWLLAIG
CFCQQGQETTPKAWSCRLVFLTAYLTYLILIAAYSAIFISFLAVQHYYVPFTDFQGLLDTGT
YRLGTYKGALYVGMFEFATNPAMRTIYEKMLVPDFDLLPEEEEEALRKICKDSNYAFAMN
SDALEEVVPSCDVMEIPGASFYLAGSLIISKTCPYHRIFNHHMENLRRFGILEKLGNLHGRP
EDVDPPNTQATMGDVISMFIILIGGIILSLICLALELVVSKYEYRP
>BgerIr168P
MRLLLILYVIQVKALDFSTIEISNITQNVLEKYHTTCLYILQQTKNQGYLPKREVEFVQFLN
KHRNLQIATISIEVLLNKESKQKCLSLIHIFSTRSIFPSGRWLMFLSKNIEKNSYFNGINIPYN
CEFTIVQRTPQDVLSLTEIYRNAPSQPLQKHILATSFDNRLQWTHQPLLERRSNLSGVTIR
VSVYPEEGMIINEQECRNTTRFILCLRDHEIWMNIARKTNVKSTFVPYTKKTVKVENKIW
TDAFAQLITNTTDVAISTYGMTPERFRVISYISKVSIQVTTFIKKQKASVSRWEAVLQPFST
TLWWVVFCSILILTAVLSSTWYLTVRMNMASKKEDYSLKNTWIVVIGSLCQQENTLSTGV
SNCLYDILNNICSIXNADNPAMRKLYEKYLVEYDSLPRGNMAGIRRLCQEERYAFTMGLKS
FTQLVPECDVIEVPRASFYIAMSFMIAKDSPYKRLFSH
>BgerIr169NI
MTPERFRVISYISNAFPQKVTTFIKKQKASVSRWEAVLQPFSTTLWWVVFCSILILTAVLSS
TWYLTVRMNMASKKEDYSLKNTWIVVIGSLCQQGYEHTPQKIPCRLVFLTAYMTYLIIFA
VYSTIFVSFLAVHIYQMPFTDFQGILDIGTYRFGTLHNKLYVSSFENADNPAMRKLYEKYLV
EYDSLPRGNMAGIRRLCQEEFIFIFSMENLRRFGIIQRTENTLSPLLKGTQTSSELPHTQAT
MEEVLPMFIILLAGFIFSVMSLAAEILRFKHECLFRLPI
>BgerIr170P
MKLLLILYVVQVTALDISMVTMISVTFGVLEKYHTSCLYIVHPTPNSDLEFIHKRKDLEFIH
LLRKRKNLQIAVISMKALLDNSEIKCNENLPLYLIMAGEELVQPYLKEFSIRSILPRGRWLIF
LSKNEKFDKYFSDIHIPYNCEFIVAQNTLQNVLSLSEVYRRDPGQSLRKNVIGTWHQDDIK
WTQTSLLQRRGNLKGGKKYLKHKCKANQRVAFCARDYEIWNAISRRTNATSTYKTYSEE
TVKIGNESWGEAIGQLIKNKADVAIHTYGMTPERFRVISFISNAFPQKVTTFIKKQKIHVSR
WGVILQPFSSRLWWTVFSSTIILTIALXYTMVNMNIFHTEEDYSFHNSWILVFGSLCQQGS
DRSPRAWPCRLVLLTIHLTYLILFAAYSAIFVSFLAVQTYHMPFTDFQGILDTRIYKFGTLQ
NKLYVGSFENADNPAMRKLYERFLVQTELLPKGNAAGVHQLXMESIRRFGLLDRINNVLS
AKLKNSQRSQDLRDPQATMEEVLPTFLILFAGIIFSLLCLLAEVILFKCRNDLTLPTEHSVN
V

>BgerIr171I
MEMILVNILLTLCAVQIKGLELSVDTMVNVTVGLLQRHHSVCLYLLISSSQADAFLWKSSS
YMKRLRESKDLQIATMSLPVLFKKTKPTWCQGNPPLYVLLQSDKATQAYLEKTEYIIPSDP
ATITIQNTTWKYPFTKLISGEADVAIGKYGMISERTFIVNYVAQAYKGRITTFVKKQGSASS
ELSQVLQPLSTGIWWTILSTFFVVTVVLTLTWNIAVGMGFSQKMEDYSIYSSWIVVLGSFC
QQGTDFAPQAWSCRVVFLTVYVTYYVIFSAYSAVFISFLAVQRYNMPFTNFKELLDTGTYR
LGVIQSAIYISIFEKASDPVLRKVYTEFIRPNLNSLPKDHIEGMRRMCQERNYAFTIVNYRIY
QTKMLCEAVEIPRAYFVVSSSFMLNKDCPYKRVLGHHVENLRRTGILKRIESKEYVHLQHS
QTVRMQATMNDVLPILYILLVGYIISIVCLCVELLRLHIGKSQLQTK
>BgerIr172NI
MKRLRESKDLQIATMSLPMLFKKTKPTWCQGNPPLYVLLQSDKATQAYLEKISRSLLSWG
RWLIFLSGDVSLETFFKDIYIPYECEFTAAQVDSRNKDRLLLTEVYKHKPQERLQKYVMA
MWSNEGFNWTEEVLIRRRGNLQNVTLKVAVKPKEKDLARTKCMDSREEFCVAVYEIWS
ELERRTNFRSEYIIPSDPATITIQNTTWKYPFTKLISGEADVAIGKYGMISERTFIVNYVAQA
YKGRITTFVKKQGSASSELSQVLQPLSTGIWWTILSTFFVVTVVLTLTWNIAV
TDFAPQAWSCRVVFLTVYVTYYVIFSAYSAVFISFLAVQRYNMAFTNFKELLDTGTYRLGV
IQSAIYISIFEKASDPVLRKVYTEFIRPNLNSLPKDHIEGMRRMCQERNYAFTIVNYRIYQTK
MLCEAVEIPRAYFVVSSSFMLNKDCPYKRVLGHHVENLRRTGILKRIESKEYVHLQHSQTV
RMQATMNDVLPIIFILLVGYIISIVCLLGELLRLHIGKSQLQTK
>BgerIr173
MILMMALLTLSAIIQAKGLELSMESIAEVTFGVLKHRHSSCIYLLQEDNSIWDTITYVKLLR
ELRFLQISVTSISSQLIKDSIAFCQNAPFYVLLHPQNTTTQYLKQIPRHLFSGSRWLIFLPAE
DISIDTFFVDVHIPYDSEITVAQHNPQNGKRLVLTEVYRHHPGACLQKHQVAIWSNEGLN
WMNKMLPRRKGNLQGVHLKVAVFPQEEVAAKAKCKDYQDEFCVIYYEIWNALQKRTNM
RSEFVSHPDGVKYIVPNASWNGPIALLISGQADVAINEYGMTPERIIVIDYIGETFLGKIKTF
VRKRVSATSNRSYMLEPFSTGLWWTIWSSLMILIMVLTLSWTASVRLGYSQNLEDYSIYSS
WIVVFGIFCQQGQEITPKNWSCRLVILTAYVTYYVTFLAYSAVFISFLAVQHYSLPFNNFKG
LLDTGTYRLGVIGTAIYLNIFENASDPVLRKLYANFLGPNLNSLPRTDVEGMNRVCEERNY
AFTVVDYSIYRTRLQCEVIEIPRAGFSIPASFLISKNNPYKRVLTHHVEELRRTGILKRNQNI
LIDKTQNFDFKRTEATFDEVLPIIYILLVGYVVSILCLCTEVIILKISKPTPNIIHFYN
>BgerIr174
MKLLILQFLTAKALQFTLKTAMDIILGALNEYHSKCVYILNSYEHGWLQDDKMAYMKYFA
KSGNIPIASISFTELLLQNGGSYYQYNRPLNVITSHDNTTKTYLTQFSSRKSMCSSKWLMFL
QANLSLDDFFIGVNIPHDCEFIVVQRLNHFENAELVLSLTEIYRQYYHHKLQKNIVATWAR
GRFNWREHSLLKRSNLQGEEDAFRSKCFGFEDKSFCILYYELWRIIEYVANIKSEYLIASNE
YPGHKIGNRTWTDAMGLMISGKADIGLNYWGMTSERAAVASYISTAFVTKLTIYVKKEPE
DSSRWNHILQPFSTWFWWTVVVSFGTLTLVLCCTGYVYDQMGVANRREDLSISSAWLPI
VGILCQQGYTNTPTTWSGRLVYLTALFTYIIIFTAYSAIFISILTVQRYRLPFHNFQELLDLDS
YNFGTIGNAMYLGIFQDASDPVLRKIYHKMLAPNVKSLPGVSYGDGLQRVCVEKRFAFTAS
EESVFAQKLSCNVLSIPETAINFPVSIMISKTSPYKRLLSHIVETLRRTGILSRIHSKSYWKTE
DPEFPKTQARFVDVLPIILILSGGICASILCLIIERTIYYSY
>BgerIr175
MKLVFILNIMCIKASEFPLDIMTSVTAGVLDRYNTACLYLIHSSSQSDTNVIYLKHLMGYRY
VQFAMTSIPALLKRSNSDRCQANRPFYVIFNATDETRTYLKEISESALYPMGRWLMFLPSV
ISTDNFFTDLNIPYDCEFIVVQQLNNSEDQQILLTELFRFSPNLPLQKHVVASWSKKKFTW
TQTSLLKRRGNLQDVTLRAAISQEVYYDFEEEFDPASSCQQQFTYFCVITYEIWNMIETLT

NATVKYVMYEDGNFSYTIGDTTWEDGIAYVLSGRADMALDSYGMTQDRIPYVSYISNLFP
GKITTFVKKYGTESSLWWRILQPFSPGFWWTTLSALIILTLLMSNTWHLNVRLGISHALE
NFGFKDSWVFVIGIFCQQGHDSTPSALSCRLVYLTAYFTCIIIFASYSAILFSILAVQHYSPPF
TDFHSLLTAGFRLGTLAGNLYVGIFGKSSDPAMRKIYDLFLKPNLHSLPKTDEEGFIRLCKE
DKYAFMSGGYNLYEDKPCEILEVSRASVDMPASYIIGKYNPYYRLLTHQIEHLRRTGLLEK
VLNADEDFTSDTKLQDTQASVADVLPIFCILLVGSIASIFCLAAEILISRYKPKKKQSRFHAR
DFAKPNAVHCKPHRGCPKPRKPDKTSSLFVRFGTNKQDNGVFYGNDSVRQRTRTFQDA
WLK
>BgerIr176
MFEKMELKLLLMLYFVHGKTLELSVNKMVNMTVSVLEHYHSTCVYLLQSTQQTDYLLKS
LSTIYVNKLVKLKDLQIAVMSISTLLAKTEVNSCQENLPFYLLFSNDNSTFEKLKQISNKGIF
SEARWLMFLATDINVTLDSLFTDINIPYNCEFLVIHPQHDEHEVVTLVLTEVYKYNLNQTL
QTNVLAKWTNEQFLWSTTSLLKRRENLQGAILREEAKVKLKCKNFNGEFCGQLYEMWKI
LETRLNLQYEYLTYSEDTLNFNSGNKTWQGAIGMLETNEADVSFDAYGVTAERLGSTNYV
SNLFAAKLVIFVKKQISGGSMWWRILQPFSIGFWWTVLSVLATLALILSNTWYITVHFGIS
QQSEGYSIYNSWVVVLGIFCQQGHDSTPSAMSCRLVYLTTYLTCIVIFASYNAIYVSLLAVQ
HYSVPFREFKGLLDIKTYHFGVEGTMINVGIFEKATDPVMRKIYDILIAPNSKSLPKNELEG
LQRICKETNYAFLTEQDSGNIYAKLLPCEIMEIPRASIVMPVSFPISKRCPYKRLFTHHMEK
LRRAGILQRIENDDISKAKDNKFTHSQAMVEDVLPILWILLLGILLSILCIIGEFLLHYFTSHK
GHTVEQNQRHYTWRQI
>BgerIr177
MELGSTIFQIGFLFSSINCLNRESDHPAEIITSVARHFQLKWVYILQHPGIDFEEVLFVTRLQ
SQLSNNHVFTTIGSSLSINQRGLCVIFENQVTEIISKVTLNYNLTWLLFHTSDLLENAFSKV
KIPFDCEFLVAKLEKDTVVISELYHSNSSLPVQYHHYGSWTRNEGLKTTRKALFQRRNSLQ
GVTFHGGIKEGPATIVSKYENSQISEIGGFIGEVWNHLMRTMKFKTKYVMAVDDGFGVQI
NGSWTGLLGMVERGEIDVITTDILMNQNRFNVVTFLDPLFEVRTYMFIQNPQNNRRDWA
NFLKPFERELWLAIVGTIIGCALFLCGTVTYQCSMNRNVDISFKPSFNSFLFYVYFAFNQQA
QDLWRHLSTSSRVVMLTSYLTTLILYVGYSGFLISYLALKMNEFPFMNFEEFLKDGSYELG
VLSNSIHYTYFKESTDHIMRLIFKKFIFPNRNNHPMSFIDGLHRVCNRKRYAFMAPVFIASS
FIKQLNCKVVEVPAAFIPSTATMAMTKNTPYKSLFSHQIKQMRFTGVLKKLQGSFILDHLH
EVETDYEVVDIEAVKPFFWFLALGIGTATAILAIEILRRGNDKIKFVIISKSRH
>BgerIr178N
ICLSKWLIFLDSEISLQIYFRNMNVLMSCEMLVAQFQEDTIILTDVYRIGPAEPLIFHYYGN
WTHDHGLKKDSETLIFERSDLNGVSINVTYINQEQLFTLTESKDSAAPKLDGYFGFIWTIL
ERRLNFTTVYLPSNDGYYGSFLENGTFNGMVNMIQNGLAHGTSCNVMMNSKRAEVVDFI
APLLEERTYVFIKEPDSIPMKWTDFIRPFSVELRCLVVGIYIILSFVLWFTQVVDKWRKRDY
RINDAMFAVFATFCTQKKIRGPCRIVFFTSHLTALVLMAGYSASLISTLASREISLPFHDFQ
GLLEDGSYKVGVLTNSAQFDNIKLMSELAIRKVYWKLMAPYVDSMPLNDEEAFQRVCSEK
FAYLSEFLETVGTKQPCRVTPVPQASIPATLSMAIAKNSPYLRLFNHLIQRMRRAGILKKLK
LQILD
>BgerIr179NI
LDSLILWRNAARVLDVGSLSLELLLGQRRHLCASPLYIIAEEQQRILNEVSVAEMFSKAKWI
VFDQTESQLKHLNISFDCEFLLANWDDGVVRLHELYRISPEYPLLVKEFGDSTFGTTSWPK
AGLYRRRRSLDGHVLKAAIQDDGIFITVIQSNEGERLGGYIGEIWCELERQMNFSTNVYIKK
PDKYLLPWSNTLAPFGPRLWICVITTMLLLTIFLSTTCAVINGLKIHDEEEFRLQNSWLYV
FGIFCQQGHDVTPRSISGRIVYFTSYLCAVVLLAGYAGNYISFLSAGRPLVLPFRNFREMLQ

DGTYKLGTKPKSAQLDFFRNATDPLFKEVYEKLIDTDDLPADYDVGFQRVCESKYAFMTS
DALMKKYAAQGSCEVIDIPQASIPGVTAMAVAKKSPYLGLINFNIQRLKDAALVKLFRERT
LKKREDVEHRDWDNISLDEVIPNFSVFILGVLVSVIVLIFECFISTLVQRRNVFKRSSESAFE
EKKTFRKNCVY
>BgerIr180N
MVSAVMRNEAEVGVGAFGVTAIRSTVVDYFTPILNTRSSLFFRKPTDLALDMKSYFKPFS
MDIWMAILLTMILLTICLTIARCLESHYESEETDFVSKLFLQESAFCVFASFCQQGSEIIPRYI
SGRTVILTSSLTSLVLLASYSAYLVSDLATRISVLPFNNFREFLADSGNKLGIGRVSFEMNYF
SESHEPVSKEIYRKLLLLTKDDLPESTLEGLQRICDSHKYAYMVTEHFAQKYFGNITCKLET
MKGDYIPFSLSMIVKKKFPYKGIINYNLEFMRRSGILKRLYELHYQTDRPLKYQKWMDAGI
ETVAPLLLFVSVGLFCSVLVLGIERIRWTTKNNKNFHFILNMSKNTSRKSSSLPKNGLRKE
VNESEFGNLC
>BgerIr181NC
MTLRQPYCNRETLQTFPVGKMCHSTSFDKRALRVEDWSLSGAWLIFVEENGSFRDIFSGI
TVPLNCEVLLAKMDSPVSLSEVYHVLHDTLEVFRVASWSPSGGLHWARGPIEPFVEITEYR
DNKPIVLSGFAMNLWHELERRMNFTTSYSMPEDKIWGTVDESGEWNGMVRMLLRKEA
EVATGLFSYRHDRMDAVKYFPTFWTSKIMVYIKEPGMEDSNRNSILQPFEFKLWLTSIGTI
FGLTFTLLLTNCITTNSANRAQRGYLSFNGFFGVLASFCQQGQDIASKMWAHKMVLLTSY
VTALLLYVAYSAIFISFLSVRRHDLPFVDFQGLLSDGSYALDIVEGTADRQYFQ
>BgerIr182N
ISVAIYVSQFLGVCSENSYILGTVAALQRHFAAKCIYFVQISEDDIKFRCVFSEHWLLFLQQD
IILKEFFSDINIPFNCEFLVAQMSDEKVDVNEVYREGPHRPLKIVNFGSWIPKYETNTFTTA
SFQNRRQSMNGYTLDVFAIQFPPFTTVINQEGHLRMGGGFGEVWNNIQTLGNFTTNITLL
SGVTFGIQEENGTWTGMVGMLEQGQADAIIAGFSMTSPRTYVLSYTRPILYTKFCLLIGQS
EKTIHLENFLSPLSSKLWFAVIASIFLYSIMFATSYYTAYKFNDTDGKKPTLYAIFFDIASTY
CMQGGIITSNIWSCRLIQVTAYAAAVNVLAAYSGSLISYLATQKEQLPFNSFEELLKNGMYK
VGVRYGTAEMSNMKDAENDIMAQLYEKLLLPDPANIVADYEQAINRMCSMKYAFWGPV
EPTLEQAKEMTTCKIQVVRGCCTGATMAIPLLKDSPYSFLIRYHFKSLMYNGVLNKVKKL
MTPTTDPATETEVLMEVDMMDVAPLMLILLIGFTSASILLIFEISIPLFRIKQPRSRNMNGF
TIVNITSKTDRSYQNNINQFSGMPIFNRTACI
>BgerIr183N
ISTLLVHKYRKSLLHMEDNMTAGEIMKHFHIPLDSEVLLMQRRNDKVDLTEFYHVAGKM
HIKHFGTWSHSKGLQGPNRAVYNRRSDFLGITFKTTSVQGPPMAVLPKGNSKIGGYFGEV
WNILERHLNFTTDLIVPLDKTYGTVKNGSWNGMISLLFNGTAEAGVGDFTISSVRAEVVD
LTIPLIEAISVIYIRRHIRPDVDWTNFLAPFSLKLWLVVLITILIMPASFIVFQPDVGFFNSLH
YVVGIFCQQGIKSAPNRCILLGIALTAIVLYGAYSANLISTLAVQKHALPFTSLEQFLQRSDY
QIHVINTTALYSELQEASDPVLKQLYERLSFTKDLPLISLAGLQKVCNQDKFAFVTSTLIAA
VMIKNLTCLVEAVPGTLMKEYLALALAKNSPFRGIINHNLLILQQSGILKRLHEKTWPQVII
PEVSWRSVDAMDLASLAALLCFGTILSLLVLVVEFFWKNIQ
>BgerIr184N
TAQTMTLEVMKFLTSRNIPTAVLLSSSRLMPVQANFCNPLRVLLSTDSHLYKMFENYKLK
GVIWLAFLEADQTVEQLLGNTYMRYDILFLVAKYDGTDVHIYEVYHVKHGGPLYIQKFGV
YRDKLITTVFDSMYMRRQSLGGHIIKTVGINEYPVVTVPVPQGNKIGGFYGKVWEVLQKSL
NFSIDVIVTEDNKWGSVVIGANGSWTGAMGFLVRHEAEAGVCGLAMTGVRAEVVSFTIPI
AIFHDRMLIQAEDTKIQSSWKNYFRIFSLTLWFTIAIFIVIIAIIFKVCYHIGSLHGIEETTIEIP
IREAIFNVLGAICQQGQASTPLSANCRFVFIMSYILSLVLVCAYSATVISFLTVKDVNLPFSNL

EELYMDDDFRLGVLNASAKFMKFQNAKTGILKDVYTKKLKPEELQLPNTVHEGLIKICST
KKYSFLTPLEKATVFFNKVPCHIFTIPYDISVLPMSIAFVKNSSFIGLFQYNLQRLRETGILR
KLHIQELYSALNPSQEAVDLRSVTVYDIVPVITLLFGGCLISIFLCIIEKIYHVTFTIKKENIFSL
ILSQEPIKNQKIKFLSSSMDHISPDYTNQDWKRKISWKVRKFKVKK
>BgerIr185P
MEVYRVHSAFPLEIHRIGNWTCTHGLQWTRISLCERRNDLQGLKLNITTSNIDPVVKVYSP
GNNMHSVGGFFGVIWCTLEEKLNFSSRFLEPQSDWGININGSWSGMIGIVTRKEADVGLS
AFSMTTDRLSVVDSLPQMLPSRNIVYIQEPKTKKLNWINFFLPFRWPLWICIFFTLVLLAIC
LTIASRLEHVNGTRELSTYTLMDSFLYVLGSFCGQGQNATPTAWTCRIVYLLSLLVGIIILAA
YSAALISFLTVQRPDLPFETLGEFLSDGSYNIFIPNGSILQDFQTSPDRTMRSIYENWVTAE
KIRDIPILERFKQVCDDPKYAFIASEYIIEAFKRWEYVKCKIIALPETGTTDILTIIVQKNSPY
GRILSH
>BgerIr186
MTTLWCYALAICFVEVYGMTVESRAYIVAALNKYMSSSCVLLPLIGKQDINRETDVIMMQ
ASLSQVGVPTLAVTRETNKTKNCTTSKPFFLFLDGGIMEHNFLKRLFTYKFIYVMQISSNT
LSKSMWLIFLDGGEGRIKDFFSDIYIPLNCLFLVVQEGYGNLMKLYEVYRVAEYLPLIVQAI
QKDSSSNMYLRRNKLHGLVFRNIVRPFIHLVQINRGVNNTVKKVTGYFGELWKELQEHLK
FSTQYIAPPNLSFGGVTPNGSWDGLIGFITRGSADVSSNGFQHTRDRISAVSFLNPILETKM
YVYIKKPRVSTNWDNFLSPFATIIWFIILVTIFIIAAFLSAAWYIVTRSPKIPFWFIAYEAIFRI
FGNFCNQGLDMQISSSGCRAIIVTSHLTTTVLLAAYSAALISSLTVQDMKLPFSTYNDLLKY
REYTLSTTAGTAMLTYFQDSPTPLLKQVYKKMLEPHLNTMPHTGHVGLKRICNRYKYAF
FTTDQEVHWSYGKTDCDIIKLPGTSFKSQVGMIIAKNSPYKDILDYYIKQFMRTGVLMRLR
KKYEPRKPEKLDPEYQELDILAMFPLLVVVLSGAVFSILVLTIELLAHRFLRRRRKTNFKEE
KVKYELKTEVKKQSIEQKILDKKIKASPRIKRPSFHSVWVDTLELLTLDLGPRIRAEHNPIIE
PHS
>BgerIr187
MTTLWCCALAICFVEVYGMTVESRAYIVAALNKYMSFSCVLLPLIGKQDINREMDVIMIQA
SLSQVGIATLVVTRETNKTKNCRTSKPFYMFLEGGTVEHNFLKRISSNTLSKSMWLIFLDG
GEARIKDFFSDIYIPLNCLFFVVQEGDGNIMKLYEVYRVAEYLPLIVQAIQEDSSSNMYLRR
NKLHGLVFRNIVLPFIHLVQITKGVNSTIKKVTGYFGEMWKELQDHLQFSTQYIAPSNLSF
GGVTPNGSWDGLIGFITRGSADVSSNGFQHTRDRISAVSFLNPILETKMYVYIKKPRVSTN
WDNFLTPFDTIIWFIILVIIVITAAFLSAVWYIVTRSPKIPFWLITYEAIFRIFGNFCNQGLDM
QISSSGFRAIIVTSHLTTTVLLAAYSAALISSLTFQDVKLPFSTYNDLLKYREYTLSTTSGTE
MLTYFQDSPRPLLQQVYKKMLEPHLNTMPHTGHVGLKRICSRYKYAFFTTDQEVHWSYG
KTTCDIIKLPRTSFKSQVGMIIAKNSPYQDILDYYIQQLMRIGVLKRLRKKYEPQKPENIDPE
YQELDILAMFPLLVVLLTGAVLSILLLIIELLAHRFHRRRRKTNFKEDKIKYELKTEVKKESI
QQKLLDKNCSIA
>BgerIr188NIC
VNTSHWTKKFASNEIWVFFLMKNEPIGEFFEAVDIPADCEVFVGQKEGELIEFSELYRSHH
SLPLEMTKKGSWSEDQGLQWANILHYKRRNNLRGLPLRIVITD
VHVGNINQWGKKLSYESWNGMVGVLAMNEADVALAGFSMRPSRLDVVDFLIPLANEKL
QNLTPKTWSCRLVYLTCYIGGVVLISAYSGFLTSFFTIQRPQLPFQTYQEILKEGTHKLLIHT
GTEMQYFLNLSLQNSTDDVMNEIYKHFIFGKSDIFPKTAREGINLVCSNQINIFLTNRHIVA
NQKDVPFKLMALTRTAIPASQGIIIRKNMPYKRIIDQ
>BgerIr189NC

MAEWLFFQSEVSPLENLFNQQYISIDVEFIVAQWHSGGIVKLKEVYHIEPNSPLQIVTVGS
WDPQYGLHWKTPGFYKRRINLNKLVLRVAVSENDLPTKLFRDKTGKITGVGGFLGKIWT
ELESRLNFRYVSRYYESIEDERGIKMKDGSWVGMIGYIDRKEVDVAIGALTMYPSCLNVTD
FLIPLLDDNTNMYIRELETLELHWSSFLSPFKRRLWLSVIAALILLTGALIFSLHMGKSSTQ
TFNFQNVLESFYCIYGAFCGQGRESIPKSWPSRIVFLTSFLIGYVLVAAYSAALVSFLTVQRS
ELPFTSFRGLLQDGTYKLGVTVGAEISYFSESKSPVMQKLYNELIVTKKNHFHENILEGFER
LCEEDKYALVTSDMWLLRLRSGDVPCNIQKVPHTKIPGSVSIAITKENPFRNLFNH
>BgerIr190NIC
LSSRFSMSIGRWILFVNIPMDKNFLKSLKIPVDCEFLIAQQENDMETTLDEVYYTNSNEQLI
THQLAILNSNKTIQVKWSSLSLYQRRDFYGSVLKAGKLFG
TRYFKPTDGIYGHISPDNSLHGILKMVNSTEVDVSLEPFDLNTKLSDVIEFLPPIWKSDLAT
YIKRPSTVQSKPGLLLAPFGASLWWCIILTLLILTLGLGIIFYVIHRCDIQDGYGFHNSWFIIT
SIFCQQGYETVLLSCSCRLLLITTLLVALIIFNAYSAVIISFLSVAKPQMPFTSFKELMDDGTY
KFEIVAKSTLYTSFEKATDPLLHEVYAKLIKSNQHKLPTNTGQGLKHLCQLEKVAFMTHSS
LFEIHLQLWPEACDLIRVPKVTLIGYNSFIISQTSPYRKFLKQ
>BgerIr192NC
NSFNLKDMIDIQKGLSIMNAQTTVLAISKFVSTMKILHCNNNSPIYIFLCSETVARPVLSQV
GTDLWGPIWLLLLPPGDTPVEGSLSGVDVPIGCEFLIAWKPREQSDYHIIEVFRVTNEGPL
QTNQYGKWLLNSGPQGWPDLELWKRRNDLKGHTFQATTIQTYEETEIGGSIGQLWKLLE
HQMNFKTRVISPLDKAWGSVDHEGQWNGMVAMLQKREVDVAVDSLIMTSDRSLVMDF
TTPILLTRYYMYVKEPRTLHRDWFSHIRPFSTQLWMIILLMMTIVACSLHLHHTLFRSTFS
AIKKLSKESLILRMFSRRLMMVTTFLTYVVLVAAYSGTLTSFLTIQKLKMPFTNFWELLES
GFVMDVVRKSAEYNYFTNSKNELIKNMYNKMLVPHEELLPPDEKVGLQTVCVKDKRVLI
ANSVFGDEAIAEANCSLISIPADMVKGTMSLGLVKDSPYRGITNY
>BgerIr193
MQLVSLLCVLVSIPLRVDSFSVEALTDVIISVQRKYTGCVFLVGSDILFDDQYHRIYRALSNA
GLSTTLLLTRTLPSRVPRMEKKCNHNTPLYAILSEDDALDAVLTQLFSNGTVSDIRALIFLR
KGDVTSFFEGLYIPPSSDILVAKKSDSVVEISEVYAVGVGRALKVNCVATWGPAASEIGWT
REGFLERRQDLEGLTLDAGITSYGIGYERVSALKGGREIETLWYFAECWKSLEMHFNFTYL
QTKLFYPTDGGIGSEDNGQWNGLVGLLKRHEIEVAVVPMSFTSSRAQVINVLTPVFLTKTI
MSIRVPKSFDNPWDLIRAPFNTALWTAIALLLLILGAQLWTTLHMGRRHGNESAVQVYPF
LYTFGLFCQNSEDVPFRSWASKCVYFSAIWFSLIIHVAYASMIIAFLTVHRTKLPFNSFEGL
LRDGTYSLGLREKTASWDYFRTSEDPVMKSLYEKLLKPQEDPPKTYQEGYSRICSRENYGY
MSPQLVTPQVTCNIIKVPGVSYTVQVAYAIRKGSPYRRLLNQGVVYMKQNGLINRILSKYYS
HRKTHEEEGQWRSVSLENVQLFFVILLGGVCLSFVMLLLEKC
>BgerIr194N
SMAHPKVDIPIWLLFLEEDHSLEDFFRDLHVPFNSKFLVAQVAGNDAYLTEVYRVSSRHP
LSETNLGVWRDGRLSMQLTNGKEDRNDLQDLTMTAAIMNSPPYTSVEEENGAIDLGGSIA
PVWKMLEKRLNFSTNYTFSPDGMSGTSIREGVWNGVIGRLQRQEVDVAVSALTITPARAF
IKKPGNLDVDWGNFWAPFEISLWVAVLIIIFCVSVVVEVCHRLNRRFGDGYLEKQQQTFFC
DSFLYVFGAFCSQGQDMTLRCMSVRTGFLVAHLTAMVILAAYSAGLISSLTIETPNLPFTNF
QEILETSTYKIGVTNSSSGEGVFRYSNSSLMREIYTKLMAPDPGNFPATALEGIRRACSMKF
VFVAQVARVKAVIDKAPCRVLDLPATDIDVSLSIAFAKGSPYRSIINKHLQAMRLGGLLHKL
QPEDRREAVAGESEIVNMGVKTDFESVNLGDAVPLLTVWCISILLGMSFLQLEQKARALLT
KRSYEPDDVEGYFRRKGI
>BgerIr195N

SVQILKDLSERGLSVSVPPWSDNTTSIGNGLMRTGHSVTIGAVLDISCISKGTLLMALFIKIL
QIHWLLLFTDQFKWNHLLRSTAQDTNQLNLSTSSVTVAMLDCYPLIPLFKINSLDKVKQV
NQSNQLRGYWSSKYGLKLVSNTLANANDNMPSLSGAILNVTAVSPFTAHQNRQMYDSAL
LQLLMEILDFRLQEVESRGRFRKSTDLWLLQQLMSGHIDISASSILVTHDRVGLAEYTIAVE
KFIPQLYYKVQSVRAARNIYILPFSIRVWATLCSLLLAITIALTFILRRETKLRQQRIEISEPD
QSTWEFSEVFLVTIGAVCQQGSDRNPTTSAARTLFIVLFALAVLTYTAYSASIISLLSSPSSV
TSTLQGILRHGSKMGLALLNIHYYHSYFKNEDNFVSRKLHQIEVSPSFLELPDGLESTVKGE
VAFCADKVEAHTYLHHSHQSEDTLCSIGEIPLLKGPEYQRAFALPLNSPYTKTLNYGLLRL
MQFGLLARERRMWFEVRTLCDSPEPPPGTSYLSIALDDIYPALYLISFGLACACALVPLESI
FHFCLRIYRKKYKTRKTAHNLIARQRVMRMMYNTQRHQQTRSVWWISRAWQRLLRSLT
NEPFPFVH
>BgerIr196N
RMLIYRAEGLPLTQDVFVLTFTGSLWLVCLAIAIILLMACFRFASLQTSKYLEGRPMPWTW
IEITLWAVAATCQQGFSKEPEGLSCKVVFLIGYLASYLLYTWFAAGVTSLLAVQGKDTHLQ
LEDVARMGIDFCAAWDGMLPDYFLNSPDPAAQALLTNQLRKERNKVHSLELMFWQLQG
DNKVAMASEHPLKHYMVSSLAMDDACQLTLSTVIGTKHLKALAIQKDSPYRNIFNYWLIR
LRESGILQLGLQKSMVTLTSCGSGPGYSSAGIGHVMSALGILGSGIAASIFLLIIELAWHYHR
TTRQQRQSQQN
>BgerIr197
MECAKLAVSHYFNDGASLTVLIEWANDNVSVLPELLDMLITIPTFIMKAAKGNLVPEDLR
EETNVLIVLEFLQNCNVKELTFLVLKHMKVLSLHISWDWRAKFLVIVETVHCNNYYNLAV
NLLKELKNWKIYKILTILLNSSNESHISFGRNLQMNVLPTLYMFLSYPHHSVDTGCPDFQT
LALTNCDNANKFVKKVDNLKLQTLSDNFFGCPIRISTFPYEPFVFPPTQVHVNDTLTILLY
TQGLEIELLHLIGQVLNLSLDFAPPPEDGELWGRLRPNGTWTGIRGDLIYGRSDVAFCGTV
LSTQSKNVMSPTIPYVRGGFVWVVPCPKPYPRWWSMFRIFSISSWISIVSSVFFAFSVTLVL
RRASSNVDAINLSDLWCILLGLSIPKMPLETYLKIFLISWVVYSLSVNTVFQAFLTGFLVDP
GFLPQLKDLEGLLNSGLEYGYLPMMDGYFDAGDNTDLKILKNRKACADPIACLFRIAVKC
DFAELISRQTLDYMKYYKFQNSKGDSTLCPFADDFVQYNVVMYLARGSPFVKHFNMVIDR
AKEAGLIDQWYRHIIYRSTLKGVKSSVELIDEGYSVLSVKHFQGVFFLYGLGVVFSVTLFVC
EKLYRRIFALENVTLNTK
>BgerIr198
MFIALIVVYGMLIEVCTCISELDQLFLCIRNITVTYYRENAAVVISLPSESEEDERSTSNLSLN
ADQWTNEVSYDALIQQQNLNQSIIMSKISTLADVASLSSLVEEKHGNYIILIQYSDNLQSVLS
SRIQFLSVIPSWNPRGRFLVVIVLAEAMNTTDILVKDILTTLWQWKLTNVNIILSASDSLNS
SSQTIKLYTWFPYSAPNKCSTVQDVVIIDTWSTELYAGFKDNTNIFPVKIVNNLHNCPIKAS
TFPLEMVSGKMTVHEGSSPKITYSEGWEVEFLELVAEHLNTTLTYLPPPPNNERWGNLE
DDGTFTGLLGDLVYNRADMGFAAWPLHPRMLLVVDPTKPYLRDGWVWWVPCAKKIPR
WRSIFMVFSTGTWTAVLLWILFAVIIMVLLARNTKGGGIQEWDIYKRVPICLSCILSIVLGV
SVAVMPRTQSLRLFFISWVWYCFAMTTVFQAFFITFLINPGLEHQVNTFEEILKSGVSFGY
NPLIDAIVSDSESEVRNRRLACNDNNKPPCLDWVAYNRNFSLLLSATFMDYILTRWYLDE
NGKPLICQAGDTFYGTHYVTYMNKGHPLLEQMNRMITRVIEAGFNAQLMERGMEKQRIQ
AAAKEREVYSDEYNNLSLDYLQGAFLIHICGVFLSFIAFLGELFGNRFLFDKNIHIAKNYCFA
YCSLKRNNRK
>BgerIr199
MKSVTFVVYWWIMCACSEEQNIINCITLIGKKYFALSMSTMVVSSRKTYCHFERSTLHDLA
TGIDSEDELLRELHTSELLPIVVSSPFAGSMLNNERNDIKIDNFIILSCGGEEDEVIDDLLEAI

QILSNENYWNPRAKYIVSVSIDGSNSTSFASIALRIFKELLWWKIVNVTIMTLKYNKEINST
SPVLNYEFYTWFPYKSKSECFDVRDVTLIDKCIVQNNQTIFTNNADLFPTKVTNDLQGCPII
AATFPIDLLVVEEESNDNTTLKYGGFEGYLISIIVEKLNLTLIYRPPPPNDEKWGGLSEDGT
VTGLLGELLYNRADVGFAAWPLHQSVLVVLDALHPISQDAWKWWVPCAAKLPRLHGVF
RMFSTHTWLSAFFSIILAIVTIRYLESMGSKFNELNECYRYRHLSSCACAVWCVFLNISVIIL
PQTNSVRTFFVFWLSYSFAMSTVFQAFLTTYLIEPVFGHQLRNFGEILVSGIAYGYNPLFDL
FVNDSEDELKYEILENRLACYEGNTPPCLDWVADYGNCTVLCSEFLLKYLLAQKYMDNQG
KPLICESEDTFFKLSYVMYMTKWNPLLDKFNSVLRHIPESGLLKNWFTNALVQKRIQAHA
IISNSIASQYNELSLMHFQGVIVVALCGLGVSFLAFMCELSMYRYIEKQKVVTNKRIKK
>BgerIr200
MLLLGIFLFYTYTTGNVEKYEEKIVQCIKTIALNHFPPFDTVVLSIYDNLDHNNLLSIGQAL
AQEIHLTEQWSIILSGPSDTNSNLMIEPEYEKHDNYIIIAYGEDEDDVYYDMVDKVEFLKQS
STWNARSRFVVLVILGSYDYNSSDIAKRLLEELWFGKILNVIVVIKQTVNAQAEAEKRLDV
YTWFPYQDSDRCLTIDEVVVIDSWLTEGNGYFQTNSNLFPEKINSNLHQCPVIAATFPIDF
VVGPPIILSESELTYDGIEVQLIKFVVEALNLTMKFRPPPPNNGKWGTLLPNSTVTGLLSEV
VLEKADIGFGAWPLHPELLTAMDATISYYRDDWLWWVPCAKKIPRWKGIALVFQTDAW
VSVFLSIALSVVVMILLTKNVQNEYIPYGNLSCFHNMWSVILGLSVVELPRTNPVRIFFTFW
VWYCLSINTIFQAFLTTFLIDPGFQHQWTGIEEVVNHNIKYGYNPGFDRILKDSPEEFTKEI
LRNRLECREYNNPPCLDWMAYHDNFSLLCSETLVRYILTNNYFDEDGKSLICRTPGLFFPL
NYVTYMTKWNPLLNQFSDKLTMLIENGMRDQWLEGELHLQQIQAAQIRRKNVAGDYFD
LSLEHCQGVFAVLVPGLVFSVLAFIGEFLNYKLTQHCRAKPKHISINIKNQRISRRVRLTTH
RVVTELKGGASQREFAP
>BgerIr201
MACFSVSNILFDKPSFIILVLLNLMTKSSANIEQQLSLCLEEIAKRHVDPASTILISYGGIGPT
TVCNRTTAETSQKMLPCSATGIWEPIFISLHNADNWNMMVIALEDVDMTNVLIHRKYAM
YILVSEHQQLDAVVNNLVQQVTKLKTSFDWNPRAKFVVILTEVNIVGENASRDISENIFTE
LWKFKIVNVIIILKARHTIDMTRGGKFHEALFDIFTWFPYHPPGRCAEDKSGILIERWIGDG
SSLGHFLNNTHLFPPKIPKDLHGCPLLVSIFEYQPLVSVKKYTSNPRRVLYDEGIEVKLLEV
LTRFTNMTIAFKEPPADGWLWGFQLPNGSWTGVSGEIIRSESDIAMDNYWYRCHLINEIE
CLTPHLSDEVRWYVPCAQPYPRWTSLTRVFKGSLWLGFLAAFIFFSFVMWYVVVKLSYVVA
SPEVQNQNYVGITKCFLNFWAIILEESANDTPHILSIRSIFLAWVIYCWAVNTIYQTFLTSFL
VDPGLQHQLSSEEEILTSDVRLGIPPTVVSVLPGLGHKKYRRNRANCDDVTVCQDRMAFK
GDLALLFSKYHLDYVIAAKYIDGNGNPLACKFDEVYCIQFVTFPVPKGMVMLHLFNGIIQH
VKEAGLLSQWWKSIQYITTLKAALGIEEIDSEYIVLAVEHLQSAFYFLFLGYGFCLLTFIGEL
MRKSKNLKKICCNRRSKTKTDKN
>BgerIr202
MKMVAPSIYVILACSVVLIPCSSATLVYPMINCLEEILNRHFLPGSTLVLLQGKEHSLIQNNA
TFRTTWFNNDINNKPMSVLDSLFLKEKYFLLLERAGQRKMKLNLKHLDYDGYILLSELED
PKVVYEDLKQRVETVRQFIDWNPKAKFIVIISQNFELSGKGNTKYILASDIFSEFWRLKIVN
VVIIFQSDFTIEDNSVVDYISLLDVYSWFPFKPVEHCGRVKDPIVVDRWILKSGKEQFLKNE
FLFPPKIPRNMKGCPLRISTFEYGPFMLGYRKKEDGSISYSEGTDFHVLTEISKILNITLHFL
PAPPDNGTWGVELENGPWDGMAGEVISGFADIGAIGVWNKCNVILELECGKSYLTDTMR
WYVPCAKPNPRWQSITRVFKASLWIGFVITYVVVGLCMYFVVTISNKIAPLELQNQAYAGF
VKCLLNFWAIILEESASNDPPEIFSIRTVFLAFVLYCWAVNTVYQTFLTTFLVDPGFQKQIS
SELEVLASGMIYHVLPATAYHDNFFLWPSIYPKMQFCSTLAECYEGVAYHDRAFCFSEVAT

DYQISVKYINADGKGIICKFDEIISSQLITFPVMKGFPKLREFDVIIQNILEAGLVNKWFNDIK
YLSILALAKTFNLPPGEYVKLSINHLQSAFYFLGIGYILSVLAFIAEIILSKKDLKRRL
>BgerIr203
MVTPSLYVLLACSAAVFPYSSAILVYPMINCLEEILNRHFLPGSTLVLLQGKEHGLLQNNTT
LWTTWPNEGIHNKVLSLLDSLFLMEKYVLLSERMGERKIKLDIKHLNYDGYILLSELEDPR
AVYEDLKERVETMRHFIDWNPKAKFIILISQKFELSGKENTKYILASDIFSEFWRLKIVNVV
IIFLSDITREDNSVVDYISLLDVYSWFPFKPVEHCGRVIDPILVDRWILKSGKEQFLKNEFLF
PPKIPRNMQGCPLRISTFEYGPMMLGYKQRDDGGVSYNDGTDFHVLVEIGKFLNMTLHFL
PPPPDMGFWGVQLENGSWNGMAGEVIRGFADIGAIGVWNKCHLILEMDCSKSYMTDNIR
WYVPCAKPNPRWTSITRVFKASLWIGFVITYIFVGLAMYFVVKISNSIAPLELQNQSYAGV
VKCLLNFWAIILEESASNDLPEIFSIRSIFLAWVLYCWAVNNVYQTFLTSFLVDPGLQKQIS
SETELFASGIPYQVFPATASHDKIFLSSKYPKILFCTEITVCFDSVAYHDKAFVYSQVATEYLI
SVKYMNADGDRIICDFDEIISSQVVTFPVMKGFPKLREFNMIIQYILEAGLVGKWFNNIKYL
SALALAKTFNLPPGEYIKLSMNHLQSAFYFLIIGYILSILAFIAEIFSMKRVQKKAKIGGK
>BgerIr204
MFQKLFYVTVISSLLIDRVESNDPTIDCLQSILERYFPPQSSLVFLQGKEDLHQPKKSRILAD
LEESSTILNVLFLMNRYSITIERAGERKINQTMKYTNHDGYILLSKLQDRHAVYQDLVDRA
SQLRHEFDWNPRAKFIIIITKSSADNGTTYDLVSDIFIELWRWKVLDVVILCRTPPKSISDD
LFPLLDVYAWFPFDPVEHCGRVRDPVVIDRWILNSGRGQFQTNENLFPPKVPRDMKGCPI
RLSTFEYGPMMLGQSTNEDGSKSYSSGIEYFLMREIAKFTNMSLYILPPPPDGGFWGIDLR
NGSWSGLAGEVMNGFSDIAAVDIWYKCNILPEMECSTPHMIDKIRWYVPCAKPNPKWSS
LTRVFKASLWGAFAAMYVIVAICMWVVVTISNHTTPKQLQNEAYDGFVKCSLNFWAIILE
ESASNDPPKIMSIRSLFLAWVLYCYAVNTVYQTFLTSFLVDPGLQKQISSEDELLASGIVYSV
FPSTYPHSVSLYTKYPKIQFCERREECLNAVAYRNEANVFSQVLTEYEISVRFVNANGDRM
VCDFDEIVSIQLIVLPVMRGNPKLKVINQVIRYLLEAGFIDQWFDNIKYLSTLALAKTFNLP
PGEYIKLSLNHLQSAFYFMFLGYGLSTMAFAGELLSKKKMFSRSLKKNQTKN
>BgerIr205
MAYNNLLFLISTIIFKVADAEVDISLCLDEIAKTYFTPGKTLVISYNSSNNDTFDSWDNVIEV
AFKSERWKIIIHNNRYDYNEDNDSDTELHGSYIIESEAQNYQDTLLDIKRQVLQLKMRRT
WNPRARFVLLLLLNYQLDAKIMCSDIFTLLWKWKVVNIVILVPSYDSINSSISMLNIYSWFP
YESFGNCGKNTDASLINIWTEGSDNKQHLMYNNSLFPEKIPKFLHGCPLKISLFEYPPMIM
GMNTNEAGTIEYSGGTEVELFRIIAEYLNMSIIYLPPNAIAWGTQLENGTVTGMLGEVISGY
SDLALDNFWYRCHVHNEIQCLTSHSIDAARWFVPCTQPYPRWTSLTRVFKLSLWLGFLA
AYIIFSLFMCLMTSLSNSFSPPKNQDKSYSNIPLCLLNFWAVILQNSVPHDPPGVHSIRIIFL
VWVLYCWAFENVYQTFLTSYLVDPGLQKQLSSEQEMLDSGITFAIPEVIVLVIPYLSTERYV
RNVYCPIFKECFDRTAHHNDLAFVFSQLHMQYITAEQYVNGDGKALICHFDEVITNQIISIP
VYKGFPLIDKFNLIILNAIEAGLMNQWLKRIVYTATLESARNFNLAPGDYIKLTTDHLQSAF
YFLLLGYAISVTTFVGELLCKKLSSIQLLRKY
>BgerIr206
MCGPSISLWVLLNYFTYIITAVNTDFTEPVQRIVENHFGVKSLTMLSETQITSNYEQNCLGK
FVDIHNIQEIRLCVFSDTLLEIMQNIFIANNLPFIYTRENTVVNYRHFRIRNDNYIILSSYSAE
QDVINDIKKQIKAINQKAGWNPRAKFVILLNQHQNHNADLASKLFAELSNFKIVNCIILMP
QRNENLALEVYSWFPFSPPGRCADVFDAFLVDRWYSNNATGTFIYNASLFPNKIPKDFNG
CPLRISTFEYIPFVGKMKYIDNSSVVYEGGLEINLMILCSELTNMTIVYRLPPTNGKWGVYM
GNGTWTGITGEITSHISDVAFSGWWYRCHLIDEIECITPHLIDAVRWFVPCPKPYPRWMS
TTRVFKTSIWLGFLSAYIVVALAMWKIVKWSNSISSRPIENEAYTSLVKCFLNFWAIILEES

ASNNPPHVVPIRAVFLMWVLYCWAVNTVYQTFLTSFLIDPGLQPHIASEDELLTSGMDFLI
PDTIMSIIPGLYSKRYWGRKVCEDVPECEDRLAFKSDIALLFSKYNVEYLAAAKYMDSDGK
SLVCKFDEVYSNQLIIAPVPKGSFILERFNDLFLRVLQSGVMDKWWKDIRYTGTLALASTL
NEVNSEYIKLTLEHLQSAFYLLFLGCIFSIIVFVSEVLCRRR
>BgerIr207
MEIQIYKLKFSLHWNSQGHFIVALIYLSPTDFEIETEVKKYHVKKVSETLYKLNVFNCIVIFC
SECNRTEEMQNCRDGSTVCNIYAYIPLYTVKSCSRRIDVTQTNSLIFHKADMWLLKNYTLF
PRTVTNNFKACPLIVSTFEDEPFVVKTKHFSNMQDTLYDNGLEVKLINFIVSSINMKLKFL
PPPSDGGKWGVLSDNGSWTGVLGELTNGRSDVAFGGVFYRCHITEDVECAMPHITDRVI
WYIPCAESYPYWSSPLRVFSPLLWLVLGSSYFLTVLIFSSLVLANTYNSNSKRNVTMCSILQ
HSGLNMWAVLLGMSETHIITHKLSIRTVFILWVMYSLTVNTLFQSYLSSFMLNPGLQKQIT
TEEDLLKSGLVYGHDHSIESVLPDLRSSQYSPTVYCDELEDCVLRLARGSAIAVMHSSISIDY
MTAARYVDSNGKALYCRLQDVFAIQHISLYLRKDNPLVSVFNTKILCAMSAGLMEYWWK
DIQHRAILSASKNSTKQNDFIVLQINHLQFAFISMLFGYIISILSFLCELFNCCFKLKK
>BgerIr208
MIVCTILVFILANIPCGESLQNELTRCLHNIAMKEFPQEKPLVFSYLWSRYAEEETVSQFSEI
LMDVRYSIHFEDFLPTLLEVFNELNQSIVVVNNDYNITKSEGPSSFYGGYVILHYVQDDIKL
QLVKLVSDSRWNPRANYIVVLIGNIKSNLHKYNVTQNIFSEFWKHNIIQVVVLININLYSRK
YEREDEDKIATNVFELYTWYPYKFPGQCAQSYDPILIDRWTTNKSYANFTENRPIFSNKLT
RYLNGCHLRISTFESEPVAMCPSGISEEGTLNYDDGLEIRVIKLIQRYTQQSIIYRAPPYHGK
WGILLPNGTWTGVLREIIDGSSDIAFGEVYYRCHLEEKIECTISYMYDETNWYVPCAQPIPK
WKSLSRVFKISLWIGFITGYIIVSILIWGVVRASNKVAKPGNESTTYINLTNCFTNLWAVILG
ISATEDIPKKTTIRMVFLLWVMYSLAVNTVYQTFLTSYMIDPGLQHQISSEDELLESNMDY
GFVRTLPVLIPDLQAQRYSRKQLCADIEKCIRRMARKKDFAVLHSKYSTDYSAYVRYVDSN
GNPLFCHLDETYSRQYVTMSVKKGHLLLGIYNRVIKAVIEGGFYDQWWKELKYLATLRAA
ANLTVPPGDYIPLSVDHMQSAFYILILGFTVAFNIFIYEVLHKKTLRMRIK
>BgerIr209
MLVFMQIIVIYHFIIYNSLSFGEISVAYISCIRDIAIRYFIQDGTLVISTDDDNHIEVDEIRELH
HIVKEFHNLFIWPIQLFSQEQNEHERYPRHQHGSYILLYQTSDLLKIKQRLLQLRKSYAWN
PRAKFLLVFLNEWIDEYELQECVKQVFSVLWNLNAIDVCALVSFSESTAMNKYRSTYVFY
VYTWFPYDLRNRLTEYDQIRQIKRWKLNTKIDSLLFFYNTSIYPQKIPDNFDGYPIRVSTFE
YEPFIMFPQTTKNNIITYGDGLEIRLLNVIVKMLNVSSVFLSPPSMEDLWGNLLGNGSWTG
AIGEILRKESDIVICGCYYPCHMSDDLECSSVYTYDEERWYVPCARPIPSWINIFLVFDKWL
WVLFILVYLLASLVVWNLIKLAKRHQSTRNTTGYTILSYCLLNMWAVILGVATPQKLPEN
VQIRCVFFLWIVYCLAINTVYQTFLVSFMVNPNLEKQISTVNEILNSGLHYAISATVQYLLP
ELSSERFQKRELCYDPEHCRYRLAHTRDFALLYSQINTDYVVALNYLNSNGKPLFCTIKET
FVRQYITMAMKKGNPILYKINSIILRILQAGLVELWWKDLKYRAALTAAKHTDDAADYHS
LTLHHFQSAFYLLLIGNTIAILIYFTKYLFQKSYLKKLNLCK
>BgerIr210
MISYFVIFLLITKSGGSSLFSNCIFTIADKFFTPSLPVVISTSKTIYQDVNPLNLNLDIDKGPDE
SDSWVKYLHDATKWPIRSFYVAVVNDQYIESSRTKEETYILISEERDGSAIVEDISFQAKRL
RTSWDWNAAGKFVVVLKNSQESHINNLARAIFSDLWISRVRNVILLTENSEMLDIYTWFP
YQPPDGCPHNVENILIDQCNENGSFVFNAPLFSAKIPKSLNSCPLYISTFEIGPAMMIKYAK
KEDNGQLNYDYGIEVEILKLFASETNQTLMFHPPPPDGGLWGFPLPNGSWTGLAGEISRS
YSDIGCAFLWNRCHLVRQNECLRPFLIDKVIWFVPCAEPYPRWTSLTRVFNLSLWVSFLV
SYIVVALVMWQIVKWRYRFSTDIIENEAYTSLVKCLLNFWAIILEESASNNPPYILSIRTVFL

AWVLFCWAMNNVYQTFFTSFLINPGIQHQLSSESEILASGLNYSTEKSIVAVYHFLHGKRY
SRITDCSDVPECVRVIAEEKSMAWLFDYYITKYFIALNFVDANGEPKVCAIPDDFAFQYITM
YVPKGTPLKRTYDMLLLYFMQGGLINLWWKNVQYIARLEMASKINLPPGEYITLSMEHLQ
SAFYFLFLGYVLSITTFILEQCVSRSKLGSPISIK
>BgerIr211
MLLLYFIIAFGGVLASDENLIHIVECLHDVLVKNFETKDSIVISTNVIDPTVKPFSNPLDGSSF
LSLILSDIHYLQEWPLFVTSISHDTKIGPRTNNQDGYILLLEGNEDELVSQLELQISLLQNSK
DWNPRGKFIIVLLETIENPEESVIAVFSELWKSKIANVVVLLHTKEVSCSSNTNNNEQFLLY
TWFPYSPPDNCGNFIDAILINCWIMENEFSGNFINNSSLFPEKIPRKFHGCPLVASAFELGP
VVLKSDPNGEYDEGIEVRLFLEFARFSNMSAKFLGPPPGEYWGIRLDNGTWTGNTGQVYR
CHVLDEFECLYPHIIDPLRWFVPCAVPFPRWTSLTRVFKLSLWLGFLASYVVISLFMFLTV
KISMFISTHPLENDAYNGLVKCMLNFWAVILEESASNFPPPVLVIRTVFLMWVLYCYAVNS
VYQTFLTSFLIDPGLQHQISTEDEVLTCGLEFGIHPTVTAVFHELDEGRYKGHISCNNFKEC
HERIALQRDLSLFFPTLPLEYIIFVKFLDSEGKPLICKLDEVYVNALVSFPVLKGYPMLFAHN
NVIQRLSEGGILNLWWNNIKYSAALSSSRNREVVNSEYITLSLKHLQSAFYALFLGYLLSCF
SFCVEYFYSKTKAYFSSNTLRLRK
>BgerIr212
MLGSAVVLCLIVQVSAQMHSYLLDSIHEIALRDFKSGIVQISDSFYSYNETCNLFNTLQSKSN
SISTCLKIDLLNYMLADLHLMKNWSIILLPHTERANEETLSNINSYILISYCEDHNCALKDIT
QQFQLLMKNHGWNPRARFLVIFTHKDLQGTKLSEELSLILWKRQVIEVIFLVASTENVNE
LSSSEKDTVLEMRSWLPYGPPGRCANISDTTILDYWLVDKSNGAGSFFNSSILFPNKFPKD
FQGCPITVSAFEFAPNIMLKKGYAPNSSHVEFDDGHEYKLFMTLVNYVNLTANFKYNPDQ
WGWNINGSWDGVTGEVIRGDIVTAPVDFWNKCHLVPEIECSVAFIIDHIKWWVPCPEPY
PRWQAMTRVFQPSLWLVFLLAYVSVAICMSIIVTVNYKIDKDVQNQQNQSYTSLVKCMLN
FWAVILEESASNNPPHMPAVRAMFLAWVLYCWAVNTVYQSFLTSFMVDPGVQHQLSSQ
DEVYASDLDIGFLRAIVTIFPELNAKKYRCSTHCENNAECHQRIAYNNNLAVMWSEVNME
YLIASKYITQDNKPLICTFDYVFGTQPVVLPFMKGFPLLPLFDNLILHVLQSGLFDYWWQY
LKYVSTLEALKDTETPDGEYVKFSLEHLESAYGFLFLGCIVALLLCIGENIHSKYHSKNPKP
QKENIPKQLKTEKFYFSPQVVSVYPRKDRS
>BgerIr213P
MNRVAKLNKTSSSKKLIMFTSFWIWTCSCGENELKIVNYVQEMAVQHLLNETSLAVSYNI
DFSLHSNLVQTHDTNNLHLRTLETSKSLIKRIYLTQFWSLQLFNKAIIDIVGICLXVEKLGA
YILIISGCCEIKTMIHDLKVQANILKEDKGWNPZTKFIVVVVNEYKDVCLEIKHSESIFSZLW
KFKIINVLSVMQTKSSGDSZKFNIYTWYPYYPNGRCRYIREPVLLDTYZYITDNQKARFLD
NANLFPNKLTENLHRCKLKVSTFELGPMSLNKIEYEDETYEFQEGIEVQLLKANAZTGRQL
MGSZSWEWNMVWYHWRSCQRVCZYCHCKYLAQVPHCSRLRMSHITDTVKWFVPCAQP
YPRWTSITRVFKASLWLGFLAAFIIVSFIMYLVVVASDRLSLEKLENQAYHGVVKVVLHFW
AVILEESASNKPPHVLSIRSVFIAWLLYFWAVKTVYQTFLTTFLVDPGLQHQISSEDELLTS
ATVRYGFDSDTVAVISGLVEDRYRHRVIFSTLQDCRVRFVTTNKFTFAFGAVTTEYQIAAR
FMDGDGNSRICHFDDVIANQLTSMTVRKGYPLLEHFNKVIRLEAGLVEKWFKDFKYTATL
NSAKEFNFPPGEYIKLSMEHLQSAFYFLXGFILSFLHFIGEIFWDKKLKSVPESNLCL
>BgerIr214
MLVLLRAMNLLQFYIFLAKVTAIISFDRNSHFNINDCLLDIMKRHFITLNTIVISINGEQVTN
HKLIEPFSCKDEFSVEYETSIITFLYESAEYPLYIIRSSQILGKRISHVRLDCHIVISHYEEHFQI
VKDLEMQVVTLTSRRLWNSHSRFIIIVSAFKNSSKELTEDIFEIFWKWDIVNLIVLFPTKRE
GENFMPINTITNSHVNETYTIIFDAYTWFPYNPVSQCGKVKQVTHLDQWILNDNDKGQFL

YNLSLFPKKIPTHMNNCPLIVSTFQFEPMVLKNKIGDFDNGIELKLIKEFTARINMSLELLV
QTDGTYWGIEMKNGSWNGMSGEVMNRISDVGMGNWYYRCHSIGSIECAYPHLIDEARW
FIPCPQPYPRWASLTRVFNPFLWLVFLISFIFMAVVMFIIVNINDCISVTPSENQAYSSFAKC
CLNFWAVILEESASNNPPRIFAIRTMFLTWVLYCWAMNTVYQTYFTSYLVDPGLQHKLSS
EEEIFSSGTAYGIPSTMFPLFPGLNSERYEKCLICDDFNKCQDRVALKGNLALLFSRFNMDY
VTASRYMDAAGKSLVCIFEEVYSNQLVSFPVKRGFLLLERLNHMSRHAIEAGLLEQWWNE
IKHSSTLDSAKDFESSSEYIKLTLNHLQSAFYFLFIGLAFSIIIFLFEFLYQRM
>BgerIr215
MAWKYKSIVITLRCFVISMWFVEQVSCTEVQSELLRCLQNIAFNYFKSEDTIVVSFKTKSDF
SASESIIFLDTTDVLLKGLTSLEVWSVKVISYQNFDCSKFELVMETNSNYVLSINSRIERIIIE
DINHQLTALKRCRNWNPRTSKFIIIANNYFSRETAAQILARLWKYNILNAIVFMEASHKEI
TISHNTEVGLSTPLFDIFTWFPYSPPGRCADVSDAILLNRWITDGKMNGRFISNTSIFPNKI
PDVLYGCQIVVSSFNFPPFAVENKSLKLTSIEARFDDGVLVSLLDIILRKLNITARYIKTSERE
RWGTRIINTTNGIIGEVIRHESDMALNLYCNVCHLYFDIECSFTYYVDQMRWFIPCPNLRP
RWLSILDVFKTSTWLIFLMTYLVMSIIMSMVEKMCNKVTRKQARNKLEYLSISLQHFGEIA
LFGGTYLSPTSRFATRSIFLTWIIFTVAINNVFQSFLTSFFVEQGQDTKITTERQLQQSTFEL
VVNPDVAGVFQELKDGRYKLEYCKSHKPCEEFYDFKGDVAYLKMSVNMAYIAAKYHGET
NKNEVVCHIDDIFVSLPLTLPFTKGFVLLEKVNKILMKAVDSGILYEIWELFLFKTAIYFKH
ESDDSTYVTFTLQHLKMAFICLLSGYSLSLIIFITETLLSRNRSYRYTNK
>BgerIr216
MFIFSCNMFTYSITFFMFMKITQNTDVRYFSQYLYDISVRHFLPRGSLIVSYFELQDSMNGI
SSNINLVVSELLKQLQHSMNWSFLLSTSLNKEKVKIQEKHDPYIVLVRSVHDMEKQINYIK
LNLELDAHSTFLIALFPDSLITDRHYLAQEILTSLWSRNIFNAVVLLPVIKEFTQKIRPLFES
SETILSFEIYTYFPFKPPDRCGKTVEAVLIELRFVGATNENSISNDSLFFLSKIPRNFNGCVFR
ISTFEYRPMVMYPVNKTNEPFYYADGLEIRFVEAMLKAMNITPVYRPPPGNKLWGTDVG
KNGTWNGIFGEVQRGESDMSIAVNVLDPDRSRLADAVYPYLGMDLVWHVPCPKPMPYW
SGLIRLFSLNLWLAFILVYVIFSVAFWLLVLLKRSSTSQYSSLEICLLSLWALILNMSASDGIP
NSKVIRLTFLLWALYCFIMNNLYQAFLTSYFVDPGLEPALTTENELLNTDLKLGFHPHMA
LRHTELNSAPYRHQIPKDNVDEGLKRLAIKEDLAFLISPYVAGYKAYFTFMTADGKPGFCH
LEEVFSIVWMSIYLQKGSPLFDIFNKYVPIVWQTGLVDKWHRDIINTAIITGHRAPTDTAS
DGDATGFSLKNLQSAFYALVLGYIVCVFSLLIEFIHKALISLNLKGNRRKRPWSIRLLN
>BgerIr217
MKIISTFLIFIIIYNQCLFLAMHENVLLTDEQRHLVHCLMNVALNYFTPNQPIAFSITKKEDS
DVMENPYFDILSNSIDGDIANYILKSLHAALQWQIRMSYLVDDPLELPFDGKEKIESYILLA
PSGDLEDMALGLGEQLNALKASTTWNPRAKYVIIVTSHLQSELEDAALTILKDLWLFYKIL
NVVSIFPRTELISTVAMTTSFDFYTWFPYQSNVKCANTEDVIVIETWILKGEGYFLKNSTLF
PNKITKNLQGCLFRVSSFELVALVNIDIFIDENGIAHKEYSGFEVKVVKEVAETLNLSLLFLD
VDGIGVQTRVNALGDLESGKTDITFGAFPLHEQVFPFADPVVSYFDDIMNWYVPCGRPVP
RMQKVMEIFTLSVWLTLGISITISAIVMNFEAKLTEKCKTKESSGYMQMKNSVLNVWAVV
MGVSVTEMPRTHRLRLYFFILIWYCFAMSTLFQTFFTSYLVNPGIMERIRTLEQLYESDLE
YRFFPENENYLKFSFPTYYSRIPLKRKLCNLSEVCLYELISSKDFTVIGHSFHTEYFTSSNNN
PKLCTLDDNIYKLSFTMHLAKGSPLIEVFDDVVRRLIEGGLVGKWWLDVKTDYWVRLLPK
DEFFFPLFLNFNSKEEEYVVLSLSHLQLAFYVLGVGCAIGLILLICETVHYRIFSRNPPKHGK
KVKFRKVKVRQHKKCHRSINNYFIFQ
>BgerIr218

MRVFRIMLASLILGICIKTNELVNEDQFFLCINKIVQNNSGLRTFMISFTLSKVHVNMIDRS
LEVDFPVASTDLVIGEIQNAYTIVYVIEAESFMIRSKIELTPVYNEKSKFCIIFLRRQEDEELL
DELSNHFLTLMQFVDIDNARYLLIMPFTLTKIMLKNISQVALKYKAHDFVILTLSSDDGKE
KLLDLYTYIPFQSNGNLFEDNVPVLINQWSSGGFLENNNIYPPKVPKSFFGREIKVSAVVN
NPFTWLEKNYTNEEGKLTYIVTGPEIEILNIICKKLNLTITFLEPHLKTNAIMGKVHNAVLE
AMYGESDVSIGTMTTMAWHFYKDMFVRSRMYTTLTEKWFVPCPIQLPREVNLSRLYFVA
FSSAMAIIMAFIFIVFTSIIHALAKKTNYTITEIPAYMSISRCFLNIWAVHLGTGTEKQPRSN
ALKILFLVSMCYAYVMNDLLRAYFKSFLIDPGMEKAVTTFEDLRNTKYYYGYQKHLTYHY
MRQINFDSESESARRDDCEDTTDCLKRIVESRNYATITCAPHVEYFTRVYYPKKTNVICAL
KDGYIQHHISLFFTLGSPYVKRFDHYLQFIVESGLMIYFEDMDKYSYVYRHNTEDFHYTNT
NEDSENTNEETSKTSGEAFNLEDVMVAFVSLLLGLTVGSLSFIWEMYTYRMVKSKIPDIST
ENNCMINTIIVSTTEPDPNK
>BgerIr219
MICIDAKSLILLNVVFSVSICDYNEHLINGIRNIESKYFKHIHIATVHIQAAMKPAIKYSLFFN
TNIEASKSPATIKEVNALNILQGLEKSRPHSVIVYDSSQNSSIWLRDLNESFGITVLSSYKKS
LIEFASYLCNKRGSTRQSYLIFIDVIQGKYFRTIDLIMKKLTKCDIYKVIIVYNNEVNSSLDVY
MHTYIHPPTVNCKHPYNIVLMDQWVVIDEGYFVRNVSLDQDRISYNFGNCSVKVSAVSYE
PHVMHSDSNDDGVFDDGFEILLLKFALQKLNASISFLPVPKSLWGLKLKNGTWTGILGNV
FEKRADIAICGLMLLLKRVADFDFVWPIDTSNFYWVVPCAKPYPPWSSILRVYHPYTWIFI
FSMVLMVSAVMLQMSKYQEEVFLKNISNYLLDSWAMLLGSGVNKPPLSLSLRLCFFTCMG
YSWAINIIFQSFVTSYIVNPGLQNQINSLNSLLASGLDYGYHPDIDYFFEDDGNILLKEIVSR
RQECYNIQNCLERVVQEGDFVTISSYESIQYKNTYKYIDESGRGLLCTIKDPAFVSYKTFYLA
PGSSLRYSLNKYINVAIESGFTRYWWKYIFVRSLIKTASKRIRTLKDDYSVFLVRHLQGVFY
MLLFGYILSFIAFSCEIFQHWLLRNKLRSFSKL
>BgerIr220P
MVGTYYYRMIMYWYIYLFILSMIRIALTFLVENDKILQNNYQYSSCIVSIIERHFETRRTIYN
YFFKIQTXNNQTVRNRKQSRNHIYTEQTNILLRNIHKTLNWSLIISFPNEKYTSIQENGKYN
YILWYSADLSRQLQKFFYFTKTQDPNARFIVVYEKTGDRVHQRTCVLQVLKKLRKLNIFN
VIVIVPTKIEPSLPVLNIYTWFPYELPSGKCGHILDIILLNQWFSFKNERQFLRNSSLFPNKIP
FNFRKCPLVAGTTLFVPYSMLNTTSTHVTSEQGLEFRLFNFVTNNMNLSTKFMLSPVGID
QWGRKLRNGTWTGLYGYLAYEKADVVFAAMSERFERVSIMDSSIPYINGGLVWVVPCAQ
QLPRWKSIFMVFSPSVWFISFIVMFISSLIFFSLSRFQNKKQIQLKSFNRKLSDSFLNMLAVL
LGFSAAIVPDIGIFRFFYALLIIYSLAMNTVYQSFVSSYLVDPGKEKQIQTLDEILKSGMRYGF
NPGLDSAFQDNSDSQIIEVKRHREACYNETECLERSNQFAVLTDRLQITYHRTYKFLDNSG
ISHFCVTNHDFIESFKAFHLTKRNPMLREFNRHICIALESGLVVKWWKDMLDTLRIKAIAL
ERVSTFDNNYSVLDLSHLQSIFYTLLFGLCLAVIVLLIETLQHLGQNNLDRSSERRAVRWH
TWVD
>BgerIr221
MEYCGAICASLINIADKYFKRSMPVILYFILENKVSGPKSLSVEQCQFTNINLLIQNIHQLGF
FNLLVHNHTVLSENVYNNIGKQFSYLIYIEDSRSDHLEIFIESYLKTLNIWEAKGRFVVFVE
HSDKSTRLLVLNCFWKYNVIDVVVLEEITDMNVLSVYNSPNLPSVKDESLKAIGIYGWTSF
QEPGNCEKIIILLDVFVSIDGGHFVSNTSLFKRDNTYKFNMCPLVVSTFENHRVVRSRKVT
NGKVTFERGWEVYLLNIILEALNASVYFVEHDPSVKHGGHYNNGTAYGLTGDLQYYRTDI
AFAGLPIIEAVTKIGDPTVSYSKIKYGWLVPCAKPLPHWESIFRIFAPELWIAYLGCIFVSSV
VMTLIAKCRKRILFLKYETFSTFSDSVCSFYAVIIGGAMSVPKSETLRLFYFSWICYGFAFNT
VLQTFLTSFLVQPLMDHQISSVDEMLDSGIAFGYGRGYDRIFEKIEGKKFQTILHNRQNCST

DEVCLQRLAYKSDFAVFFNRLRFEYQVKYKYSDKETGRLLICSIPEVSYTSNYAMFVPKGH
FVLSLMNTVIRRVLEAGLYLKFQTDDEYLEKVRTHAGSKQFSESEYFQLEVNHLQAPFTIL
LFGYVVSALMFVSEIVYYRLCRPVSLSLGAKL
>BgerIr222
MRLSQELLLLSAPSVVLCFSNVLDKYLQLSSCVKEIYEKHFIPNANILVSLSIQDDVFESSRT
FNQYASVTVKMFVDNLIEELHTLSTYSITVTSPKSVIVKMWDCKLGNVSYVLLCRWTNTN
SSKCVHDQIQVLHLMNCLNRRANFLFVVVTDGDIVESQSVVRDILEELWKWRILNAVVLL
SNLSSSPGTNSVTFDIYTWFPYQGIEQCTQVQKVFKINSCTSNGLLSNLPLFPSKIPKTVKGC
PITAAIVVTPLIAEAPRIILGKNGTSKPHFDKGWEIKIWKILVEKMNLTEEYIESNFRFALNS
TSAGIKQLILSLRADVGFGSWPLLWRNIKIFDASSSLHRIEFVWLVPCAQVHPLWTGMFRI
FDIYVWIAGTVMSFTTAFIFSYISKCSGEIGRFSNYHQCLLSLWGMLLGISLSILPIRTHVRLV
YLAWMVFCLSINTVFQTYFTSFMIQRVHQHQISSFDEILNSKIEFGYPEGIGEIFRFSDDPKT
IKMKQKRKLCQDIKTCLSRVAYKRDYCLLYSRRLIDFDLTHKYLDEKGDPLICMVKETFFS
GLGTIYFQKGHPLFDRFDLLINRIIEGGFYDFMVRRDMEKAKVRAAVRFVHSVAFEYSVLK
LQNLQAVVFMLLIGQLASFVTFLGELICSRCHVT
>BgerIr223
MLKIENVSSHGHFGQLLMDSMMSVADTYFVPNRTVVLSLPLKIVSEDTIRHLLGNRDEAN
SIQFVNNLLENLHSKNKWPLLTSRSMEQLQEKDETDWDTSNDDKHSSYIVFVENFDEDH
LNHTLLDIQQQIMTLESFSAWNPRGTFVVCLFTDRFGPQYMIKEILRLLFRWEIVNVIVMV
PYYETDRFIVNVYTWFPYLLPSGSCGKILDVILIDTWILKLSGGQFLWNIPLFPKKVPNDLN
GCPLSGTTFEFEPFVIFNEELGKGTNASIAGGLDIELMFCLTKAMNMDLNFKIPPGNFRGG
SQLENGTWTGLRGDLIGGNADIAFASLLNGEDKTTFESTRIYFTDRFTWTVARAKPYPRW
LAMIRVFAPSTWLYGFLSLIVGAIIMRTIFVFKIVQEKEVTPRYWGLVNCLTSVWAVFLEEG
VASMPESLPLRMFFLCFVIYALAINTVFQAFFTSYIVNPGLLHQISDFDELLSADLVYTYYH
YVMDKFLTDDFKQHLEPKVYCDPYDCFDVVATEYNCATFSGRVLVAYIIDFVVKKPGKHA
IYPFPDDLFQLNSVMLVPKGSHLLNRFDEVITYIVEAGLPDQWMKNILTSRAMKAGLVAL
TDLSDEYEDLTMNHLQGAFMFLLMGIALASVLFIFELLTKSLCEKNNRAWKCKITTDSMCI
PCRN
>BgerIr224P
IKIENVSSQGHFGRLLMDSTMSVADTYFVPNRTVVLSLPQKIVSGETSRHLLGNRDDANSI
QFVNNLFKNLHSKNKWPLLTSRSMEQLQEKNETDWDTSNDDKHSSYIVFVENFDEDHL
NHTLLDIQQQIMTLESFSAWNPRGTFVVCLFTDRFGPQYMIKEILRLLFRWEIVNVIVMVP
YYETDRFTINVYTWFPYLLPSGSCGKIYXIDTWILKSSGGQFLRNTPLFPKKVPNDLNECPL
RGTTFAFEPFVIFNEELGKGTNASVVGGVDIELMFCLTNAINMTLYFKIPPGNFRGGSQLE
NGTWTGLRGDLIEGNADIAFSGLLSGEDKTIFDSTRIYYSDRFTWTVARAKPYPRWLAMA
RVFAPSTWLYGLLSLIVGAIIMRIIFVFKIVQEKEVTPRYWGLVNCLTSVWAVFLEEGVASM
PESVPLRMFFLCFVIYALAINTVFQAFFTSYIVNPGLLHQISDFDELLSADLVYTYYHYVMD
KFLTDDFKQHLEPKVYCDPYDCFDIVATEYNCATFSGRILVKYIIDFIVKKPGKHGLYPFPE
DLFQLNLVMLLPKGSHLLNRFDEVITHIIEAGLPDQWMKNILTLRAIEAGVLALTDLSDEY
EDLTMSHLQGAFMFLLMGSALASVVFISELLTKSLCKKKNKNRS
>BgerIr225
MHATMTVADTYFIPDRTVVLSLPLNIVSEDVKRHLLDNNIDTYFEANPTQFVNNLLENLH
RTNKWPILTSRSIEVQKERDMTERDTSNDDIHSSYILFVGIFEVNLNYIIEDVRQQVKTLEM
YSAWNPRGKFVVCLFTDILVTQDIVREILRLLFNWEIVNVVVMVPYYETDSLNETSTVNVY
TWFPYVPPSGSCGKFQDIVLMNSWVLNASGEHFLQNTPFFPSKVPNDLNGCPLRASTFEF
HPFIICDEKLGKGTNMSIVDGLEIRLIYCLAEAMKMNLILRIPPGNFRGGSQLENGSWTGLR

ADIIDGLADIVFASLLSGEDEVIFDSTRIYFTDRFTWFVARAKPYPRWLAMARVFAPNTWF
FGFLSIILGGIIMRVIFLFKIVQSKEVSPKYWRLVTCLTSVWAVFLEEGVGFMPRSTPLRTFF
LSFVIYALAINTVFQAFFTSYIVNPGLLHQISDFDELVTSDLIYAYYYRVLDKFLTSEFKQKL
KVKLFCDPFTCLDMVATNYKYATFCGRALLAYIVDALVKQDGKHDIYAFKEDLFQINSVM
LVPKGSHLLNRFDEVMTYIVEAGLPNQWLKTILDIRGIKAGVIALTDLSDEYSALTMNHLQ
GAFFFLLMGIALGLITFILEVLTVLFCKKKKKK
>BgerIr226
MMLWGHIAIVFLLSPGRAELFNPSEDSLNLQTVDCIIDIADKYFTVERTIVLSQNLQPSFLID
NILERLNFIALWPIVVSRPKNGEDFSNINQNARDKHHSHVMHIAYSNGSIDRPLFDLKEQL
GDLKSFSSWNCRGKYVLILTGAQDAQNKKDLVREVLKEFWLWKIYNVIVLLPSLKERDFV
FIEVYTWYPYQLPSGNCGSIQEIVLLDSWMSSESQKGKFQKNSDLFPAKMNNMNGCVFRV
WTINFAPFIIVQNNTIVGGSDIQIVTALAKYMNATLTFDIFVGKERKGQKLPNGTWTGLRG
KLHYDQADIVYGSLLGNYDNHVYFDQSRPYFFDDFTWLLKRSPPYPRWLGVTRVFTTSA
WVLHFLTIFLTGAIAYLLSTFPNSEFWNFSKCIISVWAAFLGAGMPDMPRDTVLRVVFLF
WIVVSFGVNSVFQAFFTSYQIDPGHRHQISTADELYSSKIVYIFSTPIDKFFTPQVLKKLVPR
VRCEAVSCLYEVAVDKNEAAVIAGGGVIDYHRENVRKVTNREVFVFPEAMFQLQIITLLQK
DSRHLVRVNEYNARLVESGLVKFWLSCLMEQRKIEAQTLALEVLIDDYVDLEMSHLQGAF
IFLIIGIAASVICFFAELAMVKFRLKKLFNFKN
>BgerIr227
MILSFMVNVLTSITIFLVFSIHAERILEAEMTDQDDILIASCIKAIGQQYFFNDKPLITTSQLR
TDFETIHNLENTILEELHNNLMWPMFLSQPAQSYVENQWSTDRYEKIHNYILILDYQYQD
HNKTINDLRLQIQKLKTYGVWNSRGKFLISMKVSVPSEEVQDIIKMVLNETWVSYIYNIIVL
TPIADFNSTQEIEIYTWFPYQLPSGSCGVLQEPVLLDKCMRNEGNVEFLKNEPLFPNKVPD
DLQGCPVISMTYMFEPYLKTEGGIWNNTINDGSELSFMQCISNHINISLEIRTCPDPTDWP
ALREELRANHSINVMFAALLINIVDFELFDGTTVYHSERFTWTVPRADMYPKWQGITRVF
TATNWALQFLTIAFVSVLITILFRIRYAKSGEYWNLSKSLLSMWAVYLGDGSPNEPKEMVV
RLFFLSWVFQAFALNTVFQAFFTSYQIDPGRQHQIAEFDELQATKPTYLVTKPLEIFFTPD
VLKTMYPRPYCYPDDCLEYTATHKRSGMFCGREYFKYKIDQTVKRVHGHDLFTFNYDLG
ENSFVFYTQKGFPLFSRLNSMVGRIFQAGLYNYWIDNVIEIKRIEAGIVKLEVLDEFVKLSL
EHLQGACMFYFIGILSSTILFLCELGYYSAQKKLRKK
>BgerIr228N
WSTDKYEKIHNYILVLDYQYQEYNRTVNDLRVQIQNLKSYRAWNSRGIFIIAIRTNVPPTE
VQSLLKLILNEAWLSYIYNIVVLIPKLVCNSTKEIEIYTWFPYQMPSGSCGVFQEPVLLDKC
MRNEDNVEFLKNEPLFPNKIPNDLQGCLLKSMTYMFEPYIQTKDKTWNGTIIGGSEIKLM
QCIAKHVNATLQIDMTADFTSWRDLREQLANRTIDVMFAAQLISLVDFELFEGTSVYHSE
RFTWTVPRADMYPKWQGITRVFTPANWALQFLTIALVSLFITILFRIRYAKSGEFWNLSKS
MLSMWAVYLGDGSPNEPKGLIVRVFFLTWVFQAFALNTVFQAFFTSYQIDPGRQHQIADF
DELRATKPTYLFPVPLEMFFTSEVLETMKSRPYCYPDDYLEYVAKYKRSGMFCEREFFKY
HIDQTVKRVHGHELYTFKSDLGENSFVFYTQKWFPLFPRINSIVERIFEVGLYNYWIDNVIE
IKRIEAGLVKLEVMDEFVTLSLEHLQGTCIFFVIGILTSLVVFICEICIYTMKKLKTKTK
>BgerIr229P
MCGFSLQILKLIPILMHFSSQAERIAEIEVIGQDQILTANCIKAISQQSFINSIPLIITSHIRTEW
QTIHNLEDILLEELCDNLIWPMFISQPQQSNVEKEWTTDKYEKLHNYILVLDYKYQEYNK
TINDLRQQIQQLKSYRAWSSQGIYIVTKEVSVPREDVQNLVKMVLNEAWLSNIYNIVVLIPI
ADLNSTEEIEIYTWFPYQMPSGSCGVLQEPAMSDKCMSTKGNMKLFKNEPLYPNKVPND
SQGCPVTSMTYMFEPYVNTKDHIWNGTIIGGSEVSMMQSIENTZILLCKZELFRTSHILQH

YGMHZZQILQZMLCLQLYCSEEATFNGMSVYHSERFTWAVPRADMYHKWKGITRVFTPA
NKALQFLTIAFVSLFITILFRTYKIZKVRRILKPLKKFVINVGCVFRRCIPKCTERNDCLVIFS
CLDIASSCYYTVFQAFLHQLPIDPGRQHQIFDFDEFQSSKPTYLFHEFLEFFFNPEVLKTIKP
HPICMPIVCLEYVVQHKRSGLFWGRDFFLNIVSMEQZYGYVVTKFFTFNYDVGESSFVFYT
QKGFPLFPRMNIIVERIFQAGLYDFWIDSLITEXKIIEAGVVKLEVMDGFVELSLEHLQGACI
FYLTGIAISSIVFLCELGVYLLQKFKMNIK
>BgerIr230
MILIINRYHEQLIIALNFITILMHSSSHATRISDIEVIDQVHVLIANCIKAIGQQYFIHDMPLIIT
SQLRTDWQTIHNLEDLLLEEFHDNLIWPMFISQPEQSNIEKEWNTDKNEKIYNYILNLDY
QYHEYNTTIDDLRQQVQQLKSYRAWSSRGIYVVTMDDIVSQEEVQNLVKMVLNEAWLSY
IYNIVVLIPIAYFNSTKEIEIYTWFPYQKPSGSCGVLQEPVLLDKCMSTEGNVKFLKNEPLFP
NKVPNDLQGCPVTSMTYMFEPYVYTKDHIWNETIIGGSELSMMRSIEKHMNFTLQMRIV
PDVTLYPAIRHALVANHSIDVMFVALLLRGTDFELFDGSSVYHSERFTWAVPRADIYPKW
KGITRVFTPANWALQFLTIAFVSFFITILFRIRHAKSGEFWNLSKSMLSMWAVYLGDGSPN
EPKEMVVRLFFLSWVFQAFAINTVFQAFFTSYQIDPGRQHQIADFDEFQASEPTYLFHESL
EFFFLPEVLKTMKPHPFCIPDICLEYVVHHKRSGMFWGREFFKYSVDGTVKRVNGTEVYV
FNYDVGESSFVFYTQKGFPLFPRMNQIVERIFQAGLYDFWIDSLITEKRIAAGLVKLEVMD
EFVELSLEHLQGACIFNLIGIAISSVVFLCELGVHFLMKLKTKIK
>BgerIr231P
VLGFIVILMHISSHASIISEIEVIDQDHIMIANCIKAIGQQYFINDMPLIIISQLRTEWQTIHNF
EDILLEEFHVNSIZPMFISQPEQSNVEKEWNSNKYEKIQNYILNLDYQYQEYNTTIDDLRQ
QIQQLKSYRAWSSRGIYVVTMNDIVSQEEVQNLVKMVLNEAWLSYIYNIVVLIPLAYFNST
KEIEIYTWFPYQKPSGSCGVLQEPVLLDKCMSTEGNVKFLKNEPLFPNKVPNNLQGCTVTS
MTYMFEPYLKTKDHIWNGTLIKGSELSMMQSMXKHMNFTLQMTIVPDKTLYPAIRYALV
ANHSIDVMFGALLLRGIDFELFDGTSVYHSERFTWAVPRADMYPKWKGITRVFTPANWA
LQFLTIAFVSLFITILFRIRHAKSGEFWNLSKSMLSMWAVYLGDGSPNVPKEMIVRLFFLS
WVFQAFAINTVFQAFFTSYQIDPGRQHQIADFDEFQASKPVYFYPQPLEYFFNPEVVKTMI
PHPFCIPDICLEYVVHHKRSGMFWGREFFKYSVDGTVKRVNGTEVYVFNYDVGESSFVFY
TQKGFPLFPRMNAIVERIFQAGLYDFWIDSLITEKRIEAGLVKLEVMDEFVELSLEHLQGA
CIFYLIGIAIS
>BgerIr232N
YFNSTKEIEIYTWFPYQKPSGSCGVLQEPVLLDKCMSTEGNVKFLKNEPLFPNKVPNDLQG
CPVTSMTYMFEPYVYTKDHIWNETIIGGSELSMMRSIEKHMNFTLQMRIVPDVTLYPAIR
HALVANHSIDVMFVALLLRGTDFELFDGSSVYHSERFTWAVPRADIYPKWKGITRVFTPA
NWALQFLTIAFVSFFITILFRIRHAKSGEFWNLSKSMLSMWAVYLGDGSPNEPKEMVVRL
FFLSWVFQAFAINTVFQAFFTSYQIDPGRQHQIADFDEFQASEPTYLFHESLEFFFLPEVLK
TMKPHPFCIPDICLEYVVHHKRSGMFWGREFFKYSVDGTVKRVNGTEVYVFNYDVGESSF
VFYTQKGFPLFPRMNAIVERIFQAGLYDFWIDSLITEKRIEAGLVKLEVMDEFVELSLEHLQ
GACIFYLIGIAISSVVFLCELGVHFLQKLKIKIK
>BgerIr233P
KYCEDNVKLLKNEPLFPNKVPNDLQGCTVTSMTFMFEPYVKTKAHIWNGTVIGGSELSM
MRSIEKHMNFTLQMGIVPDVTQFPALRYALLANHSIDVMFVALLLRGTDFELFDGSSVYH
SEQFTWAVPRADMYPKWKGITRVFTPANWALQCLTIVLVSLFLTILFRIRYANSGEFWNL
SXVFLSMWAVYLGDSSPNAPKEMIVRLFFLSWIFQAFALNTVFQAFFTSYQIDPGRQHQIA
DFDELQASKPTYLFHESLAFFFLPEVLKTMKPHPICMPYLCLEYVVQHKRSGMFWGREFF

KYSVDGTVKRVNGTEVFIFNYDVGESSFVFYTQKGFPLFPRINEIVERIFQAGLYDFWIDSLI
TEKIIEIIVKLEVMDEFVELSLEHLQGACIFYLIGIAISLVEFLCELGVHFL
>BgerIr234
MSIINGYHERITTVLGIIAILFHPSSHAARISDIEVIDQDHILIANCIKAIGQQNFINDMPLIITS
QLRMDWQTIHNLEDLLLEEFHDNSIWSMFISQPEQSNVEKEWNTDKYEKIPNYILVLDYQ
YQEYNKTINDLRQQIKQLKSYRAWYSRGLYVVTMEVIVPQEEVQNLVKMVLNEAWLSYIY
NIVVLIPIADFNMTEEIDIYTWFPYQMPSGSCGVLQEPVLLDKCMITEGNVMFLKNEPLFP
NKVPNDLQGCTVTSMTFMFEPYVNTKDHIWNGTVIGGSEFSMMQSIEKHMNFTLQMRIV
PDITQFPALRYALIANHSIDLMFVALLLREIDFQLFDGTSVYHSERFTWAVPRADMYPKW
KGITRVFTPANWALQFVSIAFVSLFITILFRIRHGKSGEFWNLSKSMLSIWAVYLGDGSPNE
PKEMVVRLFFLSWILQAFAINTVFQAFFTSYQIDPGRQHQIADFDEFQASKPTYCYPQPLE
FFFNPEVMKTMIPHPFCIPIVCLEYVVQHKRSGLFWGREFFKYSLDGTVKRVNGRELYIFN
YDVGESSFVFYTQKGFPLFPRMNEIVERIFEAGLYDFWIDSLITEKRIEAGLVKLEVMDEFV
ELSLEHLQGACIFYLIGITISSVVFLCELGVHFLQILKRKIK
>BgerIr235
MSTINLSYERITTVLGIIAILFHPSSRAAKISEIEVIDQDHILIANCIKAIGQENFIHDMPLIITS
QLRMDWQTIHNLEDLLLEEFHDNSIWSMFISQPEQSNVEKEWNTDKYEKIHNYILVLDY
QYQEYNTTINDLRLQIQQLKSYRSWYSRGIYVVTMEVSVPREDVQTLVKMVLNEAWLSYI
YNIVVLIPIADFNITEEIEIYTWFPYQMPSGSCGVLLEPILLDKCISTEGNVKLLKNEKLYPN
KVPNDLQGCTVTSMTYMFEPYVKTKDHIWNGTIIGGSERSMMQSIEKHMNFTLQMRIVP
DVTLYPAIRHALVANHSIDVMFVALLLRGIDFQLFDGTSVYHSERFTWAVPRADMYPKW
KGITRVFTPANWALQFLTITFVSFFITILFRIRYEKSGEFWNLSKSLLSMWAVYLGDASPNA
PKEMIVRLFFLSWILQAFAINTVFQAFFTSYQIDPGRQHQIADFDELQASKPTYLFHESLEF
FFLPEVLKTMKPHPICMPYLCLEYVVQHKHSGLFWGREFLKYSIDRTVKRVHGSELYMFN
YDVGESSFVFYTQKGFPLFPRMNQIVERIFQAGLYDFWIDSLITEKRMEAGLVKLEVMDEF
VELSLEHLQGACIFYLIGIAISLVVFLCELGVHFLQKFKM
>BgerIr236
MFIMIKALISIITFIIFLTHAERILEDDEEDQEATLIARCIKAIGQQNFINGLPLITTFQLRSDL
QTINNLENILLEEFHDNLIWPMFISQPAQSNVEKEWTTDKYEKIHNYILVLDYQYQEYNK
TINDLRLQIQQLKSYRAWYSRGKYVVTMEVSVPREEVQTLVKMVLNEAWLSYIYNIVVLIP
IADFNSTKEIEIYTWFPYQMPSGSCGVLQEPVLLDKCMSTEGNVKFLKNEPLFPNKVPNDL
KGCPLTVMTYMFEPYIKTEDGIWNNTIIGGSELSLMECLKEHMNISLELRIVSNFSYQWLA
TRAELSVNRSIDAMFAALLINDEDFERFDGSSVYHSERFTWTVPRAEMYPKWQGITRVFT
LANWALQFLTIVFVSVFITILFRIRYSKSGELWNLSKSLLSMWAVYLGDGSPNEPKEMIVR
LFFLSWVFQAFALNTVFQAFFTSYQIDPGRQHQIADFDEFQATKPTYLYPDPLEIFFPSDIV
KIVKKHRYICYPDDCMAYVATHKRCGLLWGREYFKYSIDQMVKRVHGHDLYTFNYNLGE
ESFVFYTQKGFPFFPRMNSVLGRIFQGGFYDYWIDMLIVEKRIQAGIVKLEVMDEFVELSL
AHLQGACMFFGLGILTSSLLFLCELGFHAAHKFRIKNN
>BgerIr237P
MTITIKVFSYITSFMIYFTYADETIMEIDLENQLDILIANCIKAIGRQHFINGRPLIITSHLRLD
WQTKNNLEDILLEEFHDNLIWPMFISQPAQSNVEKEWTTDKYEKIHNYILVLDYQYQEYN
KTINDFRQKIQQLKSYRAWYSRGIYIVTMEIIIPQAEVEDLVKMVLKEAWLSYIYNIIVLIPIA
NYNRTEEIEIYTWFPYQKASGYCGILEEVVLLDKCMRTKDEVKFLENKPLFPDKVPNDLQ
GCPLSSITYTFEPYIQTKDKIWNGTIIGGSEIKLMQCIAKHVNATLQISTTVDSTSSRELREN
LANRSVDAMFAAQLISLIDFELFEGTSVYHSERFTWTVQRADMYPKWKGITRVFTPANW
ALQFLTIALVSLFITNLFRIRYANTGEFWNLSKSLLSMZAVYLGDGSPNEPKDLIVRLFFLT

WVFQAFALNTVFQAFFTSYQIDPGRQHQISNYDEFRASKPIYVYPLQLEMFFTSKVLKSMK
PSYNCFAEECLEYIAKHERSGMFCGREFFZYNIDQTVKRLHGHEFYTLNSDLAQHSFVFYT
QKRFPLFPRINSIVERIFQAGLYKYWVDNIIETKRIEAGIVKLEVMDEFVTLSLEHLQGACM
FFIFGISISFLLFLCEFCVYSVQRLLRKHK
>BgerIr238
MQSEMLTVLVFKVTSIATIFASSQLNLEIIRDVLETKQRTEVFSECIKTIGLEHFTLRRSLMI
STTSELEIDRLGSGERNLAGDLQTKLLQEFHHLQILPTVLSKPPQSLLEEEWNLKNVDTYN
NYEYILILRHQQFAQREIINFLREQIEHVSNYKTWNNRGKFIVAITNTVLHEEATKIVEAVL
QELWSFWIYNVIVMFYTADDISLGSVKIYTWFPYHMPSGNCGVLYEVVHLDTCMRSEENT
TFLKNESLYPNKIPLILNGCPMRIATVTFEPYILTEGDDLTGGADPELIWTITQHLNMSLQV
KIRRSNGTWPGLDNEVQEGHAEVVFAALLLFNLDYALRLELTTLYHTEHFTWVIARAEM
YPRWLAISRVFTPSNWAIQLFSIAFVSVFINVLYRIRYSKSGGELWSVSKSMLSMWAVYLG
DGSPKEPKDILVRIFFLSWVLQAFAINTVYQAFFNTYEIDPGRRHQIATFHEVREADLIYAF
NKPLNPFFRKEVLETFVPRFECEIFDCLVYAAKNKKGAMFAGREFFIYHINEVLREDQTHE
LYVVPDDYGEVHFVMYVKRGYPLLPRINDVMTRAFQAGLYNHWIDLILEPRRIAARLISLQ
VLDEFTELNLSHLQGAYIFYFLGISICIIAFVGELFIYVLKMCISKCAAH
>BgerIr239N
VNTVWLLSLGETYTLIPSNEVLNQHITECITYIAKTYFVLDRTLVFSQISKPSFPNSTALHKL
HSIGRWPIVITSDEREKEHFPKSTEINHDKHFSYIIYLQQCGEVNYAVTQKLNKLLEFLKSIT
SRNSKARYLLVITCEGEIKKKKILYDILKNTSLGKMNDIVVLIPFASGKNTSIIEAYTWYTAQ
LPTEYCGSLDEISLIDSSISNQNENGICHFKTNLYREKATKKYQGCTLRASTINFPPYITLEN
GSVADGLDVVLMNTLAKTMNVTLRYNLYFGKERKGRLLPNGSWTGLREHLHSDKADFIF
GAFLNNYFDYVTFDISTSYYSDDFTWLVKHSETFPRWLGATRVFTSSTWILHFSVIFLTGC
TAFFISNSSIFQRSESWNLSKCILAVWAAFLGTGMPPVPSNTVLRVLLIAWIIFSFATNTVF
QAFFTSYQINPGYEHQISDVEELYKSKIIYAFSNPLDKFLEPNILEKLTPRIRCEAVSCIRLVA
TMKKDSADLTGRDIVNYHREQLRKQENGHELYMFREKMFQLNFVLFMQRDSLFLNQVN
EFIVRVVEGGLPVHWMKSIMEKKKIEANMLGLHTLGDDYVDLQMSHLQGAFIFLVIGIAFS
ILSFIVEIIMFRIRNRIS
>BgerIr240P
VLGLITIFMVVLSLGERIFKLEVTYKDDMIASCIIAIGQQYFIHDMPLIITSHIRTEWQTTHN
LEDILLEEFHHNLIWPMFISQPAQRNTEREWSTDKYEKIHNYILVLDYQYQXYNRTVNDL
RVQIQNLKSYRAWNSRGIFIIAIRTNVPPTAVQSLLKLILNEAWLSYIYNIVVLIPKLVYNST
KEIEIYTWFPYQMPSGSCGVFQEPVLLDKCMRNEDNVEFLKNEPLFPNKIPNDLQGCLVK
SMTYLFEPYIQTKDKTWNGTIIGGSEIKLMQCIAKHVNATMKINLTADFTSWRGLREQLA
NRTIDIMFAAQLISLVDFELFDGTSVYHSERFTWTVPRADMYPKWKGITRVFTPANWTL
QFLTIAFVSVLITILFRIRYAKSGEFWNLSKSMLSMWAVYLGDGSPNEPRDLIVRLFFLSWV
FQSFALNTIFQAFLTSYEIDPGRQHQIADFDEFQASKPTYMFPLPLEMFFTSEVLETMKSR
PYCYPDDCLEYVAKHKRSGIFCGREFFKYHIDQTVKRVHGHELYTFKSDIGENSFVFYTQK
WFPLFPRINSIVERIFEAGLYNYWIDNVIEIKIIEAGLVKLEVMDEFVTLRLEHLLGTCILFVI
GILTSLVVFICEICIYTMKKLKTKT
>BgerIr241P
YSQNTRYILIQKEKSFCKMYYFDCIKAIGQQSFINSMPLIITSHIRTEQTIHNLEILLEELCDN
LIWPMFISQPEQSNVEKEWTTDKYEKIPNYILVLDYQHQEYNKTINDLRQQIQQLKSYRA
WSSQGKYVVVTMEVGVPREEVQTLVKMVLNEAWLSNIYNIVVVIPIADFNSTKEIEIYTWLP
YQMPSGSCGVLQEPVLLDKCMSTEGNMKLFKNEPLYPNKVPNDLQGCAVTSMTYIFEPYI
NTKDHIWNGTIIGGSELSMMRSIENXMNFTLQMRIVPDVTQFPAIGYLLVANPSIDVMFAA

LLLRLSDFQLLDGMSVXSERFAWAVPRADMYPKWKGITRVFTPANWALQFLTIAFVSLFI
TILFRIRYEKZGEFWNLSKSLLSMWVVYLGDASPNAPKEMIVRLFFLAWILQAFAINTVFQ
AXFTSYQIDPGRQHQIVDFDEFQSSNPTYLYHEFLDFFSSRGPENNET
>BgerIr242N
VLGFIAILTHIPSHAARISETEVLGIIAILFHPSSHAARISEIEVIDQDHLLIANCIKAIGQQNFI
HDMPLLIKSQLRMDWQKNYNLEDLLLEKFHDDLIWPMFISQPEQSNVEKNWSTDKNEKI
HNYILVLDYQYQEYNTTIDNLRHQIQQLKSYRAWYSRGIYVVTMEVGVPREEVQTLVKMV
LNEAWLSNIYNIVVVIPIADFNSTKEIEIYTWFPYQMPSGSCGVLQEPVLLDKCMSTEGNV
KFLKNEPLFPNKVPNDLQGCTVTSMTYMFEPYVKTKDHIWNGTIIGGSELSMMQSIEKH
MNFTLQMGIVPDITLYPAIRHALVANHSIDVMFVALLLRGIDFELFDGSSVYHSERFTWAV
PRADMYPKWKGITRVFTPANWALQFVTIAFVSFFITILFRIRYEKSGEFWNLSKSLLSMW
AVYLGDSSPNAPKEMIVRLFFLSWILQAFAINTVFQAFFTSYQIDPGRQHQIADFDEFQAT
KPTYLFHESLEFFFLPEVLKTMKPHPICMPYLCLEYVVQHKRSGLFWGREFLKYSIDRTVK
RVHGRELYMFNYDVGESSFVFYTQKGFPLFPRMNQIVERIFQAGLYDFWIDSLITEKRMEA
GLVKLEVMDEFVELSLEHLQGACMFYLIGIAISLVVFLCELGVHFLQKFKMKIK
>BgerIr243N
IKIVAIIVISGNFLNTSIAVGFSLVDTEQNTLCIDSCVLAIIEKNFLPSKTAVINLENRKLQNYL
LPRLLNVKWPVILSLSIPLAENFDPFFWDPENNIEFGGYVIILTVQNKKHDEIITDLLESITF
LQSFLSWNSRRKFIIELIDSISLHERESILKLILSQLWKRNIVEVVVLVELSRYKVGIYSWFPY
QLPWGNCGVLENVIPLDYWIFNNSRGNFLHNTDLFSNKIPKTMSGCPIRVSTAHFPPFSII
DNKNGPSNSITITGLDVKIVQAIAKKMDMILDLIPVYDPYPWGKFENETWKGLRGDLLYD
EADIAIAGWAINFKDHLLLDDTERYFTDRITWYVPRAKPNPRWASIARVFASLTWTIFFV
SIFISAFIYWLLSRILSIFEVVLSSKSKINSVLEAWAIVLGVSVPTQPYYSSLRLYFISWVIYCF
GINTVFQAFFTSYLIDPGYEHQVSNVEELVESNLRLVLSVYLDEFFDSELLSNPSRKLIMDS
PQQCIDFAASNDGVSTMLSRSFVKSEIDGYLAKNITFSLSNFVEDVTNIHLVMMIQKGSPYV
ERLNDIIMHLVEAGIPVKILNEIVNLKHYISAESNDYVVMSVSHLESSFTLFAVGIAFGIVSFV
LELLTAALK
>BgerIr244N
CVTTFIFINLWKTNGNIHYRTNENDAVINCLLQICMQYFSEKHTLAVSYDNSIASTISNTDT
LMSTFVRLHRSSRWPIVVLNLNFNATPRDPLDWDTTNNDKHGGYIVFVDHKITRQIADL
KSLSAWNSRANFITIPIRNSQTLSRDEVAVIFDELWNFKVSKVALLQLTLASGTVSVYSWF
PYKNPSGNCGQILAAVVIDNWKQDSGFERFSNNKSPFSDKMSNGLSLCPIKIGTANYPPFV
YIPPNVHGVTHLINIDGADVKLLRLIIQILNASSTVKVARESIMWSPKLANETWGGLKGLLL
YNQCDIVITGANTYNLHLYFDHTYTYYDIKIKWYVSVAEEYPRWLSSFRVFELESWIAFIST
IIISSVVLRFLDACSNIKSRYCHIRNCFLDVWSIALYTSIDRIPNSISIRIYYICFTAFSFVMNTL
FQTFVTSFFVNPGSERQASSIEDLFDFGYEFALYEDLNNVINDELQRKLHPRINCSIPVECR
SFAAHRSKAATPANSVVMRYLGNRFISQNVSVPTFAFDDNMNYFHVVMFLQKDSPFLET
VNRIVIRLFEAGIPEKFYYDMAFSKRLLERTAIVEEVTQDYVAVSLPQLQSGFGFLLLGLAFS
VVGFLLEIVV
>BgerIr245P
MEEIKSLFLQVAAMLVTNIYLLLALKSESLNLIDCIATISRQYLDPTHILTFSKSIDRQITIMH
NFTYGEYDIQKTIYSNSIFEDLILEMLHKITRWSLVISPIPYNYEHEKDYDDINGPKHGSYILI
SSCHTVDNMSDVTDIQTQILEMSDLVSWNMRARILAILSCPRYSIMETHFAVKSLFEIMRD
IPLFNVVILTQEPTLQKVDVFSWYPMKLPTGKCGKLEDVKLVDTFHNGNFIHGVDLFPEK
FPKDMQQCPLTIGVRFFPPYVFEDNDDILGIGGLEPTIISYIANYLNSSVKIVRYPRNISFFGA
GMYIDTKYADIVIGNLQYDYIFLVRYRYLFPHFIDRYTWFVPRAEQFPRWLCIIRVFEISTW

IVLFLIMLFAAFVNYLLILFHPIEPETRKKFTSFTKILCNTWSILLGIALPKLPKRISIRVFFIL
FVFFSFTVNLVLQTYMMTFILNPGYLHQMDSVEELVESDLCVFLSYLFIGYLEYIYFSLKSV
EFIMDEEELMEKALAQKDSAIFTSRYNVLSYFTDIEVLNNRYYEFSKYSLHLCSAFMVGRG
ZPLADKVNSVIKRLVEAGLTEKIMEDVIRRRELGQYNIEDEKLMPLAITHLQVTFIFLLIGFC
LGLIVFIIELSFERVRDFVVRFLCIFSKCWMVFQKRYL
>BgerIr246
MVIVIIDGDLILNNEDYKVIDCIAAIGEQYLDANHILSFSKSIDHFDNVFVNSTYGGYMDRK
PMYRNNIIEDLLLGMLHRKTNWFIIISPMYYNFELNKVVDNPSYPKHGSYIFTSSCHFGDN
MSDITDIMQQMYQMYSLFSWNERAKILAILSCPRDSDNETALAVKALFKRMKSASVYNAV
ILVPQPSLGKVDIYNWYPYQLPSGKCRELHGVELVDTFENGRFIHAVDLFPNKFPRNFQGC
SLIIAASFYPPYVFEESEDDGDGFMWIGGLEPRIISYIANYLNANVTILKYPRNISFYEVARYI
YMNQADIVIGNLQYSYGFLLFNKYLFPHFIDKYTWFVPRADQFPRWLCITRVFDLSTWIVL
FLIVILTAFVSYLIIYLQFSLIKIKKESASFDQILCYSWSVLLGIALPKLPQSMPVRMFFILFVIF
CFAVNSIVQTYLITFILDPGYLHQMDSIKELLDSNLQVFMSDLFFGYLDYIWYSMESVNYIN
DEEELMEKALESRNSAIFTTRNILSYFTDIGKIGRRYFEFSKYSVHLCSVFLVDRGWPFVDI
VNSVIMRLVQAGLNKKIMVDVLNSRKLKRHHFGNEVFVPLAITHLQAAFIFLFIGMILGLIA
FLIELSIQRFRFYVARYTDHDKIPSNFTYLP
>BgerIr247
MDSSRLPYVLFVMIKFIRGDNDYENQLRNALETITNKYFSGNNIMVSHMEFDLLGNFPTH
EGNLEFLNRLLFELHNYERWSLMADQPDNSMFRHKLDNLKCDNYIIFTKYEDSLVNLEN
QLLRLIDSDSWNPRAKFVVVFFNYKYSQCAMNVSRETLTLLWERKIYNAILLITGTASFRN
IVNDLNPDAIHVYTWFPYQNSEECNEVKSVILLDEWVFNLGKFIQNAELFPPKIPSNLSKC
NLIVSTMTVPPLIWEPITAEDGEIVYKDGLEIHVLYVIAEALNANLKFLAPEETFWVKVTN
KTMMYQIDELVDRRADIAVHGLVQRLEFKQYGDFTRYYYCDTIRWYVPCARRLPSWKNII
NVFYPDVWIAGILTILSVSALFKLLAIISLVFKLAPEKDIYRKVYSSLTLTVSVFLEISVPVMP
KTTSVRIFFISWVYYSFAVSTIFQTFLTSYLIQPVYEQQISNLEELFNSDKKYIIYDFYDDYILE
SYGKFGTELLSKRILCQSLVSCYESIHSNVPTGEVAVLWSDFLIKYYTNNGLVDKESGRRYL
CEIEEMPVLTANIVFYLQKDSPLLETINIISSRLVESGFMANEVNLLWNALRIKEKRILFPSL
ANDYFNLSFEYMQSIFYLLLFGYFLSLVIFLFEELHYYKILKTT
>BgerIr248
MKLFSFFTLVILLKGCQPSNDVGLLTTYEDSVIKCVTTIANNHFSSQHPLHVSVYKPQVKS
MERTLMKNGMHPKPDSVLIDYLLQKISEESKWSLQVTILGSTGYATVTHEDPVKKSSYLIF
IWEDEEEIIIESLIDQMEELHFDSSWNSKAAFLIVIFVQNSLSGSSIALIAVENLWKYYNVLN
SVVLLCPNFPNFVNMGSKHTSSNNLQLFTWFPYISKDHCDQVNEVVLIDQWIISRNEFIFN
LPIFIDKLPKNLLGCPIKVSTSEVPFQVKLISQHTNRNNKTVYTFTGLEMLCLFYVKDALNF
TLEFLVHAGNDHFNSHLSMITEVFEGKSDVAVSSFPLHAKLLEMVDPTVTYFPCYLRWYV
PCGRSVPRMAKLVHIFTLSVWITMAIVLLSTISTIWCLGRTTNKSKVKELSSYRKISSVIYNV
FSVIFGVSVPQLPRTSKLRAFFSLWVWYCFAISMIFQVYFTTFLVDPGIQKSIRTLEELLNSG
FKYACNKEWDDFIKFTTPEYYSKINLEKVYCKDFECYEFVLNSSDFLTVSSSPLFAYYTSSS
LHTTNLPLCSIEEDIFRMDVVMYLQKGNPLVKYFNSAIRALMEFGLLSKYHQDMKILLKAE
GISSIYSSDEFSVTDYFVFSTAHLIFAFYILGMGYSLSSLAFIAEMLYAAEFQFRKC
>BgerIr249
MAMVCYSHLYNQITMKPHILAIFVYLFPISSSIQESKFPTTEELHLTRCVKHILYKHIEPIPG
NLVLISLPFKEYDTGQYALQYLLTTQIADYMMYSVHEELIWPLQVVFQSDNSQDQEYTDR
KDGICCYIIFAWSSDDHTNTIDSIIYQMGGLATSTSWNPQALFIIALTDKVVRTPKDVTLKIL
RELWISYKVLNSLILINPLTESHNVFEIYSWFPFRDQNKCDNVQESVILDRWSIDAKGKFV

EENNLLPNKIPSNLHGCPLQVAVLSIKPYSEIFPTYQKDINKTVYIIDGYDGNVLLMIFQVM
NISLQPTADSLTNTNVSLFQRASQALSNLMFGAELAVGSLPIHPVAAYYVEFTFQYQESIFK
WFVPCPKPYSRLEKITRIFSIMLWITILLIFIFGSFFLWILAKYCKTMKMKELQTYAKLSNCF
CYLLSVTLGISVWKMPCSSALRSFFIAFIFYCFSIGIIFQTYFTSFLVNPGFHKAIKTMEDLLS
SSLDYGYDEIFDFFVEGTYPKKASDHRIKCSQDECFHRYLKDDFATPGTDFHTKYLLLKSG
SNKLICSLEENVVSKTSTFYLRKGSPFKEIFSKIIMHMTQGGILKKIEDDYLFKVRMEHSEIR
NKFYEDNVNNNDYFVFTTSHLQVAFYILYGGYTLSCVQFLCEILYMIAIHIYEDGFKCSLSVI
SRE
>BgerIr250
MLMYTIIMLITFIIICGGQKRFNDEGLLLCIKKITHLHFSTGTSIHVSIPLREQYSWNNTNVAI
QYPTQMVEFLLKDMQEELLWPVQVSNRKAEKVERSMECSTKLYNYIVLLWEETSEDDIV
SNLIYFMEDIEFSGILHGQAKFLVVLKVLPETSVKILTKSILKELWKRYQIINVILLLQVKFLP
SSVEILNKEVSVFDLYSWYPYKSRSICDDVSSAVLVDRWFLVNENVMSNYVELYPKKIGRN
LHGCPLKVSTTAAGLGVFETGTTINANIKNNSKNNVSYTGSEIQCLLYIANALNMTVYYLP
PPLVQPTHVNTHTAMLMKVIEGISHVTLSNFPLFDFINKLADATISYANTHIKWFVPCSKP
IPRAGIIVRIFSMSAWLLMTLVFVQVTTVLWLLAKNSNRRALTESNAYNTIDGCFYSVWA
VTMGVCVPQMPRTTRTRAFFLLLVCYSFVMSTVFQIFFTSFLVDPGIRKQIDNFYELLNSG
YTYLYEHNWDYFVNLASNEYHEQILLKKEECTDDDCLYAMLKAADKVTISSTSYVEYFSST
VLHVSSEHKPFCSIEEPIYRMDIVMYMAKRSPLIEFFNEAIRRQLETGLNYKYEWDMKAQL
RLHYISLENYTSHENINAPEVIEMQYFVISLLHLDAAFYILLVGYSFSFVSLLLEILHTSMNLF
>BgerIr251
MFRGFAIILVLPIIYLVNMEDNIITLKMPNIVDLHKIIEMKHPNVMACLLGILEKYFLVERSV
LIILPEFDGALNDTAVEVLESTFKRTILPVVTSTTTNRSVWIEIERDGGYLILLWGMEDEESI
SDYLLDLLEFLQHTPSWNPRGKFIVAVSESSNTSSISTAKYILKTIWNSSYVVNIVIFVVSSL
NNYINLYTWFPYKSGQCGKVENVVLLDICVSERFLWSSELFTSKIPNNLNGCALRISTWDF
APYAILVDNYTDDNGEIIYKYSGFEVEYLILASEAMNMKLVYRHHETGPAINTFMGQIQDI
ADARVSDIAVGAFPLHPFILYYADATIPHMFTSVKWFVPCAKPMSRMKKILHMFRPSLWI
TLSLIFLLAAIVIWKTAKRADPEDYRKFIDCVYNVWAIILGVSVPKLPRSSSLRLFFLVFLLF
AFSLNILFQALIVSFLIEPGYEKQIETFDQLVESGLKFAEHPLMKNLFIFTSYQEQKRLKSVE
CLDYEACLEDLINGESFTTIGTQFHAEYKAVTLPGSGTPCSLDADIASGGVVMYIGKGNPLL
PRLNSLIRKCLEAGLGIKYWSDYTRAWKLKTEVDLSSGEDGYFVFTIIHLQVPFMILGLGTT
LGLLMFLSEIFFRIFVMLERSEMVV
>BgerIr252
MAGLGTLLLFHTIFSVPKDLVRCITNIIYKHFHPGQTILTSLPTKGTQVDQILHSLHQLSLW
PFHISAIGNEILDRTFEDVTKHQGYLIFTSVTEDDIIEDIVNQLEEIKMSPYWNPRARFLVVS
TTKSNNIIKSMLQEMWNYYEISNVVVLVGSELYTWIPYLSHEVCTEVSPFVIDKCGKTSLN
KLLLYREKIILKYHGCPLRVMPIETLPIYVIEYQKIGSDGKPFYNYAGVEIEYFKAVMSVLNF
TVIYKPKRATDALTSRISALSNLVSGKVDIIFGGMAFHPKAFDLASPTWPIVVEVMNWYVP
CGLPADRVEKLMSMFTFSVWGAIAVTFLVCSITSWQFSRKALMENRWLRDASSSFYSVW
GLTLGVTLPHQPRSSAIRLLFTFITWYCFAVNTIFQTSFTSVLVNPGVKNQIKTLKDMNSSN
LVYYFKGDMDNYLNSTFPSYYMSLKIPRKECQFQESCLLEYFSKDNAAIVGYKISTEYFLLG
ALPASSDIPQLCSLHETINYLYFAMYQVKGNPLIESFNFVIRTMSETGLSLKLERDVKSSFRY
QTWPHMKLDLDEKYFIVKDNIQFFVFRIIHMTFAFYLLSVGLAISFLLLLGELIYHAGAF
>BgerIr253
MESRIFIKLYSIALCHVLLIRLPYAVCLPNDNILCVRDVIYNYFIPGQPLFVSLTNTHENAQRI
LQNVHQFILWPLVMSIPGYVRADVDFSDYAKHGGYLIYTSQNEHEVLELLINQIEEVKKCP

CWNPRAKFLIVTSENTSLDLAQEIAQELWTSYKIINLIIMINFNLYTWMPYQSPEVCAKVM
VFIVDTCKMSEKTILRNNVSLYPEKIPKFFSGCPLRVTPVQSAYISVVPFINEQNETDYFGIE
FEFFKLVIESLNFTLLYRPSVSGDGYYVRLNALADVALGAIDVTFGSIPVHPIAMAYADPTV
PYVEEILKWYVPCGKPYGRIEKVFLIFKLSVWVTILIPLILVATFIWIFTRCKSGERRILSGFA
KCVFTVWAITIGVSTNGIPRHPSLRIVFLALIWYCFALSTVFQILFTSILVDPGLRKQIENIDE
LYRSDSIYYYNFGLDSFMKAACPEFYENIPLQKKECTGDEGDCLVEYFTQSNFVIVGFIFYT
ELYLLGAIPPSKSAPKMCTLNENIYKLMYALHFEKGSPLIGRFNYIIRRLIESGFFDKLFRKM
KRVLRYFDWNIINGVDEELDQYFVFEFHHLKLSFYILLIGCIFGFGSLLLENVRGRRLKQHF
LRN
>BgerIr254
MILCCILSFIIMAVSIQNIPTCIINIAQRYFIPGCPVLVSTTSHNSDYERFLQTLYVKFVWPVY
VYSKNLTLANFEYRTENRNYIVLTSKNVEVIEHLIQTLEEIKNSVGWTRKIRILVVTEEGTK
KLAQNIVNRIWMHFRIVNVMILVGYKIFIWKPFQSYSLCANVDLFSTVSCQMLNTHSTALD
ADISPDQMIRNFHGRTFTFIPVEGRATVIRNETKDGYTFTGLEAGIFNLIIDVLNMSVTYKP
LRNNNSHYARVHALEELSNNEFDGTFGGQVMTSTSISSYDSTIPYMLEPIKWYVPCGKPLS
RTQKVGTIFSRCLWLSIIVVGISIIHVFRSLAITESQESASYKTLTSCFYLVWAVTLGVSVPEM
PRLWKQRLVFITFVWYNFVLGMVFQTFFTSILVDPGVSQQVRNFQELQETNFTLYIDYRL
EKVFNQLVLPNYGRIKLRQKYCSHDYICIVEYLTIPDVTFLSFESYTRVTMLELLPPGHNVP
KFCVMDETPYMFYFSIHLKRGHHLASVFDRIIRLLTENGLLNKAYKEFDNYVRYEKVKNV
PALHIIENKESESFIFGISHLQLAFQFLSAGYFLSGISFICEVLYTMYHQKLGHPNNVYLEKH
YC
>BgerIr255
MALLETLLLLQSLVLMPEDLATCVVEIIYRHFTSGRPILVSQPIDGQNINGILHLLHNSSTW
PFQVSSPGAETLYSEFEDYAKHHSYLIFIWISDEDDDIMEMLGTQFEDIEVSSCWNPRANF
LIVIYGPLSEEREEVAQSIAEELWSTYKIININILLPEFEYEAEQFDDNYDSTEVDQDAYDEE
NEYTKDKIISTKINSFELYSWAPYISQDICAVVHIYLSDRWLVTDQKFEKGVDLYAPKMSN
NFYGCKLSVSPIETPPVYVMATNYTDEDGNIHYEYFGYETELMKFIVQFLNFTIDYRYIPPG
DPYDTRVRILGDLNMGLTDLAFGCFPLHPIVITYADPCESYIADDVRWLVPCGKPIPRMQK
VGDIFSPSLWCALGLVIIISAGVMWQITKYSKGETSAYKTISICLLNLWAIMLGAGVTDMPR
THTQRTVFLFLVWYAFAINLLFQTYFTSILVDPGISDQISSREQLYESNLEYHYQYGFDIYIA
RAYPEYHNEIPLTRKECSYKDTCIVDYYGDKDIATIGSQLYTECFLLVALPADSDIPQLCLIE
EIVAQFRYSMYVGIGSPLVHPLNMVLRRIIESGVKNKIEKDARDFLRYHKLSDINIELDDSH
FSKDEVIEYFVFAMSHMKMVFYMLGLGCALGGVLLLGEILYFKTKTKI
>BgerIr256P
MVRNFVLLFPLMVYSHQNIASCVIITAQKYFIPKQPVLISIPSNSSHYSTILQELHIKSLWPL
HVFSSNVSNINFSFRPVNZNNLIITARIGEVLADLKSRFQLLKNSVRWNYRTKVLVVAEMIS
KNMVEEIIETFWKQFRIVNVIVLVGFELYTWNPFQSDGQCADVYVFPFGNCFIVDGLTVLN
TSGTELSSYGKPRNLHGCTFTFIPIESKHNVFHNDTPEGSNLVGIEAGIFELILEVLNMSVVY
QSIAYKNKFDARVQALRQLSDMDTDGTFGAQIMTYTGILFADATVPYIEEPLEWYVPCGR
PSSRIQKVAAIFSGNLWIAILAVFISVSSVMWWIAKNEQEESMSYKILVSTTSLIYAITLGIST
KEPRLWKNRLIYIVFVWYTFAMGVLFQTFFTSILVNPGTNEQVKNFQELQETNLTFYIEPE
FETFFNRIVLSNYDRLRLRTRYCTNNCVFEYVSLPDVTFLGFKTYTKLAMLEVLPPGITIPK
LCTMDETPYKFYFNVQFKKGHFLVKEFDRVIRLLIENGLVLRSYHTFTTYVRFVKLENLKL
VKIIDIENSQYFVFKLFHMQLAFEILFVGYTIGWIMFSFEILYSKFNSHKSLYM
>BgerIr257P

MYHRLIILFTLMTSSHQSMSSCLISTAQNYFIPYQPVLISISSHYFPHHDILEAIHQKSLWPL
NLFSSNTSNINFTFRPVNVNNLLVTSIIDGTVDDLKTRFQQIEYSVCWSHRTRILIVSEEGSK
KLVQDIIETFWKQYRTINVIFLVGFNLYTWNPFQSQDQCANVRVFSFGNCIMTDGLTVLN
TSGTELSSNERPINLHGCTFAFIPLESRHNVVCEKTEYGCRFIGIEAEIFNLIVKVLNISVEYY
TLANKSNFDARVQALRLISDPAIDGIFGAHIMTLTGIHFADATIYYIEEPLEWYVPCGRPSS
RTQIVVAIFSGTLWIAMFAVFMFVSTVMWWFAKTEKGKECHTKLWXSVFLAFASTLGISA
RAPHLWKNRLIFIMFVWYNFAVQVVFQTFFTSILVDPGINEQIKNYQKLQETSYTFYIERE
FEAFFNQFVLSNYGRMTLKSZYCSNNCVFEYVSLPDVTFLGFKTYTKLAMLEMLPLGISIP
KLCSMNETPYKLYFHVQFKRGHFLVKEFDKVIQLLIENGLILRVYDTFTTYVKSVKIKSSKL
LNNFNTENNEYFIFKLFHLQLAFEFLLVGYILSCTSFALEILYDISNLCFNMQV
>BgerIr258P
MSNKIVLLLILHLTLSEQQIYKLNMNQIHLLECITSILYRYCPHGSSLMISMPNELNESYSKQ
YLKLDTSLNTNYFDEITIFIEMIHQKMLWLLCLSSPSDKLEFEDGNKHEVYVMYIWDDDN
SDSVRKLDDQLGAIFRLNHRAKFVVIVRSLFHSGLEQEIAEIMWYSYKIWDVVIIFDEFTGK
NIDTWDNTSESYNLYIWLPYQSANNCENTNPFVIDTWTNIDGGKFLNGNSLFPNKIPNNL
HGCPLKVLAISTMPTMVVLNNYTDENGITKQNYTGNEFHYFDFLAKAVNASLIYIPSPLGD
NIAIRWETFSTLLSGKADLIFGAFPLHPLLTPFADPTLSYLSDVMRWHVPCGKPIPRMDKV
TKIFAPNVWLALSFVALAAVAIMWLSANHLPMDEMRTYSSISECSLAVWAIILGVSVSDLP
RSNTLRHVXSWSSYAISIIFQTLFTSYLVEPGLGRQIKTLEELFSSDLTYNYRNELDEYLKMS
SPEYYRSISLTRQKCENNDFCVLESFTNDNIAEVFFQLTTECLLLSLLAPEKELPLICTIEEDI
LRVYYAIYFYKGNPLVQTFNGKIRQLFETGLANKIHSDLKLKWRYTNWSHLSNHSFNEIES
DQDGYFVFSLTHLYFAFYIIVMQCFIGLFSLILEIIHYKFSKRL
>BgerIr259
MKKLKIAIFKLILKYYWLCMTSNALYFEINQYVEENSVVKYIMATVYRNFDPEWSVMLSIL
NEVPENYQNNMDITLSIIRELNWGATWSFQISCPGAMEPDLEFVNNVYHNGYIVFTWPD
EDGEITGNLIDQMESLKMSTTWNSRALFLVILLGQRSNTPDTVAQEVAEELGKNYNAINIT
ILVCQESIDMDIKTHSSEKKYASFKLYSWLPYQSSDICSEVKVVLISHWEMSSQETEENVTL
LQRKIPNNLLGCQLSVGLIYFEGGFVQFDSVVDEEGNIDYNYWGLEFELYKLVQQALNFTP
VYYRYGPGDGLEIRYAAMRDISNGLLDIMFGGFPWNELLVPFVDSVSPYLQEIVTWNVPCS
SQLSRMEKVSKIFDVSVWSTMFLTFCLSIFTIWLIARSTSRHAVEEHSCYRQVAYNLYNSFA
ITLGVSVSKMPVTGRVRMIFIIVVVYCFAISSIFQTFFTSILVDPGMDTQLKTFKEMVESGFL
YYYNPTTDNFINITDSEYHDSMILEKVECLPNKFCLEYVQGKNRVRVAFIFLSEIVLLYTIPP
GEEIPKLCTLDENIFILHYSLHLAKGSPLLGLIDITVLYLKESGLVDKTINDFKNKVRYMVPN
DNETFELSFQENSEYFVFSLAHLYLAFYFLLYGYASGVAILILELLVNILNLKYIFENYM
>BgerIr260
MFPGIIWIIPALISTTDDIGFCVYNIAERYFTEHYPLYISWEYYVNSLLYKLHDAALWTVKL
TFPEKEQSTFEFSNEDKQYNYMVFTSGKENLIDQMEELKMRPNWNPRGKFIVVGSHLNV
QDVAQELWTNYNVVNVLILNSDFERMYLFSLEPYQCSKVDLLLIDSWHVTEKSSTHNSSS
LSTRKTQTDYQGCPLRSGAMHCPTFVKPLNGFDYWGVEADLFKFVARRLNMTVIYEPFA
YSDSYEGASKVLEALSVGAIDVAFGCLPWHPVMNELADTTTTHLQDTLKWMVPCGKPLS
RTTKIGQTFSPPLWTTMFFVFVSVVITLYLYAVNSSDESRVYRSFSNCFYFVWAVTIGISVPI
QPKTSKIRLVFIILVWYCFVMSMVFQTYFVSNLVHPGFGNQVGSLEELYREGYTLYVQSGT
ADFFDEALGNNYDEIKLPIQQCPFRACLDEYLNLPKVVTITCRIFIEYQLLERLPLGKNAPK
LCMMEEDMYVIFYGFHLQKGSPLLGSFNLMVRRTLESGVYEKIKSDLKDVFRYTQLNESE
MLNVPLEDIEEYFVFSMVHLAIAFYVLVFGSLLSFVLFLFENVANLVHKTNTSGV
>BgerIr261

MVSEAVILPWILPSLLAVPDDISTCVSDIVRQHFSQLHPVHMSLSSTGYDIDRLLYALNTEE
NWTLQISSSGDQTPDFLVEDLFKHQNYIIFTAQQEEDVLEELIDQLEYIKMKPDWNARAK
FLVVTSRNGSEELVQEIAEEMWKSYSIAHLLILIPTYQRIHMYTWRPFQSDKLCFEISVSLV
AVWKQHIGTIYLTPNKLLSDILPRNFHGCPLNVAAILGSTYAFIVNNSKEVEYHGVEVDYL
RFIADVLNFTIQFYPPEVGNNAELRKSALVHLTEGVIDIAIGGFPWHQYLVHFADTSTSYIE
DIFKIYVPCGKPFSRIYKVAHTFSMGVWIAVFSGLVIVSLTLWLLAQQFQENSNPYKSVFSC
LYENWAVLLGVSVSQMPQNKLLRLLLIHFVWYSTAMTVVFQSFFVTNLVEPGYDSQVNN
LEELHLKKYDLCLPPGIDELLTRQIIPNYQEITLTRRHYNYTKCLIQYFTVSDISMIGITLSTE
LRLLSILTPNKEPPEVCVLKENLYKLMYGQQFVKGSPLLVAFNRLIRTGLESGLFQNIMNN
FKNELRFMHSNDSESLRLGNDEVVQFFVFSINHLFIAFLALGTGLSFSFIVLILEYLVIKIRNC
RNLSISKEIRFLE
>BgerIr262
MDALTIYQKNSIEESLAECIINISTKYFDEYKPVLVETPKNWYSNQYPYVEYGGKFIELLHK
ANQFPLIIYGMEAYLARLKVHVGSYIVFMPPVLRDIDNLYIEHMLNALLKWAYNYKGKVIL
ASLEKMTSQEIRNLLTLALNHELLHTILLKPRTMSQKPKNNHLDKIDIYNWSPNDQSNICL
RTVDKINHSDIWISHTKSFSVNTNLFPSYRKIDFNGCTLRTHFRDAPPFTFVFPKERLTAG
TVVEIFGCIKKYNNCKINYIPKKRQDTLIVHIYFPVLQDPIHYDQDELRDQLTYPHFVSEFT
WYVPKGKQHPRWTSLIKTFTSLVWYLVLLTFVFGSCTMWLLRKSVGRGSNPTNQNNHG
FLVDAILTHLGYGIIYRYKGFIPSTFLMFWLYYCLIINTAYQSAFFGLLINPGHIPTLQTSKQV
EESPLIKQSFGFMLTDSQFWSRFLIYKVCQNDECFKAFTENPPRALLCDTWHAKYHISRH
GENRYVPINEIVGTELISLVVYQLRGILLDVFNKVLGRLTAGGVIVQMTKEINSKIPYMRKN
LIIGTAFSLTMDHLQVAFILLWIGYVLSLSVNVDEFFVYFVINWYYLLCIKYQVQFITLL
>BgerIr263
MLHLKLSFLLFYILGCSWALLTLYKENSIEEYLAECIINISTKYFDEDKPVLVETPTHWFKYI
YPYNKYGGKFIELLHKANQFTLIIYGSEAYMAHLKVHVGSYIVLMPPVLRDIDNLYIKHML
GALSNWAYNSRGKIIVASLEQMASQEIRNLLTMAMKLELLHTILLKPRTLSQKLKDNHLD
NIDIYNWFPNEQSNICSRTVNKINKSDTWISQTKMFSVNTNLFPSHRKIDFKGCTLRANFE
TYPPYSFVYPEIRGTAGPVVDIFVSIMKYINCIIIFIPPMGEDEYILHLFFPVTLDPIHYAQYER
RDRLTYPHMVRDITWYVPKGKQHPRWSSLIKTFTSVVWYLVLLTFVFGSCTMWLLRKSE
RLGSNPTNQNNHGILVDAMLTHLGIGVVYRYKGFISSAFFMLWLYYCLIINTAYQSTFFGLL
INPGHFPTLQTSKQVEESTLIKQTFGIQNSQSPFWSRFLKYKYCQNPMECFEALSKYQTHA
ILTDTWQAMVRVSMLGDNIGLYVPINEIVGTELLSIEINQLRGILLDVFNKVLGRFTDSGIIN
QMIERINRQYSYMRKNPAIGTAFSLTMDHLQVAFFFLLVGYVLSVSLNVFEFLFNFVTNW
YYLLCIKYQLQSLKLLLL
>BgerIr264P
MFFKLFFLLFYTWGCSWTQLILYQKNSIEECIINISTKYFDKDKQVLVETPTNWYSDQYPY
VEYGGKFIELLHKVSQFSLIIYGMAAYIARLKVHVGSYIILMPPVLRPIDNIYITHVFAALNF
WAYNYRGKVIVASLEKMTFQEIRNLLTVALNHELLHTILLNPRSLSIKSKDNSLVNIDIYN
WSPNEQSNICLGTVDKINHPDIWISQTKSFLVNTNLFPSHRKIDFNGCTLRTHFMDTPPYT
FVFPKQRRTAGTVLEIFGCIIKYNCKINYIPLTRQGTPEVHIYFPILLDPIYYDQDELRDQLTY
PHLVSEITWYVPKGKQHPRWTSLIKTFTFSVWYLALLTFVFGSSTMWLFRKSERRGNHP
THKNYHGFLLDALLAHLGYGTIYRYKGFIPSAFLMFWLYYCLIINTAYQSAFYGLLINPGHI
PTLQTSKQVEESALIKQSFGIMLTNLPFWSRFLKYKVCQDDECFMAFTENPPRALLSDTW
HARYHISKHGENRYVPZNEIVGTAYLSIHVYQLRGILLDVFNKVLGRLTAGGIIVQMTKEIN
REISYMRKNPVIGTAFSLTMDHLQVAFILLLIGYVLSLSLNVVEFFVYFVINWYYLLCIKYQL
QFITLL

>BgerIr265P
MLHFKLFFLLFYIWRSSWALLTLYQENSIEESLADCIINISTKYFHKDKPVLVETPTNWSP
HHYPYIEYGGKLIELLHKENQFSLIIYGIDAYLARLKGHVGSYIVLMPPVLRDIDNLYIEHMF
EALFMWAYNCRGKVIVASLEQMTSXNLLTLALNHELLHTILLKPKTVSINTKDNHLDSIDI
YNWFPNEQSNICSRTVNKINNSDTWISQSKSFLVNTNLFPSHRNIDFNGCTLRTYFPDYPP
YTFVYPEVRSTAGTVVEIFLSIMKYINCKISYNSLMREDILKVHIFSPVPQDPIYYGQRELRD
QLTYPYLVXITWYVPKGKQHPRWTSFIKTFTSLVWYLVLLTFIFGSCTMWLFCKSERLRS
NPTNTNNHGVLVDAMLTHLGIGVFYRYKGFIPSAFFMLWLYYCLIINTAYQSAFFGLLINP
SHIPALQTSKQVEESALIKQTFGIMITESPFWSRFLKYKACQTRNECVDVLAKHPTRAILSD
TWHARYHISKHGENKYVPINEIVGTEFLSNQVNQLRGILLDLFNKVLGRLTDSGIIDQMTK
EINREYSYMRKNRVIGTAFSLTMDHLQVAFILLLIGYVLSLSLNLVKVFVNFVINWYYLLCI
KYQLQVIT
>BgerIr266P
MLLLKLVFLFYILGCSWALLTLYQEKSIEESLAECIINISIKYFDEDRPILVETSTNWFPRRYP
YSEYGGKLIELLHKENQFTLIIYGIEAYLARVKVHVGSYIVLMPPILRPSLDNLYIEHMLQAL
FKWAYNCRGKVIVASLEQMASPEIRDLLTLAMNLELLHTILLKPRTLSQKPKDNHLDNIDI
YNWFPNEQNNICSRTVNKINHSDTWISQTKMFSFNSNLFPLARQIDFHGCTLRTHFPTIP
PYSFVYEEVGRTAGPVVDIFLTIMKYINCTIIYNPPKREDTFRVHIFFPVPLDPTIYVQNKYR
DQLTYPHMVSEITZYVPKGKQHPRWTSLIKTFKSLVWYLVLLTFIFGSCTMWLLRKSERF
RSKLTSKNNHRVLVDAILTHLGYGIIYRFKGLIPSAFFMLWLYYCLIINTAYQSAFFGLLINP
GHSPALQTSKQVEESALIKQTFGLVTTVSQFWSRFLKYKVCPSSAECFENPTHAILCDTWQ
AMFHLSRLRENMYVPINEIVGTKFLTIQVNQIRGILLDLFNKVLGRLVDSGIIDQMIKDINR
EYSYMRKNPVIGTAFSLTMEHLQVAFFLLLIGYVLSLSLHVVEFFVNFFINWYYLLCIKYQL
KLIKLL
>BgerIr267P
MLRFKLFFLLFZTWGSSWAQLTIYQENSIEESLAECIINIGTKYFDEDKPVLVETPTNWFPL
RYPYIEYGGKLIELLHKANQFPLIIYGIEAYFAQLKVDVZSYIVLMPPVLRPLDKEYIKHMLG
ALFDCAYNCNGKVIVASLEQMASPDIRNLLTLAMNLELLHTILLKPKTLSIKTKDNHFDNI
DIYNWFPNEQSNICSRTVNKINNSDTWISQKKTFSVNSNLFPSHRKIDLNGCTLRTHFPAY
PPYTMIFEEKQITAGTLVNIFHSVKKYINCTMMFTAPMREDTYRLHIYFPVPLDPLHYVQN
EIRDQLTYPHMFQYITWYVPKGKQQPRWTSLIRTFTSLVWFLLLLTFIFGSCTVSPFYAES
XVVYKYKGLIPSVFFMLWLYYCLIINTAYQSAFYGLLINPGHFPALQTSKQVEESALIKQTT
GIMNPGSSFWSRFLKYKPCHNRKECLEENQSRALLCDTWYGKSHISKIGDNIYVPINEIVG
TKLLSIQVNQLRGILLSVLNKVLGRFADGGIIDQMTKEFNNIYMPTNPVIETAFSLTMDHL
QVAFYVLLIGYVFSLSLNVIEFCVNVIIYZFYLLCIKYQLQFSRLL
>BgerIr268
MLLLKLSFLLFYIFCSSLALLTLYRENSIEESLAKCIINISTKYFDKDKPVLVETPTNWFPHH
YPYIEYGGKLIELLHKENQFSLIIYGIEAYLARLKVHIGSYIVLMPPVLRDIDNLYIEHMLGAL
FTWTYNCKGKVIVASLEQMASPEIRNLLNRAMNLELLHTILLKPRTLSQKHKDDHLDNID
IYNWFPNEQSNICSRTINKINHSDTWISQPNKFLFNTNLFPSDRKIDFKGCTLRVHFTHFP
PHAIVNSELRESAGTIVSIFYSIMKYINCTMQYIPRPRADTSSEHIYFPIPLDPLDYDQNEIRD
QLTYPHMVRDITWYVPQGKQQPRWTSLIRTFTSLVWFLLLLTFIFGSCTMWLLGKSERFG
SNSTYKMNHGVLTDAMLTHLGVGVVYRYKGLIPSVFFMLWLYYCLIINTAYQSAFYGLLIN
PGHFPALQTSKQVEESTLHKVTSIFLNTASPFWSRFLKFRHCHDVELCFDSLADNQMLAL
LCTTWEGTSQYRILVLSTKKYVPINEIVGTIHLSIQVNQLRGILLDVFNKVLGRFADGGIIDQ

MTKEFNNIFLRKNPLIETAFSLTMDHLQVAFYLMLIGYVLSLSLNVIEFCVHFIINWFYLLCI
KYQLQFAKL
>BgerIr269
MIFKLFFLLFYTWGSSWAQLTIYQENSIEESLTECIINISTKYFDEDKPVLVETPTNWYSDQ
YPYVEYGGKFIELLHKANQFSLIIYGIEAYLARLKVHVGSYIVLMPPVLRPIDNLYIKHMLNA
LLKWAYNYRGKVIVASLEKMTSKKIRNVLTLALNHELLHTILLKPRTMSQKPKDNHLHNI
DIYNWSPNEQSNICLRTVDKINHSDTWISQTKSFLVNTNLFPSIRKIDFNGCMLRTHFNDA
PPYTFVYPKKRRTGGTVVYIFGCIIKYINSKINYIPPTPQGTSSVHIYFPILLDPIHYDQDELR
DQLTYPHLVSEFTWYVPKGKQHPRWTSLIKTFTSLVWYLVLLTFVFGSCTMWLLRKSVG
RGSNPTNQNNHWFLVDTILTHLGYGIIYRYKGFIPSTFLMFWLYYCLIINTAYQSAFFGLLI
NPGHIPALQTSKQVEESALIKLSFGIIFTDSPFWSRFLKYKVCQNDECFNAFTENTPRALLR
NIWEATHHINLFGENRYVPINEIVGTAYLSIQIIQLRGILLDVFNKVLGRLTAGGIIVQITKEI
NRNISYTRKNAVIGTAFSLTMDHLQVALFLLLIGYVLSLSLNVVEFFVYFVINWYYLLCIKY
QLQFITLL
>BgerIr270P
MQYFKLFFLLFYIWGSSWALLTFYQEHSIEESLAQCIINISTKYFDKEKPVLVETPTNWFPG
HYPYIKYGGKLIELLHKVNQFPLIIYGAEAYVASLKVDVGSYIVLIPPILRQIDNIYLQSMITA
LLNWAYNCRGKVIFASLDLMTSRDIRNVLNLALSYELLHTIILKPRTLSLKIKDNYLDNIDI
YNWFPNEQSNICSKTINKINHSDTWISQAKSFLANANLFPSDRKIDLKGCTLKSQFXLSSE
QRSTAGTLVSIFHSIMKLINCKIVYTPALPDDTNGVHLYFPLPQDPVFYDEEERREQLTYPH
MVSDITWYVPKGKQLPRWTSLIRTFNSLVWYLVLITFVFGSCTMWLLGKFEKIGSNSTNK
TNHGVLADAMLTHLGVGVVYRYKGLISSVFFMLWLYYCLIINTAYQSAFFGLLINPGHFPA
LETSRQVEESALIKQTVGIQSTYSVFWSRFLKYRSCYDHKVCFENIAKNQMRAILCNTWLA
KSYYSMFGDNMYVPIKEIVGTZLLSIHVIQLRGILLSVINKVLGRFVDGGIIDQMTQEINNEY
MRKNPVISTAFSLTMDHLQVAFYLLLIGYVLSFSLNVIEFYVNFIVNWIYLLCIKYELQFVRL
L
>BgerIr271
MQHFKLFFLLFYIWGSSWALLTLYQENSIEESLAQCIINISTKYFDKDKPVLVETPTNWFP
GHYPYIEYGGKLIELLHKVNQFPLIIYGGEAFIAQLKVHVGSYIVMMPPVLRQVDNIYIQNMI
AALLKFAYNCRGKVIFASLEQMASPDILKLLNRALTYEFLHSILLKPRTLSHKTKDNHLDNI
DIYNWFPKEQSNICSKTINKINHSDTWISQRKRFLLNTNLFPSDRKIDVKGCTLKSHVPFF
PPYTFVDSELRINSGTIVSIFHAIMKHINCIILYTPPVPDNTNSVYLYFPVMLDPADYGEYEL
RDQLTYPHIISDITWYVPTGKQQPRWTSLIRTFTSLVWYLVLSTFVFGSCTMWLLGKYER
LGSNSTNKTIHGVLADAMLTHLGVGVVYRYKGFIPSAFFILWRYYSLIINTAYQSAFFGLLI
NPGHYPTLQTSKQVEESALVKETVGIQGVDSQFWSRFLKYRSCYDTNVCFGSLAKNQTHA
ILSNAWFAKFHYSMFGENKYVPIKEIVGTRLFSIQVLQLRGILLDVFNKVIGRLADGGIIDQ
MAKEFINHFLRNNPVICTAFSLTMDHLQVAFYLLLIGYVLSFSLNVIEFCVNFIINWFYLLCI
KYQLQFVRLL
>BgerIr272
MPNIKVMFLLFYIWTLSSSKLTHFEENLIEEYLAECIINISSRYFVDDKPVLVATPINLYPGR
YPYLKYGGKLIEMLQKENKFSLIIYGAEAYLGKLRVRVGSYIVLLPPILRPLDELYVKGMFRG
INAFAYNFRGKTIVASVEQDTSSDNEKLQPIKVLKYAFEFEQPNTILLEPRSKSNKYNRKYL
DKIYIYNWSPNEQSNICSYNIDKINHSDTWISRKRRFLFNTNLFPTYGNINFKGCKLILEVG
NQPPFAFIEYSEDMIFGTVLNLFDYIKEYINCTVVYIIRQYQNTYPFIIHIDYLTPLDPINYAK
DVVPDKLIYPYMRHEITWHVPIGAKKSRWMSLVKTFSAFVWSLVLITFVFGSFTMWLLGR
SARLDAGVKDEINYGILADAMLTHLGVGVTYRHKGFIAGTFFMLWLYYCLVINTAYQSAFY

GLLINPGSIPPLETIKEVEKSDLVKVITSVYPASLFWSRFMKYKFCSDAELCINQIAVKQTKA
ILINKWMGKFYHRSYNDLKYVPIKEIVGTQLLSIQVFKLHCILFSVLNKVLGRFTNAGILDH
MTEKYTRGYLHNTPLTKTEFSLSMDHLQVAFYLLLIGCMLSLSLNVIEFCVHFIINWFYLL
RIRYYLSIIRLI
>BgerIr273
MKYPKVIFVLLYICAVSSPKLTHLEEHSIEESLAECIINISSRYFDEDKPVLVETPVNWFPGR
YPYLEYGEKLIHMLQKENKFSLIIYGINGFLVETKVHVGAYIVLLPPILRSLDELFVERMLAA
IHLMAYNFRGKTVVATVEDMTSSDKKKLQPIHVLKYAFKSELLNAILLVPRFLSKGKKLNN
IYIYNWSPNEQSNICSYNIDKINHSDTWISQKKSFIFNTNIFPPHSKIDLKRCKLNLLLHNEP
PFVIFSYKTRTVSGIITKLIDTILDYINGTLTYILPEHHQLPIVHFYFPLPLDPIYYEPDAIRDR
VTYPYMRQEVTWFVPEGVQKDRWTSLVKTFSALVWSLVLLTFIFGSFTMWLINRSARISS
NVTEEINYGVLGDAMLTHLGGGVNYRYKGFIASSFFMLWLYYCLVINTAYQSVFYGLLINP
GYLPALETSKEVEESHLIKQITFAYPASAYWSKYLKYRYSSNAATGLKSVAVSRTHALLTTV
WKAKFHYKSFSNNRYVPIKELVATHLYSIQVFKLNSIFFNLFNKVLGKFADGGIIDQMTNQ
YNKHSFRQNSPTTALSLSMDHLQVACYVLLIGCTSSLSVNIIEICVHFVISWFYLLSIKYNLK
FMSLL
>BgerIr274
MKYPKVIFVLLYICAVSSPKLTHLEEHSIEESLAECIINISSRYFDEDKPVLVETPVNWFPGR
YPYLEYGEKLIHMLQKENKFSLIIYGINGFLVETKVHVGAYIVLLPPILRSLDELFVERMLAA
IHLMAYNFRGKTVVATVEDMTSSDKKKLQPIHVLKYAFKSELLNAILLVPRFLSKGKKLNN
IYIYNWSPNEQSNICSYNIDKINHSDTWISQKKSFIFNTNIFPPHSKIDLKRCKLNLLLHNEP
PFVIFSYKTRTVSGIITKLIDTILDYINGTLTYILPEHHQLPIVHFYFPLPLDPIYYEPDAIRDR
VTYPYMRQEVTWFVPEGVQKDRWTSLVKTFSALVWSLVLLTFIFGSFTMWLINRSARISS
NVTEEINYGVLGDAMLTHLGGGVNYRYKGFIASSFFMLWLYYCLVINTAYQSVFYGLLINP
GYLPALETSKEVEESHLIKQITFAYPASAYWSKYLKYRYSSNAATGLKSVAVSRTHALLTTV
WKAKFHYKSFSNNRYVPIKELVATHLYSIQVFKLNSIFFNLFNKVLGKFADGGIIDQMTNQ
YNKHSFRQNSPTTALSLSMDHLQVACYVLLIGCTSSLSVNIIEICVHFVISWFYLLSIKYNLK
FMSLL
>BgerIr275
MYLSHLFLLIHTLASGHSILSSFEDSSLEQNFAECVLNISNTYFDKRFSVLIQTPSTWYPEDH
PNHKYGDKLIQMLHINNSFPHIVFGSTTFRQIRELYSRDIASYIVLFPTTAKDEDIEYIMSVF
EPIFYQGPQFKAKLIFVSLTKQQTIEGTTLSTKAFLLKIALKAGIERAVYLTPENERNKSRSK
SYNSIDVFSWSVNEQSDICSGHLDNIKYIDSWNTEEKKFLLNADLFPLRSEINLRGCEIKVIL
GHMPPYSYSIDDKKFGEIVILINDTVHQMNGKIRFVDFEDDNHISFPLVYSTERQRDAIEST
YPHIAEEIKWFVPSGLEVPRWQSLIRTFSSGTWCLVLLTFAFGICTMWLLGKSPGHLNNTR
VNSNSMLVISAMLSHLGVGVVERYKGFVATLFFTLWLYYCLIINTAYQSAFFGFLVNPGHF
PTIKTFNELEISGLNMYRLFNLYLENESFWSSFNKYTVCPTFTECYVKVSNDRTHAVLDDT
YSAKLLLRRFRDSNGHPKFVPIDETVGTMLTAIGINELQSVLSTAFDKNLNRFVVAGFMEK
SRNDGVFSYNLEYKTANYHPIFALAVRHIRGSFYLLLIGLVLSFLVFLFENFNHFIHNGLFLY
YIKHYLYIQKLV
>BgerIr276P
MYLSYILLLIYTLGSCNSTWTLFEDSSLEKNISECVLDISKTYFDKHFPVLIQTPSTWYPENH
PNHKYGDKLIQMLHIENHFXGDTTFRZIRETLVHDIRSYIVLVPPTVSEEDLDYIISPFQLID
ENSPHIKAKLIIASLIIRRTFRSTTLSIKASLLNLGLNSGLDRAVYLTQVANSIKVFSWSVND
QTYICSGNLDIIKYFDTWITKEKXFLLKTDLFPPRQEINLRGCEVRVVLGHIPPYSYYNRNK
LIGEVSVLINDTIHQMNGNIRLVNFEERHHIAFPAIYSEERNRDANRATYPHIPLEVTWFV

QSGLEVPRGQSLVRTFSSITWCLVLLIFTFGIVTMWLLAKSPGHLNNTSANTNSMIIISAML
SHLGIGVPERYKGFVATLFFTLWLYYCLIINTAYESAFFFGFLINPGYFPTIKTFHELETSGL
NMYRQFNLNLEEENFWSRFXRYTVCRTVTECYSKVSIYRTHAVLDDTYSAKLVSLLTRFX
SNGYPKFVPIDETVGTMLISIGINDLQSVFSTAFNKXNRLVEAGIVEKSKHDGVFSNNLGHK
TKHYGTIFALALRHVQGTLFLLLIGFLLSFFVFIFENFNNFIRNLIYLYYIIYYLKLM
>BgerIr277
MKKRKMFFSHLLLFMYTFGSSHSILTSFEDNSKEQHFAECVLDISNTYFDKHLPVMIQTPS
TWYPENHTNHKYGDKLIQMLYIQNHFSVVVFGDTTHRQIRKTYIRDIASYVVLVPPIVSEE
DMDCIISLFQLIYDHAPQFRAKLIIASLMKQQSFEGIPIKKFLLNIALTAGLERAIYLTQEISD
SINVYSWSINDQKNICSGFLDNVNYLDTWITEKKMFLFNTDFFPLRPEINLRGCEVKVLLG
HIPPYSYYYDNKRIGEIGILINDTVRQMNGKVRLVNFEDENHISFPVVYTMNRQRDTIESTY
PHIAQEISWFVPSGLEVPRWQSLIRTFSSVTWYLVMLTFAFGVVTMWLLAKSPGHINNTS
ANTNSMLLISAMLNHLGVGVAENYKGFVATLFFTLWLYYCLIINTAYQSAFFGFLVNPGNI
PTIKTFHELETSGLKLYRLFTLNFEAEIFWSSFIKYTACPTVAECYSKVSNDRTYAVLDDTY
SAKLMLRGFRDSNGHPKFVPLDETIGTMLIVIGINDLQSILSTAFNNNMNRLVEAGIIEKSK
KDVLFSYNLGLKTANYHPIFALTLRHVQGTFYLLLIGLLLSFSVFLLF
>BgerIr278P
MNLMFILMFZIWCSCSSLLTAPKENILEQHLAECALKISSTYFDSNFPVVVHTPSTFYPPGH
RNYKYGDKLIQALEAQFYSPILVVGNRNNRNCTWQIEVQKPGSIIIVVPPTYTSKDFDYIFH
TYRTIRHCAFNPSAKIIIVISQDNGVIVYDKPYPLFLLSHSFKFGYLNIIVLEPKSIRTGNGTT
RIIRIFGFRINDQRNICSRELDKIRHVETWATEEKKFLFNSSVFPLQGKLNLKKCYLTVAVL
NRFPYSIRDTDSTLGFFGPFAWFLFCITKLLNVKLSIVKDYKVADIVFPSLYDPLNIKVWHK
LTYPYFREDITWYVPLGHEVPRWQGLVRAFNPLLWCLILLTSALGTFTMWLLQKSERHS
TAPSNRGFLETLSSALLTHLGAAVAERYKGFVAVLFFMLWLYYCLVISTAYQSSLFGLLVYP
GHSPAIQTLKELEESGLIMERTYSVTNAPAGIWGEQMKYKYCNDATIKCLNKVWVDRTH
ALSDSAWLAKIFAGRCRDKRGNLQLEYLKEFIGTSYYCVLVDRFFSFLVPVFNNVIGTFVAS
GIFLF
>BgerIr279P
MARTFYSTYIELVRYRIIIIIRENMNLMFMLMFYIWCSCSSLLTAPKENILEQHLAECALKIS
STYFDLNLPVVVHTPSTFYPPGHRNYKYGDKLILALQDQFYSPIIVVGNRNNRNCAWQIEV
QKPGSIIIVIPPTYTSQDVDYIFHTHETIRHCALNPSAKIIIVISQDNGAIVYDKPYPHLLLSIS
FKFGYLNIIALEPKIIPTGNGTTRIIRIFGFRINDQRNICSRELDKIRHVETWATEEKKFLLNS
SVFPLQGKLNLKKCYLKMAVLGTFPYSIPFTNSTLGFYGPFAWFLFCITKLLNVKISIVKDY
KVADIVFPSLYDPLNIKVWHKLTYPYFREDITWYVPLGHEVPRWQGLVRAFNPLLWCLIL
LTSALGTFTMWLLQKSERHSTAPSNRGFLEALYSALLTHLGAAVTERYKGXAVLFFMLWL
YYCLVISTAYQSALFGLLVHPGHSPAIQTLKELEESGLIMERTYSVTNAPAGFWWKQMQY
KMCNRSAIECLKKVSADRTHAFSDSTWIGKLFAGQCRDKRGHLQLEYLKEFTITVLYCVLL
KRFFSFLVPVFNNVIHTFVASGIFVFKIDLVVTEWLLWNRNLDDQIESVMTFSLHELQGEF
YLLLIGFLLASLVLAMEFLVHFIGVRFSITSISSLLVRLRLR
>BgerIr280P
MHFQLIVIFCILSRCKTLLVAPKANILEQNLAESVVTIRLTNFDTNFPVVVNTPSTFYPPGH
RNYKIGDKLIQTLQNQAHLALIVVGKSKNRNSVWQAGILKPGSIVIMIPPIYTFQDVQYILD
TYAMIRNSAHNPGAKVAIFLCEESRAIVGDQALPLYLLNVSFRFGYLDIIVLEPKSVSTGNG
TRRDIRILNFAINEQRNICSRKLDKINHVETWVTEEKKFFVNSSLYPIRGKLNLRRCVLKVL
IRNSFPYSIVSKTARLGFMGPIENVLXVAKSINVAFEVTDEYELSDLHFPSMYDQRNRNFW
HKLTYPYFGQDIVWYVPPGREIPRWQGLVRAFSPLLWSLILLTSAFGTLTMWLLQNSERH

STAPSHRGILAALSSAFITHLGAAVTERYKGFVTVLFFMLWLYYCLIINTAYQSALFGLLVY
PGHLPPVQTLKELEESGLIMERLYSVDTGSEGFWMGEMKYKLCNRPGYHCLRQVAFDQT
HAISGTIWISKLHGEPYRDTRENLKLVTLKELVGRMLYCLHITRCFVLLGPEFNTIIRRLVN
SCLLSYWLVDYMCQWKLLHSDEIVEIESVMTFALYQLGGEFYLLLIGFVLASLAFAIELFFH
LILLWFYVSTITVSLDRVRLL
>BgerIr281
MFLKLFLTFFMWYTCKSLLTVRQNNILEHKLAECVVKISLTYFDASLPLAVHTPSTYYPPL
HRNYKYGDTLIQTLQNQGHLPIIVVGNRNNRNSIWKKDFFKPGSVVIMIPPMYTSEDVKY
VLDTHVMIRDYAYNPSAKVTIVMCEESEAIVFGKELPHYLIYVSFRFRYLDILVLEPRSISRF
GVTISIIRILGWTISEQRNICSRDLDKVKYLETWVTADKSFLLNSSLYPVFGKSNLRKCVFTV
AILPSFPYAVPLKSANLGVTGPLEVILYLVGKSVNVAFKAATETKTADILFPSMYDPTSRKF
WYTLTYPYFGQDIVWYVPPGREIPRWESLVRAFSPLLWSLILVTCALGSLTMWLLQKSER
HSTANSNGGILMALSSAFCTHLGTGITERNKGIVAVLFFMLWLYYCLIINTAYQSALFGFLV
YPGHLPPIQTFKELEESGLIMERSYASYGTNGTFWEGQDKYKLCEAGKIVCTRKVAIDRTH
ALSGISFIGKLYGDRYRDVRGNSKLVPLKEYVGNVLYCLRIHWYPMFFVPVFNTIIRRVVTS
GLYFKWIDDAVFKWNLVYIDEVDQMEPVTSFSLHQLQGEFYLFFIGFLLASIVFVFEFLYH
FIVLWFYVSRITFFLDRVRLA
>BgerIr282
MHFMLIVVFCILNSCRSLLIAQKQDILEHSLAECVVTISLKYFDTNLPVVVHTPSTFYAPRH
KNYKYGDKLIQTLQSQGHLSIIVVGNRSNRNSIWQTKVLKPGSVIIMIPPIYKTKDLEYVFYT
YVMIQDYAYNPSAKVLIVISDENKIIVDDLPFPQCLLNLSFKFGYLDVIVLEPNSVVSGNINF
SRVRILGWTINEQSNICSGKLDKIKHIETWVTQERKFLFNSKLYPEQGKLNLRRCILKIMIN
PTFPYSNPWMNASLGIVGPIALIHYLVAKSINVRYQSDYEKYDIEFPSIYDPVIRKHWYRFT
YPYFGHDIVWYVPPGREIPRWQGLVRAFSPLLWSFILLTSAFGTLTMWLLQKSERHSTAP
SHRGILAALSSALLTLLGVAVTERYKGFVAVLFFMLWLYYCLIINTVYQSELFGRLVYPGHF
PPIQTLKEIEESGLIMERTYFYNLGPGSFWAEQMKYKYCEGKINECVKKVAGNHTHALTG
NVWLGKMHSVQFRDTKGNSKLVPLKELVGTLMICLSISMDFVFLVPVFNKIISRIINFGLLN
HCTNLVIRQWNLAFRDKVVEIKSVMTFSVYQLQGQFYLLMIGLVLASFVFAFEFFVYFIRL
RFYVSRIRFSLDRMYLG
>BgerIr283P
MFLKLFLTFFMWYTCKSLLTLPQNNILEHNLAECVVKXSLTYFDAKLPLVVHTPSTYYPPL
HRNYKYGDTLIQMLQNQGNLPIIVVGNGGNRNSKWQKEFFKPGSVVIMIPPIYTSKDVKYV
LDTHVIIRDYAYYPSAKVTIVMCEEREAIVFGKELPHYLIYVSFRFRYLDILVLEPRSISRFGV
TISIIRILGWTISEQRNICSRDLDKVKHLETWVTADKSFLLNSSLYPIRGKTNLRRCVFKVGI
VRSFPCAVPFEPAPLGFIGTIEDILYFVGKSVNVAFQAFEDLLEADIVFPSIYDPMFRKSWY
MFTYPYFGQDIVWYLPPGREIPRWESLVRAFSPLLWSLILVTSAFGTLTMWLLQKSERHS
SAPSHRGILVVLSSALLTHLGASVAERNKGNVAVLFFMLCLYYCLIINTAYQSALFGLLVYP
GHLPPIQTFKELEESGLIMERSYMAHGPPGTFWNEQMKCKGCEGGSIVCTRKVAVDRTHA
LSGISLLGKIHRGRYRDVRGNSKLVHLKEYVGNILYCLQINWYPAFLVPLFNTIIRRVLNSGL
YSKWIDEIISRCIQVYREEYVQIEPATSFSLHQLQGEFYLFLIGFLLASIVFVFEFLFHFILLWF
NVLRITFFLDRVHLA
>BgerIr284P
MHLQYILMFCMWCSCRSHLTAPNVNVLEENLAECVIKISLTYFDANLPVVVHTPSTFYPP
GHKNYKFGDKIIEALQSHSHSPIIVVGNRNNRNCTRKTEPQKPGSVVIVIPPTYTAQDVKYI
FETYPMIKNCAHNTSARVTIVLSVETEIIVRGKTFSHYLLNVSFKYGYLNIIVLQPQLVSTRN
GRKRIIRIFGFRINDQRDICSAELDKMKHFETWVTEEKKFLLNSSLYPERGKFNLRRCVLTI

AITPIFPYANVFKSAPLGIIGTLEDILYLAGKKINLAFRLTKDFDAAEIAFPTLYDSKNRGLW
HKLTYPYFRQDIVWYVPAGREIPRWLGLFRAFSPLLWSLILLTFALGTFMMWLLHMSER
HSTAPSNRRFLASLSSAFLTHLGISVTERYKGFIAVLFFMLWLYYCLIINSVYQSALFGRLVY
PGHFPPIQTLKEIEESGLIMERAYTVATEPGGFWZEQMKYKRCEGHHVSCIKKVTVDHTH
AIAGSTWLGKLYGGIFRDTKGHIKLVPLKELVANMLCCIHFNWLSVILVDVFNTVIRRIINS
GLYFKWADHAVSSWNMGMRHKLVQIEPVTSFSLHQLQGEFYLIMIGLLLASIVFVFEFFIH
FILYWFYISRITFFLDRVRLA
>BgerIr285
MFLKIFLTFFMWYTCKSLLMVPQNNILEHNLAECVVKISLTYFDTNLPLVVHTPSTYFPPL
HRNYKYGDTLIQILQSQGHLSIIVVGNRITCQTNVLKPGSVVIMIPPIYTAKDLKYISATYVM
IRKCAENHSAKVAIVLSDENEVIVGDKAFYHFLLNESFKFGYLDIIVLEPKFVSTGNGATRII
RVFGFTINEQRNICSQELDKINHAETWATEQKKFLLNSSLYSVRDKLNLRRCALKIFIISSFP
YAQATKENGFGFIGPLYDFLYFVAKSINVKFLSINDKRVGDLYFPSVYDPGNRNIWYKFTY
PYFGHDIAWYVPPGREIPRWQGLVRAFSPLLWSLILLTSSFGTLTMWLLQKSERHSTAPS
DDGILAALSSALLTHLGASVAERNKGIVAVLFFMLWLYYCQIINTAYRSALFGLLVYPGHLP
PILTIKELVESGLIMERQYTVTYARTGFWWEQMQYKMCKGSKVECMRKVAIDHTHALTG
TTWIHKLYGDQYRDTRGNLKLVPLKELVGRILVCLDINRFFVFLVPVFNTIISRIVSSGLFFK
WMGEDILRHKLLHKAEIVDIESVMVFSLYQLEGEFYLLLIGFVLASLAFIVECFFHFLVLWV
YVITITAFLDSVRLM
>BgerIr286P
MHIKLIVVFSILNSCRSLLIAPKENILEHNLADCVGTISLTYFDTYLPVVVHTPSTFYAPRHK
NYKYGDKLIQTLQSQGHLSIIVVGNRSNZNSIWQTZFLKPGSVVIMIPPIYTAKDLKYISATY
VMIRDCAENHSAKVAIVLSDENEVIVGDKAFYHFLLNESFKFGYLXILVLEPKFVSTGNGTT
RIIRIFGFTINEQRNICSRELDKINHVETWLTXEKEFLLNSSLYPVRGKLNLRRYVLKAFIGL
STPFAFVSKTPLGFSGTVVNIIYFVAKSINVVFQITKDDELSDVYSPSMYEPMNRKLWYRFT
YPYFGQDIAWYVPPGREIPRWQSLVRTFNPLLWCLILLTSALGTFTMWLLQKSERHSSAP
SNRGFLETLSSALLTLFZZSIRPISQHYFDFWCILDICPNSDVERTYKFGSNYTFFSAFESFW
MGEMKCEVCKTPVIGLCLRKVAIDKTHALTGTTWISKLYGDQYRDTRGNFKLVPLKELVG
SILYCLRVNNFFVLLVPVFNTIISRIVNIGLFFKWMEELIWQHKLLHNAEVVDIESVMTFSL
YHLEGEFYLLLIGFVLASMAFVIECFFHIIVLWFYVPAITAFLYRVTLM
>BgerIr287P
MNLIYILMFYIWCSCNSLLTDPKENILEQQLAECALKIRLTYFDPNIPVVVHTPTTFYPPGH
RKYKYGDKLIQALHSQFYSPIIVLGNKNNKNCTWQIEVQKPGSIIIVIPPTYRGEDXAIVYNK
PYPHFLLSHSFKFGYLNIIVLEPKSIRTGNGTTRIIRIFGFRINVQRNICSQELNKIKHVETWA
TEEKKFLLKSXGNLNLKKCYLTVAVLNRYPYZIPFKNSTLGFLGPFEWFLYYITKSLNVKLS
IIEDYNVADLAFPSLYDTLSIKIWHKLTYPYFREDITWXVPLGQEIPRWQSLVRAFNPLLW
CLILHTSALGTFTMWLLKKSERHSTAPSNRGFLTALSSAFLTHLEAAVTERYKGFVAVLFF
MLWLYYCLIISTAYQSALFGLLVYPGHSPPIQTVKELEESVLIREKTYSVTNARTGFWWKQ
MKYKMCKDITIKCLNKVWIDRTRALSDSDWIGKLFAGQCRDKRGNVQLVYLNEVIGTGH
YCVLVDRFFSFLVPVFNNIIRTFVASGIFLFEIDLVVTEWLLADRNFDVQIESVMKFSLHEL
QSEFYFLLIGLVLASFVLAIEFLVHFLHLRFSVIAIASLLDHVRLR
>BgerIr288P
MNLIFIVMFYIWCSCSSLLKAPNGNILEQHLAECALTIRLTYLDPNFPVVVHTLSTYYPPGH
MNYKSGDKLIQALQSQFYSPTIVVGNRNNRNSTWQIEVQKPGWIIIVIPLPYTSQXSDYDYI
FHTHETIRHCALNPSAKIMIVISQDNGAIVYDKPYSHFLPSLSFXFGYLDIIVLEPKLIRTGNG
TTRNIRIFGFRINDQRNICSRELNKIKHVETWATKEKKILLNSSVFPLQGKLNLKKCYVKVA

VLGTFPYAIPFTNSTLGFFLGPLNGFFIVFPNYZMSNFQLLZTIMZRILLSQVFMIHZTYKYD
TCZAIRTSERILLGTFPLDKKCPDGKEPLQCGCCKSQRDTALLRPTEGSWRLCLPSVLTFLG
VAVTERCKGFVAVLFFMLWLYYCLIISTAYQFALFGLLVYPGHLPPIQTVKELEETGLTME
RTZALSDSTWIGKLFASQCPDKRGNSQLEYLKEXLLYCVLVKRFFSFLVPVLNNVIRTFVSS
GIFIFKIDLVVTEWLLADRNFDVQIESVMTFILHELQGEFYLLLIGFLLASLVLAIEFLVHLIG
VRFYITAILSLLDRLRLR
>BgerIr289P
MHFQLIVIFCILSRCKTLLIAPKENILEQNLEECVVTISLTNFDTNFPVVVHTPSTFYPPGHR
NYKIGDTLIQTLQNQAHIALIEVGKSKNRNSVWQTGILKRGSIVIMIPPKYTPQDVEYILDT
YVMIRNNAHNPGAKVAIVLCEELGALWEIKHFLIIYZSFFFRFGYWDIIVLEPKSVSTGNDT
RRDIRILNFAINKQRNICSRKLDKINHVETWVTEEKKFFVNSSLYPTRGKLNLGRCVMRVL
ITNSFPZSIISKTARLGFMGPLEIVLLFVAKSINVAFEVTNEYKLSDLHFPSMYDPWNRISW
HKLTYPYFGQDIVWYVPPGREIPRWQGLVRAFSPLLWSLILLTSAFGTLTMWLLQKSERH
STAPSDGIVFCITHTFRCFCRZEEQRDCRCSVFYIMVVYVCLINNTAYQSAIFGLLVYPGHLP
PVQTLKELEESGLIMERVYSVSTKSESFRMGEMKYELCNRTGLCLHQVAVDQTHATSGTI
WINKLHLEKYRDTRGNLKLVTLIELVGSMLYCLHITRYFVSLVPEFNTIIRRLVNSGXVLHS
DEFVEIESDMTFSLYLFGGEFYLLLIGFVLASLAFAIELYFHLLLLWFYVSTITVSLHRVRLR
>BgerIr290P
MFLKPFLTFFMWYTCKSLLTVPENNILEHNLAECVVKINLTYFDANFPLVVHTGSTYYPPL
HRNYKYGDTLIQTLQNQGHLPIIVVGNSTNRNSTWQKDFFKPGSVVIMIPPMYTSKDVKY
VLDAHVMIRDYAYNPSSEVTIVICDESEAIVLEKELPHYLIYVSFRFRYMDIIVLEPRSISRFG
VTITIIRILGZTISEQRNICYRDLDKVKLFETWVTADKSFLLNSSHYPVRGKSNLRKCVFKVA
ILPSFPYAVPVKSANLGVTGPLEDILYLVGKSVNVAFKAAKVPKKR-
IFFPSMYDPMSRKSZFTFTYPYFGQDIVWYIPPGREIPRWESLVRAFSPLIWSLMLFTCALG
SLTMWLLQKSERHSTARSDGRILMALSSAFRTHLGASVTERNKGIVAVLFFKVKVVLLSN
NQYXSESSWMGEMKYEICNRTGLFQPQVSVDQTHAISATIWISQLHGKPYZDTRGNLKLV
TLIELVGSKLYCLHITGYFVLLVPELNTIIRRLVNSGLLSFWLVDYMCQWKLLHSDEIVEIES
VMTFSMYERGGEFSLLLIGFVLTSLAFAIELFFHLLLLWFYVSTISLHRVRLL
>BgerIr291P
MPFKLIVLFCILNSCRSLLIAPKDNILEHSLAECALKISPTYFDTDMLVVVHTPSTFYPPGHK
NFMYGDKLIQTLQCQGHLSIIVVGNRSNRNSIZQTKVLKLGSVIIMIPPIYKTKDLEYVLDTY
VMIQDYACNPNAKVLIVISGENKIIVVDLPFPQCLLNLSFKFGYLNVIVLEPNSVVSGNISFS
TVRILDWTINEQTNICSRKLDKIKHIETWVRZPKRENSFSILVSSQYRGSZIZGDVFZKLCZS
FPYSIPLKNASLGIIGPIAFIHYLVAKSINVZFQSTNSILHDIDFPSIYDPVIRKHWYRFTYTYF
GQDIALYVPSGCEIPQWQGLVRAFRPLLWSXIHLTSAFGTLTMWLLQKSERYSTAPSDSGF
LVALSSALLTFLGVAVTERYKGFVAVLFFMLWLYYCLINNTVYQZEHFGRLVYPGHFPPIQ
TLKEIEESGLXMERTYFYNPGPGSFWAEQMKYKHSEAXCVKKVAVDHTQALTGNVWLGK
MHSVQFRDTKGNSKLVPLKEFVGNIRICISISTVFVFFXPVFNKIISRIINFGLLNHCTNLVIG
QWNLVYRDKVVEIKSVMTFSVYQIQGKFYFLMIGLMLASLVLVIEFLVY
>BgerIr292P
MHLQYILMFYMCCSFRSHLTFQNEDDLEENLAECVISISFTYFDANLPVVHTPTTFYPPGH
RNYKFGDKLIQALQSHSHSPIIVVRNKNNRNCTRKTEPQKPGSIVIVIPPKYTAEDVDYIFD
AYPXLRICAHNASAKVTIVLSDETEIIVGSTAYPNYLLNVSFKFGYLNIIVLMPQLFLTGNGT
KRIIHIFGFRINDQRDICSAELDKMKHVETWVTEEKKFLLNSSLYLERGKINLRRCVLTVAI
KPIFPYAYNFKSAPLGIIGSLEYILYFVGKTINVAFRVTKDNYRTKISFPTMYDPKNRDIWH
KMTYPYFGQDITWYVPAGQEISRWQGLLRAFSPLLWSLILLTSALGTLTMWLLHKSERHS

TAPSNKGFMVALSSAFLTHLGASVTESNKGFFAVLFFMLWLYYCLIXNTAYQSTLFGLLVY
PGHFPPIQTLKEIEESGLIMERAYTKATDPGGFWWEQMKYKZCDGDDEFCLKKGVYZSPH
AIVGSTWLGKLYEGVYRDSKGNMKLLPLKKLVANMMFCIHFNWFSVVLIDVFNTIISRIIN
SGFFFKWTNSALSHYKMGLFQQVVQIESVRTFSLHHLLGTFYLFFIGFLLASIVFVLEFFIHF
ILLWFFVSRIKFFFGRVRSS
>BgerIr293P
MHLKLFLTFFMWCTTSXLRTVTQNSFLERNLAECVVTISLTYFNTNFLLIVHTFSTFYPPG
HNILRVWGZSNPDFAKPRZFAYNIVIENGINRNSILQNEVLKPGSLVIMIPPTYSXKELDYIF
ATYVMIMDYAHNSNVKVTIMFSYENEIDIDDIAHYLVNVSFKFGYLDIIILDPKLVSIGDGST
KIIRMLGXTINEQRNICSRELDKINHVETWVTEEKKIILKZYLTPVRGKLNFRRCVXKVFILP
SLPFAMVCRTALMGFMGPIQEILYFVAKSINITFQLTKDPNVADHNFSSLYDPLNRIFWFR
FTYPYFGQDFFVVRTTWIPRWQGLFRAFSPLLWFLILLTFALDTFTMZFLQKSERQTTAPS
DGGXMAALSSELKTLLGVAVSERYKGFVAVLFLLWLYYCLIIINTIYQSAFLDFWCXNLPSI
QTLKELSESXLIMEITYLVFGEGRYWEGQMKYKPCEETNSVCMRKVAVDQTHALSGTIWI
RKLYGDQYRDRR
>BgerIr294P
ALAHIGLLSYRQDKTTESPIIVVGNRNNRNCTRKTEPQIPGSIVIVILPTYTVHDVNYIFDTZ
PMIKNCAHNATAKITIVLSDEIEVTVGVKAXAHYLLHVYFKFGYLNIIVLLPQLVSTNNGSK
RIIRIFGFRINDQHDICSGELVKMKHVEIWITEEKKFLFNSSIYSEQEKINLRRCVLKIAIAHI
FPYANAIKSAPLGIVGVLEYILLFVGRTINVAIRATKDSRXAQLSFPCAYDTTNRHVWHRLT
YLYFRKDITWYVPSGREIPRWQGLVRAFRLLLWSLVFITSALGTFTMWLLQKSERDSTAS
SNRWFLVAFSSAFLTHLGVSVSERYKGFVTVLFFMLWLYYCLLINTAYQSSLFAHLVYPGY
FPXQTFSELEESGLIMERAYTVTTVNKGFWREQMKYKLCEGDEVSCIEKVTIDRTDAIAGS
TWLGKQYGGVYRDSKGVQYMKLVSLKKQVANMLYGIHFNWLGVVLVDAFNTILRRLINS
GIZFNRTDHTVDVWHQAISDQVVQIEPVTTFSLHQLQGEFYLFLVGFLMASFVFVFEFFIH
FIVLWFYDSRITFFLDRVRIA
>BgerIr295
MNLIFIVMFYIWCSCSSLLTAPKENILEQHLAECALKISSTYFDPNFPVVVHTPSTFYPPGH
RNYKYGDKLIQELQAQFYSPIIVLGNRNNRNCTWQIEVQKPGSIIIVVPPTYTAQDFDYIFH
TYRTIGHCAFNPTANIIIVTSQAIGAIIYDKPYPHFLLSISFKFGYLDIIVLEPKSMRTGNGTKI
IRIFGFRINDQRKICSQELNKIKHVETWATEEKKFFLNSSVFPLQGKLNLKKCYLKVAVLGT
FPYSIPFTNSTLGFFGPFEWFLYCITKLLNVKISIVKDYNVADIVFPSLYDPLNIKIWHKLTY
PYFRQDITWYVPLGREIPRWQSLVRAFNPLLWCLILLTTALGTLTMWLLQKSEGHSTAPS
VGGILAALSSAFLTFLGVAVTERYKGFVAVLFFMLWLYYCLIISTAYQSALFGLLVHPGHSP
PIQTFKELEESGLIMERTYSVTNATAGIWWKQMQYKMCNGSTIECLKKVSADRTHALSDS
TWIGKLFAGQCRDKRGHSQLEYLKEFTITVLYCVLVKRFFSFLVPVFNNVIHTFVASGIFVF
KIDLVVTEWLLWNRNLDDQIESVMTVSLHELQGEFYLLLIGFLLASLVLAMEFLVHFIGVR
FSITSISSLLDRLRLRQRHDKQNSKV
>BgerIr296P
MHFQLIVIFCILSRCKTLLVAPKENILEQNLAECVVTISLTNFDTNFPVVVHTPSTFYPPEH
RNYYKSGDKLIRTLQNQAHLALIVVGKSKNRNSVWQAGIQKPGSIVIMIPPTYYTPQDVEYI
LDTYVMIRNSTHNPGAKVAIVLCEESEAIVGDQALPHYLLNVSFRFGYLDIIVLEPKSVSTG
NGTRRDIRILNFAINEQRNICSRKLDKINHVETRVTQEKKFFVNSSLYPIRGKLNLRRCVLK
VLITNSFPYSIVSKTARLGFMGPIENVLFFVAKSINVAFEVTNEYKLSDLHFPSMYDQRNRN
FWHKLTYPYFGQDIVWYVPPGREIPRWQGLVRAFSPLLWSLILLTSAFGTLTMWLLENSE
RHSTAPSHRGILAAFSSAFITHLGAAVTERYKGFVAVLFFILWLYYCLIINTAYQSALFGLLV

YPGHLPPVQTLKELEESGLIMEMLYSVDTGSEGFWMGEMKYKLCNRPGYHCLRQVAFDQ
THAISGTIWISKLHGEPYRDTRGKLKLVTLKELVGRMLYCLHFTRYFGILVPEFNTIIRRLV
NSGLLSYWLVDYMCQWKLLHSDEIVEIESVMTFSLYQLGGEFYLLLIGFMLASLAFAIZLFF
HLILLWFYVSIITVSLDRVRL
>BgerIr297P
MFLKLFLTFLMWYTCKSLLTVPQNNILEHNLAECVVKLSLTYFDTNLPLVVHTPSTFYPK
GHRNYKYGDTLIQTLQNQANLPVIVVGNTNHRKSIWKTKLLKPGSVVIMIPPTYTAQDVR
YIRDTYVMIRNYTHNPSAKVAIVLSDENEVIVGDQAIYHFLLDESFNFGYLDVVILEPKLAS
TSNGTTRIIRIFGFTINEQQNICSRELDKINHVETWLTEEKEFLLNSSIYPVRGKLNLRRCVL
KVFVIQSFPYAVVDEDRPLGFIGALEDFLYFVGKZINVAFRVTKDAKVADIRFPNMYDPMN
RKFWYAFTYPYFGQDFVWYVPPGREIPRWQGLVRAFSPLLWSLILLTSSFGTLTMWLLQ
KSERHSTAPSHGGILAALSSALLTHLGASVAERNKGFVAVLFFMLWLYYCLIINTAYQSALF
GFLVYPGHLPPIQTIKKLEESGLIMERFYTALVPNGTFWSEQMKYKLCEEGKIVCTRKVAV
DQTHALSGISWFGKLYGNRYRDIRGNSRLLTLKESVGTMLYTLNIRRFFVLLVPVFNTIISR
IVNSGLFFYWIDEYIWSFYNMYSDKIVDIESVMVFSLYQLEGEFYLLLIGFVLASLAFEIELFF
HLILLWFYVSTITVFLDRVCLM
>BgerIr298
MGVKFVFVITVLLVCDSFPKLTSNSEPTVEKQIAKFVADVIRVTYSNTHLPIFMITPGTFYP
PSHRNSKYGDELIQMAQDNGHILIGNEKYRPMKLSNNLVAESYVILIPPIKSQSDFQYIVQM
FHIILDFGYNPLGKMIFVCSVESEIRLENLPLPQFLLNFALKFDFVNSIVIEPKFKANSTFSEY
SKNFTIYNWKINEQNNICTGKLDNIRYFHSDLSKVDMLADVFLQEKLNFKNCALKLSISKS
FPFDYIINDILYIVSQQINVKFLVSDDIWNAHVSFPGYYGTNDRGMVHSYTYPYFRHDLTW
WVPSGQGIPRWLSFIRAFSPKLWFLILLTFAFGTCTIWLLQKSPMTDNIPKNGHIMVLLST
LFTHLGIGFADRYKGPAATLFFTLWLYYCLIINTVYQSQFFGFLVYPGNFPPITTLQELEESG
LVMERGIKIIGDEISQWYFRKYNICKHSLYSCLQKVAYDRTHAVLTDIESIMYQIASFQDIFS
NPLIVPLHEVVGSLLFSIQIHKLQGVLKNVFDSLLHNLVSTGLVGSEVMWLRGRFQANDDS
PHVFSLVHLQSSFYLLLIGLSLAFFLYITEILVHFIGNAFFLYLAKNYI
>BgerIr299
MLLISGLCCSSVLRQDRNYRRITIVWCIFTIIYAQGDCRLLLIYSKNHILEQYLAECVANITTN
YFDKDFPIVISTPKPIQSNPIYEYGNLLIQKLQSLNQFCLIILGNKNNETLKNVEKFTTESYVF
VLPPNYRKKKVILFFDNVKSVLDEAFQSRGKVLIVSFIGNFLEVNGKPLSHHLLNLSFKFCFI
QTLVLVPRTEISDGIIVNIRIFSWTINEQNNICSGKLDKIDQLDTWMSKKRTFLLNSNLFPL
QKTLNLKGCALKYHTTRFLPFSTKICLDNYCGPIEHILPAVSKAIHTTFKFTKRVKEGYVVF
PAYYKPNSTRKKNWRTYPHFRQDVIWFVPSSSEVPQWQSLVRTFNPLIWFLIAVACVFGS
FTMWILGSHSKYSSETSARLCISSVLMCTLLSHLGVGVAERYKGVVAVLFFMLWLYYCLVI
NTAYQSALSGLLVNPGYLPAIETLEELEESGLIMERRLTENGGSFWTKFNRYQRCQGSEQA
CFKKVSIDRTHALIHGSWYGKLYSNLFRDIYGKPQFKTLNQVEGTIYLCVYFTRFSCVLYSV
FESVMQRLVSAGLIEKWISWTLWVWNLKQGVVRGDTVFAFTLFHLQGSFYLLVFGLSLAS
LVFVIEILAYYIQNYCHLFIIIFYLYHKRSL
>BgerIr300
MILKCVFVASYLLVSCWSKLSIPNDEDLEEYSMAECVYNISSVYFDRDLPIFVQAPRKITNH
RYFNYMEKIIQKLQIQNELSIILLGGTKFRPIKNSENIMPGSYIIVLPTSLNAADLNYMTDTF
LQIAFFAYNPKGRAVIVNPEEKSLIVQHKTLPHFSLSIAWGYEFLQAIFLNPKPDVGLNPNR
TNTNFDVYSWSINDQIDLCSGEINKIRHFDTWLSQERRFSRHSKLFGVNEDVDLKGCKLN
LFMQSMPPYSWITRDGNYDGAVAKFLDFASGLINVSLKISQDIENMHIGFPSYYTDEPIIKI
WPKTYPHILKKITWFVPSGSEIPRWQSLWRTFSPLLWFLILLTFTFGTFTIWLLHKSTGES

IFSVFLFSLLTHLGIGVRDRYKGFVATLFFIIWLFYCLIINTAYQSELFRLLVNPGHFPAIQTL
KELGESHLIMERVLVFTGNESYLGNFIMHYNECDGSSVIECYKKVAIDRSHAILESADIYTIE
VISTSVEFSDEFGNPKLVPLKESLGTLYICLQISRMAGLLLDVLDNTLQKFVSAGIFDFWSSS
VHDTFRRQFITRNIIDQFVFSLDHLQGSFILFFLGHLLATVIYIIDISTHLIHYYFFLYIIAGCLS
RVYLL
>BgerIr301
MILKCVFVACYLLVGCWSQLSLPNEEELDEEYSMAECVYNISSVYFDRDLPIFVQTPRNTT
DHRYLNYMQKIIQKLQIQNQFSVTLLGGTKFGPIKNAETIKPGSYIIVLPTSITVDDLNFMID
TFLQIVSYAFNPKGKVVIVNPEETGAVVNGKTFPHLVLSFAWFSNFLHAIFLNPEPDAGLN
PNWTNTNFNVYGWRINDQTDLCSGDLDNIRLFDTWLSQEARFSRDSRLFQVDENLDLKG
CELHLDWSDLPPHSWVTSNGDYGGSVGNVLDVVSDLINVSFRTNQDLKNPHIGFPSYYTD
EAQRKSWPKTYPHIFAKLTWFVPSGSQIPRWQSLWRTFSPWLWFLILLTFTCGTFTIWLL
HKSTGESIFSVFLSSLLTHLGVGVTDRYKGLVATLFFTIWLFYCLLINTVYQSELFRLLVNPG
NFPAIQTLKELEESHLIKERDIETTEERSYLGYMIKQYNRCESHPLIECCKKVAIDRSHAILA
RDSDTIKVISTSVEFSDEFGNPKLVPLKESLSTLYFCLQILRMSGLLLDVLDNTLQTFISAGL
FDFWSSSDHDIFRRLFIARDIIDQFVFSLDHLQGSFILFFLGHLLATVIYIIEISTHFTLNCIFL
YTIAGCLSRVYLL
>BgerIr302C
MIQKCVFVASYLLVSCWSQLSLPNEEELDKEYSMAECVYSISSVYFERDLPIFVQTPRNTTD
HRYFNYMQKIIQKLQIQNQFSVILQGGTKFGPIKTAETIKPGSYIIVLPTSLKAADFQFMADT
FVQIILYASNPKGKLVIVNPEEKGVVVNGKTFPHLVLSFAWLSNFLQAIFLNPEPDVGLNP
NRTNTNFDVYGWTINDQTDLCSGDLDNIRLFDTWLSQEARFSRDSRLFQVDENLDLRGC
ELHLDWGDLPPYSWITLSGNYGGTVGKFLDVISHLINVRFRTTQDLKNAHIRFPSYYIDTA
QRRSWQQTYPHIFEKLIWYVPSGSQIPRWQSLWRTFSPWLWFLILLTFTCGTFTIWLLHK
STGECIFSVFLSSLLTHLGIGVPDRYKGLVPTLFFTIWLFYCLLINTAYQSELFRLLVNPGNF
PAIQTLKELEESHLIMERGIETNEEGSYLGNFIMQNNPCPAYSLLECYKKVAIDRSHAILTT
DSDTTKVVSTSVEFSDEFGNPKLVPLRESLGTLYFCLQILRMSGLLLDVVDNTLQNFISAGL
FDFWSSSVHDRFRRHFISRDIIDR
>BgerIr303
MILKCVFVASYLLVSCGSQLSVPNEEELDEEYSMAECVYNISSVYFDRDLPIYVQTPRNTTD
HRYLNYMQKIIQKLQIQNQFSVIFLGGTKFRPIKNSETIKPGSYIIVAPTPLNAADLNYMMD
TFLQIMLYAFNPKGKVVIVNPEETGVVVNDITFPHLVLYFAWLSNFLQAIFLNLEPDAGLN
PNRTNTNFNVYGWTINEQTDLCSEQIENIRLFDTWLSQESRFSRDSNLFQVNENIDLKGC
ELHLFWQILYPYSWITPSGNYSGGVRKFLDVISHVINVRFRTTQDFENAHIAFPSYYTDEA
QTKSWPKTYPHIFEKLTWFVPSGSQIPRWQSLWRTFSPWLWFLILLTFTCGTFTIWLLHK
STGESIFSVFLSSLLTHLGIGVSDRYKGLVATLFFTIWLFYCLLINTVYQSELFRLLVNPGNF
PAIETLKELEESYLIMERVIKTTGRGSYLGNMIMQYNKCESHPLIECYKKVAIDRSHAILTSD
SDTINVISTSVEFSDEFGNPKLLPLKENVGILYFCLQILRISGLLLDVLDNTLQKFISAGLFDF
WSSSGHDRFRRHFITHDIIDRFVFSLDHLQGSFILFFLGYLLATVIYMIEISTHFTLNYFLLY
TIAGCLNRVYLL
>BgerIr304P
MILKCVFVACFLLVSCWSQLSLPNEEELDEEYSMAECVYNISSVYFDRDLPIYVQTPRNTT
DYRYLNYMEKXIQKLQIQNQFSVILLGGTKFGPLKNAETIKPGSYIIVLPTSLKAADFQFMM
DTFVQIDYYAYNPKGKLVIVNPEETGVVVNGKTFPHLVLSFAWKCNFLQAIFLNPEPDAGL
NLDRTNTNFNVYGWTINDQTDLCSGDLHNIRLFDTWLSQEGRFSRDSILFQVNENLDFKG
CELHLDMEYWPPHSWVTFNGDYGGSVVELLDVVSDLLNVSFRINQDVKNPHIVFPTYYTV

KYPNEVLSQTYPHIFEKLIWFVPSGSQIPRWQSLWRTFSPFLWFFILLTFTCGSFTIWLLH
KSTGESIFSVFLSSLLIHLGVGVSDRYKGLVATFFFTIWLFYCLLINTVYQSELFRLLVNPGN
FPAIQTLKELEESHLIMERAIATTEEGSYLGKIVMQYNKCESHPLTECYKKVAIDRSHAILTS
XDSDTIKVLSSSVEFSDEFGNPKLVPLKESLGTLNLCLLILRMSGLLLDVVDNTLQKFISAGL
FDFWSSSVHGLFRRHFIAHDIIDRFVFSLDHLQGSFILFLLGHLLATVIYIIEISTHFTLNYFF
LYTIAGCLSRVYLL
>BgerIr305P
MSLKYIIVASYLLVSCFSQLSLPNEKDFEAYSMAECVYNISSVYFDRDLPIFVQAPRNTTDH
RYLNYVEKIIQKLQIQNQMSIILLGGTKLRPMKNSEILKPGSYIIVLPTSLNAADLNFMLDTF
LQIVLYASNPKGKVVIVNSEEKGVVVSSKTFPHVGLSIAWLSNFLQAIFLNPEPDAGLNPNT
TNTNFDVYGWTIKDQTDLCSGKIDNIRLFDTWLSLEGRFSRDSKLFKVNENLDLMGCELQ
LDWSYLPPHSWLTPSGNYDGSVGKFLDVVSELLNVSFITNQDLENTHVVFPSYYVDKTLT
KAWPQSYPHIFEELTWLVPSGSQIPRWQSLWRSFSPSLWFLILLTFTCGTFTIWLLHKSSG
ESIFSALVSSLLTHLGVGVHDRYKGFVATFFFTIWLLYCLIINTAYQSEMFRLLVNPGNFPAI
QTLKELEDSHLIKERVIETTEMGSYLGNFIMQYNICESNPXECYKKVAIDRSHAILARDSDT
IKVISTSVEFSDEFGNPKLVPLKESLGTVYFCLEIMRLSGLLLDVLDNTLQQFISVGLFAFWS
SSSHDIFRSLIIHRDIIDQFVFSLHHFQGSFILFFLGHLLATVIYIFEISTHFILNYFFLETLTGF
LSRVYIF
>BgerIr306P
MILKCVFVACYLLVSCWSQLSFSYEEDFEAYNMAECVYNISSVYFDRDFPIFVQVPTKITHH
RYFNYMEKIIQKLQIQNQMSIILLGGTKFRPMKNAEIVKPGSCIIVLPSYITVADLNYMMDS
FEQMDLYAYNRKGKVVIMNTDEKGVVVNGKTLPNFTMSIAWEANFLQAIFLDPEPDAGL
NPNRTNTNFNVYSWTINDQIDFCSGKINNIRLFDTWLSQEGRFSRDSRLFQVNENLDLKG
CELHLFWQKLPPYSWITLNGNYAGTVGDLLDDVSDVINVSFKITQDVNNIHIAFPSYYTET
ALNPWKQTYPHMFEKLTWFVPSGSQIPRWQCLWMTFSPLLWFLILLTFTCGTFTIXLLH
TSTGESIFSVFLSSLLTHLGVGVRDRYKGFVATVFFTIZLFYCLIINTGYQSELFRLLVNPGYF
PAIQTLKELEESHLIMERILSVSENESYLGNFIMQYNKCESHYFMECYKKVAIDCTHAILDV
DSYTTNIISPSVELSDEFGNPKLVPMKESLGTFYYCLQVMRLPGLLLDVLDNTLEKFISAGIY
DFWSSSVHNGLRRMIIDRDIIDRFVFSLNHLQGSFILFFLGHLLASVIYIIEISTHFILNYFFLH
TIRCCLSRVYLY
>BgerIr307
MILKYVYVATYLLVSCWSMLSIPKDEDLEEYNMAECVYNISSVYFDRDLPIFFLAPRKLTH
HRYFNYMEKIIQKLQILNQFSVILLGGTKFRPIKNAETIKPGSYIIVLPTSLNAADLTYMADT
LLQIDYYAYNPKAKVVIVNPEEIGLVVNDKTLAHFSLSISWKYEFFQAIFLNPEPLADLNPT
RTNTNFNVYSWTINDQIDLCSGEIDNIRLFDTWLSQEGRFSRDSKLFQVNENLDLKGCVL
NLYWLTMPPYSWVTRGNYDGTVAMFLHYFSDLINVIFRITQDLENIHIGFPSYHTEKALST
SWPKTYPHIFDKLTWFVPSGSEIPRWQSLWRTFSPLLWFLILLTFTCGTFTIWLLHKSTGE
SIFSVFLSSLLIHLGIGVRDRYKGFVATLFFMIWLFYCLLINTAYQSEFFRLLFNPGHFSTIQS
LKELEESHLIMESVLSLYETKSYWGNLLNQYNNCLDGVRGCYRKVAFDLTHAILDSDIYIIK
LVSTSVEFSDKFGKPKFVPFKEDMGTLYLCLQVRKMSGLLVVLLDNTLQKFISGGFFDFWS
SAIHNVFTSNYINLDIIDRFVFSLDHLQASFILFFLGPLLATVMYIIEISTHFILNYFFLHTIAG
CLSRVYLF
>BgerIr308
MIWKCVFVACYLLVSCWSQLSIPKYENLEEYSVAECVYNISSVYFDRYLPIFVQAPRKLTHH
RYFNYMEKTIQKLHIQNQFSVILLGGTKFRPMKNSEIVEPGSYIIVLPTSITVSDLNYMQDT
FVLMTLYAYNHKAKVVIVNPEEKHRLEINGKTLPHFALCIAWLCDFLQAIFLNPEPATGLN

PNKTNTNFNVYTWTINDQIDLCSGRIDSTRLFDIWLSQEGRFSRDSKLFQVNENLDMKGC
ELHLGWNYLPPHSWVSDVGHYEGTVGKILDVVSDLLNVSFRTNQDLRNIHIGFPSYYTDE
GLSKTWPKTYPHIFENPTWFVPSGSQIPRWQSLWRSFSPLLWFLILLTFICGTFTIWLLHK
STVESIFLVFVSSLLTHLGVGVSDRYKSFVATLFFTIWIFYCIIINTAYQSEMFRLLVNPGNFP
AIQTLKELEESPLIMESILSFSENKSYWVEILKQYNQCESRTAMECYKKVAIDRTHAILETEI
YTINVISTSVEFSDEFGNPKLVPLKESLGTLYFCLQIQRMSSLLLDVLDNILQKFISAGLFEF
WSSSVYDIFRRNLIVRDIINRFVFSLDHLQGSFILFFLGHILATAVYIIEISTHFTLNYFFLYIIA
GCLSRVYLL
>BgerIr309P
MRVQSVFVTTYLLVSCWSKLNIPKDEXLVEYSVAECVYNISSVYFDRDLPIFVQAPRKITHH
RYFNFMEKIIQKFQIQNELSVILLGGTKFQPIKNAEIVKPGSYIIVHSTSLNAADLTFMVDAF
LQIILNASNPKGKVVIVNPEENGVVVNGKTFPHFALSVAWLSNFLQAISLNPEPVAVINTN
RKSPNFNVCGWTIYDQTDLCSKQKDYLRLFDTCLSHEARLSRVSSLIQLTKISIZRDVSYIYY
GKLCLRVLGLSDAGHYEGIVRNCHYVVSELLNVSFRTNQDLKNPHIGFPSHYTDEAQRKN
WPETYPHIFEELTCFVPSGSQIPRWQNLWRTFSKLLWFLILLTFTCGTFTIWLLHKSTRZS
IFSAFVSSLLTHLGIVVSDRYKGFVATIFFTIWLFYCLIINTAYQSELFRLLVNPGNFPAIQTL
KELEESRLIIERIFSVFENKLHWVKFLKQYNKCESNPLIECYKKVAIDRTHTILDRDSDIIKVI
STAVEFSDEFGNPKHVQLKESLGTLYFCLQIMRLSGLLLDVFDNTLQKLISAGXFDFWSSS
DHDTFRRHFIARDIIDQFVFSLDHLQGSLILFFLGHLLATVVYIIEISTHFTLNYFFLYTIAGC
LGCVYLL
>BgerIr310
MGLKCVFVASYLFVSCWSQLSLPNEEDFEAYSVAESVYNISSVYFERDFPIFVQAPRNTTDI
RYLNYAEKIIQKLQIQNQLSVIFQRGTELRPMIHVKPGSYIIVLPTSITTAELYYIKDMFEQV
ALHAYNPKGKVVIVNPEEKGVAANRDAFSHFCLTTAWLYNFLQAIFLNPAAVVGLKTDRK
TPNFNVYGWTINDQTDLCSGDLDNIRLFDTWLSQEARFSRDSKLFEVDEHLDLKGCKLNV
YMESWPPFSWLGPDGKYYGTVPIVLNSFSDLINVRFRFTHDLKIAHIAFPSYYKEETLWPK
TYPHIFEKLIWFVPSGSQIPRWQSLCRTFSPFIWFLILLTFTCGTFTIWLLNKSTGQSIFSVF
LSSLLTHLGVGVPDRYKGFVATIFFTLWLFYCLIINTAYQSELFRLLVNPGNFPAIQTLKELE
ESPLIIESLFSISEDKSHWGKFLKQYNKCVNHFECYKKVAIDRTHAIVGTEMNTMEHLYTV
RELSDEFGKPKFVQLKEDLGTLYVSLVVRRMSCLFLGLLDNTIQKCISAGLFDFWSSLVNA
LSRLVFNDRDIIDRFVFSLDHLQGSFILFFLGHLLASFIYIIEIATHFILNYFFLHTITGFLGRV
HLF
>BgerIr311P
MILKCVFVACDLFVSCWSQLYLPYNEDFEAYSMADCVYNIRSVYFDRDLPIFVQAPSKIIPH
RYFDYMEKIIKKLQIENQMSVIFQRGTKFRPIKNAETLKPGSYIILLPTSMAAADWYYMRD
SFVQMASYAYNPKGKVVIVKPEEKGVVVNSKKFPHFGLSIALLANFLQAIFLNPESVVGLN
TNRKSPNFNVYGWAINDQTDLCSEQIDNIRQLDTWLSQEARFSRESKLFEVNEDLDLKGC
QLSVYMGNWPPYSWLGYDGKYHGTVSIFLNSFSDLINVRLRATHDLKIAHIVFPSYYTAED
PSTIWPNTYPHIFEKLTWFVPSGSQIPRWQSLWRTFSPLLWFLILLTFTCGTFTIWLLHKS
TGESIFSVFLSSLLTHLGVGVSDRYKGFVATLFFTIWLFHCLIINTAYQSELFRLLVNPGHFP
EIHTLNELEESNLIMETLLSLSETNSYWVNIFRQYKKCQGNTDTDCYKKVAIDRTHAILGK
LLKXTIEYLSTMAELSDEFGKPKVIQMKEGMGTLYLCLLVRRMSGLLLDVLDNTIQKFICA
GIFDYWSSLVHDIIRRKFTDRDIIDRFIFSLNHLQGSFILLFLGHLLASFIYIIEIIIHFTLNYFF
LHTITGCLGRVHLF
>BgerIr312P

MRLQSVFGETYLLVSCWSKLSIPEDEDLLEHSVAKCVYNISSVYFDRDLPIFVKAPSKIIPHR
YFDYTEKXIHKLQIQNQVSVILLGDSKIPSIKKGEILKPGSYIFVLPTSLAIADLYYMRDSFLE
VAFNAYNPKGKVVIVNPEEKGLLVNRKTFPHYSLSIAWTNEFLQAIFLNPEPEAGLXPYRT
NANFNVYSWTIKDQIDLCSGEIDNIRLFDSWFSQEARFSRDSKLFZVNENLDLKGCELHLF
WQRLPPYSWVTPSGNYGGTVGEFLDVILHLINVRFRTTQDFKNAHIZYSGRSKYYKAKNL
PNIFEEFTWFVPTGSEIPPWQSLWRTSSPFLWFLFLLTFTCGTFTIWLLHKSTGESIFSAFL
SSLLTHLGIGVSDRYKGFLATLSFIIWLFYCLLINTVYQSELFRLLVNPGNFPAIQTLKELEES
HLIMERVLSLSETNSYWVNFLKQYKQCQGNTVIECYKKVAIDRTHAILDREMYTIEALSTM
GELSDEFGNPMLVQLKEGLGTLYVSLQIQRMSGLLLGLLDNTFQKFISAGLFVFWSSSVHD
IFRRHFIDRDIIDRFVFSLGHLQGAFILFFXGHLCASLMYMIEISTHFILNYFFLHAILGCLSR
FYY
>BgerIr313
MILKCVFVACYLWVSCWSQLSLPNEEELEEYNMAECVNNISSVYFDRYLPIFVKAPSKIIPH
RYFDYMEKIIQKLQIQNQMSVILLGDTKIRPIKKGEIVKPGSYIFVLPTSLAIADLYYMRDSL
LEVGLNAYNPKGMVVIVNPEEKGLLVNGKKFPHYSLSIAWRNEFLQAIFLNPEPDGGINPN
TTNTNFNVYSWTINDQVDFCSGQIDKIRLFDTWLSQETRFTRDSKLFQVNENLDLKGCEL
HLFWQRLPPYSWVTPSGNYGGTVGKFLDVISHLINVRFRTTQDFKNAHIGFPSYYTAENL
STIWPKTYPNIFEEFTWFVPTGSQIPRWQSLWRTFTPCLWFLIFLTFTCGTFTMWLLHKS
TGESIFSAFLSCLLTHLGVGVRDRYKGFVATLFFIIWLFYCLLLNTVYQSEFFRLLVNPGNFP
AILTLKELEESHLIMERVLSLSETNSYWVNIFKQYKQCQGNSVIECYKKVAIDRTHAILDRE
MYTIEALSTMGELSDEFGNPKLVQLKESLGNLYVCLQIQRMSGLLLDVLDNSLQKFISAGLF
DFWSSSVHDLFRRNFIERDIIDRFVFSLGHLQGAFILFFLGHSLASLMYIIEISTHFILNYFFL
HAIVGCLSRFYYF
>BgerIr314P
NSKGKVVIVNPEEKGVAANRKAFSHFCLSMAWKAQFLQAIFLNPEPVVGLNTNRKSPNIN
VYGWTVNDQTDLCSGQIDNIKLFHTWLSQEARFSRDSKIFQVNEHLDLKGCKLNVYMKN
LPPYAWVGSDGTCLGIVGMFLHSFSDLINVRLRSTHDTKIAHIVFPSYYDEEAINSVCPKTY
PNIFERLTWFVPSGTQIPRLQSLWRAFSPFLWFLILLTFTCGTFTIWLLHKSTGESIFSALV
SSLLTHLGVGVPDRYKGFVATLFFTIWLLYCLIINTAYQSEMFRLLVKPGTFPVIQTLKELE
ESHLIMESAIGSTGKESYWANIFKQYKESQFNTVIECYKKVAIARTHAILDRHMYTIEVTSK
MGEFSDEFGNPKLVPVKESLGTLYFCLQIMZLSGLLLDVLDNILQKFISAGLFVFWSSSVHD
IIRHHSIERHMIDRFVFSLGHLQGAFILFLLGHLCASLMYMIEISTHFILNYFFLHAIAGCLSR
FYFF
>BgerIr315P
MSLKQNSDWFDVCLHEEDNMSLKCVFVASYLLVSCFSQLSLPNEKDFEAYSMAECVYNIS
SLHFDSDLPIFVQAPRKLTHHRYFNYMEKIIQKLQIQNQFSVILLGGTKFRPIKNAETVKPG
SYIIVLPTSPNAADLKYMHVTFVYMSLHAYNPKGKVLIVNPEXKSVVVNRKTLPNFALSIA
WKYEFFQAIFLNPELEAGVNPDRTNANINLYSWSINDQIDLCSGEIDDIRHSDTWLSQEAR
FSRNSKLFQFKENYDLKGCELHLWWSYLPPHSWVTFNGDYGGTVGKLLDVVSELLNVSF
RTNQDLKNPHISFPSYYSYESQRKAWPHTYPHIFAKIIWLVPSGSQIPRWQSLWRTFSPW
LWFLILLTFTCGTFTIWLLNKSTGESMFSAFVSSLLTHLGIGVSDRYKGFVATLFFTIWLFY
CLIIDTAYQSEMFRLLVNPGNFPAIQTLKELEESHLIMERLIETTEKGSYLGNFIMQYKPCD
SYPHIECYKKVAIDRSHAILTRDSDTIKVISTSVEFSDQLGNPRFLPLKESLGILYFCLQIHRM
SGLLLDVLDNTLQKFISAGLFDFWSSSDHDTSRRQFIARDIIDQFVFSLDHLQGSFILFFLGH
LLATVTYFIEISTHFILNYFFLHTITGSLSRVYLL
>BgerIr316P

MSLKCVFVAFZLLVSCFSQLSLPNEEDFEEYSTAZYVYNISSLYFDRDLPIFVQAPTNTTDH
RYLNYVEKIIQKLQIQNQMSVILLGGZKYRPMKHSEIVKPLSYIIVLPTSHNASNFNFMVDA
FLQNFFYLFCTPPILRVRLXHEEKGVVVNSKTFPHVGLSIAWLSNFMQAIFLNPEPDAGLN
PNRTNTNFDVYGWTIKDQTDLCSGKIDKIRLFDTWLSLEGRFSRDSKLFKVNENLDLMGC
ELQLDWSYLPPHSWLTPSGNYDGSVGKFLDVVSELLNVSFRTNQDLENTHVVFPSYYVDK
TLTKAWPQSYPHIFEKLTWLVPSGKQIPRWQSLWRSFSPSLWFLILLTFTCGTFTIWLLH
KSSGESIFSALVSSLLTHLGVGVHDRYKGFVATFFFTIWLLYCLIINTAYQSEMFRLLVNPG
NFPAIQTLKELEDSHLIKERVIETTEMGSYLGNFIMQYNXECYKKVAIDRSHAILARDSDTI
KVISTSVEFSDEFGNPKLVPLKESLGTVYFCLEIMRLSGLLLDVLDNTLQQFISVGLFAFWSS
SAHDIFRSLIIHRDIIDQFVFSLHHFQGSFILFFLGHLLASFIYIIEISTHFTLNYFFLQILTGFL
SRVYLF
>BgerIr317P
MILKCGFVATYLLVSCWSKLSIPKDEDLEEYSMAECVYNIGSVYFEZDLPISFHAPSKITHR
RYFNYMEXIIQKLQIQNQFSVILLGSTKLRPVKNAKVLKPGSYIIVHATTFHLADIIYMVDTF
LQIALYSYNPKAKVVIVNPEETSAGINGKRLSHSVLSLAWKYNFLPAIFFNPEPAAGLNPNR
TNINFNVCSWTINDQIDLCSGEIDNIRHVDTWLSREGRFSRDSKLFHVTENLDMKGCELH
LFWTDFPPHAWVSDVGHYEGTVAKLLDVVSHLLNVSFRTTQDIKNPHIGFPCYYTENSIW
LRTYPHIFEKLTWFVPSGSQIPRWQSLWRTFSPLLWFLIXLTFTCGTFTIWLLHKSTGESM
FSVFVSSLLIHLGIGVRDRYKGFIATLFFTIWLFYCLLINTAYQSEMFRLLVNPGNVPPIQTY
KELEESPLIIERILRFSDNKSQWVEILKQYKQCHTVIECYKKVAIDRTHAVLDVDSYTINVIS
SSAEFTDEFGNPKLVPMKESLGTLYFCLQIKRLSGLLLDVLDNTLQKCNSAGLFDFWSCSV
LDKFRRHFIVRDIIDRFVFSLDHLQGSFILFFLGHLFSSVIYIIEISTHFIHYYFFLHTITGSLSR
VYLL
>BgerIr318
MILKYVYVATYLLVSCCSQWSLPYEKDLVGHSVAECVYNISSVYFDRDFPIFVQAPRNTTD
HRYLNYVEKIIQKLQIQNQMSVILLGSTKFRPTRNAKTVKPGSYIIVLPSFITLADLHYMVDS
FRQMALYAFNRKGKVVIVNPNDTDVEVNGKTLPHYALSIAWEASFLQAIFLDPEPVAELS
PTKTNTNFKVYSWSINDQTDLCSGQIDNIRLFDIWLSQEGRFSRDSKLFEVDEHLDLKGCE
LHLDWGVLHPYSWITLNGRYGGSVGELLDVVSDLINVSFRISQDLKNPHIVFPTYYREKYP
NAIWPQTYPHFFERLTWFVPSGSQIPRWQSLWRTFSPFLWFLILLTFTCGTFTIWLLHKS
SGESIFSVFMSSVLTHLGIGVSDRYKGFVATIFFTIWLFYCLIINTAYQSELFRLLVNPGNFPA
IQTLKELEESPLIIERVIETTAKGSYIGNVIMLYNECQGYSTFECYKKVAIDRTHAIFDRESDT
IKVISTSVEFSDEFGNPKFLPLKESLGTLYYCLQILRLPGLLLDVLDNTLQKFISAGLFDFWS
SSDQDKFRRHFVARDIIDHFVFSLEHLQGSFIVFFLGHLLATVIYIIEISTHFILNYFFLHIITG
CLSRVYLL
>BgerIr319P
MCLMCVFVACYLLVSCWSQLSLPNEEDFEAYSMAECVYNICSVYFDRDLPIFVQAPRQITH
LRYFNYVEKIIQKLQIQNQMSIILLGGSKFRPMRNAEIVKPGSYIIVLPTSLNAADLYYMQDT
FVHMALYAYNPKGKVVIVNPEEKGVEVNGKTLFHYSLSLAWIYEILQAIFLNPEPVAGLNP
NRRNTNFNVYSWTINNQIDLCSGQIDNIRHIDTWLSQEGRFSRDSKLFQVNEIIDLKGCEL
HLEWGNLPPYSRITPSRNYGGSVGELLDVVSDLLNVSFRISQDLENTHIVFPSYYEQTALNP
WQQTYPSIFEKLICFVPSGSQIPRWQSLWRTFSPLLWFLILLTFTCGTLTIWRLHKSTGEK
LLSVFMSQPLRXHLGIGVSDRYKGFVATLFFIIWLFYCLIINTAYQSELFRLLVNPGNFPSIQ
TLKELEESHLIMERVIETTENGSYLCNFIMQYNKCESDTLTECYKKVAIDRTHAMLDREW
DTIKVISTSLEFSDEFGNPKLVPLNESLGTVYISLQIIRLSGLLLDVLDSTLQNLISAGIFDFW

SSSDHDTFRRHFIARDIIDQFVFSLDHLQGSFILFFLCHLLATVVYIIEISTHFIHNYLFLHTIT
GFLSRVYLL
>BgerIr320P
MILKCIFIACYLLASCWSRLSLHNEZDFEAYSMAECVYNISSVYFDRDLPIFVQAPRNTTXY
RYLKYVEKIIQKLQIQNEXSVILLGGTEFQRTENAKIIKPGSYIILLPTSITVDDLYYMQDTFM
LMAFYAYNPKGKVVIVNHDEKGVDVNDKTLPHFSLSIAWKNEFLZAIFLNPEPAVGLISNR
RNINFNVFSWSINDQTDLCSGQIDNIRLFDIWLSQEARFSRDPKLLLVNENLDLNGCLLHL
FWLDFPPYSMVISSGNHDGTVVKFLDVVSDLLNVSFRTNLVLKNPHIMFPTYYIEKYPNAV
STQTYAHIFEKLTRFVLSGSQIPRWQSLWMTFSPLLWFFILLTFICGTFTIWLLHKSTGESI
FSALVSSLLTHLGIGVSERHEGFVATLFFTIWLFYCLIINTAYQSELFRLLVNPGNFPAIQTL
KELEESLLIMERFLGSIENESYLGNFIMZYNSCTDNFECYKKVAIYRTHAILDFGFALIIALSI
TDELLDESRNPKLKSLKEHVGTLYVSLLVSRMSCILPDQLDKALQKFISAGFFDLWSSLFLN
KFRSHFIDRDIIDRFVFALNHLZGSFILFFLGHLLASVIYIIDFFYLVAYYVLKZNFYRDLHFL
>BgerIr321
MILKYIFVACYILVSCWSDLSLPNEEDFEAYSVAECVYNISCVYFDRDLPIFVETPRKITHHR
YFIYMEKIIQKLQIQNQMSVILLGGSKFRPMINAEIVKPGSYIIVLPTSITVDDFYYMQETFM
QMALNAYNPKGKVVIVNPKETGVEVNDKTLPHYALSIAWNANFSQTIFVDPEPFAELSPT
RTNTNFNVYSWSINDQTDLCSGQIDNIRLFDIWLSQEGRFSRDSKLFEVNENLDLKGCVLH
LFYGPMPPYSWVSDGKYEGIVAKFLDVVSDLINVRYRPTEDLENIHIGFPSYHTEISTYWP
KTYPHFFAEITWFVPSGSQIPRWQILWRTFSPWLWLLILLTFTCGTFTIWLLHKSTGESIF
SVFVSAVLTHLGIGVRDRYKGFIPTLFFTIWLFYCLIINTAYQSELFRLMVNPGNFPAIQTLK
ELEESHLIMERAIGSTGKESSLAKFILQYNECPDYPIFECYRRVAIHRTHAILEIGYILDIDLSE
TDEFLDEFRNPKFVPLKERVATFYICLLIKRMFGSLPDLFDNTLQKFISAGIFDFWSSSLLN
EIRGNYINRDIIDRFVFSLNHLQGSFILIFLGHSLASVIYIIEISTHFILNYFFCTQLQVA
>BgerIr322
MILKCVFVACYLLVSCWSHLSTPKYEDLVEYSVAECVYNISSVYFDRDLPIFFLAPRNITHR
RYFNYMEKIIQKLQIQNQFSVILLGGTKFLPIRNAETINPGSYIIVLPTSLNAADLNYMADTF
LQIDYYAYNPKGKVVIVNPEEMSLVVNDKTLPQFSLSIAWKYEFLQAIFLNPEPDGGLNAN
RTNINFNIYSWTINDQIDLCSGEIDNIRLFDTWLSQEARFSRDSKLFQVNENLDLKGCELRL
FWQDFPPYSWESDVGHYDGTVAMFLYYFSDLINVSFKISQDVENIDIAFPAYYTETGSHTA
WPKTYPHFIEKITWFVPSGSEIPRWQSLWRTFSPWLWFFIILTFTCGTFTIWLLQKSTGEN
MFSALVSSLLTHLGVGVPDSYKGFVATLFFTSWLCYCLIINTVYQSELFRLLVKPGNFPAIH
TLKELEESHLIMESVIVTTENETYLGNFIMQYNPCLAYPIFECYKKVAIDRTHAILSFDLNL
NIVLSITDDLRDDFGNPKLVPLKEGVAHFYVSLQVTRKSGLLVDLLDNTFQKFISAGIFDFS
LSSFHNRHRRNFIDRDIIVRFVFSLNHLQGSFIVFFLGHLLASVIYIIEISTHFILNYFFLHTIT
GCLGRVYLF
>BgerIr323P
MSMKYVFVASHLLVSCWSQFSLPNEEDFEAYSMADCVYNISSVYFDRDLPIFVQAPRNTT
DYRYLNYVGKIIXFMLMDFYAYNPKGKVVIVNSEEKGVEVNDKTLPHFSLSIAWKNEFLQS
IFLNPEPDVGLNPNRTNINFNVYSWSINDQIDLCSGEIDDIRHTDXYLYLEARFSRDSKLFQ
VNEDLDLKGFVLHLRWQNLPPHSWVSDVGHYEGAVAKFLDFVSVLINFSFRTTQNLENE
HIVFPSYYTEEHLSTMWPNTYPQIFKDXITWFVPKGLQIPRWQSLWRAFSPLLWFLIXLTF
TCGTFTIWLLHKSTGENIFSVFMSSVLTQLGIGVRDRYKGFVATLFFTIWLFYCLIIKTAYQS
ELFRLLVNPGHFPAIQTLKELEESHFIIERMFSLYENKSHWVKFLKQCNKCDSDPLTECYK
KVAIDRSHAILARDSDTIKVISTSVEFSDEFGNPKLLALKESLATLYYCLQILRLPGLLLDVL

DNTLQKFISAGLFDFWXRHFIAHDIIDQSVFSLDHLHGAFILFFPGHLCAFFIYIIEISTHFTL
DYFFLHTITGCLSRVYLF
>BgerIr324
MILKCVFVASYLLVSCWSKLSIPKDEDLEEYSMAECVYNISSVYFDRDLTIFFLAPRNITHR
RYFNYMEKIIQKLQIQNQFSVILLGGIKFRPIKNAETINPGSYIIVLPTSLNAADLNYMADTF
LQIDYYAYNPKGKVVIVNPEEISLVVNEKTLPQFSLSIAWRNEFLQAIFLNPEPDGGLNANR
TNTNFNIYSWTINDQIDLCSGEIDNIRLFNTWLSQEARFSRDSKLFQVNENLDLKGCELHL
DWQGLPPYSWFTFNGNYGGSVGELLDVVSKLLNVSFRTNQDLKNPHVGFPSHYTDEPQR
ENWKKTYPFIFEKLTWFVPSGSKIPRWQSLWRAFSPLLWFLILLTFTCGTFTIWLLHNST
GESIFSVFMSSVLTHLGIGVRDRYKGFVATLFFTIWLYYCLLINTVYQSELFLLLVNPGNFPA
IQTLTELEESHLIMERILSLSDNKSQWVEILKQYNQCENHPVIECYKKVAIDRTHAILDFGL
YVNLALCMTDELLDEFRNPKLVPLKEHVGTLYVSLLVRRMSGLLLGLLDNTIQKFISAGIF
DLCSSVFLDEFRRHFIDRYFIERFVFSLNHLQGAFILFFLGHLLAYVIYIIEISTHFILNYFFLH
TVRGFLGRVCLF
>BgerIr325P
RVKKELSVILLGGTKFRPIKNAEIVKPGSYIIVLPTSLNAADLNFMADTFLQIDYYAYNPKA
KVVIVNPEEMSPVVNDKTLPHFSLSIAWKYEFLQAIFLNPEPLADLNPNRTNTNFNVYSW
TINDQIDLCSGEIDNIRHIDTWLSQDARFSRDSKLFQVKENLDLKGCELNLYWLTMPPYS
WVTRGNYDGTVAMFLHYFSDLINVSFKITQDVENIHIAFPFHYTVSGSHTTWPKTYPHFF
EELTWFIPSGSQIPHWQSLLTTFSPWLWFFFLLTFTCGTFTIWMLHKSTGEGIFSALVSSL
LTHLGVGVPDRYKGFIATLFFIIWLFYCFIINTVYQSELFRLLVNPGNFPAIQTLKELEESHLI
MERFIDATEKESYLGNLIFQYKECPAYPVFECYKRVSIDRTHAILDFNFNVMKLLAVSLTD
ELLDEFGNPKLVPLKEHVAPFYLCLQVKRISGLLVDLFDITVQKFISAGLFDFSSSSFHNRLR
RIIDRDIIDRFVFSLNHFQGSFILFFLGHLLSSVIYIIEIATHFILNYFFLHTITGCLGRVYLL
>BgerIr326P
VILKYVFVACYLLVSCWSELSLPNEEDFEAYSMAECVYNISSVYFDRDLPIFVETPRKITHH
RYFXNYMEKIIQKLQIQNQMSVIFLGGTKFRPMKNAEIVKPRSYIIVLPISITVDDLYYMQET
FLQMALNAYNPKGKVVIVNPKETGVEVNDKTLPHYALSIAWEANFSQTIFLDPETFVELSP
TRTNTNFNVYSWSINDQTDLCSGQIDNIRLFDTWLSQEGRFSRDSKLFEVNENLDLKGCE
LHLFWRNMPPYSWVTSDGKYDGIVAKFLDVVSDLINVRYRPTEDLENIHIGFPSYHTEIST
YWPKTYPHFFAEIAWFVPSGSQILLWQSLWRTFSPLLWFLILLAFTCGTFTIWLLHKSTGE
SIFSVFESSLLTHLGIGARDRYKGFIATLFXTIWLFYCLIINTVYQSELFRLMVNPGSFPAIQT
LKELEESHLIMERAIGSTGKESSLAKFILQYNECPDYPVFECYRRVAIHRTHAILENGFILNI
DLSETNEFLDELRNPKLVPLKERVATFYICLLIKRMFGFLPDLLDIKLQKIISAGIFDFWSSS
LLIEIRGNYINRDIIDRFVFSLNHLQGSFILIFLGHSLASVIYIIEISTHFILNYFFCTQLQVA
>BgerIr327P
MSLKCVFVASYMLVSCWSHLSIHKYVDLVEYSVAECVYNISSVYFERDLPIFVQAPRNTTD
HRYLNYMEKIIQKLQIQNQMSIILLGGTKFQPVKNSKFLPIKNGDTVKPGSYIIVLPISLTFS
DLKYMANTFRQIASHAFNSKGKVVIVNPDEKGVVVNGKTLPHYAMSVAWKCDFLHSIFL
NPDPVVGLSXNFNVYGWTINDQTDLCSGDLDNIRLFDTWLSQEARFSTDSKLFEVNENLD
LKGCELHLVWLDFLPYSWVTDSGNNAGTVAEFLDVVSDLLNVSFRTNQDLKNPHIVFPTY
YIEKYPNAVLPQTYPHFFVKLTWFVPSGSQIPRWQSLCRAFSPFLWFLILLTFTCGTFTIW
LLNKSTIESMFSVFVSSLLTHLGIGVRHRYKGFIATLFFTIWLFYCVIINTAYQSELFRLLVN
PGNFPAIQTLKELEESHLIMERVIDSTGEGSYLNKFIKQYNPCPPYPIFECYKKVAIYRTHAI
LDFGFNVNIVLSMTDELLDESRNPKLKPLKEHVATMYLCLHIGRMSGLLPDLLDKALQKFI

SAGIFDLWSSLFLNKCRSHYNNRDIIDRFVFSLDHLQGSFILFFLGHLLASIIYIIEIATHFILN
YLFLHTVRGCLGRVYLF
>BgerIr328P
MILKCGFVATYLLVSCWSKLSIPKDEDLEEYSMAECVYNIGSVYFDRDLPISFHAPSKITHR
RYFNYMEXIIQKLQIQNQFSVILLGSTKLRPVKNAKVLKPGSYIIVHATTFHLADIIYMVDTF
LQIALYSYNPKAKVVIVNPEETSAGINGKRLSHSVLSLAWKSNFLPAIFLNPEPDASLNPNR
TNINFNVYSWTINDQIDLCSGAIDNIRLFDTWLSQEGRFSRDSKLFQVNEDLDLKGCELHL
FWQNLPPHSWVSDVGLNEGTIAKFLDVVSVLINVSFITTQNLENVHILFPSYYTEEDLSTM
WPRTYPHIFEELTWMVPSGSQMPRWQSLWRTFSPLLWFLIILTFTCGTFTIWLHHKSTG
ESILSALVSSLLTHLGVGVSDRYKGFVATLFFTIWLFYCLIINTAYQSEMFRLLVKPGTFPAI
QTLKELEECPLIMETVLSFSETKSYWANIFKQYKECQGHTVIECYKKVAIDRTHAILDVDSF
TIKLILTSVEFSDEFGNPKLVPMKESLGNLYYCLQVIRLSGLLLDVLDNTLQKCISAGLFVF
WSSSAQDIFRRLFIDRDIIDRFVFSLNHLQGSFILFFLGHLLASFIYIIEISTHFILNYFFLHTIT
GYLSRVYLF
>BgerIr329
MSLKCVFVACYLLVSCWSQLSLPNEEDFEAYSVAECVYNLSSVYFDRDLPIFVQTPRKITD
RRYFKYMEKIIQKLQIQNQFSVILLGGTKFRPIENAETVSPGSYIIVLPTSLAIDDLYYMRDSF
VQMALYAYNPKGKVVIVNPEEKGVVVHGKPFPLVALSLAWQANFLQAIFLDPEPVAELDF
NVYSWTINDQIDLCSGEIDNIKHIDTWLTQEGRFSRDSKLFQVNENLDFKGCELNLYWAT
LPPYSWVTRGTYEGIVAEFLHHFSDLINVHFRPTQDLENVHIAFPNYYTDDGHKAVWPNT
YPHIFEKLTWLVPSGKQIPRWQSLWRTFSPLLWFLILLTFTCGTFTIWLLHKSTGESIFSVF
VSSVLTHLGIGVSDRYKGFVATLFFTIWLFYCLIINTSYQSELFRLLVNPGNFPAIETLKELE
ESHLIKKRVIEASGNGSYLNEFIMQYNKCEGTIFEFCKKVAIDRTNAILYSGLYINLVLSIMD
DLLDEFKNPKLVPLKETVGTFYVSLQVRRMSSLLHDLLDNALQIFISAGFFDFWSSSFQNM
FRSRFIDHDIIDRFVFSLNHLQGAFILFFLGHLLASVIYIIEISTHFILNYFFLHTITGCLSRIYL
F
>BgerIr330P
MILKCVSVAIYLLLSCWSQLYLPNEEDFEADSVAVCVYNISSVYFDRDLPIFFPAPRKITHRR
YFNYMEKIIQNLQIQNQFSVILLGGTKFRPIKNAETIKPGSYIIVLPTPLSAADLTYMAVTFL
QIDYYAYNPKGKVVIVNPEEIGLVVNDKTLPLFSLSIAWKYEFFQAIFLNPEPLADLNPTRT
NTNFNVYSWTINDQIDLCSGEIDNIRLFDTWLSQEARFSRDSKLFQVNENLDLKGCVLNLY
WLTMPPYSWVTRGNYDGTVAMFLHYFSDLINVSFRITQDLQNIHIGFPFYHTEKALSTSW
PKTYPHSFDKLTWFVPSGSEIPRWQSFWRTFSPLLWFLILLTFTCGTFTIWLLHKSTGESI
FSVFLSSLLTHLGIGVHDRYKGFVATLFFMIWLFYCLLINTAYQSEFFRLLVNPGHFSTIQTL
KELEESHZIMETVLRLYKTKSYWGNLLNQYNNCLDGVRGCYRKVAFHRTHAILDSDIYIIK
LVSTSVQFSDKFGKPKFVPFKEDMGTLYLCLQVRKMSGLWVGLLDNTLQKFISGGFFDFW
SSAIHNVFTSNYINLDIIDRFVFSLDQLQASFILFFLGHLLATVMYIIEISTHFIHNYFFLHTIA
DCLSRVYLF
>BgerIr331
MSLKCIFVASYLLVSCWSQLSLPNEEDFEAYSVAECVYNISSVYFDRDFPIFVQAPRNKTDN
RYLNYAEKIIQKLHIQNQMSVIFQRGTELKNVKPGSYIILLPTSITSAELYYMRDSFAQMAY
HAYNAKGKVVIVNLEEKGVVVNGKKFPHYSLSMAWLTNFLHAIFLNPAPVVGLNTNRKP
PNINVYGWTINDQTDLCSGQIDKIKHIDTWLSQEARFSRDSKLFEVNEHLDLNGCKLNVY
MQFWPPYSWLGYDGKYHGTVSIFLNSFSDLINVRLRSTHDTKIAHIGFPSYYIEQYLSTTW
PQTYPHIFEKLTWFVPSGSQIPRWQSLWRAFNPWLWFLILLTFICGTFTIWLIHKSTGESM
FSALVSSLLTHLGVGVSDRYKGIVATLFFTIWLFYCLIINTAYQSELFRLLVNPGNFPAIQTL

KELEESHLIMERIFGLNETKSYWANIFKQYKQCQRNTDIECYKNVAIDRTHAILGIEINTIE
YLSTRGELSDEFGNPKFVPLKESLGTLYFSLQIQRMFGLLIDVLDITLQKFISAGFFAFWSSL
VHDVIRHKFIDRDIIDRFVFSLNHLQGLFILFFLGHLLASLIYIFEISAHFILNYFFLHTIVGWL
SRVHLF
>BgerIr332P
MSLKCVFVACYLFVSCWSKLSLPNDEELEEYNMAECVYNISSIYFDRDLPIFVKASSKIIPHR
YFDYMEKIIQKLQIQNQMSVILLGDSKIPSIKKGEILKPGSYIFVLPTSLAIADLYYMRDSFLE
VAFNAYNPKGKVVIVNPEEKGLLVNRKTFPHYSLSIAWTNEFLQAIFLNPEPEAGLXFNVY
SWTIKDQIDLCSGEIDNIRLFDSWFSQEARFSRDSKLFZVNENLDLKGCELLLFWQRLPPY
SWVTPSGIYGGTVGEFLDVISHLIYVRFRTTQDFKNAHIGFPSYYTADDLSTIWPNSYPNIF
EEFTWFVPTGSEIPRWQSSWRTFSPFLWFLFLLTFTCGTFTIWLLHKSTGESIFSVFLSSQL
THLGVGVTDRYKDFVATLFFIIWLFYCLLINTAYQSELFRLLVNPGNFPAIQTLKELEESHLI
MERVLSLSETNSYWVNFLKKYKQCQGNTVIECYKKVAIDRTHAILDREMYTIEALSTMGE
LSDEFGNPMLVQLKEGLGTLYVSLQIQRMSGLLLGLLDNTFQKFISAGLFVFWSSSVHDIFR
RHFIDRDIIDRFVFSLGHLQGAFILFXLGHLCASLMYMIEISTHFILNYFFLHAILGCLSRFYF
F
>BgerIr333P
MSLKCVFIASYLLVSCWSLLSLPNEEDFEAYSVAECVYNISSVYFDRDLPIFVQAPRNTTDH
RYLNYMEXIIQKLQIQNQMSVIFQRGTELRPMNHVKPGSYIILLPTSITTDDLYYIKDTFVQ
MAYHAYNPKGKVVIVNREEKAVSVDPEAFPHFVLSMAWLTNFMHTIFLNPALVVGLKTN
RKSLNFVVYGWTINDQTNLCSGQIDNIRHFDTWLSQETRFSRDSKLFEVDKHLDLKRCKL
NVYMESWPPYSWLGPDGNSYGTVSMFLNSFSDLINVRFRLTHDLKIAHIVFPSYYKEEDL
WIKTYPHIFEKLIWFVPSGSQIPRWQSLWRTFSPLLWFLIVLTFTCGTFTIWLLHKSTGESI
FSAFVSSLLTHLGIGVSDRYKGFVATLFFIIWIFYCLIINTVYQSELFRLLVNPGTFPAIQTLK
ELEESPLIIERMISVFENKSHWVKFLMQYNKCESHPRIECYKKVAIDRTHAIVGTEMNTIEY
LFTIGELTDEFGKLNLVQLKEDLGTLYVSLVVRRMSGLFLGLLNNAFQNCISAGFFDFWSS
LVNDVFRRIFIDRDIIDRFVFSLDHLQGSFILFFLGHLLATALYIIEIATHFILNYFFLHTIAGC
LGRVYLF
>BgerIr334
MSLNCFFVACYLFVSCWSKLSLPNKEDFEASNMAECVYNISSVYFDRDLPIFVQAPRNTTD
HRYLNYMEKIVQKLQIQNQMSVIFQRGTELKNVKPGSYIILLPTSITAADLYYMRDSFLRIV
FYASNPKGKVVIVNPEEKGLLVNGKKFPHFCLSFAWFAKFLQAIFLNPGPVVGQNTNRKS
RSFDVYGWTINDQTDLCSGQIDNIRRIDIWLSQESRFSRDSKLFQVNQHLDLKGCKLNVY
MDSWPPYSLVGPDGKYYGTVPIVLNSFSDLINVRLRFTHDSKIAHIVFPFHYIEEDLWPKT
YPHIFESLTCFVPSGSQIPRWQSLWRTFGPLLWFLILLTFTCGTFTICLLNKSTGESMFSAF
VSSLLTHLGIGVSDRYKGFVATLFFIIWLFYCVIINTVYQSELFRLLVNPGTFPAIQTLKELEE
SPLIIESLISVSENKSHWVKILNQYNKCESQSRVECYKKVATDRTHAIVGTEMNTAEYLFTV
GKLSDEFGNPKLVQLKEGLGTLYVCLLVRRMSGLLLGLLDNTLQKCISTGLFHFWSSLVHD
AIRRNFMDRDIIGRFVFSLNHLQGSFILFFLGHLLATVIYIFEISTHFTLNYFFLYTIAGCLSR
VYVF
>BgerIr335
MSLKCIFVACYLLVSCWSQLSLPYEEDLEAYNMAECVYNISSIYFDRDLPIFVQAPRSTTHN
RYLNFMETIIQKLQIQNQMSVILLRGTELLPMKNVKPGSYIIVLPTSITAGDLYYIKDTFVQI
AINAYNAKGKVVIVNPEEKGVRVNGEAFPHFGLSIAWLANFLQTIFLNPEPIVGLNTNRKT
PNFNVYGWTINDQTDLCSGDLDNIRHFDTWLSQEGRFSRDSKLFEVNEDIDLKGCKLNLY
MKNLPPYSLLGSDGKCFGIVAMFLHSFSDLINVRWRTTHDLKTSHIVFPSYYKENALSTIY

PKTYPNIFDKVTWLVPSGSEIPRWQSLWRAFSPLLWSLILLTFTCGTFTIWLLHKSTGEGI
FSALVSSLLTHLGIGVRDRYKGFVPTIFFMIWLFYCLLINTVYQSEMFRLLVNPGTFPSIKTL
KELEESHLIMERVLSLSETRSNWVNFLKQYKQCEGHTVIECYKKVAIYRTHAILDREMYTI
EYLSTMDEFADEFGKPKLIPLKEGLGTLYVCLQIKRISGLLLGLLNNTFQKFISAGFFDFWS
SSVHVVFRSNFIYRDFIDRFVFSLNHLQGPFILFFLGHLLASFIYVFEISTHLILNYFFLHAIA
RCLRRVYLF
>BgerIr336
MKKKSLKCIFVACYLLVSCWSQLSLPYEEDLEAYNMAECVYNISSIYFDRDLPIFVQAPRST
THNRYLNFMETIIQKLQIQNQMSVILLRGTELLPMKNVKPGSYIIVLPTSITAGDLYYIKDTF
VQIAIKAYNAKGKVVIVNPEENGVRVNGEAFPHFGLSIAWLANFLQAIYLNPEPVVGLNTN
QKTPNFNVYGWTINDQTDLCSGDLDNIRHFDTWLSQERRFSRDSNLFEINEEIDLKGCKL
NVYMKDFHPYSWLGSDGKYFGTVAMFLHSFSDLINIRFSITHDLKNTHIVFPSYYKENALS
TIYPKTYPNIFDKLTWFVPSGSQIPRWQSLWRAFSPLLWSLILLTFTCGTFTIWLLHKSTG
EGIFSALVSSLLTHLGIGVRDNYKGFFATIFFMIWLFYCLLINTVYQSELFRLLVNPGTFPAI
QTLTELEESHLIMERVLSFSETRSYWGNLLQQYKQCEGHTVIECYKKVAIYRTHVILDREIY
KIEYLSTIGELSDEFGKPKVVPLKEGLGTLYVCLQIKRMSGLLLGLLNNTFQKFISAGFSDF
WSSSVNVVFRSNFIDRDIIDRYVFSLNHLQGPFILFFVGNFMASVIYIFEISTHFILNCLFLHT
IACCLSRVYLF
>BgerIr337
MFQKICLILFVCSFLFNISRTDEFEQSMAECVVNIATRFFDENFPLLIYTPGDYYVIGDKLFQ
TLYKQIPFPKIMFANTAFLQVRQQIKPESYIIIVPPVESNDDVKFIENMFTKIQEEAFNAKG
KVILVNFWMVYIEVNLHRIAHFLLQLALKYGFVHTVVLDQQLLYYGKGLIDETDIHVFSWS
INDQLNICSRFIDIITHEDTWNSMERRFELQSTLFKHNNKINMKNCVLKVGLQQYPPFVFK
FPMELVGPIVEFVEIFCKIINANCRIRNWKYTSVVRDLHIVFPAVYGTTDIFPKTWKSYPYM
RTDIAWFVPSGEKHPSWKSLFRTFSPLVWTFVIITFACGVFTMWLQQKYLGRSSGRSVNY
DIIFSTLLMHLGVGVPDRSKGFVAVMFFALFLYYTLIINTAYQSAYFGLLVDPGNSPPIQSVK
ELNESGLIMERMINTYGNDSFWWFVMEYQACKEIMPDMCYEKVALKRTHSIMDNTWMS
KMISHLYLDRRGKPKYVIIDEIIGTSFLSLLGTILSPVLGSIMDETLHRIFETGIMFMLSDKAE
RIFRYGHLHKEFQIDSIYAFSISDFQGAFFLLILGHSLAFIVFLFDVLIQFITNTLYVYVIVFSF
EFCGFKI
>BgerIr338
MHPKIFLILFVCSFLLKLSRNEEFELSMAECLVHVVIKYFDTHLPVLIYTHGTNFPIGDKVIQ
IVHNKINFSQIIFSNTVSLLVRFRTRIKPGSYIVMVPPVESTEDVEFIKGMFRKIRDEAFNPR
GKVVFVNFWMEYNTVNDLSITHYLLNLALSFGFVDAVVLDQRILDEKSKELRIHKTNISVF
TWTINEQLNICSRFIDKMTHEDTWISMERKFELQSRFFKYSNKIDMKHCVLQVSLQNYPP
FVFNTTKERTGPIVAFIMIVCKSINATCKLIDWSHSSEGVDVHLIFPAIYGIVDPYPNTWRT
YPHMRTDIAWFVPSGQKQHSWRSLYRTFSPIVWVFILIIFSFGSVTMWLLQTSHRLLTGR
NSYSDIAFSALLMHLGMSVSAKFKGFVATMFFTLWFCYCLIINTAYQSAYFGILVYPGNMA
PIKSVKELEESGLVMERNLNVYGKSSDWDNIMQYRGCAEKSSDLCYEKVAVRRTHSVMD
NVWMSKIISPKFLDRRGNPKFVVINEFVGTFYLSLLGTLLSSTLEDIVDATLHTCVESGIMQ
MLADQLAILCSYRMLVNNIQTDPIFAFSISDLQGAFYLLILGHSFAFTVSTWEVLIHFMSNT
FYAFIIVNYFKCNGFNIV
>BgerIr339P
MHPKIFXSCLFVCSFLLKLSRNEEFELSMAECLVHVVVKYFDTDLPVLIYTHGTNFHIGEKII
QIVHNKINFSQIIFSNTASLHVQFKRRIKPGSYILMVPPVESKEDVEFIKGMFRKIRDEAFNP
RGKFVFVNFWMEYNTVNDLSIPHYLLNLALQFGFVDAVVLDQRIHDEKSKELHIHKTNIS

VFTWTINEQRNICSRFIDKMTHEDTWISMERKFELQSRLFKYDNKIDMKNCVLQVSLKNY
HPFVFNITKVLTGPIVTFIMIVCKSINATCKVIDWNHSSEGVDVHLIFPAIYGIADPFPNIWR
TYPHMRTDIAWFVPSGQKQHSWRSLYRTFSPIVWIFILIIFSFGSITMWLLQKSQRLLTGR
NSYSDIAFSALLMHLGMSVSAKFKGFVAVMFFTLWFCYCLIINTAYQSAYFGILVYPGNMA
PIKSVKELEESGLVMERNLNVYSKSSVWDNILQYRGCAEKSPDLCYEKVAVRRTHSVMDN
IWMSKIISPKFLDWRGNPKFVMINEFVGTIYLSLLGTVLSYILEDIVDATLHTCVESGIIQMF
TDQFEILCSYHMLVNNIQTDPIFAFSISDLQGAFYLLILGNSLALTISTWEVLIHFNNNIFYA
YVIVYSFKCCGFKILV
>BgerIr340P
MHPKIFLILFVCSLLSKVSRNEEFELSMAEYLVHVVIKYFDIDLPVLIHTQGTNFPIGEKNIQI
VHNKINFSQIIFSNSASLHVQFRKQIKPGSYIVMVPPVESKEDVEFIKGMFRKIRDDAFNPR
SKFVFVNFWMEYNTVNDQSIPHYLLNLALNFGFVDAVVLDQRIHDEKSKELRIHKTNINV
FTWTINEQRNICSRFIDKXHEDTWISMERKFELQSRLFKYNKKIDMRNCVLRVNLQHYPP
FVINNTRGYTGPIVAFIKIVCRSINATCKLIDWNESSKGKDVDLIVPVIYGIVDPFPNIWRTY
PHMRTDIAWFVPSGQKQLSWRSLYRTFSPLVWTFIVITSSFGCITMWLLTGRNFNSDIAFS
ALLMHLGMSVSAKYKGIVAVMFFTLWFYYCLIINTAYQSAYFGILVYPGNMAPIKSVKELK
ESGLIMERHLYANGKHSASKNIMQYRVCAEKSPDLCFEKVGVRRTHSVMDNIWMSKIISP
KCLDRRGNPKFVVINEFVGTFYLSLLVTILSYILEDIVDATMQTVVESGIIQMFTEQLTIFCW
YSMLVNKFQTDPIFPFSISDLQGAFYLLILGHSLALTVSTWEVLIHFIVNIFYAYVIIYSFKCY
GFKILM
>BgerIr341
MQIKKFILILLYFWKGSKSVLKPITESRIENYLTDCILSISTTYFNKELPVLIQTPSVRRNKDF
QIGKQLFQKIQSEQIFSHIVFSISGSSKKIKITKPGSYIFILPPLLAYNDMHKTLQMYESIKQN
AHQPKGKLLVVATQKTAIPASMVYLKTSLNYGYSDVIVIEPDLAHRNINIFTWDVNEQMDI
CSGEIDNIKHSDIWISKKKKFLHKVILYPLYNRIDLRGCLVNATLAPFEPLIYFCFKRNNPSD
ILVTYDGLKMCGVFIRFLISLKQCLNFKIAIYSSINHRYRNFNFPAVHRNTHEWCACGSTYP
HFRCDYTWVVPSGKEIPRWQSLFRVFDTLIWFLITLTLSFGILTMWLLQKSPRHWSSSSST
FDTVIIYALLTHLGVGITERYKGFVASLFFLLWLYYCVLINTLYQSKYFGLLVDPEVLPPVKS
AKELENSGLILATGVHSNDDIDILKNINNYEFIRFITSFISKLATDRKHAFFTDLWNANRIS
NRYRDSRGKPQIIAIDENVVALLILMEFRKLPCVVLDIIDLIMYRVLETGLIAKWLEDETRIL
NYPYYLPPDVVRVFSFSLLQFQGAFYLLLLGVVISSFVFIIEMVFTLFCNSLIKKRFLRSMRN
>BgerIr342
MQIKTYILILLYFWKGCKSVLKPVTESRIENHLTDCILSISTTYFNKKLPVLIQTPSVRRNKD
FQIGKKLFQMLQSEQIFSHIVFSISGSSKKNNITKPGSYIIILPPLLTYNDMHKTLQMYESIRQ
NAHQPKGKLLVVATQKTNIPASMVYLKISLNYGYSDVVVIEPDFAHRNINIFTWDVNEQM
DICSGEIDNIKHSDIWISKKKKFLHKVILYPLYNRIDLRGCLVNATLAPLEPLIYFCYKRNNP
SDILLTYDGLKMCGVFIRFLISLKQCLNFKIAIYNSTNHRYRNFNFPAVHDSTHEWCACGST
YPHFRCDYTWVVPSGKEIPRWQSLFRVFDALIWFLITLTLSFGICTMWLLQKSPRHWSAS
SSTFDTVIISALLTHLGVGITERYKGFVASLFFLLWLYYCVLINTLYQSIYFGLLVDPQFLPPV
KSAKELEKSGLILATGVYLKDDAEIWKNINNYEFIRHTNPFISKLATDRKHAFFTDLWNA
NRISNHYRDSRGKPQIIAIDENVAALLILMEFRKLPCVVLDIIDSIMYRVFETGLIGKWLEDE
TKIINYPYYLSPDVVRVFSFSLLQLQGAFYLLLLGVVISSFVFIIEIVFTLFCNSLIKKRFQSSM
RN
>BgerIr343P
MQIKKCLIILLYFWNTGYSVLKPVNERRIEKYLADCILSISTTYFNLEFPVLIQTPSGRRNND
FRMGEKLFQMLQSEQIFSRIVFSISRSSKKFKNTKPGSYVIILPPLWTYNDMQKTLLMYEKI

KQNAYQPRGKLLVVATQKTTIPASNVYLEYSRRYGFSKVVVIEPDFAHRNINIFSWDVNEQ
MNICSGEIDNISHSDIWISGTKKFLHETNLYPLHNRIDLRGCILNATLPPCKPLVYFCYKHN
NPSDVLRTYNGIKMCGVFIHFLKSLREFLNFEIIIYDNSNYINRNFYFPALIDTEYELRACEN
SXPYFRYDITWIVPSGNQIPRWQSFFRVFDALIWFLIIFTLSFGILTMWLLQKSPIHZQASFS
TFDTVIISALLTHLGVGVTERYKGFVASLFFMLWLYYCLLINTLYQSKYFGLLVDPEVLPPI
KSLRELEKSGLILETGIHVGNGTELWNYLKNYEFILFTDSVYDKIARDRKNAFLTCLWNAN
RISNSYRDSRGKSQIITIDEKVATLFFFMEFKNLPCVLLEVIDSTMYRFYETGIIAKWLEYDI
RIVNYPSFLKPEVVKVFTFSLLEFQGAFXLILFGVVISLLVFVIELLFIPLSISFIKWRFRSSNR
>BgerIr344
MQMKKYILILLYFWNGCKSVLKPVTENRMENYLTDCILSISTTYFNKKLPVLIQTPSVRRN
KDFQIGKKLFQMLQSEQIFSHIVFSISGSSKKIKITKPGSYIIILPPLLTYNDMHKTLQMYESI
KQNAHQPKGKLLVVATQKTAIPASMVYSQVSLNYGFSNVVVIEPAFARSNINIFSWDVNE
QMNICSGEIDNTRHSDIWISKKKKFLHEINLYPLYNRINLRGCLVNASLSPLAPLIYFCYKR
NNPSEYLLTYDGLKMCGVFIRFLISLKQCLNFKITIYNKTNHRHRNFNFPTVLDSKHEWRA
CESTYPHFRYDFTWMVPSGKEIPRWQSLFRVFDAVIWFLIILTLSFGILTMWLLQKSPRH
WSASSSTFDTVIISALLTHLGVGITERYKGFVASLFFSLWLYYCALINTLYQSKYFRLLVDPE
VLPPIKSAKELENSGLILATGVHLKDDAEIWKYINNCEFIRYITPFISKLATDRKHAFFTHL
WEANRISNHYRDSRGKPQIIAIDKHVAAVLILMEFRKLPCVFLDIIDSIMYMVFETGLIGKW
LEDETWIMTYPHYLSPDVVRVFSFSLLQLQGAFYLLLLGVVISSFVFIIEIVFTLFRNSLIKKR
FQSSMRN
>BgerIr345
MQIKKCLIILLYFWNTGYSVLKPVNERRIEKYLTDCILSISTTYFNLEFPVLIQTPSGRRNND
FRMGEKLFQMLQSEQIFSRIVFSISRSSKKFKNTKPGSYVIILPPLWTYNDIQKTLLMYEKIK
QNAYQPRGKLFVVATQKTTIPASNVYLEFSRRYGFSKVVVIEPDFAHRNINIFSWDVNEQ
MNICSGEIDNIRHSDTWISGKKKFLHETNLYPLHNRIDLRGCILNATLPPFKPLVYFCYKH
NNPSDVLRTYNGLKMCGVFIRFLKSLGEFLNFEIIIYDNSNYINRNFYFPALIDTEYELRVCE
NSYPYFRYDITWIVPSGNQIPRWQSLFRVFDALIWFLIIFTLSFGILTMWLLQKSPIHWKAS
SSTFDTVIISALLTHLGVGVTVRYKGFVASLFFMLWLYYCLLINTLYQSKYFGLLVDPEVLP
PIESIRELEKSGLILETGIHVGNGTELWNYLMNYEFILFTDSVYDKIARDRKNAFLTSLWNA
NRISNSYRDSRGKSQIITIDEKVATLFFFMEFKNLPCVLLEVIDSTMYRFYETGIIAKWLED
DIRIVNYPSFLKPEVVKVFTFSLLEFQGAFYLILLGVVISLLVFVIELLFIPLSISFIKWRFRSSN
R
>BgerIr346
MQIKKCILILLYFWKGCKSVLKPVTESRIENYLTDCILSISTTYFNKELPVLIQTPSVRRNKD
FQIGKKLFQMLQSEQIFSHIVFSISGSYKKIKITKPGSYIIILPPLLTYNDMRKTLQMYESIRQ
NAHQPKGKLLVVATQKTAIPASKVYSQASLNYGFSNVVVIEPDFAHSNINIFTWDVNEQM
NICSGVIDNIRHSDIWISKKKKFLHEINLYSLYNRIDLRGCHVNASLSPLEPLIYFCYKRNSH
SEFLLTYDGLKMCGVFIRFLVSLKQCLNFKISIYNRINHRHRNFNFPTALDSKHEWRACES
TYPHFRYDFTWMVPSGKERPRWQSLFRVFDALIWFLITLTLSFGICTMWLLQKSSRHWS
ASSSTFDTVIISALLTQLGVGITERYKGFVASLFFLLWLYYCALINTLYQSKYFRLLVDPEVL
PPVKSAKELEKSGLILARGIYMKDDGGIWNYIDNCEFIKHTTPFISKLATDRKHAFFTDLW
DANRISNYYRDSRGKPQIIAIDENVAALLILMEFRKLPCVFLDIIDSIMYRVFETGLIGKWLE
DETWIMTYPYFLPPDVVRVFSFSLLQLQGAFYLLLLGVVISSFVFLIEIVFTLYCNSLIKKRF
QSSMRN
>BgerIr347P

MQIKKCLLLILLYFWNGCNSVLKPVTESRIEKYVADCILSISTTYFNKEFPVLIQTPIVRRNKD
FQIGEKLFQMLQSEQIYSHIVFSISRSSKKFKNTKPGSYVIILPPLLTYNDMHKTLLMHKKIK
QNAYQPKGKLLVVATQKTIIPAANVYFEFSRRYGFSKVVVIEPDFAHRNINIFTWEVNEQM
NICSGEIDNIRHSETWISGKKKFLHETNLYPLHNRIDLRGCILNATLPPFQPLVYFCYKHNN
PSDVLRTYNGLKMCGVFIHFLKSLREFLNFAIIIYDNSNYININFYFPTLIDTEYELRACENS
YPYFRYDITWIVPSGNQIPRWQSLFRVFDALIWFLIIFTLSFGILTMZLLQKSPIHWQASSST
FDTVIISALLTHLGVGVTERYKGFVASLFFMLWLYYCLLINTLYQSKYFGLLVDPEVLPPIKS
IRELEKSGLILERGIHVGSGNELWNYLKNYEFVIFAAPVYSKIARDRKNAFLTCLWNANRIS
NSYRDSRGKSQIITIDEKVATLFFFMEFKNLPCVLLEVIDSTMHRFYETGIIAKWLEDDIRIV
NYPSFLKPEVVKVFTFSLLEFQGAFYLILLGVVISLLVFVIELLFIPLSISFIKWR
>BgerIr348
MFVKYIWILLNVSTCCQSILKPLKDESTEINLAECIINIWKIYFNEDLPVFIQTPGTFEHPRH
RFAEFGQKFIQILHSQNEFPQITFDASEGNTHKRDKPILEKSGTYIVLLPVVQNMYDFKFVT
AMLLTMLHNGHSPKSRVVVIAVEKTRRRVREPASFHFLFGLLRYGFTQVILLEHKNGNGK
NIDVYSWFIEDQTNICSEYINSIIHIDTWLPQEKRFLQNTQLFRSSQITNLRGCVLKVYFAFS
APFSLPCKNGVCGIFHPIFKILKNVINFNFVTKAKELDEYDMVFPFIHEAVYTKFDCLMTYP
LYYFDMRWLVPSSLYVPRWKVLFYTFDYSIWTLVVLTFSCGSFTMWLLQKSSNHSSENSN
EGVIISALLTHLETGVGDKYKGSISAVFFLLWLYYCMIINTAYQSQFFDLLVHPMEMPEMK
TIKEVEQSGLILERTINSSFLEEDSYISFIKKYKLCKQNDSCWAGLYQKRTHAILSDTFSGKL
FLHRRRNDKEKPSAAILPEGVGTLYLSVGVRKWSLSCALQEAMDRILHRLMDAGFISRSH
TLHPWYLMFRQTKSNERPPFAFSLADLQSAFYLLILGILFSSSAFIIEKIIFYLRCRFCLQRIV
SILKYSGSF
>BgerIr349
MYNKYFWILLNLSVGCQSILKPFKNEFSEINLAECIINIWKNDLNKELPIFIQTPGNYQHPS
HRFSKVGQKLIQILQSHINFPQITFDASEANTHKRYEQGLVKPGSYIVLLPRMQSKSDYEIV
KDMLETMFWNVHSPKSKVVVAAVEKTIRRNREPNSNDFMYALLKIGFTQVVVLEPRSRS
GRYIDVYTWLINEQNNICSDNIDNIKLIDTWKPQEKKFIHGKSLFETNPNINLRGCVLNILF
SPAPPYIIPCKNGICGIINKIFKILKTLLNFNVAVKVKDIYDYDIVFPYMYDPEDTRFECLLTY
PLYYFDMRWLVPSSLEVPRWQVLFYTFNRSIWALVILTFTCGSFTMWLLQKSSIHSASNA
NEGVIISALLTHLEAGVGEKYKGSVAAVFFVLWLYYCMIINTAYQSQFFERLVHPKEMPEM
KTIKEVEQSGLIMERVVSISSSSKYLSFVNNYKHCEQNGLCWDGLKHKRTHAILADTFCGR
LMERRTKNNRQKSSITILPEGVGTLYLSLGIRKWSLSCALQEAVDRILHRLIDAGLVSWSLK
THFWDMEIINVGSNELPPFSFSLLDLQSAFYLLIFGILVSSLVFTTEKVVSYLHSYVFLQTIII
VLENLGLY
>BgerIr350P
MYIKHIWILLNFSDVCQVKLKPPKDDMSEINLAECITSIWKTYLSKELPVFIQTPGSYNHSR
HRFAKVGQKLIQVVQTQINSPQITFDASKPNTLLKVKPMLEKSGSYIVLXPCVQTKRDYNF
VESMLDIMLHNGHSPRGRVIVAFVEKTLZENTRHQILVDFLIGLLKTGFIKVILLEPRSRSG
RYIDVYTZLINEQSSICSENLGNIKRIDTWIPQEKRFKYGSHLFQSDTNINLQRCVLKIHVAP
APPYIIPCNEGICGTIVSILKVLIYQINFKYAVKAKQLDDYDIVFPYKHNEADIRFECRLTYPF
YYXDMRWLVPSSLQVPCSKVLFYTFDHLIWVLVIPTFTCGSGTMWLLQISSIHSSSNTNEG
VIISAFLAHLETGVREKYKGSVAGIXFVLWLYYCMIINTAYQSPFFERLVHPIZMPEMKTVK
EVEQSGLIMESVIPSETHPFLKIYKNVDRMVHVGMVZNINVPMPSFLVLLLVNRLLKKYEM
RGVKPSIIILPEEVFTLFVALQEVMDRILHRLTDAGIVAWSLTNHPWRREIAEADRFELPPF
AYSLVDLQSAFYLFIFGSLISLSVFTAEKNVFYVRSLVFLQSVVSMLETLKVF
>BgerIr351

MATKIYVNLSFSGNMKEETMLFKSVWVLLYCCVGFQSNMIPLEDRSIEEQLAECILNISKT
YFRKDMPVLIQTPSSYEHPHHEYFQIGGKLIEKFSSQGDFPQMNFDVSKYHNRIKTDIEQK
AGSYIFIYPTLKAIEDVHFIYRMPKNVLDYGYNPRATVIIAGLDETARIDTLSATDFMIILYK
YGFNNVIVLDPIPYPNNTQQYKYIDIFTLTINEQSNVCSGIKDKIKQIDYWVIQEKRFYLKVD
LFPDHQNLDLKNCTLDMYLQVLPPYSYICENQLCGSISYFIDLLQDHFNFNIDIRYGIGEQN
YDLIFPTFLNSKIMKYECTFSYPIFHYDIVWIIPSGTEVPKWQNLFRTFSPLIWFLIFLTFVS
GTFTIGLIQKSSMEGNEPLANNSTLPIMNGVLTHLGAAIKERSKGFTASIFFILWLYYCMIIN
VAYQSRFFELLVNPNVLPEIQTIKELEESDVVKTRAVDLPTRSKEFSYIHKYQVCNDDPCW
EDLSTDKKYNYALLSEASHAKWMTRQFRDRRGKPKIKILAENLGTLFISLGLRKFSMSCIL
HDDVDKLLHRATDAGLSNFWYNLIMDTTLKRLVGQGRAPFSLLMSDMESVFYLFIVGHL
TAIVAFLVESVIFAFKQ
>BgerIr352
MNNKNIVIIVGLFSGCQSLLSLPENRVSEEYLAECIINISKTYFDKDKPLFIQTPSSILHYRHR
YFNIGKKIIEILHNEYDFSKIIFDVSTTIPTRKTETIQKPGSLLIIIPQVETKEDFELVDLMKSSI
LKNGYNPKGKVLIAAIENTTKKKSYVNSFNIFSYLCAIGYTETIIMEPDPTGQYFYIIGSLINE
QEIICSLKNHKMKYFDSWSTKEKRFVFNKERFPTRQRLNLGGCVLKVFVPPFFPYAYLCPY
GVCGLIRNFYRILENHLHFKLKIVLDQRDTDISFPYIDIGAVTKYECLVSYPFFKIDYGWLVP
SSLQLPKWKNLFYTFSPLIWALVLLTFVCGSFTMWLLQISSNHYNQTSKDNKEVILTALLT
HLEAGVGERYKGLVAVLFFTLWLYYCMIINTAYQSQFFELLVNPIELPRIMSINELKESELA
LKRIFYYENIESVHHLAKYPTCLNVTERCWEKLGKERIYALLIDVKNGLLMSRQSRNSRGK
PKFELLDDREGSLLISLAISKWSLSCILQEEINLLLHRLQDAGMLNSYYNTILWANSLVYEK
DDFISVFSFSLTDLQSAFCLLYFGLFSASFIYTAEICIFCLRNMIFLRVIRWSLRIFTIL
>BgerIr353
MNMKDIWMVVCFLSGCEALLRPPVLWSSEEYLADCITNISKTYFDKDKPLFIQTPSSYLHT
GHRYFKIGQKLIQILHNENYFSKIIFDVSTARHVNATETTQKPGSYVIVIPRVETQEEYNFV
DLMKTSIGQVAYNPKGKAIIAAIQSTSRSPLNSLNFFVNFFETGFTEIIILEPQVSFQHLNIFG
WTINDQEDICTQKIHKLKYIDSWNNQEKRYLKNKNLFPMREKLNLNGCTLKVTVPAAPP
YVYSCPQGICGFMKDFFRIMKEVIHFKFKVVSEYSKYDVIFPHIYEQTLINYECLASYPVFNL
DFGWLVPSGLQIPQWQTLFYTFNPILWSLVILTFVCGSCTMWLLQISSKDYTKTSKNNKE
VLFTALLTHLEVGVGERYKGVVAVVFFSLWLYYCLIINTAYQSQFFELLVHPRELPSVNSIS
ELKESNLIKERYFQLSSAYYIHVAKYPRCVGNIKCYDKLAKYRTHAILTDTMNALFVARHS
KNNRGKLKLKLLPGIEGTMLMSMRMQTFSLSCIFIDEVNLLLHRLLNAGIMDMWFKRLL
WLRALVFDKDIKSIYVFKFSLSDLQSAFYLLYFGLILAICMYITEIVIFSTRYMFFLMVIRRSL
NVFNYF
>BgerIr354
MNDVDIAIEQELSGCILKLSETYFNKELPIVVQTPAMWYPRHDWKKNYGDILLHTLYHYN
QIPQVTVGYEATKMKIGYQLKTHNAVQPGSYILQIPRGDRHEDRVWIELTLLRLAHDVYN
PNENFIIALNYFPYSYQRTAKGLFTWALEKGFINVIIMIPKRNSKRSTTTYIEQLDIFGWIPD
EQENICSMEVDQIIHLDSWDVENKTFVSNTNLFPKKKLTNMHYCPINAFLGVVPPFIFPKN
GMLFGSFIEVIQEYCARSKCGLISVTDARNNHIAFPTGYGSYIHWREGEFLYPYFILDFTWY
VPSGSQVEPWKSLFKAFTFKMWLLVVICSALGSLFLWLLDKFGNVLINGNMKILSISALKN
HIGNSRNKGPGHSFFVTLWLFYCLLINTAYQSTLFGLMVNPGEYPPIETFEELKKSGLTMK
TLVEIVGREDDKYEYKLCEESCLKEIAEYRNLSTLYSTYSGELITPSFIDDRGKPKVVPLKEV
VYSNYAFGQIIQLSSLIHNTLNTLLHRASASGLIEHWNRYHIEEWLYTFSYREIERVFALSLY
HVQGAFYILGAGFLIAFVLFFIEILNYNFCGSVPIVV
>BgerIr355

MLSVMFIINGNKADISRHDIEISIEQHLSELILNITETYFDPNFPIAAQTPATWYPQHDWRK
NYGDILLQMLHYYSDVPKVTLGYKDIEFSPGYFLRTGNAVQPGSYVLFIAKPRNDYDEYSIQ
TTFDRLRYDTYSPKGKFIIAINFFRQNYLHAAHYFFYTALYRGFINVILLIPKATSKRSEALR
MKQIDIYGWAPDEQNNICIAEVDKINHLDSWDVEMKAFVKKSNLFPKKKLTNMKYCKIDI
FLGHLPPFLFHDGRMNYGSFIELIVEYCVRFKCQLNPVFTSEYSHINFPAGYGPNHKSRECE
FSYPYFILDFTWFVPSGTKVEQWKSLFKAFTVKMWLLAVFTSVIGILFLWYLEKTTIIFRN
NICKVHNDLLSIAIFGIGVNGRKIGIGYAIFLTLWLFYTLIINTAYQSTLFGLMIDPGEYPPIK
TLAELKESGLEMRSLVKVTDDRRHDINNDGWFNNDWFIQIAEYRNLSVLYSTYSGELDRQ
FKLHLDRWGKPKIVPLEDIIRKEYAYGQVTFLSCLIHDNLNTLLHRAWTFGLIHKWNGAY
REWWQKSYQQQDMGITQALSIWHFQGAFYIFGIGVFVAVAIFVVEILISFFFNTR
>BgerIr356P
ILRNDIDIAIEQVLSGLILNLSETYFNKELPIVVQTPAMWYPRYDWKKNYRDILLQTLYQYN
QIPQVTVGYEATKMKTGYQLKTHNAVEPGSYILQIPRDGRHEDRVWIELTLLRFAHDVYN
PNENFIIALNYFPYSIQSTARDLFTLALEKGFINVIIMIPQKNSKRSITTDIEQIDIFGWIPDE
QENICSMEVDQIIYLDSWDVENKTFVSNSNLFPKKKLTDMHYFPINAFLGVVPPFIFPKNG
MLFGSFIEVIHEYCARSKCGLIAVTDARNNHIAFPTGFGSYIHWREGEFLYPYFILDFTWYV
PSGSQVEPWKSLFKAFTFKMWLLVVICSVFGSLFLWFLWFLPYXGFFLTNGNLKDLSSSAL
KNQIGMRINRRNKGPGHSFFITLWLFYCLLINTAYQSTLFGLMVNPGEYPPIETFDELKKS
GLTMKTLVEIVGMEDDEYEYKLCEESCLKEIAEYRNLSALYSTYSGELIMPSFLDHRGKPK
VVPLKEIAYSNYVFGQILRLSSLIHNNLNTLLHRASASGLIELWNRYHVQEWLYTFSYPELE
RVFALSLYHVQGAFYILGAGLLIAFVLFLIEILNYYFCGSVPIVV
>BgerIr357
MLSVMFIINGNKADISRHDIEISIEQHLSELIFNITETYFDPNFPIAAQTPATWYPQHDWKK
NYGDILLQMLHYYSHVPKVTLGYKDIEYSVYFLRTGNSVQPGSYVLLIAKPKNEVDEYFIQR
TFDRFSYDTYSPKGKFIIAINFFPQNYLHAAFYFFYQALCRGFINVILLIPKATSKRSEALRM
KQIDIYGWAPDEQNNICIAEVDKINHLDSWDVEMKAFVKKSNRFPKKKLTNMKYCKIYGL
LGYLPPFLFHEGIMHFGSFIELILEYCDRFKCQFQTVFSSKKSHINFPAGYGQNHKSRECEFL
YPYFVLDFTWYVPSGTKVEQWKSLFKAFTVKMWLLVIVTSVIGILFLWYLEKTTIIFRNNI
CKVHNDLLSIAIFGIGVNGRKIGIGYGFFLTLWLFYTLLINTAYQSTLFGLMIDPGEYPPIKT
LAELKDSGLEMRSLVKIMGDKGFDIKNDDIESSNDSFIQIAENRNLSVFYSTYCGELDRQAK
LLVDRWGKPKVVPLEDIIRREYAYGQVRFLSCLIHDNLNTLLHRAWSFGLIQHWNKEYRE
WWLKNYPEQNMAIVTALSIWHFQGAFYIFGIGVFVAVAIFVVEILVSPFYNTR
>BgerIr358P
MCTKLDSVITAILRNDIDIAIEQVQSGLILNLSETYFNKELPIVVQTPAMWYPRYDWKKNY
GDILLQTLYHYNQIPQVTVGYETTKMKTGYQLKTHNAVEPGSYILQIPRDGRHEDRVWIE
LTLLRFAHDVYNPNENFIIALNYFPYSFQSTARGLFTLALEKGFINVIIMIPQKNSKRSITTDI
EQIDIFGWIPDEQENICSMEVDQSIYLDSWDVENKTFVSNSNLFPKKKLTDMHYCPINAFL
GVVSPFIFPKNEIZFGSFIEVIHEYCARSKCGLISVTDARNNHIAFPTGFGSYIXWREGEFLYT
YFILDFTWYVPSGSQVEPWKSLFKAFTFKMWLLVVICSVFGSLFLWFLEEFGNVLTNGNL
KDLSYSALKNQIEMRRNRRNKGPGHZLFITLWLFYCLLINTAYQSMLFGLMVNPGZYPPIE
TFEELKKSGLTMKTLVEIVGMEDDEYEYKLCEESCLKEIAEYSNLSALYSTXSGELIMPSFL
DHRGKPKVVPLKEVVYSNYAVGQILRLSSLIHNSLNTLLHRASASGLIELWNRYHVQEWL
YTFSYPELERVFVLSLYHVQGAFYXYILGAGLLIAFVLFLIEILNYYFCGSVPIVV
>BgerIr359
MFIINSNKGDISRNDIEISIEQHLSELILNITETYFDPNFPIATQTPAMWYPQHDWRKNYGD
ILLQMLHYYSDVPKVTLGYKAIYYAPGHFLRTGNSVQPGSYVLLIAKPKNEDDEYYIQATF

DRFLHDTYSPKGKFIIAINYFPQNYLHAAHYFFYTALYRGFINVILLIPKTTSKRSEATRMK
QIDIYGWTPEEQDDICLAEIDKINNLDSWDVEIKAFVKKSNLFPKKKLTNMKYCKIYGLLG
YLPPFLFHEGIMHFGSFIELIIEYCVRFKCQLNPVFTSEYSHINFPAGYGQNHKSRECEFSYP
YFILDFTWFVPSGTKVEQWKCLFKAFTVKMWLLVVFTSVIGILFLWYLEKTTIIFRNNICK
VHNDLLSIAVFGIGVNGRQIGIGYAIFLTLWLFYTLLINTAYQSTLFGLMIDPGEYPPIKTLA
ELKDSGLEMRSKVKVMGDKGFDIKNDIELNNDFFIQIAESRNLSVFYSTYSGELDRQAKLL
VDRWGKPKIVPLEDIIRREYAYGQVTFLSCLIHDNLNTLLHRAWSFGLIQHWNGAFREW
WLKNYPEQDMAIATALSIWHFQGAFYIFGIGVFVAVVIFMVEILLSPFHSTRQSEK
>BgerIr360
MNRIKSDILRYDVELSIEQLLSDCIQNITETYFDPNLPIALQTPAMGYPQHDWRKNYGDLL
QHTLHDNNHSPQVTLGYKDIEYAPNYFLRTVNVVLPGAYVLLVAKPLDDDEDYLIEIAFH
RFFYDTYNPKGKFIIALNYFPQDYKYTAQMFFYIALIKGFIDVIILIPKKTYRTSVAHHVKQI
DIYGWTADEQNEICSSEINKINHLDTWNIEMKTFITNSNLFPIKKMTDMKYCEIHAFLGYL
PPFIFEDERVYHGSFAELILEFCFRSKCKIVPELTSKYSHIALPSGYELTNDSRECEYLYPYFL
LDFTWFVPSGKKVEQWKCLFKVFTIKMWLLVIFTSIFGILFLWLLELIRNIIWNDIYKAHR
DLLSIALFGIGVNGRKIGLGYAIFLTLWLFYTLLISTAYQSKLFGLMIDPGEYPPIKTLEELKQ
SGLPMRSLVTVAKDTNIDQSKSLCNNMDCFSQISDHRNLSVFYSTYSGELIRQRNIYRDNW
GKPKIVPLEEIIKSTYANIQIMYLSCLIHDKLNTLLHKAWAFGLIHFWNKEYRQLWEIYHPY
KETDMVVALKIWYMQGAFYILGVGLFFAIFIFTVEISLSPY
>BgerIr361
MAAILHQDVDIAIELHMSECILKIAETHFNSVLPIAVQTPATWYPQDDLRRNYGDILLHTL
NHYSYISQVTVGYKDTEYMNSGYHLKTRNAVHPGSYILQIPSEDRKNDWYWIDLTLHRFV
HDIYNPKAKFVIALNYFPYFYQRTASILFSWALRKGFIDVIVMIPKRSSKNAMTSNVEQFDI
FGWIPNEQENICLGEINEIVHLDTWDVKTKTFLSKSNLFPMKEMTDMNYCELNTYLGGLP
PFIFIEAGFMYGAFAKLLGEYCELSKCEIIPWDRVDIDYSITFPTGFRGDHNLRECEFLYPYF
ALDFTWYVPSGSKVEPWKSLFKAFTFKMWLLVVICSVFGTLFLWLIEKFRNMFTNANAK
DTNDLSYSAQSTHLGIGVNNNNKGPGYTFFIILWLFYCLLINTAYQSTLFRLMVDPGEYPPI
ETFEELKESGLTMKTLINSTRDGDDEYELCHKECFADIADQRNLAALYSTYSGELGMDIYR
DHRGKRKIAPLKEVMFTRYAYAQIIKLSCLIHNRLDTLLHRASASGLIAHWNAFYEQLWR
LVHPQRKVQHVFALTLFHVQGAFYILGAGLVIAFAIFLLEIFNYKFHGSNHIEI
>BgerIr362P
CFLLLGFGVSAILISHCLAALLWHDDDDIHTEQLLTDCILKVRQTYFDPEFPTAVQTPGMW
YNQHRWKKNYGDVLLQELNYDSQNPLLTAGYKDILFRDEDNVAFTIQTENAVHPGSYIVY
ITDQDDGYEAFWLDGVLGRLRFDIYNPNAKFIIATDFFPPSFERTVQIIFTWALRFGFFDVI
SIIPKRKTRWSRVPNVEKIEIYGWVPDEQGNICSTQINKIKLLDTZVVZTKTLLSNTNLFPIK
KIINMKSCTFKAYLGHVPPYLFTSGGVIVZSFAVLLQEYCSLQSCVISTVNENAVGIHIQFPT
GYEKDIMSHECGSLYPYMVTELTWFVPAGAKVEPWKRLFKAFTVKLWVLVVVSCISGILFI
WFIENARKFMSNENPKAANDXLLQGNVEYRNSSVLHSKYLGELPIKLFRDRKGNPKIVPL
TEIVHIKYQFARISTLSCLVYDTLNELMHRAWTHGLVDHWNTHFTDIWHKCLGKQQIEL
VYALTLWHLQGAFYVLGVGLLXTMLVFLVEILISSFTDADR
>BgerIr363N
LLSFVGFFAILISHCLAALLWQDDNDIHIELLLTDCILKVRETYFDPEFPTAVQTPGMWYN
QHRWKKNYGDVLLQELNYDSQNPLVTAGYKDILFRDDDNVAYTIQTENAVHPGSYIVYIV
DQDDGYEAFWLDGVLGRLRFDIYNPNAKFIIATDFFPPSFERTAQIVFTWALRFDFLNVIII
TPKRKTRWSRVPNVEKIEIYGWVPDEQGNICSAQVNKIKLLDTWVVETKTLLSNTNLFPI
KKITNMKYCTFKAYLGQVPPYLFVYDGVKYGSFVELLEEYCILQSCVISTVHENALGVHIQF

PTGYEKDIMSHECGFLYPYLATELTWFVPAGAKVEPWMSLFKAFTVKMWVLVVVSFISGI
LFIWFIDKARRFLNNEIPKAVNDFFTIAVFGIAVQDRNIGPGYAFFLIIWLFYSLLINAAYQS
TLFGLMVEPGEYPPIETFEELNKSGLTMKTSIQVDNVFIGYEGCFDEDCFKEISDYRNSSVL
YSKYLGELAIKMFRDKKGKLKVVPLTEIVQTKYQIARISTLSCLVYDTLNELLHRAWSHGL
FGQWNTNYMDLLFRLLGKQQIELVYALTLWHLQGAFYVLGVGILLSMVVFQVEVLTSSFS
DDHR
>BgerIr364P
YDFLFQLLCFVGFFAILISHCLGALLCQDDDMHIELLTDCILKVRETYYFDPEFPTAVQTPG
MWYIQHRWKKNYGDVLLQKLNYDSQNPLVTAGYKYILFRDEQNVAFTIQTENAVHPGW
YIVYITDQDDGYEEFWLDGVLGRLKFDIYNPNAKFIIATDFFPPPFERTAQIIFTWALRFCF
FNVIIIIPKRTRRWSRVSNVEQIEIYGRVPDENGNICSAQVNKIKLLDTWVVERKTLLSNTN
LFPIKKITNMKYCIFEAYLGHVPPYLFVYDGVKFGSFVELLEEYCILKSCVISDVHENALGVH
IQFPTGYEKDITSHECGFLYPYLATELTWFVLAGAKVEPWMSLFKAFTVKIXIEHVYGLIL
WHLQGAFYVLWVRLLLSMLVHLVEIFSSSFNDDDREK
>BgerIr365N
VLSSAVFFRILSSISPCLAAILWHDNDIHMEQILTDCTLKISETYFDPEYPTAVQTPGMWYN
QLGWKKNYGDILLQALNHGKIPLVTAGYKDIDYKDEYNRVFFMKTLNSVHPGPYIMLLPV
QDDGYEGYWMDRVFGRLLYDLYNPKGKFVIAIDFFPPTFEETAHIIFSWTIRKSFIDVILLIP
RRTSRNPMMSNIEEIDIFGWIPDEQGNICSTEINKFKLLDTWEVKTKSLLRKTNLFPYKKL
TNMKYCTFRVNLGVAPPYLFSHGGLKYGSFAKLLREYCHQQRCVISTVNDLDHINIMFPVG
IGDEIISRECDLLYPYLTTELTWFVPAGAKAEPWMSLFKAFTVKMWVLVVVSFISGILFIW
FIENARRFLSNEIPKAVNDFFSIAVFGIAVQGRNIGPGYAFFLIIWLFYSLLINAAYQSTLFGL
MVEPGEYPPIETFEELNKSGLAMKTLIQVNNKYLNYEGCSTDCFKEISENPNKSVLYSKYL
GELIIQLYLDNRGKPKVVPLTEIVHTSYAMAQINSLSCLVSNSLNELMHLSWSSGLFKYWN
TYFTVHWSSSHPEERIELVYALTLWHVQGAFYVLGVGFLLSMLVFLAEIFISSYYDVDREK
>BgerIr366P
TCVLFVLFVNFLNINKIMADILQQDPDIFMEQQISGCILNISETYFNPDHPIAVQTPAMWYA
IHDWGKHYGDLLLQTLHSYNSIPQTNVGYKDSISENDYTTRIHNTIHPGSYILLIAEQKTDH
DVWWIAKTFYRFQYDIYNPKGKFIVAVNYFPNYYKNTVQLLFYFALLKGFIDVIILIPRRSS
RKSKVPNVEYIDIFGWIPDEQENICSLEINNVKYLDSWNVKKKAFMLNSNLFPTKKLTNM
NYCEINVYLGYLPPFIVDVDGRDISWIIRTIDYGILLEDKLCNRFSNYCRTCSHZVSDWLZT
NLISRECEYLYPYLSLDYTWYVPSGEKAEPWRSLFKAFTLKMWILVIITSVFGNLFLWFLQ
KTRFILNIAIPKSTKDFLYIAIIGIDLNNTNQGPGYACFLVLWLFYSLLINAAYQSTLFELMV
DPGEYPPIETFDELKESGLSRKSGIFVIGKEDEYNICGDTCFADLARNRNFSVFYSIFSGTLE
MDLYRDRRLKPKFVPITDILNTKYAYGQISRLSCLVHKELNSLLHRTWAFGFIQVWNSDY
KRYWDRTHSPVETEGVLAISIWHLQGAFYILGIGLTLSFSVFLFEILIYPFQGSVSC
>BgerIr367P
NCVFFAFFATFLNVNKIMADILRQDPEIFLEQDITGCILNISETYFNPDHPIAVQTPAMWYA
IYDWGNHYGDLLLQTLHSYNSIPQAKVGYKDSFLKIGYTTRIHNTIHPEFYILLIAEPKINDD
VQWIXRTFYRFQYDIYNPKGKFITAIHYIPNYYQKIAQLLFXFALFNGFIDVIILMPRRTSRK
SKIPFSEYIDIFGWIPHGQENICSLQVNNVKYLDSWDIKRKTFMLKSNLFPTKKLTDINYFQ
INAFLGNLPPFIADLDGILHGSFITLNLEYXCEIDSVIYIKQAHIRFPTGFKSNLISRECEYLYP
YLSLYFTWYVPSGEKTEPWRSLFKVFTKKMWILLVITYVSGNLFLWFLQKTRFILNIAISKS
SKDFLSFATFGIDVNSTNQGPGYACFFVLRLFYSFFINKAYQSKLFELMVDPEEYPPIQTFE
ELKESGLSRKSVTFIIGKEKEYDVCSDTCFADLARSRNFSVFYSIYSGTLEMDLYRDRRLKP

KFVPVSDIPNTKYAYGQITGLSYLVHKELNTLLHRAFGFIQVWNSDZKQYWERTHSPIESE
GVFGISLWHLQGAFYSLVTGFVVSIFVFVFEILICPFQGSASC
>BgerIr368
MLPSKIYIAFSCWVGATIYAFTTNDDNPLEDILADCILNISKTYFNNELPTVVQTPETWRR
YGIPIDTQSEKFLEKLNTNSLIPLVTIGYIEGDLRCVQRNMIKPGSYIMIIPEVQNVQDQNWI
IDMFLRLKVNVHNSKGRMIIALNWDCTDQMFVSKALLDWAHDQSFDDAVVVIPERTPKK
VSNLNIYGWLSEEQENLCAVRLNKVRRFDTWSVVKKSFLLNTNLFPIKTMINKNHCRIKV
FYGNVPFSTTMYGKKLVGAIPLILKYGIKPNFEPIEKFKDNSRHLRYPIVFKSFEINRECGFT
YPLFALYSKWYVPIGKKIAPWRSLYKAFTPGMWIFVLFTSIFGNLFLGLIQKVKKLLFGVTD
RVDNIVTNVVLTHLGVGVRDSYTGPASVLLFSLLLFYCLLINTAYQSTLFGLMVEPGEYPPI
QTIEELKASNFVLKSFAFVYYGNNKKGIKYENYEYCGVDCFMEITENTEVAVLLAKSIGDLF
RDLSRRLHGTYKVLALTEIVETEYLGITSNIYNCIMFNRLETIIFRSAAFGLIDKWNKDFYR
WWKIKVHSKFQEPDIPALSLWHLQGAFYVLLLGVFGSIIIFIAESFKYSFTTIKFWTVQTLH
SG
>BgerIr369
MLASKLYIAVFCCICAPISAFISLDVNPMEDILAECILNISKTYFNNELSTVVQTPETWRRYG
IPIDTQSEKFLETLYTNSLTPLVTIGYVEGGLHFLQQNMVKPGSYIIIIPEVQTAQDQDWIIE
MFRRFQVNVHNAKGKMIIVLNWICNVDEFFVSKALLDWASDHSFNDAVVVIPERTPKKV
SNLNIYGWLSEEQENLCAVRLNKVRRFDTWSAEKKSFLLNTNLFPTKTMINKNHCRIKVF
YGNVPPFSTTMYGKQLMGAIPLILKYGIKPKFEPIEKFKNNSRHLRYPVIFSNIGGTRECGF
TYPLFASLSKWYVPIGKKIAPWRSLYKAFTPGMWILVLFTSICSNIFLGLIQKAKKLLFGVT
DKLDNIVTNVVLTHLGFGVRDSYSGPASALLFSLLLFYCLLINTAYQSTLFRLMVEPGEYPP
IKSIEELKASNLVLKSFAFMHNNDHKTGKYENYEYCGVDCFMQITENTNVAVLVPESVGEI
FRDLSRKHHGTYKVLALREIVDTEYLGITSNIYNCIVFDRLQTIIFRAADFGLIDKWNKDYF
RWWKIKVHSKFQEPDIPALSLWHVQGAFYVLVLGVLGSIIIFIAEIFKYSFTAI
>BgerIr370P
MLGSKIYITLCCCIITPICAFISHDDFPLEDILADCILNISKTYFNNILPTLVQTPETWRRYGIP
IETQSEKFLQKFNTNSLIPLVTIGYIESDLHIVQENVVKPGSYIMIIPEMKTVQDQYWIIDMF
LRIKVNVHNARGRMIIALNWDFTDHIFISKVLLDWAHDHTFYDAVVVIPESTPKKVSNLNI
YGZLTEDQGNLCNLRLRKVRRLGTWIAEKKSFLWNTNLFPTKMMINKYQCRIKVFYGNV
PPFSTVMYGNQLVGAIPEILKYGINPKFESAENFNANMLNLRYPEIHSHVLGNRECGLTYP
LFALNSKWYVPIGKXKVAPWRSLYKAFTPEMWIFVLLTSILGNLFLGLIQNAKKLLFGLTD
RVDNIATIVILTHLGVGVRDSYTGPASVLLFSLLLLYCLLINTAYQSTLFGLMVEPGEYPPIQ
TIQELKASKLVLKSYAMILDTNNKRRLEYKNYEYCGLSCLMKITENTDVAVILPESLGEIYR
DLTREWHGTYKMLAVREIVNTDYYGITSNLYNCIIFDRLETIIFRAADFGLIDKWNKDWFR
WWKINVHSKFQETGTPALSLWHLQGAFYVLVLGVVGSIIIFIAEILKYSFTAIKSWASQNLH
YTVCKTARVLHPCDVMHPLLHEFQAMSAYVLRPQ
>BgerIr371P
MLASKIYITLFCCIATSISAFISHGDNPLEDILAHCILNISKTYFNNELPTVVQMPKTWQRYG
IPIDTKSEKFLENLNTNSLIPLVTIGYIEGDLNIVQQNMVKPGSYIMIIPEVQTTQDQEWIIE
MFLRLQVNVHNARGRMIIALNWVCTVDELFVSNALLDWAYDNSFNDAVVVIPESTPKKV
SNLNIYGWLSDEQENLCALRLNKVRRFDTWLAEKKSFLLHTNLFPTKTMINNNHCRIKVF
YGNIPPFSTTMYGKYLEGAIPFILRYSVKPNFVPVEKYNNNLRNLRYPVVFSNVGGNRECG
FTYPLFASYSKWYVPIGKKIAPWRSLYKAFTPGMWILVLFTSMFSHLFLGLIQKAKQLFLG
VTDKLDNIVMNIVLTHLGVGVRDSYSGPASVLLFCLLLFYCLLINTAYQSTLFGLMVEPGQY
PPIQSIEELKASKLVLKSFELLFDDQKTVLKYKNYGYCGQDCFTDITENTDVAVLISESVGE

VFRDISRKQHGMYKVLLXREIVDTEYFGIVSDVYNCIVLKRIEKIIFRAAASGLIEKYNNKFI
WWWKIQVHSKFAEPGIPALSLWHLQGAFYVVVMGVLGSIINILIAETFKYSFIGIKSWSGQ
NLHS
>BgerIr372
MLAPNVCIALFCCIIRPISPFISHGDFHLENILADCILNISNTYFNNDLPIVVQTPETWERYGI
PIEIQSDQFFEKLNSNSLIPLVTVGYIEGDLSFKQRNIVKPGSYIMIISEMETAQGQYWIIDM
FARLQVNVNNSRGRMIIALNWVCTFDELFVSKAILVWAYDHSFNDAVVVIPQSTPKKVPN
LNIYGWLSEEQLNVCAERLNKVRRFDTWLTEKKSFLFNTNLFPTKTMINKNHCRIKVFYG
NVPPFSIEMYGKYLEGAIPRILKYGITPFFKSYKKLRKDTSSHLIYPVNFGKGDFFRECDLTY
PLFALNFKWYVPFGKKIAPWRSLYKAFTPGMWIFVLCTSISGNLFLGLIQKAKKLLFGATD
NVDNIVTIVVLTHLGVGVKDSYTGPASVLLFSLLLFYCLLINTAYQSTLFGLMVEPGDYTPI
QTIEELKASKLQLKSFTEVFDGHNRINVHESYEFCPFDCIMEITENSDVAVLVPQSLGNVFR
DLSRKKHGSYKTLALKEIEETEYMAITFNKRNCIIFDRLQTIIFRAADFGLIDKWNKDFFIW
WKIRVNSQFKEPEIPALSLWHLQGAFCVLLLGVLGSIIIFIAEIFKYSFTAMKSKA
>BgerIr373P
MLAPNVYIALFCCIISQISDFILHDDFPLEDILADCILNISDTYFNNAFQQLCKRQKLRVDMV
FLSIHKVTNSSKNZTQTLATVGYSKGNLNNGQKNMVKPGSYIMIVPEMQTAQGQYZIRDM
FGRLQVTVQNSRGRMIIALNWICTIDELFVSKVILEWANYCSFHNAIVVIPESAPKKVPNLN
IYGWLSEEQLNVCAVKLNKVRRFDTWLTEKKSFLLNSNLFPTKXMINKIHCRIQVVYRNA
PPFSFKMYYKYLEGAIPRILNYGITPLFKSHKEFKQNPRYLRYPVDFKHTGGKCECELTYPL
FALMYRSGKKLFLGEVTIKHLLLECGXSFLGLIQKAKKIWFAATDKFDNILTIVVLTHLGVG
VRDSYPGPASVSLFSLLLFYILLINTAYQXTFFGLMVDPGEYPPIQTIKELKESKLVLKSYAL
EFDNHKIVLVYKNYEYWDFSCFLEITENSDVAVLIAESTGEIFRDFSRQKHRSYKVLAIRKI
ADTAYYGIISAKYNCIIQDRLQTLIFRASEVGLIDKWNKDFFIWYKIRVHSTFQEPEIPSLSF
WHLQGAFYVLVLGVLGSIIIFIAESFKYSFTAIKSKAG
>BgerIr374P
MLASKIYIAIFCCISTPIFAFISQEDFPLEDILADCILNISKTYFNNDLPTAVQRPETWRRYGI
PIDTQSEKLLEKLNTKSLIPLVTVGYIESDLTIIQQNIVHPGSYIMIIPEVKTIQDQYWIINMF
HRFLFNVHNARGRMIIALNZDCSVDEDFVSKALLDWAYDHSFHDAVVIIPESTSKKVSKLH
IYGWLSEEQQNVCVSRLARVRRFDTWLAEKKSFFLNKSLFPTKTKINKNQCRVKVFYGNI
PPFSTVNYGKQLVGVIPEILRYGIKPDFEPIAKFKAKVRHITYPVDFSKIEQCRECGYTYPLF
ALNSRWYVPIGKKIAPWRSLYKAFTPGMWIFVLFTSISGNLFLVLIQKAKKLLFGATDNLD
NIVTNVVLTHLGVGVRDSYTGPASVLLFSLLLFYCLLINTAYQSTLFGLMVEPGEYPPIQTIE
ELKASKLVLKSYALRYENQTIKLKYENYEYCGEDCFMEISENTDVAVILAESVGEIFRDLSR
QKHGTYKVVALREIADTHYYGLRSDNYNCIIFDRLQTIIFRAADSGLIYKWNKDFFTWWKI
EVNSKFQESEIPALSLWHLQGTFYVLVLGVLGSIIIFIAEIFKYSFTAIKSRAGQNLHSV
>BgerIr375
MFASKIYVALFCCINAPIFAFISHEDFPLEGILADCILNISKTYFNNDLPTVVQTPETWRRYG
IIVDTLNEKFLEKINTNSLIPLVTVGYIKGDLRVKQQNRIKPGSYILKVPEVQTVEDQYWIM
EMFARFQANVHNARGRMIIALNWVCSVDEVLVSKALLDFAYAHSFHDAVVVIPESTPNIV
SNLNIYGWLSEEQHNLCGLRLDKVRRFDTWLAEKKSFLLNRNLFPTKTMVNKNHCRIMV
FYGNVAPFSSTMNGKLWGAIPQILEYSINVNYEPVKNFKGNPRYLSYPVDFSNSKGSRECD
ITYPIFALNSKWYVPIGKKIAPWRSLYKAFTPGMWIFVLFTSMFGNLFLGLIQKAKKVLFG
ATDNLGNIVTILVLTHLGVGVKDSYTGPASVLLFSLLLFYCLLINTAYQSTLFGLMVEPGEY
PPIQTIEELKASKLVLKSFALVSANQKVGLKYKNYEYCGETCFMKITENTDVAVLLPEAVG

YIFRDLSRQEHGTYKVLALKEIADTTYFGITADKYNCIIFDRLQTIIFTAVDSGLIDKWNDVF
FKWWKRRIHSKFQESEIPALSLWHLQGAFYVLVLGVLGSIITFIVEIFKYSFTGIKS
>BgerIr376
MLASKIYFALLCCISTPIFAFISQEDFLLEDILADCILNISKTYFDHDLPTVVQTPETWRRYGI
PIDTHSEKFLEKLNTNSQIPLVTVGYIQDDLRIAQQNLVKPGSYIMKVPELQTRQDQYWTI
HMFRRFHINVHNARGRMILALNYVCSVDELVVSKALLDWAYGHSFHDVVVVIPESTPKN
VSKLNIYGWLSEDQQNVCVLRIGKVRRFDTWLAEEKYFILNTNLFPTKTKINKNQCRVTV
FYGNVPPFSMENLGTELVGVIPEILKYGITPNFEPADNYHENSYNLRYPLDFSSYLEYRECA
LTYPLFAVNSRWYVPIGKKIAPWRSLYKAFTPGMWSFVLFTSISGNLFLGLIQKAKKLFFG
ATDNLDNIVTIVVLTHLGVGVKDCYTGPASVLLFSLLLFYCLLINTAYQSTLFGLMVEPGEY
PPIQTIDELKASKLVLKSYALVYDYQKFTLKYENYEYCGEDCFREITENSEVAVLLPESVGEI
FRDLTRQEHGEYKVTAIREIADTQYYGIDSDKYNCIINDILETIIFRAADSGLIDKWNKYYFT
WWKIEVHSKFEDPEIPALSLWHLQGAFYVLVLGVLGSIIIFIAETFKYSFTAIKSWAGQNLH
SV
>BgerIr377
MLASKIYIALFCFMSTPISAFISHEDFPLEDILADCILNISKTYFDHDLPTVVQTPETWRRYG
IPIDTRSEKFLEKLNTNSQIPLVTVGYTEGNLLIKEQNMVKPGSYIMIIPKVNNVEDQYWII
KMFARLIFNAHNARGRMILAVNYVCSVDEVFVSKSLLVWAYDNSFNDAVVVIPQRTRKKV
SKLSIYGWLSEEQQNVCVLRLAKVIRFDTWLVEKKSFFLNANLFPTKTIINNNRCRVTVFP
GNMPPFSTSTDGKQLVGAIPEILKYCITPNYEPAEKINDQSFHLRYPVDFSSSTDYRECELT
YPLFASNSRWYVPIGKTIAPWRSLYKAFTRGMWIFVLFTSISGNLFLGLIQKAKKIFFGATD
NVDNIVTIVVLTHLGVGVKDSYTGPASVLLFSLLLFYCLLNNTAYQSTLFGLMVEPGKYPPI
QTIEEVKASKLVLKSFALYFDNQNIGLKYEKYEYCGQDCFLEITENSEVAVILPETVGEILRD
VSRQKHGTYKVVAIREIADTSYYGIDSDKYQCIIFDRLQTTIFRAADSGLIDKWNKEYFTRL
KIKIHSKFREPEIPALSLWHLQGAFYVLVLGVLGSIIIFIAETFKYSFTAIKY
>BgerIr378
MLASKIYIALFCCISTPISAFISHEDFSLEDILADCILNISKTYFNDDLPTVVQTPETWRRYGI
PIDTHSEKLLEKLNTNSQIPLVTVGYTEGKFSIRTTVKPGSYIMIIPEVNNVEDKSWVRNMF
RRLIFEVHNARGRMIIAPNWYVRSGIDEVFVSKALLDFAYDHSFHDAVVVIPLKTSKKVSK
LSIYGWLSEQQNVCVLRLENVRRFDTWLVEKKSFFLNANLFPTKTIINKNRCRVRVFRGNI
PPFSTSTDDKKLVGAIPEILEYCITPNYELAEKVDHQSFHLRYPIDFSNSADSRECDLTYPLF
ASNSRWYVPIGKTIAPWRSLYKAFTPGMWIFVLFTSISGNLFLALIQKAKRLFFGATDNVD
NIVTIVVLTHLGVGVRDSYTGPASVLLFSLLLFYCLLINTAYQSTLFGLMVEPGEYPPIQTIE
ELKASKLVLKSYVLQYDNQKESLKYENYEYCDDDCFMEITENSEVAVILPETAGEILRDISR
QKHGTYKVSAIREVADTYYFGISSDQYKCIIFDRLQTTIFRAADSGLIDKWNKEYFTRWKIK
VHSKFRESEIPALSLWHLQGAFYVLVLGVLGSIIIFIAETFKYSFTAVKY
>BgerIr379
MSFSRICLVITISFGAVNSAFISYEDDPLEEAIADCVLNISKTYFNQELPTVLLMPDTWYRYE
DPIERRTDKFLQIYSKYNDIPGVTIANTNTPRIFRQPNIIYPGSYIMMAPEKLSNLKQEWEN
WVQDMLHRIQENVENAKARFVIVLNWYSTHFTNSVSKSLLDWAYNSGFSDVIVVMSKGK
KSRKVSNLDIFGWLSEEQTNLCAARISKIRRFDMWISEKKIFVFNRNLFPSKRIVTKDDCRI
SVFHGVAPPFAYKVNGVIFGAIPELLKYGVEPNFIPFGEWKNHSHNNIVFPIKYKIQDGIRE
CSWTYPIFAADITWYVPIGHKIEPWRSLYKAFTPEMWVLVIFTSAFCNLSLWMIQKIKQVY
FGIKRDIDNSIVTTVVLTHLGVGVKDSYSGSASVLLFSLLLFYSLLINTAYQSTLFGLMVEPG
EYPPIKTIEELEASNLRLKALERTFHNGIVSSEKYEHCNATCFLQITENSDYAVLIPKQLGEI
LRGYILREHGTQKVLSLTEMVTTYYLGMSIRRINCIVFMRLESLLFRAASSGLIHKWNNEY

VQLCRIKFYKYKSQNLPKLSIWHLQGAFYLLMVGVLGSVLIFFAEIIVYNFYSMQPRTPRRH
LCFCCLTVARI
>BgerIr380
MLASKNYIALFCCICTPISAFISQEDFPLEDILADCILNISKTYFNNDLPTVVQTPEAWRRYG
IPIDTHSEKFLEKLNTKSQIPLVTVGYIEGNLLSKEPNMVKPGSYIMIIPEVQTRQDQYWIIN
MFRRLQFNVHNAKGRMVIALNYVCSVDEVFVSKALLDWAFDHSFNDAIVVILQSTRKKRS
KLNIYGWLSEEQHNLCAVRLDKVKSFDNWIAEKKSLLLNRNLFPTKTMINKNHCRIKVFY
GNMPPFSVLLLGNLLVGAIPQILKYVINPNLFPLEKEDNNLHHLTYPVDFSDNRGSRECGY
TYPLFALNFKWFVPIGKQIAPWRSLYKAFTPGMWIFVLFTSISGNLFLGLIQKAKKLFFGA
TDNVDNIVTIVVLTHLGVGVRDSFTGPASVLLFSLLLFYCLLINTAYQSTIFGLMVEPGEYPP
IQTIEELKASKFVLKSYALLYSYQNVGLLYENYEFCKEDCFLDITENSEVAVILAEYVGEMLR
DVSREKHGSYKILALREIADTRYYGISSNKYTCIVFDRLQTTIFRAADSGLIDKWNKDFYT
WFKITIHSKFEEPDIPALSLWHLQGAFYILVLGVLGSIIIFIAEIIKYSFTATKPCAGQSLHCG
>BgerIr381
MLVLKIYIALFCFISIPISAFISHEDFPLEDILADCILNISKTYFNNDLPTVVQTPETWRRYGIP
IDTHSEKFLEKFNTNTQIPLVTVGYIEGDLTIIQQNVVKPGSYIIIIPPVDDVQDQDWIINMF
ARLIFNAHNARGRMIIALNYVLSVDEVFVSKALLDWAYDHSFINAVVVIPQSTRRKRSKLN
IYGWLSEEQQNVCFFRLAKVRRFDTWLAEKKSFFLNANLFPTKTIINNDHCSLRVYHGNV
PPFSTSTYGKQLVGAIPEILKYAITPNFELFEKFDDQSYHLTYPVDFVSSEDYRECHLTYPLF
ALNSRWYVPIGKKIAPWRSLYKAFTPGMWIFVLCTSISGNLFLGLIQKAKKLFCGATDNVD
NIVTIVVLTHLGVGVRDSYTGPASVLLFSLLLFYCLLINTAYQSTLFGLMVEPGEYPPIQTIE
ELRASKLVLKSYAFHFDYHKVRLKYEKYEYCGKDCFIEITENSDVAVLIADSVGEPFRDVSR
QKHGTYKVSAIREIADTDYCGIDSYKYHCILFDRLQTTIFRAADFGIIDKWDKEYFTRWKIK
VHSKFQEPEIPALSLWHLQGAFYVLVLGVLGSIIIFVAETFKYSFTAIKY
>BgerIr382P
MLAPNVCIALFCCIIRPISPSISHDDFPLEDILADCIFNTSNTYFNNDLPTVVQRPETWERYG
IPFETQSDQFFEKLNSNSLIPLVTVGYIGGDLSFIRRNMVKPGSYIMIISEMETAQGQYWIID
MFARLEVNVNNSRGRMIIALNXVCTFDELFVSKAILDWAYDHYFNVAVAVIPQSTRCSKL
NIYGWLSEEQLNVCAERLNKXEKKSFLLNTNLFPTKTLINKNHCRIKVFHGNVPPFSIEMN
GKYLEGAIPRILNYSITPLFKSHKELKQNRPHLVYPVDSALNSKYNRECELTYPLFALNFK
WYVPIGKXIAPWRSLYKAFTPGMWIFVLSTSISGNLFLGLIQKAKKLLFGATDNVDNIVTIV
VLTHLGVGVKDSYTGPASVLLFSLLLFYCLLINTAYQSTLFGLMVELCEYPPIQTIEELKVSK
LQLKSFTEVFDGHNXHESYEFFPFDCIMAITENSNVAVLVVQSLGNVFRDLSRKKHRSYK
MLALREIEETEYMAITSNERNCIIFDRLQTIIFRAADFGLVDKXKIRVNSQFKDPEIHAHSL
WHLHGAFYVLLLGVLGSINIFMTKFSNIHSLL
>BgerIr383
MITSKIYVTIFCCISTPILAFISHEDYLLEDILADCILNISKTYFNNDLPTAVQTPETWRRYGI
PIDTHSEKFLEKLNRNSLIPLVTVGYIEGNLQTRQLNMVTPGSYIMIIPKVNTRKDHLWIIE
MFSRLQFNVHNARGRMIIALNWVCVVDELFVSKALLEWAYDQSFNDVVVVIPASTPRKV
LSWNIYGWLSEEQHNLCAVTLKNVKRFDTWLAEKKSLLLNRNLFPAKTMINKNHCRIKV
YYGNVPPFSTIYDSRIVGAIPQILRSVIKPKFEPMQKRTLKSHHIIYPVQFSSTNARECDLTY
PLFALNFKWYVPIGKTIAPWRSLYKAFTPGMWIFVLFTSVSGNLFLGLIQKTIKLFFGAIDN
VDNIVTIVVLTHLGVGVRDSYTGPASVLLFSLLLFYCLLINTAYQSTLFGLMVEPGEYRPIQT
IEELKASKLALKSNALEVFNQSLSLVYENYEYCDEDCIIEITENTDVAVILPESAGKIFRDLSR
QKHGMYKLLAIREIADTNYFGITADKFNCIIFDRLQTFIFRAADFGLIDKWNKDFFTLWEIE
VHSKFQEPEIPALSLWHLQGAFYILVLGLLGTIIIFIAEIFKYSFTAIKF

>BgerIr384P
MLPSKIYIALFCCIITPISTCISHEDFPLEFILAECILNISKTYFNDDLPTIVQTPETWRRYGIPI
DTHSENFLEKINTNSLTPLVTVGYIKDNLQIAQQNMVKPGSYIMIIPEVQTIQDQYWIINMF
ARLQFNVHNAKGRMIIALNWICTFDEYFFSKALLDWAFDHSFNDAVVIIPEITPKKVSNLN
IYGWLSEEQKNLCAVRLNKVRHFDTWLAEKKSFLLNTNLFLTKTMINKNHCRIKVFYGN
APPFSLETNDKQLEGAIPLILKYGIKPNFESIEKLKKDSRHLRYPTAFSNYGGSRECQLTYPL
FALYSKWYVPIGKKIAPWRSLYKAFTPEMWIFVLFTSIFGIIFLGLIQKAKKILFGATNVLD
NIVTILVLTHLGVGVRDSYTGPASVLLFSLLLFYCLLINTAYQSTLFGLMVEPGEYPPIQTIE
ELKASKLVLKSYVVEFDNQKIVFNYRYPNYEYZRPDCFIKITENTDVAVIIPEFMGEIFRDLS
RRKYGMYKVLALREIADIEYLGITFDKYNCIIFDRLQTIIFRAADFGLINKWNNEFFTWYK
MIIQSKFQEPEIPSLSLWHLQGAFYVLGLGLVGSIIIFIAENFTAIKSCAGRNLHNV
>BgerIr385P
MLASNIYIALFCCISTPIFAFISHEDFPLEDISAXTILNISKTYFNNDLPTVVQTPETWRRYGI
PIETHSEKFLEKINTNSFIPLVTVGYIEGDLRXIIKVPDLQTAKDQYWIIKYMFARFQVNVH
NAKGRMIIAVNYVCSVDDEFVSKALLDWAYDHSFHDALVVIPESTSKXVSKLNIYGWVSEE
QQNLCAFRLDKVRRFDTZLAEKKSLLLNTNLFPTKTLINKNHCRIMVFHANVPPFSFTAY
GKQLVGAIPEIFKYVIKPNFEPVEKLNDDSYHIRYPVSSQGNYCECDLTYPLXATHPKWYV
PIGKKXSFTPGMWIFVLITSLLGNLFLGLIHKAKKILFGATDNLDNIVTIVVLIHLGVEVKDS
YTGPASVLLFSLLLCYCLLINTAYQSTLFGLMVEPGEYPPIQLIEELKALQLVLKSNVARFHN
QTSVMKYQNYEYCGEDCFMEITENTDVAVILPESGGEIFRDLSRQEHGTYKVLALREIADN
SYYGISSDKYNCIINDRLQAIIFRAADFGIIDKWNKNFFNWWKIEVHSKFQEZEIPALSLWH
LQGAFYVLVLGVLGSIIIFIAEIFKDSFTAIKSRAGQNLHSV
>BgerIr386F
MLASKIYIVLFCCFSTQISAFISHEDFPLEDILADCILNISKTYFNDDLPSAVQTPETWRRYGI
PIDTHSEKFLETLNTESLIPLVTVGYIEGDFCFVHQNMVKPGSYIMIIPEVQTRQDQYWIIN
MFTRLQFNVHNARGRMIIALNYVCSVDEVFVSKALLDWAYDHSFNDAVVVIPQSTRKKVS
KLNIYGWLSEEQHNLCAVILDKVKRFDTWLAEKKSLLLNRNLFPTKTMINKNHCSMKVF
YGNLPPFSIVMLGNRLVGAIPQILQYVLNPNLFPAKKFNDYPRYLRYPVAFSNFEGSRECA
VTYPLFALNSRWYVPIGKQIAPWRSLYKAFTPGMWIFVLFTSISGNLFLGLIQKAKKIFFGI
SDNLDNIVTIVVLTHLGVVVRDSYTGPASVLLFSLLLFYCLLINTAYQSTLFGLMVDPGEYP
PIQTIEELKASKLVLKSFHLQFDNRNLRLVYENYEFCAGDCFMEITENSDVAVILAEYVGE
MLRDMSREKHGTYKVVALREIADTTYYGISSDKYNCIIFDRLQTIFFRAADGLIDKWNKDF
YTWFKNRVHSKFKEPVIPALSLWHLQGAFYVLVLGVLGSIIIFIAEIFKYSLTATKPCTGQSL
HCGLTDKSNPPEHCNASIAR
>BgerIr387P
MLNIYFRLFVFTGVPISALISHDDDPLGEALADCVLNICRTYFNQDLPXHFFEIFSSRSDIPQ
APIGYITANLSVPQLKLMLPGSYIMEIPRGLKKQTRPWIIQMLRRMNVNIYNPKARLVIVV
NWYPVGDVFGMVAVLLEWSYEFAFSDAIVVVPRRGKYNKLTNLDVFGWLPEDERDICLL
QVKIIRRFNTWLVEKKSFACNRHLFPVKRMRNKYHCYIKAHHGSTPPFVYYNKGKVVGS
MVELVNYAVNLVAVEHAMKKKHDPVHSILLPLTIEPTHELSICKLTYPLFNVDCMWYVPV
GHKVAPWRTLYKAFYLZCGFVFSLLLHPITYSZGZSKNVKKKILFVLKDNIDKSMLVIFILM
HLNIGVRDSYTGPASVLLFSLLLFYSMFINTAYQSTHFGLIPGEYPPIKSTEELKASNLGLKT
NEKQLIYGDHNVRYITTFGHYSHCGIDCVLEMSENSDFAVLLSKHSGEAMRGKSREKHGSY
KIVSLTEIDHTYYVAIGSDQSNCLVLTKLETLVFRAVSSGLLQKWNDZYTLMWKRDVYPH
FEESGVSALSLWHVQGAFYVCVVGLLASVFTFVVEIILYASSKD
>BgerIr388

MLMLKIYFALFVFSGGPISAFTLHDDDPLEEALADCVLNISRTYFNQDLPTVIQTPETWRK
YGVPIKTYKDTFFEIFSSRSDFPQAPIGYITANLSVPQLKVMLPGSYIMKIPRGLNKQTHAW
IIQMLRRMDVNIYNPKARLVIVVNWYPVGNVFSMVGVLLEWSYEFAFSDAIVVVPRRGKS
NKLTNLDVFGWLPEDERNICLLQVNVIRRFNTWLVDKKSFAYKRHLFPVKTMRNKYHCY
IKAYHGHLPPYVYYDKGRVVGSMVELVNYAVALVSVEHAMKKKHDPVHSILLPLTIEPTH
ELSICKLTYPLFNVDHTWYVPVGHKVAPWRSLYKAFHPLMWICVLITSTFGNIFLWLIQKI
KQLRFGMKDNIDNSLLVIFILMHLNIGVRDSYTGPASVLLFSLLLFYSFLINTAYQSTLFGLM
VEPGEYPPIKTLEELKASNLGLKTNEKQLIYGDNDVRYINTYGNYSPCGIDCVLEMSENSDF
AVLFSKNSGEAMRGKSREKHGSYKIVSLTEIDHTYYNAIGSDQSNCLVLKKLETLVFRAASS
GLLQKWNDEYILLWKRDNYPHFEETRVSALSLWHVQGAFYVLVVGLLCSVFTFVVEILLY
ASSID
>BgerIr389P
MLASKIYIELFCCISTSISTFVTHDDFPLEDILADCILNISKTYFNNDLPTAVQTPETWRRYG
IPIESHSEKFLEKLNTKSQIPLVTVGYIEGNLKIVEKNVVKPGSYILIIPDIQTIQGQYWIINMF
DKFRINVHNARGRMIIPLNYVLSVDEVFVSKALLDLAYDHSFNDAIVVIPERTPKKVSKLNI
YGWLSEEQQNVCAVRLDKVKRFDIWIAEKKSLLLNRNLFPTKTMINKNHCRIEVHYGNLP
PFANIMSGVQLEGAIPEIVKYIIYPYLMTIRKSHESLRRISYPVRFSNTVGTRECGVTYPLFAL
NFKWYVPIGKKLLLGGASIKHLLLECGFLSCSHLYPVIYSWDZFRKLKDYFFGATDNVDNIL
TIVILTHLGVGVKDCYTGPASVLLFSLLLFYCLLINTAYQSTLFGLMVEPGEYPPIQTIEELK
ASKLVLKSFALEFEKEEIKLVYENYEFCPKACFMEITENSNVAVILGEYLGEILRDMSREKH
GSYKVVAIREIADTRYYGIASGNYNCILFDRLQTTIFRAADSGLIDKWNKDFYEWFKIRVHS
KFHVSEIPALSLWHLQGAFYVLVLGVLGSIIIFIAEIFKYSFTAIKY
>BgerIr390
MLAPNVYIALFCCIIRPISAFVLHDDFPLEDILADCIVNISNTYFNNDLPTVVQTPETWRRY
GIPIETQSDQFFEKLNTNSLIPLVTVGYIGGNLYTPQLNMVTPGSYIMTVPEMQTAQGQYW
IIDMLGRLQVNVQNPRGRMIIALNWICTIDELFVSKAILEWANDCSFTDAIVVIPESTSKKV
SNLNIYGWLSEEQLNVCAVRLNKVRRFDTWLTEKKSFLLNSNLFPTKKMINKNHCRIQVV
YGNAPPFLFKKDYKYLEGAVPRILNYSITPLFKSHKELKQNRGHLVYPVDSIQNSKYNREC
DVTYPLFALNSRWYVPIGKKMSPWRSLYKAFTPGMWIFVLCTSISGNIFLGLIQKAKKLFF
GATDNVDNILTIVILTHLGVGVKDSYTGPASVLLFSLLLFYCLLINTAYQSTLFMLMVEPGE
YPPVQTIEELKASKLVLKSYALVLKNKEVRLEYENYEYCDFSCFLEITENSDFAVLLPVSVG
EIFRDLSRQEHGSYKASVIREIADTYYFGIGADKFNCIIFDRLQTLTFRASEVGLIDKWNKDF
FIFYKIKVYSTFKDPEIPALSLWHLQGAFYVLVLGVLGSIVIFIVEIFKFSFTDIKSMAG
>BgerIr391P
MLASKMYIPLFCCISSSIFAFLFQEDFPLEDILADCILNISKTYFNNDLPTAVQTPETWRRYG
IPIDTHSEKFLEKLNTKSQIPLVTVGYIEGDLSIIQQNVVKPGSYIMIIPKVNNVEDQYWIIN
MFARLIFNVHNARGRMIIALNWYVRSDVDEVFVSKALLNWPYGNSFNDAVVVIPQSFXKV
SKLNIYGWQSEHHLCVLRLAKVIRFDTWLGEKKSFFLNTNLFPTKTIINKNRCRVTVDHE
NVPPFSTIZYGQQLVGAIPEILKYVITPNFELFKQTDDQSYHLRYPVDFSSSGDYRECHLTYP
LFALNSRWYVPIGKKIAPWRSLYKAFTPGMWIFVLCTSVSGNLFLVLIQKAKNLIYRATDN
VDNIVTIVVLTHLGVGVKDCYTGPASVLLFSLLLFYCLLINTAYQSTLFGLMVEPGEYPPIQT
IEELKASKLVLKSYALRYENQTIKFKYENYEFCGRDCIMEITENTDVAVILPESVGEMLRDM
SREMHGSCKVVPIREIADTHYYGVSSDQYNCIIFDRLQTIIFRAADTGLIDKWNKDFFTWW
KIEIYSKFEEPEIPALSLWHLQGAFYILVLGVLGSIIIFIAETFKYSLTAIKFCGKNLQCVX
>BgerIr392

MLASKFYIAIFCCISTSISAFISHEDFPLEDILADCILNISKTYFNNDLPTAVQTPETWRRYGI
PIDTHSEKFLEKLNRKSLIPLVTVGYIDGDLLSKEQNMVKPDSYIMIIPKVNNVEDQYWIID
MFNRLRFNVHNARGRMIIALNYVLSVDEVFVSKALLDWAYDRSFHDVVVVIPETASKKVS
KLNIYGWLSEEQQNVCVSRLARVRRFDTWLAEKKSFFLNTNLFPTITKIDKNQCRAKVFY
GNLPPFSTVNNRKQVVGVIPEILKYVITPNFESIMKFNKPLYHFTYPVDFSSNRGSRECDFT
YPLFALNFKWFVPIGKKIAPWRSLYKAFTPGMWIFVLFTSVSGNLFLGLIQKAKKLFFGAT
DNVDNIVTIVVLTHLGVGVKDCYTGPASVLLFSLLLFYCLLINTAYQSTLFGLMVEPGEYPP
IQTIEELKASKLVLKSFALKAENQELSLVYENYEYCGEDCFMDITENSDVAVLLSEIAGEIFR
DLSRQKHGSYKVLALREIADTRYYGITSDRFNCIIFDRLQTTIFRAADSGLIDKLNKDYFTQ
NKIEIYSRFEEAEIPALSLWHLQGAFYILVLGTLGSIIIFIAESFKYSLTTI
>BgerIr393P
MLGSNIYFALFFYFGVHTSASISYEDNSLEEALADCIINISQSYFNKKQPTVWQLPEGCYPY
VSPIRKNNERVLQVLSAHSDIPQVPVGFIQTNLRAEQRNIVTPGSYVMIIPEILSYKDQEWT
MAMFSRLRVNVHNPKAKFIMALNWYCEIDETSVAKQLLEWASLRGFNDAIVIIPKRNNSN
TVSTFDIYGWVSEDQKNLCIMTKNKITRFDTWIVEKKSFYLNRHLFPDKKMLSNKQCRIT
VFRGNQPPFMYESQGMLLGAVPELLKHSVQPTFKYIDQLKHETNFLAFPITYSSEIPSRECS
LTYPLFALDATWYVPTGQKVEPWRSLYKAFTPGMWMFVLLTSTFGNVFIWLIQKFKQLF
FGMDQKLSNIVTIALLIHLGCGVTDSFNGPASISLFCLLLFYCLLINTAYQSTLFGMMVEPGE
YPPIKTLEELKASKLGLNTFELLYTDQALVTSNNYGYCGLDCILRMTENSDFAVLISKPVGE
ALRDISRYIHGTYKVISLTEIVYTEYSAIXFSYKNCIIFNRIETMLFRASASGLIDKWNKDFV
WWWQVKIYFRFEEKGIPALSLWHLQGAFYLLVVGMLVSIIIFIAEILSYPFTTMN
>BgerIr394
MLHEVIPIEKNSFPIITLYSIFDFRMLPSKICFVLFCCVISCTSDLISEEDDILEETLADCILNIS
KSYFNKDLPTIVQTPETWRKNGIPIETQSDKFLERFSTYSGIPQVIIGHIKVNLRVQQPNIVK
PGSYVMLIPPVLSEQEQYWVIKMFARMWVNVHSPKSRFIIGLNWYCTVDTNFVAEALLN
WAYDHSFYDVIVVIPKIRSNEKLPKFDIFGWLSEDQINICSERLKKIRYFDTWIAENKTFLL
NANLFPIKEMINKNHCRIKVYHGNSPPFSYKLHEKLTGGIPELLRYSITPRFVPIIKFKNDSH
YLLTPVIYNQEHGLRECKMTYPLFALDATWYVPIGHKVAPWRSLYKAFTPGMWMFVFLT
STFGNLFLWFIQRIEQLFFGMKNKTENSIFIIVVLIHLGVGVRDSYTGPASVLLFSLLLFYCLL
INTAYQSTLFGLMVEPGEYPPIKTIEELEASKLGLKTYELEYQYESTKEFTTYNNNYGYCNV
KCFMEITEDGDYAVLVAKQVGNALRDYSRRIHGSYKVLSLPETVNTYYVTICSDAYNCMLY
HKLQKILFRAASSGLLQKWNTALSWWMKIVVHAQFREQGLPDISLWHLQGAFYMLLMG
VLFSIFIFVAEILVHAFSSN
>BgerIr395P
MNSLRYLLLLSTIYRPWNGFAILLSTEYDKALEQRLADCILDISKQYFVKNLPMAIQTPGM
WDQQWSSQHGDILIKSLSKENHISHLTVGKIRSFRPNKNSIRIPGSYVILIPSLNSNGLKMVL
IMLKRFWIDSRNPEGILVIGVMECTVSTNYLSAYLRIIFSYALGLTFNRVLVLVPESEPTGET
QQFNIFSYIPTEQSDICSISIDNIKEMDTWVFKDNKFLHGSNLLPKSKKLNLKKCLLQLAGY
TYAPMITVKKRRTWGVAVNFLILFCQEHNCRITLNTTQHSHILFPVAYSETRNYDRCEVTY
PYFKRTLAWYVPAGSEIPRWQSVLRVFNPLMWILVFCTAISGTLTLWLIRMSKLQSFDNQ
PIRGHTVVITALLTHLGFGDSNTYTGVTAVAFFSLZLFYCFIINTAYQTGLFGQLVSPGYFRG
VETLDELEKSGIVMKKLMTTSNKGNASFWSSFDKYEDCGKEPISCMQEIETSKNTAVLYDS
LLGGYIAKLQQDNWGRQQIVPIRHSASTIHFAFQITRLSYILHSKMEPLIHKVVNVGLIDKW
TREETIWRKLILSEPDGKSLFAFSLWHLQGGFYLLLMGESLAIVVFFVECCVK
>BgerIr396

MDNTVEHYLAECIINISSTYFNTALSIAVQTPSNWYLQHPWDITHGETLLKILNNEIHIPIL
TYGPVPDDYNSSDSAPTPDSYIFLISAINSDEEMNVAFSMFYRVVQEVSNGGAHVIIALVEP
FNNINHIISRLQYIFSVAFYARFSRVILVVPHKSRTLNNPKEIDIYGWLPNEQVNICSRFVRI
VRFFDAWKTKERRFLLGFNLFTVDSKINLNNCQINVTYYGNYPVIYPSNKRVDGIAVLFLD
AFSKVFNCTVKYNSSKSSHMQFPVFYTNIFKSDGHELTYPYFQMDINWFVPAGIEIPRWQS
IVRVFPTLMWGLVAATSVCGTFTLWLTLKHRRSSDDHQTGGHSILITALLNQLGFGDDIEC
KGPLAVMFYILWLYYCLLINNAYQAGLYGLLVQPGQYPPIETYEELMESGLRLKTKVKIDL
GYDAWGVIKQYEDCEIQIGDAVEACLDEMASTGNFAVLVVDALGRVIAKLLYEKHGKDKIS
RINKPMGNMFLGFQISKMAYILHNAMDTLLHRAVQGGLLDKWNFDELHKWEMNRKVR
TVDTVKPFTFEHVQGGFYLLSVGILIAFLAFIFEISHNFCHKLFV
>BgerIr397
MRFAGLFLVTFCLGRESTNALIATEMNHSLERLLSQCIVNISRTYFNTELPTAIQTMGTWY
PHSQSNIFNGDTLLKMLSTNNYNSLLTLGYVTNFYKSSYKIKTGSYIIIVKSLNTETEINMM
RTMLLRIDMEFVNPRSYCVIAVMESFKTKKNQNEIVSIILSMTFHIRLLRTIVLIPNDVRTSS
ISRFEVFSWIHNEQDNICALKLNKVKYIDSWIIGENRFALGSNLFPPQTKIDLKKCIINIVHS
DFHPFIQEYKRRVDGVAVEILKIFCKNSNCRISFNGPNSPHMTFPVIYDVAHLIDADIMTYP
YFRTTFLWFIPAGAELPQWQCLIRTFSNLMWVFVFITFITGAFTLWLIQKAVDLSAKTEHS
VVMTILITHLGASDTNTYKGFVPISFFCLWLFYCLLINTAYQSGLFGVMVQPGRLPALESIQ
QLKDSKLKMKTVINVHKDMYHWAQNHEMCNFDVRSCFKDVAEGKHAILYNEESGHWT
TRLFRDRNGKNTILPLKETVGTAYFGFYVGAFSYTFHNYLDSLFHRTTSAGLIDKWTQDR
FWRFKKIILELDREHVFAFSLFHLQGAFYVLLFGLKASFVAFLIEVMLPHFQQWY
>BgerIr398
MTIFHLLKTLIFCYKCAYAVLQPSSTKFSSELTLSKCILNISKSYFNTEHPIAVQTPGLWYRH
NHWKDNFGEELLKLLSIDNYFSQLTLGYTTNIGTSLSNVFHPGSYIILIPRLKNIADGNMVL
KMIRRIYKDARNPTAKTIICIKEPFQTIQEENYVVSSILTEAFAHRLTRVVLILPKSSHREYYI
FSFKHSDQRDMCSNTINAIWCLDVWSSNKNMFLKGSNLFPKQDKLNLKNCRLNIMFTNY
TPYIYELDRQKQGMIIDFFVLFCKKVHCILKWNVGDENFVHFPTFFRSTYQTDGYEMTYPF
LRSDFVWFIPQGLEVPRWKSIALVFTPGLWVLVLITCTFGMFTFWLIQKSQNRSENVRREI
GHTEIFTVLLTHLGWSYTNTYTSLVGVVFFNLWVFYCMLINTAYQAGLLAFIVSPGQLPAI
ETVEQLYRSNLNLTSYFEVINENSLSYPFCDWLYCYNNVAEGKTAVLDDVNFGRYNAIPFQ
DSRGRKLITSIEETVSTLYITMDISTFSYVFYNEFESLFHRYVSFGLLDRYVQYGKWLYPRR
YLIPIADEVFSFSLFHLQSAFYLLLLGNTLSLGSFLLELCLFCKT
>BgerIr399
MCEKHSPFSHRMNPFGRLLEFLSCLLGCSAVLIPSETDSSLQQTLAGCILNISKTYFDTDLPI
AVQTPEMWYPRHRFANTDGERPLQLLSSQSHIPQLTLGDVGEESYTWRNAMKPGSHIFII
PHVSPREMNEVASSMIRRTLRDSRNPNARVAFLFMRPFANAAQRNDMMHHLLTTCLFS
RLLKVKLIVPRQSASSHSLDVYTFVPNEQRDICSMRINVVWKIDVWIGHESKFLYGEDLFP
SQTELNLRKCQLRLYTSHLVPFSSWFSGRFLGPGIETLALFCEIVNCTLSVSTKFKYESHILF
IRLYTEYIIYDNWMTYPYFRLDVFWFVPSGRFLPRWRSMFKVFSPLLALFTCVTAVLGTFT
LWLLQKPVDGSKSSGHSVILTALLAHLYACDNIKFRDTSGSAFFSLWLFYCLLINTAYQTGL
FGLLVYPGHEPVIQTLEQLKKSGLHMKRNIVLSGTSELAAEIMQYEYCHDGDKSCYTEVAD
SGTAAVLEDNRVGKFTTDLIKDRWGNNRMVPIEESFATFLMSFEVVNLRSLLHSHLERLL
GRLANAGLLDKWSTDLMWVVKMQTAPDKPPAESVFAFSVIHLQGPLWLLLIGWSLSIST
FFAERFIYRRVREFLKERALKW
>BgerIr400

MNALGCLLTLSCLLWGAYSALPPRAMEELSECVLNISRTYFDPKFPVVVQTPDMWHNKY
KLSYPLVRAEKMFAEHFAQLTVGYIEERPEMPSNTVKPGSYVFFIPRRSQTTVILLSNMFT
RILQDARNPAAKVIVAFIEAFDEDFDRDMTALHYLVLHVGFSRVVFVIHIAAPKSLFLVITF
PLSGQANICLYQISHMEVEDIWIADERKFFRGSALFKTEKKLNFEKCTLNIYVVIQFPFIYIY
QGGFAGSGFEALQVFCQLYNCSLLFLKKLHGDVHASFPVIYGEGIIFDGSEMSYPQFQCEVL
WFVPSGMELPRWQCMFKVFDPLMSSLVLVTAASASLTLWLFKKSGGAYGHSVLLTVLLT
HLGVGDSNSYKGPVAVSFFTLWLFYCLLINTAYQSGLFELFVNPGREPPIETLQQLEHSGLE
MKRLGGFLGVDRLTYAFFKYNNCAGHNDLLCFREVAESRTTAVLADGYFGAIRSEMYVDR
FGNKKILRLAEAFATLHLSFYFIELSQILQDAMDRLLGRFTETGIIKKWNQDQIRNLQFLYS
SDVVDVDKVFAFSLNHLQGGFYILLIGGALSCLLFAVEILLTIAIHRRLD
>BgerIr401
MNALGCLLTLSCFLWGADSALPPRAMEELSECVLNISRTYFDPKFPVVVQTPDMWHNKY
KLSYPLVRAEKMFAEHFAQLTVGYIEQRPEMSSNTVKPGSYVFFIPRCSPTTVILLSNMFTR
ILQDARNPAAKVIVAFIEAFDEEFTRDMTALHYLVLHVGFSRVVFVIPTKEQNAIIVISFPLS
GQANICLYKISHMELEDIWIVHERKFFRGSALFKTEKKLNFEKCILNIYVVIQFPFIYTYQGV
FAGSGFKALQEFCQLYNCSLSFQRKLHGNVHASFPVIYGEGIIFDGSEMSYPQFQCEVLWFV
PSGMELPRWQCMFKVFDPLMSSLVLVTAASASLTLWLFKKSGDAYGHSVLLTVLLTHLG
VGDNSSYKGPLAVSFFALWLFYCLLINTAYQSGLFELFVSPGREPPIETLQQLEHSGLEMKR
FGGFLGVDRLTYAIFKYNNCAGHNDLLCFREVAESRTTAVLADGYFGAIRSEMYVDRFGN
KKILRLAEAFATLHLSFYFTELSHILQDAMDRLLGRFTETGIINKWNQDQIRNLQFQHSIDI
ITVDKVFAFSLNHLQGGFYILLIGSVLSCLLFAVEILLTIAIHRHLD
>BgerIr402
MNALGLLVWALPPQDLSEYVLHIGRTYFDPRQPVVVQTPDSWYKRFRLETPLVGAEALF
AQHFAQVTVGPDQWLRNAVKPGAYVFFVPRPTRATIALVVHMFQRIMDDAKNPAAPVV
VALIDAFDDEVLQMAPLHYCVLHAGFSRIVFVAATRTRRTTFAALSFPLSGQSDLCAFRISH
VDTVAAEGLFGQKPLNLQRCALDVHVSLVFPFFYIYESDYVGSGLEALGVFCRLHNCSLSF
HETIRNAVHASFPVVFGGGGRMSYPHFRAEALWIVPSGAELPRWQCIFKVFGPLMSGLVL
ATAATAALTLWLLRRSGGVGGQSAVLLSVLLTHLAAGDGGSPKGPVAGAFLGLWLFYCLL
INAAYQAGLFPLLVYPGREAPIATLDQLRHSGLQLKRLGGFLGLEPSAAMDILNYDSCADY
DVPRCFRDVAESRTSAILAEGVWGTTYAGRYVDRFGHKKAEVLPQPFATAHLSFYFAELL
HVLQEPMDRLLGRLSAAGILGKWERDQIRVLQSAYSRGVPRSETVFAFDLRHLQGPFSLLL
LGTASACLLFVAEKLRHRWVRSN
>BgerIr403P
MMDLIALVALFVGSCAMLRAPELDTSEEQVLCDCLLEVSKSCFDSQLPVAVQTPEMWRTP
SARGETLLQMLSSRSDLAHVTLGIPRTNDDDLGERSHNKVRPGAFLFFTSCPRDLDRCLNL
DNLMKMRTAKDSRNSEGPAVVAVMEPFASERHRNTSVAVLLDLCLVYYFARCVVLVPRR
ATEGTSVVVDVFSRSHSFCIAENELTVVLVDTWVAEGRGFLAGADLFPANLKLDFKGCRL
TVHFDTPSFPLVFTIREQFQGPGFEVLLMFCRKFNCSLSPNVHRAPDIIFPVAYTDDYILED
VGLSYPHFLESYTWFVPSGRKFPRWKVIFRVFSPLLSLLVFLTGVLGTLTLWLLIKPSARVG
KVGYSLLTHLGVGATDYFQGPLSVAFFTLWLFYCLLINTAYQSGLFTLLVDPGEHAPLEAF
AELEKSGLHMTSIINFRSDTKYSSEVLGKYELLQCSEGKYFCFQEVARSKSRAMXSDVYKR
QEYARFFKDPLGKPQVLHLKDSFVTINAAFHAPLFLLLMRPLDRVLSIAVDSGIVDKCMRQ
AFRDFVLFFPHKNQQRTEKVFAFTFEHLQGPFYLLMTGLSLSAVAYLLEISFCYCFQRKLFL
V
>BgerIr404

MAVFAVLCCCLLSVCACSLLPPALDFSTEIRLAHFIVEVSKTYFDAALPVAVLRYSRSRRAL
GETAPRVLHERMRLPQLVLGLSSGGDAGATVNRMVPGAYVLLVPCVDRPGTRAVVQMML
RRVFRDARNPDACAVVAVAEPCVTTPTSALGAFIRDLFSRALRKGVGRAVFVKPRLVGAV
EVWDAFAQAAPALQRDICSLRVSRVAYMDTWLPHERRFIRGTDLFPPPSQGPSGRLDLK
WCTLNVSFTNSFPFIFEAEGNIFGTALEVLVVFCMRFHCRLNMAGHVGSAHLMMPVMLE
DTGSSDPSSATYPHRRDNLAWFVPAGREFPRWQSLFRVYSPLMSLLVAATSLALLLLLVA
TRRGHGRRYEGALASLCFVAWSFFSLQMSTRYQSGLFGLLLYPGHPPPLESLAQLQSSGLR
MKNSVVLFGDSSLEPRLRHFEVCSEDLFSCLRRVAESDDTAYLCLEVAGKVYGSEFTDSWG
TVRVVPLKDTFLTLYNSLLLLKLRHLLFRHLERLMSRLDDAGILDKWTRDLVGLLRRTAK
REASSLVVLLPQKAFPFALGHLGGAFYLLLFGLFLSVAQFVGELLYFRHTTG
>BgerIr405P
NLGSFLFTFLVICGGYGKLLPPHMNTLRERLLGECILNIVETHISKDLPLAITASSMWYPGW
NRSHGDTLLEMLFSHAGITQFTLGYIEQADHYPNKFTPGSIIILIPRLRSNNDVIQMCMMFQ
RIVVDSRNPRAKLIIVFMEDEELIISQIVKLLLNIGFLSNFSRLIVVTQSVHLHKSKINQMSSF
DIFGFNPNEQNDICSMDIDKISYIDTWDSGQNRFLHNATMFQLPEEVNLNGCGIRISVHHS
HGFKGVSAKLLFLFCERFNCSIELNSSEFHIEFPVRYGILSPFDTDTATYPYFRADFKWFVP
AGEEIPRWQSFFRVFTPHMWVFASCTSAIGIFTLWLMQKYKRQFSRGPGHTVLITGLLTH
LGFGDSNNFSGYGPAMFYAIWLFYCLIINTAYQSGLLSFLVYPGQFPNIETEEQLLASGLNM
KRLIGIVNRVNDSIVSQVNKYSLSDRFGXHCLTDLALNRDFAVSYESQIGVMFAGWLKEKY
GKDVILPLKKSIWTMYLAFNIPKLSSIIFTKLEQLLHRVVSVGLIEQWTRDEVILWNNIFKIL
HWESVFAFSLWHLQGGFFFLLFGNLLALFVFVLELCLK
>BgerIr406
NIGPFLSTLILVSGGYGILVPPDLDTIEERLLGECILNIVETHFSKDLPLAIETPSMWYPAWK
RSQGDTLLEMLFTHAGITKFTLGKVEKIVHKSNTFIPGSFVILVPVIESEDDVLYMTMMFQ
RILVDSRNPRAKFIIIFMKAIKPLINEIIRALLMLCWFSNFSRLIVVTQIVENKMNQMPSFDI
FGFNPNEQMDICSMHIEKISHIDIWNSEENRFLLNATMFQLPEKLNLNRCALQIGVHHHP
PFVYVLNGIKGIGAKILYLFCERFNCRIELNSSEFHIRFPVKYGILSPFDRDTATYPYFRADF
KWFVPAGEEIPRWQSFFMVFNPYMWVFAFCSSASGIFTLWLMQKYKRQFSRGPGHTVLI
TGLLTHLGFGDSNNFSGFGPAMFYAIWLFYCLIIITAYQSGLLSFLVYPGQFPNIETEEELLS
SGLNMKRLINIINRINDSIILDINKYKLCKKLASHCMVDLALNRNFAVLYDSQIGGMISGWL
KEKYGREVIVSLEQALWTMHLAFHIPRLSGILLSNFESLLHRVVSAGLIEKWTRDEIFLWN
ALFKIPYCEPVVAFSLWHLQGAFYFFLLGNLLALVVFVVELCLK
>BgerIr407C
MFHIYFSYRNLGPFLSTLSAICGGYAMLLPPDLGTTEERLLGQSILNIVETHFSKDLPLAIET
PSMWYPAWKTSHGDTLLEMLFTHSGISHFTLGCIQNTDLPTNSPNKFMPGAVIILIPNLRS
QNDVKNMLTLFQRILVDSRNPRAKLIIIFMQTVKAILKQIVRALLMVGLFSNFSRLIVLTHI
VDLYGNKLNQMPSFYVFGFNPNEQKDICSMNIDKFSHIDTWNSEENRFFHNANMFQLPE
KLNLNRCRIHIAVHHHPPFVYVSDDIKGIGAQILLLFCERFNCRTKINASQFHIEFPVKYGIL
SPFDTDTATYPYFWTDFKWFVPAGEEIPRWQSFFRVFTPYMWAFAFCTSASGIFTLWLM
QKHKRKFSRGPGHTVLITGLLTHLGFGDSNNFSGCGPAMFYAIWLFYCLIINTAYQSGLLSF
LVYPGQFPNIETEEQLLLSGLNMKRLINIINRINDTHISY
>BgerIr408
MFHIFSYRYLGHFLSTLFVLCGGYAMLLPPDLGTTDERLLGECILNIVETHFSKDLPLAIET
PSMWYPAWNRSDGDTLLEMLFTHSGMSHFTLGYIENVDVPRNSPNAFMPGAFIILIPILR
SQNDVINMFKLFKRILVNSRNPRAKLIIIFMQTEKVLINAIVRGLLMLGLFSNFSRLIVMTQI
VDSYKNEINQTPSFDIFGFNPNEQMDICSMYIDKISYIGTWSSVENRFLLNANMFQLPEKL

NLSQCGIQVAALTYPPFAYILDGIRGVAAKFVLLFCERFNCRIELNSSYFHIEFPVKYDTEYD
NDAVTYPHFKDELKWFVPAGKEIPRWQSFFRVFTPQMWLFASCTFAFGTTTLWLMQKH
KRKFSGEGSGHTVLITALLTHLGFGDSNNFSGYGPSIFYAIWLFYCLTINTAYQSALLSFLVS
PGQFPNIETEEQLLASDLNMKRLVFFIDRVNGSLGSLVNKYEICEKLSLYCLIDLALNRDFA
VLYGVRVGGIISGWMKEKYRIEVIVPLKETFWDLHFAFHIPRLSSILFAEFEPLLHRVVSVG
LIEKWTRDEALIWNDLFKMLRSEPVFAFSLWHLQGGFYLLLFGNLLRCFCGGALCKIISMA
ASQKFPTLL
>BgerIr409P
NIGSFLSTLFVICGSYAMLQPPDLETTEEKLLGECILNIVEKHFSKDLPLAIETPSMWYPKW
NRLPGDILLEMLFTHAGITKFTLGKVEKIVHKPNTFIPGSFVILIPVIESDDDILLMTLMFQR
ILVDSRNPRAKLIIIFMQTEKEHINEIVRWLLLLGLFSNFSHLLVLTSHESKINQKPSFYVFG
FNPNEQKDICSMNIDKISYIDIWSSVENRFLLNATMFQLPRKLNLNQCGIQVAPRTYPPIAY
VLDGIKGVAAKFVSVFCERFNCRIELNSSYFHIEFPVDYDTEYLYDNDVVTYPHFKDELKW
FVPAGKEIPRWQSFFRVFTIQMWVFASCTSVCGTFTLWLMQKYQRQSSGGGSGHTVIITA
LLTHLGFGDSNDFSGYGPSIFYVIWLFYCLIINTAYQSSLLSFLVSPGQFPSIETEDQLVASGL
NMKIIVFFIDRVNGSLASLVNKYEICEKLSIYCFIDLALNRNFAVLYGAQGGZIISGWMKEKY
RRELMVPLKQTFWEMHFTFHIPRLSSIIFAEFEPLLHRVVSVGLIEKWTRDKVVIWNELFK
MLRWEQVFAFSLWHLQGGFYLLLFGNLLALVVFVVELYVK
>BgerIr410P
DARNPRAKLIIIFMQTEKGLINQIVRTILMVGLFSNFSRLIVLTQIVDLHRNRIKQMPSFDVF
AFNPNEQKXICSMHIDKFSHIDTWNSEENRFLLNATMWEIKLESIWNSNSSTPPSALCYVL
DGIKRIGAKILLFCERFNCRTKLNASHFHIQFPVKYGTLSPFDTDTATYPYFWTDFKWFVP
AGEEIPRWQSFFRVFNPYMWAFAFCTSASGIFTLWLMQTYKRQFSRGPGHTVLITGLLTH
VGFGDSKNFSEYGPAIFXAIWLFYCLIINTAYQSGLLSFLVSPGQFPSIETEEQLLYSGLKMK
RLVNIINRINDIHISYINKYEICEKLAFNCLKDLALNRDFAVLYDSQIRGMISGWMKEKYGR
DVIVPLKQALWTLYLAFHIPRLSGILFSKLEPLLARVVSAGLIEKWTREEIFVWNVLYXSFY
WKPVVAFLLWHLQGAFYFFLLGDLLALVVFGLELCFK
>BgerIr411
MKENNGNICTIIKREVECFILFSYRNLRPFLSTLFAICGGYGMLLPPDLDTTEERLLGWCIL
HIVETHFSKDLPLAIETPSMWYPAWNSSHGDTLLEMLFTHAGITHFTLGYIQNTDLPRNT
PNKFIPGSFIILIPILRSQNEVENMLTLFKRILVDSRNPRAKLIIIFMQTEREFTNQIVRTLLIV
GLFSNFSRLIVLTQIVDSHQNKIKRMPSFDVFGFNPNEQKDICSMNIDKISYIDTWSSVENR
FLLNATMFQLPGKLNLNQCGIQVAPHTYPPFVYVLDGIKEVAAKCVSLFCERFNCRIELNY
SHFHIEFPVKYETEYLYDIDGASYPHFTDELKWFVPAGEEIPRWQSFFRVFTPQMWVFAC
GTSACGTFTLWLIQKYKRQFSGEGPGHTVLITSLLTHLGFGDSNHFSGYGPSTFFAIWLFYC
LIINTAYQSALLSFLVSPGQYPGIETEEQLLASGLNMKRLFVIKRVNDSVILSNINKYELCEK
LSLYCFTDMALHRDFAVLYGARIGGMISGWMKEKYRREVIVQLKQTFWDLHLAFHIPRLS
SILFAEFEPLVHRVVSVGLIEKWTRDEVILWNDLYKILRLEPVFAFSLWHLQGGFFFLLFG
NLLALVVFVVELYVK
>BgerIr412N
NIGPFLSTLLLISGGYGMLVPPDLDTTEERLLGECVLNIVETHISKGLPMAIETPSMWYPA
WKSSHGDTLLEMLFTHAGITKFTLGKVEKIVHKPNAFIPGTFVIFIPVIESEDDVLHMIMM
FQRILVDSRNPRAKVIIIFMKAVKPITNVIVRAFLMVGLVSNFSRLIVLTQIVDSHQNKIKRM
PSFDVFGFNPNEQKDICSMNIDKISYIDTWSSVENRFLLNATMFQLPEKLNLNQCGIQFAP
HTYPPFVYVLDGIKGVVAKFVSLFCDKFNCRIELNSSHFHIEFPVKYDTENLYDIDGASYPH
FRDELKWFVPAGEEIPRWQSFFRVFTPQMWVFACGTSACGTFTLWLMQKHRRQFSGEG

TGHTVLITSLLTHLGFGDSNHFSGYGLSTFFAIWLFYCLIINTAYQSALLSFLVSPGQYPSIET
EEQLLASGLNMKRQVFFIDRVNGSLESLVNKYEICEKYSLYCFIDLALNRDFAVLYGAQNG
GIISRWLKERYRREVMVPLTQTFWDLHLVFHIPRLSSIIFAEFESLLHKVESAGLIEKWTRD
EVVIWIEFFKILRWEPVFAFSLWHLQGGFYLLLLGNLLAVSVFVVELYVK
>BgerIr413P
KILLSYRDLETVLSTLFIVSASYGKLLPPDLDTTEERLLGECVVNIVETHFSKVLPLAIETLS
MWYPAWNRSDGDTLLEMLXTHSGITHFTLGYIENTDVPTNSPNKFMPGAFIILIPIIRSQN
DVKNMFSSLKRILLDSRNPRAKIIIVVLESEKVFRNKFVRALMVIELFSNFSRFIVMSLNFDS
HKNKINQMLSFDIFGFNPNEQQDNCSMDIDKISYIDTWNSDQNRFLHNTTMFQLAGKLN
LKQCRIMVSFNHHPPFIYVLNGFKGLTANFLWLFCERLNCRIKMNETQHTHVEFPVNYGS
EFYFDNNVATYPHFKTFFRVFNPHMWVFAFCTSASEIFTLWLMQKYKRQFSRGPGHTVL
ITGLLTHLGFGDSNNFSGCSPAMFYAIWLFYCLIINTAYQSGLSSFLVSPGQFPNIETEEQLL
YSGLNMKRLIFIKRLNYFVEPSHINXEMCDEVAIKCLSDLALNRDFAVLZDSQLGRLFSGW
MKKKYGREVIVPLKQTFLTLYLTFHIPRLSSILFTKFEELLYRVVSVGLIEKWTRDEVVLW
NDVFKILRWEPVFAFSLWHLQGGFYLFLLGDFLALLVFILELCLK
>BgerIr414N
IVQFSFCIAMFCCIYMFFFWCEVTAMIPYPTLNSVERRLSECILNISETYFDRNLPVAIQTPG
MWHPRHQWRGSHGETLLKMMSSLNGNPKLTLGFGGDEDTIVKPGSHIFLVPNLNKAGD
RQMAVSMLQKTEDHAINPMGHVLIGTTTRIKSENLRALLSFALHRKLFRVVVCIRRKSPID
GFDIFSWLPNEQSNICSMNIDKLLYMDSWISEEGRFLHGSTLFPSESTLNLNNCKINVSPGF
VFPLVDQFEGAGGITHIQGVAVDCVYSFCKKVNCTTIWNRNKKTHIQFPVSYGKRLTRGGC
ELTYPHFRKDFSWFVPSASEIPHWQIIFRVFNMQVWILVIVVFVAGTLTLCLTQRRFADSK
TGHSVLLTALLTHLGAGDGNKQSGFLSTHFFAIWLFYCLIINTAYNTALFRLMVYPGKLPQ
ITTLEQLKKSGLEMVRMVQINGDWDEVLEYKECMSENCLDKVIRGESVAVLFPTDLGKRY
TSRFRDHRGRPTVVILEQRFGTLLCTFRVESLSCVLHSHLETIIHRVVSSGLVDRWTRQQF
QWRPIADDTDESSLRFAFSLFHLQSAFYLLAVGLCLALLSFFAEILTVRCGLHC
>BgerIR415N
TARCSFCFEVFLICWIGAEAILLHSTNDSPEGSLSQCILNISETYFRKDLPIAIQTPAMWYPR
FEWRGSDGETLLKIMSTQNMIPKLMLGETSDINLIMGNVMIPGSFIFIIPDLNNFEDRFMYI
SMFLRTRKDSRNPTAHVVIGIMEGTERVSDLLDMLFIAFQVRLLRIVVIVKQYNSKNNSFV
GFDVFSWLPDEQNDICSEKVDNLKYMDTWISEEDRFLLGSNLFPQQISTNMRNCKVNIAP
HWQYPFIYGDKRNIDDTSQLNGVALTCLEILCEYLNCQITWNKKLKSVIKFPVRFNPEYAL
DSCESTYPYFTSNIAWFVPAGTEIDRWRSIIRVFSKQMWALIILTFLSGIVVLWLVDVPPEP
SNLSHLERVNRALLSAILTHIGGSETSWRRGYLSKYFFAIWLFYCLIINTAYISGLFILMVHP
GQIPPLKTIEELNKSGLELQRSIYGEALNWSNIKLCLEVRMDSLGCFGKIAYLRNGALLHSD
DIGKVMSKQFRDQFGKDRIVRLEETFATWYLGFNIERLSCILQRPLEVLLRRAISGGLIDKL
TRDESSLLSSFLPKKDVQRTFAFSLQYLVGAFYLLLVGNCLATIVFIIEFHFVRCK
>BgerIr416N
TARRSFCFEVFLTCWIGAEAILLPSTNDSPEDSLSQCILNISETYFRKDLPIAIQTPAMWYPR
FEWRGSDGETLLKIMSTQYMIPKLMLGETSDINLIMGNVMIPGSFIFIIPDLNNFDDRFMYI
SMFLRTRKDSRNPTAHVVIGIMEGTERVSDLLDMLFIAFQVRLLRIVVIVKQYNSKNNSFV
GFDVFSWLPDEQNDICSEKVDNLKYMDTWISEADRFLLGSNLFPQQISTNMKNCKVNIAP
HWQYPFIYNYRDVSNIDVPSRPKGVAFKCLEILCEYLNCQITWDKTLNSVIKFPLRVNPDY
SLDSCESTYPYFTSNIVWFVPAGAEIDRWRSLFRVFSKEMWALIILTFLSGIVVLWLVDVPP
EPSKVSHLERVNRALLSAILTHIGASETSRRRGYLSKYFFAIWLFYCLIINTVYNSGLFILMV
HPGQIPPLKTIEELNKSGLELQRSIYGDSSFNWQGIELCDESGIQTSKCFGKIAYLRNGALLH

SDDIGKMCMQFRDKFGKDLIVRLEETFATWYLGFNIERLSCILQRPLEVLLRRAISGGLIDK
FTGDNSLESIFSSQNDVLNTFAFSLQYLVGAFYLLLIGNCLAIIVFIIEKINFVRCTSSK
>BgerIr417P
MIAYHLFSLLSTSFVGGYRLLLPPQTDVVLEKHLSECIINISSIYFDKDLPLVIQTPGTWIQIL
GRKDTTGDTVLEILSGKIDTSQLILGYDSDIDGFYSNVVKPGAFLLIFSDLEKEQSRRTAISM
LKRVNKDSRNPKARVVIVIMKCFNSLAEKHFNLNGMLSYALLCGLVWSVVILPNAGLCQL
DPKEKPGFDILNWTPNEQADICTVGGPVNKVRYIDTWEITQKKCSLGTKLFQEVGKLNLR
KCKLNLAHSRIYPLIYLRHKTPAGVIIKILKLFCEEYNCRLTTDVRKPPLIRFPIIHLEGFSHD
SCKVTYPHFRKDYTWFVPAGAEFPRWQSIIRVFTPKIWTLVLVIFLLGSFCLWLIDTLQHK
CRNVILTALLTHLGMSHSNSYHGFVAVTFFSLWLFYCFFIYTIYQSDLFGLIVNPGQLPAMK
TEDELHNSSLIKKNSDNSXWSQVNNYEFCPYSLGVCIHEVATSQSVALLYEDELGKRISRM
YKDQWGKETVVPLVEHMASAYMSFRLERLSGVLHDPLEALLGRVVDVGLIEKWVNDEQ
WKWNMDYMKFLSKKISPFSLWHLQGAFFLLVIFLSLAVIVFFVEIGVNIFR
>BgerIr418P
MRLKCVVLLLSFWGSCESRLTLPEDRSLEPYLAQCMIEIMSKYFDKHMPMVVETPGTFLS
AENDNYHSGDKFIEMIQNQVQCSLLIIGNREFGQIAKWEQLSPRSYIFLLPPLNTNKDTEFL
RLMINRFLGHSYNFYYRPKLVLILTAEYNNPRIREIILLWCQRFFHVATGIKDTIVLQPDILS
GDGSINNVSGYSWTVNDQTNYCSAIDKVRHVDTWISDRGTFLLDEILFLQDEKLNLKGCAI
IVIYATILKXESHIFTYPHISMDVTWFVPAGREIPPWZSLFRAFSPILWTLVGLTFVFGSFTI
WLLQKSVRSSTDTFVDVFISALSSHLGIGVSDRYKSFVAVVFXMLWLFYCLVINAAYQSAFY
GLLVDPGHFPAIRTVAELEESGLVIERLLYFQISNSTMLSRLLLSFYEHNLCYNFTQCLGNG
VDKRKRAVLTPYKYKHYSLLQFTETNEHVPLAEVVGTFFESVCKLHEHYSVWLKALNTAI
ERFVTSGLVNKYSNELEYSIKLYSVHEETQPVFSFSLFHLQGAFYLLLCGLSLSALVFVTZFL
VYFYRRLIQIWAIRAYLQNVQGAHVKHTPLY
>BgerIr419P
EIIGETRNMYLKWVVZLLSFWGSCESRLTLPEDRSLEPYLAQCVIEIMSKYFDENMPMVVE
TPEAFLSHENQDYDSGDKFIEMIQNQVQCSLLIIGNREVSTIEPDKHNTPGFHILLLPPGNT
KQDKDFIMLMIKIFAKRYQTSYHGTKLVLILTAENETTQIRGEIPLFLQKYFHNNIGIRDTIV
VQPDILTEGRSINNISVYSWAVNEQTNFCNDLDKVKHVDTWISDRGTFLLDEILFLHDEKL
NLKGFTINVCSVSFAPYSGLCNDEVCMVLETVFNVVSKSLNDTVILSRNYNSTIKYAIMFPC
IYHQFMDHNLFTYPRISLDVTWFEPAGSEIPRWQSLCRAFSPIVWSLVALTFAFGSFTIWL
LQKSVRSSTDSFVDVLISALSSHLGLGVADRYKGFVTVVFFILWLFYCLVINAAYQSAFYKL
LVYPGHFPAIKTVGELEESGLVMERMLNFEFTDSIMLSRFWLSILEYNACYNFTQCFGNGA
GNRKHAVFTTYNIYKYYSPCLTNKNTHVPLDEVGGTLLLGVCK
>BgerIr420P
QLNFWGSCESRLTLPEDRSLEPCLAQCVIEIMSKYFNKNMPIVVETPETFLSSENDNYDSG
DKFIEMIQNQVQCSLHIIGNRELGQIAKGEQHSPGSYKFLLPPQNTKQDIVFTGSMFQMFL
KRRYTFYDDAKLAENHIVWFREKILAILQGYFSDSTGIRDTIVLQPDILSGDGSINNISVYSW
TVNEQTNFCSXLHKVRHVDTWISDRGTFLLDEILFLHDEKLNLKGFTINVCSVSFAPYSGL
CNDEVCMVLETVFNVVSKSLNDTVILSRNYNSTIKYAIMFPCIYHQFMDHNLFTYPRISLD
VTWFEPAGSEIPRWQSLCRAFSPIVWSLVALTFAFGSFTIWLLQKSVRSSTDSFVDVLISAL
SSHLGMGVSDRYKSFVAVIFFILWLFYCLVINAAYQSAFYKLLVYPGHFPAIKTVAELEESG
LIMGRLLYFQISNSAMLSRFLLSFYEYNFCYNFTQCFGNGAGNRKHAVFTTYNIYKYYSPCL
TNKNTHVPLDEVGGTLLLGVCKRNASYTLWLKALDTVLVRFINSGLFNFLINKVEFYNKL
HCVHDNAEPVFSFSLFHLQGAFYLLLFGLSLSAVVFLTEALVYVFSRLIQIWSIRAYLQNVH
>BgerIr421

MIGKTRNMHLKCVVLLLSFWGSCESRLTLSEDKSLEPYLAQCVIEIMSKYFDKNMPMVVE
TPGTFLSHENQNYDSGDKFIEMIQNQVQCSLLIIGNREFGQIAKWEQLSPGSYIFLLPPLNT
DKDIEFLTLMKKRFILYNYNFYYRAKLVSILTAEYNNSRISEILWCQRFFHVATGIRDTIVL
QPDILSRDVSINNISVYSWTVNEQANYCNDLDKVSHVDTWISERRTFLRDETLFLQDVKV
NLKGCAIKACIIPDAPYSGVCNDELCTPIETVINAVSKSLNATFILRRDIAGKRQCGILFPCFY
DSFQESDLSTYPHISNDLTWFVPAGSEIPRWQSLFRAFSPILWSLVALTFAFGSFTIWLLQK
SVRSSTDSFVDVLISALSSHLGIGVADRYKGFVAVVFFILWLFYCLVINAAYQSAFYGLLVDP
GHFPAIRTVAELEESGLVMERLFDFSTINRTILNTFWLSILKYDYCHSVSQCFGNVGDRLER
AVLTSYDMFKYLSPCYREYMNRHVPLSEVVTTFSSVMCLNYWYYXMWLKALDTVLERFI
TSGIFNFMINKFEFYSKLHCVQDEAEPVFSFSLFHLQGAFYLLLCGISLSSVVFVIEVLVHFF
RRLFQIWAIAVYLQIVH
>BgerIr422P
MHVKWVVLLLSFWGSCESRLTLPEEKSLEPYLAQCVIEIMSKYFDENMPMVVEAPGTFLS
HENQDYDSGDKFIEMIQNQVQCSLLMIGEREIGTIEHEELHKPGSYIFLIPPGNTNQIIKFLN
LVFKVFFGRNYNYYHAAKLVLILTAENNNSLIREENPLFLLRIFIQSGIRDIIVLQPDILTDYR
SINNISVFSWNVNEQTNFSNRLDKTKHVDTWISERETFLRDETLFLQDAKLNLRGCEINT
CIKSSIPYIDFCNDEVCTPMESVFNATSKSLNVTFLLSRDINRTDKCAINFPCIYYPLLSFHEF
NYPHISDDLTWFVPAGSEIHRWQSLFRPFSLILWILVTLTFAFGSFTIWLLQKSAKSSTTDS
FMDVLISALSSHLGIGVADGYKGFVAVVFFILWLFYCLVINAAYQSAFYGLLVDPGRFPAIK
TVEELEESGLIMERMVDVDFTGPFTSMVDVEFTDPTIPFTIWRXCYNFTQCFGNVADKPN
RAVLTTPNKYKYHSSCYTDIHKHVPLGEVIHTDLYSLCLNDGYYTLWLIALDTVLVRFITSG
LFNFLINKVELYNKLRCVPDEPEPVFSFSLFHLQGAFYLLLCGLSLSTIVFVTEVFVYFFRRL
IQIWAIRAYLQNAH
>BgerIr423
MCVGLITKVVPLVCCFFVIGGDTFLFTDSEISLEQDFAECILSISQTYLDSSLPLAVQTPGSW
DSHFKFKNLYGDIFLKTLSDRGNKSQYILGYTGNFKTRLKFDSFIILLPPLKPVIFSELLGSMI
MRISMDLGREKTKGIIIIMEHFDSEEFITIFLQSLFSFALKFTVVQIIAVVPQANPVMTSARRI
DVFSWLASVQTNICSRKVDNVRYIDTWNSEENSFIYGSNLFPSPSVSDMRGCRVNLFQHT
NYPLIYNDEQGLRGVGFQFLSMFCKRFNCLIINNWDKSRNDDVRFPVIYGRDEIYLGYLTY
PYFRVHYSWFVPAGSARPRWQSIVRVFTPLMWTFSVVACILGSCTFWLIQKCRMISVDGD
RSIKHSGLITLLTHLVLSNTINCRDPLAVTFFTAWLLYCMIINTAYQAGLFELIVYPGQFPAI
KNLEELEKSGLHKKSGIQMHNADEYWQKFNEYENCTKLYMSLCLEDVEKYGNFAYLYDQ
EYGKMFASLHKDERGKDRVLSLKEVFAIGYQGFYISNFFHILGTTPDALLHRTVSAGLIDK
WARDELHVFHMQYPRTEAETVFPLSLIHLQSPFYLLVVGLMMSLLVCLIEMSMHNLHTV
AGS
>BgerIr424
MKMNTIFLILLYFFFGSSMNLEHSNSVEYLEQELSECILSIINKYFDPNLPMVIQTSGAWDK
EQSINFSPGNQLLKILSEQNNITHIILGHTSHEERSKMNRSAVKPGSYIYVVSSLNTEYEAMI
LNSMLSVRYLNIWNSVAYCIIVVVKSFSTEQEQKRLLPYFVTNAVFIGLSRIILISHVKSVKS
VNEFIPNFDVITWQRNNEDPFCSQGWYEIRYLDTWISEERRFSLKSELFPIQRHMNNCVM
RLSLYRHFPLVYNFRGGTHGVVINFLIKFCDLYKCRMEVKSPADMFFPMVYQEPASLYGID
ITYPHLWKHLTWFVPAGKMVSRWMTIFRAFTSLMWLCVALTFISGTCVFWIIEKAKYWN
NNNNSVSAGPNILITVLRTHLCVGDTCNYEGSEAAVFFSLWLFYCIIINTAYQAGLFKLMV
NPGCLPPIQTEDQLRSSGLDMLTYIVVKGNDDSYWSKVTDYDPCTDLYKCYEIIADRKTLA
ILLDLEYGNIYKDFFKTELGAYKVIPLKEIVGTMFFGFQINTNIDSILKSRLNRLLARAKNTG
LIQKWTSDILRLRSRKYFKKEVKEVIVFSLEHSQSAFWLILVGLLFAIFAFIVEILFYSWL

>BgerIr425P
MNMFVTVLVMLCSFYASVTSLTHSNTSDYLEKTLSECVLSIINRYFDPNLAMVIQSSGSWD
KNHRINFSPGNKLLNILSDETNITLVNLGPASPYDEAMLNSAIMPGSYIFVVSSLNTEYEAK
MFNDMLYRMSLRIWNSEAYCIIVVVKSFFSVLEQNRLLPYFVSNALYVGLTRIIIISHVRTE
KSVTKLLPNFDVYSWKRYEEDRFCSQALYKIRYIDTWISEENRFLLESDLFPLQRHMDNC
VIKLLLYRHFPLIYNRSNGKPDGVIISFLRTFCEVYKCRLELKSSSPDLIFPMAYDELSLLSGN
DLTYPYIWKHYTWFVPAGKIVSRWKTIVRAFTSLMWLFVAITFISGICVFWIIERAKYWD
DNNSVRAGHNILITVLRTHLCVADTCNYEGIEAAVFFILWLFYCLIINTAYQAGLFKLMVNP
GCMPSIETEDQLKTSGLNMFTYVVITNNEESYWSKVTGYDKGSCLNVYNCFEVMADFGTF
ALLSDLDSGTIYMDYFMNELGGYKXILGTMYFGFQVDKNVDSSLKSRLNKLLIRAVENGLI
EIWTSDILFKERKGYFEVDVNKIVVFSLSHLQSAFWIVLIGILFGIFIFILETFFHLWLHV
>BgerIr426P
MEPMGLWRFTVALTWNVFYLNIMNRYFDLNLPIALQISGTRDFSSRINYSPGDTLLKTLIE
SSNFTHVNFGPKSQEDXEFTMITREIKSGSYIIVISSLNTKYEALILNNMLSRMYQYIWNSV
AYYIIVVVEAFLTEKVQEIILSYYLTNIVNIGLARIIIVSHARYVNYEKEVLPNFDIFSWQRNR
EERLCTQTWYEIRYIDTWLSKEKRFSLESDLFPLQEHMNNCVMKVSLHTLYPFVFNFQER
SYGIIVNLLVVFCELFKCRIEVDSSADIIFPILYEEDNTLYGIDVTYPHLWKHITWFVPSGKL
VSRWMTIFRAYSSLMWLCVAITFINGTCVFWIIEKAKYWDNNNSVRAGHNILITMLRTHL
CNGDTSHYESSEAAVFFILWLFYCIIINTAYQAGLFKLMVNPGCMPSIETEDQLRSSGLNMS
TYMVTNGEKNSFWSIIQIYDTCLDLFKCFEIVAEFATSAILLDSDSGKIFIDIFKNEFGENKV
APLRENVGTMYFGLEVRNNLDSALKSRLNILHDRAVNSGLIMKWKRDSSRFARREHFDS
GLKXSFCFSFNHLQSAFWLILIGLLSSILVFVVEILWFSFEDTH
>BgerIr427
MNIFFSRLIQFIVLTSITVHHLKNCSAIFQSYIDVSTLEQHLTKCITNISMTYFSNHLPLIVQT
PGTWHSTLDYQILHDRKNVHGDVLLQELNRQNHIPQIVFGFVHSIVKESRNIMKPDSYIIL
VTGTDFTQESKFISNMLFRVLKSTRNPYAKVILATTRLPGIFEHKKNISQLYLQFALYLNFY
NAIFLVPSAAKEKQIYNFDVFSWVQSEQKNICMKTLDNIKYLDTWVSKENKFLLNKYLFP
PQTAPGMKECKVRMKYGIMYPFIYMENGKLKGTFLKFIDIFSKVSNFSISNVKVEEHAHFP
ELYKSKFAQDECQYIYPNFFKVLRWYVPAGKKVPYWQSLIRAFSSTLWILVIFTFVLGSLTL
SLFQKFHSQSYSDNSIIRTCIILALMHIGVFIPIRYNSATAVFIFSVWMFYCLLIYTAYLSSLAG
LMVNPGHLSPVRTEKELKESGITMKTLFRFSGVGFGNTFSNYEFCGKDHPRVYFKEIVDTQ
SLAILSDTELAKWSIEYFDDRGKPKVIPLEDIAGTLYLSVQINDQLSCMLHKPMETILNRAV
SVGLMEKWNNDEVFKQKMAEAKLHTEQHILAFSLSHLQGAFYIIAIGLMMSIFVFIIEILVQ
AFVIKFP
>BgerIr428N
NPSLKGCFCIILLLPQSGFSGFLELDPLVSLEQTLSECILNISKTYFNKNLPLAIQTPSTWGKA
PLHVIPSHSNIKFGENLLQNLSNQNYFALVTLGYTQNFNSHENNTMKPGSYILILPGEDNK
KDMMVEDNMLWRIAKDSRNPRALTIIASMKPHSSLYLQMFIGKYFLKLAFAANLQAETIV
LLPKKENLQNSKVNTYDIDVFTWLPDEQSNFCSSEIDVIKHLDRWIGKEKRFESNENLFPP
KQITNMKRCYIRVTAVPVFPYVFSNRGSINGILMHLLDAASFKSKFKISIVDMKDMKKEKP
HLLMPNYASRVYLENECRFTYPIFEQDLTWFVPSGDKIPRYQSIIRVFSPIMWMFVTPTYII
GTLLFFFMSKFPNHFDRKQNKFNISTSLMNTLSTHLGLSVEYRCKGSLATSVFSLWLFYCF
LVNVAYQSALIGFLANPGEFPPIKTLQELDESDLVKERKVTLLSSKDSYLNNYNQYPLCGKE
PDCFTRIAKQRDVAILYDRLSGEMDIHSTYTQNGKPEIVPLTESLETFYLGILIIKLGCVIHSR
LSTIQSRLVRAGILQKILHLIKEQTSRKYFIFNESKVFVLTLSHLQGGFYLLILGILFSLILFLFE
ITHFVLK

>BgerIr429
MLFQQENSAVLLPPTIDYSIEQQALTCIFQIINDYYKTDLPLVIQTPSTWGKYSKSLTPLNT
KIHFGDILLGMLNSDRYVSQIVLGYVHDFKIRQLRNAFKPGFHIMLLSGANYVEELRMGFC
MMYRLHKNSRNSASKALIISTLPMESSEIRRKAAITIITMLWSICSQAKVIVLIPIKIKQIYDN
TLYNFDIYSWFPEEHLNLCSGKVSDVKLVNRWISKENKFNSNIELFPKEEVKDMKGCILSA
TMYEMYPFFYRDENKKLQGALLKLLQIAADKSNFKIKLVTTDEHRHITLPNIITQQAMTD
ECMYLYPHFKDQLAWFIPSGELIPRWQSLIKVPFNSFMWIMVISTFLTSSLMFWLLSKFPK
YLKKNCIEMNLVLTIMNLFLTQLAMGITNRFRGLIPMTLFILWLFYCLQINALYQSALIGFL
ANPGEIPPLRSIKELNESTLKKMSIIKFKAFKGNYLLKYNIYGHCEPATECFSRVAQHRDMA
LLSFKAVGELSMREHFTEYGRPQFLALVEYVDTIYAGIVITQLGCVLYNRMEDIQSRLVSAG
FVNSLVNDEVEKSLRTYFKSVGKEGAFVLTLSHLQGAFYISIIGIILSLIVFTSEIIYHKLNTVT
EL
>BgerIr430
MILRFLVNLLPVFHVTVSGLGHNILNTIDVEPPLLKSLAECVVKIATNYFRTDLPITILTPSK
ELGTILDILNVDMVLRSLNKQFSHSLVNLGSFVGKSAPKRLTNSVKPGSYIILPSGADDEAV
TSLVLRMVGRILVDARNPSARLLIVLMNSSKSKQEQTSKAGRFLQMMWLNLNIPNAVVVI
PYFSSEYSGNNHIYDFQIFNWNIHQQQDPCFRVLSHVNELDNWIWKDRRFVMDADLFPV
KSITDMHGCILISILNVFFPYAFYVNFKQIRGALIQSLAMIEHALNVKIIYIEKTKKIPDFITPF
LYTGVHGADECTVTYPHHIENIKLYVPAGSYVPRWKSLINIFTPLLWIFVVVTFVLGSFVF
WLLLKNLNGNANYILVLMQILLTYLAVGITYRYRGTVATSLFIIWLFYCLLINAAYQSALIGF
LANPGQYPPIRNVQELLDSNMELHSGFNVTSSAEEIASRFGEIKTCPSLYDCAWKITENRST
ALLMNDFLGDILVRGTFSSDRKPKVVALDEHFFNCYLGVRLYRYGCLLHNRVDMLMNRL
FSAGLTRKVLEDLTSLQKMHFNVLRMGSNFKLSLNHLQGAFYLFGFGMSFGLVLFTVEYIA
NQILNEIQAQKKD
>BgerIr431
MLQISKHLLLVMSFYHMSHCLMKVQHFENKLLESSTECIIRIAKTYFRADLPIMLMIPYEET
EESSKNDTNYVYDDFIIGKLNLHLNHSFLILDYIEKEEHVIRSPNKVQPGSYVLILKGSAYKS
FAMAIKMVKRILIDARNPSANLLVVSINAPKSRQDQLSIAGNLLKLFWKRLKISDAIAMVPS
IPISSNAYGQSRGFEIFNWFPKKQRGPCFRTINHITKLDHWALGDQDFKSNNNLFPVKHIK
DMNGCNLDILLKTVPPFVTFFKTKQLWGTIPEAINTIKDVLNFSINYVRNIDYTPDISAPILY
SAFSTQDNCSMTYAHHTENIKWFVPAGAPFPKWKSLTRIFSPLMWTCVVVTFISGSLTFW
LLFNATRRYTQPQTFCTVLMNTLLTYLGVGIRDKSKGPLSISFFILWLFYCMLINTAYQSSL
IGFLANPGEYPPIRSIEELLRSDLSLLSTFVFSDSTKEGLELNSYEKCPVIHTCAEKMVKYQD
TALLVSEFKGSLIIRNAISYGRKSLIVPIDENVHNLIFGIKINTFGCMLRDRVDDLMHRLFTA
GITNKARHDLMNLQKLVYTRLSQDPFKFTLSDLQGAFYLIALGIIVAFTVFIFEMIINIIITAF
V
>BgerIr432
MLNQFEIVFQVLMLYSLKTTHSTIQTLSITSTIVEDLSECIVDIAQKYFSKNLSTAVLIPTEVF
GTSSNDCMDNSDGNKIVLSLNENIVNSLLLLGDQSKPKHLSYDVKPGSFIIIVSGDIVHALR
KLMILLAQTKLPSARLLIASTIVPISVRQQTAIAKLFLGAVWDMLQMCNAIAIIPRKHQSKS
VKNNYDGFDIFNWFPGKQSDPCFKNLDKIDLLVSWNLQMKRLENNKHLFPNKQITILPGC
HLSALTLKHQLSNILSFEDMSYSLHILESVLKTKIRYIPIGKSEVLSDLKFPVARGVNISTAVE
ECSLTYPHFTENIKWFVPAGVPIPRWKSLTKIFNPLMWTCVVVAFVLGVVTSWYILKYSH
TSQQPVDMVSVIINTLLTYLAVGISDRYKGTVGTSFFVVWLFYCLIINTAYQSALIRFLADPG
EYSPIKSVQELYESDLNLITTVKFNHAVQNEIQKLNSFKHCVSHEVCMKLISEARNTAILMI

EFVGTSSIRASYSSETKKPGIVPIEENVYKIYSSIEIYTYGCLLYERLEELMHLLFSAGLTAKIT
RETLRVEKMLYSDLLNEDWTFQLKLSHLQGAFYIYVIGFLVSIIAFFLERFVHN
>BgerIr433
MNLFKYQGSIIKILIIFSIQEYDTKILQPVPVSPILKYLSDCIVNVVDKYFTRDLPIAVLVPRNT
ASIPPNNDNILGDYLINTLNKRIMNTFLILNQDNNARIMNNWMKPGSYILLISGENNLFFN
MAYHMLRTIFETTKTYASARVLVAITDIPRNQTQQVDTAREILEMIWKGLIIYDSAAIVPN
PVSELPTKRDFDGFGVFNWNPLKQRNSCLKYLSHIEYVDNWQSNIFIKNANLYPQTDLTN
MHGCKLNIRMQEHVPYAFQLIPGIIWGVFPIAIEILKSKLNFKVSYTFEATDIRPELLFPVTY
TSGNVSTHEECLMTYPHTQENIKWFVPAGAPIPRWKSLTKIFNPFLWFSVMVLYIAGSITA
WVLLESTQKEKQPKAFSVVLMDTLLTYLAMGISDKYKGTLSTAFFVIWLFYCLIINTAYQS
ALISFLADPGEYPPIRSNQELHDSDLELVSRLDFLGILDTDLLYLKHYQHRYTYEELLQRISK
KNNVAFLTNEVDGTMILLKSFSSETNKPRMTPIHENVHRVYVSMYVNTFGCLLFDRIETL
MNRLMSGGIFDNFLEKLHTGVFIKFSELENDDVFRLTLSHLQGSFYLLCVGLSMSCVTIVTE
IMLSVFIK
>BgerIr434
MGLAVNLLVVLKMVSFAIGHGQARLIAFDKVSRITESLTQSIIDIAEKYFARDAPIGVLIPSK
AAPHAWAIHREGEFLLEELNKQMIHPLISLDYHKKSQFKAKYTKPGSYILLISGESDSISTM
TFNMLSTIFETANTNTSARLLIALTTVPKTQSHQISAARTLLQIVWKALDISDVVAIVPNPP
SRSGVKHSVIAFGVFNWIPIQQRDPCFKVLNKIEHADSWLIKTKCFLNNVDLYPNKYVTD
MRGCILKAKIQEYTPYVARLDDSLLGVIPTAIGLIKQLLNLRITYSFVEVDPDLVFAVPYLFD
MSVIAHDSALIYPHLQDNFKWFVPAGAPIPRWKSLTKIFNPLMWFCVLVVFISGSLTTHLL
KNHSNKDNQNKRNYSSALLDTLLTYLAMGVSDRYKGIIPGTFFLFWLFYCLNINTAYQSAL
ISFLADPGEYPPIRTIAELYKSELELLSMIEFSGPIDQGLIELEKYNRCSSFIYCIVNILKYRNIA
VLTNEFQGRRIVLDTFSAESNKPAVLAINEVIQKVLIGMYINTHGSLLYKSIEVLMHRFITFG
FIGNYLDTYRDLENTFFSELMNEKPSVLTLSHLQGAFYLLGCGIFTSILTFLLEIKSYFFKRK
TKLMQYMSCLWNNSCM
>BgerIr435
MRHAVTLLVVSTMILFSIGYSNLISFDTVSTMTEDLAECITQIAEKYFAKDAPVGVLISNKA
DFNYWTIEREGEFLLEELNKQMILPLIILDYHKKSQFKAKYTKPGSYILFISGESDSISTMTF
RMLSTIFETANTNTSAKLLIALKTVPKTQSHQISEARTLLQLVWKELEIPDVVAIVANPPSK
LGINYSVIAFGVFNWIPIQQRDPCFKVLNKIKVVDLWLIERKCFLNNVDLYPNKYVTDMRN
CILNAKVQEYRPYVFFEDDSLWGSIHSAVKLITEILNFKIIWSNVAEYADLVFPVPYLFDMS
VIPHDSSLIYPHFQDNMKWFVPAGAPIPRWKSLTKIFNPLMWFCVLVVFISGSLTTYLLKN
HSNKGNQNKINYSSALLDTLLTYLAMGITDRYKGIIPGTFFLFWLFYCLIINTAYQSALISFL
TDPGEYPPIRTIAELHNSELKLLSLFEISGSINQGLIELQKYNRCSSIDYCMVNIMKYRNIAVL
ANQLGGSSIGLDTFSIERNKPAVIPIDEVIRKVLIGIYINTHGCLLYKSIEMLMHRFISFGFIGN
YLDNYQYVENIFYSELMNDNPSVLTLSHLQGAFYLLGCGIFTSIVAFLLEIKLYFLKRKTKL
MQ
>BgerIr436
MPFQKWMILVLLAMHPVRQTQTTLRVFDKWPSVEENLSQCLVAIAGKYFAKDLPLTVLT
SKEVLSIDSNSYIKHGNGNILIKVLIDTLKIPVESIDYQNKNLTTRNKPGAFIIMISGEDISFIL
MEFYKMYYSLAGVISRHSIVVMAVDSTGPLDSPDEEKDLAERLLRIMWDGIKITDAIVVIPS
LESSSNTKYKLNTFNVFNWDPKKQRDSCFKSLNRIEIFDTWIVDNKTFAKNANLFPNKYK
TDLHGCKLRGLIYTYPHYAYYIESPKGAIFESLRIIGNKTNFSMGYNFERINSMPDLVFPVIY
ETDKNAFPPQCSLTYPHHTDRIRWYVPSGAPIPRWKSLALVFSTFMWIFVAITFILGSLAY
WLLLSRSNKSVKFGSVFFDILSTYVEVSIPDKHKGTVAKSFFITWLFYCMIITSVYRSGLIGF

LADPGEYEPINSLEQLNRSGLKMFSAMHVFQVKDKELKDLTMYKLCESLSICRKMAIENQ
DTAILMGELQGIIFMRQSYNSEKKKNRMVPIDATVYQAYLGIEIIAHGCLLHKRVEELMHR
LFSAGLSRRVLDNEIRNERILYSELINDDPFVLTLQHLQGAFYLILIGILLALIVFSSEILIFYSK
S
>BgerIr437N
RIIKTDLLDTWYVDGKKFRNWSNLFPIKRLIELPKCILFAGLQYYPPYVSCYGKCVGSLYHA
GVIIGNFLKLVFIYNRKNTVPEVFIPYTYVNYNGSAMGDCLSTYPHFQDNIKWLVPSGAPIP
RWKSLTKVFNPFLWFSVICMYAMGSVTSWLLFNYSQSKRQNKRNFSTVILDVLLTHLAVG
ISDNYEGRISKMFFIMWLFYCLIINTAYQSALISFLIDPGEYPSIQTNQEVYDSGLKLFSNIEF
VGKIYESLKYINELEPCDDITEFIKQFRSKRIGALLSNEFSCNYVRTSTFSQQSNKPQMETID
ETLHRFYVGFYINKFGCLLYNRFEELMNRLVPTGILNHHIQILLYKQSRIYSEMFNENPFEL
SIIHLQSSLYLYCLGMCLSICLFFAELMYNKIYIKVFSD
>BgerIr438P
MLEIPWNMLYILVMSSFQMCYTKHHSLEVLSPIVESQSECIVNIAEKYFDKQQPIAVLIPAIT
ASTTSSVINVNSVDTDYLVHVLNNRMVNSLIILDFRANPKMIRNGLTPAYYILLLSGDIFDM
TFRMVYTIFGTSKSYPSASVLIASTIVPKSPSEQVATARRLLQMVWEGLVMYESIAILPHYN
NKSATKPNVENFGIFGWFPEKQQDPCLKQLDSIELIDTWIVKSNKFTKNAFLYPTKQLTD
MGGCFIYINIQNHNPLTLMENSKRPKGIMILVXNIIAYNLNMDIIYDLKATDNVTEAYAPVS
YFRDDVSDIGDCLLTYPHVQDRMKWLVPAGAPVLRWKSLIKIFSPIMWVCVVLVFVLGFA
TSWLLLEYSDEHRQNKKDLLSILMDILLNHLAMGISDTFKGLISKLFFVLWLFYCVIINTAY
QSALISFLADPGDDPSIRSLEELYNSDLKLFSRVELNGGVAEELNYLKNFEICEDLEQLIPGI
VKDRNVAILTNEYEGTVFRKYTFSQEAKKPQIEALEDNVLRTYVGIYVNKLGCLLYNRFEE
LMGRIVANGILNYQFEAMYRRLNKRYLALFNDDPFRLSLSHLQGGFYLLLIGLFISISAYFVE
KLFYNYTSR
>BgerIr439P
MMEVSWCLLQILILTSFQCGLSNIHSPKVVSPFVESMSECIVNIAEKYFDKQQPIAVLIPPKT
ISTHSIDGDYLVHALNNRMZNSLLILDYRANHKIIRNGLKPAYYILLLSGYIFDMTMNMAR
TIFETYKSYPSARIVIASTIVPKSPSEQVATARRLLQMVWEGLVMYESIAIVPHPNPKSATN
HNLDFFGIFGWIPEKQRDPCLKNLNRIDFIDSWLIRENXFDIYTTKYLPDMRGCTLTVQPR
NHPPLIYVKKSEEPGGTFLYFLKVIAKKINMKLAYLLKAKDNVCDVEIPVTYLHGNLSNMG
DCLLIYPHIQDSLKWIVPAGSVIPRWKSLTKVFSPVVWVCVVLVFLLGSVTSWLLFEYSSKK
LQNQNNLSKILIDILLNHLGMGISDLYSGWISKLFFGLWLFYCLIINAAYQSALLSFLANPGE
YPTMKSNKELYDSDIKLYSAVTFHGPLDEELGYLEDLEPSPDGMDSIVTNLKEGNTALLTN
MHEAMRYRLKTYNPETNKPQTAVLEDNVLRIYVGIYVNKFGCLLYNRFEELMSHMLESGI
VNFQLKLLSDIVEKHYALYYNLDLFKLSLTHLQGGFYLFVFGVFTXFFVVCVEILLYAYSN
>BgerIr440
MMEVSWSLLLYLVILSFQSCHSNIHLLRVVSPIVENMSQCIVNIAEKYLDKQQPIAVLIPPN
TVTATATNVNGNSIDGDYLIHALNNRMVNSLLILDYRANHKSIRNGLKPAYYILLLSGYIFD
MTIDMGRTIFESYKSYPSARIIIASTIVPKSPIEQVATARRLLQMVWEGLVMYESIAIVPNCN
PRSAMKHNLENFGIFGWFPQKQSDVCLKNLNRVDLIDSWLIRENKFVHNFDMYTTKYLP
DMRGCTLTVQPRNYPLLAYVRKSKTPTGLFLYFLKEISKKINMKLVYLLNAKDNVCDVEIP
VAYLHGNFSHIGDCLSTYPHIQDSFKWVVPAGSAIPRWKSLIKVFSPAVWAFVVLVFVLGS
LTSWLLFKYSSKKLQNRKNLSTILIDILLNHLGMGISDLYSGWISKLFLGMWLFYCLIINAA
YQSALLSFLANPGEYPSMKTNKELYDSDIKLCSHLGILGPLEEELRYLQDFESCVNVETSILS
IPKDRNTAVLTNEFEGKRCRLYTFSPDTNKPQTELLAESVLQIYLGMYVNKLGCLLYNRFE

ELMGHMVANGIVKYQFNWVSRSLEKHYALRYSRDLFKLSLTHLQGGFYLFLFGVFTSFSV
FCVEILLYGYSN
>BgerIr441P
MKRVSWSLLLILICLWFQSCYSSIHPLKVVSPIVESMSECIVNIAEKYFDKQQPITVLIHPKS
VTAHSIDGDYLVHAINNRMVNSLLIFNYRANPKMIRKALKPVYYILLVSGYXFGMMIYMGR
TIFETSKSYPSERILIASTVVPKSSSEQVATARRLLQLASEGLVMYESIAIVPNWNPRSEMT
HNLENFGVFVWFPEKQRDPCLKNLNRIDLIDSWLVREYIFIKNFDMYKSKYLPDMCGCSL
TVQPRHYPPLSYVENTKAPNGIIXKKIYMKLAYLLKDKGNTRDVEIPVAYLHGNLSNMGD
CLLMYPHIQDSLKWIVPACSAIPRWKSLTKVLRPVVWACIVLVFVLGFVTSWLLLEFSNKK
LQNKNNLSTILIDILVNHLGMGISDLYNGWISKLFFGLWLFYCLIINAEYQSALLSFLANPGE
YPTMKINKKLYDSIKLYSIVDVVGPLEEELRYLKDFEPCVNVEKSILSIPGDRNTAVLTNEF
EAMRCKWHTISPQINKAQAELLEEGVLQIYFGMYINKFGCLLYNRFEEFVGLLVSNGILKY
QFNCVSRGLQKYYSLRYSHDLFKLSLTHLQGDFYLFVFEVYTSFSVFCVEILL
>BgerIr442
MEVSWSLLLNLIVLSFQSCNSNIHPLKVVSPIVENMSKCIVNIAEKYFDKQQPIAVLIPPNTV
TATATNVNGNSIDGDYLIHALNNRMVNSLLILDYRANHKIIRNGLKPAYYILLLSGYIFDMT
MNMARTIFETYKSYPSARILIASTIVPRSPREQVATARRLLQMVWEGLVMYESIAIVPNCN
TRSTTNHSFENFGIFGWFPEKQRDPCLKNLNPIDLIDSWLIRKNKFENNFDMYTTKYLPD
MRGCTLYVLPRNYQPFAYVEKTEEPTGIFIFFLLVIARKVNMKVAYLLNTEDNVCDLEIPIT
YMHGNFSHIGDCLFIYPHIQDSLKWIVPAGSLIPRWKSLTKVFSPVVWACVVIVFLFGSVTS
WLLLRYSNNKLQNKNNLSKILIDILLNHLGMGISDLYNGWISKLFFGLWLFYCLIINAAYQS
ALLSFLANPGEYPTMKSNKELYESDIKLYSHVDLLGPLDEELGYLKDLEPCLDRVEKIVASL
KERNSALLTTVQEGFRFRLKTFNPETNKPETVVLEENVLKIYVGMSVIKFGCLLYNRFEEL
MSHMLESGIVNNQLKMRSNLVKKYYALYYNVDLFKLSLTHLQGGFYLFVFGVFTSCFVFC
VEILLFAYGN
>BgerIr443P
NFDMYTIKYLPDMRDCTLCVIAKKKZNKLTYLRNAKDNVCDVNILVAYLHGNISNMRDCL
LIYPHIQDSLKWIVPAGSVIPRWKRLTKVFSLVVWAZMVLVFLFZSVTSWLLFEYCNKKLR
YKNNFSKILIDILQNHLGLGISDLYNGWISKLFFGLLLFYCLVINAAYQSALLSFLANPGEYP
TMKSNKELYESDIKLYSAVTLLXPLEEDLGYIKSLEPAPDRVETIVTNLKERSTALLTNMHE
AMRYRLKTYNPETNKPQTVVLKNNVLQIYLGIYVNKFGCLLYNRFGELMSHMLESGIVNF
QLKLLSNIVEYHYALYYNLDLFKLSLTHQHCGFYLCVFGFFTSFFVFSFENLLSAYSN
>BgerIr444P
MMEVSWSLLQILVFSSFQCCLSNIHPLKVVSPIVESMSECIVNIAEKYFDKQPPIAVLIPTNT
VTTAALKVNDNLIDGDYLIHALNNRMVNSLLILDYRANHKIIINGLKPAYYILLLSGYIFDM
TINMARTIFETYKSYPSARIIIASTIVPKSPREQVATARRLLQMVWEGLVMYESIAIVPNWN
PRSAMNHNLENFGIFGWFPEKQPDPCLKNLNRTDLIDSWLIRENXFDIYTTKYLPDMRGC
TLTVQPRNHPPLIYVKKSEEPGGTFLYFLKIIAKKKINMKLAYLLKAKDNTCDVEIPVTYLH
GNLSNMGDCLLIYPHIQDSLKWIVPAGSVIPRWKSLTKVFSPVVWACVVLVFLFGSVTSWL
LFEYSSKNLQNKNNLSKILIDIMLNHLGMGISDLYSGWIXKLFFGLWLFYCLIINAAYQSAL
LSFLANPGEYPTMKSNKELYDSDIKLXGPLDEZLGYLEDLEPSPDRMDTIVTNLKERNTPL
LTNMHGAMRYRLRTYNPETNKPQTVVLEDNVLRIYVGIYVNKFGCLLYNRFEELMSHTLE
SSVLNFQLKLLSDIVEKHYALYYNLDLFKLSLTHLQGGFYLFVIGVFTSFLCFVLKFCCPLRV
IRFLK
>BgerIr445

MSWFFVMLFHALLLLGNSKIQSSNNVSQLVKSLSKCIVQITNKYFRQEHPITVLISTRNSSA
VSRNNFMNSNVDFILRSLNQGVNHSLVIVDYQNNTENNYKKSGSYILLITDHAESPVEITY
NMLSKIRHQLNPSLTMLIASIVVKPTEEQLYQARMVLKLTWEAFMISDAIFMVPMTVRRT
VMFGIFNWIPKKQPDPCFKTITYVDVVDCWITRIDGFHIKANLFPTKNIIDMKGCILRADV
VPHNEPLATITTDEVWGIFYDELEILRDALSLQIVYQRQNESSIADIILPLVYSATQKWFYT
NTYPYYMDNLKWYVPAGLPVPRWKSLTKIFNPCMWIFVVAIFILGSLTSWLLLKYSGVQS
LQPVDLSLVIIKILLTYLSMGISNKYQGIMATTFFTIWLFYCLIINTAYQSALICFLVDPGEYP
PIKTIKDLQESGLNLMSSISFSEHFGEDFQLINTYQRWRFDKNHPKTIWEKRDSALLTNEF
LGTFYTKVSFNSERNRPRITSIDENVKSLFFSIGIFSHGHLLFERMEELKHRLFSSGIPRVWI
NDATESQDRVFLGARNDNQFVIIKLPHIQGAFYFWAFGIITAIVVLLIEIVFGSLQMK
>BgerIr446P
MLWLLVMLFHAHILLGNSNSHSSYLSQLVKSLTECIAQISSKYFMQELPTALIIPINKFSVAS
SAKFMNSDVDFILRSLZHRINNSLVIQDYYNNTENNHMKPGFYILLLTDEVGSLLDLTYYIL
RNIHNQLNPSVNMLIASVFSKPIEEQHYMAILXLNFVWEVFIIPDAIVIVPMSIGRTVMFGIL
NWIXQKQPDPCFKTLGRINFVDXWSFRKEGFHVNANIFPSKKITDMQGCILRADVFLYEPL
AAITTSDVIWGIFYEELEVMKDDLNLQIVYQRQEEKFNRRFSVASSIFCNSKEVLZKIHIPII
WTNLNYIPAALPIPRZKSLTKIFNLCMWICVVTIFILGSLTSWLLLKYSEKRNLQSVDFSLVI
INTLLTYMSMGISNKYHGIMATTFFTIWLFYCLIINTAYQSALISFLADPGEHSPIKTVNVLQ
ESGMNFMSSINFSHHLGEEFQLTNTYELWSFVRNHPKTIWEKRGSALLANEFRGTYYTKV
SFNZKRNKPRITSIDENIKSLFFSIGIFSHGHLLFERMKELKHMLFCSGIPRKZINDVFQNKV
DLMVRNENQFIIIELPHMQGAFYLSAFGLLMALLVFFIELIFDSYKTK
>BgerIr447
MIMLWLWIMRFQALFLLGKSNIIPSNNVSKLVGSLTECIVQIARKHFTQDVPTAVLMPSNG
FDIASSTNFTNSDADFILSSLYRRINNSFVIINYRNNFIEINHTKPGSYILVIAGHDALSLLDM
SYYMLKSIRHQLNPSVTMLIVSINQNSLEQQYHMARSLLKMAWEGFIVSNAIVMIPLPTRR
KVIFGIFNWIPQKQTDYCFKALKHIAFVDYWILGKEGFHLNGNLFPSKQITDMQGCILRAD
VGFYEPFAAVTTFDELWGIFYEALEIIKVALNVQIDYQRIDESSVADIILPVFYSETEKWFYK
NTYPYYRDNLKRYVPSGLPIPRWKSLTKIFNPSMWIYVVITFILGSLTSWLLLKYSEKQNF
QPVDLSLVIINTLLTYLSMGISNKYQGIMATIFFIIWLFYCLIINTAYQSALISFLADPGEYPPI
KIIKDLQESDLNLMTSINFSHHFGEEFQVINSYQRWSFLNSDPNSIWKKRASALLTNEFRG
KMYTKVSFNSERNRPRINSIDENVKSLFFSIGIFSHGQLLYEHIDKLMHRLFSSGIPQVWMD
DVSAYLDRVYLPVGNDNQFVIIQLSHLQGAFYFWSFGMLIALVAFFIEIIFGNLQIN
>BgerIr448
MFHHLRIVLQVLLMLFPQNGESNTLIPDNISTLLENVFDCTVHIANQYFTKDLPTALFIPTL
KIMPTKYVSADYNYLDFLVQGLQQDTNQSLVMLNLLGSSIFLKKNFKIGAFILIVSGDTDAL
FITASQMLKSINKSRNPSSTLLIATTYVPKSENEEYYLARKLLRRVWRISHISDAIAVIPNKF
GDRLDAYNWSPDKHHDPCLEVLDQVDHLDSWTFERKEFLSKSNLFPKKQITDMKGCTLK
GFFYARPPLVNIRPSGFVWGTLTQELAIIANLLNFDVDTIKKEYVSMANFIVPATCHNDAF
KLDCTFIYPYHKDVMKWYVPSGELIPRWKSLTKVFNPFMWVCTVTTFIVGSVTSWLLQK
NSKSENKPTVNFSSVLIDMFSTYLAFSVSNKYNGPVASTFFVLWLFYCLIINTAYQSALISFL
ADPGEYKPISTIEELHESGLKFIGMLTSWSNLEEKALGVYAIANKSCNANNIAKCVEEASED
RSAAVFTTEFLGQMAMVMTYSPTRKKAKIIPIDGNVMELYITIAIPTHGCLLFKRVEELFH
RLFSSGIPQWINKEHFRDIRTQVKDFENEVLFATTLSHVQAAFYSWLAGIILACVLFAFEMI
FYRK
>BgerIr449

MFQHLRIVLKVLLMMFLQNGDGNNLLHDAVSPLLDNLLDCTVDIVNKYFTKDLPTAVFIP
TLGFMPTLSISSDYNYLDFLVHGLQQQTNQSLVTFDQIDSSVFSKKEIRIGSFILIVSGETDA
LFITANKVLNEILESWNPSTTLLIATTYMPKSENEEYYLARKFLRRVWRISQISDAIAVIPN
PFGDRLDVYNWSPDKRHDRCLKVLDEMDHLDSWIFEWKEFQNKSNLFPKKQITDMKGC
TLKGYFYARQPLVNIRPSGLVWGTLPSELVIISNLLNFDIDPIKKGHVSHPDFIVPATGDDD
TIDLDCTFFYPYHRDIFKWYVPSGELIPRWKSLTKVFNPFMWFCTVTTFIIGSITSWLLQK
NSKSENKPTVSFSVVLIDMFSTYLAFSVSNKHSGLVASTFFVLWLFYCLIINTAYQSALISFL
ADPGEYKPISTLEELHESGLNFIRLVNTWGSFEKRILGEYTFANLTCGRKDIVQCVEQVSKD
RSAAVITNEFLGQMAMVKTYSHIRKKARMIPIEGNVMELYISMAMFNYGCLLFKPLEELF
HRLFSSGIPQSMNKEHIRHIKTQFKDFENEVLFATTLSHVQGAFYSWLAGILMACVVFVFE
IIFHRQ
>BgerIr450
MFQHLRIVLQVLLMLFPQNGDSNNLLHDAISPLLDNLLDCTVDIVNKYFTKDLPTAVFIPT
MGFMPTLSISSDYNYLDFLVQGLQQQTNQSLVTFDQIDSSVFSKKKIRIGSFILIVSGETDAL
FITANKILNKIFESWNPSSTLLIATTYMPKSENEEYYLARKLLRRVWRISQISDAIAVIPNPF
GDRLDVYNWSPDKRHDRCLKVLDEMDHLDSWTFEWKEFLSKSNLFPKKQITDMKGCTL
KGFFYSRPPLVNIRPSGFVWGTLTSELVIIADLLNFAVDPVVEGYVSNPDFIVPTSCDDDTI
DLDCTFFYPYHRDIFKWYVPSGELIPRWKSLTKVFNPFMWVCTVTTFIVGSVTSWLLQKN
SKSKNKPTVSFSLVLIDMFSTYLAFSVSNNHNGPVASTFFVLWLFYCLIINTAYQSALISFLA
DPGEYKPISTLEELHESGLNFIKLVNPWGSFEDRILGEYNFANVTCGGIDIVQCVEQVSEDR
SAAVITNEFLGQMAMVETYSQTKKKVRMIPIEGNVMELYISMTLFDYGCLLFKPLEELMH
RLFSSGILQWMNKEHSRYFKTQFKDFENQVLFATTLSHVQGAFYCWLAGILLACVLFVFE
TIFYRKKGL
>BgerIr451
MMGILRVHLKILFLLILAHIGHTNILRHDTMSTLVEKLSECILEIADKYFTQIRPTEVFFPAK
KLSPEIYFSPLVQILHQRLNQPVIISDNRKRPLHKLKPGSYILITSGEGELKHDTTYKILQAM
VDLGLNLSANLIITTTDVQKSRNEEYYFAGISMDMIWQELQIADMIAVAPGTSTRGQIDRF
GVYNWTPEKQEDVCFKALTHVELIDSWIPERKAFLNNVTLYPKKKITDMRGCVLHAAYT
PYAPLVFRKKQYIYFGTMIELFGVVERFLNFRLTVSSRAKTSEISDFYIPIGYGDFELLVEDC
LPLYPYYSDDIRWYVPSGAPVPRWKSLIKIFNPFLWTCVATTFIVGSLTSWLLLKYTNHEE
RAVALIFVIIDTMLTYLAVGITDKYKGSVATTFFVIWLFYCLIINTAYQSALISFLANPGEETP
IKTVQELEDSGLNMLSMIDFSIGHLQELTKYEMCKKFQICIKNAVNHRDTALLLNFFGGRL
AIHHSFNSETKKPGIVAIDEGSHTLYFSSSINIHGCLLFNRVSEIIGRLFNAGLITRDLEFFYG
RDKIFFTKFLNQDPFVMTLSHLQGAFYLLTIGMTFPLFAFLCEIIFHSIYKYANLRE
>BgerIr452
MNMAFYGRLCLIILFLLFVKDGTNRLPLDSVSPVEDSLSQCIAKIANEYFNRDLPTALFIPY
REYESQSYISNNNSHVDFLVQSLHQRIDNSLVMLDYHNNPETLLRKVKLGSYILILSGEVSL
YFDLGFEVIWKIYEVAGHMSPSGRLLIATTGSPDKERIILIPKLLQVIWDVLEISEAILLLPRI
RSNNDDVIEVYKWLPEDQDDPCFLLLNRFVVLDIWIMKVNEFIAGTTLFENKLITDMKNC
KLNLTLRHYPPFVAMNDRYMLGPLVDLLFILFHTLKIRLYLNQPGVEQVANFELPVGVTN
VTNKELDINQECVAIYPYFIQHLKWVVPAGAPVPRWKSLIKIFNPLMWLCVVTTFIIGSTT
SWLLLNQSQQSLTYISALLDTLLTYVVVGISDRYKGTVATTFFLLWLFYCLIINTAYQSALIS
FLNDPGQEQPIKTIEELHKSGLHLMSMIFNAFVVESENIEETSHFDECSFMIPCLKLIAQNG
DTALLDDEVLLRLVVNKYFDTETNRHLLHTIEENAYTFYFTIAVYSHGCLIFKRMEQLMH
RMWSAGLIIKYLNGFNEVQRIIYYGVLNDDPFVITLSHLQGAFYFLTCGLFVSIIVFLIEISYW
SVLV

>BgerIr453P
MNMVFDGRLCQTTLFLIVVNSGTNPLPLDSASPLEXCIAKIANEYFNRDLPTALFIPNREYE
SHSYISSNDSHVDYLVQSLHQRIDHPLVMLDYHNNPQSLHQKVKLGSYIIILSSEHSRSNAL
MALEVISRIYVIAGEMSSSGRLLIAMTATXRSTGDDVIEVYKWFPEEQDDPCFLXWILKLN
KFNNDETLFKTDMNNCKLNATAKNYPPIVIMNTEVVFGPLASQIRILAGTLKMRIHFNAA
GLRSFPRFELPVCETYERNNELPINQECVVTYPYFMQHLKWVVPVGACVPRWKSLIKIFN
PLAWFZIVSTFLIGSTTSWLLLKQSHQSLTYISALLDTLLTYVVAGISDRYKGTVASTYFFL
WLFYCLIINTVYQXSALISFLADPGHDQPIKTIEELHKSGLHLMTIFQLFDYTQTKELQEIN
NYDSCDNNRFQCYKRVAQNRDTAILDGEEIAYLMLHRNYDSGTKKYLITNIEENVYTLYF
AMAIYSHGCLLFNRMEZLIHRMFCAGILIKXSGLSSSVSRKMNQGVLKNSQFLISVSHLQAS
FYFLMSGFTVSIIVFLIEVMYHSI
>BgerIr454P
MLLVRFFCEIIFLPLLIKGGTHPLPLESMSPVEDCLSRCIAKIANEYFNRDLPMALFIPYREH
VSHSYISTNDSHVDFLVKSLHHSIDHPLVTLDYHNNPQSLHQKVKLGSYVIVLSGEVNSYIS
MAVEVILKISEVAGHMSPSGRLLIATTGSPNKERITLIRIFFNIIWKVLEMSEAIVLMPRIGS
TAEDQTIEVYKWYPEEQDDPCLLLLNHFRFAELSITEINTXLFQDNIITDMRGCVLNISVVD
YPPLTYVKDSEFVFSLMGHQLIILFEALNLRMTLNPANLSSLPNLKLPHIVIFEGKRDLLVN
IDCVVTYPYFIENLKWLVPAGALVPRWKSLIKIFKPLMWFCVVSTFLIGCTTSWLLLKRSH
QSMTYISALLDILRTYLASGISDRYKGTVASTFFVLWLFYCLIINTAYQSALINFLNDPGQEE
PIRSLEELHESGLNLISKVRFSNAEAEEMLQINNYGTCQNNSYCLKNIVQNRNSAMLLPEY
SANFLIHDFYDSEKNKYLLTIIDESVSTLYLTIAIYTHGCLFFKRMELLVHRMWSAGLIMRSI
RHIQFISRRISLDLFNSDPYVITLSHLQGGFFFLTSGLFLSVSVFLLEILYHAL
>BgerIr455P
SNLLPQIRSTTVEDVIEVYKWFPEEQDDPCLLLLNRFSLFNFWIMKENNFIIDATLFETRFI
KDMNNCLLNATANNYPPLVMMKIGVVLGPLVQQLHILAGTLNMRILFNPAELKLFPNFEL
SVHLTYVKNKEVKEIKKIHQECIITYPYFMQHLKWVVPAGACVPRWKSLIKIFYPLMWFC
VFTMFLIGSTTSWLLLKLSHQSLTYISALLDSLLTYVVARISDRYMGTVASTFFLLWLFYCL
FINMAYQSALISFLADPGHDEPMKTIEELYKSGFHLMSRFHSKNPQTKELVEANSYETCYK
LEQCYRSVAQNRDTAILDDEQLTFSLQHYFYNSVTKKFLFTNIDESVYTWCFAMAIYTHGC
LFNQIEQLIHSMFCAGLLTRIQHLLDELSRIENLKILNNAPFVITLSHLQANFYFLMSGLFVS
IIVILIEVMYHSI
>BgerIr456
MFLVRRFCEIIFLSLLIKGGTHPLSLDSVSAVEESLSRCIAKIANEYFNRDLPTALFIPYREYE
SHSYISTNDSHVDFLVQSLHQRIDHPLVMLDYHNNPQPLLRKVKLGSYIIILSGEVSSYFDM
GFEVFRRIYEVARHMAPSGRLLIATTSSPGKKRNIIIGKLFQAVWRVLEISEAILLLPRIRSN
DDGVIEVYKWFPEDQDDPCFQLLNQFELLDIWIMKVNEFVAGTTLFENKLITDLKNCGLN
VTLHNYPPYAMRDFRDIFSPLVDQLFILCNTLKFLFNFKQPGLEPVSNFVLPIRVTYYVTNK
ELDINQECVLTYPYFIKHLKWVVPAGAAVPRWKSLIKIFNPLMWRCVVSTFLIGSITSWLL
LKQSQQSLTYISALLDTLLTYVVAAGISDRYKGTVASTFFLLWLFYCLIINTAYQSALISFLAD
PRHDQPIKTIDELHKSDLRLMSRVSISGVELEKIEVISQSTNQRHWLKRIAENRDTALLDD
ELPFGLVINNYFDTETNKHLLHIIDKNAYTFYFTIAVYSHGCLIFKRMEQLLHRIWSAGLIIR
HFNYFYKVQRRRYYEVLNDDPFVITLSHLQGAFYFLMCGLFVSIIVFLIEISYWSVLV
>BgerIr457
MGRNILHLTHSIPFSSHSSMWVRGRFCQIIFLSLVLKGGTNPLPLDSVSPVEDSLSRCVAKI
ANEYFNRDLPTALFVPYREYESHSYFSTNDSHVDYLVQSLQQRINHPLVMLDYHNNPQPL
NREVKLGSYIIILSGDVSSYIDMAIEVSVTIYKVAGHMSPSGRLLIATTGSPNKERHIIIGKLF

QVIWRALEISEAILLLPRIRSTDDNVIEVYKWFPEEQDDPCLQFLNRFVSIDIWFVKVNKFL
LNLKLFQDNVITDMRGCVLNANVNEYPPLVVRDTDSIVGTLVHQLHITCDILNLGMNLNI
GDLTSKSNMQVPAAFRTDSDMTFLQDCVATYPYFIQNLKWLVPAGAPVPRWKSLINIFNP
SMWLCVVTTFLIGSATSWLLLKENQQSVTFMSVILDTLLTYLAAGISDRYKGTVASTFFVI
WLFYCLIINTAYQSALISFLTDPGQEEPIKTIKELHNSGLRLMSRLLILNAEDDEIAEINSYEI
CEVHHLCFRSIAQNRDTALLDEELLARSLMENFFDSETNRYKLHIIDESSYTFYFTMATYT
HGCLLFKRMEQLVHRIWSAGLIIKSYDYYYMVVRRRSHELVNNNPYAIALSHLQGGFYLLA
SGLFVSLIVFLIEVMYQSV
>BgerIr458P
MAFHQMTLLGATLFLLLQIGFANIPQSFTTSTLVRSLSTCIAEISNKYFNKDLSVALLIPDRD
LRTSLYISSSHNHVDLLVRSLHQQMDHSFLILDFQKNPRPFLQKPKVGSYILVLSGKYSRFR
YSDMAYQILRAVNEVHPGVRFSTASVLIATIGSPKSPIETLNLAITLLNIVWSIARISDAIVLV
PVIGSIDFGNRFDIFNWVPEDQISPCFKILDKVRYIESWILNRNMFHSNAVLYPRKPLTSMR
GCCLNVEFHKSGFHWFVLDEIIQIKZFLQFDLCDIPVDFDITPDLELPILYYFNTPVLKYECV
AMYPYYMLKLKWFVPAGAPVPRWKSLIKIFNPLMWFCVITTFLTGSITSWLLLKRNQQYL
TFLSVLFDTLLTSLAAGISDRYKGTVASTFFLLWLFYCLIINTAYQSALISFLNDPGQDEPIR
TIEELHQSGLHLRSTIRVLNTSLKERNNVNEYELCENLNECYEHVSENRNTAVLDLDFNG
NVKRNSSYNSEINRYQIVVLDETMLTFYLTTAIYNHGCFLLKRMEELMLRLTSAGLANRH
MGQILKKQSIHFSELVNKNPYVISLSHIQGAFYCLIFGLFISCVAFVTEVIYNCSSSYLFV
>BgerIr459P
MNMVFDGRLCQIIFLPLVLKGGTNLISLDSVLTVEESLSRCVAKIANEYFNRDLPTALFIPY
REYESHSYISTNDSHVEFLVQSLHQRIDQTLVMLDYHNNPQSLDRKVKQGSYVIILSGEVSS
YFDMAYKVFVTIYEVAVHMSTSGRLLIATNGSPNKERISLIWQLFNMIWKGLQISEAIVLL
HEXSAEDQTIDVYKWYPEEQIDPCLLFLNRFGFLDSWIIKMNSFKNNVELFQNNIITDMRG
CVLNVSVENYHPFAFVRDNKISGLMVFQLVILFEAMNLRMILNPPNLESPPNIELPLMVTF
ERKNDLPRNGDCVTTYPYFTQNLKWFAPAGSLVPRSKSLIKIFNPLMWLCVVSTFLIGSTT
SWLLLKQNQKSLTYISVLLDTLMTYVAAGISDRYKGTVASTFFVLWLFYCLIINTAYQLALI
SFLNDPGQEQPIKSLEELHESGLHFITEVMFRNEYVEEMLQINNYETCNDRRYCLKMIVQN
RNAAMLFPDYQANSIFRRLYDSEILSIIDESEYTLYFTMSTFTHGCLLFNRLEQLVHRIWSA
GLKVRRTIMYQFIMNRHDLDHFNTDPYVITLSHLQGSFFFLISGLFLSVTLFLIEIMYHAL
>BgerIr460
MALADRRFQIIFLSLVLRVGTNPLPLDSLSPVEDTLSRCIAKIANEYFNNDLPTALFIPYREY
ESHSYISTNDSHVDFLVQSLHQRINHPLVVLDYHNNPQPLKRKVKLGSYIIILSGEVSSYIDM
AYKVIVTIYNIAGHMSPSGRLLIATTGSPNKEKITLISTFFHMIWKILQISEAIVSLPRIRTSA
EDYIIEVYKWYPEEQNDPCLLSLNRFSIFSIPISVIKSSFYSFELLENNIVTDMRGCVMNISVQ
ELPPHTYVKGNGIVFGPIAVQLKILFEALNLRMDLNPTNFKTHPNLELPRILSFEGNHYFHI
NHDCVATYPYFIDNLKWLIPAGAPVPRWKSLVKIFNPLMWFCVVTTFISGSTTSWLLLKQ
SHQSMTYISALLDTLMTYVAAGISDRYKGTVASTFFLLWLFYCLIINTAYQSALISFLNDPG
QEQPIRSLEELHESGLQLISKVVFEHPEGEELLEINNYESCHNNDYCLKMIAQNRNAAMLL
AENQVYFFMHRHYDSEINKYLISIIDESAYTMYLTMATYTHGCLFFKRMEQIVHRIWRAGL
IMRYIQHYQFHERRVRLDIFNNDPFVITLSHLQGGFFFLISGLFLSVTVFLIEIMYHAVDVYK
VPTISI
>BgerIr461P
NNILALLIKGGTHPQPLDSVSPVEESLSRCISKIANEYFDRDLPTALFIHYREYESHSYISTN
DSHVFFIVKZLHQMIDHSLVMLDYKNNPKYLDKMIKLGSLVILSGEVSSYISLAVEVILKMY
EVAGHMSPSGRLVIATTGSPNKERITLIRIFFNVIWKVLEMSDAIDLLPRIGSSAEDQTIEVC

KWYPEEQNDPCLLFLNQFDFAEISITEINTSLCNLILFQDYIITDMRGSVLNISAVDYPXDSE
FDLCPMGHQLIILFEALNLRMTLNPANLSSLPNFKFEGKRDLLIKKDCVATYPYFIENLKW
LIPVGASVPRWKILIKTFNPLMWFCVVTSFITGSTTSWLLLKQHQKSLTYISVLLDTLLTYV
VSGIPYTYKGTVASTFFVLWLFYCLIINTAYQSALISFHNDPGQEQPIRSLKELHESGLHLIS
KIRFPNAEAKEMQQINNYGTCQNNRYCLKXIVQNRNSAMLLPEYSANFTFHYFYDSEKNK
HLLSIIDESVYTLYLTIAIYTHGCLFFKRMEQLVHRMWSAGLIMRSIRHIQFISRRISLDLFN
SDAYVITLSHLQGGFXFLISGLLLSFTVFQIEIMYHAV
>BgerIr462
MAFDCRLCLILLFLLVVKDGIIPLPLDSVSPVEDSLSRCIGKIANEYFNRDLPTALFIPYREYE
SHSFISTNDSHVDFLVQSLHQRIDNSLVMLASHNNPQPLLGKVKLGSYIIILSGEVSLYFDLG
FEVIWKIYEVAGHMSPSGRLLIATTSSPDKERNIFIGKLFQTIWHILEISEAILLLPRIRWND
DDVIEVYKWFPEDQDDPCFLLLKQFELLDIWIMNVNEFISGAILFENKLITDMKYCKLNTT
LHHYPPFVARNDRYMFGSLVDLLFIMCRTLNIRLYLKQPGVEPVPSFELPVRVETVRNEEL
PIDKECVNTYPYFIQHLKWVVPAGAAVRRWKSLIKIFNPMMWFCVLTMFLIGSTTSWLLL
KQSHQSLTYISALLDTLLTYVVAGISDRYKGTVASTFFVLWLFYCLIINTAYQSALISFLADP
GHGQPIKTIEELHKSGLHLITMSQLFDYTQSKELQEINSYDSCDNSRIQCYRRVAQNRDIAI
LDAEEIAYLMLHHNYDSGTKKYLITNIEENVYTFYLAMAIYSHGCLMQQLIHRMFSAGILT
RLQNLLVQYSTILNQEVLNNSPFVITLSHLQASFYFLISGLFVSIVVFLIEVMYHSI
>BgerIr463
MWVRSTFGQIIFLSLLLNGGTNPLPLDSVSPVENSLSRCIAKIANEYFDGDLPTALFIPYREY
DSQSYISTNDSYIDYLVQSLHQRIDHPLVMLDYHNNPQPLLRKVKLGSYIIILSGEVSLYFDL
GFEVIWKIFKVAGHMSPSGRLLIATTSSPDKERITLIGKLFQAIWRVLEISEAILLLPRIRSNG
DDVIEVYKWFPEDQDDPCFLLLNQFELLDIWIVKGTEFIAGTTLFENKLITGMKNCGLNVT
LHNYPPYAMKDFKDKFSPLVDLLFILCDALKFRLYLNRPSLESVPNFEFPVRVKNVTNKKL
DINQECVITYPYFIQHLKWVVPAGSPVPRWKSLIKIFNPLMWFFVFTMFLIGSTTSWLLLK
QSQQSLTYISALLDTLLTYMVAGVSDRYKGTVASSFFLLWLFYCLIINTAYQSALISFLADPG
HDQPINTIEELHKSDLRLMSRVSISDEEFEKIEEISQSTNQRDWLTRIAQNRDTALLDDELP
LGLVINKFFDTETNRHLLHIIDKNAYTFYFTIAIYSHGCLIFKRMEQLLHRIWSSGLIIRHFN
YFYKVQKSRYYEVLNDDPFVITLSHLQGAFYFLICGLFVSIIAFLIEICYWSVLV
>BgerIr464
MNMAFDGRLCLITLFLLVVKGGTNSLPLRSVSAVKDSLSRCIAKIANEYFNRDLPTALFIPY
REYESHSYITTNDSHVDFLVQLLYQRIDHPLVMLDYHNSPQPLVRKVKLGSYIIILSGEVSLY
FDLGFEVIWKIFEVAGHMSPSGRLLIATTSSPDKKRNILIGKLFQAIWRLLEISEAILLLPRIR
SNGDDVIEVYKWFPEDQDDPCFLLLNHFVLLDIWIMKENELISGKTLFENKLITDIKNCGL
NVTLHNYPPLTVSNVRELFGPMVDLLFIMCNTLKIRLFLNRPGLEPVTNFELPVRETYLRN
NELPIYQECVVTYPYFIQHLKWVVPAGAPVPRWKSLIKIFNPLMWFCVFTMFLIGSTTSW
LLLKQSQQSLTYISALLDTLLTYVVAGISDRYKGTVASTFFLLWLFYCLIINTAYQSALISFLA
DPGHDEPIKTIEELHKSELHLMSMFFNVLVESEEIEEMSHFGECTFLYPCLTTVAQNGDTA
LLNDELLLRVVINKYFDAENNRNLLHTIEEIAYKFYITIAVHSHGCLIFKRIEQLQHRIWSAG
LIIRQFDYFYETQRNIYYDVLNDDPFVITLSHLQGAFYFLTCGLFVSIIVFLIEISYWSVLV
>BgerIr465P
MNMVFDDRLCQIILLLLVVKGGTNPLPLDSVSTVEESLSRCVTKIANEYFYRDLQMAFXHP
YREYESHSFISTNDSHVYFLVQSLHQTIDHPLVMLDYHNNQQSLDRKVKLGSYIIILSGEVS
AYMDMAIEVVFKIFYIAREMSPSGRLLVATTGSLNKGRITLIWQFLXNMIWKVLQISEEIVL
LPKLESSADFAEDQTIEVYKWYPEEQNDPCLLFLNRFGFLDSWIIKINSFKNNFELFQNNII
TDMRECVLNVSVENYPPFTFVRDNGISGLVVFQLVILFEAMNLRVNENPSNLKTPPNMEL

PLMVTFDRKNNFPINEDCVAXPYFTQNLNWLVPAGALVPRWKSLINIFNPLMWFCVVST
FLIGSTTSWLLLKQNQKSLTYISVLLDTLMTYVAAGISYRYKGTVASTFFMLWLFNCLIINT
AYQSALISFLNDPGQEQPIRSLEELHESGLQLITKGKFQNADVEZMLQINNYETCNDHRYZ
LKIIVQNRNTAMLVPZYQANSVSRRFYDSEINKYLITVIDESEYTMYFTMATFSHGCXLFNR
MEQLVHRIWSAGLIIRHTRMZZFIANRHDLDYFNTDPYVITLSHLQGGFFFLISGLFLSVTF
>BgerIr466P
MSLAERRFQIIFLSLVLRVGTNPLHLDYVSPVEDSLSWCIAKIANEYFVRDLPTALFIPREFE
SHSYISTNDSHVDYLVQIVHQRINHPLVILDYHNNPQPLKRKFKLWSYIIXLSGEVSSYIDM
AYTVIVTIYNVAGHMSPSGRLLIATTVSPNKERIALIRIFFNMIWKILQVWEAIVLLPRIRTS
TEDHINEVYKWYPEDQYDPCLLFVKQFSFYSIPITAIKSSPYNFNLFDNNIVTDMRGCFINI
SVQVVAPHTYGMNNEFVLGAIGTQLDILFEALNLRMNLKPTNPKSHPNLGLPXSIQLFTYN
YDCVATYPYFIDNLKWLVPAGALVPRWMSLIKILNPLMWFCVVSTFLIRFTTSWLLQKSL
TYISVLLDTLMKYVVAGIPDMYKGTVASKFFLLWLFYCLIINTAYQSALISFLNDPGQEHPN
RSLEELHESGLQLISTVVFEHPEGEELLEINNYEHCHNQENCLKKIVKNRNVALLLPENQV
YFFIHRFYDSEINKYLISIIDESAYTMYITMATFTHGSLFFKRMEQLVHRIWSAGLXTLSHZ
QGCFFLLISGLFLSITVFLIEKSCTTLFICV
>BgerIr467P
FDGRLCLITLFLLVVKGVTNPLSLDSASPLEESLSKCIAKIANEYFNRDLPTALFIPYREYES
HSYISNNNSHVDYLVQSLHQRIDHPLVMLDYHNNPQSLHQKVKLGSYVIILSAEHSRSNAE
MALEVIFRIYEIAREMSSSGRLLIATTWSPKWERQDLVVSILXIWSLLEISESIVLLPRIRSTG
DDVIEVYKWFPEDQNDPCFLHLNRFVLLDIWILKLNKFNNDATLFENRLIKDMNNCELN
ATTNSYPPLVIMNTGVVFGPLASQLRILAGTLKMRIHFNPAGPRSFPNFELPVCETYVRNN
ELPINQECLVTYPYFIQHLKWVVPAGAPVPRWKSLIKIFNPLMWFCVFIMFLIGSTTSWLL
LKQSQQSLTYISALLDTLLTYVVAGISDRYKGTVASTFFLLWLFYCLIINTAYQSALISFLAD
PGHDQPIKTIEELHKSGLHLMTIFQLFDYTQTKELQEINSYDSCENSRFQCYRRVAENRDT
AILDGEEVAYLMQRHYYDSGTQKYLITNIEENVYTLYFAMAIYSHXMLFNRMEQLIHRMF
SAGILTRLQNWLVQFSRILNQEVLNNAPFVISLSHLQASFYFLMSGLFVSIIVFLIEVMYHSI
>BgerIr468P
MSLADRCFZIIFLSLVLRVGTIPLPLDSVSTVEESLARCIAKIANEYFDRDLPKALFIPYREYE
SRSYISTNDSHVDFLVQSLHQRIDNSLVMLDYHNNPQYLRQKVKLESYIIIQGKLVYTLIWA
LKLFGKFMMLQDIZHLREDSZLLRLGHLIKRGZLZYTVAIFNMIREVLQIWEAIILLPRIRTS
AEDPIIEVYKWNPEEQDDHCLLFLNRFRIFSISIAVINTSLNNLKLFQNNIVTNMRGCVLNI
SVEDFPPITGLKGNKLGFGFMVYQLLVLFEAFNLPMNXNPANLCSLPNWGLPEIVTIGTSN
DLLINNDCIATYPYFIDNLKWLVPAGAPVPRZMSLIKIFNPLMWFCVVSTFLIGSTTSWLLL
KQNQKLLTYISVLLDTLITYVAAGISDRYKGAVASTFFMIWXSTAYQSALISFLNCPGQEQPI
RSLEELHESGLQLVTKVGFQNGGTEELLEINNYGSCHNNDYCLKMIALNRDAAMLLPEQQ
ANFLIHIFYDSQINKHLITTIDETVYTLYFTMATFTHGCLMFRLIHRIWSAGIIMRIIREIQFI
ARSSIDLFNSDPNVITLSHLQGGFYFLMSGIFVSFIVFLIEVMYHSI
>BgerIr469P
MLLVRLFCEIIFLPLLIKGGTHPLPLDKVSPVEESFSRCIAKIANEYFNRDLPMALFIPYREY
ESHSYISTNDSHVHFMVKSLHQMIDHSLVMLDYHNXPQPLEQKVKLGSYVIILSXIISNIDM
TIKVILKINEVAGHMSPSGRLIIATTGSPKWNTADLVGNFFNIIWRVLEISESTLLLPRIRST
TVEDVIEVYKWFPEEZDDPCLLLLNRFSLFNFWIMKVAGTLNMRILFNPADLKSFPNFEL
PVHVTYVKNKEIKEIKKVHQECIITYPYFMQHLKWVVLAGAPVPRWKSLIKIFNPLMWFC
VFIMFLIGSTTSWLLLKQSQQSLTYISALLDTLLMYVVAGISDRYKGTVASTYFLLWLFYCLI
INTAYQSALISFLADPGHDQPIKTIEELHKSGLHLITIFQLFDYTQTKELQEINSYDSCDNSZ

FQCYRRVAQNRDTAILDGEEIVYLMQRYYYDSGTQKYLITNIEENVYTLYFAMAIYSHGCL
LFNRMEQLIHRMFSAGILTRLQNWLVQFSRILNQEVLNNALFVITLSHLQASFYFLMSGLF
VSIIVFLIEVIYHFI
>BgerIr470P
MMPGRRFCEIIFLPLLIKDRTHSQTLDLVSPVEESLSRCVAKIANEYFNRDLPTALFIPYREY
ESHSYISTNDSHVVFIVKSLHQMIDHSLVMLDYHNNPKYLDQKVKLGSFVIILSGEVSLVVE
VILKIYEVAGHMSPSGRLLIATTGSPNKERITLIRIFFNIIWKMLEMSEAIVLIRRIGSTAEDQ
TIGVYKWFPDEEDELCLLLLNQFGFAEISITKINRSLNIGYYFKTISLQTCVFVFSISVLXLFQ
DNIITDMRVCVFNISVVGYPPLTYVKDNEFVFSLMDHQLIILFEALNLQMSLNPANLSSLPN
LKLPHIVIFEGKRDLLIKKDCVAXLVPAGASVPZWKSLIKIFNPLMWFCVVSTFLIGSTMSW
LLLKQSQQSLTYISALLDTLLTYVVTGISDRYKGTVASTFFLLWLFYCLVINTAYQSALISFL
NDPGQEQSIRNVEELQESGLHLMSRFFSKNPQNKELVEINSYETCYDSMQCYRRVAQNRD
TALLDYEQLTFALQPYFYDSVTKKFLITNIDGSVYTWCFAMVIYTHGCLLFNRIEQLIHRM
FCAGLLTRIQNLLDELWRVEYLNVLNNAPFVITLSHLQGSFYFLMNGLFVSIIVFFIEVMYY
SI
>BgerIr471
MNMVFDGRLCQIILLILLLKGGTNPIPLDSLSPVEDSLSRCIAKIANKYFNRDLPTALFIPYR
EYESRSYISTNDSHVDFLVQSLHQRIDHPLIMLDYHNNPQSLHRKVKLGSYIIILSGQVSSYF
HMAAEVIFKIYEVAREMVPSGRLLIATTSSPNKARNILIRKLFNVIWRALEISEAILLLPRIR
STTIEDTIEVYKWFPEEQNDPCLLLLNRIVLFNIWIIKVNEFIYDATLFDNKLITDMNNCELI
ATLHNYPPLVQINGRAILGTLASQLVIMCHTLKIRIYFYQMGLGTSPNFGLPVRVKNEKNK
ELLLNQECVVTYPYSIQRLKWVVPAGALVHRWKSLVKIFNPFMWFCVVTTFLIGSTTSWL
LLKQSHQSMTYISAILDTLLTYVVAGICDRYKGAVASTFFVLWLFYCLIINTAYQSALISFLA
HPGHDEPIKTIEELQKSGLHLTTNVLISNGESELNFDECTNRSHCLTRIAQSHDAALLYDDL
ILSLAINKYFDTETKRHLLHIIEETAYTLYFTIAVYTHGCLIFKRMEQLIHRVWSAGLIIRYF
NYFHELQRIRYYASLKDDPYVITLSHLQGGFYFLMCGLFLSIIVFLIEISYWSV
>BgerIr472P
LKGDTIPLPTDSVSPLEECLSRCIAKIANEZFNRDLPMALFFPYRKYESHSYISTNDSHVDFL
VQSLHRRIDHSLLMLDYHNIPQSLHQKVKLGYYIIVLSNEVSSYFDMASEVFLRIYELARHM
TPSGRLLMTGSPKGKXNYLVSRLLCQTWRVLEMSEAIVLLLRIGSSAGLDIIEVYKWFPEE
QDDPCLLYLNQFVFLDMWIIKISIFXLFQNELIRDMWECVLNVTLDDYSSLVMIINTGQVF
GAIVDQIKTISVVLNLQVKFNVTTLSNLHLPVKFGRNKELLINZDCZATYPYFKQSLKWFV
PAGAPVPRXMWFVTTFLIGSTTSWLLLKHSHQSMTYISALLDTLLTYVVAGISDRYKGAVV
STFFMLWLFYNLIINTAYQSALISFLAHPGHDEPIRNIEELHKPNLHLMSKVTVPNTEIEE
>BgerIr473
MNMVFDGRFCLRILLLLVLKGCIKPLPLDLVSPVEDSLSRCIAKIANEYFNRDLPTALFIPYR
EYDSHSYISTNNSHVDYLVQSLHQRIDHPLVMLDYHNNPQSLHQKVKLGSYIIILSGEVGSY
IDMAIYVILKIYEVAREMSASGRLLIATTGSPTFERDGIVGGLLRLIWKILQISEAIVLLPNIES
TSVHDDVIEVYKWFPEEQDDPCMLSLNPFVLLDIWILKGDQFSRGKKLFQTDLITDMRGC
TLNLSFLNYPPLVYTQDDVYVIGPLIDQIKLIIEAINLRLKIFRRNFTDFTNMKLPISLLNVK
DNDFLTYGDCVVTYPYFKQNLKWVVPAGAPVPRWRGLIKIFNPLLWFCVVATFLIGSTTS
WLLLNQNRQSQTYISALLDTLRTYVVAAISDRYKGTVASTFFVLWLFYCLIINTAYQSALIS
FLADPGRDEPIKTIAELHNSGLHLMSRVFILNEELENIGETEHYSGCTNQSHCLTRIAQNRD
TALLDDELLLGLVINKYFGTETNRYLLHIIEEIAYKFYFTITVYSHGCLIFKRMEQLIHRVWS
AGLIIRYFNDFYKIQRTAFYEVLNDDPYVITLSHLQGGFYFLICGLFVSTIVFLIEISYWSV
>BgerIr474P

VVRNNIHSLHLVFKGGYYPLPLDSVSIVEDSLSRCIAKIANEYFNRDLPTALFIPYRKYESHS
DISSDDSHVDFLVQSLHQRIYHSLVMLDYHNNPQSLHRKVKLGSYIIILSGEIRSYIDMAIYV
ILKIYEVVKEMSPSGRLLIASTGYPKWERTKLVGTFFNMIWQALKISEZFVLLPRIGSNDEG
VIEVYKWFPEEQDDPCLLFVNTFQVFDVWIFKLNIFLFNLKLFQNNIITDMRGCVLNISVS
AYPTLVRATGNKLDAGLMADQLVILFEALNLRMNLNPANLTSHPNLLLPVLATIENGIDL
QYFZIEIAZLLIPTSHRNZNGPRWKSLIKIFNLLLWFCVVATFFIGSTTSWLLLNQNQLPLT
YISALLNNLLTYVVAGISDKYKGTVASTLFVLWLIYCLIINTAYQSAXFLNDPGHZQPIRSVE
ELRGSDLHLMSNILLXNKKVEKVLRISNYETCEDNHVRLKMIAQNLKIALLIPEHHANFVM
CHFYDSEINKYLNSTIDESAYTLYLTMSFNTHGCLLFKRMEQLIHRLRSAGLLLDILDCFYL
FVYVPQNNDPYVIKLSHLQGAFYFLTSGLFVSLLVVFLIEIMNHAL
>BgerIr475P
MFFDIRLRQIISLCLVLMGGTSPLPLDSVSTVEDSLSRCIAKIANEYFNRDLPSALFILQXYES
HSYISTNDSHVDFLVQSLHQRKDHTLVMLDYHNNPQSLHQKVKLGSYIIILSGKVNSYIQM
TVEVSEIINKVAGHMSPSGRLLIASTGFPKYKRTKLVGIFFNIIWRMLEISESIVLLPQLGSTD
DDVIEVYKWFPEQQDDPCLLHLNLFVLLDILILKVNKFNKDATLFENRLIADMNSCELNA
TIHKYTPLVVMNTRVLIGPLVTQFRIMCQTLKMCIDINPADLRSIPNFQLPVHLKSALMKY
DCVVTYPYFIQHLKWVVPAGAPVPRWKSLIKIFNPMMWFSVVTTFITGSAISWLLLNQSH
HSKTYISALLDTLLTYVVAGISARYKGTVASTFFLLWLFYCLIINTAYQSALISFLADPGHDQ
PIKTIEELLKSELHLMSRFKLANSKTKELAEINIYEMCKSTGKQCYRRVAQNRDTALLDEE
XLFFLQSNFYDSGTNKFLITNIEENVYTLYFAMAIFTHGCLLFKRMEQLIHRMFCAGFLTKS
MKDVLKFARIQNQEVLNNAPFVITLSHQQGTFYFLMSGLFVSIIVFLIEVIYQSI
>BgerIr476P
ALFIPYREYESHSYISTNDSHVDFLVQSLHQRKDHTLVMLDYHNNPQSLHQKVKLGSYIIIL
SGKVNSYIQMTVEVSEIINKVAGHMSPSGRLLIASTGFPKYKRTKLVGIFFNIIWRMLEISES
IVLLPQLGSTDDDVIEVYKWFPEQQDDPCLLHLNLFVLLDILILKVNKFNKDATLFENRLIA
DMNSCELNATIHKYTPLVVMNTRVLIGPLVTQFRIMCQTLKMCIDINPADLRSIPNFQLPV
HLKSALMKYDCVVTYPYFIQHLKWVVPAGAPVPRWKSLIKIFNPMMWFSVVTTFITGSAI
SWLLLNQSHHSKTYISALLDTLLTYVVAGISARYKGTVASTFFLLWLFYCLIINTAYQSALIS
FLADPGHDQPIKTIEELLKSELHLMSRFKLANSKTKELAEINIYEMCKSTGKQCYRRVAQN
RDTALLDEXLFFLQSNFYDSGTNKFLITNIEENVYTLYFAMAIFTHGCLLFKRMEQLIHRM
FCAGFLTKSMKDVLKFARIQNQEVLNNAPFVITLSHQQGTFYFLMSGLFVSIIVFLIEVIYQS
I
>BgerIr477
MLFNIRLRQIILLSLVLKGDTNPLPLDSVSVVEESLSRCITKIANEYFNKNLPTALFIPYRKY
KSHSYISSNDSHVDFLVKSLHQRIDHSLVMLDYHNNPQSLHQKVKLGSYIIILSGEVSSYIDL
AVEVMLKMYEIVRQIKASGRLLIATTGSPTFKRNDILCKLLGLIWLIFQISEAIVLLPKKGLT
SVDDNDVIEVYKWFSEDQIDPCLLSLNSFVLLDIWILKGNKFSSGNTLFQSDLITDMRGCTL
NLSYSDYPPLVYTDDDVHVHGPLINQINLISDVLNFRIKIFKRNFTDFINMQLPIPVWNGN
NKDFHIYDDCVVTYPYFKQNLKWVVPAGSSVPRWKSLIKIFNPLIWFCVVTTFLVGSTTS
WLLLKQSHQSLTFLSVLLDTLLTYVLAGISDRYKGTIASTFFVFWLFYCLIINTAYQSALISF
LADPGRDDPIKTIEELYKSELHLMSRVSSSNVESEKTEEANHFEDCTNRIHCLNRIAQNRD
TALLDDELLQDLVINKYFDSETNRRLLHTVEEIAYTFYFTIAVYTHGCMIFKRMEQLFHRV
WSAGLIIRYFNDFNEIQRNRYYEVLNDDPYVITLSHVQGCFYFLMCGLFVSIIVFLIEISYWC
V
>BgerIr478P

LNSIWSTZDISEZIVLLPRIRSMDDDVIKVYKWFPDEQDDHCLMLLNRFVLLDIWIQKVNE
FIDDATLFENKLIKNLNNYKLNATTISYPLLFIVMNIGVVLGALVSPLRIMGRTLKMRIHFN
PSGLRSFPNFZLPVRETCVINKELLINQAYVVTYPYFIQHSKWVVPAGAPVPRWKSLIKIFN
PLMWFCVFTTFVIGSTTSWLLLKKSHHSMTYISALLNTLLKYVVAGISDRYKGTVASKFFL
LRLFYCLIINTAYXRIIQSFLNDPGQEQPIRSLEELHKSGFHLISKIVFKNTEVEEFLRINNYE
SCHNNDYCLKKITQNRNAAMLPEYLVNFLIHKFYDSEINKSLISIIDESAYTIYLTMTIYTYG
CLFFKRIEQLVHRIWSAGLIMRFIRHSQFHVRRISPFQQZSACDNNVAFAKWFLFPZSGLFL
PVTVLLIEIMYHAVSICIICHSFP
>BgerIr479
MFFDIGLRQILFLSLVLKGDTNPLPLDSVSTIEESLIKCITKIDNEYFNKDLPTALFIPYREYE
SQSFISTKDSHVDFLVKTLHQRIDHSLVMLDYHNNPQSLHLNVKLGSYIIILSGEVNSYFEL
ALEVVFKIDEIAGKMSASGRILIATTGSPIFERDGIVGGLLGMAWFILQISEAIVLLPNEDVIE
VYKWFPEDQMDPCLLILNQFVLLDIWNLERNEFSSGEKLFHSDLITDMRGCTLNISYRNY
PPLVYTEDDEYVVGPLINHIMLISDILNFRFEIFRTDLSVNDFTNMMLPIPVWDGKIKDIIIH
EYKDCVVTYPYFKQNLKWVVPAGAPIPRWKSLIKIFNPLLWFCVFTTFVIGSTTSWLLLKQ
SHHSMTYISALLDTLLTYVVAGISDRYKGVVASTFFLLWLFYCLIINTAYQSSLISFLAHPGH
DEPIKTIEELHKSGLHLISRVSISNVGFAKLEETCHFDECTNQNHCLTRIAQNRDTALLDDE
IMLGAVIDKYFDAETNRHLLHTIEEIAYPLYLTITVCSHGCLIFKRMEQLLHRVWSAGLIMK
YFKDLYKDQRRNYHEVLNEDPYVITLSHLQGGFYFLICGLFVSIIVFLIEISYWSV
>BgerIr480
MRLRQIIFLSLVLKGYSNPLPLDSVSPVEESLSKCVAEIANEYFNKDLATALFIPYREYESRS
YISTNDSHVDFLVQSLHQRIDHPLVMLDYHNSPQSLHQKVKLGSYIIILSGEVSWYLHIAYE
VFFKIDKVAGQMSHSGRLLIATTSYPKGEKNYIISSLLRQTWLVLQISEAIVLIPKIGSTNPD
VIEVYKWFPEKQYDPCLLFLNRFVLFYIWIMKRDEFFKGKKLYQNDLITDMRGCILNISFN
ELVPLVILVDDMYVAGPVIDQIRIIGHFLNLRFNFYKADRSSDRNMKLPVAVNNGENKDVL
TYDDCIVTYPYFKQNLKWVVPAGAPVPRWKSLIKIFNPLLWLCVVTTFLIGSITSWLLLKE
NQQSLTYISVLLDTLLTYVVAGISDRYKGTVASTFFVLWLFYCLIINTAYQSALISFLADPGQ
DQPIKTIEELHKSGLHLMCRLPISTVETANTSECINPRLCLITIAKNRDSALLEDEVYLSSFT
NYFLDSETNRHLLHTIEENARTFYFTINVHSHGCLIFKRMEQLIHRIWGAGLMIKCFDDISR
MLRRRYYEVLNDDPYVITLSHLQGVFYFMTTGLFVSIIVFLIEISYYHV
>BgerIr481
MFFRGRLFQIILIPLLFKGDYYPFPLDSVSTVEDSLSKCVAEIANEYFNKDLPTALFIPYREY
ESHSYIYTKDSHVDFLVQSLHQRIDHPLVMLDYHNNPQSLFKKVKLGSYIIILSGDVNSYID
MAIDVTVNIYEVARQMSPSGRLLIATTGSPNKEKTNVVGNFFYVIWHVLEISEAIVLLPRIG
SSADNDVIEVYKWFPEEQKDPCLLMLNRFELFDVWMIKKNKFLVNLKLFQNNTIKDMRG
CVLNISIVDYPELVTVKDNELGVGLMSEQLVILFQALNLRMNLNPANLSSHPNLKLPEPVK
IGENNDLLTTNDCIATYPYFIQNLKWLVPAGALVPRWKSLMKIFNPLMWFCVVTTFLIGS
TTSWLLLKENQQSLTYISALLNTLLTYLATGIFDRYKGTVASTFFVLWLFYCLIINTAYQSA
LISFLNCPGQDQPIKSVEELHESGLHLNTKIMFPNTIVEEMLHINNYETCQDNQYCLKMIA
QNRNTAVLLPELQAHFLIRNFYDLNINKYLINTIDENQDTLYLTMATLTHGCLIFNRMEQL
IHRIRSAGLLLEIFDVFSVIIKHNLQNKDPYVINLSHLQGIFYFLTSGLLMSMIVFLIEIMYHTI
YDDI
>BgerIr482
MFFRGRLFQIILIPLLFKGDYYPFPLDSVSTVEDSLSKCVAEIANEYFNKDLPTALFIPYREY
ESHSYIYTKDSHVDFLVQSLHQRIDHPLVMLDYHNNPQSLFKKVKLGSYIIILSGDVNSYID
MAIDVTVNIYEVARQMSPSGRLLIATTGSPNKEKTNVVGNFFYVIWHVLEISEAIVLLPRIG

SSADNDVIEVYKWFPEEQKDPCLLMLNRFELFDVWMIKKNKFLVNLKLFQNNTIKDMRG
CVLNISIVDYPELVTVKDNELGVGLMSEQLVILFQALNLRMNLNPANLSSHPNLKLPEPVK
IGENNDLLTTNDCIATYPYFIQNLKWLVPAGALVPRWKSLMKIFNPLMWFCVVTTFLIGS
TTSWLLLKENQQSLTYISALLNTLLTYLATGIFDRYKGTVASTFFVLWLFYCLIINTAYQSA
LISFLNCPGQDQPIKSVEELHESGLHLNTKIMFPNTIVEEMLHINNYETCQDNQYCLKMIA
QNRNTAVLLPELQAHFLIRNFYDLNINKYLINTIDENQDTLYLTMATLTHGCLIFNRMEQL
IHRIRSAGLLLEIFDVFSVIIKHNLQNKDPYVINLSHLQGIFYFLTSGLLMSMIVFLIEIMYHTI
YDDI
>BgerIr483P
MLFESRFCQIIFLPLLIKGKTHSLFDSLTPVEESLSKCVAEIANEYFNKDLPTALFIPYRKYET
HSYITTKDSHVDFLVQSLHQRIDNSLVMMDYHNNPQSLHZKVKLGSYIIILSGDVSSYIDMA
IDVILKIYEVARQMSPSGRLLIATTGSPKWERDDLMSELSLTWRILQISEAVVLLPRRAPTH
ANQDDFIGVYKWFPEDQDDPCVVYLNQFVLLDIWVIKVNEFIENTTLFENNLITDMNNCE
LNATVTXFPSLVLMNTEVLSGPLLTQLHIICETLNMRINFNPSGLRSIPNLELPVHVQNVK
NKRFLVNHECVVTYPYFLQHLKWVVPAGAPVPRWKSLIKIFNPLMWFCVITTFLIGSTTS
WLLLRDNQQSLTYISALLDTLLTYVVAGISDRYKGSVASTFFMLWLFYCLIINTAYQSALIS
FLADPGHDQPIKTIEELRKSGLHLMSGILITNADTEEMVZINRYEICDDTILCLGIIARNRNT
AMVIDELRAQVMFDDYFDSETNTYLIRTIDERVYTFYYSTAINTHGCLLFKRIEQLIHRTW
SAGLIIRVILHFQMVVRRFNLDLLNNNPYVMTLSHLQGGFFFLTSGMFLSLIVFLIEIMYRTI
SNPM
>BgerIr484C
MVFDGSLCLITLLLLVLKGGINPLPLDSVSPLEDSLSRCIAKIANKYFNRDLPTALFFPYRKY
ESHSYISTNDSHVDFLVQSLHQRIDHPLLMLDYHNNPQSLGHKVKLGSYIIILSGKVSSYIE
MAFEVFETIYNVAGHMSPSGRLIIATTGSPKWKRPNLVGHFFNMIWHSLQISESIVLLPRIR
STDDDVIEVYKWFPEEQDDPCLLLLNRLALFNIWILKQNAFILDATLFENRLITDMNNCKL
NATIYNYAPLIIMNNRIISGSFVTQFGIILETLNIRIHFSPASLTSNFELPVRVTYVKDEELLR
NPECVVTYPYFIQQLQWMVPAGAPVPRWMSLIKIFNPLMWFCVFTTFLIGSTTSWLLLKQ
SHQSLTYISALLDTLQTYVVAGISHRYKGTVASTFFLLWLFYCLIINTAYQSALISF
>BgerIr485
MSLVRRFCEIIFLPLLVMGGIPPLSLDSVSSLEDSLSRSIAKIANEYFNRDLPTALFIPYRKYE
SHTYISTNDSHVDFLVQSLHQRIDHPLLMLDYHNNPQSLGHKVKLGSYIIILSGKVSSYIEM
AFEVFETIYNVAGHMSPSGRLIIATTGSPKWKRPNLVGHFFNMIWRSLQISESIVLLPRIRS
TDDDVIEIYKWFPEEQGDLCLLLLNRFVLFNIWILKLNEFILNATLFENRLITDMNNSELN
AAIHNYAPLVLMDNRVMYGPLVYQFGIMCETLNIHIHFNPVDVRSISNFVLPVHVTYVKDE
ELLRNRECVITYPYFIQQLQWVVPAGTPVPRWKSLIKIFNPFMWFCVFTTFLIGSITSWLL
VKQSHQSLTYISALLDTLQTYVVAGISDRFKGTVASTFFLLWLFYCLIINTAYQSALISFLAD
PGHDQPIKTIEELHKSGLNLMSRFKFKNPKTKELAEINTYEMCNKGKQCFIRVAQNRDTA
LLDEEQNTFFLLRQFYDLGTKKFLITNIEESVYTLYFAIAVYTHGCLLYKRMEQLIHRLFCA
GLLTKFSNELGLIAKIQKQEVLNNAPFVITMSHLQGSFYFLMSGLFVSIIVFLIEVMYHSI
>BgerIr486P
MNMVFDGRLCQIIILLLVLKGGFNVLPLDSVSIIEESLSRCIAKIANEYFNRNLPTALFIPYRK
YKSHSYISTNDSHVDFLVQSLHQRIDHPLVMLNYHNNPQSLGRKVKLGSYIIILSGEVSSYIE
MAFEVFLTINKVAGHMSPSGRLIIATTGSPKWKRKLEGDFFNMAWLALEISESIVLLPRIGS
TDDDVIEVYKWFPEEQVDPCLLLLNRFILFNIWIMKVNKFLYDATLFEDKLITDMNNCEL
NVTINNYAPLVLMNTRVMYGPLVTQFGIMCETLKIRKHFNPVDLRSIPNDKLPVRVTYVK
DTELFKSRECVVTYPYFIQHLKWVVPAGAPVPRWKSLIKIFNPLMWFCVFTTFLIGSTTS

WLLLKHNHQSTTYISVLLDTLLTYVVAGISDRYKGALASTFFLLWLFYCLIINTAYQSALISF
LADPGHDQPIKTIEELHKSZLYLMSKHQFKNPETKELAEILIYKMCNTHMQYYRRVAQNR
DTAFLEDEQINFFWLHYLYDSGTKKFLITNIEESVYTSYFAIAIYTHGCLLFKRIEQLIHRMF
CAGLLTRVMNEIVEIAKIRNQEILTNAPFVITLSHLQSSFYFLVSGLFVSIIVFLIEIIYQSI
>BgerIr487P
MFFDIKLRQIIFLCLVLKDDTNPLPLDSVSTVEESLSKCISKIANEYFNRDLPTALFIPYKEYE
SHSHISTNYSHVDFLVQSLHQRIDHSLVMLDYQNNPHSLHQKVKLGSYIIILSGEVSSYIEM
AIYVILKIYEVAREMSPSGRLLIASTGSPKRERTKLVDTFFNMIWRVLEISESVVLLPXDCVA
TYSYLTQKLIWIVXPAGASVPRWKSLIKIFNPLMWFCVVTTFLIGFTTSLLLLKQSHQSLTY
ISAFLYILLTYVVAGISDRYKGTVASTFFVLWLFYCLIINTAYQSALISFLNDPGQEQPIRSVK
ELQDSDZHLMSNIVFRNKEVEYMLQSSNYETCDDNLYCLKIIIQNRNIALLVPEQYVKMLF
GEFYDSDMNKYLISTIDESAYTMYLTISINTHGCLLFKRMEQPIHRIWSAGLLLHILDCLYL
FVDVLQNNPYEIKLSHLQGAFYFLTKGLLVSVIVFLIETMYHTL
>BgerIr488
MIIRGRLCHIIFIHLLFKGGYYPLPLDSVSPFEDSLSRCIAKIANEYFNNDLPTALFIPYREYE
SHSYISTNDSHVDFLVKSLHQRIDHSLVMLNYHNNPQSLYQKVKLGSYIIVLSGEDISSYID
MAIEVFVVIYEVARQMSPSGRLLIATTGSRNKKGIKLAGAFFNMLWRELQISEAIVLVPGLI
ADHDIFEVYKWFPEDQDDPCLLILNRFELFDIWFIDMNKFIFNMKLFQNNIITDMRGCVL
NISISDYPTLATVTDNELEVGLMANQLVILFEALNLRMNLNPANLSSDPNMKLPELVTIEK
SNDLLINDDCVATYSYFTHKLKWLVPAGAPVPRWKSLIKIFNPLMWFCVVTTFFIGSTTP
WLLLKQNRQSLTYISALLDTLLMYLTTGISDRYKGTVASTFFLLWLFYCLIINTAYQSALISF
LNDPGQDQPIRSVEELHESGLHLISKVAFPNSEVEEILQINNYETCDSNQYCLKMIAQNRN
TAMLLPEEFADFFIQYYYDSEMNKYLITIIDKSEYALYLTMASFTHGCLIFNRMEQLIHRIW
SAGLLVELFDVVYMSVFTMEDIPQSNDPYVINLSHLQGAFYFLTSGIFVSLIVFQIEIMYQTI
>BgerIr489
MMLARRFCEIIFLPLLIKGGTYPLPLDSVSPVEDSLSRCVAKIANEYFNRNLPTALFIPYRKY
ESQTYISNNSSHVEFLVQSLHQKIENSLVMLDYHNNPQSLHQKVKLGSYIIILSGDVSSYFD
MAYTVIWKMIVVAGHMTPSGRLLIATTGSPNKERPLLIFQLFNMIWKALQISEAIVLLPGIG
SVAAAGDVIEIYKWFPDEQDDPCLLFLNQFKMVDYWITEMNKFLYNFELFQINAITDMRE
CDFNISVKVFHPLTKVEGNKLGFGFMNYQLLILLQSLNLRMNVNPGNFNSVPNFNLPELLI
FNGDEQFLTNDDCVAIYPYFIQNLKWIVPAGALVPRWKSLIKIFNPLMWLCVVTTFLIGST
TSWLLLKQNQNSLTFISALLDTLLTYVAAGINDRYKGTVASTFFLLWLFYCLILNTAYQSA
LISFLNNPGQEQPIRSLEELQESGLHLISKIMFPDAQTEEIRHINSNYEKCDDCYKMIAQNR
NTALLVPEYHANYGVFKFYDSEINTSLVGIIDETFYTFYLTISSYTQGCLFFNRMEQLVHRT
WSAGLIMRFIQDYKFQERKTRLDLFNKDPYVITLSHLQGGFFFLISGLFLSVTVFLIEIMYH
AV
>BgerIr490
MSLTDRRFQIIFLSLLLNGGTNPLPIDSVSPVEDSLSRCIAKIANEYFNRDLPTALFIPYREYE
SHFFISTNDSHVDLLVKSLHQRIDNSLVMLDYHNNPQSLHRKVKLGSYIIILSGEVSSYISMA
VEIILKIYEVAEHMTPSGSLLIATTTFPNKERTLLLWQFFNMIWKVLQISEAIVLLPGIGSVA
AEDVIALYKWFPDEQDDPCLLFLNRFKLVDYYGIIESNKFLYNFELFQNSAITDMRGCVFN
ISVELLNPFTTLNGNKLGFGLMDDQLIILLEALNLRINVNPINYNPVPNLKLPEPLIFGGDD
HLLIKKDCVATYPYFILNLNWLVPASAPVPRWKSLIKIFNPLMWLCVVTTFISGSMTSWLL
LKKIHQSMTYISALLDTLLTYVAVGISHRYKGTVATTFFLLWLFYCLIINTAYKSALISFLNY
PGQEQPISSIKELQKSGLHLISLIMFPNAEVEEILQINNYDTCYNCLEMMVQNRNTAVLLPE

YLAKFAMHNFYDSGINKHLISIIDESAYTFYLTIASYSHGCLFFKRIEQLVHRTWSAGLIMRF
MQYMQFHVRRVGLDLFNNDPYVISLSHLQGGFFFLISGLFLSFTVFLIEIMYFAV
>BgerIr491P
MLLVRRICEIIFLPLLIQGRMQPLPLDSVSPLEDSLSRCIAKIANEYFNRELPTALFIPYREYE
SHSYISTNYSHVDYLVQSLHQRIENSLVMLDYHNNPQSLQRKVKLGSYIIILSGEVSSYISMA
VEIILKIYEVAEHMTPSGSLLIATTTFPNKERTLLLWQFFNMIWKVLQISEAIVLLPGIGSVA
AEDVIALYKLSAVLKXCLLFLNRFKLVDYYGIIESNKFLYNFELFQNNTITDMRGCVFNISV
QWLPLFTTLNGNKLGPGLMNDQLLILLEALNLRMNVNPRNFSSVPNFRLPDFVTFDENN
HYMINIDCVATYPYFNEILKWLVPAGAPVPRWKSLVKIFNPLMWLCVVSTFLIGSTTSWL
LLKQSQQSLTYISALLDTLLTYVAAGISDRYKGTVASTFFLLWLFYCLIINTAYQSALISFLN
DPGQEQPIRSLEKLHESGLHLISRVKFNNAEAEEIQQINNNYEICDNCLEMMVQNRNTAVL
FPEYLAKFAMHNFYDSGINKHLISIIDESFYTFYLTISSNTHGCLFFKRMEQLVHRIWSAGLI
MRFIQIVEVRERRISFDLFNKDPYVITLSHLQGGFFFLISGLFLSFTVFLIEVMYHAV
>BgerIr492
MMLARRFCEIIFLPLLVKGGTYPLPLDSVSPVEDSLSRCIAKIANEYFNRDLPTALFIPLREY
ESHSYISNNNSHVDFLVKSLHQRIDHSLVMLDYHNNPQSLHQKVKLGSYVILLSGDVNSYF
EMAHKVIFKISEVAGHMSPSGRFLIATTRSPNKERTTLLWQFFNMIWKILQISEAIVLLPGI
GAAAAEDVIVIYKWFPEEQDDTCLLFLNRFKMVDYWITEISKFLYNFELFQNNTITDMRG
CVFNISVERFHPLTEVEGNKLGFGLMNDQLLFLLQGLNLRMNVNPRKFNPVPNLKLPDLL
IFDGENHLLINNDCVATYPYFIQNLKWLVPAGAPVPRWKSLIKIFNPLMWVCVVTTFLIGS
TTSWLLLKQNQQSLTYISALLDTLLTYVAAGISDRYKGTVASTFFLLWLFYCLIINTAYQSA
LISFLNNPGQEQPIKSFEDLQESGLHLISLIMFPDAETEEIQYINSNYEKCDDCYKMIAQNR
NTAVLLPEYHANFAMRHFYDSVINTFLIRILEESMYTFYLTMSNYTHGCLFFKRMQELVH
RIWSAGLIMRYIQHIQFHARRIGLEFFNNDPFVITLSHLQGGFFFLISGLFMSVTVFLIEITY
HAV
>BgerIr493
MSLADRCFQIISLSLVLKVGTNPHPLDSVSPVEDSLSRCIAKVANEYFNRDFPTALFIPYREY
ESHSYISTNYSHVDFLVKSLHQRIDHSLVMLDYHNNPQSLQQKVKLRSYVIILSGEVSLYYN
MAFKVIFEIYEIAKNMSPSGRLLIATTGSPNKERTLLLWQFFNMIWKILQIFEAIILLPEIES
VAAEDVIALYKWFPDEQDDPCQLFLNRFITVVYWITEISIFLYNFELFQNNTITDMRGCVF
NITVERFHPLTEVEGNKLGFGLMNYQLLILLKALNLRMNVNPRNFSSVPNLKLPDLLIFDG
DNHLLLNNDCVATYPYFIENLKWLVPAGAPVPRWKRLMKLFNPLMWFCVVTTFISGSITS
WLLLKQSHQSLTYISALLDTLLTYVAAGVSDRYKGTVASTFFLLWLFYCLFINTAYQSALIS
FLNDPGQEQPIKSFKELQNSGLHLISLIMFPNAEVEEIQQINNNYEKCDNCLEMIAQNRNT
AVLLPEYHVNFAIFQFYDSEINTFLIRIINESMYTLYFTMSSFTHGCLFFKRMEQVVHRTWS
AGLIMRFIQHIQFHVRRTGLEFFNSDPYVITLSHLQGGFFFLTSGLFLSVTVFLSEILFHAF
>BgerIr494
MSLADRCFQIISLSLVLKVGTNPHPLDSVTPVEDSLSRCIAKVANEYFNRDFPTALFIPYRE
YESHSYISTNYSHVDFLVKSLHQRIDHSLVMLDYHNNPQSLQQKVKLRSYVIILSGEVSLYY
NMAFKVIFEIYEIAKNMSPSGRLLIATTGSPNKERTLLLWQFFNMIWKILQIFEAIILLPEIE
SVAAEDVIALYKWFPDEQDDPCQLFLNRFITVVYWITEISIFLYNFELFQNNTITDMRGCV
FNITVERFHPLTEVEGNKLGFGLMNYQLLILLKALNLRMNVNPRNFSSVPNLKLPDLLIFD
GDNHLLLNNDCVATYPYFIENLKWLVPAGAPVPRWKRLMKLFNPLMWFCVVTTFISGSI
TSWLLLKQSHQSLTYISALLDTLLTYVAAGVSDRYKGTVASTFFLLWLFYCLFINTAYQSAL
ISFLNDPGQEQPIKSFKELQNSGLHLISLIMFPNAEVEEIQQINNNYEKCDNCLEMIAQNRN
TAVLLPEYHVNFAIFQFYDSEINTFLIRIINESMYTLYFTMSSFTHGCLFFKRMEQVVHRT

WSSGLIMRFIQHIQFNVRRTGLEFFNSDPYVITLSHLQGGFFFLTSGLFLSVTVFLLEIMYH
AV
>BgerIr495
MKMMLGRRFCEIIFLPLLIKGGTHPLPLDLVSPVEENLSRCIAKIANKYFNRDLPTALFIPY
REYESHSYISTNDSHVDYLVKSLHQRIDHSLVMLDYHNNPQYLDQKVKLGSYIIILSGDVSS
YFNMAHKVIWNIYDVAGHMTPSGRLIIATTWSPNKERTRLLWKLFNMVWKILQISEAIVL
LPGIRSVAAEDMIAIYKWFPGEQDDPCLLFLNRFIMVNYWMTVINKFLYNFELFQNSTITD
MRGCVFNITVRDFPPLTAVKGNKLGFGFMNYQLRILLKALNLRMNVNPRNSNSVPNFKLP
AHLIFSGHNHLHDCVATYPYNIQNLIWLVPAGAPVPRWKSLIKIFNPLMWLCIVSTFFIGS
TTSWLLLKQSHQSLTYISALLDTLLTYVAAGISDRYKGTVASTFFLLWLFYCLIINTAYQSA
LISFLNDPGQEQPIRSFEELQESGLHLISNIMFPNAKVEEIQQINNYEGCDNCLEMMALNRT
TAVLLPEYHAKFAMHRYYNSDINTFLISIIDESFYTFYFTMSSYTHGCLFFKRMEQIVHRIW
SAGLTTRYVQLIQFTVKMYYIDLFNSNPYVITLSHLQGGFFFLISGLFLSFTVFLIEIMYHAV
>BgerIr496
MMLARRFSEIIFLPLLIKGGTHPLSLDLVSPVEDSLSRCIAKIANEYFNRDLPTALFIPYRKY
ESHSYIFTNGSHMDFLVKSLHQRVNHPLVMLDYHNNPQYLERNLKLGSYIIVLSGEVSSYL
HMAHKVFSKIYEVARHMSPSGRLLIATTGSPNKERNLLLWDFFNMIWKNLQIFEAIVLVP
RIESFAAGDVIAIYKWFPGEQDDPCLLFLNRFTIADYWITEISKFLYNFELFQNNTITDMRG
CVFNISVEIFYPLTEVKGNKFGYGLMNHQLVILLEALNLRMNVNPRNFNPVPNFKLPDPLI
FDGDDHLLINYDCVATYPYFMQHFKWLVPAGAPVPRWKSLIKIFNPLMWLCVVTTFLIGS
TTSWLLLKQNQKSVTFISALLDTLLTYVAAGISYRYKGTVASTFFLLWLLYCLIINTAYQSA
LISFLNDPGHDEPIKTIEELHKSGLHLTSLFKFRNTEVEEIKQLDNYEICDNCFKVMAQNR
NTAVLLPEYYASFMIYMSYDSDINTFLITIIDESAYTFYLTISSYTHGCLFFKRMEQLVHRTF
SSGLTTRYIELVRFTEKIYYRDLLKKDPYVITLSHLQGGFFFLISGLFLSITVFLIEVMYHAV
>BgerIr497P
MLGRRFCEIIFLPLLIKGRTYLVPLETALPLEDSLSRCIAKFANEYFNRELPTALFIPYRKYE
PHSYISTNDSHVDFLVQSLHQRINHPLVMFDYNNNPQSLHRKVKLGSYVIILSGEVSSYIDM
AYKVIVTIYEVAGHMSSSGRLLIAMTGSPNKERSLLLSLLFTMIWKIFEIFEAIVLLPGIGSVA
AEDLIVIYKWFPDEQDDPCLLFLDLFIMVNYWITEINEFLYKSELFQNNTITVMRGCVFNIS
VERFHPFTTLNGNKLGFGLILHKALNLRINVNPRNFSSVPNFKLPDLLIFDGDNHLXLATY
PYFMQNLKWLVPAGAPVPRWKSLIKIFNPLMWLCVVTTFLIGSTTSWLLLKQSNQSLTYI
SALLDTLLTYVVAGISDRYKGTMASTFFLLWMFYCLIINTAYQSALISFLADPGHDEPIKIIE
ELHKSGLHLISLFWFRNTEIEEIQQINNYDKCYNCFEMMTQNRNAAVLAPEYYASFAISKF
YDSEINTFLISTIDESAYTFYLTISSZTHGCLFFKRMKQLVHRTWSAGLISRYIQRVQFIAKR
YSPDLFNNDPZVITLSHLQGGFFFLVSGLFLSVTVFLIEIIYHAL
>BgerIr498P
MFLARRFCEIIFLPLLIKGGTYPLPLDSVSPLEDTLSRCIAKIANEYFNRDLPTALCIPYREYE
SHSYISTNLSHVDFFVQSLHQRIENSLVMLDYHNNXPQSVHQKVKLGSYIIILSGKVSSYID
MAIYVILKIZEVAKEMSPSZRLLIATTRFPNWGRKNLVSTLLRQTCLILEISEAIVLLPRIGST
DDGVIEIYKWFPEQQDDPCLITLNRFALFNIWKMKVNDFIDEATLYENRFITDMNNCDVI
ATVHKYTPIVVMETGEIYGPLVTQFRIMCETFKMGIHVNPPGLRSISNFELPVRVXVVTYPY
FIQQLQWVVPAGAPVPRWKSLIKIFNPLMWLCVFTTFLIGSTTSWLLLKQSHQSLTYISAL
LDTLLTYVVAGISDRYKGTVASTFFLLWLFYCLIINTAYQSALISFLADPGNDQPIETIEELH
KSGLHLMTRFKSNNPQTKELSEINSYEMCKNKMQCYRRVAENRDTAILXFMLSYFPDSGN
RKFLITNVEESVYILYTAMAIYTHGCLLFKRMEQLNHRMFCAGLLTKAMNDVLDLARIQN
LEVLSNAPFVITLSHLQGSFYFLMSGLFVSIIVFFIEVMYNSI

>BgerIr499P
RNLALADRRFQIIFLSLVLRVGTNPLPHDSLSPVEDSLSRCIANIANEYFNKDLPTALFIPNR
EYESHSYIFTNDSHVDYLVQSLHQRIDHSLVMLDYQNNPLYLDQKVRLGSYIIILSRDVSSY
FDMARRVISKIYLVAGHMSPSGRLLIATTGSPNKERTLLLWRFFTMIWNCLQIFEAIVLLP
GKGSVAAEDVIEIYKWFPEHQDDPCLLTLNRFTIADHWITEINKFLYNFELFQNSTIRDMR
GCVFNISVEWVHPFTTLNGNKLGFGLMDDQLLILLKALNLRINVNPRNFSSVPNFKLPDFV
IFDENNDYMINIDCVATYPYFIENLKWLVPAGAPVPRWKSLIKIFNPLMWFCVFSTFLIGS
TTSWLLLKQNQKSLTFISALLDILLTYVAAGISDRYKGTVASTFFLLWLFYCLIINTAYQSAL
ISFLNDPGQEQPLRSVEELQESGLHLTSIFMFQNAKVEEIQQINNYEGCDNCLEIMVQNRN
TAVLLPEYLARFAIHNFYDSGINKHLITIIDESAYTFYLTMSSYTHGCLFFKRMEQLVHRIW
SAGLTTRYIQVIQFTAKIYYLDLFNSDPYVITLSHLQGGFFFLISGLFLSFTVFLIEIMYHAL
>BgerIr500P
MSLADRRFQIIFLSLVLRVGTIPLPHDSVSSVENSLSKCIAKIANEYFNRDLPTALFIPFREH
ESHSFIYTNDSHVNFLVQSLHQZIDHSLVMLDYHNNPKSLHQTXKLGSYVIILSGEVSLYFD
LGFEVIWKIYEVAXRLLIATTGSPDKESILFAWKFFNMIWEVLKMSEVIVLLPRIRSIAEDRI
IEVYKWCPEEQNDPCLLLLNRFRIFSIPIAVNNASLNNLKLFQNSIITNMRGCFFNISVDLN
GNKLGIGLMLYQFLILLEALNLRMNLNPANLSSLSNWGLPEMVTIDKNNDLPINEDCVAT
YPYFILHLKWPVPAGASVPRWKSLIKIFNPLIWFCVVMTFISGSTTSWLLLKQSHQSLTYIS
ALFDTLLTYVAAGISDRYKGTVASTFFVLWLFNCLINTAYQSALISFLNDPGQDEPIRIIEEL
HESGLHLISKVRFQNVEAEELLEXDYCLKNIVKNRNFAMSLPEQQANSVISHFYDSEINKYL
ISTIDESAHTLYLTMATFTHGCLMFRRMEQLVHRICSAGIIMRHISGIQFIVRRGRINIFNND
PYVITLSHLQGGFYFLMSGIFVSLIVFLIEVMYHSI
>BgerIr501P
MFFRGTLFPRMFLPLQLKGGTTSFRLDSVTPIEDSLSKCXPKIANEYFNRDLPTALLVPYR
NYKSHSYILTNDSHVDFLVHSHHZRIDHPLVILDYHNDTLSLQQKVKLGSHIIILSGEVSSYA
DMAFGVLLRILKVTEQIKSPGRLIIATTTSPNRGZDNLVSKLFHTAWLVQRISEVIVLLPKL
ESTTSFDKIEVYKWFPEQQDDPCLLHLNKFVVVDIWAIKINEFFNNEZLFPNIMIRYMRGC
VLNITTFDYAPLVMTIYNGHVIGPLIDQIYIICDALNLRINLVQPDISSVPDMMLPAPVKNM
ESNDLYECVATYPYFIQHLNGLFRRXIASLFFILWLFYCLIISTAYQSALISFLANPGHDQPIK
SIEELHKSELNLMSVSVEGISQLNDYDRCHSYLLCVKMVAQNRNTALLNEQYLIAYLYETF
TFDSGTNRYLIHTIEESAYTLYLTIAIYSIGCMLFKRMEQLIHRIWSAGLIIZNFSYFHQQEIS
VNYRLLNNNPYVITLSHLQGXYFLINGLFVSVIVFLIEIIYHAM
>BgerIr502P
AIESFSKCIIEIANKYFNKDLPTALFTSNRHLYSATXISSNHSHVEFLVQLLHQQMHHSLVN
FGYHNKIQTLRPKLKLGSYIFVLLGKVDSYVYMTNKILKAVEQAGAIFQSGRVLVALTDSPK
SYTRRYNLILKLFNSVWKILRIASAIAVLPIVKSTSFVNVFHVWTWIPEKQKDPCIRALSHIE
LVDSWIVEINYFLKNASLFANEQLWDMRGCVLNINIHNYSPLVXQNCVGTYPFHIMHLKW
LVPAGAPVPRWKSLTAIFNPFMWFCVTMAFAVGSITSWLLLKNSRKENQQSLGFLSVLTS
TLLTYLTVGIPDRYKGTEATTFFVLWLFYCLIINSAYQSALISFLAEPGEEKPIQTIDELLQSG
LHLMSRFGVLELKVMEDYDKCHDEVYCIKMVSQNRDTALLDDEHHAVRVLKQFYNSEIN
RKTVIFIEEVVYTFYFTIGTYNHGYLLFRRMENLMHRLSNAGLISRHINIYNNMFKIYYSDR
VNHNPYVIGLSHLQGAFYLLITGLFISFIVFFIEITYYRLQSM
>BgerIr503P
MFLFSVYLTPIVRLLFVIISCANFKTSDTMYEYLMEKNLSECIVSVAGKYFNKDLPTAVIIPT
KSFVDAANISINSNSNKELILKELNQRMVNTIVNFDNRMNYQLTGSYNKPGSLIVILAGERE
FLFNLVENITKAIFDHALSPSAKLIIATTNIANTSKEQVWLAKSLLQMFWKSVKIANAVVVI

PILKGHSEMQKSNFDGFGIFSWSPGMQHDPCFKXLDRVDFVDIWLLGTRQFLHNYNLFPP
IHLTDLRGCKLQTTVEKYFPYAYVWYDTSVKGVLIHALRIIFKFLNIKLQFVKPKSGLYPDF
HVPYFYGVNSKPLDCTVLYPLHQEDMKWYTATGTPVPRWKSFIKLFNPLMWTFVVATFI
VGVATSWLLLNVSTYKNKKPVDLSTVALDVLLTYLAVGIHNKYRGTVAHSFFINWLMFCL
IINTAYQSSLIRFLADPGQYEPIKTIDELFKSDLQLFVQPSHKYKKIKNLHIANNYEMCYNIT
KCIEMVAEGRSAILLPEFATFYIQVLTLDKERKKPRVIPIDEIVYSVHLGVYVRSLNCLIYNR
LEELMHRLSSAGINSRFLKLKRSRMAYYYTTLTTDHPFVFTVGHLQGAFYILLIGIFIAFAVF
IFENIQGGRKVTWY
>BgerIr504
MNWNNFIIVQSIFWTSSSIMVPNIDNTFEQSLSECILYIAKTYFGKDAPIAVQTPSTWISEFK
NLKGVSFESYGDILLQALNEKSDISLLALDYLDGEELLQKNKMYPGAYIIVLTGEYDFMMF
MQYQMISRLLIDARNKSAMMMIVSTHIPKHPLDLQFISGNLLQSAWQLFGCHQAIVMLPD
VQFTKSKVKELKYFQVYTWFPESQHDPCIRNLNKVNLLDHWIVKEQKFSIGINIFPDKKIN
NMRNCTATTKVYSMYPFAVIAPPRAWGSMLISLQMAVQTLNLRLFFIHRNSTRKTDLQLP
SPYDPNLINDECTLTYPNFQTNIKWYVPGGISIPRWKSLTKIFSPLLWVAVCVTFVMGTAT
LRILQFTNKNENGSNTLSALLLNTLAMSLGQGVIANYTGAPRKMVFLLWLFYFLVINTAY
QSALVGFLTNPGKYPPIQSLVELFDSNLHLRTSIIVEGVASEKRRQWSKYDICHNELVCLEQ
VATEKATAVLIEEPLAEWVRAKLLAKPLRDEFTSIDNSEDIVYFGISIDELGCLLHKRLDDK
FHRLFSSGIIHKLSDEYNIRYASKSQDFRVLTSFHLQGSFFILIFGILLSTSVFIYELVFHS
>BgerIr505P
MDLNAILVILTIFGTTCNFNLISNLNNAFEQSLSECIMNIAKNFFRKDLPMAVQIPSTWYAS
FKNIPDRSVQAYGDIVLKALNEGTDIPLLALDYLEETELNQPNMFIPGSYVIVLTGNYETMI
MLALRMITRLQVDLRNPSASMMIVSTDIPTSVHEQYFVAGNLLQEAWQRLGIADVIIIEPG
VQSSKSRRNKVQQFDIFTWFPEHQRKPCITNLNNVSFLDSWMVKPQIFYRGMNLYPIKXI
VDMRNCFIYVRIISLPPFAEINETTPWGSMIDIVKIIAKRLNFHVMFRKEKHDDMEFSLPTV
YDHNFKPDECMLTYPYHSAEIKWFVPPGESIPRWQSLVKIFSPLLWIIVIIAFILSVLIFWFM
RYSNDDGNVTNNFSAVVLDTLLMSLGYAVPDKYKGPFKKIIFTLWLFYCLIINNAYQSSLV
GFLTNPGEYPTIGSLEELYNSNLKLVTSIQVLGDANDERKKWNNYEICKNSFKCLAQVAIK
RDIAVLTDEPTGDKTRAILSMRSQDFRSIEEYVDISYFGIHIYRLNCLLHGPMTENLYRLYS
AGIVGKFVDENKIRYIFF
>BgerIr506
MHFKHLLAFLFLQNYFSGNCSIVSVERSQPMLEKQLALCAVRIIEKYLPQNAPVIIQTPNIW
KNSAVEIFKTSADETELLLKVMYEALANDFVIVGYTKDNETKETALQFPVSIVMLINGDQY
EHMVYMVSQYLLSISAIASIRESKIMVITTKMPVDDHIQFFMASLFLTLMWEVLYASDTIFL
IPDSRWRSEYETEVPAVDVFGWTPEEQNDPCLKEINKVSHLDCWWSVEVGILFNEDLFPS
KDIKVMKGCTFVVQTFGCPPLMIEDPLRTSVREGLYAKMIQALAEASNFEVVYKEHDFGQI
PDLSGPHVMDATRIPGTCGFPYPHFKDDIVWFVPAGAPFPTWYSLVKIFHPTMWLSVVA
TYILGCITYWGISKLELLVEGLKRELRLSLIFTNIFLSQLPGGFRYEFRNPSSLIFVPLWLIYCL
QIYTAYQSSLIGFLANPGHFSPIKSTEELFASDLELNTAIVLSSDESWDGFDSYPLCIEAQCF
ERIAEKRNLALLTTRHFSELYIAANHMKNGKPTVIPLEEVRSTQYFNLQNSIGCYMQRRM
DSVFHRLVSGGIVNKWSGDIKSRYIKEYRVKEENADVFVLTMKHLKGVFLFLIIGNLVALM
CFMIELLLPLFYNLFTYKWLHCFKK
>BgerIr507
MHFKHLVAFIFLQIYSGYCSIVSVERSQPMLEKQLALCAVRIIEKYFPQNAPVIIQTPNIWKN
SPVEIFETSADETEMFLKVMYEALANDFVIIGYIKDNETKEPALQFPVSIVMLINEDEYEHM
VYMMSEYLQRILAIANIRESKIIIITTKIPIDDHIQLYMATIFLTLIWKALYASDTIFLIPDSRW

RSEYETEVPAVNVFGWTPEEQNDPCLKELNKVSQLDCWWSDEVGFLINQDLFPSKDITD
MKGCTFVVKTFQNPPFMIKDPLRKSVREGLYAKMIQALAQASNFEVVYKEYDFGQIPDLS
GPHVMNATRIPGTCGFSYPHFKDDVMWFVPAGAPFPTWYSLVKIFHPTMWLSVVATYIL
GCITYWGISKLELLVEELKRELGLSLILTNIFLSQLAGGFRYEFRNPSSLIFFPLWLIYCLQIY
TAYQSSLIGFLANPGHFPPIKSTDELFASDLELNTAILLSTGSLNDENWDGFDSYPLCKEDE
CFERIAEKRDLALLTNRHYSELYITAHHMKNGKPTVVPLEEVRSTQYFTLQNNIGCYMQR
RMDSVFHRLVSGGIVNKWSEDCKSRYIKEYRAKKENVDAFVLTMKHLKGVFLLLIIGNLV
AFLCFMSELLLQLFHKLLFTYKWSPCFKKVWK
>BgerIr508
MHFKHLVAFLFLQNYFSGNCSIVGVDRSQPMLEKQLALCAVRIIEKYLPQNAPVIIQTPNI
WKNSPVEIFKTSADETEMFLKVMYEALANDFVIVGYTKDNETKETVLQFPVSIVMLITED
EYEHMVYMVSEYLKRIVPIASIRESKIIIITTKIPIDDHIQLYMATIFLTLIWKALYASDTIFLI
PDSRWRSEYETEVPAVNVFGWTPEEQNDPCLKELNKVSQLDCWWSDEVGFLINQDLFPS
KDITDMKGCTFVVKTFENPPFMIEDPLRKSVREGLYAKMIQALAQASNFEVVYKEYDFGQI
PDLSGPHVMKATRLPGTCGFSYPHFKDDVVWFVPAGAPFPTWYSLVKIFHPTMWFSVVA
TYILGCITYWGISKLELLVEDLKRELGLSLIFTNIFVSQLAGGFRYEFRNPSSLIFFPLWLIYC
LQIYTAYQSSLIGFLANPGHFPPIKSTDELFASDLELNTAILWNTGSPIVQIWDGFDSYPLC
MEDECFERIAEKRDLALLTKRHYSELYITAHHMKNGKPTVVPLEEVRSTEYFTLQINIGCY
MQRRMDSVFHRLVGGGIVNKWSEDCKSRYIKEYRVKKENVDVFVLTMKHLKGVFLLLIIG
NLVAFLCFMSELLLQLFHKLLFTYKWSPCFKKVWK
>BgerIr509
MPFYFAGCTMLFKHLVGFIFLQNYFSGNCSIVSVELSQPMLEKQLALCAVRIIEKYFPQNAP
VIIQTPNIWKNSPVEIFKTSADETEMFIKMMYEALANDFVIVGYTKDNKTKETALQFPISIV
MLITGDQYEHMVYMVSEYLQRIEAIASIRESKIIVITTKIPVDNRIQFFMASIFLSLIWDVLY
ASDTIFLIPDSRWRAEYETEVPAVNVFGWMPDEQNDPCLRELNRVSHLDCWWSDEVGFL
FNEDLFPSKDITDMKGCTFVVKTFEYTPLMIKDPLRTSVREGLYAKMIQALAEASNFEVVY
KEYDFGQFHDFSGPHLMNATRLPGTCGFSYPYFKDDFVWFVPAGAPFPTWYSLVKIFHPT
MWFSVVATYILGCITYWGISKLEVLVEELKRELGLSLILTNIFVSQLAGGFRYEFRNPSSLIF
FPLWLIYCLQIYTAYQSSLIGFLANPGHFPPIKSTDELFASDLELNTGILLGTESQNDESWD
GFDSYPLCMEPECFERIAEKRDLALLTTRHFSELYIAAYHMENGKPTVIPLEEVRSTGYFNL
QNNIGCYMQRRMDSVFHRLVGGGIVNKWSGDFKSRYIREYRVKKENVDVFVLTMKHLKG
VFLLLIIGNLVALMFFMIEMALKLFYKL
>BgerIr510
MPFYFAGCTMLFKHLVGFIFLQNYFSGNCSIVSVELSQPMLEKQLALCAVRIIEKYFPQNAP
VIIQTPNIWKNSPVEIIKTSADETEMFIKMMYEALANDFVIVGYTKDNKTKETALQFPISIV
MLITGDQYEHMVYMVSEYLQRIETIASIRESKIIVITTKIPVDNRIQFFMASIFLSLIWDVLYA
SDTIFLIPDSRWRAEYETEVPAVNVFGWMPDEQNDPCLRELNRVSHLDCWWSDEVGFLF
NEDLFPSKDITDMKGCTFVVKTFEYTPFMIKDPMRTSVREGLYAKMIQALAEASNFEVVY
KEYDFGQFHDFSGPHLMNATRLPGTCGFSYPYFKDDIVWFVPAGAPFPTWYSLVKIFHPT
MWFSVVATYILGCITYWGISKLELLVEELKREPGLSLILTKIFVSQLAGGFRYEFRNPSSLIF
FPLWLIYCLQIYTAYQSSLIGFLANPGHFPPIKSTDELFASDLELNTAIILGTDSPKSESWDG
FDSYPLCMEPECFERIAEKRDLALLTTRHFSELYIAAYHMENGKPTVIPLEEVRSTGYFNL
QNNIGCYMQRRMDSVFHRLVGGGIVNKWSGDFKSRYINQYRVKKENVDMFVLTMKHLK
GVFLLIIIGNLVAFLCFMIELLLQLFYKL
>BgerIr511

MKMDIPCILLYRMVIFFLLLPSGFGIFQLQNSPNMPENFITDSILHIMSRFLHNNMPIVIQTS
NIWSNFFNESNIPEEDLLLARVNNNSMSHIVLGPPIESFDYMLECKGKPGSAVLVLTDKDM
KQQIDTLYFMMLRLRIDMRNHATKFVIVSNLIPCSREEQMEYSGEFLNFLWNYFRMSDVI
VLISKPKAMRENEDSFDAITWYPNDQADTCLESLDHFIILDTWLSREKRFINGRELFTPKV
IQTMEGCTFKVLLFIWPPFIFLKKTDKGKTLIDGIYIRVLNMISEIMLIRYSFNSYPDKHIGDI
ILPVTEHKFHSDNCDYVYPYFTDDLTFFIPAGNMIPRWQSLFRCFSSNMWCFVIVSYFMG
TLAFLFFEVFDNLKGNRIFYANTVTVFFNTFCSSLGIAMKENFHSMNSYLFLTLWLFYCM
QIYTAYQASLTGFIVNPGEFSPIKTIDQLKESGFGLWQSFRLLDIIETDVDINVSLSCDSTDCF
KILLENPNSAVLTFKTYSEMFVQGYLTSNGRRPLVALEESFLKVYISLITRIGCMINKRFKLII
QRLVIGGFIQKWANTMVEHHWNMYYNNVDSDTVFSFSLNHLQGAFYLLIFGLVISTIAFV
CEFLIQLKAC
>BgerIr512
MILLTKITQILLYCTVWSADGALKLQHEANLYTQLVLEAIVNISDIYLNKDSPVIVLSHRSLC
NTPCHFNFTEKEDEDSIVQFLHESNKWTILTPGNIEKNITNIYVKTGRTSRPMSLIIILNCEE
YNMQLLLLHYMVMSLIHNRSFNPRARCVVVSTSLPSNRDQQNHVLNTFLRLTTYWWIYD
AIVIIPEITSDTSKSDFSLKSSLEIFTKYPYNQQSNCDKEVGKIIFLDRWFLIGNQIGPLRNTN
LFPSKVPKNLWNCGFVFRTSPWPPLVMTPEVDINSSIEYKDGLEIRILNQIAKSTNFKVLYS
QNNSILTQAIFGATWIQGEIGVEYTWPHFTGAVTWFVPRERELPQWLSLIKIFDPLFWLF
VLLAYILGSFTFWILGNCPLNEKELEIKGFSNMTLIFINTLGTVLSESVYGKPRRTLSQIFFIL
WLFYCLQINNAYQSSLIGVLAEPGNLPPIRNVHELIESGIQIGIQSGMQNYLDDNEQKQLKV
QNAIELNDYTKKYCLDKMAYEMNLAVLAGRVGIEFIGYTNYTFNGKPLYVPFHDNVQEGH
MAFRYSIGHLLVVRFNKIIHRLQSAGIIYKWLEEIRRKYGKHFNQNLRGKEFYVLTLSHIEG
AYYLLILGEILAFVVFLTEIIWNFYVLQSKENMGG
>BgerIr513
MCMSSALSDHRLIFCFSFQRIIMNIVWKVVILQFLLTLKLNRSKVVFRNSKTSISDSTWECI
LNIVLRFFPSDLPMVVQTPDVWNTFGYFKANPVINADGDMLLRTLNEKYIDYIFVGPVPNI
GDKMWVDVKPGSTVLLLANGNFEDSYHSLYYMMMRLHVDMRNVGMKIVVVSLLKTLTR
HEQRSKAVVFLQILWKEFSCSDVVVLMPTPRHIYDSKYTYLNAITWFPSEQSDRCLNFLDS
VNILDSWSSNDKSFMNKGNLFPLKSLHSMRDCTLKASAENFLPFSFVYGKVIGGLFSTTLN
GFSEKTGIDIEFNNEEKSEKFAIEYPLITFGPFKLDECEYIYPYFYAEITFFVPSGEPIPKWQS
LIKCLSLKVWILVIISYFLGSFTFYISVNLQNYRSLLQTRTQISQTFLLIFGTFLAVGIKENFQG
LFQRAFFALWLFYCLQISTVYQSEMTAMMVNLGEQAPIRNLKELEKSDLKLMSNLDISRLI
PDVKASKLNISQMKMCSETACFNKISNERNVAVLATRQMAELLILAYHSSSGKKQLVPLEE
NLWKFYPIIKTKTECMLNRNINSISRQFMSMGIYDYFFRQLTFLYTIYVKINQDRWKQNMS
FSLNQFQGTFFLIFLGFIISTFTFLCEIKYSKMLY
>BgerIr514
MMIANKIIFCMNIASAKMFPPEIDTSIADRFADCILNISRRYFELDLPVAVQTSSMYRNLFQ
PGIYDNQLLRVLIKENQFSQLTLGYIKYMEFEIAYSYETGNEMKFGSYLLVVSGKSIREAYD
VSFMMVDRFLVGINSRAKLVIFFTGTSLTFHQQKRLSKKLLQVALIAGFVNFIVIVPSAIQGG
NLQQLNIFGWHPNSQSNICSKKLDTIEQLDTWSFEKRAFLSNADLFPQHTYIDMKGCELHI
IIVFTYPFIYRFPRANQMVMRGSFYNVIPILNNLLNCSIRIGEPDPKYEASVHAVFPVFVHTL
RKNDDCTMIYPYFVEDYFWYVPSPLEIPRWKSLIRAFEPEMWALISLAFISGTLTLYYIQKF
MYLSHSKNDSISLTNQFLSSMKTYLGLSTIVKYKGKTAILTFILWLFYCMVINTAYQSALFG
LIVNPGRYPAIKTLKELDESGLEKVKSYQDYNGEDEVQGQHSLYFNDLPVCAYSNYFCFKK
LSENREVAVLVNNYIGDKAIKEFSVGGAPKVIAVEERYFTMHWTMEINKLACILHRPVETT

LRQLTSAGVLQKWNLQDELRENIKIAARNHTSSSFSLSLNHIYGVFYICMLGFMLAVVVFII
EILIPGFQQVFT
>BgerIr515P
MMIANKIIFCMNIASAKMFPPEIDTSIADRFADCILNISRRYFELDLPVAVQTSSMYRNLFQ
PGIYDNQLLRALIKENQFSQVTLGYIKYMEFQTAYSYETGNKMKFGSYLLVVSGKSIREAYD
LSFMMVDRLLVGINSKAKLVIFFTGTSLTFHQQKRLSKKLLQVALIAGFVNFIVIVPSAIQGA
NIQQLNIFGWNPNSQSNICSEKLDTIEQLDTWSFEKRAFLSNADLFPQHTYIDMKGCELHI
IIVFTYPFIYRFPRANQMVMRGSFYNVIPILNNLLNCSIRLRTPDPKYEASVHAIFPLFVHSL
LKNDDCNMIYPYFVEDYFWYVPSPLEIPRWKSLIRAFEPEMWALVNLAFLSGTLTFYYIQ
KFMHLSNSKNNNSISLINQFLSSMKTYLGLSTEVKFRGKTAILTFILWLFYCMVINTAYQSA
LFGLIVNPGHYPXIKTLKELNESGLKKVKSYYSYIEEDEIQDQHSLYFNDLPVCAPDNYFCF
KKLSENREVAVLVNNYIGDKAIKEFSVGGVPKVIAVQERYFTMHWTMQIIKLACILHRPVE
TTLRQLTSAGLLQKWNIQDALRENIKIAARNHTSSSSSLSLNHIYGVFYICMLGFMLAVVVF
IIEILIPGFQQVFT
>BgerIr516
MMIANKIICCMNIAHAKMFPPEIETSIADRFADCILNISRRYFELDLPVAVQTSSMYRNQFK
TGIYDNQLLRTLIKENQFSQVTLGFLKNMENRIAYSYKTGNGMKYGSYILVVSGKSIREAYD
MSSTMVNRILVGRNSKAKLVILFTGTSLTFHQQERLAKKLLQDALTAGFVNFIVIAPSAIHG
ANLQQLNIFGWDPNSQSNICSRKLDTIEQLDTWIFEKRAFQLNADLFPQRTFIDMKGCKF
LITIMFQYPFIYRFPRGNKMITRGTFYNVISILNNLLNCSIRIGPYDPKSYRIAHAEFPVIINV
KRNNDDCTMIYPYFVEDYYWYVALPLQIPQWKSFIRAFESEMWALVSFAFFSGTLTLYYI
QKFKHLSNSKNNSISLINQFLSSMKTYLGLSTVVKFRGKTAILTFILWLFYCMVINTAYQSA
LFGLIVNPGHYPAIKTLKELDESGLKKVKSYYTAKGINESFHTLYFNDLPVCAPDSYLCFQK
ISENREVAALVDNYIGDKAIQEFSIAGFPKVIAVEERYFTMHWTMEINKLACILHRPVETTL
RQLTSAGVLQKLNLQDDIEDSRRIAAKNHTSLTSSLSMNHLYGVFYICMLGFMVAVVVFNI
EILITGFQ
>BgerIr517
MMIANKIICCMIIAHSKMFSPKIDISIADRFADCILNISRRYFELDLPVAVQTSSMYRNQFKT
GIHDNQLLRTLIKENQFSQVTLGYIKYMDYPITYGYMTGNSVKYGSYLLVVSGKSIKEAYD
MSFLMVTRIIVGKNTKAKLVIVFTGTTLTFHQQKRLAKKLLQDALTAGFVNFIVIVPSAIQG
GNLQQLNIFGWNPNSQSNICSNILDTIEQLDTWSFEKRAFLSNTDLFPQHNFIDMKGCKFL
ITIMLQYPFIYRFPRGNQMIMRGSFYNVIPILNNLLNCSIRIGPLDKKHPRIAHAEFPVIVNT
KQQTDACTIIYPYFSEDYFWYVASPLEIPRWKSLIRAFEPEMWTLVGLAFIFGTLTLYYIQK
FMHLSHSKNDNNITLTNQFLSSMKTYLGLSTIVKYRGKTAILTFILWLFYCMVINTAYQSA
LFGLIVNPGHYPAIKTLKELDESGLEKVKSYYTQKVIRKDHESLYFNNLPVCAHDNYFCFK
KLSENREVAVLVNNYIGDKAIKEFSVGGAPKVIAVQERYFTMHWTMEINKLACIIHRPVET
TLRQLTSAGVLQKWNLQDELRENIKIAARNHTSSSSSLSLNHIYGVFYIFMLGFMLAVVVFI
IEILITGFQQVFS
>BgerIr518
MTFYYFRMNKLLTLMMIANKIICCMNIARVKMFPREIETSIADRFADCILEISRRYFELDLP
VAIQTSSMYRNQFQTGIYDNQLLRTLIKENQFSQVSLGFIKNMEKRTAYSYNTGNEMKYGS
YILVVSGKSIKDAYDLSSMMVDRILVGKNTKAKLVICFTGTTLTFHQQKRIAKKLLQDVLT
AGFVNLIVIVPSAIHGGNLQQLNIFGWDANSQSDICSRKLDNIEQLDTWIFEKRAFELNVDL
FPQRTFTNMKGCKFLVTINLSYPFIYIFRRRNQVIMSGSFYNVIFILNDLLNCSIRIGSYDPKS
SRISHAEFPVFHHTQRKNDDCTMIYTYFVDDYYWYVPSPLEIPRWRSLIRAFEPEMWTLV
SLAFIFGSLTLYCIQKFMHLSHSKNDNSISLTNQFLSSMKTYLGLSTIVKFRGKTAILTFILW

LFYCMVVNTAYQSALFGLIVNPGHYPAIKTLNELDESGLEKVKGYYAQDVVAEGLHSIDFN
DLPLCVNDNYVCFKKISENRKQAVLVSKYVGDKAVKEFSKGGVPKVIPVDERYFTSHWTV
EINKLACILHRPVERVLRQLTSAGLIQKWNLQDDIQENRRIAANNRIAASCSLSLNHLYGVF
YICILGFMVAVVVFIIEILTPGFQQVII
>BgerIr519P
MMIANKIICCMNIAHAKMFPPEIETSIADRFADCILNISRRYFELDLPVAVQTSSMYRNQFK
TGIYDNQLLRTLIKENQFSQVTLGFLKNMENRIAYSYKTGNGMKYGSYILVVSGKSIREAYD
MSSTMVNRILVGRNSKAKLVILFTGTSLTFHQQERLAKKLLQDALTAGFVNFIVIAPSAIHG
ANLQQLNIFGWDPNSQSNICSRKLDTIEQLDTWIFEKRAFQLNADLFPQRTFIDMKGCKF
LITIMFQYPFIYRFPRGNKMITRGTFYNVISILNNLLNCSIRIGPYDPKSYRIAHAEFPVIINV
KRNNDDCTMIYPYFVEDYYWYVASPLQIPQWKSFIRAFESEMWALVSLAFFSGTLTLYYI
QKFKHLSNSKNNSISLINQFLSSMKTYLGLSTVVKFRGKTAILTFILWLFYCMVINTAYQSA
LFGLIVNPGHYPAIKTLKELDESGLKKVKSYYTAKGINESFHTLYFNDLPVCAPDSYFCFQK
ISENREVAALVDNYIGDKAIQEFSIAGFPKVIAVEERYFTMHWTMEIIKLACILHRPVETTL
RQLTSAGVLQKLNIQDDIEDSRRIAAKNHTSFTSSLSMNHLZGVFYICMLGFMVAVVVFNI
EILIPGFQQFFT
>BgerIr520P
MMIANKIICCMITAHSKMFPPEIETSIADRFADCILNISRRYFELDLPVAVQTSSMYCNQFQ
TGIYDNZLLRTLIKENHFSQVTLGYIKYMEYRITYGYMTGNSMKYGSYLLVVSGKSIKEAYD
MSSTMVNRILAGKNTKAKLVIVFTGTTLTFHQQKRFAKKLLQNALTAGFVNFIVIAPSAIQ
GGNLQQLNIFGWNPNSQSNICSKILDTIEQLDTWSFEKRAFISNTDLFPQHTFIDMKGCKF
LITIMLQYPFIYRFPRGNQMIMRGSFYNVIPILNNLLNCSIRIGAPDPKYEASVHAVFPVIVN
AKQQNDACTMIYPYFSEDYYWYVASPLEIPRWKSLIRAFEPEMWTLVGLAFIFGTLTLYY
VQKFMQLSHSKNDNNITLTNQFLSSMKTYLGLSIIVKFRGKTAILTFILWLFYCMVINSAYQ
SALFGLIVNPGHYPAIKTLKELDESGLEKVKSYYTQKVIRKGHESLYFNNLPVCAPDSYLCF
KKLSENREVAVLVDNYIGDKAIKEFSVGGAPXVIAVEERYFTMHRTMEINKLACILHRPVE
TTLRQLTSAGVLQKWNLQDELRENIKIAARNHTSSSFSLSLNHIYGVFYICMLGFMLAVVV
FIIEILIPGFQQVFT
>BgerIr521P
MRMNKLLTLMMIANKIICCMNIARVKMFPREIDTSIADRFADCILDISRRYFELDLPVAIQT
SSMYRNQFQTGIYDNQLLRTLVKENQFSQVTLGFIEDMEDKMTYSYNTGNEMKYGSYILV
VSGKSIMEAYDMSSMMVDRILVGRNZKAKLIIVFTGTSLTFHQQKRLAKKLLQDTLTAGFV
NFIVIVSTAIDGGTLQQLNIFGWDPNSQSDICSNKLDTIEQLDTWISDKRAFLLNADLFPQR
TFIDMKGCKFYIVINMHYPFIYIFRQRNRVIMSGSFYNVIFILNDLLNCSIRIGSYDPKSYRIS
HAEFPVFLHTQRKNDDCTMIYPYFVEDYYWYVPSPLEIPRWKSLIRAFEPEMWTLVSLAF
IFGSLTLYCIQKFMHLSHSKNDNSISLTNQFLSSMKTYLGLSTIVKFRGKTAILTFILWLFYC
MVVNTAYQSALFGLIVNPGHYPAIKTLNELDESGLEKVKGYYAEDVVEEGLHSIDFNDLPL
CANDNYVCFKKISENRKQAVLVSKYVGDKAVKEFSKDGVPKVIPVEERYFTMHWTVEINK
LACILHRPVERVLRQLTSAGLIQKWNLQDDIQENRRIAANNRIASSSSLSLNHLYGVFYICIL
GFMVAVVVFIIEILTPGFQQVIT
>BgerIr522P
MMIANKIIICMNIAHVKMFPREIETSTADRFADCILNISRRYFELDLPVAIQTSSMYRNQFQ
PGMYDTQLLRALIKENQFSQVTLGYIRYMEYXGNAMKYGSYLLVVAGNSIREAYDMSSTM
VNRILAGKNTKAKLVICFTATTLTFQQQKRLAKKLLQDVLTAGFVNFIVIVPSAIQGGHLQ
QLNIFGWDPNSQSNICSKKLDTIEQLDTWSFEKRAFQMNADLFPQRTFIDMKGCKFYITII
LEYPFIYRFPRGNQMIMRVTFYNVIPILSNLLNCSIRIGPYDKKYHRFAHAQFPVIVNTQRK

NDDCTMVYPYFVKDYYWYVASPLEIPRWKSLIRAFEPEMWTLVILAFIFGTLTLYYIQKFM
HLSHSKNDDSITLTNQFLSSMKTYLGLSTIVKYRGKTAILTFILWLFYCMVINTAYQSALFG
LIVNPGHYPAIKTLKELNESGLEKVKSYNTGKEIKNSIHPLYFNDIPVCAHSNYLCFQKLSE
NREVAVLVDNYIGDKAIKEFSVGGAPKVIAVEERYFTVHWTMEINKLACILHRPVETTLRQ
LTCAGVLQKWNLQDELRENIKIAARNHTSSSFSLSLNHLYGVFYICMLGFMLAVVVFIIEILI
PGFQQVFT
>BgerIr523P
MMIANKIIICMNIAHSKMSSPKIDTSIADRFANCILNISRRYFELDLPVAVQTSSMYRNQFQ
TGNYDSHLLRRLIKENPFSQVTLGFIEDMENKIAYSYHTGNEMKYGSYLLVVSGNSIKEAY
DMSSTMVNRIVVGRNSKAKLVICFTGTSLTFHQQKKTLKKLLQDTLSAGFVNSVVIVPSTI
QGANLQQLNIFGWNPNSQSNISSRKLDTIDQLDTWIFGKRAFLSNANLFPQXSFIDMKGC
KFLITIMLQYPFIYRFPRGNQMIMRGSFYNVRPILNNLLNCSIRIGAPDPKYEASVHAVFPV
FVHILRKNDDCTMIYPYFVEDZFWYVPSPLEIPQWKNLIRAFEPEMWALISLTFIFGTXTL
YYIQKFMHLSHSKNDSISLTNQFLSSMKTYLGLSTVVKVRGKTAILTFILWLFYCMVIDTAY
QSALFGLIVNPGHYPAIKTLKELDESGLEKVKSYIDYNGEDEVQDQHSLYFKDLPVCAYSNY
FCFKKLSENREVAVLVDNYIGDKAIKEFSVGGAPKFIAVQERYFTMHWTMEINKLACILHR
PVETTLRQLTSAGVLQKWNLQDDLRESIKISARNHTSSSSSLSLNHIYGVFYICMLGFMLAV
VVFIIEILIPGFQQVFT
>BgerIr524
MMIANKIIFCMNIASAKMFPPEIDTSIADRFADCILNISRRYFGLDLPVAVQTSSMYRNLFQ
PGIYDNQLLRALIKENQFSQVTLGYIKYMELQTAYSYETGNKMKFGSYLLVVSGKSIREAYD
LSSMMVDRILVGINSKAKLVIFFTGTSLTFHQQKRLAKKLLQDALIAGFVNFIVIVPSAIQGA
NIQQLNIFGWNPNSQSNICSEKLDTIEQLDTWSFEKRAFLSNADLFPQHTYIDMKGCELYII
IVFTYPFIYRFPRANQMIMRGSFYNVIPILNNLLNCSIRLRTPDPKYDAFVHAIFPLFVHTLL
KNDDCTMIYPYFVEDYFWYVPSPLEIPRWKSLIRAFEPEMWALVNLAFLSGTLTFYYFQK
FMHLSNSKNNNSISLINQFLSSMKTYLGLSTVVKFRGKTAILTFILWLFYCMVINTAYQSAL
FGLIVNPGRYPAIKTLKELNESGLKKVKSYYSYIEEDEIQDQHSLYFNDLPVCAYSNYFCFK
KLSENREVAVLVNNYIGDKAIKEFSVGGAPKVIAVQERYFTMHWTMQIIKLACILHMPVET
TLRQLTSAGLLQKWNIQDALRENIKIAVRNHTSSSSSLSLNHVYGVFYICMLGFMLAVVVFI
IEILIPGFQQVFT
>BgerIr525
MNKLLTLMMIANKIICCMNTAHVKMFPREIDTSIADRFADCILEISRRYFELDLPVAVQTSS
MYRNQFQTGIYDNQLLRTLVKENQFSQVTLGFIEDMEDKMAYSYNTGNKMKYGSYILVVS
GKSIKEAYDLSSMMVDRILVGRNSKAKLIIIFTGTSLTFHQQKRLAKKLLQDTLTAGFVNFI
VIVPTAIQGGNLQQLNIFGWDPNSQSDICSNKLDTIEQLDTWISDKRAFLLNADLFPQRTFI
DMKGCKFYIVINMHYPFIYIFRQRNRVIMSGSFYNVIFILNDLLNCSIRIGSYDPKSYRIAHA
EFPVFLHTQLQNDDCTIIYPYFVEDYYWYVPSPLEIPRWKSLIRAFEPEMWTLVSLAFIFGS
LTLYCIQKFMHLSHSKNDNSISLTNQFLSSMKTYLGLSTIAKFRGKTAILTFILWLFYCMVV
NIAYQSALFGLIVNPGHYPAIITLNELDESGLEKVKSYYAQDVLAEGLHSINLNDLPLCAND
NYVCFKKISENRKQAVLVSKYVGDKAVKEFSKDGVPKVIPVEERYFTSHWTVEINKFACIL
HRPVERVLRQLTSAGLIQKWNLQYDIQENRRIAANNRIASSSSLSLNHLYGVFYICILGFMV
AVVVFIIEILTPGFQQVI
>BgerIr526P
MMVANKIIRCMSIAPAMMFPLEIDTSIADRFADCIVNISRRYFDIAFPVAIQMSSMYHNKF
KTAAFDNRLLHIISNENIFSQITLGFIENMNDTSVYSYDTGNLFKYGSYFVVLSGNSIKENYD
LFVQMTLRIFLGRNLKAKLIILLTIASKSFLQQKSLAKKLQFAFRFGLVSAIVIIPTAIESSNL

QQLNIFGWDPNSQSNICSMKIDTIRLLDTWVIKXREFILNADLYPEYKFIDMKGCKLIMGII
LYYPFIYTYRQRNNEIMEGSFYEFLLVLQDLLNSSIRIGPQTPYFKGLHAKFPLYMGSTLQV
HGCYITYPYFNDDYFWYVPSPLEIPRWRSLIRAFKPEMWICVSIAFVLGTLTLFCIQKFAQN
SHPKNKSVSLTNQFLISMKTYLGLSTVVNYRGKTAIITFVLWLFYCLVINTAYQSALFGLIV
NPGLYPAMKTLKELDDSGLVKETLYRANEKDQNSLYAKYFNNIPICADVNYNCFAKVSKN
RLHAILSSVFVGNKAKEYFIEDGRSLVVPIEERYFTGYLVVEINDLSCILHKPMEIILKQLVSA
GLIEKWNFQNSVLSLRRTALKNDTSSVSSFGMNHLYGVFYICFFSVLVAATVFLIEILLPAF
HQ
>BgerIr527
MMNLVTFLIIARTFCCIKFTSAKMFTSDIGSSKATNFADCILNISRRYFDNTLPVAIQISSMY
HKKYQTNTNEKQLLHTLSKENEFSHVTFGLIEDMENVSVYSVKTGNEMKFGSYLMVLSG
NSVKENFDLVSKMILRIFIGKNYKSKLIMILTNTVKTPHDQGTHATMLLRFSFRIGFEYSIVI
APTTIQGSNLQQLNVFGWNPSLQSNICSTKLDNINLLDTWNLEQRAFVLNADLFPEYAFID
MKGCILVTAIDYYYPFRIPIFKNKKLTFERSFFDFMLILEKLLNCKIRPVSQDMYIDTIHAKF
PVLVGTELQFNDCGWVZPYYIDDYYWYVPSPLQYPRWRSLIRAFKPEMWVFVSLVFIFGT
LMVFYLQKFANKSDCKNKNKSLTDQFLSSMKTHLGISTTAHYRGQNRITTFLWLFYCMVI
NTAYQSALFGLIVNPGYYPAIKTLKELDESGLEKVTEYYSDIVDQGAPYSLYYNKLPLCSNT
VYNCFKKITKQRLQTVLASSYXGKKAMEEFTENGLPQIVPIKEKYFTIYFNVEINELSCILQK
PMGITLRRLVSAGLIQRWNNAFRIIZKRRVTLNNETSLVSSFCLTHLZGVFYICILGLMLAVI
VSLTEILLPSFHQGFT
>BgerIr528
MLIYTRFRMMKLLIFLIIASKFCSIEITSTKMFTSDIDTSIANNFADCILNISRRYFDNTLPVA
IQMSSMYHQKYQPNTNENQLLYTLSKENEFSHVTLGLIEDIDNPFVYGFKTGNAMKYSSY
LLVLSGNSIRENFDLVFKMMQRIYIGRNEKSKLIIVLTNTLKTLHEQERHAKKLLQVLFKIG
FVYSIVIAPNTIHGSNVQQLNIFGWSPNLQNNICSMKISTINLLDTWNFERRAFVLNAELLP
EYTYIDMKGCVLLTGIVYYYPFIFPYFPDNKLTMGRSFFEFILILEKLLNCKFRLRSQDMSIN
IIHVQFPIFINTELQFNDCGRVYPYYSDDYYWYVPFPLEIPRWRSLIRAFKPEMWVFVSLAF
IFGTLILFYLQKFSKKRDYKNKSISLADQFLSSMKTYLGISTSVHYGGKIAIATFVLWLFYCL
VINTAYQSALFGLIVNPGHYPAIKTLKELDESGLKKVTAYYTNKVDLGGVYGLYYNNYSVC
SNSVYSCYKKVTKERLQTVLESSYAGKKAMEEFTEGGLPQIVPVKEKYLTIYIVAEIYCLSCV
LHKPMEITLRRLVSAGLIERWNNEFRITEKRRVTLNNETSLVSAFGLSHLYGVFYICILGLM
LAFIVFLTEILLPAFYQGFT
>BgerIr529
MTKLVTFLIIASKFCCIEFICARVFTPDIGSSTPNNLSDCILNIIRRYFDNTLPIAIQISSMYHK
KYETNNNENQLFHSLSKENEFSRVTLGLIQDMDNGLVYSFKTGNEIKFGSYLLVLSGNSIR
ENFDLAFKMIQRIFIGKNDKSKVIMALTNTLKTLQEQERHAKKLLQVSLQVGLVYTLVIAP
NTIHGSNVQQLNIFGWNPNLQNNICSMKLDTINLLDTWNFEQRAFVLNADLFPEYSYIDM
KGCVLYTGIGYYYPFIYPYLSNKTTTMQGSFFDFILILEKLLNCNIRLGSQDYINSIHAMFPIII
GTELQFNDCRWVYPYYSDDYYWYVPSPLEIPRWRSLIRAFKPEMWIFVSLAFLFGTLILFS
LQKFAMKHDCKNKSISLADQFLSSLKTYLGISTSVHYTGKIAIATFVLWLFYCLVINTAYQS
ALFGLIVNPGHYPPIETLKELDESGLEKVTAYPTEHVDLDGFYGLYYNNYPLCSNTAYNCF
KKVTKERLQTVLSSSFAGKKAMEEFTEGGLPQIVPVKEKYLTIYIIVEITDLSCVIQKPMEIT
LRRLVSAGLIQRWNDALSMTEKRRVTLNNETSSVSAFGLTHLYGVFYICILGLMLAVIVFLT
EILLPAFHQRFTYSV
>BgerIr530P

LFLANKVICCIEIVSAKTFLSDGATSVGSHFADCILKISRRYFDTTLPVAIQMSSMYDKKYQT
NVNENHLLNTLSKENEFSRVTLGFFENMNNDSAYNYSTGNEIKYGSYLLVLSGNNMKENF
DLAFKMTHRIYIGKNYKSKLLIFLTNSLKSFHHQEILAKKLLEFEFIVGFMSTIVILPTVIQR
KRLQLKIFGWDPNLQNNICSMKLDTIKEQDSWIFEKRDFLLNADLFPEYRFIDMKGCELF
VGVTAYYPFIFPQQNEKDEHIKGSFYKFILILQEVLNSSIRLRPQQPSFKSIHAMFPVFIGTH
LQFDDCSCTYPYYSDDYYWYVPSPQEIPRWRCLIRAFKPEMWILVSIAYLFGTMTLFCTLK
FSQNSRNKSISLADQFMSSMKTYLGISTSVHYRGKIAIATFVLWLFYCLVINTAYQSALFGLI
VNPGHYPSIKTLKELDESGLGKVTVYRTEDRDLNGLYSRYYNGLPACSNNNYDCFKKITKE
RLQAVLASTYIGKRAMIEFSQGDFPEVYPVEERYFTMYVVVHINYLSCLLHNAMQTTLRRL
VSAGLITKWNLEVSIKEMIRVALKNETSLVFSFGLNHLYGAFYICILGLMVAVIVFLIEILTPS
FYLK
>BgerIr531
MIMANKILIFIAITTANMIPQDDDNSKVNNFADCILYISLRYFDFSLPVAIQMSSMYHTKYE
TNTHENMLLNTISKENQFSRVTLGFIENMNNASVYNYNTGNEMKYGSYIIVLSGSNMKEN
FELTVKMMKRIFVGKNEKSKLLIILTNTIQGSHQQERVANILLQFVFKVGFVKTIVIIPTVIQ
DSNLELKILGWDPNSQSNICSLKLDQIKQLDTWIFKQRTFRLNADLFPEYRFVDMKGCKIF
IGINHYYPFINTFFVDKKKYMDGSFPEVLIILQKLLNSSFRECPQKPFTNKMHARFPIFIGRD
LQFNDCSYVYPYYTDDFYWYVPAPLEIPRWKSLIRAFKPEMWVFVSLAFILGTLILFYLQK
FAKYSDSKTESMSLTNHFLSSMKTYLGLSTNVHYTGKIAILTFVLWLFYCLIINTAYQSALF
GLIVNPGHYPAIKTLKELDESGLEKVRTYFSKEKDPNGLYSLYYNNFPPCSNNTLKCFKKII
NERSHAVLASVYVGKKAIEEFTEFGLPLVRQIEEKYFTMYIVVSITHLSCMLKEPMEITVRR
LVSAGLIEKWDSDFRIERITRITLNNDTSSVYSFGLNHLYGVFYISIFGLIVAANVFLIEIFLPV
FDKVLI
>BgerIr532P
MLNLVTFLIVSSKFCCIELTSAKMFTPDIDSSTDINFEDCILKISRRYFDNTLPVAIQMSSMY
HKKYESNSNEKQLLHTLSKENZFSHVTLGLIRDMDNASVYSVKTGNVMKFGSYLLVLSGK
SIRENFDLAFKMILRIFIGKNHKSKLIMVLTNTLSLHEQIRHAKKLLRYSFRVGFAFNIVIAP
TTILGSNLQQLNIFGWNPNLQTNICSMKLDTINLLDTWNFEQRAFVLNVDLFPDYRFVDM
KGCKLVTAISDYYPFIFPFSLNNKVNMEGSFYEFIIIIEKLLNCXFVLFHKACRIDIMHAEFPI
FISTELQFNDCGWVYPYYSDDHYWYVPAPLEIPRWRSLIRAFKPEMWVFILLVFIIGTIIFF
YLQKFAETSDSKNKSISLTDQFLSSMKTYLGLSTNVHYTGKIAIATFLWLYYCLVINTAYQS
ALFGLIVNPGHYPPIETLMELDESGLEKVTEYHTDKVDQGGPYSLYYNQFPLCSDTVYNCF
KKIAKERLQTVLASSYVGKKAMEEFTEGGLPQIVPVREKYFTMYLVVQISDLSCVLHKPME
ITLSRLVSAGLIQRWNEAFSITERRRVTLNNVTSPVFAFGLTHLYGVFYICILGLVLAFIVFL
TEILLPAFHQGFT
>BgerIr533
MMKLVTFLIIASKYCCIELTSANMFTPDIGPSTDINFADCILNISRRYFDNTLPVAIQMSSMY
HKKYESNSNEKQLLHALCNENEFSHITLGLIGDMDNVSVYSVKTGNEIKYGSYLLVLSGSSI
RENFDLAFKMIQRIFIGKNHKSKLIMVLTNTLSLHEQGRHAKKLLRYSFRVGFAFNVVIAP
TTILRSNLQQLNIFGWNPNLQTNICSTKLDIINLLDIWNFEQRAFVLNVDLFPEYRFVDMK
GCKLVTAIGDYYPFIFPFSLNNKVNMEGSFYHFIVIIEKLLNCKIHLGPPHMSIDIMHAQFPV
FISTELQINDCGWVYPYYTVDHYWYVPSPLEIPRWRSLIRAFKPEMWVFILLVFTIGTLILF
YLQKFAKKSDSKNKSISLTDQLLSSMKTYLGISTSVHYRGKIAMATFLWLYYCLVINTAYQS
ALFGLIVNPGHYQPIETLMELDESGLEKVTEYHTYKVDQGGPYSLYYNKFPVCSDTVYSCF
KKIAKERLQTVLASSYVGKKAMEEFTEGAQIVPVREKYFTMYLVVQISDLSCVLHKPMEIT

LRRLVSAGLIQRWNEAFSITERRRVTLNNVTSPVFAFGLTHLYGVFYICILGLVLAFIIFLTEI
LLPAFHQGFT
>BgerIr534P
MMDLVTFLIITSKFCCMEFTSAKMFTSDIGTSTGINIVDCILNNSRRYLDNSRSVAIQTSSM
YHKKYETNINEKQLLHTLSTENEFSRVIFGLIEDMDNISVYSVKTGNEMKFGSYIMVLSGNS
EKENFDLVSKMILRIFIGRNYKSKLIMVLTNTLLLHEQERQTKKLLRYSFRVRFAFIIVIAPI
TILGSNIQQLNIFGWNANLQTNICSTKLDTINLLDTWNFEQRAFTLNADLFPEYRFVDMK
GCKLVTAIGDYYPFIFPYSLNNKVNMEGSFYGFIIIIEKLLNCKIHLGPTHMSIDIMHAQFPV
FISTELQFNDCGWVYPYYTVDHYWYVPAPLEIPRWRSLIRAFKPEMWVFILLVFIFGTIIFF
YLQKLAETSDSKNKSISLTDQFLSSMKTYLGLSTNVHYTGKIAIATFLWLYYCLVINTAYQS
ALFGLIVNPGHYPPIETLMELDESGLEKVTEYHTDKVDQGGPYSLYYNQFPLCSDTVYSCF
KKIAKERLQTVLASSYVGZKAMEEFTEGDLPQIIPVRVKYFTLYIVVQISDLSCVLQKPMEII
LRKLVSAGLIQRWNDVFSITEVRKVTLNNVTSPVFAFGLTHLHGVFYICILGFMLAIIVFLK
EILLSACHQGFT
>BgerIr535
MMKLVTFLIITSKFCCIEFTSAKMFTSDIGSLTENNFADCILNISRRYFDNTFPIAIQMSSMY
HMKYQTNVNENQLLHSLSKENEFSRVTLGFIEDMDNPSVYSFKTGNKIKFGSYLLVLSGNS
IKENVDLAFKMLQRIFIGKNNKSKLIIVLTNTFKTPHKQKRHAKKVLQVSFRVGFVYSIVIG
LTTIHGSNLQQLNIFGWSPNLQTNICSMKLDTINLLDTWNFEQRAFVLIADLFPEYRYTD
MKGCVLFTGTVNFYPFIFTYYINNKLNIGGSFYNFILILEKLLNCKIRLVQQDQSIDTIHALF
PIFVGTEVQFNDCEWVYPYYSDDHYWYVPSSLEIPRWRSLIRAFKPEIWVFVSLAFIFGTLI
LFSLQKFEKKRDCKIKSISLVDQFLSSLKTYLGISTSVHYRGKIAIATFVLWSFYCLVINTAYQ
SALFGLIVNPGHYPAIETLKKLDESGLEKVTAYEIGHVDLDGIYGLYYNNYPLCSNTAYSCF
KKIAEDRMQTVLTNSFTGKKAMEEFRKGGLPQIVPVKEKYFTLYIIVEINGLSCVLQKPMEI
TLRRLVSAGLIERWNDEFRITEKRRVTLNNETSSVSAFGLTHLYGVFYICILGFMLAVVCFI
TEILLPAFYQFLF
>BgerIr536P
MMKLVTLVIIASKFCCIEFTSAKMFTSNIGSSTANNFADCILNISRRYFDNTLPVAIQISSMY
HTKYQTNTNENQLFHTFSEENEFSRVTLGLIEDMDNPLVYSFKTGNAMKFGSYLLVLSGN
SIKENFDLALQMIQRILLGKNDKSKLVMVLTNTLKTLHEQERHXKKLLQLSFTVGFVYNILI
VPTIIHGSNLQQLNIFGWNPILQTNICSMKLDTINLIDTWNFEQRTFVLNADFFPEYTFVN
MKGCALVIGIGSFYPFISPYAHNGKVTTEGSFYQFLLILEXRLNSNIRVGPQYRSIDTIHALFP
IMIGPNVRFTDCGWVYPYYSNNFYWYVPSPLEIPRWRSLIRAFKPEMWVFVSLAFILGTLI
LFYLQKFAKKRDCKSKSIALADYFLSSLKTYLGISTSIEYKGKTAIASFVLWLCYCLVINTTF
QLSLFGLIVNPGHYLAIETLKELDETGLGKVKSYFTEEVDRDYVYGLYYNKYPVCSNDVYK
CFKXAMEEFTDGGLSKIVPVKEKYZTIYVIVEINGLSCVLQKHMEVALRRLVSAGLIERWN
DQFLIEERRRVTLNNXTVSVIGLTHLYGAFYICILALMLSVIVFLTLFLLPAFYQGFT
>BgerIr537P
MPIFIRFRMKKLVTFMIIASKFCCIEFTSVKMFTSDIGSSTANNFADCILNISRRYFYNTLPV
AIQMSSMYHKKYQTNTNENQLLHSLSKENEFSRVTLGVIEDMDNPLVYSFKTGNAIKYSS
YLLVLSGNSIRENFDLVFKMMQRIYIGKNEKSKLIMVLTNALKTLHEQERHAKKFLQALFK
VGFVYSIVIARSNIHGSHVQQLNVFGWNPNLQSNICSKKLDTINLIDTWNFEQRTFVLNAD
LFPEYRFVNMKGCLLIVAIAQHYPFIYPYIQNRKLTTEGSFYQFLLILEKLINCNIRLGSQDK
SIDYTDIHALFPIIIGPNVKFTDCGWVYPYYSNDYYWYIPSPLEIPRWRSLIRAFKPELWVF
VSLAFILGTLILFYLQKFAKDRDCKSKSISLTDYFLSSMKTYLGISTSIDYKGKTAIATFVLWL
FYCLVINTAYQSSLFGLIVNPGHYTAIKTLNELDESGLEKMMGYYTDEIDLDGSCSIYYQKY

PVCSNTLYDCFKKITKERLQTVLASSYXGKKAMEEFTEGGLPQIVPVKERYFTIYMIVEING
LSRILQKPIEITLRRLVSAGLIEKWNDQFSIAEKRRVTLNNETSLVSVFGLTHLYGAFYICIL
GLMLAVIIFLTEFLLPAFYEVLFQVFRCIFV
>BgerIr538P
MQIFIRFRMKILVTFMIIASKFCCIEFTSAKMFTSDIGSSTANNFADCILNISRRYFDNTLPV
AIQMSSMYHKKYQTNINENQLLHSLSKENEFSRVTLGVIEDMDNPLVYSFKTGNAMKYSS
YLLVLSGNSIRENFDLVFKMMQRIYIGKNEKSKVIMVLTNDLKTLNEQQRHAKKILQALFK
VGFVYSIVIARSNIHGSHVQQLNVFGWNPKZQSNICLKKVDTINLIDTWNFEQNTFLLKAD
LFPEYRFVNMKGCLLIAGIAGHYPFIYPYYHNRKVTMEGSFYQFMLILEKLLNCNIGLASED
ISIYSIHAVFPIIIGPNVKFTDCGWVYPYYSNNFYWYVPSPLEIPRWRSLIRAFKPEMWVFV
SLAFILGTLILFYLQKFAVESNCKSKRFSLADYFLSSLKTYLGISTSIEYKGKTAIATFVLWLF
YCLVINTAYQSSLFGLIVNPGHYPAIETLKELDESGLEKVMSYETQEHVDDSYGLYYHKYPL
CANNVYDCFKKITKERLQTVLASSYAGKKAMEEFTEGGLPQIVPVKERYFTVYMIVEINAL
SCVLQKPMEITLRRLVSAGLIERWNDEFFISEKRRVTLNNETSSVSVFGLTHLYGAFYICILG
FMLALIIFLTEFLLPAFYQCFTSSV
>BgerIr539P
MLSYTRFRMMQLVTFLXSKQICCIEFTSTEMFTRDIRSATTNNFADCILYISRRYFDNTLPV
AIQMSNMYXKIQKNTDENQLLHTLSKENEFFRVTLGLIESMGNPSVYSSKTGNACAYLLVL
SGNSIRENFDLVFKMMQRIFIGKNDKSKQIMVLTNTLTTIHEQERHAKKLLQSLFKIGLVY
CVVIARTTIHGSNIQQLNIFGWNPNLQNNICSIKLDNINVLDTWNFEERSFVLNADIFPDSA
FVNMKDCLLFEGINQHYPFIYPFAHNRKITMQGSFYQFMLILERLLNCSIYLESQYMSRRH
AMFPIIIGTDVEFTDSGWVYPYZSNDFYWYVPSPLEIPRRRSLIRAFKPEMLLFVSLAFIFRT
LILFYLQKLAKESDYKNKSISLAVYFLSFLKTYLGISKSIDYEGKIAIATFVLWLFYCLVINTA
LESSLFGLTVNPGHYPAIETLKEFDEPGLEKVMGYYTHEVHQDGSYGLYYHRYPXCSNTVY
SCFEKVAKGRLQTVLSSRYARKKAAEEFTEGGLRQIIPVKEKYFSLYIVVEITGLFWVVQKP
MEITLRRQVSAGLIERWNNEFQITEKRRFTLNNETVLVSAFGLTH
>BgerIr540P
MMKMVTFLITASKLCCIEFTSAKMFTSDIGSATANNIZDCILNISRRYFDNTLPIAIQISSMF
RLKYQTNTNENQLLHTLSKENEFSLVTLGLIEYKGNPSVYSFKTGNAMKFGSYLLVLSGXSI
KENFDLAFKMTQRILIGLNEKSKLIMVLRNTLKTLYKQERHAKKLLQVSFRVGCVYSIVISP
SSIHGSNVQQLNIFGWSPNLQTNSCZMKLDTINLLDTWNFEQRAFVLNVEFLPEYKYTDM
KSCVLLTXTVNYYPFIFPYYINNKLTIGGSFHDFILIIEKLINCKIRLRSQHMPINTMHAMFP
SFINTEVQFNDCGWVSPYYCNDYYWYVPSPPEIPRWRSLIRALKPEIWVLISLAFMFGTLL
LFSLQKFEKKCDCKNKSISLAEQFLSTLKXKFLGISTSVHYTRKIAIATFVLWSFYCLVTNTA
YQSALFGLIVNPGHYAAIETLKELDESGLEKETAYQTGHVDQEFYGLYYNKYPLCSNTAYS
CFKKLRKDDCXFYGKKAMEVFRKGGLPQFVPVKEKYLTIYIIVEINGLSCVLQKPMEITLRR
LIERWNDEFRITEKLRVTLNNETSSVSAFGLTQLYGVFYICILGFLLAVICSITEILLPAFYQ
>BgerIr541P
MLISTRFRMMELVTFVIIASKFCCIEFTSAKMLTSNIGSSTANNFADCILNISRRYFDNTLPV
AIQISSMYHTKYQTNTNENQLFHTFSEENEFSRVTLGLIEDMDNPLVYSFKTGNAMKFGS
YILVLSGNSIKENFDLALQMIQRILLGKNDKSKLVMVLTNTLKTLHEQERHAKKLLQLSFT
VGFVYNILIVPTIIHGSNLQQLNIFGWNPILQTNICSMKLDTINLIDTZNFEQRTFVLNADLF
PEYRFVNMKDCALFIGIGSFYPFISPYAHNGKVTTEGSFYQFLLILEKILNCSIRVGPQYRSID
TIHAMFPILINSEVQFNDCGWVYPYYSNDFYWYVPSHLEIPRWRSLIRAFKPEMWVFVSL
AFILGTLILFYLQKFAKERDCKSKSIALADYFLSSLKTYLGISTSIDYKGKTAIATFVLWLFYC
LVINTAYQSSLFGLIVNPGHYPAIETLKELDESGLEKVKSYFTKEVDRDEVYGIYYNKYPVY

SNDVYTCFKKISEERLKTVLACSYEGKKAMEEFTEGGLPKIVPVKEKYLTIYVIVEINGLSCV
LHKPMEVALRRLVSAGLIERWNDQFLIEEKRRVTLNNETSSVSVFGLTHLYGAFYICILGL
MLSVIVFLIEFLLPAFYQGFT
>BgerIr542P
MMKLVTFMIIASTFYCVEFNSANILTSDIGSSPATNFANCILNISQRYLDNTLPVAIQMSSM
YHKKCQPNTNENQLLHSLSKENEFSRVTLGLIEDMDNPLVYSFKTGNAMKYSSYLLVLSG
NSIRENFDLVFKMTQRIYIGRNEKSKLIIVLTNTLKTLHEQERHAKKLLQVMFKIGFVYSIVI
APTTINGSHVQQLNVLGWNPNVQTNICLKKLDIINLLGTWNFEQNTFVLNADLFPEYTFV
NMKGCLVFTEIALHYPFIYPYIYNRTFATTEGSFYQFLLILKKLLNCNISVGTHDMSIDNTDI
DVLFPIIIRPNAQFTDCGWVYPYFSNNYYWYVPSLLEIPRWRSLIRAFKPEMWAFVSLAFI
LGTLILFZIQKFAVERNCKSKRFSLADYFLSSLKTYLGISTSIEYKGKTAIATFVLWLFYCLVI
NTAYQSSLFGLIVNPGHYTAIKTLKELDESGLEKVMSYYVYDVELEGSYGIYYHKYPLCSNN
VYECFKKITKERLQTVLASSYVGKKAMEEFTEGGLPQIFPIKERYFTIYMIMQITGFSCILQK
PMEITLKRLVSAGLIERWNDQFSIAEKTRVALNNETSLVSVFGLTHLYGAFYICILGLMLSV
IVFLTEFLLPAFYQGLSCV
>BgerIr543P
MKKLVAFLILASKLCCIEFISARMFTSDIGSSTPNNFAECILNICRTYFLSLXPIAIQLTSMYD
TKYKTNNNEIHLLHSLSKENEFSRVIZGFIENMIKGTVFSFKTGNAIKYSSYFLVFSGNSIKG
NSDLVFNMTLRIFIGKNNKSKLIMLLRNTLKTLHEQERHAKKLLQFSFIVGLVYSLVQVISP
TIIHGXNLQQLNIFGWSPNLQTDICSMKIDNINLLDTWNFEQKAFVLNFDLFPEYTFVDIK
GCVLXGTVYYYAFIFPYMNNKIVTMEGSFFDFFINSSKLIKLZNSLDSPDMSIKHLTFPVFIG
TFLYFNDGAWVYPQYSNDFYGYVPYPLEIPRWGSLIRAFKPDMWVFVSLAFIVGTZILFSL
QKFXKTKSNSLTDQFLSSLKTYLGISTSVHCKGKIAIATFVLWLFYCLVIX
>BgerIr544
MLSYTRFRNMKLVIFLTIASQIYCIDFTSAMMFTTDIGSSTTNNFADCILYISRRYFNNTRP
VAIQMSSLYDKKYLTNNNEINLLYSLSKENEFSRVTLGLIENMGNPSVYSFNTGNAMKYSS
YLLVLSGNSLKENFDLAFKMTHRIFIGKNEKSKLIMVLTNTLKTPYEQEKHAKKLLQVSFR
VGFVYSIVIAHTTIHGSKLQQLKVFGWNPNLQKNICSIKLDTIDLLDTWNFEQRSFVLNAD
LFPEYRFVNMKGCLLFQGVSQHYPFIYSYIHNRKVTTEGSFYQFMLTLETLLNCKIRLRSED
MPIYTRHTMFPVLISTDVQFNDCGWVYPYYSNDFYWYVPYPLEIPRWRSLIRAFKPEMW
VFVSLAFIFGTLILFYLQKIEKKYDSKNNSISLVDHFQSSLKTYLGISTSIEYKGKTAIATFILW
LFYCLVINTAYQSSLFGLIVNPGHYPAIKTLKELDESGLEKVKSYFTKEVDRDEDYGLYYNK
YPVCSNDVYTCFKKISEERLKTVLACSYEGKNAMEEFTEGGLPQIVPVKEKYFTLYFIVKVN
GLSCVLQKPMEITLRRLVSGGLIERWNDEFSIAEKRRVTLNNNTSLVSVFGLSHLYGVFYIC
IFGFMLAVICFITEILLPAFYRDFI
>BgerIr545
MVIHAILVDFHAENLVETHLSELTVSIASIYFDIHLPVAVQITNRITYNPRKSLKRENVYGD
DIIKKLNTECHVPQLILGPPIKPELVSGLWTNVKFGFYLLVLSPNDIFEEEKMANVMMSRLI
VSNAQKGKLVIVTTRIYNIFEEQHYTAKQILQMILNFGFINAIVITPREISDISSLQYDIFGWN
PNEQSNICGLHIDKIKHLDTWVVQHKMFLFGTNLFPKRPNVNMNLCTLIVTSGTLYPYFYI
NRFKKLAGIFYHFVMALEDALNCSTIPNTKPHKYSQIFFPAFIAETDADFQNYGIYPYFFLD
FYWYVPSPLEKLRWKCLFRAFKFETWILVFLTFIIGSLALFLLEKSGNHSENHDSNISFADQ
LMSSMKTHLCITTVIHYKGLLATTIFLLWLFYCLIVNIAYQSALFGFMVNPGFGPQIKTLKD
LDESGLEKICEFSVNGNPLDSLFGKQFSKYQVCKNIPKCVEDIVERRSQALITNSFMGRVVE
KLVSKHGHSLLTPVEERVFTIYITLGLGKLSSMIQKPVELLVRRLFTSGILNKFIEEEELYLN
RIFALNQDGNSAFSLKLSHLQSAFYLSIIGIFMAFIVFIFEIVANLKKDKI

>BgerIr546
MTTNGIFLDSHTEIIVEKEVSECIVNIVRSYFNMHLPLALQTTGTYIRRNLFVKSNMFGDEV
INKFSSENHVSVVILGPPVDMETVERMGTNVFFGSYLLVLTTTTIMKDLKIAKLMMRRLIV
NMVPNSKLVIVITRINSIFEEQLYTAKCLLQIIFEYRFVNSIAIIPREIAGVSSLQFDVFGWDP
NEQSNLCASKIDKIKHLDTWVAEERTFLLGVNLFPKRPKLNLKDCRLGLRTNMLFPFFYQ
TSKKEFTGNFLFLISHLVMDHNFSVSFDTGLNPYTQIEFPIFIDTNDANSHCSALYPHIYLD
FNWYVPSPLQIPRWKSLFKAMKSETWIVIFSILILGSLTLFLLQKFSSFTDHQSFNISFVNQL
MLSMKTQLCIPTKARYKGSTASTTFILWLFYCLIINTAYQSSLFGFTVNPGYEPQIKTLREL
NESGLEKIRLFTFNKGSLTSQFAGQFRHYGYCVSDECFKTMAQQRSMALITDSFRGGSAM
RSLTSGGHSKITVIKERAFTLYVTIGILKLSCILHEPLQSTMSKLITSGIWDKWSGVLETKKS
MEFAFNEDKSKVFAFQLTHLQGAFYLWIVGNFVAIIIFLTEINFSVLKY
>BgerIr547P
MKSNCSNSLDVNIGTSVERSISECIVNITQRYFATRLPLNVQTPGTHYKLKYLQKNNLYGD
EVLQKLSSENHVSEIIVGYLPEFSNVYRMEANMVSSYLLVLTMNNNIEDRSLVEIMLRRVV
LKGNPKAKLVIASTRIHNILQQQIYSAKRLLQMTFNFRFVNASXIVPRAILGDSLLQFDIFS
WYPTDQSNICSLNIDNIKHLDAWIAKEKSFLFGTNLFPTQPKIYMKRCSITIALGSIFPYYYT
TKYGKTSGSIWEFINTLIPVLNCSITPGEYPYSQIYFPAFIDEKDMLYQCNAMYPYDSLDFY
WYVPSSLEIPRWKCLFIALKLETWIIIFTIFVLGSFPLLLLRKSKNHSEGRYFNISISNQLMSS
LSTHLCISTSVYYKGTIATTTFTIWLFYCLIVNTAYQSALFGFMVNPGHGPHIETLKELDES
GFEKLRFFYMLEDSKNSQFGIQFSQYRLCKNYAKCMEITIERRAQSLLINKYIGRFTVSLLT
RSGHPLIVPTDERVFTLHVTMIIGRLSCIIQQPMQVTLSRLFSAGLLNKWNEQKEVRLHRA
FAMNQEIDVVFAFKLTHLQGAFYLWSVGILAVFISFLVEILSSTLNSR
>BgerIr548
MTFIALFSLFLFVVIGDARISLHDNRILDAQLSKCTKGVVQQYFSQDVPITIVDNAAIFCNSP
QKNILSNYNELIAMLYQEIDLPLIHLGCVVNFTLKNSLKSGSAILLLPDEIMEYTDLMTLCEI
YVLRLVDWLENRAVRIILVSKVTTKTKKMQMLTAYGLLQGAWGSRVQIADSIVLLPEFGL
STWSKKKTPAIDAFTWFPSDQSDVCLRELDRVKFLDRWVSERTGFLRKADLYPVKAVSD
MRGCTLKVDLILDPPLGAVFLKNKGTYFIGTYGMMLQTSAEFLNIHLSYTVRQSRRYTADI
VAPIFLTHKFFSTECIVSYPYFMDTVTWYVPVFQIPKWQGPIRVFSMSMWINVTVAYVLGS
FTFWLLGKHDQQSYRDVSTIFLNTLKTYIGVGVRVKYRGVGVLFLIVWLLYCMQIYTAYQS
ILTGFLVNPGDFPQIQTLQGSELQNFTTIGFDSDNIDEQIWNKYTDCTQQQCWTKLTEKQ
KVAVFGLKFFFDLFINMEYNNKKPGVYRITESVKDYLKVAYMDLGCIFGRSFNSVFSRLVT
SGFPDKWIRDLTHNFWMDLEHNGNDHPDAISLVHIQGILYFLLIGFLLGLILFITEIIIYKIKI
KT
>BgerIr549
MKFPHIFILIDLTITFINADIELPISDILEIQLGKCLINISNEYFKDIMPVIIERNIDQYCSKDGR
NLEENYTNLITVLSEGMNHSVIHLNCYMEYKERHDIKPGSAVLIMPNDVTTPESMVRFTS
FFVHTLFSLYGNRAMRIVVVSKHLSKSRKSQMSLASAMLFGIWRTVQIADAIVLLPAVAQE
IHSRKIYPSIDVLTWFTNDQSDVCLLKVDRVKYLDHWQYEEERFVTNTNLFPVKDVTNM
RGCELKVELKKDPPFGHFMNKNERFFNDHNFGVYGVMLEAIGDFCNISFSYFIEVDEDLSL
DIIAPIFLVPGDDIHDSCYFSYPYFKDTVTLYVPITPIPKWQGIIRVFDIEMSILIAATYVFGSF
VFWLFAKLKNQGLDDISLALMNTLRTYLGDGMHTARKYRGVISTSFLTLWLFFCIQISTAF
QSVLIVFLINPGTYPPISSIEELDKSGFKKMSSYDFVGTTDVEKEWRSYGKCYKHDCWNILG
QNSRVAVLGNRFHFDVLVSKIIGRSKVSRVTDSVKDYLPVSYIQLGCIFGRRFNSLFHRLVM
AGISNFWLLKSSYLFWRTLKNESDVEEVTSISLVHLQGLFYLVLIGFLLAFIVFVCELLFR
>BgerIr550P

METYMNLMFIILITKSTKAYIKLPAQDILEHQLAKCIVNISNTYFDHQIPVWIQXDKFILFAQ
MIIRFQEGNFTTLFNILISEMKQPLMHLNCYRKHNAIIHSKPNLAVLMLPEHDILLDDLFE
RTSIFLNMLFELYCNYSVRLILVSKHLTKTKKSQLLIASAILSAAWESYKIADAIVLIPAVGW
TNQSNPPIDIITWTPKDQYNNCLLKLNRYQRIDRWLSEDQGFLYNSNLFPEKDVIDMRGC
EMVVTLLRDPPLGPYGEGINDQSPSISLGVYGAMLKTVGEFCNIEFLFFVDTDNVGSDIVTP
TFLATGDGYHDSCMFSYPFFRETITWYVHVVSIPKWQGIIRVFSVKMWILVTTGFFFGCFT
FWLIAKLKHQGVDDPVLIFLNTLAMYLGNPGQSSIGFRGPLTGPFFTLWLFYCLQVYTAFQ
SVLIGFLIDPGTYPTVSSIDELNEHGFLKVSTYDFVGSLDNEKQWNSYDKCDGSECWNVLN
GKSKIALLGSRFYFDSYEIYTNFKIVRVVDSVQDYLVTSICMLGCIFGRRLDDLYQRLFSSGIP
NLLLEENSRYYWTKEKNRKLSTNLVTTISLIHMQGPLYVLLFGYFLSLLIFISEFIFQALK
>BgerIr551
MEICCIFLFVVIKTSVVCAQILLPKDDILEVQLAKCILNISKKYFENDKPVILERQINRDCPSD
STYFDQNYISLITLLSQELENPIIHFNCYKEHNRVTNSKPNSAILLLPEYVTQVHDFMFLTK
GFLYMLSGLFYNSAIRLVIVCKHLTRTKFEQISIAAFILAGAWEYKIADAIVLLPGVEWSTDP
AIDIITWLPKSQSSRCLHELNRFKFLDRWLGIEERFKSNGNIFPLKDVTDLGGCELVVELHR
DPPFGPYAEGITPLAYRGELGVYGHMLHIVAEFCNISLLYATDNQNATSTIVTPVFLTAGYG
AHDSCLFSYPFFRDTITWYVPVVPIPKWQGMIRVFSVKMWILVTVAFILGFLTFLLLAKMK
HQGGNDIVLILMNTLCTYLGIGIAAEFRGFTGACFFTLWLFSCIQIYTAFQSVLIVFLIDPGSY
PTVSSVEQLDELGYKKISTYDFVGEIETEIEWNSFDKCDILDCWNVYSGNVAILCSRFYYDY
YMEFSNSKIARVVDSEKDYLVSSHFKLGCILGRKFDVLNSRLISAGIPNFLLQQSSLLFWLT
KTRELDPDTYSISLIHMQGPLYILLFGFLLAVIIFIFEILYRFFHHN
>BgerIr552
MHQIVIVLLLSVNQPSVLCANPTHNNTNSLEIILADCLLNIAETYFNPDMPVLIQLATDRSL
ASVNMFFRTTGELTLKLLVQTFKYTIINFGIIYTDIVKLYYRLKPGSVLLILPNLRILIKIILTIR
ELLERMRQIFGDSALRFVIVITNVIKSKEIKKQTVFSVLQAVWFYQKVSSVIVLMPVIVKSSE
NEKIFVIDVYSWLPERQFNEESAECLTNLTVVEHFDRWISNEKKFLKNTNLFPKKQLKSM
NNCTLNVGSTIFPPFGGFPILVMKHPNPELVNGLYTGIIKVLGQLTKVNFTTRVIKTNTWN
IFCPGGVVFDEGRGYILQDCKCTRAHFRASIYWYLSTSSIPQWQSIIRVFSTPMWGLVGISYI
MGSITFWLRGGYHNNLHGFLDAFFDTFRSYLAQSFSQKFNGIISATFISLWLVFCIQVYTSY
QTALIGFLIDPGDFSQIKSIEELEVKNIELYSGLQLGKRITHSYCNIIECMNILTNKYNVAILG
DSFLFDVFSRFAPKRYGRKRMYKLDMKYMDIPIICSAHLGCMFTEQLNFVTESLLTAGILD
KWLSDIMNQIERENRNHENDGAVPLSLANLQGPFYVLIVGLLLASVGFISELMFEYLIT
>BgerIr553
MTQFLIVLPLAFASVFCTIPLQNNSNYLDKQVADCLLQIAESHFHPDMPVFIQLATDKTLA
SKNYFFNTSGENALKSFVQSFKYTIINFGIIYSDMAKEYVNLKPGSVLLILPNLRLLDQILFT
VKQYLTRIRQTFGNRNIRIAVVITNVITSKENKERLALSVLNTVWFYQKMSDVIVLIPAAVK
SPENEKMSVIDIYSWLPETQFNEKGTVCLIEITDVQQFDRWISNEKRFLKNTNLFPKKQPR
KMNNCYLDVETTIYPPFGGFPNLVMKHENPDLVNGLYVGIMKVLREMTQIEFMTRISRKT
NWKILCPIRANVSGKMAISIPECTLTRSHFLVSIYWYISVSPVPQWQGIIRVFSTPVWALVT
AAYIMGCVIFWLRGSYQNSAQGFLDAFLDVFRSYLAQSISQNFTGIISATFFILWLFFCIQIY
TSYQTALTGFLIDPGDFTPIKSLEELAVKNIDLYRGLQLNEDLNYKFCDFIECFNILTNKEN
VGVLGNSFIFDVFSRFSPKRYGRKRMYKLDIKFMEFPIMCAVHLGCMFTEQLNFVTESLLT
AGILDKWLSGIMNQIERENINYENDGAIPLSLVNLQGPFYILIVGLVFASVGFISEFVFKYLI
>BgerIr554
MLRTKTTIVIIMTIFSSNHCSIIDNINPLDMQLAYCVLNISRTYFPKDKPVIIQLPTGTFSAS
GRYAPEPSEVLMKLFVSSFKLQVINCGVLDDEMLPNKYTLKPPSAVLILTDKSLENMFALL

GTFFFRISLHFGLYPIRVVIITTFEVASKWEQFDIAHALVNEAWVRFKIYNVVVLVQEPRKY
FDEENLPAVDIFTWFPKLQNNNKFGMCLNHLSVVTHLDIWVSKDKGFLLNENLFPLKQL
KDMNQCPTAVEMNFLPPLGIFLNKIMKTENSKVMNGVYVGILTVLSELTQVNFALIQASN
FSRGWSICPVFLRYFLFGEHTAFDCILTRIHFQMSLKLYVTVSPVPSWQSLTKVFSPVMWL
LILVTYIQGCVVFWFVGRYPISFQGILDVVFLVLQSFLGITVIDNHKGYIAAGFLILWLFFCM
QIYTAYMSMLTGRLIDPGDFPAVNDLEELQKSNFNGWTTMKLQNEDIGIMKKIFPNYEEC
IWFDCLKILTTRQKAAVLGDSYMFDLFTESSPRRHGRKRMIKMDSDLITNPVCCYMKLGC
LYADIFNTVTARLLAAGITNKWLQEIGDQISRENRINENDDPEALSLVHLQGPFYVLLVGF
VFSCV
>BgerIr555P
MIKKYVVFFILLVLVSKIKSRILFAQEETTNLEFQITKCLYNMSQTYFRTAMPTLLIEVPHTF
KAYRNISXFYMLFSSASHQIZNNRPLRLQRHITFLDFILVKEVKFAPSIILVILPDVGLNIMK
KLLVPSLYKSEKQWHSSIASRLIIISTKEMPSRRVQNTLAIQIFASLHHSFKVTDVLLITPGA
GWSKESRTZPSLDVLTWFPAKQKSSSDCIEAMSDITLLDVCSETGEGFKNNVNLFPNKDW
KDMNDCLVRVQFANKLPFGPLNSGEDIMQRNGIVNGIYNGVLLTLEKITNIKFTVKGAEHE
QSIYCPTPYAISKIYHHRFKTKYHFSTDFYLYQSIITVPQWQGLFRVFSATMWLLVVSTFAL
ACVTFWLMXVEGIGSVVHIVLRSYLAIDIPKKFTZFIRVSFFSLWLFFCMQVYTSYQAAITG
FLVDPGDLAVKNKNLDKISKNLDRSIWATSEYMLHYTYDIILYLFNNSCLLNEFIEKLATEP
GAAVLGEGTSFDLFLQFIPTRHGRRRFHKVQSRIHTIPAPCFIYLGTSFVDLFNKATDRLISG
GIIDKWHKDVLFQTRWENRISVVVGPVKLSLTHFQGLFFILLVGYMLSFVVFVTEIIVKR
>BgerIr556
MMIKQHIEYLLLLAFVSMIKSRNLAVQEETTNLEFQITKCLYNMTQTYFRTAMPTLVIEVP
HKFHANRFISIFNMLFSSVSHQILNFRPYQLPRNIKDDQLIFNKELNFTPSIILVILPDVGLNI
MKKLLVSSLYTLEVIYTSIASRLIIISAKEMPSRRVQNTVAKHIFSILQDELRLNDVLLITPGA
GWSKESRTSPSLDVLTWFPAKQKSSSKYCMETMSDITLLDIWCETGEGFKNNVNLFPNKY
WKDMNDCLVLVKFSNNLPLGPLISGKDIRQTNGVVNGIYNGVLLALEQITKMKFTEGGSK
HSYTIYCPIPFMYAQVLHHRFKSRYYFNTDFYIYQSITAVPHWQGLFRVFSTSMWLLVVTS
SALACVTFWLMNNYPRSLEGIGIGYVVLIVLRSYLAIDIPQNFTGFIRVSFFSLWLFFCMQVYT
AYQAAITGFIVDPGDLAVENKDLDKIGNNLDRSMWACSEYIENYQLMVSSFHLYNQTCKL
NECIEKLATEPGAAVLGEGTSFDLLLQFIPTRHGRRRVHKVQSRTHTVPAPCFIYLGTSFVD
LFNTVTDRLISGGFIDKWHHDIWFQTKLEKRISVAVGPVKLSLTHFQGLFFILLGGYMLSF
TVFVTEILFKRYWLNFYKIYSFPKRQSYLNRL
>BgerIr557
MMMIKQHIVYLFLLASVSMIKSRHLAILEETTNIEFQITKCLYNMTQTYFRTAMPTLVIEVP
HKFHANRFISNYTRAIFNMLFSSVSHQILNFRPYQLPRNIKDDQLIFDKQLNFTPSIILVILP
DVGLNIMKKLLVSSLFTLEVIFTSIASRLIIILTKEMPSRRVQNTVAKHIFSILQDELRLNDVL
LITPGAGWSKESRTSPSLDVLTWFPAKQKSYSKYCMETMSDITLLDIWSETGEGFKNNVN
LFPIKDWKDMNDCLVPVKFSNNLPLGPLISGKDIRQRNGVVNGIYNGVLLALEQITKMKFT
EGGSKHSYSIYCPIPFMYAQLLHHRFKSRYYFNTDFYIYQSITAVPHWQGLFRVFSTSMWL
LVVTSSALACVTFWLMNNYPRSLEGIGIGYVVLIVLRSYLAIDIPQNFTGFIRVSFFSLWLFFC
MQVYTAYQAAITGFIVDPGDLAVENKDLDKIGNNLDRSMWACSEYIENYQLMLSRFLLYN
QTCKLNECIEKLATEPGAAVLGEGTSFDLLLQFIPTRHGRRRVHKVQRRTHTVPAPCFVYL
GTTFVDLFNTVTDRLISGGIIDKWHQDIWFQTKLEKKISVAVGPVKLSLTHFQGLLFILLGG
YMMSFTVFVTEILVKHYWLNL
>BgerIr558

MMLLKIWFQMYVLVSMTMCFLVKNEVQILESQLAECMVNLAERDFAQEKLVFLQLPEF
WECRHYDLNYVDSLLQVLVNKMSREMMIFGCVEDEQDVEFFKTCVLILVVKEFNEIEDYR
EFAQNSVLLAINTYHLYELRIVIILTLVPETLEQQQEISLSLISGAWVYWIKTEIIVLTPEVG
WSSSKNKTPAIDIYGWNPTEQEDPCFREINQASFLDRWISAEKGFLRNEDLFTYKGFEYD
RTCTLHVKLNRLIPYISASLNETIDVPTNEMGIYPILWDIITKKINVKFEYHEEDSDNIADYD
IEGPELIRMTDFDGNYILTYPYFKDVIWYYVPVIRVARWQGLIRVFSGFVWLFVVITFLLG
MMILWVLEKYLHKKNVSIIDSFLNTLRTYIAVGIPFDCKGILSTSFFILWLFYCMQLYTAYT
SLLTSFLAYPGEYPLVSSIEELKESSYEIWTNILFIDSTNEIEKLFLTYSLCTASDCIDKLIIEQ
DVVVIGNIELEMEIVLLKGYTSGSERTSITIMDNSVIDYYVTLVMKSVILLDYFDKQMQWLV
SSGIPYFCLNKFDAQMLYFMSYSNGPKPTTLSELQGPFYFLIGGLASSLVAFIIENVYFWKK
SI
>BgerIr559
MMLLKIWFQMYVLVSMTMCFLVQNDVQILESQLAECMASLAERHFAQEKLVFLQFPELW
VCRHYDLNYVDSLLQVLVNKMSREMMILGCVEDEQEVENFKSGVLIIVVKEFNQMEIYRQ
FAKVSVQHAVNTYILYELRIVIISTLVPETLEQQQEISLSLISGAWVYWIKTEIIVLTPEVGWS
SSKNKTPAIDIYGWNPTEQEDPCFREINQASFLDRWISAEKGFLRNEGLFTYKGFEYDRTC
TIDIKLYRLYPYVRASPSDKIHASTNEMGIYPILWDIITKKINVKFEYHEEDSDNIADYDIEG
PVLMRITDFDGNYILTYPYFIDVIWYYVPVIRVARWQGLIRVFSGFVWLFVAITFLLGMMIL
WVLEKYLHNKNVSIIDSFLNTLRTYIAVGIPFDCKGILSTSFFILWLFYCMQLYTAYTSLLTS
FLAYPGEYPLVSSIEELKESSYEIWTNIQFIDSINEIEELFLTYSLCTASDCIDKLIREQDVLVI
GNIELEQAITLLLGYTSSSERTSISKMDNSLIDYYVTLVMKSVILLDYFDKQMHKLVSSGIPY
FYLHKLDIEKQFYMSYSNGPKPTTLSELQGPFYFLMGGLATGLIAFIVEKVYFRKKRILL
>BgerIr560P
MMVLKIICASSILVSTTICFSLENDVQILESQVADCLASIARRYFPQEALILVQTPENECEYT
DVNYVDSFLQVLVKEMNYQMMILGCAQYKLNTTKLKSAILVLVMEERDDVETYLRNSFVS
YAMVRIFTRYELYVLRVVIVSTIVPSSVQQQEEIALTLLNAAWFFFXKLEIILLTPEVGWSTS
TNKMPAVDIYSWNPVEQSEPCFRELDRVSYIDRWISVQKKFERNEDLYPYKGFVNDLTCI
LHVQVQFIPPFFIIDQSHKKILNPTGIYPTIWKMITSTTNIDIQYHSPYENLKYVDAEGPAMI
RTNSLDLVVFSYPYFRVVFNCYVPLIRIDRWQGLIRVFTGLVWGLVGMSFLLGTMTLWML
EKYILKKQVSFTDSFXKSLRTYVAVGIPFDCKGLLSTSFFLLWLFYCMQIYTAYTSLLTSFLT
FPGEYPLLSSMKDLNDTTYEVWSAFTYSDSINEIEDVFMTYPSCKAHDCMEKLVRDQDIA
VIAYQSIYDARYHKKYSEHGRSRIFRLNDPVMEYYVTVRFNCMLVYEYFEVRTQWLISSGI
AARIIQTYRSRYDDYTPDQHDLNDPKSMNLSEFQGPFYFLVGGLVTSFIVFICENIYSKKYD
VLLYLIIQYTIY
>BgerIr561
MMVLKIICASSILVSTTICFSLENDVQILESQVADCLASIARRYFPQEALILVQTPENECEYT
DVNYVDSFLQVLVKEMNYQMMILGCAQYKLNTTKLKSAVLVLVMEERDDVETYLRDCFV
SYAMVRIFTRYELYVLRVVIVSTIVPNSIQQQEEIALTLLKEAWFFLANLEIILLMPEVGWST
STNKTPDVDIYSWNPVEQSEPCFRELDRVSCIDRWISVDKEFERNEDLYPYKGFVNDLTC
TLHVQPKIIPPYFIIEEDHKKILNPIGIYPTIWKMISSTINIDIQYHSSFENLKYVDVEAPVLIR
KNKLDSLPLSYPYFRTVINCYVPLIGIERWQGPIRVFTGLVWGLVGIVFLLGSMTLWILEKY
ILKKHVSLTDSFLKSLRSYVAVGIPFDCKGLLSTSFFILWLFYCMQIYTAYTSLLTSFLTFPG
EYPLVSSMKDLNDTTFEVWIVFRYLDSINEIEDNFMTYPICKSKEDCMEKLVHDQDIAVIA
DESFYDARFDKKYSEHGRSRIFRLNDPVMEYYVTARFQYMRVFEYFEKHMQWLVSSGITA
RIIQKYRSRYEFNTPDQHDLNDPKSMTLSEFQGPFYFLVGGLVTSFIVLICENIYSKKYDVLL
YLIIQYTRY

>BgerIr562P
MKMLFKINCLASILASTAICFLLENDVQILESQVAEZLASIARKYFPQETLILLQTPEKECEY
NDVNYVNNLLQVLVEEINYQMMILGCTKYKIKRTTLKTAVLVLVVXDIETYLRNSFSFRAM
TRIIYSYHLHDLRVVVVSTLVPISVQQQEQIAVSLLTDVRNYYQLKMEIIVLTTEVXSTNKTP
AVDIYGWYPAEQYDPCLRILVRVSYIDKWISVEKQFERNEDLYPYKGFVHDLTCTLHVKIE
DFRPFFVRNTSDNMIRNPFGIYPNVWNIIMSTTNIKIKYHVDSEILDAQGPVLLRTDRSLEE
FIFSYPYFRYVLNCYVPLIRIDRWQGPIRVFTGLVFCLVGMAFLLGTMTLWILEKYILRKQV
SFKDSFLKTLZTYVAIEIPFDSKGLLSTSFFILWLFYCMQIYTAYTSLLTSFLAFPGEYPLVSS
MKELNGTSYDVRHTIGFEESLNEIECFLMSYPSCNFHDCMKKLVQDQDVPVIAEDSFFDN
RLGKAYSEHGRSQIIRIDVPVLEYYVTIIFFHNLHFKYFEKLMRNLVFSVIFQREIKEYRSVH
YPDYHDISDPKPMTSELQGPFYILVGSLVTGIICIVENIYSNKNDVILHLYYGQIINEFISTLNI
HR
>BgerIr563P
MSKLQKGTQYFRISTQRYRTQSLDLIPIMLLKVCCLICIVGSRTTGFLVQNDLQILESQLSEC
LWSIAGEYFPQDALVLLQTPELECKYNDLNYIDSTLQVLVKKNNYQMLISRCFEQDLKRTT
FQTAVLVLVVDEQANIEICQIFGELTLMRILFYHNSNNLRIVIVSPKIPISVQEQEETALSLIN
GFRTTAIDLEVMSKIELIVLMPQVGWSASTKKYSAIDIYGWHKSEQNDPCFHELNKVSLLD
QWFSDERAFLRNEDLFPYDAFTHHHTCTLHVVVVEEFIPYFFLKTDGDLFGIYFIVFDIIEK
TKNIKIEYHTNESEYYDAKGIEFLDTTKFYCEHSYAYLCFKENLYFYVPLIHVPRWQGLIRV
FSGFVWLSVIISFLLGMVILWLLDKYVNRKDVSITDAFLNTLRTYIAMGIQFDCKGLVSTSF
FILWLFYCMQIYTAYTTFLITFLTYPGEYPLVHSSVELRQSSYEIWNTEYIPTPQNEIEFML
MSYQYCELQFCIFYLGEEQNIALIGRSYLKYFLYLTKLHGRQLSLDXDEPALENYIHFSFKNP
LLLEHFDKFMGWLISSGIPYQFIKQIDEIGNNFVSEENASLKPMTLSELQGPFYFLVISLMM
CLVVFIAENINARRRVCIF
>BgerIr564P
MFKFFTSIKTVYAIHIIWYSSTFHCRKLDPGDELLDLHVDTAILNIANRFFHEDKVTVVQLL
SSQDCFINPKLDSRVGTTMLQKLSSEIIIFGCFIGARNVNNIVKGSSLVLVLPFDRSDSVINAL
LARIRILVGIVDIRLLIVLEKSPVSMKGKVVLELLNFSWMNSIVDAIVMLPGEDNFELYGWH
FEKQQDPCRRMLTDVELFDVWLGADKMFRNNTNLFGKKVVSTLDGCSLRFSLYNYPPFF
YFCHEDNNSQPIPTGLSFYVLFIFRKILKLDFELSVDHTGRFDISSSVTGFYDECCTIYPYYFD
RYVWLVPVAKAPTWQGIIRVFSTPLWVLIIITFLLGSFTFWIISKLKSGKKTSVSVTLMNTL
LTYLSVSSPMKGHSYTYRLLMSVWLFHCLLLYTLYQSQLIGFLAYPGEFPSIKTLTELDNTZ
LEKWSHTRMKEHIZZCZSKFISKNKTFRSPCDHFEICLKEFIKSEKISILTSEFIYEVYKAGSR
EKILNEFVLIMSPFLMNLSCLIVKRFDALVTRLFESGLSGKFVRNFTRWVRAVFNTIEDRSA
YPVPLSQFHSPFAFLIAGIFFAFF
>BgerIr565
MFKFFTSIKTVYAIHIIWYSSTFHCRKLDPGDELLDLHVDTAILNIANRFFHEDKVTVVQLL
SSQDCFINPKLDSRVGTTMLQKLSSEIIIFGCFIGARNVNKIVKASSLVLMLPFDRSDSVINA
LLARIRILVGIVDIRLLIVLEKSPVSMKGKVVLELLNFAWMNSIADAIVMLPGEDNFELYGW
HFEKQQDPCRRMLTDVELFDVWLGADKMFRNNTNLFGKKVVSTLDGCSLKFSMYNYPP
FFYLYHKDNNSKTIPTGLSIYILFIFRKNLKVDFELSVDHTGHFDISSSVTGFYDECCTIYPYY
FDRYVWLVPVTKAPTWQGIIRVFSTPLWVLIIITFLLGSVTFWIISKLKADKKTSVSVTLMN
TLLTYLSVSSPMKGNSYTYRLLMSVWLFHCLLLYTLYQSQLIGFLAYPGEFPSIKTLTELNN
SGLEKWSHTRMIKTGRTCDHFEICLKVFIKSEKISILTSEFIYEVYKAGSREKRLKIHKVDEP
EFVLIMSPFLMNLSCLIVKRFDALVTRLFESGLTGRFVRNFTRWFRAVFNTIEDRSAYPVP
LSQFHSPFAFLIAGMLFASLSFVAEKILHRIFHGIV

>BgerIr566
MFKFITSIKTVYAIHIIWYSSTFHCRKLDPGDELLESHVDTAILNIANRFFHEDKVTVVQLL
SPQDCFINPKLDSRVGTTMLQKLSSEIIIFGCFIGARNVNYIVKGSSLVLIMPFDRSDSVINAL
LARIRILVGIVDIRLLIVLEKSPVSMKEKVVLELLNDSWMNKIVNAIVMLPGEDNFELYGW
HFEKQQDPCRRMLTDVELFDVWITADKIFRNNTNLFGKKVVSTLEGCSLRFSLYNYPPFL
YFYHEDNNSQPIATGLSIYVLFVLKKILKLDFELSEDHTRRSSVTGIYDECATIYPYYFDRYV
WLVPVAKVPSWQGIIRVFSTPLWVLIIITFLLGSFTFWMISKLKAGKKTSVSVTLMNTLLT
YLSVSSPMKGHSYTYRLLMTVWLFHCLLLYTLYQSQLIGFLAYPGEFPNIKTLTELNNSGL
EKWSHARMIKTGRTCGLLENCINEFFKSEKISILTSEFIYEVYKAGSREKRLKIHRVDEPEF
VLIMSPFLMNLSCLIVKRFDALVTRLFESGLTDRFVRIFARWFHVVFNSIEDRSAYPVPLSQ
FHSPFAFLIAGMLFASLSFVAEKILHHIFH
>BgerIr567
MKIQKICNVSEVALVLWCVWAGSVAQCRSLKIPPATEERFSLAVLDIANRFFPKDRAIVVQ
IAASTKCPEEVLFSTTLATSVLTSLNQQMIVCGCVDFDEADLHFLKGGSMIILLPSLDSFTV
MFNASLEYLNRVKRKIGILDLRLLVVSEDAPSPESREDVAVGFLNAFWVLHSIADAVMVFP
MYTIKSSPLLVYGWTLEMQNDPCFSRLTNVAFLDRWIVKEKKFRKLANLFPSKEIRSMHG
CTLKLMMYDYPPFCFVIPGINRVTGSIPVIAKRLKQKLGLNLEVVPFGRSRPHMILPYMNE
CKTAYPYYFDRQLWYVQIPHWQGIFRVWTLVFLSFLLGSVTFWLMDCASLSQALLSTLES
FLSHSASFNGSGPRFGFLLVLWLFYCLQVYTLYQSQLISFLARPGEFPPISTLEELDASDLQK
WTMVITTDVSSAKSLAKFPVCNFLGCFRKLVAHKNTSVLADELFFNVCKHFYVTGGKPEV
VPINQPHRVSFVSTFFAVLRCQIVKRMNDVIGRLYDTGNLVGSFRRVNNFISIKLVKDEPA
MFPVALSQFQSPLCVLVCGLLVAFLSFLGEVLFHRY
>BgerIr568
MNMTLHLRLCAQYVSSKYFVPDNVLVISIPVVGKKNCLDNETHRVENLEPKNSKFIQIVNC
ESNFEANNATEEFTSFRKPYYMLSDLEDSLIAVLQGHSKWSIVVYSTTNSNKIKGQFKDKY
YIVVLYIRETLESTVKNASLQIETLKERLSWNPRARFLVMINGLYYDKSNTLPNELLKLFW
EFKVINVVLIIPDFCNVLNAYTWFPYSSRSTCMHVRAVQINKWIPEGNGKFYDTNTFLFPN
KIGNDFKNCSIKASTVEIKPFVDIEFKNNRTEIHGIESRIFKFLMSNLKAKYLIKVPDSDLWG
IKLPNGTWTGMKADIFNNVTEMGFGGLLLDTELCKTFDCTIPHFRDGLVWHVPRPKQIP
NWESIYRVFTINTWLLFLVICIAVAFLLWGLGKTEEDSYFITLDRSLSHMWATILSVSVPRL
PLTPRLRFLFVIWVIYGLHIDLLYLSFLTTFLINPGYERQVQNVIELVHSKLKYGYHEGFDK
YFDDTTDEILTTIRKHRIHCEGDGSICLKRMVTKRDFANLVSSSLMEFKINAEFFDHNGQP
LYNHFKNTFLGYDFVFYLSKGSPLLDTFNDIIFRISEAGLILQWWEEMKTELRLTYAKRDI
QEESSTLSLSHLVSVYVFLSIGLAISFILFICEIIWFSCYLKRLNRRI
>BgerIr569
MIMKAILLWLTLNVESTVAEFSLENLINVREVSYFAKCVVQIWQRTISSEQTLVVSLGVQN
TTNISSNYMGSKHEDTVKSTPERDLLTGSVHSLLYEDHFQILLMSAKSVEFDSYNFIHNIGG
YLIILEHGYLNDQLNVLKVQLVRIASYLYWNPKAPFAVLVKIINSKNELNLAEKFLEILWL
WRLINITVLIFNSELESLNAYTWFPFSDRKCGKEVTDTVLQYQWRKGRIDDLFLNTIFPPK
FSGNLNGCQLIVSTFSWPPYVFNTSLSSTELKYDGVEIRLLQDISRMMNFTVKFWPPPPGN
VLWGSILSNGTWTGLVGDLVYRRSDIAFACFRTKPERYKKLDATFSYSRERYVWFVPRPE
EKLSWESMFHIFPMTVWILILPSFVLSVFALYLLANRPKGANAENFHFKNIVSLILNAWGV
MLNAAIHVTPETNPVRMFFLVWIAYCFLLINAFQTFLVSVLTSPAFENGINNVEELLNSGM
EFGFMSHFKPVFNNVSDWTHLKILKEYVPCDDISSCLDRMAYAKDFAVFTSEKNGMFRA
TSKYVDKKGKALFKPMNQYFFAYNVAMYVEKGSPFLEHMNKVIIRDFEGGFLSQSWKHL

KHFTKLQAVFSLEKSDDNTLHVLSMDHVIGIFYLHLIGNIIAFLIFLLESTFYYCNKSLLKKY
L
>BgerIr570
MQSLIILFVLIISVAMNFTQNLVNKYNVLDCTKAVWSKELTESQVLVITRPQFAKLHNSILR
DLSNSSEEKLSKFSWNDGLYNLFRAVHNKYMGIVINTINKYNMDIENEHSFKNITYNLFLY
DKESKNTTDILKQLIKELTALRSYTFWNPHSKFTVSVIQEKQIIPARTLVKLLVCEMSKHFL
INTIIIISVDQNSLENVDEFQITNKERLVGIYTWFPFKNPKFSSNDEEIVLLDIWQTNKFVRG
KQFFPYKLKTNLRGLTIRAITAFPQPTVVDSVEKVHSNGSDTKKIIFKGGIGMVTLNTVAEA
MNFTLEYLDPTQANWGTWEGIMRLIYSRKVDLVFGGASIIIHWLRVADFTIPFHFGGIVW
WVPCAQSFPRWKSIIRVFSPNLWLLGLFSIIVAGIVMRLLANKHLKTTNLEWRLYQNVAN
CMLNSWAVLLSIGVSEMPRTKTLRMFFIAWVTYCLAVNTVFQTFLTSYLINPGLEHQISNV
EEMINSDLKFGVRDQIVEGYIKGLQDKKSLEISKKMFTIKIEDALKRVAYKRDISFAEGENLI
TNLRINYVDENGKHAICMIRETLTTFNIAYFFPKGSPLTEIFSKVLRRLFEANFTGVWIKSA
NDIKILKAVYRSRLKSKDDDEDEYCALGISHLQGAFYLLVVGYGITFMVFVSEISFHIYVPKL
HDFLNK
>BgerIr571
MVSKKNIGLQSRILNKSTEKDRAIIEYSPIINNIKLDLIVESRLYTNFLSNVSMIDQQIIKCITS
VVNTHFPKFKTVVVISEKDCALGQEVLSSLHLLSEWSIISTSGSNMKLRRRHGLRQACGFIF
LLDTDEYLRNLLLELESHPVWNSRARFVVAISAEVKSNTEKIIYGILNDFWQRYVINLVVL
QPLKENMTVQIHTWYPFTKTRCRGPVNESEIIDQWIPEGGGRFLTNAPLFPNKIKNLHQC
NIIVGTRPFEPYVLHSGMFNCSKTESSPCYKSGLEISILQTLSKSINFQLKYLDPPKGDWIEIS
EYTLNKTSDIAISAFSRSGKLTVNFDCSVTYIQDNIVWFTPRGKRRSPWENLILIFSQLIWV
LLSLSFVLFSVVTWQLAKLETNEHPCYQSAVHSFLNALVVLLGLTVKLLPKTTVLMALVYT
WILFSLNIRTAYQSSLISFLTNPRFHEPIRNVEELLNSGIKYGFYPGIRSWFDDPNDPKMNE
IFKNYIKCYDNIKCLNRMAYQRDFAVAGGKFNKMYLAAKHFTRSGKMLYLPMKINISFIYI
CMMFQKGSVFLDRFNEVIGRIITGGFIGHWWEEIKSKNLEPEIVNHANEYDDDDDHFQVL
NFVHLRGSFLLYTIGLSVAIIVFLLELVCNSFYRGNSRNKMEG
>BgerIr572
MSSAKRVLVGIWFLFLRDLNSELLPQTENSMTTNYNIVQCILNICSNGIQSDVTLTIVLPFR
KYYDEMCSLTNRIHFNTCYVTDSVLSKLYSSEKWNFLVTSSEYNSEYNSTFEQPSVQCYFIV
TGETLTENTSSVFSTQLQSISRNMWNPRAKFIVLVSTSINTTVNRSTVDNVFISDIMTQLFS
QNVVNAILVVPIVSSDPEKVYNLRDIEIHSWFPFTEDHCRGSVKSTVILNTWDKTVQKFNK
NTNLFPDKLLDLHGCVVKASTFPYSPHVFSNNAGNESGVTDYKDGLEIRIFHLLSEAMNFK
VHYLPAPKTVLPWYDVRDHVKNGFSDLCFAGFIHVQEHLGIYDSTTSHTTDTLKFFRPLP
QETPHWKSLIIIFPSLLWLYILIAYFIGSFIFWVLANIQKSIKEHFTYTNWMLCFMQTFSVIL
GEAVAVRPATWYLRVLFLFWVFYCLVINTAFQSALIDVLTNPRLEAIVTSLDELLKSKLKF
GFLIGTDYFYKMNDDPVSKYLVKHQVNCEVFDKCVRRMISKKDLVLCGGELNMVYLSKTS
YSKLGEPQFHPFQETVITSHLTFVLRLGSIFLERFDKLLFRMIEGGFVVKIWDDIKMTNLEL
EEEDSREFDGHSHHHQGPKSLDLDHLQAAFSLAIIGLLAGIAAFLLELMYFTFRRCATSDN
RSSAMSEMKVRSKTNYKNHFIYRTNVKNVNKR
>BgerIr573
MFSSFQIMNFLVLLLPTAAASFSSDVSKNMATCLVNSFQHFHKKSLVIILFDVPEDVEFQFL
IYELHLREEWSVSIKSGGHIRQDSSENFFVWAPHNLNRLMNLLDELALLNSRARIIILLHNS
NGIELILKKCREINMINAVLVEYPNSSSFMQAFTWLPYEPSGTCGRSDIKPIILDRCSIENGR
IFDRNSSIFPSKIPFDLQGCPIKIATFPWPPFIFNSRGTRMTDKVYYNSGLEIKLVGEIAKVL
NMSLQYLPPPPNDSKWGRRTDSGSWTGILGEVFHGRADLAFASLAATEDRLMHLSPSVT

YWSNAVIWVVPRPQYIAGWKGMFIVFELVTWTALTIVYFIASTLIWCVAKSSHICRESAAF
CKLSSCMTLTWASTLEVAVRIPNGQILKAIFICWIIYCLGVTTVYKSSLMSFLTEPHMGPPI
RTFEQLLQSGLPLGYTLGLSEYFDDPRTQDSLRFCPDIDTCLNYVAFYGNSSLVSDEWYVR
YLIPRLYSDRTGKPLLEILGEDVLSYHIVMIFSKGHILLERFNAIICRICESGLLEKWMHDIN
RNKYIDIDEELETESNWGRLTISNLQGPFLILIFGLCISSLVMFLELSSSKRFCITHFVKF
>BgerIr574
MEVISLVMLILSWQTPSLADKNIEYHLTECLHNIADKYITSVITFTFSKNSQRQCRTGQTFN
LLAKSENSFSNNHSLCYDHSIIKKFQESFKWPLIIRQSMVSVVQQEHFSSYIIILERCCDSDV
LNFLKYEVSKVKMNKYSKLIVAITQKVTKAKIIAENVLSILRQHRIVNTIVLMPSDSDVYQN
GIGSSKFDVYTWFPYQEEGYCGNFTNPELLDRYISTHKGQNFLFRNALFPYKIPSNLHACP
LVVSTFEYAPFVTSMKNDSNVLTYEEGIEVRFLDEFARRMNLTIKYREPSPDLWGVFLEN
GSWTGAAGEVSRGLSDMTIMGFWNKGDKPGLEYSTTYVIEHLNWFVPCAKPIPRWKSLI
RVFKLSLWLGFLAAYVIVSKLMSLIAKWSNNSSENQAYSSFGRCLLFFWAIIVEESASNNPP
NVTAIRLVFLTWVLYCLAVNTVYQTFLVSFLVDPGLEHQLSSETEIIESDLALGILESIGDCC
VPNFDRYPRRDPCEDYIRCMDKMAFKNELALIFAQYNAEHFIHTRYMGDNGLPLVCKLPQ
EISIQFIATPFTIGSFFLEWYDKMVHCAIQAGLFKYWWDSLEYTTTLTTAMKFKVDDEYD
MLSMEHLESAFIFLLLGSAVSSIIFVGEIFLSSNIWRKKLNIVKS
>BgerIr575P
MISVSNDSQNDCRIRDVFSKRRELLELTTNQNIVIKVQTSLKWSLMILQIAEVPVLEDVYCS
YILILNHZYINDIVQDVKSHISKVKINKYWTVHSKVILAVTNKVSQTHIIAHKVLTVLREYKI
INTIVLIPSDSDVHQDVTGSPVLDVYTWFPYSQEGICDNVTNPVLLDRWMSFDKNENFLF
KNELFPYKIPSNFHACPVFVSTFEYAPFVLNMKKDGNIITYKEGIEVRLLDEFARRMNFTIN
YREPSPDMWGMFLENGSWTGATGEVIRGFSNMTIIGFWTTGVMTGLEYSTSYLMDQLT
WFVPCVKHIPRWKSIILVFKLSLWLGFLSAYVVSKIMSLIAKWSYNSTENQAYGSFGRCLL
FFWAIVVEESASNNPPNVTSIRLVFFTWVLFCKAINTVYQTFLISFLVNPGLEHQISTEIEIIE
SNLALGITTSVADCCIPDVDQYPRRDRCGDYRKCVERVAIKNELAFIFGQYSGEHLIRTTYM
GSDGFPLVCKLAEEVNFILITTAFSKGSFFRDRYNHIVRHTIQSGLLKYWWNSLEYRISLTT
SKFKNDDDYVKVSMEQMESVFIFLVFAYTASIMVFAIEYVVGRNIQLSRK
>BgerIr576
MRICFLIILWYSSIFANKSIQNFMLNCLHHIADRYISSTIKIIVSNNTQHNCQIEDTSNLLSAR
LNLHDTTQPHNVIKKLQMVLKWPLMILQIEDESFFQEEFSSYILITDHCYMNNIIQDIRVQ
VPKVKINTYSKLIVLVTYKVAQPNIVAEKVLSVLREYKILNVIILISSDSDIHQDSHGSSIVVV
YTWFPYEREGCCGNVSNPVLLDKCMSSDKGDNFFLKNELFPYKIPLDLHGCPLVVSSFEYP
PFVLNMKMEGNEITYQEGIEVRLLEEFARRINLTIKYREPSPDFWGLLLENGSWNGATGE
VIRGLSDMAIIGFWHKGDISGLEYSTIYQIDYLTWFVPCAKPIPRWKSLMRVFKLSLWFGF
LSAYIIVSKVMSLVAKSSYNKTKKSSQEKSYSSFGRCLLYFWAIIVEESASNNLPNVTSIRLV
FLTWILYCLAMNTVYQTFLISFLINPGLEHQISTEVEVIESDLTLGILDNVASCCLSNVENYP
RRDHCKDFTQCMDRMAFQSDLALVFAQYYAEHVIHTKYMGRNGLPLVCKLPREVSFQFV
TTPFTKGSFIREIYDKMVLRAIQSGLLEYWWDSLEYMTALTTTKFKEKDGEFVKLSIKHLE
SVFIFLLLGYIGSSITFAFEFLLTSNTWSKGQYIAKS
>BgerIr577
MKFSFLVFLWYSPIMANENLQTRMANCLQYIADQYISSTIMVIVSNNTQPKCQIRGRTTSH
LLSEKLYVDDPTSNHKVIKKLQSSFKWPLMIFQISVGHISQEGYSTYILISDHCNNKDIMQEI
RYQISKVKNNKLYSVNSKLIVAITNKLPNPRIIVENILSILREFKIVNVIVLIPSEADVSQDDF
GSSTLDVYTWFPYQDEGYCANVTNPTLLDRWISSDKGQNFLFKNALFPHKIPPNFRACPV
VVSTFEYAPFVLKMKKEDNVISFEGGIELALLLEFARRMNLTIKYKEPSPDLWGTFLENGT

WIGAPGEVGRGLSDMTVIGFWTTGVIPGLEYSVTYLIDYLTWYVPRAKPIPRWKSLIRVFK
LSLWLGFLLSYVIVSKLMSQIAKWSYNSSEDHPYSSFARCLLYFWAIIVEESASNNPPNVTA
IRFVFLTWVLYCWAINTVYQTFLVSFLVNPGLEHQISTETEIIESDLALGITESVGDCCIPDV
DRYPRRDRCGEDFRICMDRAAVKNELAVIFGHYSGEHLIHTSYMGSDGSPLVYKLQQEVT
FLFITTPFIKGSYFRDSYDKVVHRVIQAGLLKYWWDSLEYTTTLSTVANFEEEDGEYVKLS
MDHMESAFIFVIFAYTTTTVVFIVEFITGTKICKINSRSNFETQY
>BgerIr578
MKVQMFLLILEFILYTFGFMRSNYVYCILDIAEKCFPYGSPVLVSIEATNINTPSFNQHFTNY
TSVNTIFNSLHSSEKWTILAYDKQKQFNTRYIERLDYYVLVSEGDKSVTSLNRTLGSLSKN
AVWSTTAKFIVTVNRIAYEVFRKMLTNVVSVLQYWKILNVVILSEHCISANIGVNTNEELN
RSYFEMYTIYTWVPNPNPNYLCKENGRLILLDTWLGNNEIRKFTRNFSLFPNKILQRCGLE
VSTFHYEPLVYRMNAIPDDGLEIKLFNAILQATKLTAVYLPPPPGEEKWGGLVNKTWNGL
IGQLINKRSDVVFAGLVNVLQSFNIMDFTYQYLFSQNRWMVPCPKPVTRLYGFAIVFSLRL
WICHTFVHITTAYCICLLEFRNKNEGIKSWFICSLELYCVFFGGSVLKTIPRRNSVRIAFISW
VIYSMIMATVYQTYLTYFLINPGSQISSENELLRSGMKILLNPVSEKVHLDLQTDKYKRREH
CVDLTKCLKRIIIERNLAMLCADQICDYIGITDNYDSNGDRGFCGLKERFSVIWVGMIIQKG
DPMISMFNNVILRVMQADLIGHWWEYIKHRKNLEFITNMKYVDYAHKNYLDTNSQLTR
KAAYFIILLGYISSVVAFTVEQIVRLRGRIKTFNY
>BgerIr579
MFMVTVSLYTIIYLTGVKSAKKMYSVISIYFLICGQSTISSSLSLIQCFRAISLQLPVNHTTVVS
YLSRPQTSDMYEKNEMVNLFLNYLQYQPSIVYNINSQPTWRTNTFHLGRYIIFISNDSNDE
NELNINLNSHLYFLVNSISLNPRAQFVIITAECAICGPRNTAKMILTYLWKWKIAKAIVLVS
VGRSKNASYEMYTWFPYHEFGQCGKSIDIASGKWLIADRYVIKMNSLFKNNININTQGCS
MRVSTITTSFFVGPPENEYSNSLNESKLVYSKGLEIQLLKLITQAMKMKDVYIPAPESPWIL
MDNDGNLTGYAKELYSDKADIAFGGLSMTGSTRQLTDVTHSYYWDKISWYVPCGIKYPR
WKSLTRIFSTSLWVCFALSVLLTVIIVVRLSQTSGSKECYAYRSLLDTLSNTWAVIIGISITLP
KSLLIRIFFGAWICYSFTINTVFQTYLTTYLIDPGYVPHFETLDQLINSDMKLGLTYFDAEIY
NNSADLYTERMMKKIVDCTFKEYCIQWALMYKNLSIVFSTLYMDVINIVSSSQWAANGSN
LLCRIEHETVNYMRFAMLMPKGSPLFPIVNNIVLRTMEAGLFNQWIEVFLHSLKIQWKMK
IRTGLSEEYCELTLIHMQSPFYLMVLGYGISLFVFICEVILHHIWYNVL
>BgerIr580
MSLIKVLVWLVMFLSIVNSYSERHFHIFRCVMNITERYIPLEHILAISWPIDFRNSSNRNLF
KIPSVSIEHYFFNGNSEWSYSVYRPVKQSTTVEETFFYGGYIVFAHFDFHDVSNSIDVFWYQ
VYRLFSGPSWNPHAPFIVALLNSSDYPPLVAETILKVLKMFRIRKPLVVVTEKLTETLDMY
TLFPHKCSSLRVVFLDSWAMEGDGFFVLHTDLFSNKINRNFQKCPLKVFTLTAVPSVGFPI
YTFSNKTQELEIQYEDGWEIQLLKIIAQKLNMTEKYILPDWEKIGPEIDFGRPLYDGEADV
AFSAIITREHTYYDVTRGYLWDTTRWYVPCGKKYPEWESIYRICDCLSWLSWIVSIILAAG
VLSFLSRGTLESTEYKSFAGALHSACSITLGVSVACKPKTLPLRWFFLSWVIYSYALDTVFQ
AFLTTFLIDPGMVPHITNVKDLVESNMALGVFNNHVFYFNNSEDPYGMMVMNKRIDCTF
NSTCFIWAAQYHNLSLLSTEMWFNNITITYESAGIPKGALCSLEDGDVETVYYIMLLQRGS
LYLEHFNWVIDGVLESGLFEHWKQMTFHKHKGENYTLVEQSSSEGYCQFTMDVMQSVFI
VLLMGYVFSAIALIGECLCFVLTRRSKLRLSRRFVNKRLLLLESRI
>BgerIr581
MIKVTVLLSMLICLPLLNGNNLNRPLYVARCILNTAERYFPPEKTLAISWPKTNISLVNRN
PFKIHTSPIDNLFFKELNQWSICIYRTKVKWHRELFFHGGYIVFSYYDFINDRRTTRTFMH
QMMQLYTSNTWNAHAPFVVVLMNAVDNPPLVAGFYLGAMKWHKIMNSVVIISDNKTG

NLNLYTWYPEKCDSFKVVCLDTWVMEDEGFYASNADLFPAKTDRNFHGCPLRAITHPAR
PSVGLPVYTRTYLNKTYKLNISYNDGWEVKLYKIILHKLNMTEKYVIPRFGVSGDFDSYLR
SDKADVAFSAYISRYESYFSTTRGYHWDTTRWYVPCGKKYPRWDSISRMFQWLVWISWI
AGVLLSGGILTLLSRHALEAESYKSFLGSLTYSCAVTLGISVHQKPKTSPLRCFFLSWVCYSF
ALDTVFQTFLTTYLIDPGMIPAVANMKELLNSDLAMGVSYKQLFFFNNTDDPTGMRVLQ
KWINCYFNRTCYEWAAQHHNLSLLATEMWFNNISATLEKTGIGRSSLCTIEGGDVETVFT
VMFLQRGSLYLDHFNRIIDGVVESGIFRHWTQVTYHEHKLLNRTNLEKGSSDEYCQLTME
LMQSVFFLLLMGYCIGVITLISECIYHSVSGRTQMGLLDPRYS
>BgerIr582
MEYVKVIISLVLFLTIASSHLNRHFHIARCILNISERYFPADHTLAISWPKDFRTVTKTKKLK
THSPPVEEIFFEWISQWPVSIYSTFKTVTWVGERFFYGGFIVFAYFENNNNQTDAVDVFW
NQILQLYAGHSWNARAPLIVALMNTFENSPAVAKKFVRVLKRFNIKNSLVIVTDKETKTL
DLYTWFPRKCTSFKMNILDAWVMDNDGYFVLNADLFPQKTSRNFHRCPLRVYTLSVKPS
VGDPVYTYSNKSGQWKIEYEDGWEIQLLQIIAWKLNMTQKYLEPFWSSEPDGGLFTSLDT
DNADVAFAASITRHMSFFETTKGYLWDTTRWYVPCGKKHPQWDSISRIFHWVVVWLFWF
ICILLAGFVFSFLSHKTIDSKVYKSVVGSVSNACSITLGISVSQKPKSLQMRWFFFSWVCYSL
AFDTVFQTYLTTFLIDSGIIPYIRNMEELIESDMTLGISENHVKLFNNTEDQSGRRIIERRID
CTFNSTCYLWAVQNQNLSILATEMWFNDISSTFESSDIHRRSLCTIEGGDFESVYIVMMLQ
RGSIYLDYFNHVIQGVVEAGIFMQWTEMTYYEQKLQNTTQVDGTSTNAYSQLTMDMMQ
SVFLVLFMGQVLSLFALIGECLFRKMTTSKKQRRFLHIVN
>BgerIr583
MLHEAKCAAVVAILVLNTACLSHTHEEPLLGCLVSISNRHFITGRTLVALLSKEHSLPQRSL
AHNIVNLNKVEILLLRHLHEANKWSILFSLLPLTYSSVCTDSYDQHGSYMFVLNKETVGEF
DAYLKLVLEAQCWDGKTIFIAVIDFTDVQEMLLDQVLQVFKKNRINNSAVVFMNSTFGVN
VLTLVPNQFDPKNSECADVLRTYSCDEYLKTTLDVFCTRGNLNGCPVKIATTPLPPFIGTP
VINSSHPIDINYKEGSEIKMLGLILQKLNATPRYISTEGDFFSETDERGNITGYAKSLKTEAD
VAVGLFFHSSQVLSKFDVTNAYYKVSWRWYIPCPMKSRSWESIIKIFSNEVWLSIILALIVA
ALVVVCIANKAEIIDVDEHCIYKHFSGSILSIWAMVLGVGVTLMPFSVPLRILIFFWLCYSLA
IDNVFQSFLTSFLIEPIQIPYVNNFDELLNSREKYGYLYFMDRTFESSDDWRSRKILKNRILC
TDIVACSKWIAHRRNFSMMYLDSLFSYYSSKGMNVDPNGHSLICQLTGGKVNNLYIGMAM
PKGSPLLDLFNCIIDRTVEAGIFMQFMELIDHQEKLKAGIVKGRSLADDYCDISLVHLQGVF
YLLLFGHGVSLVIFAMEILISRFWFGLLFSQNRVTQFRTTFDS
>BgerIr584
MLQETLLWLLSAANTSSGVQKQLINCLMTISSTYFLQNQTLVISLLKSPENSMTEQKVLSH
LHKVQLWSHIAFIPGIPSSNAFNISDMKEISQKNYIIFMNEQENDSLVRSLSIILQQMNVTAS
WNSQSYFVISLYKTISKDSIKVLLQFLWKWRVINVIILTRGFYEEMECIDVCSWFPYSNTDQ
CSSVKYVEVLDKWILENGSFMNNKYLFPNKLTKNLNKCTLRIMPRLTPYLLVETPIEVSEK
LLYVSGWEVLLGKLIARYLNMTEEYLSLSHNTLSQYGREIGELDALMKDEVDLVYGGEDIK
ETNVGNIVSTRAYHWDIWSWYVPCATKYHTWESIFKIFSLGLWFAICLSVIALTVAMFLIS
KHIPAEKPIYSKPVFCLFYVIAIMTGVSISGMPVTIHLRIFVFLTVCYFYAVSTVFQAWLTSF
LTDPAQSGQIKSIQELLDSNIRYGYVSYLEEYFEKDTEPQSKQILLGKTICPDLIQCVNWVGK
YRNYSLVYTNTLENFLRSKSVLQEETDRSLLCKIENGDVLPITYAMVVLKGNPLLCFIDNVI
TRIVESGIFLKWKEDSFVLEKILAKSFGIHTMASEYCDLSLSHMQPVFFILLFGDGLAFITFIL
ENIFPKFR
>BgerIr585

MVRLLVYIMTYSDVLATIPLYLLSQHMSSAVMYIGENYFPISQPLAVSFPHLREFQDPGKKE
EGEEIFIGNLHVMGLWPIIVFGDQRKFYYDFTNSGLIGSYIFIVRGLCDNLQSTVELFNKRIF
ELLVHSWRSWNPKAVFLVCITSNCDVQRQKLLARYFLSILWSVRVGKAMILFEVNAYAK
WTVYFWYPYTTAMTCSPDDDLRMCDLEKEENKMKSCFPLVSSYGRNYQGCPLKIIPRISE
PFVSLPKNAYNNSTTIFYDDGWEIKLIQIISKQLNMTEIYLRTPEQFLTTNLGAVLPLLGGM
VDIALGSVDAQQVVPTIETTASYLTVKEGWYTPCARKMPRWSSFFRTLSGVVWFIVSLSLF
AATFIAVYIARREEIELTSYKKYGTTFIYIWVIILGESVPAIPHSTPLRLFFLAWVLYSFVLNT
VFQSSLTAFLVDPGYELPIRTVEELVKSGTKYGFFPGRDPLFNDSSDILSAIILKNRIPCADP
DLCLEWASSYGNITTLHFELYVQHKYWDTSFVDENDKPKLCELYDGTVYNIRIGMLMTKR
SPMLDHVNYVLNMVIEAGIFIQWKKSFFDMVRIKARGKSQKSLVDSYFAMTVTHFQSAFY
ISLLGYSLAFLTLMIELICFNIFEIRNRDSHKCNI
>BgerIr586
MNIPAEHSKEVILCMNNIIGAALEQVLSPIVVLPEDNQSQTISTILHGRIQVIPHMMTTRDT
FCSTQLTGSSRDIYIIFIDSSTNTEESLHQLTNQMKCLSNKSIISSKSRFVITILGHHSTTERVL
RLIFQYFYKFNVLNIIVVVRQYLNSDVFGIYTWFPYRHFGKCDLVDDVTLLTECPKQQVLL
HSHLFPYKIPNNLKGCPLRAQTCQFPPVTGKVILTKGTDATNDVIDYEDGWDIRLLRIIQS
MGATLKFIQPIPNTSLSNIQKIGIWTVINAYLETKEADIAIGGLPISHLVTENIDMTIPFELSP
QFWWVPCPSELHRGLSLLRIFQPIVWLFFFFALISAALILQILGNITESSRIYKSFSTCLLNIW
AMVVGVVQNSVPGSNTVRAFLLSWMCYSFVVNNILQSYLTSFFTNPGMEKPLTSVEEILDS
GLGIGYAGKLGSFLKDDDESVSKRILQGQPYLINKNILDRIGFKKNYAILEGEKMMQYYRN
KLYIKENGDHLICSVRDVYKYFMYGIGLQRTSPFLDRVNIILRRILEAGLLNQWYEETNHGL
RIAAAHRANESEEKELQQMQSAFVLMCLGHSLAFAFLLLELLYKRLIRKGIQNLRN
>BgerIr587
MYTNVCLRTITVVFLYFRFCAGYESTIQQTYILNCIQNITLKYSDSTVVISFSNENDFDQNSI
PNIFDPQQKTLGQDFESSLVERISEFLPVVTLQKSNNDVSTESVDKIKYNVYIIVVDITKYNL
KEQINSLQLKNFWNPNGKFIVGVVSDENLVKYEQLQDILHELGSYKIYNVITVSFNNSLKE
EITVGDEVLSSVGVNSWFPYEPGNKCGEGTNIKLLDVWTNEKYGTFLLNSDLFPEKAKNL
NKCPIYASSQAYKTIDEEEEIYRKNVTYVMDKYSYTLDSEIRLIRLISEVMNATLTQTFEPG
YFDKMSIVESYMACLVSISVGGLADVTYMFHPLMDALSKVADFTKSYLSNNLIFVVPCARA
LPRWQSIVRLFSPLAWLVIFFFIIVSALTMHFIAKYESKIAISDTREYLDVSYYLCTSWSVTL
GISVAQLPKSTVLRIFFAALVSYCIALNTIFQAFLTMYLIEPGYEHQIQTLEELLLSRMDYGID
KDNMGLLEGTDDSQLATFAKYSKHCDALTTCIPKVAHDRNFASILNKYKYEVLSSTTFVD
EHGRRLLCTLEDKVQTIRTVMYLSKGSPLLGRMNEIITRALEGGIFGYFSKLFSDHLEIQYG
NVGINALLEDYCDLNLEHMQSVFLVLLLGLGVSMVLFAAEIMYARSRKSIHKVRNDVFVQ
>BgerIr588
MFSLLHFLTAMDTNSSLLLQCATNLLRTYVPPAHPIILFTSPEMESYTSQILRSLNSEILWTI
YLSTTGITHSQCCIKYLSYVIILEDFHSKHIERFDQQVEELQRIEPGNPKGLYLIILMEGDRN
TAKEIIQHLWRWKIIDATIMSPSQNGEKVVVYTWFPFKSKYECTEVEDIVSLDVCVQDGCF
LEGSNLYPEKVKRNLQSCPLVYSTRDWGILSIPATTGFIDKVPFNDGLEVILFREIAKALNM
TPVFKEPIASTMVWGDYDPTINSYTGVIGDAKFGRTNISFAGLPKNYFFEDHVDSTYSYLE
SGFHWYVRCPTSRERWAILIMTFTPPVWVVLIIAFLIIGFIIMFWAKLLNESEVYKYKTLSG
SLEYLLVAFLGMSVKLHPFTWEIRIFFFLWVWSSMGVTIVFQSLFTSFLVNPGLEKQVSSV
NELIISSLVLNFDPGYDALFEDSTERENLIISKRQFCVGFLECSKRTALVGDSATILDVQNYD
LYQQMFKDQFHTELLCRIPERISPYLISMYMQKGNPLFDEVNKVIILLMEAGFVDWWWK
DVVARQKIKTVSEDSEKDAIPYFAFSMNHIGVVFVLLGLGYAVSLCVFLVEYFLEKLYFS
>BgerIr589

MDRAINIKQFLSPLATALILLLIAQTLVDSNFVEEDYEPEDITFKVKCIENIMERNFAPNKSL
LFVSVAENQKLTTSLLKNINEAIHCPILVHRSKRNVKLSHMSDQKFGNCLIVKETAKYIDG
QLPRLMKLKNWNNQANFLIFITEPTKNANKTAFSILETFWNDAKVLNVVVMLVVDQQFE
LYSWFPYRSSTICGDVTEVFLVGKFPFYGGKCVTEEYELYPYKIPKKFHSCPIIIATYSDYIGD
NIELKYVKLLLESALNFTVHVVEELKENMYIGIDWMMRKIILDQADIALKGMPLLHRLYK
MADPSLPYYETTYQWYVPCAKPLSRVHSVSRIFALSLWLCICLSLLVFAAAIWVLSRFSDE
PSAYYKLPTLFCNIWAVVLGIGASQMPTTTRLRFVFVTLVWYCFAISTVFQTYFTSYLVNP
GFGTQISSLEELLNSKMAYGFRPEIINFIQDLPQPSYRDIIRGYEKCNSSEYCIGKIEQTEAFA
AVIESWYVDRILNTENYQSVCIMNKFETFSVLITSYFMKGSYFTDVFNKYIQMCIQSGLLGI
VQAERDKTKQHYTYLNEQDSEDEVFIFSLSHLLVAFSCLFLGSGLGILILCVEIIYFRFQQTKI
NPPLA
>BgerIr590
MHPTYSRTVFGSNISRLCLKMYFCDVGCLFCRDMKVFLLVLKMTLASASLIVPVGNRDYTS
MGSVIRGILPQQFSPSRTLSVAATSTDEELMNFALKKINELASWPVHVSVLFAQAVPTNHE
DFNKIGNCIIFIKTVDDIDDLLDELLSTTSWSGRARFLVVVTEIVSNPQITALAVVQQLWEK
ACIANVVILVRTGDIFRLYSHFLYQSEQQCAVAANVELLQEWHKDCEVCFHVERDLFPEKV
GANLHGCTLRVSTTEVQPHIINTGGDNYTGLEIDYLRVIQGALNFSVLFRTPSPGINYETHY
EMLQDLQLGLSDLVIGDFPLHLFIVQLAEPTVPYIENYIKWYIPCAAEANRIGKALKMFDSS
SGLTLLASFVMISFLVWAMAKSAGDEPSSYTSITAAAHSMFSMLINVAVSKLPRSLKLRTF
VLLVIWYFFAIATVVQTILTSILVNPGFERQMTTLEELERAGIPFGSFTDMELLLNSSTTFM
ADLKLPKIMCVDLGTCAKRVILDGDLSTVTVSSVIEQMSWSQLETGGVELKICSLPGDVFR
MSYTIYFAKGNPLIDRFNNVIRRMMEGGIAQKLFLDMKFSIRLHNKKDEATVRGEKYFVF
GLTHLWVIFFYLLLGSANAALCLLLEMIFAKIAKLSPKKRPTHFEFLL
>BgerIr591
MKHYISLFLHTVLTFDAGIGISNLHAQTAMCTLEAINRYFSPGKILTISFPASFHDDLQIVEY
LLKLINNKLYRTVQVSRMSDDSSDVNDEIGVPFTNQNYVLFSWTLNEEEYVTDNIVSQIEE
LQYTVSWNPRAKFIIVIAEVQPTSPMSLAGDIFSKLWEMNNILNIAIFIINYATDISSKVQEL
YRLEAYSWFPYRSGHCGRSGEIIIVDKCEVMMNSWQFPTNISMFENKLPEDMMGCPLKVS
TKDLDPFVILLNTVVDQEGKVQYNYSGLEIQCLYFIREAMNFTLEFLPPSEGDMAESHAEQ
LSELNAGLTDIIIGTFPLTALIVQFGDYSYPLTYNTLVWLVPCPEPVTRVDMILKVFKPSVW
LTIVLVLVLSAFTFWITGLNNRGMNATEPHIYRTVSQCVVNSWSILLGVSLSELPRMYKLR
TFFLLYVCYCFVMSTVFQTFFVSFLVSPGYAKPLVSLDDLENSDIGYGYDESIETFLMLSSYH
EHERLSHRIPCDGYDSCAKRLLRKGDIGFLNLRIFIDYMSAKLGISIEGSHIICTLDENVFYFN
TVMFVKSGQPLLDRINVIIRRITEAGLMMNYWSSLQFGLFLRKKGSNNEYSCDGCDSLYFV
FQLSHLQIPFLILITGVISGIIALFIEIIVKRCL
>BgerIr592
MECHIRIIVIITMYTEFFHKGIFSTFIGTQDSIHEEFLLCLETIITQHLTEDDSLLLLTPSTEDK
KRNLHDLSNTLIHINGNTFVNNLLRTVHSRGILKIQTIGTEYEETDIYLIPPDIYVMLVTEEG
FNLQSKTEDDAAKYVKEDEEVRESSEEEEYSIEELEIFMGEQLFVLQKMIIWNVRAKFIIIV
DIKKGPNYSFHSLALEICRVLGKQNIMNIVVMVPNFNNGTFYNIGVDMFTWFPYKNGTCG
EVENVIILDQWINRTFSEGLNMFPVKIPKQFNKCPIKLAAFGIEPFIVAVDNYTHNKDNGN
TYNFRGLCAEYSLIALEELNVTVHVEKVSNDVTFDNLVDTVGGLSSGKYDVLVGPIPWIAS
AMILDLSFPTLYFTVKFQVPCSLPVPHTERILKTYHVSVWLTTLLTLVISSLVFYLSAKVHT
FEESFTFKSVISCLCNTWAILMGVSVSEQPKSSVLRIFFYIYVCYCFSISTVFQTFFTSYLVEP
GYGKPLRSLKDLLNSGFPLGVSRPLLPVVDTFEFKEMSLFKTEEFESFENASFRVITKGDM
CMPSATQYAHFIACKLGIDDVSRTICFLEEDIVTLLFATALPKGSPLLTSFNDHIMRGFEAG

FLHRYWSLFTLSLILSSTGNFNKGNNEYLVLNLSHLSPAFIVLFLGFSVSFVIFVLEIVIYSIE
MCIGSSVPRRTNVRYISSNSTPRNGRGESFE
>BgerIr593
MDYHIRMIVIITVYTEFFHNAIFSTLIGTEDAIHEEYLLCLETIITQHLTEDDSLLLLIPSTTD
KKRSLYDISKTLIHINGNTFINKILRTVQSCGLLKIQTIGTDYEEADVYLVPPAVYVVLVSEE
GFNLQFTTEDDADEYITENKPGERRVSSEGNEDFIGELERLIEDQLFVLQNMIIWNVRAKF
IIIVDMKKRNYSFHSIALEICRALGKQNIVNIVVMVPNFNNGTFYSSGVDLFSWFPYKNGTC
GEVENVIILDQWINRTFSDGINMFPVKIPKQFNKCPIKLAALGVEPFIVFADNYTHNIDNGN
TYNFRGLSAEYAQIALDELNLTVHIEMVSNDATFDNGMDVIGGISSRKYNVFVGPIPWIPS
MMILDLSFPTLYVTAKFQVPCSLPIPHMERILQTYQVSVWLSTLSTLVMSSVVFYLSAKVH
TFEESFTFKSVISCFCNTWAILMGVSVSEQPKTSILRMFFYIYVCYCLSISTVFQAFFTTYLV
EPGYGKPLKSFQDLLNSGFAYGYMDSLEPVVATLDFDEMSLIEKKETYENYENASIRIIMK
RDLFTVSATQYANFIAYKLGIDDVSKTICFLEEPIFTVPFGTAFPKGSPFLSSFNEHIVHGFE
AGFLQRYWSLFTLSLILSNTGKFQNVNNDYIVLNLSHLSPAFMVLFLGFSVSSIVFVFEIIFY
CFNTPVP
>BgerIr594
MEYHKRMIVIIIIYTEFLQNKLFSSHVGTEETVREEILLCFETILTQQLTDDDSLLFLIPSTEE
KERNQFEMFKALIKNINGNSFIDKFLRTVHSRGLLKIQTIGTDYEEKTVYLTSPSVYVMLVI
EETSNSESTIEVDKDEAGRLGKSGEEEEDEEDAIEELERLIEDQLLDLKNMVIWNARAKFI
LIVAMKKISKYSSNTVALQICRLLGNWNIINIVVMVPNFNNETFYSSAVDLFTWFPYNNGT
CGEVENVVMVDQWINNTLREGSSFFPMKIPKKFNKCPIKIAALGIEPFIVSVDNYTHNKDN
GNIYNFRGITAEYPLIALDRLNVTVHTEMVSNDVTFENILEVTGGLVGGKYDVLVGPMPW
LAAALMVDLSFPTINLNVRFQVPCSLPVPHMQRILKTYQVSVWLSTLLTLVISGFVFYLSAK
IHKFEESFTFKSVLNCLCHSLAILLGVSVPELPKTRVLRIFFFIYVCYCFSISTVFQAFFTSYLV
EPGYGKPLRTFEDLLNSGFTFGFLDALTAVTATIDINEIYLFKERETIDDFENATIRIITKGD
LSMVSATQYSIFIANKMGIDDISKTICFLDDDILTLKLATALPKGSPFLRSLNHYIMRGFEAG
FLQKYWSLFTQSLILANRGQFKNVSSDYLVLNLSHLSPAFMVLLMGFPLSFIVFLLEIFLSFF
QNMMSRTVVQ
>BgerIr595
MEYHIRLIVIFIIYTEFLHKKLFSTHIGTKDAVHEEFLLCFETILTQQLTGDDSLLILIPSTGTI
VRNQHDKFKALININGNSFIDKFLRTVHSRVILKIQTIGTDYKESNLYLIPPDVYVMILAEED
EDDVDEFNIEEEAGIGRTLEDEEREEEEEEEEEEEEEEEEEEEEEDTIAELEMLLEDQLLAL
KDMVIWNARAKFIIIVDVKRGPKYSPDFVALQICRFLGIWNIINIVIVVPNFNNGFVVDLFT
WFPYNNGTCGEVQNVVMLDQWINQTFREGSIFFHKKIPKKFNKCPIRIAAIGLEPFIVYVD
NYTHNKDIGNTYNFRGLSAESPLIALDMLNVTVHTKYLSNDASMGNALDVSAGILGGSYD
VLICPVPWIPSTMILDVSFPTIYFNLRFQVPCSLPVPHLQRILKTYQVAVWLSTLLTLVISGF
VFYLSAKVHNLEEAFTFKSVVNCLCHSWAILMGVSVPEQPKTRVLRIFFYIYVCYCFSISTV
FQAFFTTYLVEPGYGKPLRTFQDLLDAGFTFGVLPSLGILMSTVDFSEVSLFKKEETFNDFE
NATIRIMKKRDLCMISATLYSNYIANKLGVDDISKAICFLDDDLLGAKLGTGVPKGSPFLAS
LNYYTMLGFEAGFLERYSSLFTQSLILSNNGGYQTVNSEYLVLNLSHLSPAFMVLLMGFSV
SFIVFCFEICFYSFEIWKAHPVPRTKDVMFIPSTPSPRKLQKRRKYWIKIRPTPSQIMTARL
NNTISYDFS
>BgerIr596
MEYHTKIIVIFIICTEFLHKKIFSTHIGTEELLLCFETILDQQLTHDDSLLFLIPSIEEKERNQY
DIFKALINDHGNSFIDKFLKTVHSRGILKIQTKGTDYEDTTVYLIPPDVYVMIVAEENEDDA
DIQNEEAGRVGKLAIEELERLLEDQILVLQSMVTWNVRAKFIIIVAMKSISKYSSRTVALQIC

RLLGNWNIINIVVMVPNFNNQTFYISAVDLFTWFPYKNGTCGEVENVVMLDQWINGTFR
EGSNLFPMKIPKQFSKCPIKIAALGIEPFIVSVDNYTHNKNNGNIYKFRGITAEYPLIALDML
NITVHTEMVSNDVTLDNVLDVLAGLVSGKYDVLVGPIPWLASAMILDLSFPTIYFNARFQV
PCSLPVPHMQRILQTYQVSVWLSTLLTLVISGFVFYLSAKVHKFEESFTFKSVVNCLCHSLA
ILLGVSVPELPKTRVLRIFFYIYVCYCFSISTVFQAFFTSYLVEPGYGKPLRTFEDLLKAGFTF
GSLPALTPAVASIDFHEIYLFKKEEYFYDYENATIRIITKGDLCMMSATQYSNFIAYKLGIDD
ISKIICFLDYDLITLYFATALPKGSPFLPSLNYYIMRGFEAGFLQRYWSLFTQSLILSNTGRFQ
NVNSDYLVLNLSHLSPAFMVLFLGFPVSFIMFVSEVTFYSFKIRMRNSVMRHK
>BgerIr597P
MEYHFRLIVIFIMYTEFLLNKIFSTHMAXEELLLCFQTILIQQLTDDDSLLIVIPSTEKIVRNE
HEIFKTLININGNSFIDKFLRTVHYRGILKIQTIGTPZEDSTVYLIPPDVYVMIVAEENEDDA
DESIIEEEDTIVELEILLEDQLLGLINMVIWKARAKFIIIVDLERGPKYPPDFVAZQICRFLGK
WNIINIVVMVPNFNNGTYYSFVVDLFTWFPYNNGTCGEVENVVMLDQWINRTFTEDSNX
FPMKIPKKFNKCPIKIGALGIEPFIVSVDNYTHNKDNGNTFNFRGLSAEYCLIALDMLNVTV
HTEYVSNNMSVDNVIDVVARTVDGRYDVFVSPIPWIPSAAIVDLSFPTLYFNLRFQVPCSL
PVPHMQRILKTYQVAVWLSTLLILVISGFVFYLSAKVHNLEEAFTFKSIVNCLRHSWAILLG
MSLPEQPKTRVLKIXFYIYVCCCFSISTVFQAFFTSYLVELGYGKPLMSFEDLLETGFTFGIM
PSVARVMETIDIHEIVLFMKTETFHNLEDATIRIIKTGDLCTMSATLYSNYIANKLGIDDTS
KTICFLDDYLLTVKLATGLPKGRPFLTSLNYYIMLGFEAGFLHRYSSLFTQSLILSNTGEFQ
NVNSDYLVLNLSHLSPAFMVLLLGFPVSFIVFVFEIAFYSFKI
>BgerIr598P
MFLPEHSLCDSGTILTGEYKHRGILKIQTIGTPYEDSTVYLIPPDVYVMIVAEENEDDADGSI
IEEEDTIVELEILLDDQILGLINMVIWNVRAKFIIIFDLERGPKYPPDFVALQICRFLGKWNII
NIVVMVPNFNNGTYYSFVVDLFTWFSYNNGTCAEVENAVMVDQRINHTFREGSILFPMKI
PKXFNKCPIKVAALGFEPFIVFVDNYTHDKDNZNTFNFRGVSAEYCLIALDMXMSIDKLVD
VVARTVDGRYDVLVSPIPWLPSTMIVDLSFPTIYYNVRFQVPCSLPVPHMQRKLNTCQVSV
WLSTILTLVISGFVFYLSAKFHNLEEGFTFKSIAKCLCHSWAILLGVFVPEIPKTRVLRIFSYL
YVCYCFSISTVFQAFFISYLVEPGYEKPLRSFEDLLDAGFTFGSMPLLATLVATIDFHEIFLFK
RTEEFHEYENATICIMRKMDLCMMSATLYSNYIANKLGIDDTSKTICFLDDDDVLTARQG
QDYRKVAHSZQVZIITSCLVSKQVPSKDIRLYSHKVZFLSNTGGFQNEKSDFLVLNVSHLSP
AFMVLHLGFPVSFIVFVSAICFYSFKIWVVIPVPRTKSSPSLQLHLLVNCK
>BgerIr599
MTKYMDLFTRLLLLIIKEIRCVYTHNNGMVIYIEEIITAHFNPDFDLIISLPNIGDTYGTRILK
NTPYEVSDMQFEWKMLSAINQVTLWRIEVQGSSETYIDVMNHMLPKAGNYIIIFTFNFVD
DINSLLEKQIVDLRDTTHWNPRAKFLVVVTNIDTGTRKDIAFIISRSLKKFFIINVIILIPATI
VNPERRELEELDLELYTWYPFKEQNCGELKSVELVEKWNISRIHKFKNITEFYPIKIPKYF
MKCPIKISSFGIRPYIIPIEDRTYEETNNLYNLSGIMVEYIINAIEEMNLTAFFYPPVISTNLLD
AVETVKLLGISSDIVLGPFPLLSLLESMFDHTVPYFFDSVHWLVPCPGPISKVERILSTYSES
VWIAIICTFSCSALTFWFIAKRDSDNKESCAYVGMSNSFYNTWAISLGISVTKIPIDTTLRLF
FILFVWYSFAMSTIFQAFFMAYLVEPGYENPITTFEELIASNVPNFKVQSLDAVFTVISSGIL
RKFGKNRVTCVDYESCVKAIILHRNASIVTTSAYSKYIANNILSGVDKSKGVCNLNGVLASV
GMIALLDKGSPFLERINVYVTRSFEASLLKRYWSKIMTNEAFIFKNKSILKNDSLYLVFNLS
HLAPCFFILVFGYIISFVVYIVEVSFGFSKKN
>BgerIr600
MPHVDLASEFDIDREDIDMISCLEKLFHNYFVRSRPLVVSLPPSEPILTPRYICNLRPYNDIS
SLANNILETLNENMKWPIQVSSPSSLDEIPELPHLHQSYLFFLWKTREDDTLIEVLENEVE

MLRYSPSWNPRAVFIVIMTYYIDEDAKLMALHVHKSLWEEYKVVKVLVVISNSVKDLQYR
NYSKVQEEYKGLELYTASPYSPGRIECGTITDVISLSALCSTISEQPYMESYVFPSRTMNNFH
GCPIRIGIILLDPYVSEVDNYTNVESSLKYRFRGISIELPLIAFEHMNLSIHFLSPSKGITLDSY
THEFGNVKIGDSDILLGAIPLMPVTVTSSFETTVPYVFEQLKWFVPCPRPIRSVQQIILTYTI
SVWCLIAVVIMLVSCTIWLLGKRYNYSISEYLMFSNLYDCFYISWSIFIGVSISKMPKTSMLR
TLVFLYICYSFAIVTVFQAYFMSYLVEPSYEKRITTFDHLINSGLAYGANDGFKFLVQTMAL
NISARFGTNYINCGSMSTCVERVMSSRDITTLCTNGYAHITAIKMGAEHVEKVLCSLDEKSF
SGSIIALVPRGSPFLERLNTLISRSLESGLFTKFWNEYTLNILLSTKAKFDNDSDEYVSFEIN
HLASAFWVLVIGLGLGCVLCILEIAYHLTLI
>BgerIr601
MPTYYLLLLCLMTTYVCSVMDVLRVTDTNLHRSMVSCVENIIHKHFLSRITMTISVSYSDS
DDIKISSDMCLIDMIFETVHQFCSWPIIVSNSGIEVPESDIAVLQYGYIILLLLDSDGLVTDSL
QTQLEILSESPSWNPRGKFLIVLPNAGNESAQSIAFTICDILWDDYRITNVLIMTPNVDKRE
NEIEEVDTYLFDIYSAFPYRDGSCGKINNVMKVDQCFLGKHTFLKNDNLFPNKVPTNLNK
CPVVISTVGLEPYLILSRNYTENLNRDLFRVDGLGTELLKLSAEKMNFTALFLEPTFTVDA
RLIIQEVTRVQNGVSEVLAGATPLIANTLAFFDGTIPFIFDTINWFVPCPKPIARMKRVLSV
FTVSSWVAMAVVHLVTSVVFCWANLSSEGREQSFQSLSDALLHTWAVMMGLSFLHVPKA
LHLRALLILFILYCFVMSMVFQAYFKSYLVEPGYEKRLETIDDLVESEFLYGYIKMAEVMAS
TMEYSELKRFASRRTECKDLRSCTERIMFNGDLISTISFNYATYIASLRGVRYKREALCYLE
EPILSGFICVLLRKGSPLLDRFNQLFRRCLEAGLLNRYWTDLAFRTNLENIEIIENESDIYFV
FSVEHLEPIFWLLICGYGLSFTLFLWELILWNYLRYKDKLNHKKGNHQNFSLYNNGNYYF
YV
>BgerIr602
MLKFYKYILLFEVIYFEPGSFLSYEQIFQISSCLEKILLQHFNTTAPIKISLSNTILEFGEKPNK
LTASDKYNDELLDHFLQKINQDFKRLIMISVPGAEVEERDEGKAAMQVVLDQGYVFFVYIS
DPEDSLTDMLDYQVPPFKNTFSWNPRGKFIVVIMHENLQKDDIKRFAKAAFETFWDLHK
VFDVLLIIPKFQQKNTLDDLEADVLDLYTWVPYSDTNCGVVNESLLVNRWLFEEGGKFAE
ERDVFPNKLLNNFNGCPLYVVPVGFEPYVMQLRSFSREDGSEGFDMKGLGVELIFSFANK
HNFSIFFQKPVMEFQSFKIMDIGGLLLSGQVDIIVGIVPVVAPLLAFIDLSRSYMYESILWMV
PCPKTIPRMERVVTIFTVSAWIMVASSFVFASVVIWFEARSGEENKNYKTTGMCFYCSWA
VFMGVSVPELPRSDKVRCFFILYVCYCLAIITVFQAFFTTFLVEPGFKNPMKTMDDLVASG
TEYGFVSILEMFLETTTYEEHRRLKKRKYCNELPPCVKQALFKNDLAVVTAVSFTSFLASE
AGMSEKHHGCYLDELLISSGNSFGLAKGSPLINIVNNHLTKSFEGGVLDAYWSQLKHETRL
KANRTEEVMYFVFSMDHLSPVFYLLIIGLVFAFIVLFLEIIMKFFFKVNLDSTTTVELFKKV
>BgerIr603
MKYIKILFLMFIPCLKPLTCFIITNKSFCYHNSICVASCLNEILNQYYTSKQTILISSSEIKHEE
TSNSLSQISLQDGLFQTYEAVIKMITRTGQWPMLLYNAQLKLSNEKEPFFHNLNIMHVSSD
MNNDIDNETIQAQLEILQDRKSWDSRGKFVVIVHTSTSNGHNLALKICDLMWTLAKVFNI
IVLVSHFNEETELSIKDSNMNVLNIFTWFPYDDNNCGEIHQIVIRNQWLCNEEVFSMNSNL
FPSKVPRDLHGCPFRISTLGIEPYVILTKNDSNKDESPIYKVGGLALEYINLSIEKMNLTAIFF
EPTKKITFNVFFEHARSIQTGKSDILVGTVPFMSHLFDFFDPTIIYIFDSLKWIVPCPRAVPR
MEKIWKIFTLPVWIMTDLAVILVSVIFYFKAKNLFHTQNPDRHYNSFPQCLGYFHSLFVGV
SMPVIPKSANLRFLFITFCIYCFSFTVIFQTFFTTYLVDPGYEERIEAVDELGKRGIPFGFVSG
YKILLEFMGYEEFEEFVSSEVVCEDIKLCLKEVLIDGNFSTITSLYYPYYIASKMGVEDIEKAV
CFIDETLITGGITAMLQKGSPLLNRLNTLLRHCMECGLLDKYWSELNFGLKIKKGDGMLD

DNHYFVFSILHLQPIFLIFLFGLMISFIAFITEFFFGIFYRKKAKLDLVKYICYYKWKLMQAF
HI
>BgerIr604
MELSNITVLMCKRKEMLHHKLFLSVLIVSTSLERRSVREEDIASCVNAVFLSSLPVGRPLHV
SMATYEESTFKHSLTFCENVGLNLIDLMIGTTHGYLNWPLRITNVGKTIEMDITIDELVYL
TGGYLLLLFPNEDGEVYELEDQLNELQYSRSWNPRAKFVIVVPDAGNTPPKDIAMKVCDV
LWTFMKIINVLIVIPETEDFNSEHSNCGAEINRQRVPIYSWFPYDENNCGDIVDNVRLIDE
WICDNCSFHEKSNLFPSKMPNNFKGCPIKVSSIGKPPYVFLRDEHHFEHESSYDTVGGWCL
DFVYLMGTVLNFTLIFHEPLLELNKDSYVSIFTSLANDKADIAVGIIPNLVGVVERFDATIPY
DIDNVEFLVPCPSKIPKAERVLGIFTFSVWAFLMLSYSAVGITLWGLTIGRDESFNFRTISNS
LYSCWALLLGLSVPIMPKTAIVRSMFLLFVLFCLVVCYIFQAFFTTFLVEPGFEKKIETLEEII
KLRIPYGSVKFISKFSSIDKGIANYDKIVLSDVDCGFHYESCIRRVINKDNFFTIVLKRQAQYF
ASELGYKNLNRDVCFLDQKVTSGGIIFIFRKGSPLVPLVSSLMRKSAEYGILDKYWSMLKFE
ALLNSNVTKEEEDSYFVFSMDHLSPIFIFLAAGCIISSIIFILEHLLNLIVIVSERKRLFRSNKN
DCTKIRRRRQLPSNLRNQQRWSKIGRPTIEQFQYIVTIPWE
>BgerIr605P
MEVTTLCQQLLIISTTIVLSKIEPGHISSYVERILEDSFLPGLPLIVSIRSVSNEIVHRSLEQGT
YYEVLPKLVDLLLRKVHSHITWPIEVILPPIETLLSDEITYVHQGYIIFLWTNEYNEIEYLEE
QMHELQYTKSWNSRGKFIIIIPDLGKESINSLALNISQTLWRFSKILLXLIIIPD-
QTYDSDKSL--RNMSNEGISTFTSFPYEKENCDGTINTVKRIHLW--
NDCKNDLKSNFQLFSNKIPRNFQECPLRVSTVGIPPYL-
ITSHKIVDSKEIFTHVHGYSLEFIYILSELFNFKVIVNYQATQMTSEAYLKMFNHLFEDESDL
GLGIIPNAARVSDLFDMSIPYMFESIKIMIPCPNQIPRTERVFGIFQISVWVVLIISVIVISLVQ
RQLEIFHEDVHCFKSISSSLENSWAVLLGLSIHQLPKTLILRQHFFLYVLYCMVISTIFQAFF
TTYLVEPGYERQLKTIDEFIESRILYGQVQIFAKLLQAETAIQRYNKFTFSGIDCGDDYYSCIE
RIIAKKDFSALCTHSLAYYVANKMGFRDPNKVTCFIEEPLISGGNVVIFQKGNPLMLLVNEI
YERIIEGGIVNRYMSMVNNEARLRSNYT-
ENNNAYFVFSLSHISPIFAFLVIGYAISFIVVLGELFINMDWVKRFKQRAATLY
>BgerIr606
MIRSTVVALLIALTVSADNGLLNSQQTNFISCLQEILKKHFSSTMPILVSFRSSCPLDVSRNL
NTSSSVQDPLNVVNAVLKNINNLTTVLVTHATTTAPMDFQEDGVLHQGYFVFLSTCEGSK
VDEEGDVDDEDREDYEQDLVEALEIQFLKIMDSKSWNPRAKYVIIVLDNTGMDTESIVLEL
VKSFWSLARMENFILIIRNLEGDHQYDLQENAVLNLYSWFPYINGTCSEVDKVMLLDQWV
SSNGGHFSKKLDLFSYKLPRNFGSCQFVISTIGPEPYVKVARNYTDGNENQVYAVEGTGVN
LINLFAKDHNFSISYRPPITNFDIDEIIEESMLALGGSSDILCGYVPLAPAVVDFGDVTFPIAF
EAVVWIVPCPKPLGRIRRVVGIFTASVWATIILVIIFSSYVLFLQGRNYLKESFGFKNFSRCLS
GTWAVLLGVSVPELPLTHNTRSFFLLYVWYSMAISMVFQAFFVTYLVEPGYGERMSSLDD
LRRSNVPYVSIEIVDILLSQSSYTGHLLIPKYEGNIEFIDCIKDIMFHRNASTIVAVTYAYFVA
EKNGVSEVDKVVCFLDERLFYGPFSMALVKGNPLVPLLNEYFMRCFEAGIQESYKSMFKY
KTRLNAEDLYDEMEMYFVFSMQHLYPVFLLLIFGCIVSSITFVVELCSHTLHVCFHNNLLM
NCKSM
>BgerIr607
MQQASRSFTCSNMNLSRFLVTLIIASVSADSGLLSFQHNHILNCLQEILKNHFSSIMPIFVSF
SSSCPVNCSRNLNISFSVSHDLDLVNEVLTHISNVTTVLSYQSELNEGAIFPYDGILEQGYFV
FLSSCQQNEGEYDGLINDLETQFFYIIRSFSFNPRAKYVLIVSNNQETDIESLAFELVKTFWS
LAKMVNFILVINGLDAAQQHVLNIYTWYPYFNGACNEVDKVVLVDQWKFSDGGHFSRNS

DLFSYNFPKSVDACSLVVASVGPEPYVVFLRNYTNEDGNPEYEVEGVSLNLITLFGKDYNF
TIIYRPPITDFNAHDLLFEISLIFAGSGDILTGLTPLVAPAVAMADITFPIFFESILWLIPCPQP
LGRIERVVGIFTASAWATMISVIFFASAVLLLQAKHYKKEVNGFKSFPQCLSGAWAVLLGV
SVPEQPLTSNTRNFFLLYVWYSVAISMVFQAFFVTYLVEPGYKEKMITLEDLQRSNLSYAS
VQTIDFLLTTTTYTDHYLIPKLKEDLDFYKCSEMVMFNHNLSTMGFPLVAYFIAEKRAVRE
INKVVCFLEERLFYAAIGMGLVKGNPLVPVLNEYFSRCIEAGFQENYWSMLKYRTRLKAQ
DIYDEELMYFVFSMQHLNPVFLLHFFGCIVSAVMFAVELIVSRVSSKLCK
>BgerIr608P
MNLSRLLVTLIIASVSADSGLLSLQQNHISSCLQEIFKNHFSTTVRMFVSFSSPCPVDFSRNL
NISFSVSYDLDLVNAVLTHITNLTTVLSYQSELNEGAIFPDDGILEQGYFVFLSSCQQNESEH
DDLINDLETQFFYILRSLSFNPRANYIIIVSSNHGTDVGSLAFELITTFWNLAKIVNFVFLIN
GLEATQQHVLNLYTWYPYLNGACNDVDKVVLVDQWKFSDVGHFSRKSDLFSYKFPKSVD
ACSLVVASVGPVPYVVLLRNYTNEDGSPEYEVEGISLNLIILFARDYNFSIIYRPPITEINVYK
ILLEMAFIFSGSGDILTGLIPLVAPTVSLADITFPIFFESILWLMPCSQLLARIDRIVEILTASA
WAIMILVICFASAVLLLQAKHYKZEVHGFKSFPQCLSGTWAVLLGVSVPELPLTSNTRSFFL
LYVWYSVAINMVLQAFFVTYLVZPGYEEKMITLQDFRRSNLSYALVQVINVFLSYTTYTGH
YLIPQLDEDIEFYKCSEMVMFNHNLYTIGLQLVAYFIAEKRAVKEINKVVCFLEERLFYGAV
SMGIMKGNPIVPVLNEYFSRCIEAGLQENYWSMLKYRTRLKAQDIYDEEVMYFVFSMQHL
NPVFLLHVFGCIVSAVMFAVELIVSGVSSKLCK
>BgerIr609
MNVYRLLVILITASVSTDCGLLNLQQTHISKFLQEILKYYFSTTVRMFVSFSSSCPVNFSRNL
NISFSVSHDLDLVNEVLTHISNVTTVLSYQSELNEGAIFPDDGILEQGYFVFLSSCQQNEGEF
DDLINDLETQFFYIIRSFSFNPRAKYILIVSNNQGTDGESLAFELVKTFWNLAKIVNFVFVIN
GLEAARQNVLNLYTWYPYINGACNEVDKVVLVDQWKFSDGGHFSRKSDLFSYKFPKNVD
FCSLVVASAGPEPYVVLLRNYTNEDGNPAYEVEGMSLNLITLFAKDYNFSIIYRPPIIEINVH
KILMEMAFIFAGSGDILTGLIPLVAPTVSLADITFPNFFESVLWLIPCPQPLGRIDRIVGIFTA
SAWASMISVIFFASAVLLLQAKHYKKEVDGFKSFPQCLSGTWAVLLGVSVPELPLTSNTRS
FFLLYVWYSVAISMVFQAFFVTYLVEPGYEEKMVTLEDLQRSNLSYALIPTIDIFLSYTTYT
GHYLIPQLKEDIEFYKCSEMVMFNHNLSTIGLQLVAYFIAEKRAVREVNKVVCFLEERLFY
GAISMGLMKGNPIVPVLNEYFSRCIEAGLHENYWSMLKYRTRLKAQDVYDEEVMYFVFS
MQHLNPVFLLHVFGCIVSAIMFAVELIVSRVSLKLCK
>BgerIr610P
SRSFICSNMNLSRLLVTLITASVSADSGLLSFQHNHMSNCLQEILKNHFSSTVPIFVSFSSSC
PVEFSRNLNISHSVSHELDLVNEVLTHITNLTTVLSYQSELNEEAIFPEDGILEQGYFVFIST
CQQNEREYDGLINDLETQFFYITSSSSFNPRAKYILIVSNNQETNIESLAFELIKAFWSLAKM
VNFILVINGLEAAQQNVLNLYTWYPYLNGACNEVDKAVLVDQWKFSDVGHFSKNSDLFS
YKFPRNVHFCSLVVATSGPEPYVVLLRSYTNEDGNPDYEVEGISLNLISLFARDYNFKMIYR
PPITELNPPATLEEVSLIFAGSVDILTGFVXVSLGDITFPIFFESALWLIPCPQPLARIDRIFGI
FTASAWATMISVIFFASSVLLLQAKHYEKEVNSFKSFPQSLSGTWAVLLGVSVPELPLTSN
TRSFFLLYVWYSVAISMVFQAFFVTYLVEPGYEERMVTLKDLQQSNLSYSAVMSINFFLSS
TAYTGHYLIPQLEEDIEFYKCSEMVMFNHNLSTIGLQLVAYFIAEKRAVREINKVVCFLEER
ILYGAVGMGLVKGNPLVPVLNEYFSRCIEAGLQENYWSMLKYRTRLKAQEIYDEEVMYFV
FSMQHLNPVFLLHVFGCIVSAIMFAVELIVSRVYSK
>BgerIr611
MSSSFTCPDMNLHTLLLTLITASVSAGSDLLSLQQNHILNCLQEILKNHFSSTVPIFVSYSSS
CPGEFSRNLNIYFSVWHDLDLVNAVLTHITNLTTVLSYQSELNEEAIFPEDGILEQGYLVFL

SSCQGNEGEYDDLISNLETQLFYIIRSFSFNPRAKYILIVSNNQGTEVGSLAFELIKTFWPLA
NIVNFILVINGLEAAQQNVLNLYTWYPYINGACNVVDKVILVDQWKFSDGGHFSRKSDLFS
YKFPKSVEVCSLVVATVGPEPYVVLLKNYTNEDGNPEYEVEGLSLNLITLFARDYNFTINY
RPPITDLNAQEILLEMTFIYDGTVDVITGLIPHYPLSVSMADITFPNFFESILWLIPCPQPLG
RIERVVGIFTASAWATMILVIFFASSVLLLQAKYYKKEVNGFKSFPQCLSGTWAVLLGVSVP
EQPLTSNTRSFFLLYVWYSVAISMIFQAFFVTYLVEPGYEEKMITLKDLQRSNLSYASVESI
AFFLSSTTYDGYYLIPRRKEDLDFYKCSEMIMFNHNLSTIGFQSVPYFIAEKRAVREVNKVV
CFLEERILYAAIAMGLVKGNPLVPLLNEYFIRCIEAGLQENYWSMLKYRTRLKAQDIYDEE
LMYFVFSMKHLNPVFLLHVFGCIVSAIMFSVELIVSRVSSKLCK
>BgerIr612P
MNLSTLLVTLFSASVSADSGLLSLQQNHVSNCLRDILKNHFSSTMPIFVSFSSSCPVAFSRN
LNLSYSVSNDLDLVNEVLMHITNLTTVLSHQSELNEGEIFPDDCILEQGYFVFLSSCQQNES
EHDDLINALESQFFYILSSSTFNPRAKYILIVSNNQQNNVESLAFELVKKFWSLANIVNFILV
INSLDAAEHVLNLYTWYPYLNGDCNEVVQVFSVDQWKFSGGGHFSRKSDLFSYKFPRSVE
VCSLLVSSVGPEPYVVLLRNYTNEDGNPVYEVEGLSZYLVTQFARDYNFTIIYRPPITGFNL
DKAFYETSLFLAGTVDILTGLIRQAATTVSFYDVTSPIFFEYLRWLIPCPQPLGRIDRIVDIFT
ASAWTTMLLVIFFASSVLLLQANHYKNEVDGFKSFPQCLSGTWAVLLGVSVPEQPLTSNT
RSFFLLYVWYSVAISMVFQAFFVTYLVEPGYEDKMITLEDLQRSNLSYASGPSMKFFLSTT
TYIGHYSIPNLNEDFNFYKCSEMVMFNHNLSTIGLQLGSYFIAKKRAVREVNKVVCFLEEPI
LHLAIGMGLVKGNPLVPVLNEYFSRCIEAGLQENYWSMLQYRTRLKAQDIYDEEVIYFVFS
MQHLNPVFLLHVFGCIVSAIMFAVELIVSRVLQNYVNNYLRVV
>BgerIr613
MIIVCTFFTFLIIRLTISESVEKHILKCIDNIVNTYFDQSHSILVSFPNVCTNPTSHSMEVSSTA
SDCMTLIDELLTTLNNLTLVQIYVSSGNLYQSENVADSSQLYLIFVFKCEQRKKLYQNLYFE
EDIEITFEYQLFYIKNSKSWNQMAKYIIVVTDSTHERSDLLALRLTKKLWEITKTINFIILIA
RSKQANQAPNTTWDESVALDIYSWFPYENNCGEVDKMILLNQWLVTNGGQFSKDVSLFN
YKLPKDFNKCVLKVLSFGPEPYVTLEQNYTESDGNQVLELDGLGVHLIHGFGQEFNFTLHF
VTYLFNFDVSEVVDIYESINTGKCDVLIGAVPIVPFSYQVFGITTPFLFDSFVWIVPCPQQLG
RITRVLSIFTVSSWLTLISVIFLASATLNLLSRYNTREVKTFRKLRPCLMSVWAVLLGNSIPQ
MPKSSKTRSFFALYVWYCLAVSMIMQTYFITYLVEPGYEQKMRDFDDLRKSDLPYGMLSI
YEFYTVESDYNKHTTLRVSIGVTDIENCLEAVMFHHNISTVTSTYLPVYVVHKKGVQNIDN
FVCFLEEIIFSVYGAMAVQKGNPLLTLLNEYIRRSVEAGLQEKYWSYLNHKVHLQAEDAID
ELDIFFVFSITHLTPVFELLFCGYVLSALCFSIEMFLSLYLKVDYSISIRLLPFH
>BgerIr614P
MMRILIFIMADLYFATGLLNPLQHQILNCIDNIVRKYFELSGSILVSFPKVCNNTTPRQLGLS
NIPDHQMLVDEILATVNKFTIGQVHMTSIYMYDSEYTKDFVLQQGYIIVLFQCRNKSSSHK
NENYEDLENIFEDQLFYIKDSQSWNPSAKYILVVPESGYQRTDLLAFRMIEKSWKIAKVIN
FVLLIGSNIRDKQVQNENVIIDMYTWFPYQNNCGEVNKIISLNHSEFIKSGYFSKNIRLFQY
TLPTDFKKCALKISPFGPEPYVVLEQISSDSTGSEVFEIQGLGVHLIHAFVKKYNFTLNFLPR
FASYDVAEIVQLTAMINSGKPDIATGAIPVISYSHILFGLTTPFLFDSIHWLVPCPQQLGRVE
RVLSIFTVSTWLTLVVVIFMSGATIFLLSRFNKKEVKVFKNFRRCMISAWAILLCNSLPYMP
RTFSTRSFFVLYLWYCFVLTTVIQAFFVTYLVEPGYEERMKSLDDLRDSGLPYGTMTIFRW
FAFVTSYQGHKKLHGSIIVEDMGKCIEDTMFHRNISVTSTAFLPIYLANKKGVENTDSYVCF
LEGTVFSVFGSMALQRGNPLLTILSKYIQRGVEXGGLQEKYWSHLNHKTHLEAKEGYDRQ
EIFFVFSMKHLNPVFELLFCGYVVGTIGLAWEMLLSICLRKHSKSLMMLT
>BgerIr615

MSTFQKYAIIFLSTVDYKYKTKSQAVLISALLVCLFRSVNSLLNEQIQILSHEILHILIHHFAD
IRSISITFPDLCLDSKSRSLRTLEELYNEHLVKEIISSVGNFTAVNIQSASDDSDLPEDGILEQ
GYLILFFQCSHTKDNSYKNIAVDIKDIFENQLLYQIKDKLAWNARAKFLVITLPFAEEQSKT
VAFRLSQSLWDYAGVSNFLIIIPEAIQELDQCEGTSKVAIYSWFPYSNSSCGQVEDVILLDQ
WVYNNGTCSISEKFNFFSYNLPRNFQSCPLRISALGPEPYVVNNRNYTDKHGNTVHDVEG
VGVNLIHSFGQKYNFALTFLPPVQSLELDNLLLKFSDIYTNESDVLTGCIPLMHFFYAQGDV
TFPVFFESAVWVVPCPAQLSRMERIVRIFTLPSWLAIGSVMYIGTAVLCLQAHYYKGELKR
FKDCISCFLDVWAVLLSVSVSKLPKTENTRRLFIMLIWYSFVISMMFQTFFITYLVEPGYET
QFKTLDDLRESMLPYGFIQFFEFMLSQTSYKGHNTLRSSKIECVDIYECTEKVIFERNMVTL
TMQLMSYYIAENKGVRDLNNVVCFLDETLFSGGLVMSVRKGNPLLGILNDHLSRSIEGGLQ
DKYWSELKYKIRLQNQTLDDKLYFVFSFAHLSPVFMMLACGYFISIFALSVEIIYTYYVKMK
QKAIQNCPYKSKRLKYT
>BgerIr616
MNIQIHIILSILIPLTSVFSFVTVQDHILNCIENIVNTYFNDSRSILVSFPGVCQNKIFRGLNTS
FINEESHWIVDRSLSIFSNSTLVQLLIPPTSLDIQDGALEEGYFIFLFSCRDEGKLDSAEFDSG
DFDDYSDDVMNVFVDQLVYISNRQSWNSRAKYVVVIPDFAGDNSELFALRLAQRWSNIV
KVVNFIILIARNDQADQEPMTVKEETAALDVYTWVPYHNNCGEVDKIILLNQWTFNGRFL
IDVDLFSFELHKNFNMCLLNIVATGIEPFVIVKRNDTYPNGSQVFVLEGLGVQLISGFAEKF
NLSLKFLQPFKQHSIDELLEISDLINTGRADVITGFLPLNLYVTSQFDFTVTVWSIPIVWLVP
CPQKLGRVNRALSIFTISSWLTLVIVLLLSSATLCTLSKYGKEVSEFKDFGQCILSAFVVLLGI
SVAQMPETFRTRCFFGLYVWYCLAVSMVFQTFFVTYLVEPGFEERIRTVEELWKSKIPFGS
TEVTSFFAKQTKIMGYNVTEFPSKECTDSCVEDVLFKRRISTLAGTLLAEYTAKKKGVQKV
ASVVCYLDEGHIYRNLVMAVAKGNALLPILNEYLRRSVEAGFQQLYWSHLNHKVLLEGKD
LYSEPGMYFVFTVDVLAPVFHLHFCGCVLSVLCFTTEVFLSNYCKKMEKI
>BgerIr617
MTSVFMHCNMIVCFLSLAVVHGLLSPLQESILHFANHVLQNFTNYSSILVSFPNVCPQMPA
RTLNLGSSDSDHQALIDDLLSSLSSVAPAQILVSEETLREENTFDLREDAYFLFLYLCESLET
VQQEENNVDFTEMYFNYIEIVLEDQIKQIMEKPSCNLRGKYILILPMCGLYEGKHLALRIIQ
KWWSLSRMTHFIVVVPNTCTSGSTRIYPVLDIYTWFPYENNCGVVNDVVLLNQWLPKNG
GQLSNHTNLFLYKTIRNYYGCNLTVIPGGPEPFVIVDKVKTGSNNSVEPSNVQGISMYIIESF
AKWYNFSIYYLPHVIIADTKNILHGVEIFGAGIPDFTSALVPLSTVPWALSELSLPVLQESAI
FAIPCPKRLGKLSTVAAIFSTYTWLSIAVVLLMSGVVMFLQAKWKTTEIKLYRKMTECLSC
VWAILLGVSVPQMPKSLNSRLFFLLYVCYSLAISVVFQAFFVTYLVEPRYEKNFKTFSDLRE
NGVKYGFYDFAEFLLGFMGFKEQKRLEGIQCKSVVECVEGVVAHQNMTALSTKYIARYIAY
KRGVEDIDSIMCFLDEMLFSCLFLCIAVQKGSPLLPILNEHITGNQEGGLQDYYWTQLKHS
VHLRTELKTEEMYFAFSISHMGPIFGLLCAGYIISLIIFGIEIIVSSYKERF
>BgerIr618P
MTZVFTHSNMIVCFLSLTVSGLLZPLKESILHLSNHVLKNFTNYSSILVTLPKVYPHLPTRS
LNLESZDSDHQALTDDFLSSLSSVAPTQVLLSEEYLGEENEFDLREDAYFIFLYLCESPQTV
QLQENNEDLTEMYFNDIELVLEEQVLQIMAKTSCNLRGKYILILPICGPYEGKNLALRIIQK
WWSLSRMTHFIVVVPNTCTPGCTGIYPVLDIYTWFPYDNNCGVVNDVVLLNQWLPTNGG
QLSKHTNLFIYKTIRNFNGCTLTVLAGGAEPFVIVDKVKTGRNNSVESTNVQAINVHIINSF
AKWYHFSIYYLPLVTVTDTTSVLEAVETFVAGIPDLTSAAILLNPLPATMSELSLPVFKDSLI
FVIPCPRALDKLSTVVAIFSTFTWLSIAVVLLMSGVVMFFQAKWKMTEIKLYRKLTECLSC
VWAILLGVSVPQLPKSLNSRLFFLLYVCYSLAISVVFKAFFVTYLVZPRYEKHCKTFSELRD
NRVKYGFNEFAEILLAVMGFKEHQILERIMYNDVNECVERVVLHNNMSFLATEYLARYIA

YKMGADNMYSVVCFLDELVLVSSFFCVTVQRGSPLLLILNEHITGNVEGGLQEFCWSGIKH
GRNLRSEKIAEKMYFTFSMIHMGPVFGLFCFGYIICVITFASENIISSFNFH
>BgerIr619P
MTSIFRCCNVIGCYLLLTHVSRLLTSSQESILYFANQVLQNFTNSSSILVSFPNVCPHMPART
LNLGPSDSDHQALIDDFLFTLSRVAPAKVLLSEEYLGEENKFDLREDAYFLFVYLCESPDTV
QLQENNEDLTEMYFNDIELVLEEQVLQIMAKTSCNLRGKYILILPKCGPYEGKNLALRIIQK
WWSLSRMTHFIVVVPNTCTPGCTGIYPVLDIYTWFPYDNNCGVVNDVVLLNQWLPTNGG
QLAKHTNLFIYKTIRNFNGCTLTVLAGGAEPFVIVDKVKTGSNNSVESSDIQGISIYIIDSFAK
WFNFSIYYLPLVNVTDQTSLSSAVETFVAGVPDLTSAATPVSPLTATMSELSLPIFKDSSIFF
VPCPKALDKLSTVVAIFSTYTWLSIAFVLLMSGVVMFLQAKWKVTEIKLYRKLTECLSCV
WAILLGVSVPQMPISLNSRLFFLLYVCYSLAISIVFQAFFVTYLVEPRYEKHFKTFSDLRDNG
VKYGFNEISEYSMAYMGFTEHLILEKIMCKNIHECIEGVKTXKNISFLATEMTGRYIAYKMG
ADNMHSIMCFLDDVVLVTSFFCVAVQRGSPLLPILNEHITGNLEGGLQDFYWSGIKHGLNL
KSEKRAEEMYFAFSMIHMGPVFGLLCFGYIICVITFAIEKIIATFKJHRMKK
>BgerIr620I
MTSIFGHCNVIVYFLLLTVVSGLLTSSQESVLNFANHVLQNLTNSSSILVSFPNVCPHMPAR
TLNLRSSDSDHQALIDDFLFSLSRVAPAKILLSKEILREENKFDLREDAYFLFLYLCETPEKV
QILQENEDFSEMYFNDIEDVLEEQVIQIMGKASCNLRGKYILILPMCGPYEGKNLALRIIQK
WWSLSRMTHFIVVVPNRCSSDSYPVLDIYTWFPYKNNCGVVNDVVLLNQWLPLFLCIAV
QKGSPLLPILNEHITGNQEGGLQDLYWTQLKHTVHLRSEMRSADMYFAFSISHMGPIFGLL
CSGYIISLIIFGIEIIVSSYKERF
>BgerIr621I
MTSVFMHRNVIVCFLSLTVVHGLLSPLQESILHFANHVLQNFTNYSSILVSLPKVCPHLPTR
SLNLGSSDSDHQALIDDFLSSLSSIAPTQVLLSEENLREEHKFDLREDAYFIFLYLCESLETV
QQEENNKGISEIHFNDLEIVLEDQVKQIMEKQSCNLRGKYILILPMCGPYEGKHLALRIIQK
WWSLSRMTHFIVVVPNTCNSGSYPVLDIYTWFPYKNNCGVVNDVVLLNQWLPVQKGSPL
LPILNEHITGNQEGGLQDYYWTQLKHTVHLRTELKSEEMYFAFSISHMGPIFGLLCFGYMI
SLIIIGIEMIISSYKARF
>BgerIr622P
MTSIVRYSNVIVYFLVLTVVRGLLSPLQESILHFTFHVLQNFTNSSSILVSFPNVCPLMTARA
LNLGSSHFDLQALIDDFLSSLNSVAPTQVLLSEENLREEHKFDLREDAYFLFMYFCESPKT
VQQEEKNVDFTEMYFNDLEIVLEEQVKQIMEKASCNLRAKYILILPMCGPYEGKNLALRIT
QKWWSXSKMTHFIVVVPNTRTSDYSVIHPILDIYTWFPYDNNCGVVNDVVLLNQWLPTN
GGQLSNHTNLFVYKTIRNFNGCTLTVIAGGPEPFVIVDKVETGSNNSVASSDLQGISIHIIES
FAKWYNFSIYYLPLVILADTKNVIHGVEMFGEGIPDLTSALIPLGTVPWAISELSVPVIQESA
IFAIPCPKRLDKLSTVVAIFSTYTWVSIAVVLLMSGVVMFLQAKWKMTEIELYRKMSECISC
VWAILLGVSVPQMPISRNSRLFFLLYVCYSLAISIVFQAFFVTYLVEPRYEKHFKTFSDLRD
NGVKYGFYDFAEILLGFMGFKEQNILETIKCKNTNECVEGVVAHQNMTALTTKYLARYIAY
KRGVENIDSIMCFLDEMLLSSIFFCIAVQKGSPLLPILNVHITGNQEGGLQDFYWTQLKHTV
HLRTEMRSADMYFAFSISHMGPIFGLLCSGYIISLIIFGIEIIISSYKERF
>BgerIr623
MILMDVTIQRQTIKGVTMKCAMHPKRIVGKLFWSVFLPLVFPCVRGLLSEEQTHILSCIKEI
LHHHLIQTRSIVVSFSKQCEDPTTRPLGITLSSDHLHLVTEVVSTINNFTLTKVLRPSSENN
EVVEKEINSHDSDYIIFLFSCGRLEANNDTKDLEYKLEEQMQQIMHRLSFNPRGKYVFVTD
SNEEETALLASRLINKLWRIVKNVNFVFLIQNIEENILPEEFSGTNDETAMSVHVYTWFPYI
NGTCYEFDNAVLLNKWLPKNGGQFLMQENLFRYITPADFEGCPLRVATIGPEPYVIPKVR

FINNEQQLLDLEGLCVNFIHTFSEKFNFTLEFLPPLPSVELDAIFSIGIDAQSSRSDIVLGVVA
LSAVFLPYIDITRPLVFETAGWIVPCPQPVERIGRVIRIFSSCTWMFIFIISLFSSIVIFLQAAN
DEKEVKTFKTLVLCFYYAWAVLLGISVPQMPTSSNTRIFFLLYIWYSIAVNMIFQAFFVTYL
VQPGYEERIQTFDDLKKLNIPFSFPDLGFFLLDSLDYNPQTQLTYAGKCANFHSCSETVIFH
RNLSSGTIMALAYHLAHTLAVRDVNSVVCFLDENIISGYFPAGLPKGSPLFAILNEHITRITE
FGLIEHYSSQLEHEIQLEAKYKYEESELYFVFSMLHLSPIFMLLIVGWALSFVLFFLEVLYSQ
FKRGLFTKKISQQIFLRKYYSKICRFNKY
>BgerIr624C
MDETIEIQTIKEVTTRSAMHLKRIVSKIFLSVFLPFALPSVSGLLSEAQTLVLSCIKEILHQHL
IQTRSIVVSFSRPCVDPTTRPMGITVAPDHLNLVTEVLYTINNFTLTKVLKSSSEVAENENE
SFDNDYLIFLFTCNESEGENITSDLEYKLEEQMQQITERLAFNSRGKYVIITDIIEEATELLAS
RLIKTFLRIVKSVNFVFLIPNNEKNTLNVEGSGKIEENSMSVHIYTWFPYSNGTCYEFDNVV
LLNKWLPINDGKFLMKENLFRYITPADFEGCPLRVATIGPEPYVIPKVRFINNEQQLLDLE
GLCVNFIHIFSEKFNLTLEFQPTVTSLAWDGIMSIGIEAQSNRADVLLGIMPVNAMILPYVD
VTRPLLIERGAWIVPCPQPTERIGRVMRIFSSYTWMFMFVVSFFSSIVIFLQATNDEKEVKT
FKKLVLCFYNVLAVLFGISVPKMPTSSNTRIFFLLYIWYSIAVNMIFQAFFVTYLVQPGYEE
>BgerIr625P
MHPKSIVTKLFLSVFLQLALPCVSCLLSEEQTHVLSCIKEILHHHLIQTRSIVVSFTKPCEDP
KTRPLGITLASDHLHLVIEVLYTISYFTLTKVLKPSSEFAENEIDSFDIDYLISLFTCNELXLL
GISVPQMPTSSNTRVFFLLYIWYSIAVNMIFQAFFVTYLVQPGYEERIRTFEDLKKLNIPFSF
HDLGFLLLDSLDYNPQTQLTYAGHCADLYSCVETVMFHRNLSSGTFMALAYHIAHTRAVQ
DVNSVVCFLDEMIGYCFITVSLPKGSPLLAILNEHITRVTEFGLQEHYSSQLKHKIKLEAKYK
YEGSKLYFAFSMLHLSPIFMLLIVGWTLSFFLYILEVLYSRFKRDLFNKKISHQKLCKOILLK
DL
>BgerIr626
MNETIQRQSIKGVTMKFAMHPKRIVGKLFWSVFLPLVFPCVRGLLSEEQTHVLSCIKEILH
HHLIQTRSIVVSFSKPCEAPTTRPLGITLAPDHLHLVTEVMSTISNFTLTKVLKPSSEVSKN
EIDTLDSSYFIFLFSCEMLEPKNDTSDLEYKLEEQMQQIMERMSFNPRGKYVLVTDSVEEE
TALLALRLIKKFWNTLKSVNFLFLIPNIEKITLPVEGSVKNEENAMSVHIYTWFPYSNGTC
HEVDTVVLLNKWLPHNERKFLMKENLFPYITPADFDGCPLRVATLGPEPYVIPKVRFINN
EQQLLDVEGLCVNYVHTFSEKFNLTLQFQPPNTLVELNSILSIFEGIHSGKADIILGFLPVSA
LILPYMEISRPLLFDSGVWIVPCPQPIERIGRVMSIFSIYTWMFMVFILFLSSIVIFLQAENDE
KEVKIFKKVVLCFYCTWAVLLGISVPQMPTSSNTRIFFLLYIWYSIAVNMIFQAFFVTYLVQ
PGYEERIRTFDDLKKLNIPYSIPNTAFILLGSIDYNPLPQLTSARNCANLYSCVETVMFHRN
VFSVTPVALAFYIAHTRAVHDVHSVVCFLDEIIVYGYLSSGVTKGSPLHVILNEHIIRVTEFG
LQEHYSSQLKHKIQLQAKDKYEQNQLYNVFSMLHLSPIFVLLIVGWTLSFILYILEVLYSYL
>BgerIr627
MENILSLVLLLLSVSGGREINTVEYKHIVSCVEDILHRDFKNIHPVVVSVPEICRDSTPRALG
LLLKPPKQDHQQLVDELLKTVSNFTLIQMRVPSLATENEYPGNNDQTRHRGYIFLIFSCNA
DEDSEADDEEFQYILDSQINVIMEKLTFNAMAKFIVILPEIFGDNSESFALALTRRWWNMA
KMVNFIILLPHSVINIEEKEIQNGDEGAKLDLYSWSPYIDNNCDAVDKVILLDQWLPSNRD
KFLKNTSLYNFTLPTNFHGCPLNVATFGPEPYVIADPNYTMDNGVELKDVNGLSIHVLYSL
TRKYNLTLNFILHKKEISGSGNSDFLMKFLADEMHVIAGTIFPMITTLIYGDMSNPIVYDAA
KYIVPCPTPIGKMWRILKTFTSSTWAALIVVLFLASFLTQLNSRTNRKEYNMFTKFPYCLY
SNWAVLLGISTPQIPFSSRIRQLFILYVWYCFAINQVFQAYFVTYLVEPGYGKSLETLDDLR
LSKITYGWFNMAAWIIAVLDTLDFKTVAYSLRECIDVYPCTEIVISEQNLAALTSPFLAWYI

ARKKGFQNLNKIVCFLEENLFTFYFPFLVRKGFPLLSIINYHILIYMQTGLQERYLSELIHGIL
LKADERHNEDLMYTVFTMKYLGPIFMLLIFGCFISTMVFIGEIVFNFCRKKML
>BgerIr628
MTYTSLIVFQVVVLMCKICSGELVTAQQSYIISSIKDILQRDFKDTKHLVVSFQEICRDPISRS
HAVLSSSPEQDHLLLMDEMLKVISNFTLVQLKVSSTMSDNEYDGNDDQRLIQGYIILLFSC
KSDDEDSIEDGADSETAEDNPLDYVLDDQVNFLMEKMTFNPRAKFIIIIPDTHGEHPDSLT
LRVTKLLWKVTKMSNFIILIPKCLKNVSVKTEANIQEEDVALELYTWIPFEGNACEKVDEII
LLNQWMITSGGQFLKNITLYPFSLPKNFHQCPLIVVAFGTEPYVIVDEILNQENENQSQILN
GLGLNLLHTFAKKYNFTLSFTPYEKVISGERSERLIKKFLNDEIHVVGGSVILSYTTMRYGD
MSDVIIYDYFRYLVPCPLPVGKVSRELNTFTIFIWISLILVLFLSSFLIVLHSNKLTLEYKMFS
NFSYCLYSNWAVLLGVSSPAIPVCSRNRQFFILYVWYCFAINLVFQTYFVTYLIEPGYDKGL
KTVEELRLSKITYGFYDIADALLTTTDLLNFKTVAFSVKECFDLFACTEEVMFKRSLATLTT
YTLPWYVARKRGVLNVDKVICFLDGNLFQALFVFFLRGGNPLLGLINHHILRVMEGGLQE
KYKSEVKHQALLSAENVYIGEETYSVFTLPNLWPVFLLLLFGCLGSACVFIGEVVVTSSRKK
II
>BgerIr629
MKHTSLMVFLLSALIYTVGSGELSTSQQTYIISCIKDILQRDFKDTNHLVVSFREVCRDSIRR
QLEILSSSLEQDHQLLVDELLKVISNFTLVQLKISSTLPDNEFEGNDDQRLIQGYIIILFSCKF
DDDSIHDGEDSETTKDNPLDFVLDDQIIFIMEKITFNPRAKFIFIIPDTHGEHPDSLTLRFSQ
LLWKVTKMSNFIILIPKFINNVEMNSVVNIQEKEVALEVYTWIPFEGLTCDNVEKIVLVNQ
WMVTNGGLFLRNITLFPFSLPKNFHQCPLIVGAFGTEPYVIVDEIPNEEKGKQSLILNGLGL
NLLYTFSEKYNFTLRFAPYENSISGKRTNIILHQFLNDHFHILGGSVILSYTGTIYGDMSDVII
YDYFRYLVPCPLPVGKVSRVLNTFTVSVWISLILVLFVSSFLIVFNSKNLQLTLENKMFSNF
SYCLYSNWAVLLGISAPAIPVCSRSRQFFILYVWYCFAINLVFQTYFVTYLVEPGYEKGLNT
LEELRLSNITYGYYEIADGLLTTTELLNFKTVAFSVKECFDLFACTEEVMFKRSLATLTTYT
LPWYVARKRGVLNVDKVICFLDGSLFQALFVFFLRRGNPLMGLVNQHILRVMEGGLQEKY
KSEVKHQALLSAEKVYVGDEMYSVFTLPNLWPVFLLLLFGCLASACVFVIEVIITSFRKKIT
>BgerIr630
MRCITVMVLLEIAFMCTVCSRELYTAEQTYIISCIKNILQRDFKDTKHLVVSFQDVCRDPIS
RSHGFLSSSPEQDHLLLVDELLKVISNFTLVQLKVSSTMSDNEYESNDDQRLIQGYIILLFSC
KSDDGEDSETTEDNPLDYVFDDQVHFVMEKVTFNPSAKFIFIIPDTQGEPSDSLALSFTNS
LWKYSKLTNFIILIPIFLKKVEVKTIVNIDEKDIALNIYTWIPFEGNTCEKVDKIVLLNQWID
TSGGQFLRNVTLYPFSLPKDFHECPLIVGALGTEPYVIVDKNPNQGNINQSQELNGLGINLL
HTFAKKYNFALSFSPYESVMSTERVEQLITLSLNDEIHIAGGSIALGISTLRFGDVSDIIIYDQ
FRYLVPCPLPIGKLSRLLKTFTRTVWLSLLLVLILSSFLIVLHSNNLTQEYQMFSNFSYCLYS
NWAVLLGISAPAIPVCSRNRQFFALYVWYCFAINLVFQTYFVTYLVEPGYDKGLETLEELR
LSKITYGYYAVADILISTQDSINFKNVAFSVKECFDLFTCTEEVMFERSTATITSYSLPWYVA
KKRGVLNVDKVICFVNENSTFAWFGFFFRRGSPLRKLLNEHIQRVIEGGLQEKYESEVEHE
ALLSAENMYVGDEMYSVFTLANIWPIFFILIFGCLASVCVFLGEVMITLFKKKILSKILCHKK
LRLPC
>BgerIr631
MRCITVMVFLETAMICTISCGELYTAEQAHIISCIKDILQRDFKDTKHLVVSLQEVCRDSIPR
KLDILSSSPEQYHQLLVDELLKVISNFTLVQLKISSTMSDNEYEGNDDERLIQGYIMLLFSCK
SDEDSIEDVADSETTVDNPLDYVLEEQIAIIMEKLTFNPNAKFIFIIPDTQGESSDYLAHRFT
NLLWKDGKLTNFIILIPNFLKNVEVKPVVNIDIALNLYTWIPFEGNTCEKVDKIVLLNQWI
DTSGGQFLRNVTLYPFSLPKDFHECPLIVGALGTEPYVIVDKNHNGENGKQNQELNGLGL

NLLHTFAKKYNFTLSFTPYENVISSERIEQQIKLFLNDEIHIAAGSIALTFSTLRYGDVSEIIIY
DQFRYLVPCPLPIGKVSRLLKTFSRTVWLSLILVLFISSFLIVLHSNNLTQEYMLFSNFSYCL
YSNWAVLLGISAPAIPVCSRNRQFFALYVWYCFAINLVFQTYFVTYLVEPGYDKGLETLEE
LRLSKITYGYLAIADELISTTDNINFKTVAFSVKECFDLFTCTEDVMFERSSATLTSYSLPW
YVAKKRGVLNVDKVICFLKENSTEAWFGFYFRRGNPLRKLLNKHIHRVIEGGLQEKYKSE
VEHEALLSAENVYVGDEMYSVFTLTNLWPIFLILIFGCLASACVFLGEVMITLFKKKILSKG
VCHKGLRLPGKY
>BgerIr632
MELLILIMTALMFVCKSEKLYPSKQEHIILCTKHILQRDFKDTQPLVISIPEVCRESPMRSLG
FPSSSSEEDNQILGDKLLKIASNFTTVLIKIFSMVDETEFNDDLRLHQGYIILLFTCNNNGIE
EQNNFQDTEEDTELEYILENQMLFIMERITWNPRAKYLFIVTDTKGKSGDSLSLDLAKML
WKIAKIVNFIILIPNCVNCMEGVQTTTKNQENEIILDLFSWIPYANDTCDKVENNIKMDH
WVRHNEGQFLRNASLYPISLPNDFHGCPLNVATFGVEPYAIVNENYNTSITVGDDQDKPV
NGLNIDLIYTFAQKYNFTLTFLPHESAITPETMSKILEEWLLDRMHIFAGGTILFLASMVFG
DPSHSIVYDYMRFLVPCPVPIGKLWRVLNTFTVSTWLALTVVLILSSIVTSMEEFRNKQQL
RMFTKLPISLYRNWAVLLGISAPDVKISAIARQFFILYVWYCFVVNQLFQAYFVTFLVEPEY
ERKLETLEDLRLSKITFGFFSVLDNFLTTVDAVNFKTVASSTRECHDLHSCMEEVMFDRN
LAILTTSFVSWYVARKRGVSNVNKVVCFLKEDFYQTFIPLYVRRGNPILSILNEHIDRTIESG
LQQRYESGLKHQLILSAEDVYDEELMYSAFTMKQIGPVFWVLLVGYSGCIIVLVFELMISLL
RKKIKFTSK
>BgerIr633P
MHLLVLMLFFIFMCKPETLLTSKQTHIISCVEHILQRYFENTQPLVIPIPEXCRESSACNLGF
LSSFSEEDNQLLVDELFKIVSNFTAVQIKISSLLADNEFENNDDLRAHQRYIIILLSCKPNTF
NLKPQDNYESVDGYDVDLGQILEDQMATIMEKFTWNTRAKYVLIVPDTREETGDSXWKL
AKMVYFIILIPNYVSSKNTSMNQQKELVLDLYSWIPYENDTCDKVDKVTLLDKWVLENGG
NFLSNVRLYPFVLPNEFHGCPLIVGTFGVEPYVIVDGRYEMEGNEEKTVNGLNINLLYTFA
QKFNFSISFLPHHNTISSSYVNELLEYALQDKGHLVGGSTILFVTGMSVGDPTYPILYDYMR
YIVPCPLPVGKLWRVLNTFTISTWLAVIVVVLLSSIVTSLYSIHKKLSMLAKLSTYLFNNWA
VLLGTTSTEFKMSSTARQFFTLYVWYCFAINQVFQAYFVTFLVEPGHEKKMATLEDLKAS
NVSFGYFPVVESMLSSITAVNFRSFASFTREFNDPIECVZQVMFEHNLSTMTPSFLAWYIA
RKIGVSNVNKVVCLLEEDIYQTFMPLYVRKGNPILSILNAHIINIIESGLQEKYESGLKHEILL
NAEDVYDEETMFSVFTLKQMGPVFWILVFGYSSCVLAFIGEVMVSLIK
>BgerIr634
MYLYNFKLSFCTVLFFLTHAQERLTTFQNNIVSCMQDIMKHYFVNSYSVTIAFPNLCQVPN
PRSLQFQSPKLDHQVIVEKFLLYISNESPIHILNSDDNIRDLQQFDVLQEVYFLFLYECQGFE
DHFGDINHIQYEDLEEVLEFQINNILNKPWFNPRGKYILIIPECPLDECRVLSYRLTQKWWI
MSKMVHFIFVVPHINEAYSEYATHDLSYEGYKGLSISKTFDAPLTSEDSIHKSADLDILAWF
PYYNNNCGEVDEMVLLNQWFSVNGGQFSKGLNLFRYKVPAKFSGCPFITIVVGSVPYAIIT
KNYTNENGEPVLEMEGLGPFLVNSFTKRHNFSVQFLPYRELRDVNIILRTIEIFSNGKVNLI
TGAFPLYAFFVPFGEYTVPIINDAVVFICPCPRPIGRLRSVLNMFTMSTWLAVVIVLVSSSLI
LYLHTKRDKNEVKHYKNFGNCSSSSWAILLGVSAPHLPESANNRCYFAAYVWYSLAINVV
FQTFFVSFLVEPRYEQKFRTLEDLRNNDVKYGYHEFGEFLLGFVEYRKQEMLEWIECEEP
YGCIRAVITHQNMSSLSSAYIPFYISKEMGLNDVSRFICFIEETVMTGYVSMGVRKGCPLLSL
LDDHLTRSISAGLHQQYWSDLKHRVNLKAERTEEEEMYFVFSMSHLAPIFGLLGSGFIISIL
SFTVEILVYALLVKRIKQSKTNNVFKL
>BgerIr635

MTISFCALLFLLSFAQGRLTTFQTNIVSCIQDIMQHYFVNSSSVTIAFPNWYQVPTPRSLHL
KSSKMDHQVIVEEFLFNISNESPIHILNSDDNIRDLEQFDVLEEAYFLFLYSCQGIEDQNNDI
NQIQYKDIEEVLECQINNILTKPWYNPRGKYILIIPVCPRDDCRDLSYRLTQKWWIWSKMV
YFIFIVPHINETSSEYAIHDLSYEGYQAWNMSKPYDAPLTAEDCIQKSAALDILAWFPYYN
NNCGEVDEMVLINQWFSNNGGQFSKGFNLLRYKVPGKFSGCPFLTMVVGPEPYVIITNNY
TNENGEQVFEMEGLGAFLVNSFTNRYNFSSQFVPFNELREADVVFRGIQMFLNGKVNLVT
GLVPIFTFFLPFGEYTVPVTNDALVFINPCPRPIGRLRSVLNIFTMSTWLAVVIALVSAILILY
LQTKRDMNEVKHYKNFGNCSSSSWVILLGVSVPQLPESTNNRCFFATYVWYSLAINVVFQ
TFFISYLVEYEEKFRTLEDLRNNDVKYGYYDFGEFLLGFIEYRKQNILGWIQCEDVYGCIQA
VITHKNMSVLSSSYIPFYIARKKGMKDVSHSICIIEEAVITGYLSMVVRKGCPLLSLLNEHIT
RSISAGLQEQYWSDLKHRLNLKAERTEEEEMHFVFSMSHLAPIFGLLGCGFIICILTFTIENL
VYIFLVKVSKLFKNN
>BgerIr636P
MNLRNMTISFCALLFSLPFAQSRLTLTTFQTNIVSCIQDIMKHYFVKSSSVTIAFPNWCQVP
TPPSLHLKSSKIDHQDIVEEFLFNISNESPIHILNSDDNIRDLEQFDVLEEAYFLFLYSCQGIE
DQNNDINQIQYKDIEEVLECQINNILTKPWYNPRGKYILIIPVCPRDDCRDLSYRLTQKWW
IWSKMVYFIFIVPHINETSSEYAIHDLSYEGYQAWNMSKPYDAPLTAEDCIQKSAALDILA
WFPYYNNNCGEVDEMVLINQWFSNNGGQFSKGFNLLRYKVPGKFSGCPFLTMVVGPEPY
VIITNNYTNENGEQVFEMEGLGAFLVNSFTNRYNFSSQFVPFNELREADVVFRGIQMFLN
GKVNLVTGLVPIFTFFLPFGEYTVPVTNDALVFINPCPRPIGRLRSVLNIFTMSTWLAVVIA
LVSAILILYLQTKRDMNEVKHYKNFGNCSSSSWVILLGVSVPQLPESTNNRCFFATYVWYS
LAINVVFQTFFISYLVEPRYEEKFRTLEDLRNNDVKYGYYDFGEFLLGFIEYRKQNILGWIQ
CEDVYGYERXVRTYMVMSVLSTSYIPFYIARKKGMKDVSRSICIIEEAVITVYLSMVVRKGC
PLLSLLNEHLTRSISAGLQEQYWSDLKHRVNLKAVRTEEVEMYFVFSMSHLAPIFVLLGCG
FIICIITFTIENLVYLFTVKVPVLFKNN
>BgerIr637I
MILRNMTISLYALLFLLPFAQSRLTTFQTNIVSCIQDIMKHYFVNSSSVTIAFPNFCQVPIPR
SLHLQSSKLDHQVIVEEFLFYISNESPIHILNSDDNIRDLEQFDVLEEAYFLFLYSCQGIEDQ
NNDINEIQNEEIEEVLEFQINNILTKPWYNPRGKYILIIPVCPRDECRDLSYRLTKKWWTM
SKMVYFIFIVPHINETSFEYAMHGLTYEGYKGRNMSKLFDVTLTSEDNIQKSATLDILAWF
PYYNNNCGEVDEMVVTNQWFPNNGGQFHKGFNFFLYNVPRMFSGCTFLTMIVGPEPYAI
ITNNYTNENGDQVYEMEGLGAFLVNSFTKWHNFSVQFLPFNELREADVVFRGIQMFLNG
KVNLITGLLPIFPFFLPFGEYTVPFINDAVVFISPCPRAIGRLRSVLNMFTMSTWFAVVIVLL
SSSLILYLQTKRDKNEVKHYKNIANCSSSSWAILLGVSVPQLPESTNNRCFFAAYMRKGCP
LLSLLTEHITRSISAGLQEQYWSDLKHRVNLKAVRTEEVEMYFVFSMSHLAPIFVLLGCGFI
ICIITFTIENLVYLFIVKVPELFKI
>BgerIr638P
MNLRNMTISFCALLFLLPSAQSRLTTFQTNIVSCIQDIMKHYFVNSSSVTIAFPNLCQVPTP
RSMHLQSSKMDHQVIVEEFLSYVSNESPIHILNSDDNITDLEQFDVLEEAYFLFLYSCQGIE
DHNGDTNQIAYEDIEEVLEFQINNILTKPWYNPRGKYILIIPACPRDECRDLSYHLTQKWW
IISKMVYFIFIVPHINETSFEYAMHGLTYEGYKDRNMSKPFDVTLTFEDSIQKSATLDILAW
FPYYNNNCGEVDEMTLINQWFSDKGGQFSNGFNFFRYNVPAMFSGCTFLSMIFGPEPYAII
TNNYTNENGEQVFEMEGLGAFLVNSFTKWYNFSVQFLPYRELSDVDFILRTIEIFSNGKVH
LITGLIPLYAVFVPFCEYTVPIINDAVVFISPCPRPIARLRSVLNIFTVSTWLAVLIVLFSSSIIL
YLQTKRQKHEVKHYKNMGNCSSISWAISLLLMCGXFFAAYVWYSLAINIVFQTFFVSYLVE
PRYEEKFRSLEDLRNKDVKYGYNEFGEFLLGFVEYRKQEMLEWIECEELYGCIQAVITHQN

MSVLSTSYIPFHISRKKGVKDIGRSICIIEEAVVTGYISMGMRKGCPLLSLLNEHITRSISAGL
QEQYWSDLKHRVNLKAERTEEEEMYFVFSMSHLAPIFGLLGCGFIICIITFTIENIGTSGNYI
RALG
>BgerIr639N
DLEQFDVLEEAYFLFLYSCQGIEDHNNDIDQIQYEDIEEVLEFQINNILTKPWYNPRGKYILI
IPVCPRNDCGDLSYRLTQKWWMWSKMVYFIFIVPHINETSSEYAIHDLSYEGYQGSKISEQ
FDASVTSEDSIQTSAALDILAWFPYYNNNCGEVDEMVLINQWFPDNGGQFSKEFNFFRYN
VPRMFSGCTFLSMIFGPEPYAIITNNYTNENGDQVFEMEGLGVFLVNSFTKWYNFSAQLV
PFNELREADIVLRVIEKFLNGKVNLIAGLLPIVANLVPFGEYTVPLINDALVFICPCPRAIGR
LKSVLNMFTMSTWLTVVIVLFSSSLILYLQTKLHKNEVKHYKHFGNCSSSSWAILLGVSVP
QLPASTNNRCFFAAYVWYSLAINVVFQTFFVSYLVEPRYEKKFRTLEDLRNNDVKYGYYD
FAEFMLGFVEYRKQNILQWIECEDVIGCIETVIIHQNMSSLSTTRGSFYISRKMGLNDVSRSI
CIIEEAVVTVYLSMAVRKGCPLLSLLVEHLTRSISAGLQEQYWSDLKHRVNLKAERTDEVE
MYFVFSMTHLAPIFGLFGIGLIVSILSFTIEILVYAFLVKRTELLKSNNMFN
>BgerIr640P
MNLRNMTISFCALLFLLPFAQGRLTTFQTNIVSCIQDIMKHYFVNSSSVTIAFPNLCQVPSP
RSLHLQSSKMDHQVIVEEFLFNISNESPIHILNSDDNIRDLEQFDALEEAYFLFLYSCQGIED
XFIKINQIQYKDIEEVLESQINNILTKPWYNPZGKYILIIPVCPRDECRDLSYRLTQKWWIIS
KMVHFILIVPRINKTSSEYAIHDLSYEGYQDWNISKPLDEPLTSEDSIQSSAALDILAWFPYY
NNNCGEVDEMVLINQWFSDNGGQFSKGFNFFRYNVPGMFSGCTFLTVVVGPEPYVITTN
NYTNENGDQVFEMEGLAVFLVNSFTKRYNFSVQFLPYREIGNVDFIFTGLATFLNGKVNLI
TGLVPIFASFLPLAEYTVPVINDAVVFICPCPRAIGRMKSVMNIFTTSTWLAVVIVLVSAILIL
YLQTKRKMNEVKHYKNFGNCSCSSWAILLGVSAPHLPESTNNRCFFAAYVWYSLAINVVF
QTFFVSYLVEPRYEEKFRTLEDLRNNDVKYGYYDIFEFLFGFIEYRKHNILERIECGDVYGC
NQAVITHQNMSVLSTSYIPFYIARKKGMKDVSRSICIIEEAVITGYLSMAMRKGCPLLSLLN
EHITKSISAGLQEQYWSDLKHRVNLKAERTEEEEMYFVFSMSXLAPIFGLLGCGFIICIITFT
IENLVYLFMVKIPELLKNN
>BgerIr641P
MPFYVLLFLLPFAQSRLTTFQMNIVSCTQDIMKHYFVNSSSVTIAFRNWCQVPSPRSLHLQ
SSKLDHQVIVEEFLFYISNESPIHILNSDDNIRDLEQFDVLEEAYFLFLYSCQGIEDQNNDIN
QIQYEEIEEVFEFQINNILTKPWYNPRGKYILIIPACPRDEYGDLSYRLTQKWRTMSKMVY
FIFIIPLINETYSEYSIHDLSYEGYLGSNISKPFDVTMTSEDSIQKFAALEILAWFPYYNNNCG
EIDEMILINQWFSDNGGQFSEGFNFFRYNVPGMFSGCLFLTIIVGXQTYVIITNNYTSDNGD
QVFEMEGLAVFLVNSFTKRYNFSVQFLPYRGDMDLIFTAFDIFLNGTVHLITGLFPIFAFFL
PFGEYTZPIINDAVVIICPCPRPIGRLKSVLNMFTMSTWLAVVIVLVSSSLNLYLQAKLDKN
EVQHYKNVGDCSSSSWAILLGVSVPQLPKSTNNRCFFAAYVWYSLAINVVCQTFFFSYLXE
PKYEEKFRTLENLRSNDMKYGYSDILEFLLGFIEYRKNNILEWIECGDIYDFNQAVITHQN
MSVLSTSYIPFYISRKKEMKDVSRSICIIEEAVITGYLSMGMRHGCPLLSLLNKHITRSISAGL
QEQYWSDLKHRVNLKAERTEEEEMYFVFSMSHLAPIFVLHGWGFIICIISFTIENLVYVFLV
KRAELFKNN
>BgerIr642P
LYYHQKIIKFWAVLFLLRYALSGLTTLQKNVLSCIKDITKNYFVNSDSVSIYFPNRCVIPRLR
SLDFQSSKLDHQMMVEEFLIYISNESPIHIITSNDDNWELDQFDILEEVHFLFLYSCQRTED
HINNFDQIEYEDIEEVLEFQINNILTKPWYNPRGKYILIIPVCPRDDCRDLSYRLTQKWWT
MSKMVYFIFIVPHINETYSEYAMHDLTYRGYKVRNMSKPFDVTLTSEDSIQKSAALDIFAW
FPYYNNNCGEVDEMVLLNQWHFNNTEHFSNPLNLFHHTMPRQFSGCSLKTMTVGPEPY

VIITNNYTNQNGDIILEVEGLSNCLFLSFAKWYNFSVEFQYVSHITDADILLELMQTYLSENI
DIITGPIPICAVFYAFIEFSVPYMNDEVIFISPCPRAIGRLKSALNMFTMSTWFAVVSVLISSS
LILYLQTKHNKNEVKHYKNISNCSSSSCAILLGVSVPQLPESTNNRCFFAAYVWYSLAINVV
FQTFFVSYLVEPRYEERFRTLGDLQKGNVKYSQYETGEFFMGFIDFKEHNELEWTPCEND
VYECIETVLMHQNMTSITTTYIPFYIASQKGLKDVNRYICPLDSQVISGQFGIGVKKGCPLLS
ILDEHLSRSLSAGFHAQYWSDLKHRVNLEAVKTDDIKMYFVFSMVHLGPIFGLLGCGCVIS
VLSFISEIVVYNLLKKKT
>BgerIr643P
MYYLQMIFMFCAVLFLLKFSHGGITTLQKNVLSCIQDITKNYFVNSDSVSIYFPNRCDTPRL
GSLDFLSSKLDHQVIVEEFLIYLSKESPIHIITSNEDKWELDQFDILEEVYFLFLYSCQGIEDQ
NNDINQIEYEDIEEVSEFQINNILTKPWYNPRGKYILIIPVCPRDDCGDLSYRLTQKWWTM
SKMVYFIFIVPHINETSSAYAIHNLTYEGYRGWNTSKPFDAPFTSEDSFQKSADLDILAWFP
YYNNNCGEVDEIALLNQWRFNNTEHFSNPLNLFHHTMPKQFGGCPLKTMTFGTEPYIIIT
NNYTNQNGDIILEVEGLSNCLYLSFAKWYNFSVKFQHVSNIMDVDKLFELMHTYLSENIDI
VTGPVPISAIFYPFIEFSVPYMNDEVTFISPCPRAIGRLKSVLKMFAMSTWFAVVIVLVSSSL
TLYZYQAKRDKNEVKHYKNFGNCSSSSWAILLGVSVPQLPESRNNRCFFSAYVWYSLAINI
VFQTFFVSNLVEPRYEERYRTLADLRKSNVKYSLYETGEFFMGFTEFTEHNELEWTLCEN
DVFDCIETVIMHQNMTSVTTTYIPFYIASKKGLKDINRYICPLDTEVISGQFAIGVKKGSPLL
SILDEHLSRSLSAGFHAQYWSDLKHRVNLEAEKTDDIKMYFVFSMVHLGPIFGLLGCGCVI
SVLSFIAEILVYHLLKKKT
>BgerIr644P
MTLSFRCPDLLRCALLTMLICSCIQSPLTFVQLHILRCINNILHHQFPNVVSVVIAVPNLCDK
SPFHSLHLQSTESDNQLLVDEFIFTVSDVIPFQILSSEIDKTWMSSFDILDQVYFFFLYSCEE
YEEVNVDKSSESQFQAIENVLDKWWIRSRMINFIFIVPNYGKITASALTTVQTSGGIEPRIVI
PPANSFGIDTGESAVLEIYSWFPYYNNCGQVDDVVLLNQWLPDNGGKFSNEMILFQYKLP
VTFHGCPLTIMPAGTVPYIILKQNZTNDEGKKIFDFEGISLFILNTFLERYNFSKNYLPPFLT
MDLATAYEIYEVFFDGKTDIVSAVAPICSFFLYFGELSKPVINEALLFIVPCPRSLDRLTTVM
SIFTTTTWITIVSVLLLSSLLIFMLAKFSNSEVLFYKKMENCVTSTWSILLGVSIPEMPLSGKI
RHFFAIYVWYSLAINMIFQAFFVTYLVEPRYEEYFRSLEELRDAGVTYGSYEFMEIFLGFLE
FKKLYISKQNNFTNPTDCARSAILYQNLTTITTKYIPAYLAYEEGLVGLSRFVCFLENIVISSG
YMAFTVQKGSPLLPILNHHISQTLQAGLQDNYWNQLKHHLYLKAEKKNEEEMFYVFSVS
HLAPIFVLLICGYIVCAIVCAGEILVSLYFNRITKSAVNEG
>BgerIr645P
MTLCFRCPVLLRCALLTLLICSCIQSPLTFVELNILRCINNILHHQFTNVVSVVIAVPNMCDK
SSFHSLHLQSTESDHQLLVDEFLLNISDVIPFQILSSGVDKTWMSSFDILDQVYFFFLYSCEE
DEEVNVNKSSESEFQAIENVLDHQISQIMSRPSYNSRGNYILIIQLCPDQRCEELCQHLTEK
WWIRARMINFILLYRITVQXESAVLEIYSWFPYYNNCGQVNDVVLLNQWLPDNGGKFSNE
MIFFQYKLPITFHGCPLTIIPAGTVPYNILKQNYTNDEGNTIFDFEGISIFILNTFLERYNFSK
NYLPPFLRMELATAYEIYGIFYDGKTDIVAATVPISTFFFYFAAFSKPVLNEALLFIVPCPRS
LDRLTTVMSIFTTTTWITIISVLLLSSLVIFMLAKFSNSEVLFYKTMENCVTSTWSILLGLSI
PEMPLSGKIRYFFAIYVWYSLAINMIFQAFFVTYLVEPRYEEYFKSLEELRDAGVTYGSYEF
MEFYLGFLDFQNLYISKQINFTNPTDCARSVILHQNLTTITTKYIPAYLAYEEGLVDLNRFV
CFLENQMIVSGYMAFAVHKGNPLLSILNHHISQTMQAGLQENYWNQLKHRLYLKAEKRN
EEEMFYVFSISHLSPIFVLLICGYIVCAIVCAGEILVSLYFSRKTK
>BgerIr646P

MTENQNMLLIITIFFQGVQSMNTSSFMFTNIYPDVIGNIVHHNFPNTKVICIITHYQASNSL
QILLDLTLQVINQKLLLNLLILNVDPEEETTGITIQESNFLLFVSTCEDEDLYILEDDLNHLM
QEHSWNVEYKFLVIVLDDSSSSPKQCAQTIMELLWGFVYADNAVILIRNTDLEEIQAYTVF
SYENGNCGKLGEVVLLRQWKFGETSRSTYDGMYPSKLPQYFKGCTLTVGSFGPEPYIVRK
GLSNYSELQLSGLCIELLSIFAQHNHINLFFHEPTERIESNAVLSKIVSLQNGELNVLAGAIA
KITFFLQVMDISDTXIYDTAKWLVPCTDPMPKVERVLTVFCTMTWVGIIIIFICASLILWCQ
ARFICFEPFSRLSKCFSASWAVLLAVSVPTMPQSFELRSLFLVYVWYCFTIITVFQAYFTTY
LVEPGYYEGIHTFDDLVKSQIQYASSELVELMLLSSGYNETARIRKDVIKXTDITVAIKRVM
FQKDIFXKGKPSSIENEHLSRSLQGGLLEKYWSMLNHEMHLKANNTNTQDSDYVVFSLEH
LNLFFFLLLFGYISSSMIFLFEVLVHFINNIRIFKTFLKRFQMDKDFSI
>BgerIr647P
MENQNTLLIITIFFQAVQSMNTASFMFTNIYPDVIENVLHQNFPNSNVLCIITHYQASNZFQ
ILLDLILHTINQNLLLNLLILNVDQKEETTGITVQEFNFLLFXVNTCEDDDLYILEDDLNHP
MQEHSWNVEYKFLVIVLDESSNSPKHCAQNIMELLWGFVYADNAVILIRNSDLEEIPSYTK
FPYENGNCGKLGEMVLLRQWIFGENSSSSYDGIYPSKLPQYFKGCTLTVGSFGPEPYIVRKG
LSNNSESQLTGLCIELLSIFAQQLYLSLSFREPTVQIEYKTVLSDIVSLQNGELNVLVGAVAI
MPIFLDFMDISDTYIYDTVKWLVPCPEPMPKVERVLTVFSAMTWVGIIIVFICASFIIWCQA
KFICFEPFSRLSKCFSASRAVLLAVSVPTMPQSFQLRSLFLVYVWYCFAIITVFQAYLMTYL
VEPGYYEGIHTFDDLVKSEIQYASSELVELLLLSSGYNETAZLQKGGVKSTDTTVTITRVIFQ
KDIASVVPIYFANYIASVSGVSDKSKVVCLLDKALITTSLGYGFKKGNPLRSKMNISLTRSLQ
GGLLEKYWSMLNHEVNLKANNTNTQDSDYVVFSMEHLSPFFFLLLFGYILSSMIFLFEVLV
HFINNIRIFKTFRFQKKDFSI
>BgerIr648P
MENQNTLLITTIFFQGVQSMNIASFMLPNIVYPDVIENVVLHNFPNYNVLCILTHYQTSNSL
QILLDLTLPTINQKLLLNLLILNVDPEEETTGITIQQTNFLLFVSTCEDDDLSILEDDLSHLM
QEYSWIVEYKFLVIVLDDSSSSPKNRAQVIMELLWGFIYADNAVILIRNTDLEEIQAYTVFP
YENGNCGKLEEVVLLRQWKFGETSSSKYDGMYPSKLPQHFKGCTLTVGSLGPEPYIVRKR
LSNTSELQLTGLCIELLSIVAQQLQFNLSYREPIIQIESNAVISEIVNLQTGEVNVLAGAVAVI
NIVLDMMDISDTYIYDTAKWLVLCPGPMPKVERVLTVFSKITWVGIIIVFICASLIFWCQA
MFVCFEPFNRLSQSFSATWAXLLAVSVPTIPQSFQLRSLFLLYVWYCFAIITVFQAYFTTYL
VEPGYYEGIRTFDDLVRSEIQYASFELIELTLLASCYNKTAZLRKGVIKSTDTTATIKRVMFQ
KDMFSIVAIYFADYIASVSGXDKSKVVCLLDESIITTSLGYGFKKGNPLRSKMNIYLXRSLQG
GLLEKYCSMLNYEMYLKANNTHTEDDDYVVFSMEHVSPFFLLLLFGYILSSIIFLLEVLVHF
MNNLRISKTFIKTF
>BgerIr649P
MENQNTLLMITIFFQGVQSMNTTSFIFTNIYPDVIENIVHHNFPNSKVLCILTHYQASNSLQ
ILLDLTLQIINQKFLLNLLILNVDPEEETTGITIQESNFLLFVSTCEDDDLYILEDDLNHLMQ
EHSWNVEYKFLVVVLDDSSSSPKHCAQNIMTLLWELVYADNAVILIRNTDLEEIQAYTVFP
ZENGNCGKLGEVVLLRQWKFGGTSNSSYDGIYPSKLPQYFKGCTLTVGSFGPEPYIVRKGL
NNNSELQLSGLCIELLAIFAQHHHINLFFREPTEGIESNAVLSNIVSLQTGELNVLAGAIAKI
TFFLEVMDISDTYIYDTAKWLVPCPEAIPKVERVLTVFTTITWIGIIIVFICASLMFWCQAKF
ICFEPFSRLSKCFSASWAVLLAVSVPTMPQSFQLKSLFLLYVWYCFAIITVFQAYFTTYLVE
PGYYEGIRTFDDLVQSEINYASFELLEVLLLATGYNTTARLRKSGIKSTDVTATIKRVMFQK
DIASMVVIYFANYIASVSGVTDKSKVVCLLDKALITSSLGYGFKKGNPLRSKINIYLSRSLQG
GLLEKYWSMLNHEMHLKANNTNTQDSDYVVFSMEHLSPFFALLPFGYTLSFMIFLVEAV
VHFVNNLRIS

>BgerIr650P
MENQNTLLISAIFFQGVQSMHTAPLMFTNIGIYPDVIENVVLHNFPNSNVLCILTHYQTSN
SLQILLDLTLKTINQNLLLNLLILNVDPEEETTGITVQESYFLLFVSTCEDDDLITLEDDLNQ
LMKQHSWNVEYKILVIVLDDSSSSPKXCAQIIMELLWGFVYADNAVIVIRNTDFAEIQAYT
VFPYENGNCGEMGEVVLMRQWKFGENSSSSYDGLYPSKLPQHFKGCTLTVGSVGPEPYIV
RKSLSNNSELQLTGLCIELLSIFAQQLHLSLSFREPTVQIEYNAVLSDIVSLQNGELNVLVGG
VAIMPIFLDLMDISEAYIYDTVKWLVPCPEPMPKVERVLTVFTRITWIGIIIVFICASLILWC
KAKFICFEPFSRLSQSFSATWAVLLAVSVPTMPQSFQLRSFFLLYVWYCFAIITVFQAYFTT
YLVEPGYYEGIHTFDDLVKADIQYASYELIDILLLSSGYNETDRLRKGSIKSTDITVTITRVIL
QKDIASIAGIYFANYIASVNGVNDKSKVVCLLDKALITTSLGYGFRKGNPLRSRMNIYLSRSL
QGGLLEKYWSMLNHEMNLKAMNTNTQDSDYVVFSLEHLSPFFVLLVFGYILSSMMFLFE
VFVLFINNIRIFKTFLKRFQRKDFSN
>BgerIr651
MEIQNTLLITTIFYQGVQSMNTPSFMLTNIYPDVIQNVVLHNFPNYNVLCILTHYQASYSLQ
ILLDLTLQTINQKLLLNLFILNVDPEEETTGITIQQSNFLLFVNTCEDDDLSILEDDLTHLM
QEHSWNVEYKFLVIVLDDSSSSPKHCAQIIIELLWGFIYADNAVILIRNTDLEEIQAYTVFPY
ENGNCGKLGEVVLLRQWKFGDPSSSTYDGMYPSKLPQYFKGCTLTVGSLGPEPYIVRKRLS
NTSELQLTGLCIELLSIVAQQLQFNLSFREPIIQIEPNAVISEIVNLQTGELNVLAGAVAVINI
VLDMMDISDTYIYDTVKWLVPCPGPTPKVERVLTVFSKITWVGIIIVFICASLIFWCQAMFV
CFEPFSRLTQSFSATWAVLLAVSVPTMPQSFQLRSLFLLYVWYCFAIITVFQAYFTTYLVEP
GYYEGLKTFDDVVQSEIQYASFEIAELTMLSSGYNVTAQLRKGVIKSTDTTATIKRVMFQK
DMFSIVAIYFANYIASVSGVIDKSKAVCLLDESIITTSIGYGFKKGNPLRSKMNIYLSRSLQGG
LLEKYWSMLNHEMHLKANNTYTEDSDYVVFSIEHLSPFFFLLLFGYILSSMTFLLEVVVHF
INNFRI
>BgerIr652
MMESASFTPISEVENMYLSQQYQHTVVCILRIVIRYFLPIRILIISLSLPVNPFSELMIAQLNE
QVHCPLLIVISSEINSVSQDFGERMYPSHYIMIIESKTDFYRQMYLLLTQDKTLKFNPKARIII
MIVGEGNHELLQIIGKKLCKFKIHNVVMILPTSDDTLDFYTWLPYENLNQCGKFEGAVLID
QWSQEHEDFIFKTDLFPQKIPNDFQGCPIFLSNTSSDKHIHIKAYIEAMETFLLFPLFEKMN
VKRINHAPRSVIDVVTGHTLQDFNSQIIDPVFSHPHMSTELKWFVPCSEPNMRQGHISKVF
RNTLWFMISCVLLTMISVTFFIHRTVQQQESLSYSGVSSCFYNLWALLMRSAVTEMPRTY
RLRLFLLLWIIYCFSICTVFEAHFTSYLVDPGLEKEISTVHEMMAKGFLPIFGKKEKGLWCE
KNDYTKTVCNKPKVRICDEPNPCLMYVMRWKNVSVLTTTLEEEIISSSAGVSFRFCTLKNE
VMQLNYALTIDQYSVYSEKIYDLSLLISEFGLVERMRRHFFRSLRLMMKEVKYTTIKQNVL
SVVRRVHKMAASTDDIEYFALALPHLFEPVCILVIGYGLSILVALIELNQARILKILKKINKYI
QK
>BgerIr653P
MNKHLKHTLIITQLFTVIREINTSTENITELYSDVIQKIVLLNVPVSSVLTIYTHNRFSNSFQS
IADVTLKKINQQLLWSSVISNVENNQHRENTAFPIRQPHYLIFATCEHEDHSLLEDDMDQL
MQEYPWNHEYKFLVVVSYHPSSSPEKCVQNIMKLLWGFIYTDNVVILIRNTDLEEIQAYTV
FQYENGNCGKLGXVVLIRQWKFEEKSSSSYEGMYPSKLPQYFKDCTLTVGSYGPEPYIVRK
SLSNNSELQLTGLCIELLSIVAQHLHLNLSFREPPNQLEYNDILSEFVNLDTRVLDVLVGGV
PLMTIVLDIMDISNSYVYDTIKWLVPCPLPMPRVEKILTVFSRLSWGAIITVFICVSVVLWG
QANFINFGPFSKLSQSFSATWAVLLAVYIPIMPQSFQLRSLFIVYVWCCIAIITVFQAYFTTY
LVEPGYYEGIHTSDDLAKADIQYAYFEIFELVFSGIGYNEPNRLQSGRIKSIDLTGSIKRVMF
QRDIASVVSPYFANYIASVGGATDKSKVVCLLDEAFVSSSIGYSFKKGIPLRSNLNTYLTRSL

QGGLLEKYWSMLNQEIYLLGNNTNTEGSDYVVFSMEHLSPFFFLLFLGYILZSLIFFFEVLTI
GSARLLNNVLHKV
>BgerIr654
MSSAVYLISTTLLLTWTISPGNTSSRYIASQLDYIIHRHFTEGEIIFVSSSNISKDETVDVLLE
KLNQNGSWNILFNNFEMIEEGSVYSISDERSLHCCILFIWEQYDTDGIMETLSHQLRNLQS
YVVSFNRQSKFIIVCNENQRQSITPAEIIELMWDLFKIVNVVVLLPEDDDYTNNPMIHLYT
WFPYEEGQCGEVQHVVLIDEYNGTFRYNANLFPPKVPLDLRGCTLMAGTFEYLPSVILKN
KSIEEDGRVSYDFTGYEIEYLKLAGAAMNATLKYISPTSGEITMVFMDRFSAIMVGLVDLGI
GMMAYHSDLVPFADPTVIYDRDVWQLKFFVPCAEPVTRMQKILDVFSVEVWIGTMLISIL
SMVVLWRLNVFSLSNGSKESRTFSSIQHSFCNVFGVLLGVSVTEMPRTWRLRILVMQFICF
SLIMNTVFQAFFVTLLVEPGFQKQITTLEDFRESGIPYCSHELISSFLVLFSYTQYMSLTSFE
HEDCYSSYIFGNNISFIASSIRLAYFAAEKGFLYKRKFCSLEDDVYSGMSCMYFTKGHPLTD
RMNEVIQRIVEADLVGKYWSELNWSIMLKDGMGVATGKSDAFFVFTLDHLKPVYLLLFIG
SFLSAFVFFAEILCNCIVKQLKKSTQ
>BgerIr655
MINSVTTLLVSLVLLNIKANNTQKINETGVILCLEYIVNNYFFMGRSVMVSLPDETKSHIKS
ERSLFPEGKQLYYEDVASTLLERLNKKQEWATQTTVSNVMFPIMSTEFMDDTDKHYNYII
FTPTFEKESNRPIVEAVDLVIADYNDQSSLNIHGRYVVVVLNNGIKSPPETAMSIFNIMNW
LWPVYNVLVLIPYTQDKVQTLQLYSWFPFQLNGRRAVLLDEWMMGNKSKLLNGNNLFP
SKIPKKFSTKTLEVQPLADELVMYYKGNYTDENNELRHLYEGPEIDILNIIIEHLNISLKFDG
SKYAYLPLVTRIEDALTILSLGPLADLAVGSLPLHHKTINWMEFSHPYFITGLTWYVPCPK
PYPRLKKIAETFPIEVWVLFLFVLILVVFVLYFMSVHTVGKTSSTYNSVTMCFYSMWAVIL
GVSVPQMPLSSSTRYLFLLFVWYSFAMNLMFQTFFTSYLVDPGIIDQLRSIDELLKSDVPM
GYNREACSYFLSGIKDNTSEQIIKKGIDCPDKEYNSCILKVVRDKNFSTLRSEFYIEHFVKTT
MPKKIKPLCSLDDRYKIIYIVTYFKKRSPYLESFNKIFRRLDEVGIVAKKIRDFQESWRYKLS
PDTGKYDEESNDDYFVFSLDHMTISFYVIVVGCVLSSIMLIAELVFFTLKK
>BgerIr656
MRYLKSEVTSTIIFAVIIIMSMKTGNTLSTEENNLLLCLEYIVKHYFFLGRSVMVSFTDKSIS
YTKEHRSLLTEQKSFYYEDIGARLMRQLNEELGWATQTTLTYGSQPIMSVEFMQDTDKH
HNYIIFVPTLEEEDRKVIADAVENVIDDYNEQVSLNIHGKYVIVIVDKGIQSPRETALAILTR
MCKWWPVHDSLVIIPHKGAHDAHTLQLYTRFPLEPNGQRAILLDEWLMEKMAKLQKGN
NLFPSKIPSKFSTKPLQVQMLADEPVLYYKGKYTDENKTVHHLYEGPEIDILNMITGHLNL
SLNFSEPYTYGSLVDRIWAVLTITGTGIVVNMTIGSLPLHPKTIDWMEFSHPYLITGLTWF
VPCPKQYPRLKKIAETFPREVWATFFLVLIITACIIHFISLHTFRKDSSIFNSLVMCFYCMW
AVILGVSVPDMPHTSIIRSLFLLFVWYAFVMNTLFQTFFTSYLVDPGIIDQIRTIDALLNASI
PMGYYRYAHNYLFPDKTDPIETLMKSRGENCDEENYKTCLLKVIIGQNYSTLRSEFHAEYF
VKTTMPSKRNPLCSLDHRFTTFYICAYFQKRSPYLKSFNSILRRIDEAGLLTKRLKDFKES
WRYEISDDASKYDVEENDDYFVFSLEYMLIAFYSLVAGCALSSAVFIAESIFSMIHELKSTR
MTTPQKNDN
>BgerIr657
MKVHVTIVICGIIFIELLKNRYTISQTLTIGHLNLVACINSIVKDYMNFSGPMILTYSITSSDN
PQRTLLTEAVNVQFMDSVLKTLCENPNLSLIVFQPNTIEYINLHFQGCVFIYRLQDENEDV
SVDIPWQMSFFISSNEKFNSEVRFVIVILCSYRSVDSAKLVQLLSEIWESYKILNVVIVIPHFC
LILPTFVNILPDFDYNVNNGSLDIFTFFPYFINQCNEVNQIHFLDSWLGNGKYPYLRNTDIF
PEKITSNLHKCPLRVGTHEFPPIVVVERKNGTVESVNGYETSFIEFLFDNLNASIQYVVIPKS
WNNDLRVDTEKLAHKLYFDEVDIIYGALPLFDEMVKIASPSVPYFDTVIEWIVPCPVKISQ

VGRILNVFPVPVWICIALTFVLITLTVTFRAKYLCPGTKHEISNFLTINESFYNIWSAAMSVS
VSRMPQNQGVRVIFLLWISYCLCINTVFQAFFTMYLVTPRVGRKLSTVKDLIDSRTSIHYRQ
EFKILFNYSNDEEDIYFRNNEDYCTFISNCLYDVVKKRAYATISPSILTEFYIANWSLLDSHL
PVCKLENSILKMDVVSYLSKRNPITSRINKLTRAIIECGIMSKNRRDLMNEIRFDRHSKNTE
DTEEKSENNYFVFKLSHLSYVFLSLFSSYIAGMFVLVVEVTIHLITKTQLSCKVNNVHKATF
KHSNHESRLHEEYRGLNRRKNNY
>BgerIr658F
MRLFCLLIMFEMYLIVSFSYTLPQSSTSQYKDFINCIYSIVNKYFHYNDLMIISHSTIPDDNP
QRRMFPQNADEVYLDLMLKNLSENVNLPLSVMQQYQTNFINIYLHSFVLIYWLQDENEDI
SMSILRQLNPIINSNTQFDSEARFMFGIMCSNKRVDSSMLIQLLSIIWESYKILNVIIVIPHLC
LNVSSVNILHNVDVETNNGSLDVITSFPYKSINQCNEVDQIYILDKWLGNEKNIFLKNENLF
PQKIANNLHGCPLKVGTHYFPPAVTVMEENGIVKNVSGYETGFLELILKALNITIQYVVVQS
SPDHNLRVDAKHLLHKLLKGEVDLIYGGLPLRKVLTEVASPTVSFCETVIEWIVPCPVQVH
HLGRLLNVFPISVWLCLALSFIFMTFAITFVANYFCSDTNCEISNFRTLVQSCYNIWSVCMC
VSVSRMPEKHKVRVMFLLWVWYAFCIDTVFEAFFTTYLVNPGIGKKSATVKDLIESETEIG
YRDEFKLLFENSFDDEDIFMLNRRVDNCREPDICLQRAIKSNTFATISPSILTEYHRAFWS
WHDSHLPVCKLENEIITIDLVSYLSQKNPIRRPINSITVTLIECGIMAKYMKDMTNHLRME
RQLKNNEEEDGIFEGEYFVFKMSHLKYAFFSLFGGYIIGMFVLVVEINLDRIHVVSLYFTNK
RQNRLDFKNHDPRLLRNHRRTDREKRETFIYFRCKY
>BgerIr659P
GHIYQYFNSNFRICHAVGEYYKFLLFIYLGPIVNIVSLQRNLEITNVSNIIHCLSKAALPNFIQ
YQSVFVSLPVSYLNGSNIIPIEQLIIKNEWDVRTSIEGHETDLYITWESNEKYHNFIILFEFKE
MDEEYIIDSMNDRFSNLQNGGSLNYRAKFIVTLTIQNCKSLEKLVFTIMKELWKYYSILNV
LLLILEPMDGDSLSENSANWILSAFTSNISCGIIQRTELIGKCTQDKIHSFLNSRMSYRKNNL
NLFNGCSIKLISYLVPPVIMRASNDSDSEYFGIEVSSILTILNKLNITPIYQIIPGKTTDVFKQF
FTTIQQLSPSSTDIAIGALPFSGYFNPFSESTIAYENVQINWVIPCPEESSRWLAFTETFSLPV
WLCICFEFLIVSVSLKLLAQCAKFKTVPESSNYSTFVSSLYNMFSVTLGVSVSKMPRTSNLR
VLFFTWVYYCIVITLIYQTFVTGFLMNPGYEHGIESLEELIDSGIPYGFFGEVHRFDELDPTY
EIISKNKINCPSIYRCLEMPIKYRNFATIGYDFLIQYFKIKLSFYGHSLPICKLPVDVFQYRIST
YMSKGNPLLATFNTVIRRFLEAGLYVKWRDDYLSQFKFDGKSIDGDDVDYTEPSKYDLVS
SKAFSLTQLQVVFYMLLFGLLISFCVLFSEMLYFKICLTSNRSVKPTRIRQISLYQNRLKINK
PKYYKHRVNYYIHESSYYN
>BgerIr660
MVTICYLHFRSWLSIEGYLLLLYMETVVSKIGLESATVIRSNLSKTTKCLTNAVSSTFVQDQ
PIYISVPETYSNGSDIFPIKDFFANNEWFFRISSEESETDLYATWEINKKYKHYIILFQFTRK
ELEEDIIVATVNERFSNLQNSFLWTHRGKFIVLFIVQGRISAQTLASRTLQELWKYYSILDV
LLLVLQATQDNSSCESCTLNNNWTWSTFTSFTECGLIKYINFSDKCIPDKINTSYVTSSPNQ
KILDMFNGCKIKIISYLVPPVVMKAPINSGSHYNGFELTSILTILNKLNITPFYKILPDNTKD
HLQQFIYTIQELVPSSTDIAIGALPFSGYINPFSAESTIPYGSTQLLWIVPCPDESSRWLAFTE
TFSLPVWLCICFEFIIVSISMTFLSQCSKFKSVREISNYSTLSSSLYNTFSVTLGVSVSEMPRSS
NLRIVFLTWVYYCIVITMIYETFVTGFLVNPGFEHGIQTVEELIASGIPYGTLGDFHDFADSD
NVYQIISKNRMECASYFKCLEMPIKYRNFATISDGFHIDYFKIRLSFYGHPLPVCSLPLEVFQ
FSFSTYMSKGNPLLGVFDKIIRHFLEADLYVKWTDDYMSQLKLDGKSLDGDDVDYSELNK
YDLGTSTSFSLVQLQVVFYMLLFGLLFSLLVLLLEILHFKIICNRTSYKSVLTTQTPLNQTQN
KNIRMTQSSLYKSRVHFSQTKYYRQKITFVMNDFLNEI
>BgerIr661

MWRITCALIYCFGLYNDLIKNIIQAEPRMPSMTDYVSNIFKQYNEEGGTVFISLPKAENLIA
EVRSLNMKLTLGSLVTDNILKSLNQKLNFPVKIQNSEKNVSNGENRLNYILYYLVFFRFTSE
TNLEENIKQQFLNFELSLNPTGMYFLCAIEEFSGQNEELAHQILRRVNQLYNIWNILLILPK
QRHPENFHCQQTEDTKENLQLDTYSLNPMGIFKPGNCSSQFEIGLVNRFPNINKTRNIEVP
VNLNGCTLHISPSLLAPYTLIEEIKDQNRTVTYNYSGLEVAYLHLVAEKLNLTLKFHPPISG
NAFETRFRSLQAIQNGNTDIVLGGMILLPLSLRIAEPTLPYIKQELAWIFPCSQPFTRLEKIL
DMYTLYVWILILSVLNTIPFALWFSSKFDYNSRISEPNSYKSILNCLCIVWAISLSSSVKDTP
TKLRTKFIFIIFVYYSIVMSTIFQAIFITFLVEPGFPKALETFEELLHSDFKYGTNSGFETWFS
VSGYNEFNKFTGITTNCEDIENCVIRLLRREPIILMSCRNYINYYSSTFIEVERKWCSLQRDI
FSVSFSMYFRKGSLLLKRFNNVLRRCIESGLIEKYWEEMKLRRSSFNAKEHNNFISSEELSI
YNNMYFVFSLNHLKLAFIVLLLGYGISSCVFLIELILKYINVCFMSMCEPEVSRRLTGHGPQR
AVKPRSFCFMKSEHQTYCPKYAKECLYSY
>BgerIr662
MIINFIFQMWGITIIALLFFSRLKNCLLNNEVLAELHMSSMADYLSNILKQQNEKGDTVFISI
PAPENVTVRARVLNSVSTTESLIIEKTLKSLNQNLNFPIKIQNSSKEVSSEEHNINHAPYYLV
FLRITLESEIEDNIEEQFNSIERTKSLDSTGMHLICIVNELSGHQEEIAQRILRRIHKLYNIWN
SLLIFPKQKCLNISENSIYQQTEGNEFNLPFETYSLNPSAIFSSRNCSSQYEISKWTNANDTT
FLLRNTNLPENLNGCTLHVSPSVIPPFASIEENSNQDESVTYKGLEIAYLQLIAENLNLTLQF
HPPMPGNVFETRFRNLQIIRNGNTDILIGGMVLFPFTLQYGEATIPYVKHELRWVFPCSEP
CAHFEKILDIYTVTVWVFVFLVLKLIPITLWFFSKYDCNSGISDPNVYKSIYNCICIVWAISV
GVSVKHTPKTLKTRLIFIIFVYYSISISTIFQAFFITFLVEPGFPKALETFDELLKSDFKYGKNE
GFEDWLKISGYHEFNKFSNNYIICEEIETCVDKLLKKEPITMTCCPEYINYHSSTFIEVPRK
WCTFKQAIFSVNFSMFFQKGSLLLNRFNYLLRRCVEYGLVEKYWREMKLNRSSFNAEVN
NNFINSDVLMNNDDTYFVFSVHHLKPVFIVLLLGYSISVCSFLTEFLLKLTNNWFMKTEYR
NNCLL
>BgerIr663
MILTFLIFHLIISVTYSHTLSIQQEQYLVSCVKLITEKYLAAHNTLIVSTPDLQRNIPEFTYDL
KTYDHGESFSIEHFVQTVLEESVWPLILFQTSEGFTPESTAVKYTYHGYLIITWPDETGDIL
GNLIQQLTKLSDVKYLNTRGRFLLLIFIENNLVSETALYISQEIFKTFLIFDSLILISNFNEIIN
VSQLEDKVFFYTWLPYISKDDVILLYEDSFQKNTCITPDLNLFPVKLPEKFNTDPVIAETYI
SEPIVKLKGNYTDENGEIRYDFEGPEIYIFKLLMQNLNLTYHFVKPEQNEDIYYQILGLAFN
LMQRNTDIAFGGIPIYLESSLILDHTISYYSSGDKWFVPCPKPIPRLERISKIYRWSVWLLMA
VVFLLITLVIFLSARYSVDNCNYYHSIQLCFINVWAIVMGVSARMPGAFTLRCLFLMFIWTS
YAISTVFQAFFTSYLVDPGLTKQIETIEELIFSGIEFGTNEVKGIAVDEKDWRMKYLSANRK
NCSEYKTCLLRVIKEGNFATLRSEFFARHFVETTMPKRLKPLCSIPDTFISHFITFYLAKSSP
LLEPFNKLLRRMMEAGLIEYIMQNFMENWRFEIDPNAELEIDDIPGRIFFVFSTEHLSLAF
YVLALGYVLGFVSVLFELMLNKM
>BgerIr664
MMIHQIKDQQLSDLHRRRSSLTLASIVSLLNKIHHLIMHRCLFPLLSLYLQMGLIHFLAMM
SASAQNDTESCLAHLVLAYYTEGQPLLVSMPSTNSISLLSRADHFKTADYLMELLNNHHH
TSLRVFNSNSTSTRNNNFRLYNFEELPKGNIIFLWCDGENDDHLEMLMTVVGNAAETLSL
NFTAQFLIIAYNFPPESPQELAFSILEYLYTNLNIYNVVVLTVSQSDANTEIQSSILDKKLPPP
KLELFTWFPLHIRQSPTFDDIIFLGECSSNDSPLHKLALYPNAIPKSFFGKKVKVSAVIQEPV
SWFKGTSIDQDNETQYLISGQEIDLLMIVCNKLNLTVTFQKPIPSNEGIDKKVVATMLELM
NGISDIAIGSIPLQSPFRVQFEHCGAVGYYLEKWFVPCPEQIPRIQRIAGIFQWTTSLLLCFV
FALFVMSMWTAAVLTKETESTAFANIGCCFINIWLLYLGGSVKDVPKAFVLRMLFIICIWY

AYAMSQLFQAFFTTYLVEPGYEKPISTFEELLNSSLELGFEESLSYYFKTHFLMDFIGLLRN
RIHDCLNLENCLHRVINDRSYATASYESWVNYFVKLKYPSSHRVLCSLNDHVLTNQVTIFL
SKASPYANSFNRIIQYAWDTGVINYLHERNEDSYRYATNTGDGGDGESSQDYFIFSLQHLK
MSFFVLFLGCGLGFLALVFETILSYLKNRQVPSRLSYTTGLKSATQP
>BgerIr665
MRQFLLLTLATVYSFNSENHTLFQMLNESKSFARNPQLAKSVYRILKRQVSSKETVVVMV
TTDASSNIAIQEIQTHWPVCVTGTYHIFEQSDLIEPQKYNYFVILTSLEEPLEVQLDELNYQS
LLNPRGWYLVVSRRHVDFPELEAQSLLEVTWENYFILDSLIMIQDEYHIYTWYPYHASEK
KIIHLDFGSWSFPQKIPTKFFNTTLKLVTFEMEPILHYIGNETGLLNFEGPEVKLLNLVLDS
LNLSHVITILERENTFEELGYKVVDYLSTRYADLFFASQPILRGPSSLLDFTISYYTSGYKWY
VPCPRPFSRIKRVTSIFSVKVWGLMLISFVLVIIIMWRVGLNKFESKYYEALCNCFITVWSIL
LGASAPVKPNTKILRMLFLLWTCFSYAVCTVFQTFFTTFLVDPGSYALVNEVEDICRLGME
FGYTYELDNIMFDFNKNWMCGGVGRVNCSMEKYAGCMDRVMKDDKFTYFGIEFYADYF
RIVNISRGGQPLCSLSGYYKTLNIVTYLTRGSHFLAPINRVLEKIIESGIFLKFVDEMKEGWR
IRSGSEAVHSDPYEDIEYFVLTVANLQISFWFLGGGLAISLLVLLVELAIQF
>BgerIr666
MLAFISLSSIFLQAVFSQSFTPGEDLAIYVHSVLLRQFASGRSLSVSLPNWQQCGSTVKFGVI
SSILKFLHDDIFWPLNVFHYNNDKPEANSSNLLKMQGYLIFTLPCPDVEDITADLGKQISGL
SSLEVWNAKSQFFIVVTNMMDQFIGELANDILQELWNYNVFKAFVIIPIIAEFNTAIKKPEL
MPKFNLYTRIPYHSAKICLKFSDAFLLEQWSKEFNNNSVSLKKILKHTIPTNFNSCPLRVLA
HSKEYKWEVDNKGEKRLKYYDPLLLLLSVVLEKLNITLVQTPPKVINDSYKEMENVLENV
FFGETDIGSGVIMDFWPAMFADFARNGIAYQCVWYVPCKQYKSRVTTVTRIFNASVWFTI
ASSILLTSFMMKIITIICTHKGVSESSLFKSISNCLCSLWAVLLGVGLHKLPSSSSLRTLFLIFV
CYSFGLNTIFQTYFTSLLVDPGYEHQIESVEEFLRSNLEFGYFPAFDYHFFVSSKKSDKEILK
RRIYCNDRKYCFDRVNREGDFVYFELAVIGELYKRLYDEDARMCSLDQGTIYTIATIYMKR
DNVLLGIIDRKLLELFEAGVTMKFKYEQILAYKIGILKVPELQANESFVPAYNLKEKDYRYV
QLGLTHLQVAFYLLMLGISLSIISLIIELFYITKNKTNL
>BgerIr667
MPYRNKAFEINIKIFLKCVYTITFITIISSSDQITSTNKIPIHRQMLKCVNNVLRRYFNPAWPI
ILSLPDESEKESAPNIINLMPRSDGSHLNKMLIQTFMVEYDWPLVILGPFESTETNIKVPRQ
ENFLIMTQDIKDFDEMEEQITDLRESQFWNPKGNFLVLVSNSEVSLSFQESFAYDVLHKI
WYDYKILKSILVIHDMKSTPIIQTYAWSAEQTATECLKFQRIVLLDTCNYNFTSNEVSENK
VPTNFKNCPIRIMTPVRDYYTITNEENQTSYQFDEPELTILDLVIQKLNLFPVVTPPFPKDS
DFYTNTVATISDVFFGKVDLGIGYLFLDTEPTKFTDPSLPYHFSYGYRWYVPCGRKLSRVT
TMSRIFSATFLLALIVSIALAVFIMKLLASDRNRNLKLSIYKSSIMCLYCIWAVLLGVSVPAQ
PESLKLRTFFTFLIWFSLAINMVFQTIFTSYMTDPGLENTIQDYTELINSNMELGCEYNLDI
AFNRSVDERDTEVRNRHINCTDPIYCLSRVEKTKNFAYLAKQHDAKWYSKNRKNPLMCI
LPDGHITFLLSMYFTKGSYFRDPFNAVICTLMEAGLVTKIFSDHLDAKAVGHKETAWVKR
TVNISLDENSGDDEYITLSLFHMEISFYFIFVGYIVSFAVFIVESLLNVINLR
>BgerIr668
MPYRNKAFEINIKIFLKCIYAIFFITVLTSADQITSTNKIPIHRQMLKCVNNVLRRYFNPAW
PIILSLPDESEKESAANIINLMPRTDGSHLIKMLIETFIVEYDWPLVILGPFESTETNIKVPRQ
ENFLIITQDIKDFDEMEEYGIEQITDLRESQFWNPKGNFLVLVSNSEVSLSFQESFASDVLH
KIWYDYKILKSILVIHDMKSTPIIQTYAWSAEQTATECLKFQRLVLLDTCNYNFTSNEVSE
NKVPTNFKNCPIRIMTPVRDYYTITNEENQTSYQFDEPELTILDLVIQKLNLFPVVTPPFPK
DSDFYTNTVATISDVFFGKVDLGIGYLFLDTEPTKFTDPSLPYHFSYGYRWYVPCGRKLSR

VTTMSRIFSATFLLALIVSIALAVFIMKLLASDRNRNLKLSIYKSSIMCLCCIWAVLLGVSVP
AQPESLKLRTFFTFLIWFSLAINMVFQTIFTSYMTDPGLENTIQDYTELINSNMELGCEYNL
DIAFNRSVDERDTEVRNRHINCTDPIYCLSRVEKTKNFAYLAKQHDAKWYSKNRKNPLM
CILPDGHITFLLSMYFTKGSYFRDPFNDVICTLMEAGLVTKIFSDHLDAQAVGHKETAWVK
RTVNSSLDENSGDDEYITFSLLHMEISFYFIFVGYIVSFAVFVVECLINYVYLNVINLRH
>BgerIr669
MEMHFLIVSTTFCFIWSLTCSRNNALLTPTQQHIASYVSKIARRYFKPGVPILVSLPIKNEN
GDASSDEEENVSEEEGSRVSLIENPIKTEQIHILNSLLVVLNEAMLWPLKVTTLGYTFEIST
DIVAPIIRKDEGYLVFTPSDMNSEEIMYDFEDQMIDISGTILWNPKVDFLFVVTDIIAESPHI
LAEKILRKYWNEYNVLNSLIMIPTVSESSADNKTMIKVNIQTYSWRTHQSKGKCMQFADA
YLVDQFFLGMKLNETRENNLKNNKIPAKFEGCPLKVQTPTKNYKIERDQNNETVVKYFEV
ELYFLDIAMKHLNLSIVYQPPLPVIRNYVQNVVKATTTLVMGDSDIALGYMPIGDEMIQFA
DSSMPYMFLEVGWYVPCGRMVSRITTLSRIFPFSVWLTLAISLSLSAMTVKLQASCFSFQA
ETSTYKKFSTSSLLVWAIILGVGVPEMPQSSRVRLFFILFVWYSFAVNMIFQVFFTAYLVNP
GTKAQIQNFQKLLDSGIEFGFDPFVEYHINNSPDKRFIKAMVLHQECRDRHYCFGRVDITG
DYAYMDLEFLADWYRSVRKDARMCRLDDGTILMMIASHFRKDSVFTGVLDSIFFRLVEAG
IAIKLRKEFLEEIISDNTKFRWLDRIVNVTLVDPEAEDYDVKDEYVQYSLIHTQATFYLLFM
GYGISSIVIIVELIFYKVYNKYKNIEQ
>BgerIr670
MFCFIDMENATIFTSIYIQTMSSLLSPEYQYLGLYIENVLQRHLAPGLPLLISLPQFDIGMTS
QESSYLQEIESLLYILNEKSTWSLTIICCMRSNFVNYYSLEPTKQGYLIFLHPDRYANENNN
NFINEISLLSAKKLLHFKGQVFVILNSESPHIFIPDVLKFLWKFNIIKVFVIGVRVNKSKDEKS
FLGYSFSLYTRKPYHSNNKCLEFSNVIVLEKWKSGQKLTNLTQTFQHTIPNNFNGCPMKIS
SCSGNYKLLKQNEKMILRNYESLTQLLYIALEKLNITTIQNLPSKEINHIDIQRRVLLDVVLN
KADIGSGVLLVKPASEIADYMRYYLINYVHWYAPCKQYKSQVTTLTGIFDMSVWFLIFSSI
MSSAVIMTFICSFYTRNGVIESTTFQSISRSLCSLWAVLLGVGLFRLPRTFCLRSILLVFVCYS
FSLNTIFQTYFTSLLMNPGYEHHISTINELLSSGLEFGYNPGYDYHFQGSPHSRDIQILKRRV
ACYNRSYCFDRVSKSGDFCYLDLEFIADMYKCRRKDVRMCTLHDGSMYAYSTIYMKKGSH
LITIIETVFIQLYEAGIGFKLKEKQYAAFKSGKLNSSWFTNEAIENIGITLDNKDALLMLKHL
QVAFYLKVIGILLSCVSFIMEIILHKFKIFCT
>BgerIr671
MIVTPFAQVAMSLLFLRREAFVQYIHNVIERHCSQEETLLISYLHSGPAENNESLAMNNFL
TSRTEDKWPAIILHSPDKNRIKFGRPIKVQTYLIFVHYLPKNFNGFINQVESISYFLWWNTE
AAFFIVISNVFSDSPQSAGLKILQTLWERYGIIKTFVIVQTLNRSETIPEAQLSFHLYTRLPTH
NESLCMAFSSIILLEQWFWDNDNKFPNQSFNKIHSIPTNFNGCPLKVSSALKEYKTVGYNN
TALTLEYYEVEMTFLYVLFEKINATLVHNAPHTNSELYSDDLFTSYMDVVLKEADIAVGGI
PAIEEISLFFKIARSYFRYRAIWFVPCRQYNSRVESLSRVFDNYTWLIIVTSLLATAITTKYL
ALLHDTESVSFRYFSTSLLLSLWATVLGVGISDLPQTLNLRILFSIFLWYSIAINTIFLTYFTSFI
LDPGFKDRVHNFDQLLSSGIKFGFDEVYDYHFLVTQDKSYLKVLNHREICLDRKECFDRVS
ITGDFAYLDNSQSMLMYSILNKDPRICALDDGGVSTPMAMIMHKDSVLIEPMNHVISKLFE
AGLPQKLEQDYLLLYKSGKIISKWFSKNGTVNSYNNEKNEYEPLGLDHTHIAFYFLILGYIL
GMLGVFVELMHYRLKMHGKLVQ
>BgerIr672C
MAEILNVFYLLFCTVIIVSMVGICQSLQNIQKLEQYRLVECINNVLDSYFTKGLPILLSLPDEI
ERNSVSSFKNLLPRNDDSHIVELFIKTIYEEHNWSVVLEGPEEEFGNVLLEPKRQINANYLL
IIPFISNLDEMEHYGFQQLLNLMHTQIWNPRGNYIIVSRSSLKSAEEFALYVLNKLWYDYKI

LNSFVLFTDLEEDMHDALKIETYTWIPQQTIKECLIPKYVVLAQRCEIKNANNYINSDSRIR
NKIPSNFNKCPIIVATSVKDYFWETNENNETYLRFEDADIVVLDIILKQLNMTRLTQVYPK
TRDYRTNSVNALISAITGVAEIALGHIAILARTRNFCESSVPYTIYRFKWFVPCGRNISRLSS
MGRIFTFSFLLVLMVTILLTIVVMVFLASVGNYRKSESRNYTFFSNCSEYAWAVLLGVSVPK
LPTSLKLRCFFISFVWFSYGVNMVFQIYFTSYLTEPGLQHVVEDYTELMHSELNIIYRPNLF
YYLNSSVDKRDSETIKRGVSILDSMYCFNQVDKTESFAYLAQEYDSRWYSHNLKNKRICTL
PDGGFIFMSAMHFPKGSYYVDSFNRLLSIMLEAGLIVKLEDNRVQSVVDKRADSSTAYMM
EYSNISYEETN
>BgerIr673
MMFFSEPHNIILFLVFFTRQFNLHLKCQLISFLGGCTVLYAITLCCSLKGSIPTSELQGFPNF
EENLVISIGEVMNWFFVRNRAVFISFPNNCNQEEETIEYDKANSSFKTLINYSQLRKCLQD
TVFEEINAKFNWPIFVHYPGSDLTEIKFQYKEDNYLIFSGHDLYELDTRFSEIILSKSWNSR
ANFLIVLFEKINESPENIVRTISEFCWKVYRIYNILIMIPHSIFTSGENKFVIDGLTWYPYSGH
KKCEMDLDIVHQFRQYDGFHSILRNITPLMENKLYRNFHGCTITVSTPLKNYFWEDNGKT
LVYKEPQIDFLGYVCEMLNLSLTFHNKTAHPTQGHYENTFDSVVRVTIGTADVALGDISID
TRAAQWADFTVSFQENIFLWHVPCSSQISRIGTFSRIFDYDVWITLVITYISCVFVIWLLSVR
YKKQASTYKTLARTFTNLWAVILGTSASQMPRTSSVRMFFICYVVYSYAINTVYQTLFTSL
LVDPGLEKQIETLEELLDSGIELRYDPYLNYVLDTLDDWRYTEVFKKVKNGSMRLHALNR
LSVKRDIGVLENTIVRDIYLQHRNEKICSISDGYIKFKMTMYVKRGSYLLEYLNFAIFCFME
AGFMNKLMEDFSSSLVANGFDYAWFRNERTAKFLEKEYEADSNEYFPFGVKHLQMSFFL
FIFMHILNIVVFFTEMVFSKKNSRPVVKSQNNLIYLP
>BgerIr674
MLILLLATLSANENLFLSTEQQHLVTSVLTIIQNQFTPGRSIYISLPSYEVPGDFIFVAEILIKL
VSKGINWPTNILRSNEEPPRKYYYDSGHSYILFLECKVEIMDYLGKQLVQLKELTSWEPRA
KFLFILNKCVTEQPANIISTLFDELWGFAGIHNVVLLTFHSFEHLIDEKNCIFAYTWHVFNK
NGKCLKVNEVILINQWLMEDQGRFLNSNIIFQDKELKSFFGCPIRVITTSFEPLISLSTTELD
ENGITRFKLKGMEVGIFELITRKLNLNVLYNELYPPNMSLFDKIYEAYVEMLLGQADVIFG
AQPISKRTSELSFEAFPYDFVEYMWFVPCPRQVSGIDKVAKIFTISVWLSMLLVMLLIVTL
MKWISKKHEYNVYKSVASCLLTVYAVTLGISVSQLPRTFQTRFIFLLFVVYSYCISFIFQCFF
TTYLVSPEIEGRITSIEDLLKSDIALGHNNDLSQIIVDLANKNLVKVFHKSRECPHETYACLL
RLLERNDLAVLSSITEFKLSLVTSNRSLDTVPCVINEYFSNLMLTIHLRRGSPLADRFNYMS
MAVLEYGISKKIVNDFFATLQLERGTTIDHLDEDSKSEIKYFPFSVTNLKLANYVICIGSMV
GLFVLIGEIIYFNFNIEEICFKRNGQSVRRFAVQSEKTKIKLKRRVYRSNGFQHIHKK
>BgerIr675
MYIMTPRDRKLYYVLVIIIELLKVNGLAIYPEEDRQLLNCIETILKEHLSLNDAILVSSKELF
HNSQNNSRRSLLGPIELPHISQQFLKNIYKDNAIYKHMSDIFIGEPEYDIQFHKTSACVIFLS
SEERDEDTVRLLQSRMIDLVFRYSFWNSRAKHIIILDGNSKENKTELVHCLLKMLLDEYM
VFDTTFIIRYVNPKASPINFLDIYTWFPFDLKSHIPILFNQWVLEGDGHFLREVNLFPSKLP
KDFKGYKLEVLFTISETMYLNNGMNASFDYYTYDDISGGEIEVIKLISECTNIDFRFIHPQSE
NIPFFDRYKTTILNFALGKGDLYMSMATSMKDALLWSDMTFPHVVTGSKVYIPCPKPNPR
LESISKIFDISVWLFVSTTYVLVTTTLWLIGRSTKNYHTIETSFNTLGMCSYNTFSLSLGMSA
MEMPRTFKLRIIFLSFIWYSFSVSLIFQTFFTSILVDPGKGKLFTDLDDILDSGIEFGSLANLD
YLYYRDEDDPRAKYMEKHRKDCHRFEVCLQQVVHDRNFVQIVYDYWARRYTATHLTQD
KKSLCSLDGFYSLLFISLYLRKNSPFLEIFDRLIMLINASGFIAKHFQDEEHNWRLIGMKNT
KSLEKVQPNDNYFVFNFTHLELAFLCLFIGFVVSAVSFSIELVMKRKSIVQK
>BgerIr676

MDTAEVLTFELCYMYRSIYLVQPNLAQADQLLMKNVQSEAGKNIFLIAFAQNYSGIPIFLE
AVIPTSELLLVYVVTSLETEFDASLVSLFLEAFSNHKIYNVIILIPSQKINNEVRSLDLYTWKP
FEPVTNCGKFKNVVKEDKWGFEGQGSFTTNTNLFPKITPVNFKKCPIIISSITQKIGFDNTE
ISPIARFVELDLVLILGQKLNLHPTYDYQVEKIVFPDIFIGAPPLNSLNGFNFSAANRFLGTY
THFTTQLKWYIPCPKPIFRHGNILRVFAIPLWLCLISVAILTAITVHYLNTRTISLNKTLPKP
DFTSIFLKIWGLMLSTSTDFGVLNFKFRTVVIFWVVFCFFVSIIFQSFFTTFLVEPGVENKIS
NVKELIESGIERRTSKFQYNIWKNVFDKNGISVIVDEQFSIEKSLVRYIVHNNIAIIGDASQV
DIYCRLAGAVLKFTCCHLEDQIFSVYYSMIITRSSFFGSLVNKLLIRIIETGVIKKLELNAEVIT
KLATNHLQVKSISKFLLVNTSGYFSLAVYHLNVTFYLLLMGYIASIITFIIECTHYYILRYFK
>BgerIr677
MEDDFLTIQDYHIVACLKEIVQQHLPQDEYIIISLPPFEKSWSPERVLRRSNSYSVAKDIAQ
VFIKELAGIYRIVVLLEPNEMLVNNIWYQSLSQTFVIAFWPNWNNIQLILDSLSPTLDLHLI
VVLRMPYNCMSVPKIVQDLAALKVFNLLVLIPCESVASTHFNSISISKEKAISLCIYSWYPYE
PINNCGEFKNVVKIDEWLLEGHGRFLQNVNLFPKKSPINFEKCVFIFDILTSSKFKTPSISGV
SYLTLFLEYLIIGLFINKLNLTAIHTSMNTKVTIPDVIIRSPPLNVLNFAEYEMNNLTAAQPY
QVTETKWFVPCPHPFIQHGKFYKVFSYPLWIIIIALTLLIAFTIHLLIKIANAYENKSEIYVTN
IVHILLIMWGALLGEGVKIRPVNYVFRTIVLSWILFSFMISIVFQSFFTSFLIEPGMEKAISNL
KELMSSNVNLRAPEIEYDIWNDTFKEYNMTVKTDRMFNEQMYFEEYRRYQNFAFVGDVS
YANFYCPPVHKYQAHRCCNIDDQTITVYYSFLIPRSSFFRDLFPMVLFRVVESGLVNENKR
MIHEHPGFQKQEWRTVSVSKFPLPSEEYFTFSLKHVQVMIYLLITGYTVSVIAFIVENKLYS
RWN
>BgerIr678
MDLHTSIILLMVVTPIFSLVLLPTEEEFVITCLKRIAEQYFTEKSKLVISLPDLEKRPQVDRY
LSISSTKSTSMDMAQLIAKELSYMSKLILIIQPNVENMNSMLNATYTLSSYQVLIVFFECTN
LSKLYFEILNTKPDFIVILVLTSHYSDFENETVTRLFTIFSKAKTFNLLLLIPNTHTQSKREEI
KSLALYTWYPYDPVENCGRFNKAVKVSEWVLEGEGKFTEDSKLFPQVVTANFTGCAIIAE
RFFENANKNPPDAIRFLLEYVEYPYLNMMADVLNMDVILSELDNFKMFNDVLIGALPLNV
ISPLVYESPLQPFRAAQLKWFVPCPQPILRHGNFIRVFSYEIWICIIVVTLLITFITHIFYKYIN
GFKLSSDFSCTFLTVWGILTGVSTDLRSGNLKIRIVLVIWVCYCLVISTIFQTLFTGFLIEPGV
DGGISDIDQLNNSNFLRGIPIVEYITLLNILNKSNIFSITNVHCTFIECLNHYLKRNDFAFYAE
VSFMSYFCDSLGLRCCTIDDMAVTVHYSLLIAKNSYYFDVISNTFLRVIESGIMTMIRGKAE
ELHKVSGSDILIGTDDSLVHRSFEDYFIFSLNHLQVTFYLFGIGCFISSVIFVCEMLNPKLKLK
QFPSEIMK
>BgerIr679
MDLDFSVILLMVISPISSLVLLPTEEKHFITCLKRISEQYFTDLSKFVISLPFLEDSRSSERSLS
TSNTKSTTMNMAELLSRELNSMSKTIVIIQSNEKNVESVLTGSNLMYTQHVLIVFFPSKIIS
AVLSTKSDFVVILIFTSHYYELENEIVPAFFKTFATGKIFNLLLIIPKMISQESNMVEIKSLTIY
TWYPYDPIQNCGKFKKAVKVNEWVLEDGGKFTGATPLFPQVVSTDFNGCELVSRRIIPNT
DYTVGIHYLVEIFELEHVKISAEVLNMKLTVIEEAQKGIIPDVLLGALTLNSLPKFAYEGGLQ
PYRATQLKWFVPCPVSILRHGNFIRVFSLHIWIILLIITVLTASVIHLLYIATNDSKLRHSFSS
TLLKVWGVLTGVSIDLGPANLKFRIMLITWVSYCLLISTVFQAFFTGFLIEPGFYVRISNIVQ
LNNSDLIRGIPLEDYVPLNDVIRESGIFSITNVTCSFVECLFQYLAGNDFAMFSDVSDMDFF
CDYMGYHCCMIDDMSITVYYSFLITRNSYFFDAISTIVLRIVESGIMSKIERDIIERPTIKESVL
VTGSLDELVHDSSEDYFIFALHHLKVAFYFLGMGSFISLIIFLFERFISVIYFKIFFSKLLK
>BgerIr680

MEIHFSILLLMIAQCSAAILLPEEETHVITCLKKISQQYFTTLSKFIILLPDVEETSPHRDRHL
LNRNTSSTTTDMAQSLLKEVNSMNKLVFFIQPDSLNPKNITPWLEVPGPIYTNDVVIVFFT
YNNLETSITKFMHSKPELVIILIVTSEYSEFEDDIVQRCLGVFAEANLFNVLFLLPNLHISKS
KRTHVKSLSLYSLYPNYPIKNCGVMDNRMKVNVWKLDEEGKFLDNTEFFPKVVASGFKG
CEIRIKRSKLEGSYLGFNSLLENIGIMYLEMATNAIGMKLKDYKWDEKLPADVYMDVMSL
KTLNFFSTEFPTHIFREIKLKWHVPCPQRMARHGNFIRVFNYPIWLQMFIVAILFILLIHCL
YKSYKDSKLKPDFPDTFLRIWSILTGVSTEVNTSIYKIRIVFFIWVLFCLIVSTVFQAFFTGFL
IEPGMYVPISNMKELNRTGITRAIPLHEYLLILHSLTETDALYIANSECYFEMCWYYYMTT
KNFSFLADTLFMGPLGRGLGFPTCTIEDNSITVYAAFFIPRNSYYFNIINNFIIQIAESGLPEF
LDKYVAEEMEMALGPMISSVQASSEDYFIFGMRHLKLIFVSLGIGYLISSVVFAFENFHHKL
CLQRFNKRF
>BgerIr681
MKTCNFISFMLQENTTTMDLLFSILLMVIAMCSASVLLPKEETHVITCFQKISEQYFTTLNK
FIILLPDVEKTSPQEDRHFLNTNSPTTTTTDLAQSLITEINGLSKLVFVIQPDILVVKNITTW
LEVPGPVYTNDVLIVIFTNINLKIVLSQYIHSKPDLIVILIFTSFYTELEKEVIIMCLELFAKVK
LFNVLILIPHTNNDKSKKAEVESLYIYSLYPNYPIKNCGVIDNRLNINVWFLDGEGSFLDST
EFFPEVVSSGFKGCQISVRMSKLESSTFGFMTLLDNLGLMYLRMAANAIGMKVKVYNEDEI
PSTDIDLFMEVMSLNSLNFFSTEFPTYIFREIKMKWHVPCPKPISRHGNFIRVFSNTIWLQL
FLIAILFGLVIYWVYKSSNYSNSALDLSSTFLKIWSILTGVSTEVRTDNHKIILGFFIWTLFCL
IMSTVFQAFFTGFLIEPGMYVPISNMKELNSTGVTRAIPLREYTYILYALSENDALDLVNSE
CTITGCWYGYANGNNFSFLADNLLMSIIGSGLGFQRCTLDDNALTMYTTFYIPRNSYFFKII
NQLIIRIAESGIPVFFERYYVKEMDSTLGPIMKLDQRETDDYFIFGMHHLKLIFYSLGIGYLI
GLVVFVFEKFHYKLCFLKLKN
>BgerIr682
MMEFHFSILLIITQCSAAVLLPEQETHVITCLKQISQLYFTTLSKFIIILPDVEETSPHRDRYL
LNTNSPSNTTTDMAQSLLREINGMNKLVFLIQPELLDLENITTWLEVPGPLYTNDVIIVFF
TYKNLKTAITKFMHSKPELLIILIITSEYDDFRGPVFQKCLEIFAKAQLFNVLILKPGFHVNK
SKERVVKALYLYSWYSNYTIPNCGVKDNRVKVNTWLLDEGGKFVDKPEFFPEIGASGFKG
CHIYVRKFVWTDQYSGFNVFIEKMGYMFLKIAAKALGIKIKVRGPDDDHVIDVYMDILSLK
SLTTFSNSRPTCNFREITLKWHVPCPVSVSRHGNFTRVFKYPTWLQLFLVAILFGLIIHWL
YKNCKYLEIAEDFSSTLLKIWGILTGVSADIGTDNYKIRIAFFIWVLFCLIISTVFQAFFTGFLI
EPGKHLPIANIKELNRTGITRAVPFEEYDIILFSLSESDLLSLSNAGCTLDYCSYLYMSGKNF
SFLADTVLTNPMFQSMRYESCTIDDNAIRVYTTFYIPRNNYFFQIINGLVGSITEAGIPPVVE
RSTLEDMETKFLPPIGLDEDESDKYFIFGMHHLRLIFYALGIGYVVSLITFAFEKLHYKLYY
QRVKQF
>BgerIr683
MMEFHFNILLMVITQCSAAVLLPEEETYVITCLKQISQLYFTTLSKFIIILPDMEETSTHRDR
YLLNTNSPSNTTTDMAQSLLTEINGMNKLVFLIQPELLDLENITTWLEVPGPIYTNDVIIVF
FTYNNLKTAITKFMHSKAELLIILIATSEYDDFRGPMLQKVLETFAKAQLFNVLILKPGFHV
NKSKERVVKTLYLYSWYSNYTIPNCGVKDNRVKVNTWLLDEGGKFVDKPEFFPKIVASGF
KGCHFYVRKFVTSGQYVGFNVFTERMGYMFLKAAKALGIEVKFRGPDEDHVIDVYMDIMS
LKSLTTFSNSRPTYNFREITLKWHVPCPESVSRHGNFIRVFKYPTWLQLFLVGILFGLIIHW
LYKNCKDLEIAGEFSSTLLKIWGILTGVSADIGTDNYKIRIAFFIWVLFCLIISTVFQAFFTGF
LIEPGMHLPIANIKELNRTGITRAVPFEEYDLILYSLIESDLLSLSNAGCTVASCSYLYLSRKN
FSFLADTVLTKPMFQSLRHRSCTIDDNAIRVYTTFYIPRNNYFFQIINEIVARITEAGIPPVV
ERSILEDMETKFLPPIGLDEDESDKYFIFGMQHLRLIFYSLGIGYVVSLITFAFEKLHYKLY

>BgerIr684
MMEFHFSILLMVFTQCSAAVLLPEEETYVITCLKQISQLYFTTLSKFIIILPDMEETSTHRDR
YLLNTNSPSNTTTDMAQSLLTEINGMNKLVFLIQPELLDLENITTWLEVPGPLYTNDVIIV
FFTYKNLKTAVIKFMHSKAELLIILIVTSEYDDFRGPMLQKVLETFAKAQLFNVLILKPGFR
VNKSKERVIKALYLYSWYSNYTIPNCGVKDNRVKVNTWLLDEGGKFVDKPELFPEIEPSGF
KGCHIYVRKFVRSGRYVGFNVFMERMGYMFLNIAAKALGIKVQVRGPDDDHVIDVYMDIL
SLKSLTSFSNSRPTYNFREITLKWHVPCPVSVSRHGNFTRVFKYPTWLQLFLVAILFGLIIH
WLYKNCKDLEIAVDFSSTLLKIWGILTGVSADIGTDHYKIRIAFFIWVLFCLIISTVFQAFFT
GFLIDPGMHMPIANIKELNRTGITRAVPLEEYDFLLYSLTESGVLSLSNAECALDVCWYFYL
SRKNFSFLADMLLMNPMSKGIGYKTCAIDDHAIRVYTTFYIPRNNYFFQIINEIVARITEAGI
PVMVERSILRDMKTKFVSPSNVDEDESDNYFIFGMHHLKLIFYSLAIGYLVGLITFAFEKLH
YKLYYQRVKQF
>BgerIr685P
MGLHFSILLMVIALCSASVLRPKEETLVITCMQRISQQYFTTLSKFIIILPEVEKIPKZSTTTD
MTQSLLTEINGMNKLVFLIQPDILNQKNLTPWLEVPGPIYTNDVVIVFFTYNNLKIAMKQF
MYSKQELIIILILTSKYSEIGDDMIQGCLEIFAKEKLFNVLLLIPNLNISKPKHKEVKSLYIYS
WYSNYTILKCGEMDNRVKVNEWLLEENGKFVDSTPLFPSVVTSGFKGCKINIIKNSIRKRH
IGFDEIIEGLGILYLKMAARAIGMKIKVRNHDETFSGDVVHMQVLTLNSLNYFSNEIPTHTF
REIKLKWHVPCPESISRHGNFIRVFTYPTWLQLFLSAILFILLIHRLFKSSKDSKLTLEFSSTI
LKIWSILTGVSVNMGTGNYKIRIAFCIWIWFCLIISTVFQSFFTGFLIEPGMHLRIADMKDL
NRSDIIRGIPLTEYQFLAYALFENNAVQLMNYECHIFLCWYCYMTMRNFSFLADTLLMNQ
IVRXLKFPSCTIDENAITVYTTFYVRRNSYLFDKINDLIIRIRESGISAMYESFVMQEMDTKLI
SPNELIQASSDDYFIFGMHHLQLTFYFLGVGYLIGLIVFTFEKLHYKMSTKLQ
>BgerIr686P
MMYLIFSVLLIVIAQCSAADLLQGEETHVITCLKRISQQYFTTLSKFIIILPDVEETSTHRDRY
LLNKNSPSTTTDMAQSLLTEINGLSKLVFVIQPGILVVKNISTWLEVPGPIYTNDVIIVFFTY
NNLKTAVIKFMHSKPELIITLIVTSEYSEFDDDMVQSCLELFAKANLFNVLILLPDLHISRSK
RTQVKSLSVYSLYPNYPIKNCGVMDNRMKVNVWKLDEEGKFLSNTEFFPQVVASGFKGC
EIRIKRSKLEGFYLGFNSLLENIGIMYLKKATNAIGMNLKVYKWDKNLPADVYMNVMSLK
ALNYFSTEFPTHIFREIKLKWHVPCPQRISRHGNFIRVFNYLIWLQLFIVAILFGFLIHCLYK
SSKDSKLALDFPYTLFRIWGILTGVSADIGTNNYKTRIVFFIWVLFCLTISTVFQAFFTGFLI
EPGMYVPISNMKELNRTGITRAIPLQEYLLLILHSLTZNDALYIANSECSFQTCWYYYITTKN
FSFLADTVLMGPFGRGLGFPTCTIEDNSITVYAAFFIPRDSYLFNIINNLIIRIAESGFTEFFE
RNLAEDMEMKLGPMVGSVQASSEDYFIFGMRHLKLIFYLLGIGYLISLVIFAFEKFHYKLCL
ERFQEYFNRPYCKCV
>BgerIr687
MEIHFSILLLMIAQCSAAILLPEEETHVITCLKKISQQYFTTLSKFIILLPDVEETSPHRDRHL
LNRNTSSTTTDMAQSLLKEVNSMNKLVFFIQPDSLNPKNITPWLEVPGPIYTNDVVIVFFT
YNNLETSITKFMHSKPELVIILIVTSEYSEFEDDIVQRCLGVFAKANLFNVLFVLPNLHISKS
KRRHVKSLSLYSLFPNYPIKNCGVRDNRMKVNVWKLDEEGKFLDNTKFFPKVVASGFKG
CEIRIKRSKFEGSYLGFNSLLQTIGIMHLKAATNAIGMKLKVYKWDKKISADVYMDVMSQK
TLNVYSTEFPTHIFREIKLKWHVPCRQRMSRSGNFIRVFDYSIWLQLFIVAIFFGFLIHCLYK
SSKDSILTNDFPDTFLRIWSILTGVSTEVNTSIYKTRIVFFIWVLFCLIVSTVFQAFFTGFLIE
PGMYVPISNMKELNRTGITRAIPLQEYLLLILHSLTENDALYIANSECSFQTCWYYYITTKNF
SFLADTVLMGPFGRGLGFPTCTIEDNSITVYAAFFIPRVSYFFNIINNFIIQIAESGLPQFFDK

YVAEETEMEFGPMVGSVQASSEDYFIFGMSHLKLIFVSLGIGYLISCVVFAFEKFHHKLCLQ
RFNKRF
>BgerIr688
MKILNYISCILQEYTKTMELHFSILLLMIAQCSAAILLPEEETHVITYLKIISQLHLTTLSKFII
FLPDVEETSPNEERYFINQNPSSTSTDMAQSLLTEINGMNKLIFLIQPDILNLKDLTPWLEI
PGPIYTNDVVIVFFTNNNLGTAITKFMHSKPELILIFIVTSEFSYFEEDMVQKCLEVFAKAKL
FNVLFLLPNLHISKSKRTQVKSLLLYSLYPNYPIKNCGVMDNRMDVNIWSLEDEGKFVDT
KPLFPEVVASGFKGCEISATRNIIEGSYLGLNSLVEDIGIMYLKIAAKAMGMKLKVYDGNEE
FSADVYMNVLSLNSLNHFKFEFPTHIFLEIKLKWHVPCPQSISRHGNFIRVFSYQIWLQFFL
AAILFALVIHRLHKSSDDSKLSRLYFSYTLLRIWSILTGVSAEVDSSVYKIRIVFFIWVLFCLI
VSTVFQAFFTGFLIEPGMYVPISNVKELNSTGITRALPVKEYISLIYSITESDAMDIADSECSF
QMCWIRYGTRKNFSFLADTLLMGPLGRGLGFQICTIEDNAITVFTTFYIRRESYFFKLINLW
IIRIAESGISAMLERYVVEGLERKLGPKVTLDQASSDDYFIFGMRHLKLVFFSLGIGYLISSVV
FAFEKFHHKLGLRRFNKIF
>BgerIr689
MDLQFSILLVVIALCSASVLFPKEETHVITCFQRISEQYFTTLNKFIILLPDVEKTSPHEDRYF
LNTNSPTTTTDLAQSLLREINGLSKLVFVIQPDILVVKNITTWLEVPEPVYTNDVLIVIFTKI
NLKTVLSQFIHSKPDLIVILIFTPFYTELEEEVIIMCLELFAKGKLFNVLILIPHTNIDKSKKAE
VESLYIYSLYPNYPIKNCGVIDNRLNINVWFLDGEGSFLDSTEFFPEVVSSGFKGCQISVRSS
ELETFGFMTFLDNIGLMYLRLAAKAIGMKLKVYNEDEIPSTDIDLFMEVMSLNSLNFFSTE
VPTYIFREIKMKWHVPCPKPISRHGNFIRVFSNTIWLQLFLIAILFGLVIYWHYKSSNYSNS
ALDLSSTFLKIWSILTGVSTEVGTDNYKIRLGFFIWTLFCLIISTVFQAFFTGFLIEPGMYAPI
SKLEELNSTGITRAIPLREYIYILYTLSENDALDISNSVCTITGCWYGCVYWNNFSFLADNFL
TGITARAFGFQSCTLDDNAITMYATFYIPRSSYFFKIINQLIIRIAESGIPVFFESYYAKEMDS
MLGPIMKLDQGETDDYFIFGMRHLKLIFYSLGIGYLIGFVVFVFEMFHYKLCFLKFKNSYLC
IKYAKLDVSGRVCVFVRGISRVQNTQ
>BgerIr690
MMDLQFSILFMVIALCSTSVLLPEEETHIITCLKRISQQYFTTLNKFIILLPNVENTPAHGDR
YFLNQNPTSTTTDMAQSLLKEINGMNKLVFLIQSELADLTKMKSIATWLEGPGPLYTNDV
VIIFFTNTNVGTVVSKFMNSKSELIVILIFTSEYSEIEAYIVEIYLEIFAKSKFFNVLLLIPHLHT
TKSKHSEVKSLSIYSWYPYDPIENCGVFHEPVLVNVWVLENEEGKFLDETVLLPKVVLNGF
EGCEISVKRWFSNNNEYFGFNSFMENIGIAYLEIIASAMGMGLKVYNAHDKHSPDVEIAVL
NLNSLNFFSTECPSQSYREIKLKWHVPCPQSVSRHGNFIRVFKYPTWLQLFLVAIFFGLIIH
WLYKSSKNSEITPDFSGTLLRIWGILTGVSTYEGSENYKIRIFFFIWVLFCLTISTVFQAFFT
RFLIEPGMYVPISNMKELNRTGITRAIPLHEYDFIVYSLVENDVFSVVNSDCSFGLCSYYYM
NWDNFTFLADATLMIPMAKAAGFQSCAIEDNAIIIYNTFYIPRNSYVFNKINNLIIRITQCGL
AAMMERYVLQYMETKTVSEKGFLNASDQKSSDEYFIFGMQHLKVIFYLLLTGYVMSSILFV
FEKFHYYVCKH
>BgerIr691
MEFHVSILLMAMTVCSASVLLPEEETHVITCLKRISEQYFTTLSKFIILLPDMEDTSPPHRE
RHSLNTNIRSTTTDMAQSLLTEINGMSKLVFVIRPDILNVKNITTWLDTPGPLYTNDVLIVI
FTDINLKTVLSQFVHPKPDLIVILLFTSLFTEIGEDLIQMCLEVFARGKLFNVLIIIPHTYIDK
SERTKVESLYIYSWYPNYPIKNCGVMDNRVKVNVWSMEEGGKFLDSTEFFPEVVASGFKG
CRLSVSRSTAKDSSFGFIEILENIGFMYLKIAANATGLKLRAYKEDDIHSIDVYIDIMSLKSLN
YFSTEIPTYIYREIKFEWYVPCPQQISRHGNFVRVFSYTIWLQLFLVAILFGVIIHWLYKSSN
SSEIALDFSSTLLKIWSILTGVSTDMGSDNYRTRLVFFIWTLFCLIISTVFQSFFTGFLIEPGM

YAPISNMKELNRSGITRAIPLEEYFSVLFALSETDALDIANTKCSTHFCWYGYIGRNNFSFL
ADNLLIGPMGRGFKYRSCTIEDNAITMYTTFYIPRNSYFFKIINNLIIRIAESGIPVYLERYIV
KDMHSMLGPIIGIDQGETDDYFIFGMHHLKLIFYSLGTGYVIGLIAFVFEKFHYKLCYQK
>BgerIr692
MMEFHFSILLMVIAECSASVLLPEEDTHAISCLKRISQLYFTTLSNFIIILPDVEETSTHRDRY
LLNTNSPSTTTDMAQSLLTEINSMNKLVFVIQPELLDLENITTWLQVPGPLYTNDVIIVFFT
YNNFKTAITKFMHSKAELLIILIVTSEYDDFRGPVFQKCLEIFAKAQLFNVLILKPGFHVTKS
KERVVKALHLYSWYSNYTIPNCGVKDNPVKVNTWLLDEGGKFVDKPEFFPEVVASGFKG
CRIYVRKFVRSDRYVGFNVFMERMGYMFLNIAAKALGIEVRVREPHDDQVIDVYMDILSL
KSLTTFSNSRPTSSFREITLKWHVPCPESVSRHGNFIRVFKYPTWLLLFLVAILFGLIIQWL
YKNCKDLEMALDFSSTLLKIWGILTGVSVDIGTDNYKVRMAFFIWVLFCLIISTVFQAFFTG
FLIDPGMHLPISNIKELNRTGITRAVPFEEYDFLLYSLTESDVFSLSNAGCTLDVCWYFYLS
RRNFSFLADMLLMNPMLKGTGYKTCALDDHAVRVYTTFYIPRNNYFFKIINEIVTRITEAG
IPGMVERSILRDMRTDFVSPSGLDEDESDEYFIFGMHHLKLIFYSLAIGYVVGLITFAFEKLH
YKLNYQRVKQF
>BgerIr693
MYFLYLFQENTVMQMFQCYIILYVFPSFHGYLLLPREEIGIANCMEDIVDYYIPRLNSFVIL
MPNTDNISHIIKTLSTSINSNIIDMAETFIKSLNDTSKFIFSVQSRSEYINFLLKNNSHFLREP
VVVTFFVSTESTALFFRILQLNVEFIVILIFTSLYSEHKNEMVSMMLKSLSEMKVYSVFVVIP
NLRNTKSGRKDIKSLSLYSYFSTENCGRFHKAVKLNEWIFEGRGRFLKQKEELPRRMSSNF
KNCIIILEPLKIDYNASTEFLRIAELLQLNYWVFVAEKLSLRVRYSYEMRELVPDAYIGTIPL
NILDRLPNTNPTQPYQVSHLKWYVPCPERILRHGNFIRVFSYTIWIILFIVVILTGYVIHKLY
NNADSSIFLRNFSFTLLNMWGVLTGVSTDNGPTCFKLRIITTAWVWFCFIISTVFQAFFTS
FLIDPGMKITISNMEELKKTNIIRAATLVEYSWLLGILEENDILSIVHTHCSFEDCCNFYINN
NNISFIAEDSSMNLVCGLYGYQCCVIDEFTVPVYYSFLVLRNSYFVKIISDYVLRITESGFPDQ
IVRYYSGNLVSNSHKTKTSVLSKSVSTIVQVADDYFPFTLQHLKVAFYLLLIGYLASTITFV
WEKVH
>BgerIr694
MMNILFVYISFVVIHAHFSMETDLILTKEEKKVVNGLNELILHNFATDMPILLLFLENGSNE
NPVDHAFSRLPPIPDYHRFSTVFVATLNNETNWCLITRTFFPQRNYYLDNITVQLENIIYE
VAEQYIIFLMPIVEMNIEETFINTVVYLIKQLLKEERLVGHPRFIFVINIPEHIQMTFFSFLRM
YLAKILVILGYSKCLVIIPKWQSRNDQFSVHVVKILIEKDLTGICALQNLVTRQFLWLGLNE
TFSPINSFPELKTDSLKGCPMNLRYFKNYSSILSTSQKILEFETVIMEEACKFLQMHSTFTD
AKPDIIYGGVILYNQAFQNFYSFPYLSTHLNWYVPCAKSVHRHGNIFKIFQWSLWLAMFV
TLFVVSLVVVYWIHKSRRQNYIKTYGSITYCLCNLWAVMVGLSMYQIPQESRMRTFLFLWM
SYCLVMSTVFQALFTSFLIEPGFENQISKIHELSSSNLLKHTDQVKLTYWCNSFENEKKLCN
ETIVSLNGIHEYVQQLQLFNNLSVLASKLEIATITFNNMKHRFCEVEESVVPLYYSSLMRRD
SVFTNPFNEVLLKMTEFGIIENLKNNYFKNLHTFKDTLYNNTENSEINSDYVVINLYHLQF
TFCLLIFGHSFAIAVFLLEEVFYFGRHLRNVCNSFTK
>BgerIr695P
MLFLYLGLLLIMENVFTIRPHRILNKEHEMVAQGLKDLILHNFTPNLPIYLLIPKNRNDVN
TGDRVLTQFSTSLNDDYFSTAVLATLNNEINWFLLTSRFSLEPEYRYKTHQQNRRTHKME
LDYVISVQYIVFIWPTAKFQIKDAFLNAMIYLLNKALKPKTVIGQPHYIFIISIHGSARMPFLS
FLQRSAAEALAMMGYYKCTLLLPQWRHRNETFYVHIIRVSVDEHLSNGCALKNLVTEEYK
WLALNGSSTPKDSFSEIKAKSLKGCPIKLTYFKNDSSVAQAIQGLETLIFKEIIQFLQMNITF
NNKIPDIGFGSVFLFNNIGQNCYSFSHLSTRIKWYVPCAKAISLHGNIFKIFQZSVWVIMFIIL

FIFSPVAYWIRKYDRNKPFLNTHEYVSSYFCNMWAIMLGISLDQMPQEFRMRIFLLLWIYY
SLAMSTIFQALFTSFLIEPGTGKQISTIEELASSNFIKNSDEMKLSSWCRAFKNDETILCNESI
IVPNGFTEYVHRIQFYDNISILASELEIDMVIINNLGYRFCSIEESVLPMHYSSVMRRDAVFM
NPINKVLLKMTEFGIIKHLKGNYFQSLRSFKKESPNHQENSDMHADYFVLKLSHLQLSFHI
LTFGLCVSLIVFIFELFNNKCHHQIKI
>BgerIr696P
MLFLLVILILHTYGTLSSHYSHFLSFEQRNVVKDVKNIIIHNFIPNTPIYVSVPEKSNNGDLN
NRSLNHSPPCHNDNHFSNALLAALSNEVQWPLVIFZPPYNLENHTWPNSVNQIAVQFIVF
VWPTPKSDNRGTFIRSLSYLYKYIRQKVAITNPPRVIIVINHIEISGENFYSRLRTNATKLFAI
LSLYKVLIVIPQWRERGKHFYIQLLKIFVNEHITSACSLRNLTVMESIRTAHNGGALIKNVF
HEVQTESLKGCSITVMYLDHPSLLSGAIQRIEMLIFKEICNNLHVNAVSNTEKPDIIFSGVVL
EPNQLREAIFSFPHLSTHLKWYIPCSESVARHGNLFKTFHCSVWLVMALTFVIFLLLDYWL
HTYDKNNPHTRRYGSLSKCFSKMWASMLGMSVIGMPPDFRLRAFFFLWICYCLVMSTVF
QTLFTSFLIEPGNKNQILTIEELINSNLETRTDYSNSHYWCKTFQNSKRLCNESIVMINGFH
EHLHQIQSFNNISVLASELQIEIITMNNLGYRFCAIEDASVPMFYVSVVKSNIFFIKQYNEVL
LRMTEYGIIKYLKTNYFKTLHTFKNIVFRSDENTELSSKYFIMNLSHVRLIFYLLLFGHCIAS
FVFVAELYNLLQV
>BgerIr697P
MDKVILLLLLLIGIGTGNMNLLSTEEILIVNCVREIALRYYKSNSLAVVMYPSTDVQYSVPMK
KSLGTAKTVSNVNYLIGSLIKQLSIEMEWDVEVVSPFRRNQKLETKYKTHVQNIGQYILILS
TEDVKEIDEVWNQLATQIGHFITNIFLQPRHSFVIIILRGFGKVVFLKLLEVFSRIVLEHGILN
FIILANRSAPNGRRKLVLYTSVPEYTCGNSRFVKINEWIQEGNGTFLRETNLFPEKLSKNVS
ACELYIHETGDNNEDFILREIRRTIFVDIFRKLGIKIVSNRSNFRRLYHITYPFNMFFNDYNI
QQTQLHFYPYXQETLNCYVPCQKPRPKIGNIMAVFNWSSWFGMFVALFCTVLIARQLHR
NAESSYYGSIPSCLYCIWAVMTTVSVPVQPRTERLRLFFILWVWFSFVMNLVFQAFFTSFL
TEPGFEKQISSLEELKHINITIVCPKSEASFFGIDDQTVTDAMQNKIYFTNSSSMKCLIHHVL
SHSNEATIAKDLDADIIFERGTVLCSLSDFNLITFYTSLMDRRDFLYEVINYSLQTYIESGM
MTGIMNIFRSEKDSMTKRLQNSSVFHSPTSYYSLNLSHIAVTFHLLIAGYCLCLIIFVAEILM
SKYSILSEENYINST
>BgerIr698
MKLVIILLLPMVIISKHLDLLSNNEAHIVYCAREIALRYYKRNSLGIIIYPSQTKTEVSMKRSL
ANSEIDYNANHVTGSIINYLNMEMKWDIKILSLSISHFDIQEWPIINFQNIGQYILIFSTDGM
KNPSHGLHQQHLHFINRISIKPRNTFQIIIVLGSNLSDTTLEYTSTFAFYSKTLNTIILHSQNK
HRNNEHLILYTWVPDMSQCWQYGGKLEVNKWIKIGEGKFESDKDLFPTKITRNMPGCKV
RIEDTGDGKSDLTISSLTNVIYYDIIKRFGYKLAHHSKRHSDHSVMFVAMPLVDIYFSRLHI
TSDSFYPYLQANLRLYVPCNRPKPKMGNILAVFSLSLWFLIFVTILLTSLITYGLHRKTEFL
HYREIMLCFYCIWCVLTSVSVPQQPRTGRVRALFIIWVSFCFVISLIFQAFFTSFLIEPGMQK
SISTSKELEKSNLKIIQCEGELTIRNAPNSYNELFRKKMIFSKQKTIEMLINMSLSNNYSFAIF
AKDVDAKAFPENHRLCSIQDYNIISLFTAYMFRRDILFSPIKFAFEQYYESGIMAVLVNKFL
EPYRRSIMRQLQHSDEEEMYFKLTTSHLTISFYILLGGCALSFLFVVTEIIFSKF
>BgerIr699P
MNLLFQLILVASVMSTEIHYLGKNDRHIVNCIREIARRYFRSIALAVFLSPDDENIEDQLGN
NLNTNEDFLTEAVIAGLNMELSWNLDIVSLPTQNATYFLHKRWKFNYWQNVQQYILIMA
VKEKDCERQFSKKIMNFINSVNPHVTKSFTVIILTSGERIZHCSLGVEAIPMSNVVLVSNGM
VSDIENDGLYDSYLEKFLHIYSWINFQGEICMSLKKIVKIDEWVMEGSGYFARGIKLFSSKP
YRNLTGCNINNENLDKFSKGNVLKVFTAVHSDIYKKLSIGISNDLYAYMNTVFHELTPVSS

RLYFQSLGMVPFYPRFADHFKVYVPCKKPKPRMGNIMSVFSYSLWLLLVLIIFSVVLASHIL
YKRKEAKCYSSLSLCFYSIWAVMMSVSVSEQPRTTRLRMFFCLWIWFCFIINLIFQGVFTSF
LIDPGFQTHVSNLHELLQSNFKILFPPSYIQFISETVETVNPNAHIELYFEGIFELFSKHSLTN
DNCALGIQEMDIKWLNMKDTKRKRLCALEDIDIFNFHTSYMLWNNALFQHIDKALEQIF
ESGIFGYIENNVIESTYLDNKIDILQYVYTEERTESKYFEFTIGHVRISFFLLLAGQLISLLIYLI
EVCVWKLKKA
>BgerIr700
MKHSKWKNSSDQKSLSKCLTKYLLAFIGISSLSISKATGHEVLSTEEQIANCVTEIARRHFK
LIPLTVVISEEVTSTALEPRSPGNPSIKLLDQLIIAALNNQMKWDLNTVTFGPSRANHSVDT
LEMIFQYNNIKQNIIIVEVDESDEYGWLSQVEGWISLLLKSSAPLVITIVKNAPKHKEALLTE
LYSTFLRIGFLDIVTILHGCSEEKLCTNSTAYLIIYTWMPQKNDCRNNDFIPVTSKEIVEIDK
WILEGRGRFEKEADLFPEKLPQRLTGCKLYINKETSSLTELSLWKMFTDLADTLQIQTVD
DPMIANTISCTTSALEPNDISLNTFTPLPLLPPVTSNVKVYIPCNKPKPRSGNIISVFSWSV
WLGLFFATFFVILVTPRFYNKYETSHYSTVSSSTFCTWAVLMSVSVPEQPYTNRLRMFFIF
WVWICFVMSVIFQNFFTSFLVDPGFQKQISKLEELKTANVTIVYFFLELIFFEDLNGFGCIT
PNIMDCLVSQCSDDVMNSDCAFMGMEILAKLINSKQRGKGKVCALKDVTRFQLFTCSMAP
GTILFDQISRAWQRSFESGIMNYIQDYHAELNYHKEYTQLFVQASLHEESTSMYFILSLNHL
KVTFYLLITGYCVSFLLLFLELYFPKLFSRRK
>BgerIr70
MKLIALALLPTLLTLAQIEFLSEEGRHLVYCAREVVRQHFQSSTIVVVPPVLGQNEPISKRD
LGSATFPEISTLVGTVIKELSKECEWDLIVARPARKITWYVFVEDIWTQLATSKQFVVIVPR
EDFDDSTLRELCEFIFWKPYVLVLLIFQGNVRHYKVEYDVSSAGYCALNAIVVPNVPMKHE
ERALGLYTWIRDENDCGMYKETLEVDKWIMEGEGRFQKNVEEFFPEKLATQLRNCYVGT
GRAPMGESDPREAENKYNKAFILMYHAFLDIFSALGMKVESKKPTLNQVLLTSHSIKYLN
RELLKKSSFGPRLKLLYPRFATNYKIYVPCKIPNSRLGNIFAVFSWSLWLLISFVTLLVVVM
AKKLCKNTKHVGETRHFRSVSSCLYSVWAVIVSVSVPEQPRSKKLRVFFISWVWFCNALSF
VFLAHFTSFLIEPGVEKQISTFDELKKSNYRLLLPPDATSISDEDTSVKLREDMNNPYSGER
CEKNLLDCFIENYRSDSKIALYAESSEVKMLTLKYKLKDNLCAITDYNYLSHYTALMYSINT
LYKHIERALQHYHEAGLTEVAEGKILKVLYDEDRVAKVKENLNVDYGETEESQMYFEFTL
LHLWLSFLLLTGGHLLSFVFLILEKIIYKLCVKSTSDSHLEE
>BgerIr702
MVLTKLSNIIHNFLLLNYTFRMINILFIFHQYEYNYINSFILKHVYLTVYRIKKMDTKRILFL
LIFIPVVLMTPSDLMICLEEVISKQFLEGQVLVFSLPSIIPVSNLSSLNSIDSDFVNRFFENISE
KTKWPLLISTDSQINDADIPEPHHGYVIFLFPDEDLISSLEIQIESLQTFSFSYNRRGKFIIVV
LAEDVEDSQIISKEILNTVWSKDNIVNAIVILRAVDERNSHKDEELYDIYSLFPFRSEHCGES
DSIDLLDQWTASEGFLKGNELMPEKVPKDLMGCVLNVVITLVPPLVIINETYTENEGNVIF
DFDGINSVFTSFLSEAMNFSINLVKDISWNFLYGRLLDGKGVSIGANVMVPQMYSYADLST
PYVFTDVFVYAPCAKQNPQSGNVLNVFSTSVWLVSVAVLLLAAMLFFILQKNDMRLTSYN
TYSSCVLNVWAVFLSLSVSSMPLDSYFRIFFFLVVCFCFAMNTIFQSFFTSYLIDPGYEKQLE
SIEDLNEKGLPFLFDEFLIFAEELSCTKLLSFFKHAQLCEPYEQCMKDMIKNQNSSFIQSKL
KTEYFASCLGVKKMCTVWHYDVLGFVIAVSRGHPLLAKFNYFIRNCLEHGFLEIYWSHFI
WKTLLLHYRNSNEENEYSYQAFALIHLQICFLFLATGYVLSIWMWCLECVCYKYVFQVRK
VHTVTRC
>BgerIr703
MNLELNNFEENVFSVLYRVTKMAMKMLSLLFFFPVAIMKPLHIMICLEEIISKQFLLGEIVV
FSLPPTIPDEYMLIIDAAISNISSKEKWPLIISTDTQSTDAYIPEPQHGYVIFLLPDEDILTSLE

VQIQNLQTFSFSYNRRGKFIIVVLLLVEVFVDSHILSNEILNTVWNKDNIVNAVVIIPSMVKY
DATMNEDREVYDIYTLFPFRSEHCGEFNNSDLLDQWSFSEGFLTGNELMPEKLPNDLLGC
FLHIYNTPPFVFVSNYTDTQGNVVYDIKGINTIFLNFLSEAMNFSIKYDIEKGWNSVFERLS
ESRFIAVGAYLMFPHSYAFADISTPYLYGDFLLYVPCAKKNPSSGNVLKVFSTVVWLLSISVI
LLVSVLFFILQRNDSTKLCYNTLPSCFLNAWAVFLSASVPSMPTGSMFRIVFFLFVCFSFVL
NTVFQSFFTSYFIDPGHEKQIGSIEDLNEMGITFLFSDLINYVEQLSGVKFVSNFKYTKYCEP
YSECCKAMLKFQNCSIIENHFKVEYYASYVRVKGLCTAWRYEKIGMVFLMSRGHSILAKFN
YFIKKCIENGIFDHYWSIILWIHRVAHIYGENEDYYNTFGIDHLQICFSFLGIGYILSIYTFCLE
>BgerIr704P
MKIISLLFFVPVTIMTPLNIMICLEKIISKQFCPGEVIVFSLPSTIPDEYMLIIDGAIANISKKA
MWPILISTDSQSTDADIPEPQNGYVIFLVPAEDLGTSLEIQIETLQTFSFSYNRRGKFIIVVLL
EEFENSHILSNEILNTVWNKDNIVNAVVIVRAMDKYDATMNQDSEVYDIYSLFPFRSEHC
GEFNNSDLLDQWTLSKGFLEGKELMLEKLPNDLQGCFLHIDVHNIPPIVVISNYTDTQGNV
VYDIHGIGLVFLNYISEAINFSIKFEIDKDWKSLFEIFSERRCIAVGQQVMMPHLYTYADMPI
PYHYSEYLLYVPCAKKNPSSGNVLKVFSTAVWLLSVSVLLLASVLFYILQKNYSTPVCYNTI
SSCILNAWAVYLSASVPSMPTGNKFRLFFFLFISFCIALSTVFQZFFTSYLIDPGNEKQIZNIE
DINEMGVTFLSNNAVIWLEQLTGVNFVSTFKYIKHCVQTYKTCCMEMLKYQNSSILENRL
FVEYYASVLRVDKLCTVWRYERVGMVFLVSRGHPILAKFNYLIQKCLENGFFERYWPLIL
WSNRVVHVTDENEDVFYYNSFGLDHLQICFSFLGIGYILSICTLGWEYFYYKYYKKYRKQ
>BgerIr705
MNRKIIQLIVVFIPMTLMTPLNIMICLEEIISKEFRPGEVIVFSLPSTIPVEDMSMIDETISNIS
SKEMWPLLISTDSQITDADIPEPHHGYVIFLLPDDDIISSLEVQIETLQTFSFSYNRRGKFFIV
VLLEGNDDPHVLSKEILNTIWIKDNIINAIVIVPAVNIHNAPRNEEREVYDIYSLFPFRSEYC
GESNDTDLLDQWSFSEGFLKGNELMPEKLPNDLLGCVLLIDIYPVPPFVFMTKHYSDGQTI
YAFDGITLVFLNFISDAMKFSIKFLFDKGWEGVLRRMMEGRGISVGAVIMIPQTYSYADLSI
PYAFSDYLLYVPCAKRNHSSGNVLKVFSTVVWLVSVSVLLLVSILFFILQRNDSKVAGYKT
WLSCILNVWAVFLSVSVSGMPMASQLRMFFFTFLCFCFALNTVFQSFFTSYLINSGFERQIE
SIKDLNEMGVTFLYNEYLFFTEQFTGVRFVSNFKYTKYCEPYDLCCLEMLQYQNSSVLENN
FKVEYYASILGVDSLCTIWRYDKTGIVIAFSRGHPVLAKFNFFIQKCLENGFLERYWSIFLW
RNRVLNVNNKNEVSFPFSSFGLEHLQTCFSFLVIGHILSIFTLCSEYVCYKYYNK
>BgerIr706C
MYKYSKYNKGNLNSILFHNICYVVFRPMKMIRKIIPLMVVFIPMTSMTPFNIIVCLQEIISKE
FCPGDVIVFSLPSTIPVEDIPIIDEAISNISSKEMWPLLISSDSQTTDAVIPEPHHGYVIFLFPD
DDIITSLEVQIETLQTFSFSYNRRGKFIIVVLLENNDDPHSLSKEMLNTIWIKDNIINAVVIV
PAVDTHDSEMDEDTKLYDIYSLFPFRSEYCGESNDTDLLDQWTLSEGFQKGNELMPEKLP
NDLLGCVLQVDIHNEPPFVFLTENYSDVQVIYNIEGITMVFLNFISEAINFSIKFLIDKGWNS
VFGRLMEGRGIAVGAYLMIPQIYSYADVTIPFVYCDFLLYVPCAKRNPSSGNVLKVFSTAV
WLVSVSVLLLVSVLFFILQRNDMKLAGYNTLSSCVLNAWAVFLTASVPGMPKASQFRMFF
SLFLCFCFALNTVFQSFFTSYLIDTGYEKQIESIEDINEMGVTFLFDENIYLAEQFTGVKFLS
NFKYTKSCELYRTCCMEMLKYQNSSILENKFKVEYYASMLRVDNHCNIWRYDATGIVIPV
SRGHPILAKFNYFIQKCLENGFLQRYWSLFLFSNRV
>BgerIr707
MIRKIILLLTVVFIPTMLMTPLEIMICLQEIISKEFCPGKVLVFSLPSTIPLGDMSIIDEAISNIS
SKEMWPLLISSDSQSTDADIPELHHGYVIFLLPDEDIITSLEVQIEDLQTFSFSYNRRAKFIIV
VLLEDNDDPHNLSKEILNTIWIKDNIVNAVVVVRAVDIHDAQKNEETKVYDIYSLFPFRSE
YCGESNDTDLLDQWTLSKGFLNGNKLMHEKLPNDLLGCVLLIDIYHVPPFVFIKENYIDTQ

KYDFDGISVVFLNFISEAMKFSTKFLIDKGWDGVIGRMMDGRGIGVAAYVMLPQSYSYADL
SIPYAFTNYLLYVPCAKKNPSSGNVLKVFSTAVWLVSVSVLLLVSILFFILQRNDPKVAGYK
TWLSCILNVWAVFLSASVPSMPMASQFRMFFFTFLCFCFALNTIFQSFFTSYLIDTGYERQI
ESIEDLNEIGVTFLSNENIFFTEQFTGVRFISNFKYTKLCESYDECCMEMLKFQNSSILEDKF
KIEYYASIVRVDGLCTIWRYDTTGYVIPVSRGHPILGKFNYLIQKCLENGCFERYWSLFLWS
NRVLSANNDNEESFSYSSFGLEHIQICFSFLVIGYILSICTLCSEHVCYRYYNK
>BgerIr708I
MNRKIIQLIVVFIPMTLMTPLNIMICLEEIISKEVRPGEVIVFSMPSTIPVEDMSIIHETISNIS
SKEMWPLLISTDSQITDADIPEPHHGYVIFSGNVLKVFSTVVWLVSVSVLLLVSILFFILQRN
DSKVAGYKTWLSCILNVWAVFLSVSVSGMPMASQLRMFFFTFLCFCFALNTVFQSFFTSY
LINSGFERQIESIKDLNEMGVTFLYNEYLFFTEQFTGVKFVSNFKYTKHCEPYDVCCMEML
KYQNSSVLENNFKVEYYASILGVDSLCTIWRYDKTGIVIAFSRGHPVLAKFNFFIQKCLENG
FLERYWSIFLWRNRLVNVNNKNEERFPYSSFGLKHLQTCFSFLVIGHILSIFTLCSEYVCYY
NK
>BgerIr709
MNRKIILLIVVFIPMTLMTPLNIMICLEEIISKEFRPGEVIVFSLPSTIPVEDMSIIDETISNISS
KEIWPLLISTDSQITDADIPEPHHGYVIFLLPDDDIISSLEVQIETLQTFSFSYNRRGKFFIVV
LLEGNDDPHVLSKEILNTIWIKDNIINAIVIVHAVNEESEVYGIYSLFPFRSEYCGDSNDTDL
LDQWSFSEGFLKGNELMPEKLPNDLLGCVLHIDIYPVPPFVFMTKNYTDGQTIYAFDGITL
VFLNFISDAMNFSIKFLFDKGWEDVLRRMMEGRGISAGEVMMIPQSYSYADLSIPYAFSDY
LLYVPCAKRNHSSGNVLKVFSTVVWLVSVSVLLLVSILFFILQRNDSKVVGYKTWLSCILNV
WAVFLSVSVSGMPMASQLRMFFFTFLCFCFALNTVFQSFFTSYLINSGFERQIESIKDLNE
MGVTFLYNEYLFFTEQFTGVRFVSNFKYTKYCESYDECCMEMLKYQNSSILENNFKVEHY
ASILGVDNLCTIWRYDKSGVVIAFSRGHPVLAKFNFFIQKCLENGFLERYWSIFLWRNRLV
NVNNKNEERFPYSSFGLKHLQICFSFLVIGHILSIFTLCSEYVCYKYYNK
>BgerIr710
MIRKIILLIVVFIPMMLMTPLNVMICLQEIISKEFRPGEVLVFSLPSTIPVGDMWIIDEAISNI
SSKEMWSLMISTDSQSTDADIPEPHHGYVIFLLPEETIITSLEGQIENLQTFSFSYNRRAKFII
VVLLDVNDDPHTLSKEILNTIWIKDNIVNAVVIVRAVDIHYAQIDEDTKLYDIYSLFPFRSE
YCGESNDTHLLDQWTLSEGFLNGTELMPEKLPNDLLGCVLLIYVYPVPPYVFIEENYIDTE
VSYDFDGLTLVFLNFISEAMNFSTKFIIDKGWDGVIRRLMEGRGISVGAIVMIPQMYSYADL
SVPYAYTDYLLYVPCAKKNPLSGNFLKVFFTSVWLISVMVLFIVSTLFFILQKNEFNLDGYN
TFSSCILNVWAVFLSVSVPCMPTATQFRIFFFLFLCFCFALNTVFQSFFTSYLIDTGYGKQIE
SIEDLNEMGVTFLSNEFINYGEQITGLSFLSNFKYIKYCDSYRECCMEMLKYRNSSVLEEKF
KVEYYASILGVDNLCTIWRYDKTRIVIAVSRGHPVLDKFNYFIQKCLENGFLERYWSIFLW
RNKVVNVNKENEEIFSYSSFGLKHLQICFSFLVIGHILSIFTLCSEYVCYKYYNK
>BgerIr711P
MIRTLTLLLLVCIPVMITTPLHIMICLEQIISKQFRPGEVIVFSLPSTIPDEDMLIMDGAIANIS
GKEMWPLLISTDSQSTDAEIPEPQNGYVIFLVPDEDLITSLEIQIETLRTFSFSYNRRGKFIIV
VLLEEFEDSHILSNEILNTVWNKDNIINAVVILRCMVKYDATMNEDREVYDIYSLFPFRSE
DCGEFNNSDLLDQWTLSKGFLTGNELMPEKLPNDLQGCFLHIDIYHVPPFVFISNYTDTQ
GNVVTDMHGIVLVFLKLISEAMNVSMKFEIDKGWDTVYGRLMEGRGIAAGAYLMIAQLYS
YADPTIPYVYSDFLLYVPCAKKNPZSGNVLKVFSTAVWLVSVSVLFLVSTLFFILQQKDLKL
AGYDTFSSSILNVWAVFLSASVSCMPTNSHFRVFFLLFVCFCFALNTVFQSFFTSHLIDPGY
EKQIESIEDMNEMGMTLMYDEFTYLAEQFTGVKFVSLFKHSKICSVNCHVIMFKYQNSSLL
QNKFKMEYYASTIGVNKLCTARQYDKIGMVINVSRGHPILAKFNFYIRKCLENGFLERYWS

IFVWNNIILNMNNGKENSDSYCPFGLNHLQMCFLFLVIGHILSICTLCSEYVWFKYFSKWS
TNWFNGKIH
>BgerIr712P
MCSVFFTGLQKMAMKILSLLFFVPVAIMTPLNIMICLEEIISKQFRPGEVIVFSLPSTIPDED
MLIIDGAISNISSKEKWPLLISSDSQSTDADIPEPQNGYVIFLVPDEDLLTCLEIQIETLQTFS
FSYNRRGKFIIVVVLEEFEDSHILSNEILNTVWNKDNIVNAVVIIRAMVKYDATMNQDREV
YGIYSLFPFQSEHCGEFNNSDLLDQWTLSEGFLKGNDLMPEKLPKDLLGCFLHIDIYNIPPL
VFISNYSDTKASYDIDGIILVFLNFISEAMNFSIKFEIDKGWNSIYERLLEGRCIAVGGYIMVP
HSFSYADVPIPYLYGDFLLYVPCAKKNPSSGNVLKVFSTVVWLLSLSVLLLVSVLFFILQRN
DSTLFCYNTLPSCVLNAWAVFLSASVPGMPTGSMFRIFFFLFVCFCFALNTVFQSFXTSYFI
DPGNEKQIENIDDLNEMGVTFLFNDFINFVEQLTGNKFVSDFKYTKHCEPYDECCKEMLK
FENCSILENHLKVEYYASVVRVDRLCTVWRYERAAMVFLMSRGHPILASFNYLIEECFENG
FFERYWSLILWSNRVMHITDETEDVFYYSTFGLDHLQICFSFLAIGYILSICTFCLEYCYYKY
FKIYRKL
>BgerIr713P
MKMLTLLFFPVTIMTPLHIMIFLEEIISKQFLPGEILVISLPSTIPDEDMRIIDGAISNISSNEK
WPLLISTDSQNTDAEIPEPQNGYVIFLVPDEDLLASLEIQIETLQTFSFSYNRRGKFIIVVLLE
EFEESHILSNVILNTVWNKDNIVNAVVIVRAMEKYDATMNKNREVYDIYSLFPFRSEHCG
EFNNSELLDQWTLSEGFVKGNDLMPEKLPNDLLGCFLHIDIYNTLPFLFVSNYTDPQGNV
FYDIKGINTVFLNSXLSEAMNFYIKFEIDKGWNSVFERLSEGSGITVAAFLMLPHSYAFADI
PIPYLYSDFLLYVPCAKKNPSSGNVLKVFSTVVWLLSMZMLLLVSVLFFILQRNDSTKICYN
TLPSCILNAWAVFLSASVPSIPTGICSEXTKWASHFDZFDFIYFVZQLQGVKFVSNFKYTKY
CEPYSECCKMLEYQNSSILENHFKVEYYASLVRVDGLCSIWRYEKVGMVFLMSREHPILAK
FNYFIQKCIENGFFDRYWSFILWINRVTRITENDVIYYNTFGIDHLQICFSFLGIGYMLSICTF
CLEYFYYKYYEIYRK
>BgerIr714
MAKKMLSLLFFVPVAIMTPLNVMICLEEIISKEFRPGEVIVFSLPSTIPDEDMLIIDGAIANIS
SNEKWPFLISTDSQSTDAEIPEPQNGYVIFLVPDEDLLTSLEIQIETLQTYSFSYNRRGKFIIV
VLLEEIEESHILSNEILNTVWNKDNIVNAVVIVRAMNKYDPTMTQDREVYDIYSLFPFRSE
DCGEFNNSDLLDQWTLSEGFLKGNDLMPEKLPKDLLGCSLHIDIYNTPPFVFISNDSDTQA
SYSFDGIILVFLNFISEAMNFSIKFEIDKGWNSVFERLSEGRCIAVGAYIMLPHSYAFADIPIP
YLYSDFLLYVPCAKKNHSSGNVLKVFSTAVWLLSVSVLLLVSVLFFIFERNDSTKNCFNTLP
SSILNAWAVFLSTSVPNMPTESKFRLFFFLFVCFCFALNTVFQSFFTSYLIDPGNEKQIESVE
DINEMGVTFLFNDFIYFMEQLSGVKFVSNFKYTKYCEPYSKCFKEMFKFQNCSILDNHLKV
EYYASLLRVDRLCTIWRYERVAMVFLMSRGHPILASFNYLIQKFLENGFFERYWSLILWIN
RVAHITDENEDVFNYRSFGLNHIQICFSFLGIGYILSICIFCLEYCYNKYYKTYRKW
>BgerIr715
MTMKILSLLFFFPVAIMTPLHIMICLEEIISKQFLPGEIVVFSLPPIIPDEYMLIIDAAISNISSK
EKWPLIISSDSQSTDADIPEPQNGYVIFLLPDEDIITGLEVQIETLQTFSFSYNRRGKFIIVVL
LEEFEDSHILSNAILNTVWNKDNIVNAVVIVCAMDKYDAAMKRDRELYDIYSLFPFRSEHC
GEFNNSDLLDQWTLSEGFLTRNELMPEKLQADLLGCFLHIDVYNTPHVGFVSNYTDTQG
NVVYDIQGIVLVFINFLSEAMNFSIKYEIDEGWNSVFERLSEGRCITVGAYVMLPHSYAFAD
IPIPYLYSDFLLYVPCAKKNPSSGNVLKVFSTVVWLLSMSMLLLVSVLFFILQRNDSTKICY
NTLPSCILNAWAVFLSASVPSIPTGSMFRIVFFLFVCFCRALNTVFQSFFTSYFIDPGNEKQI
GSIEDLNEMGLTFLFGDILYFVELLTGVKFVSNFKYTKYCEPYSECCKEMLKYQNSSILENH

FKVEYYASYVRVNGLCSVWRYDNVGMVFLMSRGHPVLAKFNYFIQKCIENGFFDRYWSF
VLWINRVTHITENVDVIYYNTFGIDHLQICFSFLGIGYMLSICTFCLEYFYYKYYKI
>BgerIr716P
MKIISLLFFLPVTIMTPLNIMICLEEIISKQFRPGEVIVFSLPSTIPVEELLIIDGAIANISRKAM
WPVLISTDSQSNDAEIPEPHHGYVIFLVPAEDLVTSLEIQIETLHSFSFSYNRRGKFIIVVLLE
EFEDSHVLSYEILNTVWNKDKIVNAIVIVRAMDKYAATMNQDSEVXYDIYSLFPFRSEHCG
EFNNSDLLDQWTLSKGFLEGKELMLEKLPNDLQGCFLHIHIHNIPPIVVISNYTDTQGNVV
YDIHGIGLVFLYYISEAINFSIKFEIDKDWYSLFERFSERKCIAVGQQVMMPHLYTYADMPI
PYHYSEYLLYVPCAKKNPSSGNVLKVFSTAVWLLSVSVLLLASVLFYILQKNYSTPVCYNTI
SSCILNAWAVYLSASVSSMPTGNKFRLFFFLFVSFCFALNTVFQSFFTSYLIDPGNEKQIESV
DDINEMGVTFSSNDFIYFMEQLSGVKFVSIFKYIKHCVEPYKTCCMEMLKYQNSSILENKLF
VEYYASVLRVDKLCTVFRYERVGMVFLMSKGHPILAKFNYLIHNCLENGFFERYWSLILWS
NRVHVTDENEDVFDYNSFGLDHLQICFSFLGIGYILSICTFCWEYFYYKYY
>BgerIr717
MIRKIILLLIVVFIPMMLMTPLEIMICLQEIISKEFCRGEVLVFSLPSTIPVGDISIIDEAISNISS
KEMWPLLISSDSQSTDADIPELHHGYVIFLLPDEDIITSLEVQIEALQTFSFSYNRRAKFIIVV
LLEDIDDPHNLSKEILNTIWINDNIVNAVVVVRAVDIHYAPMDEVRELYDIYSLFPFRSEYC
GESNDTDLLDQWTLSEGFLNGTELMPEKLPNDLLGCVLLIDIYHVPPFVFIKENYIDPQKY
DFDGISVVFLNFISEAMNFSTKFLIDKGWDGVIGRMMDGRGIGVAAYVMLPQSYSYADLSI
PYAFTNYLLYVPCAKKNPSSGNVLKVFSTAVWLVSVSVLLLVSILFFILQRYDSKVAGYKT
WLSCILNVWAVFLSASVPSMPMASQFRMFFFTFLCFCFAVNTVFQSFFTSYLIDTGYERQI
ESIEDLNEMGVTFLSNENIFFTEQFTGVRFVSNFKYTKFCESYDECCMEMLKFQNSSILED
KFKIEYYASIVRVDGLCAIWRYDTTGYVIPVSRGHPILGKFNYLIQKCLENGCFERYWSLFL
WSNRVLSANNDNEESFSYSSFGLEHIQICFSFLVIGYILSICTLCSEHFCYRYYNK
>BgerIr718P
MVQLAKALLITYLFQAVITTTLQLSTCLEKILTQQFPPGRVIAFSLPSZSPDYEERSFSTNSE
MVSTNDLIAHLSNKMEWLLIISTDSQSADKDIPDPQHGYVILLFPDEDIVQSLEIQIEALRTF
SYSHNRRGKFIVVVLDEDIHNSQSIAKDLLVTLWASRWRTIALVQKVEKYKVXTRSVYDD
YTMFPYRSEKVCGESEEVLLIDQWRPFGGFRNGEDLFSPKVSSDLMGCTLTIEFSYNPPFH
MLYNHTDEQGNTLYKVEGINYQIFEFVSKGMNFSMAYVAWESLDLSMGRLMEGRHINGG
VYALTAKFFTLTDLZIPHAFLQHRVYVSKAGPNPVSGHFMEVFSRSLWSISVLVLLVVATIF
WLHGKEDNCLASFRTFFGCLQNTZAVILSVSVDQMPRKTNFZILFSLFVIYCFAVNIVFQSF
FTSFLIEPGYEYQTNSINDVNDRCMSVIFESYIKGLDFYLNWNVLSQFKYHRECDNILKCLE
YVCRNGKGAIFAKEPGTKFCLSLSDGLFNDQEQLHSIFTLGPIAIALHFCEGHPLLERFXRR
CLDSGLPDKYYSNLIRNNALKNTKAHVKESDSYVVFNLRHLYQSFYFHGIVCALSTAIFILE
HFIVHIGKRMKAIVYRKQHSKRKTCQFRICNK
>BgerIr719
MIQLAGALWITLMFPAVITTTLQLSTCLEQILNQQFPPGKVIVISLPSWSPDYEERSLSTNS
ETVSTNYLIAHLSKKMEWPLIISTDSQSADKDIPDPQHGYVILLFPDEDIVQSLEIQIEALRT
FSYSYKRRGKFIVVVVDEDIQNSQSIAKDLLATLWAMDNIVNSIVLIQSLENKGNLTSSLYN
VYTMFPYRSEEECGKSEEVQLIDQWRQDGGFQNGEDLFPPKVPSDFMGCTLTIEYDYNPP
FHILSNYTDSEGNIVNEVEGINHELLEFVSEGMNFTLAYAPLSTLGYAITHLIEGRQVNMGL
YTVTAQSFSYADMSIAYAFLKYEVYVPKSRPNSVSGNFLEVFSNSVWSISLLVILLGALLFW
GFQKEDEGLATYKTFSGCLLNTWAVFLSASVDEMPTKTDFRIFFLLFVTFCFAVNTVFQSF
FTSFLIEPGFEYQINSIEDLNERHLPVLCIEHQLVLDLYLNWNIYHLFSHQRICENTPRCLED
VYRYRLGATVIEMHESKFHMSVSGVLFNAGEQVHRILTVTTSPIAVAFTKGHPLVERFNLF

LQRCLESGLPDNYYTHMIRKYALRNMKTVDTEDESFVVLGLIHLQMGFLFHGIGCALSIFL
FLFEYFHVQGKRNNKTPGERVKRTKTKVRRNKRRLGWNQSQEKISDGQTQRGAIALYLP
>BgerIr720
MIQLAGALWITLMFPAVITTTLQLSTCLEQILNQQFPPGKVIVISLPSWSPDYEERSLSTNS
ETVSTNYLIAHLSNKMEWPLVISTDSQSADKDIPDPQHGYVILLFPDEDMVQSLEIQIEALR
TFSYSYNRRGKFIVVVLDEKMQDSHSIAKDLLVTLWAMDNIVNSIVLIQSVENKENLTSSL
YNVYTMFPYRSEEECGKSEEVLLIDQWRQDGGFQNGEDLFPPKVPTDFMGCTLTIEYDYN
PPFHILSNYTDSEGNVVNEVEGINHELLQFVSEGMNFTLAYAPLSTVGYAITHLMEGRQVN
MGLYTLTAQASSFADLSIAYAFLKYELYVPKSRPNSVSGNFLEVFSNSVWSISLLVILLGALL
FRGFQKEDEGLATYKTFSGCLLNTWAVFLSASVDEMPTKTNFRIFFLLFVIFCFAVNTVFQ
SFFTSFLIEPGFEYQINSIEDLNERHLPVLCVEHQLVLDLYLNWNIYHLFSDQRICENTPRC
LEDVYRYRLGATVIEMHESDFYMSLSGVFFNAGAQVHRILTAATCPIAVAFTKGHPLRERF
NLFLQRCMESGLPDNYYTHMIRKYALRNMKTVDTEDESFVVLGLIHLQMGFLFHGIGCAL
SIFLFLFEYFHVQGKRNNKTPGERVERTKTIVRRNNRRLGWNQSQEKISDGRTQRGTIALY
LP
>BgerIr721P
MIQLAGALFITLFFPAVITTTLQLSTFLEQILSQQFPPGRVIVFSLPSWAPDYEERSLSANSE
TVSTNYLIAHLSNKMEWPLIISTDSQNIEKMIPDPQHGYVILLFPDEDIVQSLEIQIEALRTF
TFSYNRRGKFIVVVLEEEVQDSQIIAKDILITLWRMNNIVNSIAVTQTVDKLTMZVYNIYTI
FPYRSREASGEXEEVLLIEQWRQDGGFQNGEDLFPQKVPYDLMGCTLTVEFGYHPPFHMI
SNHTDAQGNTVYKVEGINYLLFEFISKGMNFSMAYVVWKSMGNAVGLLMYGRNINGGVY
TVTAQVFSLVDLSIPHSFLKFQVYVSKAVANSVSGNFLSVFSSSVWLGSVLVLLLTAALFSW
YQKQDKGLASYSSLSGSFLNTWAVFLCVSVDRMPRNTNFRIFFLLFVTYCFAVNTVFQSFF
TSFLIEPGFEAQIDSIQDINDRLMPVIFESKNSELDLYLNWNAYSLFHYHKECDNFSDCLEE
VYKHRRGATFAERYATEYAMSLNGVFHHGKHLHTIFNLGTIAIAFPLSKGHPLLERFNVFL
RRCLESGLPDNYYSNLIRNNVLKNWRAIKSEDESYVVFNLNHLQMGFLFHGIGCALSICLF
VLEYLYVK
>BgerIr722P
MFQLTGALLITLLFPAVITTFELSTSLEKILTQQFPPGRVIVFSLPSWSPDYEERSLSANSEM
VSTNDLIAHLSNKMEWPLIISTDSQSDDKDIPDPQHGYVILLFPDEDIVQSLEIQIEALRTFS
FSYNRRGKFIVLLLQEDIQDPQLISKDILLTLWKMDNIVNSITLIQTVEDQDKIYKIYTMFPY
RSEEACGESEEIFLLDQWTQVRGFQNGEDLFPPKVPSDLMGCSLTVEFSYNPPFHMLSNY
TDEQGNTVYQVEGINYLIYEFISKGMNFSMAYVAWESVGLSMGRLMEGRNINGGLYALTP
LMTSIVDFLNAHFHLEHHVYVFKARPNSVSGNFLEVFSSSVWAISVLVLVLGAILFRWYQK
EDDCLASYRTLSGCLLNMWAVFLSASVDQMPRKTDFRIFFSLFVIYCFAVNTVFQSFFTSF
LIEPGYEEQIDSFRKVNDRRMPLMFESYLLFFDSTHNLKLLKHFDYHKECEDLRLCLEDVY
KYRKGAIFSEKYATEFHLSNDGIINEKEQLFXIYTLGSTAIAFPISKGHPLLERFNIFIRRCLE
SGLPNNYYSHLIRNNALKSRRVHGKVEESFIVFGLNHLQVCFGFYGIGCVLSISLFILEYLHA
KRVNRKKS
>BgerIr723P
MFQLAKALLITHLFQAVTTTTLQLSTCLEKILTQQFLPGRVVVFSLPSWSPDYEERSLSTNS
EMVSTNDLIAHLSNKMEWPLIISTDSQSIEKDIPDPQHGYVILLFPDEDIVQTLEIQIEALRT
FTFSYNRRGKFIVVVLEEDIQDPQQISKDILLTLWKMDNIVNSITLIQTVEDQDKVYKVYT
MFPYRSEEACGESEEIFLLDQWTNDRGFLNGEDLFLPKVPTDLMGCTITVEFSYNPPYHM
LSNYTDEQGNTVYQVEGVNYALLGFIAQAMNFSMTIVAWETLGNSMARLLEGRNINGGV
YTLSAQVSSVADLLIPHSSLKHQVYVFKARPNSVSGNFLEVFSSSVWXSVTVLFLGAMLFR

WYQKEDDCLASYRTFSGCLLNTWAVFLSASVDQMPRKTDFRIFFSLFVIYCFAVNTVFQSF
FTSFLIEPGFEYQIDSFEDVNVRRMPIMFETYLLELEFILNLDLYNLFDYHRQCDDLRVCLK
DVYEYRKGAIFSEKYATEFYLSLDGIISNEKVLLHDIFTLGTTDIAFPLSKGHPLLERFNVFL
RRCLESGLSDNYYSNLIRINALKNRRPFQSVDESYVHLNLIHLKMCFLCYGVGCAFSIALFV
LEYLHVQRKERNKPIALRKKRLVLQHRLKRKSNVRTQRATISLYLP
>BgerIr724P
MFYEIGPWAMDNIVHSIALIKKIEKNNNLTRSVZDVYTMFPYRSEKVCGESEEVLLIDQWR
PFGGFRNGEDLFSPKVSSDLMGCTLTIEFSYNPPFHMLYNHTDEHGNTVYKVEGINYQIFE
FVSKGMNFSMAYIAWESLDLSMGRLMEGRHINGGVYALTAKFFTLTDLSIPHAFLQHRVY
VSKAGPNPVSGHFMEVFSRSLCSISFLVLLVVATIFWLYGKEDNCLASFRTFFRYLQNTWA
VFLSVSVDQMPRKTNFRIFFSLFVIYCFAVNKLFQSFFTCFLIETGYEYQINSINHINDKRMP
ESYIKGLDFYLNWNVLSQFKYHRECDNILKCLEDVCRNGKGAIFAEKHGTKFYLSLSDGFF
NDQEQLHSIFTLGSIAIALPFSKGHPLVERXLRRCLESGLPDKYYSNLIRNNALKNTKXERD
SYVVFNLRHLYQSFYFHGIGCALSTAIFILEHFIVHIGKRMKAIVYLRQHSKRQNCQFRICNK
S
>BgerIr725
MIHLAGVLFMTLLVPAVVTTTLQLSTCLEQILTQKFPNGKVIVISLPSWCPDYEERSLRANS
ETVSTNYLIAHLSNKMEWPLIISTDSQSIEKDIPDPQHGYVIFLFPDEDIVQSLEIQIEALRTF
SYSYNRRGKFIVVVFDEKVQDSHSIAKDLLVTLWAMDNIVNSIVLIQSVENKENLTSSLYN
VYTMFTYRSEEECGKSEEVLLIDQWRQDGGFQNGEDLFPPKVPTDFMGCTLTIEYDYNPP
FHILSNYTDSEGNIVNEVEGINHELLGFVSQGMNFTLAYVLASTIGYAITHLIEGRQVNMGL
YTLTAQVFSFADLSVPYAFLKYEVYVPKSRPNSVSGNFLEVFSNSVWSISLLVILLGALVFR
GFQKEDEGLATYQTFSGCLLNTWAVFLSASVDEMPTKTNFRIFFLLFVTFCFAVNTVFQSF
FTSFLIEPGFEYQINSIEDLNERHLPVLCVEHQLVLDLYLNWNVYHLFSHQRICENTPRCLE
DVYRYRLGATVIEMHESKFHMSVSGVLFNAGEQVHRILTATTSPIAVIFTKGHPLVERFNL
FLQRCLESGLPDNYYTHMIRKYALRNRKTDDTEDKSFVVLGLNHLQMGFLFHGIGCALSIS
LFLFEYLHVQGKRGNKTRDERVKRTKTKVRRNNRRFGWNQSKEKISYGQRQRGAIAIYLP
>BgerIr726P
MIQLAGALFITLFFPAVITTTLQLSTFLEQILSQQFPPGRVIVFSLPSWAPDYEERSLSANSE
TVSTNYLIAHLSNKMEWPLIISTDSQNIEKMIPDPQHGYVILLFPDEDIVQSLEIQIEALRTF
TFSYNRRGKFIVVVLEEEVQDSQIIATDILVTLWRMDNIVNSIAVTKSVEKLTMSLYNIYTM
FPYRSREACGESEEVLLIEQWREDSGFQNGEDLFPQKVPYNLMGCTLTVEFGYNPPFHMI
SNHTDAQGNTVYKVEGINYLLFEFISKGMNFSMAYVVWKSMGNAVALLMDGRNINGGVY
TVTAQVFSLADLSIXHVFLKFQVYVSKAVANSVSGNFLAVFSSSVWLVSVLVLLLTAALFS
WYQKQDKGLASYSSYSGSFLNTWAVFLCVSVDRMPRNINFRIFFLLFVTYCFAVNTVFQSF
FTSFLIEPGFEAQIDSIQDINDRLMPVIFESKNSELDLYLNWNAYSLFHYHKECDNFSDCLE
EVYKHRRGATFAERYATEYAMSLKGVFNHGKHLHTIFNLGTIAIAFPSSKGHPLLERFNVF
LRRCLESGLPDKYYSNLIRNNVLKNWRAIKSEDESYVVFNLNHLQMGFLFHGIGCLLSICLF
VLEYLYVK
>BgerIr727
MFQLTGALLIAVLIPAVTTTTIELSTCLEQILTQQFNPGRVIVFSLPSWSPHYEERSLSANSE
MVSTNDLIARLSTKMKWPLVISTDSQSADKDIPDPQHGYVILLFPDEDIVQNLEIQIEALRT
FTFSYNRRGKFIVLLLQEDIQDPQLISKDILLTLWKMDNIVNLITLIQTVEDQDKIYKIYTMF
PYRSEEICGESEEIFLLDQWTQVRGFQNGEDLFPPKVPTDLMGCSLTVEFSYNPPFHMLSN
HTDEQGNTVYQVEGVNYALLGFIGQAMNFSIAVVPWETLGNSTARLLEGRNINGGIYTLS
AQVSSIADLLIPHSSLKHQVYVVKARPNSVSGNFLEVFSSSVWAVSVLVLVLEAILFRWYQK

GDEFLASYRTFSGCLLNTWAVFLSASIDQMPRKTNFRIFFSLFVIYCFAVNTVFQSFFTSFLI
EPGYEAQINSINDANDRRIPIMYESYLLEFQFILNLNIYDLFNFHRECDDFRVCLKDVYEYR
KGAIFSEKYATEFYLSLDGIISNEKVLLHDIFTLGTTYIAFPLSKGHPLLERFNVFLRRCLESG
LSDNYYSNLIRINALKNRRPFQSVDESYVDFNLIHLKMCFFCFGVGCAFSIALFVLEYLHVQ
RK
>BgerIr728P
MDGPTALLLVALFPKTMMAQLQLMMCVQDIISKQFSPGQVIVFSLPTTRSIDTSGIDZLMA
NISQNANLLFVVSADHQSADASIPEPQHGYVIFLPSGEDVLEGLGSQIEVLESFSFSYNRRGK
FVVVVLEEKVQDSEHISKIILSSMWRRNNIFNALVVVQSKKKSTFDLFSLFPFESEECGESD
AVALLDQWTFPTGFQHGDGLQLTKEPPNDLKGCSTTITVSIHEPFVIVTKNQTNNNGDTV
LELEGLDLVFLDFIKEGMNFSVIFDVATDDYAKEYGRLLDGRSINIGNYFLIPHQYSYADLT
TPYVITAAELFVPCSRQKPHSGSVLRVFAPSVWFALVAVLLLGAMVFLLFHKNGMESRTYS
TFCECLLNAWAVFLSTSVASPQNVHFRIFFFAFVVHCLPVXTVFQSLFTSFLIDPGYESEIET
IDDVNERGLTVMFNGFMKYTEGLVGIKFLEMFKYTRHSEDNASWLEGLLKHRNASILYDK
FRIEQYAAKEGIAELCAIWTYAKTGLVFLLSKGHPLLKRFDLLIRRCLEAGFLDRYWSLLKT
RNILANNNMNESKSDYTVFGVLHLQNYFLFLAIGCFLSTTAFVFEYMHNRH
>BgerIr729P
MIQLTLKLILVTFPIVWTNPFHLMTCVEQIISRNFPPGQVIVFSLPSEKLPNTQRSFPNTKD
EIESINYLISNLSNTTKWPLLISTDSQEIERDIPEFQHGYVIFLFPEEDILSILEIQIEALRXVD
QWTSEAGFQSGSDHFPQKVPPDLKGCVLTVVAVNDPPVNILTNYTDDQGDLKYTHEGIDS
VFLKFASRGLNFSIKFVERIDSNDWHRLITEENAISVGAFPHTPQLASFVDVSLPDVFLPFS
LYVPNAKRHSASGDFLRVFSGLAWAVILLFLVLVSILLRLYQGEDKIESYSTLSGCLLNAWA
VHLSVSVESMSRNAEFRVFFIFFVVYCFSVNTIFQSFFTSFLIEPGFERQIQSIQDLNDRDMP
LLMYEGHKIVETFFGFKLTELFKYVKLYDDGLKCLELVYNQRKGATLAEQHVAEFLSANE
EETKLFNIWTVVTAFQGFAFKKGLPIQDRFNLLVQRCHESGLPQRYYSDLIRNKSKENNSE
SQDGGESFVAFAVVHLAVCFLLLGLGYVISLCVFSAECVHKMCSLRTKS
>BgerIr730
MFQLAKALLISFAFPEIMTRTLQLSTFLEQILTEQFSPGSVIVFSLPSWSPDYEVRSLSANSE
MVSTNGLIELSNKIKWPLVISTDSQSIDKDIPDPQHGYVILLFPDEDIVQSLEIQIEALRTFSF
SYNRRGIFIVVVLEENIQDPQLKSKDILLTLWKMDNIINSIAVTPTVEKSTMSVYNVYTMFP
YISDNTCGESDEVVFIDQWIHDGGFRNGEDLFPPKVPSDLNGCTLTIEFSYNPPFHMLSNH
TDAQGNNVYKVEGINYMVFEFISKGMNFSMAYVAWESAGLSMGRLMEGRNINGGVYALT
PYLCSIADFLIPHSYLKHQVYIFKARPNSVSGDFLEVFSSSVWAVSVFVLLLGATLFRWYQM
EDDCLTSYRTFSGCLLNTWAVFLSASVDQMPRKTNFRIFFYLFVIYCFAVNTVFQSCFTSFL
IEPGYEKQIHSMREVNDRHMPLMYENLLLYYDFSYNLNILKRFDYHIECEDLFVCLEDVYK
HRKGAIFSEKFATEFYFSNDGIIFNEKEHLYNIHPVGSIAIAFVTSRGHPLLERFNVFIRRCL
ESGLPDNYYSHLIRNNALKSKRIHGKEEESFIVFGLLHLQMCFEFYGIGCILSITLFILECLHA
KRVNRNKAIVGCDQHICR
>BgerIr731P
MFHLAKALLISFVFPEIMTRTLQLSTFLEQILTEQFSPGRVIVFSLPSWSPDYEVRSLSANSE
MVSTNGLIAHLSNKIKWPLVISTDSQSIDKDIPDPQHGYVILPFPDEDIVQSLEIQIEALRTF
SFSYNRRGKFIVVVLEENIQDPQLKSKDILLTLWKMDNIINSIAVTPTVEKSTMSVYNVYT
MFPYRSDNTCGESDEVVFIDQWIHDGGFRNGEDLFPPKVPSDLNGCSLTIEFSYNPPFHML
SNHTDAQGNNVYKVEGINYIIFEFISKGMNFSMAYVAWESVGLSMDRLMEGRNINGGVYA
LTPYFCSFADFLIPHSYLRHQVYIFKARPNSVSGDFLEVFSSSVWAVSVFVLLLGAMLFRW
YQKEDDCLTSYRTFSGCLLNTWAVFLSASVDQMPRKTDFRIFFSLFVIYCFAVNTVFQSFF

TSFLIEPGYEKQIHSMREVNDRHMPLVYEXFLLYYDFSYNLNILKRFDYHIECEDLFVCLED
VYKHRKGAIFSEKFATEFYFSKDGIIFNEKEHLYNIHPVGSTAIAFVTSRGHPLLERFNVFIR
RCLEXGLPDNYYSHLIRNNALKSKRIHSKAEESFIVFGLIHLQVCFMFYGVGCILSITLFILEC
LHAKRVNRNKVKVGCGQHICR
>BgerIr732
MFPISPLIAYALFPAVMMAPFHLMTCLEQTISRHFPTGHVIVFSLPSEEISTPRRSIHNANE
KIVSISDVIAYVSNKTKLPLLISTDSQEFERDIPEPQHGYVIFLFPEEDILDSLEIQIEALRSYT
FSYNRRGRFVVVVLDGWLGKSQTLAKDILITLWKMDNIVNSVVMIRTSGDIENTSDEEHF
GLYDVYTLFPYQSEHCGESEDVVLVGQWTPANGFENSDTLFPEKVPPDLRGCIWTAEISYS
PPYGFLNNYTDVNGNVAYELEGVNSVFLQFASRGMNFSLRFVKWLSLNDWYVRMTNAN
VISIGYNALSPQLCSFADVTVPHFYISIDLYVPRALRNAVSGNFLQVFSVLVWSLLVLLLILV
AVLFRSYQRDETSLSRAPFSECLLKAWAVHLSVSVDCASKKPEFRVFFILFVLYCFAVNTIF
QSFFTSFLVEPGFEKQIETIEDLNQRDMPLFLTDGMKLFEYFTGFSLSEKFKTVESCSDTTK
CLENVCNRRSGAVLVEKHFAEYQQALAGVGDDQKLYNIWTVVSFFLVFTFRKGLPLQERF
NSFIHKCLESGLPHRYYCELIRNITIENVNRNLESTDLFIAFNLAHLKMCFLLLGLGYVVSLF
VFISERNSTLMCKRGH
>BgerIr733P
MEHVLQLLLSWAAPNNLMTFLEQTVSQNIPEGHVIVFSLPSESWPSETITANFHEKTKWP
LLVSTDSQSIEKDIPEPQDAYVVFLFPDEDLVESLEIQVEVLRTFSFSFNRRGKFFVVVLDE
DLEDPQAMSRNLLVTLWNMDNIVNAVTLVSRRRSAYQMYALFPFRPSKNCGKSEDVVRV
GEWTPENGFESGAEAFPQKAPSDLKGCTLTIEAGYNPPFSWLHDTGNYRYEALGINSVCL
KGASKGMNFSMDFLYYESAGEWFRRVTEMNVVSVLPFELTAQLCSIVDVSVPYFFESYDL
LVPRARRNSVSGNFLQVFSSPVWALSALLLILAGVLFRFYESQSSSCSRSLMEALAVCLSVS
VDRLPQXRAFFVLFVVHSFAMSTIFQSFFTSFLVEPGTEKQITTIQEVNDHQMELMVKRGI
KEYESISGVQFTNQFRSVRSCEIPRQCVAYVHKHRNSTILDERHVVDLLQSQEGVGDEAKL
FSAMTLVAPALVIPFKKGFPLQDRFNWHLRGCLESGLADKYYADLKRESVPLKTDEDSSY
VVLGVVHLKVCFALFGLGSLLSVATFLFEHCFCFKRKRL
>BgerIr734
MSIVSVMFLSFSELSEMIKGFLLLLGVLHTKTTTLNMIMCLEEILSSQFLPGEVLVFSFPASI
SSPESTSNVSIEFEIINDMLANISNQMTWPIMISTDTQIVEKDIPESHHGYVIFLFPDDDIILS
LEDQISALESFTFSFNRRRKFIVVVLDEFIKDSKLTSQNILATLWKIGNIVNAITLLKNKTEQ
EEHSIYDIYSMFPFQSEECGESDAVNLLDQWLSSGRFRYGNTLFPQKVPSDLHGCNLKILV
GVYPPFVIITTNITSGEGDPLTDLEGINLVFLTYLSRAMNFTMTFESSGHSSKKAFGNLLDN
RGINIGWYGMIPQLLSYGHFTYPYFATVVQLYVPCARQNPTSGNMFRVFSTPVWLVLMLV
LLSETVLLWCYQKNHLSGSSSDLLLKFFLNTLGVLLSVSVDISGTSINFRKYLCVFLYFSFAV
SMVFQSFFTSFLIDPGYEKAIENVQDINERRIPFISNEFLTAMQYIVQNDCITGVELDQYCED
LFECSLEMLRHQNSCTYSEKYTLEYEANVRGIRVGTKFCTICEGSNHNFVFVLSRGHPLLIN
FNHYIQRCLESGLPYQYWSILVSGLNAKNWNSITTNRDTYSALEVFHLKMSFVMLLTGYSL
SIFAHLAEHVYCRYYSKNPYLKYKQ
>BgerIr735
MVKIIQVLLLLGLFLQTVLMTSLQMMCLEEIIARNFPPGQVIVFSLPSTASESEPRHLQLTH
DVSDDITFINSFIAQVNEKTKWPLAISTEPQSVDAVIPEPQHGYVIFLTPSEDIVDDLQIQME
NLQTFGFSYNRRGKFVIVVLQQNIEDSYLLSKEILTVIWEIDNIFDSVTLIRASGHDQEGIYN
VYALFPFAPESCQQDKVLLVDQCTGEGVFEKPNTLPTRKVPSDMRGCPLRVNVWIIPPYTI
LTNNYTGNETAHITDVEGIDLVFLNFIREAMNFSIQLHSIDENYLIPLNNFLERRGVFIGKA
AITPHVSSYVDFTIPYDFTNYYLFVPCAKPNPTSGNVLTVFSSSVWFISMAVLFLSAMVFW

YFQKSSADSESYGTFSACLLNAWAVFLSASSNCLPMRTKMRTFFLLFVFHCFALNTIFQSF
FTSYFIDPGYEKQIESIEELNERRIPIVHLPDAEDSEVLWGFKLLTKIKYKMACSNYTECISY
MIKYQNASMKEDERVLKYMATIQGIYSKIHCPILSITTIGASMYLTKGDPLLEGFNYIIQKCV
ESGLTHRYWSILTSKNLLKNYKKVKQDSGSYTALAFFHLQTSFLFLLIGSSVGSCVWILEFIY
HRRHLGNFLFNRF
>BgerIr736
MFSHFLRTVKMNKIIQLSLLSLFLQTVFMTSLQMMMCLEEIITRNFPPGQVIVFSLPSTASE
SEPRHLQLTHDVSDDITFINSFIAKVNDRTKWPLVISTEPQNVDAVIPEPQHGYVIFLTPSE
DIVDDLQIQIDNLQTFGFSYNRRGKFVIVVLQQQIEDSYLLSKEILTAIWETDNILDSVTLIR
ASGHDQEGICNVYALFPFASEHCQQDKVLLVDQCTGDGVFEKSNTLPTRKVPSDMRGCPL
RVNVWIIPPYTILTSNNTGNETAHITDVEGIDLVFLNFIREAMNFSIELHSISVDYMVPLNS
FMEGGGIFIGKSSITPHFFSYFDLTVPYVFTHYLLFVPCAKPNPTSGNVLAVFSSSVWLTSM
AVLFLSAMVFWYFQKNCADSETFGTYSACLLNAWAVFLSASVNCLPLRNKMRTFFLLFVF
HCFALNTIFQSFFTSYFIDPGYEKQIESIEELNEMQIPILRFPDGEDTEHMWGFQLFMKFKK
KLVCSDYADCTSYMIKYQNASIKEDERVLEYVATKLGVQNKVLCPIMSVFTIAASIYLSKGD
PLRESFNKIIQKCVESGLANRYWSILTAKNLLSKGKQDSDSYTALAFFHLQTSFLFLLIGHS
VGVCLFILEILYNKCLKDDACSIVSSGGRLFGCANNV
>BgerIr737
MVKIIQLLLILLLSLFLQTVMMTSMQMMMCLEEIIARNFPPGQVIVFSLSSTASESEPRHLQ
LTHDVSIDITFINSLIAQVNDKTKWPLLISTGPQGVDAVIPEPQHGYVIFLTPSEDIVDDLQI
QMDNLQTFGFSYNRRGKFVIVILQQQIEDSYLLSKEILTAIWETDNILDSVTLIRASGHDQQ
GICNVYALFPFASERCQQDKVLLVDQCTGEGVFEKSNSLPTKKVPSDMHGCPLRVNVWIIP
PYTILTNNHTGNETAHITDIEGIDLVFLNFIREAMNFSIELRSFSYDYMVSMNSFLEGRGVF
IGKGAVTAQFFAYFDFTIPYVFLRSFLYVPCAKPNPTSGNVLTVFSSSVWLTSMAVLILSAM
VFWYFQKLCSDSESYGTYSGCLLNAWAVFLSGSVIALPMRTGLRIFFFLFVFYCLALNTIFQ
SFFTSYFIDPGYEKQIESIEEINEKRIPVLIYSVAQYIEDLSGFKLYAKFKRKLQCSSYVECASY
MIKYQNSSVNEDEKVLNYIASTLGVYSKIDCPILPISIVGSSMYLSKGDPLLESFNDLIQKCVE
SGLSNRYWSFLTAKNVLQTYRNEEEDNDSYTALAFFHLQTSFLFLLIGCCVGTCVLILEFLY
SKCSKEKSSLPIIISRKPNF
>BgerIr738
MDKVIKLLLLLLILFLQSQTVLMTSVQMTMCLEEIITRNFPSGEVIVFSLPSNASESEPRHLQL
THDVSDDITFINSFIAQINDKTKWPLLISTEPQSVDAVIPEPQHGYVIFLTPSEDIVDDLQIQ
MENLQTFGFSYNRRGKFVIVILQQNIEDSYLLSKEILTTIWKSDNILDSVTLIRASGHDQQGI
CNVYALFPFASERCQQDKVLLVDQCTGEGVFEKSNSLPTKKVPSDMHGCPLRVNVWIIPP
YTILTNNHTGNETAHITDIEGIDLVFLNFIREAMNFSIEVRSFSYDYMVSMNSFLEGRGVFI
GKGAVTAQFFAYFDFTIPYVFLRSFLYVPCAKPNPTSGNVLTVFSSSVWLTSMAVLILSAM
VFWYFQKLCSDSESYGTYSGCLLNAWAVFLSGSVIALPMRTGLRIFFFLFVFYCLALNTIFQ
SFFTSYFIDPGYEKQIESIEEINEKRIPVLIYSVAQYIEDLSGFKLYAKFKRKLQCSSYVECASY
MIKYQNSSVNEDEKVLNYIASTLGVYSKIDCPILPISIVGSSMYLSKGDPLLESFNDLIQKCVE
SGLSNRYWSFLTAKNVLQTYRNEEEDNDSYTALAFFHLQTSFLFLLIGCCVGTCVLILEFLY
SKCSKEKSSLPIIISRKPNF
>BgerIr739
MDKIIQLLFLLSLFLQTVFMTSLQMTMCLEEIITRNFPPGQVVVFSLPSDVLDAEPRHLELT
HDVSDDITFINSFIAQVNDKTKWPLVISTEPQSVDQVIPEPQHGYVIFLTSSQDIVDDLQIQ
MENLQTFGFSYNRRGKFVIVVLQQNIEDSYLLSKEILTVIWESDNILDSVTLIRASEDDPQGI
CNVYALFPFASEHCQQDKVLLVDQCTGDGVFEKSNTLPIRKVSSDMRGCPLRVNVWIIPPY

TILTSNYTGNETAHITDIEGIDLIFLNSIREAMNFSIELHSISVDYLVPMNSFLEGRGVFIGKA
SITPHFSSYFDLTVPHVFTHYLLFVPCAKPNPTSGNVLKVFSSSVWLTSMTVLFLSAMVFW
YFQKNSESYGTYSACLLNAWAVFLSASVNCLPLRTKMRTFFLLFVFHCFALNTIFQSFFTS
YFIDPGYEKQIESIEELNEKLIPILHFPGAEDNEHMWGFQLFMKFKNKLVCSDYADCSSYMI
KYQNTSIKEDDRVLEYMATKLGVHNKVLCPIMSVYTIAASIYLSKGDPLLESFNKIIQKCVE
SGLASRYWSLLTAKNLLSKGKEDSDSYTALASFHLQTSFLFLLIGHSASVCVWILEFLYNKC
LKEDACSIVSSGCANIV
>BgerIr740
METKTAVMLLAILMVKLEMATQLQNILTCLEEIISTQFSPGEVIVFSLPYTSIDIKHRPVISS
HAIREMNPLDALIVNIASKANIQIIISTDYQISDQDILEPHHGYVLFIPSHEDVIESIEYQINNL
KSFSFSFNRRGKFMVVFLQQEVQDSLLLSKKVLKSLWDWERINAITIITGSNQMNQQSINI
YSLFPYDSEECGESENLKILDKCSEQGKLVNQKHLLPRKVPSNLNGCKFKVYIWILPPYTIEI
RNRTENDHFDIDNFEGIDLIYLQYVSKAMNFSLQLDFAGYDIDETLNLFLKGNGIYIGGAL
MTAVFDSFSDPTKSYFFQDHRLFVPCARRIRSSGNILEVFTVSVWFLFVTVLFLKAILFWCL
QKNDKVNSYRTLSGCMLNVWAIVLSVSVDGLPVRTKFRIFFYMFVSYAFVVSMIFQSFFTS
FLIDPRHEKEIESIQDLNDKSIPIIYAYSLENEDIQMGNNLVKRIRNIKKCENYFGCLVDMLK
YQKYCVREERYILEYLGSTHGLYDGFDKIYCSIMSIYTPGVSILLVQGDPLLERFNYFIQKCV
ESGISNRYLSLSTSKRLVQNYKNQEEDHSSYLPFGLFHFRICFLLLVVGYSVSICVWFLELIV
SKNTLERLYEYSLNAIHSVKNLW
>BgerIr741
MKNSRTTKMLVDLLASGLLPKASTIAFLCLEQMIATNFSPGEVIVFSMPSNFSDTKLCPVN
SAECFSTEISAINNLLSNVNSKTEWLIVISTDYQSGDMTIPELQHGYVLILMPMEDQVESAV
TQIDYLQSFSFSYNRRAKFIVVVLQKDVNDTHLLSQQILQKLWDLDKIVNSVVLICECENIV
YNIYTLMPYRSDDCGESDEVQLIYQCTAESISAEISLQLPEKLSLDMHGCNLRVNVRVREPY
IVPIESKFDGIDDFEGIDLIYLNFIAEAMNISLKLNFAARKYDDTFIHFLNEGGIYIGGAPLTG
VMFPFVDLTIPYSSLNYMLFVPCAKRNPISGNVLKVFSVNVWLILIAILLIGAVLLWYLHKS
LSFSMCLMNTWAIFLSVSVDCMPIKTEVRVFFLVFLSYCFAISIIFQSFFTSFLIDPGYENQIN
SFEDLNEKHIPFVYYKGYEELELVSETKILSKMKYKLECVDYEKCLLEVLKYGNGSVMDEK
GILEYLSYSNGIYTDHCTILSTFNIGVCIYLQKGDPLTEKFNYFIQKCLEVGLADRYSSLTKIN
LQSKVYEESSSYSVLGLFHLQTSFSILILGYSFSTSVWLLEFIFSKSHQINM
>BgerIr742P
MTKTGRIIYAVVFLSAIFTTVKNSVKIMKCLEEIITSNFSPGKVIVLALPVHSDYTERSLLNF
EDEKASIYSLISNLNSKEMWPLVISTDYQINEKDIPEPHHGYVIFLLSYENILDSIERHVENL
RTFNFSYKRRAKFIIVILQQDVPDTLSISTDVLKKLWQLDNIANVVTLSRGREQSSYDIHSIF
PFHSEDCEESETVALLEECTSEGAFLNHKDLFPEKVPLDLHGCTFSVFIRILPPYTIYTKIGK
DNNSADINNFGGFDMVYLWFLSEAMNFSLKLEFYSIDAIAVSTIFIEREIKRGVLVGAANM
HPFYLSWADLTKSYIFEDNCLFVPCARPNPTFGNVLKVFTTSVWLSSILCLILAAVLLSCFN
RYNIESHSYTTFSGCLLICWAIFLSVSIDTLPVKTESRLFFLVFLSFSFAIEYNLSILFYFFPHR
TRIRNQIETLDDINERRVTFTYYDGYEYEESVRGTNLLTKXLNSNIKKVCPDYFEGLLDIVK
YRNSSGKLDRNSVKYVSSTYGFYSPLDRYDCTIMSFTKPGISMLLQKGNPLIETFNNFIQIAL
ESGAAERYISLTTSKNFVRNYNKHNDSSZGTSYTVLGFLHLQMSFLFLVFGYVLSLSVWLM
ELLHYIFIIKRINQKCLLRSVLVGPQHTRFSW
>BgerIr743P
MLEIYKKGKIICIVFLSCVMLTTTLSTSVQMMKCLEEIITSNFSPGKVIVLALPVSSDYTERSL
LNFEDEMASIYSLISNLNSKEMWPLVISTDYQINEKDIPEPHHGYVIFLLSYENILDSIERHV
ENLRTFNFSYKRRAKFIIVILQQDVPDTLSISTDVLKKLWQLDNIANVVTLSRGREQSSYDI

HSIFPFHSEDCEESETVALLEECTSEGAFLNHKDLFPEKVPLDLHGCTFSVFIRILPPYTIYT
KIGKDNNSADINNFGGFDMVYLWFLXEAMNFSLKLEFYSIDAIAVSTIFIEREIKRGVLVGA
ANMHPFYLSWADLTKSYIFEDNCLFVPCARPNPTFGNVLKVFTTSVWLSSILCLILAAVLL
SCFNRYNIESHSYTTFSGCLLICWAIFLSVSIDSLPVKTESRLFFLVFLSFSFAMNTIFQSFFT
SFLIEPGFEKQIETLDDINERRVTFTYYDGYEYEEHLSGTDLLSKLKYKKVSPDYFEGLQDI
VKYRNSSGKLDRNSVKYVSSTYGFYSPLEKYDCTIMSFNKQGISILLVKGNPLIETFNYFIQI
ALESGVADRYISLTTSKNLVQNYNKHNEGSTTYTALGLLHLQMCFLFLVFGYAMSISVWI
MEFVHFKFTLKKMN
>BgerIr744
MVIFSYFLLCLLITTPVNAFMCVEEIISKYFSPGQSIVISVPSVSPDFKHRFLNSSITLSDNIIAI
SAFIRSINQKEDWPILISTDSYISYQEAPLTHLQDGYVIFLLSDEEEPDFTSILISQLNSLQSFD
FSYNRNGKFIIVVCQHSNLDPLAISKNILKEMFDTDNIINAVVILPGGGHHLQHNAQLENSK
QVYDIYSLFPFDSEYCARGDNVSIIGKCLYEGQFLKDRELFPQKVPLNLNNCKLIISLYPNEP
YFILSQNYTVDNYTYYKFEGIEMVFLNYASEVMNFSYEFQILGGDNLYEIYSNGLIAIMAKK
TNIIMGMFPLFPQLCAFADPTVPYIFTSSEWFVPCAKQNPTSGNILKVFDISVWLVTVVVL
VFSSLVFLCLSKIKEKSKSYNNLSYCFLYIWAILISVSAEHIPVGTQFRMFVLLFVLFSFVLSTI
FQSFFTAFLIEPTFEKQIKTFEDHNERGIKFASGEMIESLSVMVGTYFQDKINHVVNCPDYI
PCMIEGLKYNNISFLGEKYSTLFMGSQLGLTASPGKTFCTLLTVGSGPVCMYFSKGHPILER
INFLIRLCFESGFLGMYWSQLTTDKARQYTVQEENDEYTAFNLFHLYTSFLYLLFGYAISLS
SFFFEVSFKHLRKVSAK
>BgerIr745
MKVLKTQNMLMLISFFLSLIPASGSHFIAYIETLISTYFSPGQVIVLSLPYTVSPITKRSLDISI
YQTDEYTSLNTLVNNINKKLTWPLVISTANPIFEFQTPEPQHGYIIFLKPKEEYYSLLNSLST
QIDYLQGFSSSYNRKAKFIIFVPDQVILENRKLISTELLKELMNMDKIVNAIVILQTNPEVSN
ENEQERKMEYDIYSIFPYNSTFCGKGDETILLRQWIHEGRFTNTDDLFPQKVPSNLNGCIL
NISTSIRPPYSIMNQNFTDCEGNVHYELTGIEMLFLTYLKDALNFTVNFELLEGKDLISTYIT
GINYMIDKVSDIYIAMFPILPFFIAYADPTIPYFFTSLEWYVPCAKPNPTSGNILKVFTIPVW
VVLILVLFLAAAVFYYLAKNNKDSKVIFSDCLLSTWALLLSLSAENLPVSTKFRIFLFLFISFS
LTINTVFQSFFTSFLIDPGFEKPIESIQDINRKNIPIARSLMTEYITLALSNNMLDKVTTQEV
PCSDYDQCIQIALKYQNMTILAEKYKTEYVGSALGINVGTGKKYCSLDTVASGGISMYLAKG
DPLVDVFNTLIQRCLEAGFLGKYWSEFTTLNALQNKNDQEESSDSYVVLGVTHLKMCFSF
LLLGNFLGLIVLLIECCYHKQN
>BgerIr746
MLFKRKYLLIAFLANAFFQKSSTIPIQMMCMDEIISTHFISGQVIVFSLPTIVPEIKQRYLKSS
AYSFDEDIMINNLLYEVNNKIRWPVVISSESVEEMLEVPQPQHGYIIFLVSDDDMTIIENLE
NHISNLNTGFSFNRKGKFIIFVNDRQLEDSKTLSRDILKTIWDMEKILNAIVIIPTYEKAVDS
PQYDIYSLLPYESEKCGKGENIILLDRCNYRGKLSRNDLFPQKIPQDLHGCKLIVTTMQNKP
YVMRTETYTEDGSVIEHYNGLDMVFLMYVSEAINFRVEFQETNATSTPAFYSRSLEYLLDK
RTDVSIGLFVLAPHFLSFGDPTIPYIFSSLDWYVPCARPNPKTGRVMDVFTDSVWMVVFLV
LFQAAFLFRLFGCSEMESEVYKSFSDCLMNTWAIFLASSTSHPSTNEFRCFFLVFVCYCFA
MTTIFQSFFTTFIIEPGFEKQIESIEDMNKRKIYFAQHRAAETLATLTSSTLMNGIKYNERC
EDYTTCMEISIKYQNISIISEKYMMEFMSSYLANSATTKVCCTFISVVTGGICMYFPKGDPLV
DTFNLYIQRCLENGFLVRYWSLLSRRKINYHQEEPNDSYSSYTRFSLFHLHYYFLTMLVGY
GFSFVLLLVECVCNFFWKNYEL
>BgerIr747P

MIQKQRINIIAIIAIIVFLPMKSTSMQMINCMEEIISKYFTPEQVIVFSLPATLSDMEQRSLLL
SEDTSAIDILLSRIGEKEIWPLIISRENNIDDYDIAQSQHGYVVLLMYDEKYPNIVDTLVNQI
VSLRGFAVPYNRKGIFIIAVVNWSSGCSYSISKQILNRLWKSDKILNAIVLISAANNNCSSTK
SQELDKTPLYDVYSLFPFESERCGMGEDVILLDQWTHEGRFLNGSKLLPPKVPKQLNGCSL
STSACEYFAPFVMVQKNHSARDGEXELLGIEMLVLKFIKEVMNFSVKIQSIYSKTLLETYIK
SFLRLLEGDTDIAIGGIPMWSEFNIFGDPKIWYTQAETGLYVPCARPKAVSGNILKVFTTQV
WLLSIAVLLLVATLIWHHLNMGFAPZSHTSFTSSILNVWAILLSVSTENRPPNMQYRLLLF
LIIYFSFVMNIVFQSFFISFMIEPGFEDEIKSIEDLNDRHIPIAMEPPLEKLIELLDLKYEGKYV
VQCPLYYKCTYDMFRYRNVSTFVERRFIEFIGTTIYEAPLNRGVRKNYCPIMSFGKFPLLMY
FQKGDPLLDRFNVLIRRCLEGGFLDIFAVQVKTGTTLVELKNGFQAKFPLPTDEDKELLKQ
EDSTVYEVLRPSHFKTSFIFLLIGYNLSFIVCLLEWTYNKYS
>BgerIr748
MEILQLAKLFIFVILFQYNFAQLKHPKDTEILLEEHLSDCILDIYQNHLPKSLPVIVQTTNT
WYQKFKPILEKHTIDQDGDILLKKLNQNYISQVTVRFTEDFRFGIRLNKTTSALKVGSYILL
LSGKVLSEILDSLIYMIAHIVKAFLNPLSRLVLATSWTPNSNQERNYFQTAVLNKIWQMCEI
SDAIVVVPSKDGAFDVVQWLPDDQTASCMHPLRETKLVDRWFHAKNNFEFGNELFPRK
RIEDIPHCTIKVSKSNHWPYLYSHFGRKIIGVLPSIIAESLKGKPFQVSFARHRTSISGGLYFP
FYLEPREFLDECNVLYPYFVEDTVWCVPYPGQLPRWKSVIQPFDTCLWFFVLGTFLFGYA
TFWLIHKVRHPNQDDQIKIFLNTLSTQLLGGIPDVYKGPTVALFFILWMFYSLLIGTAYQSA
LFSCILDPGYYESIENIENLKRVTNYPRMTEGKESYKDVRAISLAKFVNITYEGKKLALLSTR
ATAEFLFAIYHRVSILPITTDHLHITTLLHGIKYRCVLHAYLQKVMSRFLQFGIVHKFYMFY
VSQTLARLSADVDENPLPLLMWHLQGAFYLCVTGIITSVLVFVAEHLKYRYIRLLQTYKNM
LS
>BgerIr749
MSVLKNAFLVIILVQLQFQSNESLLRHPSGTERSVEKHLTDCLVSILKSHYPKMPPISVQTPI
SWLKFFRRIKSHNLLDLYGENVLQSLHNNFVPHRSISSEGDSSLVKKTSKVTEVHVLLLSGS
TKWVQFSLARSMIQRITEEMHNPKARVIFATGTAPDTVLEQKLTAMKLMQMAWSNARF
ADAIVLMPDLISSSSVEFNVFGWQLRDQKDECLREVTRAKLLDRWSSDEEKFLKGVELFP
PKLMKHMNQCEINAFLIQMPPMHRSSRKGVIAGPYFKVFCLLEELLNVELFIQGNTNMET
DFVFPVLYGNGDSQSDCRRTYPYLKDDVTWLVPGPVQVPQWQSLVRIFNPTMWLPVTVV
FVLGLVTFWVIENTDQNRNKLSFVQLALNTLCTYLAIGITYKHFKLLPLLFFALWLMYCLQ
IYTAYQSALIGFLLNPGYIPPIRSYSELTQSGLERLAMISAVGLDESQAQSYAYRRCPSTIFCL
RRTIDERDAAILYYRSVTQITSLTREINIVPIEDNVDTVYLSIYSLSGGCILHGYLETLLGRLF
DSGILHMWTQDTLQEAISTLRALQINVKNVVLTLSHLQGAFFLFVLGSIISCIAFLVEILLKH
NRGMQFYEQYCKDIVKAFREKTWGSIKISKCKIINCKHQKK
>BgerIr750N
VKHLFLYLTILCLKYSEGKIVLPVDEDTYFEVNLAKCILDISRIYLKEDSATIIQTSNMWYKIF
ERKPTFGETFIKILNKFNNIPYLSWGYSKRVLSTRKNLIKPGSYILLIDNLQEEWEITLMFR
RFYVDAYNANGKFIIATNKKLQYIEREKLLKMIFTTALEKGFTQIIAVLPYSTSRNLTQHSL
KGFNVYGWLPNDKNNIFSQAVHKINYFDTWCIQLNTFLHKVNLFPIKHPIKNKHCFINML
FGSYPPFIFEIFGRLMGSFSYLIQEFSKQLNCKITTKPKSHYHIIFPMEYKYDATDCEYSYPYF
WTDLTWYVPSGSKIPPWKSLIQAFTPQMWYFVIFIYAFGSLFLWLFQKTSQYSTDKCKNR
EVNILMVALLTHLGHATRDIFKGPAAILFFTIWLFYCLLINTAYQSALFGFMIDQGEYPAIK
TLNELKNSKLTMKTNLNVTNSENNKEVDGEFQICNGDCIGHVAHNRNEALLIDTYNGEIY
MNVYRDKKGYPLIVPLTEVVRTMYLAAYTANLSCILHYKLETIIHRSVSAGLIEYWNEYQK
RMVYKMYSEIEPVNFLSLWNLQGAFYTMTIGLIVACFAFLVEIFLYFSTN

>BgerIr751N
NEVVWGTENEDYTIISNCAVAASEQYFNTYSTIIIVSSTLTFSTTKYLHSSAYYPNFFHVSFT
NPDPQTCLFEKNIPYTDFRQEQNVKRYLESITTFNKHKIVLQNNDDHKMNIFLKTFIRSFQ
ATSKWQIIIPPIVDQFQVYHNSYFYAIIVHPTTHINQTVLNIQNTLFYIVETKTLNSNTSFLV
VLTEHPKPDTVRQIFILFFYYELFDVVVMEEDMLGTVNLFRGKINFRARYCNIVHKVINTD
KCKRQTNEIILTRVKKISKQLEASDFQHCHINLVYENHPPLTIKTRNPNSDRANVKKNGISI
ALLDIVFRHMKMTHRVLDNTGKICAYVMVNTGLSLLRRDHSVIYYIHEHNWFVRVSGVNS
KWSIITGAFEPLLWISVVLCVLLIGFASWLLTTSYGQENKEYCSLSSCLIITWGILLNVAVKM
PSSIRIRCIVLCFIIYSAAINTVYQTLLTTYMMNPGRSYQVNSYDELTTLGYDLALTDDENIY
RLYGDIIPQNNLKMIYEEHVLLYALEKANTAMFLDELYLIHYYNGICSGNLDIKFHKIPDKS
FSVYHHLVFTNPRIVSKVSQLLRRIEESGLTMKVANDIANPTGISKWTLRSTELKETYFAM
SVSHFKIAFIFLLFAHGISIVVFICEIACG
>BgerIr752N
IIWNALTVFFGKGNFIREDTISIILDCLIATCQTYIKSDSTILVSTNANIELPTMAFKSQYLYS
VYDIFFNNIIPNTCSVATSIPYIDLRKDQNIFKIKQKYELFHKRNNKSLVRTNDERVVTMLN
KLIPRIHMESRWKVLLPPFRNEYTILNHENVFIMIINSARELKSILLNSAKLFTSLADTNSL
NYKGLFLIIITGNSSQFTTTFILSMFFALEIFNVVIMQEVEQGKVYLFRGEKQSRKCRMLEKI
EVINTCVKTGTGVMLTSKSKLRRHIEVSEFQGCDILLIYKHYPPLTIKTFNNEKDLSEQMNI
KERGVTLILLDLVLKIMNISHGVSADGKNRAYSFARVNCEKYDFTNRFVPYYSYTSHWFVR
LAEVHYRWSSVFRVFQITTWITVIISVLLAGFISWIFSTFENEEFHLYNNLSSCFLNTWGIIL
NLGVKLPRTTKLRYFILSWIIFSLAINTVYQTFVTSFMVDPGRRHQVNSYEELLALKYDLFV
TLVEDLYTEYGENVPQNLKLVYNVIDVLLFALAKPETAIFLNEELLRYNYNNLCGGNLGIN
FHKISNSSYPSYFTISFNNIWIVQRVSQLFKRLDESGIAKKIASDVIDPIGVRRWTLSRTNLD
EEYVTMSLLHFQSAFIFLLFFNCISFFIFLCEIILHFSYFRT
>BgerIr753N
IIWNALTVFFGKGNFIREDTISIILDCVIATCQKHIKSDSTILVSTNANIELPTMAYKSQYLYS
VYDIFLNNIIPKTFSVDTSIPYIDLRKDQNIFKIKQKYELFHKRNNKSIIKTNDERVVTMLNK
LIPRIHMESRWKVLLPPFRNEYTILNHDNVFIMIINSARELKSILLNSAKLFTSLADTNSLN
YKGLFLIIITGNSSQVTTTFILSMFFTLEIYNVVVMQEVEQGKVYLFRGKKQSRKCRMLEKM
EVINTCVKTGTGVMLTSKSKLRRHLEVSEFQGCHILLVYRHYPPLTIKTFNNEKDLSEQMN
IKERGVTLILLDLVLKIMNISHGVSADGKNKAHSFARVNCEKYDFPNRFVPYYSYTSHWFV
RLAEVHFRWSSVFRVFQITTWITVIISVLLAGFISWIFSTLANEEFQVYNNLSSCFLNTWGII
LNLGVKLPRTSKLRYFILSWIIFSLAINTVYQTFVTSFMVDPGRRHQVNSYEELLALKYDLI
VTLEEDLYTEYGENVPQNLKLVQNVIDVLLFAITKPETAIFLNEELLMYNYNNLCGGNLGI
NFHKISNSSYPSYFTIFFNNIWIVQRVSQLFKRLDESGIAKKIASDVIDPIGVRRWTLSRTNL
DEEYVTMSLLHFQSAFILLLFFNCISFFIFLCEIILHFSYFRT
>BgerIr754P
IIWNALTVFFGKGNFIREDTISIILDCVIATCQKYIKSDSTILVSTNANIELPTMAYKSQFLFS
VYDIFXKNMIPKTCSVDTSIPYIDLRKDQNILKIKEKYELFHNRNNKSIIRTNDERVGTMLN
KLIPRIHMESKWKILLPPFRNEYTILNHDNVFIMIIKSARELKSILQNSAKLFSSLANTNSLN
YKGLFLIIITGNSSQVTTTFILSMFFTLEIYNVVVMQEVEQGKVYLFRGEKQSRKCRMLEKI
EVINTCVKTGTGVMLTNKSKLRRRIEVSEFQGCHILLIYKHYPPLTIKTFNNEKDLSEQMNI
KNVIDVLLFAITKPETAIFLNEELLMYNYNNLCGGNLGIRFHKISNSSYPSYFTIFFNNIWIV
QRVSQLFKRLDESGIAKKIASEVIDPIGVRRWTLIRTNLDEEYVTMSLLHFQSAFIFLLFFNC
ISFFIFLCEIILHFSYFRT
>BgerIr755

MGELTYFISLQIIWNILTMFFSKGNLVNEEKVPNILDCALATCEMYIKSDSTILFSTNSEIEF
EVDYSNEDYSKSNDTFLKNIVPKTCSVSNVIPFINFNDDHNVFWLKNKFELMNNINNKILA
RTKDEKVVTLMNKLIPRIQMTSKWKILQPPFIHEFNILKQDNFFIIIINSAVNLTAIEINTDN
ILLSFINGNSVNLKASYLIILTDNADEDIIIMIMAVFYYFELFDVIIMQEDEHGIVRLFRGEKH
STACRAVKKIEVINTCVRDGSGVMFTSNSELRKEIGVNEFQGCHILLHYGHYPPLTIKSENH
VSGQLNIKETGISLTLLDLVLQHMKISHGTLENNPLTNKLFSYARLNTEKSIIYANSILSYYP
HTYNWFVRVAEIHPRWSSISRVFQITTWITVVITVLLAGFASWIFSIYANDESLVYNKLSSC
FLNTWGIILNVGVKLPQTIKLRCVILSWIMFALAINTVYQTFVTSFMVDPGRLHQINSYDEL
INSKYNLILTVEQDIHRKYGEKVPQNLKLLNNVKNVLMFALVNPETAIFLNEEILMYNYNK
LCEGNLGINFHKIYDSSYSSYFHLYFSNIWIVQRVSELLERLDESGISMKIANDIIDPIGLQR
WAVSKVNLEEEYVTMSLLHLQSAFIFLFLLHGISVSVFLYEIIFFSYCRERLI
>BgerIr756NP
IIWNILTVFFSKGNLVNKEKVPNILDCVIATCEKYIKSDSTLLFSTNSEIEFEVNYSNEDYSTS
YHTFLKHKVPNTCSVSNVIPFIDFNVDHNVYWLKNKFELMDNKNNKMLVRTKDEKVVT
LMNKLIPRIQMTSKWKILLPPFTHEFNILXKTTFFIIIINSARHLTAIEINTNNILVSFPNGNS
LNFKASYLIILTNNADEDTITMNMNLFYYSEEFDVIVMLEDDQGIVHLFRGEKHSKKCRTV
KKIEVINTCVRDGSRVMFTSNSELRKEIRVSLFQGCHILLSYSPYPPVTIKSENQVSGQLNIK
ESGISLILLDLVLQHMNISYGILETNPITNKLFSVAKLNIEKSIMYANNILSYYPHTYYWFVR
VAEVHPRWSSISRVFQINTWITVVISVLSGGFASWIFSIYANDESLVYNRLSSCFLNTWGIIL
NVGVKLPRTTKLRCIILSWIMFALAINTVYQTFVTSFMVDPGRLHQINSFDELINSNYNLIL
TSQQDIHRKYGDNLPQNLKLLNNAKDVLLFALANPETAIFLNEEVLMYNYNKLCEGNLGI
NFHKIYDSSYSSYFYIYFTNIWILQRVSELLERLDESGISMKIANDIIDPMGLKRWALSKVNL
EEEYVTMSLLHLQSAFIFLFLLHGISVSIFLYEIIFFSYCRECLI
>BgerIr757N
IIWNILTVLFTKGDSVNEGRISNILDCVVATCEKYIKSDSTLLFSTNSEIEVIDYFHNNGFIISY
DIFMKNVVPKTCSVSNVIPFIDCTKEHNRFWLKYKFEIINNRHNKSLVRTKDEKVVTLISE
LIPALQMASKWKILLPPFIHEFNILKQDNFYIIIINSARDLTAIEINTNNMLKSLSNGNSLNL
KASFLIILTDNADEDTITLIMNVFYYLELYDVIVMQEDEHGIVHLFRGEKHSRKCRTVAKIE
VINTCVRYGSGVMFTSNSEIIKETGVNEFQGCHILLHYEHYPPLTIKSENHISGQMNIKERGI
SLTLLDLVLQHMKISHGILETNLITNNFFPIARLNVEKSIAFSNSFPSYYPHTNYWFVRVAE
VHPRWSSISRVFQISTWITVVISVLSSGFASWIFSIYENDESLVYNRLSSCFLNTWGIILNVG
VKLPRTTKLRCVILSWIMFALAINTVYQTFVTSFMVDPGRLHQINSYDELINSKYNLILTTE
QDVHRKYENNLPQNLKLVSNAKDVLLFALANPETAIFLNEEILMYNYNKLCEGNVGINFH
KIHDSSYSSYFHLYFNNIWIVQRVTELLGRLDESGISMKIVNDIIDPIGLQRWALSKVNLEEE
YVTMSLLHLQSAFIFLFLLHGISFSIFLCEIIFFYYCRERERERV
>BgerIr758N
IIWNIITVLFSKGNLVNEGRIPNILDCVIATCEKYIKSDSTLLFLTNSEIEVIDYIHNDDYLTSY
YDIFMKNVVPKTCSVSNIIPFIDFNKDHNVFWLKYKYEIINNRNNKSLVRTKDEKVVTLMS
ELIPALQMASKWKILLPPLIHVFNIVKQDNFFIIIINSARDLIAIAINTDNMLKSLSNGNSVN
FKASFLIILTDNTDEDTITLIMAVFHYSEFFDVIIMQEDEQGMVQLFRGEKHSSACRTVKKI
EVINTCVRDGSGVMFTSNSERRKEKGVNEFQGCHILLYYGHYPPLTIKSENHVSGQMNIKE
RGISLTLLNLVLQHMNISYGILENNPITNKLFSYARLNTEKSIVYANNILSYYPHTYYWFVR
VAEVHPRWSSISRIFQITTWIAVVINVLLAGFASWIFSIYANDESLVYNRLSSCFLNTWGIIL
NVGVKLPRTSKLRCVIISWIMFALAINTVYQTFVTSFMVDPGRLHQINSYDELINSKYNLIL
TIEQDIYTKYGENLPQNLKLLNNAKDALLFALAKAETAIFLNEEILMYNYNKLCEGNPGIN

FHKIHDSSYSSYFYIYFTNIWILQRVSELLERLDESGISIKIANDIIDPMGLKRWALSKVNLEE
EYVTMSLLHFQSAFIFLFLLHGISFSIFLCEIIFISYCRESLT
>BgerIr759N
IIWIILTVLFSKGNLVNEEKVPNSLDCVIATCEKYIKSDSTLLFSTNSEIELHINYLNEYYLPS
YHNIFLKNTLPKTCSVSTCVPFVDFNKNHNFSWLKRKFQIMNNKNNKSLVRTKDEKVVT
LMNKLIPALQMASKWKILLPPFIHEFNLLKEDNFIIMIINSARNRTAIETDTDNILKSFPNG
NAVNLKSSFLIILTDNADEDTITLIMNVFYYSEVFDVIVMMEDEQGIVRLFRGEKHSRRCRT
VKKIEVINTCVRDESGVIFTSNSELRKEIGFSEFQGCHILLMYGHYPPLTIKSENHLSRETNI
KETGISLILLNLVFQQMKISHGIFKDNPITNRLFSYGRLNTEKSIVYANSILSYFPHTNYWFV
RVAEVHPRWSSISRVFQITTWITVVICVLLGGFASWIFSLYTNEEYLVYNNLSYCFLNTWGI
ILNVGVKLPCTTKLRCVILSWIMFALAINTVYQTFVTSFMVDPGRLHQINSYDELINSKYNL
LLTVENNIHRKYGEKVPRNLKLFDTVKDVLLFALAKPETAIVLNEEILMFNYNKLCEGKLG
INFHKIYDSSYSTYFHIFITNIWIVQRASELLERLDESGISMKITNDIIDPMGLKSWALRRAN
LEEEYVTMSLLHLQSAFIFLFLFLGISFSIFLCEIIFFSYCREREFNLVDLKKI
>BgerIr760N
IVWNILTVLFSKGNLVNEEKFPNILDCVIATCEKYIKSDSTLIFSTNSEIELDFNYSNEHYLT
TYYNIFLKNILPKTCSVSYGITFIDFNKNHNVAWLKRKFQMVNNKNNKSIVRTKDEKVVT
LINKLIPALQMASKWKILLPPFIHEFNTLKQDNFIIMIINSARNRMAIEIDTDNILRSFPNGN
SINLKASFLIILTNNVDEYTVALIMALFYGSELFDVIVMQEDKQGMVQLFRGEKHSSACRTV
NKIEVINTCVREGSRVLFTSNSELRKEIGVNEFEGCHILLYYGHYPPLTIKSENHVSGQMNI
KDRGISLTLLDLVLQHMKISHGIIENHPLTNKLFSFAQLNTENSIVYANSILTYYPHIYYWF
VRVAEVHPRWSSISRVFQITTWITVVISVLSGGFASWIFSVYANDESLVYNRLSSCFFNTW
GIILNVGVKLPRTTKLRCVILSWIMFALSINTVYQTFVTSFMVDPGRLHQINSYDELINSKY
NLILTIDQDIYMKYGENLPQNLKFLNNAKDALLFALAKAETAIFLNEEILMYNFNKLCEGN
LRINFHNISDSSYSSYFYTFFSNIWILQRASELFVRLDESGISMKITNDIIDPMGLKRWALSK
VNLEEEYVTMSLLHLQSAFIFLFLLHGISFSIFLCEIIFLSYCRERV
>BgerIr761NP
IIWNILTVFFSKGNLVNKEKVPNILDCVIATCEKYIKSDSTLLFSTNSEIELDVNYLNEHYLP
SYYDIFLKNIVPKTCSVSNIIPFIDFNKNHNISWLKRKFQIITNKNNKSLVRTQDDKVVTLM
NKLIPALQKASKWKILLPPFIHEFNLLKEDNFIIMIINSARNLMAIEIDIKNILESFRIGNTVN
LKSSFLIILTNNADEDTINLIMNVFYCSESFDVIVMQEDEQGIVHLFRGEKHSRKZRTVKKI
EVINTCVRDGSKIMFTSNSELRKEIGVNEFQGCHILLDYGHYPPLTIISENHVSGQMNIKER
GISLILLDLVLQHMNISHGTLENNPITNRLFLYAHLNTEKSSVFANSILSYYPHTYHWFVRL
AEVHPRWSSVSRVFQITTWITVVFSVLLSGIASWIFYIFANEEYLVYNRLSTCFLNTWGIIL
NVGVKLPRTTKLRCVILSWIMFALAINTIYQTFVTSFMVDPGRLHQINSYDELINSKYNLLL
TVEHDIHRKYGEKVPQNLKLFDTVKDVLLFALAKAETAIFLNEEILMYNYNKLCEGKLGIN
FHKIYDSSYSTYFHIFITNIWIVQRVSELLGRLDESGISMKISNDIIDPMGLQRCALSKVNLEE
EYVTMSLLHLQSAFIFLFLLHGISFSIFLCEIIFLSYCRERD
>BgerIr762NP
IIWNIITVLFSKGNLVNQERIPNILDCVIATCEKYIKSDSTLLFLTNSEIEVIDYFHNDDYLISY
YDIFMKNVVPTTCSVPNIIPFIDFNKDHNVFWLKYKFEIINNRNSKSLVRTKDEKVVTLMS
ELIPALQMASKWKILLPPFIHEFNILKQDNFSIIIINSARDLIAIEINIDNMLKSLSNGNSVNF
KASFLIIVTDNADEDTITLIIAVFYCSELFDVIVMLEDEQGLVRLFRGEKHSRKCKTVKKIEV
INTCVRDESGVMFTSNSQLRKEIGFTEFQGCHILLDYGHYPPLTIKSENHLSREMNIKETGI
SLILLDLVFQQMKISYGILENKPISNKLFSYARLNTQIYLSFLNSILSYYPFTNYWFVQVAEV
HPRWSSIFZVFEISTWITVVISVLLSGFTSWIFXIFSNVESLEYNRLSSCFLNTWGIILNVGVK

LPRTTKLRCVFLSWIMFALAIHTVYQTFVTSFMVDPGRLHQVNSYDELINSKYNLIVPIEEV
IYTMYGEKLPQNLKLLNNAKDALLFALANPETAIFLNEELLMYNYNKLCEGNVGINFHKIY
DSSYSTYFNIYFSNIWIVPRVSELLNRLDESGISKKIAYDIIDPMGLQRWALRRTNLEEEYVT
MSLLHLQSVFIFMFFLHGISLTIFVCEIIIFSSWRMGVNYKSKYF
>BgerIr763NP
IIWNIITVLFSKGNFVNEGRTPNILDCVTATCEEHIKSDSTLLFLTNSEVEVIDYFHSGNYLIS
YYDIFMKNVVPTTCSVSHIIPFIDFNKDXLFCLKYKFEIINNKNNKSLVRTKDEKVVTLMSE
LIPALQMASKWKILLPPFIHEFNILKQDNFYIIIINSARDLIAIEINTDNMLKSLSNGNSVNFK
ASFLIILSDNADEDTITLIMNVFYYLELYDAIVMQEDEHGIVHLFGGEKXSRKCRTVNKIEVI
NTCVRDESGVMFTSNSQLRKEIGFTEFQGCHILLDYGHYPPLTIK---
SENHLSREMNIKETGISLILLDLVFQQMKITHGIVLKNKPISNKLFSYARLNTEKYLSFLNSI
LSYYPFTXYWFVQVAEAHPRWSSIFRVFEITTWITVVIGVLLSGFASWIFSIFANQESLEYN
RLSSCFLNTWGIIFNVGVKHPRTTKLRRIMFAPTINTVYQTFVTSFMVDPGRLHQINSYNE
LRDSRYNLILTIEQDIHIKYGENLPQNLKLVSNVKDVPLFALANPETAIFLXEEIIMYNYNKL
CEGNLGINFHKIYDSSYSTYFNIYFSNIWFVPRVSELLNRLDESGISKKIAYDVIDHMELQR
WALSRTNSEEEYVTMSLLHLQSVSIFLFFLHGISFTIFLCEIIIFSFCRMSATYKFKYF
>BgerIr764
MTLLNLLLHYLSFESTQNIMTSCIVKTSQEFFSNGQTLLFVPYTLGQCKGSTHTSQVWNYS
DHQATFIIGDDSSCIVRQYIRMVDYAKLNLKDQAEFIEEYYYAYTEGVKTYCVANRLDYVE
EEILKMFHGRNTWPMGVFPFDTTFLWASEDEIFGCILLFHFSGDYKELENDVFSLLLVLN
NHWLLKQSGYYLLALFGTVDHSRASKAILQNFGSVILRLHQVVLLIADDNSSTVSIYNFNPY
DPPSGRCGWFINEKKIGECTPSENALKLFGNESFYKKIFPKLEGCYITVSAMPEEPLIIGDTS
PEPNTSMLSNQSVAFVVARMIFEQLNVAGLTLNYPQEFSLEGYSLTMNEGFLTFNYATSV
WYHALTYYWFVPMAETYPRWSTLTRVFTAPVWVSGILALFLSALVFKLLSDQYKQSLGLF
RSLMHSLAVNVGVSVAMSGPLQHSIFLSSWLIYSMAINTVFQTFFTSYLVDPGHQHQIDSF
EELILGEYDLYFSSRDVLHSFVGNNVTPHMKIVTTEDAALLGALNTSKGAAFISEEAMVYF
LNKICDGRLGKRLHRVKDHSLQHHAFITLSDTTMMQPFMKIMGRLVASGIPTKLVNDILY
PRGAPRSLEFDDFTGEYFSFSLKHQQSTFFLYFIGVGLGLVFFLIEVFFF
>BgerIr765P
MSDRMYAKSSILNPRQNADELLACVLKTSERYFGSHGDLYFLDFAQNCLTKVSSALLNVTL
YNITATIEKECVLNQTIRIFEFGLNFNSNKKKWLRMCSEIFELNGNKVIHTCIINKVDYLKS
RILEGFHSARRWSIVIPHFLTNFGYLNKKNICCYVMLLQYNSNHTNITKEVYDQISHLSNSG
LLLQNSKFLVILDREGNPKVKTMTILRIFEIFHIYNVILLMPKSLQAHFRVDIYTSFPYKQPF
GRCGSFLKALLIDTCVPKGVNTSFTRKHSFNLNSIVNLDGCFVLPFGLSDEPLVINDHTFET
VFGISTESVAFILSKYILNHMNISEITFEKSIDSEHIVSLLLLNQQLHQFTGDPFIRYYSTSHY
WFVPMAGZYPRWSTITRVFTSDVWGVWLLTLVIAMIIFKFFSSEPLDIVLCLLNALAVILG
QSVEVRNNLQVHIFFLFWLVYGIAINIVFQAYFTSYLVDPGKPHQLDSFEELVQNKYNLIFY
SPDMLYFRRKFDVLPSVKIVAGGEAALAVALSSPRSAIFTSEESMIHFYNNICGGNIQKRLH
KMQDEKYQVHTGMMVMNPLLANNFKKYMDRLIEFGFNIKIAHDILYPKVNHRQVLITLD
LNEIYFPLSLKHLISAVYLYFLGLLMAILVFICELICYVIKMRI
>BgerIr766
MYILISEMFDNLNFDLQTCCFIIGSALILSKHIVISEETNILSNCVIETCKSWFTTQDYIVYILQ
VTEYMYDMKGENQHGHFSSQTEILRNPKYNYCSKEDLYITLYKYTPGKTEPETDKLKNVY
VTLTGVEDEILRKLFMIERWSVLVNPISYSGANNLPHMKNHIILLSYSNDIMLLIVKLEKQI
MLLISHGLNRNGRYVLILIGRDISEGVVLYVLTQMFGHGILNVLVLVQVTSELVNLYTSELY
VEYPKATVLFNTCYNRKYGVTFKQNATQYPDHSTMDLQNHCFSPGSSIVPFTLFKENKIF

DGLDFRIAQLFARYVNANVLCTAQTTERFLVNTLDTYINPQYLASKQDPFVSHYYTVRYTF
FVPVAESYPRWSTLTRVFSPSTWICGFFYLLLTGILLRIFARPEDPQRSTCECLLYSWSTVL
AIGVDLPRSTALRIFFLSSITCSLSITTVFQSFFTSYLVNPGLLHQIDSLQDLEEFNFSLIFDSV
KTLMKFVPASRLRKQSYFLYQGQAFIYALNTPKTAIFTYDDLFTCITEYMCSEAEISYHKFT
EDVMQFHVGLSSQDFPVLQDRLNTFLRRIVEAGIPDKIMADLTDPIGLRKAALKNLDLSGE
YVQMTLQHLQSPFYFWLFGLAVTLFIFVIEYLLKCIILRFPDRPCWLIKYKTRREKKQRINI
>BgerIr767
MQFSSLQGMLIVVISGGLVSLEGSEPITGCVVEIIRRIFPTNKTIYFAFVDPDEDSENFFMRN
ESACPLQVNSIYFQPAFTLFTNGPSQRLNATRDETCPTKKVKPKDLFYFPASEPYEEQTEY
QTQRTVCKVVRGDEFSEENAILKEIHSQNEWKVITAVFNSRILGLRLNNENGYLILLRYTS
DFESFMESLKIKQLELEEMAYMNSIFIIIILGKPTDMKIINSLFFFLHKEIRSFDAIVMTRLIP
EEIEIFTSVRDNCGRFKEVISLNKWLNGSFVKDFHYSKQKFESSFNGCELLIRANENPPFVI
VNENTQELDGLEVRILEYITKHLGMFPIFNLVNENERQEIWIGSNTLYNPRELFYIERYYVV
QYEWVVPLAESYPRWSSITRVFSVTVWVATIICSILITIIMYCIQNGHQKLSEARGGRLQYL
DIWAIFLGVSVEIPQWNPLRIVFLAWVIFSFAFSMIFQAFMTSYFTDAGKQHQIDTYDELIH
SNITFAFDSILMTVRHTCFTGRDGYVTTQGTLFLMLFWSQNSNVAIMTGKDSFDYTMPKI
CTAVKFLRIIGYSVESDMKLIIRQNSPYLPRINQLTIRMVEGGIPAKIMKTITNPKDIMTQSQ
STEILSDYSALSLQHVVCSFLFLGIGYLLSLISFIVELIIPRSIHLSMSLG
>BgerIr768
MSFYTSSTQITIFLVVISIRLIESSNPIAECTVEIVRRLFSPASTIVFGFIDPDEYASHLGVNWS
CPLKINSFYFESYSQFFPERPSVVIKSNKTTNEKCSKQNIPKGDLIYFPASNLHGDEYEERK
QWLLCNIIRGGQFSEDENAILKELHLKIEYKVVVGDLYSQSLGLHYDAENVYLIILRYNIGA
DSLMEYLDSKLYKLRKMAYSNSIFIIIILGKFEGLEIIYRLFIFLHDKVGSYEAVIIATGISEVIH
IFTSERDSCGGLKNVHLNEWTNGRFVKDFYYKKQQFGNFEGCRIFIRATENPPFVTFNNG
LDGIEIRLLRHITKHLGLNPIFNSIAPEGNEEIWIGAMHLFNSRLFTYLERYYTLQYQWYIPI
ARSYPRWSSITRVFSATVWGFTFLTSILISVAIWCIKNAQQTRARLQIGLDIWAIFLSISVRL
PIWTPLRMVFLTWVIFSFVFSTIFQAFMTSYFTDLGKQHQIDTYDELLQSNVTFVFESLTE
VLGHIRLTGRDGYFTFGNTMYLLLFWTQNPNVAIFAGNDLLVYTLSKICESVNAHKITDYS
VGKVLKMLVRPNSPYLSRINQLTNRLVEGGIPSKIIKTITNPKGIQTQIEVKQYLKDYSPIST
EHFVCIFVFLGLGYVLSLVGFLLEVIFFKVTHL
>BgerIr769P
MKSLSNYKLKNNFRFQIILLFAAVFEADTLENDEQIAECTIEIFKSIFSPGSTIFFSFMNPDE
NVSVEDEVNCPLKVHCPYFVPAVAPFPKDEPTRRKINTTREKTCPTTRIIDKNELZTIIPKF
LMYQETRSYYILQFXCKIVRGGQYSEVKNAILKHLHLQNTWKISVADLYDSLLYYVIDNGY
LLDFKSLKHFLISERPKIQGIGLWNPLAVVIVILGKSGDIEIINKLXLFLYYEIRLFDSIIVSKG
NEGEINVFAPIRDNCGKFKEVEHLNKCVNGTFVEDFYYSKQFQNNLSGCEIITRATNSPPF
VKIDEDTEEIDGLEGRLLNHISNHLGLIPRINKLRQNEQEDIWIGTMFTFTFIQFTYIETYYT
LQYTWFVPLAKNYPRWSSITRVFSGTVWMSTVLCSILITIIMWCIQSRHHKLSGTQDGIQIL
LGIWAIFLGVSVEIPHWTPLRIVFLAWFIFSFAFSMIFQAFLTSYFTDSGKHHQIDAYDELL
CSNVTFAFDSLFMIFEHNRRASZDGYLALGGTXDLMLFWSQNSNVAIFTATDLFVYTLPMI
CGSVEFHRLSGYAIDSHMKFLIRNTSPYLSRINQLTKRMVEGGLPAKIMKTVTNPSGMKIQ
SESTEIISDYEVLSALHVVCSFLFLGVGYLLSLIAFVIEISILNILKH
>BgerIr770
MEAILYLWCKKLHNFYYFFQITTLLGAIGTASLDNNYPIAECTAEIFKETFSPGSTIFFAFME
PYKEKPNFDYLSCPLQVKDILHPIAYTYFPQAKPGVRRFNSTIKRCYTKTVIRQKDLIYMPD
PQSHEEVIEHRIKWGLCTIFRGGQFSEQENYVLKQLHLEKRWKLTVGDLSSPLLNNGIDGG

YLIMLKYNSDVDSLINYLGTQRPRLQYIAYRNTKVVIVILGTSADIILVNKLLSFFYNVVEFS
DVIMIAKGIENEIYVYTSVRDNCGRFKEVSALKKWVNGQFVKNFNLQQHETNFKGCEIVII
ANENPPFVIFNDDTERVDGLEGRLLDHITKHLGLIPRFNSFKENETQEIYIGINHMFNYKYL
PYIERYYTLQYEWFIPRAKAYPRWSSITRVFSGTVWISTILSSILISITLCCIQNAYQKLSGTP
CTMGYFMNIWAISLGISIKLPTNTALRMIFLTWIIFSIAFSTIFQAFMTSFFTDAGRQHQINT
YDELMQSNLTFVFDSLLLLARHRYLTNRGGYVTFHGTIDSLLFWAQNPNVAVMIGKDFFI
YNLPRICTSVKFHKIRGYAVEREVKFMIRKSSPFLFRINQLTKRLLEGGIPSKIMNTIINPKG
LKVGNKGNRILDVYKPISTWHVICSFVFLGIGYLVSLIVFAVEVSMSKLFKIS
>BgerIr771N
IIAFLGIVSASKISVNEYAPPILECSIEVLRSLHTSGTTIFSGFMNTIEVFGNLSVNEEFSCDW
EINKLDANYTYSTTSSPTKAPEDQNLPVCSVDRTIDEVVYIPSRELEDLKIWPHLKWQLCH
ISRGSIFSTQENNLLRLLHLSNEWEVTVADIRHPMTLYCHNNVYLFVLRHATNIVSMMQQ
LSETSATIEHIANRNGIVVIVLLGKRADRLAVYTLSAFFHFVPRLFDIIVIASGAKGEVNVYT
PIRDKCGHLKEVLLLNTWVEGKFKTDFKYKKQEFVNLAGCTLVIRAFNSTPFVIFDEREVF
VDGLEIRLLHEIAKHLKVVLRVYPFRLLIPGEIWSGAMHLFSFRDLSYIQRYYTLQYEWLVP
LSKSYPRWSSITRVFSGNVWILTILGSILIAITVSCIQSTYQKLYLAQIRMQHFLDIWAVFLG
VSVEQPGLTPLRIVFLAWVLFSIAFSTIFQAFMTSYFTDAGRQHQIDSYEELLESNLSIAFDS
LPMLGRYNFLIKRGTFLARRGTLELLLYWLQNPNVAIMTSTDIFEYTLPKICDLNREVKFH
RVRGYFVEEDVKFLIQKNSAYLFRINQLTRRLVEGGIPSEIMKTITNPKGIKHGSTDNQMD
DYSPLSTLHVVCPFLFLGLGYLISVTVFLIEVATFKLRGGSGEK
>BgerIr772NP
IHLFCLRINLFVLFQMKNLLRNLLWAGSVWSLTHYEELMDCAIEICRRSLPASTQLFVILTY
FNFDYDEFPSYQNEMEDTNTNYSGIGKCSVNTPTSLLDYVYINITSSEVQLGEAPDWNLYP
PFRWGLFNGEENYLVKNLHSTELWPITSLTFGKVTGHQNINCVILMVHLTPSENILRQIDI
QLRFQKDVLEVASQLSARIIVIIIGMYRNKAVADVFLEVLSFRYDMFDALVILPETDTEKIN
VFSWTPFQMPSADCAEFKEIQLLDIZQGGRFMMNSTFERLTFPIRFNGCTLHALGLANPPF
VIRKDDGSGLDGIEVQLLQIISKQLGLLLNASVVVQFDGVEDLQFDYVIGLTSWLSSVSTPT
EPYYTFDYRWFVPRAQSYPRWHIILRVFSASVWIYLILTLLLVAAVFRIMFKIRFSIATIQNE
SNLDFTSEIVQYGDFGQFVMDLWANILFVSVSKLAISNPFRILLIAWIVFSIAFATVFQAYM
TTYYTNTGKQNQMNSYRELINSNNTLIYDSAEMAARNIFMSERPAYMFVHTLEMLNYAI
QVPNTALFADEDLFTYNLQMICDLNRTVQFHKCTEDSIQQNAKLTNKRTTPYLKGVNRIT
RLLVEAGVPNKIMKSLTDPTGTRYNDQNMENLSEEYVSLSMEHLSFVFSVLAIGLGISTLFL
VLEIISRRLSLLLNNILLVSTLKKYLSTQFSNM
>BgerIr773N
IVTIILVIAMGTSIGLLYDEELMDCAVEICKRSLSSSNRIFISLETHSSNLSRYGQTEEDQVPH
ALFPNKRKKRSSSVVRSCSVKKSPDSLVKAYINNTGLLLLPGSDFPVIWDLRTPTRKGYFN
DIQNYLIKKLHSTGAWKIVVSAFKKFDLYEFREFKGYIIILSYLEESFKVITEQLLGQYDAIRY
VAMTTSKIVVVVIGKLARSTIAHLILAHLAFFDAYNAVVILREMYTKHVKIYSWTPFRGPSG
NCNKLESVLLLDTWLNGRLLRNISFSDQIFPERFDGCTLEAVGVHNPPLVIADDSDTVIVG
GLEIQLLQIISSALGLKLKAWLGRVFRVDVDIWIGVGHFIVSKLPVAMEQYHTLEFRWFVP
VAKSNPRWAVVLRVFSLNVWGFLLISLLFTAGVLLITYKILLYNTERNSSKQTKLLETYESS
QYNNFVECILNLWAIVLNVTVDQKPRTAPLRILFIAWTVFSVSVVTVFQAYMTSYYTDTG
KQHQINSYEELVATNFTLAFDSTKMSDRNSILGKAPAYYFPRTLDMLNYAYQVPKTAVNS
DKDLFAYNFQKLCDLNETVKFHKFRETVEMPISLLNGRASPYLGQINKLTRRLVEAGVPN
QIMKTLTDPTGRNQDVKVKVNLSDEYVPLRMRHLFFIFTVLGLSLGSSSAVLLCELMLGKC
IFREKKILHHHNEFPNNGKSYNRYKEISRPTHPQINYTNHKYNNYWFT

>BgerIr774N
NYLLVSLLRPMLSTEGLLKVHDNNNNKQQAHCIVEIFKHFFAPSDDIYVSLSVSRSDYLGN
KKKKKTNSGNEIDPKTIHICSFNETITKDKHYTELNEDGIHRINFILDVTLDTDNPFKLNPR
DKDIEPRFLIDNLFNYTSVYYLWRFTVGTRSFRPKIGDYDGYIVFIDSIDLESDLERHVVLE
DKHQFLTMKILFVFLGVLDDTKLNYLFSLLNETSIYDVIAVDIENDNGIMNIFSWELDVCK
RFRKIVPLDVCMHGNLGTNKYSYIQHRPKHFHECAIEVIGYHNPPYSTTISGFVRDGIEYNL
TKEITSHLGLNSLHGFISSSTSKFQIKIGSEEGHKSRSQYMERIYTTHYAWFVPRSSYKVRW
PNLTGIFSQEVWWCVLLALILVSLLLEDALYTWAIFLNVSVHKKPQIDRIRIVFLTWVLFTI
SFSTVFQAYITTYFTDPGMEHQVDSIKELQMSDLSLTIERNTWQYWHIINLNKAANVFEK
ESVCSIVFALQNPKVALLTTIEKFYYNLYILCGPERRADFHKINENVMTVHRTLDMHRGSQ
FLFMVNQVTRHLVESGIVDKIVQDFLRPTGLQTTVHIQNQDYCPLTCIHLMFSFLVFCALL
CCSLAVFICELFAHYLFKMCEF
>BgerIr775N
TCLIVFLLIHKTKELLKIDVNEKFEYCIAEVFKHFFTFDWAICVTLPLKPLPSPSVRHASQIL
NSSGHSTPMLRRHIPMCSFNDTMELYIHYQELDNRRRVKEYEYFNTIYHIEEEFHMYDPP
VRKVDDKNILDIFLKHISSYLLWRFAIATFSYQIYNTCEGYVIFLNSNDLETDLSSHGYLKYN
YHTKKVKLLIVFLGILDGFRLNTLFRLINRTPIYDVIILDSNKRNSTMNMFSWELDACRSFK
KVIHLNVCSNGKFIRNMSMRTRNFPKRFDICEIELVGAHSPPYSITNNESYVKDGIECKLVQ
EISSHLGFKLKYGFLNYDPTIFQIQIGTEKAYQVQFLRYMEKYYTTYYTWFVPRARTYPRW
SNLARVFTNDVWGSVLVTLIIVSVFLESGLNTWAVFLSVSVHQKPVTTRFRIVFMAWLLFC
ISFSTVFQAYMTTYFTDPGKEHQIDSVNDLLMSNLTLSIDDISWQVLQILNMNKSTKLFIPG
RMCSIIYALQTRYVALLSTNEEFLYNIPLLCNNSCAKDFHKFSKSETNVHRVLIMNEHSPFL
FRVNEITRLLIESGIVNKIVSDYLNTTDLDHEIHVRNVLLDEYHPLAVLHVVFAFLFLSIFLL
VSLVVFIGEFIVRFLPQPF
>BgerIr776N
KSEGLLLKYDEDIRITKCISEICKKYFPSNSDIFVSLPTKFNSGFGHFMSHFFKNNRTFQIPIN
TEKKKHPICKLDKQIIPTDYDVIDPFQEDPLEQNPTRSFYEQILFHLDGRDKEIQAKHFVD
HLLKSMTSQESWKFVIYDPTMAKNLSQNEYYNGFILMINGDAVEQNIDRLNYLKEHPSIL
TARIVVVCLGTLGKSKVSSIFTILKETLVYDVIVVETRDRKGNFVMYFWELDQCRRIKNVT
HFNICNVGEFNLNISLIDQQRPKRFDGCKLQLVGIDNPPYSIMKSNKKVSNGIEYKVAKEIS
SYLGFNVTYGSFSSITKYQIITGIEDEFVVHPLTYMPRYYTVEHRWYVPFAGTQPRWSNIT
RVFTTDIWLCIAALLILVSFLLKSGLNTWGVLLNISVKQNNDGIRFRAVFFAWVTFSIIFINV
FQAYMTSYFTDPGRHQIDSIEKLKASNLTLAIEKVDWEISYVLSLNKSAKAFSPGTCPIIFAL
QNPNTALLQADDEISYNIPRITHMNQTVNFYKFNNIIMTVHKTLYMDKRSHYVSRINEVT
QHLVTAGILDRIIGKFLYPKGRKPGRYDRKNLLPEYYTLNLGHLFCGFLFLDIGVSISCVIFV
AECVLSKCMRTDATYSKCDLIKN
>BgerIr777N
WFYIILVLTWKSESIFKSDTDNQITKCIVELCKMYFPSDSEIFISLSTVLDESFVRYVQSIKK
MKNDSVLQFEKLPRQSPICLLNKKVDDYYDLNPSSLPYLFFQPIVYFSQKCPFRLTGPCKET
QAKHFVNKFFQYIIPQQFWKIIVYASTAPYYTGCIQAQYDYNGFILLINEDDVEVDLLRVTY
YLEQPSILSAKVIVIYLGTLDASRLSSIFSILNETSVYDVIVVDTGNQTGNVNMYSWELDQCK
NMKEIVNLDVCRFGGFNTNISLIAQQRPKRFDGCKLEVVGTHNPPYSIIENDTVVDGMEYK
LVKEISSYLGFSIINESFSYITKYQIQTGVEDGYGMHPLTYMPRYYTVEYTWYVPLAESQPR
WSSISRVFTVDIWLCVAVLLIAVSFLLDSVLQTWGVLLNIAVKQSHNTLRFRAVFFAWVSF
SIIFSNIFQAYMTSYYTGPGRSDQIDSIEKLKESNLTLAIENIDWELSHVSLLNKSAKVFSPG
TICPMIFALQNPNIALLQAGDQISYNVPRIKDLNHTINLYKFNNIIMKVHKTLYMDKLSPYV

SQVNEVTKRLVTTGTVQKVIQSFLHSNGVKSESYARKNLQIEYHRLKVSNLFCGFIFFGVGV
CVSLIVFITEYIVIYV
>BgerIr778N
SLYIILVLTQRTEEILFKSDEDVQIARCVAELCKRYFPSESEIFISLSNVLDKGFVWHVKRLK
EENDSIIHLPNPPRMIPTCSLNNKIINIYNLDPARMNFLMQREIQNLYEQCSLKLVGPCKET
EAKNFVDHLLQYISSQDFWKFIVYSPGMPVVWSCRNEHYDYDGFIALINEDDVEVNMFRF
RHLLDQPAILTAKLVVVYLGTLDTSKVKLIFRILNETSVYDVIVVDTGNKTGNVNIYSWEL
DQCKNFQRVVLLDVCRFGEFNRNISIRDQHRPKRFEGCKLELVGTHNPPYSIIENVTVVDG
IEYNLVKEISSYLGLCVTYGPLSYITKYQIQTGVEDEFKILPITYMPRYYTVEYTWYVPLARS
QPRWSSITRVFTVDIWFCVAVLLIAVSFLLDSVLKTWGVLLNVAVKQPDCTLRFRAVFFA
WLSFSIIFGNVFLAYMTSYYTNPGKSDQIDSIEKLQESNLTLAIENLGWELSHVSLLNKSAK
VFPPGTTCPIIFALQNPNIALLLAYDQLSYSIPRIKGLNRTVKFYRFNDIIMKVHKTLYMDKL
SPYVSRVNEVTKSLVTTGIVERIIERFLYPNGLKPGSYERKTSQYYRLKMNHLFCGFLFFGF
GVCVSIVIFIAECVIFIN
>BgerIr779
MEYICSSINLQLVQCAAEVSPFFFPEGSSISVILHVPPWLQREKRESNVSETGINGSNQKRYE
NCTNYERRIYSYKGEVEKRSKYSTPKYNHQRKFISPMRKFTFIHMLDTFLRLINANYSWKL
STHVFKSRWGSLHEKFDGFILILNGEELDYEISSCELYIKSTLWFPKPNVILILTGINPNIELV
FNFFEKYSIYNSIIISEDESTNVVVTAWNADSCGSFSEMYFMASCTSNVFSQTKRNYTTKYP
SNFNGCWLAALGVNNPPFSVNSDDMNLTLIDGVDFKVIEIIAHYIGARLNSTTNYIPESYES
IGSNINLQSEKKPITYLQRYYTQTFAWFVPCAKTNIRWSSLTCVFSAEVWFCFLASFIFISLT
MQDSFNAWAIFLNVAVYRRPSSMHMRLLFMSWVLFSYSFVMVFQTYMTSCFTDPGKQH
QIDSLEEVLTSDLNLVVDMSQDDSWHIMMGNRKHFFMFAYDTVNMLRFTSEKPNTAVL
TSEEVFLYNYPRMCERTETSEFYMVRNGAMSVHMTFVIDNSSRYIPFINRIIIRLVESGVVN
KLVENYVDPTGLKKGINDAEHFTSYSPLSKFQMFSTFLYLFIGLLLSFIVFFGELFVVRLHAL
AECIRILYIRDCSSMTGRF
>BgerIr780
MVFCMIMVYMTINIQMTSGCSSITLQLVQCAVEVSKVFFPEGSSISVMLHVPSRSQREKRE
YNVNVKIDRPNEKRYYGNCSDNKTRYYSFKLEYFGEAEEHSPYITPKNQQWKLIPPIRKFS
SSYILDYFLRLVNFNQSWKFYTHVIINQWDKLHEKLDGFIIFLNSDQLHYEITTYSLYIKSNF
WFPKPKIMVIIIGINPDLLPVFNFLDKYSLYGSIIISENESRDVIVSSWIADNCGSFSQSYFFES
CTSNIFSITKQNYTVHLPPNFNTCSLAAFGVHNPPFSVNSDDMNLTLLDGVDFKIIEIIAHY
MGLRLNSTTHYVPESYDYIGSNINLESEKKPITYLQRYYTQTFAWFVPCAKTNIRWSSLTR
VFSADVWFCFLASFIFISLAMQDSFNAWAIFLNVAVYRRPSSMHMRLLFMSWVLFSYSFV
MVFQTYMTSCFTDPGRQHQIDSLEEVLTSDLNLVVDMSQDDSWHIMMGNRKHFFMFAY
DTVNMLRFTSEKPNTAVLTSEEVFLYNYPRMCERNGTSEFYMVRNGAMSVHMTFVIDKS
SRYIPFINRIIIRLVESGVVNKIVENYVDPTGLKKGISIAKHVPKSYGPLSKFQMFSTFLYLWI
GFLLSFVVFFGELLVGGLHGLSRFVVISIQTH
>BgerIr781NP
IHASLFLPSTETLTFKNLENISECSSEVAKHFFPRGSSLFLSLPTDFQMLRIKREYLASNTQT
TSYEENSNYNCSDIQVNGKNGLMKTYKSYDGQGMYYVNKQQWSIDAPIRNFTFGPIIDIFL
KNIHSYQSYKMLSKQITVNDGYIQEKFDGFILMLHANNLESEIARHIKIRSNFMYPKPRIMV
ILLGETLDLNIVFDFLVQDALHQSVVLLTEESGIVNIFSWITDKCGRMQNITLLSFCSGRVN
EILNISEANTSSSHYGCNLTLFGFSNPPFSKSLKNFTLTDGVDFQLIQNIAHHLGLNIINSGY
TDVNEFVGSDMVVNQSFRKETMTYMQRYYTQKFAWFVPRASSQVHWSSLTRVFSPTVW
LCLFVCLLVVSISVGNTLHTWAVLLNVSVNRQPRTLKLRILFFTWLLFSISLSTVFQTYMTS

YFTDPGRQHQIDSIEELLESDLDFLVDGFKPNVWPTMLRKGYXFTNGNEGMLQYASQRR
NIAILVSEEEFLYNYPRICGFKRKQQFHMIRDGATGYNRYLFMAFSSPYLPRVNEITKRLVE
SGVVEKIVDNFINPLGFKHQINTQTKFLIESGYTPLTAFQVFSSLLYLTIGLFLSCLIFLGELL
LCRFPRFNAM
>BgerIr782N
VMILASHVIPFSTILPSKINEKLTECSLEVAKLFFRSGSSLFLSLPSHLQKFRNQSEYGTKYV
KTEENINKLHSTIEKSQLISSMKGPMYNQKTYIIEESNWKSSLSTRNLHAEFVTSNLLRKM
HAYLSFKIVIKVLTEEAEYVYEKFNGFILILNASTLRSDMKRNLKIESNFSFPKPRVMIVLLG
TNAELDILYNFLIRNSLYECLVLLENESGVVNILSWIPDKCGRLKSITYQERCNGRKFDGSQ
NIYIPHRLSVVYGCNISILGTNNPPFSKAINEYFLVDGIDLKVIKIIIRHMGLNINENSYRVNS
EEFIGTGINIEKSFKMEEMLFMRSYYTQKFTWFVVKTGTFRRWSSLTRVFSPEVWFCVFL
SLVAVSVAIGDTLTTWATFLNVSVSRHPHTLKVRIIFFSWMLFSISFTNVFQTYMTSYFTEP
GRQHQIDSIEEVLQSDLDFIVDTVQTRIWPIMLGNRIPYLFFSYDNINMLQFVSERPNSALL
TSEEILLYNYPKLEKKIEFHMVREGGIDARRYLFMSPSSRYLPQVNEIIRRLVESGVVQKIVE
EFVDPVGLMNGFTTQSIFLSEYIPLTTFQMFSTFLYLALGLVLSCIICVGEIYTFKFVSFINFQ
V
>BgerIr783
MFHILQFMLFFLPLLTGLSVKHQDKLQLGKCIRKIMEQIFPGGGDIYVSLTMKDLESLPFIR
EHIGILKRSAEYAFDETSVDKNNEAPARRNYSSQQNHPIKCLKTFYDTLHPHAIYKRSSSD
EIFYMSRERIKWANAMNHFLPQTSDWSGVSNRNISMGAIKDSVLLQIHSQQSWTLVVETF
RKNFVQRYAYRRFDGFLLFLLAEDIEDLFLIDHISMSCSLPKGKIILIILGKDNKKLDNVFNK
LNTLSLYETIVIDHNSSTVNILTVYPEKCGSFGNISILNSWKDGKFIQNIKHLKFHRLKGIQG
CNLYILGRNHPPFLITEKGSPTGIIKDGIDFRLIQLIAFHWDLNIQTTTLYERRKDVVYSDYT
HPFFESKIHPLTYMQRFYTLKLAWFVPRAQSYLHWSSVTRVFSTEVWLSVALVIILVSLSL
KSSLITVAVLLNISVPKMPKSNRIRFVFFTWIIFSFAFTSVFQVFMTSFFTDPGFQHQIDTIE
EMDSSGLNQSLDSFHSEYYYLMIGNKSFYIVFLKGNSQLLKYAVNNSNTAVMTSVEAFLHY
YPRVSKRRDATVFHMLSEDSLSITRFFTMDSTSAFLPFLNEIIRRLVEGGIVDRIAEDFVDP
EGLRRGYNDLKWYNKYAPLSIFEIFSAFLYLCSGLGLSFSVFIFEMIIFSASRYMKI
>BgerIr784
MDWTWYFIVITTISESLNSKVDEQGIANCVVELCKRIIPSGSTIFISLPIANYTLYSFGEENPF
PVIVKKRTFNLEYSSFCWTNKSTISAFYVVKNPAYCPARDKIYVSNSLNYYQDYLLHYMHL
LQRWCIVLSPTFYKGDHVNLLSTKFYYFILLITYEDNDVQNTLSELRKHLKLLMDSDTFNI
FATFILVVTGEIWFSKLSDTIFSYLQMRYVYHIYLVVQTEEYISVYTWFPYRSPSGECGSFKE
SVLLFTYIGRSLITNDETPKADIFSNCTLSTRLLTREPFTILEKVSSKVSYVKDGLFPRLLKAI
ATHMKFSDKTCTIEFNKCDINIQDSLYWSDQSVTNFFTIESTFFLLHSERYPGWSSVLRVF
DFTVWLSFVLSLLITGLFLIYLSRFFNDSDLYRSFVSSILNLWSVILGISVSEIPHSFPLRASF
MSWVIFSLCINTVFQTYVTSYLIDPGFQHQIDTIEELKNLNYSIGICESTVYDYFADETEFMG
QSYFFIRNSDCLLFSLLVPNSAVLISEEVFIYNIQRLCDTYDIPYYHKFSRIVLNLHAGLELFT
SRDAHKTMNVLIARLVSAGIPDKLMRDVLYSNGMGLKVGLTFVDSEYAPISLEHMQSPFL
AMFIGFLISIIVFGGEKLVYSVM
>BgerIr785
MNNIMFRKTYTTIYNIFYALIRPILNVLTIEIKCYVTEIMDWTWYIIILTTVRISWNFKVDEQ
GLASCLVELCKRIIPTGSTIFFSSPIVNYSHYGKGHAYPFQFKKRTFNLNHNRLCNHNSRISA
FYLEKNPAYAPVKDKINISHSLENCQNSILYFLHLLQRWPITLSPIFSSVDNRLQKTRFNYYI
LLTRHKNSVLQSVVSDLKKHLEMLIDSETFNSQAKFIIVLTGEIGFYILADTLINYLHLKSVY
EVFIVLQTEDSISVYTWFPFRYPSGDCSFFKKSILLYTYLYGSLVKKHDYFETDIYSNCTVGT

AVRRQEPFTILEKAESGKLYIKDGLFPRVLKIIATQMKFSDEGCTEEIGNCDTIARDSMYRN
DQSVTNFFTIELTFFMLHSERYPGWSSVLRVFTWAVWLSFVSCMLATGLFFIYCSRFVYDS
HLYRSFVSSVLHLWTVILGSSVREIPHSFALRLSFISWVIFSLCVNTVFQTYVTTYLIDPGFK
RQIDTLEELKNLNYNLGICESTVFDHFAVTTEFMGQSYLFTETRDCILFSLLVPNSAVLINE
EAFIYNIQRICKAYDIPYYHKLTKTVINMNAGLEFITSREVHKKMNLLIGRLVPAGIPDKLV
RDVMNENGIGLKVGLTFENKEYEPISIAHLQSPFLVMFIGFVCSLIALCGEKLFYSVRL
>BgerIr786
MYILQVQNLRCINLQTKNPAIWSAVSETCKRIFPTGRTIFVSVPFTIGYHPKPTGAPHIQYS
NIDKKNITRCIVQTPKLQMFHFMLNGTRLIKLETENLWNPDLEGKIKFHSSNAEGYIIRSL
QECARWPLLISPLHHLNNPEVLVKQNVVIILKLANNDSWTLRTALNVNLYRFTESPAFTQ
GSRFLIVVLEVSLTRELIYQIFRQMSAHKASLTDVYVLTQDATDDSVKLFTSYPYEPPSGKC
GKFSEAVLVDIWVENANGGRFVNNSDINLNKAPKQLQNCCIHLKLDEGVKMGIEKEIARV
VAKKLSGREETCFSYIVLHPRSGDMVFQPELAVRFYTYTLRFVVPLATSNVAWSLITKVFT
MSLWLVVIFCLFCAAFVLKCLAVSSLNKQNSGYNGIVLCLMNTWSALLGIGVSTMPLHYPI
RIFFFSWVMYSLCVNTIFQTFFNSYFIVPGRQHQVDSVKELEELNTTLIFHDFGTVYRFISF
YGLGDRTYFMYLLNAFPYFFSTPNTALFTNDFEFSYFKKTLCRDETSFYHHKFSGDLMQM
NDDIFINDPLLRPRFNSLIFGLVEGGITEKMMMDMFNPTGNTQIMNKMEEFYPLSMIHLS
SAFILLITLHVFSCFIFVVECFASCCRSNL
>BgerIr787P
MYVLHVQNLGCTSINLQTKNPAIWSAVAETCKRIFPTGRTIFVSVPFTLRYNTNYASIRLQE
SDVDKKNITRCIVETPRIQMFHLMLNGTRLINLEPEYLWDTDLGKKINFNSSNAEDYIIRSL
QECARWPLFISPLDHLNNPEVLVKQNVVIILKLAANDSWSLRTALSANVQMFTESPAFYQ
GSTFLIVVLGVSLPRKLIFKIFREMVLHTTILNDVYVLTQDATDDSVKLFTSYPYEPPSGEZG
KFSEAVLVDIWVEHANGGRFVNNSDINLNKAPEQLNNCCIGLQLDEDMKIGIEKKIARVIS
KKMSGTEETCLRDIVLHHRSDESWFQPALAVRFYTYTLRFFVPHATSNVAWSLITKVFTM
SLWLVVIFCLFCAAFVLKCLAVSSLNEQNSGYNGIVLCLMNTWSALLGIGVSSMPIHYPIRIF
FFSWVLYSLCVNTIFQTFFNSYFIVPGRKHQVDSVKELEELNTTLIFHDFGSVMWFISHYGL
GDRTYFMYLLNAFPYFFSTPNTAMFTNDFVFSYFKKTLCKDDTPFDHYVFSGDLMQIHDII
FIQDPLLRPRFNSLIFGLVEGGITEKMMRDMFNPSGITQIVNKMEEFYPLSMIHLSSAFILLI
PLHLLSCFIFVVECFTSLFL
>BgerIr788
MYVLHVLNLVCTSINLQTKNPAIWSAVAETCKRIFPTEIPIFVSVSFTIEYNTKYESIHLHYS
DIDKKNITRCIVETPRLQMFHLMLNGTRLIKLEPEKLWDADLGKKINFNSSNAEDYIIRSL
QKCARWPLFISPLDHLNNPEVLVKQNVVIILKLANNDSWSLRKALNANEYRFRESPAFTQ
GSRFLIVVLGVSLRRELIFQIFREMSVHKTSLNDVYVLTQDATDDSVKLFTSYPYEPPSGEC
GKFSEAVLVDIWFENANGGRFVNNSDINLKKSPKQLQNCCICLQLHEGMKIGIEKEIARVIA
KKLSGREETCLSNIALHPRSGDTVFQPELAVRFYTYTLRFFVPLATSNVSWSLITKVFTLSL
WLVVIFCLFCAAFVLKCLTVSSINKDHSGYNGIVLCLMNTWSALLGIGVSSIPLHYPIRIFFF
SWVLYSLCVNTIFQTFFNSYFIVPGRQHQVDSVKELEELNTTLIFHDFGSYTWFISIYGLGD
QTYFMFLFNAFPYFFSTPNTALFTNDFEFSYFKKTFCRDDTPFYHHVFSGDFMQIHDSIFI
NDPLLRPRFNSLIFGLVEGGITEKMMRDMFNPTGNTQILNKMEEFFPLSMIHLSSAFMLL
VPLHLLSCFVFVVECFAYLLL
>BgerIr789
MIHLEVMAAIILLQDNPAIWNAIAETSKRIFPRQQTIVVSLPLTIEFQGTITTLPILDVMKTE
VKKDDVCLIEKSKIEMRYFTMNGTRIKHFSSEEELILKDHVIPKFQTSYLDDAILKKIHEICT
RPILVSPIKDAHFPGLLSKKSFILLLNVEDDDASKLHWDYLNQHVLMIKYSVQSFDTGQKF

LVVIIGVPGTKKMVLRILDAMSYVIFNINDVYVLTQGPNHGTMELYTWYPYDLPSGDCGKL
KDVILLDTWIEAENGGKFVQNLNFSTNKIPSQITGCCFDMMDPLGRAGVEKAITKLVSEKI
SGDQSLCRGRIEVSSKSIIFINQPEVSARFFMIVYRFFIPVSKSYVSWSHITDVFHVSAWLFV
VCSLIISAFFLKWLMACTFEQEYSMFKNIVLCALTTWSALLGVSVPTAPISTPMRIFLFSW
MLYSMCIATVFQAFFNSYFVVPGRQHQVDSVQELEATNTTFLFNSLDTFTRFAMTYDLAH
HSFLLDKLMAYKYFLSVPNTALFTNTIEFSHYMKKICGKERLQVYHKLTGDDMQFTDDLV
TYDPLVRSRFNDFVTRIVESGISEKIMNDLWNHASDDEYFFQLEKLKDTFFPLSVLHLWSA
FGVISLLYICSLLVFISELTCSSRKKN
>BgerIr790
MLEDNPAIWNSIAETAKRIFPPHQTIFVTLPFSVLTYGEIIETQPSAALTTFKVKNDVYCSM
DQREFVAYYWTLNGTRVQHIDMYEEYDKTNEHIQFNTSHLEDLIIKNIHDVCMRPVLVSP
LVYPYIPGLLDKKSFIVLLKLDVNNDVNYESEYDTGKNWREVILILRYSTYAFDSGARFLVA
IIDLPCSKEKVLKMFRVMNFDFNLNDVYILTQGSNPGTMELFTWYPYELPSGECGKPRDA
VLLDTWVDTEDGGHFVKNLDFSLNKNPTDIVNCCLEIPHAQDGIRSKIVSLVSRKMNRNS
ETCTEVINVIPRQNSFDYPQFRARFFTFDFRFFVPVAESYFSWSSITDVFHVSAWMFVIFSF
LTAAFFVKWLAGSSYCVENPGYNEIVRCLMSSWSMLLGIGDSILPINAPMRIFLFSWILYSM
CIGTVFQAFFNSYFVVPGRLHQIDSVQELAENNATLLFVSYDTFMRFSIAYNLAHYSYFVYQ
ANAYKYMINTPNTALFSNTLEFSYYMTQYCGKQTSRTFHRFSGNEMQVDDFLIVSDALIQ
PLLKRIVTQLVEGGIPEKIIREKLNPSGDYANMFELEKVTDNFFALSFLHLWSVFGLILALH
VLSLFIFISELLWHEIKNYRISRRIRRTFLS
>BgerIr791N
LIFWAAAVIKGAIPAIPILEDNPAIWNAIAETTNRIFPPLQPIFVSLSPTFDFGDAIHKLPML
TRTKIKLKNGGPCVTDQPLLDAYYWILNGKRLQHPDLNNEKYKMSTAVKFNTSHLEDIIL
KKIHDVCKRPVLVSPVLYPYVPGLLDKKSFVLLLKVNKNDEEGYDLPESWRQTIYIFRYST
LTFNTGSRVLIVILDMVYSKEKIIKIFKSINTPKININDVYILTQGSNPGTMDLYTWYPHELP
SGECGNFKETILLDTWVESDEGGKFVKNLNLSLNKSPIDMHNCCLKLEPFNPIKFGVETEI
VKLVSRKMNRDSKTCKSVIFVNTRSQGWRQSLVSVRFFTFDFRFFVPVAESYFSWSSITDV
FHVSAWLFVIFSFLAAAFFVKWLAGSSYCVENPGYNEIVRCLMSCWSLLLGMGDSILPINA
PMRIFLFSWILYSMCIGTVFQAFFNSYFVVPGHQHQIDSVQELAETNATLLFTSYDTFTRFS
PTQSLGKVSYLLFYWDAYKFMMSTPNTALFSNSLEFSYYIKHVCKQQTPRTYHQFTGDEM
QVDDDMYVSDPLIRSHFNKLVTQVVEGGIAEKLIKDTFITFGDEANDFQLQKMKDNFFAL
SFLHLWSVFCVLLLLHMGSLLVFIFELVYHGTKQ
>BgerIr792N
TSWFFLGLALGSKEIKLISHKNEDVILNCILEASIRFFDHQHPVAVIPYAFELVVMSNHTTW
TKKINIDLNNKCVRQPVILTKFSPPNSFNWDSLLTMRFEWTNRIEQALAKLHSSQRWNIL
LGSSNYIFPLDTYKNYIVFIPNIDDENELFLYLCLHLNKSARFLLVFMYGANDDEEIIMSYFQ
FTTCFRDALILLWKENSERVDVKTTHPYTLPYGQCGVDFKFMHLDTWVQDETGGSFLKN
NADFHINKVPDKLKCCEITLYDAPPHFPPYKFIKSNLHGQVTNDGIDVRILSHINDAMMQN
ENCETEEEIFVVNTLQPHEFSFSTRYILFPYFGLRYTYFVPKAESYPRWTWFTAVFSRSVW
ICFIVSLLATALAMRCLATSNLNQYSETYNDIGYCLLKSWSVFMGIGVYIPPKIYFHIVFFSW
LSFTICVTTVFQAFITSFLVVQVKEHQIDTLEELREKGFTVIYDSTSKLKQSSGLNITRAFFA
EIEHAYMYVVNTPKTALYSTEAYFQYNQNTLCAKDQISAYHKLELHQDSVTFYGLTFLVD
TIIEHKLHHVLKMLSVVGIIDKIFNDVVDPTGRRLMPERSSRLDYEPMDIGYLQSCFYSLLC
GLSLSIAVFIVECFVKHRWK
>BgerIr793N

TSWFFLNLAQGRKEIKLISHKNEDVISNCILESSIRFMDHQHPIAVIPYAFELDMMSNRTKF
DCKIINMDANNKCVQQPVTSRKCSPPNFFNWDNLLSVRYEWTNRIEQVLAKLHSSQRWN
ILLGSSNYIFPLDTYKNYIVFIPNIDQHDPKLLLYLCMNLNKSARFLLVLMYGANDDEQITK
SYFDFTQCFRDSLILLWKENSERVDVETTHPYTLPYGQCGIDFDSMHLDTWVQDETGGSF
LNNNTEFYINKVPDKLKCCEITLFATYPHFPPYKFIKSNLHGQATYDGIDVRILSHINDAMT
QSENCKTKEEILVINTLQPHDFSFFTRHIPLPYLGLRYTFFVPKAESYPRWTWFTAVFSRS
VWICFIVSLLATALTMRFLATSKLNECSETFKDIGYCLLLSWSVFMGIGVNIPQKTTFRTVF
FSWVAFTICVTTVFQAFITSFLVLQVKEHQIDTLEELREKGFTVIYDSTSKLKQSSGLNITRA
FFAEIEHAYMYVVNTPKTALYSTEAYFQYNQNTLCAKDQISAYHKLKHNQDSITFYGLTLL
LDTIIEHKMHHVLKMLKVVGIIDKIFNDVVDPTGRRLMRESFSRLDYEPMDVGYLQSCFYS
LFCGLSLSTGVFIVECFVKYWWK
>BgerIr794
MFFSALLILLTANWYLLHFTETTELLSQKNQDTIVNCILQTSIKYLDHKHPIAVIPNAYQVG
DHVQHTPYEMSNVTVDEKQKCVIVPMTLNMFINPKNFDKENTITVNLTWTYTMELVLR
KLHTAQRWPLLLGSTNYIYPLNVYRNYIVFIPGIPSFGNILNLWFLCINFRKSARYLVVLINS
SKDNEELLRRFHQLTFCFRDVLLLVWNQTSEQVDVKGTVPYTLPYGRCGKIDHFFDVET
WIQDDSGGSFLKNADLRFFNVPDKLKCCNVSLGMVFDDLPPYNFRKLESNGEVIFDGIDV
RLLKHINDGMVDNENCTRIHQIYAINTLQTEEINVVMGQDPYPYDSLKYTIFVPEADSYPR
WAWFTAVFSTNVWISFLISLSLTALAMRCIACSKFNSCSGNYKNISYCLLNVWSIFAGVGV
NKIPASVSLRTLFFSWIAFSICVCTVFQAFITSFFVLQFKERQIETLQELQEDDFTIVYDSLKK
YFLNHDSKIASTCFEEIEHAFLYAINTQKTAFYSTENSFQLNMNRRCYNDRVSPYYKFKDY
DDSNNYYGLMIFYDGVLQDKMRDILKRLTTTGIINKVFSDIVEPTGQKWLSVNPSILEYET
MSIGYLQSCFYSLLCGVILSLIVFFMEICVKMWESKFTRHR
>BgerIr795NI
VNWSLLHLNQATELLSQKNQLLSHKNQDTIVNCILETSIKYLDHNHPIAVIPNAYQVGENV
QHTPYEMRNVTIDERQKCVNGPMTLKMYINPKNFDIETLQELQEEDFTIVYDSLKKYFLN
HDSKIASTCFEEIEHGFLYAINTLKTAFYSTENSFQLNMNRLCYNGRVSPYYKFKDYDDSN
NYYGLMLFSDGVLQDKMRDILKRLATTGIIRKIFSDIVEPTGRKWLSVNLSILVYETMSIGY
LQSCFYSLICGIILSLIVFFMEICVKMRESKSTRHR
>BgerIr796F
MFQFVAIVDNTPICIKNTHSYKVLHKNTKSFKLSNICFSSQTQANWSLLHLNQATELLSQK
NQDTIVNCILQTSIKYLDHKHPIAVIPNAYQVGVNVQHTPFEIKNVTVDERQKCVNGPKTL
KMYINPKNFDKNNIITVNLTWTYTMELVLDKLHTAQRWPLLLGSTNYIHPLNVYRNYIVF
IPGIPSFGNILNLWFMCINLRKSAKYLVVLMNSHKDNEKLLRKFSQLTYCFRDFLVLVWN
QTSEQVDVKATLPYTLPYGRCGKTHHFFDVDTWIQDESGGRFLKNADLRFFKVPDKLKCC
NVSLGMVSDDLPPYNFRKLESNGEVIFDGIDVRLLKHINDAMVDNENCSRIYPFYAINTLQ
TEEINVVSGQDPYPYDNLKYTIFVPEADSYPRWAWFTAVFSTNVWISFLISLSLTALAMRC
IVCSKFNSCSGNYKNISYCLLNVWSIFAGVGVNKIPETVSFRTLFFSWIAFSICVGTVFQTFIT
SFFVLQIKEHQIETLHELQEEDFTIVYDSLMKYFLNHDSKIASTCFEEIQHAFLYAINTQKT
AFYSTENSFQSNLNRLCYNDRVSTYYKFKDYDDSNNYYGLMLGFDGVLQEKMRDVMKRL
TTTGIIKKVFSDVVDPTGRRWLSVNPAILEYETMTMGYLQSGFYSFICGIILSLIVFFLEICVK
IYQERKSIRHR
>BgerIr797N
IEVTLCIPLLTQDIKLVSHKNEEVFMNCVLQASLKFLDHQHPVAVIPSGFGKAISKLRYIERI
EKNRFKLNGTQKCLKEVERLEVKSRSESYDFEKPITVNFTWTQLTESVLGKLHRSQRWSF
LLGSTSFVFPLDVYKNYIIFIPAGQTPMDFFYLCLKFNKAARFLFVIMNSAKENLTIIKKFGI

KTDMTCFRDILIILWDINSDRVFIKTAFPYTQPYGQCGKINKLLDLDIWILDENGGSFVNNA
DFHPNKIPDKFKCCDILAYKVENYFAPFHFQKKRLGRMGTTADGIDIRIATHIHDALTSNE
NCSILKYIHLTNTLQPIMFTFVSVANPYPYQCLVYTYFVPKSGTYPRWAWFSTVFTLNLWI
CFFVSLLVTAVAMKYIANSKNSHDSGNFKTMTCCIINAWSIFVGLGVNSIPRNVSFRFILFS
WIAFSLCVSTIFQVFVTSFLVVQINEHQVDTVEELRDKGFTVIYDSFINFFRKFDANSSSHYI
GPNDRAFQYAINTPKTALHSTEFSFQFNLNTLCKDNDVPIYHKFKHHEDSTNYNGLALVF
EGPIEHKVREVLKRLTNGGIPEKIIRDVTDPKGRNVLSESSSIFQYEPMDIGYLRSCFYSLLS
GLSMSILIFLLEVLIKRKFI
>BgerIr798
VNWCSSHGFHLEMDRNENIVINCVVQASAKFLDYEHPAAFIPSGFGNAISRLRYEDKIDKN
RFKFNSTQKCHKEDNWKVAYNVSKAIDWEVPIVVNFTWNHVRESVIGKLHSSEHWSFLL
ASSKYVYPLDTYRNHIIFVLPGETLNDFFYLCIKFNKPGRYVFVLTNSPEENNKIIKRSLIFIH
VTCFKDFLILVWDRNSDRVEIKTALPFTPPYGVCGSIFKLIDLDTWVMDEKGGSFVKNANL
RLEKYPEKIKCCHISVFTPIKISPPFYFETERFKYDGIDARIAKHLEDAMTDNRNCEGMPIR
DLFITNTQLPILYSFVTVHEPYPYQNLQYTYFVPKAGAYPRWNWFSSVFSTNLWVCFFVSL
SVTTISLRFLATSKNTLHFGNYKNITYCLMNSWSVFVGIGVDKIPQGFRFRTVFLSWIVFCV
CISTVLQAFITSFLVLQVQDHQIDTVEELQEKGFTIVHDNFYKFLVNHDSSQESHYIRPSTR
AFQFAINTPKTALYSTEFGFQYNVNTLCNRSDTPQYYMFKHYQDSINFNGLVMMFNGDLE
PRMRQLLKGLTNGGLPDKIFRDVTDPTGRRVLSEKSPLLQYDPLDVGYVQSCFYCLLGGLS
LSFLLFCVEMFVNVFWSRG
>BgerIr799N
VNWCLSRGFNLETDRNENIVINCVVQASAKFLDLELPIAFIPSGFGNAISRLRYPDKIEKNR
FKFNSTQKCFKEDGWEVADSVSKAIDWQVPIIVNFTWNHLRESLMGKLHSSERWSFLLA
SSKFVYPLDTYKNHIIFVLPGETLKDFFYLCMKYNKSGRYVFVLTNNPEENKKIIDSFFMRI
HVICFKDFLILVWDRNSDTVEIKTALPFTPPYGVCGSVFKLIDLGTCVSDEKGGSLVNNTNL
RLEKYPEKIKCCHISVFTAIKVLPPFYFETEPFKYDGIDARIAKHLEDAMTDNRNCKGLPIR
DLLITNTQLPILYSFVTVHEPYPYQNLQYTYFVPKAGAYPRWNWFSSVFSTNLWVCFFVSL
SITTISLRFVATSSNTKHFGNYKNVTYCLMNSWSVFVGIGVDKIPQGFRFRTVFSSWIVFCV
CISTVFQAFITSFLVLQVQDHQIDTVEELQEKGYTIVHDNFYKFLVNYDSSQESHYFSPSTR
AFQYAINTPKTALYSTQFGFQFSLNTLCNRSDTPQYHKFKHYQDSINFNGLVMMFNGDLE
PRMRNVLKGLTNGGIPDKIFRDVTDPTGRRVLSESSPALQYAPMDIGYVRSCFYCLLGGLS
LSFIVFCVEIFVNVFWSRG
>BgerIr800N
LNWCLPNFAEAIKWTSHKNEDVIINCVVQASIKFLENQHPVAVIPNRFGKAVTRFKYGDK
MKINYLRLNGSHRCYEELPGDILKSISNVTDWERPVTVKFTWTHVPESVMGKLHSTEHW
SFLLGSSSYIAPLDTYKNYIIFVHAGQTPKDFFYLCLKFNKSARFMFIIMNSAKANQQIIRSF
KEKTRATCFRDILVLVWNRNSGEVGIKTIFPYTRPHGHTNKGYKLFDLDTWVMNAEGGS
FVNNVHLHPSKIPETLKCFNFTVFTGNPQFPPFTMLTEHIRTELNGRTTRRVEFDGIDNRI
AKLVQDALMDNENCITIERIYITNTLQPIMHTFVTLPDPYPYQNLVYTYFVPKAGTYPRW
NWFSSVFSSNLWASLFVSLFVTAVCLKCIASSKTNQKSDGNFKDISYCLMNVWSVFVGAGI
ANIPQSTNVRTVLLSWIVFCVTVSTVFQAFITSFLVVQVQEHQINSVEELQEKDYATIHDSF
INFFLNFNRNATSHYVEPASRAFQYSINTPKTALFSTEQFFQFNLNTLCKEFELPSYHKFT
NFEDSTNFNGLVLMFDGVIEHKFRQVLKRLTNAGIPDKVFSDFVEKLGRRVLAEKRPAML
YEPLGNDYLQSNFYCLLCGLLISCFVFIAEIVSCRLA
>BgerIr801N

VDWLNLAEGTILIPNRNDDVIVSCVLQASSKYFEPEEPIAFVPPIADAKHFEFEVDNRTEYY
TLDTGKKCVSKTTKLYKYNLIKSFVFSNFVNLDTVRFEWTHTTEYILSSLHSSEAWQLALA
SVNFTKELNKYKNFIIFLPGLKYVFNLAYLCPLMNKSAKFILIFANTYEETQEMEAIFIQMV
QIACFRDFLVLVRDINSLQVSIKTALPYTPPYGYCGTILELIHIDTWNYDEKERGFLKNAQF
KIEKVEKKLRCCQIQEEILLEDLPPYYFKVESRYDGVDVRLVSYLNNALTDMEGCDNMRHI
TVSNSVQEESYNLEYGKSATPYHSLRYTYFIPMSESYHLWMWFTAVFEKNVWISFLVSLTI
TAATMRFLASSSQLSSYKSVTYCLLNAWSVFVGLGVGNIPQSCSFRTVMISWIVFCLCVSTV
FQTFVTSHLVLQSREHQLDTVEELHQHGYKIIYDITLKAVQNSEFNNNTTPLYQRSDHAFL
YAINTPKTALYTTEESFHYNLKTLCANNELPPYHKLKHYEDSNNFYELIFIEDRFLQYKIDD
VMKKLGTAGIVKKIFRDVVNPIGRKVLSESSPVLEYEPMDIGYLQSCFYALLSGLLLSIVVFFI
EVLVNRRGVFS
>BgerIr802P
MLFYTYGTFSTSLQVTSLLLVQGIKLPFDENEDTIASCVLESSIKFFQHEYPVAVVPQIIHAIE
STDSDRMFTRQNITFDTDMKCVHEPTELKGDRVFKYLDASKFDNHAVYFLSWSHIAEKSL
RRLHSSEFWPLFLASSRNIVPQNTFKSYIIFVPDEEYFFYLTRWCIALNRAAKFILVFSNNA
EENTYDIPFELGFTCFKDVLILIWDRNSDRVYVKTALPYTPPYGLCGMFRQLIDLDTWIQD
ANGGSFQKNADFRLNKIPHKLKCCVIENSFRNWSSPPYYFPVSTSADTGPTYDGVDIRLIN
HINDEITDLENCEEKEIMQVTNTITTITSSLLEQKEAYPYHSLRYSYFVQRAGTYPRWIWF
TSVFNKCLWMTFFVTLGLIAVVMRWVSSSRLILQSCYYKNIIYCLLNAWSVFLGQGANNIP
ENIKFRIVFLTWIIFTLCVSTVFQTFVTSFFVFQVKDHQIDSPEELPGEEYTVVYDSIFKILTL
YDESLLSIYIHSGKNAFQYAVNTPKTALYSTEEYFKYNERKFCRNNEIPRYHKFKYYEDSN
NYNGLLFVIDGLLQNKMNRVLQKLSTGGMIQKIFNDVVDPSGRNILSEKYSELDYEPMELG
YLZSCFYSLICGLSLSLIVFCLEISLKRRCRYF
>BgerIr803N
VTSLILGQGIKLPSYENEDVLVNCVLQSSIKFFQHGFPVAVVPLMIDAIEITDSETMFMSQNI
TFDTGKKCVQQSAEFEGNRLFKYFDVSKFENHAIFFLKWSHIAERSLRRLHTSEFWPLFLA
SSRNIVPLNTYKSYIVFVPDVEYLAHLPHWCLLVNKGAKYILVFSNTAEENRYAINILIELQF
VCYKDILILIWDRNSHRVYVKTSLPYTPPHGLCGEVLQLIDLDVWIQDGSGGSFQRNADFR
LNKVPNKLKCCVIDDTITNDFSPPYNFLLSTSADTGPTYDGVDIRILNHINDEMTDLEKCE
VKVRIFVTNTIMTMATSLLEQRVAYPYHSLRYSYFVQRAGTYPRWTWFTAVFNKILWMIF
FVTLGLIAVVMRWISSSRLILDCSSYKNIIYCLLNAWSVFLGQGANNIPENIKFRIVFLTWIIF
TLCVSTVFQTFVTSFFVFQVKEHQIDTPEELPGEEYTVVYDSISKMLTFYDESLPSISIQPFK
QAFQYAVNTPKTALYSTEEFFKYNERKWCKSDEIPRYHKFKHYEDSNNFNGLLFNLDGLL
QDKMDRVLQKLAIGGIIQKIFNDVVNPSGRKILSEISSELVYEPMELGYLQSCFYSLICGLSLS
LIVFCLEIFLEGRFRRF
>BgerIr804N
VTSLLLVQGIKLPFDENEDLIVNCILESSLKFFQQGSPIAVVPQIIDAIEINDSGSMFKTQNIT
FDTDKKCVQEPTEMERHNLFKYFDVSKFENHLTFLLTWSHIAERSLGKLHSSEFLPVFLAS
SRNIVPLNTFKSYVIFVPDNESVVLLMYWCILLNKDAKFILVFSNTAEENRYAINILFELEFV
CYKDVLILIWDRNSDRVYVKTSLPYTPPHGLCGEVRQLIDLDVWIQDANGGSFQENAEFR
LNKIPHKLKCCIIQDTYINDASPPYYFTERTFEEGPPTYDGVDIRLIAHINDEITDLENCEGN
VIIYVHNTILTMASTLVDQKVAYPYHSLRYSYFVQRAGTYPRWTWFTAVFNKNLWMTFFI
TLGLIVVIMRWISSSRLILHSCYYKNVIYCLLNAWCVFLGQGANNIPENVKFRIVFLTWIIFT
LCVSTVFQTFVTSFFVFQVKEHQINTPEELPAEEYTVVYDSISKILTFYDENVRAISIQPFKH
AFQYAVNTPKTALYSTEEFFKFNERNWCKMDEIPRYHKFKYYEDSNNFHGLLFSIDGVLQ

NKLDRILQKLATGGIIQKIFNDVVDPSGRRILSEISSELEYEPMELAYLQSCFYSLICGLSLSLI
VFCLEIVLKWRYRYFLKTHKMF
>BgerIr805N
VTSLLLVKGIKLPFDENEGTIASCVLESSIKFFQHGSPVAVVPQIIHIIEPNYTDRIFTPENITF
DTDMKCVQEPTDLQGDRVFKYFDVSKFENHAVYLLGWSHIAGKSIGKFHSSEFWSMFLAS
SRNIVPLNTFKSYIIFVPEEEYLLYLIRWCDALNRSAKFILVFSNSAEENTYAMDIFFEFEFL
CYKDVLILIWDRNSDRVYIKTALPYTPPYGLCGMVRQLIDLDVWIQDGSGGSFQKNIDFRL
NKIPHKLKCCVIENSFSDWFSPPYYFPLSTSADTGPTYDGVDIRLLNHINDEIMDLENCEEK
RRIQVTNTILNPASTLVEQRVAYPYHSLRYFYFVQRAGTYPRWTWFTAVFNKILWVIFFV
TLGLVAAIMRWISGSGLLLDSSCYQNIIYCLLNSWSVFLGQGANNIPENIKFRIVFLTWIIFT
LCVSTVFQTFVTSFFVFLVKEHQIDSPEELPAEEYTVVYDSVSKMLTLYDETLPSICIQSVKH
AFQYAVNTPKTALYSTEEFFMYNERKWCKNDEIPHYHKFKYYEDSNNFNGLLFNLDDLL
QEKINHVLQKLATGGIIHKIFNDVVDPSGRNILSEKYSELEYEPMELVYLQSCFYSLICGLSL
SLIVCFFEIFLKRR
>BgerIr806P
MTTLLLVQGIKLPSYENEDIIVNCVLESSIKFFQHGSPVAVVPQIIDGMETTDSETMFATHN
ITFDADKKCMQEPTILEGNRLAKYFGVSEFENYAIYFHNWSHIAERSLXKLHCSEFWPMFL
ASSRSIVPLNTFKSYIVFVPDDEFLINIQEWCILLNRGAKFILVFSNTADENIHALDILIEVEF
ACYKDVLILIWDRNSDRVSVKTALPYTPPHGLCGEVRQLIDLDVWIQDGSGGSFQENVDF
QLNKIPNKLKCCVIQDTFTNWFSPPFYESTSEDDTTYDGVDIRLLNHINDEIADLENCEEK
EFMHVTNTILNPASSLLEQRVAYPYHSLRYSYFVQRAGTYPRWTWFTAVFNKILWMTFF
VTLGLIAVVMRWISSSRLILDSGCYKNIIYCLLNAWSVFLGQGANNIPENIKFRIVFLTWIIF
TLCVSTVFQTFVTSFFVFQVKEHQIDSPEELPGEEYTVVYDSVSKILTLYDKSVPSICIMPFK
YAFQYAVNTPKTALYSTEEFFKYNERKWCKNDEIPRYHKFKNYEDSNNYNGLLFNMNGV
LQDKMNRVLQKLATGGIIQKIFNDVVDPSGRKILSEKYSELDYEPMELDYLQSCFYSLICGL
SLSFIVFCLEVVLKGRCRYF
>BgerIr807P
MLGQGIKLPSYENEDVLLNCVLESSIKFFQHGHPVVVVPQMIDAIETSDSETMFTPQNITF
DTDMKCVQQRTELEGNRLFKYFDFLKFENHVKYVHSWSFISERTVRKLNSSQFWPLFLAS
SRNIVPLNTFKSYIIFVPEVEYLIHLPFWCLIVNKAAKFMLVFSSTEEENKRTLDLIYEMNF
ACYKDFLILIWDRNSDRIHVKTALPYTPPHGLCGAVRQLIDLDVWIQDGTGGSFQRNSDFR
LDKIPHKLQCCKIDESLTSLHSPPYYFLESTSLKYASIFDGVDIRVLSHVNDAIMINENCSDK
DIIFVTNTLLSVANSLFHQALAYPYHSLRYSYFVPRAGTYPQWNWFTSVFNELLWASFFV
ALGSVAFVMRWISSSGFDCRPGCYKDVIFCLLNAWSILLGLGANNVPENIKFRIVFLSWIIF
TLCMSTVFQTFVTSFFVLQVRKHQLDSPEELQLFTIPHGKCWLFMTKMFLPSAFGHSNRP
SSMPZRRPKQPCIQQKNFLSTTRESGAKVTKFLVTTNSNIEDSNNZNGLLFNMDGVLQDK
MDRVLQKLATGGMIKKIFNDVVDSARRRILSEISSELEYEPMELGYLQSCFYSLICGLSLSLI
VFCLEVVLKGRCRYF
>BgerIr808NP
VTSLILGQGIKLPSYENEDVLVNCVLESSIKFFQHGSPVAVVPLMIDAIDITDSETMFMSQNI
TFDTGKKCVQQSTELEGNRLFKYFDVSKFENHAIFFYKWSHIAERSLGRLNTSEFWPLFLA
SSRNIVPLNTYKSYVVFVPDVEYLAHLPHWCILVNTGAKFILVFSNTAEENRYAVEMLFEL
EFVCYKDVLTLICDKNSDRVYVKTALPYTPPHGLCGEVRQLIDLDVWVQDGSGGSFQRND
DFRLNKVPNKLKCCVIDDTFTNGFSPPYYFPLSTSADTGPTYDGVDIRLLNHINDERTDLE
NCEVKVRIFVTNTIMTMASSLLEQKVAYPYHSLRYSYFVQRAGTYPRWTWFTAVFNKIL
WMIFFVTLGVIAVVMRWISSSILILDSGCYKNIIYCLLNAWSVFLGQGANNIPENIKFRIVFP

TWIIFTLCVSTVFQTFVTSFFVFQVREHQIDTPEELPGEEYTVVYDSLSKMLILYDESVPSISI
QPFKHAFQYAVNTPKTALYSTEEFFKYNERKWCKSDEIPRYHKFKHYEDSNNYNGLLFN
MDGVLQDKMDRVLQKLATGGIIQKIFNDVVDPSGQKILSEISSELEYEPMELGYLQSCFYSL
XCGLSLSLIVFCLEIVLKGRFRRF
>BgerIr809NP
VTSLLLVQGIKLPFDENDDVIVNCVLQSSIKFFQHGSPVAVVPQIINTIETTYTDRMFTPENI
TLDTDMKCVQKPTELQGDRVFKYLDVSKFETHAIFFLSWSHIAEKSLGRLHSSEFWALFL
ASSRNIVPLNTFKSYIIFVPEDEYLVYLTRWCEALNRGAKFILVFSNIAEKNTYVIGVFFEIEF
LCYKDVLILIWDRNSDRVYVKTALPYTPPHGLCGEVRHLIDLDTWIQDMNGGSFQKNVDF
RLNKIPNKLKCYVFANSFPHLFSPPYYFSVSTSADTGPTYDGVDIRLLNHINDEITDLENCK
QVRRMQVTNTILNPVSTLLEQKEPYPYHSLRYSYFVQRAGIYPRWTWFTTVFNTFLWMIF
FVTLGLIAVVMRWISSSRLILHSCYYTNTIYCLLNAWSVFLGQGVNNIPENZKFRIVFLTWII
FTLCVSTVFQTFVTSFFVFQVKEHQMDTPEELSGEEYTVVYDSIYKVLTLYDESLLSLSITSV
IHAFQYAVNTPKTALYSTEEFFKYNEQKLCENDEIPRYHKFKYYEDSNNFNGLVLIIDNVL
QDKMNRVLQKLASGGIVQKIFNDVVDPSGRNILSETYSELEYEPMELGYLQSCFYSWICGL
LFSLVVFCLEIGLKGRCRYF
>BgerIr810NP
VTTLLLVQGIKLPSGKNEDVIVNCVLESSIKFFQHGSPVAVVPQIIDTIDILHLIQXMRCVQQ
PTKLEGKLLLKYYDVSKFNIQSIFILSLSYIAERSLGRLHSSEFWSLFVASSRNIVPLNTFKNY
IIFVPDEEFVEYLIHWCVLLNTDGKFVLVFSKAANENRYAMGVLLELEFACYKDNLILIWN
RNSDRVYVKTALPYTPLYGLCGMVRQLINLDVWIQDANGGSFRRNADFRLNKIPHRLKCC
EIKNTYMKFFAPPYNFIGSTSQNGRNYDGVDIRIIAHINDEITDIENCDEQSTIFVHNTILITA
NTLFDQKVAYPYHSLRYSYFVQRAGTYPRWIWFTAVFNKILWMTFFVTLALIAVFMRWIS
SSRLILDSSCNNNIVYCLLNAWSVFLGQGANNIPENIKFRIVFLTWIIFTLCVSTVFQTFVTS
FFVFQVKEHQIDTPEELSDEEYTVVYDSVSKILTLYDESLLSISIHSVKHAFQYAVNTPKTAL
YSTEEFFKYNERNWCKNDEIPRYHKFKYYEDSNNFFGLLFSVDGILQYKMDRVVQKLATG
GIIQKIFNDVVDPSGRRILSDISSELEYEPMELGYLQSCFYSLICGLSISLIVFCIEIVLKRRCR
>BgerIr811N
VTSLLLVQGIKLASDKNEDVILNCVLESSIKFFQLGSPVAVVPQMIDLIDTSDSETMFKTQNI
TLDTDKKCVQEPTEMERHSLFKYLDVSKFENHGKIFLSWSHIAERSLWKLHSSEFLPLFLA
SSRNIVPLNTFKSYVIFVPDNESVVLLMYWCILLNKDAKFILVFSKTAAENNFAMNVLFDF
DFMCFKDNLILIWDINSDRVYVKTAFPYTPPYGRCGRVLQLIDLDVWIQDANGGSFRENA
EFGLNKIPHKLKCCIIQDTYINDDSPPYYFTERTFEEGPATYDGVDIRLIAHINDEITDLENC
EGNMIIYVHNTILTMASTLVDQKVAYPYHSLRYSYFVLRAGSYPRWTWFTAVFNQILWMI
FFVTLGLIAVVMRWISSSRLILHSTYYKNIIYCLLNAWSVFLGQGANNIPENVNFRIVFLTW
IIFTLCVSTVFQTFVTSFFVFQVKEHQIDTPEELPDEEYTVVYDSISKILTFYDENVRSISIQP
FKHAFQYAVNTPKTALYSTEEFFKFNERNWCKKDEIPRYHKFKYYEDSNNFNGLLFNIDG
VLQNKMDRILQKLATGGIIQKIFNDVVDPSGRNILSETYSELEYEPMELGYLQSCFYALICGL
SLSLIVFCLEIVFKRRCRYF
>BgerIr812N
VTSLLLVKGIKLPFDENEGTIASCVLESSIKFFQHGSPVAVVPQIIHIIEPNYTDRIFTPENITF
DTDMKCVQEPTELQGDRVSKYFDVSKFENHAVYLLGWSHITEKSLGKFHSSEFWSMFLA
SSRNIVPLNTFKSYIIFVPEEEYLLYLIRWCDALNRSAKFILVFSNRAEENTYAMDIFFEIEFL
CYKDVLILIWDRNSDRVHVKTALPYTPPYGLCGMVRQLIDLDVWIQDGSGGSFQKNTDFR
LNKIPNKLKCCVIENSFSDWFSPPYYFPLSTSADTGPTYDGVDIRLLNHINDEIMDLENCEE
KRRMQVTNTILNPASTLVEQRVAYPYHSLRYSYFVQRAGTYPRWTWFTAVFNKILWVIFF

VTLGLVATIMRWISGSGLLLDSSCYQNIIYCLLNSWSVFLGQGANNIPENIKFRIVFLTWIIF
TLCVSTVFQTFVTSFFVFLVKEHQINSPEELPAEEYTVVYDSVSKMLTLYDETLPSICIQSVI
HAFQYAINTPRTALYSTEEFFKYNERKWCKNDEIPRYHKFKYYEDSNNFNGLLFNLDDLL
QDKVNHVLQKLATGGIVHKIFNDVVDPSGRNILSETYSELEYEPMELVYLQSCFYSLICGLS
LSLIVCCFEIFLKRR
>BgerIr813NP
MTTFLLVQGIKLPSYENEDIIVNCVLESSIKFFQHGSPVAVVPQIIDGMETTDSETMFATHN
ITFDADKICVQEPTILEGNRLAKYFGVSEFENYVIYFHNWSHIAERSLWKLHCSEFWSMFL
ASSRSIVPLNTFKSYIVFVPDDEFLINIQDWCILLNRGAKFILVFSNTADENIHALDILIEVEF
ACYKDVLILIWDRNSDRVYVKTALPYTPPHGLCGEVRQLIDLDVWIQDGSGGSFQENVDF
QLNKIPNKLKCCVIQDTFTNWFSPPFYESTSEDDTTYDGVDIRLLNHINDEIADLENCEEK
EFMHVTNTILNPASSLLEQRVAYPYHSLRYSYFVQRAGTYPRWTWFTAVFNKILWMTFF
FTLGLIAVVMRWISSSRLILDSGCYKNIIYCLLNAWSVFLGQGDNNIPENIKFRIVFLTWIIF
TLCVSTVFQTFVTSFFVFQVKEHQIDSPEELPGEEYTVVYDSVSKILTLYDKSVPZICIMPFK
HAFQYAVNTPKTALYSTEEFFKYNERKWCKNDEIPRYHKFKNYEDSNNYNGLLFNMNGV
LQDKMNRVLQKLATGGIIQKIFNDVVDPSGRKILSEKYSELEYEPMELGYLQSCFYSLICGH
SLSFIVFZLEVVLKGRCRYF
>BgerIr814N
VTSLLSVQGIKLPFDENEDTIASCVLESSIKFFQHGYSVAVVPQIIHVIETSYTERIFTPENITF
DTDMKCVQEPTELQGRRVFKYFDFSKFENLAVYLLGWSHIAEKSLGRLHSSEFWSLFVAS
SRNIVPLNTFKSYIIFVPDEEYLLHLTRWCDALNRGAKFILVFSNNAEENTYVMGIFFEIEF
LCYRDVLILIWDTNSDRVYVKTALPYTPPHGLCGEVRQLIDLDVWIQDGSGGSFQKNVDF
RLNKIPNKLKCCVIENTFPDWPSPPYYFSVRTSADTGPTYDGVDIRLMNHINDEITDLENC
KEKRRMQVTNTILNPESILLQQRVAYPYHSLRYSYFVQRAGAYPRWTWFTAVFNKILWM
IFFVTLGLIAVVMRWISGSRLLVDSSCYKNIIYCLLNAWSVFLGQGANNVPENVKFRIVFLT
WIIFTLCVSTVFQTFVTSFFVFLVKEHQIDSPEELPAEEYTVVYDSVSKILTLYDESVPSICIQ
SVKDAFQYVINTPKTALYSTEEFFKYNERKWCKNEDIPRYHKFKYYEDSNNFNGLLFNLD
AVLQDKMDRVLQKLATGGIIQKIFNDVVDPSGRNILSETYSELEYEPMELGYLQSCFYSLIC
GLSLSLIVFCFEIFLKWRCS
>BgerIr815NP
VILLLLVQGIKLPSGKNEDVIVNCVLQATIKFSQHGSPVAVVPQIIDTIDDDDDSDKVYKTQ
NITFDTDMKCVQQPTKLEGNLLSKYYDVSKFKIESICILSLSYIAERSLGKLHSSEVWSLFVA
SSRNIVPLNTFKSYIIFVPDEEFVEYLILWCVLLNTDGKFVLVFSKAANENIHALDIVFELEF
ACYKDVLILIWDRNSHRVYVKTALPYTPPYGLCGKVRQLINLDVWIQDGNEGSFQRNVDF
RLNKIPHKLKCCEIKNTYMHFFAPPYNFIGSTSQNGRNYDGVDIRIIAHINDEITDIENCDE
QSTIFVHNTILIRASTLVDQKVAYPYHSLRYSYFVLRAGTNPRWIWFTAVFNKILWMIFFV
TLGLIAVVMRWMSSSRLILHSCYYKNTIYCLLIAWSVFLGQGANNIPENMKFRIVFLTWIIF
TLCVSTVLQSYVTSFFVFQVKEHQMDSPEELSDEQYTVIYDSLLKMLAFYNDSVPSICILSF
KHAFQYAVNTPKTALYSTEEFFKYNERNWCKNDEIPRYHKFKHYEDSNNFHGLLFNVDG
VLQYKMDHVLQKLATGGIIZKIFNDVVDPSGRNILSERYSELEYEPMELGYLQSCFYCLFCG
LSVSLIVFCLEIGLKGRCVYF
>BgerIr816NP
NYSILEKNPTAEPGSNPDTNEQQSALPSESSVLQSTIKFFQHGYALAVVPQIIHANETTYSD
RMLTSENITLDTDMICVQEPTELQGDRVFKYLDVSNFENHAVYLLGWTHIAEKSLGRLHS
SEFWSMFLASSRNIVPLNTFKSYIIFVPDEEYLLYLTRWCNVLNRGAKFIZVFSNIAEEITYV
IGVLFEIEFLCYRDVLILIWDRNSDRVSVKTALPYTPPYGLCGMVRQLIDLDVWIQDGSGGS

FQKNIDFRLNKIPNKLKCCVIENSFPDWPSPPYYFSVNTSADTGPTYDGVDIRLLNHINDEI
TDLENCKEKRRMQVTNNILNPASSLLEQRVAYPYHSLRYSYFVQRAGTYPRWTWFTAVF
NKILWVTFFVTLGLVAAVMRWISGSRLLLDSRCYQNIIYCLLNAWSVFLGQGANKIPENIK
FRIVFLTWIIFTLCVSTVFQTFVTSFFVFLVKEHQIDSPEELSAEEYTVVYESVSNMLTLYDD
TLPSICIESVKHAFQYAVNTPRTALYSTEEFFKYNERKWCKNDEISRYHKFKYYEDSNNFN
GLLFNLDGLLQDKINHVLQKLAIGGIVHKIFNDVVDPSGRNILSETYSELEYEPMELVYLQS
CFYSLICGLSLSLIVFCLEIFLKRRCRYF
>BgerIr817NP
VTWLLLVQGIKLPFDENEDTIASCVLESSIKFFQHGSPVSXVPQILHAIETTDSDRMFTNEN
ITFDTVMKCVHEPTELZEVRVSKYLDVSKFENHAVYLLGWSHVTEKSLGRLHPSELWPLF
AASIRNIVPLNTFKSYIIFVPDDEYLVHLTRWCDALNRGAKFILVFSNTADENIHALDILFX
VCFKDVLILIWDRNSDRVYVKTALPYTPLCGLCGMIRQLIDIDVWIQDGSGGSFMKNVDFR
LNKIPNKLKCCVIENSFPDLYSPPHYFRESTSSXTIFQTFVTSFFVFQVKKHQIDSPEELPVE
EYTVIYDSLLKMLAFYNESVPSISIESIKHAFQYAINTPKTALYSTEEFFKYNERKWYKNDEI
PRYHKFKYYEDLNNFNGLLFDMDVLLQDIMNHVLQMLATGGMIKKXLKKIFNNPSGRNIL
LETYSELEZEPMELVYLQSWKMKGELG
>BgerIr818N
VTSLLLVQGIKLPFDENEDTIARCVLESSIKFFKHGSTVAVVPQIIHANETNYSDRMLTPENI
TFDTDMKCVQEPTELLGDRVFKYLDVTKFENHAVYLLGWSHIAEKSLGRLHSSEFWSMF
LASSRNIVPLNTFKSYIIFVPDEEYLLYLKHWCVVLNRGAKFILVFSNIAEENTYVIGEFFEIE
FACYKDALILIWDRNSDRVYVKTILPYSPPYGLCGVAHQLIDLDVWIQDGSGGSFQKNVDF
HLNKIPTKLKCCVIENSFPDLFSPPYYFSVNTSSDTGPTYDGVDIRLLNHINDEIADIENCKQ
LGRIQVTNTILNPASTLLEQKVAYPYHSLRYSYFVQRAGTYPRWTWFTTVFNKFLWVTFF
VTLALIAVVMRWISSSRQILHSCYYTNIIYCLLNAWSVFLGQGANNIPENIKFRIVFLTWIIF
TLCVSTVFQTFVTSFFVFQVKEHQIDTPEELSAEEYTVVYDSVSKILTLYDETLPSISIPSVK
HAFKYAINTPRTALYSTEEFFKYNERKWCKNDEIARYHKFKYYEDSNNFNGLLLIIDHVLQ
DKINRVLQKLATGGIIYKIFDDVVDPSGRDILSETYSDLEYEPMELGYLQSCFYSLICGLSLSL
IVFCFEVFLKRRCRYF
>BgerIr819NP
MTTLLLVQGIKLPSYENEDIIVNCVLESSIKIFQHGSPVAVVPQIIDGMETTDSETMFATHNI
TFDTDXKCMQEPTILEGNRLAKYFGVSEFENYAIYFHNWSHIAERSLRKLHCSEFWSMFL
ASSRSIVPLNTFKSYIVFVPDDEFLINIQDWCILLNRGAKFILVFSNTADENIHALDILIEVEF
ACYKDVLILIWDRNSDRVYVKTALPYTPPHGLCGEVRQLIDLDVWIQDGSGGSFQENVDF
QLNKIPNKLKCCVIKDTFTNWFSPPFYESTSEDDTTYDGVDIRLLNHINDEIADLENCEEK
EFMHVTNTILNPASSLLEQRVAYPYHSLRYSYFVQRAGTYPRWTWFTAVFNKILWMTFF
VTLGLIAVVMRWISSSRLILDSGCYKNIIYCLLNAWSVFLGQGANNIPENIKFRIVFLSWIIF
TLCVSTVFQTFVTSFXVFQVKEHQIDSPEELPGEEYTVVHDSVSKILTLYDKSVPSICIMPFK
HAFQYAVNTPKTAMYSTEEFFKYNERKWCKNDEIPRYHKFKNYEDSNNYNDLLFNMNG
VLQDKMNRVLQKLATGGIIQKIFNDVVDPSGRKILSEKYSELDYEPMELGYFQSCFYSLICG
LSLSFIVFCLEVVLKGRCRYF
>BgerIr820N
VTTLLLVQGIKLPFDENEDTIASCVLQSSIKFFQHGSPVAVVPQIIHANETTYTDRMLTPEN
ITLDTDKKCVQEPIEFQGDRVFIYLDVSKFENHAIYFLGWSHIAEKSLGRLHSSEFWTLFLA
SSRNIVPLNTFKSYIIFVPDEEYLVYLTRWCEALNRGAKFILVFSNSAEENTYVMGIFFEIEF
LCYRDVLILIWDTNSDRVYVKTVLPYTPPYGLCGEVRQLIDLDVWVQDGSGGSFQRNSDF
RLNKIPNKLKCCVIANTFPDWPSPPYHFSVNTSAGIGPIYDGVDIRLLKHINDEITDLENCE

EKRRLQVTNTITTVTSSLLEQKKVAYPYHSLRYSYFVQRAGTYPRWTWFTAVFNQVLWM
IFFVTLGLIAVVMRWISDSRLLVDSSCYKNIIYCLLNAWSVFLGQGANNIPENIKFRIVFLT
WIIFTLCVSTVFQTFVTSFFVFQVKEHQIDTPEELPDEEYTVVYDSVSKILTLYEESVPSTCI
ESVKHAFQYAVNTPKTALYSTEEFFKYNERKWCKNDEIPRYHKFKYYEDSNNFNGLLFNL
DALLQDKMDRVLQKLAIGGIVHKIFNDVVDPSGRNILSEISSELEYEPMRLGYLQSCFYSLIC
GLSLSLIVFCFEIVLKWRCR
>BgerIr821NP
VTWLLLVQGIKLPSYENEDVIINCVLESSIKFFQHGSTVAVVPQIKGDMETTDSDEMFKTQ
NIXTFDTDKKCVQESTMLEGNRLIKYFDVSEFENHVIFYLRWSHLAERTLRRLHSSEFWPL
FLASSRNIVLLNTCKSYIIFVPDLEYLGQLPHWCLLLKRGAKFILVFSNTAAENIHALDILFE
LEFVCYKDVLILLWDRNSVRVYVKTALPYTPPYGLCGMVRQLIDLYVWIQDGSGGSFQEN
VDFQLNKIPNKLKCSIIQDSFINGFSPPFYESISEDDTTFDGVDIRLLKHINDAITDLENSEEK
VRIFVTNTIQNPASTLLEQRVVYPYHSLRYSYFVQRAGTYPRWTWFTGVFNKTLZMTFFV
TLVLIAVVMRWIYSSRLVFHSCZYKNFNYCLLNAWSVFLGQGANNIPENIKFRIVFLTWIIF
TLCVSTVFQTFVTSFFVFQVKEHQIDTPEEFPGEEYTVVYDSVSKVLTLYDKYVSSICILPFK
HAFQYVLNTPKTALYSTEEFFKYNERKWCKNDEIPRYHKFKSYEDSNNFNGFLFNIDSXL
PEKINRVLQKLATGGIIXKIFNDVVDPSGRNILSEISSELEYEPMELXAHGTCFYSWICGLSL
SFVVFCLGVVFKERCRHF
>BgerIr822N
VTSFLLVQGFNLPSEENENVIVNCILESSIKFFEHGHPVAVVPQMIDAIETSDSETMFTPENI
TFYADKKCVQQPIELEGSRLFKYFDFLKFENHVKYVHSWSFISERTLRKLHSSEFWSLFLA
SSRNIVPLNTYKSYIIFVPEVEYLIHLPFWCIIVNKAAKFMLVFSSTEEENKRTLDLIYEYYF
MCYKDFLILIWDRDSDRVHVKTALPYTPPHGLCGAVRQIIDLDVWIQDGSGGSFEINSDFR
LDKIPHKLQCCKIDEAMTSLHSPPYYFLESTSPKYGPKFDGVDIRVLSHVNDAITINENCSD
KDIIFVTNTLLSVANSLFHQVLAYPYHSLRYSYFVPRAGTYSQWNWFTSVFSELLWASFFV
ALGSVAFVMRWISSSGFECRPGCHKDVIYCLLNAWSILLGLGANNIPENIKFRIVFLSWIVF
TLCVSTVFQTFVTSFFVLQVKKHQMDSPEELPAEDYTVVYDSPLKMLAFYDENVPSICFRS
FKQAFQYAVNTPKTALYSTEEFFKYNERKWCQSDEIPRYHKFKHYEDSNNYNGLLFNMD
GVLQDKMDRVLQKLATGGMIKKIFNDVVDPSGRNILSETYSVLEYEPMERGYLQSCFYSLI
CGLSLSLIVFCLEVVLKGRCRYC
>BgerIr823N
VTSLLLVQGIKLPFDENEETIASCVVESSIKFFQNGSPVAVVPQITHANETTYSDRMFTPEN
ITLDTDMKCVQEPTELQGDRVSKYLDVSNFENLAVYFLGWSHIAEKSLGRLHSSEFWPLF
VASSRNIVPLNTFKSYIIFIPDDEYLLYLTRWCDVLNRGAKFILVFSNIPEENTYVLGVFFEIE
FLCYRDVLILIWNRNSDRVYVKTTLPYSPPYGLCGVARQLIDLDTWIQDMNGGSFQKNAD
FRLNKIPHKLKCCVIKNSFPDFFSPPYYFPLSTSADTGPTYDGVDIRLLKHINDEITDLENCK
QVGRMQVTNTILNPTSTLLEQKVAYPYHSLRYSYFVQRAGTYPRWTWFTAVFNTFLWM
TFFVTLGLVAVVMRWISSSRLILHSCYYKNIIYCLLNASSVFLGQGANNIPVNINFRIVFLTW
IIFTLCVSTVFQTFVTSFFVFLVKEHQIDSPEELPTEEYTVVYESVSKILTLYEESVPSICIESV
KHAFQYAVNTPKTALHSTEEFFKYNERKWCKNDEIPRYHKFKYYEDSNNFNGLLFNMDA
LLQEKMDHVLQKLATGGIIQKIFNDVVDPSGRNILSEISSELEYEPMELGYLQSCFYSLICGL
SLSLIVFCFEIVLKRRCR
>BgerIr824N
LEQKVAYPYHSLRYSYFVQRAGTYPRWTWFTAVFNNILWMIFFVTLGLIAVVIRWISDSR
LLVDSSCYKNIVYCLLNAWSVFLGQGANNIPENIKFCIVFLTWIIFTLCVSTVFQTFVTSFFV
FLVKEHQIDSPEELPTEEYTVVYESVSKILTLYEESVPSICIESVKHAFQYAVNTPKTALHST

EEFFKYNERKWCKNDEIPRYHKFKYYEDSNNFNGLLFNMDALLQEKMDHVLQKLATGGI
IQKIFNDVVDPSGRNILSEISSELEYEPMELGYLQSCFYSLICGLSLSLIVFCFEIVLKRRCR
>BgerIr825CP
MTTLLLVQGIKLPSYENEDVIVNCVLESSIKFFPHGSPVAVVPHIMDAVNVDDSEAMFTLQ
NITLDTGKKCVKGTTNLEGNRLFKYFDVSKFENHAIYFLSWLHIAERSLVKLHSSEFWSMF
LASSRNIVPLNNFKSYIVFVPDEEZLIYLPHWCILLNRGAKFILVFSNNAEQNRYAIDILFQL
EFVCYKDVIILIWDRNSDRVHVKTALPYRPPYGYCGIIRQLIDLDVWIQNANGVSFQINADL
RLNKIPNKLKCCIIEDTFTNGFLAPYYFPVSTSPDTZTTYDGVDIRLLKHINDKITDLENCEE
KMGIFVTNSIMKTLSSLLEQKVAYPYHSLKYSYFVQRAGTYPRWTWFTAVFNKILWMIFF
VTLGLIAVVMRWIIYCLLNAWSVFLGQG
>BgerIr826N
VTSLLLVQGIKLPFDENEEVIVNCVLESSIKFFQHGSPVAVVPQIINTIETTYTDRMFTPENI
TLDTDMKCVQKPTELQGDRVFKYLDVSKFETHAIFFLSWSHIAEKSLGRLHSSEFWALFL
ASSRNIVPLNTFKSYIIFVPEDEYLVYLTRWCEALNRGAKFILVFSNIAEENTYLIGVFFEIEF
LCYRDVLILIWDRNSDRVYVKTTLPYSPPYGLCGEVRQLIDLDVWIQDGSGGSFQRNVDFR
LNKIPNKLKCCVFANSFPHLFSPPYYFLVSTSADTGPTYDGVDIRLLNHINDEITDLENCKQ
VRRMQVTNTILNPVSTLLEQKEPYPYHSLRYSYFVQRAGIYPRWTWFTTVFNTFLWMIFF
VTLGLIAVVMRWISSSRLILHSCSYTNTIYCLLNAWSVFLGQGANNIPENLKFRIVFLTWIIF
TLCVSTVFQTFVTSFFVFQVKEHQMDTPEELSGEEYTVVYDSIYKVLTLYDESLLSLSITSVI
HAFQYAVNTPKTALYSTEEFFKYNERKWCENDEIPRYHKFKYYEDSNNFNGLLLIIDNVL
QNKMNRVLQKLATGGIIHKIFNDVVDPSGRNILSEKYSELEYEPMELVFLQSCFYSLICGLS
LSLIVCCFEIFLKWRGR
>BgerIr827NP
VISLWLVQGIKLPADKNEDVIINCVLESSIKFFQHGSPVAVVPQITDGIDINDTDIMYKTQNI
IFDTDMKCIQQPTELEGKPVFKYFDVSNFENHGFSILSZTHIDERSLGRLHSSEFWPLFLAS
SRNIVLLNAFKSYIIFVIDQEYLGYLANWCMLLNQGAKFILVFSSTAKENLFAIDMLFEIAFA
CYKDSLILLWGRNSDRVSVKTALLYTPPYGLCGMVRQLIDLDIWIQDAKGGSFQRNADFR
LNKIPNKLKCCVIVNTFAKWFSPPYHFVESTSEDGPTYDGVDIRLIAHINDAITDLQNCKEK
YRIFVHNTIVTTTSTLFDQKLSYPYHSLRYSYFVQRAGTYPRWTWFTAVFKKILWMTFFV
TLGLIAVVMRWISSSRLILESCCYKNIVYCLLNAWSVFLGQGVNNIPDNIKFRIVFLTWIIFT
LCVSTVFQTFVTSFSVFLVKEHQIDSPEELPGEEYTVVYDSVSKILTLYNESVPSICIRSIKNA
FQYAVNTPKTALYSTEEYFKYNERKWCKNNEIPRYHKFKHYEDSNNFNGLLFDMDGLLQ
EKMDRVLQKLATGGIIQKIFNDVVDPSGRNILSEISSELEYVPMELGYLLSCFYCLFRZASLY
FVWRLVLKRCGYF
>BgerIr828NP
VTSLLLVQGIKLPFDENEDVIASCVLESSIKFFQHGSPVAIVPQIIYANETTYSDRMLMPENI
TLHTDMKCVQEPTDLQGDRVFKYLDVSKFENHAIYLLGWSHIAEKSLGRLHSSEFWPLFV
ASSRNIVPLNTFKSYIIFVPDEEYLVYLTRWCGALNRGAKFILVFSNNAQENTYIMGIFLEIE
FLCYRDVLILIWDTNSDRVYVKTALPYTPPYGFCGEVHQLIDLDVWIQDGSGGSFQKNVDF
RLNKIPSKLKCCKIENSFPDWPAPPYHFSVNTSAYTGPIYDGVDIRLLKHINDKITDLENCE
EKRILQVTNTITTVTSSLLEQKVAYPYHSLRYSYFVQRAGTYPRWTWFTAVFNNILWMIF
FVTLGLIAIVMRWISDSRLLVDSSCYKNIIYCLLNAWSVFLGQGANNIPENIKFRIVFLTWII
FTLCVSTVFQTFVTSFFVFLVKEHQIDSPEELPDEEHTVVYDSVSKILTLYEESVPSICIESVK
HAFQYAVNTPKTALYSTEEFFKYNERKWCKNDEIPRYHKFKYYZDSNNFNGLLFNLDALL
QDKLDRVLQKLATGGIVHKIINDVVYPSGRNILSEISSELEYEPMELGYLQSCFYSLICGLSLS
LIVFCFEIVLKQRCR

>BgerIr829NP
VTLLLLVQGIKLPSNENSILESSIKFFQNESPVAVVPQIIHETTYSDRMLTPENITLDTDMKC
VQEPTELQGDRVFKYLDVSKFENLAVYLLGWSHIADKSLGRLHSSEFWPLFVASSRNIFSL
NTFKSYIIFVPEEEYLLYLTRWCETLNRGAKFILVFSNTAEENTYVIDVFFQIEFLFYRDVLI
LIZDTNSDRVYVKTAXPYTPPYGLCGRVLRLIDLDVWFQDGCGKSFQKNVDFRLNKIPNKL
KCCVIASTFLDWPSPPYYFPVSTSADTZPNYDGXDIRLLHHINDEIADLENCKDKVRIFVTN
TILNPGSGLLEQKVAYPYHSLRYSYFVQRAGTYPRWTWFSTVFTKFLWMIFFVTVGLIAVV
MRWISGSRLILHSCYYKNIIYCLLNAWSVFLGQGANNIPENMKFRIVFLTWIIFTLCVSTVF
QTFVTSFFRFXQTVLYSTEZYFKYNERNWCENDEILRYHKFKYYEDSNNFNGLLLIIDNVL
QNKMNRVLQKLATGGIIHKIFNDVVDPSGRNILSEKYSELEYEPMELVFLQSCFYSLICGLS
LSLIVFCLEIFLKWRCR
>BgerIr830NP
MTTLLLVQGIKLPSYENEDIIVNCVLESSIKFFQHGSPVAVVPHIMDAFDVDDSEAMFTLQ
NITLDTGKKCVQGTTDLEGNRLFKYFDVSKFENHAIYFLSWLHIAERSLVKLHSSEFWPIF
LASSRNIVPLNTFKSYIVFVPDEEYLIYLPHCCILLNRGAKFILVFSNTAEQNRYAIDILFELE
FVCYKDVIILIWVRNSDRVHVKTALPYRPPYGHCGIVRQLIDLDVWIQNANGGSFQINADL
RLNKIPNKLKCCVIEDTFTNGFSAPYYFPVSTSAVTGPTYDGVDIRLLNHINDKITDLENCE
EKMRIFVTTSIMKTSSSLLEQRVAYPYHSLXYSYFVQRAGTYPRWIWFTAVFNKILWMIFF
VTLGLIAVVMRWISSSRLILDSSCYTNTIYCLLNAWSVFLGQGANNIPENIKFRIVFLTWIIF
TLCVSTVFQTFVTSFFVFQVKEHQIDTPEELPGEEYTVVYDSLSKILTFYDEGVRSISIQPFK
NAFQYAVNTPKTALYSTEEFFKYNERNWCKNDEIPRYHKFKHYEDSNNYNGLLFNIDGVL
QDKMDRILQKLATGGIIQKIFNDVVDPSGRRVLSEISSVLEYEPMELVYLQSCFYSLICGLSL
SLIVFCLEIVLNRR
>BgerIr831NP
VTSLLLVHGIKLPLDENZDVIVNCVLEASIQFFQHGSPVAVVPQIIHAIETTDTDRVFTPENI
ALYTDMKCVQEPTELQGDRVFKYLDVSKFENHAVYFLGWSHIAEKSLGRLHSSDFPLFXF
LASSRNIVPLNTFKSYVIFVPDYEYLLYLTRWCDALNGGAKFILVFSNSAEENTYATNILFE
FTCYKDVLILIWDRDSDRVYVKTAYPYTPPYGLCGMVDQLIDLDVWIQDGTGGSFQRNVD
FRLNKIPHKLKCCMIKNSFPDFFSPPYYFPVNTSADAGPTYDGVDIRLLKHINDEIADLENC
KQVERMQVTNTILHPASTLLEQKVAYPYHSLRYSYFVQRAGTYPRWTWFRTVFNNILWM
TFFVTLGLIAVIMRWISSSRLLLDSSCYKNVIYCLLNAWSVFLGQGANNIPENIKFRIVFLT
WIIFTLCVSTVFQTFVTSFFVFQVKEHQIDTPEELPDEEYTVVYDSVSKILTLYDESVSSICID
SVKHAFQYAVNAPKTALYSTEEYFKYNERKWCKNDEIPPYHKFKHYEDSNNFNGLLFDM
DALLQDKVNRVLQKLATGWIIQKIFNDVVDPSGRNILSETYSELEYEPMELVYLQSCFYSLI
CGLSLSLIVCCFEIFLKRR
>BgerIr832N
MTTLLLVQGIKLPSYENEDIIVNCVLESSIKFFQHGSPVAVVPQIIDGMETTDSETMFATHN
ITFDADKKCVQEPTILEGNLLSKYFDVSKFENYVIYFHNWSHIAERSLVKLHSSEVWSMFL
ASSRNIVPLNTFKSYIVFVPDAEFLNNIQHWCILLNRGAKFILVFSNTADENINALDILFELE
FACYKDVLILLWDRNSDRVYVKTALPYTPPHGLCGIVRQLIDLDVWIQDGNGGSFQKNVD
FQLNKIPNKLKCCVIQDSLINWFSPPFYESISEDDTTYDGVDIRLLNHINDEMSDLENCQVK
ERMHVTNTILNPASRLLEQRVAYPYHSLRYSYFVQRAGTYPRWTWFTAVFNKILWMTFF
VTLGLIAVVMRWISTSRLILDSGCYKNIIYCLLNAWSVFLGQGANNIPENIKFRIVFLTWIIF
TLCVSTVFQTFVTSFFVFQVKEHQIDSPEELPGEEYTVVYDSVSKILTLYDKSVPSICIMPFK
HAFQYAVNTPKTALYSTEECFKYNERKWCKNDEIPRYHKFKNYEDSNNYNGLLFNMNGV

LQYKMDRVLQKLATGGIIQKIFNDVVDPSGRKILSEKYSELDYEPMELGYLQSCFYSLICGLS
LSFIVFCLEVVLKGRCRYF
>BgerIr833NP
DFLILIZDRNSDRVHVKTALPYTPPHGLSGAVCQLIDLDVWIQDGRGGSFEINSDFRLDKIP
HKLQCCKIDESLTSLHSPPYYFLESTSSLKYGPIFDGVDIRVLSHVNDAITINENCSDKDTVF
VTNTLLSVANSLFHQVLAYPYHSLRYSYFVPRPGTFSQWNWFTSVFNELLWASFFVALGL
VALVMRWISRSGLECRPGCYKDVIYCLLNAWSILLGLGANNVPDNIKFRIVFITWIVFTLCV
STVFQTFVTSFFVLKVKKHQVDAPEELSGEDYTVVYDSPLKMLAFYDENVPSICFRSFKQA
FQYAVNTPZTALYSTEEFFKYNERKWCQSDEIPRYHKIKHYEDSNNYNGLLFNMDGVLQD
NIDRVLQKLATGGMIQKIFNDVVDPSGRRILSEISSELEYEPMELAYLQSCFYSLICGLSLSLI
VFCLEVVLIGKCRYF
>BgerIr834N
VTSMLTVQDIKLPFHENEDTIANCVLESTIKFFQRGYPVAVVPQIIHAIDKTDSERMFTPEN
STLDTDNKCVPESTEIQNERVFKYLDISKFETHAIFFLSWSHIAEKSLGTLHSSEFWPLFVA
SSRNIVPLNTFKSYIIFIPDEEYLVHLIRWCDALNRGAKFIFVFSNSAEENTYAINILFEFTCY
KDVLILIWDRNSDRVYVKTALPYTPPHGLCGEVLQLIDLDVWIQDGSGGSFQRNADFRLN
KIPRKLKCCVIENSFSDRFSPPYYFLLSTFADTGPTYDGVDIRLLNHINDEITDLENCDEVER
MQVINTIPTVTSSLLEQKVAYPYHSLRYSYFVQRAGTYPRWTWFTSVFNISLWVIFFVTLG
LIAVVMRWISSSRLLLDSSCYKNVIYCLLNAWSVFLGQGANNIPENIRFRMVFLTWIIFTLC
VSTVFQTFVTSFFVFQVKEHQIDTPEELLDEQYTIIYDSQLKILALYNESVPSICIQSVKHAF
QYAVNTPKTALYSTEEYFKYNERKWCENDEIPRYHKFKHYEDSNNFNGLIFNMDALLQD
KMNRVLQKLATGGIVHKIFNDVVDPSGRNILSETYSELEYEPMELGYLQSCFYSLICGLSISL
IVFCLEIALKGRCCWF
>BgerIr835NP
MTTLLLVQGIKLPSYENEDIIVNCVLESSIKFFQHGSPVAVVPQIIDGMETTDSETMFATHN
ITFDTDTKCMQEPTILEGNRLSKYFDVSEFENYAIYFHNWSHIAERSLWNLHCSEFWSMF
LASSRSIVPLNTFKSYIVFVPDDEFLINIQDWCILLNRGAKFILVFSNTADENIHALDILIEVE
FACYKDVLILIWDRNSDRVSVKTALPYTPPHGLCGEVRQLIDLDVWIQDGSGGSFQENVDF
QLNKIPNKLKCCVIKDTFTNWFSPPFYESTSEDDTTYDGVDIRLLNHINDEIADLENCEEK
EFMHVTNTILNPASSLLEQRVAYPYHSLRYSYFVQRAGTYPRWTWFTAVFNKILWMTFF
VTLGLIAVVMRWISSSRLILDSGCYKNIIYCLLNAWSVFLGQGANNIPENIKFRIVFLTWIIF
TLCVSTVFQTFVTSFFVFQVKEHQIDSPEELPGEEYTVVYDSVSKILTLYDKSVPSICIMPFK
HAFQYAVNTPKTALYSTEEFFKYNEXKFKNYEDSNNYNGLLFNMNGVLQDKMNRVLQKZ
ATGGIIQKIFNDVVDPSGRKILSEKYSATGGIIQKIFNDVVDPSGRKILSEKYSCKYF
>BgerIr836NP
VTSFLLVQGFNLPSEENEDVLVNCVLQSSIKFFQHGHPVAVVPQMIDAIETSDSETMFTPE
XNADKKCVQQPIELEGSRLFKYFDFLKFENHVKYLHSWSFISERTLRKLHSSEFWSLFLAS
SRNIVPLNTYKSYIIFVPEVEYLIHLPFWCIIVNKAAKFMLVFSSTEEENKRTLDLIYEYYFM
CYKDFLILIWDRDSDRVHVKTALPYTPPHGLCGAVRQIIDLDVWIQDGRGGSFEINSDFRL
DKIPHKLQCCXHLFLESTSPKYGPKFDGVDIRVLSHVNDAITINEDCSDKDIIFVTNTLLSVA
NSLFHQVLAYPYHSLRYSYFVPRAGTYSQWNWFTSVFSELLWASFFVALGSVAFVMRWIS
SHGFECRPGCHKDVIYCLLNAWSILLGLGANNIPENIKFRIVFLSWIVFTLCVSTVFQTFVTS
FFVLQVKKHQMDSPEELPAEDYTVFYVSPLKMLAFYDENVPSICFRSFKQAFQYAVNTPK
TALYSTEEFFKYNERKWCQSDEIPRYHKFKHYEDSNNYNGLLFNMDGVLQDKMDRVLQK
LATGGMIQKIFNDVVDPSGRNILSETYSELEYEPMELGYLQSCFYSFICGLSLSLIVFCLEVVL
KGRCRY

>BgerIr837NP
YTGCPNKIPNKLKCCVIENSFSDWFSPPYYFSLSTSADTGPTYDGVDIRLLNHINDEIMDLE
NCEEKRIMQVTNTILNPASTLVEQKVAYPYHSLRYFYFVQKAGTYPRWTWFTAVFNKIL
WVTFFVTLGLVAAVMRWISGSRLLLDSSCYKNIIYCLLNAWSVFLGQGANNIPENIKFRIVF
LTWIIFTLCVSTVFQTFVTSFFVFLVKEHQIDSPEELPAEEYTVVYDSVSKMLTLYDETLPSI
CIQSVIHAFQYAINTPRTALYSTEEFFKYNERKWCKNDKIPRYHKFKYYEDSNNFNGLLFN
LDDLLQEKINRVLQKLATGGIVHKIFNDVVDPSGRNILSEKYSELEYEPMELGYLQSCFYSLI
CGLSLSLIVCCFEIFLKRR
>BgerIr838NP
VTSMLRVQGIKLPFDENEDTIANCVLESTIKFFQRGYPVAVVPQIIHAIDKTDSERMFTPEN
STLDTDNNCVPESIEFQKERVFQYLDVSKFETHAIFFLSWSHIAENSLGRLHSSEFWPLFV
ASSRNIVPLNTFKSYIIFIPDEEYLVYLIRWCDALNRGAKFIFVFSNSAEENTYATNILFEFTC
YKDVLILIWDRNSDRVYVKTSLPYTPPHGLCGEVLQLIDLDVWIEDGSGGSFQRNADFRLN
KIPRKLKCCVIENSFSDRFSPPYYFLLSTSADTGPTYDGIDIRLLNHINDEIADLENCDEVER
MQVINTITTVTSSLLEQKMAYPYHSLRYSYFVQRAGTYPRWTWFTAVFNVSLWVIFFVTL
GLIAVVMRWISSSRLLLDSSCYKNVIYCLLNAWSVFLGQGVNNIPENMRFRIVFLTWIIFTL
CVSTVFQTFVTSFFVFQVKEHQIDTPEELLDEQYTIIYDSQLKILALYNESVPSICIQSVKHAF
QYAVNTPKTALYSTEEYFKYNERKWCENDEIPRYHKFKHYEDSNNFNGLIFNMDSLLSLX
KMNRVLQKLATGGIIQKIFNDVLDPSGRNILTENYSELEYEPMELGYLQSCFYSLISGLSISLI
VFCLEIALKGRC
>BgerIr839NP
MTTLLLVQGIKLPSYENEDVIVNCVLESSIKFFPHGSPVAVVPHIMDAVNVDDSEAMFTLQ
NITLDTGKKCVQGTTDLEGNRLFKYFDVSKFENHAIYFLSWLHIAERSLVKLHSSEFWSMF
LASSRNIVPLNTFKSYILFVPDEEYLIYLPHWCILLNRGAKFILVFSNTAEQNRYAIDILFELE
FVCYKDVIILIWDRNTDRVHVKTALPYIPPYGHCGIVRQLIDLDVWIQNANGGSFQINADL
RLNKIPNKFKCCVIEDTFTNGFSAPYNFPVSTSADTGXTYDGVDIRLLSHINDKITDLENCE
EKXSSSLTLZZRRQVVYWNPYHSLRYSYSYPRWTWFTAVFNKILWVIFFVTLGLIAVVMR
WISSSRLILDSSCYSNTIYCLLNAWSVFLGQGANKIPENIKFRIVFLTWIIFTLCVSTVFQTFV
TSFFVFQVKELSLIHI
>BgerIr840NP
VYKRQIYFLSWLHIAERSLVKLHSSEFWPIFLASSRNIVPLNTFKSYIVFVPDEEYLIYLPHW
CILLNRGAKFILVFSNTAEQNRYAIDILFELEFVCYKDVIILIWDRNTDRVHVKTALLYIPPY
GHCGIVRQLIDLDVWIQNANGGSFQINADLRLNKIPNKFKCCVIEDTFTNGFSAPYNFPVS
TSADTGXTYDGVDIRLLSHINDKITDLENCEEKXSSSLTLZZRRQVVYWNPYHSLRYSYSYP
RWTWFTAVFNKILWVIFFVTLGLIAVVMRWISSSRLILDSSCYSNTIYCLLNAWSVFLGQG
ANNIPENIKFRIVFLTWIIFTLCVSTVFQTFVTSFFVFQVKEHQIDTPEELPGEEYTVVYDSL
SKILTFYDEGVRSISIQPFKNAFQYAVNTPKTALYSTEEFFKYNERNWCKNDEIPRYHKFK
YYEDSNNFNGLLFNIDGVLQDKMDRILQKLATGGIIQKIFNDVVDPSGRKILSETYSELEYD
PMELGYLQSCFYSLICGLSLSLIVFCLEIVLNRR
>BgerIr841N
VTSLLLVQSIKLPSEVNEDVVVNCVLQSSFKFFQHGSPVAVVPQIIDAIDINDSLKMFTPQNI
TFGIDKKCVQDPTKLEGNRLFKNFDISKFENHAIFFHSWSHIAERSLGKLHSSEFWPLFLA
SSRNIVPLNTFKSYIIFVPDDEFLIYMQHWSILLNKGAKFILVFSNTAEENAHALDILFTLEF
ACYKDNLILIWDKNSDSVCVKTALPYTPPYGVCGIILQLVDLDTWIQDGNGGFFQRNADFR
LNKIPNKLKCCVIEDTYHDDSPPYYFFESTTDYGPTYDGVDIRLIAHINDEITDLENCEEKV
RIYVYNTIMTMASSILEQKVAYPYHSLRYSYFVQRAGTYPRWIWFTAVFNKILWMTFFVT

LGMIAVVMRWISSSRLLLDSNCYKNIIYCLLNAWSVFLGQGANNIPENIKFRIVFLTWIVFT
LCVSTVFQTFVTSFFVFQVKEHQVDTPEELSGEEYTVVYDSLSKILTFYDESVPSICIRPFKH
AFQYAVNTPKTALYSTEEFFKYNEKKWCQSDEIPRYHKFKHYEDSNNYNGLLFNVDGVL
QDKMDHILQKLATGGMIKKIFNDVVDPTGRRILSEISSVLEYEPMELGYLQSCFYALICGHS
LSLIVFCLEIVLKGRCRYF
>BgerIr842NP
VTSLLLVQGIKLPSYENEDVIINCVLEASIKFLQHGYPVAVVPQIIHAIDKTDSERMFTLQNI
TLDANKKCVHEPTLLEGNRLSKYSDVSKFENQVVYFLGWSHIDERSLGKLHSSEIWSLFLA
SSRNIVPLNTFKSYXIFVPDEEYLFYLPQWCNIZYRGAKFILVFSNNAEENRFAIDIPFELEF
TCYKDVLILIGDRNSDRVXKTALPYTPPYGLCGMVLQLIDLDVWIQDGNGGSFQRNADFR
PNKIPHKLKCCVIKNSFQDXSPPYYFSVSTSSDTGPTYDGIDIRLLKHTNDEITDLENCEEV
GRIQVTNTILNPASSLLEQKVAYPYHSVRYSYFVQRAGIYPRWTZFTTVFNKFLWKIFFVT
LGLIAVVMRWISSYRLILHSNYYKNIIYCLLNAWSVFLEQGANNIPENIKFRIVFLTWIVFTL
CVSTVFQTFVTSFFVFQVKEHQIDTPEGLPGEEYTVVYDSLFKILTLYDESLLSTSINSVKHA
FQYAVNTPKTALYSTEEHFKYNERKWCKNYEIPRYHXSNNYNELLLIIDNDLQNKINSVLQ
KLATGGIIHKIFNDVDPSGRNILSETYSELDYEPMELGYLQSCFYSLICGLSLSLIVFCSEIFLK
RRCR
>BgerIr843N
VTSLILGQGIKLPSYENEDVLVNCVLESSIKFFQHGHPIAVVPQLIDAIETADSEAMYSAENI
ANFTFDTTRKCVQCSENFEGVSISKYFDLFKFEDHMIFFHNWSHITERSLWKLHNSECWS
IFLASSRNIFPLNTYKSYIIFVPVGEYLVYVADWCIILNRGAKFILVFSNSAEGNRHTIDMLL
ESSFACYKDVLFLIWDRDSDRVHVKTALPYTPPHGLCGLVRQIIDLDVWIQDERGESFQKN
ADFRLHKVPNKLRCCIIENSFSNDLSPPYNFIESTPADSSPTYDGVDIRLLSHINEAMTDLE
NCEEREIIYVYDTILTMANVLVKKPPYPYHSLRYSYFVPKAGTYPRWTWFTAVFSKIFWTS
FFVSLGLMAIVMRWISSSRLYLDSSCYKNIIYCLLNAWSVFLGHGASNIPVNIKFRILFLSWI
VFTLCVSTVFQTFVTSFFVLQVKEHQIDSPPELPDEEYTVVYDSTLKHVTFYDENVQSISFQ
SFKHAFQYAVNTPKTALYSTEEFFKYNERKWCKNNEIPRYHKFKHYEDSNNYNGLLFNID
GLLQDKMDRVLQKLATGGMIKKIFNDVVDPSGRKILSGITFELDYEPMELAYLQSCFYCLL
CGLSLSLIVFALEYVLKGRCRSCLKNTQNILTVSSNAIMGKFFCFE
>BgerIr844NP
VTSLILGQGIKLPSYENEDVLLNCVXLCTSIKFFQHGHPVAVLPQMIDAIETVDSETISGVEK
IANFTFDTTQKCVQCPEHFEGVTILKYFDFLKFENHAIFFHSCSHITERSLWKLHSSELWPL
FLASSRNIFPLNTYKSYVVFVPDEEYMVHLPLWCIILNRGGKFILVFSNIAEQNKRAMDWL
TELEFVCYKDVLILIWDRNSDRVYVKTALPYTLPHGLCGEVRQLIDLDVWIKDAHGGSFQE
HADFRFDKIPNKLKCCVIEDSFSNDYSPPYNFMESTSNDTGPTYDGVDIRLLAHINEAMTD
LENCEERDKIYVSNRILTMANVLEKKPPYPYHSLRYSYFVTKAGTYPRWTWFTAVFSKIF
WGSFFATLGIIAMVMRLMSVSRHLLRTDCYKNIIYCLLNAWSVFLGQGANNIPENVNFRIV
FLTWIIFTICVSTIVFQTFVTSFFVLQVKEHQIDTPPELPDQEYSVVYDSTLKHLIFYDENVR
SIRFRSFKHSFQYAVNTPKTALYSTEEFFKYNERKWCKNNEIPLYHKFKHYEDSNNYNGL
LFNIDGLLQDKMDHVLQKLATGGIVQKIFNDVVDPSGRNILSETYSVLEYKSSELGYLQSCF
YSLICGLSLSLIVFCMEIVLKRRCRYF
>BgerIr845NP
ATSLLLEEGIKLPSYENEDVILNCVLESSIKFFQHGYPVAVVPQMIDVIETTDSETIFTPENIT
YDADKKCVQQPTEFEGNSVPRYFDFWKFESHVMYVHSWSYTSEIALRKLHSSQFWPLFL
ASSRHIFPLNTFKSYIIFVPDLEYLVHLPFWCFVLNKGAKFLLVFSNTAEENERAIYLIFDNE
FICYKDTLIIMWDRXEREIIRVTNTILFATSGILELSAPYPYHSLGYLYFIQTAGTYPRWTWF

TAVFNKILWMTFFVTLGLIVVIMRWISSSRLFLDSSCYKNIIYCLLNAZSVFLGQGANNIPE
NIKFCIVYLTWILFTLCXSTVFQTFVTRFFVFQVKZHQINTPEELPDDDYTVVYDSPFKILTF
YDKDVPSIFFRSFKHAFQYAITTPRPALYSTEEFLKYNEZRWCNTDEIPRYHKFKHYEDSN
NYNGLLFNVDGILEETLGGVLQKLATGGIIKXIFNDVVDPAGRRILSEISSELEYEPMELGYL
QSCFYSLICGLSLSLIVFLLEVALSGRCR
>BgerIr846NP
VISLWLVQGIKLPSGKNEDVILNCVLESSIKFFQHGSPVAVVPQITDGIDINDTDIMYKTQNI
TFVTDMKCVQQPTELERKRVFKYFDVSNFENHGFCILSLTHIDERSLGRLHSSEFWPLFLA
SSRNIVLLNAFKSYIIFVIDQEYLGYLANCCMLLNKGAKFILVFSSTAKENLFAIDMLFEIAF
ACYKDSLILLWGRNSDRVSVKTALLYTPPYGLCGMVRQLIDLDIWIQDAKGGSFQRNADF
RLNKIPNKLKCCVIVNTFAKWFSPPYHFVESTSEDGPTYDGVDIRLIAHINDAITDLQNCKE
KYRIFVHNTIVTTTSTLFDQKLSYPYHSLRYSYFVQRAGTYPRWTWFTAVFKKILWMTFF
VTLGLIAVVMRWISSSRLILESCCYKNIVYCLLNAWSVFLGQGANNIPDNIKFRIVFLTWIIF
TLCVSTVFQTFVTSFFVFQVKEHQIDTPDELPGEEYTVVYDSVSKILTLYNESVPSICIRSIK
NAFQYAVNTPZTALYSTEEYFKYNERKLCKNDEIPRYHKFKHYEDSNNFNGLLFDMDGLL
QEKMDRVLQKLATGGIVQKIFNDVVDPSGRNILSKISSELEYEPMELGYLQSCFYCLICGLSL
SLIVFCLEFGLKGRCLYF
>BgerIr847N
VTSLILLGQGIKLPSYENEDVLVNCVLQSSIKFFQHGHPVAVVPQMIDAIETSDSGAMYSAE
NIANFTFDTTKKCVQSSENFEGVSISKQYDLFKFENHAIFFHSWPHITERSLWKLHTSEFW
SMFLASSRNIFPLNTYKSYVVFVPDEEYLGYLPLWCMILNRGAKFIFVFSNTGEGNRYAID
MLLEINFACYKDVLFLIWDKNSDRVHIKTALPYTPPLALCGEVRQLIDLDVWIQDESGESF
QKNADFHLNKIPNKLRCCIIEYSFSNGFSPPYNFIESTPIDSSPTYDGVDIRLLAHINEAMTD
LGNCQEREIIYVSDTILTMANVLEKKPPYPYHSLRYSYFVPKAGTYPRWIWFTAVFSKIFW
TSFFVSLGLMAIVMRWISSPRLILDSSCYKNIIYCLLNAWSVFLGHGASNIPGNIKFRIVFFS
WIVFTLSVSTVFQTFVTSFFVLQVKEHQFDSPPELPDEEYTVVYDSTLKHLTFYDENVQSIS
FQSFKHAFHYAVNTPKTALYSTEEFFKYNERKWCKNNEIPRYHKFKDYEDSNNYNGLLF
DSHGVLQEKMDRVLQKLATGGIVQKIFNDVVDPSGRKILSGITFELDYEPMELGYLQSCFY
CLLCGLALSLIVFALEYVLKGRCRYF
>BgerIr848N
VTSLLLVQGIKLASDKNEDVILNCVLESSIKFFQHGSPVAVVPQMIDLIDTSDSETMFKTQN
ITLDTDKKCVQEPTEMERHSLFKYLDVSKFENHGKILLSWSHIAERSLWKLHSSEFLPLFL
ASSRNIVPLNTFKSYIIFVPDNENVVLLMYWCILLNKDAKFILVFSKTAAENKFAMNVLFD
FDFMCFKDNLILIWDRNPDRVYVKTAFPYTPSYGRCGRVLQLIDLDVWIQDENGGSFKEN
AEFRLNKIPHKLKCCIIQDTYINDDSPPYYFTERTFEEGPPTYDGVDIRLIAHINDEITDLDN
CEGNMIIYVHNTILTMASTLVDQKVAYPYHSLRYSYFVQRAGSYPRWTWFTAVFNQSLW
MIFFVTLGLIAVVMRWISSSRLILDSTYYKNIFYCLLNAWSVFLGQGANKIPENVKFRIVFL
TWIIFTLCVSTVFQTFVTSFFVFQVKEHQIDTPEELPDEEYTVVYDSISKILTFYDENVRSISI
QPFKHAFQYAVNTPKTALYSTEEFFKFNERNWCKKDEIPRYHKFKYYEDSNNFNGLLFNI
DGVLQNKMDRILQKLATGGIIQKIFNDVVDPSGRKILSEISSELEYEPMELGYLQSCFYALIC
GLSLSLIVFCLEIVLKRRCRYF
>BgerIr849N
VICFVLHLTQGIKLVSYKNEDAILNSVLQTSVKFFEYQQPIGVVPYITSVVTKEASIKQKRQN
VTLDANKKCIYQTMRLKQIRLFNSVDHKIVNNFLDLGFTWTRTASAILGKLHSAELWSLH
LASTDYQYFPFNTYKSYVILIPGLKYTQSFIYVCQILNNAARVILVLMHSSLENAQLLMFLK
GVLDLFCFRDFVLLVWDRNSERVHVKTALLYRSLNGYCGSVDEFTGVDTWIQNGNGGFFL

KNTDLHQNRLPHKLRCCKIVYDITDTELPPYYYSLNSSEISKEYDGFDIRLLNQINEFVTNT
ENCNEVKEILVCNTIQSEEYNLLYQRPAYPYHSLKYTYFVPEAASYPRWAWFTSVFNKDV
WVCCIVALGVMAVSLKCIATSMHTRCAQSYKSFAYCVMNAWAIFVSIGVDNIPQSTSFRIA
FFSCVVFALCVSTVFQSFVTSYLVFQDKEHQIDTLQELLREGYRVTYDSALKTLLHFDDNN
KVFFFESSDHAFLYAINTPKTALYSTEEYFHYNQLQLCVNDELPPYHKFKHYEDSNNFNGL
VFITDGVLQNKFNRVLQKLAVAGIIQKLFRDVVNPTGRNMQSERNYGLEYQPTNVHCLQS
SFYFLISGLSLGITAFFIEMAIKSNSVRILVSKVSRALKR
>BgerIr850N
VHWYLVHLTVCIKLNIIENEEVIAKCVVESSIKFLDSRYPVAFVPFAIINSTVVLKKIESHRL
HMDINDTCVPKKTLITKATFPKTSKWEFSANMTWTTTSEAVLRKLHSAEFWPILITSRNY
EHPFIDYKNYIVFIPPEDWYGPELERTPFWGFKYGHAAKYIFVLSNKPKQNGRILQGLVVD
APFSSLRDLLILVWHVNSKQVVIRNTVPYIPPHGQCGIYKELVRVNTFVKNKNGGAFVKKF
DLSFNKIPDKLKCCNIEQLPNVPQLPPYTFSKPKPNGGTFKDGVDIRILKIVTDAMSDNRN
CETKLPMLVVNTLQLSTQNLFLSQTPYPYHGIRYTYLVRKAGSYPRWTWFTTVFSKDLWI
AFFVSLSLTALAMRFLASSKHCQTSDAYQSTTYCLMSAWALFVGLGVSNIPQNFGFRTVFF
SWIAFTICVSTVFLAFTTSFLILQVKEHQIDSVDELRQEGIEVITDVLEKTKLNLDSNLSSFY
FNHVEHAFIHVVNTPNTSFYSTEYYFQSYMNRLCRLIDDVPRFHKFTEYEDSNNFYGLVFI
ADGVLQGRMNNVLKMLVNGGIVEKIFRDVTDPTGRRILSEKPPILEYDPMDIVSLQSCFYC
LLCGLSTSVLVFLVEILIKVFNKYFSKCSRKTK
>BgerIr851N
VILCSLLYANGMTLKVTNHEESALAESVLLASVKFLDPNEAIAVIPTMTLIRRGKNNHFDE
HDHPELFAIGGDRCLNQSSLEDFEILANLRSKFPSYLFDTMSVLDYHFGFYDELVLKKLHSS
EKWPIFLASKSSYAAFSTHKSFVVFYYGSKFADSLEFLCRVLNPVSKLIVIFVAKKWRSVDA
SRFALAKTLGHHLLDVLILKWEEGSRHVSLETFLPYEKPSGFCGVVREFVHLDTWVYTGN
GIEFQKNGSLHLRKVPPVLKCCKVVSSGSKADKPFDIYDGDELAYDGIDVRLLKHVNDVMT
SSDDSCHKIGKLRVTNSARAKFYRYHPSRNYPYQNLKYCFLIPEAKFFNHWSWFTFVFRT
SLWVFCVSCLGLVAAVLKAMASLNKYRDYKGVCHCVLNMWAVLLGIGTNLPACRQIRIVV
LSWVLFSLCVNTVFQTYVTSYFIVQVQEHQIDSLEELEKDGYTFVYDNKLKYRSHNNKSIL
TFNIESAFLLAINSPKTALYTAKEFFVYNANTLCTRNELPKYHEVSSFEDSNNFLGMQFED
SPLLQSRMNKVLQKLANAGIVQKIWNDVVNPKGMEIVTSSAKPTDLGFEMVSLIYLQSAF
YALVFGIGLSFVVFLHELLIHSLL
>BgerIr852P
MNMNFILNWLIFEQASVIMNDLSMYWLVLCLVNAALILIFQLFWFSLPPANGFQHQKVNC
RNGHALAESVRQVSLKLFNTSEPLVVFPAVTINLETENNRSIGEALQFSYHDLNVDGKCVN
SRVTLELFKFSSDGNRIGLAEFHDSQSNCEFQRWTFNEGLVLEKLYSTEKWPVFLASNNSL
QALIGYTNVIGFVYRIEHISNLAYFITTVFIPLKIILILIAQKNCSTDLERALQEFPAGRFQDV
MVLKWEIDSDYVLLRSFQPYEQRSGFCERNFRLVYLNTWIHGSFVKNFTLRLNKIPKVVN
CCEIATWFSAREPFSFVETDSEGNRIMDGTFTRTVKILNDVMTSRRNCREIGTIFAKYGFN
DLEDASSSYWYHSLTYTYFVPRAKSYHRWTWFTSVFRISVWMCCFSSLGITAKAISLFAST
RNSDNSYGRLTQCIVCLWASLLGIGIGRMPQTTTVRLLLLSWVVFSFCISMVLQSYFTSFFV
VQFEEHQVDTVEZLASEGYTVVFDAIGKYFQLNYNESFSIEPERDAFLHAVNTPKTALYTT
EEFFKYFAKRNCRKNELPTYHKFSSYEDSNNYFGVGLPSDRLLQYRLDVVMCRLAAGGIIQ
KIWNDVVNSNRMEVISKRSQKHSRFDYEPMNLFSLKSCFYILLFVHSASAVIFLLELTRTLP
FYQCSWNFFRALVFKIRIRFVRYGSSSSG
>BgerIr853N

ANAALILIFQLFWFSLPPANGFQQQKVNYRNGHALAESVRQVSLKFFDTSEPLVVFPAVTI
NLETENNRSIGESLQFSYHDLNVDDKCVNSRVTLELFKFSSDGNRSGLAEFHDAQSDCEFQ
PWSFNEGLVLEKLYSSERWPVFLASNNSLRAFIGYKNVIAFVYRIKHISHLAYFITSVFIPLK
IILILIARKNNSTDLERALQDFPAGRYQDVMVLKWEIDSDTVSIRSFQPYEQRSGFCERNFR
LVYLNTWINGSFVKKFTLRLNKIPKVVNCCEIATWFSAREPFSFVETDSEGNRIMDGTFTR
TVKILNDAMTSRRNCREIGTIFAKYGFNDLEDASSSYWYHSLTYTYFVPRAKSYHRWTWF
TSVFRISVWMCCFSSLGMTAKAISLFASTRNSDHSYGRLSQCIVCLWASLLGIGIGRMPQTT
TVRVLLLSWVVFSFCISMVLQSYFTSFFVVQFEEHQVDTVEELASEGYTVVFDAIGKYFQL
NYNESFSIEPERNAFLHSVNTPKTALYSTEEFFKYFAKWNCRKNELPTYHKFSTYEDSNN
YFGVGLPSDRLLQYRLDVVMCRLAAGGIIQKIWNDVVNSNRMEVIAKRSQKHSRFDYEPM
NLFSLKSCFYTLLFVHSASVVVFLLELTKTLPFYQCSWNFVRALGFKIRIRFTRNGGSSSA
>BgerIr854N
ESVSFSLILSEGIKLKPRNNEDVLADSVLAAASYKFFNQKEKIIVLPTIDVSFPNLEKFYYMD
SETVSLNYEDLCVDSGAELTIRADPGNSLANFTARDYFDTMNNFTYLSLGRIESHVLEILHS
SENWQMLLPYNTRMAFARNVNAVVFVFNYGEVAILASYLQGLVSDKSPFKVMLILAVEEN
AEDKEEHSKYPWYYYAASLNDCLILKWVQNSEEVTVYAINVYGFCGRCRKIIYLDTWLKS
NNQGSFLHNTSLQMNKVPKALNCCRIFLGPNVTSDSRNSKKLKGINYNMNIYFKILEHINE
AMTNTANCNKVALLIVLIDHKGVASVDSLFPPQKYTYFVPKARWRHRWSWFTSVFKPSV
WFLFVCSLGIAATTINIIADRGQVAVEILNYTKISLCMMTLWTVILGAGVGATPRSNRVRIL
FFSWVAFSFVVSMIFHSYFTRYFMLQIQDHQIQSKAELESKGCTFVFDKYRNKRSVYADE
MSDSFLYALNRPKTALFSTKQMFTYYATKLCKPEELPEFYEFQSSEYLLKMKMTFASAPLL
QNKLDELRECTTEIFINS
>BgerIr855NP
VAVYFTLALASGMKLSPKKTEDVLANSVLAAAHKFFDQQEPIIVFPTMYSVGSDFVKLTHL
DTLISVLDERKLCVEGGTTVKTRSDPKHSLANFTKSDVDNIIDNFELNPFGSSEEFVLKMV
HSSGHWQLILPEVRSGETLLAQYINVMAFASDLWHVQLLADYLRLKVTVRSPFRVILIIML
EEEDESLDGWFYDASGLHDCVIIKWAGNSEVVTLHTVNTYELPYGFCGRFNKLIYLDTWL
KQDNQESFLHNNSLQMNKIPKSLNCCRIEFDPDDFLPYYFSRNNSKFTRGFSIIZMEQZQA
PETALTCEVLKZKZLKDSPNLZMCYSLLGSSLTSFHWRPRWSWFTSVFKTSVWITFVCCL
GLVVTTIKLAADRGSVAELKSYSTISRCMMNLWAAILGVGVDRVPHSSRVRILFFSWVAFS
FIVSMIFQSFFTSYFMLQVQDYQIDTKEELEREGYMLVYENFLSYIHFRNERSIYVEEISDSF
LYAINKPKTALFSTKAMFNYFAATFCKPEELPLYYEFXMGMTFKKDPLLQNKLDTLIYNLI
TTGITEKIWNDYFNPKRQEFVKSTLNPPELNYEAMDFMYLQSCFLTLFFGLSLSGGVFILE
LIVQKVSVLKLI
>BgerIr856N
EAVNYSLTLEHELQFSPKKNEDVLAESVVAACSKFFNKEDPTFAFPTIDRNFPDLNPYLN
MDEFTSALKSGEKCVERDAKVTMRADTSQSLPNFTRTQYTFGPFGSSEQFVLQTLHSSGN
WPIIIFTNITVYRVLLAMKINILAFANSPGDIHILKLFITTSSLINYPYRILLVLILEENLAIDS
WFSGDASQLHDCLILKWMENSEVVTVYTVNVYEEPFGFCGQFKKLIYLDTWLKQGNQGS
FLHNNSFRLNKIPKSLNCCRIGSSIYLEPYSIAQKNSRGIIHFDGIIGKLLEHINGVMTNTRNC
DKVGIIDLLIGYFQPINQVLLFPTEKYTYLVPMAEWQLRWSWFTLVFKPSVWFLFVFSLCV
VAAIMKAIAGRGQLGEALNYTNISRCMMNMWAAILGVGVGDTPRSSRVSILFLSWVAFSFI
VSMIFQSYFTSYFMLQVQDHQIQTKEELEREGFTIAFDRIVTYFLHRSKDSLFANVESDAFL
YAINKPKTALYSTKAMFTYFATKLCKPEELHNYYEFEGLGEPAAVGMSLWHNPLIQIPLN
ELLTRLVTTGITQKVWNDYFNPKQLDIVKSSQQPPELFYERLNFAYLQSCFLVLAFGLSLSF
VVFIFELIIHSTNVVSLKVKLFHILSVIISFFCMIHHFLLKCLKFKIKFSRK

>BgerIr857NP
ASLTLSYGINFDAKKNEDFLADCVVAASSKLFKQDEPILVLPTVAASYSGLLELETQTLFLR
DDDLCVHRDTKITFSWYPTQSLANSSHIYSMIHNFTINPFGSVEEFVLKMLHTSENAPILLP
NNNLLLADNINVIAFANIYLHILDLIFYAHDALSAZLQLKVMVVLILDEEEDIEEELSSILYDI
SRLGDCLILKWVRNSEVVTVYTVNVYEELYGFCGDFKKLIYLDTWLKKDDQGYFLHNNCF
KLYKVPNNXLYCCZIFYDNYYPPYSYIQKKSKGITYFDGIVNRLLTDINRAMTNTKHSDKIG
LIGARTVNFGNRIAGTLFPSQKQTYLVPMARWHLRRSWVSSVFRPRVWVVFIFSLSIEATII
KLTAERGQDAETANYTNVSGCVMKLZAAILGARVTAIILLFSLVAFSFSVTTIFQSYFTSYL
MLRVQDHQIQTKEELEREGYTIVYDNIKSYMYYGNERSNSIEDIHGAFRYAINTPKTALSA
LKSEINRYAKHLCKPGEVPQYYTFQSFEGIMKMGLAFHKDPLLQNQLDKLTNRLITAGISK
QIZDQYFIPKRRDIVGSSLQPPELNYEPLSLVYLQSCFLILIIGLALSGIVFILELIIHRCSAPPL
NVTLFHILSFTALLCRIYHILLIC
>BgerIr858NP
VAVYYSLTLGNEMEFSSKENEEVLAESIVAATTKFFDQEDPIMVFPTIDRNHSTTDMFLD
MKEFTSFLKSEGKCVERDANITMYTDPIQNIHNVTHTQYIFGPFGSSEQFSLRTLHSSZNW
PIILYTNTIGYPVRLAIKINVLAFAYSYWDIYILMHFFKEITGSRYPYKIMLVLILEEELEIDS
WFKRSSNLHDCLILKWVENSEVVIVYTVNVYEEPYGFCGQFKKLIYLDTWRKQDNQGLFL
LNASLSLNKVVKSLNCCRIGTRKYFAPYSFVQNNSRGIISYDGIFGSLIKHINEVMTNTRNCE
NVGIIDMAIYYFEPILQVQLFHTEKYTYLVPMAQWKLRWSWFTSVFKLSVWIIFVCSLCVV
TILIKLIADRGQVAETVNYSLISRCMMNMWAAILGVGVDGVPRSSRIRILFFSWVIFSLVVS
TIFESYFTSYFMLQIQDHQIQSIEELENGGYTIAFDRMEKFLQYRNKRSFYALDMSVAFLYA
INNPKMALYSTKAIFTYFATKLCKPEELSNYYEFEARQDPETVGMELRNDPLIQIQLDKLM
TRLVTAGITQKVWNDYFNPKRLDIVKSSLQSPGLYYEPLNLAYLQSCFFVLIFGLSISSVVFI
LELIIHRTNAISLKEKLSHVLTIIISLSCTIYRFLLKCLKFKIKFSRK
>BgerIr859NP
VAVNFSLTISNGIKLSSKKNEDVLADSVVAASVKFFNQVEPIIVYPTMEEAFASKFIPRYVYL
ETSTVIFKDEDVCVPSGRQVTINSDPKNALGNLNLEEFYYFYDLENLTFTSLGISEQFVLKS
LHSTENMSIVLPSSWTVSFRLLTEFTXIIAFAYEFYDINLLAAHLRYHNRLLRIIIVLILKERV
SSIDKWFEDAALLQDCLILKWVENSQVVTVYTVNVYEEPYGFCGELQKLIYLDTWLQKDN
HGSFLRNNSLRINKIPKSLRCCRLYNNDYVVPYAYTTYSQKGIIYFEGIVNRLLHHINEVMT
DTRNCEKVGIMQVSLGFYNYYTQESLFPPQKLTYIVPMARWHHRWSWFTLVFKPSVWMI
FVCCLAFAATMIKLIAGRGQVAETTSYNSISHCLMNMWAAILGVGVYAIPRSSRVRILFFS
WVTFSLVVSTIFQSFFTSYFMLQIQDHQIQTKEELESEGYTFVFDGSSSYLSYRNNRSVFIED
NIGGIQYMMNTPKTALFNTKEMFSYYASQYCKRGESPGYYYELPSFEESRTLRIKMLNCLL
LQNKLDQLVTRLKITGLIQKLYNDFFHSWRQEIVKSSLQPPELDYEPMSFVHLQSCYFILIF
GLTLSGIVFILELIIHRVSVVSLNRVLYLILNSIITLLCRIYQVVFECLKFKIKFFKN
>BgerIr860NP
VAVKFSLSISDGIKVSSKRNEDVLADSVVAASVKFFNQGESIIVYPTLDAVFGSDVIWKLWH
LERRTVIFKQEDLCIANGRKDATLSSDPNHTLGNFTYDEYAKLFDAIDRFNSFGSYEEFVL
KKLHSSGNWSITLASNVTARQTLLGQHTNIIAFAHNFWHVSTLAFHVRALKSVARIVLILIL
EERYPSIFYWFRKAAYLQDCLILEWVEKSEAVTLYTVNVYEESYGFCGKLKYINYLDTWIK
QDSRGSFVRNCSLRMNKIPKYLRCCRLSNNNYVVPYAYYNRNQKGFIYFEGILNRLLNHVN
EVITDARNCEEIDTIEVHINNFHGLRMVDSLFPPQKYTYIVPMARWHHRWSWFTPVFKT
NVWIIFVCCLAFVAITITVIAGRGIVVESPSYNSISHCMMNMWAAILGVGVDAIPRSTRVRIL
FFSWVTFSLIASTIFQSXTSYFMLQLQDHQIQTKEEIEAEGYTIVFETFLSYLRYRSNRSVFF
DDFVSAFQYMINTPKTVAFTTKQMFTFYATRFCKPGEFPGYFYELPSFERLLKIGMFLYW

HPLLQKKLDEVLTRLISTGITQKVYNDFFNPGRQDIVTSSFKPPELDYQPLTLVHLQSCFLI
LMYGLILSVIVFILELIIHXVVSLKRVLSRILNVIVALLYRIYRFMLKCLKFKNKFFSK
>BgerIr861NP
VAVNFSLTISNGIKLSSKKNEDVLADSVVAASVKFFNQVEPIIVYPTMEEAFASKFIPRYVYL
ETSTVIFKDEDVCVPSGRQVTINSDPKNALGNLNLEEFYYFYDLENLTFTSLGISEQFVLKS
LHSTENMSIVLPSSWTVSFRLLTEFTNIIAFAYGFYDINLLAAHLRYHNRLLRIIIVLILKERX
SSIDKWFEDAALLQDCLILKWVENSQVVTVYTVNVYEEPYGFCGELQKLIYLDTWLQKDN
HGSFLRNNSLRINKIPKSLRCCRLYNNDYVVPYAYTTYSQKGIIYFEGIVNRLLHHINEVMT
DTRNCEKVGIIQVSLGFYNYYTQESLFPPQKLTYIVPMARWHHRWSWFTLVFNPSVWMI
FVCCLAFAATMIKLIAGRGQVAETTSYNSISNCLMNMWAAILGVGVYAIPRSSRVRILFFS
WVTFSLVVSTIFQSFFTSYFMLQIQDHQIQTKEELESEGYTFVFDGSYSYLSYRNNRSVFIE
DNIGGIQYMMNTPKTALFNTKEMFSHYASHYCKRGESPGYYYELPSFEESRTLRIKMLNG
LLLQNKLDQLVTRLKTTGLTQKLYNDFFQPWRQEIVKSSLQPPKLDYEPMTCVHLQSCYF
ILIFALTLSGIVFILELIIHRVSVVSLKRVLYLILNSIITLLCRIYQVVFECLKFKIKFFKN
>BgerIr862N
VAVKFSLSISDGIKVSSKRNEDVLADSVVAASVKFFNQGESIIVYPTLGAVFGSDVILKLWHL
ERRTVIFKQEDLCIANGRKDATLSSDPNHTLGNFTYDEYAKLFDAIDIFNSFGSYEEFVLKK
LHSSGNWSIALASNVTVRQTLFGQHTNIIAFAHNFWHVSTLAFHVRALKSVARIVLILILEE
RYPSIFYWFRKAAYLQDCLILEWVEKSEAVTLYTVNVYEESYGFCGKLKYINYLDTWIKQD
SRGSFVRNCSLRMNKIPKYLRCCRLSNNNYVVPYAYYNRNQKGFIYFEGILNRLLNHVNEV
ITDARNCEEIDTIEVHINNFHGLRMVDSLFPPQKYTYIVPMARWHHRWSWFTPVFKTNV
WIIFVCCLAFVAITITVIAGRGIVVESPSYNSISHCMMNMWAAILGVGVDAIPRSTRVRILFF
SWVTFSLIASTIFQSFFTSYFMLQIQDHQIQTKEEIEAEGYTIVFETGLSYLRHRTNRSVFFD
DFVSAFQYMINTPKTVAFTTKQMFTFYAMRFCKPGEFPGYFYELPSFERLMTIGMFLYWH
PLLQKKLDEVLTRLISTGITQKVYNDFFNPGRQDIVTSSLKPPELDYQPLTLVHLQSCFLIL
MYGLILSVIVFILELIIHIVSVVSLKRVLSRILNVIVALLYRIYRFMFKCLKFKNKFFSK
>BgerIr863NP
VAVKFSLSISDGIKVSSKRNEDVLADSVVAASVKFFNQGESIIVYPTLDAVFGSDVIWKLWH
LERRTVIFKQEDLCIANGRKDATLSSDPNHTLGNFTYDEYAKLFDAIDRFNSFGSYEEFVL
KKLHSSGNWSITLASNVTARQTLLGQHTNIIAFAHNFWHVSTLAFHVRALKSVARIVLILIL
EERYPSIFYWFRKAAYLQDCLILEWVEKSEAVTLYTVNVYEESYGFCGKLKYINYLDTWIK
QDSRGSFVRNCSLRMNKIPKYLRCCRLSNNNYVVPYAYYNRNQKGFIYFEGILNRLLNHVN
EVITDARNCEEIDTIEVHINNFHGLRMVDSLFPPQKYTYIVPMARWHHRWSWFTPVFKT
NVWIIFVCCLAFVAITITVIAGRGIVVESPSYNSISHCMMNMWAAILGVGVDAIPRSTRVRIL
FFSWVTFSLIASTIFQSXHTSYFMLQLQDHQIQTKEEIEAEGYTIVFETFLSYLRYRSNRSVF
FDDFVSAFQYMINTPKTVAFTTKQMFTFYATRFCKPGEFPGYFYELPSFERLLKIGMFLY
WHPLLQKKLDEVLTRLISTGITQKVYNDFFNPGRQDIVTSSLKPPELDYQPLTLVHLQSCF
LILMYGLILSVIVFILELIIHVLXVVSLKRVLSRILNVIVALLYRIYRFMFKCLKFKNKFFSK
>BgerIr864N
VILVSLLFVDGRNLKLKMNDEQVLADVILEASKKFFDPNRSIAFLPRTSVNYARKEEYADLI
SQTFFTYENIYKCNEGRSTLDTELHSSTVFNGTAQHLTDISQRIHLKYSLKYYDKLALKKLL
MSGEWSIFIPSNESTTSFFLHRNFIGLIYNRFSVRPLVEMYSYMNPYSRTILVLIATKSCPKC
AHQLMKTVKLIGRNLGDVLILRWREGTRVVDVLTFMPYEEPFGFCGNIRKIVHLDTWVQ
NEDGGSFLLNENLRPVKVPKTLRCCVFVNFGLYEPFYIESPKGLDGVDVRLLNHLNEVDT
NSTDCPEIGRILLLNTVNEALGSHYPGRDYPYFSLSFSYYVERAGRYNRWSWFTAVFRNNL
WTICIVSLGITSTVIRLLALARKNGEISSYNSTFQCILNLWSALWGTGASIPQDCKIRIILFSW

IVFCLCTNTVFQTYVTSYFVVPTQKHQIDTVEEIERERFKVIADPISRYPKFDKNEMVATW
KISESFLLAVNTKESALYTTEGYFKYNVKKLCREEEMPSYHKFSAHEDSNYFIGMLPFLDP
LLQSRVNKVLQRLATAGIVQKIWKDIVDPKGHEIVTSSLKSEKLEYEPMSLFYLQSAFYVQ
VFGLGTSGIVFFLEMAIPILRRMWLNYWRTVPETGSGWY
>BgerIr865NP
LVSLISVNCITFKLNINEEVIVDAFLEVSKXLVSLISVNCITFKLNINEEVIVDAFLEVSKNPIQ
SNGIPAZRFRZIHRLNYALKHYDKLTLKNLLVPEDWPIFISSSDESITSFFMNKNFIGFIYN
WWSFESLADLYHPMNPSSRSTLVLITKKTCLKCSLQIMNILKLIGRNLGDVSILSWKEGTR
FVNIETFLPZEKPFGICGNIRETVFIDTWVQSEDGGXFLLNANLLPSRVPKTLRCCRFHSYS
LYEPFYIDDSSERVDGVDVRPINHVNEALTRFDDCKVISNILLVNTVAEGFRSEYPDRDYPY
FFLSYSYFVERAGRYNRWSWFTSDFRNSLWILCILSLGTVSTAIRLLAYASNTGEGTNYNS
TCRFTLNLWSSLWGAGAAMPHNAKIKIILIFWLIFCICLNTVFQTYVTSYFVVQTQKHQID
SVEEIEQEGFTVIVDPLCKNPIFDNNKNIAIRKIGKPFLWTVIFYNSALYTTKGYFKYNAKK
LCKNNELPSYHKFITVEDSNYFVGMFSFLDPLLQRRVNIVIERLATAGIVQKIWNDIVDAKE
IDIVTSLKSTNLDYEPMGLFYLQLAFYVQVFGLGSSGIVFFTEIALSTLWKNNGSVVGKELQ
RL
>BgerIr866NP
IILVFLHFGNCIKLKLNRNEEVISDAILEVSKKFFDHNQPIAFLPRTVRNSEEWIHSRKIYWY
KKTVKFNVSKCNNGFPTVTTDVHPPIPYNGTAAELFSRTHRLNYSLKHYDKLTLKTLLMS
ENWSIFISSDESITSFFTNKNFIGFIYNWWSFQSLADFYELMNASSRTILILIAKKTCPKCSL
QIINKLKLIGRSMGDVLILSWKEGTRFVNIETFLPYEKPFGFCGDIREMVYLDTWVQSEDG
GSFLFNANLRPNKVPKILRCCRFVSFGLYEPFYINDSLKGVDGVDVRLLNHVNEVFTSYDD
CREDGKILLVNTVSENLRSDYPGQDYPYISFSYSYFVEKAGKYNRWSWFTSVFRNSLWTF
CIVSLGIASLAIRLFTLARYTGKGSRYNSTCQCIMNLWSVLWGAGAEIPQDTRLRAILISWIL
FCLCLNTVFQTYVTSYFVVQTQRHQIDTVEEIIQEGYTIVVDPISRYPTFHNNENVAIKRISQ
SFLWTMNVKKSALYTTEGYFKYNAKKLCKXKKTVQNNELPSYHKFSTVEDSNYFVGMLS
FLDPLLQRRVNKVFERLATAGIVQKIWNDIVDTKETDIVTTSLKPTKLDYEPMGLFYLQSA
FYVQVFGLGSSGIVFFLEITLTILRRIWDGYWRSFATDPLFAVGRY
>BgerIr867N
VVWFSLLFVNGMEFKINNNNVIADSVLQASVKFFDPHQPIAIIPRSEINIQESTNSVKNVFT
YETRPFIGHICNKILTDEIQYSLNMTASMNVDHFQKHYILKFSWSFLDEIILRKLYMCENW
SIFPATNSTYSMLFAQKSVIAFIYDVQSTSTILELCSFMNPDVKIMLVIIGERACPKCASKAL
QYLKDMNEPVLNVLILTWEEESNFVILQTFLPYNNTMGFCRELEYFYLDTWVENEGGGG
GFFSKNISLRSNKTPKRLRCCQITPFQKGEPYVIVDSLRDMEQSDGVDIRLIKHLNDVLTST
ENCKDIGKVYVLNAAYSIDMYHPSCDYPYQGLRYSFFIPRARSYNRWSWFISVFQTSLWVL
CIMFLVLIAVTTKLFASLKHAKDHSKFASFSYCIITFWSILLGFGIHSMPRRHMIRILFLSWV
LYSLCINNIFQTYVTSYFAVQIREHQINSLEELQQEGYKLVYHSYWKQFKNFETKLTTMIPL
IEKSFLLAINSPKTALYTTNEYFKYNVKKLCNENELSPYHEISTLEDSNHFFGLHFPDDPIL
QNKMNQIFKRLATAGIVQKIWNDVVNPSGKEVVISSVKPPELLFEPMSLIYLQSSFYFMFF
GYFISVILFMLELFHRRVFICHCF
>BgerIr868
MTEKSMFKIITALLFLLFSTLGEELKININEDVLAECILHASIRFFHPEQAIAVIPQLQYINDD
NTNMMPRVSEFSLTGKQCLGKFKQKTIYHYKPIDELNNYKIGLSRIVFYWKHVHELTLRK
LHSSGQWPVFAVSNSTYSTLFAYKSLIVFFSAEVSGLDLFRLFDSPSRIMVVVTVDKNCLKC
RSQAMAFLKNIAGLLQDSLILLWEQESTNVSLQTFLPYQEPYGVCGYVHKVIYLDTWINDG
NGGAFLENANLTLIKIPETLICCKIVPLDLIDSILNVLDFRLLDYANKIMTNSTKCKFVDSRY

VATSMASKLLFIDPRRHYPYKTLVYSYFVPRAKYHSRWSWFISVFETNLLMLCTVALVIMS
FLIKFFSSFKSINDAPSYGNLSHCSMLLWSSLLGVGVNTLPRSIKIRILFFSWIVYSLCVSTVF
QTAVTSYFIAQSGEHQIDTIEELELEGFETVHNVDSNPSFSTTNYMETLLPSDSFKYAINTP
KTALHTTKELFKFHSKELCEEEEMPSYHKFSQYEDTIFIGMEIRNDPLLQSRLNQILERLAT
SGIVEKVWKDIVWNAFSSPQKSLILRYESLNLIYLQSAFYFLLFGLISSVVSFLLELFFHINVL
MKFRSFCQEFVLSTVIEVLRF
>BgerIr869N
GIGLSIVNVNGKRYLNYQRIEVLTDSVMQACNKFFYPNEPITILPQSEGNYRMWETIMSSVI
GPKPTKYTGDVCFHESAPKSIALRYGVDLIYLRMAKTHVELLHLIHNLTYNWSTYDGFILK
QLHLSQNWTFMLPSTKSIHILLMQKNFLIFVYDVQSVSGLDDLRDYLKPECKVIIVIISLTDC
RKCAEMIYEYFKDGTYVGLDLLFLRWEENSRFVAFETYFPYEESMRCGSIIHFQRLDTWV
QNKTGRSFKKNANISPNKVPKVIRCCKITVKAYQEDEPFVIIFDTPKGKMMGGVDIIILHHI
NEKMTDIDNCGNKIGTIYVFNKGGNQETWLHPTFIYPYHSLRYSFYVPKAKTYNRWLWF
TSVFKTSLWMFCIVTLGLAASVIKLFTLRNFHDLASYSDLCHCIVNLWSAFLGIGITLPLSG
KIRILLFSWILYSFCLSTVFQTYVTSYFMLKVQEHQIDTTEELKLYKYMTVHETITNFLRNY
NNQSVLLGSISQSFIYAINNPNTALYTTDEFLKYNTMKLCDEDELTPYHRFSTFEDSNNYV
GLQVFRDQILQTRMNQLLERLATAGIVQKIWNDVVNPKGISITSSSIRSSEFNFEPMTLRYL
QSSFFILASGFGISLIIFILESTHNKIYLCFVKRRI
>BgerIr870NP
VTSVCLTFIEGSKLDFSGEESILPNCVLQTSLKIFEHTEPLAVIPAIKEISZPVPQYTRRSQVL
MIEAEGVCVKNKMEINRVPLQETEMPANFNVVCDWLHIDEIILEKINSAHIWSIVLALVNP
KDFQYNYKNFVVFIHDIQDILHLLYYYMQRNLNGKILIVLSDNVVGHDNLKNLLIEYLVPLF
LKDFLVLSRDELSEEILLQTVKAYGINYDLITLDSWEMDENGGTFKRNANLNINKFTDKLR
CYKFTRDINGKISPPFIFTKANSPGKTIYDGVDVRLLNIIENIMTSLEGCTDIKEIAVDNTIKD
GTPSYVAEKYPYPYHSLKYNFFVPVIKSKSRWIWFTHVFSTNLWVGCIVSLMVTALALKLF
STTKYNRDILNYSSVSYCLLNVWALFLCIGVNKMPQSLXVLRLGFSCIVCCFCLSMVFQTFV
TSFFVIRANDNQIDSLQQLKENNYSIVYDSPVKTRINFFNKMDPALFTEIQHAFLYSINKPK
TTLYTSEELFYYNLNDLCKNELPEYKQFSTHEDSNNYHGLVLLYDYPLQKRMDILLQRLN
HAGITEKIFKDVVNPSRHVMMKVRKTFLEFEKIGLLHLNSCFYFLALGLVLSTFVFLVEVK
VRWLFKC
>BgerIr871
MKFSTISSILKFTNCLIHISVYNITWNSPETGQMTVVYLKSRLVIDFVQNFLLQVHWTSEIA
VTTSELNFTEAFGRWKEGMVWQVVWKEQQMLVNNLVLVTENVSEIERMFVNLKSGCSV
LNGFWNPRADYFIYHITSSENACGLLNSKALLKSIWEKFRILNIHFMCVFYSKFSWKIQSEF
TSTEYLQKELKDLKGYPLRVSMFERRPTAMKTASGEFSGVDGLVLRALAEYMNFIPIIQEP
KDGTEYGYLEKTNQTFTGSLGDILYNKADFSANTRFIKDYATPDIEFTSPVMFDNLCIIVPK
AELLPHWLAPFRAFTFQVFYVIFLTYLTCSIIYYTLKHSLATPISVSECLFELFPVFLSLSVVR
MPRSVSEKSLLISCLAFGMIISALFQSRLVTVISKEDFAPDINTLEELAESGLSVGTRSLNLLD
ESPLLKNKLLLLSTEDSVIGNIAFRRNIAGMTRLSSAQIHVLEYLNNDGSPQLHIVKECLRF
YALAYVVEKGSPLLKRMNQILWRLIESGLIKRWTEESVFFRGGANVSTLRVFSLEDLQLAF
LLLAGGLGIAVFIFVLYEKRNLQN
>BgerIr872
MIWKVCKQFFIDDYVTVVNALNLKSFIVKDFVSKLVFQIHDSMDIPASSFSFPLSPKSGTKT
KLFNHSDAFRKISNYDFGITRENILKVHYESEILRIANKRVCKRGSQQFQNKNKYPAYPIAA
KYDTVKELHYTQKNIILVAENEKRILKYLCSLKWLEFGWNSEAIFIIFLYVDGGNLNNYED
QHRFLLSSIWKEFRLRNVILIELSEKNEDFLSKDVFRFNPFTGGQSRIGELLVFDRSSIKSVE

DLRKCGVGNFKGYPLKVTMFERQPTVVPVEDCINSTVKYEGLDAFFIYTLAYYLNFTLILH
HPIDGEEYGYLTEDGSYTGMLGDIIYGKADISMNALFIKTYGAENVEFTDSAYHDKVCVIVP
KGKKIPKWLAIFTAVNSDAMSSLLIIYFVNSVIFYTLKKIHSIYILKSIRNDIGLSVALLEMLR
PFVSSPFPKIPKTTSLRIFFGACLLLGLILTSSMQGIFFAVIANPYYYPDINTLEALDKSNLKIY
TSSPSLIDTFGDPSSSENFDTTVMNSLSRKVSSISGEKDVWEMIAFRQSAAAITRKSDFQVP
QGRYQSPDGSALLHLMQYCPRSYLLGYLIPKGSPYLHEINRCVFKLVESGLFEFHKRKINTN
KEDSLNVGQSKIQNEKAKVFSFEELQIAFYILIVGWICSILSFILEYIFGKKSHKKKGRHFKTV
SFKKNYIQYCH
>BgerIr873
MFLSFSEEQMENLTPVVIIMVISFLVVQTETRILTVKTDINRRYYNIYSHLDYIKKMECVFIE
NIGSENISDIFSLTIQYIQETMDIPMIVLTPKSHDVEISHSSGCVHLVFLNHNITLSENTENN
GSNYDYWDPRSKYIFIVSNAKKLSEDDIFTIFEWLWQTHKIIRVRMLTIYEDYSTKFYNTE
MKIWKYDPFKKELNNVHSVSFNRGNHNLNGQPIRVSMFIHYPTSYFPNSLKGNITSLTNA
NDFHLGKFLGVDGQMLFNIATFLNFTPKILRYTREMSFGFLLKNGTYTGALGQIVFKHADI
AFNSRFIKQYNTTKIEYTRIILNDQVCIIVPKANLIPRWRRMLLSFNTQLWMILLCTCFLIVI
FWHILRKINKYKRAEWFIICETFQMLLLSGILHPPKKLSERCFYASCLIFFLVITNAFQSFLV
TNITYPNYEPDINTLEELDNSNLPIWSRSQDNADIFRDLDIPVMERLLKKFSVFNGTGNQL
LKHVSNIKDAAAIVRATSSPYTESTYVAEDGSQLIHTVDECPASYQLSYIVPKGSPYLPVFN
MFILRMNEGGFTDKWHKDGIRVRSLKGEKHGEREALKVFTIEDLQLSFYIYVSGVFASTVV
FIAELLI
>BgerIr874
MRFLLVIIFCIIILMIKIMTGTGSMSSLYSKVQTYKSPEKELVRIFIKILECCLTRNSWHIIVVV
PVPTVISNMLLYHIHYAQNTPVVILKGNQHRLVNNFSSSTLSLTPTYLRYPKAFIIILNKSTP
STFYAVASKLVKLVYWNSRARILLIFTEPIESSVSEVVEEIFVKCWKENIVNIVILLQEKYDL
NSTRIRVLTYDSLKLPSNLDVTEEIFNGDMNFAYQDKLRNLHKSTIRSSMFLHYPNSMPLI
NNGDKIYFGGPDGNLITTFIKHLNASLETVSPMRDVDYGFKSFNGTITGSAGDIVYNRADIA
SNSRKIDINLLDVIEFSYPHSTDEIYFVVAKSQLIPPYMKFLLPYPKTVWYLIICTLLMATPI
WYYIRNYSQGTTLISAFLQIYRVFLSISLAYSPKIIKERLFFIMWTVFGLIVTSAYQGCLISFLT
IPIYFPDIETLQELDQSGYEIFMNPGADSWPTVEDPKDVLFKLSKKFIKETQNLQSLLKVIS
TRNKAILMDKQQADYLINLRKFSRDGHPLLNKVEECVYSNLVSYGVKKNSLYLVKFEEVIR
RVMEGGFFQKWTQDTKRLKILDGTLSDNQESSVKKEPLSLSQLQTPFFILIFGLIVSAFYLCI
ELCC
>BgerIr875
MYCISITILFIFIGKHSVSALFYSNDLPLLFENCVNQISPALIIIYNETIFPNEIVQLIHESYDFL
VITMGNTNSFTLLALTHDIYHLHYQILLFIWDQNPFDFCYVANRIQMSFSSISFHVVLIFYD
AYEGEEELKATMNTYSVRPGRTIEHFGIFCPLNVVLQQNSLNSSDMLKDFTCHPLYCIKHE
QNYVKDNSMSNQMLQHQNLSLATESTQLKVALFPDLTKAIPVIKNNNLINYEGSDAYTVN
VIAEYLNMNIVWIPQSDNTTFGYRDINGTITGTSGDVAYGRADIAANSRYMKINWPEVQY
TYPHDTDSLCFVVPKSKRVPQYKNIFLPFSNSLWTALLCTIFLSSITWYWIRKLYYKNLVGI
TIIAAFLEIYGSFLVGMFNLQPNLTIERLYIIVWVMFGIIITSSFQGSLTSFLSVPKYLKDINNL
HELDMSGFKLIVYEGIDSIISLNPDDYIMKRLWNKFKYLSDFTMVPDMMIKTQNTGCMFN
EYSARYFHRMKKYTKDGYPLLHWVKENILTSYSVYEVPRNSRYLHKFNVVITRLVESGIRR
KWDNDVLYQAMLQGNITNEVSPKTARIQSLSLYHMQTAFYILSIGLFGSLLEFAIELLFPS
>BgerIr876
MLLTIAMKVFFYILCLLSIVSSSHLSLISKSTEDISQTTKFNYELIQDSSFWMACSYHFNSTR
TLTLIADEIKNNVIYNDTVSTLTSLLVHQLNIPIEVMSTEFFKYSSVFKSPPVNKKELPVSKL

EYPPESSHSYLILSSSYKRTCDYIRLNDPTKTEWKTKDNYLVLIILHDAWKMNNIKKDVEI
FEAFWNRKILNVMLLEKHLGSMNGEHDSISVYNPLQNTEKKEVIRVELSDFSLLPKTYLER
TWKLNNFSLKVTMFESFPNAIMKCEPVCKYTGRDWEVIHNLAEYMNFEPIVFTPTDGGK
FGFKNKRGTFTGAMKDLADRTADISGNERYIKFYDTELIAFTMPAFYTKQLVVIVPKAQKL
SSWEAVSSSFNFYFWMYLLIVFMTSTVLWYIIRKIHGRVYYISHLLDTMSVFLTMSVNFLT
RATSHSQRIFLSFSMLFSLVVMCLIQSSLLDAVAHPHYNRDIKTLAELDNAGLSIVTLDPNL
IDTFDESERMRNLSSKLTYMNVSSDTLLQRIILYRNISFLTSNGEAVWFLGKYPNALHIVKE
YPREYFVSYMIHKDSPYATRIHNLLGKMSAGGLVLKWDEETRYQLQLEASSLGRNYFQNT
QSLKVFKLVDFYLAFTAWGVGILLGFIVLWLENSVDYRMNKYRVSIKV
>BgerIr877
MKVIVFIVCVIQFKTGLLASLVALKTAEISETIQLRNDFVRDTSFWTTCSFHFNSTRTLTLIL
DKVFHEVWETVTSDLASLLVDKLNIALEVFSVEFYPLQRAQSDKTFGEEITKLEYPPESSH
SYLIVSCCHFETRLYIQTNDTSKEQWKTKDNYMVLIILHPNWQNKHFRDDVEIFEELWRR
KILNVILLEKHIGPTNRGQDSINVYNPLQNTEKKVVIRVSLNDFALLPTTYLERTWKLGNY
SLKVTMFDSFPNAVMKCEPVCSYTGRDWEVIKNLAEYMNFKPVVFNPTDGKKSTFKTNI
EKFTGAMKDLIEKTADISGNERYIKYYDSDDIAFTMPAFYTKRLVVIVTKAKKLSSWEAIYS
SFNFYFWMYLLIVFIISMILWYIIRKLQGKVYYISHLLDTMSVFLTMSVNFLTRATSHSQRIF
LSFSMLFSLVVMCLIQSSLLDSVAHPHFNKDIMTLAQLDATGLQIVTLDPNLLDTFDDSEY
MGNLSSKLTHKNEHSDSLIENIVLHRNICLLTSDGDAVWFLGKYPNVLHVVPEYPREYFVS
YMIHKDSPYATRIHNLLGKMSAGGLVLKWDEDTRYTLQLEALAIGRNYFQNKESMKVFK
LVDFYLAFAVWGVGVTAGFIFLLIENSLLYRINIYRVSPLDSQH
>BgerIr878
MFLQHIIILFHICICCESLLKLPHNDDLEHHIAECVLNIIVNHYDVDLPLLVHTPGKKYPIGD
EIIQKLHNENRFTQITFRNIGPLRMAFEKPIKVGYYVMIFPPLNRDKNIELLNKMLEFTIMI
VYNRKAKMIFICLKNRKVPHSKETYVDKLLAVSVRANILDTIALEPQLLPNATINNISVTG
WKANEQSNLCSLKLDNIRQLDTWMTDKKVFQHNFTLFPTDGEINLWGCKLRFSMDFYY
PLQYHNTVNKKEMIGPMVEYYKLIMRLLNVTFVAIFYQTKDTHINFPYSYSLADFDDGQCS
MTYPYFLMDFTWFVPAGLQNSRWKCIFNTFNRLTWFFVLLMFIYGCCTTYVLQKTPKHS
TSPVSSDLFMALLSALLNHLGVGVRESYKGPVANIFFVLWLFYCMIINTAYQSAFYGLLVN
PGYISPIETLEELEESELIMETAIVIKELKESEAPSSLDYVLNYKSCGSTLEECFKKVAIDRTH
AVYASRIEGTMMSYLYSDRKLIPLVTPLNKIINREYIVTVFTRLHCILQPVFEKTLSRYLTTG
IINHHVEDCSTSYHNLQKRKRVHERIFGFSLQHLQGAFYILLVGHFLSCVIYVNEIILYSISKY
FYLSVIMIYLKSIRLF
>BgerIr879
MLVSYPYCKINQSIIILKNQHFKMKICPIWFSTMFLCVESIYCELLELHESTMSNVSRNITDF
LDSSNFLSICENHFNSSRILSIITEDSKMGNWTFFINKMIQTLHQQNGIPVIVMNMNIEPLK
FEVGGIPAIEPLASQAIRDYPAESEHSYLLVVHDVDNLTRYIHQTDNEQQTLWNPKDNFTI
IIAPILNQDCSSSNITVLDTMWQKRWILNAIVILKYFIECHSYSDAIFTYNPFQKNDMNDYG
IIVNTNAQLDYIPKTYLERTWNLGGYPLKVSLYHVFPTAQKRCWPNSCTPNCWYEYMNQ
EFYMSSESSEQNQSYMFSQRICNNCSCTYEGRDWEVLQNLAIYMNFTVSIQNDSDGDIDD
QISNLKLKVSDLAFNERFMKSYDEPDIEFTMPAFYTRKIVILVSKAQKIPIWSVIFEYFSGYF
WLYFTLTFVSSCVFWYILRRTHENVSQLTNALDMLALFLTMSVNFITKIAISSQRILLSCCL
FFSLIVMCCFQSSLLDVVSHPHFKPDINTLKQLDEAEIPIFTLDPSLRDTFENSESIYMKNLN
TRVRYDRNLAEDRILQEIVINKNCSLLTSMTEAHWYLGKYPNGLHIVKEYPREYFVSYIIPR
GSPYATRIHNLLGKMSQAGLVTKWDEDSNYILKLEAIRDRKTYQTTSSGSVFAFYNLIFSFL
VYGTGIIAGVLVFIVELSKWIDLSVRFRNPNDSKIGKAYIVL

>BgerIr880
MRIYSTLIHLLCFYLTSVFSQLFEFLESNLIRGPTQISDFLESPMFSFISKDHFSSTKILSIVIET
NYEENWRYFSNKIVEKLLGQIGIPQLIISMKQYMDGNSDAEDGNAARKKIINYPAESQHSY
LIIAHDALNLNEYLSSNQNPEKKFWNPKDNFTIVLTLLSKSDCLNLDFSQSLLDNLWQSSK
IINVILIVKYFIECDDYEDAVFIFNPFSNNGNNGYGRLITINAELDHIPKTYLELTWNLNKYP
LRVSLFNVFPTAQRKCNNCPFVCNFDKASYVSPEAKNYVCNDNYEGRDWEVLKNLATYM
NFTVRIVPEAYTGDISETLSNLKNKVSDVAFNEHYMKLYNGSDIEFTMPAFYTRQIVVLVP
KAQVLPIWNVMCMYFSGYFWIYFAITFLSSCFFWYFLRRRQQNVSQLINALDMLAIFLTM
SVNFITKIAISSQRILLSCCLFFSLIVMCCFQSSLLDVVTHPHFKPDINTLKQLDEAEIPIFTLD
PSLIDTFENSESIYMKNLNARVMYQKNLTEDLILQEIIINKNCSLLTSMTEAYWYLGKYPN
HLHIVKEYPREYFVSYIIPRGSPYATRIHNLLGKMSQAGLVTKWDEDSNYILKQQALRKGI
NYRTTTSDIFVLYNLLFCFLVFGAGIVAGVLVFIVELSTWLDISVRIRNPNDSKIGKIYMVK
>BgerIr881
MKLNYILNSLLCFYVPSVYSKLLEFLEENYIRETTAIANFLESPMFLSVCEDHFNSTRILSILV
QDNYTECWKFFINEIIEKMHEHISIPEEVINITIPRAFGGGGSGTGGAGDGHHGQHPPSESG
IQPLLPEEIKEYPAESQHSYLLIAHDAMDLKWYLDQLGNEQAKLWNSKDNFTIIIAPMSLE
DCSKSSFSSNTLTTLWQRKWVLNAVVILKYLIDCKGKNDTIFTYNPFHRNGNFYGEIITSN
AQLDHIPKTYLEHTWNLLGYPLKVSLFDVFPTARHKCSNCEFSCASEYKISFTTTERHMCN
QTDSFMDPQTVCCNCSCTFKGRDWEVLKNVATYMNFSIIISNSGIGGDIYEAINSFKNKFS
DFAFNERFMKYYKQADIEFTMPAFYTKKIVILVYKAQKFPIWRVIYKYFSGYFWLYFTITF
VSSCIFWYYLRRNHENVSQLTNALDMLAVFLTMSVNFVTRIAISSQRILLSCCLIFSMVVMC
CFQSSLLDVVANPHYKPDINTMEQLDEAEIPIFTLDSSLLDSFEQSESIHMIHLNERVRYDE
TLTEDKILKDIIRYKNCSLLTSMTEAHWYLGKYPNSLHIVKEYPREYFVCYIIPRGSPYATRI
HNLLGKMSQAGLVWKWDHDSSYELKLEAFRQRKNYKTSDDSSVFILYNLIFSFMVFGAGI
SAGILVFIVELSSWLDITVRIRNPDDAEIGKAYMVSDF
>BgerIr882
MQLIRLLIVIMRIVFDLELMTSSFTETICNHVMRSFKEAEDWNMKTKFWDVYSLHFEYSN
TITVMLFRYNIYEINDILNVILQQIIGDVEKDLIIFTIPKTYPIKFINDNYQYNLETRTPYLIIY
VNDFVMKRTAYMTMEYPQSNWTSRNNYLFLKLYSTTSNWWGNFTDTSILDDMWFNYR
VVNSVIFITHKKISSDNNEEIVLYNPFVTGKDNRIIRTNLGKNYTKNDINLNIVRSLNNHKII
IVSIDNCSSVPRSALKITTDSMHKTRNLNVMDTLEKYMKFRKNIMELLYYCFLGTPERGV
MNLLLTRNADIIVSDLYVQYHGTNKIEFTTPVLLSRKLVIIVPKSEIYPPWKIFITYFSTIVW
VKLFLLFGVCVLVWHMLRHFQNNNAVSSSQPVMRFINESIDILNLMMSTVVPFVQKIKPIS
QRLFICVWLIGSLIGGINFQSSLLNSFAHPYRGEDINTLEELDEIGLRILCHDMNILDALNESI
SYRNLVGKLKYISNETTAVDNMLIYRNASLLTSDERASMYLKKYPEKLHVIPHYPREYLVS
YMIPTGSPYVTKLQNIMGKLNEAGLILKWNTEELYRVNIKIKAEYFQTDSEEMFSMKDLQ
VLFYIWGIGSISAVTVLVLENIIQRITCFGKNIPNISLYS
>BgerIr883
MVFSGSCILSSLLLLGVILSRAMLEPVIKHFYSSNDVLKKPTLFWNISNCVLEETNVFSVHIL
VDNIRVDEFLPNDIIKEVHKVFKVPVLVFQESVQKHHNSTEEIIKLQAMYRKKRSPVGTSYI
IVGETLRVVLLYINNFNPRWRSDAVVLFLILNNSGENEDDIAYEVLFQIIWIEYSIKKAIILSV
SDEFERVFAYNPFEMQDNFIFGQIFKVDKFEASEISKHLCSNNFNMNGYLFKASVFQVQNS
STDTKTMHDYILETLSSQMNFTPELHRPEEIGAIEFPNGTIYGCLTDLGYENVDIILEPKLL
KNYRTLDLDFVTPVLRFAKMCVLVPKATLIPSWESIFKCFSWDLWLVLLINFWLCVTFWR
FLIKISSNFQNNPKLKFIETLSDTLDLFIPKSFPKLVQFTLTSERVFVSSCMLFSIIVTCIFQAV
LFKILKNPAAHKEINTLEDLYNSGLPYFAIDIEKYQAFIDIDIEIARKLSEKIKINRKGERLVK

NIAYNRNASVLIAEPFGCYLKEEFPNFNKYLHIADECPVFYMASYIVPKGSIYLPLISKLVRK
MFEAGLTKLWYDNTYEEDFLPYRFKYRKTYIKSEEQKPFMLNDLLVAFIFLLAGLILGMIIF
VVEISVTNRQFKGQTAEHSSQPVLTIKTV
>BgerIr884
MQIILVLKLLILVLTEASIERKLDVNTANHILNENAVSNARLLIIHKILSCYLLEGNALFFYLI
GNNVDKYISSNLVYVSNEIPVFIYTIESPYKTYKWNVSNKIYPKETLNLIGKTLTFVLFLDAE
YIDNFNIGIQFLKTLPQWNSRAKFFVVSFQKGNLNLSVKMEWLFYESWHNLMLNVIAVII
KVDENFGNVEEPSQFFSYNPFIGTKHLNSKLSELGTSFKVIKMFMNSIAYRECSRSNCIILD
EYLFGYKFKNMNGYNLRVSIHDHTPKSFVELNADGTVKDVGGTDGKMLRTLSNLMNFGV
ILLSPTHKQHTNSSEIEEIIAGSASDLVDNRADISINSKFIVKIQDELEYTYPHNKEALCIVVP
KANIIPRSHGIFLPFDPCLWALCSMATVITAIFWYTIKRIMFGNASFLNVIFHAVALCISVPI
RSHIFQKHGRLLFFLWVYSSMIISTMYQSSLISSLVVPKFFKDVDTLQELDEKNLKLAMFPG
LRNSALTSSTNRVRISLSQKTMVTTQTLTSCLDNLIKNNDICCVYDEFSADFVVRQKKYIR
NGVRLLHVMDECLAWYPEAYRVRKDWPLLSRLNTLIQRIMESGLHTKWTNDIKCNAVR
QEKLSLLSFNNNVVLNLSHLQSAFYILTAGLCVSTLVFLREIRPFH
>BgerIr885P
MIFFSFQMEKRKMIFFCYLIQICYSMIPLPSTKSKPSDVFLLECLDNIAKTNFHQNDLLLLQ
TPYTYKNVRSFSYDAVIDVEFEEELVTILKQWPIVILGSSVFETIYVHSLQYQNIVLVLDGKD
FNAQYYLLGTMMSRMRDVHWNPSARVIIVAKGYNIKVTYRYATRNLFSLQWLMFKAINT
ILILPGKAIVNNIAQTVFDIYTFQPNMHKGVCFERVENISLIDRWISREKRFMFGNKLFPTK
FPKNLLGCIVRGRLRFGPPVVTASADQVTGTXIIILNIAALFYRFNITRSELKVISYDNVTSGK
ENIILGTDMYPHKYSYYYEQTYPYYLHNFIWNVPFRKEIPYWKSLLKIFNPNMWVIVIITYI
AYSLTFFFVKAMKSYGNQYPNFKDSFFQTLFMTIGFSTPYKRNIVLSDLLFLLGLFYCLQIY
TAYQSTLISVLTRISRYHPIADLNELKDSDLQLYSTVECMTDEGNQVCHRNYSRSVTMING
QDTVSNFERMVAQTDIAILSTEYVSRIMLQHFFAEKKPSFTLINESVGYYFNTMIVTKGSPL
LAYINRVIGTLENAGIIKMWLQKIKYHHNRVKAQSTHLARYVSLNVDRLQGAFFLLLMGL
TTATITFLIEKIKNIKK
>BgerIr886
MNILQSGFTMEKKNFCVLFLYQWISIVFSEVPIPSSNNIQDVQLIQCLNYITETYFPKNELLL
LATPLTWSHLPPHDDNVVIDIIFEDTIIDILKDWPIIILGDINFTDKYQPFLRYHYAVFILHC
KDVNSQYLLLVAMLKKIEVIHWNPSAKVIIVAKGYHHGVSNQYVANIMLYAEWHLLRSLN
VILLIPSITKINNVSTSVIDVYTFYPNLQSGYCFQKVTKSSLIDRWHTQEKRFIFENNLFPTNI
PKHMPGCTVLAKYQINSPWVAYYGEQNQVSGTDVYILNIVSHLYNFQIVYSHIDESKLENY
QIEMVNVITGIINIIIGVESKKHVHFHDVTYPYYLIHYIWNVPFGRQVPYWRSLLRIFSPIM
WTAVLIAYIFYSVSFFLIHSFISYDRKNILQNLYNSIFYTLCMTIAVSIPREPKKFALKVLFSF
GLFYCMQIYTAYQSSLISVLTSTITYPTISNLDELDSSDLQLCSLISWVHDEGNQLNERTYKK
PIKYIAVNDLDDYVERIAFSADIALLCDAFTISQHISKGNPTFTRLKENAGYFFGTMYLPKG
SPLVMYIDNVVGILQSAGIINLWLKEINFYSTGAKEEEAIASDDPIVLKIQHLQGSFYLLFVG
LGIAIVRFMFEKCTFKP
>BgerIr887P
MENTNFCVLFLYQWLCFAFSEVPVPSSNNIQDVQLIRCLKNITETYFPKDELLLLLATPIAW
SHIAPQRDDVVIDILFEDSIIDILKHWPLIIIGDITHTDDYQLXLRYHYAVFILYCKDFNSQYL
LLAAMLKKIESMHWNPSAKVIIVAKGYHHAVSNQYVANNLLYVEWYLLRSLHVILLIPSN
TKMNNVSTSVIDVYTFYPNLQSGYCFQKVTKASFIDRWHATEKRFIFENNLFPTNIPQNM
SGCTVLAKYHINSPRVAHYGEQNQVSGTDVVILNIASHLYNFKIAYSEIDLTKYKNYQIELK
NVIYGLINIIIGDDSNQFTYRYYYDLTYPYCLTHFIWSVPFGRYVPYWRSLLRIFSPIMWTA

VLLVYIFYSVSFFLVYSFMSYGRNTTLQNLYDTIFYTLRMTIGFSIPRKPKKIALKVLFSFGLF
YCMQIYTAYQSSLISVLTSRIRYPTFSTLEELENSDLQICSVIQWLYDEGGQLNKRTYKKPIE
YIAFRDLEDYVDRIASSADIALLCDAYTISRYNSKGNPKFTSLKENAGYMFGTMRLTKGSPL
VMYIDNVVGILQSAGIIDLWMKTIKFYSTSSEEAKKAIASNGPIVLKIQHLQGSFYLLFVGLC
IAIVSFILEKRTFKP
>BgerIr888P
MEKTNFCVLLLYQZICFAFSEVPIPSSNNIQDVQLIQCLKNITETYFPKNELLLLATPFAWS
HIPPQRDDVVIDILFEDSIIDILTHWPLIFIGDLTHTDHYHLLMRYHYAVFILNCKDFISZYL
LMAAMLKKIEFMHSNPSAKVIIVANGYHHGVSNQYVXMLFVEWHVLLSLNVILLIPSNTIV
NNISSSVIDVYTFYPNLQRGYCFEKVTKTSLIDHWHTQEKRFIFENTLFPINITKNMTGCTV
LAKYQINSPRVAYYGEHVSGTDVVILNIASHLYNFKIAYSEIDLTNYENYQKELKNVVYGLI
NIIKDESNHITYKYYYDLTYPYCLIQYIWSVPFGRHVPYWRSLLRIFZSDHVDSSSLSVHFLL
CLFLIHSFMSYGRNTTLQTLYDSIFHTLGMTIGISILREPKKFAMKLLFSFGLFYCLQIYTAY
QSSLISVLTRRIRYPTLSTLEELENSDLQLRSVIQFLYDEGGQLNKRPYKKPIEYIALRDFED
YVDRIASSADIALLCDACTISQRISEGNPKFTKLKENAGYFFGTMHLTKGSPLVMYIDNVVG
ILQSAGIIDLWMKTINFYSTSSKEAKEAIASDGPIVLKIQHLQGSFYLLFVGLGIAIVTFIFEK
RTFKP
>BgerIr889
MEKTNFCVLFLYQWICFAFNEVPIPSSNNIQDVQLIQCLKNITETYFPKDELLLLATPIAWS
HIAPQRDDVVIDILFEDSVFDVLKHWPLIIFGDLTHNDRYQLNMRYHYAVFILNCKDFSSQ
YLLLAAMLKKIEFTHWNPSAKVIIVAKGNHHGVSNQNVANDLLFVEWYLLRSLNVILLIPS
KSKINNVSTSVIDVYTFYPNLQRGYCFEKVTKTSLIDRWHTQEKRFIFEKNNFFPTNIPKH
MPGCTVLAKYHVNPPRVTYYGEQNHLSGTDFVILNIASRLYNFKIAYSKIENYDTELNNVIL
GIINIIIGGDSNQFTYKYYYDLTYPYCLIHFIWSVPFGRHVPYWRSLLRIFSPIMWTAVLIIYI
VYSVSFFIVHSFMSYGRNTTLQNLYDTIFYTLRMTIGVSIPRKPKKFALKVLFSFGLFYCMQI
YTAYQSSLISVLTSRIRYSTLSTLEELENSDLQLCSLIRWSSDVVSQLNKRTYQKPIQYIEFR
DFQDYVDRISSSADIALLSDAYTISRYNSKGNPKFTSLKENAGYMFGTMHLTKGSPLLMYI
DNVVGILQSAGVIDLWLKRNKFYSTSTEEAKEAIGYHGPIVLKIKHLQGTFYLLYFGLGIAIV
TFIFEKSTYKP
>BgerIr890
MEKTNFCVLFLYQWICFAFNEVPIPSSNNIQDVQLIQCLKNITETYFPKDELLLLATPIAWS
HIAPQRDDVVIDILFEDSVFDVLKHWPLIIFGDLTHNDRYQLNMRYHYAVFILNCKDFSSQ
YLLLAAMLKKIEFTHWNPSAKVIIVAKGNHHGVSNQNVANDLLFVEWYLLRSLNVILLIPS
KSKINNVSTSVIDVYTFYPNLQRGYCFEKVTKTSLIDRWHTQEKRFIFEKNNFFPTNIPKH
MPGCTVLAKYHVNPPRVTYYGEQNHLSGTDLVILNIASRLYNFKIAYSKIENYDTELKNVIV
GIINIIIGDDSNQFTYRYYYDLTYPYCLIHFIWSVPFGRHVPYWRSLLRIFSPIMWTAVLIIYI
FYSVTFFIVHSFMSYGRNTTLQNLYDTIFYTLRMTIGVSIPRKPKKFALKVLFSFGLFYCMQI
YTAYQSSLISVLTSRIRYSTLSTLEELENSDLQLCSLIRWSSDEVGQLNKRTYQKPIQYIEFR
DYQDYVDRISSSADIALLSDAYSISRYNSKGNPKFTSLKENAGYMFGTMHLTKGSPLLMYI
DNVVGILQSAGVIDLWLKRNNFYSTSTEEAKEAIGYHGPIVLKIKHLQGTFYLLFFGLGIAIF
TFLFEKRTFKP
>BgerIr891P
MEKTNFCVLFLYQWICFAFSEVPIPSSNNIQDVQLIXLKNITETYFPKNELLLLATPFAWSH
IPPQRDDVVIDILFEDSIIDILKHWPLIFIGDLTHTDQYHLLMRYHYAVIILYCKDFNSQYLL
LAAMLKKIEYLPWNPSAKVIIVTKGYHHGVSNQYVANNLLFVEWHILRSLNVILLIPSKSKF
NNVFTSVIDVYTFYPNLQRGYCFEKVTKTSLIDRWHSQEKRFIFEKNNLFPTNIPKHMPGC

TVLAKYLINPPRVAYYGEHISGTDVVILNIASHLYNFKITYSETDLTKYENYQIELKNVVHG
LINILLGDESNHFSZYYDVTYSYCLIHYIWNVPFGRQVPYWRSLLRIFSPIMWTAVLIAYIFY
SVFXFLIHSFLSYDRNNILQNLYDSIFYTLCMTIAVSIPRDPKKFALKVLFSFGLFYCMQIYT
AYQSSLISVLTSTITYPTLSNLDELDNSDLQLCSVIQWLFDEGGQLNKRTYKKPIAYIALRDF
ENYVERISSSADIALLCDAYNISQHISKANPKFTRLKXNAGYVFGTMHLTNGSPLVMYINN
VVGILQSSGIIDLWMKTINFYSTSNKEAKEAIVSDGPIVLKIQHLQASFYLLL
>BgerIr892N
HWNPSAKVIIVAKGYHHGVSNQYVANNLLFVEWHILRSLNVILLIPSKFKINNVYTSVIDVY
TFYPNLQRGYCFEKVTKTSLIDRWHTQEKRFIFEKNNLFPTNIPKHMPGCTVLAKYLINPP
RVAYYGEHISGTDVVILNIASHLYNFKITYSETDLNKYENYQIELKNVVHGLINILLGDQSN
HLSYYYDVTYPYCLIHYIWSVPFGRLVPYWRSLMRIFSPIMWTAVFLVYIFYSVSFFLIHSF
MNYRRNTTLQTLYDSIFHTLGMTIGISIPREPQKFAMKLLFSFGLFYCMQIYTAYQSSLISVL
TSRIRYPTLSTLEELENSDLQLCSVIQFLYDQGGQLNKRTYKKPIEYIALRDFEDYVDRISSS
ADIALLCDAYIISQRISKGNPKFTRLKENAGYFFGTMHLTKGSPLVMYIDNVVGILQSAGIID
LWMKTINFYSTSSEDAKEARASDGPIVLKIQHLQGSFYLLFVGLCIAIVSFIFEKRTFKP
>BgerIr893P
NLFHYKFTTSNFYVLFLYQWICFAFSEVLIPSSNNIQDVQLIQCLKNITETYFPKNELLLLAT
PFAWSHIPPQRDDVVIDILFEDSIIDILKHWPLIIIGDLTHTDHYQLLLRYHYAVIILNCKDF
NSQYLLIGAMLRKIAFMHWNPSAKVIIVAKGYHHGVSNQYVANNLLFLNWHVLRSLNVIL
LIPSNTKINNVSTGLIDVYTFYPNLQSGYCFQKVTKSSLIDHWHTQEKRFIFENNLFPTSIP
KNMSGCTVLAKYQINSPRVAYYGVHVSGTDVVILNIASHLYNFKIAYSEINLTKYQNYQME
LKNVVRGLINILLGDESNHFSYYYDVTYPYCLIHYIWNVPFGRQVPYZRSLLRIFSPIMWTA
VLIVYIFYSLSFFLIHSFLSYDRKNILQNLYDSIFYTLCMTIAVSIPREPKKFALKVLFSFGLFY
CMQIYTAYQSSLISVLTSTITYPTLSNLDELDNSDLQLCSVIQWLFDEGGQLNKRTYKKPIE
YIALRDFENYVERISSSADIALLCDAYTISQHISKANPKFTRLKENAGYFFGTMHLTKGSPL
VIYIDNVVGILQSSGIIDLWMKTINFYSTSSKEAKEAIVSDGPIVLKIQHLQGSFYLLFVGLGI
AIVSFIFEKRTFKP
>BgerIr894P
MIILQSGFTMEKKNFCVLFLYHWICIAFSEVPIPSSNNIQDVQLIQCLNYITETYFQKNELLL
LATPFAWSNIPPHDYNVVIDIIFEDTIIDILKDWPIIILGDINFTDKYQPFLRYHYVVFILHCK
DVNSQYLLLAEMLQKIKVIDWNPSAKVIIVAKGYHHGVSNQYVANIMLYAEWHLLRSLNV
ILLIPSKSKINNVYTSVIDVYTFYPNLQRGYCFQKVTKASLIDRWHTQEKRFIFENNLFPTNI
PKHMPGCTVLAKYRFNSPRVSFYGEHVSGTDVVILNIASQLYNFKIAYSETDLTKYENYQIE
LKNVVHGLINILLGDESNHFSYYYDLTYPYCLIHYIWSVPFGIHVPYWRSLLRIFSPIMWTA
VLIVYIFIQLFXLIHSFMSYGRNTTLQTLYDSIFHTLGMKIGISIPREPKTFALKLLFSFGLFYC
MQIYTAYQSSLISVLTSRIRYPTLSTLEELENSDLQLFSLIQFIYDKGGQLNKRLYNKPIEYIT
LRDFEDYVDRIASSADIALLCDAYTISQRISKRKPKFTRLKENAGYMFGTMHITKGSPLVM
YIDNVVGILQSAGIIDLWMKTINFYSTSSKEAKEAIVSDGPIVLKIQHLQGSFYLLFFGLCIAI
VSFIFEKRTFKP
>BgerIr895CP
MEKTNFCVLFLYQWICFAFCEVPIPSSNNIQDVQLMQCLKNITDTYFPKNELLLLATPFAW
SNLPPQRDNVVIDILFEDSIIDILKHWPLIIIGDITHTDEYQLLLRYHYAVFILYCKDFNSQYL
LLAAMLKKIEFMHWNPSAKVIIVAKGYHHRVSNQYVANNLLFVEWHILRSLNVILLIPSKS
KINNVYTSVIDVYTFYPNLQRGYCFEKVTKASLIGRWHTQEKRFIFEKNNLFPTNIPKHMP
GCTVLAKYQFNSPRVSFYGEHVSGTDVVILNIASHLYNFKITYSRINLTKFENYQIELNNVF
RGLINIIIGDESNHFSNYYDATYPYCLIHYIWSVPFGIHVPYWRSLLRIFSPIMWTAVLIVYIF

IQLFXFLIHSFMSYGRNTTLQTLYDSIFHTLGMKIGISIPREPKTFALKLLFSFGLFYCMQIYT
A
>BgerIr896P
MEKTNFCVLFLYQWICFAFSEVPIPSSNNIQDVQLIQCLKNITETYFPKNELLLLATPFAWS
HIPPQRDDVVIDILFEDSIIDILKHWPLIFIGDLTHTDHYQLLLRYHYAVFILNCKDVNSQYZ
LMAAMLKKIAFMHWNPSAKVIIVAKGYHHGVSSRYVANNFLFVEWHVLRSLNVILLIPSK
TKVNNISSSVIDVYNFYPNLQSGYCFQKFTKASLIDHWHTQEMRFIFENNLFPTNIPKHMP
GCTVLAKYKINSPRVAFYGEHVSGTDVVILNIASHLYNFKITYSGINLTKYENYEIKLKNVVY
GLINIIIGDESNHLTYKYXIHISIPREPKKFAMKLLFSFGLFYCMQIYTAYQSSLISVLTSRIRY
PTLSTLEELENSDLQLCSVIQFLYDEGGQLNKRTYKKPIEYFALRDFEYYVDRIASSADIALL
CDAYTISQRISKGNPKFTRLKENAGYMLGTMHLTKGXPLVMYIDNVIGNLQIAGIIDLWMK
TINFYSTSSEEAKKAIASHGPIVLKIQHLQGSFYLLCIAIVVFIFEKRTFKP
>BgerIr897
MEKKNFCVLFLYQWICITFSEVPIPRSNNIQDVQLIQCLNYITETYFPKNELLLLGTPLTWS
HLPPHDDNVVIDIIFEDTIIDILKDWPIIILGDINFTDKYQPFLRYHYAVFILHCKDVNSQYLL
LVAMLKQIEVIHWNPSAKVIIVAKGYHHGVSNQYVANILLYAEWHLLRSLNVILLIPNNTK
VNNVSSSVIDVYTFYPNLQSGYCFQKVTKASLIGRWHTQEKRFIFENNWFPTSIPKNMSGC
TVLAKYQINSPWVAYYGEQDEVSGTDVCILNIASQLYNFQIVYSHIDETKLKNYQIEMINVI
NGIINIIIGAESKKHVHFHDVTYPYYLIHYIWNVPFGRQVPYWRSLLRIFSPIMWTAVLIVYI
FYSVSFFLIHSFIRYDRKNILQNLYDSIFYTLCMTIGVSIPREPKRFALKVLFSFGLFYCMQIY
TAYQSSLISVLTSTITYPAISNLDELDNSDLQLCSLISWGDDEGSQLNKRTYKKPIKYIKVIDF
DYYVDRIASSADIALLCDAFTISQHISKGNPTFTRLKENAGYFFGTMYLPKGSPLVMYIDNV
VGILQSAGIIDLWLKEINFYSTGAKEEEAIASEDPIVLKIQHLQGSFYLLFVGLGIAIVRFIFEK
FTFKP
>BgerIr898
MEKSNFCVLFFYQWICFSFSEVPIPSFNNIEDVQLIQCLKNITETYFPKNELLLLATPFAWS
HIAPQRDDEVIDILFEDSIIDILKHWPLIFIGDLTHTDHYHLLMRYHYAVFILYCKDFNSQYL
LLAAMLKKIEYLHWNPSAKVIIVAKGYHHGVSNQYVANNLLFVEWHILRSLNVILLIPSKS
KINNVYTSVIDVYTFYPNLQRGYCFEKVTKASLIGRWHTQEKRFIFEKNNLFPTNIPKHMQ
GCTVLAKYLINPPRVAYYGEHVSGTDVVILNIASQLYNFKIAYSETDLTKYENYQIQLKNVV
HGLINILLGDESNHLSYYYDVTYPYCLIHYIWSVPFGRHVPYWRSLLRIFSPIMWTAVLIVY
VFYSVSFFLIHSFKSYGRNTTLQTLYDSIFHTLGMTIGISIPREPKKFAMKLLFSFGLFYCMQ
IYTAYQSSLISVLTSRIRYPTLSTLEELENSDLQLCSVIQFLYDEGGQLNKRTYKKPIEYIALR
DFEDYVDRIESSADIALLCDAYTISQRISKGNPKFTRLKENAGYMFGTMHLTKGSPLVMYI
DNVIGILQSAGIIDLWMKTINFYSTSSEEAKEAIASDGPIVLKIQHLQGSFYLLFVGLCIAIVS
FIFEKRTFKP
>BgerIr899
MEKTNFYVLFLYQWICFAFSEVPIPSSNNIEDVQLIQCLKNITETYFPKNDMLFLATPLAW
SHIPPQRDEVVIDILFEDSVIDILKHWPLIIIGDLTHTNHYQLLMRYHYAVFILNCKDFNSQY
LLIGAMLRKIAFMHWNPSAKVIIVAKGYHHGVSNQYVANNLLFLEWHVLRSLNAILLIPSN
TKINNVSTSVIDVYTFYPNLQRGYCFQKVTKASFIDRWHTQEKRFIFENNLFPTSIPKNMS
GCTVLAKYQINSPRVAYYGEHVSGTDVVILNIASHLYNFKIAYSEIDLTKYQNYQMELKNV
VHGSINILLGDESNHFSYYYDVTYPYCLIHYIWNVPFGRQVPYWRSLLRIFSPIMWTAVLIA
YIFYSVSFFLIHSFIRYDRKNILQNLYDSIFYTLCMTIAVSIPREPKKFALKVLFSFGLFYCMQI
YTAYQSSLISVLTSTITYPTLSNLDELDNSDLQLCSVIQWLFDEGGQLNKRTYKKPIAYIALR
DFENYVERISFSADIALLCDAYTISQHISKANPKFTRLKENAGYFFGTMHLTKGSPLVMYID

NVVGILQSSGIIDLWMKTINFHSTSSKEAKEAIVSDGPIVLKIQHLQGPFYLLFVGLGIAIVSF
IFEKRTFKP
>BgerIr900
MEKTNFCVLLLYQWICFAFSEVPIPSSINIQDVQVIQCLQNITETYFPKNELLLLTTPFAWS
HISPQRYDVVIDILFEDSIIDILKHWPLIFIGDLTHTDHYHLLMRYHYAVFILNCKDFNSQYL
LMAALLRKIELMHWNPSAKFIIVAKGNHHRVSNQYVANNLLYLQWYLLRSLNVILLIPSN
TKINNMSTSVIDVYTFYPNLQRGYCFQEVTKASLIDRWHSQEKRFIFENNLFPTNIPKHMP
GCTVLAKYQINSPRVAYYSKDKLVSGTDVVILNIASHLYNFKISYSEINLTKHQNYQLEIRN
VVNGLINIIIGGEANQLTYYYDLTYPYCLIQYIWSVPFGRHVPYWRSLLRIFSPIMWTAVLIV
YIFYSVSFFLIHSFMSYGRNTTLQNLYDTIFYTLRMTIGVSIPRKPKKFALKVLFSFGLFYCM
QIYTAYQSSLISVLTSRIRYPTLSTLEELENSDLQLCSVIKWLYDRRGQLNKRTYKKSIQYIA
ANDLDVYVDRIASSADIALLCDAYAIRQHISEGNPKFTRLKENAGYFFGTMHLTKGSPLVM
YIDNVVGILQSAGIINLWMKTINFYSTSSKEAKEAIASDGPTVLKIQHLQGSFFLLFVGLCIAI
VSFIFEKLKLKP
>BgerIr901
MSRQQNLLNCLLNITERYYNTKEPIQLMTSSFWENGTNEDRTMITDIYLQFLHEPQNFPIL
SPIHYTKQVKLLPWRGLAGGIIVILNQDDLEDQIICLSILLFVSWKYMSLNKYTKVIVLTTYS
SQNSESEEEFVLKLLRETWIFAKVYRLRRWSRFKNTIVLMSSNVSNHVDVFGWLPEKQSD
RCIKELSTVTLLDTWIAENETFKYNNDLFPVKEYPGLRLCVLFYEVWGWQPYAYEETFW
KNGEWHGVFLGPEIRVIKETAAASNFYVIMREGGETANFQAVEQGGIDIFGVSPLREYFMP
SFDHSGVHYSETKAWHVPAGKPIPSWQGLLRIFNPQLWSLVISAHLLVSVVFWLLKKFGL
ETRAGDGSIIAACLYTLRMSLGISVPVKSNSRLFLALLSISLFYYLQVYTAYQSSLIGFMTHPG
TLQAIKNLKELEMSGIELASALRYTHDFLPIGGSEYLGRNVSYFDKNTVNRLGCEGNLAVM
ASNTFVDFRASFTDEVHCKPRYQMLDDELESTYMVLYMTKGNLFFERIDDYITRMQSGGL
IHKWFQEEMTKNSKIFINYTKRFSKLTIHHLQGAYYVYFTGLILSISTFMLEVFYYCSNKILK
KMS
>BgerIr902
MENFITSQNILMFFVLFVNYNYNYLCRQQNIYKLLMFLVLLPSFKEGLALKSQSRWGKSDF
YSLTHPKYAYSPISRQFLTCLHHISSRYVTQNKSIAILDYPSLTYPTYPDIDVIHQEIHDRYQI
LQTPYGNTKISRLHGMIISIPQLFVVLLPDIANGQKVCFILGKLLEEIQEGVKNTEALIVVVA
TVQLHPKQRYNLALCLIAVTWEVLKVNNVIVLLPESTVNESFVIDIFGWLIEHQKTPCIASL
DTVTLFDRWLSSKGGFEYNNDLFPAKSVFGLQNICVLKILLRKFTPYIYGPYGEQLIGYYSE
VLSAFMRNTKVKLTFRWDARENTDILSLLSFGNSYFADECQNIYPYFLVDLIWLVPSGEPI
PKWQSLIRIFNYNMWACTILIFILGSLTFWLISKHEDHPKSIVQIITYVFLTHIDQGSGDIYK
RHLSILLFSVWLFYCLQINTAYKSALVGFLLNPGEYPPIRSLQELEESGIERAIAVSGSKEPIT
KYIKCSDEFNCIKRIVTKRHLAILINKDLITPKYLSQYATEDGRVTIVPLYETMYTLYLTFRF
NNIGCMFFKRFHSSLVRIIAFGHLHRMHRIIDYTTSKLHYSRVKKEEATIISLSHLQGAFIILF
IGLVFAFIVFWGEFMVYYFKNKCKSQVMTL
>BgerIr903
MKSFNLFCGIIRCILFLQFTNIYETEAKLSKDVSRCVLDIYKRYFQMEGPTVVQTYAGWKGY
LLNERNFTPYFEFEDALVGSLFATSQNTFMIAADTEYSQYFTNTMVKASNFIFVLDCGSIEL
QMHMIEMFILRIRRDLRNRASRLLIVSTYVFLSMKQLETFARLLLNIAWALMLNENSIILLP
DVCPSYDNCQFVRFNVFSFALDKQSNPCSKLLNCVSLLDTWDTINKKFLANNNLFPDQKI
KNMKNCSLDMFLAQWSPFIVIDYKGKSFYGPFAELLKTTARYINFQMEVTVDPEKVEVEN
SPYGIIGPAIMTMFGGFGEGQALNSYFMDDLSIYVPAGAQISKWKGLIRIFTGELWFLVAM
TYFFGSVTFWLLSKPAYRPSYINALLGTMKTYLSMGITFNHTGLSISLFFIFWLCYCLVINT

AYQSTLISFLVDPGESKPIGSLQELEESGIPRGRSVRYAYDDIIPEYILSFNDCTGTDCIDRIVD
STGMALVGGKTITDLIISSAYTKRGRPLIVSLPDWSLKAYLTFTIYLGDSFKRHFEFIVRTLK
SAGILNKWDEEGILEMRKFFRTDLDEDYSPVLQLTHFQGPFYILLIGLAFAFLYFIIEKVKFI
YSHHK
>BgerIr904P
MMYHKYISIFWLLSPSCLALLKLAKDTSIEQHMEECILEISRTYFNTELPMIVQTPSTFYEL
TERHSTHGERLIQKLYEQNLFVQLMYGHTGVHLVTETEFIMYPGSHIIILPSTNILEYFYTT
FLLIKQVMLGSFNTGGRVIIVSTSRSIRYMDGKKLPHFLLDGVFKYGYVETIVLEPSAASKN
FKIFSWSINEQKDIMLGKCXIKYMDTWISKERRFLLNTNLFPKMPTINLNNCTIYVFAKET
TWFALYAENTLSGTVVHVISELCARFNCKVTYVKDPKFSKCHILFPVYYDPSYRNIGNSMT
YPYFRRDITWFVPSGLEVPRWKSLVRAFNPLVWFLILSTFTSASCIMWMLQKSQEHWEN
HSPNVGNALMSALLTHLGVGVEERYKGFIATLFFTLWLFYCLIINIAYQSSLFGLLIYPGVLP
HIENLNELEESGLNMTRAAYFGDDETYWYKFNKYRLCGYTTNECFTKVSEYRSHAVLMD
VEKGKTWSTIFRDAHGKNRVQPLNQLMGTMLASIAAFKLPCVFQSILDSLMHRISSSGLLR
YWSHYYGTWLWKMFFFRARHQVNIRVITLLSSRRFPFVAHWSCVVTVCVWDRNFSFIYF
KGFLFLVH
>BgerIr905
MHRKFVWILFFASYCTSQLTLPTYYPLEHDFANCVANITDTYLDFHQPIVLQMQSPYNRII
RDNVVQALQIRNNFPLIILGNTARVTNRIRPGAFIIMLPVVYDKSDVYFIHEMLKKSSNINL
KAKIILVLSWQGGTTNINDKTISLYLLEKLSRYGYVNTLILDPVYEGESVFTNNNINILSWEIN
EQSEFCSELDKIKHLDTWLCREKTFIYNTNLFPTSKNVDLRGCKLNVVAYPLYPFANNCP
NHYCGTISHILMSIIKDINVTLLDFRKGSHNQIDVLLPIIMDAEKLSDCVFSYPYFFLDLIWY
VPTGREIPRWQSLFRTFNSLTWFLIILTFCFGCLTMWLLEMSPSHRANIATTANTAFAITA
LLCHLGVGVADKYKGVIATTFFSLWLYYCFIINTAYQSAIFGLLVDPGRYEPIQTYKELLHS
DLVMERLFVLSEDRGYIYWEKYGKTYPLCGDSVEQCFRKLSDSQSHALLAYTDMGNTLSN
YFKDKRGIPTYEALSEVEGTAFYTIKFSRWNCILHSSFEKICQGFYSAGIIDHLNKLMKWTY
KMENREPEAETIFAFSLYHLQGAFYLLLIGLSVSLLMYIAEIITHSISN
>BgerIr906P
MAVKLFFLLLFINGGCASMLLPIAENALEQSLAECALYIIPKHFDKNLPILVYLPRAWYPVR
HPNQRIMQNLIEMFHIQNNVFELVLGQVRSFRMRGTTIGAFVIVIPPLESESDLHYFRNMF
EMIDKYKRRAKLLVIALYISSNLKTSNSTFSHILLDFALIKEYVDTEPVVDVSLNKSNIKVFG
WRINEQSNICSGYLDKKKZLNTWVSKEKXFLESADVFTVQHKVNFKGCTLNAVVVNGPP
HSIVDVKPZSNLSWEILVHIGKRVNAKIRWRDFSHRKDSDIHIYFHSVLXVFALILTYPHFT
VDYTWIVPSGAELPRWHCLFRAFPPFLWLFLLIIFAVGGCTMWLIDRSHRSTDFFKVGVA
YLMSALLTHLGVGIVDVHKVFAAGLFFTLWLFYCLVINTAYQSAFYGCVVDSGTHLQYKHL
KNSRNLIWLWZGILIIISVISIDTTNVEHLLKYKYCHSAATCFGNZIKNKSHAILDGTWSIKM
TSFFHREKYGRLPFQHLTQFRATFYYSLELGVLSTILRDDVERIDNLFVTAGFPKNWENIL
TFLIKRKASKSDXFFAFDIDHMQGGYSLLVTGLLLEIFLFVLEICSHFIFGTLHLLFLKYYVQF
LKYVGL
>BgerIr907
MNYILQVEGTIMDVKLFFLLLFINGGCASMLLPIAENALEQSLAECALYIIPKHFDKNLPILV
YLPRAWYPVGHPNHKIMQHLIEMLHIQNNVFQLVLGQVPSFRMRGTIIGSYVLVIPPLEFQ
SDLLYFRNVFEMIGKYKNFRGKLLVIAFYISSNLKSNNSTFSHILLDFAFIKEYVDTIVLQPE
TVVDVSLNKPNIKVFGWRINEQSNICSGYLDKTKELDTWISKEKKFLDSADLFTVQQKVN
FRGCTLHAIITHFPPFAIVDGKPLPSLLWEILVHIGERVNAKIRWKNMSHRKKSDIHIYFPS
VLMPDNIYILMFTYPHFPQDFTWLVPSGAELPRWYCLYRTFPPVVWFLVLLTFALGGCT

MWLFDRSHGSTDFFKVGVMSALLTHLGVGIADLHKGFVAALFYTLWLFYCLVINTAYQSA
FYGCVVNPGNSPAIQTVGELENSDLVLVRNLNFMNFYIDNANTEYLMKYKICQNRKACYN
NLINNKTYAILESMWEIERMKLLHHDKYGRFPFQPLTQFITTFYFSFELDVLSSILSDHVER
IVNVAVCAGLPERWKNMLIYLTKRNTGTRKSDDPFLAFDIDHLQGGYLILVTGLLLAIFLF
ALEICRHFTLGTLYLLVL
>BgerIr908P
MAVKLFFLLLFIYRGCASILMPIAENAIEQSLAKCALHIIHKYFDKNLPILVYLPRAWYPVR
HPNHRIMQNLIEMFHIKSHFLEIVLGEVQSFQMRGTAVGSFVIVIPPLESERDVHYFRNVIE
IIGKYNSRGKLLVIAFYISSNLKPNNSTFAHIILDFAFSEEQVDAIVLQPETVEHIPLNNSNIK
VLGWRINEQSNICSGYLNKTKELDTWISKEQKFLDNADLFTMQQKVNLRGCILKAVVFNI
PPYAIVGQKDRPVVVRYILIEIGNRVNAKIKWKSKSNGEDSDIHXFFPSVLEPELVYDFTFT
YPHFSLDHIZIVPSGAELPRWNCLFKTFPTFVWFLVLSSFAVGGCTMWLLDRSHRSTDFF
KVAVMSALLTHLGVRIVDVHKGFVSAQFFTLWLLYCLMINIAYQSAFYGCLVDPGQLPAIQ
SFQELEKSDLVMVRNFNFLTDNSANIEYMLKYKFCQNSVTCYNNLIKNKTHAILENTWAA
KMTEHFYRDQWGRVPFHPLTEFIATFSFSFELGILSSILKDEVESIVNSAVSAGLLEKWISM
MIVINKRKFINSNDPVFGVDINHLKGGYCLLLIGLLLAIFLFMLEICGHFIFGRLHLLFIKYYV
QFMIYM
>BgerIr909P
MFLKQFVIFTLCIWAPCTSLLMPVLKSPLEQALEECVLDITLRYFEKDAPILVEIPGSRFPFK
HPNHNFGQDLIRMLHNQNKPLQVMLRITRTZCRESRIGSYVILLPPIKTIRDLSYVNKVLE
NIYTYVFKPGAKVLFITMFSQWKPKEMSFTQILLQLAFKNDFVDTIVLEPEKEGKMFSDNS
SNINMLGFSINDQSDICSEHLYKIKLLDSWMSREKRFLQRTDLFKPRKEINFNGCFLKLSVE
YSIPHVLYSQNGAVGPVMEFLKEMTNRVNANTIQVDEEKMQVAFPKNLRPTKHYLWQM
SYPHFRKDMRWVVPSGRELPRWKSLFKTFSPLVWGLVLVAFGFGGCTLWLLERSASRNS
FSKIFDAVIMSALLTHLGCGIADRYKGYIGTTFFVVWLYYCIIINTAFQSAFYGSLLKPGHFP
TIQTVKELEQSGLRMERNFDFVGDGFSGKLIKSFAKYKICPKAYDCYKILATKGALAVFDN
EFSIKMYKFLHVDKQGYPKFSLLNERYTFYQTVFFRFFSTVLLDTYDHIANLFIDTGLSHR
WDNYFTAKLNHILTSFAQNDLTAFSZDHLQVAFNLLGIGLMLAALSFLVENCFRLICCGLY
LIFIRWCMQNFYHFMSFIQ
>BgerIr910
MWLQHILSLYCFWGCYGNLLAPTEDSYIEKHVFECILHISKTHIEKEKPLLVLTHNNTFQS
QNKSLQYDKITQILHNEFQYPIISFGVNNIIRNYREIKPNSNVVIYPPIETERKVVRELLRKL
KFFPNAKGKYIIAATRLYNNVEDRIKVSRFLIQSVFKLNVIHIIALVPYKTDNQSHLLIDVFT
WNPNEQKNICYEIIDKVKLIDVWNTHTKRLLYNENLFPTLPKIHMRECRVHVKSGFNFPF
IIRDKHGVITGSMMNIIRIIHEDMRCRVVLGNYVENEHIAFPFVYSPLNRISECSLTYPHFRQ
DVTWFVPPGLEYPRWQSLVRSLSPVVVWALILLIFVIGSYVIKLLGNYESEQNANVTAILTAL
QTYLGSEASDRFKGPLAVSFFSIWLFYWLIINTAYQSAMFGLLVNPGQMPPVKTFKELKES
GLQMKKAITFDSLMNSTSNLFLLRDEVVNYEPCTADKCYINLTKYRNIAVFDTAVRGNLY
KKFKLDERGRYQLYPLDENIASVHIGIAIRKLSCILYNKLEKTLRRLNTAGFINKWNEDIIM
GWRYDNEQYFDSGGVAFAFTLSHVQGTFYLLLIGLMLALIAFIIETVVYTLRS
>BgerIr911P
MWLQHILSLYCFWGCYGNLLAPTEDSYIEKHVFECILHISRTHVEKEKPLLVLTHNNSLQS
QNKSLQYDKITQILHNEFQYPIISFGVNNVIRNYREIKPNSNVVIYPPIETERKVVRELLRKL
KFFPNAKGKYIIAATRLYNNVEDRIKVSRFLIQSVFKLNVIHIIALVPYKSDNQSHLLIDVFT
WNPNEQKNICYETIDKVKLIDVWNTHTKRMLYNENLFPTLPKIHMRECRVHVRSSFNFP
FIIRDKHGVITGSMMNIIHIIHEDMRCRVVLGNYVENEHIAFPFVYSPLNRISECSLTYSHFR

QDVTZFVPPGLEYPRWQSLVRSLSPVVWALILLIFVIGSYVIKLLGNYDSEHNANVTAILTV
LQTYLGSEASDRFKGPLAVSFFSIWLFYCLIINTAYQSAMFGLLVNPGQMPPVKTFKELKES
GLQMKKAITFDSLMNSTSNLFLLRDEVVNYEPCTADKCYINLTKYRNIAVFDTAVRGNLY
KKFILDERGRYQLYPLDENIASVHIGIAIRKLSCILYNKLEKTLRRLNSAGFINKWNEDIIMG
WRSDNVQYFDSGGVAFAFTLSHVQGTFYLLLIGLMLALIAFIIETVVYTLRS
>BgerIr912
MITQNLLRFFIFWLHALCCYAVISTSDSEDSLETVLSECIVKISSTYFNPNLPLTIQASNTWH
YPYEQNFKRGEIFLQMLSQENFVPRVIVGHKFSMVLDKIDKIGSIIIFLPYVNNDREGMLVL
EMILASIHKSRKKNSQIIIGAMTIYEEFEVRKRNAKPLLIVANAKLSDNAIVLFPYPSPSNNS
SYHVDVFGWLPEEQKTSIAQIDNVKCMDTWIGETKSFLNNFYLFPIKALTDMGRKVVNVA
MNDFFPFCFQDHRMAKGSFFFVLMWFCEKYNCRVKNTGIIDSVHIVFPTIYEPRYESLGCE
FTHPQFSTQLQWFVPSGTEIPRWQSLVRTFSGMMWVFVAVVFATGVSIFFLIESCWCDVF
DLVGNLDTALTTVTLTHLGLGVSYKCTGLVTCLFFTLWLFYCLLINTAYQSALLGQLISPGE
YPPIRSHEELMAANIKLKTTFYFTLGRDSFWVDKIRKYEFCHLTECFVDVERHKDQAVLS
DRYLGMKEAALRIDVQGKHQVLEMPDLEGTMYFLANVRLGSCIFRGALEKEIQRLESSGII
KHWNELFLKEFQELNARQEISFLFSFSLNHLQGPFYVLLIGLVFALVVFVSETLLHHFK
>BgerIr913N
FSFIALFLLLISCISYALRYKGDEMSLENVIAGCILNISKTYLNPEHLLAVLTPSKWFHPRDP
SHQIGNNLSQIIYEQFTYTQVIFSFEKSPGLLRVLKPGSFIFVIPPINSVRVKRLMYLMFMEA
SNYIGNSRGRVIIALMWPIDKEKSHAYASVFLQSALVTTFPDAIVLVPKIVRSNLLDNEKFF
EVDVFGWKLHKQINICSKNIDTIEYLDTWVSNQSRYLKGNTLFESNQDTNMNGCTISAAIY
HIDPFVYFHKPTGKYAGLFMFFLSQLCKRINCRIQPISDLQGSILSFPVIYGTWFTPNECQFL
YPHFKVELNWFVPSGSRFPQWQCLVRTFSPLIWVSSFVVFCCGSFVFWFLEKGYIRPIHRA
GNIVELAILTVLVTHVVGGVTDRYRGAIGGSFFVLWLFYCLVINTAYQSALYGVIVYPGEEP
PIETLQELAESGLQRKTLYKNPVYGNFVDPAVDVHLYEFCTQTQTLCFKEAGRSRTQTLLS
DLDMGLTISSHWPDEHEKPQMVPLVESYGTVYLTANISTLSCAVFDSASRLLESFVESGIFS
YWRNRLNFILRYYVVKHPKRVKPEFSLWHLQGIFYLLLIGWLLAFLVFLIEAFLHFRKRFV
QNLI
>BgerIr914N
FSLNGMFLLLISCISYVFSQNEAGISLENDVVGCILKVIGTYLDPESLLAVITPGKWFHPRDP
RHEIGNNLSQVLHEQFSSPQVVLSFDNSSKLSQFLKPASIIFLMTPITSERTKRLLTLTFLKA
TEYLGDSRARVIIAKLWVSDDEITRTLSKVFLNYALVQGFPDAVVLAPKKHNYMTGEHNS
FDIDVFGWQLSKQKYICSLYIDEIEYLDTWISDESKFLRGSSLFDSGRHTNLNGCELPVKLL
QFEPYVSIEERTASGIFMAFLWYLCKKINCVVAHGINWKKCIITFPVMYDTTQTSTQSTYL
YPHFKTEFTWFVPSGSKLPQWQCLFKSFSPLVWLSVLLTLAAGSFVFWLLEKGQSRPIRRS
GNVEELAILTAVVTHILVGVNVSYRGTTAVSFFILWLFYCLVINTAYQSALYGFMIYPGEVS
PIRTLEELDEFELQRINTYKTRDMSKDIESSAVYDLYDFCTQTQSECFKEAGYTRTQAVLG
DRDMGMVLSSHWLDQYGNPQMVPLKENVFTMYLTARILTLSNQVFDSASMLLDSLVERG
IFSKMRNEYYFKLRRHIAKRYIAKIYGFSLFHFQGIFYLLMIGWVMAFLVFLFEIFLHFRNR
MKGAMHKI
>BgerIr915NP
FGLNGILLLQMLCMSYTFRGKGGEISLENDLAGCILNISKTYLNPEQLLAVVTPGRWYGPR
DPRHQIGNNLSQILHEQFNFPLVLYSFVNSSQVSEYLKPDSLIFLIPPVTSNRIRRMLKFMFF
EASDYLGNSRAKVVIAKMWISVEEESRDLATFYLNCALLRGFLDAIVLAPKKPHDHISGSR
SSFDIDVFGWNLSKQRNMCSKYIDKIDYLDTWVSNESRYLLGNNLFQSNEDTDLGGCVLP
LSASIMQPYFIFNEKRGSGLFMEFLHHLTNKINCRLRLVSNMEDSLISFPIKYLTIEARKDC

KNLYPHFKLELTWFVPSGSKLPQWQCLFKAFSPLVWVSVLFTLSSGSFVFWLLEKAQNRP
IVRSGNIEELAILTAVVTHVLVGVKLRYKGAVAVCFFVLWLFYCLVINTAYQSALYGFIVYP
GKIPPIETLQEMDDSGLQRISTIKSDDRVKGASDMFDNYDFCTQTQSLCFKEAGSTRTQAIL
ADRDMGLLISZFWLDQHQKALMVPLKENVVTMYLTAEILSLPCXASTLLESFIESGIFSHK
RSQYDLVLSYKGVRHSSTRNYAFSLFHLQGIFYLLVIGWLMAFLVFIFEVLIHFRKGSMHNF
N
>BgerIr916N
LAINGLFLLRMSFVSYILNQIRADFSLENDLSGCIVNIGKMYLNQEGVIAVVTPRKWYQPPD
QKHQIGSNLSQILHEQLTFPQVIFSFKHYSKSLYAMKPETIIFLVPSPFSSIDKQLLFLMFAE
TTKHFSNARAKFIIALMIPTDQDLAQSTAEFLFNAALACGYPDAILLASKEPHNHSPGKHT
SHDIDVFGWKVGKQRNICSRKIDTVEYLDTWITNESRFLIGGDLFKSNEDTNMNGCFLGV
TSFNIDPFSYFDKDAGRYKGLFIFFLNVLCTKLNCRLRSSSELPYSNIEFPFIYLQSFPKEECS
YFYPHFIMEVTWFVPSGSKLPQWQCLFKAFSPLVWIFLLITLSSGSFVFWLLERGHNGHIH
RAGNIVELAIMTALVTHILVGVKMRYRGAIPVSVFVLWLFYCLVINTAYQSALYGFMVYQG
EIPPIRTLRELEESGLQRKSMFKNESSFHRNEVSDIFTLYDFCEETQFDCFVEAGNTRTQAL
LSPIDMAMMHLNYWLNQNEKPKMVPLKETVATVFPTAKFKTLSCQEFNVANKLLESFVE
TGVYSFYKSRYRYFYNFHHSRRSKKDDSFSLWHIQGIFYLLLFGWLLSILVFLFEVFSNCHY
RVIQHVI
>BgerIr917P
MVMALVKNKEKKLSNFFFRLTLSGIILLQILCICYGLNHSEADLLVEKYSSGCIQSISEKYFN
PGRLLAVLTPSKWYYPEDLGYQIGNTLSQILHEQFNFPLVIFSFEISTGLAYVLKPDSFIFLIP
SASSDRSEKLLYLMFLETRKYFANSRGKLILALMYPLEEKRAREVANIFLNIALFATFPHAII
LIPRIVDNCKSAKERCYDIDVFGWTLRKQSNICSGNIDAVEYLDTWVSNQSRFLVGRSLFE
PQQDTNMNGCAIGVRTSHFDPYVNFNKRAHKYNGPFILFLQILCKEVNCRVRFNNRFSDS
NINFSVVYQTWYRQEECQFFYPHFKLELTWFVPSGSKLPQWHFFLKTFSPVVWVSVLFTS
FFGIFVFWLLEKSQNQPFRRAGIIEELVILSAVVTYVLVGVKLRYRGAVAVCFFVLWLFYCL
IINTAYHSALYTLISYPGKISPIRTLQELADSWLQKKNLYKISVLDYGNVSSEAIHLYEVCNQ
SQSFCFKEAADSQTQAILVDKDMGKILSNYWFDQHEKPQMVPMQEPVVTIFLTAKFPSLA
CQVFDLASKILDSFIETGIFIHWRNNFNDNLRHYHLLQSTEKHPRFSLWHFQGIIYLLLIGZ
LISFLVFLFEVFLHFRKRFIQIYIEVPLRILTIY
>BgerIr918NP
LAINGLFLLRMSFVSYILNQIRADYSLENDLSGCIVNIGKTYLNQKGVIAVVTPRKWYQPRD
PKHQIGSNLSQILHEQLHFFQVIFSFVHYSELLYVMLPETVIFLAPSTFSSRNEKLLFMMFA
ETTRHFGNSRAKFIIALMTPTDQDLAQSTAEFFFNAALTIGYPDAILLASKKPRNQGXDID
VFGWKVGKQRNICSGKIDAVEYLDTWVSNESRFLVGSNLFKSKQDTMNGCILGVKSFDLD
PFSHFIKETGNYKGLFIYFLGQVCEKINCRLGSSNYLSYSNIKFPFIYGELFTYEKCRFFYPHF
KVEVTWFVPSGARLPQWQCLFKAFSPLVWICLLITLSSGSFVFWLLERGRNGHIHRAGNIV
ELAIMAALVTHILVGVKVRFRGAIPVSVFVLWLFYCLVINTAYQSALYGFMVHPGEIPPIRT
IPELDESGLQRKSMFKNASINFHSSQFTDMLRLYDFCEVTQFDCLIEAGNTRTQALLSDID
MGMMLSNYWLDQREKPKMVPLKENVATIFLAAEFTPSVCQLFDTASKLLESFVETGIHNF
LKDRFKYGFNFNNLRHLKKVSHSLSLWHLGGIFYLLLVGLLFSFFVFILEMFSQFKNRFTH
NFI
>BgerIr919N
LAINVLYLLRMSFASYILNQIRADFFALENDMSGCIVNISKTYSYFSQEGVIAVITPRKWYQR
HDPKRQIGSNLSQILHEQLNFPQVIFSFDRFTGILYLMTPETIIFLAPSTFSSRIQNYLFEMF
AETAKHFSNARAKFIIALMKPIDQDLAQSTAEFFFNAALTIGYPDAIVLAPKKPHNHASGK

HTSYDIDVFGWKVGKQRNICSGKIDTVEYLDTWVSSESRFLVGSNPFKSNQDTNMNGCIL
GVKSFDLDPFSHFNKETGKYKGLFIYFLGQVCEKINCRLGSSNYLSYSNIKFPFIYGELFTYE
KCRFFYPHFKVEVTWFVPSGARLPQWQCLFKAFSPLVWICLLITLSSGSFVFWLLERGRN
GHIHRAGNIVELAIMAALVTHILVGVKVRFRGAIPVSVFVLWLFYCLVINTAYQSALYGFM
VHPGEIPPIRTIPELDESGLQRKSMFKNASVNYYTSQFTDMLLLYDFCEVTQFDCLIEAGNK
QTQALLSDIDMGMMLSNYWLDQREKPKMVPLKENVATIFLAAQFTPSVCQLFDTASKLL
ESFVETGIHNFLKDRFKYGFNFNNLRHLKKVSHSLSLWHLGGIFYLLLVGLLLSFFVFILEM
FSQFQNRFTHNFI
>BgerIr920P
MNFFTICLWATIFDTSYCRLQPLNDYKSMERYMSECGLNIVRNYFDTKLPIAVQTPGMW
HIDPDLETPGDILLQILNHLNRIPLLTVGPKTIKGSKITNKVKPGSYIVLLPSSICNSQWRLE
KMFRGFYYDMHNARGRLIIMASSCPKDDDELMDFLTCALGMALTAGYMNAIALIPGKGP
TKSSREYNISKIEIFGWLAEEQTNIHSWRIDMINFFDDFNIHKRVFRLKENLFPIKGELSQK
TALSKXFMEQYAKETNCELVYAVHAPYHVAFPIMYGTHFTPRECEFTYPYDGVSFAWYVP
YGVKIPPWTSLIRALSPKMWLLVLISSAFGTISLLVLHRLKKINIGEDTGTKMILYAIGTHLG
VGVNARYKGPASLLFVSTWLFYCLLINTAYQSSLLGLTITPGYVTPMNTKQEIIDSKMKMV
SNIIFQDHIRHDETTFYTYCNNTCIEENLEKRDRAVFSFERQSRLDFLRDLEGHPYYKRLSE
TDMRYYTSMQVLGLSCLIYDKLNLLSHRAFVSGLYLKQTKDSRIREEIMYSSWAKVESVLA
LKPWHLQGAFYLLAIGLILGFLAFIFENIKMLNLIRCFTVK
>BgerIr921
MMNSAPEACICLLLTVLPWTTFSSLEMPSSISVLETEFINCVLDICNKYSNMYEGVVVTSH
NLILTKEVKRGNIPSYDFITEAMLHEMNKSENLTSFVSAERVKDYQHLCKGGLTHFAGFIF
AGVFIMDEHNFVGHNNSFQINMMSCLLWHNPDSFFIIVDTRKNIPPGSHELIFSMLLVNCK
FVNVVILSDTHDVKYERPIIAVKGWFPLRNGNITSGWITSTTTLSYYWRSEFGEKQNSFAN
VDLFPSKRVNNFEGSTVFMKPDAEGIPHIFVNVDENTQLLTASGPLAYFLEIAANRLNFTV
KYSSRINNEIMTGIKGPVFLWSTKRDSDFIHPYGMGGFTWYIPTCRRLPYWQSIIRVFSSKL
WIMVLSSYLIISFVLWIFESANLNRSRSDSETISNILIYTMSPLLGNSGNIHFKKVVSILLFSL
WLFYSLQINTAYQSSLVGYLTNPGELPLLKTIKELESSGIELTMVAQEEESFDGLIDILKDEG
MRTNGIKRKSKLGSNLNTFLETGCNVAFLAVTPMHDYIVYMSGFYNGKPLFIAMNKGIAY
SNLALRCFKGSFLWEPLDEIFFQLQSSGIFNKWVQDLYENDTIKREHQTSAHLIALSLKNL
QGAFYILVIGLAVAFFAILYELILGTDSTKEIRDWKVEENM
>BgerIr922
MMKIISIMYAIMLFVKLPETAFSSLNIPSSISSLETEFVNCILEICRQHSTKFQGLVVTSEYLIL
RKSGKLGNSHPYEFIAENLLYEINNSQNLTIISNVERNSNPILCFGNSYFPDYNNVLGIFIIDT
IDPEENFYALFFAMMSCLFWRNPDSFFIIVDMRGDIPPGSHETIFSYLWNPIKAVNVIIVHV
HYKSPTIAVHGWIPSKNINYTNWKITSTSILNYYNMSSSNEKGHFFYDTDIFPPKQINDFQ
GNTLVVKPDPNIEPLIFQNMDENSTFLSFTGPLIYFLETVSTRYNFKILFTLDEISSSIGNIYG
PFMLTESIKMYNVHNLYPICMGGYLWYVPMCRMLPYWSSIIRIFRFELWMIVLLSYLFTSF
TLCLLESKSQRNSSNNSTVSTDIAIVLINTMLPLIAHSGNLDFKKSVSTLLFSIWMFYSLQIN
TAYQSSLVGYLTNPGNLPPLQSIKDLDTSDVDLIMVVQEDSVFDIFSGILRELGMTRVNDIK
RTYKLGRNLSHFIESGCNVAFLAINPENDYVIYTNGFYNGKALFIPMEKEIMYGNIGFSAKN
GRFLYKHLNRIFLRLQSSGIYNKWIQDIYKNGTNKRAHEYQKNLIALSLKHLEGPFYFLALG
LAISSSVVIFEIIFGLYNRRNNKIVIKRRSHTVTVRYRRRVPAGNYFY
>BgerIr923P
MNLHYLVLDNTTYIKTNLVKYLLFSTMKLVSIIYTFFLLTTLQGTQFSSLKIPSSISSLETAFI
NCILEICRKHSTTFQGLVVTSINLIRKTEKFEKSNPYDFIAEQLLYEINDSQNLTIFTNVERM

TNAGLCFANASNFTDRNNLAGVFILDTRDNEENFYMSFTSMVTCLFLRNPDSFFIFVDMR
ESILPGTYELIFSNIWLQWKAVNVIVLSHASAKMYDFPIIIVLGWLPSKNIDYTDWQITSMS
TLNYYICSSLSEKGHFFYDTDIFPSKQINDFHGNIVLVKPDPNSEPFIFQDMNEIQLFSVTGP
LINFLETAAKILNFKIIFTQENRSSSIGIIYGPHILIDLEKKYSVKNYPIGMGGYLWYVPICRKL
PYWHSIIIMFRFELWMIVLLSYLFTSFTLCLLESKSQRNSSNNSTASTNIVIVLINTMLPLLA
QSGNLDLKKSVSTFLFSIWLFYSLQINTAYQSSLVGYLTNPGYLPPLQSIKDLDSSDVELIMV
VQKDTVFDIFSNILRELGMTRVNEIKRTYKLGRNLSHFIENGCNVAFLAATPEIDYIIYTNG
FYNGKALFVPMEKXIMFSNIFFGVFNGSFLHKPLNNIFLQLQSSGIYSKWMQDTYENATNK
FAHQYEEHLIALSLKHLQGALYFFILGLAFSTSAVILEIVFSFYNSPT
>BgerIr924N
VLLIILLVSTGHHTRQPPSDAKCISEAALHFAPEGSTLVFLRQLYDKYKSRIRILYEKLAMKT
EAMNVTSDVCPTITTQIKTFKSNDLLTSIQRKNMFEMILQPFSCKQDKEPIYEVLATLHSY
NKWPILIPEYEKQVFNYSQADRRVFFVVMERLIESNEFRGMIELIRFMKLGGPNNLFIIVIT
QEYATQVFQVRTLFQELFRFTLRNVIAIIRLDRNVLMAYTYTFGKPSHHESPCTMKIQILKI
GRCHLSIGWTIFAAPKNIGGATYWIKCSFLTNVLPMNTILMHQPDENLNDRRAANDKELL
VTGLIPLALRTCINKLKRGERIEFVDYRHSVTKKQRSIVLLTSDDMPQAYKLEEFNNGQLW
IYFYTMKYNWYVKNAESYPRWTSIVRVFSPSTWVCFFTSVFVAAMIFRVLKQNLSFAESLS
DAWATVIGVAVTKMPSTAESRIFFMCWIAYSLAINTVFQTFVVSYFFNPGLQYQINSYKEM
MDLNYSLVNTMQQPFQYHLTGEEYHLGDPIASYSFLSTNPNSAAFLNEELLLIYLQLFCNA
SSVSDIFKTREQEQVHVYLISSNYPIMHTMSRMIIRMREGGILAQIVRHFLLHTEITHARTL
FGAEEYEDMSLVHAQSLVVVYCMGNAFSVLVFICELLKKSNFVTHS
>BgerIr925N
VFLLILMASTGHQKRHLTSEVKCISEAALHWVPEGSTLVFLRQLYDKYKFSYRFTEESVNS
EVINITSDVCPILTEMEIINKSAMHVSRKDLVELTLQPFSCKQDKGPIYKTLATLHSYNRWP
ILIPEYEKQEFDYSDADRRVFFVVMERLIESNEFRGMLDLVRFMNLGSPNNLFIIIITEQYA
NQVFQVHNIFEMLLDLYMHNIIVIVRHDRNVLMAYTFSFGKPSPRQSPCTMKIQFLNIGRC
HYHNGWTILAPPKTIHEVTYWTRCCFTTNIFTSNLMLMNQPDEDLNDRLAATDEPLILT
GLTPVMLVTAIKHLKPDEKVEFIDRRYSWVKKQKSIILGMLDNTGQPYMLDPLNNGQLWI
YFYTMKYNWYVKKAEPYSRWTSIIRVFSPSTWVSFFTSVFVAAMIFRVLKQNRSFAESLSD
AWATVIGVSVNRMPYTVATRVFFMCWIMYCLAINTIFQTYVVSYFFNPGLQYQINSYKEM
LDLNYSFVNTMNNPFQYHLTGEKYQFGDPIACYEFLSAYPNSAAFLNEELLLLQLKFFCNK
SSVSDIVKTQEQEQVHIYLISVNYPIMHSVKKIITRMREGGILENTVKHLLQYSEIFYPTASF
RGEEYQDMSLIHAQSVFVAYGMGNAFSILVFICERLIKKVKLI
>BgerIr926NP
VLLITLMVSTGYQTRQQMPEVKCISEAALHWVPEGSTLVFLRQLCDKYKFSFKFKTZMED
TDVTNVTLDFCSATSTIFNYNKSLLTLSRKNIAELTLQPFTCKQDKDPIYQVLATLHSYNR
WPILIPEYENQEFNYSDADHRVFFVVMEKLNEEIEFRGIVDMMRFMNLGSPNNLFIIVITQ
QYINQVFQILKMFEILVRFSIHKVIVVVRHNHNILMAYKFSFGKPSHYQSPCTLKLKIFNIGG
CHFRNSWKILAPPRKIYERMLWTTCYFLTSSFTSNSILILQPNAELNNQFASKDEALDVTG
LLPRVLFTAIHYIKLGEHLYFIDERHSKYKKERSIMVGTSDTLGQNYILEEFNNGRLWTYFY
TMNYNWYIKSAESYPXWTSIIRVFSPSTWVSFFTSVFVAAMIFRVLKQNRSFTESLSDAW
ATVIGVSVTRMPRTVATRVFFMCZIMYCLAINTIFQTFVISYFFNPGLQYQKKSYKEMKDL
NYSFVNTLLQPFHFHIIGEKYHHENPIACYSYLSAYQNSAAFLNKELLLLHLQWYCNVSSVS
DIFKIQEEYQVHIYLFSNNFPVMKTVSKLTTRMREGGILAXDDTRLLWYSDIVYRKTAFRG
EEYQEMSLIHAQSLFVVYGMGNAFSILVFFVNY
>BgerIr927N

VFLLTLMVSTGYQKRQPTTEVKCISEAALHWVPEGSTLVFLRQLYDKYKFSYTITEKSVNS
EVINITSDVCPIMTEMEIINASAMSESRKDLVELILQPFSCKQDKGPIYKTLAMLHSYNRWP
ILIPEYEKQEFNYRDGDHRVFFVVMERLIERNEFRGMLYLIRFMNLGSPNNLFIIVITQQYA
NTVFQVRNIFEMLLDLYMHNIIAIVRHESNVLMAYTFSFVKPNPRQSPCTMKIQFLNIGRC
HLHNGWTILAPPTTIHEVTYWTRCYFLTNVFTTNLILMNQPNEDLNDRLAAIDEPLILTG
LAPFMLVTSIKHIKRDEKVQFIDQRYSLVKRKKSIIFGTSENIAQPYMLDALNNGQLWTYF
YTMKYNWYVKKAEPYPRWTSIIRVFSPSTWVSFFTSVFVAAMIFRVLKQNRSFGGSLSDA
WATVIGIAVTRMPRMVATRVFFMCWITYSLAFNTIFQTFVVSYFFNPGLQYQINSYKEMK
DLNYSFVNTMDEPFQYHLTGEKYQFGDPIACYEFLSAYPNSAAFLNEELLLLQLLIFCNKSS
VSDIVKTQEQGQVHIYLISVNYPIMHSVKKIITTMTEGGILDHTVKHLLQYSEILYPTASFRG
EEYQDMSLIHAQSVFVAYGMGNAFSTLVFICELLIKKSN
>BgerIr928NP
VFLVSLMVLTGYQKRHPTSEVKCISEAALHWVPEGSTLVFLRQLYDKYKFSFKFKAGMEII
DTDVTKVTLYFCLGTSTIFNYNKSLLTLSRKDIAELTLEPFTCKQDKDPIYEALATLHSYNR
WPILIPEYEKQEFNYSDTDHZVFFVVMEKLNEEIEFRGIVQMMRFMNLGSPNNPFIIVITQ
QYINQIHQXFSIHKVIVVVRHNHNILMAYKFSFGKPSHYQSPCTLKLKIFNIGGCHFRNSWK
ILVPPRKIYETMLWNTCYFLTSFFTSNSMLMLQPNAELNNQFASKDEALDVTGLLPRALS
TAIHYIKPGKHLYFIDERHSKYKKERSIMVGTSDTIFZSYILDALNNGZLRTYFYTMNYNW
YVKRAEPYPRWTSIIRVFSPSTWVSFFSSVFVAAMIFRVLKKNRSFAESLSDAWATVIGVS
VTRMPRMVTTRVFFMCWIMYCLAINTIFQTFVVSYFFNPGLQNQINSYKEMLDLNYTFVN
TLLQPFHFHIMGEKYHHGNPIACYSFLSAYQNSAAFLNKELLLLHLQWYCNVSSVSDIFKIZ
EEYQVHIYLFSNNLPVMKTVSKLTTRMREGGILAKMITQFLZYSDILDRKTACRGEEYQEM
SLIHAQSLFVVYGIGNAFSILVFICELLKKKIKLI
>BgerIr929N
VFLLTLMVSTGYQKRQQTSEVKCISEAALHWVPEGSTLVFLRQLYDKYKFSYTSTEKSVNS
EVINITSDICPITTEIQIINASDMTESSKDFGELNLQPFSCKQDKGPIYKTLAMLHSYNRWPI
LIPEYEKQVFDYSDADHRVFFVVMERLIESNEFLGMIDLIRFMNLGSPNNLFIIVITQQYTN
HVFQVYGIFETLLDLYIHNIIVIVRHNRNVLMAYTFSFGKPSSHQSPCTLKIQFHNIGRCHL
HNGWTILAPPTIHEVTYWTRCYFLTNIFTTNIMLMNQPDEDLNDRLAAIDEPLILDGLTP
VMLMTSIKHLKRDAKVEFFDERYSLVKKQKSIIFGTSDNIPQSYMLDVVNKGQLWTYFYT
MKYNWYVKRAEPYPRWTSIIRVFSPSTWVSFSSSIFVAAMIFRVLKQNRSFAESVSDAWA
TVIGVSVTTMPRTVATRVFFMCWIMYCLAINTIFQPFVVSYFFNPGLQYQINSYKEMKDH
NYSFVNTMEEPFQYHFTGEKYQYGDPIACYSFLSAYPNSAAFLNKELLLLYLQWYCNVSSV
SDIVKTQDQGQVHIYLISVNYPIMHSVKKIITRMTEGGILDEMVKHQMQYSEIFYPTASFRG
EEYQDMSLIHAQSVFVAYGMGNAFSILVFICELLIKKVKLI
>BgerIr930NP
VFLITLMVSTSYQKRQPTSEVKCISEAALHWVPEGSTLVFLRQLYDKYKLSFRFNAGPIRID
KDYTNITSNFCSGTSTIINYNKSLMTVTRKNIAELTLQPFTCEQDKDPIYEVLATLHSYNR
WPILIPEFENQEFNYSDADHRVFFVVMEKLNEEIEFRGIVEMIRFMKLGSPNNLFIIVITQQ
YTNQVIQIQKMFEILVRFKLHKVIVVVRHNHNLLGAYKFSFAKPSHYQSPCTLKIQIFNIGG
CHFRNSWKILAPPKKIHETTYWAKCYFLINLYTTNALLLIQPYIELNNQFASNDEPIIYVAG
LMPAVLTTAIDYLKPGEDIKFIDERHSKYKKERSIMVGTSDTISQNYILEEFNNGRLWTYFY
TMNYNWYVKRAEPYPRWTSIIRVFSPSTWVSFFSSVFVAAMVFRVLKQNQSFPESLSDAZ
ATVIGVAVTRMPRTVATRVFFMCWITYSLAINTIFQTFVVNSFFNPGLQYRIISYQEMKDL
NYSFINSMIRPFHYHIIGEKFLSGNSLACYSFLSTYPKTAAFLNKELLLIFLRVLCNESSVSVI

FKIQEEHQVHIYLFSNNFPVMKSVSKMITRMTEGGIVDNMVTQLVYSDNLLQKTSLIRGEE
YQEMSLIHAQSLFVVYGMGNAFSILVFICELLNKKIKLI
>BgerIr931N
VFLITLMVSMGYQKRQSTSEVKCISEAALHWVPEGSTLVFLRQLYDKYKFSTTFTEKSVNS
EVINITSDVCPITTEMEIINASAMHESRKDLVDLNLRPFSCKQNKGPIYKTLATLHSYYRWP
ILIPEYEKQEFNYSDADRRVFFVVMERLIESKEFRGMLDLIRFMNLGSPNNLFIIVITQQYA
NTVYQVHNIFEMLLDLYMHNIIVIVRHDPNVLMAYTFSFGKPSPRQSPCTMKIQFLNIGRC
HLHNGWTILAPPKIIHEVTYWTRCYFRTNIFTTNLMLMNQPDEDLNDRLAAIDEPLILNG
LTPVMLMTSIKHLKPDEKVQFIDERYSWDKKQKSIIFGTSDNIAQPYTLDALNNRQLWTY
FYTMKYNWYVKRAEPYPRWTSIIRVFSPSTWVSFFTSVFVAAMIFRVLKQNRSFAESLSD
AWATVIGVSVNRMPYTVATRVSFMCWIMYCLAINTIFQTYVVSYFFNPGLQYQINSYKEM
LDLNYSFVNTMNNPFQYHLTGEKYQFGDPIACYEFLSAYPNSAAFLNEELLLLQLKFFCNK
SSVSDIVKTQEQEQVHIYLLSVNYPIMHSVKIIITRMTEGGILDHTVKHLLQYSEILYPTASF
RGEEYEDMSLIHAQSVFVAYGMGNAFGILVFICERLIKKVKLI
>BgerIr932N
VFLITLMVSTGNQTRQQTSEVKCITEAALHWVPEGSTLVFLRQFYDKYKFSYTITEKSINSE
VINITSDVCPIMTEIEIIKTSAMSESRKDLVELILQPFSCKQDKRPIYKTLAMLHSYNRWPILI
PEYEKQEFDYSEADRRVFFVVMERLIESNEFLGMIDLIRFMNLGSPNNLFIIVITQQYANQV
FQVHNIFEMLLDLYMHNIIVIVRHDRNVLMAYTFSFGKASPRQSPCTMKIQFLNIGRCHLH
NGWTILAPPKIIHEVTYWTRCYFRTNIFTTNLMLMNQPDEDLNDRLAAIDEPLILDGLTP
VMLMTSIKHLKPDEKVQFIDERYSWDKKQKSIIFGTSDNIAQPYTLDALNNRQLWTYFYT
MKYNWYVKRAEPYPRWTSIIRVFSPSTWVSFSSSVFVAAMIFRVQKQNRSFAESLSDAWA
TVIGVSVTRMPRTVATRVFFMCWIMYCLAINTIFQTYVVSYFFNPGLQYQINSFKEMKDLN
YSFVNTMHDPFQYHLMGEKYQFGDPIACYSFLSAYPNSAAFLNEELLLLQLKFFCNKSSVS
DIVKTQEQEQVHIYLLSVNYPIMHSVKKIITTMTEGGILDHTVKHLLQYSEILYPTASFRGE
EYQDMSLIHAQSVFVAYGMGNAFSILVFICELLIKKVKLI
>BgerIr933NP
VFLVSLMVSTGYQKRQPTSEVKCISEAALHWVPEGSTLVFLRQLYDKYKFSFKFKTGMEIV
DTDVTNVTLDFCSGTSTIFNLNKSLVTLSRKNIAELTLQPFTCKQDKDPIYQVLATLHSNN
RWPILIPEYEKQEFNYSDADHRVFFVVMEKSNEEIEFRGIVEMMRFMNLGSPNNLFIIVIT
QQYINQVYPILKMFEIFVRFSIHKVIVVVRHNHNILMAYKFSFGKPSHYQSPCTLKLKIFNIG
GCHFRNSWKILAPPRKLYETMLWNTCYFLTSFFTSNSMLMLQPNAELNNQFASKDEALD
VTGLLPRALSTAIHYIKPGKHLYFIDERHSKYKKERSIMVGTSDTLGZNYILEEFNNGRLWT
YFYTMNYNWYVKRAEPYPRWTSIIRVFSPSTWGLIFHICLCCRYDLQSHEPNQSFRESLSD
AWATVIGVTVTRMPRTVTTRVFFMCWIMYCLAINTIFQTFVVSYFFNPGLQNQINSYKEM
LELNYTFVNTLLQPFHFHIMGEKYYHGNPIACYSFLSAYQNSAAFLNKELLLLHLQWYCN
VSSVSDIVKTQDEYQVHIYLFSNNLPIMKTVSKLTTRMREGGILAKMIIQFLWYSDILYRKN
AFRGEEYQEMSLIHAQSLFVVYGMGNAFSILVFICELLKQKIKLI
>BgerIr934P
IYFINTLTMYFKYIEFFETLLDLYIHNIIVIVRHNRNVLMAYTFSFGKPSPHQSPCTLKIQFL
NIGRCHLHNGWTILAPPTIHEVTYWTRCYFLTNIFTTNIMLMNQPDEDLNDRLAAIDEPLI
LTGLAPVMLMTSIKHLKRDAKVEFIDERYSLVKKQKSIIFGTSDNIPQSYMLDVVNKGQLW
TYFYTMKYNWYVKRAEPYPRWTSIIRVFSPFTWVSFSSSIFVAAMIFRVMKQNRSFTESLS
DAWATVIGVSVTRMPCTVATRVFFMCWITYSLAINTIFQTYVVSYFFNPGLQYQINSYKEM
KDLNYSFVNTMHDPFQYHLTGEKYQYGDPIACSSFLSAYPNSAAFLNKELLLLYLQWYCN

VSSVSDIIKTQDQGQVHIYLISVNYPIMHSVKKIITRMREGGILDEMVKHQMQYSEIFYPTA
SFR
>BgerIr935NP
VFLLILMVSTGYQTRQQMPEVKCISEAALHWVPEGSTLVFLRQLYDKYKFTFRFTVGVHR
DETDYTNITSDFCSGRQXFNYNKSLMTLSRKNIAELTLQPFTCKQDKDPIYEVLATLHSYN
RWPILIPEFEKQEFNYSDADHRVFFVVMEKLNEEIEFRGMVEMILFMNLGSPNNLFIIVIT
QQYINQVFQIQKMXLVRFSMHKVIVVVRHNHNILMAYKFSFAKPSHYQSPCTLKIQIFNIG
GCHFRNIWKILAPPRKIYVTTYWAKCYFLINVFTPNLLLLVQPNTELNDLFASNDEELHVT
GLMPAVLSTAIDYIKSGEKTHFFDERHSKYKTERSIMVGTSDTLAQNYILEEFNNGRLWTY
FYTMNYNWYVKRAEPYPRWTSIIRVFSPFTWVSFFSSVFVAAMIFRVLKLNRSFPESLSDA
WATVIGVSVTRMPRTVATRVFFMCWITYTLAINTVFQTFVVSYFFNPGLQYQINSYQEMK
DLNYSFINSMIRPFHYHLTGEKYLNGNSIACYSFLSAYPKTAAFLNKELLLIFLQVFCNVSEV
SDIFKIQEEHQVHIYLFSNNFPVMKTVSKMTSRMREGGILDSMITRLLYANILDPKTSLIRE
EEYQEMSLIHAQSLLVVYGMGNAFSILVFICELLNKIKLI
>BgerIr936NP
VLLLILMVSTGYQKRQPTSEVKCISEAALHWVPEGSTLVFLRQLYHKYKFSFRFNAGAIRV
DKDYTNITSDFSSVTSTIFNYNKSLMTVTRKRIAELTLQPFTYKQDKDPIYEVLATLHSYNR
WPILIPEYEKQEFNYSDADHRVFFVVMXKINEEIEFRGIVEMMRFMTLGSPNNLFIIVITQQ
YINQVFQIQKMFEIFVRFSIHKVIVVVRHNHNILMAYKFSFAKPSHYQSPCTLKIQIFNIGGC
HFRNSWKILAPPKKIHETTYWAKCYFLINVFTPNLLLLIQPNAELNELLAANDEQLLVTGL
MPAVLSTDIDYINPGEKTHFFDERHSKYKTERSIMVGTSDTIAQKXWQIMDLFLYNHIQLV
RQKGZALSSLDQHHPSVFTFHTSFFVAATIFSPEQNRSFPESLSDTWATVIGVSVTRMPRT
VATRVFFMCWIMYCLAINTIFQTFVVSYFFNPGLQYQIXSYKAMLDLNFTFVNTLLQPFHF
HIMGEKYHHGNPIACYSFLSAYQNSAAFINKELLLLHLQWYCNVSSVSDIVKTQDEYQVHI
YLFSNNFPIKKTVSKLTTRMREGGILAKIITLLLWYSDIVDQKTSFIRGEEYQDMSLIHAQSL
FVAYGMGNAFDILVFICELLNKIKSI
>BgerIr937NP
VLLLILMVSTGYQKRQPTSEVKCISEAALQWVPEGSTLVFLRQLYDKYKFSYTITEKRVNSK
VINITSDVCPIMTEIEIIKTSAMSESRKDLVELTLQPFSCKQDKGPIYKTLSTLHSYNRWPILI
PEXMKTSDADRRVFFVVMERLIESNEFRGMLDLVRFMNLGSPNNLFIIVITQQYANTVFQ
VRNIFEILLNLYMHNIIVIVRDDRNVLMAYTFSFGQPSPRQSPCTMKIQFLNIGRCHLHND
WTILAPPTTVHEVTYWTRCCFLTNVFTTNLMLMNQPDEDLNDRLAAIDEPLILTGLAPV
MLMTSIKHLKRDEKVQFIDERYSSVKKQKSIIFGTSDNIPQSYMLDALNNGQLWTYFYTM
KYNWYVKRAEPYPRWTSIIRVFSPSTWVSFFTSVFVAAMIFRVLKQNRSFAESLSDAWAT
VIGVAVTRMPRTVATRVFFMCWITYCLAVNTVFQTFVVSYFFNPGLQYQINSYKEMKDLN
YSFVNTMEEPFQYHLTGEKYQFGEPVACYEFLSAYTNSAAFLNEELLLLQLLIFCNKSSVSD
IVKTQEQDQVHIYLISVNYPIMRSVKKIITRMREGGILDNTVKHLLQYSEIFYPTASFRGEEY
QDMSLIHAQSVFVAYGMGNAFSILVFICELLIKKSN
>BgerIr938NP
YDKYKFSFKFKTGMEIVDTDFTNVTLDFCSGTSTIFNLNKSLVTLSRKNIAELTLQPFTYKQ
DKDPIYEVLATLHSNNRWPILIPEYEKQEFNYSDADHRVFFVVMEKSNEEIEFRGIVEMM
RFMNLGSPNNLFIIIITQQYINQVYQILKMFZIFVRFSIHKVIVVVRHNHNILMAYKFSFGKP
SHYQSPCTLKLKIFNIGGCHFRNSWKILAPPRKLYETMLWNTCYFLTSFFTSNSMLMLQP
NAELNNQFASKDEALDVTGLLPRALSTAIHYIKPGEHLYFIDERHSKYKKERSIMVGTSDT
LGZNYILEEFNNGRLWTNFYTMNYNRYVKRAEPYPCWTSIIRVFSPSTWVSFFSSVFVAA
MIFRVLKQNRSFAESLSDAWATVIGVSVTRMPRMVATRVFFMCWIMYCLAFNTIFLTFVV

SYFFNPGLQYQINSYKEMLDLNYTXVNTVLQPFHFHIMGEKNHHGNPIACYSFSVSDIFKI
QEEYQVHIYLFSNNFPVMKTVSKLTTRMREGGILAKMITRLLWYSDILDRKTAFRGEEYQ
EMSLIHAQSLFVVYGMGNAFSILVFICELLNKKIKLI
>BgerIr939
MEPKFPNQLAKVLLLILMVSTGYQKRQQTSEVKCISEAALHWVPEGSTLVFLRQLYDKYK
FSYTITEKSINSEVINITSDICPIMTEIEIINASAMSETRKDLVELILQPFSCKQDKGPIYKTLS
TLHSSNRWPILIPEYEKQEFNYRDADHRVFFVVMERLIERNEFRGMLSLIRFMNLGSPNN
LFIIVITQQYANTVFQVRNIFEILLTLYMHNIILIVRHDPNVLMAYTFSFVKPSPRQSPCTMK
IQFLNIGRCHLHNGWTILAPPTTVHEVTYWTRCYFLTNVFTTNLMLMNQPDEDLNDRLA
AIDEPLILTGLAPVMLMTSIKHLKPDEKVQFIDQRYSLVKRKKSIILGTSENIAQPFMLDAL
NNGQLWTYFYTMKYNWYVKRAEPYPRWTSIIRVFSPSTWVSFFTSVFVAAMIFRVLKQN
RSFGGSLSDTWATVIGVAVTRMPRTVATRVFFMCWITYSLAINTIFQTFVVSYFFNPGLQY
QINSYKEMLDLNYSFVNTMPYPFQYHLTGEKYQLGDPIACYEFLSAYPNSAAFLNEELLLL
QLKFFCNKSTVSDIFKTQEQGQVHIYLISVNYPIMHYVKKIITRMREGGILDEMVKHQMQY
SEIFYPTASFRGEEYQDMSLIHAQSVFVAYGMGNAFSILVFICELLMKKVKLI
>BgerIr940N
VFLLLILMVSTGYQKRQHTSEVKCISEAALHWVPEGSTLVFLRQLYDKYKFTFRFTVGVHRD
ETDYTNITSDFCSGTSMIFNYNKSLMTLSRKNIAELTLQPFTCKQDKDPIYEVLATLHSYN
RWPILIPEYEKQEFNYSDAVHRVFFVVMEKLNEEIEFRGIVEMMRFMNLGSPNNLFIIVIT
QQYINQVFQIQKMFEILSRFSIHKVIVVVRHNHNILMAYKFSFAKPSHYQSPCTLKIQIFNIG
GCHFQNSWKILALPRKIYETTYWAKCYFLIDVFTPNLLLLIQPNAGLNDLFASNDEALHVT
GLMPAVLSTAIDYMKPGEKTHYFDERHSKYKKERSIMVGTSDTIAQNYILEEFNNGRLWT
YFYTMNYNWYVKRAGSYPRWTSIIRVFSPSTWVSFFSSVFVAAMVFRVLKQNRSFAESLC
DAWATVIGVSVTRMPRTVATRVFFMCWITYTLAINTIFQTFVVSYFFNPGLQYQIKSYQE
MKDLNYSFINSMIRPFHYQLTGEKYLNGNSIACYSFLSAYPKTAAFLNKELLLIFLQVFCNV
SEVSDIFKIQEEHQVHIYLFSNNFPVMKTVSKMTSGMREGGILDNMITQLLNSNILDQKTS
LIRGEEYQDMTLIHAQSVFVVYGMGNAFSILVFICELLNKKMKVI
>BgerIr941N
VFLITLMVSTGYQKRQPTSEVKCISEAALHWVPEGSTLVFLRQLYDKYKFSFRFNAGPIRID
KDYTNITSNFCSGTSTIINYNKSLMTVTRKNIAELTLQPFTCEQDKDPIYEVLATLHSYNR
WPILIPEYEKQEFNYSDAVHRVFFVVMEKLNEEIEFRGIVEMMRFMNLGSPNNLFIIVITQ
QYINQVFQIQKMFEILSRFSIHKVIVVVRHNHNILMAYKFSFAKPSHYQSPCTLKIQIFNIGG
CHFRKSWKIIAPPRKVLETTYWTECYFLINLYTTNALLLIQPYIELNSQFASNDEPIIYVAGL
MPAVLTTAIDYLKPGEDIKFIDERHSKYKKERSIMVGTSDTISQNYILEEFNNGRLWTYFYT
MNYNWYVKRAEPYPRWTSIIRVFSPSTWVSFFTSIFVAAMIFRVLKQNRSFPESLSDAWA
TVIGVSVTRMPRTVATRVFFMCWITYSLAINTIFQTFVVSYFFNPGLQYQINSYQEMKDLN
YSFINSMIRPFHYHINGEKYLGGNSLACYSFLSAYPNTAAFLNKELLLIFLRVLCNVSSVSDI
YKIQEEHQVHIYLFSNNFPVMKFVSKMITRMTEGGIVDNMVTQILHSDNIVQKTSLIRGEE
YQEMTLIHAQSLLVVYGMGNAFSLLVFICELLIKKIKLI
>BgerIr942NP
VLLLILMVSTGYQKRQPTSEVKCISEAALHWVPKGSTLVFLRQLYDKYKFSYRYTEZSVNS
EVINITSDVCPIMTEMEIINTRAMHASRKDLVELTLQPFSCKQDKWPIYKTLATLHLYNR
WPILIPEYDKQVFDYNDADHRVFFVIMERLIESNEFRGMLDLIRFLNLGSPNNFFIIVITQQ
YANQVFQVHNIFEMLLDLYMHNIIFIVRHNRNVLMAYTSFGKPSPRQSPCTMKIQFLNIGR
CHLHNGWTILAPPKTIHEVIYWTRCCFTTNIFTPNLMLMNQPDKDLNDRLAAIDEPLILT
GLTPFMLVTSVKHLKPDEKVEFIDRRYSWVKKQKSIILGMLDNSGQHYMLDALNNGQLW

IYFYTMKYNWYVKKAEPYPRWTSIIRVFSPSTWVSFSTSVFVAAMIFRVLKQNRSFAESLS
DSWATVIGVSVTRMPCTVATRLFFMCWIMYCLAINTIFQTYVVSYFFNPGLQYQINSYKE
MLDLNYSFVNTMKNPFQYHLTGEKYQFGDPIACYEFLSAYPNSAAFLNEELLLQQLKFFC
NKSSVSDIKTQDQEQVHIYLLSINYPIMHSVKKIITRMREGGILGEMVKQQLEYSEIYYPTSY
YR
>BgerIr943N
VLLLILMVSTSYQKRQPTSEVKCISEAALHWVPEGSTLVFLRQLYDKYKFSFRFNAGAIRID
KDYTNITSDFSSVTSTIFNYNKSLMTVTRKKIAELTLQPFTYKQDKDPIYEVLATLHSYNR
WPILIPEYEKQEFNYSDADHRVFFVVMEKLNEEIEFRGIVEMMRFMNLGSPNNLFIIVLTQ
QYTNQVFQIQKMFEILGRFRLYKVIVVVRHNHNILGAYKYSYAKPSHYQSPCTLKMQIFSIG
GCHFRKSWKIFAPPRKVLETTYWTECYFLINIYTTNALLLIQPYIELNNQLASNDEPIIYVA
GLMPAVLTTAIDYLKPGEDIKFIDERHSKYKKERSIMVGTSDTISQKYILEEFNNGRLWTYF
YTMNYNWYVKRAEPYPRWTSIIRVFSPSTWVSFFSSVFVAAMIFRVLKQNRSFAESVSDA
WATVIGVSVTRMPHTVATRVFFMCWITYSLAINTIFQTFVVSYFFNPGLQYQINSYQEMK
DLNYSFVNSMIRPFHYQINGEKYLGGNSLACYSFLSAYPNTAAFLNKELLLIFLRILCNVSSV
SDIYKIQEEHQVHIYLFSNNFPVMKLVSKMITRMTEGGIVDNMVTEILHSDNIVQKTSLIRG
EEYQEMTLIHAQSLLVVYGMGNAFSLLVFICELLNKKIKSI
>BgerIr944N
VIYLMCFVHGIRIPLEGIGKCASEAALAFFNKGNLVFFLPDLNSDVQHNKPIIDGGTERKRK
VQFQDRVRFSERFVVFSNILNKLHLQIHWPIVTIKYLPNYTIENPATVNGYVIVLRTVNSLA
AVNIVIETLKRMHLFSSRGHYIFVFTRMTGTITESTVFLEHLAASKVFNVLLVLPVTSNEVQ
TFTWHPYHTSTSSCRSAKQVIMYGNCAVHNNWRFHRKDQHSENINFYSECSVLTLISPFP
PFIFYKENGTVDGFTARILDVISNKMNTRLLYQQSPQGDPDYTFSGIDVSALSNANTMERF
GYKISGVNVSAPRTTNAVRDPWNTDFPAHERVHNFQKTNYKLSKADISAHSKPDSTEEN
QDVERLFTLKYCWFVRRTETYPRWDSMSRVFTFNAWVCIFTSLILAALTFRCLKLYPDFA
ESLLDSWSIFLGIGASKTPEEFSTRIFFLSWVFYSLSVNAVFQIFVVSYFLDPGLHHQTISFPE
ILDNDFILVFDTFDIAYSYLDTVNKDIVTADTTTCIKYLLKNPKVAMFLSVELFEYNIHQHF
NVLEINKMSVLKRYEQQIHIRIQFHNFVLFDRMKGLIARLQEAGLSEKLMRDIINPNGLKH
FSYSAADLEQEYMHMTLTHLHSTFVLYVFGVIFSSVVFMCELVLNSLISWTRKVTAKARFI
QVTCCGYCLQYFR
>BgerIr945N
ILRLVPVLCTACSIDLLSEERTENLAKCIVDVSNAYFTDGDTLLYLSPIKDLSTKGRSVADVL
YRKFRHLDLPLEPQETCLSTRKLLSVDKLEDWDTAEYNEYLEHYLLIDYECNFTYESSKTK
YVDTEILRSLHEDSKWPIILQPYSDGWYKFSGIVKGYVLTIQKNESLSNIENFCLMFDYSPA
LSKDSNILVIVLSGWLEEFEVYKIMNVFVKRHIHYIILLCYNKNDTFNIFTWSKLKKTLFGS
DVVDRLNEIGKCSSTFGLIKDKSWNLSKDIVNMEGRPFIHRSQYNPPFSMPFEKEGMAFRL
VGYIAKNMNSSFTGDINLPSRDFHSFHEYYDIDISVLNFDLPSLFQSGKRRIIFFYTLSYVWF
VRTAQPYDRWTSMFRVFTVSNWLLIFFALLITAIIFSFIKKSYQSLESCVQGFHVSITEFSISC
STTKCILEMWSVLLGIGVFDEPTKVSLRILLLTWIVFSLSINTIYQSFVVSYFLDPGSKHQID
TLNELIERNYTLAFDSFRSLLQYANDKRATLYVTSEVYNQEDTFLQSMSQVFNIPNSALLF
SEEIFDYFTQKYCGSGLIEKYHKFTTKSHQEHIFVNVINPLIRSRFSTLMQRLVQGGFPEKI
MRDVTDPCGRFAFMTPKVGIDNYVQMSLSHFKSSMIVYVTGIILAGTAFIWEMVIDVRFST
GIRDYHSNAKSLFDARAN
>BgerIr946NP
ILWLMPVLHITYAMNFQSKETTKKLATCIAQVSSAYFSAGDTVLYSPPIQEFPHFGTRDKSI
DITIRLENIGWEKKHLVGSETCLGEKKSVSLAALHFNESSIQKYINYVPMGPSPLIQYSSYEK

YIDTEILRLLHEEHRWLILLTPYIPNWHMFSGNVNGYILIIHRNELVLNIYNLNSMFEKCPA
LSVDTNIFAIIIGGWLQELEVNGILNELLAQCMRYIIVMFENEYGSYSIFTWTKLKKTFLKL
DVVDKLLQIGICNSEFGLLKNVSWNLTKDISNMDRRPFVHRTQYYHPLTIVERKGVAFQLI
THIAEYMNSSMLTESRIPSRDNLAFNKHYDLDXSVVHYDFNSLFRPGNGRIVFYYSLSYVW
YVQSAQLYERWSSMFRVFTVSNWFLIFLTLFITFIIFNFIRIVYQLLNIYKGECQIDISYVNIT
YTTCKPFLEMWAILLGIGVFAERTKVSLRILFLTWIIFSLSINTIYQSFVVSYFYDPGLQHSY
DEIVNRNYTMLFDSELRALPYLYNNPGSVYIASEZSELERKLVESLNFVLSVPNSAFLFSEE
LFNYYSQKYCKSALLDKIHKFSTKSIQVHSFVNVINPLLRGRFNTLMKRLVQGGFPEKIMR
DVTDPCGRVSFMSANVGVDNYVQLSLLHFQSSLILYAVGVILATITFFLEMIVVIKCGVLHV
LRNLFV
>BgerIr947N
TLWLVAILHITGAANFHSEKPSTKLATCIAYVSNAYFTAGHTVLYSSPIEEFSVLESGENSL
DITMIFENMIGVNKFLEGTETCLSERKEIAVSRLRLNELNIKKYNSYIPTAPSLAIKYSSETK
YIDTEILRILHEEHQWLIIITPYIANWLTFSGNIKGYILIIHKNDYLSNINNLNLMFENCPALS
ADTNIFVIIIGGWLEVSEVNDVMNEFLAEGMRYIVVMCEDKFGTFGIFTWEKLKKSSFQVE
FIDKLVQIGMCSSEFGFLKYDSWNLKRDISNMEGIPLVHRSRDNHPLSRLEKNGVAFRLIT
HIAEYMNSSFSSQNIISQRDSLAFRKYYDLDISVVHYDVLSLFRSGNGRIVFYYSLSYVWFVP
IAHPYDRWSSMFRVFTISNWFLIFLTLLITSIIFNVIEVLYQFLDVYKGKLQIYTENTNLAYT
TSRCILDMWSVLLGIGVFSEPRKMSLRILFLTWIIFSLSINTIYQSFVVSYFFDPGLQHQIDSF
DEILNGNYNMIFDSELRAIPYLTSKSGSVIIGTESNDLERKMLNSINIVVNSQNSAILFSEELF
NYYTQKFCKSTLFDKIHKFSTKSHQVHVFVNVINPLLRSRFSTLMKRLVQGGFPEKIMKDV
TDPCGRFTFMGRKHGVENFVQMSLLHFQSGLMLFAFGIILAVVSFFCEMTLFLQTDFIRAL
RNKFI
>BgerIr948N
TLWLISILHITGAANFHSEEPSTRLATCIAYVSNDYFTAGDTVLYSSPIEEFSVLGYGENSFD
ITMLLENMRGENIYLEGNETCLGARKEMRISSLRLNEQNIQKYNSYIPTAPLLAINYSSETK
SIDTQILRILHEEHQWLIIITPYIADWLTFSGNIKGYILIIHKNDYLSNIYNFNLMFENCPALS
ADTNIFAIIIGGWLEESEVKEVMNEFLAESMRYIVVMCEDKLGMFSIFTWGKLKKSFTKVD
FVEKLIQIGTCSSEFGLLKYDSWNLKKDISNMEDIPFVHRSHYNHPLSRVGNKGVAFRLIT
HIAEYMNSSFSDENTIPQRDWLSFKKDFDLDVSVVHYDVPSLFRSGNGRIVFYYSLSYVWF
VPSAQPYERWSSMFRVFTISNWFFIFLTLSFTSIIFNCIQILYQFLDVYKKEFKVYIDNVNVF
YTTSRCVLDMWSVLLGIGVFSEPTKVSLRVLFLTWIIFSLSINTIYQSFVVSYFFDPGLQHQV
DSFDEIVNGNYNMIFDSELRALPYVDSKPGSVFIGSESNDLERKMLDSINIVVNSPNAALLF
SEELFNYYTQKFCKSTLFDKIHKFSTKSHQVHAFVNVINPLLRSRFSTLMQRLVQGGFPEKI
MRDVTDPCGRFTLMGRKHGVENFVQMSLLHFQSGLILYAFGIILAVVSFFCEKMVFLQTD
FIHAFRNKFL
>BgerIr949N
ILWLMTVFHISGAANFHSEEPSTKLATCIAHVSSAYFTPGDTILYSSPIKEFSLLETGGQTIDI
TKLFEIMSKKTKYLEDTETCLSERKDVRISSLRLNEPNVQKYNRYIPTAPLLALQYYSETKY
IDTEILRVLHEKQQLLIISTPDISNWMVFSGNIKGYILIIHKNDYLSNINNFNLMFENCPALS
ADTNIFAIIIGGLLEESEVNDIMSIFMAEVMCEDKLGMFSIFTWGKLKKSRFKVDIVDKLIQI
GTCSSEFGFLKYDSWNLRKDISNMEGRSLVYRSRYNHPLSRVGNNGVAFRLITHIAEYMNS
SLPSQNIIPQRDSLAFRKYYDLDISVIHYDLPSLFRSGNGRIVFYYSLSYVWFVPIAHPYERW
SSMFRVFTISNWFLIFLTLLITSIIFNSIQILYPFLDVYNGEFHMYATTINVAYSTSRCFLDM
WSTLLGIGVFSEPTKLSLRILFLTWIVFSLSINTIYQSFVVSYFFDPGLQHQVDSYDDIVDRN
YTMLFDSELRALPYMFTNPGSVYISSESNQIERKLLEPINFVVNSPNSAILFSEELFNYYTQK

LCKSSLFNKVHKFSSKSHHVHVFVNVINPLLRIRFRTLMQRLIQGGFPEKIMRDVTDPCGR
FSFMTQKIGVDNYVQMSLLHFQSSLILYAVGIILAVVSFFCETLFWVQPNFIHVIWKMFFLE
CSISLSEWNICKR
>BgerIr950N
TLWLMSIMHITGAANFHTEEPSTKLAKCIAYVSNAYFTAGDTILYSSPIEEFLVLGSREKKI
DITMLFENMRWENKYLEGNETCLSARKEISVSSLHLRELKVRKYNSYIPTAPLLAINYSSET
KYIDSEILRIIHEEHQWLIIITPYIANWLTFSGNIKGYIFIIHKNDYLSNIYNFNLMFENCPAL
SADTNILAIIIGGWLDEFEVNDVLNEFLAECMCYVVVMFEDKFGKFSIFTWAKLKKSRFEV
DIVDKLVQIGTCTTEFGLLKNDSWNLKKDLTNMDRPFIHRSHYNHPLSRVGNKGVAFRLI
THIAEYMNSSFSAENTVPLKDNLSFRKNFDLDISVVHYDLSSLFRSGNGRIVFYYSLTYVWF
VPSAQPYERWSSMFRVFTISNWLFIFLTLFITSIIFNCLQILYQFLDVYKKGFKVHIDNVIVF
YTTSRCVLDMWSVLLGIGVFSEPTKVPLRILFLTWIVFSLSINTIYQSFVVSYFFDPGLQHQV
DSFDEIVNWNYTMLFDSEVRALSYMYTNPGSVYIASESNDLERKLIETMNFVVNSQKSAIL
FSEELFNYYVQMFCKSTLLDKVHKFSTKSHQVHAFVNVINPLLRSRFSTLMQRLVQGGFP
EKIMRDVTDPCGRLSFLTQKIGVDNYVQMSLLHFQSSLILYGVGIILSMASFVCELIHRVP

109 BgerOBP proteins
>BgerOBP1
MRAEFTRDSLKCTLQSDTMRFYGISLAVLSLVTVIQANEEKPFLKMNTIKIITACKQDNKA
QRDDYDAIRKSETPTTENGKCFLACLFDKFQVVQNGAFSVETFSKRLAELYKDDPDKLQK
AMNMANECKTSLNGVQETNKCQYAPKVLECFASYKTKIEVVQDLLKYQKIKSS
>BgerOBP2
MKGPTSAVLFAGLLLFASHEVPVRALEMSDMVSMAPQIISMGVEVIMNCMSENNMSIMK
TGASLMTARTIDQNAKCTVCCITSQIMITDGDKCSVNTAMIDTMVGALPKAVQDIVKKITK
GCTNAGAGKSKCQGLFDYGACIFKEGKNNIGSIFKMG
>BgerOBP3
MLLRGLIVLLGAVYCYAQSGPKEYALQCQKDYNIPDDVFKKIQWNLKATDEQNVNQRCFI
ECMLKAEGTIKNGELDQDFVVEEAKKDLVQVNLTLDEPKFRRSVATCAAQDGQGQCTRS
NNIWKCLADLLSGGLPLVS
>BgerOBP4
MRHLIVVLIAVLSLLVIEVELGTDEMRRIMQECLNQANIRSQDQLDFLKKKSMPVTTEGKC
FLGCLMEGYGYLIDGEFTEDSCEEWATRQGQSHEAAADLFQSCHSTVGDGDDRCETGPLL
WKCLLKLDNNQ
>BgerOBP5
MGGHHVVSLLCIAVMLVTPLSGAGVPDFYQQLPNFMTFLTTSQNVNPILSRVARQTKTSV
PCCGHEKFSWSIITDEFNGCTEEVGDIEKKERVPCFFECAGVKLGICDNEGYLKKDEAVELS
PKFVEGDEALENFVKQLAPKIADTVANIPDDVRKQLKCNIAVYAYLRGLHFHMELMCPIE
MQNPADECEKLRAEYRQMAEQKRG
>BgerOBP6
MTCRTKVFSNFIIILAVLHSIDGFTIEQVKKTANMVKDICIRKTAVDPEKVEQATNGNIPED
DNFKCFTKCLLEMLQAIRGDQYNSDGLIRMIKVLLPTDLGTRAITAIQQCNNAGDGLENIC
EVTYSIVVCFYKTDPEFLSLIL
>BgerOBP7
MKRITATLCVFLLAAAIRNSSAGMSEAQIKQAMKMIRQACQGKNSVSTEMLDGIQQGNFP
DDKNLKCYMKCVMGMMQSLKNGKYKPDAAIAQAKAMLPDEIKDRVIVSMDKCRNSGDG
IDDACELAYVVTKCIYASDPGAFMFP

>BgerOBP8F
MMKLHYILIVSCLFISTPISFAKMPSKGQVLQAIGMLYEHCQEKTNAEPDDISGICDGNFPE
DKVPLKEYTCCVLDMLRVMNGDKFEPDEALDNLKDLPDRVKKHLMEGINQCRNSGKNG
STCAETSYEVVKCFKATLGKYFFFPCGTDELPPEE
>BgerOBP9
MLPVVTGSVVICAILFSHLVVCEVTEDEIEKNCEEKFGVDESIFVDIDGDGNEDAIDEFDITL
KDEENEDHRCFIECLMEGYGYIKNGRLDVDFLVEEVLIELEEEGIEIDVDELRGDIAFCADQ
HAEGKCSTSYTIAKCLFNLQKKRRR
>BgerOBP10
MVSTKTFSLIVVCCLVYTVSAHYSREDDSNTVEDSGSASASRGINTGDKQISVKGSVSWQQ
TITVKQQSGAAPPHHGGHHGHRHHPHYFNMCCMVGTNLTDNPKLMKSLMKCRDEVMA
LFTNNGTFKPSPKNHLKASNCFFQCVGKEMDLVEENGSLKTKETVDFIATDYPLMELGVT
KEQIEACVITSKRSTPAPMPAEGGKVCNLVNFEAARFTKCLMDLKNLNCPTEKRVHTPIC
DHMREQWQRK
>BgerOBP11
MNMRNQIVIVLLSFYGYALCASKQGGLPKKVIEECRKATGVTEAEAQELQENDLVKDESN
EAHRCFVACILSKRGAMKDGKMVVEEAVHAAKDGFKEAGFPFDEAKYRKGLEECNKQTG
EGKCVKSYNAWKCFLSFTSKTMIKSAEDKGK
>BgerOBP12
MLTTLICFLGSLLLAFAKPTFPQSPSTAEMRNDLALIRLCNQTSPISLATMNSVLINKQLSG
VSNAEGFKCFLHCLYNNYNWMDEDGGFMLSNMKGSLEATRLDELSIEFLIYKCTSVDSSD
RCERAFKFTECFWSETKTFPDESAAVDSSDYRIDWAAK
>BgerOBP13
MNNTGLVLALLLLGVSTPHVFAALTMDQMRNAAKMIRNVCQPKTGVATELIDQISEGVF
DEENRDLKCYIKCAMQMMQSMGSNGKLRPDAAISQAKKMLPIEIRDAMIAGIDTCRNVD
KENPGLDPCDLALVATKCVYNHTKDVFLFP
>BgerOBP14
MHRELLVLVLAVVIQLALAELSLEEIKQANEILRKHCQPESGVSDDVLNGAMSGNFPEDRG
LKCYLACMMRLAHVLKNGQYRSELAVRLADDVLPSSIKDRARVVVEKCKDEGAGLADECE
MAFAIKKCSYAADPEIFYVQ
>BgerOBP15
MHNRVLLPALVVILVALLGHTHALIILTQNEGTGKLIRSKCQPESGVSTDLIEGVPKGNFPD
DKNLKCYMKCAMSMTMTMRDDKLRIDIAKIMTERAVPEPNRSRILAAIDKCQNADQGLT
DPCEIAFAATKCIHDADSEVLLFP
>BgerOBP16
MSWDTALFLVLASVLCASAHHHHQEAASSSYGHPLTDRDALLNETRRVHPYFNPRDRER
PLITTCCDLSLNKSSSHTSPPAWVKEKCQLEVIAQFETESPESSSEEDLGLDVMNRNMTVC
LLECYFREMHLSTEDGIMIYDNVLKASVSNGKSQMGVTDRMVERCVVRANIIGPAMSQHK
GYACNFAPVEFEQCILERVDLLCPEYNQIRADLCDRYREKLRRKYTI
>BgerOBP17
MNRVLLASLCLVILATVLRPTLAGMSMDQIRQAMKMMRNSCQNKAKASPELVDGIQQGQ
FPDDRNLKCYIKCAMGMMQSMKGGKLKPEAAIQQAKMMLPDEVKGRVIAALETCRNAA
DGITDACDVAMAGAKCIYDTDPEAFIFP
>BgerOBP18
MEHHLFFLSLVTLFGTLSTLHVPKLDDLSDHDYLHKEVPSYFSKSCCNYNEPNAEDEMNI
FIDCRGIINDKYKLQRPKPTGQDEEDRITMISWIWNRSTCFSYCFLHKQGMANEDGTINVE

TAMKKQKKSIPELVNPEAWTNIEKLCTKPNDDDKETYVCKKDALHFSGCVHDISEFYCPD
ALQSKGESCVKYREDTKKKYGI
>BgerOBP19
MFSFKLKLLLAVVMWVCSADPTTLQEKQNIHKQCQEEYDISDEVFAEMRNTEMILEDES
DIKKKCTVECLLLGYGMIKDGQLDVTSVLDFAKPMLEHIQQEGGEIDEENLKENISKCSDT
VSGEEENCTSSYETWVCLHDVMTKLMAGSKNQE
>BgerOBP20
MVLKRIKLILFLSFISVTFSLKPEEIGISCQQKYKLSGEDIVEMRENRMILKDETNVNQRCFI
ECLMVESNMFVNGELNQEEVKKGEKELKEFYKSHGKELNLEKFDTSIQECSSKDDEGQCM
KSYQMWKCLVSAMPTDVVGGQPLKQQHL
>BgerOBP21
MKEYFVCVYILFIVEVLYLTDAGGEFLWRMKREANITEIKCCMQEGIPTGKRGLRDSIEMR
DIGKICFEEIQNKSSDINLEELNLERRVVFLECQLQCVFDKLKKVNEEGNLKKEETASSMIG
LFMIADRKFGDIFIEKCWKNLEGHAETKVEGHVCNPAAFNIYLCFEDIANLMQNVRITE
>BgerOBP22F
MHLLLPVAIFVVFVGFASAGNPMDSLDDDQKEMLKMLHDSCVGDSGADEGLIAKAIKGDF
TDDGNLKAYMACIFQNLGAMDENGVPDMDTMISMLPASMQERGGKMIGSCKGVTGSDA
ADTAMKLNQCFYNADPGYYFMF
>BgerOBP23
MQLSIELVCLLFAICSVKADSTKSFKNVIGPIARECMTKMDNVTEDDFQAVFNRNPLETRP
AMCLLKCVYQKLGVYKDDGALKEGDDLVPIIERIYGFNDSKNAMRIQAINTCVQKANADR
GEKCEEVAQFVRCVLQNY
>BgerOBP24
MKLPICIVFIFAAISAVSAESTQSFKDAVGPLLRECMGQVDGVTEDDFQTVYNRNKVETHS
TKCLLWCLYKSLGCYLPDGQLKAGDEMMPIIDKLYGFKEFKTVFRAQVVNNCVHEVNAES
SEDCEKAADFLHCLIVHY
>BgerOBP25F
MELQMKLCLGLALFTTIAAESTQSFKDGVEPHLKKCMEKVVDATEDDFNAVYNRNPLET
HSARCLLKCLYGELDALLPDGRIKSGDDLMPIMEKLYGFKEFKTVMRTQIVNQCVDMVNS
GEARDCELASDLIHCVMQHY
>BgerOBP26
MQIIFTVNAAPYFDGLPPEAEKMLKECNETFPIDMGYLKDLNDTGSFHDEDNKTPKCFIR
CVLMKAGLMDEDFTFDAPKLKEAFKDSKYPDMDNMIDMCIAKDTETQCRCTKAYLFIKC
LMSEEITKYGDASKEITKYGDASKVKTSN
>BgerOBP27
MATVGCVCLFLLSTVVSHVQPFFLARTLEKSEANHLIRMKRGSEEAYIDDDYVLVCCDELQ
NKVNNKLTSEDIKIMEECQEEVERKMGERDEMTPIKEYFLNYSTCGNQCLFQKHGWIDEE
GNILKGVSAEIASIQMGITDASLLDSIATTCSAVARDYKENHNANYICNRTAMMFYTCSFD
MVNLHCPTEHLICNDGCDRRMERVRNQYQSLLVRKGSLRFLTL
>BgerOBP28
MLLRQTFTIIFVVTAVAASDPRLAQLDQDTRDLLNMLHDSCIEETGLDESLGWIDKARTG
EFTEDQTFKNYLGCIYMQTGALAEDGEADYDAMLAVLPEQFLERATKMVNQCRHVREN
NAPDTAFVLNKCLYKADPEYYFIF
>BgerOBP29

MDLPCTISFLLITVVCSSFVIAQERGEEFMVKAKECQKKFEISDEIYQYVLKTMQTMDENN
ASQRCLVECLLTAEGFMNNEKLDTEKIVEQTKKESEKVGLKLNENTLRTTINDCKKLGGE
GKCSASYNSWKCFFNMMMKGLPYA
>BgerOBP30F
MKNAIFASSAVFLLIGCQQILAEFSGRGMEKAKEVNAKCMQETGAGQGYFGKFLRGEIEDE
NMNIYKCFVKCVMVELASLSHDGVYDIDEEKQNIPPEILEEGHRIITLCKDTAGSDPCDTAY
MMHKCYHDANPELYRRVLHHWETIAQA
>BgerOBP31
MSSYLILSLISLICVEFVELASVSGNQEIGSETILIRNLRNAEGTHEECCGKGKTQFTEEEKK
NFEECKQQYQGKYGGDTPSRADVIRTMECVTYCVAKKNNLLDDNGYVKEESFASDMIQIY
PEDSVKKIAVANAHNCTLEGNNFAKDNWEQVKTQFCNPASYMAAHCLMNAVDMDCPK
EVQNQSDTCQKRRQHLQNMKQNY
>BgerOBP32
MLFKSLILVAVLSAFVRGHTEEEIKELEKKCQEKHSVGDDIFIKHEKEELDSFEEFDITLKD
DTVENHRCYVECLLSEFGLMKDGILELDALVHLTEVEMNKRSITIDSAKLKADVVTCTAEV
AEGHCMVSYHAVKCLYNWTKEHVDQKKAEITTAFNLRIPRMMKFGKKKLAEHRN
>BgerOBP33
MNQEYFSTNMASSAILVVFALACSAVTCFSVQDYVASLDTQEAFDLLTGLPSSDKRVAREI
DYKFPPKCCGHEGVKFSDTDKEAYEACRVAPCWMECMGKNKTALDKDGFVILEEFKAHY
KTMYADADLHDLTDKAISTCVAAANAKAKEVGPEKIDGKECNRATAYTVMCIRYKIETEC
PNADKVDSVDCTKIRAYLKEWNKKTGLKYN
>BgerOBP34
MLSLLLFVCFICGSQGGPSLFSRTTRDTQHMQLEDLYCCSLLDEYMVGLKNGISQNAVRCD
FEAEVMVDEAFNASTKINRKQLIEDCNMLCRLKLENMVDERNLTKAEGCKDYLEKHLKD
GEMKPKLIAANAECVRYSNLHAELRKNSIEKKKICSPAAFIYMTCLVQHIQMYCPEDKLSK
TEHCVNEIKKLKVTYDV
>BgerOBP35J
MGGRIILLCVLTIISPFYTGATSDYNEISNSWTDANLEKTLSRVRRGLETSQPCCGETFFKSE
SFQEEMNECQKLKFQGTDEKNIYACLYECAGKKLDLVDEKGFVKLEEFAEFCPEFVEGDK
SLQPLVEKIARENLVTSVNNLSKSIRRQYKCNPAMIFAINNLYFKLQLACPDEFKSNTEECN
NHRAEIRKMQNAEKGNKS
>BgerOBP36
MKSFQNSTAATILVILGLAQFALGFSGRAFERAKEVDEKCRKEHQVDRGYFEKFIKAEIEK
TDPPHNYKCFVKCVMVELLALNDQGEFNIDEELENVPPEILEEGHRIIKACHGTEGADGCD
TAYQIHRCYHRENPELYSLVLHYWENASQK
>BgerOBP37
MGHFSCSFIFFVATSVSWFHIGGILGSVPLKNDNFLTEMTNLNKIHPTLSRVTRGEIAVPNE
PCCGNVMFNMDAFKEEIKECMEEKGERQMAEKYCLFECVGSKRFLTDDDGKLVKDEVLE
FAPSVVEGDPNLEELAKSITKRVIDVINEMAADMMLKEKPKCNPALDLYFQFLFVEIVMTC
PENMKSKSKACKEFRRKFEG
>BgerOBP38
MFLYALFLVSVLTASQAAGPQKRCQSPAIAPQRIEKVIADCQDELKLAILQEALDDLEISTD
VLTSRTKREAFSPDERRIAGCLLQCVYRKVRAVDSSGFPTREGLVRLYADGVEERGYYIAT
AQASQQCLQAAHQRRQKSAPRGQQSCDMAYDIFECISNKISEYCSGTN
>BgerOBP39

MGSRDFIIICAVISIFNTVSTDNIFDHLTFEEERLLVSRQRREVSVDNGTDEFANHTRSCCRL
EKSEHRPTDEEMDKHDQIRRECENITDALTKNLTADKSSNIMTCFVQCIYEKHNMTNSSG
NVLDDPCILSELQRIVDFPERMMKKMSEVCWKISKNNTESEKGKFVCNRMPLHFTICAYD
VANGFCPEAQQVKSDECNKLRQTLKATFQN
>BgerOBP40
MMKLQFIFVSVLLYTMSVRCEDERFAEEKTECNKLFPTADEAEEHLRNLGSLPDESDKTA
LCFIHCIMEKTGMINEEKELQAPVIVEKLKGFPNGTEIENLEEMVEKCVEENTQEDHCEKA
YGFAKCIMTSEINTKKEE
>BgerOBP41
MMKAATAVLSFAIALSSAVGGDEEGLLPEILKQGKKCVKDGNIDIAICNELLEKSGVNEDD
PKYASCKCGLACVLKNFGTMDDSGMLKREKFTELKERIKDEDLKKEAERVFQPCYDTVGD
KRDCEAGYALCKCAVESSETVKELLKMVMEKSKED
>BgerOBP42
MNSKLLFAILSAFVAIHVASAFGNVDVFLFSPLAGEDVESPAFLSRVTRGAGKGNLFKNCC
GQRDMPAFTQEDIETKKACKDEASGNADRKEGFICAMDCMAKKSGIVDSDGNVDAAKFS
EKVQTFYDEQELKDKVKDIVPEIVSKANAQKGDGACSKAAMIALFKTKAKIELECSESLKK
SGEECDKVREWSKKFDSQ
>BgerOBP43
MALSSTYLVIAFLCAGVSSFSVGPQDMDLNLRHEAVQQVMSLIGAARHRVTREVDIVHPP
KCCGKENIVISDDDKKMMDECDPHGKENGGMTACWMECMAKKKATLDAAGNVDVAAF
TKVYLTHYVDESLKPKTEDAIKTCAEMMNKKASEMGPEEYKGNKCNRAALLTSFCVRYI
METDCPKEQLIESDECNKLREDMKTWKDKVE
>BgerOBP44
MDSFLTSLLCTIVILPTLYAIPEIEVLHRYEPLKERFLSRVVRQSNKACCGTAPLKLDMIFDL
FQECKEEIGDVGKQNEGPCLFECAGKKLEVLDEDGKMKAEEYVELCQKMAEGDEQLEEHI
KEVCTTQAEKTKDFPENIQDKYKCKLDMLIAFQGTHIEILLNCPAELKNNDAECEKMRES
MKKQIQQAQG
>BgerOBP45
MATRAVVLLLVLTCVCVTAFRIKLLDETKNKYDLERHITRFIRSTRDEDTQRVENEQDDD
EEERLEELLWCCNIPPDNMMKNETAREKEQREDEKIIQECRTEVTNILGDEAAPKKRDRI
RKEMECFMQCAYQKFQVTDGTGNIGETGIDLLMTKLELNDQRTLDKITNICMSRKQGFGK
QSDGYTCNPAANNFNHCVYDMMNLYCPDKQKPFSKRCDKHREMLKMRYEP
>BgerOBP46
MEYINLLICTFIVCANVLGQQTSNTSGESGHTNTNFFSQCCPDTGMSKNFTNEEIEMQKQC
YSDAVKDEHVKDGKPIYGCVSQCYGEKKGVVDREGSVISEAFVQQVLQLYTDESIKNVVRT
VAPKCVEHCNAHSKALRAKDPNTYSCSPAMTLVNLCTYVEIETNCPSEIRISSEKCEASRNS
MIKMKNTMFP
>BgerOBP47
MASKSVISFLLIASAAFASGSFFDYESANPLLDASDYLADYYNYNSNPHARVTRGADKKMC
CGDGTIQIEESDKEMAKKCMTENGIEMPPPGDHKHGPGGPGGPGGKDFKEHMKKKACAC
ECMGKATNMIDDDGYTKTAFIDEVVTKAPAELKDFAKTVATDCVTNMNAKAKEIGKVEA
NGKQCNPAFGMTVLCTIANMETECPAAQQVKSDDCDKRRGFFKMIKEKVAKGEEM
>BgerOBP48
MKALHGSRVVRQAADPSVCPNDQMSFLSCCGKDSFHKDLEKQMTLMFSCAVELNITNMH
PNNTDPFSCEVIQKQQEAMICLTECVTKKLNLMDDSGNLKEAEVKEMTKSQLSEDWKKE

IADKAVTTCIEKTKNPPAAMAAGNHKCSLKPLLFGHCIWNEIHANCPEDKKIKSTMCTFV
TEAMKQYQQK
>BgerOBP49
MKSNSVAAILVGAILIASQRVNVRSQDLSPASTTGADSMFPNFITAFGEIQQCMSNNNVDI
GAAITNFFTTFTYDQNFKCGYCCVLQGFAMADEHCNPQLSKVKSVAEMIPIPDIKDKIVGL
LTKCEAAGKAKENEKCEAAFAFKDCVVAEVLTIVKEKFASIAG
>BgerOPB50
MNTPVIVLFIFFLVCQTALAFDVGCLMTTGIEAMEMVESCCGENNVTLESMANVFEKKCP
RQHKCALTCIMEAVSLIQDGKIATDFLINVAQMVPVQLVKKICIPLLTKCKAAGNGEDRCE
NSHRWYKCMTTKFLATALRAFTG
>BgerOBP51
MMRVTQTVVLAFFFFQVTVALNVKCLTDNAKEIVNHAVSCGKENDVVLDDMMEVMNDT
CEKKYKCTLACLLDAVMLLQEDGNMAIDFIYYIIELAPHEEVKKELRVLFDSCESQGRGHD
KCGNAFNLFTCIKEKVPAFVLSIIFGN
>BgerOBP52
MGTCSAVVFTFFMVFQMTLALQLRCLLIAVQDLESKIHSCCNENHIDISTLHKILQDTTPR
EHRCYLACVMDDMTLMEGDNVAIDSIKMMVEIIPFKAIKNIAVEVVKRCESSGKKKDRCE
SAHYFLVCIKNEFIAFLVNMFTS
>BgerOBP53F
MGTCSAVVFTFFMVFQMTLALQLRCLLIAINDIGSKMNSCCDENHIDMSSLHKILQDTTPR
EHRCYLACMMDDLTLMEGDNVAIDSIKMMVEIIPFKAFKNFAVEVVKRCESSGKKKDRCE
SAHYFLLCIKNESLVFVVNMFTG
>BgerOBP54
MKSITLLLVVFTYGVVTAQDLGDAVSLALGAVTECTEKNGIDLMQIIASLQQKKSPDERVK
CCLACIMEKLYVLSDDGQFQVELVSGLIESLPFESARKPLTDSVKSCAGQAVSNNKCEKAW
MFTGCLKRNLPKIGLALLMG
>BgerOBP55F
MVKTSIPIVILTFCLAVALTEVDVRNAIQECQDEQGLADVESLMMERSMTLKDENNEKGR
CFIDCLMKKFGIYKNGVLDVDAVLEQGKEIIQYAKNKGIKVDENTIRTGVKDCSSKDGQGK
CMKSYRIWKCLDDLAKATLGNEM
>BgerOBP56
MFKHKLFTPLKLVHMKLHTVVIVFLIAIVFEELHAASRYIDLGHKCFRESNITSPEDIEFVR
TRSPPITLSAKCYIGCLLNGVAACYIGCLLNGVAALENGRFLKEPKSLEIWTNTDKARYKTL
YNVCHKHVGDGNNSCITGPKLWNCINKYYNCADCVPVWKYKTQQK
>BgerOBP57
MAGRILLLCIFMTVNTILNTVTSDHNVITDSWTGEINLEKRNFILSRVRRKIRTTEPCCGET
FYNYNIFEEDSKECLVESSKYGKDWKVTFPCLYECVGRELDLIDDEGYVKEDALLDFLPQL
VEEDKKLTALLEKLATPDYINSMNERCKEVRKKQKCNPAIVLIINNLYMIMQMSCPEELIN
KSDECNRHREEIKRYL
>BgerOBP58
MGGRILLLCTFTTVTTILNTDATSSYNAVSNSLTDGMNLEKSNLTLSRVKRAFKTTEPCCG
DTFFNFAVFEDDSKDCLQESVKYGTDIKVILVCLYECAGRDLNLIDDEGYVKEDSFIDFLPS
LVEPDEKLMALLLKIAKKDNTIRHFNKMSKSSKTEYKCNPAMIFLINFLYMNMHLTCPDE
ILNTSEECVDHRAKIKQVLSY
>BgerOBP59

MMDTVFFSFQFIAMITLSPMSIGDVRGSFLEASETQPSLSRTVREISTNIACCGNETFKFNL
FTDKFDECKKEIKPGLRGDSLKRCLYDCAGKKLGLVDDYGLIIKGKFLEFCKGLAQPHPKL
EALLLYLAKRGVDALNDAARELIETKRFECSIAMDNCLHGLYRSFVLNCPIELKIMSDECR
KLHKELVDFQNERME
>BgerOBP60
MCCSISIYIFFGYTAMVISSLNADSAMGTKNLVPNLTTSHKTQPVLSKATGIMAKEPCCGKV
LYNRSIIFDEFEECKWSLASPELEKAEILNCLYECAGQNIDVVNDDGFVDKEQFLDMVPKL
VNDDEDLGDHLYELAKDLVDNMNDRAKSERNKHQCNISMRYVIKNLYWLMEMQCPQEI
IDTSDRCVEMRYKMSQRQRGSAPPNELTANGTNLLD
>BgerOBP61
MCCRISIYISFVYIVVVMSSLNVGDAQDSALGSKNLVPNLTTSRKMEPILSKATSTIATEPCC
GKVLYNRSIIFNEFEECKKSLAPELERAEALICLYECAGKNLEVVNEDGFVDKEDFLDMVG
QLVNDDKDLRNLLHGLAKDLVDNMNNWAKSELKKHQCNISMRYVIKNLYWLMEMQCP
YEIINTSDRCVEMRYKMSQG
>BgerOBP62F
MRNLSTIIYFGCIFIVMVSSLISGDAVQRQNLESKFPTSKTVQHRLSRVTRQYDPTNVFYDL
EHACCGKVEFNKLIIYSDYDECKQATGVAIRSPKIRQLCIYECVGRKQNVIDEEGYLIREEFL
ELTSKFVEGDKELQEFVIELAEKVIEPANTIAAEKVRSNSTCNVAMGSAIGFVYVNYQLRCP
DHLKTNSDECKEFRRKLYKEATKGK
>BgerOBP63
MNSVLIVLLYITVQVLSMDAPGHTQQLPNLMQFFSTSQYFNHIIFRVTRQTTTSVPCCGKE
KYSLSAITNHFDGCMEEIGDVQKEDQLPCLFECAGKKLGICDDEGYIIKDQIVEMSELFVDG
DEYLQNFLVQVAAKVVDYIKNIPKERRQKTKCNTELISFCHGLYHNMEINCPEDLKNKAE
ECEKLRADMRKKSQPKSG
>BgerOBP64
MDRIMMFLLFIIVPVSSIDTSGVSGSSPKVDKMFQSLMTSDHNKPILSSLDFDAEHLICCGK
EPFKARIVTDLYPQCWREIGKKESNNAQDMICVSECVGKKLEVVDDNGSIKEDDFMDLCH
NFVEGDEKLEEFVNEICSAQIKMVKYISKATRSKYKCNLDMIMAFHGVFIKTMINCPSNLR
NKGKECENFRKSMKMNN
>BgerOBP65
MDIVWLKYFFMIYFCFSNVCSSNINKLINITLRRDSRSAADCPKASHDCCRLEKPFLDEFIA
EHQTVIRQCYAENGGMENVVAGFMKDSQGYEMKTAKQKKISKELECVLFQCLVRDLKLID
ENQLLNMSACENFVKSTFKNREVLHILIKTNEKSVNFANKYGEAFKKSPKNTGCSPVGVQ
YYIIMGYAINAYCPEHKKVGGSKCKSYLEKVKKCYESDI
>BgerOBP66
MHCLLTSFLCVVVSTLNADGVSGNTLQTEHTSMTQHLLYFVNTMSNNSRIKRGAHNPYA
ACCGIKSYKVEIFQESFGICKEELGEVSFEDELPCIFECVGKREDLIEENGNIKEEEYLDFWY
RLVERDDKMEKLVKNIAAPIITKINELPKEKRNLFECDSAMKSAFETILVLIELRCPEDMQ
NKGKVCEKTRAELRQKVGIKQTASLDL
>BgerOBP67
MDSLLNYLLCTIVLLPSLYAIPEFEVLHHYEPLKDIFLSRVVRQSDVPCCGSVPFKTRLIAQF
FEECKEEIGELEKKDEKPCLYECAGKKLEVIDEEGKINVEEYVELCERMAEGDESLEKHVR
GVCSANAESVKKIPEDVQSQFKCRLDMAAAFQGTDIELLLNCPSNMKNYDAECEKKRDHV
VKKLKQSRA
>BgerOBP68

MDSLLTYLLCTIVLLPTLYAIPEIKELHRLEPSKDRFLSRVVRESDIICCGSVPYKIDMISEFF
QECKEDLGDLEKKDELSCLFECSGKKLEVVDDEGKINVEEYVELCERMAEGDESLVEHVR
GVCSSNAESVKEIPEDVQSRLKCRLDMIAAFERTHIELLLECPSNMRNNDAECEKKRENL
MKKLKQSEG
>BgerOBP69
MDSFLASLLCTIGLLPALYAIPDMGVLQRLEPLKERFLSRVVRQSDVVCCGSVPFKIDIITEL
FGECKEELDDLEMQDEALCVYECAGKKLEVVDNKGKIKVYAYIELCQRMAEGDKTLEEYV
RGVCYENAEIAKQVPEDMQRKLKCRLDMAHAFQSTQVDLLLNCPSKMKNNDAECERLR
DDMKKTIQEAEELAMAKKT
>BgerOBP70
MDRHLTSLLCTIVILPSMYAIPEIEVLHRLEPLHERFLSRVVRQSDIACCGSVPFKTEVITEL
FGECKEEIGDLEKKDELPCMFECAGKKLEVVDSKGQIKVQKFVKLCQRMAEGDETLQEHV
RSVCSTYAENMKEVPEDTQRKFKCRLDMGLAFIQAHFEIILECPSDMMNNDPECEKIRDD
MRFKLKRAEIEETQVQG
>BgerOBP71
MDSLLTSLLCTIVILPTLYAIPEIEVLHRLEPLKEGLPSRVVRQSNKACCGSVPFKTEIITDLF
DECEENIGDLEIQDEAPCFFECAGKKLGVVDNKGKIKVQEFVELCQRMAEGDETLEEHVR
GVCYENAGSIEYVPEDVQNKSKCKMDMVLAFINTNIEILLNCPPEMNNDDPECEKIRDDM
RFKLKRAEIEETQAQG
>BgerOBP72
MDSLLTYLLCSILLLPSLYAIPEIEVLHSLEPLKERFLSRVVRQSNKACCGSVPLKSGIITDLA
QVCTDELGDLQKQDQVPCLCECAGRKLGVVDDDGYIKVEEFVKLCQRMTEGDKKLQEHII
NVCINEAERTQEIPENVQSKFKCKLDMIVAIQAVELEILFNCPPEIKNNDAECERIRNDVM
KTIEEV
>BgerOBP73
MEISAFFLVILAVCGTVATFNLHQLEDNPLLPLHLHLQRVRRGTYNYLDKCCNYKRRELK
DGMKILDECDAEMSTKYGIVEPPAEEGKEDAKAGEHVFSEAEMSYQRNKSMCFYQCLFQ
KIKMAKEDGTINVEEAIKQSKEALSELTDSAAWGHIEKFCPKPSTAHTLKYVCKTDSADY
MECMYYMGEAFCPEAITRKSETCDAFREDVKKRYEI
>BgerOBP74
MGGRILLICIFTMSSLLYAGATNNYINKTANTWSAANLEKTNFNITRVRRGIKVKTSEPCC
GEAFYTCDTFKEEMKECQAQNDNYGKKDDILICLYECVGRKLDLVDEEGFIKKEKFIEFCP
KFVEGDRGLQALVEQIARENVVSAVNKASKDVGNEFKCNPAMVFVIINVYMLLQDTCPDE
MKNKSKCIYDFRFLVKSKANIFLWLCINEVK
>BgerOBP75
MRGLLLTFVYVYIVLLISPLQTDGASKLFPLHHNVKPRKADLEGAYEYVVLPEGIDKMPEE
PCCGDVKFSFNVMLDELEECKEELDADGKDDDVHCVMECVAKKLNMLDSKGFLKADKIT
EFVPGLVEGDSKLEELLKSFTDNTLSAVNNVAAGRIADNAFAKCNFVLQLYLNYIFRLFILQ
CPEEMENNNRECNKLREKVQYSLRMKD
>BgerOBP76
MRGLLLSLRKEFIVFLLISSLHADGASELLLQIHNVKPRKADLEGVYESVLLPKGIDKMPEE
PCCGDVKFGFNGIMDEIEECKDERDADGKDDDVHCIMECVAKKLNLLDSKGFLKADKITE
FISRLAEGDSKLEGMLKSLNRGTLSTVNNMAATNKAENAFAKCNFVLQLYMQFVFNMLL
LNCPQEMQHGGIECYKLWKVVDHNFHM
>BgerOBP77

MRGLLLSLLNEFNVFILISSLHPDGASELFTHKHNVKPRKADLEGVYEYVVLPEGIDKMPE
EPCCGDVKFSFNAMMDEMEECKEERDADGKDDDVHCIMECVAKKFNLLDSKGFLKADKI
TEFVPGLVEGDPRLEEMMKSFTRGTLSAVNNMAKAMKAENAIAECNFVLQLYMRFVFNL
LILNCPEEMQNNNGECYKVRKIVLHNNRA
>BgerOBP78
MQHMSFLFIFFTAILVFWLHLGDILGLVPPNHHDLPTKPTIQYKSSNTLARVTREGQGMP
TEPCCGNVMLSIDALNEDGSECIQEKEQCLQESSCLMECIAKKRDVVDDDGYLMKEELVEF
SPLLYEGDPKLQKLSRFFTEETVESINKKAKQMVKQENLECNPSFQIYLIFLMEHIVVYCPQ
DMQKKSTECEEFRKYIAKEK
>BgerOBP79
MCYFPFIICFFAMPYKLFSLLSFSLIFVSSLQAENISQIADLESKIRSTFENSQHILTRKIRGEP
GPPKEICCGKVKFNQDILKWEMENCEEPPDSPDRTPSITCIYECAGKSQDLLDENGYMKVE
ELAEFSTFTVTGDEKLEQHVRKCSKKIIPGLNESVRKMKEDMDMDCNIAFETFMELLFSY
VMISCPREIRSESPVCIKYRQELKEYVLSEAEKIKRGEYD
>BgerOBP80
MQHIVFSFIFFTAILVSLLHLGNVLGLVPPKHHDLLTKATELYKNNISLSRAIRENQPMPVP
DEPCCGDVVFNMDAFNDDVMKCIEAGNKLKEDIRACCVMECIAKSLKVVDYDGYLLKEEL
VDLSPSFFQGDHELEKLCRTITEGLVEEMNEIVKRSMEEEKSECNPSFKLYIHTLLVHITRD
CPENMQSKSKVCTEFRKYVTSKR
>BgerOBP81F
MEGRVFFLCFFTTVTAPLYAGAATSYYSEFTNFLTDVVSLEKTNLTLTRVKRAITTSEPCC
GDVAFNFNKFEEEIEHCRIDSDVSPEEKDSALACTYLCSAKELMLVDKNGYLRPGDFLDFC
PQLVEGNDRLERLIEVIVRADLVKKINEMCKDMKRRPPCNPSTLLLIYNVYRVLQLHCPAD
MKNKSKECISHREELLKMK
>BgerOBP82JF
MGGRIFLICIFTAVTSTLCTGYNEVTNFWNDVTSLEKTNLTLSRVRRVVKTSEPCCGSIFYK
FDIFEEDSKECLADSYADNSDKRVARSCLYECVGRELDLVDSQGFVKEDAFFEFCPQLVDG
DESLQEVVEKIVKHKFVKTMNYLSKGRKIERIERHNRFVSIACPEELQNKSEECDKHRAEIS
NKLNV
>BgerOBP83
MVALSVVVILCLLLSGCTFAEPPDPPRTSSTTTTTEDPGSYTVEAKGSLQWKETITVTKSK
GGNEIKRSYTKSSQPEDKRDHHHHNGFIPKCCNAPSNKTNDIDFESRRTECMTQVEKVLG
RKVTSKLMSKDDYLTMNCFLQCTSRKVGLSEDNGSIKPKEMTDYTMADSKMIELGITSEM
VNACVITSKGNSANSKITTQGGDVCNMTNYEHIRFMKCMVRMENVNCPAKYQVQSSACD
KYRETHKK
>BgerOBP84
MCFKTLITLFSVCCFFPQFLASTPKRTTDNFMKCGGQYGIKTAEDEDKAAKAISNGTLTDD
QRCYWVCVLTELGEINKDGTLNESYAVPELLRATQRHRNDFPEEKIKSDVAECAKKESEE
KCGMSFERWRCLYSLSVKMYLKILEN
>BgerOBP85
MSIKILIFIGLLIYCVKNNLAGTYSCDETYGYTKAEETYGALGDTEAGIGTDKQKCYWECTK
KAANVLKEDGNLNDEFYRQMLKKAKDDYRLPYFDEKINSYIDECLTKIVGEGCAKTVEAF
SCVGNLTFEFDKLIYMTDCEEETGIYDEEDFDPVTMNESFRKNTLTDTQKVILN
>BgerOBP86
MGSCLVRLLISLLCLGYADVISANQKMELDGILIRHVRGTQSYYEECCGKGKPEYTDEESKA
LTECWNEAKEKIKNDAPDVNTRITECTTYCMAKRDKYVDANGYLIEEEFKRVVSDYYPEE

DVKQVVRQNAHNCTVQFNQYAKDNWEKNKDVFCNPSALEGAFCLMESVELDCPEAYKS
KTETCDKRRNRLQQKWRRNQ
>BgerOBP87
MKILIVFVLVIKLAEESTNFGNIGILQSQGEGPTVEQGNNIFEYNSFVTRKLPSLNRVRQRSR
TGTSTQENDTNCCGERLPTPERAMTAYKECTESTELDIRKLGGRCVIFQCMGKYFEVLDD
EGYVKEEEYAEAYAELHPPYMKSLVVACVADCIKINEKAKLMVDKWGVKCNPANSMMID
CMKAILEVKCPEDMQIKSPECNKRRQICAKKVQKKGYDPDD
>BgerOBP88
MGSKCSFVVIFIPFFMSVAGFSLKDVFTYDNFLAVTKTLGAQPIPLQSGTINMSEVFPDLVH
PKCCGNSMPDFSEDDAKIFMNCTKQVGLLNGTAGEIAEREIAECVGRAMGVINETGRVNL
TEFLKYVEKFYGPISMKNATQDFAPNCLECIYTAYGNDMTSNTTCNPEMLQATRCVKYSV
LKNCPEDQKNNSTSCELYRAELGGLNTTDY
>BgerOBP89
MEAGTIILILGIVTLTMSSYQSQILEHGLVINSGIVTLDGGNENISNTENGDDVNQTEFLPCC
GKVNFTLRELKQNLYQCDYYNQLKDDEEERIVCMMECLAKAADLANFTGYIRKERFLDYI
GNFYEYDDELTEHAEKVMEYFFDMIYLQMLEQRGGKFCNPQMTNYLVFLRDAVEYNCP
MHMRNYNCTQREELGIIVK
>BgerOBP90F
MEQAVLFYLLAAVTIQLHYFSEALVTGPNVQPTWSKAEEDDSEKKEAADQSICCGSIIFST
DFVRKYTKKCKKSFRKDRDDDDNTNCLLECIGKVMGVLDAEGFVKEEEFIEFWGNRFRE
DEDLKKHAIDTAEKHILHLNIRIKELVEEENIACNPSMEYMINYLDEYNIIFCPEKLMRQSE
FCEWVKETLTKKHESHIEKE
>BgerOBP91
MIADHQHVLQVLGEHYQIQSLRAKRSKDKEFEHETCCGTGQHALTNAETNAIEKCNHHL
HDDGDFDEMKGKDEKMMEKFDCYMLCYGDVTGICSATGDVNKAAMEEELKRHYPEPEV
KDLVGKLCPICIDGMTKYAKDNNNGKCCKACGHSVACILHLVDCMCPKKFQVQSNECEAL
RKEFKDMMEKNS
>BgerOBP92
MGSCVKLLIFLISIQLLDVTSGWPFEPNRKFLSSRRPIRTRNEKVGQLSRFKRQSEAEEISET
CCGTEPELTNAEEDLYQKCKKYLPAVNSTDDRDYIHGCYQECFAGHLGWLDKDGFIILEK
YISGILYFYVEPELKDLVKIYGPECVKSANKQADDDPKDDDCNSAAGRASVCVSLVVDKHC
PGGLKRKNTYCDRYFQEISGLKPQTS
>BgerOBP93
MGSCFKLLIFLISIQLLAVTSGWPFEPNTKFLSSSRPIRTRIEKVGQLSRFKRQSEAEEVSET
CCGTEPELTNAEEDLYQKCKKYLPAVNSTDDRDYILGCYQECCAGHLGWLDKDGFIILEKY
ISGFLYFYVEPELKDLVKIYGPECVKSANKQADDDPKDDDCNSAAGRANVCVSLVVDKHC
PSGLKRKNTYCDRYFQEISGLKPQTS
>BgerOBP94F
MRSSAILALISLSCAVFQLGWTFEPSRKFFSNALHAPCNESRFRRDIGDKLDVFNCCGERD
NITAEDIKIYEKCIEKLKGEKTVNISSANYSCFAECYARNKNWTDDNGFIIAERYLASAKQF
YKEVELQALVDKYGPDCVKDGNRVGKEKQTSCNPASSIVDSCLFISVDQECPAKLKKKSDI
CDKYFKALSED
>BgerOBP95
MSYLCSYLFVAFIFGVGQCLESNYRTSLRNEYYPRSEVNFETAEIWEDAEIHGIGEKEFKGI
KENGVEHSRIKRADISCCKSPHLFNITSTDKTGHKNCSKAATKNPLCADICFLTKRNMLSA
DGLVMEDAVRMVVKRGQLQRWQKNIILNAATTCTETANKQFRGKRSPQRGKIRREPDAE

MLPSLRCSKVGVTFMNCIAKALEDGCPSNLRIHTKECQSTEVDDLMSNGVAGTGQVEYEK
SPIRFN
>BgerOBP96
MAQGVLFYLLATVTIKLHYFSEALVTGPNVQLPLSKAAADDSDKQDTICCGSTGADLYFLE
EYGKNCTKWLRRGRRHHEDSTYCLLECIGNILGLVDAEGFVKEEEFVAFWGNRFPNDEN
LKKHALDVAEKHIQDLNTRIKERVIVEEEYKPCNPSMVYMFDYLNGYNVLFCPEKIKNRG
EYCDSLEEKLMKIHGSHIKRE
>BgerOBP97
MRQLLRAVVLLIAVASTLQKQEHKDKTQTLNPNRLLETPKKLCCKVENSRLLTIDMKRES
VKEKCKEEIKETIKKIKESLESGTLSGMAGGIPLASHTTICHSSCELSEFNVLDDKKFIDAEK
ARKFVEENVSEGIPRDSALKVTDECTRISNLRAQFTANSIEKGQMCNIASTYYLLCLKDLVI
LDCPEDKKVDNPECNAFIKLAKIHYDKC
>BgerOBP98
MSYLTVLFFMVVVSLLFFDQIDCGSNYDYPHFIGVHVLSRNRNKREVNDDHAVDISTLCCN
FTEPNDEKYEKYNKIFDICSDEVDSRPEFNYTIDYKDYDVENFTIDEVDGDFNFSAALDNII
YNYSSCIQECVFQKLEAINSDGNIIPDKVEEYITWLTPLTKDDLWRGLDQCGIIFPDSHRFL
CNSGPMDYLDCIIDFSELHCPLQHRTYGPGCSYDDENEAQKV
>BgerOBP99
MICFIFIVIVFTSSVTSTVEEPEVSRKVKHAEKCCPFEPQPTTSEEQSEEIRSRHEKIRDKCIQ
ELESSKVKALGDEYEKIYDCRYACVYNAIKVLDDQGNLNEDETLKLFFEVMYIPNPETIEN
SKLHCWRTLRENEQKIEDAHVCSTATREFLLCTMDMHILHCDDPIFNKEESCVKTREELK
KRYH
>BgerOBP100
MVSHYIALAVLFSVYFSAKGELTIFAFGGTLEPTIFKREVGNEANNEDCCLPTHNIKRTKEQ
IDADKERDRNITEECEKEVQEKLSEAGALDDPMQRIECNFECGYKKLQVFDDDGELIRILT
LKNFLEIMDSWNMNIFWRSGQKCWRKFQANLNSTSEGHACHPATKELADCVNEIKVLYC
KDESFNNNDRCLQYRQTLKQKYESH
>BgerOBP101
MLAASVVLFLAASPLLVSGLVLQEGGVLAYHDAAVKSSSGLYNGKDKKPLKSEKAYIEGTD
AADGNTDKTKEEQESERIEKECEEQMKTKLNVVDSNDDDEPNVQQEHEKDCFFQCLFQK
HNAADEKGNVVEKNVIIFFLKNSPFKDKAKLNKITGECIKKAKDEGKIKEADVCNPFSNYV
YHCIYEDMHMEM
>BgerOBP102
MDSILTSLFCTIMLLPALYTVTEMEAVLHRLKPSKDNEIFLSRVVREPKPNPICCGNKPLK
MEIVQVALEECKEDINGDLSKEDLMFCVFECAGRKLEVVDEIGDIKLEEFLELCERMAEGD
RKLVDHFPNVCASKVEEVKYIPQDIRSKYKCKLDMAFCIKESTLDLLLNCPDDMKHNDGL
CEYKRERLRQHQENQG
>BgerOBP103F
MCGRILLLCIFATVTTILNTGATSSHNAVSNSLADGMNLEKSNLTLSRVKRAFKTTEPCCG
DIFFNFAVFEDDPKDCLQESVKYGTDMKVVLICLYECAGRYLDLIDDEGYVKEESFIDFLPS
LVEPDEKLMALLVKIAKKDKMIRHYNKMSKWSKKEHKCNPAMFFLINMLYINMHLTCPD
EILNTSDECDAHRAKIKQALPQ
>BgerOBP104
MFSIALNVIILNVLFVSTDAFSLARSEIDWKQRHIAEMIVVQERYSFLSSSTQAQKNAEREP
WELPISRTVRAAGKAQPGQCCIKPKEELSDKMKSSYLNCFKETRPSMNKAEDNETKKHW

IRYFICLSQCVGNKYDVLDEKNFLVKDKFMAHIRNENQTDEVFTQKEEIITRCIDFSNQFSK
ERGDVDFFGKSCNMAPLAFVQCIKGFEQINCPEELKVKSSLCEMARMRIEELILIWKI
>BgerOBP105
MMKIAFAYFLSLSLVCIDSFSAIHEQNDLDQYILQVTTAQEHDSLLSLVRGQRSTSHKNSW
ELAASRTVRNAGNDQPQQCCNIPKKELTDKATNSLLACIQEVRPSGVGDEELKKHSMKQV
VCLTQCLGKKYGLLDGNGFVLKDKITEFAKTESDDEVFNQKEDIIARCTDFSKKFADERGE
VKFFEKSCNMAPLVFTQCLKGFVEVNCSEEKKVKTDVCDMSRMRLAEFIEIWKI
>BgerOBP106
MVFKVVVILCVIIASFSKVSSNRIFDGLDFEEEYSLDLRKRRETNKPEAIEEISYHTKLCCNL
ENSKRIMTQEEMIKNEELLSKCKDTSAALTNNLTDDKTRNSLTCQFQCMFEKYNLTDNA
GNILDDPTVMTVVAENSDFSESVIKKSFEVCRKISKKKMESEKIHSVCNRMAMHYILCAYD
VKNWFCPEEYQNKSEECKKHRETLKSTFEN
>BgerOBP107
MFFNMVLSGFILLFFVIYAASSAEDHVQNTEKKMLIRERREESERIQEIKDHIQTCCNVTVT
HTKTEEEETDERVIKEHCSKFVYDKLQNITTDRMKNRATCVFQCAFEQMKLADSDGNLIE
DESTLGTLGLLLSVKDEKLLKKVSTHCGKISKENAQKETEKFVCSRRPILYTLCAYDVMNW
FCKEEAIIRSKQCDALRQNLKAVFLE
>BgerOBP108
MLFRCLCFLVFFALVTASYNEEEEEEEEEEEEEEEEEELEGDFLERFKRQAEEANGTDTDD
GPPPPDGPRPVHPKMRRCCKKMPRVIPGRMLRGCGFRGGPPPPPGPPPPGGGPPGPPPPG
GGPPGPPPPGGPPPPPDGGDIDENDGGIPGPPPDDEGGEEEEEVTQGPVAGDEAPQKYKG
PSGGRGGKKCKGGRGRDKGAWMEQLACTMECAFNKSGMIKEGKVDQDAFKADMEKMK
EDKNGDVWKDAIDAALQNCSTTVPGAVKQDQKCKSGALEMAICMDNLLFANCPESLWK
SDNEKCKACRTSPENCAPAKREKKKKGD
>BgerOBP109
MGSHPIILLLAFACGATLAFRLPGQNKKIEAESSSDLANEDFIPQIVDPLSIDEVTLGRFVRS
PGEIEESPVDSELEHEEELTNFELLNLLDATLSRVARSPQDDTEGGQEGGKGKGIGKGKGK
GKGKGKGKGKGRDQEEDAAGLADDILALDIEESLLSRIARSPQDDAETEGGKGKGKGKGK
GKGKGKGKGKRKGGEEEDAGLTALDADLEGLNLESEIPSALFNSVLSRIARSAESEDAQEL
TGLESELDFDVLDEDVPLLESELLLERQVRSPQGPPEGKGPPEGKVPPAGKGRPEGKGKPS
LTSQLRDLDSEFDFDILNIGEALLNRETRSLQEGKGRPEAQGKSEFADMRNAILGLAASED
LSLLAQMENTEVGFDEDYLDREKREGKGPKSCCEGSSLFKPADEDKELKKQCIEELKNQE
GETKEKRHAHGGHGGHGKGHGKGHGRGPCVWECIMKKKGLIDADGNIVQDKAKEEVLK
KYTESWKQEIAKKYIEECAEFANKPGGWGKHGPKGDKDGHPEPAAVEEEGGPPPHKGPK
CKPTGLKFVMCMEKKVKQDCPAEHKLEGEQCKARHKHGGHGGEGGPPPPPPE

5 newly-recognized ZnevGr proteins
>ZnevGr88
MKIVRVKPIENPKQIHADEHTFYRDVRPMALVCKVLGIFSLQNILQGDGRRLKHKFLSIDAI
WGPIFIGALPIPFIEYGNDDFGRYWRIMQCLRGLITVSLSSYYDASLPEMISKMETLGVVLR
SMRSKTTERNINRHGRISGYVGVAGSVLFVSMNTFVEYVIKRVSFPRLMIHLYGTLNLIPRQ
VYVVMYIFFCYNIILLFRDVQSCWKEHVNRTDEREKQLENLRLVHVDLLRIIRLLNQAYGS
RLAFYLATIFIEVLLDLYIFFFYNNYGYIQMEYYVFNAVTFYMLTSVTDELSHVGDYIALDL
MKIRMTKLNSGCRNQVLMFLRQLIANRVQSVSAAGFCAINKGIIVSILIAVVTYFVVIYQQAS
GRN
>ZnevGr89

MAFVIEITAHRTSLTNRNESLKEEIARENKTFYRGLKPFALICKLIGVFSLQNVLQDNGCLL
KHTLFSFHTLWGPFFVGMATISNTYFETEHFASYIAMFYIIRGISISLLTSYYDKFLPEIIFRI
EEFNAIIKSNCNKTHTRRNLNTHGRLVLYIACVGYIILMCANVAVLSLINGYNFPRVVNKIC
DSFTFLTRQMFVMMYIYFCYNIKLILCSISSIWRKTVKTIVINKNVDPVPPEERLESVRLLH
AEAVQTVELINSAYGVRLLFYVTIYCSEVLLSLYEFSNRHVHLKLYFIIYSGFTLYMVTKFTE
DITTQGNVMADDVTDIPIALLNNECQQQAEMFLSQIMVKKLQVSASGLCIINKRLIISIVMG
ITTFFIAIYQLSQET
>ZnevGr90
MFIDVSSNNSVLTCTGPFKCSKFGVGLACGHLAFLLWTTYTTLVDPNANPWNLLDSLLAG
IFVIRATVTQSRSVICLNQLYTASSRFQFSSLAFFCFAVCQSFVLTFTGIKSFRSCLENVFLLLI
QFSVESIIGAMERAFELQFHSINEELETIASQKTQLKSGNFRGIRNQEESKLQRANSGHILR
KLCSKLIRMFQNAFRRQGFNRRKVTFRKTPRKLLNSDRAFRPLPWVVQASRITQHPQTKY
ERNIFARFEIPGKNILGHSYTCDLLEEIRLAHGHLSDIAHRVNCHHGPEVLMTTTVNFCKV
VLILSDFVWEIILQGSATASLITSTTISFMLQLSRILYICYRGEKVCCETDRTKDLLLKLSDST
LEPNIKQEVSIFIKQLHAKKIQFSACGFFCWNKGLILPMVGAVTTYSAMLIQFQSTGFSFK
>ZnevGr91
MFKNLVFTSNQMKVDLKREYPIMKINEDSEYKNIIHKSFRPAYLFSKALGVMPLSYKIRSS
AKVTEARNRKMNSMEFEWSWKGAIYSGLWIALLITIRYFIFITRRSPPPRVERDSDHSNST
FENFSNWSNSHPPPFPGGREHLIGSMNELLDFTCTLLALIIGVVGARKIPEIFRQLQDLDEN
ADEDGHMLLDSAERRCLSRYGRMFSVFSVIACCSYTLTGTFAASYSAISVGVPMEIILPSMF
GDLMKLGNAAGEAQFINFCFFLRQRFSVLNDNLTKLQMDTRKTKLDHWFNDSSQKAPYG
PPNHLPESLASRAHPLWGRVTVANPRVISMTKDEVADTLGRLCRWHRRLCDLVDMVASC
YELPLFVVIAYCFANSVFGVYLVITFRGPDSLLRITGVIWSVAYGCRLILIAVIPSVTVAQAKK
SRMLVERLNNRYLDDASKQEILIFISHLSSRNISFNVCGFFTLNIPFLRSIAVAIVTYVILLMQ
FKFPEN
>ZnevGr92P
MYKSKNSVGDPQCPWYHEYTSAKPLLRVSQVVGLAPVAWPNLRPVTYGIVHTCVMFIAL
FGWFIYATALTILQEYPLKEATYIVPDFCNSASLYLSSIVSLALCATANRYRPQTIMRLVAQ
ADNAVNAVCPSSVHDKTRILVTAELVVSVSGLTLLAVYDNVVWTGKLWNFHNYTGRYYA
HLINLLVVLQFSSLTLLLKQRFARINKLLKSTVITSNDSDAWSQRSILEDAAPSQLVFKSRA
RGFAPPVGVWRDQQVLALRKAHSALCEVASTVNDMYGVQILLVITSDFIGSVWPLYLLLV
TNFNSEETHTTHRTVAWSLLFLLZRCLKILLVVVSCHMAKIEGRRTGCVVHQVLLHGEDP
RVTGGCSRLLQLQMFSQQLVHTREQIDFTACGLFPLDMTLLHSVVGAVTTYIIILMQFQTK
